# Effects of interleukin 1β on long noncoding RNA and mRNA expression profiles of human synovial fluid derived mesenchymal stem cells

**DOI:** 10.1038/s41598-022-12190-9

**Published:** 2022-05-19

**Authors:** Yang-peng Sun, Yun-yang Lu, Jianyu Chen, Jia-hao Bao, Hong Zhang, Jia-dong Sun, Wen-ting Liao

**Affiliations:** Hospital of Stomatology, Guanghua School of Stomatology, Sun Yat-Sen University, Guangdong Provincial Key Laboratory of Stomatology, 56 Ling Yuan Road West, Guangzhou, 510055 Guangdong People’s Republic of China

**Keywords:** Stem cells, Molecular medicine

## Abstract

Synovial fluid-derived mesenchymal stem cells (SFMSCs) play important regulatory roles in the physiological balance of the temporomandibular joint. Interleukin (IL)-1β regulates the biological behavior of SFMSCs; however, the effects of IL-1β on long noncoding RNA (lncRNA) and mRNA expression in SFMSCs in the temporomandibular joint are unclear. Here, we evaluated the lncRNA and mRNA expression profiles of IL-1β-stimulated SFMSCs. Using microarrays, we identified 264 lncRNAs (203 upregulated, 61 downregulated) and 258 mRNAs (201 upregulated, 57 downregulated) that were differentially expressed after treatment with IL-1β (fold changes ≥ 2, *P* < 0.05). Kyoto Encyclopedia of Genes and Genomes pathway analysis found that one of the most significantly enriched pathways was the NF-κB pathway. Five paired antisense lncRNAs and mRNAs, eight paired enhancer lncRNAs and mRNAs, and nine paired long intergenic noncoding RNAs and mRNAs were predicted to be co-expressed. A network constructed by the top 30 K-score genes was visualized and evaluated. We found a co-expression relationship between *RP3-467K16.4* and *IL8* and between *LOC541472* and *IL6*, which are related to NF-κB pathway activation. Overall, our results provide important insights into changes in lncRNA and mRNA expression in IL-1β-stimulated SFMSCs, which can facilitate the identification of potential therapeutic targets.

## Introduction

Temporomandibular joint osteoarthritis is one of the most common oral diseases. Owing to the limited ability of the temporomandibular joint condylar cartilage to repair itself, there are currently no effective treatments to reverse condylar cartilage injury and osteoarthritis. Synovial fluid-derived mesenchymal stem cells (SFMSCs) tend to accumulate at sites of injury and differentiate into cartilage, an important mechanism required to maintain the physiological balance of articular cartilage^1,2^. The differentiation potential of mesenchymal stem cells into cartilage cells is regulated by the local inflammatory microenvironment^3,4^.

Interleukin (IL)-1β, an important pathogenic inflammatory factor of temporomandibular arthritis, is significantly upregulated in the pathological state of the temporomandibular joint^5^. Additionally, IL-1β inhibits the differentiation potential of mesenchymal stem cells into cartilage cells^4,6^. To date, the regulatory mechanisms underlying this biological process are not well understood. Our previous study showed that activation of the nuclear factor-κB (NF-κB) pathway and its downstream signaling molecule IL-6 played important role in regulating this process^6^. Moreover, downstream signaling in mesenchymal stem cells stimulated by IL-1β mediates the development of arthritis via induction of molecules such as IL-6, IL-8, and matrix metalloproteinases^7,8^. Therefore, studying the gene expression profiles induced by IL-1β is essential for developing novel strategies to treat temporomandibular joint osteoarthritis.

Long noncoding RNAs (lncRNAs) are noncoding RNAs with a length of more than 200 nucleotides. LncRNAs are involved in many important cellular physiological processes and closely related to the pathophysiological processes of joints. The regulation of joint homeostasis and pathogenesis by lncRNAs is complicated and widely reported^9–11^. Several studies found that lncRNA has an important regulatory role in the metabolism of articular cartilage, including the proliferation and apoptosis of chondrocytes and the degradation of extracellular matrix^12–17^. LncRNAs can also regulate inflammation activation, proliferation, apoptosis, migration, and invasion of synoviocytes in the joint^18–20^. Recently, studies have found that lncRNAs affect the differentiation potential and immune regulation function of stem cells in the temporomandibular joint^21,22^. Our previous study showed that the lncRNA *AK094629* contributed to the inflammatory activation induced by IL-1β in synovial mesenchymal stem cells of the temporomandibular joint^22^. Stem cells residing in the joint are important regulators for joint damage repair and physiological balance. Therefore, exploring the relationship and underlying regulatory network between IL-1β, stem cells, and lncRNAs in the temporomandibular joint is important.

Accordingly, in this study, we analyzed the lncRNA and mRNA expression profiles of human temporomandibular joint SFMSCs in response to IL-1β in vitro using microarray analysis to identify crucial lncRNAs and mRNAs regulating pathophysiological processes.

## Materials and methods

### Ethics statements

This study was approved by the institutional ethics board of the Hospital of Stomatology, Sun Yat-sen University, Guangzhou, China. All methods in this study were carried out in accordance with relevant guidelines and regulations. Written informed consent was obtained from all patients.

### Source of SFMSCs

Synovial fluid samples were obtained randomly from patients with temporomandibular joint osteoarthrosis after arthrocentesis and lavage treatment at the Department of Oral and Maxillofacial Surgery, Guanghua School of Stomatology, Hospital of Stomatology, Sun Yat-sen University. The SFMSCs were isolated and expanded in vitro using the method in our previous way^6^. Briefly, the upper joint compartment was expanded with about 1.5 mL lidocaine using a #8 needle and syringe; fluid samples were then withdrawn. The collected synovial fluid samples were centrifuged at 300×*g* for 5 min. After centrifugation, the supernatant was discarded, and the cells of individual samples were plated on culture dishes (Corning, New York, NY) with complete culture medium (C12571500BT, α‑minimum essential medium, Gibco, MA) supplemented with 10% fetal bovine serum and 1X GlutaMAX (35050061, Gibco, MA) at 37 °C in an atmosphere containing 5% CO_2_. After 48 h, the medium was withdrawn to remove nonadherent cells and replaced with fresh complete culture medium. After reaching approximately 80% confluence, SFMSCs were passaged at a density of 500 cells/cm^2^ (passage 2) and expanded in 10-cm plates. At approximately 80% confluence, the cells were treated for 2 h with 10 ng/mL IL-1β or an equal amount of vehicle as control. The medium was then aspirated, and the cells were washed twice with phosphate-buffered saline (PBS). Total RNA was extracted using TRIzol reagent (15596-026, Invitrogen, CA). RNA quantity and quality were measured using a NanoDrop one instrument (Thermo, USA). RNA integrity was assessed by standard denaturing agarose gel electrophoresis. The characteristics of SFMSCs were evaluated as described in our previous study^6,23^.

### Microarray

The Arraystar Human LncRNA Microarray V3.0 is designed for global profiling of human lncRNAs and protein-coding transcripts. Approximately 30,586 lncRNAs and 26,109 coding transcripts can be detected by third-generation lncRNA microarray.

### RNA labeling and array hybridization

RNA sample labeling and array hybridization was performed according to the Agilent One-Color Microarray-Based Gene Expression Analysis protocol (Agilent Technologies, Santa Clara, CA). The hybridized arrays were then scanned using an Agilent DNA Microarray Scanner (G2505C).

### Data analysis

We used Agilent Feature Extraction software (version 11.0.1.1) to analyze the array images. We then used GeneSpring GX v12.1 software (Agilent Technologies) for quantile normalization. High-quality probes were screened for further analysis (at least 3 of 6 samples of a probe are labeled as Present or Marginal). *P* value/false discovery rate (FDR) was used to assess whether there were statistical differences between two groups of samples. We used fold change to screen the differentially expressed LncRNAs and mRNAs.

### Mapping and identification of differentially expressed genes

The “RCircos” package in RStudio (R version 4.0.0) was used for mapping the differentially expressed genes in the human chromosome^24^.

### Gene Ontology (GO) and Kyoto Encyclopedia of Genes and Genomes (KEGG) pathway analyses

The functions “enrichgo” and “dotplot” in RStudio were used to analyze GO terms and visualize the enrichment results, respectively. The RStudio “enrichKEGG” function was used to analyze KEGG pathway enrichment, while “barplot,” “browseKEGG,” and “cnetplot” were used to visualize KEGG pathway enrichment results. The *P* value cut off was 0.05, and the q value cut off was 0.05 for screening. Fisher’s exact tests were performed to identify significant GO terms, and FDR was used to correct the *P* values^25,26^.

### Protein–protein interaction network analysis

The STRING database was used to analyze the interactions of proteins encoded by differentially expressed mRNAs with fold changes ≥ 2. The confidence score was set as the highest values (> 0.9). The interactions data output from the STRING database was subsequently analyzed in Cytoscape software with the “CentiScape” and “MCODE” plug-ins. Top 30 MCODE score (K-score) genes in this network were selected for further network analysis. Subnetworks were screened according to K-score value which was calculated using the “MCODE” plug-in^27,28^.

### LncRNA and mRNA co-expression network construction

The “WGCNA” package in RStudio was used to analyze the lncRNA and mRNA co-expression network^29^. The Soft Threshold value was set as 18, and the “one-step network construction and module detection” method was applied. Differentially expressed lncRNAs and mRNAs showing a fold change > 1 were analyzed. K-score, which represents the central location of a gene within a network, was used to determine the crucial genes in the co-expression network.

### Reverse transcription quantitative PCR (RT-qPCR)

Five randomly differentially expressed lncRNAs and mRNAs were analyzed by qPCR. We obtained the cDNA using a Superscript Reverse Transcription system (Takara Bio, Shiga, Japan). Relative gene expression was determined by qPCR using the SYBR Green qPCR Kit (Roche, Basel, Switzerland) and the Roche LightCycler 480 System (Roche). The 2^−ΔΔCt^ method was used to calculate gene expression levels. Primers used are listed in Table S1. The primer sequences were designed using PrimerExpress software (Applied Biosystems, Foster City, CA, USA) by a technologist at Generay Biotech Co., Ltd., Guangzhou, China and was validated in Pubmed. Primers were synthesized by RiboBio Co., Ltd., Guangzhou, China. The unique products after PCR were confirmed by melt-curve analysis using the LightCycler 480.

### Western blot analysis

Cells were lysed in RIPA Lysis Buffer (P0013B, Beyotime, China) supplemented with Protease Inhibitor Cocktail (CW2200, CWBIO, China) and phosphatase inhibitor (CW2383S, CWBIO, China) to isolate proteins, whose concentrations were determined using BCA assays (CW0014S, CWBIO, China). The proteins were then incubated at 99 °C for 10 min for denaturation and separated using 6–8% sodium dodecyl sulfate–polyacrylamide gel electrophoresis (P0012A, Beyotime, China), followed by transfer to polyvinylidene fluoride membranes (ISEQ00010, Millipore, MA). The membranes were blocked with 5% nonfat milk (232100, BD Biosciences, CA) for 1 h at room temperature. The blots were then cut and incubated with the following primary antibodies: rabbit anti-phospho-p65 (3033S, 1:1000; RRID:AB_10859369; Cell Signaling Technology, MA), rabbit anti-p65 (4764S, 1:1000; RRID:AB_331284; Cell Signaling Technology, MA), rabbit anti-IKKα (61294S, 1:1000; RRID:AB_2799606; Cell Signaling Technology, MA), rabbit anti-IKKβ (8943S, 1:1000; RRID:AB_11024092; Cell Signaling Technology, MA), rabbit anti-β-actin (4970S, 1:1000; RRID:AB_2223172; Cell Signaling Technology, MA), and goat anti-IL-6 (AF-206-NA, 1:1000; RRID:AB_2838055; R&D Systems, USA) at 4 °C overnight. After washing with PBS supplemented with Tween 20 three times for 10 min, the blots were incubated with secondary anti-rabbit IgG-horseradish peroxidase-linked antibody (7074;1:2000; Cell Signaling Technology, MA) or secondary horseradish peroxidase-Rabbit anti goat IgG antibody (EM35112-01; 1:10,000; Emarbio, China) for 1 h at room temperature. The immunoblots were then visualized using an ECL Kit (WBKLS0500, Millipore, MA), after which the protein levels were semi-quantified using Image J (version 1.52t; NIH, Bethesda, MA).

### Cytometric bead array (CBA) assay

A total of 10^4^ SFMSCs were seeded into a 6-well plate and cultured for 48 h. After corresponding treatment, the culture mediums were collected and centrifuged at 3000×*g* for 10 min at 4 °C. A BD IL-6 CBA kit (558276, BD Bioscience, USA) was used for measurement the concentration of IL-6 in the culture medium according to the manufacturer’s instructions. Results were obtained and analyzed using a Cytoflex (Beckman Coulter, Inc.) and CytExpert software (version 2.1; Beckman Coulter, Inc.).

### Immunofluorescence assay

Cells cultured in confocal dishes were fixed in 4% paraformaldehyde for 15 min. Subsequently, the cells were washed with PBS three times and treated with 0.3% Triton for 15 min. The cells were then incubated in 5% nonfat milk for 1 h at room temperature. Anti-NF-κB p65 rabbit monoclonal antibodies (4764S, 1:1000; RRID: AB_10859369; Cell Signaling Technology, MA) were added to the dishes, and the cells were incubated overnight at 4 °C. PBS was added to one dish as a negative control. The secondary antibody DyLight 594 AffiniPure Goat anti-rabbit IgG (E032320-01; 1:100; EarthOx, USA) was then added, and plates were incubated in the dark for 1 h. Plates were washed with PBS three times, and nuclei were counterstained with 4′,6-diamidino-2-phenylindole for 5 min. Finally, the cells were imaged using a laser-scanning confocal microscope (Carl Zeiss, Oberkochen, Germany).

### RNAscope analysis

To amplify target-specific RNA signals but not background noise from nonspecific hybridization, we used RNAscope, a new technology developed for in situ hybridization. A series of target probes were designed to hybridize to LOC541472 (565981; RNAscope Probe-Hs-LOC541472; ACD), after which SFMSCs were cultured in confocal dishes and analyzed according to manufacturer’s instructions. Briefly, 20,000 SFMSCs were cultured in the confocal laser-scanning dishes. The culture medium was discarded after 24 h, the SFMSCs were then washed with PBS and fixed with 10% Neutral buffer formalin at room temperature for 30 min. The sample was then washed with PBS twice. The samples were then incubated with H_2_O_2_ for 10 min. After washing with PBS, the samples were then incubated with protease III for 10 min. The samples were washed with PBS times and then incubated with the probes for 2 h. Then incubated with AMP1 for 30 min, AMP2 for 30 min, HRP-C1 for 15 min, first TSA Plus fluorescent dye for 30 min, HRP blockers for 15 min, HRP-C2 for 15 min, second TSA Plus fluorescent dye for 30 min, HRP blockers for 15 min. Then 5 ng/ml DAPI (C1005, Beyotime, China) was added and incubated in the dark for 10 min. Finally, the samples were imaged using a laser-scanning confocal microscope (Carl Zeiss).

### Transfection with small interfering RNA (siRNA)

To conduct the interference of lncRNAs, Smart Silencer (designed by RiboBio, Guangzhou, China), containing three siRNA and three antisense oligonucleotides (ASO) targeting different sequences, was used to knockdown *LOC541472* and *RP3-467K16.4* in SFMSCs. The SFMSCs were inoculated in 6-well plates and transfected when the confluence reached approximately 80%. For each well, 10 μL lncRNA Smart Silencer or lncRNA Smart Silencer NC was diluted with 240 μL Opti-MEM. The two solutions were then mixed, incubated at room temperature for 15 min, and added to each 1750-μL culture medium-containing well. The final concentration of siRNA was 50 nM. After 48 h of culture, stimulation and sample collection were performed. The lncRNA Smart Silencer NC (cat. no. lnc3N0000001-1-5) was obtained from RiboBio; the *LOC541472* and *RP3-467K16.4* Smart Silencer sequences are listed in Table S2.

### Statistical analysis

Differential expression levels of lncRNAs and mRNAs were analyzed by Student’s *t* tests between two groups. Differences between more than two groups were analyzed by ANOVA and Tukey test. Fisher’s exact tests were used for GO and KEGG pathway analysis. Statistical analysis was conducted using SPSS 22.0 (IBM, Armonk, NY). Results with *P* values of less than 0.05 were considered significant.

## Results

### LncRNA and mRNA expression changes in SFMSCs after IL-1β stimulation

**Figure 1:**
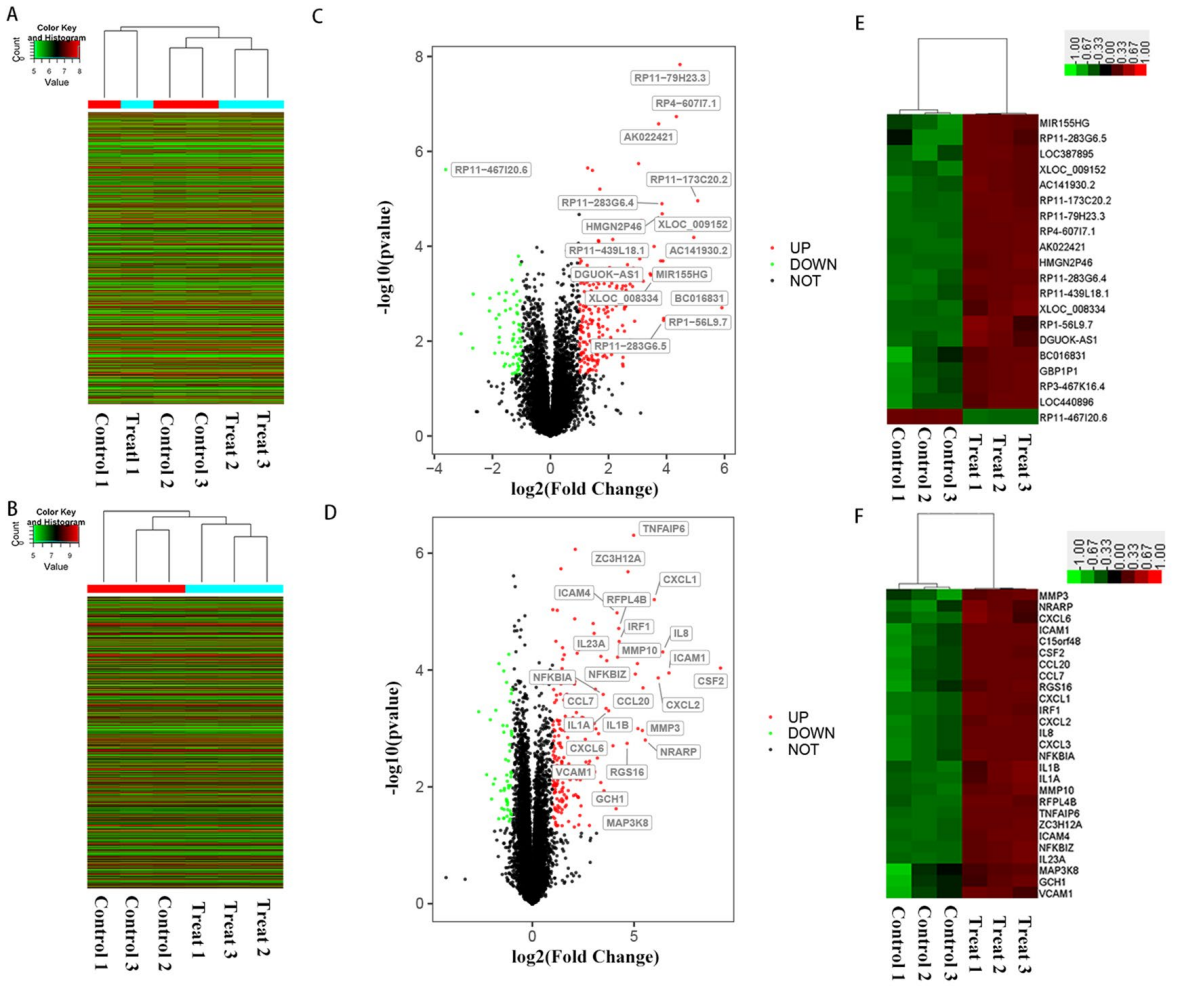
Volcano plots and heatmaps of changes in lncRNA and mRNA expression profiles in SFMSCs after IL-1β stimulation for 2 h. (**A**, **B**) Heatmap of differentially expressed lncRNAs (**A**) and mRNAs (**B**) showing a fold change > 1 in SFMSCs before and after IL-1β stimulation. (**C**, **D**) Volcano plots of lncRNA expression levels (**C**) and mRNA expression levels (**D**) before and after IL-1β stimulation. The red dots represent lncRNAs or mRNAs showing ≥ twofold upregulation, and the green dots represent downregulation (*P* < 0.05). Dots labeled with Seqname represent lncRNAs or mRNAs showing ≥ tenfold. (**E**, **F**) Heatmap of differentially expressed lncRNAs (**E**) and mRNAs (**F**) showing a fold changes ≥ 10 and *P* < 0.05 in SFMSCs before and after IL-1β stimulation. Genesymbol was used to label the genes. Volcano plots was performed using R Software created by R Core Team (2021). R: A language and environment for statistical computing. R Foundation for Statistical Computing, Vienna, Austria. https://www.R-project.org/. Heatmaps was performed using Gene Cluster 3.0 Software.

SFMSCs were isolated from synovial fluid samples which were donated by 3 temporomandibular joint osteoarthritis patients when they accepted arthrocentesis and lavage treatment. Differentially expressed LncRNAs and mRNAs in SFMSCs before and after treatment with IL-1β in vitro were detected using human Arraystar Human LncRNA Microarray V3.0. In total, we identified 14,352 lncRNAs varied slightly in expression after treatment with IL-1β in SFMSCs, including 8014 upregulated and 6338 downregulated transcripts. Of the 20,332 mRNAs varied slightly in expression, 8382 were upregulated and 11,950 were downregulated (fold change cut-off = 1.0) (Fig. 1A, B; Table S2). Among the target molecules, 201 mRNAs and 203 lncRNAs were upregulated, whereas 57 mRNAs and 61 lncRNAs were downregulated (fold changes ≥ 2, *P* < 0.05) (Fig. 1C, D; Table S4). There were 27 upregulated mRNAs, 19 upregulated lncRNAs, and only one downregulated lncRNA showing fold changes ≥ 10 in SFMSCs after IL-1β stimulation (*P* < 0.05) (Fig. 1C–F). Only 41 mRNAs and 24 lncRNAs were significantly upregulated, whereas 6 mRNAs and 1 lncRNAs were significantly downregulated among the target molecules (fold changes ≥ 2, *P* < 0.05, FDR < 0.05) (Table S5).

**Figure 2:**
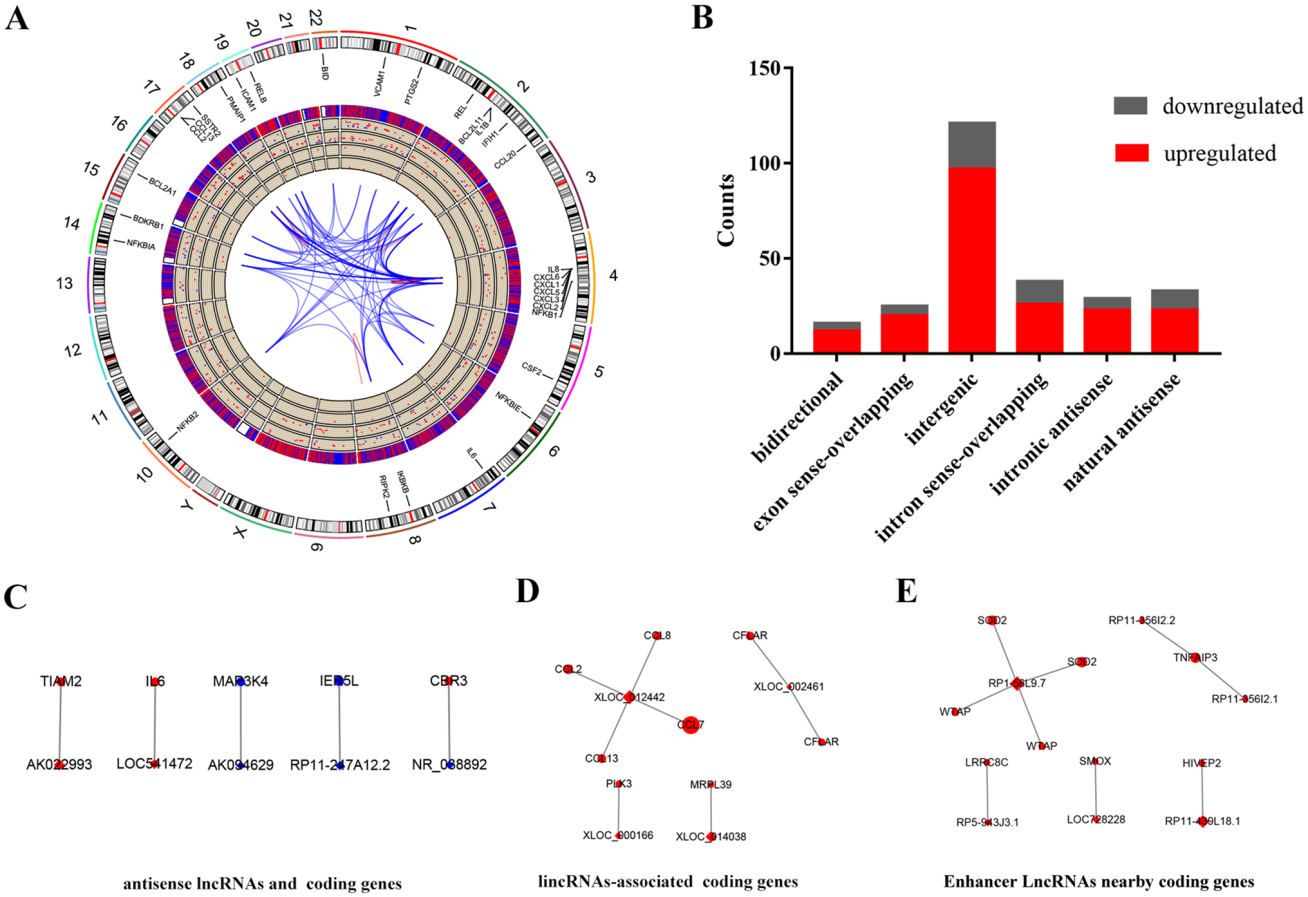
Identification of changes in lncRNA and mRNA expression profiles in SFMSCs after IL-1β stimulation for 2 h. (**A**) Circos plot showing differentially expressed lncRNAs and mRNAs on chromosomes. The first layer represents the chromosome map of the human genome. The top 30 K-score transcripts in the co-expression network of differentially expressed mRNAs are labeled in the second circle. Differentially expressed lncRNAs (blue) and mRNAs (red) with fold changes ≥ 1 are labeled in the third circle. The fourth and fifth circles represent the mean expression values of lncRNAs and mRNAs with fold changes ≥ 2 and *P* < 0.05. Red points represent upregulation, and blue points represent downregulation. The sixth and seventh circles represent mean expression values of lncRNAs and mRNAs with fold changes ≥ 10 and *P* < 0.05. Red points represent upregulation, and blue points represent downregulation. The innermost layer indicates the interaction network of the top 30 K-score transcripts labeled in the second circle. Red lines indicate linked transcripts in the same chromosome, and blue lines represent those on different chromosomes. Analysis was performed using R Software created by R Core Team (2020). R: A language and environment for statistical computing. R Foundation for Statistical Computing, Vienna, Austria. https://www.R-project.org/. (**B**) Categories of lncRNAs according to the genomic loci of their neighboring genes. (**C**) Differentially expressed antisense lncRNAs and their associated coding genes. (**D**) Differentially expressed lincRNA and associated coding gene pairs (distance < 300 kb). (**E**) Differentially expressed enhancer-like lncRNAs and their nearby coding genes (distance < 300 kb); both the lncRNA and its paired mRNA showed fold changes ≥ 2 and *P* < 0.05 among all differentially expressed lncRNAs and mRNAs. Diamonds represent lncRNAs, and circles represent mRNAs; red represents upregulation, and blue represents downregulation; different sizes represent the absolute value of the fold change. Protein names were used to label the genes in (**A**); Genesymbol was used to label the genes in (**C**–**E**). Images (**C**–**E**) were created using CytoScape 3.8.0 Software. exon sense-overlapping: the LncRNA's exon is overlapping a coding transcript exon on the same genomic strand; intronic: the LncRNA is overlapping the intron of a coding transcript on the same genomic strand; natural antisense: the LncRNA is transcribed from the antisense strand and overlapping with a coding transcript; non-overlapping antisense: the LncRNA is transcribed from the antisense strand without sharing overlapping exons; bidirectional: the LncRNA is oriented head to head to a coding transcript within 1000 bp; intergenic: there are no overlapping or bidirectional coding transcripts nearby the LncRNA.

The distribution of differentially expressed lncRNAs and mRNAs in chromosomes is shown in Fig. 2A. We identified 262 well-annotated lncRNAs with fold changes ≥ 2; these lncRNAs were classified into six categories: 16 were bidirectional, 25 were exon sense-overlapping, 121 were intergenic, 38 were intron sense-overlapping, 29 were intronic antisense, and 33 were natural antisense RNAs (Fig. 2B). We then picked out the antisense lncRNAs (corresponding to the intronic antisense lncRNAs and natural antisense lncRNAs in Fig. 2B), enhancer lncRNAs which were reported by U. A. Orom et al.^30^, and long intergenic noncoding RNAs (lincRNAs, corresponding to the intergenic lncRNAs in Fig. 2B) and their associated mRNAs. Those lncRNAs and their associated mRNAs with fold changes ≥ 2 and P < 0.05 are shown in Fig. 2C–E.

### GO enrichment and KEGG pathway analysis of differentially expressed mRNAs in SFMSCs after IL-1β stimulation

**Figure 3:**
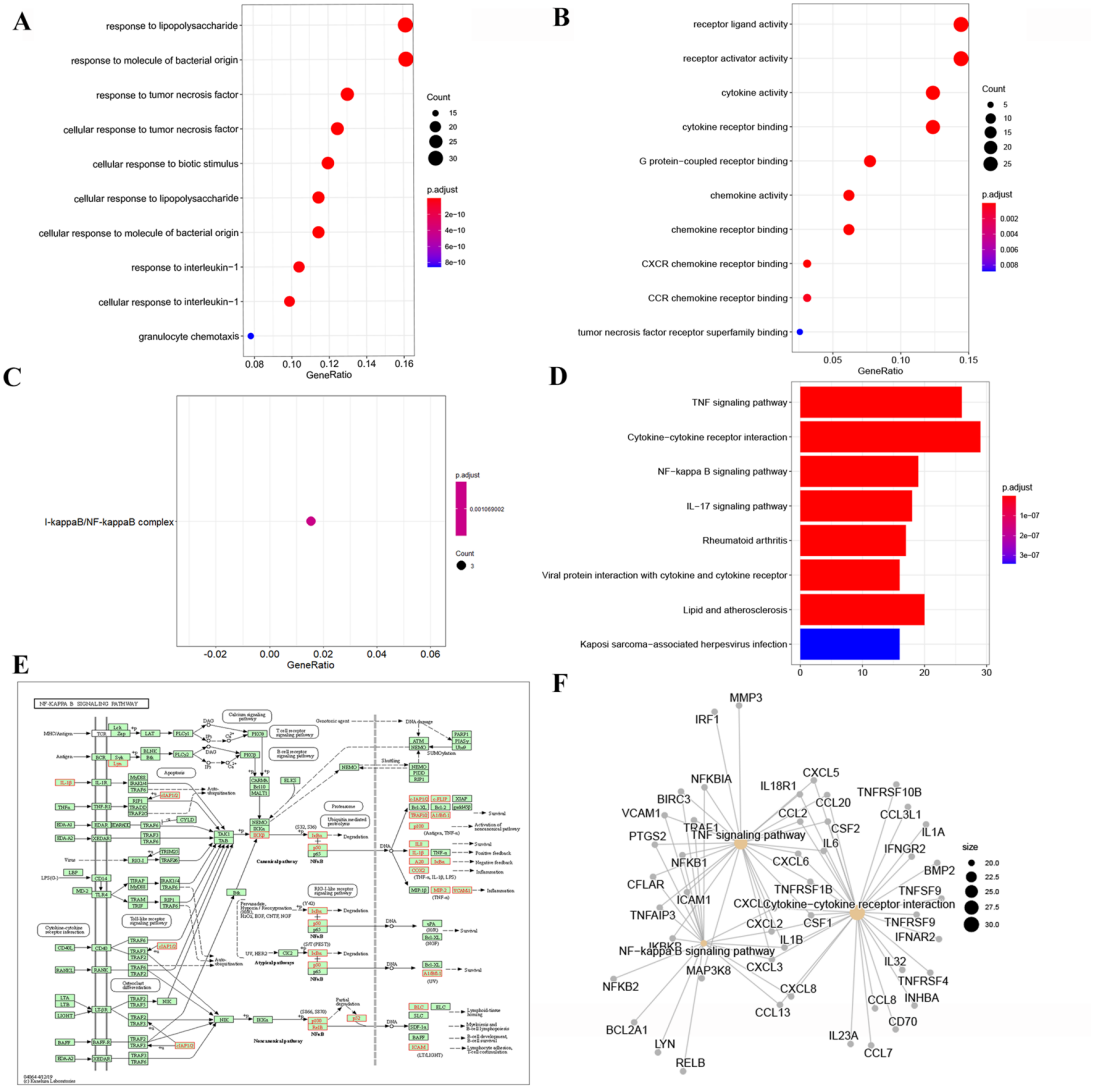
GO and KEGG pathway analysis of mRNAs in SFMSCs after IL-1β stimulation for 2 h. (**A**–**C**) GO annotations of upregulated mRNAs with the top 10 enriched genes/ratios of biological processes (**A**), molecular functions (**B**), and cellular components (**C**). *P* value cut off = 0.05, q-value cut off = 0.05. (**D**) KEGG pathway enrichment analysis of upregulated mRNAs with the top 10 most significant terms. (**E**) KEGG pathway annotations of NF-κB pathway. Genes in red are upregulated; those in black represent no significance. The KEGG map (map 04064) was developed by Kanehisa Laboratories (**F**) Genes involved in the top three significant pathways and interaction among the associated genes. Genesymbol was used to label the genes in image (**F**). Analysis was performed using R Software created by R Core Team (2021). R: A language and environment for statistical computing. R Foundation for Statistical Computing, Vienna, Austria. https://www.R-project.org/.

We subjected differentially expressed mRNAs showing fold changes ≥ 2 and *P* values < 0.05 to GO analysis. Significantly upregulated mRNAs were mainly enriched in GO terms belonging to biological processes and molecular functions, with one GO term belonging to cellular components (IκB/NF-κB complex; Fig. 3A–C). In contrast, the significantly downregulated mRNAs were only enriched in 3 GO terms belonging to the biological process category and 2 GO terms belonging to the molecular functions category (Fig. S1). According to KEGG pathway analysis, only the significantly upregulated mRNAs were enriched, whereas the downregulated mRNAs showed no pathway enrichment meeting the screening criteria (*P* < 0.05, q < 0.05). The significantly upregulated mRNAs were mainly enriched in TNF signaling pathway, cytokine-cytokine receptor interaction, and NF-κB signaling pathway according to the KEGG pathway analysis (Fig. 3D). We then visualized the upregulated mRNAs enriched in NF-κB signaling pathway (Fig. 3E). We also visualized the network constructed by genes belonging to the top three most significantly enriched pathways (Fig. 3F).

**Figure 4:**
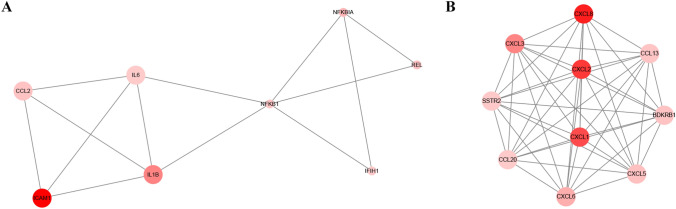
Protein–protein interaction network analysis using the STRING database. (**A**, **B**) Two core subnetworks of the network constructed by the top 30 K-score genes of Fig. S2. Different sizes represent K-score values. Different colors represent the absolute value of the fold change. Protein names were used to label the genes. Data was analyzed and created using Apps CentiScaPe 2.2 and MCODE in Cytoscape 3.8.0, Software.

To further investigate mRNA functions at the protein level, we analyzed the interactions of proteins encoded by differentially expressed mRNAs showing fold changes ≥ 2 in the STRING database. The network contained 98 nodes and 209 edges (Fig. S2A); disconnected nodes in the network were hidden. Next, K-scores were used to evaluate the importance of genes; the network constructed by the top 30 K-score genes was visualized (Fig. S2B) and found to consist of two core subnetworks (Fig. 4A, B).

### LncRNA/mRNA co-expression network in SFMSCs after IL-1β stimulation

**Figure 5:**
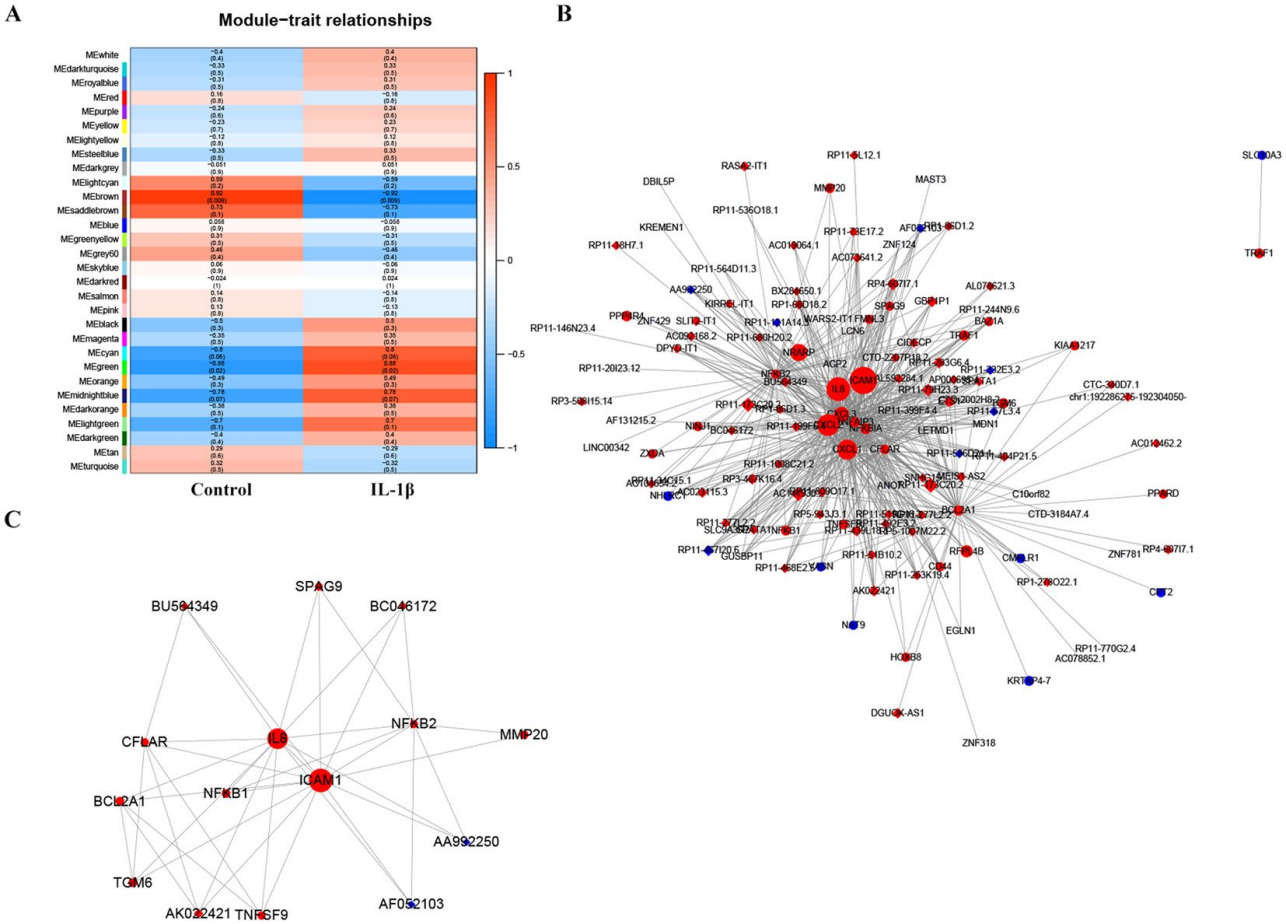
mRNAs and lncRNAs associated with the NF-κB signaling pathway co-expression networks constructed by weighted correlation network analysis. (**A**) The differentially expressed mRNAs and lncRNAs showing fold changes ≥ 1 were divided into 30 modules. The correlation indexes (upper) and *P* values (lower) between treatment and modules are listed in the heatmap. (**B**) Correlation network constructed using lncRNAs and mRNAs in MEgreen modules that are co-expressed with the upregulated genes enriched in NF-κB signaling pathway in Fig. 3F (weight ≥ 0.4). (**C**) Core subnetwork constructed from the network in (**B**). Circles, mRNAs; diamonds, lncRNAs. Different sizes represent the absolute value of the fold change. Red, upregulated; blue, downregulated. Seqname was used to label the genes. Image A was performed using R Software created by R Core Team (2020). R: A language and environment for statistical computing. R Foundation for Statistical Computing, Vienna, Austria. https://www.R-project.org/. Images (**B**) and (**C**) were created using Cytoscape 3.8.0, Software.

**Figure 6:**
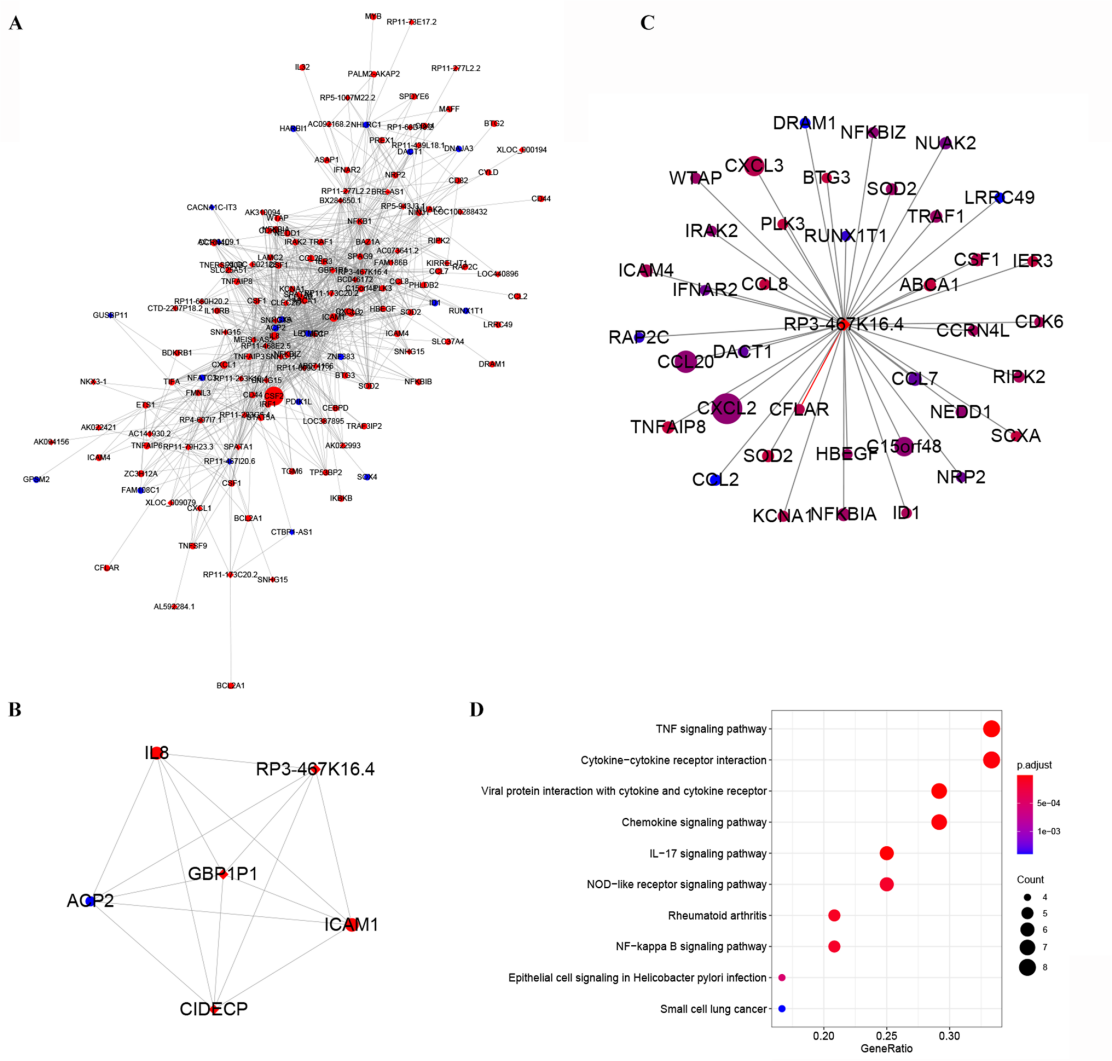
Co-expression networks of mRNAs and lncRNAs in SFMSCs after IL-1β treatment for 2 h constructed by weighted correlation network analysis. (**A**) Correlation network constructed using the mRNAs and lncRNAs in the most significant modules in Fig. 5A (MEgreen, top 1000 weight value). (**B**) Top K-score subnetwork. (**C**) mRNAs co-expressed with the top K-score lncRNA *RP3-467K16.4*. Weighted correlation network analysis of Pearson’s correlations was used to estimate associations between the lncRNA and mRNAs. (**D**) KEGG pathway enrichment analysis of the mRNAs co-expressed with *RP3-467K16.4*, with the top 10 enriched genes/ratios. Genes/ratios: number of differently expressed genes enriched in the pathway/number of all differently expressed genes associated with *RP3-467K16.4.* Circles, mRNAs; diamonds, lncRNAs. Different sizes represent the absolute value of the fold change. Red, upregulated; blue, downregulated. Seqname was used to label the genes. Images (**A**–**C**) were created using Cytoscape 3.8.0, Software. Image (**D**) was performed using R Software created by R Core Team (2020). R: A language and environment for statistical computing. R Foundation for Statistical Computing, Vienna, Austria. https://www.R-project.org/.

The functions of most lncRNAs have not yet been annotated. Therefore, we analyzed lncRNA functions by evaluating the co-expressed mRNAs. The co-expression profiles of differentially expressed lncRNAs and mRNAs showing fold changes > 1 were analyzed using weighted correlation network analysis. The genes were subdivided into 30 co-expression modules. We found that only MEgreen and MEcyan modules was obviously positive correlated with the treating factor (IL-1β, *P* value < 0.05, the genes in these 2 modules were listed in Table S6), and the MEgreen module was most significantly related to IL-1β stimulation (*r* = 0.88, *P* = 0.02; Fig. 5A). We then identified the upregulated mRNAs enriched in NF-κB signaling pathway (in Fig. 3F) and their co-expressed lncRNAs and mRNAs (weight value ≥ 0.4) in MEgreen module and then visualized the network constructed by these genes. The network contained 131 nodes and 572 edges (Fig. 5B). The most crucial subnetwork was subsequently constructed in Cytoscape and was found to contain 15 nodes and 39 edges. LncRNA *AK022421, BC046172, AF052103, BU564349* and *AA992250* were predicted to be associated with the NF-κB signaling pathway (Fig. 5C). The network constructed by genes showing the top 1000 weight values in the MEgreeen module was then visualized, and was found to contain 150 nodes and 1000 edges (Fig. 6A). The most crucial subnetwork was constructed by genes with the highest K-scores, including three lncRNAs *(RP3-467K16.4, GBP1P1, and CIDECP) and three mRNAs (ICAM1, IL-8, and ACP2; Fig. **6**B). RP3-467K16.4 sh*owed the highest K-score in the network shown in Fig. 6B. The 39 mRNAs in Fig. 6A showing co-expression with *RP3-467K16.4* were selected, and the network was reconstructed (Fig. 6C). According to KEGG pathway analysis, we found that *RP3-467K16.4* may be related to pathways contributing to inflammation activation, such as the tumor necrosis factor and NF-κB pathways (Fig. 6D).

### Co-expression relationship between RP3-467K16.4 and IL8 and between LOC541472 and IL6, which related to NF-κB pathway activation

**Figure 7:**
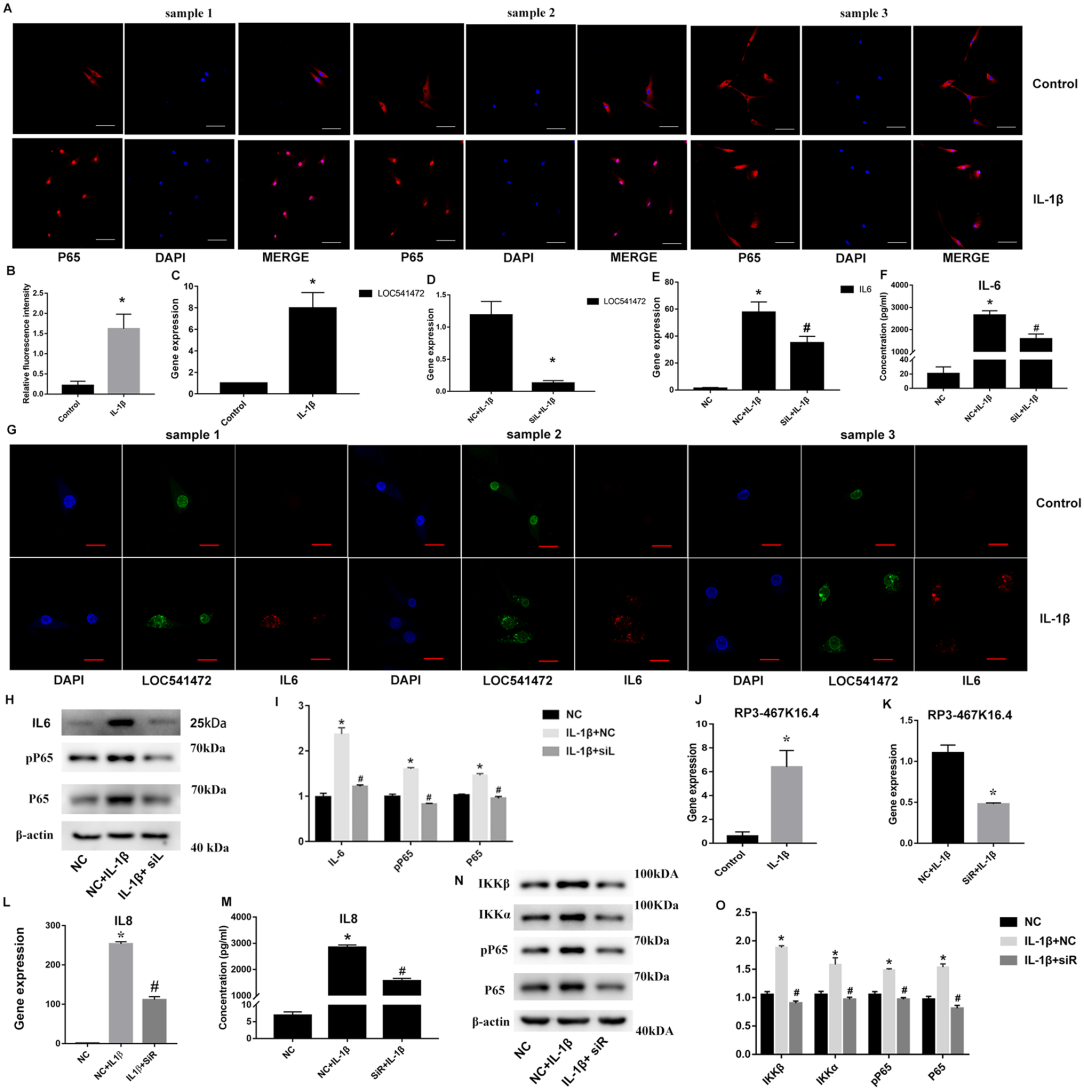
Validation of microarray data. (**A**) Cellular localization of p65 determined by immunofluorescence. (**B**) Semiquantitative analysis of immunofluorescence intensity of p65 in nucleus. (**C**) Expression of the lncRNA *LOC541472* in SFMSCs 2 h after stimulation with 10 ng/mL IL‐1β. (**D**) The efficiency of *LOC541472* knockdown using Smart Silencer was confirmed by RT‐qPCR. *IL‐6* gene expression was determined by RT‐qPCR (**E**), and IL-6 protein levels were determined by CBA assays (**F**). (**G**) Cellular localization and expression of *IL6* and *LOC541472*, as demonstrated by RNAscope assays. (**H**) Western blotting of IL6, phosphor-p65, p65, and β-actin. (**I**) Quantification of the blots shown in (**H**). (**J**) Gene expression of the lncRNA *RP3-467K16.4* after stimulation with 10 ng/mL IL‐1β for 2 h. (**K**) The efficiency of *RP3-467K16.4* knockdown using Smart Silencer was confirmed by RT‐qPCR. SFMSCs were transfected with Smart Silencer targeting *RP3-467K16.4* for 48 h and then incubated with 10 ng/mL IL‐1β for 2 h, and expression of the *IL8* gene was measured by RT‐qPCR (**L**), and IL8 protein levels were determined by CBA assays (**M**). (**N**) Western blotting of IKKβ, IKKα, phosphor-p65, p65, and β-actin. (**O**) Quantification of the blots shown in (**M**). *NC* negative control, *SiL* Smart Silencer targeting *LOC541472*, *SiR* Smart Silencer targeting *RP3-467K16.4*, *pP65* phospho p65. *P < 0.05 vs. negative control or control group. #*P* < 0.05 vs. NC + IL‐1β group. N = 3. Scale bars = 100 μm.

The NF-κB pathway was activated after IL-1β stimulation according to immunofluorescence results, consistent with our KEGG analysis results (Fig. 7A, B). To verify that the lncRNAs predicted in Fig. 2C–E were related to their associated mRNAs, we selected one lncRNA (*LOC541472*) and associated mRNA (*IL-6*) pair and analyzed their relationship in SFMSCs after IL-1β stimulation. We found that both *IL-6* and *LOC541472* were significantly upregulated after IL-1β stimulation. When we knocked down *LOC541472*, both gene and protein expression levels of *IL-6* and the *NF-κB* pathway were downregulated significantly (Fig. 7C–F, H, I). We detected strong *LOC541472* expression in the nucleus, but weak *IL-6* fluorescence in the control group. After IL-1β stimulation, both *LOC541472* and *IL-6* translocated into the cytoplasm. Interestingly, the localization of *LOC541472* and *IL-6* in SFMSCs was consistent with the RNAscope results (Fig. 7G). To verify the co-expression relationships predicted by the weighted group correlation network analysis, we selected the lncRNA with the highest K-score (*RP3-467K16.4*) and *IL-8* mRNA, which acts downstream of the NF-κB pathway, in the subnetwork shown in Fig. 6C. We found that both *RP3-467K16.4* and *IL-8* were significantly upregulated after IL-1β stimulation. Additionally, knockdown of *RP3-467K16.4* downregulated expression of the *IL-8* gene and components of the NF-κB pathway (Fig. 7J–O).

## Discussion

The etiology of temporomandibular joint disorders is complex and unclear. The inflammatory factor IL-1β is known as an important pathogenic factor for temporomandibular joint osteoarthritis^5^. One of the most important underlying pathogenic mechanisms involves the ability of IL-1β to affect joint injury repair by inhibiting the chondrogenic potential of MSCs; NF-κB pathway activation and upregulation of inflammatory cytokines, such as IL6, contribute to this process^4,6,31,32^. In addition, IL-1β can also affect the immune regulation function of MSCs in the joint. During the early phase of inflammation in the synovium, the resident macrophages secrete a large number of inflammatory factors, such as IL-1β, which shifts the synovial tissue from homeostasis toward catabolism, promoting joint destruction. The macrophages also promote CXCR3 + CD4 + Th1 cell migration into the synovium, which promotes polarization of resident macrophages toward M1 phenotype and enhances inflammatory factor secretion, creating a “positive inflammatory loop” that results in destruction progression^33,34^. The cartilage damage caused by the inflammatory immune response requires MSCs to spontaneously differentiate into chondrocytes for repair^35^. Meanwhile, the upregulated IL-1β enhances IL-1Ra secretion of MSCs, which blocks the “positive inflammatory loop” in an IL-1Ra-dependent manner, and induces macrophage polarization toward the M2 phenotype^34–37^. This is the theoretical basis of stem cell-based therapy for arthritis. In this study, we explored the effects of IL-1β on lncRNA and mRNA expression profiles of human SFMSCs, which may help us understand in more detail the regulation mechanism underlying this biological process.

The amount of differentially expressed lncRNAs and the lncRNA profile signature are likely affected by the exposure time of SFMSCs to inflammatory conditions. Here, we chose 2 h and not days because this exposure protocol was usually performed in previous study to explore the molecular function of lncRNAs^38^. In addition, we used the same method in our previous study to explored the molecular mechanism of how IL-1β affects biological behaviors of SFMSCs^6^. To further explore the underlying molecular mechanism, we used the same exposure method in this study.

In our previous study^6^, we also performed a dose–response experiment of IL-1β (0, 0.1, 1, and 10 ng/mL) and found inflammatory activation and SFMSCs concentration were positively correlated. In addition, the chondrogenic differentiation inhibition of SFMSCs by IL-1β was the strongest at 10 ng/mL. Therefore, we chose this concentration of IL-1β as it may be more helpful for us to explore the underlying mechanism of chondrogenic differentiation inhibition of SFMSCs by IL-1β.

To clarify the core subnetwork, signaling pathways, and core proteins involved in this biological process that SFMSCs in response to IL-1β, we selected differentially expressed mRNAs and performed GO and KEGG pathway analysis. We found that the differentially expressed mRNAs were mainly upregulated according to the results of volcano plots and heatmaps. Moreover, differentially expressed mRNAs showing significant enrichment in GO terms or KEGG pathways were upregulated, and few downregulated genes showed significant enrichment in GO terms or KEGG pathways, indicating that SFMSCs are activated by IL-1β stimulation. In addition, we analyzed the most significantly enriched GO terms in the biological process category and found that GO terms were mainly related to inflammatory activation, consistent with our in vitro analyses.

We also evaluated the distribution of the differentially expressed mRNAs with the top 30 K-scores and found that these mRNAs were enriched in chromosome 4, suggesting that IL-1β may have the most substantial effects on genes found on chromosome 4. Interestingly, based on analysis of the GO terms belonging to the cellular component category, we found that the differentially expressed mRNAs were only significantly enriched in the GO term IκB/NF-κB complex. In addition, from KEGG pathway analysis, we found that NF-κB pathway was one of the most significantly enriched pathways. Protein–protein interaction network analysis using the STRING database showed that some protein components of the NF-κB pathway were involved in the subnetwork with the highest K-score. Altogether, these results suggest that NF-κB pathway activation may be a core pathway in this biological process, consistent with our previous studies^6,39^.

LncRNAs play important regulatory roles in many biological processes. In this study, our microarray data identified many differentially expressed lncRNAs, indicating that lncRNAs are involved in IL-1β stimulation of SFMSCs. However, the functions of most lncRNAs remain unclear. Analyzing the relationships between lncRNAs and paired neighboring mRNAs is a common method for annotating lncRNAs. In this study, antisense lncRNAs, lincRNAs, or enhancer lncRNAs and paired mRNAs that showed differential expression were selected. We then analyzed one pair containing a downstream molecule from the NF-κB pathway (*LOC541472*, *IL-6*) and found that the lncRNA *LOC541472* was closely related to *IL-6*; both genes were significantly upregulated after IL-1β stimulation, and knockdown of *LOC541472* also downregulated *IL-6* expression and blocked NF-κB pathway activation, indicating that *LOC541472* may be closely involved in regulating IL-6 expression and the NF-κB pathway. RNAscope analysis indicated that *LOC541472* may be related to IL-6 expression and its translocation behavior from the nucleus into the cytoplasm. Further analyses of the mechanisms mediating the interactions between these molecules are required.

The functions of lncRNAs are commonly annotated by analyzing the functions of mRNAs that are co-expressed with the lncRNA. To provide some insights into the functions of lncRNAs in SFMSCs after IL-1β stimulation, we performed weighted correlation network analysis for the differentially expressed lncRNAs and mRNAs in SFMSCs after IL-1β stimulation. We visualized the highest K-score subnetwork, which contained three lncRNAs and three mRNA. Interestingly, this subnetwork contained one of the NF-κB pathway downstream molecules (*IL-8*). We then selected the lncRNA with the highest K-score (*RP3-467K16.4*) in this subnetwork and verified its function by analysis of GO terms and KEGG pathways associated with co-expressed mRNAs. We found significant enrichment of GO terms related to inflammatory activation and KEGG pathways related to the NF-κB pathway. Further evaluations showed that *RP3-467K16.4* and *IL-8* were both significantly upregulated after IL-1β stimulation, and knockdown of *RP3-467K16.4* downregulated *IL-8* expression and blocked NF-κB pathway activation. Additional studies are required to further assess the relationships among *RP3-467K16.4*, *IL-8*, and the NF-κB signaling pathway.

Both immunoregulatory function and cartilage differentiation potential of SFMSCs are important repair mechanisms for temporomandibular joint damage and joint homeostasis. In this study, we only investigated the lncRNA and mRNA profiles in untreated and IL-1β-inflamed SFMSCs. Our findings may be helpful for investigating the effect of lncRNAs and mRNAs on SFMSCs in this process. However, our study is limited in terms of investigating the immunoregulatory function of SFMSCs. As is known, MSCs can affect inflammation-related players by secreting cytokines, such as IL6 and IL8, for immunoregulation. In addition, secretion of exosomes also represents an important pathway for MSCs to participate in immune regulation. It is known that exosomes can be internalized by macrophages—the major inflammation-related player—and that the RNAs contained in exosomes can regulate macrophage polarization^40–42^. Therefore, further research should investigate the exosomal RNAs profile of IL-1β untreated/inflamed SFMSCs.

## Conclusion

There were significant differences in lncRNA and mRNA expression profiles after treatment of SFMSCs with IL-1β, and the NF-κB pathway may be one of the most crucial pathways in this biological process. We found a co-expression relationship between *RP3-467K16.4* and *IL8* and between *LOC541472* and *IL6*, which are related to NF-κB pathway activation. These results provide important insights into the alteration of lncRNA and mRNA profiles in IL-1β-treated SFMSCs as well as potential therapeutic targets.

## Supplementary Information

Supplementary Information.
