# Supplementary material for: Effects of interleukin 1β on long noncoding RNA and mRNA expression profiles of human synovial fluid derived mesenchymal stem cells
Source: Sci Rep. 2022 May 19;12:8432. doi: 10.1038/s41598-022-12190-9 (PMC9120201; doi:10.1038/s41598-022-12190-9)
Supplement: Supplementary file 1 — Supplementary Information. [file 41598_2022_12190_MOESM1_ESM.docx]

**Effects of interleukin-1β on long noncoding RNA and mRNA expression profiles of human synovial fluid-derived mesenchymal stem cells**

Yang-peng Sun^1^**^#^**, Yun-yang Lu^1#^, Jianyu Chen^1^, Jia-hao Bao^1^, Hong Zhang^1^, Jia-dong Sun^1*^, Wen-ting Liao^1*^

1 Guanghua School of Stomatology, Hospital of Stomatology, Sun Yat-sen University, Guangdong Provincial Key Laboratory of Stomatology, Guangzhou, Guangdong, People’s Republic of China

**#** The authors contributed equally to this work

*****Correspondence: Wen-ting Liao and Jia-dong Sun, Guanghua School of Stomatology, Hospital of Stomatology, Institute of Stomatology, Sun Yat-Sen University, 56 Ling Yuan Road West, Guangzhou, Guangdong 510055, P. R. China.

Phone: 008620-83870401

E-mail: liaowt9@mail.sysu.edu.cn and sunjd5@mail.sysu.edu.cn

Table S1. Primer sequences for PCR.

| Gene |  | Primer sequence |
| --- | --- | --- |
| GAPDH | F | GACAGTCAGCCGCATCTTCT |
|  | R | TTAAAAGCAGCCCTGGTGAC |
| IL-6 | F | ACTCACCTCTTCAGAACGAATTG |
|  | R | CCATCTTTGGAAGGTTCAGGTTG |
| IL‐8 | F | ACTGAGAGTGATTGAGAGTGGAC |
|  | R | AACCCTCTGCACCCAGTTTT |
| LOC541472 | F | CCAGCTTGACCCAATAAGAAAT |
|  | R | TAGAAGGAAACTACGGACGCA |
| *RP3-467K16.4* | F | CTGGGGCTGCCTTTTACTCC |
|  | R | ACCTCCATTCAGGGCTCATC |

F, forward; R, reverse.

Table S2. Target sequences of smart silencer.

| RNA | Target Sequence | |
| --- | --- | --- |
| lncRNA | | sequence |
| LOC541472 | TGGGCATTTACTCAAGTTT | |
|  | TCCTGTCTTAAGCAACGTA | |
|  | GAACCAAGTTCTCTTTCCT | |
|  | AATACTACCAGTCATCTGAG | |
|  | TTAAGCAACGTAGACACTCC | |
|  | TCTATGTAATGTGAGCTTGC | |
| *RP3-467K16.4* | AGTAAACACGATCTCCCTCC | |
|  | GAGACACCTTAAAATCCAGC | |
|  | GGGTGAAGTGATAGTAAGAA | |
|  | ATAGATTCTGGCAGAAAGA | |
|  | GCACAGAGATCGAAGTTCA | |
|  | CAAGACGATTGATCCTTTA | |

Table S3. mRNAs and lncRNAs showing fold change > 1 in SFMSC after IL-1β stimulation.

| **seqname** | **GeneSymbol** | **P-value** | **FDR** | **Fold Change** | **Regulation** | Gene type |
| --- | --- | --- | --- | --- | --- | --- |
| ENST00000006658 | SPATA20 | 0.772253631 | 0.975427771 | 1.0644373 | up | mRNA |
| ENST00000007264 | RPUSD1 | 0.948449063 | 0.996710991 | 1.0077516 | up | mRNA |
| ENST00000011619 | RANBP9 | 0.062787096 | 0.439458294 | 1.2722657 | up | mRNA |
| ENST00000011691 | SS18L2 | 0.60602007 | 0.94174007 | 1.0370858 | up | mRNA |
| ENST00000037243 | GABARAPL2 | 0.494110221 | 0.906783015 | 1.0161774 | up | mRNA |
| ENST00000083182 | APPBP2 | 0.423070245 | 0.873989281 | 1.0360345 | up | mRNA |
| ENST00000084798 | CA11 | 0.242418051 | 0.740622663 | 1.0934209 | up | mRNA |
| ENST00000156476 | KLK13 | 0.337173922 | 0.826950565 | 1.3378716 | up | mRNA |
| ENST00000161559 | CEACAM1 | 0.01625767 | 0.255922225 | 1.2204231 | up | mRNA |
| ENST00000165524 | PRLH | 0.848589346 | 0.987678166 | 1.0120153 | up | mRNA |
| ENST00000170168 | REXO1 | 0.144710505 | 0.605951408 | 1.0768091 | up | mRNA |
| ENST00000170564 | GPATCH1 | 0.980851682 | 0.998971807 | 1.0030237 | up | mRNA |
| ENST00000171111 | KEAP1 | 0.157472961 | 0.626563648 | 1.1221193 | up | mRNA |
| ENST00000175091 | LAPTM4A | 0.839501689 | 0.987032229 | 1.0339346 | up | mRNA |
| ENST00000181796 | FAM107B | 0.211921532 | 0.703329735 | 1.3112204 | up | mRNA |
| ENST00000188790 | FAP | 0.880065026 | 0.992197209 | 1.0090338 | up | mRNA |
| ENST00000194152 | PCDHB4 | 0.978863652 | 0.998661138 | 1.0125662 | up | mRNA |
| ENST00000202625 | TGM6 | 0.000409672 | 0.062231059 | 7.6556684 | up | mRNA |
| ENST00000203166 | HAUS5 | 0.207743655 | 0.698556218 | 1.3256161 | up | mRNA |
| ENST00000204604 | CHRD | 0.825886433 | 0.984306256 | 1.04064 | up | mRNA |
| ENST00000204726 | GOLGA3 | 0.538491705 | 0.923577434 | 1.0628243 | up | mRNA |
| ENST00000206765 | TGM1 | 0.56149565 | 0.928513767 | 1.1161199 | up | mRNA |
| ENST00000212015 | SIRT1 | 0.660384278 | 0.958354006 | 1.0248191 | up | mRNA |
| ENST00000215631 | GADD45B | 0.282230051 | 0.779872438 | 1.1226658 | up | mRNA |
| ENST00000216101 | RASL10A | 0.779616539 | 0.976938981 | 1.075377 | up | mRNA |
| ENST00000216733 | EFS | 0.879637942 | 0.992178089 | 1.0163165 | up | mRNA |
| ENST00000216879 | NSFL1C | 0.711744453 | 0.96592341 | 1.0409634 | up | mRNA |
| ENST00000217043 | R3HDML | 0.662385997 | 0.958354006 | 1.1682517 | up | mRNA |
| ENST00000217159 | SLCO4A1 | 0.548222282 | 0.92514258 | 1.0830441 | up | mRNA |
| ENST00000217270 | PROKR2 | 0.666340137 | 0.958354006 | 1.106534 | up | mRNA |
| ENST00000217289 | FERMT1 | 0.018678162 | 0.273264013 | 1.2003497 | up | mRNA |
| ENST00000218104 | ABCD1 | 0.473344672 | 0.899184677 | 1.2437542 | up | mRNA |
| ENST00000219162 | MT4 | 0.297679237 | 0.794023716 | 1.2607869 | up | mRNA |
| ENST00000219345 | PLA2G15 | 0.709168048 | 0.965462762 | 1.0424504 | up | mRNA |
| ENST00000219596 | MEFV | 0.502902315 | 0.910427376 | 1.2653387 | up | mRNA |
| ENST00000220420 | TGM5 | 0.499920549 | 0.909550837 | 1.1806728 | up | mRNA |
| ENST00000220676 | RP1 | 0.992605224 | 0.999798141 | 1.0029505 | up | mRNA |
| ENST00000221455 | CLASRP | 0.027034683 | 0.319947137 | 1.0408326 | up | mRNA |
| ENST00000221459 | LIN7B | 0.873086731 | 0.990580595 | 1.045568 | up | mRNA |
| ENST00000221466 | FCGRT | 0.441900956 | 0.88419144 | 1.0465852 | up | mRNA |
| ENST00000221486 | RNASEH2A | 0.980300907 | 0.998971807 | 1.0112182 | up | mRNA |
| ENST00000221496 | AMH | 0.101663918 | 0.529590777 | 1.3252069 | up | mRNA |
| ENST00000221538 | RSPH6A | 0.474978045 | 0.899688244 | 1.1895591 | up | mRNA |
| ENST00000221561 | AES | 0.001851694 | 0.105670067 | 1.979089 | up | mRNA |
| ENST00000221730 | EPHX3 | 0.176844988 | 0.656493025 | 1.0919303 | up | mRNA |
| ENST00000221742 | SLC1A6 | 0.758412476 | 0.972072638 | 1.0325876 | up | mRNA |
| ENST00000221891 | APLP1 | 0.865643415 | 0.989401142 | 1.0367172 | up | mRNA |
| ENST00000221954 | CEACAM4 | 0.007395187 | 0.190938402 | 1.5455427 | up | mRNA |
| ENST00000221978 | NKG7 | 0.03696523 | 0.359628653 | 1.2971804 | up | mRNA |
| ENST00000222005 | CDC37 | 0.125554968 | 0.578441772 | 1.0631929 | up | mRNA |
| ENST00000222115 | HAS1 | 0.945272006 | 0.996710991 | 1.0758297 | up | mRNA |
| ENST00000222139 | EPOR | 0.145010382 | 0.606427917 | 1.1979115 | up | mRNA |
| ENST00000222190 | WDR83OS | 0.555086387 | 0.926833616 | 1.0661164 | up | mRNA |
| ENST00000222266 | PSENEN | 0.133277084 | 0.589162633 | 1.0688614 | up | mRNA |
| ENST00000222275 | UPK1A | 0.752224255 | 0.970725371 | 1.0592517 | up | mRNA |
| ENST00000223167 | MYL10 | 0.599234758 | 0.940648177 | 1.2667118 | up | mRNA |
| ENST00000223357 | AEBP1 | 0.874012493 | 0.990793509 | 1.02293 | up | mRNA |
| ENST00000223862 | RLN1 | 0.160946786 | 0.632342039 | 1.6255683 | up | mRNA |
| ENST00000225275 | MPO | 0.984369093 | 0.998971807 | 1.0052634 | up | mRNA |
| ENST00000225430 | RPL19 | 0.022213983 | 0.293936579 | 1.0955461 | up | mRNA |
| ENST00000225504 | SUPT4H1 | 0.936996224 | 0.996710991 | 1.0078372 | up | mRNA |
| ENST00000225777 | SYNGR2 | 0.659302871 | 0.958354006 | 1.1357153 | up | mRNA |
| ENST00000225844 | CCL13 | 0.000534516 | 0.070570016 | 4.4812009 | up | mRNA |
| ENST00000226382 | PHOX2B | 0.509520844 | 0.911674633 | 1.1155586 | up | mRNA |
| ENST00000226574 | NFKB1 | 0.000306688 | 0.055508758 | 6.4189968 | up | mRNA |
| ENST00000228887 | GPRC5D | 0.932025847 | 0.996710991 | 1.0168836 | up | mRNA |
| ENST00000229379 | COX6A1 | 0.057896842 | 0.428369209 | 1.0832717 | up | mRNA |
| ENST00000233202 | SLC11A1 | 0.214298314 | 0.706979283 | 1.1283245 | up | mRNA |
| ENST00000233607 | APC2 | 0.146126034 | 0.608122555 | 1.1055604 | up | mRNA |
| ENST00000233712 | FAM176A | 0.052438519 | 0.412637354 | 1.8787538 | up | mRNA |
| ENST00000233840 | NEU2 | 0.797523285 | 0.980721189 | 1.0133678 | up | mRNA |
| ENST00000233997 | AZU1 | 0.18431731 | 0.66789156 | 1.2110852 | up | mRNA |
| ENST00000234420 | MSH6 | 0.303239025 | 0.798533134 | 1.0811432 | up | mRNA |
| ENST00000234961 | OPRD1 | 0.139271966 | 0.598648178 | 1.2582783 | up | mRNA |
| ENST00000236877 | SLC8A2 | 0.828945092 | 0.98552522 | 1.0389345 | up | mRNA |
| ENST00000237889 | NDUFB3 | 0.3997706 | 0.864196266 | 1.1175133 | up | mRNA |
| ENST00000239144 | HOXB8 | 0.001961963 | 0.107570744 | 2.0559017 | up | mRNA |
| ENST00000239347 | IFNA7 | 0.404172311 | 0.865617032 | 1.2723302 | up | mRNA |
| ENST00000239444 | PCDHB8 | 0.661060596 | 0.958354006 | 1.0669132 | up | mRNA |
| ENST00000239761 | MRC1 | 0.746439826 | 0.970432947 | 1.0656916 | up | mRNA |
| ENST00000240335 | TBX4 | 0.831275915 | 0.985710869 | 1.0276015 | up | mRNA |
| ENST00000240662 | KCNJ8 | 0.663806998 | 0.958354006 | 1.0920052 | up | mRNA |
| ENST00000241051 | DEPDC7 | 0.498805158 | 0.909361611 | 1.0640341 | up | mRNA |
| ENST00000241312 | CSMD2 | 0.094981254 | 0.516214612 | 1.8109655 | up | mRNA |
| ENST00000241453 | FLT3 | 0.639417653 | 0.953475593 | 1.132819 | up | mRNA |
| ENST00000242261 | TWIST1 | 0.137718592 | 0.596789644 | 1.1592522 | up | mRNA |
| ENST00000243300 | NRK | 0.456360143 | 0.891568474 | 1.2997445 | up | mRNA |
| ENST00000243578 | B9D2 | 0.421202162 | 0.872885778 | 1.1060701 | up | mRNA |
| ENST00000243673 | GPR83 | 0.386327729 | 0.855747763 | 1.0444949 | up | mRNA |
| ENST00000244051 | MOCS3 | 0.592803204 | 0.937820942 | 1.1511445 | up | mRNA |
| ENST00000244333 | LYPD3 | 0.522123694 | 0.916999248 | 1.146323 | up | mRNA |
| ENST00000244519 | BTN3A3 | 0.173332831 | 0.65166478 | 1.0970379 | up | mRNA |
| ENST00000245458 | RPS29 | 0.041025985 | 0.375308608 | 1.0626658 | up | mRNA |
| ENST00000245810 | PSPN | 0.616246913 | 0.945912508 | 1.0456982 | up | mRNA |
| ENST00000245817 | TNFSF9 | 8.58539E-07 | 0.008727906 | 4.3138843 | up | mRNA |
| ENST00000245912 | TNFSF14 | 0.255275715 | 0.75626779 | 1.0686428 | up | mRNA |
| ENST00000246163 | MAX | 0.376274948 | 0.849903337 | 1.0763831 | up | mRNA |
| ENST00000246551 | HCST | 0.05261467 | 0.413038171 | 1.2349479 | up | mRNA |
| ENST00000246554 | COX6B1 | 0.346994272 | 0.833567116 | 1.0740479 | up | mRNA |
| ENST00000246792 | RRAS | 0.981790779 | 0.998971807 | 1.0041003 | up | mRNA |
| ENST00000246802 | GLTSCR2 | 0.99628809 | 0.999872237 | 1.0015717 | up | mRNA |
| ENST00000247138 | SLC35A2 | 0.434310645 | 0.880931467 | 1.0777669 | up | mRNA |
| ENST00000248041 | CYP4F11 | 0.797762554 | 0.980749101 | 1.022756 | up | mRNA |
| ENST00000248058 | OR7A10 | 0.346276099 | 0.833163669 | 1.1339229 | up | mRNA |
| ENST00000248072 | OR7C2 | 0.081144387 | 0.486746344 | 1.2306877 | up | mRNA |
| ENST00000248076 | F2RL3 | 0.122918518 | 0.57525944 | 1.1193862 | up | mRNA |
| ENST00000248150 | GNG13 | 0.563378363 | 0.929041598 | 1.025584 | up | mRNA |
| ENST00000248342 | EIF3K | 0.395475347 | 0.861590143 | 1.0252616 | up | mRNA |
| ENST00000248451 | PNKD | 0.252258796 | 0.752621656 | 1.0931034 | up | mRNA |
| ENST00000248983 | RFPL2 | 0.494686067 | 0.907034836 | 1.1331743 | up | mRNA |
| ENST00000249007 | RFPL3 | 0.072603467 | 0.463622387 | 1.1742434 | up | mRNA |
| ENST00000249041 | GALR3 | 0.943835436 | 0.996710991 | 1.0142693 | up | mRNA |
| ENST00000249075 | LIF | 0.004605342 | 0.155233834 | 1.3653016 | up | mRNA |
| ENST00000250366 | KLK9 | 0.480429259 | 0.901538317 | 1.1517108 | up | mRNA |
| ENST00000250693 | ART1 | 0.98535194 | 0.998971807 | 1.0014452 | up | mRNA |
| ENST00000251481 | SULT1C2 | 0.393371552 | 0.860064163 | 1.1398899 | up | mRNA |
| ENST00000251642 | DHX58 | 0.048857794 | 0.40128453 | 1.4201882 | up | mRNA |
| ENST00000252250 | KRT6C | 0.509075998 | 0.911674633 | 1.3145431 | up | mRNA |
| ENST00000252252 | KRT6B | 0.736397615 | 0.968717225 | 1.030441 | up | mRNA |
| ENST00000252491 | APOC1 | 0.833952343 | 0.986475536 | 1.0257428 | up | mRNA |
| ENST00000252530 | FAM98C | 0.679172207 | 0.959969193 | 1.0361367 | up | mRNA |
| ENST00000252575 | NCAN | 0.403946819 | 0.865442226 | 1.1100967 | up | mRNA |
| ENST00000252595 | SLC27A1 | 0.115315274 | 0.560638489 | 1.1052632 | up | mRNA |
| ENST00000252599 | GLT25D1 | 0.091936007 | 0.511141068 | 1.3151063 | up | mRNA |
| ENST00000252818 | JUND | 0.571083473 | 0.933029103 | 1.1424204 | up | mRNA |
| ENST00000252945 | CYP2E1 | 0.973345196 | 0.998501532 | 1.0045829 | up | mRNA |
| ENST00000252999 | LAMA5 | 0.463283458 | 0.894989844 | 1.075233 | up | mRNA |
| ENST00000253109 | ANGPTL6 | 0.976650981 | 0.998501532 | 1.0042567 | up | mRNA |
| ENST00000253188 | SLC7A10 | 0.528724251 | 0.918883792 | 1.1049365 | up | mRNA |
| ENST00000253354 | BPIFB1 | 0.198096876 | 0.686763988 | 1.0861224 | up | mRNA |
| ENST00000253407 | C1QL1 | 0.89966185 | 0.994708735 | 1.0172015 | up | mRNA |
| ENST00000254323 | ZSWIM4 | 0.022469408 | 0.295886011 | 1.3716921 | up | mRNA |
| ENST00000254806 | WBP2 | 0.864997447 | 0.989401142 | 1.0147383 | up | mRNA |
| ENST00000255008 | SSTR4 | 0.61950599 | 0.947444797 | 1.0486739 | up | mRNA |
| ENST00000255039 | HAPLN2 | 0.352592673 | 0.835946719 | 1.1489434 | up | mRNA |
| ENST00000255082 | ACY3 | 0.584707976 | 0.93461341 | 1.0814998 | up | mRNA |
| ENST00000255136 | PABPC1L | 0.485915037 | 0.903321252 | 1.0732025 | up | mRNA |
| ENST00000255608 | BTBD2 | 0.73177825 | 0.967217713 | 1.0464979 | up | mRNA |
| ENST00000255977 | MKRN1 | 0.570693513 | 0.93296476 | 1.0395275 | up | mRNA |
| ENST00000256186 | MICALCL | 0.904531722 | 0.99508468 | 1.0060104 | up | mRNA |
| ENST00000256592 | TSHB | 0.215246531 | 0.709072014 | 1.2702608 | up | mRNA |
| ENST00000256707 | KIDINS220 | 0.449619542 | 0.888279993 | 1.2024721 | up | mRNA |
| ENST00000257209 | FHOD3 | 0.499546654 | 0.909460484 | 1.5167728 | up | mRNA |
| ENST00000257552 | MSI1 | 0.950237818 | 0.996710991 | 1.0059232 | up | mRNA |
| ENST00000257572 | HRK | 0.89117004 | 0.9937521 | 1.0094317 | up | mRNA |
| ENST00000257626 | PION | 0.320504354 | 0.814552264 | 1.3406197 | up | mRNA |
| ENST00000257863 | AMHR2 | 0.411917295 | 0.869001481 | 1.1109488 | up | mRNA |
| ENST00000258106 | EMX1 | 0.904327187 | 0.99508468 | 1.023829 | up | mRNA |
| ENST00000258443 | EDAR | 0.689522272 | 0.96108227 | 1.0526209 | up | mRNA |
| ENST00000258662 | NUDT15 | 0.926036213 | 0.995699344 | 1.0155889 | up | mRNA |
| ENST00000259631 | CCL27 | 0.8570718 | 0.988512246 | 1.0248382 | up | mRNA |
| ENST00000259633 | CD72 | 0.849844627 | 0.987678166 | 1.0633717 | up | mRNA |
| ENST00000259698 | FAM65B | 0.467955379 | 0.89714751 | 1.2137726 | up | mRNA |
| ENST00000259791 | HIST1H2AB | 0.540846066 | 0.923577434 | 1.2122075 | up | mRNA |
| ENST00000259939 | RNF144B | 0.0031498 | 0.132624561 | 2.9196926 | up | mRNA |
| ENST00000260130 | SDCBP | 0.196963368 | 0.685161614 | 1.4058146 | up | mRNA |
| ENST00000260228 | MMP20 | 0.001533482 | 0.100576613 | 6.071349 | up | mRNA |
| ENST00000260264 | POU2F3 | 0.267944907 | 0.768778917 | 1.1309259 | up | mRNA |
| ENST00000260983 | HECW2 | 0.272889178 | 0.772770327 | 1.1237434 | up | mRNA |
| ENST00000261304 | GALC | 0.313841253 | 0.808965923 | 1.0560416 | up | mRNA |
| ENST00000261336 | PZP | 0.780507005 | 0.976938981 | 1.0802801 | up | mRNA |
| ENST00000261509 | PALLD | 0.833221756 | 0.986434891 | 1.083584 | up | mRNA |
| ENST00000261862 | THSD4 | 0.95343654 | 0.996710991 | 1.0026909 | up | mRNA |
| ENST00000261897 | PRPF40B | 0.74407732 | 0.970330526 | 1.0569142 | up | mRNA |
| ENST00000261942 | FAF2 | 0.893961493 | 0.993978977 | 1.0090523 | up | mRNA |
| ENST00000262013 | SPAG9 | 0.000715588 | 0.078885568 | 1.9165083 | up | mRNA |
| ENST00000262017 | ITGB3 | 0.293179729 | 0.790364658 | 1.546295 | up | mRNA |
| ENST00000262085 | ZNF282 | 0.692047378 | 0.961451327 | 1.0320537 | up | mRNA |
| ENST00000262158 | SMAD7 | 0.012068225 | 0.222675671 | 1.2477729 | up | mRNA |
| ENST00000262262 | CD33 | 0.044461729 | 0.387482157 | 1.1508105 | up | mRNA |
| ENST00000262265 | PIH1D1 | 0.363779697 | 0.843238468 | 1.0342763 | up | mRNA |
| ENST00000262290 | LPO | 0.533716618 | 0.92058057 | 1.0476045 | up | mRNA |
| ENST00000262300 | PKMYT1 | 0.620160746 | 0.947444797 | 1.0849356 | up | mRNA |
| ENST00000262464 | FBN2 | 0.019896121 | 0.283521851 | 1.590079 | up | mRNA |
| ENST00000262765 | QRICH2 | 0.480685817 | 0.901538317 | 1.1931936 | up | mRNA |
| ENST00000262858 | MAMLD1 | 0.043934727 | 0.385691973 | 1.525582 | up | mRNA |
| ENST00000262946 | UQCR11 | 0.973110684 | 0.998501532 | 1.0020102 | up | mRNA |
| ENST00000262958 | GNA15 | 0.99682478 | 0.999872237 | 1.0007438 | up | mRNA |
| ENST00000262961 | ZFR2 | 0.692453724 | 0.961584652 | 1.0526029 | up | mRNA |
| ENST00000263398 | CD44 | 2.61133E-06 | 0.008848943 | 1.5743289 | up | mRNA |
| ENST00000263672 | SPCS2 | 0.317686634 | 0.811908136 | 1.137043 | up | mRNA |
| ENST00000263897 | USE1 | 0.913217891 | 0.995342392 | 1.0117106 | up | mRNA |
| ENST00000264009 | HSF4 | 0.285294997 | 0.781963593 | 1.1729904 | up | mRNA |
| ENST00000264183 | ARID4B | 0.162174709 | 0.634236412 | 1.1894902 | up | mRNA |
| ENST00000264265 | LXN | 0.995259115 | 0.999872237 | 1.0040889 | up | mRNA |
| ENST00000264360 | PCDH10 | 0.323136715 | 0.815948298 | 1.1460924 | up | mRNA |
| ENST00000264661 | KCNH4 | 0.281708176 | 0.779277638 | 1.0629384 | up | mRNA |
| ENST00000264734 | CLDN16 | 0.103304883 | 0.533095146 | 1.3741542 | up | mRNA |
| ENST00000264832 | ICAM1 | 0.000112317 | 0.038705735 | 104.8941687 | up | mRNA |
| ENST00000264903 | CNOT6L | 0.437207522 | 0.882234667 | 1.1258754 | up | mRNA |
| ENST00000265044 | SSR3 | 0.063447998 | 0.440687083 | 1.0567138 | up | mRNA |
| ENST00000265073 | SUB1 | 0.703336372 | 0.963625105 | 1.0279383 | up | mRNA |
| ENST00000265113 | SLC1A3 | 0.059252369 | 0.432586127 | 2.0914044 | up | mRNA |
| ENST00000265154 | ARHGEF38 | 0.652297517 | 0.956546204 | 1.123785 | up | mRNA |
| ENST00000265299 | FAM188B | 0.007663849 | 0.193257016 | 1.8165902 | up | mRNA |
| ENST00000265361 | SEMA3C | 0.202743064 | 0.692104094 | 1.1193692 | up | mRNA |
| ENST00000265437 | ST7 | 0.399591558 | 0.864032283 | 2.7854604 | up | mRNA |
| ENST00000265471 | B3GAT3 | 0.891006289 | 0.9937521 | 1.0157983 | up | mRNA |
| ENST00000265593 | CLCN2 | 0.579471041 | 0.933821303 | 1.0567201 | up | mRNA |
| ENST00000265922 | DBC1 | 0.610634288 | 0.943492388 | 1.0464802 | up | mRNA |
| ENST00000266594 | ANP32D | 0.839382535 | 0.987032229 | 1.0420011 | up | mRNA |
| ENST00000267273 | METTL21C | 0.846356472 | 0.987496831 | 1.0815913 | up | mRNA |
| ENST00000268695 | GALNS | 0.734751742 | 0.967777568 | 1.0186571 | up | mRNA |
| ENST00000268864 | RASL10B | 0.618503423 | 0.946956498 | 1.0556412 | up | mRNA |
| ENST00000269025 | LRRC46 | 0.994812651 | 0.999872237 | 1.0019446 | up | mRNA |
| ENST00000269216 | GATA6 | 0.113174309 | 0.55632728 | 2.2405943 | up | mRNA |
| ENST00000269383 | TRIM65 | 0.515900811 | 0.914429643 | 1.0870342 | up | mRNA |
| ENST00000269391 | RNF157 | 0.287902963 | 0.784247799 | 1.3708401 | up | mRNA |
| ENST00000269844 | PRDM15 | 0.064066354 | 0.442157878 | 1.5649435 | up | mRNA |
| ENST00000269878 | CIB3 | 0.591220028 | 0.937021663 | 1.055627 | up | mRNA |
| ENST00000270221 | EMP3 | 0.781303544 | 0.976938981 | 1.0249359 | up | mRNA |
| ENST00000270238 | LMTK3 | 0.508300969 | 0.911674633 | 1.0460118 | up | mRNA |
| ENST00000270631 | PTH2 | 0.242626955 | 0.740802161 | 1.1789307 | up | mRNA |
| ENST00000272462 | MALL | 0.882474201 | 0.992766819 | 1.0316053 | up | mRNA |
| ENST00000273062 | CTDSP1 | 0.91828315 | 0.995349793 | 1.0161038 | up | mRNA |
| ENST00000273353 | MYH15 | 0.398146958 | 0.863479888 | 1.2621076 | up | mRNA |
| ENST00000273550 | FTH1 | 0.663922705 | 0.958354006 | 1.1698831 | up | mRNA |
| ENST00000273905 | SLC10A6 | 0.741707675 | 0.970240145 | 1.1246211 | up | mRNA |
| ENST00000274065 | RPS3A | 0.051530436 | 0.41031217 | 1.0848957 | up | mRNA |
| ENST00000274276 | OSMR | 0.338423558 | 0.827403747 | 1.2969444 | up | mRNA |
| ENST00000274457 | FEM1C | 0.363990212 | 0.843353654 | 1.1131263 | up | mRNA |
| ENST00000274599 | ZNF300 | 0.630032049 | 0.950410442 | 1.033503 | up | mRNA |
| ENST00000274680 | FARS2 | 0.866658637 | 0.989481868 | 1.0137362 | up | mRNA |
| ENST00000274766 | KAAG1 | 0.828661965 | 0.985493382 | 1.0627692 | up | mRNA |
| ENST00000276101 | AWAT2 | 0.096059185 | 0.51782059 | 1.2633465 | up | mRNA |
| ENST00000276393 | ADRA1A | 0.28688371 | 0.782942227 | 1.167188 | up | mRNA |
| ENST00000276533 | GINS4 | 0.977135228 | 0.998501532 | 1.0202297 | up | mRNA |
| ENST00000277517 | IDI2 | 0.266430625 | 0.767064488 | 1.1496006 | up | mRNA |
| ENST00000277575 | USP6NL | 0.255424073 | 0.756286855 | 1.3307222 | up | mRNA |
| ENST00000278671 | LAMTOR1 | 0.998191268 | 0.999889342 | 1.0002629 | up | mRNA |
| ENST00000278765 | GGTLC1 | 0.047887758 | 0.398293541 | 1.3209928 | up | mRNA |
| ENST00000278795 | SMOX | 0.014741249 | 0.24380986 | 2.0831493 | up | mRNA |
| ENST00000278829 | FADS3 | 0.531929244 | 0.920228311 | 1.1106008 | up | mRNA |
| ENST00000278833 | ROM1 | 0.957970431 | 0.996933183 | 1.0281838 | up | mRNA |
| ENST00000279249 | CDC42EP2 | 0.554990024 | 0.926833616 | 1.0925533 | up | mRNA |
| ENST00000280357 | IL18 | 0.585661726 | 0.934888451 | 1.2014966 | up | mRNA |
| ENST00000280571 | RILPL2 | 0.140656785 | 0.600786526 | 1.1199329 | up | mRNA |
| ENST00000281038 | NARS2 | 0.47648589 | 0.900415348 | 1.0626879 | up | mRNA |
| ENST00000281282 | CGNL1 | 0.00791063 | 0.194808105 | 1.2414173 | up | mRNA |
| ENST00000282007 | ZC3H13 | 0.046622513 | 0.39300536 | 1.2229748 | up | mRNA |
| ENST00000282141 | CRYGC | 0.025478677 | 0.3131998 | 2.2560336 | up | mRNA |
| ENST00000282701 | BMP3 | 0.85376535 | 0.987808405 | 1.0324434 | up | mRNA |
| ENST00000283206 | TMEM87B | 0.872950304 | 0.990534615 | 1.0458045 | up | mRNA |
| ENST00000283365 | DTNA | 0.950644415 | 0.996710991 | 1.0083777 | up | mRNA |
| ENST00000284061 | DGKE | 0.505439815 | 0.910772598 | 1.1764405 | up | mRNA |
| ENST00000284110 | HS3ST3A1 | 0.697998142 | 0.962410025 | 1.0301228 | up | mRNA |
| ENST00000284425 | ABCA6 | 0.835136281 | 0.986575022 | 1.0270848 | up | mRNA |
| ENST00000284881 | C21orf91 | 0.865329501 | 0.989401142 | 1.0372399 | up | mRNA |
| ENST00000285013 | SLFN13 | 0.682060332 | 0.960111041 | 1.0463648 | up | mRNA |
| ENST00000285600 | OR4K1 | 0.557700239 | 0.926833616 | 1.2066661 | up | mRNA |
| ENST00000285949 | CYP26C1 | 0.616898672 | 0.945960598 | 1.0585149 | up | mRNA |
| ENST00000286201 | FZD7 | 0.101246686 | 0.528999035 | 1.7631501 | up | mRNA |
| ENST00000286657 | ADAMTS3 | 0.489058964 | 0.904534417 | 1.0669248 | up | mRNA |
| ENST00000286808 | CLDN17 | 0.306974286 | 0.801927429 | 1.1304933 | up | mRNA |
| ENST00000288022 | PDF | 0.883402282 | 0.992766819 | 1.006083 | up | mRNA |
| ENST00000288065 | ARMC12 | 0.464085627 | 0.894989844 | 1.051189 | up | mRNA |
| ENST00000288111 | DHRS1 | 0.284355421 | 0.78179061 | 1.1000433 | up | mRNA |
| ENST00000288710 | CCDC164 | 0.646022575 | 0.95550188 | 1.1581575 | up | mRNA |
| ENST00000289119 | ABHD3 | 0.998709067 | 0.999889342 | 1.0006518 | up | mRNA |
| ENST00000289272 | PCDHA13 | 0.691934085 | 0.961451327 | 1.1852668 | up | mRNA |
| ENST00000289361 | BTN3A1 | 0.728213305 | 0.966191059 | 1.0425926 | up | mRNA |
| ENST00000289388 | C2orf62 | 0.647400111 | 0.95550188 | 1.0813735 | up | mRNA |
| ENST00000289893 | MACF1 | 0.670400772 | 0.959531882 | 1.0500101 | up | mRNA |
| ENST00000290101 | RAP1GAP | 0.129880998 | 0.583907255 | 1.33825 | up | mRNA |
| ENST00000290219 | IFNGR2 | 0.004803484 | 0.158085585 | 3.0821847 | up | mRNA |
| ENST00000290310 | KCNE2 | 0.970539143 | 0.998245244 | 1.0041988 | up | mRNA |
| ENST00000290417 | GFRA4 | 0.830402621 | 0.985549562 | 1.012595 | up | mRNA |
| ENST00000290691 | RGL4 | 0.73117873 | 0.966852624 | 1.0327594 | up | mRNA |
| ENST00000290705 | MT1A | 0.009998483 | 0.207620683 | 1.5180225 | up | mRNA |
| ENST00000290953 | AGRP | 0.817087282 | 0.981692737 | 1.0228283 | up | mRNA |
| ENST00000291231 | OR3A3 | 0.008992126 | 0.202329498 | 1.5368041 | up | mRNA |
| ENST00000291560 | HSF2BP | 0.174282396 | 0.652872869 | 1.4119328 | up | mRNA |
| ENST00000291598 | ZNF583 | 0.181203445 | 0.664543371 | 1.1959912 | up | mRNA |
| ENST00000291744 | FCN2 | 0.230300653 | 0.727521159 | 1.2508765 | up | mRNA |
| ENST00000291860 | KIR3DL3 | 0.331084141 | 0.821853462 | 1.2026895 | up | mRNA |
| ENST00000291890 | NCR1 | 0.138507222 | 0.597943156 | 1.4304305 | up | mRNA |
| ENST00000291971 | NLRP8 | 0.703118913 | 0.963625105 | 1.0639771 | up | mRNA |
| ENST00000292169 | S100A1 | 0.987870278 | 0.999140676 | 1.0028469 | up | mRNA |
| ENST00000292377 | GPC2 | 0.109337988 | 0.547509603 | 1.2391748 | up | mRNA |
| ENST00000292513 | PTGER1 | 0.701959679 | 0.963247543 | 1.0583581 | up | mRNA |
| ENST00000292535 | CUX1 | 0.281330893 | 0.779126326 | 1.2563498 | up | mRNA |
| ENST00000292599 | MAML1 | 0.353157156 | 0.836266379 | 1.0682703 | up | mRNA |
| ENST00000292894 | THAP8 | 0.457851401 | 0.892566841 | 1.6040252 | up | mRNA |
| ENST00000292907 | COX7A1 | 0.685123803 | 0.960111041 | 1.1779623 | up | mRNA |
| ENST00000293396 | CD300LG | 0.902936403 | 0.99508468 | 1.0116215 | up | mRNA |
| ENST00000293441 | SHANK1 | 0.121132208 | 0.572400147 | 1.1956964 | up | mRNA |
| ENST00000293636 | CELA1 | 0.980074003 | 0.998971807 | 1.0075695 | up | mRNA |
| ENST00000293745 | KRT72 | 0.299908576 | 0.795797471 | 1.1172958 | up | mRNA |
| ENST00000293851 | PRSS33 | 0.363956715 | 0.843353654 | 1.0527757 | up | mRNA |
| ENST00000293973 | NTN3 | 0.58087521 | 0.933994051 | 1.3867496 | up | mRNA |
| ENST00000294066 | MAP4K2 | 0.127891043 | 0.581220503 | 1.2030816 | up | mRNA |
| ENST00000294256 | SYVN1 | 0.549058067 | 0.925428884 | 1.0858215 | up | mRNA |
| ENST00000294383 | USP24 | 0.004902332 | 0.158753988 | 1.413719 | up | mRNA |
| ENST00000294543 | TMCO4 | 0.129889839 | 0.583907255 | 1.1456526 | up | mRNA |
| ENST00000295226 | CCDC140 | 0.333255228 | 0.82335721 | 1.4064333 | up | mRNA |
| ENST00000295269 | SLC9A4 | 0.012716298 | 0.230112444 | 2.1018593 | up | mRNA |
| ENST00000295379 | BMP10 | 0.358701261 | 0.840607888 | 1.2982181 | up | mRNA |
| ENST00000295400 | TGFA | 0.820182386 | 0.982556462 | 1.1397793 | up | mRNA |
| ENST00000295718 | PTPRN | 0.161637069 | 0.633561373 | 1.1987922 | up | mRNA |
| ENST00000295911 | CLRN1 | 0.079406885 | 0.482639336 | 1.5504701 | up | mRNA |
| ENST00000295952 | SLMAP | 0.806814234 | 0.98118927 | 1.0278307 | up | mRNA |
| ENST00000296315 | ARHGEF3 | 0.00111266 | 0.087009974 | 1.1341418 | up | mRNA |
| ENST00000296387 | CLDN19 | 0.594858537 | 0.938655001 | 1.0854766 | up | mRNA |
| ENST00000296422 | SLC9B1 | 0.18074703 | 0.663780602 | 1.1520745 | up | mRNA |
| ENST00000296440 | PLXNB1 | 0.018082873 | 0.269173581 | 1.1182724 | up | mRNA |
| ENST00000296555 | FBXW7 | 0.551332479 | 0.926149092 | 1.0548582 | up | mRNA |
| ENST00000296585 | ITGA2 | 0.140101628 | 0.600174217 | 1.1429193 | up | mRNA |
| ENST00000297205 | STEAP1 | 0.933115832 | 0.996710991 | 1.0186509 | up | mRNA |
| ENST00000297268 | COL1A2 | 0.366485303 | 0.845181673 | 1.073456 | up | mRNA |
| ENST00000297313 | RGS20 | 0.442944341 | 0.884304813 | 1.0696173 | up | mRNA |
| ENST00000297375 | EN2 | 0.762856861 | 0.97349057 | 1.0723371 | up | mRNA |
| ENST00000298171 | TSHR | 0.567271504 | 0.931514776 | 1.1782683 | up | mRNA |
| ENST00000298690 | RNASE7 | 0.07006015 | 0.458026678 | 1.1821412 | up | mRNA |
| ENST00000298815 | ARHGAP42 | 0.064439442 | 0.443678541 | 1.5914502 | up | mRNA |
| ENST00000299299 | PCBD1 | 0.230041298 | 0.72737466 | 1.2408168 | up | mRNA |
| ENST00000299565 | CHRNA5 | 0.640242399 | 0.953891706 | 1.0343282 | up | mRNA |
| ENST00000299709 | SLC38A8 | 0.596830922 | 0.939608975 | 1.1186463 | up | mRNA |
| ENST00000299752 | CDH16 | 0.071331129 | 0.461586415 | 1.1683347 | up | mRNA |
| ENST00000299766 | MC4R | 0.268721425 | 0.76931062 | 1.1712945 | up | mRNA |
| ENST00000300005 | ERI2 | 0.059280016 | 0.432586127 | 1.3073754 | up | mRNA |
| ENST00000300181 | TSC22D4 | 0.64091866 | 0.953894897 | 1.0313365 | up | mRNA |
| ENST00000300190 | MS4A5 | 0.776179112 | 0.976080759 | 1.0370333 | up | mRNA |
| ENST00000300584 | TBC1D2B | 0.154978483 | 0.622362731 | 1.314458 | up | mRNA |
| ENST00000300933 | TPM4 | 0.897346162 | 0.994222243 | 1.00757 | up | mRNA |
| ENST00000300960 | IZUMO4 | 0.896300745 | 0.994194586 | 1.0094589 | up | mRNA |
| ENST00000301037 | SGK494 | 0.66595046 | 0.958354006 | 1.0625159 | up | mRNA |
| ENST00000301046 | LALBA | 0.909927423 | 0.995342392 | 1.0154425 | up | mRNA |
| ENST00000301146 | CYP2A7 | 0.641422669 | 0.954299614 | 1.0496097 | up | mRNA |
| ENST00000301175 | PRODH2 | 0.581250253 | 0.934005566 | 1.0957417 | up | mRNA |
| ENST00000301246 | C19orf33 | 0.240316203 | 0.738437735 | 1.0865293 | up | mRNA |
| ENST00000301280 | CHAF1A | 0.875179244 | 0.991094934 | 1.0505897 | up | mRNA |
| ENST00000301335 | SLC43A2 | 0.046297924 | 0.392057225 | 1.5978099 | up | mRNA |
| ENST00000301395 | GGT6 | 0.572810596 | 0.93352894 | 1.0791256 | up | mRNA |
| ENST00000301411 | NTF4 | 0.817151642 | 0.981692737 | 1.063655 | up | mRNA |
| ENST00000301421 | KLK15 | 0.39775909 | 0.863112281 | 1.0498823 | up | mRNA |
| ENST00000301452 | ACER1 | 0.416781916 | 0.870736736 | 1.0875758 | up | mRNA |
| ENST00000301454 | SLC25A23 | 0.642304779 | 0.954332328 | 1.0167959 | up | mRNA |
| ENST00000301455 | ANGPTL4 | 0.208152582 | 0.698793461 | 1.4676551 | up | mRNA |
| ENST00000301480 | ZNF560 | 0.058909121 | 0.431383592 | 1.3641335 | up | mRNA |
| ENST00000301529 | OR8J3 | 0.46723086 | 0.896646168 | 1.4513425 | up | mRNA |
| ENST00000301607 | EVPL | 0.123975685 | 0.577766088 | 1.132485 | up | mRNA |
| ENST00000301659 | GSDMA | 0.111624537 | 0.553144062 | 1.7694543 | up | mRNA |
| ENST00000301698 | PRR25 | 0.803714341 | 0.98118927 | 1.0459466 | up | mRNA |
| ENST00000301810 | ACAA1 | 0.895391693 | 0.993978977 | 1.0192221 | up | mRNA |
| ENST00000301894 | NRXN2 | 0.853088943 | 0.987678166 | 1.0217985 | up | mRNA |
| ENST00000302006 | IRX1 | 0.051667791 | 0.41031217 | 1.2069996 | up | mRNA |
| ENST00000303061 | C17orf65 | 0.207934246 | 0.698681059 | 1.085866 | up | mRNA |
| ENST00000303177 | HMP19 | 0.476809797 | 0.900429473 | 1.0729515 | up | mRNA |
| ENST00000303324 | OR2B2 | 0.730688033 | 0.966329458 | 1.0597566 | up | mRNA |
| ENST00000303532 | OR10G3 | 0.717026575 | 0.966191059 | 1.0913931 | up | mRNA |
| ENST00000303657 | RFX2 | 0.01363795 | 0.235988767 | 4.3630222 | up | mRNA |
| ENST00000303806 | HIST1H2BO | 0.627695178 | 0.949575771 | 1.1631918 | up | mRNA |
| ENST00000304094 | OR1A1 | 0.190085488 | 0.676140334 | 1.5058984 | up | mRNA |
| ENST00000304177 | C15orf40 | 0.750265901 | 0.970725371 | 1.0330783 | up | mRNA |
| ENST00000304218 | HIST1H1E | 0.989987168 | 0.999140676 | 1.007839 | up | mRNA |
| ENST00000304312 | ATP5I | 0.734726043 | 0.967777568 | 1.0097921 | up | mRNA |
| ENST00000304338 | PPP4R4 | 0.003212187 | 0.133466208 | 9.1441559 | up | mRNA |
| ENST00000304430 | NUDT6 | 0.555270822 | 0.926833616 | 1.0252007 | up | mRNA |
| ENST00000304519 | C8orf74 | 0.253796499 | 0.753972882 | 1.4055419 | up | mRNA |
| ENST00000304620 | KRT78 | 0.22016487 | 0.715236067 | 1.2317409 | up | mRNA |
| ENST00000304658 | XPO6 | 0.318757264 | 0.812850403 | 1.0523441 | up | mRNA |
| ENST00000304743 | PCNX | 0.616606105 | 0.945912508 | 1.0627336 | up | mRNA |
| ENST00000304749 | CST1 | 0.931873135 | 0.996710991 | 1.0578332 | up | mRNA |
| ENST00000304863 | UQCRFS1 | 0.568207015 | 0.931826506 | 1.0551474 | up | mRNA |
| ENST00000304915 | SMR3B | 0.822032449 | 0.982983098 | 1.0340656 | up | mRNA |
| ENST00000304926 | ZNF434 | 0.771150095 | 0.975347132 | 1.0623968 | up | mRNA |
| ENST00000305479 | WFDC13 | 0.901851887 | 0.994906068 | 1.0154631 | up | mRNA |
| ENST00000305536 | RBMX2 | 0.294868837 | 0.791301949 | 1.0825957 | up | mRNA |
| ENST00000305620 | KRT74 | 0.509474166 | 0.911674633 | 1.0484096 | up | mRNA |
| ENST00000305628 | SIGLEC7 | 0.023256524 | 0.300022844 | 1.0789714 | up | mRNA |
| ENST00000305861 | B3GALT1 | 0.836394639 | 0.986575022 | 1.066485 | up | mRNA |
| ENST00000305899 | OR10H2 | 0.840104289 | 0.987068842 | 1.0502042 | up | mRNA |
| ENST00000305904 | DYNLRB2 | 0.604403651 | 0.94174007 | 1.1403667 | up | mRNA |
| ENST00000305958 | SMOX | 0.195793816 | 0.683572955 | 7.1780966 | up | mRNA |
| ENST00000306005 | BDKRB2 | 0.008427569 | 0.198394583 | 1.5933071 | up | mRNA |
| ENST00000306030 | SNX29 | 0.431793 | 0.880121832 | 1.924989 | up | mRNA |
| ENST00000306176 | ZPLD1 | 0.568878277 | 0.93232103 | 1.2413889 | up | mRNA |
| ENST00000306390 | LRG1 | 0.703959754 | 0.963625105 | 1.1603732 | up | mRNA |
| ENST00000306402 | SORBS1 | 0.68316543 | 0.960111041 | 1.0694064 | up | mRNA |
| ENST00000306593 | PCDHGC4 | 0.752951406 | 0.970725371 | 1.0501913 | up | mRNA |
| ENST00000307394 | EME2 | 0.628191568 | 0.949690755 | 1.0636473 | up | mRNA |
| ENST00000307407 | IL8 | 4.90933E-05 | 0.02935779 | 85.0422778 | up | mRNA |
| ENST00000307493 | ACSM1 | 0.58879615 | 0.935839874 | 1.1847597 | up | mRNA |
| ENST00000308027 | SLC16A13 | 0.890561509 | 0.9937521 | 1.0352509 | up | mRNA |
| ENST00000308105 | C1orf100 | 0.014823221 | 0.244234794 | 1.248137 | up | mRNA |
| ENST00000308394 | CASP3 | 0.148553494 | 0.611786435 | 1.5226233 | up | mRNA |
| ENST00000308508 | CSPG4 | 0.446041913 | 0.886278379 | 1.1343745 | up | mRNA |
| ENST00000308946 | MYEOV | 0.757206734 | 0.971960783 | 1.0668266 | up | mRNA |
| ENST00000309166 | NRIP3 | 0.014143721 | 0.239442239 | 1.3054631 | up | mRNA |
| ENST00000309324 | C19orf70 | 0.549931727 | 0.925533435 | 1.0282458 | up | mRNA |
| ENST00000309558 | SUSD5 | 0.578325579 | 0.933821303 | 1.0820781 | up | mRNA |
| ENST00000309733 | C4orf32 | 0.548056714 | 0.92514258 | 1.0506304 | up | mRNA |
| ENST00000309989 | DAB2IP | 0.036516535 | 0.358673526 | 1.1055311 | up | mRNA |
| ENST00000310015 | SP3 | 0.981165555 | 0.998971807 | 1.0025776 | up | mRNA |
| ENST00000310169 | CHRNA9 | 0.071176498 | 0.46104346 | 1.5614066 | up | mRNA |
| ENST00000310331 | EIF4EBP3 | 0.578788091 | 0.933821303 | 1.0644995 | up | mRNA |
| ENST00000310355 | C16orf91 | 0.589330036 | 0.936090876 | 1.1088926 | up | mRNA |
| ENST00000310673 | SPESP1 | 0.562418289 | 0.929041598 | 1.1460479 | up | mRNA |
| ENST00000310677 | ADCY4 | 0.360436476 | 0.841780454 | 1.0927106 | up | mRNA |
| ENST00000310944 | LDB3 | 0.71847207 | 0.966191059 | 1.0778132 | up | mRNA |
| ENST00000311127 | HEG1 | 0.089464951 | 0.505699581 | 1.04376 | up | mRNA |
| ENST00000311303 | ABCC12 | 0.992723717 | 0.999798141 | 1.0024168 | up | mRNA |
| ENST00000311519 | GPR113 | 0.930136894 | 0.996649933 | 1.0219225 | up | mRNA |
| ENST00000311591 | OR9Q2 | 0.094886324 | 0.516112557 | 1.3866262 | up | mRNA |
| ENST00000311601 | SH3PXD2B | 0.041631214 | 0.377938997 | 1.2618084 | up | mRNA |
| ENST00000311604 | LSP1 | 0.918828585 | 0.995349793 | 1.0161911 | up | mRNA |
| ENST00000311890 | HSPB7 | 0.754109339 | 0.970944772 | 1.0357453 | up | mRNA |
| ENST00000312010 | USP36 | 0.26010728 | 0.760388386 | 1.1399516 | up | mRNA |
| ENST00000312165 | NLRP6 | 0.007257964 | 0.190657536 | 1.2920274 | up | mRNA |
| ENST00000312423 | SWSAP1 | 0.578245116 | 0.933821303 | 1.3773008 | up | mRNA |
| ENST00000312457 | GPR152 | 0.348225932 | 0.834138149 | 1.0724007 | up | mRNA |
| ENST00000312838 | RNF166 | 0.028054894 | 0.325949769 | 1.1495687 | up | mRNA |
| ENST00000312841 | HR | 0.780735024 | 0.976938981 | 1.0309047 | up | mRNA |
| ENST00000312891 | GJC3 | 0.689089688 | 0.961076385 | 1.0551183 | up | mRNA |
| ENST00000313005 | BACE1 | 0.192694292 | 0.679358478 | 1.114049 | up | mRNA |
| ENST00000313173 | HOXD8 | 0.901040819 | 0.994789506 | 1.0614559 | up | mRNA |
| ENST00000313174 | DACH1 | 0.867054134 | 0.989481868 | 1.0293509 | up | mRNA |
| ENST00000313253 | OR4D11 | 0.483113341 | 0.902654749 | 1.2054881 | up | mRNA |
| ENST00000313472 | OR8H3 | 0.635889565 | 0.951814727 | 1.0344058 | up | mRNA |
| ENST00000313639 | FZR1 | 0.630972746 | 0.950410442 | 1.4561679 | up | mRNA |
| ENST00000313965 | C2orf57 | 0.907662989 | 0.995145546 | 1.0139177 | up | mRNA |
| ENST00000314018 | BHLHA15 | 0.403223906 | 0.864786348 | 1.109341 | up | mRNA |
| ENST00000314067 | IFT43 | 0.73925849 | 0.969564098 | 1.0712434 | up | mRNA |
| ENST00000314254 | MRGPRX4 | 0.304135695 | 0.799856028 | 1.1537633 | up | mRNA |
| ENST00000314355 | CKS2 | 0.661820363 | 0.958354006 | 1.1876281 | up | mRNA |
| ENST00000314737 | ARHGAP33 | 0.526412354 | 0.917975454 | 1.0300194 | up | mRNA |
| ENST00000314933 | C1QB | 0.778571474 | 0.976938981 | 1.0406034 | up | mRNA |
| ENST00000315238 | CALML3 | 0.365498598 | 0.845008704 | 1.6167333 | up | mRNA |
| ENST00000315273 | ASAP2 | 0.890912571 | 0.9937521 | 1.0181794 | up | mRNA |
| ENST00000315392 | PLXDC1 | 0.604863328 | 0.94174007 | 1.0999059 | up | mRNA |
| ENST00000315491 | TUBB3 | 0.537052941 | 0.922702335 | 1.2508661 | up | mRNA |
| ENST00000315843 | GPR156 | 0.060146253 | 0.435030895 | 1.1608303 | up | mRNA |
| ENST00000316097 | SCAMP4 | 0.190993609 | 0.676402188 | 1.1733372 | up | mRNA |
| ENST00000316249 | KCNG2 | 0.983773953 | 0.998971807 | 1.0034035 | up | mRNA |
| ENST00000316397 | GOLGA6L1 | 0.197968237 | 0.686758264 | 1.1242738 | up | mRNA |
| ENST00000316448 | CALR | 0.840783141 | 0.987068842 | 1.0410887 | up | mRNA |
| ENST00000316737 | TSPAN16 | 0.548528903 | 0.92514258 | 1.0980184 | up | mRNA |
| ENST00000316757 | APBA3 | 0.61662217 | 0.945912508 | 1.0324747 | up | mRNA |
| ENST00000316924 | KLC2 | 0.91424344 | 0.995342392 | 1.0175121 | up | mRNA |
| ENST00000317060 | DAND5 | 0.261939924 | 0.763003229 | 1.1276575 | up | mRNA |
| ENST00000317269 | DNAJC14 | 0.605392686 | 0.94174007 | 1.0522321 | up | mRNA |
| ENST00000317726 | S1PR2 | 0.408770929 | 0.867591329 | 1.0818099 | up | mRNA |
| ENST00000317827 | TACC1 | 0.564164478 | 0.929470235 | 1.0342266 | up | mRNA |
| ENST00000317869 | HNRNPCL1 | 0.639710096 | 0.953631914 | 1.04267 | up | mRNA |
| ENST00000317951 | NKPD1 | 0.103144425 | 0.532943442 | 1.3761956 | up | mRNA |
| ENST00000317991 | GRAMD1A | 0.711155802 | 0.96568622 | 1.0208048 | up | mRNA |
| ENST00000318050 | OR4F17 | 0.347259854 | 0.833588015 | 1.0763458 | up | mRNA |
| ENST00000318083 | MAMSTR | 0.363687876 | 0.843238468 | 1.1448097 | up | mRNA |
| ENST00000318388 | NDUFV2 | 0.683780724 | 0.960111041 | 1.0428046 | up | mRNA |
| ENST00000318522 | EML4 | 0.079330986 | 0.482639336 | 1.1442372 | up | mRNA |
| ENST00000318698 | ANKFN1 | 0.287084793 | 0.782954059 | 1.136407 | up | mRNA |
| ENST00000318981 | TIAM2 | 0.026915327 | 0.319244809 | 2.1129762 | up | mRNA |
| ENST00000319420 | SHISA2 | 0.047904873 | 0.398293541 | 1.2375349 | up | mRNA |
| ENST00000319590 | KLK12 | 0.062559793 | 0.439458294 | 1.2531519 | up | mRNA |
| ENST00000319642 | USH1G | 0.009619371 | 0.205650016 | 1.3215576 | up | mRNA |
| ENST00000319928 | MOB3C | 0.148927776 | 0.61263618 | 1.2667587 | up | mRNA |
| ENST00000319977 | C17orf53 | 0.905442138 | 0.99508468 | 1.0216055 | up | mRNA |
| ENST00000320005 | CNGB3 | 0.152648239 | 0.618255775 | 1.2444907 | up | mRNA |
| ENST00000320159 | ZNF48 | 0.581431993 | 0.934005566 | 1.0906981 | up | mRNA |
| ENST00000320201 | PITPNM2 | 0.874274072 | 0.990793509 | 1.0143544 | up | mRNA |
| ENST00000320476 | SCRIB | 0.844139625 | 0.987338076 | 1.0161695 | up | mRNA |
| ENST00000320531 | WBSCR28 | 0.540840115 | 0.923577434 | 1.2063886 | up | mRNA |
| ENST00000320700 | DNASE1L2 | 0.86941693 | 0.989592579 | 1.0129244 | up | mRNA |
| ENST00000320838 | KLK8 | 0.010510463 | 0.210032734 | 1.246027 | up | mRNA |
| ENST00000320865 | B3GNTL1 | 0.588524435 | 0.93579682 | 1.0497214 | up | mRNA |
| ENST00000322038 | TSHZ1 | 0.082080773 | 0.489148905 | 1.2735401 | up | mRNA |
| ENST00000322165 | HSD17B6 | 0.710345308 | 0.965462762 | 1.1205639 | up | mRNA |
| ENST00000322348 | GCNT4 | 0.082415822 | 0.489943729 | 3.1259126 | up | mRNA |
| ENST00000322583 | PRR18 | 0.528655339 | 0.918883792 | 1.1168481 | up | mRNA |
| ENST00000323037 | ZNRF2 | 0.919466112 | 0.995349793 | 1.0092772 | up | mRNA |
| ENST00000323110 | CYP11B2 | 0.582582067 | 0.934005566 | 1.0137458 | up | mRNA |
| ENST00000323374 | SPHK1 | 0.66616175 | 0.958354006 | 1.0336077 | up | mRNA |
| ENST00000323404 | OR3A1 | 0.330882964 | 0.821853462 | 1.3057999 | up | mRNA |
| ENST00000323675 | SPEM1 | 0.277434285 | 0.777234326 | 1.1036946 | up | mRNA |
| ENST00000323813 | C18orf56 | 0.886487263 | 0.992981836 | 1.0109717 | up | mRNA |
| ENST00000324041 | KLK4 | 0.22547233 | 0.720462581 | 1.2970209 | up | mRNA |
| ENST00000324172 | SAMD3 | 0.872695797 | 0.990534615 | 1.0132979 | up | mRNA |
| ENST00000324382 | H1FOO | 0.109548249 | 0.547660436 | 1.2584431 | up | mRNA |
| ENST00000324750 | MC5R | 0.107471451 | 0.54355959 | 1.1505172 | up | mRNA |
| ENST00000325084 | CACNA1A | 0.478961408 | 0.901271944 | 1.052198 | up | mRNA |
| ENST00000325157 | ENTHD1 | 0.95759859 | 0.996922404 | 1.0171062 | up | mRNA |
| ENST00000325351 | ZNF784 | 0.078243788 | 0.480303439 | 1.0889959 | up | mRNA |
| ENST00000325921 | PRDM7 | 0.3418492 | 0.830223283 | 1.0598707 | up | mRNA |
| ENST00000326199 | RPS10 | 0.007478702 | 0.192233838 | 1.152041 | up | mRNA |
| ENST00000326505 | B3GNT5 | 0.014973231 | 0.24511734 | 4.7510405 | up | mRNA |
| ENST00000326853 | SULT1C2 | 0.510440911 | 0.912136104 | 1.0826363 | up | mRNA |
| ENST00000327042 | TMEM86B | 0.900323743 | 0.994789506 | 1.0164242 | up | mRNA |
| ENST00000327064 | CARM1 | 0.468647076 | 0.89739427 | 1.1205417 | up | mRNA |
| ENST00000327141 | AES | 0.714820307 | 0.966110889 | 1.0425322 | up | mRNA |
| ENST00000327245 | ATP10B | 0.155452399 | 0.623056705 | 1.2611261 | up | mRNA |
| ENST00000327705 | BTNL9 | 0.081961828 | 0.488911764 | 1.1032833 | up | mRNA |
| ENST00000327987 | IGDCC3 | 0.769842535 | 0.975286259 | 1.0252635 | up | mRNA |
| ENST00000328092 | NLRP7 | 0.339228927 | 0.827697414 | 1.1207167 | up | mRNA |
| ENST00000328275 | OR2V2 | 0.86544706 | 0.989401142 | 1.0179076 | up | mRNA |
| ENST00000328735 | BACE2 | 0.978541601 | 0.998661138 | 1.0055125 | up | mRNA |
| ENST00000328794 | SPDYE4 | 0.956177277 | 0.996883425 | 1.00581 | up | mRNA |
| ENST00000329106 | SULT1C3 | 0.246874981 | 0.745152522 | 3.397962 | up | mRNA |
| ENST00000329125 | GP1BA | 0.068467927 | 0.454337434 | 1.2127011 | up | mRNA |
| ENST00000329285 | ZNF599 | 0.862652227 | 0.989401142 | 1.0312701 | up | mRNA |
| ENST00000329363 | ODF3B | 0.814191468 | 0.98120545 | 1.0414561 | up | mRNA |
| ENST00000329397 | PTP4A3 | 0.400935539 | 0.864768873 | 1.1288277 | up | mRNA |
| ENST00000329402 | GAST | 0.091832115 | 0.510982637 | 1.2335231 | up | mRNA |
| ENST00000329420 | YIF1B | 0.967610403 | 0.99793927 | 1.0049869 | up | mRNA |
| ENST00000329447 | PRR23B | 0.078413945 | 0.480427915 | 1.5645317 | up | mRNA |
| ENST00000329464 | TRIM69 | 0.504828389 | 0.910568383 | 1.0468917 | up | mRNA |
| ENST00000329474 | LRRC16A | 0.492631459 | 0.906047853 | 1.071567 | up | mRNA |
| ENST00000330120 | RXFP3 | 0.931469724 | 0.996710991 | 1.0083203 | up | mRNA |
| ENST00000330198 | LMLN | 0.025718282 | 0.314812835 | 1.1786649 | up | mRNA |
| ENST00000330432 | OPRM1 | 0.649516306 | 0.95550188 | 1.0992666 | up | mRNA |
| ENST00000330475 | ADAMTSL5 | 0.990060361 | 0.999140676 | 1.0017402 | up | mRNA |
| ENST00000330514 | GJC1 | 0.008326248 | 0.198394583 | 1.2176658 | up | mRNA |
| ENST00000330574 | INTS5 | 0.405656149 | 0.866365632 | 1.1014335 | up | mRNA |
| ENST00000330636 | LYRM4 | 0.017395987 | 0.265536947 | 1.1731772 | up | mRNA |
| ENST00000330824 | GPIHBP1 | 0.013715062 | 0.236215185 | 1.2607311 | up | mRNA |
| ENST00000330891 | BEST3 | 0.397812064 | 0.863112281 | 1.0559443 | up | mRNA |
| ENST00000331336 | KRBA2 | 0.203335673 | 0.692876095 | 1.1448825 | up | mRNA |
| ENST00000331342 | C2orf82 | 0.233757663 | 0.73118887 | 1.092624 | up | mRNA |
| ENST00000331380 | HIST2H2AC | 0.739741944 | 0.969564098 | 1.0778135 | up | mRNA |
| ENST00000331427 | OTOP2 | 0.536232504 | 0.922400335 | 1.1298036 | up | mRNA |
| ENST00000331615 | TMEM106A | 0.008076551 | 0.19619167 | 1.5453557 | up | mRNA |
| ENST00000331923 | GCFC1 | 0.092991297 | 0.511788177 | 1.3320113 | up | mRNA |
| ENST00000332132 | FAM104B | 0.687721536 | 0.960243201 | 1.0347334 | up | mRNA |
| ENST00000332246 | CSGALNACT1 | 0.244822034 | 0.743498371 | 1.2406552 | up | mRNA |
| ENST00000332374 | MT1H | 0.31111824 | 0.806329643 | 1.0687759 | up | mRNA |
| ENST00000332397 | SPDYE3 | 0.134339924 | 0.590871682 | 1.7525745 | up | mRNA |
| ENST00000332411 | KRT76 | 0.054774696 | 0.419413793 | 1.2359389 | up | mRNA |
| ENST00000332427 | GPR19 | 0.780311099 | 0.976938981 | 1.1162702 | up | mRNA |
| ENST00000332578 | TMPRSS9 | 0.359453773 | 0.841033672 | 1.1115914 | up | mRNA |
| ENST00000332585 | OSBP2 | 0.572918263 | 0.93352894 | 1.0897405 | up | mRNA |
| ENST00000332859 | SUMO3 | 0.749720905 | 0.970725371 | 1.0327266 | up | mRNA |
| ENST00000333007 | TNFAIP2 | 0.053955412 | 0.41711842 | 1.9346118 | up | mRNA |
| ENST00000333149 | TRIM50 | 0.825032777 | 0.98381451 | 1.0359345 | up | mRNA |
| ENST00000333323 | FAM212A | 0.648503957 | 0.95550188 | 1.0200508 | up | mRNA |
| ENST00000333407 | FAM83F | 0.895422419 | 0.993978977 | 1.0156813 | up | mRNA |
| ENST00000333437 | TEX19 | 0.060483261 | 0.435925442 | 1.1781126 | up | mRNA |
| ENST00000333598 | C19orf59 | 0.717406506 | 0.966191059 | 1.0731288 | up | mRNA |
| ENST00000333896 | SPTBN1 | 0.032789094 | 0.34498156 | 1.1035622 | up | mRNA |
| ENST00000333999 | ACTL7A | 0.572307927 | 0.933397932 | 1.0519012 | up | mRNA |
| ENST00000334160 | KRTAP23-1 | 0.270556154 | 0.770332967 | 1.1073787 | up | mRNA |
| ENST00000334221 | C1QL4 | 0.867856635 | 0.989481868 | 1.0490802 | up | mRNA |
| ENST00000334346 | MT1B | 0.139503987 | 0.598648178 | 2.0651707 | up | mRNA |
| ENST00000334370 | MEGF8 | 0.443022271 | 0.884304813 | 1.2075081 | up | mRNA |
| ENST00000334534 | C20orf94 | 0.791559721 | 0.978822498 | 1.0316475 | up | mRNA |
| ENST00000334567 | TMEM212 | 0.000849696 | 0.083093876 | 2.4825554 | up | mRNA |
| ENST00000334662 | KRTAP10-8 | 0.449564896 | 0.888279993 | 1.0625171 | up | mRNA |
| ENST00000334994 | LRRC70 | 0.833507055 | 0.986434891 | 1.0768254 | up | mRNA |
| ENST00000335017 | H1FNT | 0.395563075 | 0.861590143 | 1.1059646 | up | mRNA |
| ENST00000335108 | C17orf82 | 0.017447557 | 0.266124324 | 1.3893382 | up | mRNA |
| ENST00000335123 | LCE1A | 0.02022695 | 0.28481199 | 1.1767904 | up | mRNA |
| ENST00000335137 | OR4F5 | 0.448919148 | 0.888279993 | 1.1613916 | up | mRNA |
| ENST00000335154 | FMNL3 | 1.85214E-06 | 0.008848943 | 2.6504469 | up | mRNA |
| ENST00000335385 | TBX10 | 0.417362952 | 0.871323908 | 1.099225 | up | mRNA |
| ENST00000335473 | MYO18B | 0.953391966 | 0.996710991 | 1.0032978 | up | mRNA |
| ENST00000335496 | SPATA21 | 0.286633592 | 0.782942227 | 1.1543258 | up | mRNA |
| ENST00000335783 | CENPQ | 0.811581779 | 0.98118927 | 1.1316302 | up | mRNA |
| ENST00000335866 | GRB10 | 0.773088882 | 0.97589756 | 1.033184 | up | mRNA |
| ENST00000335934 | TP53I3 | 0.952740952 | 0.996710991 | 1.0111049 | up | mRNA |
| ENST00000335999 | NCKAP5L | 0.531642112 | 0.920228311 | 1.0433366 | up | mRNA |
| ENST00000336727 | RGS12 | 0.973422498 | 0.998501532 | 1.0036249 | up | mRNA |
| ENST00000336985 | PRR5 | 0.428311574 | 0.877211962 | 1.1200875 | up | mRNA |
| ENST00000337554 | TSPO | 0.790487657 | 0.978460675 | 1.0156648 | up | mRNA |
| ENST00000337565 | BCL2L11 | 0.481245433 | 0.901538317 | 1.0765837 | up | mRNA |
| ENST00000337653 | CHAT | 0.332226081 | 0.822707947 | 1.1457665 | up | mRNA |
| ENST00000337752 | C5orf56 | 0.001092403 | 0.086778252 | 2.1592144 | up | mRNA |
| ENST00000337801 | AQP12A | 0.245055972 | 0.743548975 | 1.1567504 | up | mRNA |
| ENST00000337940 | NR1I2 | 0.954028062 | 0.996710991 | 1.0065096 | up | mRNA |
| ENST00000338244 | SLC23A2 | 0.022780253 | 0.297426317 | 1.2282204 | up | mRNA |
| ENST00000338270 | ERAS | 0.714710092 | 0.966110889 | 1.030431 | up | mRNA |
| ENST00000338560 | TRPV2 | 0.947620271 | 0.996710991 | 1.0171614 | up | mRNA |
| ENST00000338835 | NCR1 | 0.159128154 | 0.630864648 | 1.3476984 | up | mRNA |
| ENST00000338957 | ZC3H12B | 0.665777911 | 0.958354006 | 1.0899963 | up | mRNA |
| ENST00000339121 | ING3 | 0.512492294 | 0.91317044 | 1.0561729 | up | mRNA |
| ENST00000339123 | SMOX | 0.079456265 | 0.482639336 | 2.0472584 | up | mRNA |
| ENST00000339151 | KIF18B | 0.703267207 | 0.963625105 | 1.037924 | up | mRNA |
| ENST00000339223 | FPR3 | 0.68304668 | 0.960111041 | 1.093146 | up | mRNA |
| ENST00000339235 | FBXL14 | 0.943557847 | 0.996710991 | 1.0078191 | up | mRNA |
| ENST00000339413 | YIF1B | 0.802084047 | 0.98118927 | 1.0324393 | up | mRNA |
| ENST00000339598 | OTOF | 0.817568663 | 0.981727075 | 1.0533721 | up | mRNA |
| ENST00000339948 | ZNF648 | 0.710813486 | 0.965672846 | 1.0775972 | up | mRNA |
| ENST00000339996 | TMEM8C | 0.471740495 | 0.898908705 | 1.1192958 | up | mRNA |
| ENST00000340057 | IL17A | 0.067700449 | 0.451808679 | 1.4612447 | up | mRNA |
| ENST00000340077 | PARD3 | 0.906579909 | 0.99508468 | 1.0118216 | up | mRNA |
| ENST00000340368 | INSIG1 | 0.605871541 | 0.94174007 | 1.1981797 | up | mRNA |
| ENST00000340457 | UPK3BL | 0.540541875 | 0.923577434 | 1.0982107 | up | mRNA |
| ENST00000340790 | PALM3 | 0.552159521 | 0.926337111 | 1.1199208 | up | mRNA |
| ENST00000341048 | ANKRD55 | 0.173269778 | 0.651548201 | 1.1604741 | up | mRNA |
| ENST00000341206 | LCN6 | 0.002235689 | 0.111960638 | 1.3415859 | up | mRNA |
| ENST00000341415 | IGFL3 | 0.347096729 | 0.833588015 | 1.0712628 | up | mRNA |
| ENST00000341426 | NADK | 0.723980389 | 0.966191059 | 1.0336154 | up | mRNA |
| ENST00000341500 | INSR | 0.710086322 | 0.965462762 | 1.0844999 | up | mRNA |
| ENST00000341747 | PRR19 | 0.857947316 | 0.988512246 | 1.0047396 | up | mRNA |
| ENST00000341948 | PCDHB13 | 0.61618125 | 0.945912508 | 1.1120107 | up | mRNA |
| ENST00000342280 | GJA4 | 0.960033453 | 0.997738713 | 1.0141506 | up | mRNA |
| ENST00000342310 | LMX1A | 0.448221359 | 0.887969953 | 1.0629929 | up | mRNA |
| ENST00000342331 | ASCL4 | 0.869201079 | 0.989592579 | 1.0564261 | up | mRNA |
| ENST00000342716 | MATN4 | 0.372419858 | 0.84763089 | 1.1403138 | up | mRNA |
| ENST00000342916 | EGFR | 0.01619428 | 0.255838153 | 1.1346285 | up | mRNA |
| ENST00000342960 | NBPF10 | 0.544035088 | 0.923577434 | 1.1259538 | up | mRNA |
| ENST00000343070 | SCNN1B | 0.484502114 | 0.903250293 | 1.0814093 | up | mRNA |
| ENST00000343098 | FSIP2 | 0.138470601 | 0.597943156 | 1.0730525 | up | mRNA |
| ENST00000343200 | VCAN | 0.874277055 | 0.990793509 | 1.0192566 | up | mRNA |
| ENST00000343217 | DUSP28 | 0.771187206 | 0.975347132 | 1.0270969 | up | mRNA |
| ENST00000343267 | APOD | 0.726716888 | 0.966191059 | 1.0630856 | up | mRNA |
| ENST00000343430 | OR51F1 | 0.16459866 | 0.637911838 | 1.8373607 | up | mRNA |
| ENST00000343455 | DICER1 | 0.999961389 | 0.999961389 | 1.0000086 | up | mRNA |
| ENST00000343677 | HIST1H1C | 0.016563947 | 0.257082578 | 1.2625936 | up | mRNA |
| ENST00000343684 | NKAPL | 0.603852297 | 0.941514447 | 1.0490471 | up | mRNA |
| ENST00000343702 | NTN4 | 0.006022256 | 0.176785634 | 1.2054786 | up | mRNA |
| ENST00000343849 | CAV3 | 0.916874 | 0.995349793 | 1.0271281 | up | mRNA |
| ENST00000343916 | COL9A3 | 0.219200243 | 0.713655619 | 1.2468713 | up | mRNA |
| ENST00000344119 | ENTPD8 | 0.403622323 | 0.865202854 | 1.10561 | up | mRNA |
| ENST00000344469 | MED22 | 0.182486469 | 0.66575662 | 1.0916178 | up | mRNA |
| ENST00000344581 | KLHL33 | 0.241271831 | 0.73945497 | 1.2459534 | up | mRNA |
| ENST00000344691 | RUNX1 | 0.437247032 | 0.882234667 | 1.0709349 | up | mRNA |
| ENST00000344774 | FAM166A | 0.898033107 | 0.994326043 | 1.0094935 | up | mRNA |
| ENST00000344839 | BSPH1 | 0.233115835 | 0.731100552 | 1.0493922 | up | mRNA |
| ENST00000344941 | SLC15A5 | 0.798486404 | 0.980749101 | 1.0207651 | up | mRNA |
| ENST00000345005 | CLK3 | 0.266168704 | 0.767064488 | 1.1918976 | up | mRNA |
| ENST00000346832 | ABI1 | 0.50551194 | 0.910772598 | 1.1354835 | up | mRNA |
| ENST00000347619 | KLK8 | 0.072786318 | 0.463915802 | 1.1523372 | up | mRNA |
| ENST00000347624 | LRRC15 | 0.081741497 | 0.488477232 | 1.3974416 | up | mRNA |
| ENST00000347697 | EPN2 | 0.786397794 | 0.977479172 | 1.0221189 | up | mRNA |
| ENST00000347757 | SSX5 | 0.487644111 | 0.903757389 | 1.1006476 | up | mRNA |
| ENST00000347800 | ABCG1 | 0.243714767 | 0.742130994 | 1.1562898 | up | mRNA |
| ENST00000348075 | PTPRS | 0.719979866 | 0.966191059 | 1.0262539 | up | mRNA |
| ENST00000349038 | PTBP1 | 0.513005295 | 0.913297047 | 1.1005022 | up | mRNA |
| ENST00000349124 | PTP4A3 | 0.543074201 | 0.923577434 | 1.076212 | up | mRNA |
| ENST00000349155 | TBX3 | 0.08917189 | 0.504744674 | 1.7388434 | up | mRNA |
| ENST00000349243 | AGTR1 | 0.834547823 | 0.986575022 | 1.2352619 | up | mRNA |
| ENST00000349394 | NXPH4 | 0.113123513 | 0.55632728 | 1.0927037 | up | mRNA |
| ENST00000350092 | PTBP1 | 0.746892925 | 0.970432947 | 1.0495597 | up | mRNA |
| ENST00000350608 | DDT | 0.461991537 | 0.894754673 | 1.0481569 | up | mRNA |
| ENST00000351018 | RHOG | 0.055825638 | 0.421575513 | 1.6112005 | up | mRNA |
| ENST00000352456 | HNRNPUL1 | 0.88825363 | 0.993440915 | 1.0243443 | up | mRNA |
| ENST00000352536 | VGLL2 | 0.025775506 | 0.315133847 | 1.4264263 | up | mRNA |
| ENST00000353005 | EMR2 | 0.469322352 | 0.897810636 | 1.4192859 | up | mRNA |
| ENST00000353331 | BCLAF1 | 0.635747449 | 0.951814727 | 1.0233166 | up | mRNA |
| ENST00000353876 | EMR2 | 0.572900535 | 0.93352894 | 1.0491595 | up | mRNA |
| ENST00000354204 | KANSL3 | 0.37297683 | 0.84810582 | 1.1127078 | up | mRNA |
| ENST00000354276 | PLA2G4C | 0.633199497 | 0.951174475 | 1.1549746 | up | mRNA |
| ENST00000354293 | AP2A1 | 0.627309674 | 0.949299851 | 1.089883 | up | mRNA |
| ENST00000354419 | KIF25 | 0.852995408 | 0.987678166 | 1.0104328 | up | mRNA |
| ENST00000354591 | DACH1 | 0.610353217 | 0.94320146 | 1.1049477 | up | mRNA |
| ENST00000354650 | FYN | 0.927474182 | 0.995829678 | 1.0129682 | up | mRNA |
| ENST00000354760 | CRYBA4 | 0.62203329 | 0.948064532 | 1.0687031 | up | mRNA |
| ENST00000354882 | TMEM205 | 0.441352178 | 0.883920737 | 1.1266024 | up | mRNA |
| ENST00000354883 | SLC25A26 | 0.642155825 | 0.954332328 | 1.054898 | up | mRNA |
| ENST00000355025 | INCA1 | 0.749980182 | 0.970725371 | 1.0719602 | up | mRNA |
| ENST00000355067 | HSPB9 | 0.493926154 | 0.906630188 | 1.0340727 | up | mRNA |
| ENST00000355092 | PORCN | 0.612468108 | 0.944159427 | 1.131244 | up | mRNA |
| ENST00000355151 | MRPL52 | 0.652524195 | 0.956671614 | 1.0761275 | up | mRNA |
| ENST00000355162 | TBL1Y | 0.263881193 | 0.764495522 | 1.0852474 | up | mRNA |
| ENST00000355285 | APCDD1 | 0.855591724 | 0.988178307 | 1.0113984 | up | mRNA |
| ENST00000355497 | LMX1B | 0.049846136 | 0.40409555 | 1.1427955 | up | mRNA |
| ENST00000355502 | PDE4C | 0.862301991 | 0.989401142 | 1.0124589 | up | mRNA |
| ENST00000355653 | VAT1 | 0.595028643 | 0.938655001 | 1.0851227 | up | mRNA |
| ENST00000355728 | OR2T6 | 0.404117632 | 0.865617032 | 1.0769525 | up | mRNA |
| ENST00000355797 | MXRA7 | 0.920744164 | 0.995349793 | 1.0105462 | up | mRNA |
| ENST00000355819 | CLEC9A | 0.707352515 | 0.965401045 | 1.0777449 | up | mRNA |
| ENST00000356046 | GRHL3 | 0.117172377 | 0.564147103 | 1.1078405 | up | mRNA |
| ENST00000356175 | NF1 | 0.644374296 | 0.954657712 | 1.0232817 | up | mRNA |
| ENST00000356284 | SMOC2 | 0.744134317 | 0.970330526 | 1.0703427 | up | mRNA |
| ENST00000356467 | FAM168A | 0.630802761 | 0.950410442 | 1.0402347 | up | mRNA |
| ENST00000356628 | NRARP | 0.001583333 | 0.103180534 | 46.9245932 | up | mRNA |
| ENST00000356714 | NUDT1 | 0.951873848 | 0.996710991 | 1.0030396 | up | mRNA |
| ENST00000356897 | IL27 | 0.383774926 | 0.854109523 | 1.2391415 | up | mRNA |
| ENST00000356948 | PTBP1 | 0.730148298 | 0.966191059 | 1.0241015 | up | mRNA |
| ENST00000356956 | IGF2R | 0.810240375 | 0.98118927 | 1.021899 | up | mRNA |
| ENST00000356994 | SCRIB | 0.357976308 | 0.840264869 | 1.1198281 | up | mRNA |
| ENST00000357210 | MIB2 | 0.821856859 | 0.982940803 | 1.0282065 | up | mRNA |
| ENST00000357330 | GALP | 0.513700544 | 0.91350662 | 1.1273606 | up | mRNA |
| ENST00000357461 | TMEM229B | 0.175166903 | 0.653821873 | 1.1055642 | up | mRNA |
| ENST00000357549 | HIST1H4K | 0.859168828 | 0.988512246 | 1.0093856 | up | mRNA |
| ENST00000357596 | PHF11 | 0.551803694 | 0.92621751 | 1.1045549 | up | mRNA |
| ENST00000357627 | OR14A16 | 0.075507655 | 0.471940255 | 1.3194544 | up | mRNA |
| ENST00000357647 | HIST1H3A | 0.617576932 | 0.94659436 | 1.0787397 | up | mRNA |
| ENST00000357887 | SLC8A3 | 0.289905919 | 0.786468159 | 1.1409061 | up | mRNA |
| ENST00000357895 | NEDD4L | 0.578305168 | 0.933821303 | 1.1171671 | up | mRNA |
| ENST00000357916 | MAGEA12 | 0.830371078 | 0.985549562 | 1.040917 | up | mRNA |
| ENST00000358004 | PRELID2 | 0.455439815 | 0.891148383 | 1.086776 | up | mRNA |
| ENST00000358007 | DDX47 | 0.721672374 | 0.966191059 | 1.0430293 | up | mRNA |
| ENST00000358066 | SEMA6D | 0.043724327 | 0.385351177 | 1.1959889 | up | mRNA |
| ENST00000358078 | ASIC4 | 0.787093127 | 0.977479172 | 1.0305368 | up | mRNA |
| ENST00000358130 | HMGN5 | 0.867889358 | 0.989481868 | 1.0196499 | up | mRNA |
| ENST00000358138 | C8orf86 | 0.965482703 | 0.99793927 | 1.0079354 | up | mRNA |
| ENST00000358184 | CTSE | 0.129165062 | 0.583337191 | 1.1004581 | up | mRNA |
| ENST00000358241 | RTP2 | 0.938850609 | 0.996710991 | 1.0082541 | up | mRNA |
| ENST00000358273 | NF1 | 0.150213543 | 0.615010423 | 1.155664 | up | mRNA |
| ENST00000358400 | ABCB4 | 0.262057233 | 0.763125731 | 1.4249279 | up | mRNA |
| ENST00000358556 | EDNRA | 0.6650486 | 0.958354006 | 1.0521513 | up | mRNA |
| ENST00000358697 | ZXDA | 0.002088143 | 0.110028846 | 1.9326178 | up | mRNA |
| ENST00000358716 | BAZ1A | 0.000811177 | 0.083087642 | 1.7132603 | up | mRNA |
| ENST00000358808 | CLIP1 | 0.811507829 | 0.98118927 | 1.0245113 | up | mRNA |
| ENST00000358825 | PRDM10 | 0.338727602 | 0.827403747 | 1.1038078 | up | mRNA |
| ENST00000358849 | ABCB8 | 0.872975772 | 0.990534615 | 1.0189456 | up | mRNA |
| ENST00000358894 | COL20A1 | 0.7623654 | 0.97339948 | 1.0998705 | up | mRNA |
| ENST00000358897 | FHAD1 | 0.752710728 | 0.970725371 | 1.3550638 | up | mRNA |
| ENST00000359032 | AP2A1 | 0.553836639 | 0.926354119 | 1.0672198 | up | mRNA |
| ENST00000359161 | RFX2 | 0.705716471 | 0.964937948 | 1.0779424 | up | mRNA |
| ENST00000359227 | ELAVL3 | 0.288156348 | 0.784355942 | 1.7474025 | up | mRNA |
| ENST00000359273 | BCS1L | 0.656884056 | 0.957445617 | 1.0381823 | up | mRNA |
| ENST00000359303 | HIST1H3J | 0.633511849 | 0.951174475 | 1.0475206 | up | mRNA |
| ENST00000359320 | CMTM5 | 0.668696283 | 0.959013389 | 1.0223211 | up | mRNA |
| ENST00000359323 | DIO3 | 0.701091081 | 0.963015537 | 1.0228029 | up | mRNA |
| ENST00000359520 | TECPR2 | 0.583500931 | 0.934280817 | 1.0622425 | up | mRNA |
| ENST00000359523 | MPZL1 | 0.135227899 | 0.592787801 | 1.1401237 | up | mRNA |
| ENST00000359558 | CALCR | 0.844652542 | 0.987338076 | 1.0337643 | up | mRNA |
| ENST00000359611 | HIST1H2AM | 0.142015087 | 0.60204132 | 1.4941671 | up | mRNA |
| ENST00000359791 | NPSR1 | 0.788853341 | 0.977807732 | 1.0189207 | up | mRNA |
| ENST00000359850 | NUDT16 | 0.491825307 | 0.905806259 | 1.0621087 | up | mRNA |
| ENST00000359971 | ZNF517 | 0.009039287 | 0.202329498 | 1.1421531 | up | mRNA |
| ENST00000359987 | GNRH2 | 0.872143234 | 0.990339403 | 1.0145934 | up | mRNA |
| ENST00000360019 | CLN3 | 0.699426103 | 0.962811883 | 1.0601054 | up | mRNA |
| ENST00000360096 | ARL9 | 0.782629661 | 0.977106756 | 1.0781921 | up | mRNA |
| ENST00000360168 | SCNN1A | 0.277069897 | 0.777205298 | 1.1259158 | up | mRNA |
| ENST00000360202 | KIRREL2 | 0.929230238 | 0.996472005 | 1.0121101 | up | mRNA |
| ENST00000360238 | CUEDC1 | 0.384507001 | 0.85485898 | 1.0692222 | up | mRNA |
| ENST00000360379 | SPRR2D | 0.137481351 | 0.596770033 | 1.1657804 | up | mRNA |
| ENST00000360388 | SLC35F1 | 0.957182443 | 0.996883425 | 1.006981 | up | mRNA |
| ENST00000360409 | NRP2 | 0.000171909 | 0.044621794 | 4.1924714 | up | mRNA |
| ENST00000360617 | KLK3 | 0.101621126 | 0.529590777 | 1.178409 | up | mRNA |
| ENST00000360658 | TLR9 | 0.322217319 | 0.815372756 | 1.0370262 | up | mRNA |
| ENST00000360802 | UBAP2 | 0.009583775 | 0.205539348 | 1.3890999 | up | mRNA |
| ENST00000360957 | PPP1R13L | 0.768853541 | 0.975252991 | 1.02566 | up | mRNA |
| ENST00000361010 | CALCA | 0.737661935 | 0.969031501 | 1.0977327 | up | mRNA |
| ENST00000361101 | CYTH1 | 0.420136676 | 0.872365335 | 1.1898861 | up | mRNA |
| ENST00000361134 | SLC34A3 | 0.42173561 | 0.873398499 | 1.1634692 | up | mRNA |
| ENST00000361255 | TRPM6 | 0.721069115 | 0.966191059 | 1.0477414 | up | mRNA |
| ENST00000361297 | MAST2 | 0.002673361 | 0.122697021 | 1.0846652 | up | mRNA |
| ENST00000361300 | PHACTR3 | 0.006071821 | 0.177373936 | 2.1062336 | up | mRNA |
| ENST00000361303 | C18orf1 | 0.266638169 | 0.767248719 | 1.2108561 | up | mRNA |
| ENST00000361365 | EIF1AY | 0.499361715 | 0.909361611 | 1.0756483 | up | mRNA |
| ENST00000361509 | IGSF9 | 0.36767014 | 0.845181673 | 1.093699 | up | mRNA |
| ENST00000361570 | CDKN2A | 0.873373101 | 0.990580595 | 1.0482106 | up | mRNA |
| ENST00000361574 | R3HDM4 | 0.854068219 | 0.987877747 | 1.0191899 | up | mRNA |
| ENST00000361575 | RPL39 | 0.630113796 | 0.950410442 | 1.0498127 | up | mRNA |
| ENST00000361625 | ZFYVE9 | 0.870726484 | 0.989592579 | 1.0265723 | up | mRNA |
| ENST00000361752 | QKI | 0.619084235 | 0.947333534 | 1.0945175 | up | mRNA |
| ENST00000361779 | IL1RN | 0.091178584 | 0.50968981 | 1.6233034 | up | mRNA |
| ENST00000361825 | GATSL1 | 0.536608107 | 0.922509728 | 1.0879879 | up | mRNA |
| ENST00000361902 | GPX6 | 0.663599278 | 0.958354006 | 1.0511386 | up | mRNA |
| ENST00000361906 | TMEM184B | 0.702396119 | 0.963378163 | 1.0261232 | up | mRNA |
| ENST00000366474 | OR2T1 | 0.453346233 | 0.889563297 | 1.1343064 | up | mRNA |
| ENST00000366609 | IRF2BP2 | 0.222730417 | 0.717565338 | 1.0941273 | up | mRNA |
| ENST00000366610 | IRF2BP2 | 0.69904911 | 0.962701213 | 1.0471584 | up | mRNA |
| ENST00000366641 | EGLN1 | 0.000999417 | 0.085705167 | 1.3414522 | up | mRNA |
| ENST00000366932 | RRP15 | 0.474139684 | 0.899418411 | 1.0582518 | up | mRNA |
| ENST00000366997 | LPGAT1 | 0.865430052 | 0.989401142 | 1.010001 | up | mRNA |
| ENST00000367070 | C4BPA | 0.914201626 | 0.995342392 | 1.0170679 | up | mRNA |
| ENST00000367360 | RAET1G | 0.58791734 | 0.93579682 | 1.0285375 | up | mRNA |
| ENST00000367429 | CFH | 0.50849309 | 0.911674633 | 1.411395 | up | mRNA |
| ENST00000367818 | XCL1 | 0.222415204 | 0.71737156 | 1.191814 | up | mRNA |
| ENST00000367866 | POU2F1 | 0.975613264 | 0.998501532 | 1.0028341 | up | mRNA |
| ENST00000367980 | NR1I3 | 0.183913372 | 0.667532183 | 1.1767909 | up | mRNA |
| ENST00000368093 | SLAMF9 | 0.99472685 | 0.999872237 | 1.0016817 | up | mRNA |
| ENST00000368094 | IGSF9 | 0.165209006 | 0.638608196 | 1.117607 | up | mRNA |
| ENST00000368194 | ARHGEF11 | 0.185621957 | 0.669321303 | 1.4028094 | up | mRNA |
| ENST00000368243 | C1orf61 | 0.489283664 | 0.904620847 | 1.2405882 | up | mRNA |
| ENST00000368482 | TDRD10 | 0.233625394 | 0.73118887 | 1.277273 | up | mRNA |
| ENST00000368485 | IL6R | 0.364218903 | 0.84362654 | 1.3065473 | up | mRNA |
| ENST00000368695 | FANK1 | 0.086488311 | 0.49936346 | 1.0916411 | up | mRNA |
| ENST00000368718 | S100A5 | 0.839360531 | 0.987032229 | 1.0161583 | up | mRNA |
| ENST00000368725 | S100A7L2 | 0.185891449 | 0.669321303 | 1.1811888 | up | mRNA |
| ENST00000368733 | S100A8 | 0.961784454 | 0.99793927 | 1.0095429 | up | mRNA |
| ENST00000368738 | S100A9 | 0.473345324 | 0.899184677 | 1.5989533 | up | mRNA |
| ENST00000368744 | PRR9 | 0.181116309 | 0.664543371 | 1.1435271 | up | mRNA |
| ENST00000368750 | SPRR2E | 0.175779548 | 0.654569554 | 1.5472239 | up | mRNA |
| ENST00000368768 | LCE1C | 0.49990553 | 0.909550837 | 1.282112 | up | mRNA |
| ENST00000368773 | KPRP | 0.787298715 | 0.977479172 | 1.0569042 | up | mRNA |
| ENST00000368775 | C1orf68 | 0.595797628 | 0.938832626 | 1.1176412 | up | mRNA |
| ENST00000368784 | LCE2D | 0.244982259 | 0.743548975 | 1.3778571 | up | mRNA |
| ENST00000368789 | LCE3E | 0.130826926 | 0.585768124 | 1.4782347 | up | mRNA |
| ENST00000368802 | REV3L | 0.092383001 | 0.511338764 | 1.2017097 | up | mRNA |
| ENST00000368821 | C10orf122 | 0.697190893 | 0.962410025 | 1.0223626 | up | mRNA |
| ENST00000368985 | CTSS | 0.012304265 | 0.225175798 | 2.1901903 | up | mRNA |
| ENST00000369155 | HIST2H2BE | 0.439702881 | 0.883054028 | 1.1794972 | up | mRNA |
| ENST00000369160 | HIST3H2BB | 0.755233159 | 0.971417904 | 1.0440231 | up | mRNA |
| ENST00000369209 | HSPA12A | 0.317812687 | 0.811908136 | 1.2515936 | up | mRNA |
| ENST00000369210 | C10orf82 | 0.001221476 | 0.09206987 | 1.2484579 | up | mRNA |
| ENST00000369271 | AFAP1L2 | 0.277707037 | 0.777240591 | 1.126588 | up | mRNA |
| ENST00000369314 | POLR3GL | 0.691141386 | 0.961119378 | 1.0571483 | up | mRNA |
| ENST00000369483 | IGSF3 | 0.895087814 | 0.993978977 | 1.0123054 | up | mRNA |
| ENST00000369729 | SFR1 | 0.004316863 | 0.150034965 | 2.3192318 | up | mRNA |
| ENST00000369783 | CALHM3 | 0.972387324 | 0.998501532 | 1.0032217 | up | mRNA |
| ENST00000369849 | INA | 0.196459015 | 0.684911641 | 1.078372 | up | mRNA |
| ENST00000369886 | SAMD10 | 0.721277969 | 0.966191059 | 1.0315016 | up | mRNA |
| ENST00000369949 | C1orf194 | 0.840067636 | 0.987068842 | 1.0400633 | up | mRNA |
| ENST00000369971 | CLCC1 | 0.87281689 | 0.990534615 | 1.0149692 | up | mRNA |
| ENST00000370018 | RTEL1 | 0.624942776 | 0.948658841 | 1.057874 | up | mRNA |
| ENST00000370231 | TREX2 | 0.605858222 | 0.94174007 | 1.0444062 | up | mRNA |
| ENST00000370501 | NTSR1 | 0.092927648 | 0.511756486 | 1.1436345 | up | mRNA |
| ENST00000370634 | VGLL1 | 0.718636587 | 0.966191059 | 1.0540598 | up | mRNA |
| ENST00000371076 | UBE2U | 0.745346997 | 0.970378122 | 1.069919 | up | mRNA |
| ENST00000371116 | FOXD3 | 0.575023025 | 0.933656777 | 1.1314175 | up | mRNA |
| ENST00000371237 | C8B | 0.974217052 | 0.998501532 | 1.0062023 | up | mRNA |
| ENST00000371268 | TMEM61 | 0.105654019 | 0.537980843 | 1.1333694 | up | mRNA |
| ENST00000371336 | CASS4 | 0.97183263 | 0.998501532 | 1.009367 | up | mRNA |
| ENST00000371360 | LDLRAD1 | 0.182105408 | 0.665333517 | 1.0849037 | up | mRNA |
| ENST00000371369 | FAM70A | 0.371482203 | 0.846675738 | 1.1485516 | up | mRNA |
| ENST00000371470 | MAGOH | 0.117152468 | 0.564147103 | 1.1493233 | up | mRNA |
| ENST00000371564 | NFATC2 | 0.189298518 | 0.674719659 | 1.3916691 | up | mRNA |
| ENST00000371620 | C9orf142 | 0.951719668 | 0.996710991 | 1.0073753 | up | mRNA |
| ENST00000371835 | BRD4 | 0.216862232 | 0.711074992 | 1.1299331 | up | mRNA |
| ENST00000371852 | CH25H | 0.082948258 | 0.49126245 | 7.1656728 | up | mRNA |
| ENST00000371901 | CYP4X1 | 0.558415886 | 0.926833616 | 1.0538346 | up | mRNA |
| ENST00000372056 | LDB3 | 0.563401677 | 0.929041598 | 1.1262278 | up | mRNA |
| ENST00000372101 | KCNE1L | 0.008017047 | 0.195524479 | 1.5207968 | up | mRNA |
| ENST00000372264 | PPAPDC3 | 0.166823485 | 0.641842579 | 1.0833887 | up | mRNA |
| ENST00000372322 | FAM22G | 0.299685895 | 0.795756183 | 1.0955728 | up | mRNA |
| ENST00000372478 | C9orf50 | 0.797021547 | 0.980667273 | 1.046051 | up | mRNA |
| ENST00000372582 | IL1RAPL2 | 0.481617643 | 0.901763506 | 1.181255 | up | mRNA |
| ENST00000372584 | HIVEP3 | 0.011572608 | 0.219901175 | 3.3910252 | up | mRNA |
| ENST00000372945 | MRPS16 | 0.241265028 | 0.73945497 | 1.1228233 | up | mRNA |
| ENST00000372990 | RHBDL2 | 0.021178525 | 0.288424269 | 1.1023353 | up | mRNA |
| ENST00000372994 | C9orf16 | 0.947536216 | 0.996710991 | 1.0067027 | up | mRNA |
| ENST00000373034 | PCDH19 | 0.226067823 | 0.721636281 | 1.1809593 | up | mRNA |
| ENST00000373129 | STK40 | 0.176655213 | 0.65602809 | 1.6213441 | up | mRNA |
| ENST00000373151 | MAP7D1 | 0.355940225 | 0.838847221 | 1.2680221 | up | mRNA |
| ENST00000373176 | AK1 | 0.662367797 | 0.958354006 | 1.0635037 | up | mRNA |
| ENST00000373314 | FAM129B | 0.052645034 | 0.413038171 | 1.1667272 | up | mRNA |
| ENST00000373471 | FNDC5 | 0.474298254 | 0.899418411 | 1.0954327 | up | mRNA |
| ENST00000373491 | TBC1D22B | 0.202273821 | 0.69131473 | 1.1349776 | up | mRNA |
| ENST00000373521 | PABPC1L2B | 0.003623378 | 0.139792244 | 1.3161986 | up | mRNA |
| ENST00000373574 | WDR38 | 0.802591053 | 0.98118927 | 1.0319771 | up | mRNA |
| ENST00000373586 | EIF3I | 0.813856032 | 0.98120545 | 1.0154121 | up | mRNA |
| ENST00000373745 | DSN1 | 0.585301891 | 0.934708084 | 1.1026368 | up | mRNA |
| ENST00000373883 | TMEM198 | 0.374109565 | 0.849308269 | 1.6561704 | up | mRNA |
| ENST00000373935 | IPMK | 0.451129838 | 0.888854908 | 1.0630167 | up | mRNA |
| ENST00000373943 | STX12 | 0.710594672 | 0.965504602 | 1.1360713 | up | mRNA |
| ENST00000374094 | AGAP5 | 0.309482728 | 0.803961878 | 1.1032749 | up | mRNA |
| ENST00000374118 | KIF12 | 0.729000341 | 0.966191059 | 1.0322344 | up | mRNA |
| ENST00000374140 | RGS3 | 0.862929751 | 0.989401142 | 1.0213649 | up | mRNA |
| ENST00000374268 | FAM110D | 0.546181119 | 0.92457863 | 1.0750176 | up | mRNA |
| ENST00000374312 | PPYR1 | 0.105109251 | 0.536745347 | 1.4533752 | up | mRNA |
| ENST00000374491 | EDEM2 | 0.067822752 | 0.45182575 | 1.0578567 | up | mRNA |
| ENST00000374519 | P2RY4 | 0.407662984 | 0.867591329 | 1.0844351 | up | mRNA |
| ENST00000374767 | NIPSNAP3A | 0.869814764 | 0.989592579 | 1.0250197 | up | mRNA |
| ENST00000374798 | CYLC2 | 0.300114768 | 0.795797471 | 1.3217155 | up | mRNA |
| ENST00000374825 | BRD2 | 0.700927349 | 0.962991468 | 1.0360836 | up | mRNA |
| ENST00000374993 | BTNL2 | 0.553073713 | 0.926337111 | 1.098524 | up | mRNA |
| ENST00000375011 | GALNT12 | 0.71957463 | 0.966191059 | 1.0796675 | up | mRNA |
| ENST00000375023 | NOTCH4 | 0.512410458 | 0.91317044 | 1.0545835 | up | mRNA |
| ENST00000375043 | GPSM3 | 0.472093212 | 0.899081978 | 1.0788533 | up | mRNA |
| ENST00000375190 | TBXA2R | 0.85075396 | 0.987678166 | 1.021704 | up | mRNA |
| ENST00000375353 | FAM70B | 0.363161198 | 0.843238468 | 1.1327744 | up | mRNA |
| ENST00000375446 | NINJ1 | 0.0003572 | 0.057900793 | 4.3671697 | up | mRNA |
| ENST00000375650 | HSPA1B | 0.003548815 | 0.138758659 | 1.3957224 | up | mRNA |
| ENST00000375799 | PLEKHM2 | 0.07917152 | 0.482239465 | 1.2509762 | up | mRNA |
| ENST00000375911 | C6orf47 | 0.690927039 | 0.961105466 | 1.1366109 | up | mRNA |
| ENST00000375946 | MASTL | 0.352991896 | 0.83624275 | 1.4344329 | up | mRNA |
| ENST00000375976 | BAG6 | 0.552586388 | 0.926337111 | 1.0527628 | up | mRNA |
| ENST00000375980 | EFHD2 | 0.333966004 | 0.824079937 | 1.3405774 | up | mRNA |
| ENST00000376001 | C9orf153 | 0.009912216 | 0.207085174 | 1.2994466 | up | mRNA |
| ENST00000376072 | NCR3 | 0.066290053 | 0.449693785 | 1.1384667 | up | mRNA |
| ENST00000376093 | LST1 | 0.723050214 | 0.966191059 | 1.0204642 | up | mRNA |
| ENST00000376462 | KIAA1217 | 0.000617545 | 0.074865247 | 3.0708249 | up | mRNA |
| ENST00000376491 | CLCN6 | 0.97052873 | 0.998245244 | 1.0057595 | up | mRNA |
| ENST00000376581 | FARP1 | 0.010335828 | 0.209736088 | 1.076989 | up | mRNA |
| ENST00000376636 | VPS13A | 0.812393811 | 0.98118927 | 1.0336025 | up | mRNA |
| ENST00000376751 | RNF39 | 0.685070734 | 0.960111041 | 1.0407988 | up | mRNA |
| ENST00000376955 | ACBD4 | 0.735747134 | 0.968398491 | 1.0246773 | up | mRNA |
| ENST00000377044 | TMEM2 | 0.04577074 | 0.391180343 | 1.3304217 | up | mRNA |
| ENST00000377095 | SLC25A39 | 0.771884362 | 0.975347132 | 1.0361077 | up | mRNA |
| ENST00000377191 | XRN2 | 0.550481887 | 0.925909806 | 1.0373224 | up | mRNA |
| ENST00000377203 | CD300LG | 0.125910817 | 0.578441772 | 1.0872042 | up | mRNA |
| ENST00000377244 | SNX15 | 0.163962681 | 0.636387539 | 1.1441586 | up | mRNA |
| ENST00000377401 | HIST1H2BL | 0.90734666 | 0.995145546 | 1.0867917 | up | mRNA |
| ENST00000377658 | KLHL21 | 0.058754886 | 0.430953947 | 1.3289015 | up | mRNA |
| ENST00000377694 | IGFBPL1 | 0.77380668 | 0.97589756 | 1.104244 | up | mRNA |
| ENST00000377697 | IGFL4 | 0.481879777 | 0.902005122 | 1.0350463 | up | mRNA |
| ENST00000377726 | KRTAP4-2 | 0.697207181 | 0.962410025 | 1.1590293 | up | mRNA |
| ENST00000377828 | ESPN | 0.328101376 | 0.820939843 | 1.1837646 | up | mRNA |
| ENST00000377867 | ASB10 | 0.479788492 | 0.901538317 | 1.0446503 | up | mRNA |
| ENST00000377942 | PCDH8 | 0.556602282 | 0.926833616 | 1.1510121 | up | mRNA |
| ENST00000378207 | C10orf111 | 0.513022112 | 0.913297047 | 1.0919436 | up | mRNA |
| ENST00000378313 | C19orf54 | 0.455536868 | 0.891148383 | 1.0786088 | up | mRNA |
| ENST00000378455 | BCOR | 0.008613995 | 0.199898185 | 1.4368627 | up | mRNA |
| ENST00000378567 | PRKCZ | 0.054367309 | 0.418393692 | 1.3938549 | up | mRNA |
| ENST00000378719 | SEPT8 | 0.810834309 | 0.98118927 | 1.0411009 | up | mRNA |
| ENST00000378731 | CCNI2 | 0.37389553 | 0.849200616 | 1.0357173 | up | mRNA |
| ENST00000378817 | IL11RA | 0.096325535 | 0.51798222 | 1.1478495 | up | mRNA |
| ENST00000379033 | SEC61A2 | 0.113565006 | 0.557454702 | 1.4224071 | up | mRNA |
| ENST00000379034 | MAGEB6 | 0.661087916 | 0.958354006 | 1.0648319 | up | mRNA |
| ENST00000379119 | BCAP29 | 0.141745933 | 0.60204132 | 1.138611 | up | mRNA |
| ENST00000379133 | C9orf24 | 0.509615099 | 0.911674633 | 1.1156199 | up | mRNA |
| ENST00000379179 | SERP2 | 0.771069395 | 0.975347132 | 1.0555051 | up | mRNA |
| ENST00000379289 | TTLL10 | 0.637992459 | 0.952852353 | 1.1214042 | up | mRNA |
| ENST00000379446 | NEDD9 | 0.016211992 | 0.255838153 | 1.5864845 | up | mRNA |
| ENST00000379555 | FAM170A | 0.874986409 | 0.991094934 | 1.0109879 | up | mRNA |
| ENST00000379558 | FAM179A | 0.529917851 | 0.91899174 | 1.1935603 | up | mRNA |
| ENST00000379600 | NHLRC3 | 0.918317569 | 0.995349793 | 1.0114852 | up | mRNA |
| ENST00000379829 | OR10A4 | 0.106793495 | 0.541480474 | 1.5700348 | up | mRNA |
| ENST00000380056 | SLC4A11 | 0.20244121 | 0.691421917 | 1.1954519 | up | mRNA |
| ENST00000380067 | SLC25A35 | 0.520400787 | 0.916605148 | 1.0467333 | up | mRNA |
| ENST00000380099 | KL | 0.345478493 | 0.832533915 | 1.4107514 | up | mRNA |
| ENST00000380113 | ITPA | 0.899978742 | 0.994752372 | 1.0057834 | up | mRNA |
| ENST00000380219 | OR51B6 | 0.363680508 | 0.843238468 | 1.2276363 | up | mRNA |
| ENST00000380342 | TMEM27 | 0.360724142 | 0.841780454 | 1.1632581 | up | mRNA |
| ENST00000380378 | OR51T1 | 0.314699921 | 0.809824265 | 1.1666935 | up | mRNA |
| ENST00000380409 | RIPK1 | 0.977182125 | 0.998501532 | 1.0017741 | up | mRNA |
| ENST00000380582 | ADRA1A | 0.009397942 | 0.204717305 | 1.2833795 | up | mRNA |
| ENST00000380586 | ADRA1A | 0.467668903 | 0.896909118 | 1.112111 | up | mRNA |
| ENST00000380649 | HADHA | 0.278808721 | 0.777783404 | 1.0494686 | up | mRNA |
| ENST00000380818 | TAF9 | 0.102282287 | 0.531052978 | 1.1403429 | up | mRNA |
| ENST00000380868 | ITSN2 | 0.055858963 | 0.421575513 | 1.1542086 | up | mRNA |
| ENST00000380877 | GABPB1 | 0.062775996 | 0.439458294 | 1.1035984 | up | mRNA |
| ENST00000381175 | TH | 0.451310298 | 0.888854908 | 1.1078597 | up | mRNA |
| ENST00000381178 | TH | 0.065473301 | 0.447162633 | 1.2617541 | up | mRNA |
| ENST00000381583 | SIRPG | 0.886611856 | 0.992981836 | 1.0418272 | up | mRNA |
| ENST00000381652 | JAK2 | 0.351184523 | 0.835187247 | 1.1401832 | up | mRNA |
| ENST00000381661 | ATP6V1C2 | 0.55447186 | 0.926549129 | 1.035758 | up | mRNA |
| ENST00000381781 | ARRDC5 | 0.672356331 | 0.959531882 | 1.0374179 | up | mRNA |
| ENST00000381782 | NSUN7 | 0.54586288 | 0.92424063 | 1.0663199 | up | mRNA |
| ENST00000381799 | RHOH | 0.114358828 | 0.558391858 | 1.2200937 | up | mRNA |
| ENST00000382009 | STIM2 | 0.829155735 | 0.98552522 | 1.0909024 | up | mRNA |
| ENST00000382213 | IL32 | 0.004030199 | 0.145287251 | 3.4579125 | up | mRNA |
| ENST00000382258 | TNFRSF19 | 0.236446692 | 0.734520115 | 1.1150544 | up | mRNA |
| ENST00000382491 | SYNJ1 | 0.941092177 | 0.996710991 | 1.0121966 | up | mRNA |
| ENST00000382675 | C18orf63 | 0.45892989 | 0.892904132 | 1.0727369 | up | mRNA |
| ENST00000382822 | KRTAP19-8 | 0.978369954 | 0.998661138 | 1.0068746 | up | mRNA |
| ENST00000382826 | KRTAP20-3 | 0.27747771 | 0.777234326 | 1.3151487 | up | mRNA |
| ENST00000383640 | OR2H2 | 0.630379127 | 0.950410442 | 1.1170404 | up | mRNA |
| ENST00000383694 | FILIP1L | 0.720682342 | 0.966191059 | 1.147533 | up | mRNA |
| ENST00000383695 | OR5K3 | 0.78477637 | 0.977411899 | 1.0180545 | up | mRNA |
| ENST00000383807 | FANCD2 | 0.841770174 | 0.987123192 | 1.1417622 | up | mRNA |
| ENST00000388820 | ADAMTS7 | 0.771974852 | 0.975347132 | 1.020418 | up | mRNA |
| ENST00000388956 | OTOA | 0.811129105 | 0.98118927 | 1.1051861 | up | mRNA |
| ENST00000389347 | CCDC64B | 0.173525884 | 0.65174838 | 1.122946 | up | mRNA |
| ENST00000389774 | ARHGAP8 | 0.846082326 | 0.987338076 | 1.0193353 | up | mRNA |
| ENST00000389874 | FAM124B | 0.570782444 | 0.93296476 | 1.0448923 | up | mRNA |
| ENST00000389916 | KIF19 | 0.244109641 | 0.742391426 | 1.1283996 | up | mRNA |
| ENST00000391617 | KRTAP12-1 | 0.815014478 | 0.981277402 | 1.0489639 | up | mRNA |
| ENST00000391618 | KRTAP12-4 | 0.449751161 | 0.888279993 | 1.6017847 | up | mRNA |
| ENST00000391796 | CD33 | 0.363265438 | 0.843238468 | 1.1290009 | up | mRNA |
| ENST00000391797 | SIGLEC6 | 0.06866546 | 0.454916653 | 1.109185 | up | mRNA |
| ENST00000391806 | KLK8 | 0.201026749 | 0.689092046 | 1.1623337 | up | mRNA |
| ENST00000391909 | FKRP | 0.797982414 | 0.980749101 | 1.0209626 | up | mRNA |
| ENST00000391951 | GEMIN7 | 0.38227935 | 0.853514109 | 1.0430669 | up | mRNA |
| ENST00000391984 | CAPN10 | 0.175245899 | 0.653821873 | 1.1725851 | up | mRNA |
| ENST00000392043 | ABL2 | 0.142649715 | 0.602733584 | 1.1869977 | up | mRNA |
| ENST00000392079 | NFKBIB | 0.006705073 | 0.183552613 | 1.4597182 | up | mRNA |
| ENST00000392328 | MFSD6 | 0.792382005 | 0.978996327 | 1.1015545 | up | mRNA |
| ENST00000392432 | TMEM44 | 0.500287133 | 0.909680616 | 1.0754713 | up | mRNA |
| ENST00000392473 | CAMKK2 | 0.658900402 | 0.958354006 | 1.0370986 | up | mRNA |
| ENST00000392496 | SPHK1 | 0.821316362 | 0.982756843 | 1.0145734 | up | mRNA |
| ENST00000392588 | WASF1 | 0.943869917 | 0.996710991 | 1.0063597 | up | mRNA |
| ENST00000392863 | C6orf162 | 0.711718634 | 0.96592341 | 1.0435508 | up | mRNA |
| ENST00000392957 | EPHB6 | 0.419112466 | 0.872365335 | 1.1457732 | up | mRNA |
| ENST00000393271 | EME1 | 0.943714759 | 0.996710991 | 1.0260445 | up | mRNA |
| ENST00000393310 | LIMS1 | 0.457008687 | 0.891568474 | 2.2035791 | up | mRNA |
| ENST00000393437 | LONRF2 | 0.577304198 | 0.933656777 | 1.0497789 | up | mRNA |
| ENST00000393450 | MYL4 | 0.137720814 | 0.596789644 | 1.0948335 | up | mRNA |
| ENST00000393704 | MFAP3L | 0.599992203 | 0.940648177 | 1.0433491 | up | mRNA |
| ENST00000393716 | NR1I2 | 0.998224299 | 0.999889342 | 1.0006375 | up | mRNA |
| ENST00000393786 | SRPK3 | 0.670003317 | 0.959531882 | 1.0815134 | up | mRNA |
| ENST00000393934 | PLCXD2 | 0.918887402 | 0.995349793 | 1.0403725 | up | mRNA |
| ENST00000393962 | ANGPTL4 | 0.323083752 | 0.815915892 | 1.4122381 | up | mRNA |
| ENST00000393982 | FABP6 | 0.771945846 | 0.975347132 | 1.0530753 | up | mRNA |
| ENST00000394008 | KRTAP9-9 | 0.271732728 | 0.771737648 | 1.3383187 | up | mRNA |
| ENST00000394019 | SLC4A5 | 0.351584548 | 0.835247013 | 1.2936305 | up | mRNA |
| ENST00000394168 | KIAA0240 | 0.485140259 | 0.903250293 | 1.0409918 | up | mRNA |
| ENST00000394216 | KCNK7 | 0.836395517 | 0.986575022 | 1.0387625 | up | mRNA |
| ENST00000394510 | AKAP13 | 0.147199059 | 0.609417893 | 1.2031146 | up | mRNA |
| ENST00000394571 | PNPLA1 | 0.102459798 | 0.531296253 | 1.0914313 | up | mRNA |
| ENST00000394815 | KRT80 | 0.2180668 | 0.712269655 | 1.0999278 | up | mRNA |
| ENST00000394835 | EFCAB5 | 0.003783052 | 0.142490569 | 1.3527522 | up | mRNA |
| ENST00000394836 | RAB3IL1 | 0.512634653 | 0.913297047 | 1.0927779 | up | mRNA |
| ENST00000394903 | DNAJB12 | 0.26892381 | 0.769564942 | 1.1637326 | up | mRNA |
| ENST00000395238 | IP6K1 | 0.426875273 | 0.876424119 | 1.0902801 | up | mRNA |
| ENST00000395477 | WNK2 | 0.461629831 | 0.894667841 | 1.0743706 | up | mRNA |
| ENST00000395503 | ATP2A1 | 0.976317323 | 0.998501532 | 1.0037261 | up | mRNA |
| ENST00000395696 | CCDC51 | 0.989575207 | 0.999140676 | 1.0037185 | up | mRNA |
| ENST00000395782 | PEMT | 0.552443187 | 0.926337111 | 1.0855704 | up | mRNA |
| ENST00000395810 | RASGEF1A | 0.782066575 | 0.976984292 | 1.0227741 | up | mRNA |
| ENST00000395882 | NTRK2 | 0.122375983 | 0.57455499 | 1.4667073 | up | mRNA |
| ENST00000395891 | HECW1 | 0.02772182 | 0.32381669 | 1.127811 | up | mRNA |
| ENST00000396275 | MRGPRX3 | 0.062764423 | 0.439458294 | 2.1528409 | up | mRNA |
| ENST00000396303 | CYB5R3 | 0.149830149 | 0.614198134 | 1.1357059 | up | mRNA |
| ENST00000396364 | FXN | 0.237882336 | 0.735771951 | 1.1511863 | up | mRNA |
| ENST00000396396 | PGM5 | 0.416204681 | 0.870736736 | 1.0890431 | up | mRNA |
| ENST00000396424 | ZNF665 | 0.244755741 | 0.743408087 | 1.1702021 | up | mRNA |
| ENST00000396432 | ARHGAP21 | 0.007725759 | 0.19345927 | 1.0981847 | up | mRNA |
| ENST00000396435 | IQSEC2 | 0.537781736 | 0.923027035 | 1.2677152 | up | mRNA |
| ENST00000396693 | SEPT12 | 0.627131935 | 0.949218083 | 1.0292293 | up | mRNA |
| ENST00000396720 | GLTSCR1 | 0.417027954 | 0.870760336 | 1.062685 | up | mRNA |
| ENST00000396841 | LEUTX | 0.473122022 | 0.899184677 | 1.0625852 | up | mRNA |
| ENST00000396855 | TRIM5 | 0.942255706 | 0.996710991 | 1.0289285 | up | mRNA |
| ENST00000396857 | MAP4K1 | 0.388749756 | 0.857898453 | 1.0898885 | up | mRNA |
| ENST00000396877 | SPRED3 | 0.923396328 | 0.995456159 | 1.0182439 | up | mRNA |
| ENST00000396901 | NFKBID | 0.534225621 | 0.920652989 | 1.0642295 | up | mRNA |
| ENST00000396916 | HCFC1R1 | 0.464211698 | 0.894989844 | 1.0292984 | up | mRNA |
| ENST00000397049 | NUDT1 | 0.655922346 | 0.957336187 | 1.0266746 | up | mRNA |
| ENST00000397200 | CAPN3 | 0.482715226 | 0.902654749 | 1.2313955 | up | mRNA |
| ENST00000397566 | IRF7 | 0.597615314 | 0.940021241 | 1.0647682 | up | mRNA |
| ENST00000397630 | PRCD | 0.966012121 | 0.99793927 | 1.005629 | up | mRNA |
| ENST00000397820 | C19orf38 | 0.278236287 | 0.777440204 | 1.0967789 | up | mRNA |
| ENST00000397827 | CHRFAM7A | 0.772000902 | 0.975347132 | 1.0307011 | up | mRNA |
| ENST00000397902 | ZNF846 | 0.225885626 | 0.721329755 | 1.0871582 | up | mRNA |
| ENST00000397990 | ANG | 0.815793997 | 0.981459009 | 1.0218463 | up | mRNA |
| ENST00000398022 | TM9SF4 | 0.805451485 | 0.98118927 | 1.0272111 | up | mRNA |
| ENST00000398341 | SLC37A1 | 0.459952933 | 0.893196086 | 1.1540297 | up | mRNA |
| ENST00000398397 | TMPRSS3 | 0.541523683 | 0.923577434 | 1.1127747 | up | mRNA |
| ENST00000398453 | SMAGP | 0.666770501 | 0.958354006 | 1.0201421 | up | mRNA |
| ENST00000398457 | ABCG1 | 0.441523268 | 0.883920737 | 1.1766987 | up | mRNA |
| ENST00000398470 | KRTAP9-1 | 0.096056629 | 0.51782059 | 1.3717051 | up | mRNA |
| ENST00000398492 | PLEKHB1 | 0.905489595 | 0.99508468 | 1.021113 | up | mRNA |
| ENST00000398530 | KRTAP5-11 | 0.294701497 | 0.791301949 | 1.1620368 | up | mRNA |
| ENST00000398531 | KRTAP5-10 | 0.539722316 | 0.923577434 | 1.0813696 | up | mRNA |
| ENST00000398536 | KRTAP5-7 | 0.81492313 | 0.981277402 | 1.0309177 | up | mRNA |
| ENST00000398558 | MFSD12 | 0.47286216 | 0.899184677 | 1.0729246 | up | mRNA |
| ENST00000398610 | PCDHGA10 | 0.069960893 | 0.457987595 | 1.1378694 | up | mRNA |
| ENST00000398718 | DEFB113 | 0.335733177 | 0.825621677 | 1.526085 | up | mRNA |
| ENST00000398754 | WNT8A | 0.118465321 | 0.565973665 | 1.0715973 | up | mRNA |
| ENST00000398774 | PLEC | 0.811173774 | 0.98118927 | 1.0365203 | up | mRNA |
| ENST00000398795 | TNFSF11 | 0.764392088 | 0.973968787 | 1.0279086 | up | mRNA |
| ENST00000398812 | GRB10 | 0.107735558 | 0.544052509 | 1.3263526 | up | mRNA |
| ENST00000399044 | CDRT15L2 | 0.664529008 | 0.958354006 | 1.042126 | up | mRNA |
| ENST00000399206 | C14orf182 | 0.362772959 | 0.843238468 | 1.7358032 | up | mRNA |
| ENST00000399312 | MRPS6 | 0.859955913 | 0.988781331 | 1.0247397 | up | mRNA |
| ENST00000399562 | C22orf39 | 0.939486382 | 0.996710991 | 1.0103764 | up | mRNA |
| ENST00000399598 | UBXN2B | 0.940301616 | 0.996710991 | 1.0171229 | up | mRNA |
| ENST00000399668 | CASC5 | 0.901644838 | 0.994906068 | 1.0579715 | up | mRNA |
| ENST00000399685 | KRTAP5-3 | 0.961055253 | 0.99793927 | 1.0067661 | up | mRNA |
| ENST00000399848 | C18orf1 | 0.688524785 | 0.960626155 | 1.1036734 | up | mRNA |
| ENST00000399875 | CECR6 | 0.665882031 | 0.958354006 | 1.0460136 | up | mRNA |
| ENST00000399916 | RUNX3 | 0.129260684 | 0.583509818 | 1.3070059 | up | mRNA |
| ENST00000399921 | BACH1 | 0.167652464 | 0.642346587 | 1.5816437 | up | mRNA |
| ENST00000399974 | MTRNR2L4 | 0.763022394 | 0.97349057 | 1.093605 | up | mRNA |
| ENST00000400175 | MYL12B | 0.742137826 | 0.970330526 | 1.0400145 | up | mRNA |
| ENST00000400308 | PCBP3 | 0.686463727 | 0.960205549 | 1.0391878 | up | mRNA |
| ENST00000400365 | KRTAP10-12 | 0.176339574 | 0.65545452 | 1.2471682 | up | mRNA |
| ENST00000400368 | KRTAP10-6 | 0.109696963 | 0.548134348 | 1.2179515 | up | mRNA |
| ENST00000400374 | KRTAP10-4 | 0.623079205 | 0.948341211 | 1.1085792 | up | mRNA |
| ENST00000400485 | MORC3 | 0.159438617 | 0.631173277 | 1.204953 | up | mRNA |
| ENST00000400549 | DEFB116 | 0.896278638 | 0.994194586 | 1.0251322 | up | mRNA |
| ENST00000401466 | TMEM177 | 0.800654387 | 0.98118927 | 1.0187554 | up | mRNA |
| ENST00000401975 | GAL3ST1 | 0.456624748 | 0.891568474 | 1.321946 | up | mRNA |
| ENST00000402050 | IQCE | 0.533912519 | 0.920582542 | 1.2509656 | up | mRNA |
| ENST00000402508 | FBXO11 | 0.05937758 | 0.432586127 | 1.1262702 | up | mRNA |
| ENST00000402775 | C2orf54 | 0.984649106 | 0.998971807 | 1.0021055 | up | mRNA |
| ENST00000402966 | C22orf46 | 0.007862435 | 0.1942394 | 1.1809113 | up | mRNA |
| ENST00000402988 | IGSF23 | 0.57714115 | 0.933656777 | 1.0336288 | up | mRNA |
| ENST00000403263 | INO80D | 0.071389811 | 0.461819164 | 1.4049631 | up | mRNA |
| ENST00000404801 | ZNF792 | 0.822486188 | 0.983172978 | 1.0233894 | up | mRNA |
| ENST00000405044 | TBC1D28 | 0.672272275 | 0.959531882 | 1.0832275 | up | mRNA |
| ENST00000405093 | MYH6 | 0.413175118 | 0.869509111 | 1.0920229 | up | mRNA |
| ENST00000405148 | ALS2CR12 | 0.953532995 | 0.996710991 | 1.0157478 | up | mRNA |
| ENST00000405256 | KBTBD12 | 0.587067577 | 0.93579682 | 1.0837216 | up | mRNA |
| ENST00000405617 | PPP1R3G | 0.378362486 | 0.851828819 | 1.2155607 | up | mRNA |
| ENST00000406427 | PNPLA7 | 0.42949328 | 0.878226842 | 1.1259057 | up | mRNA |
| ENST00000406659 | DNMT3A | 0.616678165 | 0.945912508 | 1.0427416 | up | mRNA |
| ENST00000407280 | IFI30 | 0.844048475 | 0.987338076 | 1.0144671 | up | mRNA |
| ENST00000407327 | SLC44A2 | 0.98412852 | 0.998971807 | 1.0027109 | up | mRNA |
| ENST00000407997 | APOBEC3G | 0.365676005 | 0.845038366 | 1.1952222 | up | mRNA |
| ENST00000408887 | C12orf61 | 0.018127165 | 0.269217741 | 1.2284716 | up | mRNA |
| ENST00000408907 | PSMB11 | 0.664908346 | 0.958354006 | 1.0475005 | up | mRNA |
| ENST00000408957 | TIGD1 | 0.068173647 | 0.45284301 | 1.1048389 | up | mRNA |
| ENST00000409168 | SHISA6 | 0.281001142 | 0.779126326 | 1.253326 | up | mRNA |
| ENST00000409178 | C10orf55 | 0.140950163 | 0.601174472 | 1.7028669 | up | mRNA |
| ENST00000409445 | CAPS2 | 0.742364262 | 0.970330526 | 1.0821054 | up | mRNA |
| ENST00000409600 | BZW1 | 0.741994609 | 0.970330526 | 1.0861475 | up | mRNA |
| ENST00000409817 | CXCR4 | 0.328949895 | 0.821748281 | 1.1349323 | up | mRNA |
| ENST00000409858 | UAP1L1 | 0.406266124 | 0.866939845 | 1.0705442 | up | mRNA |
| ENST00000410111 | IMMT | 0.638161328 | 0.952852353 | 1.0390086 | up | mRNA |
| ENST00000411432 | METTL21A | 0.115158937 | 0.560548603 | 1.5231669 | up | mRNA |
| ENST00000411851 | TBXA2R | 0.93370115 | 0.996710991 | 1.0070156 | up | mRNA |
| ENST00000411896 | GRAMD1A | 0.200886756 | 0.68900633 | 1.1482457 | up | mRNA |
| ENST00000412135 | FGFR3 | 0.955064147 | 0.996883425 | 1.0185014 | up | mRNA |
| ENST00000412468 | SLC35G6 | 0.806486537 | 0.98118927 | 1.0797822 | up | mRNA |
| ENST00000412663 | SPOCK2 | 0.427851668 | 0.876992614 | 1.031673 | up | mRNA |
| ENST00000412723 | PIGZ | 0.659023034 | 0.958354006 | 1.030837 | up | mRNA |
| ENST00000412819 | GSN | 0.829021349 | 0.98552522 | 1.1682312 | up | mRNA |
| ENST00000413384 | SLC4A2 | 0.987635666 | 0.999140676 | 1.0043248 | up | mRNA |
| ENST00000413580 | PHOSPHO1 | 0.346180701 | 0.833163669 | 1.0827682 | up | mRNA |
| ENST00000413689 | SCN5A | 0.198351568 | 0.686799061 | 1.295731 | up | mRNA |
| ENST00000413817 | FAM116B | 0.398657548 | 0.863643939 | 1.0861087 | up | mRNA |
| ENST00000413988 | EXOC3L2 | 0.583604859 | 0.934280817 | 1.0683379 | up | mRNA |
| ENST00000415440 | ZNF101 | 0.625567994 | 0.948851902 | 1.1413971 | up | mRNA |
| ENST00000415846 | SLFN14 | 0.011865145 | 0.221526283 | 1.715586 | up | mRNA |
| ENST00000415930 | GPI | 0.335378207 | 0.825134282 | 1.0815996 | up | mRNA |
| ENST00000415950 | SCN1B | 0.753046633 | 0.970725371 | 1.0214581 | up | mRNA |
| ENST00000416165 | BAHD1 | 0.269911441 | 0.770082629 | 1.2436286 | up | mRNA |
| ENST00000416383 | C12orf70 | 0.227273675 | 0.72290573 | 1.2166173 | up | mRNA |
| ENST00000416767 | CACNA1G | 0.085933558 | 0.498103045 | 1.134078 | up | mRNA |
| ENST00000417066 | CCDC148 | 0.94784783 | 0.996710991 | 1.0284152 | up | mRNA |
| ENST00000417700 | FAM82A1 | 0.567953658 | 0.931705674 | 1.0482928 | up | mRNA |
| ENST00000418107 | HLA-DMB | 0.402855944 | 0.864786348 | 1.0956035 | up | mRNA |
| ENST00000418434 | CASP5 | 0.091659152 | 0.510719066 | 1.2389207 | up | mRNA |
| ENST00000418609 | SORBS2 | 0.594211769 | 0.938369994 | 1.1998799 | up | mRNA |
| ENST00000418635 | PPARD | 0.003388027 | 0.137400229 | 1.7820469 | up | mRNA |
| ENST00000419275 | PPIAL4G | 0.920200515 | 0.995349793 | 1.0057925 | up | mRNA |
| ENST00000419307 | DFNA5 | 0.541737875 | 0.923577434 | 1.1346096 | up | mRNA |
| ENST00000419421 | PRR22 | 0.557478611 | 0.926833616 | 1.065774 | up | mRNA |
| ENST00000419445 | PGGT1B | 0.004571671 | 0.155014562 | 1.2404506 | up | mRNA |
| ENST00000421133 | CD33 | 0.695276485 | 0.962311878 | 1.0211014 | up | mRNA |
| ENST00000421307 | LRRFIP2 | 0.921206207 | 0.995349793 | 1.0070649 | up | mRNA |
| ENST00000421453 | HSF4 | 0.372470148 | 0.84763089 | 1.0610099 | up | mRNA |
| ENST00000422127 | OBSCN | 0.086752775 | 0.499950487 | 1.1402805 | up | mRNA |
| ENST00000422354 | MEGF11 | 0.551489625 | 0.926149092 | 1.0740762 | up | mRNA |
| ENST00000422373 | FLNA | 0.153849137 | 0.620938377 | 1.1945829 | up | mRNA |
| ENST00000422986 | KLK3 | 0.167564132 | 0.642346587 | 1.1803531 | up | mRNA |
| ENST00000423118 | PKD1 | 0.973417642 | 0.998501532 | 1.002043 | up | mRNA |
| ENST00000423828 | TMBIM6 | 0.964962262 | 0.99793927 | 1.0056234 | up | mRNA |
| ENST00000424279 | TBL1X | 0.850996602 | 0.987678166 | 1.0182454 | up | mRNA |
| ENST00000424296 | AKD1 | 0.892970573 | 0.993978977 | 1.0189323 | up | mRNA |
| ENST00000424843 | LRP12 | 0.052208531 | 0.412074478 | 1.2182706 | up | mRNA |
| ENST00000424930 | ZNF419 | 0.291485501 | 0.787986325 | 1.3338435 | up | mRNA |
| ENST00000425343 | PGC | 0.348481895 | 0.834138149 | 1.0902653 | up | mRNA |
| ENST00000425534 | EIF4E3 | 0.403787455 | 0.865367868 | 1.0554343 | up | mRNA |
| ENST00000425967 | FGFR1 | 0.67333747 | 0.959531882 | 1.0698494 | up | mRNA |
| ENST00000426057 | TRPC7 | 0.343001093 | 0.831521302 | 1.2108442 | up | mRNA |
| ENST00000426613 | MAMLD1 | 0.109469806 | 0.547537537 | 1.3125024 | up | mRNA |
| ENST00000427390 | GOLGA6L6 | 0.094439856 | 0.515476821 | 1.1085258 | up | mRNA |
| ENST00000427411 | ACE2 | 0.56067422 | 0.927930666 | 1.0772903 | up | mRNA |
| ENST00000427414 | SH3D19 | 0.902487229 | 0.99508468 | 1.0064746 | up | mRNA |
| ENST00000427500 | GBA | 0.713432894 | 0.966110889 | 1.0235336 | up | mRNA |
| ENST00000427704 | PHACTR2 | 0.327149952 | 0.820196554 | 1.1246863 | up | mRNA |
| ENST00000428395 | FYTTD1 | 0.976695077 | 0.998501532 | 1.0024134 | up | mRNA |
| ENST00000429149 | IL34 | 0.33085905 | 0.821853462 | 1.2331566 | up | mRNA |
| ENST00000429416 | SMARCA4 | 0.742722732 | 0.970330526 | 1.0299969 | up | mRNA |
| ENST00000429584 | YY2 | 0.431709564 | 0.880121832 | 1.13638 | up | mRNA |
| ENST00000430046 | FCHO2 | 0.741152856 | 0.970205535 | 1.0340231 | up | mRNA |
| ENST00000430130 | LRRD1 | 0.568989156 | 0.93232103 | 1.1008316 | up | mRNA |
| ENST00000430325 | CNTD2 | 0.857370795 | 0.988512246 | 1.0138999 | up | mRNA |
| ENST00000430948 | KIAA0319 | 0.967482687 | 0.99793927 | 1.0097257 | up | mRNA |
| ENST00000431010 | HRH1 | 0.136791099 | 0.595718028 | 1.1948293 | up | mRNA |
| ENST00000431468 | SEBOX | 0.946965982 | 0.996710991 | 1.0041252 | up | mRNA |
| ENST00000431717 | C3orf52 | 0.072432707 | 0.463255488 | 1.5597839 | up | mRNA |
| ENST00000431922 | LPAR5 | 0.406885961 | 0.867591329 | 1.1450314 | up | mRNA |
| ENST00000432069 | NOL3 | 0.977844259 | 0.998570038 | 1.0028088 | up | mRNA |
| ENST00000432597 | SYCE1 | 0.410400841 | 0.86807846 | 1.1283245 | up | mRNA |
| ENST00000432825 | FBXO10 | 0.455514871 | 0.891148383 | 1.0795399 | up | mRNA |
| ENST00000432826 | TARM1 | 0.069317995 | 0.456847153 | 1.1578838 | up | mRNA |
| ENST00000432829 | DOCK8 | 0.276779893 | 0.777062798 | 1.1370818 | up | mRNA |
| ENST00000433501 | SLC25A25 | 0.371092359 | 0.846402038 | 1.1396067 | up | mRNA |
| ENST00000433651 | ANKFY1 | 0.616863978 | 0.945960598 | 1.0322705 | up | mRNA |
| ENST00000434452 | DSC3 | 0.716400054 | 0.966191059 | 1.0434697 | up | mRNA |
| ENST00000434505 | CKMT1A | 0.127233662 | 0.580546411 | 1.2852942 | up | mRNA |
| ENST00000434603 | MAP6 | 0.874581011 | 0.990905383 | 1.0400903 | up | mRNA |
| ENST00000434608 | CCK | 0.240396434 | 0.738437735 | 1.1199086 | up | mRNA |
| ENST00000434667 | SCAF4 | 0.911730258 | 0.995342392 | 1.0073871 | up | mRNA |
| ENST00000435039 | C1QTNF9B-AS1 | 0.65880975 | 0.958354006 | 1.0194811 | up | mRNA |
| ENST00000435070 | CPSF6 | 0.853263007 | 0.987678166 | 1.0185729 | up | mRNA |
| ENST00000435159 | TMEM132C | 0.8099173 | 0.98118927 | 1.093241 | up | mRNA |
| ENST00000435205 | FAM160A1 | 0.1566017 | 0.625226111 | 1.2621846 | up | mRNA |
| ENST00000435432 | NEDD4L | 0.087579477 | 0.501313605 | 1.1539732 | up | mRNA |
| ENST00000435594 | IDH3B | 0.484306196 | 0.903250293 | 1.0954288 | up | mRNA |
| ENST00000435606 | C18orf1 | 0.594765277 | 0.938655001 | 1.0868475 | up | mRNA |
| ENST00000436022 | CCDC48 | 0.325177207 | 0.817547048 | 1.1989691 | up | mRNA |
| ENST00000436235 | TAC4 | 0.616977351 | 0.945960598 | 1.0585138 | up | mRNA |
| ENST00000436881 | C9orf172 | 0.275950663 | 0.775741613 | 1.1828775 | up | mRNA |
| ENST00000437464 | ZNF469 | 0.320627976 | 0.814552264 | 1.2838956 | up | mRNA |
| ENST00000437550 | HCST | 0.0197707 | 0.282883797 | 1.1438059 | up | mRNA |
| ENST00000437936 | IGFL1 | 0.332409065 | 0.822707947 | 1.4241336 | up | mRNA |
| ENST00000438075 | LST1 | 0.023920754 | 0.303389731 | 1.1834098 | up | mRNA |
| ENST00000438167 | PLA2G10 | 0.725947179 | 0.966191059 | 1.0385173 | up | mRNA |
| ENST00000438693 | TMEM150B | 0.92355098 | 0.995456159 | 1.0180956 | up | mRNA |
| ENST00000439055 | ACOXL | 0.57675171 | 0.933656777 | 1.0820855 | up | mRNA |
| ENST00000439329 | CPB2 | 0.134349783 | 0.590871682 | 1.2795136 | up | mRNA |
| ENST00000439350 | ATP6AP1L | 0.626690474 | 0.949203235 | 1.1857741 | up | mRNA |
| ENST00000439569 | SLFNL1 | 0.754732829 | 0.971278427 | 1.0556857 | up | mRNA |
| ENST00000439817 | MGEA5 | 0.46918761 | 0.897810636 | 1.0546584 | up | mRNA |
| ENST00000440021 | FBXO34 | 0.553559405 | 0.926337111 | 1.0470097 | up | mRNA |
| ENST00000440486 | FGFR3 | 0.055644532 | 0.42131872 | 1.1387802 | up | mRNA |
| ENST00000441065 | RFPL4B | 1.943E-05 | 0.018978458 | 18.9975614 | up | mRNA |
| ENST00000441328 | AQP1 | 0.192664648 | 0.679358478 | 1.1133573 | up | mRNA |
| ENST00000441508 | C10orf105 | 0.27272768 | 0.77262076 | 1.1280054 | up | mRNA |
| ENST00000441525 | DCAF8L1 | 0.26475458 | 0.76613535 | 1.5892863 | up | mRNA |
| ENST00000441606 | ANO6 | 0.216752791 | 0.711074992 | 1.0721078 | up | mRNA |
| ENST00000442003 | CPEB2 | 0.016735966 | 0.25915892 | 1.2797698 | up | mRNA |
| ENST00000442263 | INTS6 | 0.238904223 | 0.736872476 | 1.594464 | up | mRNA |
| ENST00000442312 | SH3BP2 | 0.726752802 | 0.966191059 | 1.1049831 | up | mRNA |
| ENST00000442340 | TBX22 | 0.097253476 | 0.519831481 | 1.2017754 | up | mRNA |
| ENST00000442650 | GSTM2 | 0.830983633 | 0.985710869 | 1.0474925 | up | mRNA |
| ENST00000443581 | SCN5A | 0.008252654 | 0.197897997 | 1.1967779 | up | mRNA |
| ENST00000444303 | AMPD3 | 0.00297013 | 0.129868122 | 3.2434774 | up | mRNA |
| ENST00000444484 | BCL2 | 0.173661971 | 0.651863583 | 1.2166657 | up | mRNA |
| ENST00000444740 | CD79A | 0.409995935 | 0.86807846 | 1.1340638 | up | mRNA |
| ENST00000444761 | MCFD2 | 0.83860632 | 0.98694974 | 1.0184172 | up | mRNA |
| ENST00000445167 | SLC35A2 | 0.260097414 | 0.760388386 | 1.1869608 | up | mRNA |
| ENST00000445202 | PATE3 | 0.620742819 | 0.947874051 | 1.0928 | up | mRNA |
| ENST00000445470 | CPA4 | 0.618568853 | 0.946956498 | 1.1491426 | up | mRNA |
| ENST00000445694 | ADAM29 | 0.886139154 | 0.992981836 | 1.0074549 | up | mRNA |
| ENST00000446176 | FNBP1 | 0.046398952 | 0.392259245 | 1.3320764 | up | mRNA |
| ENST00000446461 | IQCF5 | 0.642914984 | 0.954522001 | 1.0956086 | up | mRNA |
| ENST00000447255 | NCR1 | 0.378316572 | 0.851828819 | 1.2267577 | up | mRNA |
| ENST00000447857 | CCDC155 | 0.700374859 | 0.962991468 | 1.0481968 | up | mRNA |
| ENST00000448077 | PPARD | 0.216713878 | 0.711074992 | 1.1364131 | up | mRNA |
| ENST00000448372 | SENP1 | 0.190451858 | 0.676402188 | 1.3892399 | up | mRNA |
| ENST00000448662 | SORBS2 | 0.545111995 | 0.924012779 | 1.3825062 | up | mRNA |
| ENST00000448907 | ELAVL4 | 0.657511361 | 0.957904915 | 1.0383975 | up | mRNA |
| ENST00000448933 | SEPT8 | 0.27218664 | 0.772141487 | 1.086713 | up | mRNA |
| ENST00000448943 | TWIST2 | 0.975280413 | 0.998501532 | 1.0072599 | up | mRNA |
| ENST00000449195 | IL4R | 0.67349429 | 0.959531882 | 1.0125368 | up | mRNA |
| ENST00000449498 | IFNA13 | 0.75803557 | 0.972072638 | 1.0922775 | up | mRNA |
| ENST00000449880 | ITGB4 | 0.690976369 | 0.961105466 | 1.0340409 | up | mRNA |
| ENST00000449912 | TRPM6 | 0.032689825 | 0.344901399 | 1.1287508 | up | mRNA |
| ENST00000450244 | SCN11A | 0.903150884 | 0.99508468 | 1.0106266 | up | mRNA |
| ENST00000450269 | BTBD19 | 0.028146668 | 0.326447555 | 1.549868 | up | mRNA |
| ENST00000450331 | PNPLA6 | 0.223153503 | 0.71761506 | 1.1563768 | up | mRNA |
| ENST00000450448 | EFCAB4A | 0.441407128 | 0.883920737 | 1.0347034 | up | mRNA |
| ENST00000451626 | CEACAM18 | 0.283176771 | 0.780770837 | 1.1292001 | up | mRNA |
| ENST00000451767 | C20orf26 | 0.300182328 | 0.795797471 | 1.1796509 | up | mRNA |
| ENST00000452699 | ATP13A2 | 0.685283164 | 0.960111041 | 1.0190016 | up | mRNA |
| ENST00000452783 | SUN1 | 0.835348177 | 0.986575022 | 1.0206516 | up | mRNA |
| ENST00000453464 | RNF223 | 0.317249423 | 0.811824899 | 1.2196456 | up | mRNA |
| ENST00000454345 | DGKZ | 0.139434896 | 0.598648178 | 1.1974554 | up | mRNA |
| ENST00000455191 | IQCG | 0.211277728 | 0.702600386 | 1.240056 | up | mRNA |
| ENST00000455515 | FXYD1 | 0.774498122 | 0.97589756 | 1.0416093 | up | mRNA |
| ENST00000455836 | ERP29 | 0.964491336 | 0.99793927 | 1.0063755 | up | mRNA |
| ENST00000456013 | PTPN6 | 0.149848425 | 0.614198134 | 1.5591938 | up | mRNA |
| ENST00000457378 | SUN1 | 0.706023682 | 0.965098393 | 1.0401633 | up | mRNA |
| ENST00000457424 | CEP44 | 0.309226729 | 0.803961878 | 1.1962743 | up | mRNA |
| ENST00000457476 | TANK | 0.677729692 | 0.959531882 | 1.0578284 | up | mRNA |
| ENST00000457542 | MAPKBP1 | 0.154819003 | 0.622213869 | 1.239589 | up | mRNA |
| ENST00000457772 | ST6GAL1 | 0.30151929 | 0.796581367 | 1.0544411 | up | mRNA |
| ENST00000457828 | LSS | 0.2213022 | 0.716734257 | 1.1173299 | up | mRNA |
| ENST00000458008 | FAM86A | 0.927756087 | 0.99588917 | 1.0160185 | up | mRNA |
| ENST00000458128 | PGAM4 | 0.731614214 | 0.967176866 | 1.0415555 | up | mRNA |
| ENST00000458322 | NDOR1 | 0.296187138 | 0.79250767 | 1.0504783 | up | mRNA |
| ENST00000458427 | JRKL | 0.257442849 | 0.757735833 | 1.057042 | up | mRNA |
| ENST00000458694 | PRICKLE4 | 0.509833387 | 0.911771697 | 1.050237 | up | mRNA |
| ENST00000471652 | ZC3HAV1 | 0.015658433 | 0.251276455 | 1.5477527 | up | mRNA |
| ENST00000472374 | CENPM | 0.922771899 | 0.995349793 | 1.0055485 | up | mRNA |
| ENST00000474759 | CACNA2D3 | 0.60048621 | 0.940752476 | 1.0510567 | up | mRNA |
| ENST00000478945 | DPPA2 | 0.894318044 | 0.993978977 | 1.0265761 | up | mRNA |
| ENST00000490841 | ABI1 | 0.372629234 | 0.847650212 | 1.1443518 | up | mRNA |
| ENST00000493151 | NOS1AP | 0.003011489 | 0.130211341 | 1.102009 | up | mRNA |
| ENST00000500576 | LYRM4 | 0.013085821 | 0.233796941 | 1.3007283 | up | mRNA |
| ENST00000502536 | OSMR | 0.343227775 | 0.831521302 | 1.1791858 | up | mRNA |
| ENST00000505920 | ATP5L2 | 0.534272917 | 0.920652989 | 1.0380388 | up | mRNA |
| ENST00000518069 | PCDHGA5 | 0.794019037 | 0.97921643 | 1.0479022 | up | mRNA |
| ENST00000518157 | SBSN | 0.462238874 | 0.894765983 | 1.474493 | up | mRNA |
| ENST00000519574 | GPBAR1 | 0.884447327 | 0.99276855 | 1.0185378 | up | mRNA |
| ENST00000520790 | PCDHGB6 | 0.306156088 | 0.801520161 | 1.061829 | up | mRNA |
| ENST00000521992 | CES5A | 0.327784017 | 0.820730147 | 1.1131354 | up | mRNA |
| ENST00000522353 | PCDHA3 | 0.299003302 | 0.795233272 | 1.2669299 | up | mRNA |
| ENST00000522605 | PCDHGB2 | 0.957481706 | 0.996883425 | 1.007222 | up | mRNA |
| ENST00000523079 | NRG1 | 0.759540541 | 0.972724759 | 1.112921 | up | mRNA |
| ENST00000523207 | KCNB2 | 0.838352538 | 0.986897498 | 1.0111115 | up | mRNA |
| ENST00000523390 | PCDHGB1 | 0.954947153 | 0.996883425 | 1.0083284 | up | mRNA |
| ENST00000525815 | SLC35F2 | 0.562978129 | 0.929041598 | 1.0802829 | up | mRNA |
| ENST00000525929 | PCDHA7 | 0.99741204 | 0.999889342 | 1.0005526 | up | mRNA |
| ENST00000526441 | TBX5 | 0.418870736 | 0.872328878 | 1.1605005 | up | mRNA |
| ENST00000528743 | KRTAP5-9 | 0.508264639 | 0.911674633 | 1.3603532 | up | mRNA |
| ENST00000528954 | LPXN | 0.017934435 | 0.269173581 | 1.6307527 | up | mRNA |
| ENST00000529859 | PCDHA5 | 0.346474558 | 0.833284666 | 1.7893733 | up | mRNA |
| ENST00000530656 | PRDM11 | 0.845288145 | 0.987338076 | 1.0190454 | up | mRNA |
| ENST00000531452 | RNF214 | 0.855542059 | 0.988178307 | 1.0623609 | up | mRNA |
| ENST00000534062 | RTL1 | 0.478602735 | 0.901271944 | 1.0516799 | up | mRNA |
| ENST00000535316 | USP6NL | 0.323618473 | 0.816213518 | 1.1391029 | up | mRNA |
| ENST00000535549 | ETS1 | 4.52257E-06 | 0.010216988 | 1.9612647 | up | mRNA |
| ENST00000536133 | CXorf56 | 0.91186627 | 0.995342392 | 1.017966 | up | mRNA |
| ENST00000536621 | MUC12 | 0.707434961 | 0.965401045 | 1.0510124 | up | mRNA |
| ENST00000536684 | MTRNR2L8 | 0.963518089 | 0.99793927 | 1.0026613 | up | mRNA |
| ENST00000537427 | PNPLA4 | 0.99987408 | 0.999954265 | 1.0000388 | up | mRNA |
| ENST00000537469 | IQCC | 0.729342022 | 0.966191059 | 1.0317242 | up | mRNA |
| ENST00000537559 | VEPH1 | 0.134270123 | 0.590871682 | 1.2158147 | up | mRNA |
| ENST00000537561 | NUDT16 | 0.749204154 | 0.970725371 | 1.028167 | up | mRNA |
| ENST00000538197 | CPEB2 | 0.035142197 | 0.352396712 | 1.6449597 | up | mRNA |
| ENST00000538371 | PODNL1 | 0.116979181 | 0.56398437 | 1.0760594 | up | mRNA |
| ENST00000538517 | PODNL1 | 0.957065655 | 0.996883425 | 1.0089068 | up | mRNA |
| ENST00000538575 | PRRG3 | 0.986386525 | 0.998971807 | 1.0050477 | up | mRNA |
| ENST00000538775 | NCEH1 | 0.873140541 | 0.990580595 | 1.0637481 | up | mRNA |
| ENST00000538962 | PTGR1 | 0.853126647 | 0.987678166 | 1.0378231 | up | mRNA |
| ENST00000538966 | PLXNB3 | 0.763493678 | 0.973567683 | 1.0226461 | up | mRNA |
| ENST00000539099 | FLT1 | 0.300414712 | 0.795797471 | 1.6352825 | up | mRNA |
| ENST00000539115 | ZFX | 0.749626308 | 0.970725371 | 1.1743225 | up | mRNA |
| ENST00000539718 | CD300LG | 0.911314763 | 0.995342392 | 1.0135929 | up | mRNA |
| ENST00000539851 | GPR137 | 0.486367201 | 0.903524001 | 1.0470777 | up | mRNA |
| ENST00000540010 | TRAF1 | 0.000988537 | 0.085705167 | 6.1999299 | up | mRNA |
| ENST00000540040 | MTRNR2L1 | 0.260795046 | 0.761523031 | 1.0294747 | up | mRNA |
| ENST00000540144 | HIST1H3C | 0.175996904 | 0.655114384 | 1.205663 | up | mRNA |
| ENST00000540147 | ERBB2 | 0.249466801 | 0.747444593 | 1.0799631 | up | mRNA |
| ENST00000540430 | LDHA | 0.02508712 | 0.309509304 | 1.8819437 | up | mRNA |
| ENST00000540522 | FAM157B | 0.001762235 | 0.105670067 | 1.0938743 | up | mRNA |
| ENST00000540550 | TSFM | 0.873458722 | 0.990580595 | 1.0151476 | up | mRNA |
| ENST00000540734 | RPAIN | 0.971958578 | 0.998501532 | 1.0205566 | up | mRNA |
| ENST00000540800 | NHSL2 | 0.559079517 | 0.927203296 | 1.0887929 | up | mRNA |
| ENST00000541112 | CPEB2 | 0.041261795 | 0.376372733 | 1.5898175 | up | mRNA |
| ENST00000541323 | PDGFRL | 0.87048484 | 0.989592579 | 1.093681 | up | mRNA |
| ENST00000541337 | NRG2 | 0.702462002 | 0.963399221 | 1.1007766 | up | mRNA |
| ENST00000541932 | FLT1 | 0.996643743 | 0.999872237 | 1.0006793 | up | mRNA |
| ENST00000542104 | RASSF3 | 0.531900265 | 0.920228311 | 1.0699646 | up | mRNA |
| ENST00000542437 | CCDC166 | 0.032163805 | 0.3425639 | 1.3873364 | up | mRNA |
| ENST00000542569 | SAMD8 | 0.988141187 | 0.999140676 | 1.0015293 | up | mRNA |
| ENST00000542643 | RAP1GAP | 0.219546587 | 0.714276371 | 1.0933052 | up | mRNA |
| ENST00000542713 | FKBP5 | 0.112015212 | 0.554156842 | 2.0646172 | up | mRNA |
| ENST00000543261 | DSTN | 0.545632049 | 0.924167487 | 1.0593581 | up | mRNA |
| ENST00000543264 | SHD | 0.001614603 | 0.103549765 | 1.6025226 | up | mRNA |
| ENST00000543409 | MNX1 | 0.969382759 | 0.998245244 | 1.0050448 | up | mRNA |
| ENST00000543727 | TSFM | 0.58067249 | 0.93393768 | 1.0936055 | up | mRNA |
| ENST00000545051 | SMAD7 | 0.033775218 | 0.348587681 | 1.2392878 | up | mRNA |
| ENST00000545060 | SLC13A2 | 0.525432862 | 0.917686109 | 1.2601528 | up | mRNA |
| ENST00000545103 | UBAP1 | 0.762710355 | 0.973443358 | 1.0266636 | up | mRNA |
| ENST00000545121 | CEPT1 | 0.713838865 | 0.966110889 | 1.0299935 | up | mRNA |
| ENST00000545128 | PCSK5 | 0.848081716 | 0.987678166 | 1.0680642 | up | mRNA |
| ENST00000545172 | ADCY10 | 0.249102552 | 0.747319103 | 1.319777 | up | mRNA |
| ENST00000545187 | ISYNA1 | 0.171582605 | 0.649286717 | 1.2534648 | up | mRNA |
| ENST00000545201 | PNPLA6 | 0.032075504 | 0.342493703 | 1.1542694 | up | mRNA |
| ENST00000546084 | TRAF1 | 0.001014561 | 0.085705167 | 8.7174763 | up | mRNA |
| ENST00000550422 | ADCY6 | 0.665368612 | 0.958354006 | 1.0391708 | up | mRNA |
| ENST00000554012 | TCL1A | 0.154224716 | 0.621674306 | 1.1374021 | up | mRNA |
| ENST00000556084 | PROX2 | 0.04882875 | 0.40128453 | 1.4083444 | up | mRNA |
| ENST00000559471 | SECISBP2L | 0.456558118 | 0.891568474 | 1.1213964 | up | mRNA |
| ENST00000563496 | TCF24 | 0.560036356 | 0.927772696 | 1.1408088 | up | mRNA |
| ENST00000568335 | TMEM114 | 0.873333101 | 0.990580595 | 1.0128545 | up | mRNA |
| ENST00000569714 | RNF151 | 0.984791641 | 0.998971807 | 1.0040341 | up | mRNA |
| ENST00000574641 | OCLM | 0.420880554 | 0.872753026 | 1.0565406 | up | mRNA |
| NM_000014 | A2M | 0.121032744 | 0.572400147 | 1.25792 | up | mRNA |
| NM_000021 | PSEN1 | 0.012113073 | 0.222675671 | 1.4531345 | up | mRNA |
| NM_000023 | SGCA | 0.408760653 | 0.867591329 | 1.1080759 | up | mRNA |
| NM_000025 | ADRB3 | 0.249041679 | 0.747319103 | 1.2977709 | up | mRNA |
| NM_000035 | ALDOB | 0.079751942 | 0.483313408 | 1.1529237 | up | mRNA |
| NM_000036 | AMPD1 | 0.451353216 | 0.888854908 | 1.2195016 | up | mRNA |
| NM_000039 | APOA1 | 0.80243472 | 0.98118927 | 1.0258415 | up | mRNA |
| NM_000041 | APOE | 0.885703945 | 0.992981836 | 1.0348337 | up | mRNA |
| NM_000043 | FAS | 0.993380953 | 0.999872237 | 1.0012542 | up | mRNA |
| NM_000045 | ARG1 | 0.540959746 | 0.923577434 | 1.1680505 | up | mRNA |
| NM_000046 | ARSB | 0.856244837 | 0.988315074 | 1.0081704 | up | mRNA |
| NM_000052 | ATP7A | 0.180054471 | 0.662747609 | 1.1647494 | up | mRNA |
| NM_000053 | ATP7B | 0.805362882 | 0.98118927 | 1.0228185 | up | mRNA |
| NM_000055 | BCHE | 0.320803837 | 0.814711917 | 1.6348476 | up | mRNA |
| NM_000059 | BRCA2 | 0.25658068 | 0.757240077 | 1.1395845 | up | mRNA |
| NM_000064 | C3 | 0.275902365 | 0.775741613 | 1.6192228 | up | mRNA |
| NM_000069 | CACNA1S | 0.836063373 | 0.986575022 | 1.0294517 | up | mRNA |
| NM_000077 | CDKN2A | 0.270921608 | 0.770905786 | 1.0764993 | up | mRNA |
| NM_000081 | LYST | 0.507499579 | 0.911674633 | 1.1299865 | up | mRNA |
| NM_000085 | CLCNKB | 0.007663343 | 0.193257016 | 1.7954825 | up | mRNA |
| NM_000094 | COL7A1 | 0.549693321 | 0.925533435 | 1.0397171 | up | mRNA |
| NM_000099 | CST3 | 0.967819889 | 0.99793927 | 1.0024718 | up | mRNA |
| NM_000103 | CYP19A1 | 0.655060342 | 0.957205183 | 1.0899927 | up | mRNA |
| NM_000111 | SLC26A3 | 0.684483457 | 0.960111041 | 1.0930789 | up | mRNA |
| NM_000119 | EPB42 | 0.052149145 | 0.411765599 | 1.1257396 | up | mRNA |
| NM_000120 | EPHX1 | 0.247007118 | 0.745152522 | 1.1344003 | up | mRNA |
| NM_000123 | ERCC5 | 0.801712068 | 0.98118927 | 1.0532315 | up | mRNA |
| NM_000126 | ETFA | 0.917914258 | 0.995349793 | 1.0056628 | up | mRNA |
| NM_000127 | EXT1 | 0.13235208 | 0.587843174 | 1.2344321 | up | mRNA |
| NM_000131 | F7 | 0.396561802 | 0.861995469 | 1.109855 | up | mRNA |
| NM_000132 | F8 | 0.786890634 | 0.977479172 | 1.0219557 | up | mRNA |
| NM_000137 | FAH | 0.492646054 | 0.906047853 | 1.114577 | up | mRNA |
| NM_000143 | FH | 0.567001649 | 0.931483408 | 1.0413797 | up | mRNA |
| NM_000144 | FXN | 0.506823774 | 0.911517619 | 1.0385308 | up | mRNA |
| NM_000146 | FTL | 0.889230351 | 0.993561109 | 1.046503 | up | mRNA |
| NM_000157 | GBA | 0.312904592 | 0.808280545 | 1.1511009 | up | mRNA |
| NM_000164 | GIPR | 0.456079964 | 0.891365829 | 1.0812649 | up | mRNA |
| NM_000167 | GK | 0.680818201 | 0.960111041 | 1.0437894 | up | mRNA |
| NM_000168 | GLI3 | 0.620990769 | 0.947926299 | 1.108256 | up | mRNA |
| NM_000169 | GLA | 0.600255167 | 0.940648177 | 1.11625 | up | mRNA |
| NM_000170 | GLDC | 0.59117687 | 0.937021663 | 1.0997497 | up | mRNA |
| NM_000181 | GUSB | 0.476505665 | 0.900415348 | 1.0841517 | up | mRNA |
| NM_000188 | HK1 | 0.724772159 | 0.966191059 | 1.0383158 | up | mRNA |
| NM_000196 | HSD11B2 | 0.847392697 | 0.987678166 | 1.0122398 | up | mRNA |
| NM_000202 | IDS | 0.995911437 | 0.999872237 | 1.001152 | up | mRNA |
| NM_000203 | IDUA | 0.81223242 | 0.98118927 | 1.0495336 | up | mRNA |
| NM_000209 | PDX1 | 0.90376464 | 0.99508468 | 1.0226733 | up | mRNA |
| NM_000214 | JAG1 | 0.485656191 | 0.903250293 | 1.1682492 | up | mRNA |
| NM_000215 | JAK3 | 0.951229983 | 0.996710991 | 1.009028 | up | mRNA |
| NM_000217 | KCNA1 | 5.16177E-05 | 0.029985488 | 4.5972326 | up | mRNA |
| NM_000218 | KCNQ1 | 0.077936434 | 0.479601567 | 1.0691635 | up | mRNA |
| NM_000219 | KCNE1 | 0.740299259 | 0.969794316 | 1.2282438 | up | mRNA |
| NM_000220 | KCNJ1 | 0.654928591 | 0.957205183 | 1.0438537 | up | mRNA |
| NM_000223 | KRT12 | 0.287221401 | 0.783022998 | 1.1663849 | up | mRNA |
| NM_000235 | LIPA | 0.128723803 | 0.58263855 | 1.1699308 | up | mRNA |
| NM_000236 | LIPC | 0.185106759 | 0.668407207 | 1.425094 | up | mRNA |
| NM_000252 | MTM1 | 0.775362114 | 0.97589756 | 1.0204901 | up | mRNA |
| NM_000258 | MYL3 | 0.29222275 | 0.788954556 | 1.1695392 | up | mRNA |
| NM_000260 | MYO7A | 0.621129772 | 0.947926299 | 1.0306369 | up | mRNA |
| NM_000263 | NAGLU | 0.983674796 | 0.998971807 | 1.0022096 | up | mRNA |
| NM_000266 | NDP | 0.009655263 | 0.205650016 | 2.047779 | up | mRNA |
| NM_000269 | NME1 | 0.91518459 | 0.995349793 | 1.0200841 | up | mRNA |
| NM_000271 | NPC1 | 0.648687664 | 0.95550188 | 1.0383889 | up | mRNA |
| NM_000273 | GPR143 | 0.723589252 | 0.966191059 | 1.0385311 | up | mRNA |
| NM_000278 | PAX2 | 0.823474342 | 0.983498765 | 1.0267047 | up | mRNA |
| NM_000283 | PDE6B | 0.161662227 | 0.633561373 | 1.4074526 | up | mRNA |
| NM_000288 | PEX7 | 0.815448 | 0.981459009 | 1.0512827 | up | mRNA |
| NM_000301 | PLG | 0.062155956 | 0.439379649 | 1.1567115 | up | mRNA |
| NM_000303 | PMM2 | 0.514631364 | 0.913840427 | 1.1111562 | up | mRNA |
| NM_000306 | POU1F1 | 0.058358295 | 0.429582432 | 1.427359 | up | mRNA |
| NM_000320 | QDPR | 0.841400444 | 0.987068842 | 1.0087589 | up | mRNA |
| NM_000322 | PRPH2 | 0.229419345 | 0.726452908 | 1.166438 | up | mRNA |
| NM_000324 | RHAG | 0.473820657 | 0.899418411 | 1.144678 | up | mRNA |
| NM_000325 | PITX2 | 0.949365718 | 0.996710991 | 1.0123563 | up | mRNA |
| NM_000326 | RLBP1 | 0.493403192 | 0.90641426 | 1.0382447 | up | mRNA |
| NM_000328 | RPGR | 0.906174025 | 0.99508468 | 1.0297387 | up | mRNA |
| NM_000337 | SGCD | 0.866614191 | 0.989481868 | 1.0888732 | up | mRNA |
| NM_000343 | SLC5A1 | 0.576906426 | 0.933656777 | 1.1562 | up | mRNA |
| NM_000344 | SMN1 | 0.935912027 | 0.996710991 | 1.0077933 | up | mRNA |
| NM_000347 | SPTB | 0.645504504 | 0.955130468 | 1.0537936 | up | mRNA |
| NM_000349 | STAR | 0.421854297 | 0.873398499 | 1.2788567 | up | mRNA |
| NM_000352 | ABCC8 | 0.823198205 | 0.983498765 | 1.0491394 | up | mRNA |
| NM_000367 | TPMT | 0.908455617 | 0.995329331 | 1.0122548 | up | mRNA |
| NM_000371 | TTR | 0.457547621 | 0.892189338 | 1.1011233 | up | mRNA |
| NM_000374 | UROD | 0.87543194 | 0.991094934 | 1.0113648 | up | mRNA |
| NM_000375 | UROS | 0.942477223 | 0.996710991 | 1.0077839 | up | mRNA |
| NM_000388 | CASR | 0.716379562 | 0.966191059 | 1.042903 | up | mRNA |
| NM_000389 | CDKN1A | 0.5232647 | 0.917239107 | 1.0654087 | up | mRNA |
| NM_000393 | COL5A2 | 0.005569415 | 0.169833068 | 1.2573483 | up | mRNA |
| NM_000395 | CSF2RB | 0.54931591 | 0.925481755 | 1.0579194 | up | mRNA |
| NM_000396 | CTSK | 0.906574896 | 0.99508468 | 1.0367682 | up | mRNA |
| NM_000398 | CYB5R3 | 0.276860924 | 0.777075691 | 1.1076671 | up | mRNA |
| NM_000399 | EGR2 | 0.151557428 | 0.616711191 | 1.4839406 | up | mRNA |
| NM_000402 | G6PD | 0.698718627 | 0.962701213 | 1.1204904 | up | mRNA |
| NM_000405 | GM2A | 0.541076816 | 0.923577434 | 1.0164483 | up | mRNA |
| NM_000406 | GNRHR | 0.819206287 | 0.98219491 | 1.0371882 | up | mRNA |
| NM_000407 | GP1BB | 0.633605876 | 0.951174475 | 1.029366 | up | mRNA |
| NM_000414 | HSD17B4 | 0.500249541 | 0.909680616 | 1.0465513 | up | mRNA |
| NM_000417 | IL2RA | 0.728336134 | 0.966191059 | 1.0841164 | up | mRNA |
| NM_000418 | IL4R | 0.322861669 | 0.815766149 | 1.2601045 | up | mRNA |
| NM_000423 | KRT2 | 0.532156392 | 0.920228311 | 1.1982945 | up | mRNA |
| NM_000426 | LAMA2 | 0.991791461 | 0.999548897 | 1.0044587 | up | mRNA |
| NM_000428 | LTBP2 | 0.87560313 | 0.991094934 | 1.0117439 | up | mRNA |
| NM_000431 | MVK | 0.046364403 | 0.392259245 | 1.2700587 | up | mRNA |
| NM_000435 | NOTCH3 | 0.968801705 | 0.998196842 | 1.004507 | up | mRNA |
| NM_000436 | OXCT1 | 0.834166334 | 0.986475536 | 1.0272853 | up | mRNA |
| NM_000441 | SLC26A4 | 0.542015165 | 0.923577434 | 1.153742 | up | mRNA |
| NM_000444 | PHEX | 0.774681789 | 0.97589756 | 1.032035 | up | mRNA |
| NM_000446 | PON1 | 0.442938051 | 0.884304813 | 1.2350046 | up | mRNA |
| NM_000451 | SHOX | 0.492475696 | 0.906047853 | 1.7472247 | up | mRNA |
| NM_000454 | SOD1 | 0.203348516 | 0.692876095 | 1.1119953 | up | mRNA |
| NM_000460 | THPO | 0.163078999 | 0.635317534 | 1.1005457 | up | mRNA |
| NM_000466 | PEX1 | 0.719992355 | 0.966191059 | 1.0158357 | up | mRNA |
| NM_000478 | ALPL | 0.925446925 | 0.995465766 | 1.0280899 | up | mRNA |
| NM_000495 | COL4A5 | 0.490921213 | 0.90553335 | 1.0511498 | up | mRNA |
| NM_000496 | CRYBB2 | 0.048971221 | 0.401897225 | 1.1873749 | up | mRNA |
| NM_000500 | CYP21A2 | 0.008609635 | 0.199898185 | 1.3605417 | up | mRNA |
| NM_000507 | FBP1 | 0.562710361 | 0.929041598 | 1.0759283 | up | mRNA |
| NM_000511 | FUT2 | 0.80759064 | 0.98118927 | 1.023155 | up | mRNA |
| NM_000515 | GH1 | 0.932561547 | 0.996710991 | 1.0077594 | up | mRNA |
| NM_000519 | HBD | 0.369664036 | 0.846215286 | 1.0549376 | up | mRNA |
| NM_000523 | HOXD13 | 0.858632989 | 0.988512246 | 1.0204504 | up | mRNA |
| NM_000528 | MAN2B1 | 0.021276036 | 0.288424269 | 1.2196209 | up | mRNA |
| NM_000531 | OTC | 0.793845106 | 0.97921643 | 1.0143301 | up | mRNA |
| NM_000551 | VHL | 0.340640692 | 0.828777111 | 1.0446308 | up | mRNA |
| NM_000552 | VWF | 0.132275198 | 0.587843174 | 1.1894246 | up | mRNA |
| NM_000558 | HBA1 | 0.741124569 | 0.970205535 | 1.0386713 | up | mRNA |
| NM_000561 | GSTM1 | 0.911930423 | 0.995342392 | 1.0804207 | up | mRNA |
| NM_000573 | CR1 | 0.952068301 | 0.996710991 | 1.0112603 | up | mRNA |
| NM_000574 | CD55 | 0.346345629 | 0.833163669 | 1.0818393 | up | mRNA |
| NM_000575 | IL1A | 0.000497391 | 0.067872214 | 13.4552538 | up | mRNA |
| NM_000576 | IL1B | 0.001004714 | 0.085705167 | 36.4643692 | up | mRNA |
| NM_000581 | GPX1 | 0.835321214 | 0.986575022 | 1.0241874 | up | mRNA |
| NM_000585 | IL15 | 0.214636108 | 0.707749163 | 2.2929842 | up | mRNA |
| NM_000588 | IL3 | 0.936053832 | 0.996710991 | 1.0147011 | up | mRNA |
| NM_000593 | TAP1 | 0.020916647 | 0.287349508 | 1.4787515 | up | mRNA |
| NM_000594 | TNF | 0.103982453 | 0.533896783 | 1.6346765 | up | mRNA |
| NM_000598 | IGFBP3 | 0.59568628 | 0.938746918 | 1.1212226 | up | mRNA |
| NM_000599 | IGFBP5 | 0.001975529 | 0.107570744 | 1.2474735 | up | mRNA |
| NM_000600 | IL6 | 3.22298E-05 | 0.023612787 | 2.2309858 | up | mRNA |
| NM_000601 | HGF | 0.84847993 | 0.987678166 | 1.1824108 | up | mRNA |
| NM_000603 | NOS3 | 0.153065336 | 0.619205018 | 1.1088377 | up | mRNA |
| NM_000606 | C8G | 0.813445663 | 0.98120545 | 1.0529915 | up | mRNA |
| NM_000608 | ORM2 | 0.781437152 | 0.976938981 | 1.0236508 | up | mRNA |
| NM_000610 | CD44 | 0.000660146 | 0.076919551 | 5.6708867 | up | mRNA |
| NM_000614 | CNTF | 0.373160289 | 0.848112577 | 1.3715483 | up | mRNA |
| NM_000615 | NCAM1 | 0.717460702 | 0.966191059 | 1.0432752 | up | mRNA |
| NM_000616 | CD4 | 0.257402752 | 0.757735833 | 1.2305257 | up | mRNA |
| NM_000621 | HTR2A | 0.888829613 | 0.993440915 | 1.0595798 | up | mRNA |
| NM_000625 | NOS2 | 0.422463145 | 0.873896892 | 1.0723237 | up | mRNA |
| NM_000628 | IL10RB | 0.000463914 | 0.066148041 | 1.6036565 | up | mRNA |
| NM_000629 | IFNAR1 | 0.079412956 | 0.482639336 | 1.1346386 | up | mRNA |
| NM_000631 | NCF4 | 0.710049118 | 0.965462762 | 1.0605342 | up | mRNA |
| NM_000632 | ITGAM | 0.520847216 | 0.916605148 | 1.0952549 | up | mRNA |
| NM_000636 | SOD2 | 0.000130853 | 0.042911465 | 7.866537 | up | mRNA |
| NM_000640 | IL13RA2 | 0.895324394 | 0.993978977 | 1.0406317 | up | mRNA |
| NM_000644 | AGL | 0.167601813 | 0.642346587 | 1.1948826 | up | mRNA |
| NM_000668 | ADH1B | 0.075813316 | 0.473123493 | 1.1204249 | up | mRNA |
| NM_000669 | ADH1C | 0.375579893 | 0.849862269 | 1.3934546 | up | mRNA |
| NM_000676 | ADORA2B | 0.852488369 | 0.987678166 | 1.0458352 | up | mRNA |
| NM_000678 | ADRA1D | 0.897826263 | 0.994326043 | 1.0172399 | up | mRNA |
| NM_000686 | AGTR2 | 0.016063284 | 0.25477722 | 1.0612247 | up | mRNA |
| NM_000687 | AHCY | 0.921908838 | 0.995349793 | 1.0149563 | up | mRNA |
| NM_000689 | ALDH1A1 | 0.676123452 | 0.959531882 | 1.0499696 | up | mRNA |
| NM_000690 | ALDH2 | 0.085470143 | 0.497360491 | 1.087105 | up | mRNA |
| NM_000692 | ALDH1B1 | 0.289025921 | 0.784785662 | 1.1241079 | up | mRNA |
| NM_000693 | ALDH1A3 | 0.517174596 | 0.915240133 | 1.1302978 | up | mRNA |
| NM_000695 | ALDH3B2 | 0.454150561 | 0.890261204 | 1.1341668 | up | mRNA |
| NM_000699 | AMY2A | 0.757668033 | 0.972072638 | 1.0295228 | up | mRNA |
| NM_000701 | ATP1A1 | 0.371468007 | 0.846675738 | 1.1676913 | up | mRNA |
| NM_000707 | AVPR1B | 0.714222392 | 0.966110889 | 1.1203072 | up | mRNA |
| NM_000710 | BDKRB1 | 0.000401786 | 0.061887191 | 5.2259899 | up | mRNA |
| NM_000712 | BLVRA | 0.361996714 | 0.842986736 | 1.0936656 | up | mRNA |
| NM_000713 | BLVRB | 0.938105471 | 0.996710991 | 1.006399 | up | mRNA |
| NM_000719 | CACNA1C | 0.963018574 | 0.99793927 | 1.0053101 | up | mRNA |
| NM_000725 | CACNB3 | 0.382128437 | 0.853514109 | 1.2743757 | up | mRNA |
| NM_000726 | CACNB4 | 0.864566493 | 0.989401142 | 1.030895 | up | mRNA |
| NM_000727 | CACNG1 | 0.441327321 | 0.883920737 | 1.0780974 | up | mRNA |
| NM_000728 | CALCB | 0.722639206 | 0.966191059 | 1.0261807 | up | mRNA |
| NM_000730 | CCKAR | 0.965999716 | 0.99793927 | 1.0297353 | up | mRNA |
| NM_000733 | CD3E | 0.245058119 | 0.743548975 | 1.2797381 | up | mRNA |
| NM_000735 | CGA | 0.470776957 | 0.898445976 | 1.1900557 | up | mRNA |
| NM_000742 | CHRNA2 | 0.163979654 | 0.636387539 | 1.3532657 | up | mRNA |
| NM_000750 | CHRNB4 | 0.990095592 | 0.999140676 | 1.0017869 | up | mRNA |
| NM_000751 | CHRND | 0.113359541 | 0.5567213 | 1.2517487 | up | mRNA |
| NM_000758 | CSF2 | 9.24468E-05 | 0.036683999 | 608.7565866 | up | mRNA |
| NM_000762 | CYP2A6 | 0.971273157 | 0.998501532 | 1.0024778 | up | mRNA |
| NM_000785 | CYP27B1 | 0.243676296 | 0.742130994 | 1.6089366 | up | mRNA |
| NM_000787 | DBH | 0.0905905 | 0.508246703 | 1.2193197 | up | mRNA |
| NM_000789 | ACE | 0.402201803 | 0.864786348 | 1.2016156 | up | mRNA |
| NM_000793 | DIO2 | 0.44978155 | 0.888279993 | 1.1268853 | up | mRNA |
| NM_000797 | DRD4 | 0.45029705 | 0.888519952 | 1.0934038 | up | mRNA |
| NM_000802 | FOLR1 | 0.150635828 | 0.615623649 | 1.4244417 | up | mRNA |
| NM_000808 | GABRA3 | 0.353573997 | 0.836405054 | 1.0917736 | up | mRNA |
| NM_000811 | GABRA6 | 0.748811125 | 0.970725371 | 1.0475521 | up | mRNA |
| NM_000812 | GABRB1 | 0.718244058 | 0.966191059 | 1.1196511 | up | mRNA |
| NM_000814 | GABRB3 | 0.918675919 | 0.995349793 | 1.0080527 | up | mRNA |
| NM_000815 | GABRD | 0.64468527 | 0.954657712 | 1.084415 | up | mRNA |
| NM_000816 | GABRG2 | 0.46012226 | 0.893268958 | 1.1575726 | up | mRNA |
| NM_000821 | GGCX | 0.720814975 | 0.966191059 | 1.054199 | up | mRNA |
| NM_000839 | GRM2 | 0.354260063 | 0.837147328 | 1.0566945 | up | mRNA |
| NM_000840 | GRM3 | 0.265249741 | 0.766494847 | 1.1125498 | up | mRNA |
| NM_000844 | GRM7 | 0.078513702 | 0.480536 | 1.3943465 | up | mRNA |
| NM_000848 | GSTM2 | 0.886026471 | 0.992981836 | 1.0456327 | up | mRNA |
| NM_000851 | GSTM5 | 0.111378989 | 0.552465871 | 1.2099861 | up | mRNA |
| NM_000853 | GSTT1 | 0.755483729 | 0.971497464 | 1.1230051 | up | mRNA |
| NM_000857 | GUCY1B3 | 0.046836012 | 0.394183256 | 1.1642383 | up | mRNA |
| NM_000873 | ICAM2 | 0.218533397 | 0.712342894 | 1.0876843 | up | mRNA |
| NM_000874 | IFNAR2 | 4.48281E-05 | 0.028482665 | 4.8956619 | up | mRNA |
| NM_000878 | IL2RB | 0.45751218 | 0.892189338 | 1.0347486 | up | mRNA |
| NM_000882 | IL12A | 0.573894059 | 0.933656777 | 1.1426873 | up | mRNA |
| NM_000888 | ITGB6 | 0.286528411 | 0.782942227 | 1.2404132 | up | mRNA |
| NM_000892 | KLKB1 | 0.175101567 | 0.653821873 | 1.3946535 | up | mRNA |
| NM_000893 | KNG1 | 0.588463799 | 0.93579682 | 1.1724469 | up | mRNA |
| NM_000898 | MAOB | 0.137097112 | 0.595992833 | 1.3168744 | up | mRNA |
| NM_000900 | MGP | 0.231639073 | 0.729296323 | 1.3691296 | up | mRNA |
| NM_000905 | NPY | 0.6545236 | 0.957205183 | 1.0702299 | up | mRNA |
| NM_000918 | P4HB | 0.343284751 | 0.831521302 | 1.0450865 | up | mRNA |
| NM_000924 | PDE1B | 0.957812102 | 0.996933183 | 1.0058731 | up | mRNA |
| NM_000929 | PLA2G5 | 0.029444475 | 0.333953485 | 1.2434496 | up | mRNA |
| NM_000930 | PLAT | 0.306473488 | 0.801653799 | 1.3732578 | up | mRNA |
| NM_000936 | PNLIP | 0.522637358 | 0.917200998 | 1.2041359 | up | mRNA |
| NM_000941 | POR | 0.59931908 | 0.940648177 | 1.1033137 | up | mRNA |
| NM_000942 | PPIB | 0.867045845 | 0.989481868 | 1.007255 | up | mRNA |
| NM_000943 | PPIC | 0.9866127 | 0.99909491 | 1.002993 | up | mRNA |
| NM_000954 | PTGDS | 0.285063257 | 0.781963593 | 1.247179 | up | mRNA |
| NM_000956 | PTGER2 | 0.008815653 | 0.202329498 | 2.3455344 | up | mRNA |
| NM_000958 | PTGER4 | 0.030481213 | 0.338388833 | 1.4180496 | up | mRNA |
| NM_000959 | PTGFR | 0.042662647 | 0.382084661 | 1.6353101 | up | mRNA |
| NM_000960 | PTGIR | 0.204760283 | 0.694790735 | 1.1161894 | up | mRNA |
| NM_000961 | PTGIS | 0.56507848 | 0.929921137 | 1.1598325 | up | mRNA |
| NM_000963 | PTGS2 | 0.014915711 | 0.245074119 | 3.1939376 | up | mRNA |
| NM_000966 | RARG | 0.416513264 | 0.870736736 | 1.070689 | up | mRNA |
| NM_000967 | RPL3 | 0.369581687 | 0.84620888 | 1.1189306 | up | mRNA |
| NM_000968 | RPL4 | 0.829880136 | 0.985549562 | 1.0097914 | up | mRNA |
| NM_000978 | RPL23 | 0.039584144 | 0.370545499 | 1.1661357 | up | mRNA |
| NM_000979 | RPL18 | 0.867866539 | 0.989481868 | 1.0198312 | up | mRNA |
| NM_000980 | RPL18A | 0.4854834 | 0.903250293 | 1.0587224 | up | mRNA |
| NM_000982 | RPL21 | 0.012507427 | 0.227664282 | 1.1192019 | up | mRNA |
| NM_000983 | RPL22 | 0.38783276 | 0.857110401 | 1.0908769 | up | mRNA |
| NM_000984 | RPL23A | 0.569085364 | 0.93232103 | 1.0432021 | up | mRNA |
| NM_000986 | RPL24 | 0.679871879 | 0.960007989 | 1.0116164 | up | mRNA |
| NM_000987 | RPL26 | 0.170735517 | 0.647381721 | 1.180623 | up | mRNA |
| NM_000990 | RPL27A | 0.150508936 | 0.615491439 | 1.1911756 | up | mRNA |
| NM_000993 | RPL31 | 0.066527279 | 0.450298982 | 1.0469794 | up | mRNA |
| NM_000996 | RPL35A | 0.320328073 | 0.814552264 | 1.0252291 | up | mRNA |
| NM_000997 | RPL37 | 0.00917844 | 0.203383174 | 1.0614365 | up | mRNA |
| NM_000998 | RPL37A | 0.628944623 | 0.950128593 | 1.0226156 | up | mRNA |
| NM_001001389 | CD44 | 0.000245516 | 0.052545605 | 5.0409503 | up | mRNA |
| NM_001001390 | CD44 | 6.84607E-05 | 0.031937303 | 4.7026494 | up | mRNA |
| NM_001001392 | CD44 | 0.000773362 | 0.081471496 | 1.5025288 | up | mRNA |
| NM_001001396 | ATP2B4 | 0.006379809 | 0.180561804 | 1.5239895 | up | mRNA |
| NM_001001417 | TBC1D3B | 0.947180768 | 0.996710991 | 1.0038914 | up | mRNA |
| NM_001001418 | TBC1D3C | 0.435153478 | 0.881404713 | 1.0775046 | up | mRNA |
| NM_001001433 | STX16 | 0.251408005 | 0.751378444 | 1.0541987 | up | mRNA |
| NM_001001435 | CCL4L1 | 0.075707966 | 0.472611103 | 7.3021655 | up | mRNA |
| NM_001001437 | CCL3L3 | 0.059451274 | 0.432586127 | 1.1959512 | up | mRNA |
| NM_001001520 | HDGFRP2 | 0.038576144 | 0.366748746 | 1.6022049 | up | mRNA |
| NM_001001521 | UGP2 | 0.161489801 | 0.633496168 | 1.1965528 | up | mRNA |
| NM_001001523 | RORC | 0.397105971 | 0.862344286 | 1.2377847 | up | mRNA |
| NM_001001548 | CD36 | 0.950304647 | 0.996710991 | 1.1048933 | up | mRNA |
| NM_001001549 | GRB10 | 0.41490488 | 0.870239791 | 1.1623948 | up | mRNA |
| NM_001001560 | GGA1 | 0.49687921 | 0.908665955 | 1.1328175 | up | mRNA |
| NM_001001577 | PDE9A | 0.565367704 | 0.930020725 | 1.1505058 | up | mRNA |
| NM_001001582 | PDE9A | 0.041138721 | 0.375924711 | 1.1162666 | up | mRNA |
| NM_001001659 | OR2A14 | 0.416739954 | 0.870736736 | 1.2540152 | up | mRNA |
| NM_001001666 | ANO7 | 0.537465763 | 0.922702335 | 1.0629372 | up | mRNA |
| NM_001001671 | MAP3K15 | 0.153571852 | 0.620667112 | 1.1442126 | up | mRNA |
| NM_001001676 | LCN9 | 0.243874478 | 0.742391426 | 1.2133553 | up | mRNA |
| NM_001001683 | MED11 | 0.845880757 | 0.987338076 | 1.0276841 | up | mRNA |
| NM_001001694 | IL17REL | 0.559564785 | 0.927226667 | 1.058203 | up | mRNA |
| NM_001001701 | C4orf3 | 0.877079754 | 0.991255275 | 1.0234213 | up | mRNA |
| NM_001001709 | C9orf170 | 0.761799051 | 0.97339948 | 1.0132438 | up | mRNA |
| NM_001001713 | SH3BGR | 0.797605684 | 0.980721189 | 1.0205948 | up | mRNA |
| NM_001001732 | CC2D2B | 0.054769864 | 0.419413793 | 1.4364419 | up | mRNA |
| NM_001001850 | STX19 | 0.559323893 | 0.927203296 | 1.0888124 | up | mRNA |
| NM_001001852 | PIM3 | 0.048657877 | 0.400568526 | 4.1813487 | up | mRNA |
| NM_001001872 | C14orf37 | 0.789004064 | 0.977807732 | 1.0190525 | up | mRNA |
| NM_001001936 | AFAP1L2 | 0.372041825 | 0.847209316 | 1.1818862 | up | mRNA |
| NM_001001971 | FAM13C | 0.532426824 | 0.920378445 | 1.0907514 | up | mRNA |
| NM_001001992 | USP16 | 0.53331404 | 0.920530493 | 1.0443647 | up | mRNA |
| NM_001002017 | HCFC1R1 | 0.98318809 | 0.998971807 | 1.0035495 | up | mRNA |
| NM_001002020 | PUS1 | 0.931252614 | 0.996710991 | 1.0158739 | up | mRNA |
| NM_001002026 | CLDN18 | 0.250833223 | 0.750432768 | 1.3477337 | up | mRNA |
| NM_001002029 | C4B | 0.483864457 | 0.903204457 | 1.2176397 | up | mRNA |
| NM_001002235 | SERPINA1 | 0.092889011 | 0.511682303 | 1.6315495 | up | mRNA |
| NM_001002245 | ANAPC11 | 0.772443067 | 0.975448017 | 1.013576 | up | mRNA |
| NM_001002255 | SUMO4 | 8.26393E-05 | 0.034290244 | 1.9796552 | up | mRNA |
| NM_001002264 | EPSTI1 | 0.899270796 | 0.994575817 | 1.028132 | up | mRNA |
| NM_001002295 | GATA3 | 0.277985259 | 0.777290204 | 1.3002619 | up | mRNA |
| NM_001002755 | NFU1 | 0.322128007 | 0.815372756 | 1.0477799 | up | mRNA |
| NM_001002759 | SFR1 | 0.253431853 | 0.753550225 | 1.4069262 | up | mRNA |
| NM_001002760 | BPY2B | 0.726042181 | 0.966191059 | 1.0378479 | up | mRNA |
| NM_001002836 | ZNF787 | 0.365888643 | 0.845164582 | 1.2015405 | up | mRNA |
| NM_001002862 | DERL3 | 0.888356056 | 0.993440915 | 1.007776 | up | mRNA |
| NM_001002911 | GPR139 | 0.594195193 | 0.938369994 | 1.084006 | up | mRNA |
| NM_001002913 | PTRH1 | 0.680379711 | 0.960111041 | 1.0671695 | up | mRNA |
| NM_001002914 | KCTD11 | 0.403254298 | 0.864786348 | 1.2144228 | up | mRNA |
| NM_001002919 | FAM150B | 0.743114188 | 0.970330526 | 1.0426727 | up | mRNA |
| NM_001002926 | TWISTNB | 0.450472395 | 0.888533638 | 1.0627524 | up | mRNA |
| NM_001003 | RPLP1 | 0.241537945 | 0.739795638 | 1.0220674 | up | mRNA |
| NM_001003395 | TPD52L1 | 0.937227642 | 0.996710991 | 1.0658776 | up | mRNA |
| NM_001003396 | TPD52L1 | 0.954719427 | 0.996834354 | 1.0474537 | up | mRNA |
| NM_001003665 | C1orf95 | 0.326528948 | 0.819425644 | 1.2081761 | up | mRNA |
| NM_001003678 | C11orf49 | 0.106794054 | 0.541480474 | 1.205029 | up | mRNA |
| NM_001003682 | TMEM200B | 0.230208128 | 0.727477722 | 1.4168605 | up | mRNA |
| NM_001003684 | UQCR10 | 0.810141905 | 0.98118927 | 1.0196738 | up | mRNA |
| NM_001003693 | LY6G6F | 0.031681653 | 0.341807319 | 1.1302929 | up | mRNA |
| NM_001003694 | BRPF1 | 0.338804328 | 0.827403747 | 1.1060385 | up | mRNA |
| NM_001003696 | ATP5J | 0.648055246 | 0.95550188 | 1.0290994 | up | mRNA |
| NM_001003701 | ATP5J | 0.542069899 | 0.923577434 | 1.0794816 | up | mRNA |
| NM_001003704 | MTFP1 | 0.030573494 | 0.338388833 | 1.0879302 | up | mRNA |
| NM_001003712 | OSBPL8 | 0.773306147 | 0.97589756 | 1.0662679 | up | mRNA |
| NM_001003714 | ATP5J2 | 0.293348829 | 0.790610868 | 1.0820431 | up | mRNA |
| NM_001003785 | ATP5H | 0.416290089 | 0.870736736 | 1.0784115 | up | mRNA |
| NM_001003788 | STRADA | 0.647810316 | 0.95550188 | 1.0513854 | up | mRNA |
| NM_001003793 | RBMS3 | 0.348911503 | 0.834138149 | 1.0943028 | up | mRNA |
| NM_001003794 | MGLL | 0.111492214 | 0.552757787 | 1.1261391 | up | mRNA |
| NM_001003796 | NHP2L1 | 0.573112944 | 0.933656777 | 1.0150847 | up | mRNA |
| NM_001003811 | TEX11 | 0.82179331 | 0.982940803 | 1.0645656 | up | mRNA |
| NM_001003841 | SLC6A19 | 0.360636474 | 0.841780454 | 1.1386547 | up | mRNA |
| NM_001003891 | MED15 | 0.392109023 | 0.859461046 | 1.0730823 | up | mRNA |
| NM_001003895 | CDY1B | 0.966159623 | 0.99793927 | 1.003187 | up | mRNA |
| NM_001003897 | MANBAL | 0.773291995 | 0.97589756 | 1.0136717 | up | mRNA |
| NM_001004019 | FBLN2 | 0.469341989 | 0.897810636 | 1.1326766 | up | mRNA |
| NM_001004023 | DYRK3 | 0.072784619 | 0.463915802 | 1.2775887 | up | mRNA |
| NM_001004052 | OR52B2 | 0.321011912 | 0.814934973 | 1.2922628 | up | mRNA |
| NM_001004057 | GRK4 | 0.34032696 | 0.828487518 | 1.0799222 | up | mRNA |
| NM_001004067 | NOMO3 | 0.597352775 | 0.939753684 | 1.0408388 | up | mRNA |
| NM_001004125 | TUSC1 | 0.113618546 | 0.557454702 | 1.3543591 | up | mRNA |
| NM_001004313 | TMEM220 | 0.20660555 | 0.698066071 | 1.2191741 | up | mRNA |
| NM_001004318 | PAPL | 0.146527267 | 0.608122555 | 1.2145365 | up | mRNA |
| NM_001004339 | ZYG11A | 0.960917705 | 0.99792516 | 1.0140095 | up | mRNA |
| NM_001004342 | TRIM67 | 0.54681124 | 0.924803608 | 1.0720686 | up | mRNA |
| NM_001004343 | MAP1LC3C | 0.00310885 | 0.131960624 | 1.2245883 | up | mRNA |
| NM_001004353 | C9orf173 | 0.173905471 | 0.652129479 | 1.0682167 | up | mRNA |
| NM_001004419 | CLEC2D | 0.004154956 | 0.147175197 | 6.8263384 | up | mRNA |
| NM_001004432 | LINGO4 | 0.27232217 | 0.772141487 | 1.1221891 | up | mRNA |
| NM_001004440 | FAM19A3 | 0.947311371 | 0.996710991 | 1.0021149 | up | mRNA |
| NM_001004460 | OR10A2 | 0.050509716 | 0.406628421 | 1.2560444 | up | mRNA |
| NM_001004461 | OR10A6 | 0.318503774 | 0.812422372 | 1.3002741 | up | mRNA |
| NM_001004470 | ST8SIA6 | 0.060871743 | 0.436633496 | 1.5031191 | up | mRNA |
| NM_001004486 | OR13H1 | 0.198687456 | 0.687087939 | 1.1830816 | up | mRNA |
| NM_001004490 | OR2AG2 | 0.510313803 | 0.912097614 | 1.0767868 | up | mRNA |
| NM_001004492 | OR2B11 | 0.368840638 | 0.845559574 | 1.10242 | up | mRNA |
| NM_001004691 | OR2M7 | 0.68464694 | 0.960111041 | 1.0546549 | up | mRNA |
| NM_001004746 | OR5T2 | 0.91564697 | 0.995349793 | 1.019068 | up | mRNA |
| NM_001005169 | OR52I1 | 0.655943853 | 0.957336187 | 1.1804441 | up | mRNA |
| NM_001005178 | OR52W1 | 0.122326263 | 0.57455499 | 1.6548716 | up | mRNA |
| NM_001005180 | OR56B1 | 0.405267797 | 0.866165103 | 1.3764908 | up | mRNA |
| NM_001005181 | OR56B4 | 0.666891641 | 0.958354006 | 1.0746157 | up | mRNA |
| NM_001005195 | OR8B12 | 0.569636168 | 0.932573151 | 1.0863724 | up | mRNA |
| NM_001005198 | OR8G5 | 0.022121868 | 0.293783036 | 1.1841402 | up | mRNA |
| NM_001005199 | OR8H1 | 0.469249977 | 0.897810636 | 1.1551769 | up | mRNA |
| NM_001005218 | OR5B21 | 0.666273048 | 0.958354006 | 1.1213887 | up | mRNA |
| NM_001005242 | PKP2 | 0.875809826 | 0.991148023 | 1.0120645 | up | mRNA |
| NM_001005271 | CHD3 | 0.616120372 | 0.945912508 | 1.0877196 | up | mRNA |
| NM_001005273 | CHD3 | 0.196746877 | 0.685161614 | 1.145634 | up | mRNA |
| NM_001005274 | OR4A16 | 0.093294111 | 0.512441525 | 1.3001933 | up | mRNA |
| NM_001005279 | OR6K2 | 0.841426184 | 0.987068842 | 1.0336679 | up | mRNA |
| NM_001005286 | OR6F1 | 0.141515762 | 0.601858131 | 1.1030594 | up | mRNA |
| NM_001005291 | SREBF1 | 0.644531159 | 0.954657712 | 1.1160975 | up | mRNA |
| NM_001005328 | OR2A7 | 0.848882555 | 0.987678166 | 1.075274 | up | mRNA |
| NM_001005333 | MAGED1 | 0.344523012 | 0.831973758 | 1.0711447 | up | mRNA |
| NM_001005335 | HNRNPL | 0.283234286 | 0.780770837 | 1.102434 | up | mRNA |
| NM_001005367 | TTYH1 | 0.647945207 | 0.95550188 | 1.0612897 | up | mRNA |
| NM_001005374 | LRSAM1 | 0.277256103 | 0.777205298 | 1.296098 | up | mRNA |
| NM_001005376 | PLAUR | 0.503174306 | 0.910475258 | 1.1148699 | up | mRNA |
| NM_001005377 | PLAUR | 0.914939742 | 0.995349793 | 1.0099653 | up | mRNA |
| NM_001005386 | ACTR2 | 0.671954924 | 0.959531882 | 1.0327277 | up | mRNA |
| NM_001005388 | NFASC | 0.329428764 | 0.821768501 | 1.1514685 | up | mRNA |
| NM_001005404 | YPEL2 | 0.039182975 | 0.369512172 | 3.437624 | up | mRNA |
| NM_001005413 | ZWINT | 0.99824985 | 0.999889342 | 1.0011524 | up | mRNA |
| NM_001005415 | MARCH2 | 0.408938123 | 0.867591329 | 1.1927986 | up | mRNA |
| NM_001005416 | MARCH2 | 0.971484454 | 0.998501532 | 1.0106498 | up | mRNA |
| NM_001005463 | EBF3 | 0.643110711 | 0.954522001 | 1.0582919 | up | mRNA |
| NM_001005464 | HIST2H3A | 0.867673819 | 0.989481868 | 1.1475852 | up | mRNA |
| NM_001005467 | OR8B3 | 0.558925475 | 0.927153474 | 1.3316596 | up | mRNA |
| NM_001005469 | OR5B3 | 0.348338944 | 0.834138149 | 1.1066592 | up | mRNA |
| NM_001005474 | NFKBIZ | 0.000712688 | 0.078885568 | 4.1542486 | up | mRNA |
| NM_001005490 | OR6C74 | 0.532880433 | 0.920530493 | 1.0560798 | up | mRNA |
| NM_001005498 | RHBDF2 | 0.695915807 | 0.962410025 | 1.1213858 | up | mRNA |
| NM_001005526 | SF3B1 | 0.083478147 | 0.492392714 | 1.1096112 | up | mRNA |
| NM_001005567 | OR51B5 | 0.606798548 | 0.942291918 | 1.1217117 | up | mRNA |
| NM_001005612 | EDA | 0.475803344 | 0.900161309 | 1.3314924 | up | mRNA |
| NM_001005738 | FPR2 | 0.526225198 | 0.917975454 | 1.1116143 | up | mRNA |
| NM_001005743 | NUMB | 0.092169074 | 0.511207518 | 1.213939 | up | mRNA |
| NM_001005744 | NUMB | 0.027349879 | 0.321618131 | 1.2845413 | up | mRNA |
| NM_001005746 | CACNB4 | 0.78949956 | 0.978053425 | 1.0447991 | up | mRNA |
| NM_001005751 | FAM21A | 0.018446809 | 0.271518921 | 1.2197182 | up | mRNA |
| NM_001005781 | SUMO1 | 0.848035153 | 0.987678166 | 1.0169011 | up | mRNA |
| NM_001005785 | DAZ2 | 0.856945127 | 0.988512246 | 1.0284674 | up | mRNA |
| NM_001005861 | RYK | 0.480861494 | 0.901538317 | 1.0415499 | up | mRNA |
| NM_001005862 | ERBB2 | 0.720030041 | 0.966191059 | 1.0653191 | up | mRNA |
| NM_001005911 | IP6K2 | 0.901159161 | 0.994807691 | 1.0191999 | up | mRNA |
| NM_001005914 | SEMA3B | 0.301180578 | 0.796100301 | 1.1687188 | up | mRNA |
| NM_001005915 | ERBB3 | 0.079513708 | 0.482639336 | 1.0509837 | up | mRNA |
| NM_001005918 | ATP7B | 0.232415161 | 0.730317988 | 1.2196773 | up | mRNA |
| NM_001005920 | JMJD8 | 0.649426386 | 0.95550188 | 1.0891436 | up | mRNA |
| NM_001006618 | MAPKAP1 | 0.0230626 | 0.298858368 | 1.1793048 | up | mRNA |
| NM_001006624 | PDPN | 0.016380327 | 0.255922225 | 1.476747 | up | mRNA |
| NM_001006625 | PDPN | 0.020255843 | 0.284825234 | 1.5059258 | up | mRNA |
| NM_001006634 | ARHGAP17 | 0.556578645 | 0.926833616 | 1.1017618 | up | mRNA |
| NM_001006641 | SLC25A25 | 0.257165079 | 0.757669958 | 1.1502997 | up | mRNA |
| NM_001006655 | FAM149A | 0.886726424 | 0.993055447 | 1.0667144 | up | mRNA |
| NM_001006665 | RPS6KA1 | 0.010225607 | 0.20936306 | 1.6041099 | up | mRNA |
| NM_001006666 | APOBEC3F | 0.015286134 | 0.248240953 | 1.1418469 | up | mRNA |
| NM_001006932 | RPS6KA2 | 0.850186926 | 0.987678166 | 1.0410673 | up | mRNA |
| NM_001006943 | EPHA8 | 0.363911797 | 0.843353654 | 1.1045671 | up | mRNA |
| NM_001006947 | UHRF1BP1L | 0.396729551 | 0.862081791 | 1.062331 | up | mRNA |
| NM_001007068 | SDCBP | 0.129047481 | 0.582935655 | 1.4668259 | up | mRNA |
| NM_001007070 | SDCBP | 0.223375194 | 0.71761506 | 1.3783026 | up | mRNA |
| NM_001007073 | RPL32 | 0.084764871 | 0.495305754 | 1.0221971 | up | mRNA |
| NM_001007075 | KLHL5 | 0.700652635 | 0.962991468 | 1.1266165 | up | mRNA |
| NM_001007090 | C8orf48 | 0.69878739 | 0.962701213 | 1.1536732 | up | mRNA |
| NM_001007255 | KLHDC9 | 0.15048876 | 0.615491439 | 1.09238 | up | mRNA |
| NM_001007257 | PRDM2 | 0.907511537 | 0.995145546 | 1.0159368 | up | mRNA |
| NM_001007267 | PLA2R1 | 0.994155524 | 0.999872237 | 1.0014294 | up | mRNA |
| NM_001007273 | DUSP13 | 0.972176182 | 0.998501532 | 1.0028386 | up | mRNA |
| NM_001007466 | TULP4 | 0.734461644 | 0.967777568 | 1.0367127 | up | mRNA |
| NM_001007468 | SMARCB1 | 0.845008947 | 0.987338076 | 1.0150338 | up | mRNA |
| NM_001007523 | F8A2 | 0.184619597 | 0.668017166 | 1.1352633 | up | mRNA |
| NM_001007525 | NWD1 | 0.608503373 | 0.943053241 | 1.0643152 | up | mRNA |
| NM_001007530 | CDRT15 | 0.564802272 | 0.929820378 | 1.112304 | up | mRNA |
| NM_001007553 | CSDE1 | 0.954179278 | 0.996710991 | 1.00605 | up | mRNA |
| NM_001007559 | SS18 | 0.557630791 | 0.926833616 | 1.1222101 | up | mRNA |
| NM_001007565 | TFG | 0.843696196 | 0.987338076 | 1.0186968 | up | mRNA |
| NM_001007593 | SMPD1 | 0.34686349 | 0.833567116 | 1.0356829 | up | mRNA |
| NM_001008211 | OPTN | 0.017216325 | 0.263784715 | 2.8398384 | up | mRNA |
| NM_001008218 | AMY1B | 0.258051492 | 0.758836353 | 1.1207295 | up | mRNA |
| NM_001008219 | AMY1C | 0.81340981 | 0.98120545 | 1.0128841 | up | mRNA |
| NM_001008225 | CNOT4 | 0.467279234 | 0.896646168 | 1.0604777 | up | mRNA |
| NM_001008237 | TTC32 | 0.00678232 | 0.183552613 | 1.1760879 | up | mRNA |
| NM_001008387 | REG3G | 0.145122281 | 0.606482829 | 1.2206053 | up | mRNA |
| NM_001008388 | CISD2 | 0.352419375 | 0.835946719 | 1.0574978 | up | mRNA |
| NM_001008390 | CGGBP1 | 0.695487532 | 0.96236244 | 1.0217522 | up | mRNA |
| NM_001008394 | EID3 | 0.620236047 | 0.947455997 | 1.0534057 | up | mRNA |
| NM_001008395 | C7orf59 | 0.353535346 | 0.836405054 | 1.0812663 | up | mRNA |
| NM_001008410 | STEAP3 | 0.755178435 | 0.971417904 | 1.0386224 | up | mRNA |
| NM_001008486 | SLC41A3 | 0.79708868 | 0.980667273 | 1.0420666 | up | mRNA |
| NM_001008496 | PIWIL3 | 0.053311098 | 0.415136437 | 1.4855856 | up | mRNA |
| NM_001008505 | OPRM1 | 0.190455686 | 0.676402188 | 1.2349938 | up | mRNA |
| NM_001008530 | LGMN | 0.749430625 | 0.970725371 | 1.0898297 | up | mRNA |
| NM_001008535 | AKAP14 | 0.516073794 | 0.914546356 | 1.0790775 | up | mRNA |
| NM_001008539 | SLC7A2 | 0.535367657 | 0.921528548 | 1.0819869 | up | mRNA |
| NM_001008541 | MXI1 | 0.971039644 | 0.998441289 | 1.0143819 | up | mRNA |
| NM_001008564 | NUPL1 | 0.190132458 | 0.676189108 | 1.0545842 | up | mRNA |
| NM_001008657 | TCOF1 | 0.634792399 | 0.951279916 | 1.0556113 | up | mRNA |
| NM_001008661 | CCBL2 | 0.984424874 | 0.998971807 | 1.0078952 | up | mRNA |
| NM_001008701 | LPHN1 | 0.522441038 | 0.917200998 | 1.0556387 | up | mRNA |
| NM_001008707 | EML1 | 0.319768445 | 0.813912372 | 1.2242926 | up | mRNA |
| NM_001008711 | RBPMS | 0.763107058 | 0.97349057 | 1.0161568 | up | mRNA |
| NM_001008739 | C6orf226 | 0.805827965 | 0.98118927 | 1.0376805 | up | mRNA |
| NM_001008800 | CCT3 | 0.518114652 | 0.916125349 | 1.0490968 | up | mRNA |
| NM_001008801 | ZNF468 | 0.662896937 | 0.958354006 | 1.0454292 | up | mRNA |
| NM_001008895 | CUL4A | 0.999719389 | 0.999954265 | 1.0000455 | up | mRNA |
| NM_001008910 | STK16 | 0.449545085 | 0.888279993 | 1.1342977 | up | mRNA |
| NM_001008925 | RCHY1 | 0.953775544 | 0.996710991 | 1.0230614 | up | mRNA |
| NM_001009184 | GRINA | 0.571111032 | 0.933029103 | 1.0382865 | up | mRNA |
| NM_001009552 | PPP2CB | 0.753373787 | 0.970725371 | 1.033613 | up | mRNA |
| NM_001009566 | CLSTN1 | 0.738119982 | 0.969207218 | 1.0269058 | up | mRNA |
| NM_001009611 | PRAMEF4 | 0.154664886 | 0.622213869 | 1.0664135 | up | mRNA |
| NM_001009616 | SPANXN5 | 0.825102592 | 0.98381451 | 1.0436275 | up | mRNA |
| NM_001009812 | LBX2 | 0.640821631 | 0.953894897 | 1.0651605 | up | mRNA |
| NM_001009923 | TMEM230 | 0.586461745 | 0.935504488 | 1.0719122 | up | mRNA |
| NM_001009936 | PHF19 | 0.868102857 | 0.989481868 | 1.0444902 | up | mRNA |
| NM_001009955 | SSBP3 | 0.667179573 | 0.958354006 | 1.0517847 | up | mRNA |
| NM_001009958 | ZNF655 | 0.706591134 | 0.965401045 | 1.0288915 | up | mRNA |
| NM_001009960 | ZNF655 | 0.813748363 | 0.98120545 | 1.0233927 | up | mRNA |
| NM_001009984 | C20orf194 | 0.841359929 | 0.987068842 | 1.0150192 | up | mRNA |
| NM_001009991 | SYTL3 | 0.000327817 | 0.056781055 | 2.2613598 | up | mRNA |
| NM_001009997 | C10orf62 | 0.864851266 | 0.989401142 | 1.0365097 | up | mRNA |
| NM_001009998 | SSBP4 | 0.478129871 | 0.901271944 | 1.0283478 | up | mRNA |
| NM_001009999 | KDM1A | 0.582376444 | 0.934005566 | 1.0363675 | up | mRNA |
| NM_001010 | RPS6 | 0.112831962 | 0.555760453 | 1.0637177 | up | mRNA |
| NM_001010847 | LRRC38 | 0.506770954 | 0.911506285 | 1.1295538 | up | mRNA |
| NM_001010855 | PIK3R6 | 0.983858077 | 0.998971807 | 1.008487 | up | mRNA |
| NM_001010858 | RNF187 | 0.296188244 | 0.79250767 | 1.188443 | up | mRNA |
| NM_001010859 | C22orf42 | 0.600986055 | 0.941023551 | 1.2864851 | up | mRNA |
| NM_001010867 | IBA57 | 0.696161119 | 0.962410025 | 1.1168637 | up | mRNA |
| NM_001010890 | PRAMEF9 | 0.183253335 | 0.666292349 | 1.1342365 | up | mRNA |
| NM_001010892 | RSPH4A | 0.407470458 | 0.867591329 | 1.4624881 | up | mRNA |
| NM_001010895 | ERCC6L2 | 0.680986603 | 0.960111041 | 1.0596858 | up | mRNA |
| NM_001010898 | SLC6A17 | 0.082429491 | 0.489943729 | 1.3653997 | up | mRNA |
| NM_001010906 | NUGGC | 0.884980407 | 0.992981836 | 1.0646131 | up | mRNA |
| NM_001010909 | MUC21 | 0.078589903 | 0.480619753 | 1.3668712 | up | mRNA |
| NM_001010915 | PTPLAD2 | 0.191828371 | 0.67759806 | 1.1586094 | up | mRNA |
| NM_001010927 | TIAM2 | 0.003484084 | 0.138138947 | 2.3165572 | up | mRNA |
| NM_001010931 | HGF | 0.867576024 | 0.989481868 | 1.0834069 | up | mRNA |
| NM_001010934 | HGF | 0.250914636 | 0.750565892 | 1.3058575 | up | mRNA |
| NM_001010942 | RAP1B | 0.352437458 | 0.835946719 | 1.1335146 | up | mRNA |
| NM_001010982 | AFMID | 0.871308809 | 0.98985097 | 1.0255545 | up | mRNA |
| NM_001010986 | ATP11C | 0.070920996 | 0.460531803 | 1.180674 | up | mRNA |
| NM_001011 | RPS7 | 0.985622958 | 0.998971807 | 1.004379 | up | mRNA |
| NM_001011547 | SLC5A9 | 0.458151951 | 0.892646883 | 1.0435023 | up | mRNA |
| NM_001011666 | CREB5 | 0.398384711 | 0.863627032 | 1.0829875 | up | mRNA |
| NM_001011668 | CHCHD7 | 0.579412665 | 0.933821303 | 1.1740222 | up | mRNA |
| NM_001011669 | CHCHD7 | 0.543861782 | 0.923577434 | 1.204766 | up | mRNA |
| NM_001011670 | CHCHD7 | 0.676819054 | 0.959531882 | 1.1231147 | up | mRNA |
| NM_001011671 | CHCHD7 | 0.592399452 | 0.937765934 | 1.1662749 | up | mRNA |
| NM_001011719 | ARSH | 0.244920713 | 0.743548975 | 1.1384816 | up | mRNA |
| NM_001011722 | ARHGEF10L | 0.563812364 | 0.929266617 | 1.0805738 | up | mRNA |
| NM_001011878 | DEFB121 | 0.947311052 | 0.996710991 | 1.0105708 | up | mRNA |
| NM_001012 | RPS8 | 0.843739459 | 0.987338076 | 1.0187292 | up | mRNA |
| NM_001012267 | CENPP | 0.691011491 | 0.961105466 | 1.1291498 | up | mRNA |
| NM_001012277 | PRAMEF7 | 0.975135513 | 0.998501532 | 1.0031109 | up | mRNA |
| NM_001012279 | SOGA3 | 0.917163421 | 0.995349793 | 1.0107725 | up | mRNA |
| NM_001012334 | MDK | 0.904639394 | 0.99508468 | 1.0274207 | up | mRNA |
| NM_001012337 | ROPN1B | 0.449958764 | 0.888279993 | 1.4953068 | up | mRNA |
| NM_001012361 | WDR31 | 0.891288156 | 0.9937521 | 1.0312937 | up | mRNA |
| NM_001012393 | OPCML | 0.899699667 | 0.994708735 | 1.0070576 | up | mRNA |
| NM_001012409 | SGOL1 | 0.696603124 | 0.962410025 | 1.2132575 | up | mRNA |
| NM_001012418 | MYLK4 | 0.840562421 | 0.987068842 | 1.0418641 | up | mRNA |
| NM_001012446 | FAM221B | 0.575607785 | 0.933656777 | 1.0751044 | up | mRNA |
| NM_001012454 | FAM71F2 | 0.304598452 | 0.800038203 | 1.2863577 | up | mRNA |
| NM_001012456 | SEC61G | 0.556123212 | 0.926833616 | 1.1074869 | up | mRNA |
| NM_001012478 | U2AF2 | 0.305261798 | 0.801056074 | 1.0723963 | up | mRNA |
| NM_001012505 | FOXP1 | 0.131522296 | 0.586786184 | 1.18907 | up | mRNA |
| NM_001012509 | SLC45A2 | 0.235024675 | 0.732452741 | 1.206692 | up | mRNA |
| NM_001012511 | GOSR2 | 0.033908967 | 0.349511805 | 1.1139909 | up | mRNA |
| NM_001012514 | ITM2C | 0.624238706 | 0.948341211 | 1.0788838 | up | mRNA |
| NM_001012516 | ITM2C | 0.79422849 | 0.979253685 | 1.0373036 | up | mRNA |
| NM_001012633 | IL32 | 0.071845492 | 0.462266629 | 2.8077168 | up | mRNA |
| NM_001012635 | IL32 | 0.048293424 | 0.398851601 | 1.3098656 | up | mRNA |
| NM_001012750 | ABI1 | 0.031385965 | 0.341322176 | 1.2646344 | up | mRNA |
| NM_001012751 | ABI1 | 0.037021488 | 0.359628653 | 1.2299295 | up | mRNA |
| NM_001012752 | ABI1 | 0.088251101 | 0.502399895 | 1.3235873 | up | mRNA |
| NM_001012763 | GNRHR | 0.180273625 | 0.663104378 | 1.0909583 | up | mRNA |
| NM_001012965 | KLK6 | 0.046097185 | 0.391970965 | 1.2605419 | up | mRNA |
| NM_001012969 | ADAL | 0.613603718 | 0.944706254 | 1.0501499 | up | mRNA |
| NM_001012971 | FAM209A | 0.539041027 | 0.923577434 | 1.0672023 | up | mRNA |
| NM_001012977 | PABPC1L2A | 0.561252498 | 0.928452106 | 1.1042709 | up | mRNA |
| NM_001012984 | C16orf86 | 0.136293142 | 0.595042335 | 1.0983896 | up | mRNA |
| NM_001012993 | C9orf152 | 0.82754297 | 0.984990263 | 1.0265453 | up | mRNA |
| NM_001012994 | SNX30 | 0.444049949 | 0.885088246 | 1.077845 | up | mRNA |
| NM_001013 | RPS9 | 0.194467928 | 0.682224934 | 1.0930866 | up | mRNA |
| NM_001013355 | OR2G6 | 0.798022141 | 0.980749101 | 1.0490824 | up | mRNA |
| NM_001013399 | CCNC | 0.919028814 | 0.995349793 | 1.005362 | up | mRNA |
| NM_001013407 | PRAMEF5 | 0.412654259 | 0.869333523 | 1.1012027 | up | mRNA |
| NM_001013435 | PAGE5 | 0.572692752 | 0.933460721 | 1.1251356 | up | mRNA |
| NM_001013439 | FXR1 | 0.588800852 | 0.935839874 | 1.061683 | up | mRNA |
| NM_001013622 | FAM53A | 0.456720357 | 0.891568474 | 1.0429124 | up | mRNA |
| NM_001013632 | TCTEX1D4 | 0.026017108 | 0.315620427 | 1.7085546 | up | mRNA |
| NM_001013642 | TRNP1 | 0.371824284 | 0.846997196 | 1.1255214 | up | mRNA |
| NM_001013649 | C2orf68 | 0.556189092 | 0.926833616 | 1.0575718 | up | mRNA |
| NM_001013653 | LRRC26 | 0.787338993 | 0.977479172 | 1.0289591 | up | mRNA |
| NM_001013699 | H3F3C | 0.369087736 | 0.84565745 | 1.0784301 | up | mRNA |
| NM_001013706 | PLIN5 | 0.114475397 | 0.558534358 | 1.1785728 | up | mRNA |
| NM_001013742 | DGKK | 0.911508698 | 0.995342392 | 1.0211981 | up | mRNA |
| NM_001013841 | STAP2 | 0.173484607 | 0.65174838 | 1.1650588 | up | mRNA |
| NM_001014433 | CUTA | 0.655017387 | 0.957205183 | 1.0144626 | up | mRNA |
| NM_001014434 | LHX9 | 0.28699922 | 0.782954059 | 1.2638015 | up | mRNA |
| NM_001014444 | CRYM | 0.509062365 | 0.911674633 | 1.3274357 | up | mRNA |
| NM_001014446 | OCIAD2 | 0.510364098 | 0.912097614 | 1.0579207 | up | mRNA |
| NM_001014451 | DDX19B | 0.67160308 | 0.959531882 | 1.0280204 | up | mRNA |
| NM_001014764 | EMC6 | 0.108094801 | 0.544140502 | 1.2077563 | up | mRNA |
| NM_001014796 | DDR2 | 0.027135089 | 0.320017762 | 1.3375188 | up | mRNA |
| NM_001014838 | CUTA | 0.304315398 | 0.800038203 | 1.0303299 | up | mRNA |
| NM_001014842 | TM9SF1 | 0.200715647 | 0.68900633 | 1.1080071 | up | mRNA |
| NM_001014975 | CFH | 0.010225503 | 0.20936306 | 1.2245745 | up | mRNA |
| NM_001014977 | BANF2 | 0.157442792 | 0.626563648 | 1.1045946 | up | mRNA |
| NM_001014985 | GLTPD2 | 0.058017955 | 0.429109154 | 1.236231 | up | mRNA |
| NM_001014999 | SLX1A | 0.771010034 | 0.975347132 | 1.0414073 | up | mRNA |
| NM_001015 | RPS11 | 0.345311053 | 0.832533915 | 1.0137454 | up | mRNA |
| NM_001015072 | UFSP1 | 0.010536759 | 0.210032734 | 1.0878618 | up | mRNA |
| NM_001015880 | PAPSS2 | 0.620644373 | 0.9478661 | 1.0394805 | up | mRNA |
| NM_001015892 | TAF9 | 0.070498831 | 0.458976058 | 1.1481549 | up | mRNA |
| NM_001016 | RPS12 | 0.948817493 | 0.996710991 | 1.0016228 | up | mRNA |
| NM_001017 | RPS13 | 0.063742633 | 0.44127178 | 1.0671466 | up | mRNA |
| NM_001017367 | C4BPB | 0.190530151 | 0.676402188 | 1.3105204 | up | mRNA |
| NM_001017368 | RFFL | 0.804796143 | 0.98118927 | 1.0731884 | up | mRNA |
| NM_001017390 | SULT1A4 | 0.820822227 | 0.982756843 | 1.019765 | up | mRNA |
| NM_001017403 | LGR6 | 0.35827515 | 0.840546205 | 1.0563339 | up | mRNA |
| NM_001017404 | LGR6 | 0.603910362 | 0.941514447 | 1.0697808 | up | mRNA |
| NM_001017417 | CT45A1 | 0.884521995 | 0.99277401 | 1.0136335 | up | mRNA |
| NM_001017418 | SPRR2B | 0.380883783 | 0.853514109 | 1.077478 | up | mRNA |
| NM_001017420 | ESCO2 | 0.991317541 | 0.999477747 | 1.0079469 | up | mRNA |
| NM_001017438 | CT45A6 | 0.544354621 | 0.923577434 | 1.0666937 | up | mRNA |
| NM_001017440 | CALN1 | 0.245298297 | 0.743589595 | 1.0611558 | up | mRNA |
| NM_001017523 | BTBD11 | 0.52867398 | 0.918883792 | 1.0634107 | up | mRNA |
| NM_001017915 | INPP5D | 0.080011842 | 0.483876854 | 1.091991 | up | mRNA |
| NM_001017963 | HSP90AA1 | 0.840834082 | 0.987068842 | 1.1100556 | up | mRNA |
| NM_001017980 | VMA21 | 0.09879933 | 0.522577515 | 1.19667 | up | mRNA |
| NM_001017986 | FCGR1B | 0.281345958 | 0.779126326 | 1.2470026 | up | mRNA |
| NM_001017992 | ACTBL2 | 0.643879244 | 0.954605197 | 1.0379278 | up | mRNA |
| NM_001018016 | MUC1 | 0.556729733 | 0.926833616 | 1.0678227 | up | mRNA |
| NM_001018017 | MUC1 | 0.037073805 | 0.359628653 | 1.1147061 | up | mRNA |
| NM_001018036 | TSHR | 0.545782571 | 0.92424063 | 1.1043971 | up | mRNA |
| NM_001018038 | VPS13A | 0.615344225 | 0.945385967 | 1.076133 | up | mRNA |
| NM_001018066 | NTRK2 | 0.528223774 | 0.918883792 | 1.0537169 | up | mRNA |
| NM_001018070 | CORO1B | 0.921065179 | 0.995349793 | 1.0052703 | up | mRNA |
| NM_001018078 | FPGS | 0.844921484 | 0.987338076 | 1.0146143 | up | mRNA |
| NM_001018082 | ADIG | 0.37675918 | 0.85040519 | 1.1267891 | up | mRNA |
| NM_001018108 | SERF2 | 0.903396413 | 0.99508468 | 1.0132304 | up | mRNA |
| NM_001018136 | NME1-NME2 | 0.479680968 | 0.901538317 | 1.0409437 | up | mRNA |
| NM_001018159 | NAE1 | 0.698908309 | 0.962701213 | 1.0334669 | up | mRNA |
| NM_001019 | RPS15A | 0.327462421 | 0.820319134 | 1.0676738 | up | mRNA |
| NM_001020 | RPS16 | 0.010363623 | 0.209736088 | 1.1239389 | up | mRNA |
| NM_001023 | RPS20 | 0.654245905 | 0.957205183 | 1.0305473 | up | mRNA |
| NM_001023567 | GOLGA8B | 0.989531688 | 0.999140676 | 1.0048123 | up | mRNA |
| NM_001023587 | ABCC5 | 0.767972455 | 0.975114674 | 1.1037051 | up | mRNA |
| NM_001024 | RPS21 | 0.920283163 | 0.995349793 | 1.0058738 | up | mRNA |
| NM_001024024 | GCH1 | 0.009434395 | 0.204717305 | 11.4742944 | up | mRNA |
| NM_001024070 | GCH1 | 0.011656101 | 0.22025265 | 11.4811321 | up | mRNA |
| NM_001024071 | GCH1 | 0.007630001 | 0.193257016 | 11.4709802 | up | mRNA |
| NM_001024074 | HNMT | 0.376033423 | 0.849862269 | 1.4879128 | up | mRNA |
| NM_001024075 | HNMT | 0.526575142 | 0.917975454 | 1.0781452 | up | mRNA |
| NM_001024211 | S100A13 | 0.55674121 | 0.926833616 | 1.0600684 | up | mRNA |
| NM_001024216 | FBLIM1 | 0.030930218 | 0.340185047 | 1.3038772 | up | mRNA |
| NM_001024227 | ARF1 | 0.213938867 | 0.706596013 | 1.0359186 | up | mRNA |
| NM_001024372 | BAALC | 0.653443063 | 0.956860606 | 1.1032533 | up | mRNA |
| NM_001024466 | SOD2 | 0.000122604 | 0.040865428 | 7.0778399 | up | mRNA |
| NM_001024594 | C1orf53 | 0.613248724 | 0.944565792 | 1.095191 | up | mRNA |
| NM_001024601 | C1orf227 | 0.046135861 | 0.391970965 | 1.5631585 | up | mRNA |
| NM_001024607 | C7orf66 | 0.598270988 | 0.940397814 | 1.0819462 | up | mRNA |
| NM_001024613 | FEZF1 | 0.923161848 | 0.995430245 | 1.0021193 | up | mRNA |
| NM_001024628 | NRP1 | 0.915043991 | 0.995349793 | 1.0095122 | up | mRNA |
| NM_001024629 | NRP1 | 0.338052059 | 0.827403747 | 1.5986007 | up | mRNA |
| NM_001024630 | RUNX2 | 0.376346839 | 0.849903337 | 1.5736278 | up | mRNA |
| NM_001024644 | XCR1 | 0.390315965 | 0.858920947 | 1.1819709 | up | mRNA |
| NM_001024647 | RAB3IP | 0.752302313 | 0.970725371 | 1.1506035 | up | mRNA |
| NM_001024662 | RPL6 | 0.439670114 | 0.883054028 | 1.1546863 | up | mRNA |
| NM_001024666 | SH3KBP1 | 0.407277636 | 0.867591329 | 1.2546597 | up | mRNA |
| NM_001024680 | FBXO48 | 0.026754441 | 0.318484367 | 1.2618826 | up | mRNA |
| NM_001024683 | ZNF688 | 0.500854006 | 0.909680616 | 1.0471746 | up | mRNA |
| NM_001024736 | CD276 | 0.091643705 | 0.510719066 | 1.1073001 | up | mRNA |
| NM_001024808 | BCL7A | 0.640259695 | 0.953891706 | 1.0540899 | up | mRNA |
| NM_001024843 | TNRC6B | 0.76086622 | 0.972945894 | 1.0493178 | up | mRNA |
| NM_001024844 | CD82 | 0.002077398 | 0.109993922 | 2.0862355 | up | mRNA |
| NM_001024845 | SLC6A9 | 0.464309483 | 0.894989844 | 1.1624133 | up | mRNA |
| NM_001024847 | TGFBR2 | 0.109437292 | 0.547509603 | 1.0614271 | up | mRNA |
| NM_001024916 | CBWD5 | 0.88018262 | 0.992197209 | 1.0136491 | up | mRNA |
| NM_001024921 | RPL9 | 0.642216624 | 0.954332328 | 1.0377137 | up | mRNA |
| NM_001024937 | MINK1 | 0.293327683 | 0.790610868 | 1.1259073 | up | mRNA |
| NM_001024948 | FNBP1L | 0.329898605 | 0.821768501 | 1.4195957 | up | mRNA |
| NM_001025 | RPS23 | 0.095665952 | 0.51703353 | 1.1069333 | up | mRNA |
| NM_001025071 | RPS14 | 0.753098889 | 0.970725371 | 1.0133784 | up | mRNA |
| NM_001025077 | CELF2 | 0.139132431 | 0.598648178 | 1.2797914 | up | mRNA |
| NM_001025081 | MBP | 0.326123864 | 0.818914463 | 1.2001974 | up | mRNA |
| NM_001025088 | CELF4 | 0.176755918 | 0.656282198 | 1.1473146 | up | mRNA |
| NM_001025091 | ABCF1 | 0.044780034 | 0.38849334 | 1.2075008 | up | mRNA |
| NM_001025092 | MBP | 0.387880602 | 0.857122965 | 1.2068175 | up | mRNA |
| NM_001025105 | CSNK1A1 | 0.614561387 | 0.944827645 | 1.0515841 | up | mRNA |
| NM_001025108 | AFF3 | 0.061531624 | 0.437893235 | 1.6220957 | up | mRNA |
| NM_001025160 | CD97 | 0.692101201 | 0.961451327 | 1.1185356 | up | mRNA |
| NM_001025161 | CYP2D6 | 0.654504891 | 0.957205183 | 1.0460267 | up | mRNA |
| NM_001025194 | CES1 | 0.681933528 | 0.960111041 | 1.1095181 | up | mRNA |
| NM_001025195 | CES1 | 0.344453694 | 0.831959196 | 1.1301045 | up | mRNA |
| NM_001025199 | CHI3L2 | 0.381324525 | 0.853514109 | 1.2860979 | up | mRNA |
| NM_001025201 | CHN1 | 0.955205878 | 0.996883425 | 1.0291735 | up | mRNA |
| NM_001025243 | IRAK1 | 0.432804776 | 0.88061258 | 1.1883477 | up | mRNA |
| NM_001025247 | TAF5L | 0.270357607 | 0.770199084 | 1.0849419 | up | mRNA |
| NM_001025356 | ANO6 | 0.585603818 | 0.934869412 | 1.0683485 | up | mRNA |
| NM_001025366 | VEGFA | 0.006655796 | 0.183552613 | 1.5468252 | up | mRNA |
| NM_001025367 | VEGFA | 0.113197224 | 0.55632728 | 1.3472451 | up | mRNA |
| NM_001025368 | VEGFA | 0.061491149 | 0.437893235 | 1.4725865 | up | mRNA |
| NM_001025369 | VEGFA | 0.069604352 | 0.457632202 | 1.4154249 | up | mRNA |
| NM_001025370 | VEGFA | 0.03131153 | 0.341171508 | 1.364337 | up | mRNA |
| NM_001025390 | AMPD3 | 0.001775167 | 0.105670067 | 4.7999103 | up | mRNA |
| NM_001025596 | CELF1 | 0.16008706 | 0.6318948 | 1.0579128 | up | mRNA |
| NM_001025598 | ARHGAP30 | 0.725136191 | 0.966191059 | 1.0473512 | up | mRNA |
| NM_001025603 | RFX5 | 0.252417012 | 0.752621656 | 1.1870146 | up | mRNA |
| NM_001025604 | ARRDC2 | 0.854503599 | 0.988044084 | 1.055721 | up | mRNA |
| NM_001025778 | VRK3 | 0.687060468 | 0.960205549 | 1.0335808 | up | mRNA |
| NM_001025930 | TTLL3 | 0.503281976 | 0.91053927 | 1.0370021 | up | mRNA |
| NM_001029 | RPS26 | 0.211131904 | 0.702369538 | 1.149663 | up | mRNA |
| NM_001029835 | CCM2 | 0.22372235 | 0.717822772 | 1.2540021 | up | mRNA |
| NM_001029863 | C6orf120 | 0.895801049 | 0.993978977 | 1.0258387 | up | mRNA |
| NM_001029871 | RSPO4 | 0.587805324 | 0.93579682 | 1.0874042 | up | mRNA |
| NM_001029881 | CIB4 | 0.043241215 | 0.383753983 | 1.121528 | up | mRNA |
| NM_001029896 | WDR45 | 0.572882553 | 0.93352894 | 1.0826199 | up | mRNA |
| NM_001029954 | CDNF | 0.069018198 | 0.455905782 | 1.5433615 | up | mRNA |
| NM_001029964 | TTLL13 | 0.200865455 | 0.68900633 | 1.2893111 | up | mRNA |
| NM_001029989 | KIAA0101 | 0.877616979 | 0.991306146 | 1.0930585 | up | mRNA |
| NM_001030 | RPS27 | 0.766616667 | 0.974840715 | 1.010648 | up | mRNA |
| NM_001030003 | HNF4A | 0.107929193 | 0.544140502 | 1.1587841 | up | mRNA |
| NM_001030010 | ALDH3B1 | 0.243237809 | 0.741678336 | 1.0572279 | up | mRNA |
| NM_001030015 | OPN4 | 0.625074605 | 0.948700498 | 1.100932 | up | mRNA |
| NM_001030018 | APRT | 0.991512729 | 0.999495983 | 1.0016198 | up | mRNA |
| NM_001030060 | SAMD5 | 0.437425846 | 0.882322896 | 1.0967846 | up | mRNA |
| NM_001030287 | ATF3 | 0.898736185 | 0.99453218 | 1.0130725 | up | mRNA |
| NM_001030311 | CERKL | 0.788666743 | 0.977754404 | 1.0479068 | up | mRNA |
| NM_001031 | RPS28 | 0.541122969 | 0.923577434 | 1.0599303 | up | mRNA |
| NM_001031617 | COX19 | 0.914264465 | 0.995342392 | 1.0117422 | up | mRNA |
| NM_001031618 | SPDYE2 | 0.549828488 | 0.925533435 | 1.0940899 | up | mRNA |
| NM_001031672 | CYB5RL | 0.219153369 | 0.71361728 | 1.1272561 | up | mRNA |
| NM_001031681 | CTNS | 0.810493035 | 0.98118927 | 1.0236693 | up | mRNA |
| NM_001031685 | TP53BP2 | 0.003729987 | 0.141753453 | 2.7322662 | up | mRNA |
| NM_001031694 | SCMH1 | 0.572687089 | 0.933460721 | 1.0537871 | up | mRNA |
| NM_001031698 | PRPF40B | 0.774397651 | 0.97589756 | 1.0154351 | up | mRNA |
| NM_001031700 | FAM198B | 0.999305654 | 0.999897336 | 1.0004996 | up | mRNA |
| NM_001031702 | SEMA5B | 0.078272761 | 0.480303439 | 1.0758446 | up | mRNA |
| NM_001031709 | RNLS | 0.588501039 | 0.93579682 | 1.0565141 | up | mRNA |
| NM_001031710 | KLHL7 | 0.04253933 | 0.381521685 | 1.2613553 | up | mRNA |
| NM_001031711 | ERGIC1 | 0.684719098 | 0.960111041 | 1.0778079 | up | mRNA |
| NM_001031713 | CCDC90A | 0.246206413 | 0.744847716 | 1.1111357 | up | mRNA |
| NM_001031714 | INF2 | 0.484410235 | 0.903250293 | 1.0863648 | up | mRNA |
| NM_001031715 | IQCH | 0.127860473 | 0.581220503 | 1.4894269 | up | mRNA |
| NM_001031716 | NABP1 | 0.498498625 | 0.909361611 | 1.2284068 | up | mRNA |
| NM_001031719 | DHRS12 | 0.865433266 | 0.989401142 | 1.056646 | up | mRNA |
| NM_001031738 | TMEM150A | 0.003665186 | 0.140604851 | 1.2490049 | up | mRNA |
| NM_001031739 | ASB9 | 0.998341503 | 0.999889342 | 1.0006539 | up | mRNA |
| NM_001031745 | RIBC1 | 0.132258477 | 0.587843174 | 1.1586008 | up | mRNA |
| NM_001031746 | VSTM4 | 0.199907573 | 0.68900633 | 1.153258 | up | mRNA |
| NM_001031749 | LYPD5 | 0.415694662 | 0.870382888 | 1.04587 | up | mRNA |
| NM_001031800 | TIPRL | 0.213780264 | 0.706323665 | 1.0956171 | up | mRNA |
| NM_001031801 | LIMK2 | 0.963919201 | 0.99793927 | 1.0099845 | up | mRNA |
| NM_001031804 | MAF | 0.785350869 | 0.977411899 | 1.040306 | up | mRNA |
| NM_001031848 | SERPINB8 | 0.056445545 | 0.423551663 | 1.6700411 | up | mRNA |
| NM_001031850 | PSG6 | 0.24428863 | 0.742654967 | 1.3050195 | up | mRNA |
| NM_001032278 | MMP28 | 0.449294685 | 0.888279993 | 1.0966096 | up | mRNA |
| NM_001032280 | TFAP2A | 0.852763709 | 0.987678166 | 1.0174903 | up | mRNA |
| NM_001032281 | TFPI | 0.961130518 | 0.99793927 | 1.0102593 | up | mRNA |
| NM_001032282 | KLF10 | 0.008821443 | 0.202329498 | 1.272261 | up | mRNA |
| NM_001032288 | UBE2V1 | 0.533363541 | 0.920530493 | 1.1259221 | up | mRNA |
| NM_001032291 | PSRC1 | 0.338006223 | 0.827394958 | 1.1038194 | up | mRNA |
| NM_001032293 | ZNF207 | 0.75186998 | 0.970725371 | 1.0202622 | up | mRNA |
| NM_001032387 | SUOX | 0.049960797 | 0.40410612 | 1.1905112 | up | mRNA |
| NM_001032409 | OAS1 | 0.798605561 | 0.980749101 | 1.1025428 | up | mRNA |
| NM_001032410 | USP16 | 0.446626515 | 0.886733447 | 1.0698113 | up | mRNA |
| NM_001033002 | RPAIN | 0.588667766 | 0.93579682 | 1.0875512 | up | mRNA |
| NM_001033030 | FAIM | 0.155406587 | 0.623056705 | 1.3952819 | up | mRNA |
| NM_001033056 | GLUL | 0.345589774 | 0.832533915 | 1.0831648 | up | mRNA |
| NM_001033117 | SRGAP3 | 0.463588375 | 0.894989844 | 1.0681872 | up | mRNA |
| NM_001033519 | WIPI2 | 0.773991879 | 0.97589756 | 1.0396308 | up | mRNA |
| NM_001033520 | WIPI2 | 0.025973289 | 0.315604326 | 1.2384785 | up | mRNA |
| NM_001033551 | TOM1L2 | 0.592539411 | 0.937776808 | 1.0317396 | up | mRNA |
| NM_001033553 | SPECC1 | 0.122776181 | 0.574983504 | 1.8139516 | up | mRNA |
| NM_001033555 | SPECC1 | 0.571721861 | 0.933299883 | 1.0595418 | up | mRNA |
| NM_001033564 | C6orf225 | 0.138888445 | 0.598648178 | 1.1220446 | up | mRNA |
| NM_001033576 | UNC45B | 0.089422139 | 0.505598147 | 1.3354006 | up | mRNA |
| NM_001033578 | SGK3 | 0.768796863 | 0.97524194 | 1.034333 | up | mRNA |
| NM_001033582 | PRKCZ | 0.90278121 | 0.99508468 | 1.0058527 | up | mRNA |
| NM_001033583 | ACOT9 | 0.276152869 | 0.775838072 | 1.1048632 | up | mRNA |
| NM_001033602 | MTUS2 | 0.309120549 | 0.803961878 | 1.2595112 | up | mRNA |
| NM_001033667 | LY9 | 0.538632435 | 0.923577434 | 1.1222088 | up | mRNA |
| NM_001033677 | CABP1 | 0.548254477 | 0.92514258 | 1.0666937 | up | mRNA |
| NM_001033756 | VEGFA | 0.076454741 | 0.47552089 | 1.4143432 | up | mRNA |
| NM_001033886 | CXCL12 | 0.362222012 | 0.843173209 | 1.1798436 | up | mRNA |
| NM_001034172 | SLC25A52 | 0.002532478 | 0.11946717 | 1.4306806 | up | mRNA |
| NM_001034850 | FAM134B | 0.734370692 | 0.967777568 | 1.0706794 | up | mRNA |
| NM_001034852 | SMOC1 | 0.931845458 | 0.996710991 | 1.0386685 | up | mRNA |
| NM_001034915 | ESRP1 | 0.672338921 | 0.959531882 | 1.0562071 | up | mRNA |
| NM_001035005 | C18orf32 | 0.793866727 | 0.97921643 | 1.0261673 | up | mRNA |
| NM_001035223 | RGL3 | 0.815727566 | 0.981459009 | 1.0220284 | up | mRNA |
| NM_001035230 | RABGAP1L | 0.222493855 | 0.71737156 | 1.0468117 | up | mRNA |
| NM_001035247 | UFD1L | 0.679340575 | 0.959969193 | 1.0248239 | up | mRNA |
| NM_001035258 | RPL38 | 0.625486756 | 0.948847028 | 1.0122977 | up | mRNA |
| NM_001035267 | RPL41 | 0.100814468 | 0.527707289 | 1.1624586 | up | mRNA |
| NM_001035505 | BOLA3 | 0.966781951 | 0.99793927 | 1.0098386 | up | mRNA |
| NM_001035511 | SDHC | 0.714811094 | 0.966110889 | 1.0182451 | up | mRNA |
| NM_001035512 | SDHC | 0.644727777 | 0.954657712 | 1.0239754 | up | mRNA |
| NM_001035513 | SDHC | 0.537431669 | 0.922702335 | 1.0289287 | up | mRNA |
| NM_001035516 | DMKN | 0.074192395 | 0.469200549 | 1.1162153 | up | mRNA |
| NM_001035521 | GTF3C2 | 0.280452519 | 0.778877286 | 1.1917196 | up | mRNA |
| NM_001036646 | HHLA3 | 0.343192575 | 0.831521302 | 1.0894582 | up | mRNA |
| NM_001037 | SCN1B | 0.858488734 | 0.988512246 | 1.0674618 | up | mRNA |
| NM_001037171 | ACOT9 | 0.334988883 | 0.824836547 | 1.0565621 | up | mRNA |
| NM_001037175 | SUSD4 | 0.55082276 | 0.925960004 | 1.1129248 | up | mRNA |
| NM_001037281 | PARD6A | 0.328177729 | 0.820977969 | 1.7147638 | up | mRNA |
| NM_001037293 | PALM2 | 0.814095943 | 0.98120545 | 1.0450249 | up | mRNA |
| NM_001037328 | STAU1 | 0.163292647 | 0.635463018 | 1.131241 | up | mRNA |
| NM_001037329 | CNGA4 | 0.785271931 | 0.977411899 | 1.0395819 | up | mRNA |
| NM_001037332 | CYFIP2 | 0.6294203 | 0.950410442 | 1.0719036 | up | mRNA |
| NM_001037335 | PRIC285 | 0.526150832 | 0.917975454 | 1.3078273 | up | mRNA |
| NM_001037339 | PDE4B | 0.000293101 | 0.055178904 | 4.6076262 | up | mRNA |
| NM_001037340 | PDE4B | 0.690371669 | 0.961105466 | 1.0197634 | up | mRNA |
| NM_001037536 | SCML1 | 0.6795139 | 0.959969193 | 1.0235138 | up | mRNA |
| NM_001037540 | SCML1 | 0.009657317 | 0.205650016 | 1.5984202 | up | mRNA |
| NM_001037558 | HEPN1 | 0.465157632 | 0.895224488 | 1.1853399 | up | mRNA |
| NM_001037582 | SCD5 | 0.302358551 | 0.797857839 | 1.0671314 | up | mRNA |
| NM_001037666 | GATSL3 | 0.180166694 | 0.662944156 | 1.248784 | up | mRNA |
| NM_001037668 | DEFB107A | 0.767236492 | 0.975033576 | 1.082482 | up | mRNA |
| NM_001037866 | RGPD6 | 0.327199206 | 0.820196554 | 1.1947652 | up | mRNA |
| NM_001037954 | DIXDC1 | 0.354806843 | 0.83766056 | 1.1168919 | up | mRNA |
| NM_001038707 | CDC42SE1 | 0.541862765 | 0.923577434 | 1.0482508 | up | mRNA |
| NM_001039132 | ICAM4 | 0.00031435 | 0.05606455 | 10.4114408 | up | mRNA |
| NM_001039140 | C20orf27 | 0.937153416 | 0.996710991 | 1.0062529 | up | mRNA |
| NM_001039178 | ATP5J2 | 0.426117809 | 0.875717896 | 1.0308578 | up | mRNA |
| NM_001039367 | ATP6V1E1 | 0.892597087 | 0.993978977 | 1.0106346 | up | mRNA |
| NM_001039372 | HEPACAM2 | 0.56657067 | 0.931095609 | 1.0478468 | up | mRNA |
| NM_001039396 | MPEG1 | 0.437116673 | 0.882217212 | 1.1451276 | up | mRNA |
| NM_001039469 | MARK2 | 0.127523052 | 0.580991132 | 1.2074935 | up | mRNA |
| NM_001039481 | ETNK1 | 0.035984508 | 0.356229271 | 1.5143849 | up | mRNA |
| NM_001039500 | VWA5B1 | 0.585732689 | 0.934928328 | 1.0967617 | up | mRNA |
| NM_001039517 | RUSC1-AS1 | 0.730180671 | 0.966191059 | 1.0298363 | up | mRNA |
| NM_001039523 | CHRNA1 | 0.293609244 | 0.791102989 | 1.266185 | up | mRNA |
| NM_001039547 | GK5 | 0.466796558 | 0.896044904 | 1.067495 | up | mRNA |
| NM_001039567 | RPS4Y2 | 0.89868845 | 0.99453218 | 1.0106999 | up | mRNA |
| NM_001039577 | CCNL2 | 0.954097736 | 0.996710991 | 1.0077807 | up | mRNA |
| NM_001039585 | PTGFR | 0.011069254 | 0.215781465 | 2.1737521 | up | mRNA |
| NM_001039592 | SPAG8 | 0.61462871 | 0.944827645 | 1.0903112 | up | mRNA |
| NM_001039618 | CREBZF | 0.794162901 | 0.979253685 | 1.0201392 | up | mRNA |
| NM_001039716 | DIO1 | 0.384560228 | 0.85485898 | 1.3242417 | up | mRNA |
| NM_001039763 | TMEM232 | 0.541607408 | 0.923577434 | 1.2860203 | up | mRNA |
| NM_001039775 | AIM1L | 0.310614522 | 0.805412658 | 1.0920078 | up | mRNA |
| NM_001039803 | CDK20 | 0.872949775 | 0.990534615 | 1.0104886 | up | mRNA |
| NM_001039842 | OXLD1 | 0.981994459 | 0.998971807 | 1.0021136 | up | mRNA |
| NM_001039845 | MDH1B | 0.883067988 | 0.992766819 | 1.0299592 | up | mRNA |
| NM_001039846 | IZUMO4 | 0.863536488 | 0.989401142 | 1.0064362 | up | mRNA |
| NM_001039876 | SYNE4 | 0.212269725 | 0.704056777 | 1.1318929 | up | mRNA |
| NM_001039877 | STRN4 | 0.472464556 | 0.899184677 | 1.0546752 | up | mRNA |
| NM_001039920 | ZNF384 | 0.223647835 | 0.717791282 | 1.2487327 | up | mRNA |
| NM_001039936 | CHN2 | 0.409933707 | 0.86807846 | 1.0671218 | up | mRNA |
| NM_001039937 | INTS6 | 0.700200177 | 0.962991468 | 1.054038 | up | mRNA |
| NM_001039948 | SGSM1 | 0.69923439 | 0.962701213 | 1.0294744 | up | mRNA |
| NM_001039958 | MESP2 | 0.605512231 | 0.94174007 | 1.0735857 | up | mRNA |
| NM_001039970 | PTPN5 | 0.667717107 | 0.958354006 | 1.015216 | up | mRNA |
| NM_001040007 | RSPO4 | 0.809589539 | 0.98118927 | 1.0623171 | up | mRNA |
| NM_001040011 | SWI5 | 0.120989222 | 0.572400147 | 1.1698672 | up | mRNA |
| NM_001040033 | CD53 | 0.930493524 | 0.996710991 | 1.01288 | up | mRNA |
| NM_001040057 | FAM133B | 0.120005267 | 0.57061438 | 1.0924051 | up | mRNA |
| NM_001040060 | SPP1 | 0.637128982 | 0.952507828 | 1.3162737 | up | mRNA |
| NM_001040061 | C3orf72 | 0.341783567 | 0.830223283 | 1.1344816 | up | mRNA |
| NM_001040102 | OPALIN | 0.321703256 | 0.815271211 | 1.1860175 | up | mRNA |
| NM_001040109 | MLN | 0.596737238 | 0.939585033 | 1.0722449 | up | mRNA |
| NM_001040114 | MYH11 | 0.646512444 | 0.95550188 | 1.078729 | up | mRNA |
| NM_001040125 | PQLC2 | 0.14711665 | 0.609351845 | 1.136958 | up | mRNA |
| NM_001040134 | PALM | 0.123891783 | 0.577744894 | 1.2688544 | up | mRNA |
| NM_001040146 | CHTF8 | 0.67959196 | 0.960007989 | 1.0949542 | up | mRNA |
| NM_001040147 | SERPINB7 | 0.073647781 | 0.467143085 | 1.2842675 | up | mRNA |
| NM_001040160 | C16orf13 | 0.708143438 | 0.965462762 | 1.0441659 | up | mRNA |
| NM_001040162 | C16orf13 | 0.793436669 | 0.979128905 | 1.0240744 | up | mRNA |
| NM_001040167 | LFNG | 0.226827869 | 0.722135151 | 1.0831454 | up | mRNA |
| NM_001040177 | AKR1E2 | 0.28564741 | 0.782403763 | 1.0979885 | up | mRNA |
| NM_001040182 | CLDND1 | 0.951114883 | 0.996710991 | 1.003848 | up | mRNA |
| NM_001040195 | AGTRAP | 0.739478326 | 0.969564098 | 1.0283659 | up | mRNA |
| NM_001040197 | AGTRAP | 0.539753763 | 0.923577434 | 1.2988827 | up | mRNA |
| NM_001040217 | FAM63A | 0.495388624 | 0.907415174 | 1.1114287 | up | mRNA |
| NM_001040261 | DCLK2 | 0.287052081 | 0.782954059 | 1.2804615 | up | mRNA |
| NM_001040273 | TYSND1 | 0.778550386 | 0.976938981 | 1.0529055 | up | mRNA |
| NM_001040275 | ESR2 | 0.193521273 | 0.680621781 | 1.4745009 | up | mRNA |
| NM_001040283 | DMRTC2 | 0.717538183 | 0.966191059 | 1.0280871 | up | mRNA |
| NM_001040411 | C9orf131 | 0.53892569 | 0.923577434 | 1.193073 | up | mRNA |
| NM_001040427 | HAGH | 0.623458416 | 0.948341211 | 1.0673623 | up | mRNA |
| NM_001040429 | PCDH17 | 0.493455122 | 0.90641426 | 1.0506062 | up | mRNA |
| NM_001040436 | YARS2 | 0.113962995 | 0.557835051 | 1.1758625 | up | mRNA |
| NM_001040439 | MAPK8IP3 | 0.833359613 | 0.986434891 | 1.0282018 | up | mRNA |
| NM_001040443 | PHF11 | 0.722540595 | 0.966191059 | 1.047936 | up | mRNA |
| NM_001040445 | ASB1 | 0.016064548 | 0.25477722 | 1.5368423 | up | mRNA |
| NM_001040451 | RUFY1 | 0.619014871 | 0.947298687 | 1.0246481 | up | mRNA |
| NM_001040458 | ERAP1 | 0.036725564 | 0.359628653 | 1.1079689 | up | mRNA |
| NM_001040662 | METTL21D | 0.78841985 | 0.977641695 | 1.012185 | up | mRNA |
| NM_001040663 | GAGE1 | 0.723608084 | 0.966191059 | 1.0981239 | up | mRNA |
| NM_001040664 | PPAN-P2RY11 | 0.700167266 | 0.962991468 | 1.0315862 | up | mRNA |
| NM_001040665 | STEAP2 | 0.843693814 | 0.987338076 | 1.0535722 | up | mRNA |
| NM_001040666 | STEAP2 | 0.788960086 | 0.977807732 | 1.0875241 | up | mRNA |
| NM_001040703 | DEFB105B | 0.518980523 | 0.916145597 | 1.3089463 | up | mRNA |
| NM_001040709 | SYPL2 | 0.482104147 | 0.902092906 | 1.1378771 | up | mRNA |
| NM_001040710 | FAM228A | 0.620585936 | 0.9478661 | 1.0228679 | up | mRNA |
| NM_001040874 | HIST2H2AA4 | 0.152718226 | 0.618416047 | 1.3658859 | up | mRNA |
| NM_001040875 | GDAP1 | 0.65288259 | 0.956714149 | 1.0747494 | up | mRNA |
| NM_001042355 | CYLD | 0.001326792 | 0.093667829 | 1.7427502 | up | mRNA |
| NM_001042358 | PTPN20B | 0.207069495 | 0.698556218 | 1.0947179 | up | mRNA |
| NM_001042359 | PTPN20B | 0.72976066 | 0.966191059 | 1.0318987 | up | mRNA |
| NM_001042368 | RALGDS | 0.94462843 | 0.996710991 | 1.0126722 | up | mRNA |
| NM_001042385 | PREPL | 0.003219341 | 0.133466208 | 1.3754466 | up | mRNA |
| NM_001042387 | PTPN20A | 0.163799406 | 0.63628397 | 1.2760284 | up | mRNA |
| NM_001042388 | PPP4R1 | 0.196992472 | 0.685161614 | 1.0945701 | up | mRNA |
| NM_001042406 | HMGCLL1 | 0.845108284 | 0.987338076 | 1.0441145 | up | mRNA |
| NM_001042413 | GLIS3 | 0.445443556 | 0.885807324 | 1.0524654 | up | mRNA |
| NM_001042414 | PSPC1 | 0.915594393 | 0.995349793 | 1.0135606 | up | mRNA |
| NM_001042417 | RUFY2 | 0.991130062 | 0.999376923 | 1.0012119 | up | mRNA |
| NM_001042440 | CAST | 0.134347879 | 0.590871682 | 1.1964422 | up | mRNA |
| NM_001042452 | MST4 | 0.058404204 | 0.429582734 | 1.626978 | up | mRNA |
| NM_001042453 | MST4 | 0.117516721 | 0.564147103 | 1.337399 | up | mRNA |
| NM_001042454 | TGFB1I1 | 0.937379884 | 0.996710991 | 1.0120721 | up | mRNA |
| NM_001042459 | FILIP1L | 0.693423026 | 0.961584652 | 1.2106585 | up | mRNA |
| NM_001042462 | TRAPPC5 | 0.028791755 | 0.329985324 | 1.1718763 | up | mRNA |
| NM_001042463 | TMEM80 | 0.962889709 | 0.99793927 | 1.0064395 | up | mRNA |
| NM_001042471 | GSDMB | 0.851686144 | 0.987678166 | 1.0227153 | up | mRNA |
| NM_001042478 | AJAP1 | 0.32225413 | 0.815372756 | 1.1043546 | up | mRNA |
| NM_001042500 | DEFA1B | 0.560564263 | 0.92782421 | 1.089376 | up | mRNA |
| NM_001042507 | LGALS7B | 0.3605163 | 0.841780454 | 1.1161498 | up | mRNA |
| NM_001042517 | DIAPH3 | 0.905788099 | 0.99508468 | 1.0559766 | up | mRNA |
| NM_001042522 | SPRED3 | 0.750704493 | 0.970725371 | 1.0305351 | up | mRNA |
| NM_001042529 | COASY | 0.139480489 | 0.598648178 | 1.0647942 | up | mRNA |
| NM_001042540 | NCBP2 | 0.428458411 | 0.87728262 | 1.2220236 | up | mRNA |
| NM_001042544 | LTBP4 | 0.396192248 | 0.861906782 | 1.0721004 | up | mRNA |
| NM_001042548 | FEZ2 | 0.942721244 | 0.996710991 | 1.0073438 | up | mRNA |
| NM_001042572 | CHD2 | 0.148075291 | 0.611080968 | 1.1722213 | up | mRNA |
| NM_001042573 | ENGASE | 0.421820404 | 0.873398499 | 1.0880525 | up | mRNA |
| NM_001042575 | TMPRSS7 | 0.031029556 | 0.340185047 | 1.2548918 | up | mRNA |
| NM_001042583 | CD1E | 0.398959314 | 0.863643939 | 1.2450891 | up | mRNA |
| NM_001042586 | CD1E | 0.2350962 | 0.73249698 | 1.150449 | up | mRNA |
| NM_001042587 | CD1E | 0.461512361 | 0.894667841 | 1.0651555 | up | mRNA |
| NM_001042678 | RHOC | 0.718956967 | 0.966191059 | 1.0290162 | up | mRNA |
| NM_001042681 | RERE | 0.519719881 | 0.916187634 | 1.0862589 | up | mRNA |
| NM_001042693 | FAM159A | 0.322547859 | 0.815372756 | 1.1417532 | up | mRNA |
| NM_001042702 | DFNB59 | 0.643296688 | 0.954522001 | 1.2033585 | up | mRNA |
| NM_001042704 | CLCNKA | 0.677169592 | 0.959531882 | 1.0590047 | up | mRNA |
| NM_001042705 | IQCJ | 0.067643715 | 0.451808679 | 1.1206533 | up | mRNA |
| NM_001042728 | RARG | 0.550179425 | 0.925693985 | 1.0752436 | up | mRNA |
| NM_001043318 | C14orf43 | 0.159711637 | 0.631516339 | 1.195744 | up | mRNA |
| NM_001043353 | TPM3 | 0.79093144 | 0.978592956 | 1.0376381 | up | mRNA |
| NM_001044 | SLC6A3 | 0.272688935 | 0.772618648 | 1.1281559 | up | mRNA |
| NM_001044264 | MSMP | 0.636552332 | 0.951995735 | 1.0859879 | up | mRNA |
| NM_001044305 | SMAP1 | 0.600720615 | 0.940757262 | 1.0626521 | up | mRNA |
| NM_001044385 | TMEM237 | 0.455112512 | 0.891148383 | 1.1616228 | up | mRNA |
| NM_001044387 | ZNF557 | 0.549355126 | 0.925481755 | 1.0500932 | up | mRNA |
| NM_001044390 | MUC1 | 0.471752356 | 0.898908705 | 1.0792153 | up | mRNA |
| NM_001044392 | MUC1 | 0.36369892 | 0.843238468 | 1.1341035 | up | mRNA |
| NM_001044723 | CSNK1G3 | 0.082558322 | 0.490238261 | 1.3136838 | up | mRNA |
| NM_001047434 | DPH3 | 0.455983982 | 0.891365829 | 1.0376321 | up | mRNA |
| NM_001048166 | STIL | 0.331199946 | 0.821853462 | 4.0705043 | up | mRNA |
| NM_001048241 | UBL5 | 0.678416865 | 0.959825429 | 1.0218806 | up | mRNA |
| NM_001050 | SSTR2 | 0.046205805 | 0.392057225 | 2.7543331 | up | mRNA |
| NM_001054 | SULT1A2 | 0.496061843 | 0.908151395 | 1.0533399 | up | mRNA |
| NM_001058 | TACR1 | 0.793047752 | 0.979003983 | 1.0456274 | up | mRNA |
| NM_001059 | TACR3 | 0.674174507 | 0.959531882 | 1.1928378 | up | mRNA |
| NM_001061 | TBXAS1 | 0.434115805 | 0.880931467 | 1.5301871 | up | mRNA |
| NM_001066 | TNFRSF1B | 0.00398213 | 0.14487712 | 2.7310918 | up | mRNA |
| NM_001068 | TOP2B | 0.621196636 | 0.947926299 | 1.0523706 | up | mRNA |
| NM_001069 | TUBB2A | 0.001308274 | 0.093126586 | 1.6958936 | up | mRNA |
| NM_001076682 | NCAM1 | 0.915802682 | 0.995349793 | 1.0034087 | up | mRNA |
| NM_001076780 | PKD1L2 | 0.439941861 | 0.883272235 | 1.0387333 | up | mRNA |
| NM_001077181 | CDC14B | 0.084364007 | 0.494319593 | 1.26801 | up | mRNA |
| NM_001077182 | FSCN2 | 0.070762172 | 0.46010121 | 1.2345959 | up | mRNA |
| NM_001077241 | SLC25A45 | 0.04362145 | 0.385351177 | 1.1899946 | up | mRNA |
| NM_001077261 | NCOR2 | 0.414905168 | 0.870239791 | 1.047192 | up | mRNA |
| NM_001077263 | TMPRSS13 | 0.404283896 | 0.865617032 | 1.2895326 | up | mRNA |
| NM_001077268 | ZFYVE19 | 0.952046175 | 0.996710991 | 1.0039436 | up | mRNA |
| NM_001077395 | DPH5 | 0.491969295 | 0.905806259 | 1.0738839 | up | mRNA |
| NM_001077418 | TMEM231 | 0.697368703 | 0.962410025 | 1.083794 | up | mRNA |
| NM_001077443 | HNRNPC | 0.326831884 | 0.819869378 | 1.111777 | up | mRNA |
| NM_001077470 | NR1I3 | 0.003344933 | 0.136839396 | 1.0941145 | up | mRNA |
| NM_001077471 | NR1I3 | 0.912205293 | 0.995342392 | 1.0218061 | up | mRNA |
| NM_001077472 | NR1I3 | 0.126967155 | 0.580406758 | 1.099457 | up | mRNA |
| NM_001077474 | NR1I3 | 0.524144557 | 0.917428132 | 1.0438127 | up | mRNA |
| NM_001077475 | NR1I3 | 0.702677596 | 0.963478781 | 1.073449 | up | mRNA |
| NM_001077479 | NR1I3 | 0.477260052 | 0.900450218 | 1.0829856 | up | mRNA |
| NM_001077481 | NR1I3 | 0.318939203 | 0.812974553 | 1.1429895 | up | mRNA |
| NM_001077492 | KLK5 | 0.742854869 | 0.970330526 | 1.0710692 | up | mRNA |
| NM_001077494 | NFKB2 | 0.017740265 | 0.267776588 | 1.2984863 | up | mRNA |
| NM_001077498 | FAM222B | 0.614410774 | 0.944827645 | 1.0223511 | up | mRNA |
| NM_001077500 | KLK10 | 0.307561616 | 0.802348229 | 1.1539176 | up | mRNA |
| NM_001077511 | TCF19 | 0.929919199 | 0.996643872 | 1.0440986 | up | mRNA |
| NM_001077628 | APH1A | 0.549763055 | 0.925533435 | 1.1190698 | up | mRNA |
| NM_001077654 | TNFAIP8 | 0.000644898 | 0.076684828 | 8.447393 | up | mRNA |
| NM_001077663 | URGCP | 0.002795366 | 0.124912935 | 1.2001208 | up | mRNA |
| NM_001077664 | URGCP | 0.472066766 | 0.899081978 | 1.0639869 | up | mRNA |
| NM_001077704 | MIER1 | 0.645691039 | 0.955194282 | 1.1192486 | up | mRNA |
| NM_001078 | VCAM1 | 0.00194814 | 0.107343066 | 3.9272905 | up | mRNA |
| NM_001078170 | RGPD2 | 0.462987834 | 0.894989844 | 1.091164 | up | mRNA |
| NM_001078172 | FAM127B | 0.840576691 | 0.987068842 | 1.024326 | up | mRNA |
| NM_001079512 | FAM18A | 0.032737362 | 0.344901399 | 2.0285508 | up | mRNA |
| NM_001079519 | FAM177A1 | 0.022490287 | 0.295888941 | 1.2230412 | up | mRNA |
| NM_001079525 | PAICS | 0.978670574 | 0.998661138 | 1.0030169 | up | mRNA |
| NM_001079530 | CFC1B | 0.207493474 | 0.698556218 | 1.3614507 | up | mRNA |
| NM_001079533 | CPEB1 | 0.84019877 | 0.987068842 | 1.0407126 | up | mRNA |
| NM_001079536 | ART5 | 0.221128209 | 0.716640124 | 1.1093312 | up | mRNA |
| NM_001079670 | CAB39L | 0.259057269 | 0.759547173 | 1.1380068 | up | mRNA |
| NM_001079684 | KIAA1191 | 0.691950899 | 0.961451327 | 1.0370574 | up | mRNA |
| NM_001079691 | N4BP2L1 | 0.192416052 | 0.678822446 | 1.2659205 | up | mRNA |
| NM_001079804 | GAA | 0.983869397 | 0.998971807 | 1.0031092 | up | mRNA |
| NM_001079808 | PGA4 | 0.112551109 | 0.555164763 | 1.0813072 | up | mRNA |
| NM_001079811 | GLB1 | 0.745256542 | 0.970378122 | 1.1523492 | up | mRNA |
| NM_001079818 | ITGA6 | 0.955068627 | 0.996883425 | 1.0148434 | up | mRNA |
| NM_001079846 | CREBBP | 0.55367158 | 0.926337111 | 1.0603206 | up | mRNA |
| NM_001079858 | GPR64 | 0.864615019 | 0.989401142 | 1.0167269 | up | mRNA |
| NM_001079859 | GPR64 | 0.722652093 | 0.966191059 | 1.0375614 | up | mRNA |
| NM_001079860 | GPR64 | 0.761852702 | 0.97339948 | 1.038355 | up | mRNA |
| NM_001079862 | DBI | 0.003515489 | 0.13849526 | 1.4553351 | up | mRNA |
| NM_001079863 | DBI | 0.689868043 | 0.961105466 | 1.0439802 | up | mRNA |
| NM_001079864 | TAX1BP1 | 0.947975285 | 0.996710991 | 1.0125033 | up | mRNA |
| NM_001079872 | CUL4B | 0.409174231 | 0.867591329 | 1.0734541 | up | mRNA |
| NM_001079881 | PRKD2 | 0.164799554 | 0.638119216 | 1.1276605 | up | mRNA |
| NM_001079910 | LRRIQ1 | 0.917208189 | 0.995349793 | 1.0066824 | up | mRNA |
| NM_001079911 | DMP1 | 0.966401473 | 0.99793927 | 1.0039042 | up | mRNA |
| NM_001080122 | PRNP | 0.750160761 | 0.970725371 | 1.0300722 | up | mRNA |
| NM_001080125 | CASP8 | 0.920009127 | 0.995349793 | 1.0141129 | up | mRNA |
| NM_001080156 | ARHGAP9 | 0.64230589 | 0.954332328 | 1.0697157 | up | mRNA |
| NM_001080395 | AATK | 0.837901135 | 0.986897498 | 1.0197218 | up | mRNA |
| NM_001080396 | FAM155A | 0.949831198 | 0.996710991 | 1.0066231 | up | mRNA |
| NM_001080400 | PLIN4 | 0.589537117 | 0.936090876 | 1.055153 | up | mRNA |
| NM_001080407 | GLB1L3 | 0.176324828 | 0.65545452 | 1.4583183 | up | mRNA |
| NM_001080412 | ZBTB38 | 0.049315478 | 0.402199077 | 1.5103876 | up | mRNA |
| NM_001080424 | KDM6B | 0.29211868 | 0.788954556 | 1.1154259 | up | mRNA |
| NM_001080429 | CAMSAP3 | 0.563631484 | 0.929146073 | 1.0769735 | up | mRNA |
| NM_001080441 | TTC36 | 0.31515263 | 0.810240865 | 1.0947091 | up | mRNA |
| NM_001080448 | EPHA6 | 0.429704693 | 0.878226842 | 1.4466684 | up | mRNA |
| NM_001080453 | INTS1 | 0.244128914 | 0.742391426 | 1.0555043 | up | mRNA |
| NM_001080461 | UNCX | 0.989474923 | 0.999140676 | 1.0020348 | up | mRNA |
| NM_001080462 | TMEM202 | 0.404326429 | 0.865617032 | 1.096223 | up | mRNA |
| NM_001080470 | ZNF697 | 0.301589607 | 0.796663621 | 1.183625 | up | mRNA |
| NM_001080472 | FITM2 | 0.329139188 | 0.821768501 | 1.2039318 | up | mRNA |
| NM_001080473 | MFSD2B | 0.457009966 | 0.891568474 | 1.0656467 | up | mRNA |
| NM_001080489 | GLOD5 | 7.50576E-05 | 0.033623631 | 1.2614322 | up | mRNA |
| NM_001080496 | RGP1 | 0.283012325 | 0.780627498 | 1.0483866 | up | mRNA |
| NM_001080500 | VWC2L | 0.557846541 | 0.926833616 | 1.0802843 | up | mRNA |
| NM_001080503 | CCDC159 | 0.772413948 | 0.975448017 | 1.0175072 | up | mRNA |
| NM_001080506 | TMEM150C | 0.183165791 | 0.66623548 | 1.2382659 | up | mRNA |
| NM_001080510 | METTL23 | 0.954405795 | 0.996711624 | 1.0052449 | up | mRNA |
| NM_001080512 | BICC1 | 0.412002371 | 0.869001481 | 1.0641754 | up | mRNA |
| NM_001080515 | FAM163B | 0.125675563 | 0.578441772 | 1.5026619 | up | mRNA |
| NM_001080538 | AKR1B15 | 0.262579501 | 0.763125731 | 1.4877867 | up | mRNA |
| NM_001080546 | TMEM218 | 0.459592386 | 0.892922349 | 1.0598957 | up | mRNA |
| NM_001080554 | GSG1 | 0.80272193 | 0.98118927 | 1.0848554 | up | mRNA |
| NM_001080770 | KIR2DL4 | 0.362634584 | 0.843238468 | 1.4191394 | up | mRNA |
| NM_001080779 | MYO1C | 0.03613993 | 0.356565658 | 1.3798271 | up | mRNA |
| NM_001080791 | C15orf57 | 0.888772708 | 0.993440915 | 1.023808 | up | mRNA |
| NM_001080821 | ZNF799 | 0.924095102 | 0.995456159 | 1.0104943 | up | mRNA |
| NM_001080830 | PRAMEF12 | 0.407819467 | 0.867591329 | 1.0681069 | up | mRNA |
| NM_001080843 | GSTT2B | 0.807929084 | 0.98118927 | 1.0264166 | up | mRNA |
| NM_001080851 | DMRTC1B | 0.466000323 | 0.895615707 | 1.0398879 | up | mRNA |
| NM_001080956 | PLAGL1 | 0.308719113 | 0.803800359 | 1.0668002 | up | mRNA |
| NM_001080975 | REPS2 | 0.1996161 | 0.688831389 | 1.0628498 | up | mRNA |
| NM_001080976 | DSE | 0.609446304 | 0.943053241 | 1.0584773 | up | mRNA |
| NM_001081438 | LILRB4 | 0.152602434 | 0.618255775 | 1.1034946 | up | mRNA |
| NM_001081442 | LILRB5 | 0.726120179 | 0.966191059 | 1.0399619 | up | mRNA |
| NM_001081443 | LILRB5 | 0.868705261 | 0.989481868 | 1.0137065 | up | mRNA |
| NM_001081461 | JMJD6 | 0.519429573 | 0.916145597 | 1.0548991 | up | mRNA |
| NM_001081491 | NXF1 | 0.222610041 | 0.717433439 | 1.1300713 | up | mRNA |
| NM_001081560 | DMPK | 0.28138646 | 0.779126326 | 1.2211169 | up | mRNA |
| NM_001081637 | LILRB1 | 0.893739663 | 0.993978977 | 1.0124921 | up | mRNA |
| NM_001081677 | SCN3A | 0.934960196 | 0.996710991 | 1.0118904 | up | mRNA |
| NM_001081752 | ELN | 0.892302402 | 0.99393483 | 1.0743339 | up | mRNA |
| NM_001081753 | ELN | 0.158615543 | 0.629877192 | 1.1145461 | up | mRNA |
| NM_001081754 | ELN | 0.508432889 | 0.911674633 | 1.1673941 | up | mRNA |
| NM_001081955 | RGS9 | 0.20722322 | 0.698556218 | 1.0538962 | up | mRNA |
| NM_001082480 | ZNF623 | 0.770205719 | 0.975286259 | 1.0665694 | up | mRNA |
| NM_001082537 | TCTN1 | 0.958242619 | 0.996933183 | 1.008686 | up | mRNA |
| NM_001082538 | TCTN1 | 0.75766726 | 0.972072638 | 1.0641828 | up | mRNA |
| NM_001082619 | PIH1D2 | 0.584903533 | 0.934705544 | 1.1298323 | up | mRNA |
| NM_001082959 | SCARB1 | 0.706098055 | 0.96513517 | 1.0226594 | up | mRNA |
| NM_001083113 | ZGPAT | 0.130422892 | 0.584602788 | 1.0941716 | up | mRNA |
| NM_001083116 | PRF1 | 0.629529141 | 0.950410442 | 1.0271546 | up | mRNA |
| NM_001083335 | ZFP112 | 0.112722785 | 0.555472532 | 1.1416576 | up | mRNA |
| NM_001083536 | FGD3 | 0.686835328 | 0.960205549 | 1.0366401 | up | mRNA |
| NM_001083601 | NAA60 | 0.524276877 | 0.917428132 | 1.0994471 | up | mRNA |
| NM_001083615 | MYO3B | 0.842114415 | 0.987123192 | 1.0258045 | up | mRNA |
| NM_001083899 | GP6 | 0.030309968 | 0.337447626 | 1.2772091 | up | mRNA |
| NM_001083910 | SIRPB1 | 0.372058853 | 0.847209316 | 1.0602396 | up | mRNA |
| NM_001083913 | WBP1L | 0.441149132 | 0.883920737 | 1.0818936 | up | mRNA |
| NM_001083926 | ASRGL1 | 0.492940668 | 0.906130999 | 1.0534859 | up | mRNA |
| NM_001083956 | ZNF655 | 0.44337446 | 0.884425653 | 1.0548443 | up | mRNA |
| NM_001084393 | DDTL | 0.34126193 | 0.829571684 | 1.0449322 | up | mRNA |
| NM_001085 | SERPINA3 | 0.256344161 | 0.757240077 | 1.0718869 | up | mRNA |
| NM_001085367 | ZNF655 | 0.232897359 | 0.731089872 | 1.1613872 | up | mRNA |
| NM_001085372 | C11orf83 | 0.438871804 | 0.882895421 | 1.0931981 | up | mRNA |
| NM_001085375 | C1orf226 | 0.329577748 | 0.821768501 | 1.114401 | up | mRNA |
| NM_001085384 | ZNF154 | 0.598993488 | 0.940648177 | 1.160416 | up | mRNA |
| NM_001085398 | TLX1NB | 0.914093337 | 0.995342392 | 1.0229451 | up | mRNA |
| NM_001085427 | ARSA | 0.379009707 | 0.852573556 | 1.1322929 | up | mRNA |
| NM_001085447 | CCDC173 | 0.03264402 | 0.344901399 | 1.2787053 | up | mRNA |
| NM_001085452 | FAM75A1 | 0.028491897 | 0.327950578 | 1.2397023 | up | mRNA |
| NM_001085471 | FOXN3 | 0.818773618 | 0.982093332 | 1.0197639 | up | mRNA |
| NM_001088 | AANAT | 0.924233469 | 0.995456159 | 1.0108174 | up | mRNA |
| NM_001090 | ABCF1 | 0.961537637 | 0.99793927 | 1.0026786 | up | mRNA |
| NM_001092 | ABR | 0.108771677 | 0.546082252 | 1.2258018 | up | mRNA |
| NM_001097592 | XAGE1A | 0.973533936 | 0.998501532 | 1.0049926 | up | mRNA |
| NM_001097611 | KNCN | 0.655110088 | 0.957205183 | 1.0228563 | up | mRNA |
| NM_001097615 | POLR2J3 | 0.705514836 | 0.964798549 | 1.0257302 | up | mRNA |
| NM_001097620 | TMEM184A | 0.104918353 | 0.535979888 | 1.1575046 | up | mRNA |
| NM_001098270 | MGAT3 | 0.252311862 | 0.752621656 | 1.1181454 | up | mRNA |
| NM_001098398 | COPA | 0.71728158 | 0.966191059 | 1.0216113 | up | mRNA |
| NM_001098403 | ZNF295 | 0.00782066 | 0.193678037 | 1.571658 | up | mRNA |
| NM_001098406 | GAGE12J | 0.211420626 | 0.702730777 | 1.119891 | up | mRNA |
| NM_001098407 | GAGE2D | 0.063221295 | 0.440210745 | 1.1658125 | up | mRNA |
| NM_001098408 | GAGE12C | 0.610286381 | 0.943169861 | 1.1377175 | up | mRNA |
| NM_001098424 | DLG1 | 0.767769406 | 0.975033576 | 1.0386815 | up | mRNA |
| NM_001098475 | TDRD10 | 0.773497593 | 0.97589756 | 1.0386641 | up | mRNA |
| NM_001098479 | HLA-F | 0.744680661 | 0.970330526 | 1.0678757 | up | mRNA |
| NM_001098482 | CRTC1 | 0.117295482 | 0.564147103 | 1.2302601 | up | mRNA |
| NM_001098484 | SLC4A4 | 0.974636016 | 0.998501532 | 1.004732 | up | mRNA |
| NM_001098486 | SLC17A3 | 0.513154527 | 0.913452797 | 1.0982938 | up | mRNA |
| NM_001098497 | SGSM1 | 0.832141324 | 0.98620142 | 1.0353746 | up | mRNA |
| NM_001098503 | PTPRJ | 0.524980382 | 0.917632491 | 1.0431429 | up | mRNA |
| NM_001098504 | DDX17 | 0.712402583 | 0.966072854 | 1.0425987 | up | mRNA |
| NM_001098506 | CEACAM21 | 0.040220155 | 0.371915487 | 1.3310152 | up | mRNA |
| NM_001098507 | ZNF207 | 0.856040804 | 0.988315074 | 1.0100082 | up | mRNA |
| NM_001098511 | KIF2A | 0.813929865 | 0.98120545 | 1.0164517 | up | mRNA |
| NM_001098514 | C16orf89 | 0.399544169 | 0.864021702 | 1.0622156 | up | mRNA |
| NM_001098519 | LRRC43 | 0.499138496 | 0.909361611 | 1.1936471 | up | mRNA |
| NM_001098520 | HTATIP2 | 0.069583655 | 0.457632202 | 1.3564151 | up | mRNA |
| NM_001098524 | FAM129C | 0.375081983 | 0.849710156 | 1.2105483 | up | mRNA |
| NM_001098531 | RAPGEF3 | 0.346195685 | 0.833163669 | 1.0819502 | up | mRNA |
| NM_001098579 | MRVI1 | 0.061462991 | 0.437893235 | 1.1244896 | up | mRNA |
| NM_001098620 | PCBP2 | 0.859962622 | 0.988781331 | 1.0204306 | up | mRNA |
| NM_001098622 | NANOS3 | 0.707179038 | 0.965401045 | 1.075212 | up | mRNA |
| NM_001098632 | AKT1S1 | 0.063893147 | 0.441793638 | 1.1913059 | up | mRNA |
| NM_001098671 | RASGRP2 | 0.327489708 | 0.820319134 | 1.2797996 | up | mRNA |
| NM_001098783 | RPP14 | 0.92128464 | 0.995349793 | 1.0085773 | up | mRNA |
| NM_001098791 | MID1IP1 | 0.046109698 | 0.391970965 | 1.0804615 | up | mRNA |
| NM_001098817 | INO80C | 0.663598955 | 0.958354006 | 1.0299428 | up | mRNA |
| NM_001098821 | TMEM91 | 0.221958687 | 0.716912415 | 1.0506171 | up | mRNA |
| NM_001098834 | GBX1 | 0.568277632 | 0.931867151 | 1.1599538 | up | mRNA |
| NM_001098844 | TMEM236 | 0.605674983 | 0.94174007 | 1.1578769 | up | mRNA |
| NM_001098845 | ANXA8L1 | 0.44187694 | 0.88419144 | 1.1195181 | up | mRNA |
| NM_001099221 | TIFAB | 0.663429219 | 0.958354006 | 1.1069233 | up | mRNA |
| NM_001099270 | ZBTB34 | 0.388970832 | 0.85800274 | 1.4105612 | up | mRNA |
| NM_001099274 | TINF2 | 0.686664403 | 0.960205549 | 1.040395 | up | mRNA |
| NM_001099280 | HEATR7A | 0.862159866 | 0.989401142 | 1.0346885 | up | mRNA |
| NM_001099293 | KIF4B | 0.797472475 | 0.980721189 | 1.0147407 | up | mRNA |
| NM_001099338 | FAM22A | 0.987329818 | 0.999140676 | 1.0013051 | up | mRNA |
| NM_001099400 | SGCE | 0.311855916 | 0.806920827 | 1.1496614 | up | mRNA |
| NM_001099402 | CCNK | 0.446072763 | 0.886278379 | 1.0761554 | up | mRNA |
| NM_001099403 | PRDM8 | 0.398795704 | 0.863643939 | 1.4445265 | up | mRNA |
| NM_001099407 | DZANK1 | 0.337563118 | 0.827187565 | 1.0590324 | up | mRNA |
| NM_001099431 | CLEC1B | 0.375790365 | 0.849862269 | 1.2316478 | up | mRNA |
| NM_001099433 | JAKMIP1 | 0.055071415 | 0.419524921 | 1.1897763 | up | mRNA |
| NM_001099436 | ULK3 | 0.381013323 | 0.853514109 | 1.3810794 | up | mRNA |
| NM_001099439 | EPHA10 | 0.220163198 | 0.715236067 | 1.1622373 | up | mRNA |
| NM_001099456 | NPW | 0.24679925 | 0.745152522 | 1.0614298 | up | mRNA |
| NM_001099645 | RPL22L1 | 0.189311616 | 0.674719659 | 1.1870796 | up | mRNA |
| NM_001099667 | ARMS2 | 0.577650319 | 0.933787388 | 1.1511601 | up | mRNA |
| NM_001099687 | TP53TG3B | 0.121589951 | 0.573155624 | 1.1460545 | up | mRNA |
| NM_001099692 | EIF5AL1 | 0.826664424 | 0.984436279 | 1.0296839 | up | mRNA |
| NM_001099693 | RPL31 | 0.802518075 | 0.98118927 | 1.0138527 | up | mRNA |
| NM_001099746 | SYBU | 0.941524664 | 0.996710991 | 1.0089689 | up | mRNA |
| NM_001099772 | CYP4B1 | 0.876096897 | 0.991213905 | 1.0763536 | up | mRNA |
| NM_001099773 | CYP11A1 | 0.954320952 | 0.996710991 | 1.0131546 | up | mRNA |
| NM_001099777 | C3orf55 | 0.952206997 | 0.996710991 | 1.0370235 | up | mRNA |
| NM_001099855 | MCF2 | 0.038516685 | 0.366631229 | 1.4322194 | up | mRNA |
| NM_001099856 | IKBKG | 0.970888695 | 0.998387059 | 1.0040115 | up | mRNA |
| NM_001099858 | C7orf25 | 0.425744756 | 0.87545849 | 1.1123232 | up | mRNA |
| NM_001099922 | ALG13 | 0.91316562 | 0.995342392 | 1.0052408 | up | mRNA |
| NM_001099952 | ITPR1 | 0.395108058 | 0.861590143 | 1.0636742 | up | mRNA |
| NM_001100111 | LOC286238 | 0.831814065 | 0.986141573 | 1.0533533 | up | mRNA |
| NM_001100120 | ECE2 | 0.534956508 | 0.921054766 | 1.1012917 | up | mRNA |
| NM_001100164 | PHACTR2 | 0.233037253 | 0.731100552 | 1.188918 | up | mRNA |
| NM_001100165 | PHACTR2 | 0.418166535 | 0.871927185 | 1.0711984 | up | mRNA |
| NM_001100166 | PHACTR2 | 0.424285648 | 0.874817544 | 1.0860714 | up | mRNA |
| NM_001100169 | IMMT | 0.082066749 | 0.489148905 | 1.0816332 | up | mRNA |
| NM_001100390 | IQCE | 0.942017007 | 0.996710991 | 1.0118775 | up | mRNA |
| NM_001100400 | PDS5A | 0.102391917 | 0.53121522 | 1.0736502 | up | mRNA |
| NM_001100421 | C21orf91 | 0.68581916 | 0.960111041 | 1.0783014 | up | mRNA |
| NM_001100425 | KIAA0895 | 0.850162849 | 0.987678166 | 1.0216048 | up | mRNA |
| NM_001100595 | SKA2 | 0.70965341 | 0.965462762 | 1.0392435 | up | mRNA |
| NM_001100619 | CABLES1 | 0.7789059 | 0.976938981 | 1.0669102 | up | mRNA |
| NM_001100620 | TROAP | 0.74518325 | 0.970378122 | 1.0319631 | up | mRNA |
| NM_001100625 | CENPN | 0.401636519 | 0.864768873 | 1.218469 | up | mRNA |
| NM_001100814 | TMEM55B | 0.620012954 | 0.947444797 | 1.1134476 | up | mRNA |
| NM_001100818 | PID1 | 0.074783121 | 0.469867247 | 1.1960848 | up | mRNA |
| NM_001100876 | PHYHD1 | 0.95595321 | 0.996883425 | 1.0038669 | up | mRNA |
| NM_001100878 | C8orf73 | 0.553000167 | 0.926337111 | 1.1823294 | up | mRNA |
| NM_001100879 | ST20 | 0.137825272 | 0.596789644 | 1.3085718 | up | mRNA |
| NM_001100916 | MBOAT4 | 0.457303651 | 0.891884683 | 1.1048538 | up | mRNA |
| NM_001101 | ACTB | 0.641425267 | 0.954299614 | 1.0516263 | up | mRNA |
| NM_001101330 | LOC728819 | 0.811962022 | 0.98118927 | 1.0542505 | up | mRNA |
| NM_001101340 | C19orf76 | 0.381767715 | 0.853514109 | 1.8943714 | up | mRNA |
| NM_001101387 | PIRT | 0.060530654 | 0.435971562 | 1.1155983 | up | mRNA |
| NM_001101401 | SBK2 | 0.028149352 | 0.326447555 | 1.2070337 | up | mRNA |
| NM_001101404 | SH2D7 | 0.675664418 | 0.959531882 | 1.0384028 | up | mRNA |
| NM_001101426 | ISPD | 0.660352801 | 0.958354006 | 1.0587461 | up | mRNA |
| NM_001101653 | COMMD9 | 0.899204729 | 0.994575817 | 1.0068503 | up | mRNA |
| NM_001101663 | NBPF24 | 0.377641897 | 0.851054649 | 1.1608335 | up | mRNA |
| NM_001101669 | INPP4B | 0.735824724 | 0.968398491 | 1.1182251 | up | mRNA |
| NM_001101801 | FAM13B | 0.952076131 | 0.996710991 | 1.0170098 | up | mRNA |
| NM_001102 | ACTN1 | 0.9668927 | 0.99793927 | 1.0112563 | up | mRNA |
| NM_001102367 | C14orf159 | 0.765732612 | 0.974638504 | 1.0809249 | up | mRNA |
| NM_001102368 | C14orf159 | 0.610703292 | 0.943527304 | 1.0376918 | up | mRNA |
| NM_001102406 | ALPK1 | 0.279202438 | 0.777816764 | 1.1552655 | up | mRNA |
| NM_001102416 | KNG1 | 0.475991282 | 0.900275303 | 1.0805952 | up | mRNA |
| NM_001102420 | ZFAND5 | 0.013500972 | 0.235623837 | 1.3477524 | up | mRNA |
| NM_001102467 | AQP12B | 0.084775921 | 0.495305754 | 1.1419235 | up | mRNA |
| NM_001102560 | PPAPDC1B | 0.683964363 | 0.960111041 | 1.0624424 | up | mRNA |
| NM_001102562 | MARCH11 | 0.884053449 | 0.992766819 | 1.0129819 | up | mRNA |
| NM_001102596 | DTX2 | 0.539271179 | 0.923577434 | 1.0702024 | up | mRNA |
| NM_001102597 | CEACAM20 | 0.758987897 | 0.972369912 | 1.1199861 | up | mRNA |
| NM_001102598 | CEACAM20 | 0.964528578 | 0.99793927 | 1.0216501 | up | mRNA |
| NM_001102608 | COL6A6 | 0.971624445 | 0.998501532 | 1.0086413 | up | mRNA |
| NM_001102612 | PGPEP1L | 0.383300183 | 0.853658125 | 1.1702147 | up | mRNA |
| NM_001102653 | OTUD4 | 0.144438291 | 0.605627057 | 1.1770035 | up | mRNA |
| NM_001102654 | NTF3 | 0.013209089 | 0.234704726 | 1.4403394 | up | mRNA |
| NM_001102663 | NBPF16 | 0.241303209 | 0.73945497 | 1.2138681 | up | mRNA |
| NM_001102667 | PSMA4 | 0.025053096 | 0.309277202 | 1.1488114 | up | mRNA |
| NM_001103149 | PNMAL1 | 0.275462728 | 0.775184524 | 1.2673493 | up | mRNA |
| NM_001103170 | AADACL3 | 0.206284664 | 0.69755672 | 1.2467027 | up | mRNA |
| NM_001104 | ACTN3 | 0.415131544 | 0.870239791 | 1.0766101 | up | mRNA |
| NM_001104545 | FAM70A | 0.443250992 | 0.884425653 | 1.0908631 | up | mRNA |
| NM_001104558 | ELOVL7 | 0.014103359 | 0.239157213 | 4.0754734 | up | mRNA |
| NM_001105192 | TLE3 | 0.882903465 | 0.992766819 | 1.0820549 | up | mRNA |
| NM_001105209 | LAMA4 | 0.88786287 | 0.993391292 | 1.0200573 | up | mRNA |
| NM_001105248 | TMC5 | 0.479462599 | 0.901538317 | 1.3193894 | up | mRNA |
| NM_001105518 | SWT1 | 0.201400062 | 0.689610625 | 1.9811182 | up | mRNA |
| NM_001105519 | C2orf70 | 0.375936673 | 0.849862269 | 1.0734563 | up | mRNA |
| NM_001105533 | CPED1 | 0.273923561 | 0.774088254 | 1.2038575 | up | mRNA |
| NM_001105537 | ZNF142 | 0.022312636 | 0.294616576 | 1.2147704 | up | mRNA |
| NM_001105543 | PLXNA4 | 0.355416679 | 0.838369858 | 1.1245899 | up | mRNA |
| NM_001105556 | THEMIS2 | 0.518260403 | 0.916125349 | 1.1253041 | up | mRNA |
| NM_001105581 | LRRC30 | 0.864019332 | 0.989401142 | 1.0430283 | up | mRNA |
| NM_001105663 | NUDT7 | 0.820158625 | 0.982556462 | 1.0457908 | up | mRNA |
| NM_001107 | ACYP1 | 0.775597155 | 0.97589756 | 1.0385113 | up | mRNA |
| NM_001109689 | ZNF250 | 0.847441536 | 0.987678166 | 1.0246058 | up | mRNA |
| NM_001109974 | SYNPO | 0.837549334 | 0.986775289 | 1.0243948 | up | mRNA |
| NM_001110354 | ZP3 | 0.901044833 | 0.994789506 | 1.0294212 | up | mRNA |
| NM_001110503 | TMEM87A | 0.247644467 | 0.745868458 | 1.0699865 | up | mRNA |
| NM_001110533 | C1orf177 | 0.765795783 | 0.974657894 | 1.0230134 | up | mRNA |
| NM_001110556 | FLNA | 0.176146041 | 0.6551228 | 1.185904 | up | mRNA |
| NM_001110792 | MECP2 | 0.081041929 | 0.486347254 | 1.1456777 | up | mRNA |
| NM_001111 | ADAR | 0.584636639 | 0.93461341 | 1.0342531 | up | mRNA |
| NM_001111031 | ACVR1C | 0.663402417 | 0.958354006 | 1.0927677 | up | mRNA |
| NM_001111032 | ACVR1C | 0.01795202 | 0.269173581 | 1.2409082 | up | mRNA |
| NM_001111097 | LYN | 0.310547439 | 0.805412658 | 1.4460712 | up | mRNA |
| NM_001111101 | CNRIP1 | 0.541638876 | 0.923577434 | 1.1225601 | up | mRNA |
| NM_001111270 | ARHGEF25 | 0.377602381 | 0.851054649 | 1.1241985 | up | mRNA |
| NM_001111284 | IGF1 | 0.680824834 | 0.960111041 | 1.0227246 | up | mRNA |
| NM_001111307 | PDE4A | 0.237899115 | 0.735771951 | 1.1191918 | up | mRNA |
| NM_001111308 | PDE4A | 0.707149246 | 0.965401045 | 1.0631794 | up | mRNA |
| NM_001111309 | PDE4A | 0.366704417 | 0.845181673 | 1.0745883 | up | mRNA |
| NM_001112707 | TLK2 | 0.918699755 | 0.995349793 | 1.0171983 | up | mRNA |
| NM_001112741 | KCNC1 | 0.406512553 | 0.86737467 | 1.1692797 | up | mRNA |
| NM_001112808 | FPGT-TNNI3K | 0.450177727 | 0.888463871 | 1.0725207 | up | mRNA |
| NM_001113178 | NFAT5 | 0.103520001 | 0.533123263 | 1.1226231 | up | mRNA |
| NM_001113205 | TSTD1 | 0.198643915 | 0.687087939 | 1.1187345 | up | mRNA |
| NM_001113207 | TSTD1 | 0.969546171 | 0.998245244 | 1.0046678 | up | mRNA |
| NM_001113239 | HIPK2 | 0.006987502 | 0.186688411 | 1.3294405 | up | mRNA |
| NM_001113324 | TEN1 | 0.509015013 | 0.911674633 | 1.1185282 | up | mRNA |
| NM_001113347 | ECE1 | 0.040734695 | 0.374589699 | 1.3687567 | up | mRNA |
| NM_001113348 | ECE1 | 0.607222371 | 0.942627675 | 1.0695772 | up | mRNA |
| NM_001113349 | ECE1 | 0.019059511 | 0.27719455 | 1.4943152 | up | mRNA |
| NM_001113363 | TBC1D14 | 0.615036582 | 0.94515077 | 1.1022481 | up | mRNA |
| NM_001113397 | ZNF385B | 0.226231007 | 0.721639289 | 1.2467149 | up | mRNA |
| NM_001113475 | NOXRED1 | 0.117776709 | 0.564437853 | 1.112334 | up | mRNA |
| NM_001113492 | SEPT9 | 0.413633888 | 0.86961884 | 1.0525069 | up | mRNA |
| NM_001113493 | SEPT9 | 0.934974636 | 0.996710991 | 1.014428 | up | mRNA |
| NM_001113495 | SEPT9 | 0.376949208 | 0.850530607 | 1.0921294 | up | mRNA |
| NM_001113496 | SEPT9 | 0.718125037 | 0.966191059 | 1.0488141 | up | mRNA |
| NM_001113525 | ZNF276 | 0.531718522 | 0.920228311 | 1.0887022 | up | mRNA |
| NM_001113546 | LIMA1 | 0.513212483 | 0.913475987 | 1.1285744 | up | mRNA |
| NM_001113755 | TYMP | 0.184572416 | 0.668017166 | 1.1638381 | up | mRNA |
| NM_001114091 | CDC27 | 0.077296428 | 0.477977791 | 1.0892985 | up | mRNA |
| NM_001114100 | SEZ6L2 | 0.806296233 | 0.98118927 | 1.0503135 | up | mRNA |
| NM_001114107 | PDLIM3 | 0.98199782 | 0.998971807 | 1.0042988 | up | mRNA |
| NM_001114120 | DEPDC1 | 0.240431724 | 0.738437735 | 1.2069867 | up | mRNA |
| NM_001114171 | FOSB | 0.668806551 | 0.959036237 | 1.1160865 | up | mRNA |
| NM_001114173 | CTSC | 0.112935128 | 0.55597991 | 1.074869 | up | mRNA |
| NM_001114331 | CLCN7 | 0.052718869 | 0.413038171 | 1.2257428 | up | mRNA |
| NM_001114598 | ASPDH | 0.956301146 | 0.996883425 | 1.018213 | up | mRNA |
| NM_001114614 | MFGE8 | 0.653639989 | 0.956927437 | 1.1102571 | up | mRNA |
| NM_001114635 | PLAG1 | 0.734536471 | 0.967777568 | 1.0928833 | up | mRNA |
| NM_001114726 | PRRT4 | 0.456100372 | 0.891365829 | 1.1445033 | up | mRNA |
| NM_001114734 | PABPC4L | 0.882198729 | 0.992766819 | 1.2152078 | up | mRNA |
| NM_001114735 | BCL2A1 | 3.88066E-05 | 0.026300499 | 7.7905634 | up | mRNA |
| NM_001114752 | CD55 | 0.200461 | 0.68900633 | 1.0931186 | up | mRNA |
| NM_001114937 | SH2D1A | 0.312789754 | 0.808086566 | 1.18846 | up | mRNA |
| NM_001114974 | SMTNL2 | 0.172830879 | 0.650632622 | 1.2048268 | up | mRNA |
| NM_001114978 | TP63 | 0.403239777 | 0.864786348 | 1.1774583 | up | mRNA |
| NM_001115 | ADCY8 | 0.76833404 | 0.975142015 | 1.0897837 | up | mRNA |
| NM_001115016 | KANSL3 | 0.485829441 | 0.903250293 | 1.0599546 | up | mRNA |
| NM_001117 | ADCYAP1 | 0.896768613 | 0.994222243 | 1.0160612 | up | mRNA |
| NM_001119 | ADD1 | 0.054434032 | 0.418738677 | 1.2197614 | up | mRNA |
| NM_001122 | PLIN2 | 0.808383215 | 0.98118927 | 1.030959 | up | mRNA |
| NM_001122606 | LAMP2 | 0.960443738 | 0.997738713 | 1.0104391 | up | mRNA |
| NM_001122607 | RUNX1 | 0.007580199 | 0.193257016 | 1.2077178 | up | mRNA |
| NM_001122674 | ABCD3 | 0.663098187 | 0.958354006 | 1.1436928 | up | mRNA |
| NM_001122731 | MICAL3 | 0.055291846 | 0.419636174 | 1.3653469 | up | mRNA |
| NM_001122742 | ESR1 | 0.755667538 | 0.971497464 | 1.045197 | up | mRNA |
| NM_001122752 | SERPINI1 | 0.087749907 | 0.501332443 | 1.2379615 | up | mRNA |
| NM_001122821 | SET | 0.27179629 | 0.771810358 | 1.1080264 | up | mRNA |
| NM_001122824 | TACC1 | 0.134111502 | 0.590871682 | 1.1258631 | up | mRNA |
| NM_001122837 | SLC50A1 | 0.733193059 | 0.967777568 | 1.0334329 | up | mRNA |
| NM_001122839 | SLC50A1 | 0.703593734 | 0.963625105 | 1.065304 | up | mRNA |
| NM_001122848 | SLC6A12 | 0.997704654 | 0.999889342 | 1.0006126 | up | mRNA |
| NM_001123041 | CCR2 | 0.677962858 | 0.959531882 | 1.1489919 | up | mRNA |
| NM_001123066 | MAPT | 0.179540069 | 0.661905473 | 1.1183473 | up | mRNA |
| NM_001123225 | SYCE3 | 0.588240819 | 0.93579682 | 1.1167046 | up | mRNA |
| NM_001123228 | TMEM14E | 0.453012994 | 0.889563297 | 1.0820058 | up | mRNA |
| NM_001123355 | PPP6C | 0.84203329 | 0.987123192 | 1.0306843 | up | mRNA |
| NM_001123363 | RGPD6 | 0.893977235 | 0.993978977 | 1.0261034 | up | mRNA |
| NM_001123385 | BCOR | 0.000948759 | 0.084526081 | 1.6473583 | up | mRNA |
| NM_001123392 | TBC1D3H | 0.714565727 | 0.966110889 | 1.0350179 | up | mRNA |
| NM_001123395 | CLDN19 | 0.166055023 | 0.640243452 | 1.285822 | up | mRNA |
| NM_001124 | ADM | 0.013630859 | 0.235988767 | 2.9623249 | up | mRNA |
| NM_001124758 | SPNS2 | 0.514184524 | 0.913526716 | 1.0831037 | up | mRNA |
| NM_001126049 | KLLN | 0.753294383 | 0.970725371 | 1.0436522 | up | mRNA |
| NM_001126050 | HDGF | 0.033032219 | 0.346127643 | 1.1765334 | up | mRNA |
| NM_001126057 | DMKN | 0.648135141 | 0.95550188 | 1.1041588 | up | mRNA |
| NM_001126058 | DMKN | 0.966282969 | 0.99793927 | 1.014756 | up | mRNA |
| NM_001126112 | TP53 | 0.284290979 | 0.78179061 | 1.0711396 | up | mRNA |
| NM_001126113 | TP53 | 0.037109036 | 0.359628653 | 1.2138074 | up | mRNA |
| NM_001126114 | TP53 | 0.565848874 | 0.930458047 | 1.0396069 | up | mRNA |
| NM_001126123 | ENOSF1 | 0.702506239 | 0.963399221 | 1.0878263 | up | mRNA |
| NM_001126130 | GINS3 | 0.277892187 | 0.777287651 | 1.1892161 | up | mRNA |
| NM_001126131 | POLG | 0.730187587 | 0.966191059 | 1.1350687 | up | mRNA |
| NM_001126133 | TNNT1 | 0.808289991 | 0.98118927 | 1.0695331 | up | mRNA |
| NM_001126242 | TP73 | 0.005592177 | 0.169833068 | 1.3189313 | up | mRNA |
| NM_001126328 | LNX1 | 0.373214874 | 0.848112577 | 1.075698 | up | mRNA |
| NM_001126337 | TUFT1 | 0.365818766 | 0.845164582 | 1.0521366 | up | mRNA |
| NM_001127173 | CADM3 | 0.992503827 | 0.999781402 | 1.0034844 | up | mRNA |
| NM_001127184 | CFLAR | 1.33426E-05 | 0.016955107 | 4.23717 | up | mRNA |
| NM_001127197 | ELF4 | 0.019398087 | 0.280114993 | 1.5717145 | up | mRNA |
| NM_001127203 | PCID2 | 0.042955742 | 0.382891781 | 1.160068 | up | mRNA |
| NM_001127208 | TET2 | 0.365363086 | 0.844953778 | 2.1306306 | up | mRNA |
| NM_001127219 | ACCS | 0.907928599 | 0.995148479 | 1.0123789 | up | mRNA |
| NM_001127222 | CACNA1A | 0.756695102 | 0.971824502 | 1.0367663 | up | mRNA |
| NM_001127227 | CD59 | 0.335750322 | 0.825621677 | 1.1528799 | up | mRNA |
| NM_001127231 | AUTS2 | 0.908485231 | 0.995329331 | 1.0258579 | up | mRNA |
| NM_001127232 | AUTS2 | 0.043774898 | 0.385351177 | 1.2564351 | up | mRNA |
| NM_001127241 | BBC3 | 0.491579242 | 0.905806259 | 1.0694642 | up | mRNA |
| NM_001127242 | BBC3 | 0.836354601 | 0.986575022 | 1.060149 | up | mRNA |
| NM_001127258 | HHIPL1 | 0.571746928 | 0.933299883 | 1.0421632 | up | mRNA |
| NM_001127320 | UBAP2L | 0.728861604 | 0.966191059 | 1.0251608 | up | mRNA |
| NM_001127322 | CBX5 | 0.746642205 | 0.970432947 | 1.0487932 | up | mRNA |
| NM_001127361 | RNF19B | 0.00903489 | 0.202329498 | 2.3928799 | up | mRNA |
| NM_001127385 | CTXN3 | 0.007304381 | 0.190890337 | 1.3851806 | up | mRNA |
| NM_001127392 | C11orf9 | 0.895813042 | 0.993978977 | 1.0106651 | up | mRNA |
| NM_001127393 | C14orf2 | 0.537501793 | 0.922702335 | 1.056374 | up | mRNA |
| NM_001127453 | DFNA5 | 0.66452732 | 0.958354006 | 1.1461184 | up | mRNA |
| NM_001127457 | CRY2 | 0.278676166 | 0.777783404 | 1.2267794 | up | mRNA |
| NM_001127493 | ANK2 | 0.024308361 | 0.304521009 | 1.1576535 | up | mRNA |
| NM_001127496 | SPRY4 | 0.892878478 | 0.993978977 | 1.0556325 | up | mRNA |
| NM_001127641 | LAMB3 | 0.905851473 | 0.99508468 | 1.020034 | up | mRNA |
| NM_001127660 | MFN2 | 0.418526832 | 0.87232061 | 1.1209058 | up | mRNA |
| NM_001127691 | RHD | 0.49802367 | 0.909116151 | 1.0253385 | up | mRNA |
| NM_001127692 | PCCA | 0.245364086 | 0.743589595 | 1.143615 | up | mRNA |
| NM_001127708 | PRG4 | 0.664536812 | 0.958354006 | 1.4144396 | up | mRNA |
| NM_001127711 | TMEM14B | 0.890410712 | 0.9937521 | 1.0147251 | up | mRNA |
| NM_001127716 | ETFA | 0.58166445 | 0.934005566 | 1.0188024 | up | mRNA |
| NM_001127891 | MMP2 | 0.814982502 | 0.981277402 | 1.049809 | up | mRNA |
| NM_001128127 | GK | 0.069707207 | 0.457632202 | 1.1870637 | up | mRNA |
| NM_001128128 | ZEB1 | 0.202369064 | 0.691421917 | 1.3625088 | up | mRNA |
| NM_001128141 | DPEP1 | 0.930325611 | 0.996700407 | 1.0163412 | up | mRNA |
| NM_001128160 | UBP1 | 0.005677956 | 0.170618233 | 1.3191458 | up | mRNA |
| NM_001128167 | PAK3 | 0.818488699 | 0.981973932 | 1.0460597 | up | mRNA |
| NM_001128172 | PAK3 | 0.634810441 | 0.951279916 | 1.0336536 | up | mRNA |
| NM_001128178 | NPHP1 | 0.448918174 | 0.888279993 | 1.1278129 | up | mRNA |
| NM_001128205 | SULF1 | 0.607554801 | 0.942627675 | 1.129422 | up | mRNA |
| NM_001128209 | SGCD | 0.954334561 | 0.996710991 | 1.0170888 | up | mRNA |
| NM_001128210 | SPRED2 | 0.400184064 | 0.864237287 | 1.0869184 | up | mRNA |
| NM_001128211 | NUDCD1 | 0.092604532 | 0.511338764 | 1.2149449 | up | mRNA |
| NM_001128215 | LIPM | 0.497246608 | 0.908789022 | 1.2484784 | up | mRNA |
| NM_001128228 | TPRN | 0.398787021 | 0.863643939 | 1.0695041 | up | mRNA |
| NM_001128302 | LYRM1 | 0.08026434 | 0.483876854 | 1.4435826 | up | mRNA |
| NM_001128309 | TSPAN14 | 0.574865019 | 0.933656777 | 1.1076798 | up | mRNA |
| NM_001128423 | MPV17L | 0.545236371 | 0.924012779 | 1.06289 | up | mRNA |
| NM_001128590 | CYP21A2 | 0.201784372 | 0.690328163 | 1.1704942 | up | mRNA |
| NM_001128591 | PSMG4 | 0.351523421 | 0.835247013 | 1.0795664 | up | mRNA |
| NM_001128592 | PSMG4 | 0.936653872 | 0.996710991 | 1.0544484 | up | mRNA |
| NM_001128608 | MAPKBP1 | 0.364542963 | 0.843907627 | 1.3349285 | up | mRNA |
| NM_001128613 | NUDT22 | 0.155055443 | 0.62254883 | 1.0298859 | up | mRNA |
| NM_001128619 | LUZP6 | 0.24569158 | 0.743916784 | 1.0828797 | up | mRNA |
| NM_001128631 | DCAKD | 0.009360296 | 0.204533521 | 1.2321948 | up | mRNA |
| NM_001128834 | PLP1 | 0.760105218 | 0.972883253 | 1.0998851 | up | mRNA |
| NM_001128840 | CACNA1D | 0.297137946 | 0.793643704 | 1.2647626 | up | mRNA |
| NM_001128848 | SMARCA4 | 0.247054037 | 0.745152522 | 1.0524655 | up | mRNA |
| NM_001128852 | SRRT | 0.370669075 | 0.846330273 | 1.1593895 | up | mRNA |
| NM_001128853 | SRRT | 0.370323829 | 0.846215286 | 1.1708824 | up | mRNA |
| NM_001128917 | TOMM40 | 0.953882951 | 0.996710991 | 1.0121024 | up | mRNA |
| NM_001128918 | MARK3 | 0.673855703 | 0.959531882 | 1.0356444 | up | mRNA |
| NM_001128919 | MARK3 | 0.424948998 | 0.874854499 | 1.0637442 | up | mRNA |
| NM_001128921 | MARK3 | 0.542455127 | 0.923577434 | 1.0235848 | up | mRNA |
| NM_001128923 | SH3D19 | 0.046567376 | 0.392866345 | 1.1123311 | up | mRNA |
| NM_001128929 | ROBO2 | 0.61787248 | 0.946754755 | 1.0773629 | up | mRNA |
| NM_001128931 | NAF1 | 0.390848896 | 0.858920947 | 1.1173768 | up | mRNA |
| NM_001128934 | SYNPO2 | 0.746361781 | 0.970394407 | 1.569871 | up | mRNA |
| NM_001129729 | PLEKHG4 | 0.546188959 | 0.92457863 | 1.0655731 | up | mRNA |
| NM_001129731 | PLEKHG4 | 0.439185113 | 0.882895421 | 1.1131778 | up | mRNA |
| NM_001129778 | GRAPL | 0.52990831 | 0.91899174 | 1.1264047 | up | mRNA |
| NM_001129828 | CSAG3 | 0.385904932 | 0.855546731 | 1.1815636 | up | mRNA |
| NM_001129829 | CACNA1C | 0.665605409 | 0.958354006 | 1.0526513 | up | mRNA |
| NM_001129835 | CACNA1C | 0.642034853 | 0.954332328 | 1.0857494 | up | mRNA |
| NM_001129842 | CACNA1C | 0.82423188 | 0.983582732 | 1.0060268 | up | mRNA |
| NM_001129884 | TEX264 | 0.004194201 | 0.147792896 | 1.5521988 | up | mRNA |
| NM_001129895 | HGC6.3 | 0.869573608 | 0.989592579 | 1.0337648 | up | mRNA |
| NM_001129899 | ZNF673 | 0.965102366 | 0.99793927 | 1.0100624 | up | mRNA |
| NM_001130001 | C3orf55 | 0.866532887 | 0.989481868 | 1.0609088 | up | mRNA |
| NM_001130002 | C3orf55 | 0.442838546 | 0.884304813 | 1.3314622 | up | mRNA |
| NM_001130004 | ACTN1 | 0.731538759 | 0.967176866 | 1.0722386 | up | mRNA |
| NM_001130006 | GSPT1 | 0.568673517 | 0.932312606 | 1.0625315 | up | mRNA |
| NM_001130010 | C15orf41 | 0.824171397 | 0.983568283 | 1.0206788 | up | mRNA |
| NM_001130021 | ATP6V0A1 | 0.084540738 | 0.494805206 | 1.2536356 | up | mRNA |
| NM_001130025 | FAM115C | 0.834099985 | 0.986475536 | 1.0564725 | up | mRNA |
| NM_001130036 | PLEKHB1 | 0.285225897 | 0.781963593 | 1.1251505 | up | mRNA |
| NM_001130046 | CCL20 | 0.021452873 | 0.288950479 | 2.0922956 | up | mRNA |
| NM_001130050 | DOCK9 | 0.004243345 | 0.148495162 | 1.4713091 | up | mRNA |
| NM_001130056 | EEF1D | 0.870830417 | 0.989640119 | 1.0066227 | up | mRNA |
| NM_001130059 | ATF7 | 0.608666436 | 0.943053241 | 1.0384967 | up | mRNA |
| NM_001130069 | SUMF2 | 0.910786338 | 0.995342392 | 1.0174964 | up | mRNA |
| NM_001130071 | EPN1 | 0.678709109 | 0.959891867 | 1.0480351 | up | mRNA |
| NM_001130072 | EPN1 | 0.71265547 | 0.966110889 | 1.0222359 | up | mRNA |
| NM_001130084 | ABLIM2 | 0.920162059 | 0.995349793 | 1.0162133 | up | mRNA |
| NM_001130085 | ABLIM2 | 0.719181936 | 0.966191059 | 1.0369663 | up | mRNA |
| NM_001130089 | KARS | 0.594879061 | 0.938655001 | 1.0791856 | up | mRNA |
| NM_001130103 | COL13A1 | 0.092148128 | 0.511207518 | 1.1335387 | up | mRNA |
| NM_001130111 | FAM108A1 | 0.692215608 | 0.961544561 | 1.0430418 | up | mRNA |
| NM_001130142 | VWA5A | 0.094159796 | 0.514224271 | 1.2058401 | up | mRNA |
| NM_001130172 | MYB | 0.001314545 | 0.093126586 | 2.4752923 | up | mRNA |
| NM_001130182 | DNAJA4 | 0.95244093 | 0.996710991 | 1.0134315 | up | mRNA |
| NM_001130183 | DNAJA4 | 0.194464271 | 0.682224934 | 1.0990463 | up | mRNA |
| NM_001130413 | SCNN1D | 0.901277756 | 0.994830583 | 1.0179614 | up | mRNA |
| NM_001130447 | HAUS2 | 0.513944343 | 0.91350662 | 1.205878 | up | mRNA |
| NM_001130448 | C15orf62 | 0.395022025 | 0.861590143 | 1.2108711 | up | mRNA |
| NM_001130475 | THAP5 | 0.754945726 | 0.971417904 | 1.0416657 | up | mRNA |
| NM_001130487 | NDRG4 | 0.138208678 | 0.597376455 | 1.104572 | up | mRNA |
| NM_001130520 | ZNF195 | 0.617687058 | 0.946691789 | 1.0823618 | up | mRNA |
| NM_001130677 | C17orf96 | 0.112358854 | 0.554686607 | 1.5920131 | up | mRNA |
| NM_001130682 | GUCY1A3 | 0.71731418 | 0.966191059 | 1.052639 | up | mRNA |
| NM_001130690 | PDE10A | 0.671190116 | 0.959531882 | 1.0297744 | up | mRNA |
| NM_001130710 | LSM5 | 0.897320987 | 0.994222243 | 1.017507 | up | mRNA |
| NM_001130716 | PLAC8 | 0.762028845 | 0.97339948 | 1.0365357 | up | mRNA |
| NM_001130720 | AHCYL2 | 0.669896393 | 0.959531882 | 1.0422383 | up | mRNA |
| NM_001130723 | AHCYL2 | 0.85191459 | 0.987678166 | 1.0189318 | up | mRNA |
| NM_001130725 | PSMB5 | 0.8523308 | 0.987678166 | 1.0095257 | up | mRNA |
| NM_001130726 | CCDC149 | 0.938516721 | 0.996710991 | 1.0116761 | up | mRNA |
| NM_001130820 | RABL5 | 0.762159485 | 0.97339948 | 1.0311814 | up | mRNA |
| NM_001130823 | DNMT1 | 0.891785364 | 0.9937521 | 1.0314237 | up | mRNA |
| NM_001130861 | CLDN5 | 0.757114987 | 0.971960783 | 1.0734624 | up | mRNA |
| NM_001130865 | DMRT2 | 0.414314819 | 0.869769512 | 1.1445392 | up | mRNA |
| NM_001130913 | ZNF720 | 0.760228015 | 0.972883253 | 1.067484 | up | mRNA |
| NM_001130914 | BTG3 | 8.56074E-05 | 0.034811383 | 1.5457021 | up | mRNA |
| NM_001130916 | TGFBR1 | 0.687720005 | 0.960243201 | 1.094133 | up | mRNA |
| NM_001130918 | TTLL6 | 0.730014439 | 0.966191059 | 1.0791828 | up | mRNA |
| NM_001130921 | RABL2B | 0.803329193 | 0.98118927 | 1.0247518 | up | mRNA |
| NM_001130923 | RABL2B | 0.518260563 | 0.916125349 | 1.1149333 | up | mRNA |
| NM_001130929 | C7orf73 | 0.209535551 | 0.700367256 | 1.0883081 | up | mRNA |
| NM_001130964 | PLCD1 | 0.184575536 | 0.668017166 | 1.4199173 | up | mRNA |
| NM_001130965 | SUN1 | 0.829129316 | 0.98552522 | 1.0143473 | up | mRNA |
| NM_001130979 | DYSF | 0.107261238 | 0.542766422 | 1.2965826 | up | mRNA |
| NM_001130980 | DYSF | 0.532580417 | 0.92042723 | 1.1684316 | up | mRNA |
| NM_001130982 | DYSF | 0.569155055 | 0.93232103 | 1.0621011 | up | mRNA |
| NM_001130983 | DYSF | 0.554494865 | 0.926549129 | 1.0454929 | up | mRNA |
| NM_001130984 | DYSF | 0.924909627 | 0.995456159 | 1.0055337 | up | mRNA |
| NM_001130993 | RBP1 | 0.151342136 | 0.61652741 | 2.3128391 | up | mRNA |
| NM_001131 | CRISP1 | 0.271480805 | 0.771561047 | 1.1203939 | up | mRNA |
| NM_001131008 | PTPN12 | 0.628188818 | 0.949690755 | 1.1318197 | up | mRNA |
| NM_001131009 | PTPN12 | 0.501259677 | 0.909965336 | 1.1838972 | up | mRNA |
| NM_001131015 | CIZ1 | 0.68092328 | 0.960111041 | 1.079267 | up | mRNA |
| NM_001131023 | PEX5 | 0.720663337 | 0.966191059 | 1.1332838 | up | mRNA |
| NM_001131027 | PDLIM4 | 0.022008605 | 0.292481125 | 1.3021383 | up | mRNA |
| NM_001134364 | MAP4 | 0.799921558 | 0.981152656 | 1.0261896 | up | mRNA |
| NM_001134366 | GAD2 | 0.804642854 | 0.98118927 | 1.0899337 | up | mRNA |
| NM_001134367 | SLC6A6 | 0.542173265 | 0.923577434 | 1.1142951 | up | mRNA |
| NM_001134374 | SPATC1 | 0.655497273 | 0.957222998 | 1.0356657 | up | mRNA |
| NM_001134381 | TBC1D5 | 0.411045299 | 0.868434057 | 1.3917131 | up | mRNA |
| NM_001134395 | C7orf50 | 0.528710554 | 0.918883792 | 1.1410997 | up | mRNA |
| NM_001134402 | CYB561D1 | 0.24873629 | 0.747319103 | 1.1096638 | up | mRNA |
| NM_001134407 | GRIN2A | 0.413015349 | 0.86947899 | 1.6566392 | up | mRNA |
| NM_001134408 | GRIN2A | 0.495149931 | 0.907415174 | 1.0472763 | up | mRNA |
| NM_001134418 | LEPREL1 | 0.110487301 | 0.549968727 | 1.5193866 | up | mRNA |
| NM_001134422 | CDV3 | 0.083401867 | 0.492251581 | 1.3013836 | up | mRNA |
| NM_001134423 | CDV3 | 0.025975441 | 0.315604326 | 1.4942318 | up | mRNA |
| NM_001134431 | TOR2A | 0.447534392 | 0.887387289 | 1.1239474 | up | mRNA |
| NM_001134432 | AZI2 | 0.970521403 | 0.998245244 | 1.0032021 | up | mRNA |
| NM_001134433 | AZI2 | 0.999227032 | 0.999897336 | 1.0001822 | up | mRNA |
| NM_001134437 | PHLDB2 | 0.000232862 | 0.050909248 | 2.3924888 | up | mRNA |
| NM_001134439 | PHLDB2 | 0.001477147 | 0.098148183 | 2.0906041 | up | mRNA |
| NM_001134450 | TMEM130 | 0.152438557 | 0.618255775 | 1.2621096 | up | mRNA |
| NM_001134453 | DSG4 | 0.330901283 | 0.821853462 | 1.1683384 | up | mRNA |
| NM_001134492 | HS2ST1 | 0.934230646 | 0.996710991 | 1.019621 | up | mRNA |
| NM_001134647 | AFAP1 | 0.317869993 | 0.811908136 | 1.1044685 | up | mRNA |
| NM_001134658 | SLC35G1 | 0.015489789 | 0.249554977 | 2.6322849 | up | mRNA |
| NM_001134665 | TRMT10A | 0.146591568 | 0.608265259 | 1.0962585 | up | mRNA |
| NM_001134670 | HOGA1 | 0.386301209 | 0.855747763 | 1.0805243 | up | mRNA |
| NM_001134672 | FAM204A | 0.329538581 | 0.821768501 | 1.0785574 | up | mRNA |
| NM_001134673 | NFIA | 0.22968142 | 0.726716875 | 1.065297 | up | mRNA |
| NM_001134707 | SARDH | 0.470712278 | 0.898445976 | 1.0475847 | up | mRNA |
| NM_001134734 | C1orf94 | 0.331814724 | 0.822255387 | 1.0908096 | up | mRNA |
| NM_001134738 | BCL6 | 0.079992098 | 0.483876854 | 1.4499349 | up | mRNA |
| NM_001134745 | LRRTM4 | 0.894947221 | 0.993978977 | 1.0208056 | up | mRNA |
| NM_001134832 | AHI1 | 0.279972561 | 0.778697234 | 1.1503833 | up | mRNA |
| NM_001134875 | C14orf80 | 0.726545831 | 0.966191059 | 1.0633811 | up | mRNA |
| NM_001134876 | C14orf80 | 0.602914085 | 0.941464987 | 1.0135241 | up | mRNA |
| NM_001134877 | C14orf80 | 0.933695315 | 0.996710991 | 1.0098889 | up | mRNA |
| NM_001134878 | KIF9 | 0.963404132 | 0.99793927 | 1.0019473 | up | mRNA |
| NM_001134944 | GHRL | 0.28590641 | 0.782635081 | 1.5930953 | up | mRNA |
| NM_001135040 | DCTN1 | 0.084475635 | 0.494805206 | 1.1449801 | up | mRNA |
| NM_001135041 | DCTN1 | 0.513949815 | 0.91350662 | 1.062773 | up | mRNA |
| NM_001135057 | LRRC15 | 0.083823201 | 0.493058711 | 1.1048913 | up | mRNA |
| NM_001135095 | FNDC3B | 0.00095202 | 0.084526081 | 1.5103276 | up | mRNA |
| NM_001135099 | TMPRSS2 | 0.778267543 | 0.976938981 | 1.0315554 | up | mRNA |
| NM_001135105 | KCNK16 | 0.963284029 | 0.99793927 | 1.0085396 | up | mRNA |
| NM_001135113 | IGFL2 | 0.475258901 | 0.899815707 | 1.0373378 | up | mRNA |
| NM_001135146 | SLC39A8 | 0.392701576 | 0.859694706 | 1.2738882 | up | mRNA |
| NM_001135153 | SLC39A14 | 0.00264751 | 0.122061602 | 1.8698167 | up | mRNA |
| NM_001135154 | SLC39A14 | 9.26751E-06 | 0.014870836 | 2.0096106 | up | mRNA |
| NM_001135163 | SIGLEC11 | 0.122808405 | 0.574983504 | 1.1196461 | up | mRNA |
| NM_001135171 | C1QTNF7 | 0.634706035 | 0.951279916 | 1.1561746 | up | mRNA |
| NM_001135187 | AGFG1 | 0.894024779 | 0.993978977 | 1.0623373 | up | mRNA |
| NM_001135190 | ARAP1 | 0.925394141 | 0.995465766 | 1.0047533 | up | mRNA |
| NM_001135208 | FKBP2 | 0.112677106 | 0.55538204 | 1.0784802 | up | mRNA |
| NM_001135211 | FKBP6 | 0.413413808 | 0.86961884 | 1.0648112 | up | mRNA |
| NM_001135239 | LDHA | 0.380735671 | 0.853514109 | 1.1866044 | up | mRNA |
| NM_001135240 | C1orf226 | 0.294240282 | 0.791230449 | 1.0816092 | up | mRNA |
| NM_001135243 | TCOF1 | 0.524962102 | 0.917632491 | 1.0930085 | up | mRNA |
| NM_001135248 | MFAP2 | 0.836118556 | 0.986575022 | 1.0134554 | up | mRNA |
| NM_001135254 | PAX7 | 0.063638223 | 0.440919074 | 1.0942539 | up | mRNA |
| NM_001135555 | EPB41L2 | 0.719235285 | 0.966191059 | 1.0867405 | up | mRNA |
| NM_001135575 | C6orf228 | 0.12425028 | 0.578223093 | 1.0912828 | up | mRNA |
| NM_001135585 | SLC2A5 | 0.928286483 | 0.996053205 | 1.0100518 | up | mRNA |
| NM_001135592 | RPS27A | 0.124646907 | 0.578441772 | 1.184842 | up | mRNA |
| NM_001135599 | TGFB2 | 0.714590254 | 0.966110889 | 1.0581301 | up | mRNA |
| NM_001135604 | ESM1 | 0.066758292 | 0.450692395 | 2.9799775 | up | mRNA |
| NM_001135610 | PRDM2 | 0.38751162 | 0.856773191 | 1.0837755 | up | mRNA |
| NM_001135636 | PIP5K1A | 0.05480409 | 0.419413793 | 1.1809466 | up | mRNA |
| NM_001135637 | PIP5K1A | 0.127447848 | 0.580991132 | 1.1709769 | up | mRNA |
| NM_001135638 | PIP5K1A | 0.108021251 | 0.544140502 | 1.2500424 | up | mRNA |
| NM_001135650 | EEF1E1 | 0.674858248 | 0.959531882 | 1.0629745 | up | mRNA |
| NM_001135676 | C8orf40 | 0.719132223 | 0.966191059 | 1.0397495 | up | mRNA |
| NM_001135685 | LTK | 0.064809961 | 0.4445551 | 1.2024936 | up | mRNA |
| NM_001135704 | ACBD4 | 0.576298528 | 0.933656777 | 1.1973534 | up | mRNA |
| NM_001135732 | TOM1 | 0.648102158 | 0.95550188 | 1.0569497 | up | mRNA |
| NM_001135740 | PNCK | 0.544164635 | 0.923577434 | 1.1096216 | up | mRNA |
| NM_001135747 | NEIL2 | 0.242274819 | 0.740610726 | 1.1752667 | up | mRNA |
| NM_001135748 | NEIL2 | 0.611393368 | 0.943894921 | 1.0201847 | up | mRNA |
| NM_001135750 | PSMG4 | 0.249454092 | 0.747444593 | 1.2276758 | up | mRNA |
| NM_001135751 | DERL3 | 0.2301266 | 0.727446213 | 1.1307442 | up | mRNA |
| NM_001135753 | ECD | 0.946341761 | 0.996710991 | 1.0127477 | up | mRNA |
| NM_001135772 | LRP5L | 0.038186353 | 0.365047918 | 1.1401487 | up | mRNA |
| NM_001135773 | SPATA2 | 0.566362524 | 0.930978716 | 1.1179621 | up | mRNA |
| NM_001135775 | CACFD1 | 0.613481327 | 0.944660887 | 1.4711768 | up | mRNA |
| NM_001135820 | TMEM2 | 0.112031433 | 0.554156842 | 1.3107234 | up | mRNA |
| NM_001135821 | FDPS | 0.311306725 | 0.806503245 | 1.0683156 | up | mRNA |
| NM_001135823 | SSPN | 0.357321239 | 0.839643099 | 1.0497494 | up | mRNA |
| NM_001135825 | RNF185 | 0.500813864 | 0.909680616 | 1.097397 | up | mRNA |
| NM_001135861 | PHPT1 | 0.782576473 | 0.977106756 | 1.0205812 | up | mRNA |
| NM_001135862 | PHF21B | 0.368572953 | 0.845559574 | 1.1662383 | up | mRNA |
| NM_001135914 | KCP | 0.291296658 | 0.787795113 | 1.2126843 | up | mRNA |
| NM_001135917 | DOLPP1 | 0.21177862 | 0.703266283 | 1.1793367 | up | mRNA |
| NM_001135920 | KIAA1432 | 0.415548096 | 0.870382888 | 3.8811512 | up | mRNA |
| NM_001135955 | TRPC4 | 0.045593029 | 0.39096051 | 1.6390408 | up | mRNA |
| NM_001135958 | TRPC4 | 0.033500539 | 0.347704395 | 1.4696687 | up | mRNA |
| NM_001136003 | C2CD4D | 0.253991599 | 0.754221876 | 1.1221217 | up | mRNA |
| NM_001136004 | MICAL3 | 0.355478386 | 0.838369858 | 1.2252145 | up | mRNA |
| NM_001136007 | FXYD3 | 0.693508793 | 0.961632734 | 1.0516702 | up | mRNA |
| NM_001136017 | CCND3 | 0.579041955 | 0.933821303 | 1.0829559 | up | mRNA |
| NM_001136021 | NFATC2 | 0.988350155 | 0.999140676 | 1.0020314 | up | mRNA |
| NM_001136023 | NFE2 | 0.213490548 | 0.705954358 | 1.4601114 | up | mRNA |
| NM_001136024 | NHS | 0.721729456 | 0.966191059 | 1.0381143 | up | mRNA |
| NM_001136029 | DEPDC5 | 0.477502982 | 0.900611376 | 1.1088911 | up | mRNA |
| NM_001136037 | LIMS2 | 0.944589662 | 0.996710991 | 1.0229367 | up | mRNA |
| NM_001136038 | ZNF415 | 0.045173699 | 0.389384436 | 1.1312365 | up | mRNA |
| NM_001136042 | PTGES3L-AARSD1 | 0.955982727 | 0.996883425 | 1.0079625 | up | mRNA |
| NM_001136046 | ZMYND15 | 0.862764968 | 0.989401142 | 1.0262532 | up | mRNA |
| NM_001136109 | CASP5 | 0.233355435 | 0.731130266 | 1.1526042 | up | mRNA |
| NM_001136129 | APP | 0.544153246 | 0.923577434 | 1.0532503 | up | mRNA |
| NM_001136134 | RPL28 | 0.728782994 | 0.966191059 | 1.0323469 | up | mRNA |
| NM_001136136 | RPL28 | 0.480950604 | 0.901538317 | 1.1048846 | up | mRNA |
| NM_001136137 | RPL28 | 0.573591001 | 0.933656777 | 1.0422581 | up | mRNA |
| NM_001136138 | RPS6KC1 | 0.147511571 | 0.609998801 | 1.0889029 | up | mRNA |
| NM_001136157 | OTUD5 | 0.458258789 | 0.892667725 | 1.0484083 | up | mRNA |
| NM_001136159 | OTUD5 | 0.108429637 | 0.54515118 | 1.099547 | up | mRNA |
| NM_001136179 | EGR2 | 0.315866355 | 0.810545544 | 1.3186847 | up | mRNA |
| NM_001136200 | C10orf32 | 0.550216989 | 0.925693985 | 1.0170305 | up | mRNA |
| NM_001136214 | CLEC18A | 0.452607262 | 0.889207734 | 1.1016849 | up | mRNA |
| NM_001136215 | ARTN | 0.271691093 | 0.771737648 | 1.1243034 | up | mRNA |
| NM_001136217 | TMEM51 | 0.062776518 | 0.439458294 | 1.911139 | up | mRNA |
| NM_001136265 | IFFO2 | 0.404922838 | 0.866165103 | 1.1327981 | up | mRNA |
| NM_001136268 | DAZAP2 | 0.158167462 | 0.628711796 | 1.1953902 | up | mRNA |
| NM_001136269 | DAZAP2 | 0.444311601 | 0.885088246 | 2.9640402 | up | mRNA |
| NM_001136275 | AKIRIN1 | 0.275768283 | 0.775616353 | 1.1129196 | up | mRNA |
| NM_001136473 | LITAF | 0.007179921 | 0.189833754 | 1.4453525 | up | mRNA |
| NM_001136475 | VASH2 | 0.677371971 | 0.959531882 | 1.0424473 | up | mRNA |
| NM_001136485 | C11orf86 | 0.209610413 | 0.700367256 | 1.1372055 | up | mRNA |
| NM_001136495 | C1orf198 | 0.795487003 | 0.979995258 | 1.0653647 | up | mRNA |
| NM_001136498 | CISD3 | 0.894709669 | 0.993978977 | 1.0157344 | up | mRNA |
| NM_001136504 | SYT2 | 0.386701949 | 0.856062164 | 1.0822523 | up | mRNA |
| NM_001136508 | C1orf185 | 0.709621594 | 0.965462762 | 1.1115667 | up | mRNA |
| NM_001136528 | SERPINE2 | 0.206127176 | 0.697334067 | 1.4396494 | up | mRNA |
| NM_001136530 | SERPINE2 | 0.001748883 | 0.105670067 | 4.1384787 | up | mRNA |
| NM_001136540 | APOL1 | 0.502604688 | 0.910335149 | 1.1513541 | up | mRNA |
| NM_001136541 | APOL1 | 0.116045925 | 0.562308327 | 2.7987578 | up | mRNA |
| NM_001136562 | AKAP2 | 0.001145056 | 0.088098571 | 1.98362 | up | mRNA |
| NM_001137548 | FAM25C | 0.364263102 | 0.84362654 | 1.0813468 | up | mRNA |
| NM_001137552 | LRRFIP1 | 0.589619191 | 0.936090876 | 1.0712607 | up | mRNA |
| NM_001137601 | ZBTB42 | 0.213695853 | 0.706323665 | 1.1339662 | up | mRNA |
| NM_001137608 | ZNF732 | 0.746135974 | 0.970394407 | 1.0158836 | up | mRNA |
| NM_001137668 | CASP8AP2 | 0.658241289 | 0.958354006 | 1.0234478 | up | mRNA |
| NM_001137669 | RGSL1 | 0.826198338 | 0.984374112 | 1.0309909 | up | mRNA |
| NM_001137675 | ATXN1L | 0.835138966 | 0.986575022 | 1.0343402 | up | mRNA |
| NM_001139442 | TTLL11 | 0.777120228 | 0.976841327 | 1.0462375 | up | mRNA |
| NM_001139443 | BEST1 | 0.367446542 | 0.845181673 | 1.1511755 | up | mRNA |
| NM_001139444 | BET3L | 0.788606999 | 0.977739953 | 1.0351305 | up | mRNA |
| NM_001139456 | SVOPL | 0.102156164 | 0.53053362 | 1.1558731 | up | mRNA |
| NM_001139500 | FGF13 | 0.786596654 | 0.977479172 | 1.0353303 | up | mRNA |
| NM_001139514 | DACH2 | 0.295647055 | 0.791662837 | 1.0830343 | up | mRNA |
| NM_001140 | ALOX15 | 0.458327573 | 0.892667725 | 1.7280763 | up | mRNA |
| NM_001141 | ALOX15B | 0.202815468 | 0.692118849 | 1.3400521 | up | mRNA |
| NM_001141917 | FAM205A | 0.444252176 | 0.885088246 | 1.0215705 | up | mRNA |
| NM_001141919 | XG | 0.552087622 | 0.926337111 | 1.0649399 | up | mRNA |
| NM_001141936 | C4orf48 | 0.739762338 | 0.969564098 | 1.0283761 | up | mRNA |
| NM_001141969 | DAXX | 0.957687955 | 0.996933183 | 1.0056586 | up | mRNA |
| NM_001142274 | CLASP1 | 0.374524917 | 0.84936907 | 1.0852029 | up | mRNA |
| NM_001142276 | APLP2 | 0.881806177 | 0.992766819 | 1.0235891 | up | mRNA |
| NM_001142291 | MGRN1 | 0.59252387 | 0.937776808 | 1.0711046 | up | mRNA |
| NM_001142293 | ARFGAP3 | 0.099943483 | 0.525350189 | 1.1079632 | up | mRNA |
| NM_001142299 | SQSTM1 | 0.473087329 | 0.899184677 | 1.2734188 | up | mRNA |
| NM_001142300 | CCNYL1 | 0.442986597 | 0.884304813 | 1.7036126 | up | mRNA |
| NM_001142307 | GTF2H1 | 0.223821139 | 0.717828939 | 1.1446448 | up | mRNA |
| NM_001142314 | DUSP19 | 0.25288474 | 0.753245318 | 1.2797513 | up | mRNA |
| NM_001142318 | TXNL4B | 0.12560626 | 0.578441772 | 1.1835681 | up | mRNA |
| NM_001142324 | SLC18A1 | 0.279055125 | 0.777816764 | 1.1167186 | up | mRNA |
| NM_001142348 | SCN4B | 0.119231094 | 0.568456822 | 1.1588204 | up | mRNA |
| NM_001142352 | ST6GAL2 | 0.57487647 | 0.933656777 | 1.0317051 | up | mRNA |
| NM_001142364 | ALG5 | 0.976263249 | 0.998501532 | 1.0020212 | up | mRNA |
| NM_001142386 | PDK3 | 0.185834763 | 0.669321303 | 1.1518746 | up | mRNA |
| NM_001142393 | NEDD9 | 0.069010194 | 0.455905782 | 1.5896124 | up | mRNA |
| NM_001142397 | CTIF | 0.943161563 | 0.996710991 | 1.0075414 | up | mRNA |
| NM_001142398 | FAHD1 | 0.942011346 | 0.996710991 | 1.0126265 | up | mRNA |
| NM_001142416 | AIMP1 | 0.473454349 | 0.899184677 | 1.058414 | up | mRNA |
| NM_001142447 | ATP1B4 | 0.691048633 | 0.961105466 | 1.1373952 | up | mRNA |
| NM_001142467 | HES4 | 0.845992049 | 0.987338076 | 1.0383734 | up | mRNA |
| NM_001142469 | C16orf45 | 0.206919261 | 0.698502807 | 1.1517602 | up | mRNA |
| NM_001142495 | MYO5A | 0.294967415 | 0.791301949 | 1.0808642 | up | mRNA |
| NM_001142498 | SIRT1 | 0.647872143 | 0.95550188 | 1.0503182 | up | mRNA |
| NM_001142505 | ABCG4 | 0.102956553 | 0.532378596 | 1.1671759 | up | mRNA |
| NM_001142523 | IRAK3 | 0.004746013 | 0.15793115 | 1.4260091 | up | mRNA |
| NM_001142537 | LGALS12 | 0.58989995 | 0.936114189 | 1.0932132 | up | mRNA |
| NM_001142538 | LGALS12 | 0.909612033 | 0.995342392 | 1.0080812 | up | mRNA |
| NM_001142544 | CTF1 | 0.692323255 | 0.961562704 | 1.0347233 | up | mRNA |
| NM_001142555 | ADCK4 | 0.395533793 | 0.861590143 | 1.1148875 | up | mRNA |
| NM_001142556 | HMMR | 0.322816003 | 0.815766149 | 1.1816162 | up | mRNA |
| NM_001142576 | IMPDH1 | 0.002705924 | 0.122805466 | 1.5737452 | up | mRNA |
| NM_001142614 | EHBP1 | 0.961897957 | 0.99793927 | 1.0085932 | up | mRNA |
| NM_001142619 | STRA6 | 0.94167581 | 0.996710991 | 1.0085498 | up | mRNA |
| NM_001142640 | TNRC6C | 0.559417851 | 0.927203296 | 1.127255 | up | mRNA |
| NM_001142643 | CASKIN2 | 0.278160393 | 0.777440204 | 1.3903018 | up | mRNA |
| NM_001142644 | SPHKAP | 0.064778134 | 0.444527127 | 1.2379437 | up | mRNA |
| NM_001142645 | TMEM194B | 0.853065067 | 0.987678166 | 1.0414126 | up | mRNA |
| NM_001142673 | ATG13 | 0.421107637 | 0.872879684 | 1.1039244 | up | mRNA |
| NM_001142677 | CHID1 | 0.899056435 | 0.994575817 | 1.0266075 | up | mRNA |
| NM_001142683 | CCDC121 | 0.170733505 | 0.647381721 | 1.2299705 | up | mRNA |
| NM_001142699 | DLG2 | 0.843900612 | 0.987338076 | 1.0197299 | up | mRNA |
| NM_001142749 | KIAA1324L | 0.26870789 | 0.76931062 | 1.2770014 | up | mRNA |
| NM_001142766 | PCDH15 | 0.88998667 | 0.9937521 | 1.0322805 | up | mRNA |
| NM_001142776 | CHAC1 | 0.010584471 | 0.210159643 | 1.2856769 | up | mRNA |
| NM_001142777 | HSD3B7 | 0.142344192 | 0.602652383 | 1.1379942 | up | mRNA |
| NM_001142784 | IL11RA | 0.839286467 | 0.987032229 | 1.0175391 | up | mRNA |
| NM_001142798 | C17orf49 | 0.560205406 | 0.92782421 | 1.12747 | up | mRNA |
| NM_001142799 | C17orf49 | 0.583210028 | 0.934269626 | 1.1007 | up | mRNA |
| NM_001142853 | HES6 | 0.834112768 | 0.986475536 | 1.0457365 | up | mRNA |
| NM_001142854 | C21orf56 | 0.269090029 | 0.769715598 | 1.0418277 | up | mRNA |
| NM_001142864 | PIEZO1 | 0.097257474 | 0.519831481 | 1.160793 | up | mRNA |
| NM_001142885 | TMOD2 | 0.333248825 | 0.82335721 | 1.1019398 | up | mRNA |
| NM_001142946 | C11orf21 | 0.84352632 | 0.987338076 | 1.0386769 | up | mRNA |
| NM_001143677 | SGK1 | 0.145806027 | 0.607759658 | 1.0697182 | up | mRNA |
| NM_001143685 | CES5A | 0.055343832 | 0.419636174 | 1.1417163 | up | mRNA |
| NM_001143687 | DCST1 | 0.511666876 | 0.912662856 | 1.0598436 | up | mRNA |
| NM_001143758 | LRRC27 | 0.044068328 | 0.386039315 | 1.4280794 | up | mRNA |
| NM_001143760 | EIF5A | 0.531258092 | 0.919882712 | 1.1736201 | up | mRNA |
| NM_001143761 | EIF5A | 0.693965463 | 0.96187237 | 1.0323748 | up | mRNA |
| NM_001143782 | FKBP11 | 0.11119959 | 0.552114792 | 1.1811093 | up | mRNA |
| NM_001143784 | FES | 0.947644618 | 0.996710991 | 1.0110095 | up | mRNA |
| NM_001143819 | TPCN1 | 0.868566839 | 0.989481868 | 1.0105107 | up | mRNA |
| NM_001143820 | ETS1 | 0.00318932 | 0.133152455 | 2.4669698 | up | mRNA |
| NM_001143821 | PLEKHA5 | 0.710421424 | 0.965462762 | 1.1094828 | up | mRNA |
| NM_001143823 | ZFP161 | 0.019337228 | 0.280031712 | 1.1071774 | up | mRNA |
| NM_001143824 | SLC38A4 | 0.912030126 | 0.995342392 | 1.0363404 | up | mRNA |
| NM_001143830 | GAS2 | 0.684721148 | 0.960111041 | 1.0483262 | up | mRNA |
| NM_001143836 | NOX4 | 0.714921807 | 0.966131151 | 1.3003604 | up | mRNA |
| NM_001143842 | TMEM106C | 0.835531305 | 0.986575022 | 1.0172719 | up | mRNA |
| NM_001143843 | TMEM106C | 0.38547637 | 0.85515381 | 1.1934139 | up | mRNA |
| NM_001143854 | RPH3A | 0.938065546 | 0.996710991 | 1.0309862 | up | mRNA |
| NM_001143883 | PPIAL4B | 0.880537677 | 0.992287164 | 1.0076589 | up | mRNA |
| NM_001143888 | BSDC1 | 0.132084426 | 0.587843174 | 1.5360757 | up | mRNA |
| NM_001143889 | BSDC1 | 0.145773514 | 0.607759658 | 1.335803 | up | mRNA |
| NM_001143890 | BSDC1 | 0.226960278 | 0.722378894 | 1.2545541 | up | mRNA |
| NM_001143913 | FAM76A | 0.020713321 | 0.286034385 | 1.2095058 | up | mRNA |
| NM_001143941 | RBM24 | 0.946037181 | 0.996710991 | 1.0126841 | up | mRNA |
| NM_001143943 | EFCAB2 | 0.903578664 | 0.99508468 | 1.0230127 | up | mRNA |
| NM_001143971 | CDYL | 0.145117922 | 0.606482829 | 1.1584011 | up | mRNA |
| NM_001143982 | CHRDL1 | 0.575947519 | 0.933656777 | 1.0544076 | up | mRNA |
| NM_001143983 | CHRDL1 | 0.921923086 | 0.995349793 | 1.0388727 | up | mRNA |
| NM_001143984 | PTPMT1 | 0.000197913 | 0.047904279 | 1.1404182 | up | mRNA |
| NM_001143986 | TLE6 | 0.622769661 | 0.948341211 | 1.0893826 | up | mRNA |
| NM_001143993 | RASSF7 | 0.91332569 | 0.995342392 | 1.0277953 | up | mRNA |
| NM_001144026 | NDOR1 | 0.931042927 | 0.996710991 | 1.0066067 | up | mRNA |
| NM_001144030 | NAT10 | 0.374044457 | 0.849308269 | 1.1646733 | up | mRNA |
| NM_001144038 | TMEM25 | 0.926741496 | 0.995829678 | 1.0088033 | up | mRNA |
| NM_001144058 | NTM | 0.544501244 | 0.92372126 | 1.0611049 | up | mRNA |
| NM_001144059 | NTM | 0.874091481 | 0.990793509 | 1.0434133 | up | mRNA |
| NM_001144068 | ZNF772 | 0.316273593 | 0.810808526 | 1.0810046 | up | mRNA |
| NM_001144663 | CDH17 | 0.40150836 | 0.864768873 | 1.0887528 | up | mRNA |
| NM_001144757 | SCG5 | 0.969445914 | 0.998245244 | 1.0046541 | up | mRNA |
| NM_001144761 | TLE2 | 0.858040419 | 0.988512246 | 1.0104281 | up | mRNA |
| NM_001144762 | TLE2 | 0.752831151 | 0.970725371 | 1.040894 | up | mRNA |
| NM_001144772 | NSMAF | 0.693389871 | 0.961584652 | 1.0659421 | up | mRNA |
| NM_001144822 | CD58 | 0.026522975 | 0.318338323 | 1.6174635 | up | mRNA |
| NM_001144823 | DENND4A | 0.140846006 | 0.601036902 | 1.2831955 | up | mRNA |
| NM_001144830 | FMO5 | 0.452247028 | 0.888854908 | 1.14641 | up | mRNA |
| NM_001144832 | TTC39A | 0.412455119 | 0.869219033 | 1.0984462 | up | mRNA |
| NM_001144871 | VSTM5 | 0.972391446 | 0.998501532 | 1.0042329 | up | mRNA |
| NM_001144875 | DOK3 | 0.087713719 | 0.501332443 | 1.0960174 | up | mRNA |
| NM_001144878 | IMPA1 | 0.210377142 | 0.701096222 | 1.0911006 | up | mRNA |
| NM_001144884 | SLC30A7 | 0.028694058 | 0.329236786 | 1.2153698 | up | mRNA |
| NM_001144890 | SLC23A3 | 0.196795842 | 0.685161614 | 1.0676517 | up | mRNA |
| NM_001144895 | CD209 | 0.308963975 | 0.803961878 | 1.0848664 | up | mRNA |
| NM_001144896 | CD209 | 0.638123955 | 0.952852353 | 1.0473656 | up | mRNA |
| NM_001144897 | CD209 | 0.039836087 | 0.371365115 | 1.3881676 | up | mRNA |
| NM_001144913 | FGFR2 | 0.567294548 | 0.931514776 | 1.1477609 | up | mRNA |
| NM_001144914 | FGFR2 | 0.080242376 | 0.483876854 | 1.3557406 | up | mRNA |
| NM_001144917 | FGFR2 | 0.616507029 | 0.945912508 | 1.1444891 | up | mRNA |
| NM_001144918 | FGFR2 | 0.114215969 | 0.558096391 | 1.1015995 | up | mRNA |
| NM_001144932 | PSMB5 | 0.988677502 | 0.999140676 | 1.0035767 | up | mRNA |
| NM_001144951 | GLYCTK | 0.70394343 | 0.963625105 | 1.0423055 | up | mRNA |
| NM_001144952 | SDK2 | 0.10765369 | 0.543907835 | 1.1891946 | up | mRNA |
| NM_001144972 | SNX20 | 0.246904725 | 0.745152522 | 1.0475164 | up | mRNA |
| NM_001144994 | C2orf72 | 0.578632841 | 0.933821303 | 1.0962599 | up | mRNA |
| NM_001144999 | ITGAV | 0.00164927 | 0.104139643 | 1.2535982 | up | mRNA |
| NM_001145000 | ITGAV | 0.04103221 | 0.375308608 | 1.2013282 | up | mRNA |
| NM_001145007 | MUC7 | 0.808846048 | 0.98118927 | 1.0172693 | up | mRNA |
| NM_001145009 | BTN3A1 | 0.874669432 | 0.990905383 | 1.0072683 | up | mRNA |
| NM_001145018 | CCDC153 | 0.558413134 | 0.926833616 | 1.0904317 | up | mRNA |
| NM_001145026 | PTPRQ | 0.736241314 | 0.968717225 | 1.19931 | up | mRNA |
| NM_001145031 | PLAU | 0.338584995 | 0.827403747 | 4.9506627 | up | mRNA |
| NM_001145033 | C11orf96 | 0.015180481 | 0.247513663 | 4.8029851 | up | mRNA |
| NM_001145044 | SLCO3A1 | 0.072473698 | 0.463255488 | 1.2368916 | up | mRNA |
| NM_001145057 | IL18BP | 0.941772102 | 0.996710991 | 1.00213 | up | mRNA |
| NM_001145064 | GATSL2 | 0.382353885 | 0.853514109 | 1.0904104 | up | mRNA |
| NM_001145073 | USP27X | 0.905859146 | 0.99508468 | 1.017495 | up | mRNA |
| NM_001145076 | EML4 | 0.066104884 | 0.449693785 | 1.2865098 | up | mRNA |
| NM_001145097 | SKIL | 0.272202925 | 0.772141487 | 1.2516073 | up | mRNA |
| NM_001145099 | SLC2A6 | 0.065303578 | 0.446303313 | 1.1234374 | up | mRNA |
| NM_001145101 | BTBD18 | 0.160522313 | 0.632016252 | 1.0681635 | up | mRNA |
| NM_001145102 | SMAD3 | 0.367143498 | 0.845181673 | 1.1373616 | up | mRNA |
| NM_001145104 | SMAD3 | 0.557820917 | 0.926833616 | 1.0276605 | up | mRNA |
| NM_001145122 | CAPN14 | 0.091268393 | 0.50968981 | 1.2793424 | up | mRNA |
| NM_001145124 | FAM75C1 | 0.500250835 | 0.909680616 | 1.1226043 | up | mRNA |
| NM_001145125 | MKRN1 | 0.852019008 | 0.987678166 | 1.0138009 | up | mRNA |
| NM_001145136 | CPT1B | 0.46216376 | 0.894754673 | 1.0950932 | up | mRNA |
| NM_001145138 | RELA | 0.116322677 | 0.562576753 | 1.3345643 | up | mRNA |
| NM_001145140 | CXorf49 | 0.067693055 | 0.451808679 | 1.1835763 | up | mRNA |
| NM_001145143 | HTR3D | 0.125195264 | 0.578441772 | 1.0558981 | up | mRNA |
| NM_001145146 | CRHR1 | 0.377852529 | 0.851340605 | 1.1694072 | up | mRNA |
| NM_001145147 | CRHR1 | 0.399969274 | 0.864205663 | 1.1442689 | up | mRNA |
| NM_001145148 | CRHR1 | 0.893457803 | 0.993978977 | 1.0147484 | up | mRNA |
| NM_001145196 | FAM75A6 | 0.945130965 | 0.996710991 | 1.0139398 | up | mRNA |
| NM_001145204 | SHISA9 | 0.875106326 | 0.991094934 | 1.0133814 | up | mRNA |
| NM_001145207 | HBS1L | 0.252591352 | 0.752702237 | 1.6989104 | up | mRNA |
| NM_001145210 | ANKRD65 | 0.824687141 | 0.983779567 | 1.0253749 | up | mRNA |
| NM_001145224 | GOLGA6D | 0.994067426 | 0.999872237 | 1.0029085 | up | mRNA |
| NM_001145260 | NCOA4 | 0.050748775 | 0.407674476 | 1.3727045 | up | mRNA |
| NM_001145275 | ZFY | 0.031735827 | 0.341807319 | 1.4440496 | up | mRNA |
| NM_001145276 | ZFY | 0.147067315 | 0.609351845 | 1.4636351 | up | mRNA |
| NM_001145279 | OPRM1 | 0.813059838 | 0.98120545 | 1.0469355 | up | mRNA |
| NM_001145284 | OPRM1 | 0.14429748 | 0.605523499 | 1.1122743 | up | mRNA |
| NM_001145291 | PDE6B | 0.527627603 | 0.918678174 | 1.1155723 | up | mRNA |
| NM_001145292 | PDE6B | 0.412685925 | 0.869333523 | 1.0411438 | up | mRNA |
| NM_001145306 | CDK6 | 0.000757837 | 0.08067192 | 2.0375253 | up | mRNA |
| NM_001145312 | ETV3 | 0.068626123 | 0.454916653 | 1.2955969 | up | mRNA |
| NM_001145349 | IPP | 0.60769913 | 0.942627675 | 1.054339 | up | mRNA |
| NM_001145353 | ELF1 | 0.651815017 | 0.956204521 | 1.0306144 | up | mRNA |
| NM_001145357 | SIN3A | 0.087024879 | 0.500185639 | 1.3925296 | up | mRNA |
| NM_001145398 | TEF | 0.77897474 | 0.976938981 | 1.0434813 | up | mRNA |
| NM_001145399 | MPPED2 | 0.900972035 | 0.994789506 | 1.0194833 | up | mRNA |
| NM_001145400 | ADAD2 | 0.785283308 | 0.977411899 | 1.01852 | up | mRNA |
| NM_001145409 | NONO | 0.500964393 | 0.909754202 | 1.0950471 | up | mRNA |
| NM_001145412 | NFE2L2 | 0.159372968 | 0.631041393 | 1.3793428 | up | mRNA |
| NM_001145413 | NFE2L2 | 0.083138888 | 0.491675352 | 1.4140705 | up | mRNA |
| NM_001145436 | LSS | 0.217836255 | 0.712065391 | 1.1401143 | up | mRNA |
| NM_001145453 | GFRA1 | 0.696383087 | 0.962410025 | 1.0598709 | up | mRNA |
| NM_001145460 | HOPX | 0.307585624 | 0.802348229 | 1.1006897 | up | mRNA |
| NM_001145466 | NCR3 | 0.654664404 | 0.957205183 | 1.0811953 | up | mRNA |
| NM_001145473 | LOXHD1 | 0.021889419 | 0.291648534 | 1.2770721 | up | mRNA |
| NM_001145511 | NFIA | 0.35747818 | 0.839643099 | 1.0755117 | up | mRNA |
| NM_001145513 | SCRN1 | 0.203264294 | 0.692876095 | 1.1606764 | up | mRNA |
| NM_001145522 | RAI14 | 0.603426033 | 0.941484604 | 1.1928701 | up | mRNA |
| NM_001145523 | RAI14 | 0.863229718 | 0.989401142 | 1.0550813 | up | mRNA |
| NM_001145525 | RAI14 | 0.858816354 | 0.988512246 | 1.0382452 | up | mRNA |
| NM_001145526 | AFMID | 0.454716612 | 0.890855478 | 1.0765289 | up | mRNA |
| NM_001145542 | ZSCAN18 | 0.024737293 | 0.307244129 | 1.3056996 | up | mRNA |
| NM_001145544 | ZSCAN18 | 0.967781272 | 0.99793927 | 1.0084462 | up | mRNA |
| NM_001145545 | C16orf82 | 0.641882058 | 0.954332328 | 1.1227972 | up | mRNA |
| NM_001145547 | RBM17 | 0.593436417 | 0.938165712 | 1.0596085 | up | mRNA |
| NM_001145635 | TRIM45 | 0.619428647 | 0.947444797 | 1.0735538 | up | mRNA |
| NM_001145640 | ZFR2 | 0.806123191 | 0.98118927 | 1.0285479 | up | mRNA |
| NM_001145648 | RASGRF1 | 0.321745657 | 0.815271211 | 1.269744 | up | mRNA |
| NM_001145662 | GATA2 | 0.917393179 | 0.995349793 | 1.030877 | up | mRNA |
| NM_001145666 | GLG1 | 0.957458457 | 0.996883425 | 1.0068741 | up | mRNA |
| NM_001145670 | SORBS2 | 0.040066666 | 0.371915487 | 1.2812331 | up | mRNA |
| NM_001145671 | SORBS2 | 0.338756387 | 0.827403747 | 1.0700644 | up | mRNA |
| NM_001145673 | SORBS2 | 0.867165657 | 0.989481868 | 1.0616836 | up | mRNA |
| NM_001145720 | ZBTB8B | 0.34614134 | 0.833163669 | 1.3026387 | up | mRNA |
| NM_001145727 | IFLTD1 | 0.207828377 | 0.698556218 | 1.1278952 | up | mRNA |
| NM_001145783 | MEF2BNB | 0.706816574 | 0.965401045 | 1.0372747 | up | mRNA |
| NM_001145785 | MEF2B | 0.67610873 | 0.959531882 | 1.0655353 | up | mRNA |
| NM_001145794 | ANTXR2 | 0.010895225 | 0.213674567 | 1.9653992 | up | mRNA |
| NM_001145795 | SH2B1 | 0.770014477 | 0.975286259 | 1.0334317 | up | mRNA |
| NM_001145812 | SH2B1 | 0.769738744 | 0.975286259 | 1.0427835 | up | mRNA |
| NM_001145841 | TNNI2 | 0.974757638 | 0.998501532 | 1.0045693 | up | mRNA |
| NM_001145845 | ROBO1 | 0.439114961 | 0.882895421 | 1.0751042 | up | mRNA |
| NM_001145853 | WFS1 | 0.054334427 | 0.418298968 | 1.1877616 | up | mRNA |
| NM_001145860 | POP1 | 0.663394098 | 0.958354006 | 1.08676 | up | mRNA |
| NM_001145862 | MTMR11 | 0.828260683 | 0.985325387 | 1.1106791 | up | mRNA |
| NM_001145873 | CD8A | 0.26791693 | 0.768778917 | 1.1445475 | up | mRNA |
| NM_001145903 | C2 | 0.406037053 | 0.866541972 | 1.0666659 | up | mRNA |
| NM_001145946 | SLCO1C1 | 0.252135357 | 0.752621656 | 1.426532 | up | mRNA |
| NM_001145975 | SLC13A2 | 0.227855248 | 0.723854154 | 1.3511735 | up | mRNA |
| NM_001146 | ANGPT1 | 0.843909505 | 0.987338076 | 1.1048044 | up | mRNA |
| NM_001146008 | SLCO5A1 | 0.769844288 | 0.975286259 | 1.0392352 | up | mRNA |
| NM_001146032 | FCHO2 | 0.290369651 | 0.786543531 | 1.4181056 | up | mRNA |
| NM_001146033 | OR56A5 | 0.907842633 | 0.995145546 | 1.0206134 | up | mRNA |
| NM_001146039 | GORAB | 0.001797099 | 0.105670067 | 1.9903232 | up | mRNA |
| NM_001146082 | MBOAT7 | 0.632614527 | 0.951123179 | 1.0642741 | up | mRNA |
| NM_001146105 | PARP9 | 0.740194154 | 0.969794316 | 1.0413654 | up | mRNA |
| NM_001146155 | PTGR2 | 0.150772789 | 0.615920812 | 1.1048886 | up | mRNA |
| NM_001146156 | GSK3B | 0.22246881 | 0.71737156 | 1.1054512 | up | mRNA |
| NM_001146157 | FAM25A | 0.69015766 | 0.961105466 | 1.0182893 | up | mRNA |
| NM_001146170 | TATDN3 | 0.613917 | 0.944827645 | 1.0231984 | up | mRNA |
| NM_001146171 | TATDN3 | 0.58152823 | 0.934005566 | 1.2534342 | up | mRNA |
| NM_001146172 | SLAMF9 | 0.765586745 | 0.974574862 | 1.1789116 | up | mRNA |
| NM_001146178 | IP6K2 | 0.84526105 | 0.987338076 | 1.1017698 | up | mRNA |
| NM_001146181 | LOC649330 | 0.103734355 | 0.533622448 | 1.1914298 | up | mRNA |
| NM_001146190 | ZNF407 | 0.672918308 | 0.959531882 | 1.0642018 | up | mRNA |
| NM_001146191 | MPZL1 | 0.018857391 | 0.275239397 | 1.2535174 | up | mRNA |
| NM_001146197 | CCDC168 | 0.004786446 | 0.158085585 | 2.6912734 | up | mRNA |
| NM_001146203 | RASGRP4 | 0.436857337 | 0.882131629 | 1.1525043 | up | mRNA |
| NM_001146207 | RASGRP4 | 0.157222234 | 0.625935125 | 1.0584095 | up | mRNA |
| NM_001146210 | SPDYE6 | 0.000716105 | 0.078885568 | 1.9306187 | up | mRNA |
| NM_001146216 | TACC1 | 0.184900706 | 0.668140272 | 1.2043049 | up | mRNA |
| NM_001146218 | WRB | 0.934485878 | 0.996710991 | 1.0177724 | up | mRNA |
| NM_001146227 | RPS20 | 0.633094658 | 0.951174475 | 1.0429105 | up | mRNA |
| NM_001146256 | ZDHHC15 | 0.410011499 | 0.86807846 | 1.1708433 | up | mRNA |
| NM_001146322 | GRAMD3 | 0.165831131 | 0.639669852 | 1.1924713 | up | mRNA |
| NM_001146334 | NACAD | 0.657934785 | 0.958309968 | 1.0793281 | up | mRNA |
| NM_001146340 | NKX1-2 | 0.115550057 | 0.561243135 | 1.1929284 | up | mRNA |
| NM_001146345 | PQLC1 | 0.840794337 | 0.987068842 | 1.0143249 | up | mRNA |
| NM_001146684 | RNF222 | 0.243479419 | 0.741870658 | 1.131731 | up | mRNA |
| NM_001146686 | GMNC | 0.491407341 | 0.905806259 | 1.6232859 | up | mRNA |
| NM_001146696 | KDM4C | 0.966142301 | 0.99793927 | 1.004784 | up | mRNA |
| NM_001146698 | RBM19 | 0.169273879 | 0.645717918 | 1.1910952 | up | mRNA |
| NM_001146728 | HRASLS5 | 0.423010594 | 0.873989281 | 1.2116487 | up | mRNA |
| NM_001148 | ANK2 | 0.538659225 | 0.923577434 | 1.2405842 | up | mRNA |
| NM_001151 | SLC25A4 | 0.909750165 | 0.995342392 | 1.0128996 | up | mRNA |
| NM_001155 | ANXA6 | 0.56415234 | 0.929470235 | 1.0632906 | up | mRNA |
| NM_001157 | ANXA11 | 0.534232563 | 0.920652989 | 1.0837073 | up | mRNA |
| NM_001159 | AOX1 | 0.226346394 | 0.721667695 | 1.1345014 | up | mRNA |
| NM_001159286 | HDAC10 | 0.469648609 | 0.89787452 | 1.0543243 | up | mRNA |
| NM_001159295 | ADAD1 | 0.436712879 | 0.882102748 | 1.0775663 | up | mRNA |
| NM_001159353 | REG4 | 0.144757385 | 0.605951408 | 1.350794 | up | mRNA |
| NM_001159378 | MTHFSD | 0.586816547 | 0.93579682 | 1.1404092 | up | mRNA |
| NM_001159387 | B4GALNT2 | 0.511068461 | 0.912481293 | 1.0536926 | up | mRNA |
| NM_001159391 | GUK1 | 0.81347357 | 0.98120545 | 1.0102488 | up | mRNA |
| NM_001159398 | FCRL1 | 0.749741841 | 0.970725371 | 1.1287813 | up | mRNA |
| NM_001159495 | BANF2 | 0.635030919 | 0.951399945 | 1.0178018 | up | mRNA |
| NM_001159524 | ZNF735 | 0.950487626 | 0.996710991 | 1.011122 | up | mRNA |
| NM_001159707 | BTNL8 | 0.536844459 | 0.922650285 | 1.1067952 | up | mRNA |
| NM_001159727 | PLBD2 | 0.478958886 | 0.901271944 | 1.0673736 | up | mRNA |
| NM_001159740 | LTA | 0.40386955 | 0.865367868 | 1.1637801 | up | mRNA |
| NM_001159770 | SLC39A11 | 0.592721424 | 0.937820942 | 1.0481886 | up | mRNA |
| NM_001160001 | NRG1 | 0.388636043 | 0.857898453 | 1.3709374 | up | mRNA |
| NM_001160102 | LPO | 0.744487296 | 0.970330526 | 1.036186 | up | mRNA |
| NM_001160125 | KLF6 | 0.093815065 | 0.513134818 | 1.2845247 | up | mRNA |
| NM_001160183 | ZNF138 | 0.49297794 | 0.906130999 | 1.2221664 | up | mRNA |
| NM_001160184 | PLEKHN1 | 0.093879709 | 0.513134818 | 1.2513237 | up | mRNA |
| NM_001160213 | PMFBP1 | 0.096464528 | 0.518318389 | 1.1199752 | up | mRNA |
| NM_001160233 | ATP1A1 | 0.483999884 | 0.903250293 | 1.0838671 | up | mRNA |
| NM_001160244 | RPAIN | 0.482278958 | 0.902253937 | 1.0839685 | up | mRNA |
| NM_001160246 | RPAIN | 0.558276658 | 0.926833616 | 1.0691934 | up | mRNA |
| NM_001160266 | RPAIN | 0.703433186 | 0.963625105 | 1.0411019 | up | mRNA |
| NM_001160301 | DPYD | 0.229795056 | 0.726859868 | 1.1694788 | up | mRNA |
| NM_001160355 | LY6K | 0.840258647 | 0.987068842 | 1.0225347 | up | mRNA |
| NM_001160372 | TRAPPC9 | 0.23006819 | 0.72737466 | 1.2595114 | up | mRNA |
| NM_001160392 | TRPT1 | 0.95434875 | 0.996710991 | 1.0039993 | up | mRNA |
| NM_001160423 | IGF2BP1 | 0.617575851 | 0.94659436 | 1.0694161 | up | mRNA |
| NM_001161342 | TMEM171 | 0.453193021 | 0.889563297 | 1.3062612 | up | mRNA |
| NM_001161346 | CHFR | 0.087025579 | 0.500185639 | 1.2006809 | up | mRNA |
| NM_001161347 | CHFR | 0.898920751 | 0.99453218 | 1.0111839 | up | mRNA |
| NM_001161354 | PLEKHA4 | 0.136828802 | 0.595718028 | 1.1527849 | up | mRNA |
| NM_001161403 | LIMS2 | 0.04757371 | 0.397835916 | 1.327739 | up | mRNA |
| NM_001161440 | PTPRH | 0.738189726 | 0.969207218 | 1.0174331 | up | mRNA |
| NM_001161441 | SH2D2A | 0.724249079 | 0.966191059 | 1.0575897 | up | mRNA |
| NM_001161442 | SH2D2A | 0.12792454 | 0.581220503 | 1.103866 | up | mRNA |
| NM_001161454 | CYBASC3 | 0.027443412 | 0.322334413 | 1.2828657 | up | mRNA |
| NM_001161473 | ALDH3B1 | 0.542730494 | 0.923577434 | 1.0819915 | up | mRNA |
| NM_001161476 | WDR25 | 0.498982985 | 0.909361611 | 1.1488381 | up | mRNA |
| NM_001161504 | ALDH4A1 | 0.979952692 | 0.998971807 | 1.0010262 | up | mRNA |
| NM_001161520 | COG5 | 0.347775438 | 0.834082222 | 1.0655039 | up | mRNA |
| NM_001161527 | TNIP2 | 0.001820675 | 0.105670067 | 1.6525 | up | mRNA |
| NM_001161561 | TNIK | 0.860944692 | 0.989013989 | 1.0258637 | up | mRNA |
| NM_001161562 | TNIK | 0.582457202 | 0.934005566 | 1.0516363 | up | mRNA |
| NM_001161565 | TNIK | 0.425409641 | 0.875271081 | 1.0488801 | up | mRNA |
| NM_001161573 | MAFF | 0.001369965 | 0.094832054 | 2.1316489 | up | mRNA |
| NM_001161574 | MAFF | 0.004898927 | 0.158753988 | 1.8702621 | up | mRNA |
| NM_001161587 | GYS1 | 0.630908827 | 0.950410442 | 1.0774911 | up | mRNA |
| NM_001161656 | MYB | 0.010564257 | 0.210159643 | 4.793836 | up | mRNA |
| NM_001161657 | MYB | 0.00169875 | 0.10523567 | 5.9155709 | up | mRNA |
| NM_001161659 | MYB | 0.002153206 | 0.110274545 | 5.5240667 | up | mRNA |
| NM_001161660 | MYB | 0.653394917 | 0.956860606 | 1.0515875 | up | mRNA |
| NM_001161661 | WWC1 | 0.913067492 | 0.995342392 | 1.0145514 | up | mRNA |
| NM_001161725 | PPP2R5C | 0.39538026 | 0.861590143 | 1.0959128 | up | mRNA |
| NM_001161726 | PPP2R5C | 0.011894127 | 0.221863669 | 1.1967515 | up | mRNA |
| NM_001161780 | PDP1 | 0.358658037 | 0.840603483 | 1.1197324 | up | mRNA |
| NM_001162498 | LPAR6 | 0.322975094 | 0.815844155 | 1.0848852 | up | mRNA |
| NM_001162499 | CAND2 | 0.326484502 | 0.819425644 | 1.1041704 | up | mRNA |
| NM_001162501 | TNRC6B | 0.138831619 | 0.598648178 | 1.2916429 | up | mRNA |
| NM_001162895 | KIAA0040 | 0.505024851 | 0.910568383 | 1.2553381 | up | mRNA |
| NM_001162916 | TYW3 | 0.771172288 | 0.975347132 | 1.0135194 | up | mRNA |
| NM_001162936 | CXorf68 | 0.323670827 | 0.816213518 | 1.2639197 | up | mRNA |
| NM_001163034 | RPTOR | 0.673262658 | 0.959531882 | 1.0202349 | up | mRNA |
| NM_001163120 | RDM1 | 0.656971544 | 0.957445617 | 1.0591942 | up | mRNA |
| NM_001163149 | ETV1 | 0.44431739 | 0.885088246 | 1.2448715 | up | mRNA |
| NM_001163258 | FAM63A | 0.007983227 | 0.195524479 | 1.2600967 | up | mRNA |
| NM_001163285 | EWSR1 | 0.7672901 | 0.975033576 | 1.0338804 | up | mRNA |
| NM_001163286 | EWSR1 | 0.44220793 | 0.88419144 | 1.1088587 | up | mRNA |
| NM_001163287 | EWSR1 | 0.676865837 | 0.959531882 | 1.0496697 | up | mRNA |
| NM_001163296 | MARK2 | 0.341334937 | 0.829649963 | 1.0840193 | up | mRNA |
| NM_001163297 | MARK2 | 0.054574184 | 0.419253535 | 1.0951852 | up | mRNA |
| NM_001163377 | QPCTL | 0.028749701 | 0.329689184 | 1.0986493 | up | mRNA |
| NM_001163418 | FAM172A | 0.542365275 | 0.923577434 | 1.0874727 | up | mRNA |
| NM_001163438 | CXorf69 | 0.604315486 | 0.94174007 | 1.1088538 | up | mRNA |
| NM_001163474 | ZNF746 | 0.092042048 | 0.511207518 | 1.19028 | up | mRNA |
| NM_001163499 | FBXO24 | 0.922747114 | 0.995349793 | 1.0123279 | up | mRNA |
| NM_001163678 | SHOX2 | 0.703289218 | 0.963625105 | 1.1696387 | up | mRNA |
| NM_001163724 | SMIM1 | 0.829923178 | 0.985549562 | 1.08595 | up | mRNA |
| NM_001163857 | TEX33 | 0.709094191 | 0.965462762 | 1.0821358 | up | mRNA |
| NM_001163926 | LRTM2 | 0.070828457 | 0.460237837 | 1.4401292 | up | mRNA |
| NM_001164095 | COPS7A | 0.512203597 | 0.91317044 | 1.0751344 | up | mRNA |
| NM_001164104 | MOV10L1 | 0.823453097 | 0.983498765 | 1.0597233 | up | mRNA |
| NM_001164105 | MOV10L1 | 0.056126745 | 0.422065984 | 1.4048914 | up | mRNA |
| NM_001164106 | MOV10L1 | 0.015352474 | 0.248644932 | 1.2693995 | up | mRNA |
| NM_001164115 | CASS4 | 0.867769022 | 0.989481868 | 1.0145932 | up | mRNA |
| NM_001164165 | GPR75-ASB3 | 0.863764597 | 0.989401142 | 1.0294071 | up | mRNA |
| NM_001164261 | PPIAL4D | 0.38828508 | 0.857737097 | 1.3305488 | up | mRNA |
| NM_001164278 | SLC37A4 | 9.45294E-05 | 0.036683999 | 2.7483562 | up | mRNA |
| NM_001164281 | TRAF3IP2 | 0.015781529 | 0.252653581 | 1.6341052 | up | mRNA |
| NM_001164283 | TRAF3IP2 | 0.000855141 | 0.083093876 | 1.6222877 | up | mRNA |
| NM_001164317 | FLNB | 0.911123707 | 0.995342392 | 1.0121706 | up | mRNA |
| NM_001164326 | BRD8 | 0.649893542 | 0.95550188 | 1.032596 | up | mRNA |
| NM_001164342 | ZBTB20 | 0.00496151 | 0.159869138 | 1.2555737 | up | mRNA |
| NM_001164372 | GPN3 | 0.504310013 | 0.910568383 | 1.0744178 | up | mRNA |
| NM_001164379 | FAM180B | 0.029478496 | 0.333953485 | 1.2434947 | up | mRNA |
| NM_001164380 | STAU2 | 0.51712605 | 0.915233884 | 1.164474 | up | mRNA |
| NM_001164386 | RAPGEF6 | 0.860991826 | 0.989013989 | 1.0254548 | up | mRNA |
| NM_001164388 | RAPGEF6 | 0.333163859 | 0.82335721 | 1.0384484 | up | mRNA |
| NM_001164404 | GOLGA6C | 0.109859068 | 0.548539924 | 1.1860552 | up | mRNA |
| NM_001164407 | TLCD2 | 0.973013669 | 0.998501532 | 1.008618 | up | mRNA |
| NM_001164444 | CBY3 | 0.239299542 | 0.737634672 | 1.2799174 | up | mRNA |
| NM_001164446 | C6orf132 | 0.035961834 | 0.356229271 | 1.301962 | up | mRNA |
| NM_001164468 | TMED7-TICAM2 | 0.103102751 | 0.532943442 | 1.1556194 | up | mRNA |
| NM_001164469 | TMED7-TICAM2 | 0.013472648 | 0.235533862 | 1.123107 | up | mRNA |
| NM_001164470 | MS4A12 | 0.259937826 | 0.760388386 | 1.1455671 | up | mRNA |
| NM_001164530 | ZNF135 | 0.08056957 | 0.484779682 | 1.217952 | up | mRNA |
| NM_001164541 | DISC1 | 0.421113359 | 0.872879684 | 1.1906926 | up | mRNA |
| NM_001164616 | FZD6 | 0.563083426 | 0.929041598 | 1.0759277 | up | mRNA |
| NM_001164623 | EIF2C2 | 0.15149747 | 0.616665978 | 1.1123251 | up | mRNA |
| NM_001164664 | MAST4 | 0.504913566 | 0.910568383 | 1.2492245 | up | mRNA |
| NM_001164691 | AMHR2 | 0.338146151 | 0.827403747 | 1.1340263 | up | mRNA |
| NM_001164694 | IYD | 0.363245117 | 0.843238468 | 1.1319319 | up | mRNA |
| NM_001164720 | CC2D2A | 0.746287217 | 0.970394407 | 1.0157859 | up | mRNA |
| NM_001164749 | NPAS3 | 0.412117968 | 0.869119647 | 1.081606 | up | mRNA |
| NM_001164771 | SLC7A2 | 0.392520085 | 0.859680391 | 1.3012459 | up | mRNA |
| NM_001164796 | SLC25A26 | 0.703953595 | 0.963625105 | 1.0710943 | up | mRNA |
| NM_001164829 | C1orf210 | 0.185264154 | 0.668759953 | 1.2897646 | up | mRNA |
| NM_001164841 | LYRM4 | 0.583252231 | 0.934269626 | 1.1392135 | up | mRNA |
| NM_001165412 | NFKB1 | 0.015818051 | 0.252840098 | 4.0507481 | up | mRNA |
| NM_001165415 | LDHA | 0.290616022 | 0.786596721 | 1.3948653 | up | mRNA |
| NM_001165416 | LDHA | 0.290288565 | 0.786468159 | 1.2083832 | up | mRNA |
| NM_001165418 | SLC25A11 | 0.398239491 | 0.863588452 | 1.0850992 | up | mRNA |
| NM_001165921 | SERPINF2 | 0.673560885 | 0.959531882 | 1.0607206 | up | mRNA |
| NM_001165927 | MKS1 | 0.298075658 | 0.794295449 | 1.2879575 | up | mRNA |
| NM_001165938 | STARD3 | 0.592915751 | 0.937853046 | 1.039983 | up | mRNA |
| NM_001165960 | ALOXE3 | 0.289640462 | 0.786034421 | 1.2243081 | up | mRNA |
| NM_001165975 | PDE1B | 0.011096444 | 0.216104302 | 1.9658519 | up | mRNA |
| NM_001166 | BIRC2 | 0.026489274 | 0.318338323 | 1.8104868 | up | mRNA |
| NM_001166006 | EPB41 | 0.448264891 | 0.887969953 | 1.1754594 | up | mRNA |
| NM_001166034 | SBSN | 0.48306269 | 0.902654749 | 1.4148348 | up | mRNA |
| NM_001166060 | GLRB | 0.560483532 | 0.92782421 | 1.1789955 | up | mRNA |
| NM_001166108 | PALLD | 0.978129665 | 0.998641374 | 1.0089543 | up | mRNA |
| NM_001166109 | PALLD | 0.927581633 | 0.995829678 | 1.02339 | up | mRNA |
| NM_001166119 | LEF1 | 0.602978411 | 0.941464987 | 1.0645042 | up | mRNA |
| NM_001166160 | PPP1R9A | 0.140681913 | 0.600786526 | 1.2814531 | up | mRNA |
| NM_001166175 | NKX2-5 | 0.179825901 | 0.662598807 | 1.1136973 | up | mRNA |
| NM_001166209 | SYNPO | 0.984032386 | 0.998971807 | 1.0045684 | up | mRNA |
| NM_001166212 | CLCF1 | 0.042403133 | 0.380635986 | 1.3636093 | up | mRNA |
| NM_001166226 | CEP120 | 0.708420017 | 0.965462762 | 1.0250819 | up | mRNA |
| NM_001166237 | GSDMD | 0.803777394 | 0.98118927 | 1.0148669 | up | mRNA |
| NM_001166247 | GRIK2 | 0.590720641 | 0.936566755 | 1.1657162 | up | mRNA |
| NM_001166252 | LPPR4 | 0.982216491 | 0.998971807 | 1.002843 | up | mRNA |
| NM_001166270 | HAUS4 | 0.773701564 | 0.97589756 | 1.0187203 | up | mRNA |
| NM_001166276 | ARHGAP25 | 0.729119608 | 0.966191059 | 1.0447927 | up | mRNA |
| NM_001166279 | PAN2 | 0.635285732 | 0.951501252 | 1.0268366 | up | mRNA |
| NM_001166290 | KCNJ11 | 0.445773421 | 0.886229119 | 1.0242449 | up | mRNA |
| NM_001166306 | TMEM44 | 0.8207973 | 0.982756843 | 1.0700318 | up | mRNA |
| NM_001166345 | MDFIC | 0.997976477 | 0.999889342 | 1.000302 | up | mRNA |
| NM_001166373 | MARCH1 | 0.349462254 | 0.834138149 | 1.1175089 | up | mRNA |
| NM_001166392 | TPBG | 0.13583926 | 0.594011904 | 1.3636339 | up | mRNA |
| NM_001166395 | CHST4 | 0.636091824 | 0.951827088 | 1.0532751 | up | mRNA |
| NM_001166420 | HDAC8 | 0.775630963 | 0.97589756 | 1.0143736 | up | mRNA |
| NM_001166422 | HDAC8 | 0.367306748 | 0.845181673 | 1.0870174 | up | mRNA |
| NM_001166436 | ITIH1 | 0.360377801 | 0.841780454 | 1.100991 | up | mRNA |
| NM_001166449 | ITIH4 | 0.709330196 | 0.965462762 | 1.0454439 | up | mRNA |
| NM_001166496 | SLC16A1 | 0.2321033 | 0.730050958 | 1.1778783 | up | mRNA |
| NM_001166498 | HPSE | 0.99453428 | 0.999872237 | 1.0016374 | up | mRNA |
| NM_001166624 | CFHR3 | 0.487638311 | 0.903757389 | 1.0610731 | up | mRNA |
| NM_001166663 | CD244 | 0.204436634 | 0.694503867 | 1.2961926 | up | mRNA |
| NM_001166692 | C11orf91 | 0.58248887 | 0.934005566 | 1.1179367 | up | mRNA |
| NM_001166695 | SLC1A3 | 0.016086056 | 0.254919482 | 2.7245335 | up | mRNA |
| NM_001166698 | FAM13C | 0.671372098 | 0.959531882 | 1.0498167 | up | mRNA |
| NM_001166702 | FAM104B | 0.038037348 | 0.364284204 | 1.3772912 | up | mRNA |
| NM_001166703 | FAM104B | 0.942639947 | 0.996710991 | 1.0084758 | up | mRNA |
| NM_001166704 | FAM104B | 0.751704155 | 0.970725371 | 1.0396675 | up | mRNA |
| NM_001167 | XIAP | 0.602945502 | 0.941464987 | 1.1029803 | up | mRNA |
| NM_001167574 | UPK3A | 0.55434444 | 0.926549129 | 1.2013325 | up | mRNA |
| NM_001167580 | SV2B | 0.581109647 | 0.934005566 | 1.0547366 | up | mRNA |
| NM_001167599 | NEU4 | 0.800600356 | 0.98118927 | 1.0166321 | up | mRNA |
| NM_001167602 | NEU4 | 0.451421714 | 0.888854908 | 1.1158471 | up | mRNA |
| NM_001167606 | RAB35 | 0.461182454 | 0.89460367 | 1.0455393 | up | mRNA |
| NM_001167609 | GLI1 | 0.207047676 | 0.698556218 | 1.1988842 | up | mRNA |
| NM_001167667 | CDH18 | 0.175536291 | 0.653962789 | 1.4539932 | up | mRNA |
| NM_001167670 | TMEM239 | 0.01359157 | 0.235988767 | 1.3918862 | up | mRNA |
| NM_001167676 | LOC100128071 | 0.329493318 | 0.821768501 | 1.0919146 | up | mRNA |
| NM_001167681 | FLI1 | 0.042820797 | 0.382359436 | 1.1991271 | up | mRNA |
| NM_001167733 | VARS2 | 0.99269315 | 0.999798141 | 1.0012691 | up | mRNA |
| NM_001167738 | NAV1 | 0.569157699 | 0.93232103 | 1.07193 | up | mRNA |
| NM_001167820 | BLCAP | 0.962764484 | 0.99793927 | 1.0027596 | up | mRNA |
| NM_001167830 | TMED5 | 0.262301205 | 0.763125731 | 1.2071687 | up | mRNA |
| NM_001167871 | ATP5SL | 0.751650007 | 0.970725371 | 1.0193188 | up | mRNA |
| NM_001167916 | VEPH1 | 0.017634796 | 0.267375595 | 1.402075 | up | mRNA |
| NM_001167931 | IL1RAP | 0.039280594 | 0.369575679 | 1.2507647 | up | mRNA |
| NM_001167987 | INCA1 | 0.633288133 | 0.951174475 | 1.0841505 | up | mRNA |
| NM_001168217 | ALS2CR11 | 0.856041456 | 0.988315074 | 1.0480587 | up | mRNA |
| NM_001168222 | TBC1D17 | 0.406573519 | 0.867413724 | 1.0609979 | up | mRNA |
| NM_001168236 | ABL2 | 0.86418408 | 0.989401142 | 1.0373596 | up | mRNA |
| NM_001168237 | ABL2 | 0.838084485 | 0.986897498 | 1.0402846 | up | mRNA |
| NM_001168238 | ABL2 | 0.399096211 | 0.863643939 | 1.0803019 | up | mRNA |
| NM_001168239 | ABL2 | 0.624579474 | 0.948341211 | 1.0934071 | up | mRNA |
| NM_001168271 | GPR156 | 0.143696063 | 0.604141513 | 1.2439738 | up | mRNA |
| NM_001168280 | WWTR1 | 0.567016279 | 0.931483408 | 1.0303915 | up | mRNA |
| NM_001168298 | CXCR2 | 0.12477329 | 0.578441772 | 1.1322266 | up | mRNA |
| NM_001168301 | KLHL13 | 0.423080352 | 0.873989281 | 1.1782811 | up | mRNA |
| NM_001168303 | KLHL13 | 0.986168777 | 0.998971807 | 1.0119649 | up | mRNA |
| NM_001168338 | PLG | 0.586938626 | 0.93579682 | 1.0552823 | up | mRNA |
| NM_001168341 | TTC39B | 0.574527897 | 0.933656777 | 1.2759142 | up | mRNA |
| NM_001168342 | TTC39B | 0.755625406 | 0.971497464 | 1.0723141 | up | mRNA |
| NM_001168361 | PCDH11X | 0.899227252 | 0.994575817 | 1.0131856 | up | mRNA |
| NM_001168363 | PCDH11X | 0.124341538 | 0.578369571 | 1.1286007 | up | mRNA |
| NM_001168368 | GALNTL1 | 0.149608417 | 0.614016619 | 1.088309 | up | mRNA |
| NM_001168370 | CUL7 | 0.725611818 | 0.966191059 | 1.0493772 | up | mRNA |
| NM_001168409 | RIMS1 | 0.95433671 | 0.996710991 | 1.039193 | up | mRNA |
| NM_001168468 | PDZD3 | 0.120362017 | 0.571550658 | 1.2707981 | up | mRNA |
| NM_001168648 | CNKSR2 | 0.828520181 | 0.985493382 | 1.0633059 | up | mRNA |
| NM_001168682 | SAP25 | 0.941307941 | 0.996710991 | 1.0068034 | up | mRNA |
| NM_001169 | AQP8 | 0.532256569 | 0.920302794 | 1.1378789 | up | mRNA |
| NM_001169107 | FAM21C | 0.542150664 | 0.923577434 | 1.0489955 | up | mRNA |
| NM_001170402 | CDC20B | 0.944735911 | 0.996710991 | 1.0166249 | up | mRNA |
| NM_001170414 | TJP2 | 0.065188481 | 0.445966419 | 1.6499272 | up | mRNA |
| NM_001170418 | MOG | 0.082806231 | 0.490850228 | 1.0994069 | up | mRNA |
| NM_001170569 | CXorf56 | 0.323471564 | 0.816213518 | 1.12802 | up | mRNA |
| NM_001170630 | TJP2 | 0.355577631 | 0.838390774 | 1.233934 | up | mRNA |
| NM_001170692 | CAGE1 | 0.184162455 | 0.667687419 | 2.2362647 | up | mRNA |
| NM_001170716 | BCAR1 | 0.986711015 | 0.99909491 | 1.0015116 | up | mRNA |
| NM_001170738 | IQSEC3 | 0.803668677 | 0.98118927 | 1.0447371 | up | mRNA |
| NM_001170750 | SRPX | 0.348908138 | 0.834138149 | 1.04911 | up | mRNA |
| NM_001170760 | SRPK3 | 0.927026717 | 0.995829678 | 1.0120487 | up | mRNA |
| NM_001170792 | FAM82A1 | 0.574483217 | 0.933656777 | 1.1168205 | up | mRNA |
| NM_001170804 | LARP4 | 0.815740246 | 0.981459009 | 1.0201114 | up | mRNA |
| NM_001170808 | LARP4 | 0.285160299 | 0.781963593 | 1.0610007 | up | mRNA |
| NM_001171088 | CLCN2 | 0.55374293 | 0.926337111 | 1.0981094 | up | mRNA |
| NM_001171133 | FAM3A | 0.423769999 | 0.874798629 | 1.1923321 | up | mRNA |
| NM_001171155 | PET100 | 0.155476629 | 0.623056705 | 1.1083118 | up | mRNA |
| NM_001171161 | SIGLEC10 | 0.622144261 | 0.948131965 | 1.0657724 | up | mRNA |
| NM_001171167 | CAMTA2 | 0.638084846 | 0.952852353 | 1.0624756 | up | mRNA |
| NM_001171174 | CX3CR1 | 0.542122639 | 0.923577434 | 1.1784943 | up | mRNA |
| NM_001171191 | GDPD2 | 0.880055585 | 0.992197209 | 1.0156641 | up | mRNA |
| NM_001171507 | MCFD2 | 0.051836125 | 0.41031217 | 1.0856052 | up | mRNA |
| NM_001171510 | MCFD2 | 0.961963038 | 0.99793927 | 1.0034388 | up | mRNA |
| NM_001171622 | VEGFA | 0.031691683 | 0.341807319 | 1.4791966 | up | mRNA |
| NM_001171624 | VEGFA | 0.013737751 | 0.236215185 | 1.6260258 | up | mRNA |
| NM_001171653 | ZEB2 | 0.031685903 | 0.341807319 | 1.2916409 | up | mRNA |
| NM_001171654 | KLHL5 | 0.600366889 | 0.940648177 | 1.1304264 | up | mRNA |
| NM_001171660 | CYB5R3 | 0.262188303 | 0.763125731 | 1.1701138 | up | mRNA |
| NM_001171815 | RNF166 | 0.148896115 | 0.61263618 | 1.143754 | up | mRNA |
| NM_001171816 | RNF166 | 0.316848887 | 0.811663295 | 1.2892226 | up | mRNA |
| NM_001171818 | PPARD | 0.006832372 | 0.183994427 | 1.2810839 | up | mRNA |
| NM_001171878 | MCF2 | 0.071772731 | 0.462237305 | 1.1534683 | up | mRNA |
| NM_001171906 | NUDT16 | 0.435457258 | 0.881627543 | 1.0711758 | up | mRNA |
| NM_001171909 | CXorf40A | 0.703983626 | 0.963625105 | 1.0412249 | up | mRNA |
| NM_001171933 | CDH23 | 0.473117167 | 0.899184677 | 1.0829828 | up | mRNA |
| NM_001171946 | SUN1 | 0.202813662 | 0.692118849 | 1.1421654 | up | mRNA |
| NM_001171990 | SETD9 | 0.488112787 | 0.903757389 | 1.1057454 | up | mRNA |
| NM_001171993 | HPD | 0.660049279 | 0.958354006 | 1.0546949 | up | mRNA |
| NM_001172 | ARG2 | 0.937759248 | 0.996710991 | 1.0388157 | up | mRNA |
| NM_001172132 | HCK | 0.090785863 | 0.508828962 | 1.6762801 | up | mRNA |
| NM_001172219 | SCMH1 | 0.850604093 | 0.987678166 | 1.0155285 | up | mRNA |
| NM_001172220 | SCMH1 | 0.763880554 | 0.973773636 | 1.0151768 | up | mRNA |
| NM_001172222 | SCMH1 | 0.57517742 | 0.933656777 | 1.0481389 | up | mRNA |
| NM_001172303 | MASTL | 0.394326502 | 0.86105621 | 1.3216643 | up | mRNA |
| NM_001172304 | MASTL | 0.343109731 | 0.831521302 | 1.4380355 | up | mRNA |
| NM_001172418 | BTBD9 | 0.970046621 | 0.998245244 | 1.0063294 | up | mRNA |
| NM_001172428 | KLHL7 | 0.129017474 | 0.582929618 | 1.2324579 | up | mRNA |
| NM_001172430 | AMPD3 | 0.006627326 | 0.183552613 | 3.8242245 | up | mRNA |
| NM_001172436 | PHKA1 | 0.953362342 | 0.996710991 | 1.0146655 | up | mRNA |
| NM_001172440 | ENDOU | 0.939809923 | 0.996710991 | 1.0039466 | up | mRNA |
| NM_001172566 | MYD88 | 0.664946511 | 0.958354006 | 1.0284075 | up | mRNA |
| NM_001172567 | MYD88 | 0.555915265 | 0.926833616 | 1.1188673 | up | mRNA |
| NM_001172623 | NEO1 | 0.139365567 | 0.598648178 | 1.1378076 | up | mRNA |
| NM_001172639 | CELF1 | 0.4519838 | 0.888854908 | 1.0776745 | up | mRNA |
| NM_001172640 | CELF1 | 0.207450693 | 0.698556218 | 1.0762161 | up | mRNA |
| NM_001172673 | CELF5 | 0.790203131 | 0.978438986 | 1.0442205 | up | mRNA |
| NM_001172684 | CELF6 | 0.674888005 | 0.959531882 | 1.070642 | up | mRNA |
| NM_001172685 | CELF6 | 0.894166255 | 0.993978977 | 1.0216512 | up | mRNA |
| NM_001172696 | TSFM | 0.523527524 | 0.917239107 | 1.0843419 | up | mRNA |
| NM_001172779 | LRRC34 | 0.866276465 | 0.989481868 | 1.0155373 | up | mRNA |
| NM_001172895 | CAV1 | 0.442070636 | 0.88419144 | 1.1692143 | up | mRNA |
| NM_001173454 | PDHA1 | 0.634805884 | 0.951279916 | 1.0105725 | up | mRNA |
| NM_001173464 | KIF21A | 0.175373447 | 0.653962789 | 1.1654387 | up | mRNA |
| NM_001173466 | AAAS | 0.810515908 | 0.98118927 | 1.0280601 | up | mRNA |
| NM_001173486 | PRRG1 | 0.405375115 | 0.866165103 | 1.1017148 | up | mRNA |
| NM_001173490 | PRRG1 | 0.200677287 | 0.68900633 | 1.2999477 | up | mRNA |
| NM_001173514 | TYROBP | 0.163280524 | 0.635463018 | 1.2923391 | up | mRNA |
| NM_001173525 | TROVE2 | 0.548648663 | 0.92514258 | 1.1511248 | up | mRNA |
| NM_001173541 | BANP | 0.707109715 | 0.965401045 | 1.0438864 | up | mRNA |
| NM_001173542 | BANP | 0.848648714 | 0.987678166 | 1.0297718 | up | mRNA |
| NM_001173989 | C9orf86 | 0.875893796 | 0.991187881 | 1.0083763 | up | mRNA |
| NM_001174063 | FGFR1 | 0.690431545 | 0.961105466 | 1.0252318 | up | mRNA |
| NM_001174064 | FGFR1 | 0.883863454 | 0.992766819 | 1.0394119 | up | mRNA |
| NM_001174068 | SYTL4 | 0.000871232 | 0.083163758 | 2.018054 | up | mRNA |
| NM_001174082 | NLRP2 | 0.776631308 | 0.976577486 | 1.1283624 | up | mRNA |
| NM_001174117 | DMXL2 | 0.628889618 | 0.950128593 | 1.1328727 | up | mRNA |
| NM_001174120 | ZFYVE27 | 0.116612543 | 0.562996237 | 1.1397214 | up | mRNA |
| NM_001174121 | ZFYVE27 | 0.718046647 | 0.966191059 | 1.0275176 | up | mRNA |
| NM_001174124 | GOLGA7 | 0.924948808 | 0.995456159 | 1.0103214 | up | mRNA |
| NM_001174127 | SLC11A2 | 0.386697166 | 0.856062164 | 1.1722311 | up | mRNA |
| NM_001177314 | RRAS2 | 0.68040951 | 0.960111041 | 1.0444668 | up | mRNA |
| NM_001177383 | CPEB2 | 0.014195152 | 0.240112996 | 1.628751 | up | mRNA |
| NM_001177384 | CPEB2 | 0.007361318 | 0.190938402 | 1.4370676 | up | mRNA |
| NM_001177387 | ATXN7 | 0.159810406 | 0.63153842 | 1.0831344 | up | mRNA |
| NM_001177428 | TRPV4 | 0.679861454 | 0.960007989 | 1.0621251 | up | mRNA |
| NM_001177431 | TRPV4 | 0.368165828 | 0.845312226 | 1.0672966 | up | mRNA |
| NM_001177465 | MAMLD1 | 0.004150591 | 0.147175197 | 1.3274625 | up | mRNA |
| NM_001177515 | ABHD16A | 0.156974937 | 0.625751326 | 1.0769978 | up | mRNA |
| NM_001177520 | ALPL | 0.686831802 | 0.960205549 | 1.2106551 | up | mRNA |
| NM_001177548 | SIGLEC6 | 0.329928184 | 0.821768501 | 1.0613898 | up | mRNA |
| NM_001177562 | PPP2R1B | 0.751387176 | 0.970725371 | 1.0300802 | up | mRNA |
| NM_001177563 | PPP2R1B | 0.11776632 | 0.564437853 | 1.1362212 | up | mRNA |
| NM_001177660 | HABP2 | 0.917896088 | 0.995349793 | 1.0097074 | up | mRNA |
| NM_001177665 | RIF1 | 0.633944782 | 0.951174475 | 1.0326751 | up | mRNA |
| NM_001177675 | PNPLA5 | 0.523614654 | 0.917239107 | 1.0495821 | up | mRNA |
| NM_001177701 | IFT27 | 0.080566686 | 0.484779682 | 1.1168405 | up | mRNA |
| NM_001177704 | FREM1 | 0.460065858 | 0.89324475 | 1.2359389 | up | mRNA |
| NM_001177711 | RBMS3 | 0.002875519 | 0.12765297 | 1.176955 | up | mRNA |
| NM_001177842 | SRPR | 0.560537267 | 0.92782421 | 1.0519856 | up | mRNA |
| NM_001177972 | VIT | 0.460576962 | 0.893982093 | 1.1788785 | up | mRNA |
| NM_001178010 | CDC45 | 0.395584636 | 0.861590143 | 1.1669375 | up | mRNA |
| NM_001178011 | CDC45 | 0.879280742 | 0.992149178 | 1.0795042 | up | mRNA |
| NM_001178017 | DBI | 0.023523792 | 0.301884214 | 2.3743781 | up | mRNA |
| NM_001178020 | BEAN1 | 0.609455833 | 0.943053241 | 1.1081819 | up | mRNA |
| NM_001178031 | SF1 | 0.785665372 | 0.977479172 | 1.0142853 | up | mRNA |
| NM_001178033 | ZC4H2 | 0.957512119 | 0.996883425 | 1.010937 | up | mRNA |
| NM_001178041 | DBI | 0.129001503 | 0.582929618 | 1.9626691 | up | mRNA |
| NM_001178042 | DBI | 0.171961886 | 0.649834644 | 1.1449652 | up | mRNA |
| NM_001178044 | SLC44A4 | 0.410635083 | 0.868335658 | 1.0524713 | up | mRNA |
| NM_001178054 | TAC3 | 0.12767403 | 0.580991132 | 1.5750704 | up | mRNA |
| NM_001178055 | PARP8 | 0.595566444 | 0.938746918 | 1.2037067 | up | mRNA |
| NM_001178057 | ACE | 0.68671071 | 0.960205549 | 1.3147513 | up | mRNA |
| NM_001178065 | CASR | 0.986153185 | 0.998971807 | 1.0021066 | up | mRNA |
| NM_001178080 | STAT6 | 0.200861216 | 0.68900633 | 1.0737557 | up | mRNA |
| NM_001178091 | BCAT1 | 0.978009467 | 0.998641374 | 1.0017511 | up | mRNA |
| NM_001178092 | BCAT1 | 0.966367893 | 0.99793927 | 1.003785 | up | mRNA |
| NM_001178093 | BCAT1 | 0.821255409 | 0.982756843 | 1.0395352 | up | mRNA |
| NM_001178094 | BCAT1 | 0.390804253 | 0.858920947 | 1.0787616 | up | mRNA |
| NM_001178096 | F3 | 0.577325471 | 0.933656777 | 1.5369766 | up | mRNA |
| NM_001178097 | C12orf74 | 0.304120388 | 0.799856028 | 1.0627579 | up | mRNA |
| NM_001178108 | ZNF185 | 0.537429049 | 0.922702335 | 1.0546478 | up | mRNA |
| NM_001178110 | ZNF185 | 0.823919996 | 0.983568283 | 1.0184931 | up | mRNA |
| NM_001178117 | MINPP1 | 0.001992001 | 0.107649983 | 1.2262456 | up | mRNA |
| NM_001178118 | MINPP1 | 0.133246147 | 0.589162633 | 1.7425331 | up | mRNA |
| NM_001178119 | ABI1 | 0.260989266 | 0.761652614 | 1.190224 | up | mRNA |
| NM_001178120 | ABI1 | 0.178657945 | 0.65979922 | 1.1372183 | up | mRNA |
| NM_001178121 | ABI1 | 0.057889563 | 0.428369209 | 1.2175798 | up | mRNA |
| NM_001178129 | SEMA3E | 0.717781106 | 0.966191059 | 1.0180958 | up | mRNA |
| NM_001178130 | EGF | 0.805009396 | 0.98118927 | 1.0348873 | up | mRNA |
| NM_001178138 | TFDP2 | 0.367638417 | 0.845181673 | 1.0647303 | up | mRNA |
| NM_001178142 | TFDP2 | 0.883740853 | 0.992766819 | 1.0089351 | up | mRNA |
| NM_001179 | ART3 | 0.010467244 | 0.209736088 | 1.3049425 | up | mRNA |
| NM_001182 | ALDH7A1 | 0.770672207 | 0.975347132 | 1.0352118 | up | mRNA |
| NM_001184691 | NPNT | 0.436088628 | 0.881677506 | 1.0872499 | up | mRNA |
| NM_001184693 | NPNT | 0.306072803 | 0.801520161 | 3.8026953 | up | mRNA |
| NM_001184697 | MEPE | 0.054811611 | 0.419413793 | 1.8429242 | up | mRNA |
| NM_001184703 | GYG2 | 0.023331838 | 0.300052453 | 1.0955393 | up | mRNA |
| NM_001184726 | TCN2 | 0.769975008 | 0.975286259 | 1.0191018 | up | mRNA |
| NM_001184729 | PIGT | 0.897994317 | 0.994326043 | 1.0089958 | up | mRNA |
| NM_001184733 | SLC22A8 | 0.05743947 | 0.42700171 | 1.283613 | up | mRNA |
| NM_001184735 | ASTN2 | 0.952937895 | 0.996710991 | 1.0034416 | up | mRNA |
| NM_001184744 | PTH1R | 0.735229729 | 0.96794858 | 1.0424822 | up | mRNA |
| NM_001184745 | PPP1R3F | 0.704943104 | 0.964529151 | 1.119275 | up | mRNA |
| NM_001184747 | PAFAH1B2 | 0.487034093 | 0.903621334 | 1.23567 | up | mRNA |
| NM_001184748 | PAFAH1B2 | 0.38317624 | 0.853514109 | 1.1392713 | up | mRNA |
| NM_001184760 | CLTA | 0.982406788 | 0.998971807 | 1.0015787 | up | mRNA |
| NM_001184771 | CPXCR1 | 0.548297418 | 0.92514258 | 1.2959745 | up | mRNA |
| NM_001184773 | SEZ6L | 0.319080117 | 0.812974553 | 1.1925743 | up | mRNA |
| NM_001184774 | SEZ6L | 0.924438329 | 0.995456159 | 1.0111661 | up | mRNA |
| NM_001184776 | SEZ6L | 0.960310749 | 0.997738713 | 1.0088073 | up | mRNA |
| NM_001184794 | PARD3 | 0.787279649 | 0.977479172 | 1.0255161 | up | mRNA |
| NM_001184823 | VDAC2 | 0.89006616 | 0.9937521 | 1.0289769 | up | mRNA |
| NM_001184830 | VSIG4 | 0.587771466 | 0.93579682 | 1.114308 | up | mRNA |
| NM_001184836 | GPR64 | 0.404316542 | 0.865617032 | 1.0669903 | up | mRNA |
| NM_001184837 | GPR64 | 0.851076185 | 0.987678166 | 1.0256898 | up | mRNA |
| NM_001184854 | KLHDC4 | 0.960424961 | 0.997738713 | 1.0085903 | up | mRNA |
| NM_001184866 | FCRLA | 0.885582711 | 0.992981836 | 1.0295473 | up | mRNA |
| NM_001184867 | FCRLA | 0.445434146 | 0.885807324 | 1.1395391 | up | mRNA |
| NM_001184870 | FCRLA | 0.47010132 | 0.898057982 | 1.26884 | up | mRNA |
| NM_001184871 | FCRLA | 0.160423291 | 0.631980676 | 1.0636087 | up | mRNA |
| NM_001184873 | FCRLA | 0.905380618 | 0.99508468 | 1.0099559 | up | mRNA |
| NM_001184879 | CD84 | 0.390853508 | 0.858920947 | 1.1302042 | up | mRNA |
| NM_001184881 | CD84 | 0.790115257 | 0.978438986 | 1.0304704 | up | mRNA |
| NM_001184882 | CD84 | 0.880822662 | 0.992287164 | 1.1108214 | up | mRNA |
| NM_001184883 | PLCB3 | 0.928651311 | 0.996166427 | 1.0205379 | up | mRNA |
| NM_001184897 | PHF8 | 0.591776239 | 0.937303262 | 1.0373757 | up | mRNA |
| NM_001184940 | FAM219A | 0.409408027 | 0.867634354 | 1.0931721 | up | mRNA |
| NM_001184976 | NCKAP1L | 0.561263271 | 0.928452106 | 1.163951 | up | mRNA |
| NM_001184977 | UQCC | 0.974594772 | 0.998501532 | 1.0024322 | up | mRNA |
| NM_001184996 | RNF32 | 0.490388742 | 0.905264563 | 1.0991942 | up | mRNA |
| NM_001185 | AZGP1 | 0.608851412 | 0.943053241 | 1.0203865 | up | mRNA |
| NM_001185011 | NCAPH2 | 0.996156426 | 0.999872237 | 1.0012757 | up | mRNA |
| NM_001185039 | ACOX1 | 0.002476833 | 0.118591515 | 2.0673791 | up | mRNA |
| NM_001185054 | ADD2 | 0.072011323 | 0.462605549 | 1.3286693 | up | mRNA |
| NM_001185059 | ZCCHC6 | 0.117640659 | 0.564253331 | 1.2020995 | up | mRNA |
| NM_001185061 | AQP1 | 0.02841521 | 0.327830694 | 1.2142038 | up | mRNA |
| NM_001185062 | AQP1 | 0.317236397 | 0.811824899 | 1.0942976 | up | mRNA |
| NM_001185077 | ARHGDIA | 0.288615029 | 0.784514546 | 1.1686051 | up | mRNA |
| NM_001185085 | ATP12A | 0.455641031 | 0.891148383 | 1.1428768 | up | mRNA |
| NM_001185090 | GRIN1 | 0.006247036 | 0.179020559 | 1.4536052 | up | mRNA |
| NM_001185092 | NIT1 | 0.29513297 | 0.791537205 | 1.1340176 | up | mRNA |
| NM_001185093 | NIT1 | 0.809435247 | 0.98118927 | 1.0247626 | up | mRNA |
| NM_001185099 | CD22 | 0.763361696 | 0.973567683 | 1.0435824 | up | mRNA |
| NM_001185106 | PLCXD2 | 0.804014788 | 0.98118927 | 1.0250675 | up | mRNA |
| NM_001185107 | CD1E | 0.07389983 | 0.468039773 | 1.1813504 | up | mRNA |
| NM_001185113 | CD1E | 0.978546185 | 0.998661138 | 1.0026078 | up | mRNA |
| NM_001185176 | CES3 | 0.86737353 | 0.989481868 | 1.0201624 | up | mRNA |
| NM_001185177 | CES3 | 0.73768786 | 0.969031501 | 1.0721167 | up | mRNA |
| NM_001189 | NKX3-2 | 0.728050424 | 0.966191059 | 1.086024 | up | mRNA |
| NM_001190 | BCAT2 | 0.713103533 | 0.966110889 | 1.0485272 | up | mRNA |
| NM_001190201 | CES4A | 0.538660125 | 0.923577434 | 1.0709427 | up | mRNA |
| NM_001190316 | IP6K2 | 0.564030428 | 0.929400006 | 1.0351133 | up | mRNA |
| NM_001190317 | IP6K2 | 0.168490228 | 0.643936713 | 1.3267263 | up | mRNA |
| NM_001190326 | DGCR8 | 0.395730161 | 0.861639069 | 1.0519333 | up | mRNA |
| NM_001190349 | DMKN | 0.633728301 | 0.951174475 | 1.1201889 | up | mRNA |
| NM_001190415 | TAF6 | 0.84622628 | 0.987401591 | 1.0244221 | up | mRNA |
| NM_001190455 | CHRNA7 | 0.492450594 | 0.906047853 | 1.070195 | up | mRNA |
| NM_001190457 | CORO2B | 0.868398355 | 0.989481868 | 1.0164739 | up | mRNA |
| NM_001190462 | LOC643037 | 0.259128367 | 0.759601783 | 1.2173493 | up | mRNA |
| NM_001190470 | MTRNR2L2 | 0.488254001 | 0.903785537 | 1.0402539 | up | mRNA |
| NM_001190478 | MTRNR2L5 | 0.245665291 | 0.743916784 | 1.2427584 | up | mRNA |
| NM_001190481 | CLSPN | 0.950733867 | 0.996710991 | 1.0468919 | up | mRNA |
| NM_001190720 | IKBKB | 0.009475396 | 0.204959264 | 2.2765793 | up | mRNA |
| NM_001190726 | RERG | 0.401108479 | 0.864768873 | 1.5620477 | up | mRNA |
| NM_001190728 | RALGPS1 | 0.314835109 | 0.809871892 | 1.1602227 | up | mRNA |
| NM_001190730 | RALGPS1 | 0.938263364 | 0.996710991 | 1.0131477 | up | mRNA |
| NM_001190737 | NFIB | 0.807415583 | 0.98118927 | 1.083822 | up | mRNA |
| NM_001190764 | TMEM238 | 0.7582689 | 0.972072638 | 1.0314241 | up | mRNA |
| NM_001190787 | MCIN | 0.274163377 | 0.774314458 | 1.1491906 | up | mRNA |
| NM_001190789 | NCF2 | 0.952332686 | 0.996710991 | 1.0043222 | up | mRNA |
| NM_001190791 | ZNF317 | 0.925002618 | 0.995456159 | 1.00786 | up | mRNA |
| NM_001190807 | CYB5A | 0.010315847 | 0.209736088 | 1.236921 | up | mRNA |
| NM_001190811 | DAZL | 0.886121652 | 0.992981836 | 1.0343179 | up | mRNA |
| NM_001190818 | ORC1 | 0.889203049 | 0.993561109 | 1.0281928 | up | mRNA |
| NM_001190848 | CNOT4 | 0.521427978 | 0.916644975 | 1.2137363 | up | mRNA |
| NM_001190919 | THRA | 0.927383944 | 0.995829678 | 1.0238138 | up | mRNA |
| NM_001190972 | LOC100127983 | 0.892003532 | 0.99376525 | 1.0065448 | up | mRNA |
| NM_001190977 | YAF2 | 0.362469974 | 0.843173209 | 1.0392299 | up | mRNA |
| NM_001190981 | IL6ST | 0.019972977 | 0.283521851 | 1.1938167 | up | mRNA |
| NM_001190986 | CRISP3 | 0.350190456 | 0.834344847 | 1.1126186 | up | mRNA |
| NM_001191003 | GSTO1 | 0.866701612 | 0.989481868 | 1.0162044 | up | mRNA |
| NM_001191016 | CASP12 | 0.146273828 | 0.608122555 | 1.333777 | up | mRNA |
| NM_001191049 | SEC31A | 0.822327653 | 0.983107122 | 1.0150068 | up | mRNA |
| NM_001191323 | GREM1 | 0.537217173 | 0.922702335 | 1.161703 | up | mRNA |
| NM_001193270 | MSL3 | 0.237208387 | 0.735183924 | 1.3222992 | up | mRNA |
| NM_001193283 | LEKR1 | 0.607600848 | 0.942627675 | 1.0647292 | up | mRNA |
| NM_001193301 | SEMA4A | 0.708916609 | 0.965462762 | 1.0330103 | up | mRNA |
| NM_001193302 | SEMA4A | 0.827742016 | 0.985006148 | 1.0358461 | up | mRNA |
| NM_001193304 | TMEM127 | 0.902867626 | 0.99508468 | 1.0122438 | up | mRNA |
| NM_001193307 | SAMD9 | 0.751428299 | 0.970725371 | 1.0664701 | up | mRNA |
| NM_001193311 | C7orf10 | 0.236205911 | 0.734460585 | 1.2196881 | up | mRNA |
| NM_001193329 | C9orf3 | 0.528640495 | 0.918883792 | 1.1023962 | up | mRNA |
| NM_001193331 | C9orf3 | 0.187401338 | 0.671419231 | 1.2705097 | up | mRNA |
| NM_001193335 | ASPN | 0.592151481 | 0.937519382 | 1.1007778 | up | mRNA |
| NM_001193338 | FAIM3 | 0.886132462 | 0.992981836 | 1.0240567 | up | mRNA |
| NM_001193339 | SLC13A3 | 0.744225717 | 0.970330526 | 1.0452905 | up | mRNA |
| NM_001193375 | NDUFA11 | 0.471377055 | 0.898908705 | 1.4026775 | up | mRNA |
| NM_001193376 | TERT | 0.918670741 | 0.995349793 | 1.0097737 | up | mRNA |
| NM_001193379 | UCKL1 | 0.124719123 | 0.578441772 | 1.1133639 | up | mRNA |
| NM_001193388 | UNKL | 0.80805443 | 0.98118927 | 1.0439971 | up | mRNA |
| NM_001193389 | UNKL | 0.582081377 | 0.934005566 | 1.2302916 | up | mRNA |
| NM_001193414 | TUBA8 | 0.922460559 | 0.995349793 | 1.0194432 | up | mRNA |
| NM_001193417 | DDX3X | 0.605982107 | 0.94174007 | 1.0252603 | up | mRNA |
| NM_001193424 | SUV39H2 | 0.099943662 | 0.525350189 | 1.101972 | up | mRNA |
| NM_001193460 | MSRB3 | 0.634792543 | 0.951279916 | 1.0665737 | up | mRNA |
| NM_001193473 | FOLH1 | 0.422161684 | 0.87361534 | 1.2047354 | up | mRNA |
| NM_001193483 | LIMS1 | 0.501666858 | 0.910145661 | 1.0218665 | up | mRNA |
| NM_001193522 | FAM65A | 0.74343236 | 0.970330526 | 1.0461969 | up | mRNA |
| NM_001193524 | FAM65A | 0.734671727 | 0.967777568 | 1.0391425 | up | mRNA |
| NM_001193530 | CCT6B | 0.869760392 | 0.989592579 | 1.011603 | up | mRNA |
| NM_001193557 | ENY2 | 0.730631142 | 0.966329458 | 1.0173337 | up | mRNA |
| NM_001193572 | CSRP1 | 0.008415934 | 0.198394583 | 1.296788 | up | mRNA |
| NM_001193600 | SCP2 | 0.801705766 | 0.98118927 | 1.0355591 | up | mRNA |
| NM_001193613 | TUBD1 | 0.278696902 | 0.777783404 | 1.2413715 | up | mRNA |
| NM_001193621 | PINLYP | 0.051741319 | 0.41031217 | 1.2764972 | up | mRNA |
| NM_001193622 | PINLYP | 0.479584688 | 0.901538317 | 1.4404128 | up | mRNA |
| NM_001193623 | LOC147646 | 0.847542875 | 0.987678166 | 1.0408337 | up | mRNA |
| NM_001193628 | LOC147670 | 0.006351619 | 0.180328763 | 1.5147788 | up | mRNA |
| NM_001193640 | CRB1 | 0.549984997 | 0.925533435 | 1.0435201 | up | mRNA |
| NM_001193654 | C17orf62 | 0.820370532 | 0.982666057 | 1.0316763 | up | mRNA |
| NM_001194947 | EIF4G1 | 0.701959958 | 0.963247543 | 1.0471586 | up | mRNA |
| NM_001194956 | MATR3 | 0.028167453 | 0.326447555 | 1.1489813 | up | mRNA |
| NM_001194986 | TRABD2B | 0.857641972 | 0.988512246 | 1.0344585 | up | mRNA |
| NM_001195005 | C11orf70 | 0.790268515 | 0.978438986 | 1.0283431 | up | mRNA |
| NM_001195007 | PPIE | 0.430933839 | 0.879515151 | 1.0425224 | up | mRNA |
| NM_001195076 | C19orf81 | 0.071505173 | 0.461977497 | 1.1675868 | up | mRNA |
| NM_001195127 | LOC100288524 | 0.697576486 | 0.962410025 | 1.0365885 | up | mRNA |
| NM_001195129 | PRSS56 | 0.06942027 | 0.457076726 | 1.2841704 | up | mRNA |
| NM_001195156 | ZMYM6NB | 0.616411365 | 0.945912508 | 1.0739582 | up | mRNA |
| NM_001195220 | ZNF783 | 0.01317326 | 0.234534775 | 1.3262225 | up | mRNA |
| NM_001195243 | LOC100287482 | 0.029484194 | 0.333953485 | 1.1592269 | up | mRNA |
| NM_001195259 | TGFBR3L | 0.767106198 | 0.975033576 | 1.0918254 | up | mRNA |
| NM_001195305 | BBIP1 | 0.39509407 | 0.861590143 | 1.099054 | up | mRNA |
| NM_001195306 | BBIP1 | 0.826000066 | 0.984306256 | 1.0215943 | up | mRNA |
| NM_001195307 | BBIP1 | 0.749839772 | 0.970725371 | 1.024429 | up | mRNA |
| NM_001195545 | LRRC3C | 0.762642199 | 0.973443358 | 1.0673134 | up | mRNA |
| NM_001196 | BID | 0.001214183 | 0.09206987 | 2.7606583 | up | mRNA |
| NM_001198858 | HLA-DQB2 | 0.971494533 | 0.998501532 | 1.0041023 | up | mRNA |
| NM_001199 | BMP1 | 0.750029985 | 0.970725371 | 1.0285421 | up | mRNA |
| NM_001199417 | ARHGAP23 | 0.568007517 | 0.931705674 | 1.0220402 | up | mRNA |
| NM_001199979 | PLSCR2 | 0.894252731 | 0.993978977 | 1.0119204 | up | mRNA |
| NM_001200 | BMP2 | 0.001635166 | 0.103894345 | 2.0658659 | up | mRNA |
| NM_001202439 | NCR3LG1 | 0.005360398 | 0.16793161 | 1.7963463 | up | mRNA |
| NM_001204068 | ANKS1B | 0.713075269 | 0.966110889 | 1.0248955 | up | mRNA |
| NM_001204069 | ANKS1B | 0.330216546 | 0.821769369 | 1.1246546 | up | mRNA |
| NM_001204070 | ANKS1B | 0.61016383 | 0.943169861 | 1.0713003 | up | mRNA |
| NM_001205266 | DEFB4B | 0.370813158 | 0.846330273 | 1.1963701 | up | mRNA |
| NM_001205271 | PRSS46 | 0.98255138 | 0.998971807 | 1.0032858 | up | mRNA |
| NM_001205281 | PPP5D1 | 0.713535218 | 0.966110889 | 1.1297911 | up | mRNA |
| NM_001205288 | LIMS3L | 0.383373615 | 0.853658125 | 1.5572839 | up | mRNA |
| NM_001206 | KLF9 | 0.042467224 | 0.381043071 | 1.198322 | up | mRNA |
| NM_001206670 | PLA2G4E | 0.24085453 | 0.739116169 | 1.1090033 | up | mRNA |
| NM_001206920 | SLC35F4 | 0.782207842 | 0.977014981 | 1.0701672 | up | mRNA |
| NM_001207 | BTF3 | 0.274445637 | 0.774561497 | 1.102155 | up | mRNA |
| NM_001207020 | SHISA8 | 0.231401212 | 0.729136134 | 1.0883535 | up | mRNA |
| NM_001207068 | BZW1 | 0.920802498 | 0.995349793 | 1.0256867 | up | mRNA |
| NM_001207069 | BZW1 | 0.450452425 | 0.888533638 | 1.1653882 | up | mRNA |
| NM_001215 | CA6 | 0.460819993 | 0.894196612 | 1.0438171 | up | mRNA |
| NM_001218 | CA12 | 0.608227899 | 0.942927156 | 1.0287257 | up | mRNA |
| NM_001220 | CAMK2B | 0.626864901 | 0.949203235 | 1.0397682 | up | mRNA |
| NM_001220480 | PPFIA2 | 0.644450921 | 0.954657712 | 1.1899376 | up | mRNA |
| NM_001220488 | CDH13 | 0.064711206 | 0.444345911 | 1.1933098 | up | mRNA |
| NM_001220489 | CDH13 | 0.554359404 | 0.926549129 | 1.1137319 | up | mRNA |
| NM_001220490 | CDH13 | 0.873548212 | 0.990580595 | 1.0417028 | up | mRNA |
| NM_001220491 | CDH13 | 0.18161166 | 0.664924727 | 1.1332926 | up | mRNA |
| NM_001220492 | CDH13 | 0.157029797 | 0.625751326 | 1.1329729 | up | mRNA |
| NM_001220769 | IKZF1 | 0.636550172 | 0.951995735 | 1.0973094 | up | mRNA |
| NM_001223 | CASP1 | 0.914833918 | 0.995349793 | 1.0881545 | up | mRNA |
| NM_001225 | CASP4 | 0.012943773 | 0.232279602 | 1.3666004 | up | mRNA |
| NM_001227 | CASP7 | 0.105795544 | 0.538296946 | 1.5369088 | up | mRNA |
| NM_001228 | CASP8 | 0.034615154 | 0.35197585 | 1.2219705 | up | mRNA |
| NM_001231 | CASQ1 | 0.586253124 | 0.935318466 | 1.0559773 | up | mRNA |
| NM_001236 | CBR3 | 0.009375627 | 0.204533521 | 2.1283351 | up | mRNA |
| NM_001238 | CCNE1 | 0.704007953 | 0.963625105 | 1.1115032 | up | mRNA |
| NM_001241 | CCNT2 | 0.955083251 | 0.996883425 | 1.0044779 | up | mRNA |
| NM_001242307 | PITRM1 | 0.627214828 | 0.949272881 | 1.1349558 | up | mRNA |
| NM_001242309 | PITRM1 | 0.912264144 | 0.995342392 | 1.0268563 | up | mRNA |
| NM_001242348 | LOC100287177 | 0.853069827 | 0.987678166 | 1.0891806 | up | mRNA |
| NM_001242366 | SLC46A1 | 0.584382574 | 0.93461341 | 1.0758996 | up | mRNA |
| NM_001242480 | LOC389831 | 0.938935023 | 0.996710991 | 1.0351364 | up | mRNA |
| NM_001242559 | MAP4K4 | 0.140858786 | 0.601036902 | 1.0562759 | up | mRNA |
| NM_001242560 | MAP4K4 | 0.10319899 | 0.532945334 | 1.2208621 | up | mRNA |
| NM_001242608 | NCAM1 | 0.465691298 | 0.895570502 | 1.3601997 | up | mRNA |
| NM_001242671 | C20orf78 | 0.039542086 | 0.370322288 | 1.19445 | up | mRNA |
| NM_001242672 | TTC34 | 0.211935527 | 0.703329735 | 1.1028722 | up | mRNA |
| NM_001242758 | HLA-A | 0.433863492 | 0.880923255 | 1.199728 | up | mRNA |
| NM_001242804 | LOC100505549 | 0.393149681 | 0.859795559 | 1.0719876 | up | mRNA |
| NM_001242812 | LOC284385 | 0.16028035 | 0.631980676 | 1.241304 | up | mRNA |
| NM_001242901 | LOC100131094 | 0.842494124 | 0.987182488 | 1.0475249 | up | mRNA |
| NM_001243042 | HLA-C | 0.693426823 | 0.961584652 | 1.0742556 | up | mRNA |
| NM_001243079 | PLK5 | 0.148940618 | 0.61263618 | 1.2112346 | up | mRNA |
| NM_001243186 | PIM1 | 0.030895726 | 0.340185047 | 5.04693 | up | mRNA |
| NM_001243247 | NPRL3 | 0.687520774 | 0.960205549 | 1.0729154 | up | mRNA |
| NM_001243538 | LOC283710 | 0.414023224 | 0.86961884 | 1.0570944 | up | mRNA |
| NM_001247 | ENTPD6 | 0.546585594 | 0.924803608 | 1.1488917 | up | mRNA |
| NM_001248 | ENTPD3 | 0.651464859 | 0.956204521 | 1.0662929 | up | mRNA |
| NM_001249 | ENTPD5 | 0.511353344 | 0.912481293 | 1.0591617 | up | mRNA |
| NM_001250 | CD40 | 0.26419214 | 0.764950604 | 1.4182685 | up | mRNA |
| NM_001251 | CD68 | 0.01240007 | 0.226521311 | 1.2077827 | up | mRNA |
| NM_001252 | CD70 | 0.001014056 | 0.085705167 | 3.455059 | up | mRNA |
| NM_001252404 | TMEM249 | 0.96778797 | 0.99793927 | 1.0060368 | up | mRNA |
| NM_001252507 | C14orf132 | 0.672904652 | 0.959531882 | 1.1257472 | up | mRNA |
| NM_001253 | CDC5L | 0.070358248 | 0.458686035 | 1.1503193 | up | mRNA |
| NM_001253791 | LOC440335 | 0.743823381 | 0.970330526 | 1.0638938 | up | mRNA |
| NM_001253793 | LOC440335 | 0.331531718 | 0.822036938 | 1.1765406 | up | mRNA |
| NM_001255 | CDC20 | 0.910117623 | 0.995342392 | 1.011508 | up | mRNA |
| NM_001256368 | LOC100144595 | 0.262094276 | 0.763125731 | 1.1234625 | up | mRNA |
| NM_001256395 | RHBG | 0.538441165 | 0.923577434 | 1.0873506 | up | mRNA |
| NM_001256396 | RHBG | 0.186291092 | 0.669888134 | 1.0807258 | up | mRNA |
| NM_001256526 | C9orf69 | 0.542858432 | 0.923577434 | 1.0672959 | up | mRNA |
| NM_001256686 | CSNK2A1P | 0.703513384 | 0.963625105 | 1.047798 | up | mRNA |
| NM_001256795 | LOC100129083 | 0.056097221 | 0.422065984 | 1.0816989 | up | mRNA |
| NM_001256852 | LOC100287144 | 0.799251099 | 0.980917337 | 1.0517821 | up | mRNA |
| NM_001256861 | LOC100287441 | 0.920379385 | 0.995349793 | 1.0145393 | up | mRNA |
| NM_001257 | CDH13 | 0.102440802 | 0.531296253 | 1.1482276 | up | mRNA |
| NM_001257964 | BOD1L2 | 0.862269069 | 0.989401142 | 1.0102747 | up | mRNA |
| NM_001258 | CDK3 | 0.474456003 | 0.899495615 | 1.1086649 | up | mRNA |
| NM_001264 | CDSN | 0.434187993 | 0.880931467 | 1.0553044 | up | mRNA |
| NM_001266 | CES1 | 0.485477906 | 0.903250293 | 1.1725295 | up | mRNA |
| NM_001267 | CHAD | 0.100290042 | 0.526354447 | 1.1996901 | up | mRNA |
| NM_001270 | CHD1 | 0.948037745 | 0.996710991 | 1.0044035 | up | mRNA |
| NM_001270601 | ASCL5 | 0.493304269 | 0.906383343 | 1.0900269 | up | mRNA |
| NM_001271 | CHD2 | 0.85073419 | 0.987678166 | 1.0294211 | up | mRNA |
| NM_001273 | CHD4 | 0.821148906 | 0.982756843 | 1.0452899 | up | mRNA |
| NM_001274 | CHEK1 | 0.847669775 | 0.987678166 | 1.0522451 | up | mRNA |
| NM_001284 | AP3S1 | 0.475729708 | 0.900105754 | 1.0478473 | up | mRNA |
| NM_001286 | CLCN6 | 0.706865451 | 0.965401045 | 1.0353685 | up | mRNA |
| NM_001289 | CLIC2 | 0.581372269 | 0.934005566 | 1.1035421 | up | mRNA |
| NM_001297 | CNGB1 | 0.114080176 | 0.557835051 | 1.2220557 | up | mRNA |
| NM_001300 | KLF6 | 0.155200312 | 0.623007454 | 1.2836866 | up | mRNA |
| NM_001302 | CORT | 0.602244692 | 0.941406011 | 1.1531631 | up | mRNA |
| NM_001305 | CLDN4 | 0.104661787 | 0.535611239 | 1.4878159 | up | mRNA |
| NM_001307 | CLDN7 | 0.794826944 | 0.979537918 | 1.0464095 | up | mRNA |
| NM_001317 | CSH1 | 0.128225321 | 0.581807014 | 1.1497021 | up | mRNA |
| NM_001318 | CSHL1 | 0.930896133 | 0.996710991 | 1.0068924 | up | mRNA |
| NM_001320 | CSNK2B | 0.313580237 | 0.808703036 | 1.0320878 | up | mRNA |
| NM_001321 | CSRP2 | 0.563302195 | 0.929041598 | 1.2491556 | up | mRNA |
| NM_001328 | CTBP1 | 0.41631664 | 0.870736736 | 1.0810857 | up | mRNA |
| NM_001331 | CTNND1 | 0.539195084 | 0.923577434 | 1.1580474 | up | mRNA |
| NM_001333 | CTSL2 | 0.338316015 | 0.827403747 | 1.0857706 | up | mRNA |
| NM_001337 | CX3CR1 | 0.033217188 | 0.346375523 | 1.3927246 | up | mRNA |
| NM_001351 | DAZL | 0.196884797 | 0.685161614 | 1.3333067 | up | mRNA |
| NM_001352 | DBP | 0.936794993 | 0.996710991 | 1.009364 | up | mRNA |
| NM_001354 | AKR1C2 | 0.000419105 | 0.063120244 | 1.4559538 | up | mRNA |
| NM_001357 | DHX9 | 0.49242257 | 0.906047853 | 1.0693216 | up | mRNA |
| NM_001359 | DECR1 | 0.324882906 | 0.817191271 | 1.203408 | up | mRNA |
| NM_001375 | DNASE2 | 0.399811331 | 0.864196266 | 1.1408489 | up | mRNA |
| NM_001379 | DNMT1 | 0.923421248 | 0.995456159 | 1.0298898 | up | mRNA |
| NM_001380 | DOCK1 | 0.94647371 | 0.996710991 | 1.0143005 | up | mRNA |
| NM_001386 | DPYSL2 | 0.32403241 | 0.816485317 | 1.0776772 | up | mRNA |
| NM_001393 | ECM2 | 0.083681795 | 0.493003289 | 1.3892167 | up | mRNA |
| NM_001395 | DUSP9 | 0.780817711 | 0.976938981 | 1.0410812 | up | mRNA |
| NM_001397 | ECE1 | 0.775100072 | 0.97589756 | 1.0312225 | up | mRNA |
| NM_001398 | ECH1 | 0.46741849 | 0.896646168 | 1.0845273 | up | mRNA |
| NM_001399 | EDA | 0.558200599 | 0.926833616 | 1.3755716 | up | mRNA |
| NM_001404 | EEF1G | 0.963500174 | 0.99793927 | 1.0048769 | up | mRNA |
| NM_001405 | EFNA2 | 0.172678205 | 0.650463738 | 1.2617508 | up | mRNA |
| NM_001415 | EIF2S3 | 0.980978495 | 0.998971807 | 1.0104905 | up | mRNA |
| NM_001424 | EMP2 | 0.198973344 | 0.687546912 | 1.37071 | up | mRNA |
| NM_001426 | EN1 | 0.672533965 | 0.959531882 | 1.1180452 | up | mRNA |
| NM_001430 | EPAS1 | 0.190919972 | 0.676402188 | 1.2805607 | up | mRNA |
| NM_001432 | EREG | 0.044578919 | 0.387767262 | 4.8533286 | up | mRNA |
| NM_001433 | ERN1 | 0.005745402 | 0.17178751 | 1.4209003 | up | mRNA |
| NM_001440 | EXTL3 | 0.627823632 | 0.949611308 | 1.026715 | up | mRNA |
| NM_001441 | FAAH | 0.272964863 | 0.772770327 | 1.0776488 | up | mRNA |
| NM_001442 | FABP4 | 0.444687177 | 0.88545487 | 1.3638988 | up | mRNA |
| NM_001447 | FAT2 | 0.340005885 | 0.828118322 | 1.1051537 | up | mRNA |
| NM_001451 | FOXF1 | 0.559436545 | 0.927203296 | 1.0927338 | up | mRNA |
| NM_001453 | FOXC1 | 0.00994411 | 0.207155377 | 1.5028389 | up | mRNA |
| NM_001454 | FOXJ1 | 0.124310269 | 0.578369571 | 1.145983 | up | mRNA |
| NM_001457 | FLNB | 0.578613283 | 0.933821303 | 1.0656691 | up | mRNA |
| NM_001458 | FLNC | 0.784838073 | 0.977411899 | 1.0325457 | up | mRNA |
| NM_001465 | FYB | 0.019526965 | 0.281049008 | 1.5968425 | up | mRNA |
| NM_001467 | SLC37A4 | 0.070010159 | 0.457987595 | 1.1237377 | up | mRNA |
| NM_001478 | B4GALNT1 | 0.01025603 | 0.20936306 | 1.3730062 | up | mRNA |
| NM_001491 | GCNT2 | 0.444879132 | 0.885663616 | 1.2503495 | up | mRNA |
| NM_001493 | GDI1 | 0.662907702 | 0.958354006 | 1.0238345 | up | mRNA |
| NM_001495 | GFRA2 | 0.499243342 | 0.909361611 | 1.0521785 | up | mRNA |
| NM_001497 | B4GALT1 | 0.396114925 | 0.861880738 | 1.2695796 | up | mRNA |
| NM_001501 | GNRH2 | 0.583549829 | 0.934280817 | 1.0603777 | up | mRNA |
| NM_001505 | GPER | 0.528978463 | 0.918932853 | 1.2964208 | up | mRNA |
| NM_001511 | CXCL1 | 6.23173E-06 | 0.012670363 | 63.6688294 | up | mRNA |
| NM_001519 | BRF1 | 0.30549699 | 0.801185951 | 1.0795947 | up | mRNA |
| NM_001528 | HGFAC | 0.778699277 | 0.976938981 | 1.0285024 | up | mRNA |
| NM_001539 | DNAJA1 | 0.049543845 | 0.40293018 | 1.3405977 | up | mRNA |
| NM_001544 | ICAM4 | 1.05067E-05 | 0.015258694 | 17.9016211 | up | mRNA |
| NM_001545 | ICT1 | 0.877066645 | 0.991255275 | 1.0113871 | up | mRNA |
| NM_001546 | ID4 | 0.07744003 | 0.478473478 | 1.5081347 | up | mRNA |
| NM_001552 | IGFBP4 | 0.895119069 | 0.993978977 | 1.0266753 | up | mRNA |
| NM_001553 | IGFBP7 | 0.124644462 | 0.578441772 | 1.0532226 | up | mRNA |
| NM_001555 | IGSF1 | 0.168119534 | 0.642977391 | 1.1045095 | up | mRNA |
| NM_001556 | IKBKB | 0.000175241 | 0.044621794 | 1.8094519 | up | mRNA |
| NM_001558 | IL10RA | 0.308650962 | 0.803800359 | 1.0822026 | up | mRNA |
| NM_001561 | TNFRSF9 | 0.001331823 | 0.0936977 | 2.453929 | up | mRNA |
| NM_001565 | CXCL10 | 0.062200597 | 0.439379649 | 5.3236883 | up | mRNA |
| NM_001569 | IRAK1 | 0.809313527 | 0.98118927 | 1.0449864 | up | mRNA |
| NM_001570 | IRAK2 | 0.000260413 | 0.052661938 | 3.2340364 | up | mRNA |
| NM_001572 | IRF7 | 0.894175865 | 0.993978977 | 1.0099286 | up | mRNA |
| NM_001586 | TEX28 | 0.093664198 | 0.512619241 | 1.1033058 | up | mRNA |
| NM_001606 | ABCA2 | 0.159757079 | 0.63153842 | 1.0863584 | up | mRNA |
| NM_001607 | ACAA1 | 0.973797548 | 0.998501532 | 1.0044611 | up | mRNA |
| NM_001621 | AHR | 0.825170408 | 0.98381451 | 1.0768756 | up | mRNA |
| NM_001624 | AIM1 | 0.0145857 | 0.243678265 | 4.6703895 | up | mRNA |
| NM_001625 | AK2 | 0.890608616 | 0.9937521 | 1.0129505 | up | mRNA |
| NM_001627 | ALCAM | 0.001696231 | 0.10523567 | 1.4823026 | up | mRNA |
| NM_001628 | AKR1B1 | 0.162274239 | 0.63437028 | 1.5046353 | up | mRNA |
| NM_001630 | ANXA8L2 | 0.055910139 | 0.421647851 | 1.2111467 | up | mRNA |
| NM_001635 | AMPH | 0.861036261 | 0.989013989 | 1.0334668 | up | mRNA |
| NM_001644 | APOBEC1 | 0.438223058 | 0.882682266 | 1.2138111 | up | mRNA |
| NM_001651 | AQP5 | 0.579000767 | 0.933821303 | 1.0476401 | up | mRNA |
| NM_001672 | ASIP | 0.599970044 | 0.940648177 | 1.226444 | up | mRNA |
| NM_001682 | ATP2B1 | 0.079574966 | 0.482639336 | 2.6242464 | up | mRNA |
| NM_001683 | ATP2B2 | 0.023540454 | 0.301884214 | 1.2016125 | up | mRNA |
| NM_001684 | ATP2B4 | 0.153783066 | 0.620938377 | 1.1329303 | up | mRNA |
| NM_001686 | ATP5B | 0.550460753 | 0.925909806 | 1.038047 | up | mRNA |
| NM_001694 | ATP6V0C | 0.831563004 | 0.985965651 | 1.0380558 | up | mRNA |
| NM_001697 | ATP5O | 0.09508173 | 0.516597924 | 1.1396747 | up | mRNA |
| NM_001699 | AXL | 0.033634579 | 0.348374048 | 1.1187854 | up | mRNA |
| NM_001706 | BCL6 | 0.103356999 | 0.533123263 | 1.2773202 | up | mRNA |
| NM_001708 | OPN1SW | 0.335013782 | 0.824836547 | 1.1830508 | up | mRNA |
| NM_001710 | CFB | 0.001015884 | 0.085705167 | 2.5788574 | up | mRNA |
| NM_001717 | BNC1 | 0.386726882 | 0.856062164 | 1.1824401 | up | mRNA |
| NM_001719 | BMP7 | 0.081013282 | 0.486318878 | 1.2632437 | up | mRNA |
| NM_001722 | POLR3D | 0.121140952 | 0.572400147 | 1.3701025 | up | mRNA |
| NM_001724 | BPGM | 0.005069102 | 0.162819877 | 1.322441 | up | mRNA |
| NM_001728 | BSG | 0.108074002 | 0.544140502 | 1.274985 | up | mRNA |
| NM_001729 | BTC | 0.218551789 | 0.712342894 | 1.0921396 | up | mRNA |
| NM_001730 | KLF5 | 0.000134131 | 0.04328803 | 1.9415584 | up | mRNA |
| NM_001731 | BTG1 | 0.009080607 | 0.202886702 | 1.7034997 | up | mRNA |
| NM_001735 | C5 | 0.798655463 | 0.980749101 | 1.0121729 | up | mRNA |
| NM_001736 | C5AR1 | 0.212410303 | 0.704178424 | 1.52029 | up | mRNA |
| NM_001740 | CALB2 | 0.086284637 | 0.49936346 | 1.1644246 | up | mRNA |
| NM_001748 | CAPN2 | 0.406863918 | 0.867591329 | 1.0929558 | up | mRNA |
| NM_001751 | CARS | 0.533804017 | 0.92058057 | 1.0419791 | up | mRNA |
| NM_001752 | CAT | 0.727833321 | 0.966191059 | 1.0174146 | up | mRNA |
| NM_001753 | CAV1 | 0.605710557 | 0.94174007 | 1.1625389 | up | mRNA |
| NM_001760 | CCND3 | 0.194524964 | 0.682263509 | 1.078627 | up | mRNA |
| NM_001763 | CD1A | 0.063341882 | 0.440687083 | 1.2274213 | up | mRNA |
| NM_001764 | CD1B | 0.315879326 | 0.810545544 | 1.1162026 | up | mRNA |
| NM_001765 | CD1C | 0.680379357 | 0.960111041 | 1.1251572 | up | mRNA |
| NM_001769 | CD9 | 0.983290428 | 0.998971807 | 1.0064508 | up | mRNA |
| NM_001773 | CD34 | 0.260546215 | 0.761124374 | 1.1847218 | up | mRNA |
| NM_001779 | CD58 | 0.004573062 | 0.155014562 | 1.5539201 | up | mRNA |
| NM_001785 | CDA | 0.557626658 | 0.926833616 | 1.248852 | up | mRNA |
| NM_001788 | SEPT7 | 0.682460064 | 0.960111041 | 1.0354925 | up | mRNA |
| NM_001790 | CDC25C | 0.920318672 | 0.995349793 | 1.0249746 | up | mRNA |
| NM_001792 | CDH2 | 0.267725028 | 0.768622602 | 1.3011912 | up | mRNA |
| NM_001793 | CDH3 | 0.366640921 | 0.845181673 | 1.1396092 | up | mRNA |
| NM_001798 | CDK2 | 0.013395296 | 0.23544335 | 1.4374946 | up | mRNA |
| NM_001801 | CDO1 | 0.863459835 | 0.989401142 | 1.0777406 | up | mRNA |
| NM_001803 | CD52 | 0.933998994 | 0.996710991 | 1.0087052 | up | mRNA |
| NM_001805 | CEBPE | 0.171133033 | 0.648430269 | 1.1359545 | up | mRNA |
| NM_001814 | CTSC | 0.868170642 | 0.989481868 | 1.0399993 | up | mRNA |
| NM_001818 | AKR1C4 | 0.273910132 | 0.774088254 | 1.2398558 | up | mRNA |
| NM_001819 | CHGB | 0.815945366 | 0.98149313 | 1.0624122 | up | mRNA |
| NM_001822 | CHN1 | 0.96573261 | 0.99793927 | 1.0205098 | up | mRNA |
| NM_001823 | CKB | 0.689171093 | 0.96108227 | 1.1103899 | up | mRNA |
| NM_001824 | CKM | 0.509679534 | 0.911674633 | 1.4734753 | up | mRNA |
| NM_001826 | CKS1B | 0.072823391 | 0.463930261 | 1.2803584 | up | mRNA |
| NM_001836 | CMA1 | 0.818727505 | 0.982093332 | 1.0442654 | up | mRNA |
| NM_001838 | CCR7 | 0.410224254 | 0.86807846 | 1.0996442 | up | mRNA |
| NM_001841 | CNR2 | 0.197133556 | 0.685161614 | 1.2378605 | up | mRNA |
| NM_001843 | CNTN1 | 0.985755242 | 0.998971807 | 1.0068947 | up | mRNA |
| NM_001844 | COL2A1 | 0.465774335 | 0.895570502 | 1.038413 | up | mRNA |
| NM_001845 | COL4A1 | 0.280311478 | 0.778821055 | 1.0513416 | up | mRNA |
| NM_001848 | COL6A1 | 0.117548438 | 0.564147103 | 1.0993911 | up | mRNA |
| NM_001855 | COL15A1 | 0.978595362 | 0.998661138 | 1.0071604 | up | mRNA |
| NM_001856 | COL16A1 | 0.530017568 | 0.91899174 | 1.1087125 | up | mRNA |
| NM_001860 | SLC31A2 | 0.169141439 | 0.645576073 | 1.1550875 | up | mRNA |
| NM_001862 | COX5B | 0.208866472 | 0.700205031 | 1.0801653 | up | mRNA |
| NM_001865 | COX7A2 | 0.424767491 | 0.874854499 | 1.1331182 | up | mRNA |
| NM_001869 | CPA2 | 0.693414726 | 0.961584652 | 1.0727426 | up | mRNA |
| NM_001870 | CPA3 | 0.303661071 | 0.79933155 | 1.888871 | up | mRNA |
| NM_001873 | CPE | 0.851065415 | 0.987678166 | 1.1147362 | up | mRNA |
| NM_001883 | CRHR2 | 0.562040141 | 0.928830379 | 1.0822049 | up | mRNA |
| NM_001885 | CRYAB | 0.785213775 | 0.977411899 | 1.064641 | up | mRNA |
| NM_001888 | CRYM | 0.31623661 | 0.810808526 | 1.0563771 | up | mRNA |
| NM_001899 | CST4 | 0.294081972 | 0.791146775 | 1.5031567 | up | mRNA |
| NM_001907 | CTRL | 0.740832004 | 0.970153053 | 1.0269341 | up | mRNA |
| NM_001914 | CYB5A | 0.02267975 | 0.29673403 | 1.2716233 | up | mRNA |
| NM_001916 | CYC1 | 0.82080454 | 0.982756843 | 1.0214217 | up | mRNA |
| NM_001923 | DDB1 | 0.155523061 | 0.623056705 | 1.1232012 | up | mRNA |
| NM_001924 | GADD45A | 0.074152568 | 0.469200549 | 1.3114811 | up | mRNA |
| NM_001928 | CFD | 0.909027791 | 0.995333785 | 1.0296388 | up | mRNA |
| NM_001935 | DPP4 | 0.391610552 | 0.859202394 | 1.0633469 | up | mRNA |
| NM_001937 | DPT | 0.129713511 | 0.583907255 | 1.1680514 | up | mRNA |
| NM_001938 | DR1 | 0.382581763 | 0.853514109 | 1.0739279 | up | mRNA |
| NM_001941 | DSC3 | 0.911698752 | 0.995342392 | 1.0167969 | up | mRNA |
| NM_001945 | HBEGF | 0.000258071 | 0.052661938 | 2.5996919 | up | mRNA |
| NM_001946 | DUSP6 | 0.081209914 | 0.486780649 | 2.013491 | up | mRNA |
| NM_001954 | DDR1 | 0.63594202 | 0.951814727 | 1.0893965 | up | mRNA |
| NM_001955 | EDN1 | 0.030897169 | 0.340185047 | 1.3319847 | up | mRNA |
| NM_001963 | EGF | 0.212559191 | 0.704442294 | 1.2412713 | up | mRNA |
| NM_001964 | EGR1 | 0.655301968 | 0.957222998 | 1.1138895 | up | mRNA |
| NM_001965 | EGR4 | 0.791598383 | 0.978822498 | 1.0423186 | up | mRNA |
| NM_001968 | EIF4E | 0.455233369 | 0.891148383 | 1.1985949 | up | mRNA |
| NM_001978 | EPB49 | 0.683900953 | 0.960111041 | 1.0905118 | up | mRNA |
| NM_001987 | ETV6 | 0.380726982 | 0.853514109 | 1.1433573 | up | mRNA |
| NM_001991 | EZH1 | 0.546914999 | 0.924803608 | 1.227881 | up | mRNA |
| NM_001992 | F2R | 0.257501413 | 0.757782419 | 1.0815033 | up | mRNA |
| NM_001993 | F3 | 0.539783197 | 0.923577434 | 1.5521393 | up | mRNA |
| NM_001997 | FAU | 0.524709455 | 0.917632259 | 1.0330492 | up | mRNA |
| NM_002003 | FCN1 | 0.360186256 | 0.841759421 | 1.0632833 | up | mRNA |
| NM_002006 | FGF2 | 0.034918677 | 0.352338753 | 1.6842701 | up | mRNA |
| NM_002009 | FGF7 | 0.697802612 | 0.962410025 | 1.0900477 | up | mRNA |
| NM_002015 | FOXO1 | 0.008383922 | 0.198394583 | 1.5095142 | up | mRNA |
| NM_002017 | FLI1 | 0.395954492 | 0.861849835 | 1.046062 | up | mRNA |
| NM_002018 | FLII | 0.319041169 | 0.812974553 | 1.2125365 | up | mRNA |
| NM_002021 | FMO1 | 0.354772428 | 0.83766056 | 1.2994077 | up | mRNA |
| NM_002023 | FMOD | 0.611507584 | 0.943894921 | 1.0759095 | up | mRNA |
| NM_002028 | FNTB | 0.31727996 | 0.811824899 | 1.0999602 | up | mRNA |
| NM_002029 | FPR1 | 0.666621004 | 0.958354006 | 1.0771078 | up | mRNA |
| NM_002038 | IFI6 | 0.484866063 | 0.903250293 | 1.1036057 | up | mRNA |
| NM_002040 | GABPA | 0.049095563 | 0.402023817 | 1.1568415 | up | mRNA |
| NM_002041 | GABPB1 | 0.011711003 | 0.220344574 | 1.4071531 | up | mRNA |
| NM_002045 | GAP43 | 0.417467702 | 0.871363651 | 1.1820911 | up | mRNA |
| NM_002049 | GATA1 | 0.027775746 | 0.32381669 | 1.4690208 | up | mRNA |
| NM_002051 | GATA3 | 0.223586626 | 0.717768501 | 1.0987918 | up | mRNA |
| NM_002053 | GBP1 | 0.092155693 | 0.511207518 | 2.8577072 | up | mRNA |
| NM_002055 | GFAP | 0.920878553 | 0.995349793 | 1.0109104 | up | mRNA |
| NM_002064 | GLRX | 0.24535069 | 0.743589595 | 1.0789175 | up | mRNA |
| NM_002069 | GNAI1 | 0.059584254 | 0.432821385 | 1.0746783 | up | mRNA |
| NM_002070 | GNAI2 | 0.890086312 | 0.9937521 | 1.010879 | up | mRNA |
| NM_002078 | GOLGA4 | 0.977314911 | 0.998501532 | 1.0028972 | up | mRNA |
| NM_002084 | GPX3 | 0.439107867 | 0.882895421 | 1.0579939 | up | mRNA |
| NM_002085 | GPX4 | 0.101390427 | 0.529124784 | 1.0898008 | up | mRNA |
| NM_002086 | GRB2 | 0.946183663 | 0.996710991 | 1.0032313 | up | mRNA |
| NM_002088 | GRIK5 | 0.661156017 | 0.958354006 | 1.0423772 | up | mRNA |
| NM_002089 | CXCL2 | 0.000136348 | 0.043316065 | 72.9340279 | up | mRNA |
| NM_002090 | CXCL3 | 7.76277E-05 | 0.033623631 | 35.8795776 | up | mRNA |
| NM_002092 | GRSF1 | 0.603229419 | 0.941484604 | 1.0493366 | up | mRNA |
| NM_002093 | GSK3B | 0.042384261 | 0.380634622 | 1.1957503 | up | mRNA |
| NM_002094 | GSPT1 | 0.383786376 | 0.854109523 | 1.0950201 | up | mRNA |
| NM_002097 | GTF3A | 0.817098335 | 0.981692737 | 1.0238476 | up | mRNA |
| NM_002101 | GYPC | 0.665037301 | 0.958354006 | 1.0174432 | up | mRNA |
| NM_002103 | GYS1 | 0.93717964 | 0.996710991 | 1.0143 | up | mRNA |
| NM_002112 | HDC | 0.135208402 | 0.592787801 | 1.1355098 | up | mRNA |
| NM_002114 | HIVEP1 | 0.00963917 | 0.205650016 | 2.2117183 | up | mRNA |
| NM_002121 | HLA-DPB1 | 0.952957149 | 0.996710991 | 1.0090831 | up | mRNA |
| NM_002122 | HLA-DQA1 | 0.675818319 | 0.959531882 | 1.0894678 | up | mRNA |
| NM_002123 | HLA-DQB1 | 0.832040285 | 0.98620142 | 1.0930664 | up | mRNA |
| NM_002125 | HLA-DRB5 | 0.518948245 | 0.916145597 | 1.2204371 | up | mRNA |
| NM_002130 | HMGCS1 | 0.421791867 | 0.873398499 | 1.0704274 | up | mRNA |
| NM_002139 | RBMX | 0.345356358 | 0.832533915 | 1.1105588 | up | mRNA |
| NM_002141 | HOXA4 | 0.99080509 | 0.999376923 | 1.0023296 | up | mRNA |
| NM_002145 | HOXB2 | 0.707083248 | 0.965401045 | 1.0459717 | up | mRNA |
| NM_002152 | HRC | 0.935582623 | 0.996710991 | 1.0096324 | up | mRNA |
| NM_002160 | TNC | 0.009298686 | 0.204533521 | 2.0264408 | up | mRNA |
| NM_002166 | ID2 | 0.01735653 | 0.265474108 | 2.5155932 | up | mRNA |
| NM_002167 | ID3 | 0.034146616 | 0.349933975 | 1.3181294 | up | mRNA |
| NM_002178 | IGFBP6 | 0.874387338 | 0.990846067 | 1.0077157 | up | mRNA |
| NM_002184 | IL6ST | 0.299425721 | 0.795703014 | 1.0985181 | up | mRNA |
| NM_002185 | IL7R | 0.00621049 | 0.178855089 | 1.6938945 | up | mRNA |
| NM_002187 | IL12B | 0.770705016 | 0.975347132 | 1.0743943 | up | mRNA |
| NM_002189 | IL15RA | 0.321332431 | 0.815220381 | 1.0662884 | up | mRNA |
| NM_002191 | INHA | 0.154369473 | 0.621759137 | 1.1269968 | up | mRNA |
| NM_002192 | INHBA | 0.002349349 | 0.115379162 | 4.1053067 | up | mRNA |
| NM_002193 | INHBB | 0.961442639 | 0.99793927 | 1.0070371 | up | mRNA |
| NM_002196 | INSM1 | 0.542405415 | 0.923577434 | 1.2767912 | up | mRNA |
| NM_002198 | IRF1 | 3.25181E-05 | 0.023612787 | 19.2190684 | up | mRNA |
| NM_002199 | IRF2 | 0.055701875 | 0.421334923 | 1.1375936 | up | mRNA |
| NM_002201 | ISG20 | 0.347345192 | 0.83359566 | 1.6834595 | up | mRNA |
| NM_002210 | ITGAV | 0.112903477 | 0.555958703 | 1.1374506 | up | mRNA |
| NM_002214 | ITGB8 | 0.006020812 | 0.176785634 | 1.6329967 | up | mRNA |
| NM_002218 | ITIH4 | 0.490089607 | 0.905264563 | 1.2079324 | up | mRNA |
| NM_002220 | ITPKA | 0.356673586 | 0.839337376 | 1.1220811 | up | mRNA |
| NM_002223 | ITPR2 | 0.11920642 | 0.568456822 | 1.2711968 | up | mRNA |
| NM_002224 | ITPR3 | 0.878889727 | 0.991983232 | 1.0348693 | up | mRNA |
| NM_002227 | JAK1 | 0.089531732 | 0.505936401 | 1.1109809 | up | mRNA |
| NM_002228 | JUN | 0.081485565 | 0.487857627 | 1.611134 | up | mRNA |
| NM_002229 | JUNB | 0.182725935 | 0.666042256 | 1.4908275 | up | mRNA |
| NM_002231 | CD82 | 0.000173326 | 0.044621794 | 2.6546025 | up | mRNA |
| NM_002235 | KCNA6 | 0.318163357 | 0.812165395 | 1.2137318 | up | mRNA |
| NM_002240 | KCNJ6 | 0.444982848 | 0.885721054 | 1.1201326 | up | mRNA |
| NM_002241 | KCNJ10 | 0.216850098 | 0.711074992 | 1.0957575 | up | mRNA |
| NM_002247 | KCNMA1 | 0.956750509 | 0.996883425 | 1.0207786 | up | mRNA |
| NM_002251 | KCNS1 | 0.772669411 | 0.975589298 | 1.0273082 | up | mRNA |
| NM_002254 | KIF3C | 0.07588227 | 0.473352674 | 1.2485143 | up | mRNA |
| NM_002258 | KLRB1 | 0.43658961 | 0.882102748 | 1.1178864 | up | mRNA |
| NM_002267 | KPNA3 | 0.543592869 | 0.923577434 | 1.056133 | up | mRNA |
| NM_002272 | KRT4 | 0.898259425 | 0.994360027 | 1.0272562 | up | mRNA |
| NM_002274 | KRT13 | 0.2846267 | 0.78179061 | 1.18169 | up | mRNA |
| NM_002278 | KRT32 | 0.671496657 | 0.959531882 | 1.0968084 | up | mRNA |
| NM_002284 | KRT86 | 0.753352016 | 0.970725371 | 1.0462597 | up | mRNA |
| NM_002285 | AFF3 | 0.126977227 | 0.580406758 | 1.3774306 | up | mRNA |
| NM_002287 | LAIR1 | 0.891623789 | 0.9937521 | 1.01999 | up | mRNA |
| NM_002290 | LAMA4 | 0.783622958 | 0.977275912 | 1.0133721 | up | mRNA |
| NM_002293 | LAMC1 | 0.665280378 | 0.958354006 | 1.0475027 | up | mRNA |
| NM_002294 | LAMP2 | 0.93281997 | 0.996710991 | 1.0247398 | up | mRNA |
| NM_002295 | RPSA | 0.911187539 | 0.995342392 | 1.0098701 | up | mRNA |
| NM_002297 | LCN1 | 0.605795732 | 0.94174007 | 1.1136238 | up | mRNA |
| NM_002305 | LGALS1 | 0.270726085 | 0.77070887 | 1.0364887 | up | mRNA |
| NM_002306 | LGALS3 | 0.88251496 | 0.992766819 | 1.0098614 | up | mRNA |
| NM_002313 | ABLIM1 | 0.952337107 | 0.996710991 | 1.0076132 | up | mRNA |
| NM_002317 | LOX | 0.687421474 | 0.960205549 | 1.0331124 | up | mRNA |
| NM_002334 | LRP4 | 0.763105421 | 0.97349057 | 1.0738064 | up | mRNA |
| NM_002336 | LRP6 | 0.643237172 | 0.954522001 | 1.0574464 | up | mRNA |
| NM_002342 | LTBR | 0.355576137 | 0.838390774 | 1.067189 | up | mRNA |
| NM_002343 | LTF | 0.241822001 | 0.740247655 | 1.0908729 | up | mRNA |
| NM_002344 | LTK | 0.728347589 | 0.966191059 | 1.0311143 | up | mRNA |
| NM_002345 | LUM | 0.256523129 | 0.757240077 | 1.2478706 | up | mRNA |
| NM_002348 | LY9 | 0.186447104 | 0.669888134 | 1.1522582 | up | mRNA |
| NM_002350 | LYN | 0.040315844 | 0.371915487 | 2.2072308 | up | mRNA |
| NM_002353 | TACSTD2 | 0.942245336 | 0.996710991 | 1.0152506 | up | mRNA |
| NM_002354 | EPCAM | 0.919533068 | 0.995349793 | 1.0144565 | up | mRNA |
| NM_002356 | MARCKS | 0.450409262 | 0.888533638 | 1.1834174 | up | mRNA |
| NM_002357 | MXD1 | 0.031739731 | 0.341807319 | 1.5957358 | up | mRNA |
| NM_002371 | MAL | 0.581130611 | 0.934005566 | 1.0211818 | up | mRNA |
| NM_002374 | MAP2 | 0.143133374 | 0.603147721 | 1.271551 | up | mRNA |
| NM_002376 | MARK3 | 0.478429274 | 0.901271944 | 1.0404825 | up | mRNA |
| NM_002381 | MATN3 | 0.873673383 | 0.990604909 | 1.0591513 | up | mRNA |
| NM_002387 | MCC | 0.015423576 | 0.249037809 | 1.9424026 | up | mRNA |
| NM_002389 | CD46 | 0.141993176 | 0.60204132 | 1.2223632 | up | mRNA |
| NM_002393 | MDM4 | 0.511153943 | 0.912481293 | 1.0529073 | up | mRNA |
| NM_002395 | ME1 | 0.663075968 | 0.958354006 | 1.1678451 | up | mRNA |
| NM_002398 | MEIS1 | 0.262688528 | 0.763125731 | 1.3585971 | up | mRNA |
| NM_002401 | MAP3K3 | 0.337998777 | 0.827394958 | 3.2966335 | up | mRNA |
| NM_002402 | MEST | 0.921210989 | 0.995349793 | 1.0112958 | up | mRNA |
| NM_002404 | MFAP4 | 0.146455529 | 0.608122555 | 1.078005 | up | mRNA |
| NM_002413 | MGST2 | 0.964533131 | 0.99793927 | 1.0122169 | up | mRNA |
| NM_002416 | CXCL9 | 0.387093714 | 0.856594404 | 1.1686664 | up | mRNA |
| NM_002419 | MAP3K11 | 0.018004903 | 0.269173581 | 1.6305465 | up | mRNA |
| NM_002421 | MMP1 | 0.021185514 | 0.288424269 | 5.2465025 | up | mRNA |
| NM_002422 | MMP3 | 0.001085031 | 0.086778252 | 42.4069445 | up | mRNA |
| NM_002425 | MMP10 | 6.01733E-05 | 0.030586101 | 18.2714864 | up | mRNA |
| NM_002426 | MMP12 | 0.086159541 | 0.498802898 | 2.6419612 | up | mRNA |
| NM_002427 | MMP13 | 0.190615878 | 0.676402188 | 1.315442 | up | mRNA |
| NM_002428 | MMP15 | 0.627047741 | 0.949218083 | 1.0673476 | up | mRNA |
| NM_002434 | MPG | 0.941888106 | 0.996710991 | 1.0061009 | up | mRNA |
| NM_002437 | MPV17 | 0.464613924 | 0.894989844 | 1.0438261 | up | mRNA |
| NM_002441 | MSH5 | 0.710005088 | 0.965462762 | 1.1302929 | up | mRNA |
| NM_002443 | MSMB | 0.360799349 | 0.841780454 | 1.4110694 | up | mRNA |
| NM_002444 | MSN | 0.032409996 | 0.343869895 | 1.2873579 | up | mRNA |
| NM_002447 | MST1R | 0.650714788 | 0.955894361 | 1.0176983 | up | mRNA |
| NM_002449 | MSX2 | 0.994470104 | 0.999872237 | 1.0004678 | up | mRNA |
| NM_002451 | MTAP | 0.324995505 | 0.817191271 | 1.0773873 | up | mRNA |
| NM_002456 | MUC1 | 0.886451424 | 0.992981836 | 1.0228203 | up | mRNA |
| NM_002461 | MVD | 0.210888042 | 0.701961023 | 1.1501795 | up | mRNA |
| NM_002468 | MYD88 | 0.664231061 | 0.958354006 | 1.0295908 | up | mRNA |
| NM_002470 | MYH3 | 0.870613181 | 0.989592579 | 1.0439259 | up | mRNA |
| NM_002475 | MYL6B | 0.024790238 | 0.307526004 | 1.185863 | up | mRNA |
| NM_002479 | MYOG | 0.00032543 | 0.056781055 | 2.9105834 | up | mRNA |
| NM_002482 | NASP | 0.693689522 | 0.961812388 | 1.0963413 | up | mRNA |
| NM_002483 | CEACAM6 | 0.926938846 | 0.995829678 | 1.0103431 | up | mRNA |
| NM_002484 | NUBP1 | 0.782766296 | 0.97711225 | 1.0178378 | up | mRNA |
| NM_002490 | NDUFA6 | 0.888961971 | 0.993470148 | 1.0071463 | up | mRNA |
| NM_002492 | NDUFB5 | 0.407919999 | 0.867591329 | 1.0945864 | up | mRNA |
| NM_002502 | NFKB2 | 0.000738383 | 0.079656701 | 2.5435067 | up | mRNA |
| NM_002503 | NFKBIB | 0.000175573 | 0.044621794 | 1.7654122 | up | mRNA |
| NM_002506 | NGF | 0.006357348 | 0.180328763 | 1.6140928 | up | mRNA |
| NM_002507 | NGFR | 0.371527933 | 0.846675738 | 1.1140119 | up | mRNA |
| NM_002508 | NID1 | 0.512918979 | 0.913297047 | 1.0594757 | up | mRNA |
| NM_002516 | NOVA2 | 0.524260318 | 0.917428132 | 1.042849 | up | mRNA |
| NM_002518 | NPAS2 | 0.004405362 | 0.152329639 | 1.3820744 | up | mRNA |
| NM_002519 | NPAT | 0.897594101 | 0.994326043 | 1.0142669 | up | mRNA |
| NM_002522 | NPTX1 | 0.508690532 | 0.911674633 | 1.8670636 | up | mRNA |
| NM_002523 | NPTX2 | 0.097019775 | 0.519517004 | 1.2356321 | up | mRNA |
| NM_002527 | NTF3 | 0.221763346 | 0.716734257 | 1.3006652 | up | mRNA |
| NM_002532 | NUP88 | 0.983230799 | 0.998971807 | 1.0040123 | up | mRNA |
| NM_002534 | OAS1 | 0.954229289 | 0.996710991 | 1.0272318 | up | mRNA |
| NM_002536 | TBC1D25 | 0.866783534 | 0.989481868 | 1.0438653 | up | mRNA |
| NM_002537 | OAZ2 | 0.005447999 | 0.168942963 | 1.2444942 | up | mRNA |
| NM_002541 | OGDH | 0.431740515 | 0.880121832 | 1.0565701 | up | mRNA |
| NM_002546 | TNFRSF11B | 0.309128741 | 0.803961878 | 1.4353333 | up | mRNA |
| NM_002557 | OVGP1 | 0.007914199 | 0.194808105 | 1.0551629 | up | mRNA |
| NM_002558 | P2RX1 | 0.625009727 | 0.948689643 | 1.0990563 | up | mRNA |
| NM_002559 | P2RX3 | 0.077876154 | 0.479375706 | 1.2411773 | up | mRNA |
| NM_002562 | P2RX7 | 0.974367456 | 0.998501532 | 1.0101183 | up | mRNA |
| NM_002569 | FURIN | 0.272273651 | 0.772141487 | 1.124656 | up | mRNA |
| NM_002581 | PAPPA | 0.623501811 | 0.948341211 | 1.1193244 | up | mRNA |
| NM_002585 | PBX1 | 0.479655946 | 0.901538317 | 1.0810561 | up | mRNA |
| NM_002587 | PCDH1 | 0.023867868 | 0.303389731 | 1.2333603 | up | mRNA |
| NM_002594 | PCSK2 | 0.619788825 | 0.947444797 | 1.1198994 | up | mRNA |
| NM_002596 | CDK18 | 0.906551907 | 0.99508468 | 1.0132564 | up | mRNA |
| NM_002600 | PDE4B | 0.560786442 | 0.927965324 | 1.1226045 | up | mRNA |
| NM_002601 | PDE6D | 0.939506965 | 0.996710991 | 1.0069618 | up | mRNA |
| NM_002605 | PDE8A | 0.674480218 | 0.959531882 | 1.0541573 | up | mRNA |
| NM_002606 | PDE9A | 0.674990823 | 0.959531882 | 1.0742734 | up | mRNA |
| NM_002607 | PDGFA | 0.021346721 | 0.288424269 | 1.3372059 | up | mRNA |
| NM_002615 | SERPINF1 | 0.807626576 | 0.98118927 | 1.0827299 | up | mRNA |
| NM_002617 | PEX10 | 0.509384172 | 0.911674633 | 1.0721071 | up | mRNA |
| NM_002619 | PF4 | 0.666657345 | 0.958354006 | 1.0486413 | up | mRNA |
| NM_002622 | PFDN1 | 0.5956536 | 0.938746918 | 1.0816489 | up | mRNA |
| NM_002623 | PFDN4 | 0.908957868 | 0.995333785 | 1.0269228 | up | mRNA |
| NM_002624 | PFDN5 | 0.884128008 | 0.992766819 | 1.0221045 | up | mRNA |
| NM_002626 | PFKL | 0.348257474 | 0.834138149 | 1.21683 | up | mRNA |
| NM_002627 | PFKP | 0.342102777 | 0.830550068 | 1.1651031 | up | mRNA |
| NM_002630 | PGC | 0.621663182 | 0.947997886 | 1.0713437 | up | mRNA |
| NM_002636 | PHF1 | 0.122818874 | 0.574983504 | 1.0794937 | up | mRNA |
| NM_002638 | PI3 | 0.895986881 | 0.994117613 | 1.0398029 | up | mRNA |
| NM_002643 | PIGF | 0.63032758 | 0.950410442 | 1.0606926 | up | mRNA |
| NM_002651 | PI4KB | 0.009353387 | 0.204533521 | 1.2381336 | up | mRNA |
| NM_002653 | PITX1 | 0.940747618 | 0.996710991 | 1.0542818 | up | mRNA |
| NM_002657 | PLAGL2 | 0.044315773 | 0.386873462 | 1.4132353 | up | mRNA |
| NM_002658 | PLAU | 0.230644512 | 0.728071705 | 1.529319 | up | mRNA |
| NM_002659 | PLAUR | 0.570079499 | 0.932828856 | 1.0618932 | up | mRNA |
| NM_002661 | PLCG2 | 0.253190457 | 0.753288068 | 1.1837802 | up | mRNA |
| NM_002665 | PLGLB2 | 0.60904461 | 0.943053241 | 1.1209695 | up | mRNA |
| NM_002667 | PLN | 0.010386163 | 0.209736088 | 1.2827192 | up | mRNA |
| NM_002675 | PML | 0.087678142 | 0.501313828 | 1.2890021 | up | mRNA |
| NM_002690 | POLB | 0.737001674 | 0.968834005 | 1.0455682 | up | mRNA |
| NM_002691 | POLD1 | 0.933481712 | 0.996710991 | 1.0115763 | up | mRNA |
| NM_002696 | POLR2G | 0.902960576 | 0.99508468 | 1.0087258 | up | mRNA |
| NM_002701 | POU5F1 | 0.69315901 | 0.961584652 | 1.0286246 | up | mRNA |
| NM_002703 | PPAT | 0.329956112 | 0.821768501 | 1.0706213 | up | mRNA |
| NM_002716 | PPP2R1B | 0.276826512 | 0.777075691 | 1.2494231 | up | mRNA |
| NM_002718 | PPP2R3A | 0.021183782 | 0.288424269 | 1.4684672 | up | mRNA |
| NM_002727 | SRGN | 0.033663295 | 0.348493947 | 1.8618977 | up | mRNA |
| NM_002729 | HHEX | 0.559213548 | 0.927203296 | 1.036633 | up | mRNA |
| NM_002730 | PRKACA | 0.279901917 | 0.778697234 | 1.0866341 | up | mRNA |
| NM_002732 | PRKACG | 0.282633644 | 0.780457321 | 1.1962661 | up | mRNA |
| NM_002735 | PRKAR1B | 0.501204307 | 0.909946064 | 1.0804296 | up | mRNA |
| NM_002748 | MAPK6 | 0.267066233 | 0.76755337 | 1.1171248 | up | mRNA |
| NM_002751 | MAPK11 | 0.546350742 | 0.92469697 | 1.0568979 | up | mRNA |
| NM_002753 | MAPK10 | 0.397066223 | 0.862344286 | 1.1330667 | up | mRNA |
| NM_002758 | MAP2K6 | 0.513520658 | 0.91350662 | 1.2234501 | up | mRNA |
| NM_002766 | PRPSAP1 | 0.590495845 | 0.936429414 | 1.0675762 | up | mRNA |
| NM_002769 | PRSS1 | 0.616461399 | 0.945912508 | 1.0799134 | up | mRNA |
| NM_002771 | PRSS3 | 0.034379121 | 0.351431016 | 1.1933101 | up | mRNA |
| NM_002772 | TMPRSS15 | 0.98524881 | 0.998971807 | 1.004017 | up | mRNA |
| NM_002773 | PRSS8 | 0.808376122 | 0.98118927 | 1.0312919 | up | mRNA |
| NM_002778 | PSAP | 0.996500292 | 0.999872237 | 1.0007098 | up | mRNA |
| NM_002782 | PSG6 | 0.140426654 | 0.600490836 | 1.1487278 | up | mRNA |
| NM_002786 | PSMA1 | 0.109156785 | 0.547155834 | 1.0551665 | up | mRNA |
| NM_002788 | PSMA3 | 0.191901922 | 0.67769911 | 1.1354116 | up | mRNA |
| NM_002790 | PSMA5 | 0.686976627 | 0.960205549 | 1.0313922 | up | mRNA |
| NM_002791 | PSMA6 | 0.729108958 | 0.966191059 | 1.0801728 | up | mRNA |
| NM_002795 | PSMB3 | 0.648880154 | 0.95550188 | 1.0354736 | up | mRNA |
| NM_002796 | PSMB4 | 0.702706416 | 0.963478781 | 1.0176751 | up | mRNA |
| NM_002799 | PSMB7 | 0.729798991 | 0.966191059 | 1.0239112 | up | mRNA |
| NM_002800 | PSMB9 | 0.009527499 | 0.204959264 | 1.283202 | up | mRNA |
| NM_002802 | PSMC1 | 0.355986491 | 0.838847221 | 1.1603607 | up | mRNA |
| NM_002809 | PSMD3 | 0.939908147 | 0.996710991 | 1.0103272 | up | mRNA |
| NM_002812 | PSMD8 | 0.848769854 | 0.987678166 | 1.0097228 | up | mRNA |
| NM_002818 | PSME2 | 0.001619557 | 0.103549765 | 1.3207215 | up | mRNA |
| NM_002820 | PTHLH | 0.018801283 | 0.274617595 | 2.7260953 | up | mRNA |
| NM_002823 | PTMA | 0.702037225 | 0.963247543 | 1.0448757 | up | mRNA |
| NM_002825 | PTN | 0.181856731 | 0.664924727 | 1.1531828 | up | mRNA |
| NM_002827 | PTPN1 | 0.005680315 | 0.170618233 | 2.4585605 | up | mRNA |
| NM_002828 | PTPN2 | 0.094850809 | 0.516057441 | 1.1657352 | up | mRNA |
| NM_002831 | PTPN6 | 0.771957474 | 0.975347132 | 1.071747 | up | mRNA |
| NM_002832 | PTPN7 | 0.9261062 | 0.995699344 | 1.0130579 | up | mRNA |
| NM_002834 | PTPN11 | 0.942449466 | 0.996710991 | 1.0039998 | up | mRNA |
| NM_002835 | PTPN12 | 0.070673162 | 0.459816555 | 1.3117022 | up | mRNA |
| NM_002836 | PTPRA | 0.033740291 | 0.348587681 | 1.2038609 | up | mRNA |
| NM_002838 | PTPRC | 0.869505011 | 0.989592579 | 1.0081148 | up | mRNA |
| NM_002839 | PTPRD | 0.981020553 | 0.998971807 | 1.0038113 | up | mRNA |
| NM_002841 | PTPRG | 0.633901099 | 0.951174475 | 1.0948124 | up | mRNA |
| NM_002849 | PTPRR | 0.129779231 | 0.583907255 | 1.3913546 | up | mRNA |
| NM_002851 | PTPRZ1 | 0.91710798 | 0.995349793 | 1.0222679 | up | mRNA |
| NM_002852 | PTX3 | 0.060626712 | 0.435971562 | 2.2835283 | up | mRNA |
| NM_002855 | PVRL1 | 0.692983438 | 0.961584652 | 1.0146895 | up | mRNA |
| NM_002868 | RAB5B | 0.1444386 | 0.605627057 | 1.129321 | up | mRNA |
| NM_002871 | RABIF | 0.793892325 | 0.97921643 | 1.1130997 | up | mRNA |
| NM_002872 | RAC2 | 0.287699986 | 0.783907278 | 1.1582991 | up | mRNA |
| NM_002882 | RANBP1 | 0.850053635 | 0.987678166 | 1.0400823 | up | mRNA |
| NM_002883 | RANGAP1 | 0.472172576 | 0.899115872 | 1.19007 | up | mRNA |
| NM_002888 | RARRES1 | 0.005485777 | 0.16971815 | 1.6458392 | up | mRNA |
| NM_002891 | RASGRF1 | 0.082668146 | 0.49074708 | 1.1695325 | up | mRNA |
| NM_002894 | RBBP8 | 0.760632876 | 0.972931992 | 1.0530347 | up | mRNA |
| NM_002904 | RDBP | 0.936568612 | 0.996710991 | 1.0069601 | up | mRNA |
| NM_002905 | RDH5 | 0.992171134 | 0.999743458 | 1.0030315 | up | mRNA |
| NM_002908 | REL | 0.006180763 | 0.178714838 | 7.0437262 | up | mRNA |
| NM_002921 | RGR | 0.890990793 | 0.9937521 | 1.0219928 | up | mRNA |
| NM_002923 | RGS2 | 0.118690839 | 0.56661708 | 1.8876684 | up | mRNA |
| NM_002925 | RGS10 | 0.144466419 | 0.605627057 | 1.1237122 | up | mRNA |
| NM_002928 | RGS16 | 0.001800694 | 0.105670067 | 25.213203 | up | mRNA |
| NM_002929 | GRK1 | 0.530620374 | 0.919193443 | 1.1047981 | up | mRNA |
| NM_002942 | ROBO2 | 0.280430954 | 0.778877286 | 1.2382725 | up | mRNA |
| NM_002943 | RORA | 0.149090931 | 0.612758601 | 1.2129199 | up | mRNA |
| NM_002947 | RPA3 | 0.911604213 | 0.995342392 | 1.0249943 | up | mRNA |
| NM_002952 | RPS2 | 0.389943647 | 0.858788371 | 1.0310796 | up | mRNA |
| NM_002959 | SORT1 | 0.967984205 | 0.99793927 | 1.0041111 | up | mRNA |
| NM_002966 | S100A10 | 0.576808334 | 0.933656777 | 1.0530184 | up | mRNA |
| NM_002970 | SAT1 | 0.005152808 | 0.164469214 | 2.0932092 | up | mRNA |
| NM_002979 | SCP2 | 0.936377026 | 0.996710991 | 1.012048 | up | mRNA |
| NM_002980 | SCTR | 0.677353385 | 0.959531882 | 1.0282805 | up | mRNA |
| NM_002981 | CCL1 | 0.110112481 | 0.549130968 | 1.1584124 | up | mRNA |
| NM_002982 | CCL2 | 0.001774821 | 0.105670067 | 5.228113 | up | mRNA |
| NM_002984 | CCL4 | 0.062259137 | 0.439379649 | 8.893865 | up | mRNA |
| NM_002985 | CCL5 | 0.970044123 | 0.998245244 | 1.0057264 | up | mRNA |
| NM_002986 | CCL11 | 0.041127582 | 0.375924711 | 1.159343 | up | mRNA |
| NM_002989 | CCL21 | 0.456338433 | 0.891568474 | 1.1498436 | up | mRNA |
| NM_002990 | CCL22 | 0.555750907 | 0.926833616 | 1.0571497 | up | mRNA |
| NM_002993 | CXCL6 | 0.001976889 | 0.107570744 | 15.5808341 | up | mRNA |
| NM_002994 | CXCL5 | 0.00037096 | 0.058467857 | 6.9497496 | up | mRNA |
| NM_002999 | SDC4 | 0.007719421 | 0.19345927 | 1.7634565 | up | mRNA |
| NM_003000 | SDHB | 0.50418017 | 0.910568383 | 1.1415333 | up | mRNA |
| NM_003001 | SDHC | 0.850567629 | 0.987678166 | 1.0242527 | up | mRNA |
| NM_003002 | SDHD | 0.67850779 | 0.959881742 | 1.0206509 | up | mRNA |
| NM_003006 | SELPLG | 0.437569359 | 0.882430095 | 1.0820829 | up | mRNA |
| NM_003009 | SEPW1 | 0.871407614 | 0.989857511 | 1.0161136 | up | mRNA |
| NM_003013 | SFRP2 | 0.959614889 | 0.997591263 | 1.0409707 | up | mRNA |
| NM_003014 | SFRP4 | 0.743493174 | 0.970330526 | 1.1040101 | up | mRNA |
| NM_003016 | SRSF2 | 0.375687991 | 0.849862269 | 1.0652363 | up | mRNA |
| NM_003019 | SFTPD | 0.47693094 | 0.900429473 | 1.1323887 | up | mRNA |
| NM_003022 | SH3BGRL | 0.957912969 | 0.996933183 | 1.0097804 | up | mRNA |
| NM_003023 | SH3BP2 | 0.475344357 | 0.899815707 | 1.1998013 | up | mRNA |
| NM_003028 | SHB | 0.008864939 | 0.202329498 | 1.8258164 | up | mRNA |
| NM_003034 | ST8SIA1 | 0.931903499 | 0.996710991 | 1.0213592 | up | mRNA |
| NM_003039 | SLC2A5 | 0.765191247 | 0.974315408 | 1.0915622 | up | mRNA |
| NM_003045 | SLC7A1 | 0.032042834 | 0.342454646 | 1.191516 | up | mRNA |
| NM_003047 | SLC9A1 | 0.367426539 | 0.845181673 | 1.1547691 | up | mRNA |
| NM_003052 | SLC34A1 | 0.37713975 | 0.850710713 | 1.111757 | up | mRNA |
| NM_003055 | SLC18A3 | 0.02107063 | 0.288296123 | 1.2257437 | up | mRNA |
| NM_003057 | SLC22A1 | 0.076560428 | 0.475659052 | 1.2044617 | up | mRNA |
| NM_003059 | SLC22A4 | 0.551774127 | 0.92621751 | 1.1108333 | up | mRNA |
| NM_003062 | SLIT3 | 0.999443321 | 0.999897336 | 1.0003347 | up | mRNA |
| NM_003068 | SNAI2 | 0.727874036 | 0.966191059 | 1.1959879 | up | mRNA |
| NM_003073 | SMARCB1 | 0.494264907 | 0.906903176 | 1.0567473 | up | mRNA |
| NM_003075 | SMARCC2 | 0.830176554 | 0.985549562 | 1.0306329 | up | mRNA |
| NM_003080 | SMPD2 | 0.688299664 | 0.96055024 | 1.0523887 | up | mRNA |
| NM_003081 | SNAP25 | 0.114646678 | 0.558779196 | 1.4089189 | up | mRNA |
| NM_003087 | SNCG | 0.40957642 | 0.867822547 | 1.0706572 | up | mRNA |
| NM_003093 | SNRPC | 0.969771004 | 0.998245244 | 1.0040515 | up | mRNA |
| NM_003094 | SNRPE | 0.81417926 | 0.98120545 | 1.029074 | up | mRNA |
| NM_003095 | SNRPF | 0.687288017 | 0.960205549 | 1.031 | up | mRNA |
| NM_003096 | SNRPG | 0.874239885 | 0.990793509 | 1.0194038 | up | mRNA |
| NM_003104 | SORD | 0.954135458 | 0.996710991 | 1.0121235 | up | mRNA |
| NM_003109 | SP1 | 0.960532023 | 0.997779446 | 1.0134618 | up | mRNA |
| NM_003114 | SPAG1 | 0.057302439 | 0.426454314 | 1.5423034 | up | mRNA |
| NM_003115 | UAP1 | 0.021781229 | 0.290701669 | 1.3448274 | up | mRNA |
| NM_003117 | SPAM1 | 0.829152886 | 0.98552522 | 1.0450094 | up | mRNA |
| NM_003121 | SPIB | 0.771743653 | 0.975347132 | 1.0395578 | up | mRNA |
| NM_003122 | SPINK1 | 0.352583741 | 0.835946719 | 1.6048075 | up | mRNA |
| NM_003124 | SPR | 0.436010002 | 0.881677506 | 1.0171115 | up | mRNA |
| NM_003128 | SPTBN1 | 0.14222594 | 0.602445379 | 1.1451902 | up | mRNA |
| NM_003130 | SRI | 0.811163813 | 0.98118927 | 1.0246416 | up | mRNA |
| NM_003137 | SRPK1 | 0.12037084 | 0.571550658 | 1.155942 | up | mRNA |
| NM_003139 | SRPR | 0.216378685 | 0.711073448 | 1.1774699 | up | mRNA |
| NM_003141 | TRIM21 | 0.037836245 | 0.363212377 | 1.3571807 | up | mRNA |
| NM_003150 | STAT3 | 0.007644614 | 0.193257016 | 1.4195004 | up | mRNA |
| NM_003151 | STAT4 | 0.280060291 | 0.778697234 | 1.9061258 | up | mRNA |
| NM_003152 | STAT5A | 9.50821E-06 | 0.014870836 | 2.3390196 | up | mRNA |
| NM_003157 | NEK4 | 0.188312826 | 0.673545538 | 1.1364287 | up | mRNA |
| NM_003164 | STX5 | 0.685210977 | 0.960111041 | 1.0338076 | up | mRNA |
| NM_003170 | SUPT6H | 0.051156286 | 0.40916979 | 1.4835789 | up | mRNA |
| NM_003172 | SURF1 | 0.339671283 | 0.828117387 | 1.7696303 | up | mRNA |
| NM_003174 | SVIL | 0.248450013 | 0.746819289 | 1.1100314 | up | mRNA |
| NM_003176 | SYCP1 | 0.115671586 | 0.561475615 | 1.8317642 | up | mRNA |
| NM_003177 | SYK | 0.710062781 | 0.965462762 | 1.0301414 | up | mRNA |
| NM_003181 | T | 0.638433264 | 0.952916247 | 1.0311141 | up | mRNA |
| NM_003182 | TAC1 | 0.390904309 | 0.858920947 | 1.659202 | up | mRNA |
| NM_003183 | ADAM17 | 0.366983462 | 0.845181673 | 1.1775285 | up | mRNA |
| NM_003189 | TAL1 | 0.777035069 | 0.976821563 | 1.0280757 | up | mRNA |
| NM_003190 | TAPBP | 0.001930338 | 0.107205943 | 1.47702 | up | mRNA |
| NM_003196 | TCEA3 | 0.975284344 | 0.998501532 | 1.018817 | up | mRNA |
| NM_003204 | NFE2L1 | 0.372146938 | 0.847311483 | 1.1382504 | up | mRNA |
| NM_003212 | TDGF1 | 0.274263445 | 0.774381942 | 1.4422256 | up | mRNA |
| NM_003221 | TFAP2B | 0.796333638 | 0.980562956 | 1.0321975 | up | mRNA |
| NM_003224 | ARFRP1 | 0.560489211 | 0.92782421 | 1.0236401 | up | mRNA |
| NM_003234 | TFRC | 0.912572245 | 0.995342392 | 1.0129998 | up | mRNA |
| NM_003239 | TGFB3 | 0.935228904 | 0.996710991 | 1.0179313 | up | mRNA |
| NM_003242 | TGFBR2 | 0.105152045 | 0.536769112 | 1.0580619 | up | mRNA |
| NM_003243 | TGFBR3 | 0.356273748 | 0.838932592 | 1.0726834 | up | mRNA |
| NM_003248 | THBS4 | 0.018681732 | 0.273264013 | 1.2323494 | up | mRNA |
| NM_003254 | TIMP1 | 0.034596579 | 0.35197585 | 1.1178542 | up | mRNA |
| NM_003255 | TIMP2 | 0.799198417 | 0.980917337 | 1.0308892 | up | mRNA |
| NM_003263 | TLR1 | 0.005393532 | 0.168450524 | 1.2637779 | up | mRNA |
| NM_003265 | TLR3 | 0.509727781 | 0.911674633 | 1.063932 | up | mRNA |
| NM_003273 | TM7SF2 | 0.456748084 | 0.891568474 | 1.1536816 | up | mRNA |
| NM_003278 | CLEC3B | 0.77705646 | 0.976821563 | 1.1202967 | up | mRNA |
| NM_003280 | TNNC1 | 0.347005315 | 0.833567116 | 1.1192069 | up | mRNA |
| NM_003281 | TNNI1 | 0.965006786 | 0.99793927 | 1.0056497 | up | mRNA |
| NM_003286 | TOP1 | 0.167515499 | 0.642346587 | 1.2917872 | up | mRNA |
| NM_003287 | TPD52L1 | 0.767424342 | 0.975033576 | 1.2436466 | up | mRNA |
| NM_003291 | TPP2 | 0.56606382 | 0.930638763 | 1.0323564 | up | mRNA |
| NM_003294 | TPSAB1 | 0.755574089 | 0.971497464 | 1.0311174 | up | mRNA |
| NM_003295 | TPT1 | 0.799925581 | 0.981152656 | 1.0400879 | up | mRNA |
| NM_003300 | TRAF3 | 0.058268865 | 0.429558128 | 1.4285133 | up | mRNA |
| NM_003304 | TRPC1 | 0.856853487 | 0.988512246 | 1.0539883 | up | mRNA |
| NM_003311 | PHLDA2 | 0.043157173 | 0.383508423 | 1.3237003 | up | mRNA |
| NM_003313 | TSTA3 | 0.852191737 | 0.987678166 | 1.0118943 | up | mRNA |
| NM_003322 | TULP1 | 0.369091295 | 0.84565745 | 1.1502109 | up | mRNA |
| NM_003324 | TULP3 | 0.998450047 | 0.999889342 | 1.0002436 | up | mRNA |
| NM_003326 | TNFSF4 | 0.912050848 | 0.995342392 | 1.0179792 | up | mRNA |
| NM_003327 | TNFRSF4 | 6.55967E-05 | 0.031755073 | 2.8086841 | up | mRNA |
| NM_003331 | TYK2 | 0.213228915 | 0.705626675 | 1.3642317 | up | mRNA |
| NM_003332 | TYROBP | 0.260543725 | 0.761124374 | 1.1727864 | up | mRNA |
| NM_003333 | UBA52 | 0.458286103 | 0.892667725 | 1.0537047 | up | mRNA |
| NM_003335 | UBA7 | 0.894583135 | 0.993978977 | 1.0320167 | up | mRNA |
| NM_003337 | UBE2B | 0.383129144 | 0.853514109 | 1.0597825 | up | mRNA |
| NM_003341 | UBE2E1 | 0.085898008 | 0.498103045 | 1.1633746 | up | mRNA |
| NM_003343 | UBE2G2 | 0.530547889 | 0.919193443 | 1.0320252 | up | mRNA |
| NM_003344 | UBE2H | 0.653557517 | 0.956875599 | 1.0422415 | up | mRNA |
| NM_003355 | UCP2 | 0.499243981 | 0.909361611 | 1.1724618 | up | mRNA |
| NM_003358 | UGCG | 0.912721859 | 0.995342392 | 1.0241677 | up | mRNA |
| NM_003364 | UPP1 | 0.47916359 | 0.901538317 | 1.2451694 | up | mRNA |
| NM_003369 | UVRAG | 0.062890573 | 0.439491843 | 1.0780502 | up | mRNA |
| NM_003374 | VDAC1 | 0.800162431 | 0.98118927 | 1.0437545 | up | mRNA |
| NM_003377 | VEGFB | 0.966779638 | 0.99793927 | 1.0057341 | up | mRNA |
| NM_003380 | VIM | 0.493837513 | 0.906630188 | 1.0292851 | up | mRNA |
| NM_003391 | WNT2 | 0.223119971 | 0.71761506 | 1.0947194 | up | mRNA |
| NM_003395 | WNT9A | 0.85427657 | 0.987950129 | 1.1551392 | up | mRNA |
| NM_003399 | XPNPEP2 | 0.343994295 | 0.831639952 | 1.2557991 | up | mRNA |
| NM_003407 | ZFP36 | 0.116821709 | 0.5636495 | 1.2687397 | up | mRNA |
| NM_003410 | ZFX | 0.090816606 | 0.508828962 | 1.455557 | up | mRNA |
| NM_003411 | ZFY | 0.088677347 | 0.503065796 | 1.5021823 | up | mRNA |
| NM_003413 | ZIC3 | 0.349706397 | 0.834138149 | 1.1899004 | up | mRNA |
| NM_003414 | ZNF267 | 0.003005762 | 0.130211341 | 2.1968796 | up | mRNA |
| NM_003417 | ZNF264 | 0.813112959 | 0.98120545 | 1.0341528 | up | mRNA |
| NM_003420 | ZNF35 | 0.948230446 | 0.996710991 | 1.0085967 | up | mRNA |
| NM_003426 | ZNF74 | 0.183847493 | 0.667497719 | 1.2505085 | up | mRNA |
| NM_003427 | ZNF76 | 0.461531256 | 0.894667841 | 1.0831683 | up | mRNA |
| NM_003437 | ZNF136 | 0.602382954 | 0.941406011 | 1.0759129 | up | mRNA |
| NM_003439 | ZKSCAN1 | 0.606029167 | 0.94174007 | 1.0219734 | up | mRNA |
| NM_003440 | ZNF140 | 0.794258927 | 0.979253685 | 1.0527017 | up | mRNA |
| NM_003443 | ZBTB17 | 0.360905279 | 0.841780454 | 1.1679673 | up | mRNA |
| NM_003447 | ZNF165 | 0.362701996 | 0.843238468 | 1.1685936 | up | mRNA |
| NM_003451 | ZNF177 | 0.903162602 | 0.99508468 | 1.0108552 | up | mRNA |
| NM_003452 | ZNF189 | 0.050209584 | 0.405586514 | 1.2074315 | up | mRNA |
| NM_003459 | SLC30A3 | 0.960030355 | 0.997738713 | 1.0092145 | up | mRNA |
| NM_003465 | CHIT1 | 0.757270842 | 0.971960783 | 1.0735189 | up | mRNA |
| NM_003468 | FZD5 | 0.93074974 | 0.996710991 | 1.0152924 | up | mRNA |
| NM_003475 | RASSF7 | 0.612955656 | 0.944423643 | 1.0570305 | up | mRNA |
| NM_003480 | MFAP5 | 0.859736562 | 0.988752971 | 1.0970936 | up | mRNA |
| NM_003481 | USP5 | 0.815672203 | 0.981459009 | 1.0333406 | up | mRNA |
| NM_003485 | GPR68 | 0.595276935 | 0.938707475 | 1.0757939 | up | mRNA |
| NM_003486 | SLC7A5 | 0.265643264 | 0.766537754 | 1.380323 | up | mRNA |
| NM_003492 | TMEM187 | 0.775350209 | 0.97589756 | 1.0409011 | up | mRNA |
| NM_003498 | SNN | 0.002647186 | 0.122061602 | 1.7356513 | up | mRNA |
| NM_003504 | CDC45 | 0.944935314 | 0.996710991 | 1.0564202 | up | mRNA |
| NM_003506 | FZD6 | 0.270331387 | 0.770199084 | 1.0971532 | up | mRNA |
| NM_003511 | HIST1H2AL | 0.828124274 | 0.985325387 | 1.1696728 | up | mRNA |
| NM_003523 | HIST1H2BE | 0.480713369 | 0.901538317 | 1.1158924 | up | mRNA |
| NM_003540 | HIST1H4F | 0.630615695 | 0.950410442 | 1.0749018 | up | mRNA |
| NM_003543 | HIST1H4H | 0.727095544 | 0.966191059 | 1.0323823 | up | mRNA |
| NM_003550 | MAD1L1 | 0.801653637 | 0.98118927 | 1.0931385 | up | mRNA |
| NM_003551 | NME5 | 0.411038355 | 0.868434057 | 1.0851221 | up | mRNA |
| NM_003557 | PIP5K1A | 0.16045073 | 0.631980676 | 1.1842187 | up | mRNA |
| NM_003564 | TAGLN2 | 0.707782095 | 0.965436846 | 1.0307947 | up | mRNA |
| NM_003565 | ULK1 | 0.256279671 | 0.757240077 | 1.0927416 | up | mRNA |
| NM_003567 | BCAR3 | 0.009032472 | 0.202329498 | 1.3550411 | up | mRNA |
| NM_003568 | ANXA9 | 0.343643593 | 0.831536584 | 1.1700282 | up | mRNA |
| NM_003573 | LTBP4 | 0.891186726 | 0.9937521 | 1.0236037 | up | mRNA |
| NM_003574 | VAPA | 0.690425181 | 0.961105466 | 1.0360397 | up | mRNA |
| NM_003578 | SOAT2 | 0.389376059 | 0.858189054 | 1.0756508 | up | mRNA |
| NM_003579 | RAD54L | 0.81247405 | 0.98118927 | 1.0834551 | up | mRNA |
| NM_003582 | DYRK3 | 0.041849217 | 0.378824525 | 1.5366251 | up | mRNA |
| NM_003598 | TEAD2 | 0.718574018 | 0.966191059 | 1.084535 | up | mRNA |
| NM_003601 | SMARCA5 | 0.048479169 | 0.399707405 | 1.1654393 | up | mRNA |
| NM_003603 | SORBS2 | 0.080905646 | 0.486246998 | 1.3015553 | up | mRNA |
| NM_003607 | CDC42BPA | 0.66310252 | 0.958354006 | 1.0409683 | up | mRNA |
| NM_003612 | SEMA7A | 0.944793201 | 0.996710991 | 1.0100538 | up | mRNA |
| NM_003615 | SLC4A7 | 0.514172737 | 0.913526716 | 1.2277501 | up | mRNA |
| NM_003617 | RGS5 | 0.612800133 | 0.944398719 | 1.1338728 | up | mRNA |
| NM_003621 | PPFIBP2 | 0.935923369 | 0.996710991 | 1.0134048 | up | mRNA |
| NM_003624 | RANBP3 | 0.41993571 | 0.872365335 | 1.0979528 | up | mRNA |
| NM_003625 | PPFIA2 | 0.561528884 | 0.928513767 | 1.1039444 | up | mRNA |
| NM_003626 | PPFIA1 | 0.565273732 | 0.930020145 | 1.0754265 | up | mRNA |
| NM_003629 | PIK3R3 | 0.000884263 | 0.083478018 | 1.4219525 | up | mRNA |
| NM_003632 | CNTNAP1 | 0.601465175 | 0.941048131 | 1.0779922 | up | mRNA |
| NM_003633 | ENC1 | 0.083833561 | 0.493058711 | 2.7099782 | up | mRNA |
| NM_003634 | NIPSNAP1 | 0.452219755 | 0.888854908 | 1.1655337 | up | mRNA |
| NM_003641 | IFITM1 | 0.613124143 | 0.944540087 | 1.2115081 | up | mRNA |
| NM_003644 | GAS7 | 0.98595785 | 0.998971807 | 1.0016256 | up | mRNA |
| NM_003645 | SLC27A2 | 0.041200696 | 0.376152917 | 1.7345224 | up | mRNA |
| NM_003654 | CHST1 | 0.991532263 | 0.999495983 | 1.0010955 | up | mRNA |
| NM_003659 | AGPS | 0.420131192 | 0.872365335 | 1.1226179 | up | mRNA |
| NM_003671 | CDC14B | 0.713061821 | 0.966110889 | 1.0594698 | up | mRNA |
| NM_003672 | CDC14A | 0.290576139 | 0.786596721 | 1.057046 | up | mRNA |
| NM_003676 | DEGS1 | 0.857593856 | 0.988512246 | 1.020019 | up | mRNA |
| NM_003684 | MKNK1 | 0.615176169 | 0.945266163 | 1.0431433 | up | mRNA |
| NM_003685 | KHSRP | 0.481999479 | 0.902092906 | 1.085176 | up | mRNA |
| NM_003686 | EXO1 | 0.746525642 | 0.970432947 | 1.1743403 | up | mRNA |
| NM_003687 | PDLIM4 | 0.01429561 | 0.241161654 | 1.177872 | up | mRNA |
| NM_003688 | CASK | 0.412865433 | 0.869353641 | 1.1090401 | up | mRNA |
| NM_003692 | TMEFF1 | 0.795152044 | 0.979701559 | 1.0301906 | up | mRNA |
| NM_003695 | LY6D | 0.410370191 | 0.86807846 | 1.2280309 | up | mRNA |
| NM_003705 | SLC25A12 | 0.500795111 | 0.909680616 | 1.0871639 | up | mRNA |
| NM_003706 | PLA2G4C | 0.733876955 | 0.967777568 | 1.1470099 | up | mRNA |
| NM_003707 | RUVBL1 | 0.605616926 | 0.94174007 | 1.0490097 | up | mRNA |
| NM_003709 | KLF7 | 0.084900857 | 0.495565119 | 1.3647109 | up | mRNA |
| NM_003711 | PPAP2A | 0.701670642 | 0.963236071 | 1.0622473 | up | mRNA |
| NM_003712 | PPAP2C | 0.761463625 | 0.973287132 | 1.0503026 | up | mRNA |
| NM_003715 | USO1 | 0.188839636 | 0.674422532 | 1.1279763 | up | mRNA |
| NM_003724 | JRK | 0.125284551 | 0.578441772 | 1.1620724 | up | mRNA |
| NM_003728 | UNC5C | 0.551451478 | 0.926149092 | 1.2194235 | up | mRNA |
| NM_003733 | OASL | 0.921331183 | 0.995349793 | 1.0191812 | up | mRNA |
| NM_003734 | AOC3 | 0.151216812 | 0.616500055 | 1.0967356 | up | mRNA |
| NM_003735 | PCDHGA12 | 0.985731068 | 0.998971807 | 1.0030971 | up | mRNA |
| NM_003739 | AKR1C3 | 0.711622065 | 0.96592341 | 1.1930461 | up | mRNA |
| NM_003744 | NUMB | 0.028366167 | 0.327830694 | 1.5702065 | up | mRNA |
| NM_003750 | EIF3A | 0.994611081 | 0.999872237 | 1.0006517 | up | mRNA |
| NM_003754 | EIF3F | 0.494406107 | 0.906930718 | 1.0773746 | up | mRNA |
| NM_003758 | EIF3J | 0.689566602 | 0.96108227 | 1.0245146 | up | mRNA |
| NM_003760 | EIF4G3 | 0.593238036 | 0.938070909 | 1.0397011 | up | mRNA |
| NM_003761 | VAMP8 | 0.262112687 | 0.763125731 | 1.0735974 | up | mRNA |
| NM_003768 | PEA15 | 0.985934505 | 0.998971807 | 1.00136 | up | mRNA |
| NM_003774 | GALNT4 | 0.18543469 | 0.669014284 | 1.1419434 | up | mRNA |
| NM_003775 | S1PR4 | 0.374211826 | 0.849321997 | 1.0776928 | up | mRNA |
| NM_003776 | MRPL40 | 0.339804464 | 0.828118322 | 1.1104738 | up | mRNA |
| NM_003786 | ABCC3 | 0.577743744 | 0.933815962 | 1.2416419 | up | mRNA |
| NM_003791 | MBTPS1 | 0.334326507 | 0.824174306 | 1.0659233 | up | mRNA |
| NM_003798 | CTNNAL1 | 0.036858545 | 0.359628653 | 1.137065 | up | mRNA |
| NM_003799 | RNMT | 0.65698012 | 0.957445617 | 1.0285814 | up | mRNA |
| NM_003805 | CRADD | 0.681950765 | 0.960111041 | 1.0265842 | up | mRNA |
| NM_003808 | TNFSF13 | 0.156521112 | 0.625223427 | 1.0982769 | up | mRNA |
| NM_003815 | ADAM15 | 0.82598959 | 0.984306256 | 1.0188434 | up | mRNA |
| NM_003818 | CDS2 | 0.900895716 | 0.994789506 | 1.0165228 | up | mRNA |
| NM_003820 | TNFRSF14 | 0.912920476 | 0.995342392 | 1.023567 | up | mRNA |
| NM_003821 | RIPK2 | 0.000644949 | 0.076684828 | 5.4350477 | up | mRNA |
| NM_003825 | SNAP23 | 0.461995176 | 0.894754673 | 1.0499969 | up | mRNA |
| NM_003828 | MTMR1 | 0.721595896 | 0.966191059 | 1.0318689 | up | mRNA |
| NM_003830 | SIGLEC5 | 0.983074073 | 0.998971807 | 1.0011433 | up | mRNA |
| NM_003842 | TNFRSF10B | 0.000360555 | 0.057900793 | 2.1341064 | up | mRNA |
| NM_003845 | DYRK4 | 0.265083168 | 0.766340249 | 1.1471728 | up | mRNA |
| NM_003846 | PEX11B | 0.530422842 | 0.919086179 | 1.037733 | up | mRNA |
| NM_003854 | IL1RL2 | 0.028017529 | 0.325701768 | 1.1407333 | up | mRNA |
| NM_003855 | IL18R1 | 0.010226062 | 0.20936306 | 2.8491025 | up | mRNA |
| NM_003856 | IL1RL1 | 0.935628487 | 0.996710991 | 1.0548936 | up | mRNA |
| NM_003859 | DPM1 | 0.423153181 | 0.873989281 | 1.0562925 | up | mRNA |
| NM_003867 | FGF17 | 0.199653396 | 0.688843178 | 1.0458494 | up | mRNA |
| NM_003870 | IQGAP1 | 0.775332843 | 0.97589756 | 1.040086 | up | mRNA |
| NM_003872 | NRP2 | 0.000911405 | 0.084230411 | 2.2129333 | up | mRNA |
| NM_003873 | NRP1 | 0.174296377 | 0.652872869 | 1.0668404 | up | mRNA |
| NM_003874 | CD84 | 0.76696505 | 0.975033576 | 1.0497879 | up | mRNA |
| NM_003877 | SOCS2 | 0.577641545 | 0.933787388 | 1.0611226 | up | mRNA |
| NM_003878 | GGH | 0.610086419 | 0.943169861 | 1.0732694 | up | mRNA |
| NM_003879 | CFLAR | 8.04342E-05 | 0.034070596 | 3.4015517 | up | mRNA |
| NM_003880 | WISP3 | 0.153607214 | 0.620667112 | 1.0991542 | up | mRNA |
| NM_003882 | WISP1 | 0.457149125 | 0.891668841 | 1.0401252 | up | mRNA |
| NM_003885 | CDK5R1 | 0.390828023 | 0.858920947 | 1.1648044 | up | mRNA |
| NM_003887 | ASAP2 | 0.107818941 | 0.544052509 | 1.206455 | up | mRNA |
| NM_003890 | FCGBP | 0.104386628 | 0.535011073 | 1.3671422 | up | mRNA |
| NM_003891 | PROZ | 0.009886581 | 0.207085174 | 1.1536874 | up | mRNA |
| NM_003896 | ST3GAL5 | 0.802085453 | 0.98118927 | 1.0233119 | up | mRNA |
| NM_003897 | IER3 | 0.000156617 | 0.044621794 | 4.2619899 | up | mRNA |
| NM_003898 | SYNJ2 | 0.592864684 | 0.937845232 | 1.1176918 | up | mRNA |
| NM_003900 | SQSTM1 | 0.462416572 | 0.894903269 | 1.2987346 | up | mRNA |
| NM_003901 | SGPL1 | 0.011455767 | 0.218702965 | 1.4420853 | up | mRNA |
| NM_003916 | AP1S2 | 0.180359985 | 0.663244568 | 1.131818 | up | mRNA |
| NM_003919 | SGCE | 0.970481201 | 0.998245244 | 1.0072178 | up | mRNA |
| NM_003930 | SKAP2 | 0.877722435 | 0.991306146 | 1.0205395 | up | mRNA |
| NM_003932 | ST13 | 0.602910664 | 0.941464987 | 1.10457 | up | mRNA |
| NM_003937 | KYNU | 0.933671466 | 0.996710991 | 1.1464325 | up | mRNA |
| NM_003940 | USP13 | 0.461798322 | 0.894728749 | 1.0869948 | up | mRNA |
| NM_003944 | SELENBP1 | 0.913968626 | 0.995342392 | 1.0660593 | up | mRNA |
| NM_003945 | ATP6V0E1 | 0.285275336 | 0.781963593 | 1.0753056 | up | mRNA |
| NM_003948 | CDKL2 | 0.382753617 | 0.853514109 | 1.1233371 | up | mRNA |
| NM_003951 | SLC25A14 | 0.528694534 | 0.918883792 | 1.1080567 | up | mRNA |
| NM_003955 | SOCS3 | 0.126018258 | 0.578441772 | 2.8950905 | up | mRNA |
| NM_003959 | HIP1R | 0.901014664 | 0.994789506 | 1.026716 | up | mRNA |
| NM_003971 | SPAG9 | 0.063928234 | 0.441804507 | 1.3925367 | up | mRNA |
| NM_003973 | RPL14 | 0.495295186 | 0.907415174 | 1.0392236 | up | mRNA |
| NM_003979 | GPRC5A | 0.043855979 | 0.385351177 | 1.5520286 | up | mRNA |
| NM_003980 | MAP7 | 0.942690771 | 0.996710991 | 1.0226314 | up | mRNA |
| NM_003987 | PAX2 | 0.253891189 | 0.754108603 | 1.1368543 | up | mRNA |
| NM_004000 | CHI3L2 | 0.357255328 | 0.839643099 | 1.1783737 | up | mRNA |
| NM_004013 | DMD | 0.113899477 | 0.557835051 | 1.1385664 | up | mRNA |
| NM_004015 | DMD | 0.782887197 | 0.977203173 | 1.0431196 | up | mRNA |
| NM_004018 | DMD | 0.789156741 | 0.977807732 | 1.0260277 | up | mRNA |
| NM_004027 | INPP4A | 0.951319423 | 0.996710991 | 1.0045734 | up | mRNA |
| NM_004034 | ANXA7 | 0.575961059 | 0.933656777 | 1.0767499 | up | mRNA |
| NM_004035 | ACOX1 | 0.014713593 | 0.24380986 | 1.3574314 | up | mRNA |
| NM_004038 | AMY1A | 0.966257348 | 0.99793927 | 1.0017834 | up | mRNA |
| NM_004041 | ARRB1 | 0.208117827 | 0.698793461 | 1.2486414 | up | mRNA |
| NM_004045 | ATOX1 | 0.132692976 | 0.588376689 | 1.0573957 | up | mRNA |
| NM_004049 | BCL2A1 | 2.97329E-05 | 0.02325109 | 8.3974812 | up | mRNA |
| NM_004053 | BYSL | 0.16833721 | 0.643478528 | 1.0781537 | up | mRNA |
| NM_004055 | CAPN5 | 0.824134903 | 0.983568283 | 1.0335404 | up | mRNA |
| NM_004064 | CDKN1B | 0.455604321 | 0.891148383 | 1.1442255 | up | mRNA |
| NM_004067 | CHN2 | 0.000474592 | 0.066547663 | 1.4093335 | up | mRNA |
| NM_004071 | CLK1 | 0.087299491 | 0.500533133 | 1.2392891 | up | mRNA |
| NM_004073 | PLK3 | 0.000156573 | 0.044621794 | 3.0285519 | up | mRNA |
| NM_004081 | DAZ1 | 0.329556075 | 0.821768501 | 1.1009127 | up | mRNA |
| NM_004086 | COCH | 0.377525673 | 0.851053291 | 1.3434435 | up | mRNA |
| NM_004090 | DUSP3 | 0.026943935 | 0.319244809 | 1.2265506 | up | mRNA |
| NM_004091 | E2F2 | 0.290160667 | 0.786468159 | 1.514652 | up | mRNA |
| NM_004102 | FABP3 | 0.78528876 | 0.977411899 | 1.0125514 | up | mRNA |
| NM_004103 | PTK2B | 0.948056942 | 0.996710991 | 1.0022939 | up | mRNA |
| NM_004112 | FGF11 | 0.313299153 | 0.808636478 | 1.0505885 | up | mRNA |
| NM_004113 | FGF12 | 0.713296429 | 0.966110889 | 1.0779602 | up | mRNA |
| NM_004120 | GBP2 | 0.090123564 | 0.507386748 | 3.3243243 | up | mRNA |
| NM_004122 | GHSR | 0.622778187 | 0.948341211 | 1.0889662 | up | mRNA |
| NM_004126 | GNG11 | 0.477749255 | 0.900908722 | 1.0683075 | up | mRNA |
| NM_004132 | HABP2 | 0.38505133 | 0.855147437 | 1.0547859 | up | mRNA |
| NM_004137 | KCNMB1 | 0.812503286 | 0.98118927 | 1.0279284 | up | mRNA |
| NM_004140 | LLGL1 | 0.306145998 | 0.801520161 | 1.0830461 | up | mRNA |
| NM_004146 | NDUFB7 | 0.649047912 | 0.95550188 | 1.0120566 | up | mRNA |
| NM_004164 | RBP2 | 0.098229831 | 0.521464473 | 1.4081988 | up | mRNA |
| NM_004165 | RRAD | 0.00738165 | 0.190938402 | 4.4981135 | up | mRNA |
| NM_004168 | SDHA | 0.221864107 | 0.716818852 | 1.0522908 | up | mRNA |
| NM_004171 | SLC1A2 | 0.9271307 | 0.995829678 | 1.0133457 | up | mRNA |
| NM_004182 | UXT | 0.475996435 | 0.900275303 | 1.0725231 | up | mRNA |
| NM_004183 | BEST1 | 0.869532683 | 0.989592579 | 1.0365343 | up | mRNA |
| NM_004195 | TNFRSF18 | 0.018140286 | 0.269217741 | 1.2460913 | up | mRNA |
| NM_004196 | CDKL1 | 0.641660806 | 0.954332328 | 1.0635619 | up | mRNA |
| NM_004198 | CHRNA6 | 0.51536764 | 0.914319064 | 1.1328215 | up | mRNA |
| NM_004200 | SYT7 | 0.662411382 | 0.958354006 | 1.1094597 | up | mRNA |
| NM_004202 | TMSB4Y | 0.899693909 | 0.994708735 | 1.0284994 | up | mRNA |
| NM_004211 | SLC6A5 | 0.310207763 | 0.805036317 | 1.2850502 | up | mRNA |
| NM_004217 | AURKB | 0.672812061 | 0.959531882 | 1.0431124 | up | mRNA |
| NM_004219 | PTTG1 | 0.994309493 | 0.999872237 | 1.0028772 | up | mRNA |
| NM_004220 | ZNF213 | 0.855212923 | 0.988178307 | 1.0170906 | up | mRNA |
| NM_004221 | IL32 | 0.000583615 | 0.073247308 | 3.310154 | up | mRNA |
| NM_004225 | MFHAS1 | 0.033752241 | 0.348587681 | 1.4264357 | up | mRNA |
| NM_004226 | STK17B | 0.096936453 | 0.519207575 | 1.1793887 | up | mRNA |
| NM_004228 | CYTH2 | 0.69023901 | 0.961105466 | 1.0239645 | up | mRNA |
| NM_004231 | ATP6V1F | 0.365895385 | 0.845164582 | 1.048411 | up | mRNA |
| NM_004233 | CD83 | 0.510374511 | 0.912097614 | 1.0572827 | up | mRNA |
| NM_004240 | TRIP10 | 0.068745288 | 0.455138128 | 1.2242204 | up | mRNA |
| NM_004242 | HMGN3 | 0.240740611 | 0.739051504 | 1.0729392 | up | mRNA |
| NM_004246 | GLP2R | 0.400029464 | 0.864237287 | 1.1629678 | up | mRNA |
| NM_004258 | CD101 | 0.195107219 | 0.682833964 | 1.1645377 | up | mRNA |
| NM_004263 | SEMA4F | 0.409089631 | 0.867591329 | 1.4441867 | up | mRNA |
| NM_004271 | LY86 | 0.321423691 | 0.815220381 | 1.0519878 | up | mRNA |
| NM_004273 | CHST3 | 0.564638075 | 0.929798441 | 1.0821881 | up | mRNA |
| NM_004277 | SLC25A27 | 0.860888646 | 0.989013989 | 1.0536024 | up | mRNA |
| NM_004285 | H6PD | 0.777916354 | 0.976938981 | 1.0493795 | up | mRNA |
| NM_004286 | GTPBP1 | 0.104348744 | 0.535011073 | 1.1502016 | up | mRNA |
| NM_004287 | GOSR2 | 0.795149664 | 0.979701559 | 1.0507875 | up | mRNA |
| NM_004292 | RIN1 | 0.513712447 | 0.91350662 | 1.1954159 | up | mRNA |
| NM_004299 | ABCB7 | 0.996403609 | 0.999872237 | 1.0003232 | up | mRNA |
| NM_004302 | ACVR1B | 0.756516325 | 0.971789861 | 1.0391221 | up | mRNA |
| NM_004307 | APBB2 | 0.739489817 | 0.969564098 | 1.0275931 | up | mRNA |
| NM_004322 | BAD | 0.369684038 | 0.846215286 | 1.3360488 | up | mRNA |
| NM_004332 | BPHL | 0.696165104 | 0.962410025 | 1.0404612 | up | mRNA |
| NM_004333 | BRAF | 0.438419122 | 0.882830305 | 1.061876 | up | mRNA |
| NM_004339 | PTTG1IP | 0.966349479 | 0.99793927 | 1.006953 | up | mRNA |
| NM_004344 | CETN2 | 0.504366822 | 0.910568383 | 1.0752444 | up | mRNA |
| NM_004355 | CD74 | 0.258516082 | 0.75933964 | 1.1711876 | up | mRNA |
| NM_004364 | CEBPA | 0.531102552 | 0.919715279 | 1.1151246 | up | mRNA |
| NM_004365 | CETN3 | 0.862267215 | 0.989401142 | 1.0181407 | up | mRNA |
| NM_004368 | CNN2 | 0.938893411 | 0.996710991 | 1.0077428 | up | mRNA |
| NM_004371 | COPA | 0.870224816 | 0.989592579 | 1.0175691 | up | mRNA |
| NM_004374 | COX6C | 0.265711741 | 0.766537754 | 1.0810808 | up | mRNA |
| NM_004388 | CTBS | 0.716867561 | 0.966191059 | 1.0455884 | up | mRNA |
| NM_004394 | DAP | 0.538762885 | 0.923577434 | 1.0234439 | up | mRNA |
| NM_004396 | DDX5 | 0.987669485 | 0.999140676 | 1.0012848 | up | mRNA |
| NM_004406 | DMBT1 | 0.41620118 | 0.870736736 | 1.352157 | up | mRNA |
| NM_004408 | DNM1 | 0.945691209 | 0.996710991 | 1.0101406 | up | mRNA |
| NM_004409 | DMPK | 0.650454902 | 0.955894361 | 1.0344743 | up | mRNA |
| NM_004411 | DYNC1I1 | 0.463646852 | 0.894989844 | 1.2042742 | up | mRNA |
| NM_004414 | RCAN1 | 0.001843103 | 0.105670067 | 1.9701803 | up | mRNA |
| NM_004417 | DUSP1 | 0.420387822 | 0.872365335 | 1.2047337 | up | mRNA |
| NM_004419 | DUSP5 | 0.130165105 | 0.584037049 | 1.9924971 | up | mRNA |
| NM_004421 | DVL1 | 0.674243551 | 0.959531882 | 1.0683145 | up | mRNA |
| NM_004422 | DVL2 | 0.982359762 | 0.998971807 | 1.0023819 | up | mRNA |
| NM_004426 | PHC1 | 0.103859574 | 0.533896783 | 1.0606196 | up | mRNA |
| NM_004427 | PHC2 | 0.059369386 | 0.432586127 | 1.8180139 | up | mRNA |
| NM_004428 | EFNA1 | 0.101714144 | 0.529590777 | 1.668458 | up | mRNA |
| NM_004430 | EGR3 | 0.385555319 | 0.85515381 | 1.5127725 | up | mRNA |
| NM_004436 | ENSA | 0.601559443 | 0.941056135 | 1.0655585 | up | mRNA |
| NM_004439 | EPHA5 | 0.471795859 | 0.898908705 | 1.0843899 | up | mRNA |
| NM_004449 | ERG | 0.786757249 | 0.977479172 | 1.122522 | up | mRNA |
| NM_004454 | ETV5 | 0.329628631 | 0.821768501 | 1.0645385 | up | mRNA |
| NM_004455 | EXTL1 | 0.578848206 | 0.933821303 | 1.0638971 | up | mRNA |
| NM_004456 | EZH2 | 0.582568512 | 0.934005566 | 1.2755683 | up | mRNA |
| NM_004457 | ACSL3 | 0.284111919 | 0.78179061 | 1.3341458 | up | mRNA |
| NM_004458 | ACSL4 | 0.344627312 | 0.8319832 | 1.0990384 | up | mRNA |
| NM_004464 | FGF5 | 0.083253445 | 0.491989753 | 1.9555124 | up | mRNA |
| NM_004475 | FLOT2 | 0.259488468 | 0.759999933 | 1.18851 | up | mRNA |
| NM_004477 | FRG1 | 0.944147501 | 0.996710991 | 1.011161 | up | mRNA |
| NM_004483 | GCSH | 0.471773273 | 0.898908705 | 1.0794383 | up | mRNA |
| NM_004487 | GOLGB1 | 0.635986501 | 0.951814727 | 1.0507303 | up | mRNA |
| NM_004494 | HDGF | 0.312563166 | 0.807809113 | 1.1214356 | up | mRNA |
| NM_004496 | FOXA1 | 0.2278369 | 0.723854154 | 1.1882617 | up | mRNA |
| NM_004497 | FOXA3 | 0.665228613 | 0.958354006 | 1.024141 | up | mRNA |
| NM_004501 | HNRNPU | 0.216841994 | 0.711074992 | 1.1014043 | up | mRNA |
| NM_004502 | HOXB7 | 9.94014E-05 | 0.036966313 | 1.8309262 | up | mRNA |
| NM_004509 | SP110 | 0.159530746 | 0.631415053 | 1.083811 | up | mRNA |
| NM_004510 | SP110 | 0.945507768 | 0.996710991 | 1.0072869 | up | mRNA |
| NM_004513 | IL16 | 0.523825158 | 0.917348245 | 1.1293407 | up | mRNA |
| NM_004519 | KCNQ3 | 0.671311382 | 0.959531882 | 1.1073935 | up | mRNA |
| NM_004520 | KIF2A | 0.196987687 | 0.685161614 | 1.2409371 | up | mRNA |
| NM_004527 | MEOX1 | 0.514679703 | 0.913840427 | 1.208315 | up | mRNA |
| NM_004528 | MGST3 | 0.54624505 | 0.9245966 | 1.0537578 | up | mRNA |
| NM_004530 | MMP2 | 0.401649668 | 0.864768873 | 1.1170339 | up | mRNA |
| NM_004542 | NDUFA3 | 0.268339409 | 0.768988803 | 1.0409494 | up | mRNA |
| NM_004543 | NEB | 0.422198718 | 0.87361534 | 1.0768497 | up | mRNA |
| NM_004545 | NDUFB1 | 0.464248001 | 0.894989844 | 1.0491264 | up | mRNA |
| NM_004547 | NDUFB4 | 0.067081042 | 0.451445852 | 1.1256438 | up | mRNA |
| NM_004549 | NDUFC2 | 0.701000507 | 0.962991468 | 1.0377613 | up | mRNA |
| NM_004553 | NDUFS6 | 0.214716443 | 0.707887861 | 1.1093684 | up | mRNA |
| NM_004556 | NFKBIE | 0.001782676 | 0.105670067 | 3.6494967 | up | mRNA |
| NM_004558 | NRTN | 0.687736647 | 0.960243201 | 1.0587834 | up | mRNA |
| NM_004565 | PEX14 | 0.864474308 | 0.989401142 | 1.0385942 | up | mRNA |
| NM_004566 | PFKFB3 | 0.018242381 | 0.26991728 | 1.9201879 | up | mRNA |
| NM_004567 | PFKFB4 | 0.117813647 | 0.564437853 | 1.337636 | up | mRNA |
| NM_004571 | PKNOX1 | 0.401802579 | 0.864786348 | 1.0431108 | up | mRNA |
| NM_004584 | RAD9A | 0.410125917 | 0.86807846 | 1.0634264 | up | mRNA |
| NM_004585 | RARRES3 | 0.05787558 | 0.428369209 | 1.2156624 | up | mRNA |
| NM_004586 | RPS6KA3 | 0.027449413 | 0.322334413 | 1.3838157 | up | mRNA |
| NM_004588 | SCN2B | 0.996352417 | 0.999872237 | 1.0005313 | up | mRNA |
| NM_004591 | CCL20 | 0.000201933 | 0.048302273 | 43.3646913 | up | mRNA |
| NM_004594 | SLC9A5 | 0.408861422 | 0.867591329 | 1.076805 | up | mRNA |
| NM_004595 | SMS | 0.062647693 | 0.439458294 | 1.2565954 | up | mRNA |
| NM_004596 | SNRPA | 0.279474706 | 0.777968199 | 1.3223307 | up | mRNA |
| NM_004598 | SPOCK1 | 0.623196535 | 0.948341211 | 1.0853775 | up | mRNA |
| NM_004602 | STAU1 | 0.116766556 | 0.563517119 | 1.0918576 | up | mRNA |
| NM_004604 | STX4 | 0.034868691 | 0.352338753 | 1.2983446 | up | mRNA |
| NM_004605 | SULT2B1 | 0.396739097 | 0.862081791 | 1.0339468 | up | mRNA |
| NM_004607 | TBCA | 0.897860991 | 0.994326043 | 1.0156884 | up | mRNA |
| NM_004612 | TGFBR1 | 0.505430735 | 0.910772598 | 1.1219945 | up | mRNA |
| NM_004615 | TSPAN7 | 0.671738972 | 0.959531882 | 1.0718472 | up | mRNA |
| NM_004618 | TOP3A | 0.691559072 | 0.961451327 | 1.0475659 | up | mRNA |
| NM_004625 | WNT7A | 0.224028153 | 0.718105062 | 1.4235842 | up | mRNA |
| NM_004630 | SF1 | 0.774269391 | 0.97589756 | 1.0252271 | up | mRNA |
| NM_004636 | SEMA3B | 0.849057815 | 0.987678166 | 1.0508106 | up | mRNA |
| NM_004639 | BAG6 | 0.874812332 | 0.991012053 | 1.0254301 | up | mRNA |
| NM_004656 | BAP1 | 0.394144695 | 0.861008404 | 1.0729756 | up | mRNA |
| NM_004657 | SDPR | 0.369430808 | 0.846072048 | 1.1440383 | up | mRNA |
| NM_004658 | RASAL1 | 0.039700118 | 0.370777582 | 1.1601973 | up | mRNA |
| NM_004662 | DNAH9 | 0.700069636 | 0.962991468 | 1.0638793 | up | mRNA |
| NM_004665 | VNN2 | 0.279513794 | 0.777970495 | 1.217077 | up | mRNA |
| NM_004667 | HERC2 | 0.54781443 | 0.92514258 | 1.0819874 | up | mRNA |
| NM_004670 | PAPSS2 | 0.139173422 | 0.598648178 | 1.2995259 | up | mRNA |
| NM_004672 | MAP3K6 | 0.111956856 | 0.554156842 | 1.3407872 | up | mRNA |
| NM_004673 | ANGPTL1 | 0.642987775 | 0.954522001 | 1.0453559 | up | mRNA |
| NM_004675 | DIRAS3 | 0.539889796 | 0.923577434 | 1.157883 | up | mRNA |
| NM_004683 | RGN | 0.666637518 | 0.958354006 | 1.0618404 | up | mRNA |
| NM_004686 | MTMR7 | 0.517403943 | 0.91546111 | 1.0531947 | up | mRNA |
| NM_004688 | NMI | 0.041909127 | 0.378877886 | 1.3984093 | up | mRNA |
| NM_004693 | KRT75 | 0.618127415 | 0.946938411 | 1.1320609 | up | mRNA |
| NM_004694 | SLC16A6 | 0.319816103 | 0.813931781 | 1.2348347 | up | mRNA |
| NM_004696 | SLC16A4 | 0.008876247 | 0.202329498 | 1.1461596 | up | mRNA |
| NM_004698 | PRPF3 | 0.671610915 | 0.959531882 | 1.0564772 | up | mRNA |
| NM_004705 | PRKRIR | 0.933087217 | 0.996710991 | 1.0037443 | up | mRNA |
| NM_004708 | PDCD5 | 0.708063289 | 0.965462762 | 1.0363074 | up | mRNA |
| NM_004712 | HGS | 0.357643129 | 0.839676686 | 1.1347391 | up | mRNA |
| NM_004717 | DGKI | 0.159677238 | 0.631516339 | 1.3576434 | up | mRNA |
| NM_004723 | ARHGEF2 | 0.002137586 | 0.110028846 | 1.8691393 | up | mRNA |
| NM_004728 | DDX21 | 0.006393076 | 0.180561804 | 1.2142116 | up | mRNA |
| NM_004734 | DCLK1 | 0.000720976 | 0.078885568 | 2.327314 | up | mRNA |
| NM_004735 | LRRFIP1 | 0.218178295 | 0.712269655 | 1.1551493 | up | mRNA |
| NM_004736 | XPR1 | 0.829872583 | 0.985549562 | 1.0422157 | up | mRNA |
| NM_004739 | MTA2 | 0.246269849 | 0.744874529 | 1.125032 | up | mRNA |
| NM_004741 | NOLC1 | 0.033316408 | 0.346667968 | 1.2031438 | up | mRNA |
| NM_004742 | MAGI1 | 0.853952126 | 0.987855862 | 1.0522957 | up | mRNA |
| NM_004747 | DLG5 | 0.775268322 | 0.97589756 | 1.0241071 | up | mRNA |
| NM_004749 | TBRG4 | 0.848170714 | 0.987678166 | 1.0319528 | up | mRNA |
| NM_004758 | BZRAP1 | 0.10117303 | 0.528804637 | 1.1981433 | up | mRNA |
| NM_004759 | MAPKAPK2 | 0.749896791 | 0.970725371 | 1.0841184 | up | mRNA |
| NM_004764 | PIWIL1 | 0.003100961 | 0.131960624 | 1.4040909 | up | mRNA |
| NM_004766 | COPB2 | 0.938884684 | 0.996710991 | 1.0032579 | up | mRNA |
| NM_004767 | GPR37L1 | 0.671155882 | 0.959531882 | 1.0368242 | up | mRNA |
| NM_004768 | SRSF11 | 0.678024258 | 0.959531882 | 1.0226403 | up | mRNA |
| NM_004776 | B4GALT5 | 0.16083376 | 0.63226359 | 1.6144885 | up | mRNA |
| NM_004783 | TAOK2 | 0.670125983 | 0.959531882 | 1.0190096 | up | mRNA |
| NM_004787 | SLIT2 | 0.695644343 | 0.96236244 | 1.1026264 | up | mRNA |
| NM_004789 | LHX2 | 0.159318685 | 0.631041393 | 1.1816336 | up | mRNA |
| NM_004794 | RAB33A | 0.950352438 | 0.996710991 | 1.0179756 | up | mRNA |
| NM_004798 | KIF3B | 0.236665159 | 0.73465251 | 1.4375584 | up | mRNA |
| NM_004803 | SLC22A14 | 0.057460177 | 0.42700171 | 1.3684302 | up | mRNA |
| NM_004811 | LPXN | 0.023293798 | 0.300022844 | 1.1876426 | up | mRNA |
| NM_004817 | TJP2 | 0.165806676 | 0.639669852 | 1.2521576 | up | mRNA |
| NM_004820 | CYP7B1 | 0.221551507 | 0.716734257 | 1.0965727 | up | mRNA |
| NM_004824 | CDYL | 0.644072107 | 0.954605197 | 1.0533238 | up | mRNA |
| NM_004827 | ABCG2 | 0.331215191 | 0.821853462 | 1.1655924 | up | mRNA |
| NM_004832 | GSTO1 | 0.922890618 | 0.995349793 | 1.0114607 | up | mRNA |
| NM_004845 | PCYT1B | 0.652660578 | 0.95670626 | 1.0385727 | up | mRNA |
| NM_004846 | EIF4E2 | 0.935212221 | 0.996710991 | 1.005976 | up | mRNA |
| NM_004851 | NAPSA | 0.147453193 | 0.609973207 | 1.2042544 | up | mRNA |
| NM_004853 | STX8 | 0.571163773 | 0.933029103 | 1.0188626 | up | mRNA |
| NM_004862 | LITAF | 0.100410301 | 0.526713683 | 1.2885419 | up | mRNA |
| NM_004877 | GMFG | 0.375494285 | 0.849862269 | 1.0960665 | up | mRNA |
| NM_004878 | PTGES | 0.021251187 | 0.288424269 | 1.6407254 | up | mRNA |
| NM_004887 | CXCL14 | 0.334252503 | 0.824174306 | 1.2851422 | up | mRNA |
| NM_004889 | ATP5J2 | 0.023924162 | 0.303389731 | 1.2094691 | up | mRNA |
| NM_004892 | SEC22B | 0.603499106 | 0.941484604 | 1.053279 | up | mRNA |
| NM_004894 | C14orf2 | 0.359343566 | 0.841033672 | 1.1095667 | up | mRNA |
| NM_004897 | MINPP1 | 0.037799926 | 0.363212377 | 1.2253727 | up | mRNA |
| NM_004899 | BRE | 0.86943776 | 0.989592579 | 1.0357139 | up | mRNA |
| NM_004905 | PRDX6 | 0.717211858 | 0.966191059 | 1.0678312 | up | mRNA |
| NM_004906 | WTAP | 0.000383111 | 0.05991852 | 3.8742671 | up | mRNA |
| NM_004907 | IER2 | 0.0686483 | 0.454916653 | 1.5675164 | up | mRNA |
| NM_004918 | TCL1B | 0.801255184 | 0.98118927 | 1.0627024 | up | mRNA |
| NM_004922 | SEC24C | 0.647878732 | 0.95550188 | 1.0399369 | up | mRNA |
| NM_004924 | ACTN4 | 0.38893075 | 0.85800274 | 1.0682769 | up | mRNA |
| NM_004926 | ZFP36L1 | 0.172154612 | 0.6499464 | 1.4804165 | up | mRNA |
| NM_004936 | CDKN2B | 0.271999887 | 0.771957246 | 1.3039235 | up | mRNA |
| NM_004938 | DAPK1 | 0.350721885 | 0.834911159 | 1.0944013 | up | mRNA |
| NM_004943 | DMWD | 0.470054596 | 0.898057982 | 1.0881883 | up | mRNA |
| NM_004947 | DOCK3 | 0.349745609 | 0.834138149 | 1.4282446 | up | mRNA |
| NM_004954 | MARK2 | 0.503367174 | 0.91053927 | 1.0722312 | up | mRNA |
| NM_004957 | FPGS | 0.849460781 | 0.987678166 | 1.0621397 | up | mRNA |
| NM_004958 | MTOR | 0.905164086 | 0.99508468 | 1.0091658 | up | mRNA |
| NM_004970 | IGFALS | 0.143395369 | 0.60362622 | 1.1447462 | up | mRNA |
| NM_004973 | JARID2 | 0.016410338 | 0.255922225 | 1.8578244 | up | mRNA |
| NM_004975 | KCNB1 | 0.797597157 | 0.980721189 | 1.0483766 | up | mRNA |
| NM_004980 | KCND3 | 0.82720426 | 0.984825466 | 1.0809698 | up | mRNA |
| NM_004992 | MECP2 | 0.055221619 | 0.419634622 | 1.1399047 | up | mRNA |
| NM_004998 | MYO1E | 0.732175809 | 0.967416074 | 1.0340204 | up | mRNA |
| NM_004999 | MYO6 | 0.832959549 | 0.986434891 | 1.0255508 | up | mRNA |
| NM_005003 | NDUFAB1 | 0.798391562 | 0.980749101 | 1.0336385 | up | mRNA |
| NM_005005 | NDUFB9 | 0.177074907 | 0.657106589 | 1.0953673 | up | mRNA |
| NM_005008 | NHP2L1 | 0.81806016 | 0.981818735 | 1.0082891 | up | mRNA |
| NM_005013 | NUCB2 | 0.380363346 | 0.853407568 | 1.070431 | up | mRNA |
| NM_005014 | OMD | 0.621632603 | 0.947997886 | 1.2408821 | up | mRNA |
| NM_005015 | OXA1L | 0.229546108 | 0.726627973 | 1.2106618 | up | mRNA |
| NM_005017 | PCYT1A | 0.587515141 | 0.93579682 | 1.0308793 | up | mRNA |
| NM_005021 | ENPP3 | 0.176980282 | 0.656875337 | 1.2823295 | up | mRNA |
| NM_005026 | PIK3CD | 0.358975996 | 0.840767187 | 1.1090731 | up | mRNA |
| NM_005029 | PITX3 | 0.821523209 | 0.982772998 | 1.0278273 | up | mRNA |
| NM_005034 | POLR2K | 0.814275647 | 0.98120545 | 1.0185205 | up | mRNA |
| NM_005044 | PRKX | 0.784276486 | 0.977347603 | 1.0427078 | up | mRNA |
| NM_005050 | ABCD4 | 0.282838711 | 0.780627498 | 1.2169446 | up | mRNA |
| NM_005063 | SCD | 0.980514642 | 0.998971807 | 1.0106812 | up | mRNA |
| NM_005066 | SFPQ | 0.698529692 | 0.962701213 | 1.0436659 | up | mRNA |
| NM_005067 | SIAH2 | 0.088043718 | 0.501570433 | 1.2919463 | up | mRNA |
| NM_005068 | SIM1 | 0.126949512 | 0.580406758 | 1.2833812 | up | mRNA |
| NM_005070 | SLC4A3 | 0.298904831 | 0.795233272 | 1.1547153 | up | mRNA |
| NM_005073 | SLC15A1 | 0.17816403 | 0.658983275 | 1.5361783 | up | mRNA |
| NM_005074 | SLC17A1 | 0.224015867 | 0.718105062 | 1.1111463 | up | mRNA |
| NM_005076 | CNTN2 | 0.729832815 | 0.966191059 | 1.050079 | up | mRNA |
| NM_005077 | TLE1 | 0.06936517 | 0.456861883 | 1.7237806 | up | mRNA |
| NM_005078 | TLE3 | 0.817450349 | 0.981727075 | 1.1280588 | up | mRNA |
| NM_005080 | XBP1 | 0.07132554 | 0.461586415 | 1.4363178 | up | mRNA |
| NM_005082 | TRIM25 | 0.04486437 | 0.38849334 | 1.2800662 | up | mRNA |
| NM_005086 | SSPN | 0.263022035 | 0.763125731 | 1.0882025 | up | mRNA |
| NM_005087 | FXR1 | 0.918864127 | 0.995349793 | 1.009425 | up | mRNA |
| NM_005089 | ZRSR2 | 0.921263686 | 0.995349793 | 1.020306 | up | mRNA |
| NM_005098 | MSC | 0.001773362 | 0.105670067 | 2.5798556 | up | mRNA |
| NM_005100 | AKAP12 | 0.74752444 | 0.970620903 | 1.3058282 | up | mRNA |
| NM_005101 | ISG15 | 0.114482166 | 0.558534358 | 1.0948324 | up | mRNA |
| NM_005103 | FEZ1 | 0.535147945 | 0.921242952 | 1.0550787 | up | mRNA |
| NM_005108 | XYLB | 0.754534662 | 0.97114634 | 1.0420449 | up | mRNA |
| NM_005110 | GFPT2 | 0.066679382 | 0.450526863 | 1.9286079 | up | mRNA |
| NM_005115 | MVP | 0.269241834 | 0.76973577 | 1.1426913 | up | mRNA |
| NM_005118 | TNFSF15 | 0.720642811 | 0.966191059 | 1.448383 | up | mRNA |
| NM_005119 | THRAP3 | 0.613704214 | 0.944789436 | 1.0596232 | up | mRNA |
| NM_005120 | MED12 | 0.667588544 | 0.958354006 | 1.0377713 | up | mRNA |
| NM_005127 | CLEC2B | 0.973793785 | 0.998501532 | 1.0334987 | up | mRNA |
| NM_005128 | DOPEY2 | 0.643947256 | 0.954605197 | 1.081579 | up | mRNA |
| NM_005130 | FGFBP1 | 0.85095945 | 0.987678166 | 1.1925566 | up | mRNA |
| NM_005132 | REC8 | 0.976206594 | 0.998501532 | 1.0034715 | up | mRNA |
| NM_005133 | RCE1 | 0.42287371 | 0.873989281 | 1.0966993 | up | mRNA |
| NM_005138 | SCO2 | 0.006545385 | 0.182886161 | 1.2023316 | up | mRNA |
| NM_005139 | ANXA3 | 0.333747671 | 0.824014287 | 1.4275643 | up | mRNA |
| NM_005140 | CNGA2 | 0.826784489 | 0.984490906 | 1.0129422 | up | mRNA |
| NM_005153 | USP10 | 0.16500888 | 0.638373173 | 1.0555207 | up | mRNA |
| NM_005155 | PPT2 | 0.988349475 | 0.999140676 | 1.0017094 | up | mRNA |
| NM_005157 | ABL1 | 0.06145357 | 0.437893235 | 1.0985199 | up | mRNA |
| NM_005175 | ATP5G1 | 0.810842875 | 0.98118927 | 1.0401695 | up | mRNA |
| NM_005177 | ATP6V0A1 | 0.64065904 | 0.953894897 | 1.0455994 | up | mRNA |
| NM_005178 | BCL3 | 0.724400315 | 0.966191059 | 1.0334214 | up | mRNA |
| NM_005186 | CAPN1 | 0.155368774 | 0.623056705 | 1.3119665 | up | mRNA |
| NM_005187 | CBFA2T3 | 0.744720205 | 0.970330526 | 1.054346 | up | mRNA |
| NM_005191 | CD80 | 0.891476813 | 0.9937521 | 1.0095301 | up | mRNA |
| NM_005195 | CEBPD | 0.000338733 | 0.05739273 | 5.0596 | up | mRNA |
| NM_005204 | MAP3K8 | 0.023675085 | 0.302362963 | 17.3403326 | up | mRNA |
| NM_005210 | CRYGB | 0.62351698 | 0.948341211 | 1.150535 | up | mRNA |
| NM_005212 | CSN3 | 0.043452171 | 0.384598968 | 1.1917495 | up | mRNA |
| NM_005213 | CSTA | 0.762914008 | 0.97349057 | 1.0610496 | up | mRNA |
| NM_005214 | CTLA4 | 0.694969096 | 0.962214902 | 1.0459428 | up | mRNA |
| NM_005219 | DIAPH1 | 0.273935225 | 0.774088254 | 1.0974902 | up | mRNA |
| NM_005221 | DLX5 | 0.04564614 | 0.39096051 | 2.3812182 | up | mRNA |
| NM_005224 | ARID3A | 0.602919526 | 0.941464987 | 1.0984947 | up | mRNA |
| NM_005225 | E2F1 | 0.804474412 | 0.98118927 | 1.1216767 | up | mRNA |
| NM_005226 | S1PR3 | 0.572276432 | 0.933397932 | 1.0787168 | up | mRNA |
| NM_005228 | EGFR | 0.269477446 | 0.769905254 | 1.1246977 | up | mRNA |
| NM_005229 | ELK1 | 0.105549977 | 0.537720406 | 1.246299 | up | mRNA |
| NM_005230 | ELK3 | 0.003105289 | 0.131960624 | 1.341421 | up | mRNA |
| NM_005231 | CTTN | 0.440418478 | 0.883544565 | 1.131726 | up | mRNA |
| NM_005235 | ERBB4 | 0.611914887 | 0.943964604 | 1.0902669 | up | mRNA |
| NM_005238 | ETS1 | 0.00593342 | 0.175346348 | 1.8530218 | up | mRNA |
| NM_005239 | ETS2 | 0.044029406 | 0.385988468 | 1.4878482 | up | mRNA |
| NM_005240 | ETV3 | 0.057686196 | 0.427646201 | 1.4859062 | up | mRNA |
| NM_005250 | FOXL1 | 0.029975078 | 0.335897836 | 1.4316267 | up | mRNA |
| NM_005251 | FOXC2 | 0.087824991 | 0.501417299 | 2.0753308 | up | mRNA |
| NM_005252 | FOS | 0.43274556 | 0.88061258 | 1.5601748 | up | mRNA |
| NM_005254 | GABPB1 | 0.043428528 | 0.384577017 | 1.3781831 | up | mRNA |
| NM_005255 | GAK | 0.027132543 | 0.320017762 | 1.1728226 | up | mRNA |
| NM_005258 | GCHFR | 0.947330324 | 0.996710991 | 1.0209757 | up | mRNA |
| NM_005259 | MSTN | 0.96769107 | 0.99793927 | 1.0420972 | up | mRNA |
| NM_005262 | GFER | 0.435030513 | 0.881331247 | 1.2615471 | up | mRNA |
| NM_005264 | GFRA1 | 0.709592902 | 0.965462762 | 1.0551441 | up | mRNA |
| NM_005274 | GNG5 | 0.871171222 | 0.98985097 | 1.0108787 | up | mRNA |
| NM_005275 | GNL1 | 0.648413955 | 0.95550188 | 1.046393 | up | mRNA |
| NM_005282 | GPR4 | 0.563081753 | 0.929041598 | 1.0471809 | up | mRNA |
| NM_005284 | GPR6 | 0.727626996 | 0.966191059 | 1.0564122 | up | mRNA |
| NM_005285 | NPBWR1 | 0.614059163 | 0.944827645 | 1.0514585 | up | mRNA |
| NM_005290 | GPR15 | 0.880019591 | 0.992197209 | 1.0277744 | up | mRNA |
| NM_005293 | GPR20 | 0.985624719 | 0.998971807 | 1.0015559 | up | mRNA |
| NM_005296 | LPAR4 | 0.453400769 | 0.889563297 | 1.0963904 | up | mRNA |
| NM_005297 | MCHR1 | 0.836052037 | 0.986575022 | 1.0697249 | up | mRNA |
| NM_005298 | GPR25 | 0.645072303 | 0.954828387 | 1.1343197 | up | mRNA |
| NM_005304 | FFAR3 | 0.777632564 | 0.976938981 | 1.1241523 | up | mRNA |
| NM_005309 | GPT | 0.04430576 | 0.386873462 | 1.3412757 | up | mRNA |
| NM_005317 | GZMM | 0.823538048 | 0.983498765 | 1.0526023 | up | mRNA |
| NM_005324 | H3F3B | 0.524179366 | 0.917428132 | 1.0648164 | up | mRNA |
| NM_005327 | HADH | 0.407493419 | 0.867591329 | 1.0390168 | up | mRNA |
| NM_005329 | HAS3 | 0.055702748 | 0.421334923 | 1.3307921 | up | mRNA |
| NM_005332 | HBZ | 0.569196387 | 0.93232103 | 1.0658911 | up | mRNA |
| NM_005335 | HCLS1 | 0.294822708 | 0.791301949 | 1.3170179 | up | mRNA |
| NM_005336 | HDLBP | 0.725547113 | 0.966191059 | 1.0218733 | up | mRNA |
| NM_005337 | NCKAP1L | 0.038993828 | 0.368411952 | 1.2011417 | up | mRNA |
| NM_005338 | HIP1 | 0.8296105 | 0.985549562 | 1.031606 | up | mRNA |
| NM_005345 | HSPA1A | 0.006578128 | 0.183214382 | 1.7678681 | up | mRNA |
| NM_005348 | HSP90AA1 | 0.842622774 | 0.987190965 | 1.0244878 | up | mRNA |
| NM_005355 | KIF25 | 0.331346377 | 0.82187807 | 1.1932472 | up | mRNA |
| NM_005360 | MAF | 0.209553629 | 0.700367256 | 1.0837302 | up | mRNA |
| NM_005371 | METTL1 | 0.099103093 | 0.523009123 | 1.1779923 | up | mRNA |
| NM_005375 | MYB | 0.003644569 | 0.140237595 | 7.0326952 | up | mRNA |
| NM_005378 | MYCN | 0.520117127 | 0.916394575 | 1.1124667 | up | mRNA |
| NM_005380 | NBL1 | 0.633930398 | 0.951174475 | 1.0921338 | up | mRNA |
| NM_005386 | NNAT | 0.713882452 | 0.966110889 | 1.0407602 | up | mRNA |
| NM_005391 | PDK3 | 0.550900207 | 0.925960004 | 1.1155092 | up | mRNA |
| NM_005393 | PLXNB3 | 0.761362971 | 0.973219662 | 1.024965 | up | mRNA |
| NM_005409 | CXCL11 | 0.200020849 | 0.68900633 | 1.1466534 | up | mRNA |
| NM_005416 | SPRR3 | 0.820122815 | 0.982556462 | 1.0460196 | up | mRNA |
| NM_005418 | ST5 | 0.674842064 | 0.959531882 | 1.040279 | up | mRNA |
| NM_005419 | STAT2 | 0.943403623 | 0.996710991 | 1.0116445 | up | mRNA |
| NM_005421 | TAL2 | 0.901779841 | 0.994906068 | 1.0260447 | up | mRNA |
| NM_005425 | TNP2 | 0.373249623 | 0.848112577 | 1.1113188 | up | mRNA |
| NM_005426 | TP53BP2 | 0.000351155 | 0.057900793 | 2.0053167 | up | mRNA |
| NM_005427 | TP73 | 0.709181038 | 0.965462762 | 1.0418118 | up | mRNA |
| NM_005431 | XRCC2 | 0.904191799 | 0.99508468 | 1.0134014 | up | mRNA |
| NM_005432 | XRCC3 | 0.489611187 | 0.904897251 | 1.0662644 | up | mRNA |
| NM_005433 | YES1 | 0.554507663 | 0.926549129 | 1.0628718 | up | mRNA |
| NM_005435 | ARHGEF5 | 0.37085271 | 0.846330273 | 1.1143167 | up | mRNA |
| NM_005438 | FOSL1 | 0.009115712 | 0.203095117 | 1.7639247 | up | mRNA |
| NM_005440 | RND2 | 0.29791434 | 0.794295449 | 1.1396154 | up | mRNA |
| NM_005442 | EOMES | 0.402406077 | 0.864786348 | 1.2180146 | up | mRNA |
| NM_005444 | RQCD1 | 0.955960712 | 0.996883425 | 1.0023929 | up | mRNA |
| NM_005446 | P2RX6 | 0.661511456 | 0.958354006 | 1.2004176 | up | mRNA |
| NM_005447 | RASSF9 | 0.221998847 | 0.716912415 | 1.1876737 | up | mRNA |
| NM_005449 | FAIM3 | 0.11528538 | 0.560638489 | 1.2378221 | up | mRNA |
| NM_005451 | PDLIM7 | 0.918157916 | 0.995349793 | 1.0137099 | up | mRNA |
| NM_005454 | CER1 | 0.184574834 | 0.668017166 | 1.6929416 | up | mRNA |
| NM_005455 | ZRANB2 | 0.3374601 | 0.827187565 | 1.0663024 | up | mRNA |
| NM_005456 | MAPK8IP1 | 0.244991484 | 0.743548975 | 1.1049594 | up | mRNA |
| NM_005458 | GABBR2 | 0.353575715 | 0.836405054 | 1.1901059 | up | mRNA |
| NM_005460 | SNCAIP | 0.226422569 | 0.721797379 | 1.1324674 | up | mRNA |
| NM_005468 | NAALADL1 | 0.550550948 | 0.925928432 | 1.0205855 | up | mRNA |
| NM_005470 | ABI1 | 0.042070999 | 0.379087707 | 1.0981306 | up | mRNA |
| NM_005472 | KCNE3 | 0.828638021 | 0.985493382 | 1.0970077 | up | mRNA |
| NM_005488 | TOM1 | 0.748376508 | 0.970725371 | 1.0439546 | up | mRNA |
| NM_005494 | DNAJB6 | 0.405967057 | 0.866541972 | 1.1763973 | up | mRNA |
| NM_005496 | SMC4 | 0.769223303 | 0.975270932 | 1.063199 | up | mRNA |
| NM_005502 | ABCA1 | 0.000212942 | 0.048871331 | 8.5710059 | up | mRNA |
| NM_005511 | MLANA | 0.169313262 | 0.645746997 | 1.1016028 | up | mRNA |
| NM_005514 | HLA-B | 0.488801026 | 0.904304137 | 1.1861625 | up | mRNA |
| NM_005516 | HLA-E | 0.324525036 | 0.816842093 | 1.1486673 | up | mRNA |
| NM_005520 | HNRNPH1 | 0.213484497 | 0.705954358 | 1.1019446 | up | mRNA |
| NM_005522 | HOXA1 | 0.439596449 | 0.883054028 | 1.0959332 | up | mRNA |
| NM_005523 | HOXA11 | 0.197837703 | 0.686504248 | 1.2178608 | up | mRNA |
| NM_005525 | HSD11B1 | 0.813337302 | 0.98120545 | 1.030167 | up | mRNA |
| NM_005526 | HSF1 | 0.308554585 | 0.803724978 | 1.1108828 | up | mRNA |
| NM_005528 | DNAJC4 | 0.890812024 | 0.9937521 | 1.0228701 | up | mRNA |
| NM_005529 | HSPG2 | 0.043699232 | 0.385351177 | 1.2925189 | up | mRNA |
| NM_005532 | IFI27 | 0.912255053 | 0.995342392 | 1.061707 | up | mRNA |
| NM_005533 | IFI35 | 0.019669562 | 0.282298294 | 1.129607 | up | mRNA |
| NM_005538 | INHBC | 0.135397763 | 0.593245913 | 1.1164212 | up | mRNA |
| NM_005541 | INPP5D | 0.081289777 | 0.48711575 | 1.1088678 | up | mRNA |
| NM_005551 | KLK2 | 0.649585125 | 0.95550188 | 1.0817373 | up | mRNA |
| NM_005554 | KRT6A | 0.479816512 | 0.901538317 | 1.1917099 | up | mRNA |
| NM_005561 | LAMP1 | 0.521358072 | 0.916644975 | 1.0450789 | up | mRNA |
| NM_005562 | LAMC2 | 0.00074926 | 0.080178677 | 2.415313 | up | mRNA |
| NM_005566 | LDHA | 0.447877806 | 0.887721928 | 1.1704895 | up | mRNA |
| NM_005568 | LHX1 | 0.856401322 | 0.988372845 | 1.0164344 | up | mRNA |
| NM_005577 | LPA | 0.780662781 | 0.976938981 | 1.0315924 | up | mRNA |
| NM_005590 | MRE11A | 0.962759024 | 0.99793927 | 1.0094068 | up | mRNA |
| NM_005591 | MRE11A | 0.974874705 | 0.998501532 | 1.0055945 | up | mRNA |
| NM_005595 | NFIA | 0.070276616 | 0.458686035 | 1.0689754 | up | mRNA |
| NM_005600 | NIT1 | 0.286788075 | 0.782942227 | 1.1168997 | up | mRNA |
| NM_005605 | PPP3CC | 0.097909804 | 0.520535504 | 1.4859298 | up | mRNA |
| NM_005619 | RTN2 | 0.473768254 | 0.899411578 | 1.0617722 | up | mRNA |
| NM_005623 | CCL8 | 0.000173495 | 0.044621794 | 4.1802736 | up | mRNA |
| NM_005631 | SMO | 0.402617845 | 0.864786348 | 1.1636614 | up | mRNA |
| NM_005633 | SOS1 | 0.606103079 | 0.94174007 | 1.2322778 | up | mRNA |
| NM_005635 | SSX1 | 0.291386969 | 0.787934547 | 1.0586172 | up | mRNA |
| NM_005636 | SSX4 | 0.485415612 | 0.903250293 | 1.0688038 | up | mRNA |
| NM_005640 | TAF4B | 0.005623735 | 0.170012215 | 1.9942547 | up | mRNA |
| NM_005645 | TAF13 | 0.794874882 | 0.979537918 | 1.0337315 | up | mRNA |
| NM_005647 | TBL1X | 0.676760524 | 0.959531882 | 1.0361906 | up | mRNA |
| NM_005669 | REEP5 | 0.128834081 | 0.582780013 | 1.077713 | up | mRNA |
| NM_005672 | PSCA | 0.051085556 | 0.408925797 | 1.1201512 | up | mRNA |
| NM_005676 | RBM10 | 0.48338372 | 0.902908388 | 1.2238539 | up | mRNA |
| NM_005677 | COLQ | 0.640574231 | 0.953894897 | 1.070953 | up | mRNA |
| NM_005679 | TAF1C | 0.719763974 | 0.966191059 | 1.0317308 | up | mRNA |
| NM_005686 | SOX13 | 0.571813725 | 0.933299883 | 1.053984 | up | mRNA |
| NM_005687 | FARSB | 0.610097193 | 0.943169861 | 1.0648617 | up | mRNA |
| NM_005690 | DNM1L | 0.486265714 | 0.903524001 | 1.0586776 | up | mRNA |
| NM_005694 | COX17 | 0.485651698 | 0.903250293 | 1.0554552 | up | mRNA |
| NM_005697 | SCAMP2 | 0.151177633 | 0.616500055 | 1.077151 | up | mRNA |
| NM_005711 | EDIL3 | 0.908946021 | 0.995333785 | 1.0125012 | up | mRNA |
| NM_005713 | COL4A3BP | 0.42881134 | 0.877386753 | 1.0864042 | up | mRNA |
| NM_005727 | TSPAN1 | 0.007368053 | 0.190938402 | 1.1331483 | up | mRNA |
| NM_005729 | PPIF | 0.075955269 | 0.473352674 | 1.1391919 | up | mRNA |
| NM_005730 | CTDSP2 | 0.53250699 | 0.920422692 | 1.0594255 | up | mRNA |
| NM_005736 | ACTR1A | 0.844553112 | 0.987338076 | 1.0150259 | up | mRNA |
| NM_005746 | NAMPT | 0.838074035 | 0.986897498 | 1.0210273 | up | mRNA |
| NM_005748 | YAF2 | 0.74461343 | 0.970330526 | 1.0132584 | up | mRNA |
| NM_005755 | EBI3 | 0.545211894 | 0.924012779 | 1.1142357 | up | mRNA |
| NM_005756 | GPR64 | 0.771030564 | 0.975347132 | 1.0347821 | up | mRNA |
| NM_005778 | RBM5 | 0.402791023 | 0.864786348 | 1.0708978 | up | mRNA |
| NM_005779 | LHFPL2 | 0.014309828 | 0.241161654 | 1.5348705 | up | mRNA |
| NM_005780 | LHFP | 0.45411106 | 0.890261204 | 1.1562218 | up | mRNA |
| NM_005793 | NME6 | 0.941027887 | 0.996710991 | 1.0218917 | up | mRNA |
| NM_005807 | PRG4 | 0.787175442 | 0.977479172 | 1.2855412 | up | mRNA |
| NM_005809 | PRDX2 | 0.360954421 | 0.841780454 | 1.04086 | up | mRNA |
| NM_005816 | CD96 | 0.154346801 | 0.621759137 | 1.1813443 | up | mRNA |
| NM_005819 | STX6 | 0.024812386 | 0.307613066 | 1.4863403 | up | mRNA |
| NM_005822 | RCAN2 | 0.294066572 | 0.791146775 | 1.1674885 | up | mRNA |
| NM_005824 | LRRC17 | 0.964773053 | 0.99793927 | 1.0211594 | up | mRNA |
| NM_005826 | HNRNPR | 0.28100698 | 0.779126326 | 1.0678971 | up | mRNA |
| NM_005827 | SLC35B1 | 0.83621702 | 0.986575022 | 1.0269894 | up | mRNA |
| NM_005829 | AP3S2 | 0.297997102 | 0.794295449 | 1.0805037 | up | mRNA |
| NM_005830 | MRPS31 | 0.296755013 | 0.793167205 | 1.0745038 | up | mRNA |
| NM_005838 | GLYAT | 0.048677177 | 0.400568526 | 1.24486 | up | mRNA |
| NM_005842 | SPRY2 | 0.237868125 | 0.735771951 | 1.6724346 | up | mRNA |
| NM_005852 | CHD3 | 0.905611904 | 0.99508468 | 1.0128485 | up | mRNA |
| NM_005855 | RAMP1 | 0.624318637 | 0.948341211 | 1.0377285 | up | mRNA |
| NM_005858 | AKAP8 | 0.87039482 | 0.989592579 | 1.0264126 | up | mRNA |
| NM_005866 | SIGMAR1 | 0.498215494 | 0.909116151 | 1.0662359 | up | mRNA |
| NM_005872 | BCAS2 | 0.536237063 | 0.922400335 | 1.1790293 | up | mRNA |
| NM_005875 | EIF1B | 0.237103244 | 0.735183924 | 1.1530121 | up | mRNA |
| NM_005877 | SF3A1 | 0.580604113 | 0.93393768 | 1.0344618 | up | mRNA |
| NM_005886 | KATNB1 | 0.352685099 | 0.835951672 | 1.0763375 | up | mRNA |
| NM_005891 | ACAT2 | 0.07184244 | 0.462266629 | 1.1243782 | up | mRNA |
| NM_005893 | CCIN | 0.36637872 | 0.845181673 | 1.1004406 | up | mRNA |
| NM_005900 | SMAD1 | 0.270502141 | 0.77028705 | 1.0392759 | up | mRNA |
| NM_005902 | SMAD3 | 0.503711688 | 0.910568383 | 1.0936479 | up | mRNA |
| NM_005907 | MAN1A1 | 0.512439618 | 0.91317044 | 1.1138563 | up | mRNA |
| NM_005917 | MDH1 | 0.547426123 | 0.925055514 | 1.0276068 | up | mRNA |
| NM_005920 | MEF2D | 0.936769634 | 0.996710991 | 1.0040734 | up | mRNA |
| NM_005921 | MAP3K1 | 0.435900293 | 0.881677506 | 1.1243096 | up | mRNA |
| NM_005923 | MAP3K5 | 0.017718279 | 0.267776588 | 1.6017014 | up | mRNA |
| NM_005928 | MFGE8 | 0.367006231 | 0.845181673 | 1.2654474 | up | mRNA |
| NM_005931 | MICB | 0.884869676 | 0.992981836 | 1.0258326 | up | mRNA |
| NM_005937 | MLLT6 | 0.299285356 | 0.79562655 | 1.0842083 | up | mRNA |
| NM_005940 | MMP11 | 0.947361614 | 0.996710991 | 1.0076789 | up | mRNA |
| NM_005941 | MMP16 | 0.4262298 | 0.875717896 | 1.0846121 | up | mRNA |
| NM_005943 | MOCS1 | 0.043152393 | 0.383508423 | 1.2406563 | up | mRNA |
| NM_005949 | MT1F | 0.045839548 | 0.391271907 | 1.210014 | up | mRNA |
| NM_005950 | MT1G | 0.00893892 | 0.202329498 | 2.1384379 | up | mRNA |
| NM_005952 | MT1X | 0.034596957 | 0.35197585 | 2.1346194 | up | mRNA |
| NM_005953 | MT2A | 0.129911342 | 0.583907255 | 1.8827699 | up | mRNA |
| NM_005954 | MT3 | 0.107979907 | 0.544140502 | 2.1459677 | up | mRNA |
| NM_005973 | PRCC | 0.804965668 | 0.98118927 | 1.0228141 | up | mRNA |
| NM_005978 | S100A2 | 0.423908406 | 0.874798629 | 1.0362922 | up | mRNA |
| NM_005981 | TSPAN31 | 0.780763488 | 0.976938981 | 1.0341379 | up | mRNA |
| NM_005985 | SNAI1 | 0.595519278 | 0.938746918 | 1.1046815 | up | mRNA |
| NM_005988 | SPRR2A | 0.282950577 | 0.780627498 | 1.7564086 | up | mRNA |
| NM_005994 | TBX2 | 0.920847299 | 0.995349793 | 1.0496309 | up | mRNA |
| NM_005998 | CCT3 | 0.904117971 | 0.99508468 | 1.0087137 | up | mRNA |
| NM_005999 | TSNAX | 0.919123852 | 0.995349793 | 1.0053833 | up | mRNA |
| NM_006006 | ZBTB16 | 0.836366692 | 0.986575022 | 1.0608819 | up | mRNA |
| NM_006009 | TUBA1A | 0.979416403 | 0.998971321 | 1.002836 | up | mRNA |
| NM_006014 | LAGE3 | 0.306625751 | 0.801653799 | 1.0850535 | up | mRNA |
| NM_006015 | ARID1A | 0.743675703 | 0.970330526 | 1.0440171 | up | mRNA |
| NM_006026 | H1FX | 0.885045072 | 0.992981836 | 1.0129579 | up | mRNA |
| NM_006028 | HTR3B | 0.210037097 | 0.700767028 | 1.539176 | up | mRNA |
| NM_006033 | LIPG | 0.068812341 | 0.455285558 | 1.6010529 | up | mRNA |
| NM_006039 | MRC2 | 0.194011502 | 0.681590151 | 1.1962049 | up | mRNA |
| NM_006041 | HS3ST3B1 | 0.060066984 | 0.435030895 | 1.2467674 | up | mRNA |
| NM_006044 | HDAC6 | 0.001877182 | 0.105670067 | 1.1256692 | up | mRNA |
| NM_006058 | TNIP1 | 0.507774139 | 0.911674633 | 1.1174553 | up | mRNA |
| NM_006065 | SIRPB1 | 0.366565855 | 0.845181673 | 1.1208863 | up | mRNA |
| NM_006066 | AKR1A1 | 0.504412265 | 0.910568383 | 1.0273108 | up | mRNA |
| NM_006068 | TLR6 | 0.101039465 | 0.528378192 | 1.3537261 | up | mRNA |
| NM_006072 | CCL26 | 0.152760433 | 0.618463784 | 1.2195182 | up | mRNA |
| NM_006074 | TRIM22 | 0.97214071 | 0.998501532 | 1.0313529 | up | mRNA |
| NM_006077 | MICU1 | 0.142981751 | 0.603147721 | 1.0674837 | up | mRNA |
| NM_006087 | TUBB4A | 0.084143248 | 0.493737521 | 1.2928181 | up | mRNA |
| NM_006098 | GNB2L1 | 0.80861862 | 0.98118927 | 1.0198329 | up | mRNA |
| NM_006116 | TAB1 | 0.635120034 | 0.95145488 | 1.0830267 | up | mRNA |
| NM_006120 | HLA-DMA | 0.733402569 | 0.967777568 | 1.1172229 | up | mRNA |
| NM_006122 | MAN2A2 | 0.437429412 | 0.882322896 | 1.0553696 | up | mRNA |
| NM_006129 | BMP1 | 0.69476868 | 0.962202557 | 1.0320342 | up | mRNA |
| NM_006133 | DAGLA | 0.749352945 | 0.970725371 | 1.0272688 | up | mRNA |
| NM_006149 | LGALS4 | 0.798892743 | 0.980813744 | 1.026589 | up | mRNA |
| NM_006150 | PRICKLE3 | 0.602430643 | 0.941406011 | 1.1390628 | up | mRNA |
| NM_006155 | SEPT2 | 0.146341011 | 0.608122555 | 1.0911376 | up | mRNA |
| NM_006160 | NEUROD2 | 0.125549006 | 0.578441772 | 1.1091959 | up | mRNA |
| NM_006162 | NFATC1 | 0.00021571 | 0.048871331 | 2.0663701 | up | mRNA |
| NM_006164 | NFE2L2 | 0.17722154 | 0.657170957 | 1.1181104 | up | mRNA |
| NM_006169 | NNMT | 0.190192606 | 0.676255734 | 1.3007291 | up | mRNA |
| NM_006177 | NRL | 0.121601033 | 0.573155624 | 1.1912473 | up | mRNA |
| NM_006183 | NTS | 0.579386804 | 0.933821303 | 1.0491598 | up | mRNA |
| NM_006187 | OAS3 | 0.345409934 | 0.832533915 | 1.5692997 | up | mRNA |
| NM_006202 | PDE4A | 0.40761883 | 0.867591329 | 1.3060493 | up | mRNA |
| NM_006211 | PENK | 0.964310108 | 0.99793927 | 1.0477188 | up | mRNA |
| NM_006216 | SERPINE2 | 0.463839001 | 0.894989844 | 1.1728591 | up | mRNA |
| NM_006219 | PIK3CB | 0.659841785 | 0.958354006 | 1.0491102 | up | mRNA |
| NM_006224 | PITPNA | 0.087303289 | 0.500533133 | 1.0671268 | up | mRNA |
| NM_006225 | PLCD1 | 0.858313098 | 0.988512246 | 1.0295642 | up | mRNA |
| NM_006228 | PNOC | 0.644361 | 0.954657712 | 1.0287264 | up | mRNA |
| NM_006229 | PNLIPRP1 | 0.575399974 | 0.933656777 | 1.0684689 | up | mRNA |
| NM_006235 | POU2AF1 | 0.965593139 | 0.99793927 | 1.0050792 | up | mRNA |
| NM_006249 | PRB3 | 0.519150118 | 0.916145597 | 1.0721102 | up | mRNA |
| NM_006253 | PRKAB1 | 0.893981467 | 0.993978977 | 1.0163992 | up | mRNA |
| NM_006255 | PRKCH | 0.637861088 | 0.952852353 | 1.1195024 | up | mRNA |
| NM_006257 | PRKCQ | 0.125151009 | 0.578441772 | 1.2241292 | up | mRNA |
| NM_006263 | PSME1 | 0.281834713 | 0.779398875 | 1.1336005 | up | mRNA |
| NM_006273 | CCL7 | 0.000454513 | 0.066148041 | 12.3195865 | up | mRNA |
| NM_006274 | CCL19 | 0.938766366 | 0.996710991 | 1.0128965 | up | mRNA |
| NM_006277 | ITSN2 | 0.792090975 | 0.978996327 | 1.0173238 | up | mRNA |
| NM_006278 | ST3GAL4 | 0.62814903 | 0.949690755 | 1.0447428 | up | mRNA |
| NM_006280 | SSR4 | 0.851939227 | 0.987678166 | 1.0166268 | up | mRNA |
| NM_006281 | STK3 | 0.802253439 | 0.98118927 | 1.0360764 | up | mRNA |
| NM_006282 | STK4 | 0.026510197 | 0.318338323 | 1.4552822 | up | mRNA |
| NM_006284 | TAF10 | 0.890410207 | 0.9937521 | 1.0242505 | up | mRNA |
| NM_006287 | TFPI | 0.868376791 | 0.989481868 | 1.0400083 | up | mRNA |
| NM_006288 | THY1 | 0.864591069 | 0.989401142 | 1.0337376 | up | mRNA |
| NM_006289 | TLN1 | 0.741384106 | 0.970240145 | 1.0241686 | up | mRNA |
| NM_006290 | TNFAIP3 | 1.60499E-05 | 0.018129212 | 7.9496672 | up | mRNA |
| NM_006295 | VARS | 0.274620727 | 0.774745196 | 1.0572325 | up | mRNA |
| NM_006301 | MAP3K12 | 0.039419173 | 0.370286706 | 1.3326355 | up | mRNA |
| NM_006305 | ANP32A | 0.817727215 | 0.981727075 | 1.0589143 | up | mRNA |
| NM_006313 | USP15 | 0.002490934 | 0.118591515 | 1.1951344 | up | mRNA |
| NM_006317 | BASP1 | 0.017736187 | 0.267776588 | 1.251642 | up | mRNA |
| NM_006328 | RBM14 | 0.690229042 | 0.961105466 | 1.0533902 | up | mRNA |
| NM_006334 | OLFM1 | 0.520450937 | 0.916605148 | 1.266985 | up | mRNA |
| NM_006337 | MCRS1 | 0.552928177 | 0.926337111 | 1.0287701 | up | mRNA |
| NM_006344 | CLEC10A | 0.068915573 | 0.455528425 | 1.1568423 | up | mRNA |
| NM_006348 | COG5 | 0.36881363 | 0.845559574 | 1.0824042 | up | mRNA |
| NM_006350 | FST | 0.496008215 | 0.908134987 | 1.1319724 | up | mRNA |
| NM_006356 | ATP5H | 0.410703492 | 0.868341245 | 1.0356353 | up | mRNA |
| NM_006375 | ENOX2 | 0.893090675 | 0.993978977 | 1.016475 | up | mRNA |
| NM_006386 | DDX17 | 0.741003188 | 0.970189763 | 1.0194856 | up | mRNA |
| NM_006396 | SSSCA1 | 0.965314947 | 0.99793927 | 1.0056404 | up | mRNA |
| NM_006398 | UBD | 0.056679609 | 0.424304055 | 1.2259512 | up | mRNA |
| NM_006404 | PROCR | 0.653239661 | 0.956860606 | 1.1580085 | up | mRNA |
| NM_006407 | ARL6IP5 | 0.905187866 | 0.99508468 | 1.0109764 | up | mRNA |
| NM_006417 | IFI44 | 0.716225097 | 0.966191059 | 1.0453172 | up | mRNA |
| NM_006420 | ARFGEF2 | 0.782186008 | 0.977014981 | 1.0247099 | up | mRNA |
| NM_006422 | AKAP3 | 0.677290262 | 0.959531882 | 1.0951455 | up | mRNA |
| NM_006424 | SLC34A2 | 0.865761782 | 0.989426402 | 1.0201184 | up | mRNA |
| NM_006432 | NPC2 | 0.774975796 | 0.97589756 | 1.033571 | up | mRNA |
| NM_006433 | GNLY | 0.095292899 | 0.516597924 | 1.2763088 | up | mRNA |
| NM_006435 | IFITM2 | 0.465316531 | 0.895338213 | 1.169715 | up | mRNA |
| NM_006440 | TXNRD2 | 0.667791018 | 0.958354006 | 1.0723626 | up | mRNA |
| NM_006441 | MTHFS | 0.256964733 | 0.75762862 | 1.1416564 | up | mRNA |
| NM_006442 | DRAP1 | 0.804635795 | 0.98118927 | 1.0457657 | up | mRNA |
| NM_006449 | CDC42EP3 | 0.35974261 | 0.841302824 | 1.263901 | up | mRNA |
| NM_006451 | PAIP1 | 0.575512287 | 0.933656777 | 1.07134 | up | mRNA |
| NM_006457 | PDLIM5 | 0.911838176 | 0.995342392 | 1.0141621 | up | mRNA |
| NM_006464 | TGOLN2 | 0.706261818 | 0.965294118 | 1.027312 | up | mRNA |
| NM_006465 | ARID3B | 0.494342492 | 0.906930718 | 1.1244433 | up | mRNA |
| NM_006470 | TRIM16 | 0.009850699 | 0.206905383 | 1.4083703 | up | mRNA |
| NM_006476 | ATP5L | 0.477284674 | 0.900450218 | 1.027101 | up | mRNA |
| NM_006483 | DYRK1B | 0.747227563 | 0.970527074 | 1.0460932 | up | mRNA |
| NM_006486 | FBLN1 | 0.451756883 | 0.888854908 | 1.2318213 | up | mRNA |
| NM_006487 | FBLN1 | 0.981682135 | 0.998971807 | 1.0089598 | up | mRNA |
| NM_006491 | NOVA1 | 0.247105107 | 0.745152522 | 1.3044143 | up | mRNA |
| NM_006497 | HIC1 | 0.674001775 | 0.959531882 | 1.0502528 | up | mRNA |
| NM_006499 | LGALS8 | 0.637300492 | 0.952624144 | 1.0597832 | up | mRNA |
| NM_006506 | RASA2 | 0.015999663 | 0.254584533 | 2.3080151 | up | mRNA |
| NM_006509 | RELB | 0.015778514 | 0.252653581 | 3.1993548 | up | mRNA |
| NM_006515 | SETMAR | 0.394015919 | 0.861008404 | 1.0931219 | up | mRNA |
| NM_006516 | SLC2A1 | 0.020627294 | 0.286034385 | 3.1671558 | up | mRNA |
| NM_006517 | SLC16A2 | 0.805101407 | 0.98118927 | 1.0482039 | up | mRNA |
| NM_006519 | DYNLT1 | 0.497185344 | 0.908789022 | 1.0648469 | up | mRNA |
| NM_006520 | DYNLT3 | 0.877951208 | 0.99136421 | 1.0107516 | up | mRNA |
| NM_006521 | TFE3 | 0.900620087 | 0.994789506 | 1.0067704 | up | mRNA |
| NM_006526 | ZNF217 | 0.08783866 | 0.501417299 | 1.4238115 | up | mRNA |
| NM_006528 | TFPI2 | 0.002254348 | 0.112066979 | 7.0195798 | up | mRNA |
| NM_006531 | IFT88 | 0.561630661 | 0.928606538 | 1.0741849 | up | mRNA |
| NM_006533 | MIA | 0.536388053 | 0.922509728 | 1.0297699 | up | mRNA |
| NM_006538 | BCL2L11 | 0.020554796 | 0.286034385 | 1.8882189 | up | mRNA |
| NM_006542 | SPHAR | 0.171278026 | 0.648737859 | 1.0856099 | up | mRNA |
| NM_006549 | CAMKK2 | 0.711951657 | 0.96592341 | 1.0621481 | up | mRNA |
| NM_006551 | SCGB1D2 | 0.221209592 | 0.716640124 | 1.1076809 | up | mRNA |
| NM_006553 | SLMO1 | 0.626896392 | 0.949203235 | 1.1019553 | up | mRNA |
| NM_006555 | YKT6 | 0.797835187 | 0.980749101 | 1.0372157 | up | mRNA |
| NM_006558 | KHDRBS3 | 0.381212866 | 0.853514109 | 1.057866 | up | mRNA |
| NM_006560 | CELF1 | 0.071915394 | 0.462423718 | 1.1034212 | up | mRNA |
| NM_006561 | CELF2 | 0.039536011 | 0.370322288 | 1.118572 | up | mRNA |
| NM_006571 | DCTN6 | 0.672057521 | 0.959531882 | 1.050556 | up | mRNA |
| NM_006576 | AVIL | 0.967125393 | 0.99793927 | 1.0112323 | up | mRNA |
| NM_006577 | B3GNT2 | 0.03755437 | 0.362147666 | 1.2782461 | up | mRNA |
| NM_006578 | GNB5 | 0.844298701 | 0.987338076 | 1.0345142 | up | mRNA |
| NM_006581 | FUT9 | 0.53421875 | 0.920652989 | 1.1240602 | up | mRNA |
| NM_006585 | CCT8 | 0.248350044 | 0.746629174 | 1.1355846 | up | mRNA |
| NM_006588 | SULT1C4 | 0.931082248 | 0.996710991 | 1.0084121 | up | mRNA |
| NM_006589 | FAM189B | 0.556494682 | 0.926833616 | 1.0589714 | up | mRNA |
| NM_006598 | SLC12A7 | 0.902217404 | 0.995003485 | 1.0263979 | up | mRNA |
| NM_006606 | RBBP9 | 0.403046481 | 0.864786348 | 1.0551464 | up | mRNA |
| NM_006607 | PTTG2 | 0.937708946 | 0.996710991 | 1.0326273 | up | mRNA |
| NM_006612 | KIF1C | 0.945783621 | 0.996710991 | 1.0089368 | up | mRNA |
| NM_006614 | CHL1 | 0.265420236 | 0.766537754 | 1.0911949 | up | mRNA |
| NM_006618 | KDM5B | 0.289313933 | 0.785298708 | 1.0893722 | up | mRNA |
| NM_006622 | PLK2 | 0.021000007 | 0.287801352 | 2.385228 | up | mRNA |
| NM_006632 | SLC17A3 | 0.577779107 | 0.933815962 | 1.0923361 | up | mRNA |
| NM_006634 | VAMP5 | 0.041976403 | 0.378937196 | 1.10028 | up | mRNA |
| NM_006636 | MTHFD2 | 0.810162984 | 0.98118927 | 1.0862784 | up | mRNA |
| NM_006640 | SEPT9 | 0.027284859 | 0.3212251 | 1.2479923 | up | mRNA |
| NM_006641 | CCR9 | 0.097398617 | 0.520175647 | 1.2634949 | up | mRNA |
| NM_006644 | HSPH1 | 0.748085341 | 0.970725371 | 1.0508394 | up | mRNA |
| NM_006645 | STARD10 | 0.194680663 | 0.682390954 | 1.3721072 | up | mRNA |
| NM_006646 | WASF3 | 0.096819153 | 0.518991327 | 1.0639116 | up | mRNA |
| NM_006647 | NOXA1 | 0.459345003 | 0.892904132 | 1.0664904 | up | mRNA |
| NM_006650 | CPLX2 | 0.672042152 | 0.959531882 | 1.0393287 | up | mRNA |
| NM_006659 | TUBGCP2 | 0.858251086 | 0.988512246 | 1.0151586 | up | mRNA |
| NM_006671 | SLC1A7 | 0.147285356 | 0.609651031 | 1.2751897 | up | mRNA |
| NM_006677 | USP19 | 0.462869657 | 0.894989844 | 1.0873949 | up | mRNA |
| NM_006684 | CFHR4 | 0.126804017 | 0.580406758 | 1.3564596 | up | mRNA |
| NM_006700 | TRAFD1 | 0.720391571 | 0.966191059 | 1.0373275 | up | mRNA |
| NM_006706 | TCERG1 | 0.338308699 | 0.827403747 | 1.2258103 | up | mRNA |
| NM_006708 | GLO1 | 0.681666829 | 0.960111041 | 1.0224788 | up | mRNA |
| NM_006710 | COPS8 | 0.789697381 | 0.978197097 | 1.0158385 | up | mRNA |
| NM_006714 | SMPDL3A | 0.09221334 | 0.511207518 | 1.1216931 | up | mRNA |
| NM_006720 | ABLIM1 | 0.903930212 | 0.99508468 | 1.014595 | up | mRNA |
| NM_006721 | ADK | 0.580046412 | 0.933893479 | 1.0886553 | up | mRNA |
| NM_006725 | CD6 | 0.810183213 | 0.98118927 | 1.0221135 | up | mRNA |
| NM_006732 | FOSB | 0.975626411 | 0.998501532 | 1.0053725 | up | mRNA |
| NM_006734 | HIVEP2 | 0.022550948 | 0.295888941 | 2.5413872 | up | mRNA |
| NM_006738 | AKAP13 | 0.30953244 | 0.803961878 | 1.0536244 | up | mRNA |
| NM_006742 | PSKH1 | 0.519782823 | 0.916187634 | 1.1585828 | up | mRNA |
| NM_006744 | RBP4 | 0.973560104 | 0.998501532 | 1.0099371 | up | mRNA |
| NM_006745 | MSMO1 | 0.508134713 | 0.911674633 | 1.0911501 | up | mRNA |
| NM_006746 | SCML1 | 0.179502934 | 0.661888586 | 1.1203468 | up | mRNA |
| NM_006757 | TNNT3 | 0.553697272 | 0.926337111 | 1.0419774 | up | mRNA |
| NM_006759 | UGP2 | 0.099726427 | 0.524750962 | 1.2583873 | up | mRNA |
| NM_006760 | UPK2 | 0.409359789 | 0.86762256 | 1.1886421 | up | mRNA |
| NM_006763 | BTG2 | 0.000890947 | 0.083478018 | 7.6838119 | up | mRNA |
| NM_006765 | TUSC3 | 0.984282195 | 0.998971807 | 1.0052191 | up | mRNA |
| NM_006769 | LMO4 | 0.912073382 | 0.995342392 | 1.01739 | up | mRNA |
| NM_006778 | TRIM10 | 0.333382669 | 0.823381801 | 1.3669065 | up | mRNA |
| NM_006786 | UTS2 | 0.461210807 | 0.89460367 | 1.0571702 | up | mRNA |
| NM_006789 | APOBEC2 | 0.150024985 | 0.614362133 | 1.4752919 | up | mRNA |
| NM_006793 | PRDX3 | 0.525387821 | 0.917686109 | 1.0305251 | up | mRNA |
| NM_006795 | EHD1 | 0.997633362 | 0.999889342 | 1.0004697 | up | mRNA |
| NM_006806 | BTG3 | 0.001712417 | 0.105422342 | 1.5856139 | up | mRNA |
| NM_006808 | SEC61B | 0.810641239 | 0.98118927 | 1.0151216 | up | mRNA |
| NM_006809 | TOMM34 | 0.343829549 | 0.831629639 | 1.0691458 | up | mRNA |
| NM_006813 | PNRC1 | 0.967412917 | 0.99793927 | 1.0038586 | up | mRNA |
| NM_006816 | LMAN2 | 0.033572204 | 0.347905227 | 1.4609669 | up | mRNA |
| NM_006821 | ACOT2 | 0.650355804 | 0.955894361 | 1.0420894 | up | mRNA |
| NM_006829 | C10orf116 | 0.887237174 | 0.993189793 | 1.0148603 | up | mRNA |
| NM_006834 | RAB32 | 0.766047681 | 0.974734429 | 1.0656628 | up | mRNA |
| NM_006857 | SNRNP27 | 0.962143241 | 0.99793927 | 1.0068786 | up | mRNA |
| NM_006865 | LILRA3 | 0.271726686 | 0.771737648 | 1.2508147 | up | mRNA |
| NM_006866 | LILRA2 | 0.79264862 | 0.979003983 | 1.0116695 | up | mRNA |
| NM_006867 | RBPMS | 0.801608924 | 0.98118927 | 1.0638309 | up | mRNA |
| NM_006869 | ADAP1 | 0.880201705 | 0.992197209 | 1.0304349 | up | mRNA |
| NM_006870 | DSTN | 0.530714517 | 0.919278204 | 1.0701643 | up | mRNA |
| NM_006884 | SHOX2 | 0.28135881 | 0.779126326 | 1.4099571 | up | mRNA |
| NM_006886 | ATP5E | 0.036884118 | 0.359628653 | 1.0719408 | up | mRNA |
| NM_006888 | CALM1 | 0.402983175 | 0.864786348 | 1.086973 | up | mRNA |
| NM_006895 | HNMT | 0.942301832 | 0.996710991 | 1.0217402 | up | mRNA |
| NM_006896 | HOXA7 | 0.506195524 | 0.911278334 | 1.0840288 | up | mRNA |
| NM_006902 | PRRX1 | 0.022234628 | 0.293936579 | 1.2279604 | up | mRNA |
| NM_006903 | PPA2 | 0.834780709 | 0.986575022 | 1.0163103 | up | mRNA |
| NM_006908 | RAC1 | 0.910486182 | 0.995342392 | 1.0104993 | up | mRNA |
| NM_006909 | RASGRF2 | 0.55334787 | 0.926337111 | 1.0772351 | up | mRNA |
| NM_006913 | RNF5 | 0.819588072 | 0.982331503 | 1.0822641 | up | mRNA |
| NM_006922 | SCN3A | 0.085345168 | 0.497345359 | 1.0867225 | up | mRNA |
| NM_006923 | SDF2 | 0.604411108 | 0.94174007 | 1.0569051 | up | mRNA |
| NM_006925 | SRSF5 | 0.348137581 | 0.834138149 | 1.2363838 | up | mRNA |
| NM_006928 | PMEL | 0.19373884 | 0.681151321 | 1.3767321 | up | mRNA |
| NM_006929 | SKIV2L | 0.463046035 | 0.894989844 | 1.0583373 | up | mRNA |
| NM_006930 | SKP1 | 0.474292375 | 0.899418411 | 1.1317247 | up | mRNA |
| NM_006931 | SLC2A3 | 0.095459516 | 0.516597924 | 1.1589306 | up | mRNA |
| NM_006933 | SLC5A3 | 0.261178521 | 0.761986179 | 1.2280418 | up | mRNA |
| NM_006942 | SOX15 | 0.157921938 | 0.62798139 | 1.1760992 | up | mRNA |
| NM_006944 | SPP2 | 0.477233799 | 0.900450218 | 1.1735281 | up | mRNA |
| NM_006952 | UPK1B | 0.693827577 | 0.961812388 | 1.2478227 | up | mRNA |
| NM_006954 | ZNF33A | 0.166645518 | 0.641468509 | 1.1028828 | up | mRNA |
| NM_006955 | ZNF33B | 0.589878491 | 0.936114189 | 1.1024124 | up | mRNA |
| NM_006977 | ZBTB25 | 0.489242237 | 0.904620847 | 1.0335438 | up | mRNA |
| NM_006981 | NR4A3 | 0.540306668 | 0.923577434 | 1.0635534 | up | mRNA |
| NM_006983 | MMP23B | 0.346305005 | 0.833163669 | 1.0925616 | up | mRNA |
| NM_006995 | BTN2A2 | 0.024302308 | 0.304521009 | 1.1694896 | up | mRNA |
| NM_006997 | TACC2 | 0.568038994 | 0.931705674 | 1.0489693 | up | mRNA |
| NM_007005 | TLE4 | 0.71849887 | 0.966191059 | 1.0652257 | up | mRNA |
| NM_007006 | NUDT21 | 0.966382986 | 0.99793927 | 1.0054314 | up | mRNA |
| NM_007008 | RTN4 | 0.237477016 | 0.73558542 | 1.2541609 | up | mRNA |
| NM_007009 | ZPBP | 0.482703325 | 0.902654749 | 1.1226606 | up | mRNA |
| NM_007013 | WWP1 | 0.571900518 | 0.933338778 | 1.0282872 | up | mRNA |
| NM_007017 | SOX30 | 0.622268174 | 0.948166379 | 1.0446291 | up | mRNA |
| NM_007021 | C10orf10 | 0.846849676 | 0.987678166 | 1.0169058 | up | mRNA |
| NM_007023 | RAPGEF4 | 0.756837213 | 0.971833663 | 1.0613845 | up | mRNA |
| NM_007026 | DUSP14 | 0.780543242 | 0.976938981 | 1.0472918 | up | mRNA |
| NM_007027 | TOPBP1 | 0.890497335 | 0.9937521 | 1.0302929 | up | mRNA |
| NM_007036 | ESM1 | 0.051073603 | 0.408925797 | 2.1242273 | up | mRNA |
| NM_007044 | KATNA1 | 0.314031959 | 0.809136712 | 1.0596124 | up | mRNA |
| NM_007045 | FGFR1OP | 0.640737171 | 0.953894897 | 1.1037511 | up | mRNA |
| NM_007047 | BTN3A2 | 0.913914379 | 0.995342392 | 1.0090387 | up | mRNA |
| NM_007050 | PTPRT | 0.560529608 | 0.92782421 | 1.0943716 | up | mRNA |
| NM_007051 | FAF1 | 0.324926026 | 0.817191271 | 1.126781 | up | mRNA |
| NM_007054 | KIF3A | 0.83526102 | 0.986575022 | 1.0344819 | up | mRNA |
| NM_007067 | KAT7 | 0.979167086 | 0.998867352 | 1.0044148 | up | mRNA |
| NM_007072 | HHLA2 | 0.002791056 | 0.124912935 | 1.452491 | up | mRNA |
| NM_007084 | SOX21 | 0.656578442 | 0.957445617 | 1.113869 | up | mRNA |
| NM_007085 | FSTL1 | 0.771725491 | 0.975347132 | 1.0208108 | up | mRNA |
| NM_007096 | CLTA | 0.718043566 | 0.966191059 | 1.0793416 | up | mRNA |
| NM_007097 | CLTB | 0.950126183 | 0.996710991 | 1.011518 | up | mRNA |
| NM_007098 | CLTCL1 | 0.52214657 | 0.916999248 | 1.2107257 | up | mRNA |
| NM_007104 | RPL10A | 0.78931035 | 0.977896413 | 1.0343346 | up | mRNA |
| NM_007110 | TEP1 | 0.058604218 | 0.430469997 | 1.2068238 | up | mRNA |
| NM_007115 | TNFAIP6 | 4.92756E-07 | 0.008727906 | 31.5628739 | up | mRNA |
| NM_007117 | TRH | 0.235977161 | 0.734183264 | 1.1491154 | up | mRNA |
| NM_007118 | TRIO | 0.465701434 | 0.895570502 | 1.0879217 | up | mRNA |
| NM_007128 | VPREB1 | 0.91869324 | 0.995349793 | 1.0115457 | up | mRNA |
| NM_007131 | ZNF75D | 0.782450281 | 0.977106756 | 1.0889424 | up | mRNA |
| NM_007135 | ZNF79 | 0.688157422 | 0.96055024 | 1.0684665 | up | mRNA |
| NM_007155 | ZP3 | 0.444263772 | 0.885088246 | 1.1374012 | up | mRNA |
| NM_007157 | ZXDB | 0.027696265 | 0.32381669 | 1.217816 | up | mRNA |
| NM_007174 | CIT | 0.797147284 | 0.980667273 | 1.0413915 | up | mRNA |
| NM_007175 | ERLIN2 | 0.818905753 | 0.982103868 | 1.0248621 | up | mRNA |
| NM_007182 | RASSF1 | 0.380438243 | 0.853407568 | 1.1116128 | up | mRNA |
| NM_007184 | NISCH | 0.198321148 | 0.686799061 | 1.1248105 | up | mRNA |
| NM_007185 | CELF3 | 0.94336599 | 0.996710991 | 1.0062478 | up | mRNA |
| NM_007187 | WBP4 | 0.073003879 | 0.464657997 | 1.1240781 | up | mRNA |
| NM_007199 | IRAK3 | 0.066209241 | 0.449693785 | 1.7768691 | up | mRNA |
| NM_007203 | PALM2-AKAP2 | 0.001091723 | 0.086778252 | 3.0146491 | up | mRNA |
| NM_007207 | DUSP10 | 0.993202258 | 0.999872237 | 1.0008859 | up | mRNA |
| NM_007209 | RPL35 | 0.752047214 | 0.970725371 | 1.0177172 | up | mRNA |
| NM_007213 | PRAF2 | 0.560751568 | 0.927965324 | 1.0579375 | up | mRNA |
| NM_007232 | HRH3 | 0.54139982 | 0.923577434 | 1.079583 | up | mRNA |
| NM_007238 | PXMP4 | 0.095795435 | 0.517208636 | 1.0990449 | up | mRNA |
| NM_007255 | B4GALT7 | 0.8461217 | 0.987338076 | 1.0334614 | up | mRNA |
| NM_007261 | CD300A | 0.375486071 | 0.849862269 | 1.1197577 | up | mRNA |
| NM_007262 | PARK7 | 0.583867075 | 0.934397621 | 1.0250742 | up | mRNA |
| NM_007264 | GPR182 | 0.689839893 | 0.961105466 | 1.0527884 | up | mRNA |
| NM_007266 | GPN1 | 0.34078035 | 0.828854153 | 1.1347776 | up | mRNA |
| NM_007268 | VSIG4 | 0.962274486 | 0.99793927 | 1.0134665 | up | mRNA |
| NM_007270 | FKBP9 | 0.702368827 | 0.963378163 | 1.0630665 | up | mRNA |
| NM_007272 | CTRC | 0.821293528 | 0.982756843 | 1.0426764 | up | mRNA |
| NM_007274 | ACOT7 | 0.755939422 | 0.971522741 | 1.0579104 | up | mRNA |
| NM_007279 | U2AF2 | 0.70902206 | 0.965462762 | 1.0565266 | up | mRNA |
| NM_007281 | SCRG1 | 0.776176022 | 0.976080759 | 1.1375584 | up | mRNA |
| NM_007283 | MGLL | 0.252454922 | 0.752621656 | 1.7293398 | up | mRNA |
| NM_007286 | SYNPO | 0.985422567 | 0.998971807 | 1.0036285 | up | mRNA |
| NM_007292 | ACOX1 | 0.002001362 | 0.107649983 | 1.5496601 | up | mRNA |
| NM_007293 | C4A | 0.016421132 | 0.255922225 | 1.6732211 | up | mRNA |
| NM_007300 | BRCA1 | 0.096397727 | 0.518096374 | 1.3007246 | up | mRNA |
| NM_007309 | DIAPH2 | 0.405502399 | 0.866165103 | 1.0227035 | up | mRNA |
| NM_007313 | ABL1 | 0.281562083 | 0.779126326 | 1.179994 | up | mRNA |
| NM_007318 | PSEN1 | 0.009010181 | 0.202329498 | 1.4807123 | up | mRNA |
| NM_007327 | GRIN1 | 0.436364214 | 0.881836517 | 1.0879219 | up | mRNA |
| NM_007346 | OGFR | 0.02730248 | 0.321246536 | 1.2882538 | up | mRNA |
| NM_007350 | PHLDA1 | 0.748687104 | 0.970725371 | 1.0705246 | up | mRNA |
| NM_007352 | CELA3B | 0.122387357 | 0.57455499 | 1.1270783 | up | mRNA |
| NM_007353 | GNA12 | 0.828407924 | 0.985442892 | 1.0293608 | up | mRNA |
| NM_007355 | HSP90AB1 | 0.755812177 | 0.971522741 | 1.0199273 | up | mRNA |
| NM_007361 | NID2 | 0.724951424 | 0.966191059 | 1.0518081 | up | mRNA |
| NM_007370 | RFC5 | 0.540973445 | 0.923577434 | 1.0824656 | up | mRNA |
| NM_007374 | SIX6 | 0.185858193 | 0.669321303 | 1.1969553 | up | mRNA |
| NM_009590 | AOC2 | 0.069754354 | 0.457793905 | 1.2898305 | up | mRNA |
| NM_012062 | DNM1L | 0.003996458 | 0.145099977 | 1.3955655 | up | mRNA |
| NM_012063 | DNM1L | 0.360162075 | 0.841759421 | 1.0494126 | up | mRNA |
| NM_012068 | ATF5 | 0.055012605 | 0.419520587 | 1.1603813 | up | mRNA |
| NM_012071 | COMMD3 | 0.952895193 | 0.996710991 | 1.0048501 | up | mRNA |
| NM_012072 | CD93 | 0.988729326 | 0.999140676 | 1.0020718 | up | mRNA |
| NM_012088 | PGLS | 0.93766939 | 0.996710991 | 1.0183701 | up | mRNA |
| NM_012098 | ANGPTL2 | 0.490997809 | 0.90553335 | 1.157883 | up | mRNA |
| NM_012100 | DNPEP | 0.968930163 | 0.998196842 | 1.0066552 | up | mRNA |
| NM_012114 | CASP14 | 0.145871883 | 0.607759658 | 1.1554119 | up | mRNA |
| NM_012116 | CBLC | 0.437052123 | 0.882217212 | 1.0465796 | up | mRNA |
| NM_012118 | CCRN4L | 0.000652132 | 0.076919551 | 3.9803381 | up | mRNA |
| NM_012121 | CDC42EP4 | 0.290084872 | 0.786468159 | 1.0816808 | up | mRNA |
| NM_012124 | CHORDC1 | 0.621324819 | 0.947979605 | 1.2274637 | up | mRNA |
| NM_012125 | CHRM5 | 0.194351746 | 0.682224934 | 1.2357811 | up | mRNA |
| NM_012147 | DUX2 | 0.90196482 | 0.994906068 | 1.0224592 | up | mRNA |
| NM_012151 | F8A1 | 0.376028745 | 0.849862269 | 1.1012969 | up | mRNA |
| NM_012154 | EIF2C2 | 0.041026264 | 0.375308608 | 1.1666795 | up | mRNA |
| NM_012168 | FBXO2 | 0.998048666 | 0.999889342 | 1.0007925 | up | mRNA |
| NM_012184 | FOXD4L1 | 0.157002516 | 0.625751326 | 1.6636577 | up | mRNA |
| NM_012188 | FOXI1 | 0.303163195 | 0.798533134 | 1.1601725 | up | mRNA |
| NM_012191 | NAT6 | 0.585088316 | 0.934708084 | 1.0689147 | up | mRNA |
| NM_012193 | FZD4 | 0.723691802 | 0.966191059 | 1.0420993 | up | mRNA |
| NM_012198 | GCA | 0.949853136 | 0.996710991 | 1.0227195 | up | mRNA |
| NM_012199 | EIF2C1 | 0.453153156 | 0.889563297 | 1.1223083 | up | mRNA |
| NM_012212 | PTGR1 | 0.964266873 | 0.99793927 | 1.0015807 | up | mRNA |
| NM_012215 | MGEA5 | 0.677010585 | 0.959531882 | 1.0257006 | up | mRNA |
| NM_012217 | TPSD1 | 0.211252436 | 0.702600386 | 1.1316933 | up | mRNA |
| NM_012224 | NEK1 | 0.758386711 | 0.972072638 | 1.0854168 | up | mRNA |
| NM_012230 | POMZP3 | 0.182516595 | 0.66575662 | 1.1257793 | up | mRNA |
| NM_012231 | PRDM2 | 0.252425073 | 0.752621656 | 1.1101411 | up | mRNA |
| NM_012232 | PTRF | 0.728134676 | 0.966191059 | 1.014704 | up | mRNA |
| NM_012234 | RYBP | 0.82416075 | 0.983568283 | 1.0510805 | up | mRNA |
| NM_012235 | SCAP | 0.331799194 | 0.822255387 | 1.1110421 | up | mRNA |
| NM_012244 | SLC7A8 | 0.909264908 | 0.995342392 | 1.0158004 | up | mRNA |
| NM_012245 | SNW1 | 0.314452429 | 0.809707041 | 1.0698363 | up | mRNA |
| NM_012249 | RHOQ | 0.823488943 | 0.983498765 | 1.0881934 | up | mRNA |
| NM_012250 | RRAS2 | 0.910640266 | 0.995342392 | 1.0131263 | up | mRNA |
| NM_012252 | TFEC | 0.912587991 | 0.995342392 | 1.0249202 | up | mRNA |
| NM_012253 | TKTL1 | 0.103474934 | 0.533123263 | 1.1964098 | up | mRNA |
| NM_012265 | RHBDD3 | 0.874511002 | 0.990891534 | 1.0315843 | up | mRNA |
| NM_012266 | DNAJB5 | 0.247761257 | 0.745868458 | 1.398991 | up | mRNA |
| NM_012276 | LILRA4 | 0.866167072 | 0.989481868 | 1.0213645 | up | mRNA |
| NM_012278 | ITGB1BP2 | 0.683253938 | 0.960111041 | 1.055366 | up | mRNA |
| NM_012281 | KCND2 | 0.410428794 | 0.86807846 | 1.2723037 | up | mRNA |
| NM_012294 | RAPGEF5 | 0.816330559 | 0.981643774 | 1.0377257 | up | mRNA |
| NM_012298 | CAND2 | 0.293745985 | 0.791146775 | 1.0771399 | up | mRNA |
| NM_012300 | FBXW11 | 0.494453895 | 0.906930718 | 1.1226992 | up | mRNA |
| NM_012301 | MAGI2 | 0.357608542 | 0.839676686 | 1.1658547 | up | mRNA |
| NM_012302 | LPHN2 | 0.884320277 | 0.992766819 | 1.1648393 | up | mRNA |
| NM_012306 | FAIM2 | 0.54851363 | 0.92514258 | 1.0367494 | up | mRNA |
| NM_012307 | EPB41L3 | 0.756228061 | 0.97167109 | 1.071297 | up | mRNA |
| NM_012309 | SHANK2 | 0.684857609 | 0.960111041 | 1.1784054 | up | mRNA |
| NM_012317 | LDOC1 | 0.022986114 | 0.298247393 | 1.169792 | up | mRNA |
| NM_012322 | LSM5 | 0.974591883 | 0.998501532 | 1.0043341 | up | mRNA |
| NM_012331 | MSRA | 0.970732628 | 0.998277062 | 1.0120994 | up | mRNA |
| NM_012334 | MYO10 | 0.003257203 | 0.134059627 | 2.129463 | up | mRNA |
| NM_012335 | MYO1F | 0.023564378 | 0.301884214 | 1.82447 | up | mRNA |
| NM_012339 | TSPAN15 | 0.412799547 | 0.869353641 | 1.0834816 | up | mRNA |
| NM_012341 | GTPBP4 | 0.923787339 | 0.995456159 | 1.0162507 | up | mRNA |
| NM_012342 | BAMBI | 0.005287309 | 0.166411099 | 3.2756064 | up | mRNA |
| NM_012344 | NTSR2 | 0.178362021 | 0.659475654 | 1.2889162 | up | mRNA |
| NM_012364 | OR1Q1 | 0.552662335 | 0.926337111 | 1.5663547 | up | mRNA |
| NM_012369 | OR2F1 | 0.130876113 | 0.585829729 | 1.1461489 | up | mRNA |
| NM_012384 | GMEB2 | 0.617766536 | 0.946742234 | 1.0845099 | up | mRNA |
| NM_012388 | BLOC1S6 | 0.110268569 | 0.549639753 | 1.0947992 | up | mRNA |
| NM_012392 | PEF1 | 0.439211988 | 0.882895421 | 1.1069564 | up | mRNA |
| NM_012398 | PIP5K1C | 0.771436827 | 0.975347132 | 1.0229247 | up | mRNA |
| NM_012400 | PLA2G2D | 0.123247032 | 0.576324437 | 1.772196 | up | mRNA |
| NM_012410 | SEZ6L2 | 0.941115358 | 0.996710991 | 1.0073112 | up | mRNA |
| NM_012418 | FSCN2 | 0.352370705 | 0.835946719 | 1.0698512 | up | mRNA |
| NM_012420 | IFIT5 | 0.638249862 | 0.952852353 | 1.0864226 | up | mRNA |
| NM_012421 | RLF | 0.333934416 | 0.824079937 | 1.1914162 | up | mRNA |
| NM_012423 | RPL13A | 0.118472552 | 0.565973665 | 1.0938887 | up | mRNA |
| NM_012424 | RPS6KC1 | 0.097731363 | 0.520535504 | 1.2138365 | up | mRNA |
| NM_012429 | SEC14L2 | 0.200210098 | 0.68900633 | 1.2292409 | up | mRNA |
| NM_012448 | STAT5B | 0.268357573 | 0.768988803 | 1.1077514 | up | mRNA |
| NM_012451 | SYNGR4 | 0.309520702 | 0.803961878 | 1.112404 | up | mRNA |
| NM_012452 | TNFRSF13B | 0.051387136 | 0.41031217 | 1.142971 | up | mRNA |
| NM_012456 | TIMM10 | 0.971521526 | 0.998501532 | 1.0096927 | up | mRNA |
| NM_012459 | TIMM8B | 0.507984389 | 0.911674633 | 1.0693686 | up | mRNA |
| NM_012465 | TLL2 | 0.346581682 | 0.83333713 | 1.1285899 | up | mRNA |
| NM_012472 | LRRC6 | 0.78174991 | 0.976938981 | 1.0329913 | up | mRNA |
| NM_012475 | USP21 | 0.062923507 | 0.439491843 | 1.1942984 | up | mRNA |
| NM_012479 | YWHAG | 0.177837872 | 0.658188638 | 1.2195826 | up | mRNA |
| NM_012481 | IKZF3 | 0.887905762 | 0.993391292 | 1.0439394 | up | mRNA |
| NM_012486 | PSEN2 | 0.143190846 | 0.603264875 | 1.0547682 | up | mRNA |
| NM_013233 | STK39 | 0.913461573 | 0.995342392 | 1.0037904 | up | mRNA |
| NM_013238 | DNAJC15 | 0.922691279 | 0.995349793 | 1.0061323 | up | mRNA |
| NM_013241 | FHOD1 | 0.419922125 | 0.872365335 | 1.4428496 | up | mRNA |
| NM_013251 | TAC3 | 0.85890582 | 0.988512246 | 1.0155178 | up | mRNA |
| NM_013253 | DKK3 | 0.715737888 | 0.966191059 | 1.0706409 | up | mRNA |
| NM_013255 | MKLN1 | 0.676910122 | 0.959531882 | 1.0829156 | up | mRNA |
| NM_013262 | MYLIP | 0.30239889 | 0.797857839 | 1.1050834 | up | mRNA |
| NM_013269 | CLEC2D | 0.000275381 | 0.053149627 | 5.5895332 | up | mRNA |
| NM_013272 | SLCO3A1 | 0.045323459 | 0.389977391 | 1.2674745 | up | mRNA |
| NM_013280 | FLRT1 | 0.621195169 | 0.947926299 | 1.0445412 | up | mRNA |
| NM_013289 | KIR3DL1 | 0.916053634 | 0.995349793 | 1.0496584 | up | mRNA |
| NM_013293 | TRA2A | 0.228420297 | 0.724599197 | 1.0906026 | up | mRNA |
| NM_013304 | ZDHHC1 | 0.136934646 | 0.595872872 | 1.2116685 | up | mRNA |
| NM_013313 | YPEL1 | 0.571583246 | 0.933223365 | 1.0359619 | up | mRNA |
| NM_013314 | BLNK | 0.900670396 | 0.994789506 | 1.0163906 | up | mRNA |
| NM_013324 | CISH | 0.377289886 | 0.850827191 | 1.2324115 | up | mRNA |
| NM_013327 | PARVB | 0.861272667 | 0.989120869 | 1.0526295 | up | mRNA |
| NM_013329 | GCFC1 | 0.139781443 | 0.599332835 | 1.2951926 | up | mRNA |
| NM_013336 | SEC61A1 | 0.988413205 | 0.999140676 | 1.000726 | up | mRNA |
| NM_013338 | ALG5 | 0.668299797 | 0.958736264 | 1.026289 | up | mRNA |
| NM_013346 | SNX12 | 0.879603887 | 0.992178089 | 1.008623 | up | mRNA |
| NM_013347 | RPA4 | 0.002865612 | 0.127491517 | 2.0320298 | up | mRNA |
| NM_013349 | NENF | 0.804516768 | 0.98118927 | 1.032944 | up | mRNA |
| NM_013356 | SLC16A8 | 0.250762115 | 0.750330436 | 1.1380887 | up | mRNA |
| NM_013358 | PADI1 | 0.141883418 | 0.60204132 | 1.2079978 | up | mRNA |
| NM_013371 | IL19 | 0.360963203 | 0.841780454 | 1.0728697 | up | mRNA |
| NM_013372 | GREM1 | 0.186698555 | 0.6701898 | 1.2082254 | up | mRNA |
| NM_013373 | ZDHHC8 | 0.562789415 | 0.929041598 | 1.0332484 | up | mRNA |
| NM_013378 | VPREB3 | 0.101702718 | 0.529590777 | 1.1344853 | up | mRNA |
| NM_013379 | DPP7 | 0.954092399 | 0.996710991 | 1.0106921 | up | mRNA |
| NM_013380 | ZFP112 | 0.981259816 | 0.998971807 | 1.0021031 | up | mRNA |
| NM_013389 | NPC1L1 | 0.770970494 | 0.975347132 | 1.0641182 | up | mRNA |
| NM_013399 | C16orf5 | 0.770433024 | 0.975347132 | 1.0261845 | up | mRNA |
| NM_013402 | FADS1 | 0.72592341 | 0.966191059 | 1.0473534 | up | mRNA |
| NM_013409 | FST | 0.458989847 | 0.892904132 | 1.1341675 | up | mRNA |
| NM_013410 | AK4 | 0.451081066 | 0.888854908 | 1.0937227 | up | mRNA |
| NM_013416 | NCF4 | 0.795742196 | 0.980137767 | 1.0830048 | up | mRNA |
| NM_013437 | LRP12 | 0.028999842 | 0.331249882 | 1.3523504 | up | mRNA |
| NM_013442 | STOML2 | 0.697166141 | 0.962410025 | 1.0286185 | up | mRNA |
| NM_013448 | BAZ1A | 0.003935779 | 0.144623204 | 1.677735 | up | mRNA |
| NM_013449 | BAZ2A | 0.261589634 | 0.762395628 | 1.7160346 | up | mRNA |
| NM_013451 | MYOF | 0.493041867 | 0.906130999 | 1.0921336 | up | mRNA |
| NM_013453 | SPANXA1 | 0.219787491 | 0.714537777 | 1.337377 | up | mRNA |
| NM_013941 | OR10C1 | 0.352597164 | 0.835946719 | 1.3419516 | up | mRNA |
| NM_013952 | PAX8 | 0.112406941 | 0.554722799 | 1.2264637 | up | mRNA |
| NM_013956 | NRG1 | 0.420530513 | 0.872472081 | 1.4600345 | up | mRNA |
| NM_013957 | NRG1 | 0.600373372 | 0.940648177 | 1.2603981 | up | mRNA |
| NM_013959 | NRG1 | 0.634275638 | 0.951249707 | 1.2756487 | up | mRNA |
| NM_013960 | NRG1 | 0.574217876 | 0.933656777 | 1.2614406 | up | mRNA |
| NM_013962 | NRG1 | 0.300655524 | 0.795797471 | 1.1306058 | up | mRNA |
| NM_013964 | NRG1 | 0.513908706 | 0.91350662 | 1.257131 | up | mRNA |
| NM_013978 | BNIP1 | 0.656300062 | 0.957440295 | 1.0705806 | up | mRNA |
| NM_013979 | BNIP1 | 0.131178919 | 0.586182371 | 1.2143481 | up | mRNA |
| NM_013986 | EWSR1 | 0.203518179 | 0.693120873 | 1.0773962 | up | mRNA |
| NM_013987 | PARK2 | 0.489751241 | 0.904991569 | 1.062416 | up | mRNA |
| NM_013988 | PARK2 | 0.028819942 | 0.330122286 | 1.1372684 | up | mRNA |
| NM_013993 | DDR1 | 0.981069425 | 0.998971807 | 1.0025516 | up | mRNA |
| NM_013994 | DDR1 | 0.223348104 | 0.71761506 | 1.2903243 | up | mRNA |
| NM_013995 | LAMP2 | 0.736623541 | 0.968717225 | 1.0309228 | up | mRNA |
| NM_013996 | TAC1 | 0.440737771 | 0.883920737 | 1.4381696 | up | mRNA |
| NM_013997 | TAC1 | 0.28674278 | 0.782942227 | 1.8161489 | up | mRNA |
| NM_014000 | VCL | 0.950682982 | 0.996710991 | 1.0075681 | up | mRNA |
| NM_014002 | IKBKE | 0.074950119 | 0.470573816 | 1.3176178 | up | mRNA |
| NM_014007 | ZBTB43 | 0.496115992 | 0.908168754 | 1.1624136 | up | mRNA |
| NM_014011 | SOCS5 | 0.513966083 | 0.91350662 | 1.0725608 | up | mRNA |
| NM_014012 | REM1 | 0.046459196 | 0.392453681 | 2.2867425 | up | mRNA |
| NM_014014 | SNRNP200 | 0.844580801 | 0.987338076 | 1.0246229 | up | mRNA |
| NM_014017 | LAMTOR2 | 0.883244399 | 0.992766819 | 1.0095647 | up | mRNA |
| NM_014018 | MRPS28 | 0.398837455 | 0.863643939 | 1.1309028 | up | mRNA |
| NM_014030 | GIT1 | 0.331193753 | 0.821853462 | 1.176618 | up | mRNA |
| NM_014038 | BZW2 | 0.384442386 | 0.85485898 | 1.1344147 | up | mRNA |
| NM_014042 | ANAPC15 | 0.82345294 | 0.983498765 | 1.0269747 | up | mRNA |
| NM_014047 | C19orf53 | 0.613837783 | 0.944827645 | 1.0411835 | up | mRNA |
| NM_014057 | OGN | 0.148899656 | 0.61263618 | 1.4749776 | up | mRNA |
| NM_014062 | NOB1 | 0.868640854 | 0.989481868 | 1.0151313 | up | mRNA |
| NM_014063 | DBNL | 0.801070512 | 0.98118927 | 1.0183372 | up | mRNA |
| NM_014069 | PSORS1C2 | 0.246410883 | 0.744874529 | 1.0815924 | up | mRNA |
| NM_014078 | MRPL13 | 0.888537903 | 0.993440915 | 1.0155533 | up | mRNA |
| NM_014080 | DUOX2 | 0.099348068 | 0.523708821 | 1.1548842 | up | mRNA |
| NM_014089 | NUPL1 | 0.03169168 | 0.341807319 | 1.2011687 | up | mRNA |
| NM_014110 | PPP1R8 | 0.519758702 | 0.916187634 | 1.0816057 | up | mRNA |
| NM_014143 | CD274 | 0.070941649 | 0.460531803 | 5.218223 | up | mRNA |
| NM_014172 | PHPT1 | 0.956864989 | 0.996883425 | 1.0051694 | up | mRNA |
| NM_014181 | LGALSL | 0.013529886 | 0.235723775 | 1.4177291 | up | mRNA |
| NM_014182 | ORMDL2 | 0.523172699 | 0.917239107 | 1.0756768 | up | mRNA |
| NM_014187 | TMEM208 | 0.575493521 | 0.933656777 | 1.1219658 | up | mRNA |
| NM_014189 | ADD1 | 0.230185577 | 0.727477722 | 1.0985848 | up | mRNA |
| NM_014207 | CD5 | 0.072374335 | 0.463255488 | 1.4729917 | up | mRNA |
| NM_014212 | HOXC11 | 0.743699962 | 0.970330526 | 1.042087 | up | mRNA |
| NM_014213 | HOXD9 | 0.466704396 | 0.896037184 | 1.0567328 | up | mRNA |
| NM_014218 | KIR2DL1 | 0.233224384 | 0.731100552 | 1.2188721 | up | mRNA |
| NM_014220 | TM4SF1 | 0.806764091 | 0.98118927 | 1.0228959 | up | mRNA |
| NM_014224 | PGA5 | 0.06213894 | 0.439379649 | 1.1168775 | up | mRNA |
| NM_014225 | PPP2R1A | 0.737445996 | 0.968965205 | 1.0153863 | up | mRNA |
| NM_014227 | SLC5A4 | 0.238907307 | 0.736872476 | 1.1141915 | up | mRNA |
| NM_014232 | VAMP2 | 0.832853035 | 0.986434891 | 1.0424357 | up | mRNA |
| NM_014234 | HSD17B8 | 0.059320652 | 0.432586127 | 1.2467685 | up | mRNA |
| NM_014238 | KSR1 | 0.861601329 | 0.989401142 | 1.0560628 | up | mRNA |
| NM_014240 | LIMD1 | 0.947612773 | 0.996710991 | 1.0073668 | up | mRNA |
| NM_014249 | NR2E3 | 0.493159439 | 0.906265159 | 1.0494635 | up | mRNA |
| NM_014258 | SYCP2 | 0.728719058 | 0.966191059 | 1.0635681 | up | mRNA |
| NM_014275 | MGAT4B | 0.996844241 | 0.999872237 | 1.0003358 | up | mRNA |
| NM_014278 | HSPA4L | 0.05243363 | 0.412637354 | 1.2689636 | up | mRNA |
| NM_014289 | CAPN6 | 0.433825374 | 0.880923255 | 1.2105123 | up | mRNA |
| NM_014292 | CBX6 | 0.424741064 | 0.874854499 | 1.0440574 | up | mRNA |
| NM_014297 | ETHE1 | 0.804211554 | 0.98118927 | 1.0228289 | up | mRNA |
| NM_014303 | PES1 | 0.773878758 | 0.97589756 | 1.0414812 | up | mRNA |
| NM_014310 | RASD2 | 0.760659401 | 0.972931992 | 1.0756029 | up | mRNA |
| NM_014314 | DDX58 | 0.032818505 | 0.34498156 | 1.401667 | up | mRNA |
| NM_014317 | PDSS1 | 0.029864157 | 0.335098261 | 1.184348 | up | mRNA |
| NM_014325 | CORO1C | 0.637468495 | 0.952666356 | 1.1319511 | up | mRNA |
| NM_014328 | RUSC1 | 0.395932953 | 0.861849835 | 1.0359205 | up | mRNA |
| NM_014329 | EDC4 | 0.529627416 | 0.91899174 | 1.0699634 | up | mRNA |
| NM_014330 | PPP1R15A | 0.054008932 | 0.417146607 | 1.6278661 | up | mRNA |
| NM_014331 | SLC7A11 | 0.724858059 | 0.966191059 | 1.0358873 | up | mRNA |
| NM_014334 | FRRS1L | 0.879581213 | 0.992178089 | 1.0201406 | up | mRNA |
| NM_014336 | AIPL1 | 0.709512119 | 0.965462762 | 1.0299795 | up | mRNA |
| NM_014344 | FJX1 | 0.017326693 | 0.265275841 | 1.64014 | up | mRNA |
| NM_014346 | TBC1D22A | 0.830565219 | 0.985549562 | 1.0276799 | up | mRNA |
| NM_014350 | TNFAIP8 | 0.001227176 | 0.09206987 | 9.5632158 | up | mRNA |
| NM_014357 | LCE2B | 0.493982354 | 0.906630188 | 1.22472 | up | mRNA |
| NM_014359 | OPTC | 0.019236947 | 0.27881743 | 1.3887655 | up | mRNA |
| NM_014360 | NKX2-8 | 0.666313515 | 0.958354006 | 1.028358 | up | mRNA |
| NM_014365 | HSPB8 | 0.160336263 | 0.631980676 | 1.474893 | up | mRNA |
| NM_014370 | SRPK3 | 0.863754308 | 0.989401142 | 1.0404878 | up | mRNA |
| NM_014376 | CYFIP2 | 0.207521796 | 0.698556218 | 1.1105091 | up | mRNA |
| NM_014390 | SND1 | 0.765367809 | 0.974418178 | 1.0202021 | up | mRNA |
| NM_014391 | ANKRD1 | 0.745877121 | 0.970394407 | 1.1252277 | up | mRNA |
| NM_014425 | INVS | 0.084969716 | 0.495725758 | 1.0805839 | up | mRNA |
| NM_014435 | NAAA | 0.441034333 | 0.883920737 | 1.0948586 | up | mRNA |
| NM_014442 | SIGLEC8 | 0.108420201 | 0.54515118 | 1.2993181 | up | mRNA |
| NM_014447 | ARFIP1 | 0.751514319 | 0.970725371 | 1.0514398 | up | mRNA |
| NM_014449 | GPR162 | 0.719838248 | 0.966191059 | 1.0669073 | up | mRNA |
| NM_014450 | SIT1 | 0.107795437 | 0.544052509 | 1.3096775 | up | mRNA |
| NM_014468 | VENTX | 0.90932197 | 0.995342392 | 1.0321048 | up | mRNA |
| NM_014470 | RND1 | 0.31658099 | 0.811181435 | 1.3218141 | up | mRNA |
| NM_014471 | SPINK4 | 0.10357254 | 0.533123263 | 1.2321526 | up | mRNA |
| NM_014472 | R3HCC1L | 0.162626869 | 0.63494386 | 1.1203754 | up | mRNA |
| NM_014495 | ANGPTL3 | 0.173579455 | 0.65174838 | 1.1807371 | up | mRNA |
| NM_014504 | RABGEF1 | 0.5795277 | 0.933821303 | 1.035097 | up | mRNA |
| NM_014517 | UBP1 | 0.000251024 | 0.052661938 | 1.3609142 | up | mRNA |
| NM_014519 | ZNF232 | 0.404872865 | 0.866165103 | 1.1724949 | up | mRNA |
| NM_014521 | SH3BP4 | 0.31231748 | 0.807482072 | 1.2189237 | up | mRNA |
| NM_014547 | TMOD3 | 0.429622144 | 0.878226842 | 1.1172061 | up | mRNA |
| NM_014548 | TMOD2 | 0.414351146 | 0.869769512 | 1.0967097 | up | mRNA |
| NM_014570 | ARFGAP3 | 0.10081314 | 0.527707289 | 1.0960145 | up | mRNA |
| NM_014573 | TMEM97 | 0.17371617 | 0.651863583 | 1.1372313 | up | mRNA |
| NM_014576 | A1CF | 0.253526705 | 0.753559682 | 1.127428 | up | mRNA |
| NM_014583 | LMCD1 | 0.916620013 | 0.995349793 | 1.0604033 | up | mRNA |
| NM_014589 | PLA2G2E | 0.846420055 | 0.987514349 | 1.03185 | up | mRNA |
| NM_014591 | KCNIP2 | 0.582242114 | 0.934005566 | 1.0504009 | up | mRNA |
| NM_014600 | EHD3 | 0.081579904 | 0.488129303 | 1.280552 | up | mRNA |
| NM_014606 | HERC3 | 0.609620534 | 0.943053241 | 1.0419956 | up | mRNA |
| NM_014608 | CYFIP1 | 0.38288423 | 0.853514109 | 1.099966 | up | mRNA |
| NM_014612 | FAM120A | 0.734768585 | 0.967777568 | 1.0250178 | up | mRNA |
| NM_014615 | KIAA0182 | 0.480540717 | 0.901538317 | 1.0630612 | up | mRNA |
| NM_014626 | TAAR2 | 0.068081374 | 0.452806835 | 1.4979651 | up | mRNA |
| NM_014628 | MAD2L1BP | 0.734636409 | 0.967777568 | 1.0295019 | up | mRNA |
| NM_014629 | ARHGEF10 | 0.741508492 | 0.970240145 | 1.0230022 | up | mRNA |
| NM_014631 | SH3PXD2A | 0.571381381 | 0.933118573 | 1.0660348 | up | mRNA |
| NM_014640 | TTLL4 | 0.543578613 | 0.923577434 | 1.0334044 | up | mRNA |
| NM_014641 | MDC1 | 0.812379542 | 0.98118927 | 1.0340062 | up | mRNA |
| NM_014644 | PDE4DIP | 0.446992959 | 0.88691918 | 1.0800992 | up | mRNA |
| NM_014654 | SDC3 | 0.228443547 | 0.724599197 | 1.2116102 | up | mRNA |
| NM_014655 | SLC25A44 | 0.085305947 | 0.497259322 | 1.2516189 | up | mRNA |
| NM_014659 | PPIP5K1 | 0.170788422 | 0.647381721 | 1.0628715 | up | mRNA |
| NM_014662 | DEPDC5 | 0.328350251 | 0.821170223 | 1.0690933 | up | mRNA |
| NM_014665 | LRRC14 | 0.275263146 | 0.77515932 | 1.065445 | up | mRNA |
| NM_014689 | DOCK10 | 0.41079614 | 0.868341245 | 1.0890939 | up | mRNA |
| NM_014690 | FAM131B | 0.918726983 | 0.995349793 | 1.0273888 | up | mRNA |
| NM_014698 | TMEM63A | 0.293825762 | 0.791146775 | 1.1638337 | up | mRNA |
| NM_014701 | SECISBP2L | 0.723383583 | 0.966191059 | 1.0842468 | up | mRNA |
| NM_014705 | DOCK4 | 0.125579043 | 0.578441772 | 1.0910652 | up | mRNA |
| NM_014731 | PROSAPIP1 | 0.988802492 | 0.999140676 | 1.0017108 | up | mRNA |
| NM_014734 | KIAA0247 | 0.003464704 | 0.138138947 | 1.8413079 | up | mRNA |
| NM_014736 | KIAA0101 | 0.965386452 | 0.99793927 | 1.0250039 | up | mRNA |
| NM_014751 | MTSS1 | 0.008894593 | 0.202329498 | 1.4210253 | up | mRNA |
| NM_014755 | SERTAD2 | 0.009891831 | 0.207085174 | 1.3195634 | up | mRNA |
| NM_014759 | PHYHIP | 0.02215404 | 0.293936579 | 1.0669481 | up | mRNA |
| NM_014760 | TATDN2 | 0.893438821 | 0.993978977 | 1.0199563 | up | mRNA |
| NM_014761 | IST1 | 0.915083147 | 0.995349793 | 1.0153792 | up | mRNA |
| NM_014764 | DAZAP2 | 0.614109252 | 0.944827645 | 1.0380295 | up | mRNA |
| NM_014767 | SPOCK2 | 0.807987027 | 0.98118927 | 1.0168191 | up | mRNA |
| NM_014771 | RNF40 | 0.422905139 | 0.873989281 | 3.0238187 | up | mRNA |
| NM_014772 | CTIF | 0.899164375 | 0.994575817 | 1.0226345 | up | mRNA |
| NM_014775 | SFI1 | 0.524931005 | 0.917632491 | 1.3214057 | up | mRNA |
| NM_014779 | TSC22D2 | 0.815839008 | 0.981459009 | 1.0276023 | up | mRNA |
| NM_014780 | CUL7 | 0.318964117 | 0.812974553 | 1.1436265 | up | mRNA |
| NM_014784 | ARHGEF11 | 0.127072165 | 0.580406758 | 1.1490645 | up | mRNA |
| NM_014787 | DNAJC6 | 0.048254495 | 0.398851601 | 1.2146645 | up | mRNA |
| NM_014791 | MELK | 0.935086334 | 0.996710991 | 1.0379817 | up | mRNA |
| NM_014795 | ZEB2 | 0.018369646 | 0.270998737 | 1.4164851 | up | mRNA |
| NM_014807 | C2CD2L | 0.840834183 | 0.987068842 | 1.0691402 | up | mRNA |
| NM_014808 | FARP2 | 0.294170255 | 0.791146775 | 1.3312901 | up | mRNA |
| NM_014810 | CEP350 | 0.047866865 | 0.398293541 | 1.3269811 | up | mRNA |
| NM_014817 | TRIL | 0.297416413 | 0.793891363 | 1.1940801 | up | mRNA |
| NM_014826 | CDC42BPA | 0.435792315 | 0.881677506 | 1.0619997 | up | mRNA |
| NM_014829 | DDX46 | 0.999089187 | 0.999897336 | 1.0001248 | up | mRNA |
| NM_014831 | TRANK1 | 0.999117168 | 0.999897336 | 1.0002731 | up | mRNA |
| NM_014836 | RHOBTB1 | 0.822306164 | 0.983107122 | 1.0793311 | up | mRNA |
| NM_014849 | SV2A | 0.117482022 | 0.564147103 | 1.2685648 | up | mRNA |
| NM_014855 | AP5Z1 | 0.198772932 | 0.687087939 | 1.303493 | up | mRNA |
| NM_014856 | DENND4B | 0.929794002 | 0.996643872 | 1.0310623 | up | mRNA |
| NM_014860 | SUPT7L | 0.570779525 | 0.93296476 | 1.0428992 | up | mRNA |
| NM_014875 | KIF14 | 0.871353171 | 0.98985097 | 1.1019895 | up | mRNA |
| NM_014876 | JOSD1 | 0.799778126 | 0.981152656 | 1.0275721 | up | mRNA |
| NM_014879 | P2RY14 | 0.669275809 | 0.95930319 | 1.0482206 | up | mRNA |
| NM_014880 | CD302 | 0.469379824 | 0.897810636 | 1.3441989 | up | mRNA |
| NM_014886 | NSA2 | 0.218354159 | 0.712269655 | 1.08764 | up | mRNA |
| NM_014889 | PITRM1 | 0.741544405 | 0.970240145 | 1.0748551 | up | mRNA |
| NM_014893 | NLGN4Y | 0.099405073 | 0.523769488 | 1.1661028 | up | mRNA |
| NM_014900 | COBLL1 | 0.423262727 | 0.87412674 | 1.2289568 | up | mRNA |
| NM_014902 | DLGAP4 | 0.681073342 | 0.960111041 | 1.0629858 | up | mRNA |
| NM_014903 | NAV3 | 0.003749585 | 0.142058481 | 1.9864559 | up | mRNA |
| NM_014908 | DOLK | 0.908887283 | 0.995333785 | 1.0394188 | up | mRNA |
| NM_014909 | VASH1 | 0.534322377 | 0.920652989 | 1.0702314 | up | mRNA |
| NM_014912 | CPEB3 | 0.006738579 | 0.183552613 | 1.6712358 | up | mRNA |
| NM_014913 | ADNP2 | 0.80457148 | 0.98118927 | 1.045139 | up | mRNA |
| NM_014914 | AGAP1 | 0.741592074 | 0.970240145 | 1.0685549 | up | mRNA |
| NM_014915 | ANKRD26 | 0.317455253 | 0.811824899 | 1.0436636 | up | mRNA |
| NM_014920 | ICK | 0.483571649 | 0.903010541 | 1.1253382 | up | mRNA |
| NM_014921 | LPHN1 | 0.437080981 | 0.882217212 | 1.0824451 | up | mRNA |
| NM_014927 | CNKSR2 | 0.766337554 | 0.974737263 | 1.1046843 | up | mRNA |
| NM_014931 | PPP6R1 | 0.965724311 | 0.99793927 | 1.006618 | up | mRNA |
| NM_014932 | NLGN1 | 0.347484228 | 0.833732515 | 1.1326533 | up | mRNA |
| NM_014934 | DZIP1 | 0.519987249 | 0.916389075 | 1.0829112 | up | mRNA |
| NM_014936 | ENPP4 | 0.344797966 | 0.8321226 | 1.2651399 | up | mRNA |
| NM_014940 | MON1B | 0.989103714 | 0.999140676 | 1.0007467 | up | mRNA |
| NM_014943 | ZHX2 | 0.344438492 | 0.831959196 | 1.1886208 | up | mRNA |
| NM_014944 | CLSTN1 | 0.547640028 | 0.92514258 | 1.054652 | up | mRNA |
| NM_014945 | ABLIM3 | 0.013962332 | 0.238556417 | 1.596494 | up | mRNA |
| NM_014946 | SPAST | 0.589729886 | 0.936090876 | 1.0227813 | up | mRNA |
| NM_014947 | FOXJ3 | 0.960186159 | 0.997738713 | 1.0035474 | up | mRNA |
| NM_014948 | UBOX5 | 0.302794733 | 0.7981878 | 1.0806463 | up | mRNA |
| NM_014953 | DIS3 | 0.977534243 | 0.998501532 | 1.0025211 | up | mRNA |
| NM_014956 | CEP164 | 0.233684936 | 0.73118887 | 1.1745642 | up | mRNA |
| NM_014957 | DENND3 | 0.008927145 | 0.202329498 | 2.2440754 | up | mRNA |
| NM_014964 | EPN2 | 0.792807691 | 0.979003983 | 1.0351315 | up | mRNA |
| NM_014966 | DHX30 | 0.368233838 | 0.845312226 | 1.0394591 | up | mRNA |
| NM_014970 | KIFAP3 | 0.290606468 | 0.786596721 | 1.0873371 | up | mRNA |
| NM_014974 | DIP2C | 0.739429155 | 0.969564098 | 1.0354018 | up | mRNA |
| NM_014983 | HMGXB3 | 0.083264644 | 0.491989753 | 1.0884863 | up | mRNA |
| NM_014992 | DAAM1 | 0.515666278 | 0.914422683 | 1.0810133 | up | mRNA |
| NM_014999 | RAB21 | 0.719340412 | 0.966191059 | 1.039749 | up | mRNA |
| NM_015001 | SPEN | 0.222035238 | 0.716916063 | 1.1644089 | up | mRNA |
| NM_015004 | EXOSC7 | 0.184837146 | 0.668108241 | 1.0943479 | up | mRNA |
| NM_015009 | PDZRN3 | 0.002024665 | 0.108616058 | 1.3392122 | up | mRNA |
| NM_015023 | WDTC1 | 0.432001392 | 0.880287969 | 1.0505213 | up | mRNA |
| NM_015036 | ENDOD1 | 0.212768744 | 0.704792133 | 1.0987047 | up | mRNA |
| NM_015037 | KIAA0913 | 0.241308466 | 0.73945497 | 1.0587777 | up | mRNA |
| NM_015038 | KIAA0754 | 0.468236929 | 0.897203143 | 1.1535237 | up | mRNA |
| NM_015042 | ZNF609 | 0.32819737 | 0.820977969 | 1.0634248 | up | mRNA |
| NM_015043 | TBC1D9B | 0.529276802 | 0.91899174 | 1.0633305 | up | mRNA |
| NM_015050 | FTSJD2 | 0.649087819 | 0.95550188 | 1.0307517 | up | mRNA |
| NM_015062 | PPRC1 | 0.037853975 | 0.363212377 | 1.2053936 | up | mRNA |
| NM_015074 | KIF1B | 0.000332331 | 0.056781055 | 1.2160136 | up | mRNA |
| NM_015079 | TBC1D2B | 0.079549059 | 0.482639336 | 1.1808071 | up | mRNA |
| NM_015082 | FSTL4 | 0.209682096 | 0.700387116 | 1.1122311 | up | mRNA |
| NM_015086 | DDN | 0.433390715 | 0.880817674 | 1.1737458 | up | mRNA |
| NM_015089 | CUL9 | 0.856603599 | 0.988448602 | 1.0180888 | up | mRNA |
| NM_015092 | SMG1 | 0.752332151 | 0.970725371 | 1.0297164 | up | mRNA |
| NM_015093 | TAB2 | 0.210071871 | 0.700768052 | 1.2906358 | up | mRNA |
| NM_015102 | NPHP4 | 0.932548463 | 0.996710991 | 1.0075999 | up | mRNA |
| NM_015110 | SMC5 | 0.238480193 | 0.736444734 | 1.1956493 | up | mRNA |
| NM_015111 | N4BP3 | 0.195947344 | 0.683831343 | 1.4723695 | up | mRNA |
| NM_015114 | ANKLE2 | 0.001660587 | 0.104529571 | 1.7353753 | up | mRNA |
| NM_015115 | DCUN1D4 | 0.643311774 | 0.954522001 | 1.2544829 | up | mRNA |
| NM_015116 | LRCH1 | 0.80153924 | 0.98118927 | 1.0218989 | up | mRNA |
| NM_015125 | CIC | 0.316271475 | 0.810808526 | 1.1508725 | up | mRNA |
| NM_015136 | STAB1 | 0.402585461 | 0.864786348 | 1.1444284 | up | mRNA |
| NM_015137 | EFR3A | 0.589089744 | 0.936024748 | 1.1290846 | up | mRNA |
| NM_015150 | RFTN1 | 0.759315069 | 0.972558522 | 1.0317921 | up | mRNA |
| NM_015154 | MESDC2 | 0.315894299 | 0.810545544 | 1.1087293 | up | mRNA |
| NM_015158 | KANK1 | 0.920155328 | 0.995349793 | 1.0321694 | up | mRNA |
| NM_015163 | TRIM9 | 0.242868028 | 0.741106522 | 1.095942 | up | mRNA |
| NM_015166 | MLC1 | 0.349704411 | 0.834138149 | 1.1594138 | up | mRNA |
| NM_015172 | PRRC2C | 0.13800429 | 0.596873691 | 1.1920163 | up | mRNA |
| NM_015173 | TBC1D1 | 0.776777821 | 0.976652443 | 1.0119413 | up | mRNA |
| NM_015175 | NBEAL2 | 0.480934182 | 0.901538317 | 1.169151 | up | mRNA |
| NM_015179 | RRP12 | 0.944695416 | 0.996710991 | 1.0040267 | up | mRNA |
| NM_015184 | PLCL2 | 0.501613445 | 0.910145661 | 1.42919 | up | mRNA |
| NM_015186 | VPS13A | 0.521008351 | 0.916644975 | 1.1446776 | up | mRNA |
| NM_015187 | SEL1L3 | 0.321410968 | 0.815220381 | 1.4959866 | up | mRNA |
| NM_015188 | TBC1D12 | 0.140494536 | 0.600490836 | 1.1811402 | up | mRNA |
| NM_015189 | EXOC6B | 0.722792484 | 0.966191059 | 1.0199675 | up | mRNA |
| NM_015191 | SIK2 | 0.716423753 | 0.966191059 | 1.0554565 | up | mRNA |
| NM_015192 | PLCB1 | 0.060580825 | 0.435971562 | 1.3095081 | up | mRNA |
| NM_015193 | ARC | 0.366453298 | 0.845181673 | 1.2053034 | up | mRNA |
| NM_015194 | MYO1D | 0.082199937 | 0.489396524 | 1.0796477 | up | mRNA |
| NM_015197 | PACS2 | 0.280317356 | 0.778821055 | 1.1007389 | up | mRNA |
| NM_015198 | COBL | 0.54048077 | 0.923577434 | 1.0482301 | up | mRNA |
| NM_015199 | ANKRD28 | 0.15468885 | 0.622213869 | 1.0792826 | up | mRNA |
| NM_015201 | BOP1 | 0.877123434 | 0.991255275 | 1.0174721 | up | mRNA |
| NM_015213 | DENND5A | 0.30064234 | 0.795797471 | 1.1689544 | up | mRNA |
| NM_015226 | CLEC16A | 0.945805399 | 0.996710991 | 1.0108914 | up | mRNA |
| NM_015227 | POFUT2 | 0.368599991 | 0.845559574 | 1.4047486 | up | mRNA |
| NM_015230 | ARAP2 | 0.911159494 | 0.995342392 | 1.0154592 | up | mRNA |
| NM_015238 | WWC1 | 0.21042366 | 0.701136326 | 1.4330108 | up | mRNA |
| NM_015246 | MGRN1 | 0.045011794 | 0.389144768 | 1.0903732 | up | mRNA |
| NM_015247 | CYLD | 0.072047574 | 0.462687451 | 1.4068219 | up | mRNA |
| NM_015251 | ATMIN | 0.283040818 | 0.780627498 | 1.0662159 | up | mRNA |
| NM_015254 | KIF13B | 0.286873918 | 0.782942227 | 1.1320369 | up | mRNA |
| NM_015256 | ACSL6 | 0.015557868 | 0.250096132 | 1.3117899 | up | mRNA |
| NM_015260 | SIN3B | 0.203106999 | 0.692764888 | 1.2338286 | up | mRNA |
| NM_015263 | DMXL2 | 0.415713566 | 0.870382888 | 1.1052745 | up | mRNA |
| NM_015266 | SLC9A8 | 0.003202195 | 0.133416043 | 1.2081572 | up | mRNA |
| NM_015269 | ZCCHC11 | 0.78455381 | 0.977411899 | 1.0746079 | up | mRNA |
| NM_015270 | ADCY6 | 0.065003353 | 0.445228144 | 1.1333132 | up | mRNA |
| NM_015272 | RPGRIP1L | 0.995846168 | 0.999872237 | 1.0004541 | up | mRNA |
| NM_015274 | MAN2B2 | 0.114902849 | 0.559704057 | 1.1058008 | up | mRNA |
| NM_015277 | NEDD4L | 0.42822329 | 0.877211962 | 1.1638031 | up | mRNA |
| NM_015279 | TBC1D30 | 0.499729123 | 0.909460484 | 1.1237804 | up | mRNA |
| NM_015285 | WDR7 | 0.004148124 | 0.147175197 | 1.2038936 | up | mRNA |
| NM_015289 | VPS39 | 0.780176871 | 0.976938981 | 1.0242544 | up | mRNA |
| NM_015299 | KHNYN | 0.847783007 | 0.987678166 | 1.0186787 | up | mRNA |
| NM_015310 | PSD3 | 0.01317206 | 0.234534775 | 1.3063705 | up | mRNA |
| NM_015312 | KIAA1109 | 0.884468302 | 0.99276855 | 1.0067953 | up | mRNA |
| NM_015313 | ARHGEF12 | 0.646454748 | 0.95550188 | 1.0439443 | up | mRNA |
| NM_015315 | LARP1 | 0.777518047 | 0.976938981 | 1.0327508 | up | mRNA |
| NM_015316 | PPP1R13B | 0.736227301 | 0.968717225 | 1.0839655 | up | mRNA |
| NM_015330 | SPECC1L | 0.168003333 | 0.642921845 | 1.123115 | up | mRNA |
| NM_015338 | ASXL1 | 0.973967684 | 0.998501532 | 1.0054841 | up | mRNA |
| NM_015340 | LARS2 | 0.270003145 | 0.770135843 | 1.0556384 | up | mRNA |
| NM_015341 | NCAPH | 0.862356677 | 0.989401142 | 1.0196794 | up | mRNA |
| NM_015348 | TMEM131 | 0.973285176 | 0.998501532 | 1.0033455 | up | mRNA |
| NM_015350 | LRRC8B | 0.399196065 | 0.863703388 | 1.1161627 | up | mRNA |
| NM_015359 | SLC39A14 | 0.056474944 | 0.423551663 | 1.609772 | up | mRNA |
| NM_015364 | LY96 | 0.424486693 | 0.874854499 | 1.111626 | up | mRNA |
| NM_015367 | BCL2L13 | 0.621110853 | 0.947926299 | 1.0394748 | up | mRNA |
| NM_015368 | PANX1 | 0.011416254 | 0.218702965 | 1.6123841 | up | mRNA |
| NM_015374 | SUN2 | 0.614601593 | 0.944827645 | 1.0455218 | up | mRNA |
| NM_015376 | RASGRP3 | 0.662469002 | 0.958354006 | 1.0934895 | up | mRNA |
| NM_015391 | ANAPC13 | 0.718666536 | 0.966191059 | 1.0704339 | up | mRNA |
| NM_015397 | DCAF12 | 0.966552704 | 0.99793927 | 1.0040263 | up | mRNA |
| NM_015404 | DFNB31 | 0.298878976 | 0.795233272 | 1.0651662 | up | mRNA |
| NM_015407 | ABHD14A | 0.508797846 | 0.911674633 | 1.0555021 | up | mRNA |
| NM_015409 | EP400 | 0.878598236 | 0.991764342 | 1.0138484 | up | mRNA |
| NM_015414 | RPL36 | 0.851237139 | 0.987678166 | 1.0048313 | up | mRNA |
| NM_015415 | MPC2 | 0.796917663 | 0.980667273 | 1.0344522 | up | mRNA |
| NM_015424 | CHRDL2 | 0.079784858 | 0.48336881 | 1.3246206 | up | mRNA |
| NM_015429 | ABI3BP | 0.226765188 | 0.722135151 | 1.1399114 | up | mRNA |
| NM_015433 | METTL21B | 0.420511135 | 0.872472081 | 1.0692454 | up | mRNA |
| NM_015443 | KANSL1 | 0.013594081 | 0.235988767 | 1.1743023 | up | mRNA |
| NM_015457 | ZDHHC5 | 0.000147309 | 0.044621794 | 1.4617109 | up | mRNA |
| NM_015458 | MTMR9 | 0.200444702 | 0.68900633 | 1.1823887 | up | mRNA |
| NM_015459 | ATL3 | 0.854063202 | 0.987877747 | 1.0273862 | up | mRNA |
| NM_015466 | PTPN23 | 0.429934289 | 0.878446786 | 1.0741722 | up | mRNA |
| NM_015474 | SAMHD1 | 0.577432323 | 0.933700811 | 1.0396505 | up | mRNA |
| NM_015475 | FAM98A | 0.506589004 | 0.911415165 | 1.0682942 | up | mRNA |
| NM_015476 | TPGS2 | 0.952629586 | 0.996710991 | 1.0089411 | up | mRNA |
| NM_015481 | ZNF385A | 0.509479705 | 0.911674633 | 1.0205783 | up | mRNA |
| NM_015482 | SLC22A23 | 0.363096132 | 0.843238468 | 1.0612008 | up | mRNA |
| NM_015488 | PNKD | 0.927254539 | 0.995829678 | 1.0121588 | up | mRNA |
| NM_015492 | C15orf39 | 0.890854458 | 0.9937521 | 1.0116609 | up | mRNA |
| NM_015500 | C2CD2 | 0.77142579 | 0.975347132 | 1.0367843 | up | mRNA |
| NM_015510 | DHRS7B | 0.233999595 | 0.731381183 | 1.2155381 | up | mRNA |
| NM_015523 | REXO2 | 0.841973224 | 0.987123192 | 1.0202636 | up | mRNA |
| NM_015525 | IBTK | 0.730653674 | 0.966329458 | 1.0164531 | up | mRNA |
| NM_015526 | CLIP3 | 0.650645961 | 0.955894361 | 1.0518307 | up | mRNA |
| NM_015531 | C2CD3 | 0.074747658 | 0.469867247 | 1.1518773 | up | mRNA |
| NM_015532 | POLR2M | 0.223220873 | 0.71761506 | 1.1329042 | up | mRNA |
| NM_015540 | RPAP1 | 0.028423928 | 0.327830694 | 1.1989998 | up | mRNA |
| NM_015541 | LRIG1 | 0.010632168 | 0.210507384 | 1.1523647 | up | mRNA |
| NM_015547 | ACOT11 | 0.459495176 | 0.892904132 | 1.0388685 | up | mRNA |
| NM_015554 | GLCE | 0.524649353 | 0.917632259 | 1.2454736 | up | mRNA |
| NM_015556 | SIPA1L1 | 0.808831061 | 0.98118927 | 1.0685894 | up | mRNA |
| NM_015562 | UBXN7 | 0.467994035 | 0.89714751 | 1.0864295 | up | mRNA |
| NM_015564 | LRRTM2 | 0.543340215 | 0.923577434 | 1.0753499 | up | mRNA |
| NM_015567 | SLITRK5 | 0.616588437 | 0.945912508 | 1.0203909 | up | mRNA |
| NM_015570 | AUTS2 | 0.961337956 | 0.99793927 | 1.0100509 | up | mRNA |
| NM_015576 | ERC2 | 0.390392368 | 0.858920947 | 1.1845258 | up | mRNA |
| NM_015577 | RAI14 | 0.285924325 | 0.782635081 | 1.4381105 | up | mRNA |
| NM_015585 | C20orf26 | 0.916488667 | 0.995349793 | 1.0208175 | up | mRNA |
| NM_015589 | SAMD4A | 0.054780669 | 0.419413793 | 1.6991016 | up | mRNA |
| NM_015590 | GPATCH4 | 0.840647092 | 0.987068842 | 1.018564 | up | mRNA |
| NM_015595 | ARHGEF26 | 0.342703165 | 0.8313124 | 1.0802674 | up | mRNA |
| NM_015599 | PGM3 | 0.09074409 | 0.508827591 | 1.1918331 | up | mRNA |
| NM_015603 | CCDC9 | 0.286677131 | 0.782942227 | 1.1035901 | up | mRNA |
| NM_015610 | WIPI2 | 0.989129832 | 0.999140676 | 1.0018461 | up | mRNA |
| NM_015626 | WSB1 | 0.790303866 | 0.978438986 | 1.050942 | up | mRNA |
| NM_015627 | LDLRAP1 | 0.584857605 | 0.934705544 | 1.1012165 | up | mRNA |
| NM_015629 | PRPF31 | 0.710380066 | 0.965462762 | 1.0301247 | up | mRNA |
| NM_015634 | KIAA1279 | 0.029768919 | 0.334953885 | 1.2424462 | up | mRNA |
| NM_015635 | GAPVD1 | 0.433778062 | 0.880923255 | 1.0992161 | up | mRNA |
| NM_015636 | EIF2B4 | 0.63150665 | 0.950475493 | 1.0580063 | up | mRNA |
| NM_015638 | TRPC4AP | 0.999644921 | 0.999940005 | 1.0000131 | up | mRNA |
| NM_015666 | GTPBP5 | 0.558206311 | 0.926833616 | 1.1333332 | up | mRNA |
| NM_015669 | PCDHB5 | 0.986837754 | 0.999122857 | 1.0140811 | up | mRNA |
| NM_015672 | RIMBP3 | 0.682095471 | 0.960111041 | 1.0827299 | up | mRNA |
| NM_015678 | NBEA | 0.521188378 | 0.916644975 | 1.2985058 | up | mRNA |
| NM_015680 | CNPPD1 | 0.44946029 | 0.888279993 | 1.0526533 | up | mRNA |
| NM_015681 | B9D1 | 0.47160818 | 0.898908705 | 1.0536568 | up | mRNA |
| NM_015687 | FILIP1 | 0.323323047 | 0.816114734 | 1.1011923 | up | mRNA |
| NM_015689 | DENND2A | 0.585954303 | 0.93505319 | 1.08424 | up | mRNA |
| NM_015691 | WWC3 | 0.110448871 | 0.549968727 | 1.120695 | up | mRNA |
| NM_015694 | ZNF777 | 0.264916596 | 0.76613535 | 1.242219 | up | mRNA |
| NM_015695 | BRPF3 | 0.105474589 | 0.537720406 | 1.4204373 | up | mRNA |
| NM_015700 | NFU1 | 0.070761051 | 0.46010121 | 1.0607583 | up | mRNA |
| NM_015703 | RRP7A | 0.682783106 | 0.960111041 | 1.0683068 | up | mRNA |
| NM_015704 | DESI1 | 0.751724448 | 0.970725371 | 1.0281664 | up | mRNA |
| NM_015705 | SGSM3 | 0.085508626 | 0.497360491 | 1.1485473 | up | mRNA |
| NM_015714 | G0S2 | 0.166105747 | 0.640243452 | 3.0997057 | up | mRNA |
| NM_015715 | PLA2G3 | 0.284654529 | 0.78179061 | 1.1739475 | up | mRNA |
| NM_015723 | PNPLA8 | 0.141481738 | 0.601858131 | 1.1943904 | up | mRNA |
| NM_015726 | DCAF8 | 0.678683484 | 0.959891867 | 1.0642558 | up | mRNA |
| NM_015727 | TACR1 | 0.306813895 | 0.801817496 | 1.1113343 | up | mRNA |
| NM_015831 | ACHE | 0.538514292 | 0.923577434 | 1.0624951 | up | mRNA |
| NM_015836 | WARS2 | 0.36976646 | 0.846215286 | 1.1187605 | up | mRNA |
| NM_015846 | MBD1 | 0.804012526 | 0.98118927 | 1.005888 | up | mRNA |
| NM_015850 | FGFR1 | 0.177481767 | 0.65741652 | 1.0591337 | up | mRNA |
| NM_015853 | UBXN1 | 0.067767526 | 0.451819382 | 1.1668979 | up | mRNA |
| NM_015859 | GTF2A1 | 0.879698297 | 0.992178089 | 1.0179695 | up | mRNA |
| NM_015864 | FAM65B | 0.31134389 | 0.806503245 | 1.1605662 | up | mRNA |
| NM_015866 | PRDM2 | 0.091890791 | 0.511125402 | 1.2730232 | up | mRNA |
| NM_015868 | KIR2DL3 | 0.552943666 | 0.926337111 | 1.1805994 | up | mRNA |
| NM_015874 | RBPJ | 0.933204861 | 0.996710991 | 1.0034372 | up | mRNA |
| NM_015892 | CHST15 | 0.338708975 | 0.827403747 | 1.1018112 | up | mRNA |
| NM_015896 | ZMYND10 | 0.719191011 | 0.966191059 | 1.0299967 | up | mRNA |
| NM_015898 | ZBTB7A | 0.710548753 | 0.965504602 | 1.006027 | up | mRNA |
| NM_015905 | TRIM24 | 0.20474912 | 0.694790735 | 1.2666495 | up | mRNA |
| NM_015907 | LAP3 | 0.712992811 | 0.966110889 | 1.0190199 | up | mRNA |
| NM_015917 | GSTK1 | 0.997578013 | 0.999889342 | 1.0006758 | up | mRNA |
| NM_015925 | LSR | 0.769064611 | 0.975270932 | 1.0353597 | up | mRNA |
| NM_015931 | C3orf32 | 0.477250187 | 0.900450218 | 1.0678717 | up | mRNA |
| NM_015932 | POMP | 0.884880884 | 0.992981836 | 1.0114713 | up | mRNA |
| NM_015937 | PIGT | 0.069783644 | 0.457827646 | 1.0450208 | up | mRNA |
| NM_015954 | DERA | 0.884246897 | 0.992766819 | 1.0102792 | up | mRNA |
| NM_015973 | GAL | 0.532044607 | 0.920228311 | 1.36356 | up | mRNA |
| NM_015975 | TAF9B | 0.248973174 | 0.747319103 | 1.0810919 | up | mRNA |
| NM_015985 | ANGPT4 | 0.180180474 | 0.662944156 | 1.1169303 | up | mRNA |
| NM_015989 | CSAD | 0.390856711 | 0.858920947 | 1.0503128 | up | mRNA |
| NM_015993 | PLLP | 0.010387934 | 0.209736088 | 1.1508754 | up | mRNA |
| NM_016021 | UBE2J1 | 0.679838691 | 0.960007989 | 1.051415 | up | mRNA |
| NM_016032 | ZDHHC9 | 0.154812895 | 0.622213869 | 1.0611868 | up | mRNA |
| NM_016049 | EMC9 | 0.887107315 | 0.993099104 | 1.0122747 | up | mRNA |
| NM_016050 | MRPL11 | 0.589851158 | 0.936114189 | 1.0290162 | up | mRNA |
| NM_016053 | CCDC53 | 0.313960383 | 0.809054816 | 1.1045609 | up | mRNA |
| NM_016056 | TMBIM4 | 0.663622785 | 0.958354006 | 1.0421384 | up | mRNA |
| NM_016059 | PPIL1 | 0.728578679 | 0.966191059 | 1.0522682 | up | mRNA |
| NM_016060 | MED31 | 0.315183833 | 0.810240865 | 1.1557593 | up | mRNA |
| NM_016062 | FAM96B | 0.605383567 | 0.94174007 | 1.0208555 | up | mRNA |
| NM_016066 | GLRX2 | 0.935751318 | 0.996710991 | 1.0087132 | up | mRNA |
| NM_016068 | FIS1 | 0.69018266 | 0.961105466 | 1.0603201 | up | mRNA |
| NM_016069 | PAM16 | 0.592544691 | 0.937776808 | 1.0506347 | up | mRNA |
| NM_016079 | CHMP3 | 0.332396658 | 0.822707947 | 1.0367739 | up | mRNA |
| NM_016080 | GLOD4 | 0.728315745 | 0.966191059 | 1.0506985 | up | mRNA |
| NM_016084 | RASD1 | 0.177629188 | 0.657722936 | 1.4301697 | up | mRNA |
| NM_016089 | ZNF589 | 0.300662131 | 0.795797471 | 1.1603171 | up | mRNA |
| NM_016096 | ZNF706 | 0.051740655 | 0.41031217 | 1.0634209 | up | mRNA |
| NM_016098 | MPC1 | 0.898916198 | 0.99453218 | 1.0143314 | up | mRNA |
| NM_016107 | ZFR | 0.295252872 | 0.79157694 | 1.0898736 | up | mRNA |
| NM_016111 | TELO2 | 0.567939594 | 0.931705674 | 1.0470028 | up | mRNA |
| NM_016112 | PKD2L1 | 0.193041194 | 0.680109784 | 1.7411928 | up | mRNA |
| NM_016118 | NUB1 | 0.062780856 | 0.439458294 | 1.2296959 | up | mRNA |
| NM_016128 | COPG1 | 0.979752341 | 0.998971807 | 1.0023118 | up | mRNA |
| NM_016134 | CPQ | 0.935679593 | 0.996710991 | 1.0266314 | up | mRNA |
| NM_016139 | CHCHD2 | 0.567743866 | 0.931705674 | 1.0261568 | up | mRNA |
| NM_016152 | RARB | 0.888727241 | 0.993440915 | 1.042316 | up | mRNA |
| NM_016167 | NOL7 | 0.478482527 | 0.901271944 | 1.0646781 | up | mRNA |
| NM_016170 | TLX2 | 0.43171812 | 0.880121832 | 1.1952705 | up | mRNA |
| NM_016201 | AMOTL2 | 0.322395465 | 0.815372756 | 1.3115002 | up | mRNA |
| NM_016210 | C3orf18 | 0.435628178 | 0.881645489 | 1.1633146 | up | mRNA |
| NM_016219 | MAN1B1 | 0.747482862 | 0.970620903 | 1.0963855 | up | mRNA |
| NM_016226 | VPS29 | 0.725533105 | 0.966191059 | 1.018037 | up | mRNA |
| NM_016229 | CYB5R2 | 0.451286556 | 0.888854908 | 1.1114206 | up | mRNA |
| NM_016237 | ANAPC5 | 0.351055101 | 0.835187247 | 1.0962397 | up | mRNA |
| NM_016246 | HSD17B14 | 0.803597987 | 0.98118927 | 1.042638 | up | mRNA |
| NM_016247 | IMPG2 | 0.570917054 | 0.933029103 | 1.0296966 | up | mRNA |
| NM_016248 | AKAP11 | 0.367129879 | 0.845181673 | 1.0994548 | up | mRNA |
| NM_016249 | MAGEC2 | 0.556676364 | 0.926833616 | 1.142237 | up | mRNA |
| NM_016262 | TUBE1 | 0.576414001 | 0.933656777 | 1.1091908 | up | mRNA |
| NM_016269 | LEF1 | 0.52297706 | 0.917239107 | 1.154634 | up | mRNA |
| NM_016276 | SGK2 | 0.000969015 | 0.085290119 | 1.7620323 | up | mRNA |
| NM_016283 | TAF9 | 0.976801992 | 0.998501532 | 1.0028184 | up | mRNA |
| NM_016286 | DCXR | 0.385494834 | 0.85515381 | 1.0807294 | up | mRNA |
| NM_016290 | UIMC1 | 0.921478126 | 0.995349793 | 1.0187632 | up | mRNA |
| NM_016304 | RSL24D1 | 0.774541102 | 0.97589756 | 1.0260434 | up | mRNA |
| NM_016310 | POLR3K | 0.905707624 | 0.99508468 | 1.0144002 | up | mRNA |
| NM_016321 | RHCG | 0.938994427 | 0.996710991 | 1.0135941 | up | mRNA |
| NM_016337 | EVL | 0.939462535 | 0.996710991 | 1.0072822 | up | mRNA |
| NM_016341 | PLCE1 | 0.888465667 | 0.993440915 | 1.0305679 | up | mRNA |
| NM_016346 | NR2E3 | 0.760224574 | 0.972883253 | 1.0601174 | up | mRNA |
| NM_016350 | NIN | 0.234579332 | 0.731962397 | 1.1125371 | up | mRNA |
| NM_016351 | ADAM22 | 0.943583591 | 0.996710991 | 1.0074159 | up | mRNA |
| NM_016357 | LIMA1 | 0.483028615 | 0.902654749 | 1.1064632 | up | mRNA |
| NM_016361 | ACP6 | 0.192856104 | 0.679693241 | 1.0582045 | up | mRNA |
| NM_016362 | GHRL | 0.307081729 | 0.80210505 | 1.102795 | up | mRNA |
| NM_016368 | ISYNA1 | 0.625989147 | 0.948901166 | 1.0511815 | up | mRNA |
| NM_016370 | RAB9B | 0.317445352 | 0.811824899 | 1.2470893 | up | mRNA |
| NM_016381 | TREX1 | 0.788239094 | 0.977641695 | 1.0504918 | up | mRNA |
| NM_016382 | CD244 | 0.816969337 | 0.981692737 | 1.0310628 | up | mRNA |
| NM_016388 | TRAT1 | 0.740292592 | 0.969794316 | 1.0239052 | up | mRNA |
| NM_016401 | C11orf73 | 0.424279712 | 0.874817544 | 1.0352345 | up | mRNA |
| NM_016410 | CHMP5 | 0.685027164 | 0.960111041 | 1.0144707 | up | mRNA |
| NM_016433 | GLTP | 0.953192439 | 0.996710991 | 1.003109 | up | mRNA |
| NM_016437 | TUBG2 | 0.956712758 | 0.996883425 | 1.0087675 | up | mRNA |
| NM_016438 | HIGD1B | 0.149188747 | 0.613036702 | 1.2961124 | up | mRNA |
| NM_016440 | VRK3 | 0.369799317 | 0.846215286 | 1.0316989 | up | mRNA |
| NM_016442 | ERAP1 | 0.833988483 | 0.986475536 | 1.0195929 | up | mRNA |
| NM_016445 | PLEK2 | 0.912828857 | 0.995342392 | 1.0440309 | up | mRNA |
| NM_016454 | EMC4 | 0.742711473 | 0.970330526 | 1.0246259 | up | mRNA |
| NM_016456 | TMEM9 | 0.657049141 | 0.957445617 | 1.0816585 | up | mRNA |
| NM_016462 | TMEM14C | 0.556927085 | 0.926833616 | 1.0372955 | up | mRNA |
| NM_016470 | C20orf111 | 0.196219115 | 0.684309956 | 1.4410446 | up | mRNA |
| NM_016478 | ZC3HC1 | 0.633384862 | 0.951174475 | 1.0301992 | up | mRNA |
| NM_016483 | PHF7 | 0.681123199 | 0.960111041 | 1.1600964 | up | mRNA |
| NM_016484 | PDZD11 | 0.408981852 | 0.867591329 | 1.0489627 | up | mRNA |
| NM_016490 | FAM178B | 0.915704277 | 0.995349793 | 1.0079271 | up | mRNA |
| NM_016492 | RANGRF | 0.602431321 | 0.941406011 | 1.06435 | up | mRNA |
| NM_016495 | TBC1D7 | 0.690766537 | 0.961105466 | 1.0491895 | up | mRNA |
| NM_016500 | CXorf26 | 0.938443668 | 0.996710991 | 1.0073802 | up | mRNA |
| NM_016505 | ZCCHC17 | 0.100888261 | 0.527859014 | 1.1555102 | up | mRNA |
| NM_016507 | CDK12 | 0.261520437 | 0.762395628 | 1.1952738 | up | mRNA |
| NM_016508 | CDKL3 | 0.781158384 | 0.976938981 | 1.0182049 | up | mRNA |
| NM_016509 | CLEC1B | 0.010911367 | 0.213728231 | 1.1581041 | up | mRNA |
| NM_016526 | BET1L | 0.055775525 | 0.421571734 | 1.3778686 | up | mRNA |
| NM_016529 | ATP8A2 | 0.319331885 | 0.813412163 | 1.2161854 | up | mRNA |
| NM_016531 | KLF3 | 0.004432207 | 0.152997695 | 1.5998606 | up | mRNA |
| NM_016532 | INPP5K | 0.896950768 | 0.994222243 | 1.0259314 | up | mRNA |
| NM_016535 | ZNF581 | 0.742412681 | 0.970330526 | 1.0297795 | up | mRNA |
| NM_016542 | MST4 | 0.037076915 | 0.359628653 | 1.2800455 | up | mRNA |
| NM_016545 | IER5 | 0.473518116 | 0.899184677 | 1.2226204 | up | mRNA |
| NM_016546 | C1RL | 0.836633892 | 0.986725216 | 1.0710427 | up | mRNA |
| NM_016551 | TM7SF3 | 0.965519758 | 0.99793927 | 1.0022926 | up | mRNA |
| NM_016563 | RASL12 | 0.034709135 | 0.352074963 | 1.6737082 | up | mRNA |
| NM_016569 | TBX3 | 0.074071196 | 0.468871594 | 2.1047137 | up | mRNA |
| NM_016570 | ERGIC2 | 0.908915756 | 0.995333785 | 1.0108367 | up | mRNA |
| NM_016574 | DRD2 | 0.166520079 | 0.641349924 | 1.0613435 | up | mRNA |
| NM_016582 | SLC15A3 | 0.069574512 | 0.457632202 | 1.4929871 | up | mRNA |
| NM_016583 | BPIFA1 | 0.509132573 | 0.911674633 | 1.1182497 | up | mRNA |
| NM_016584 | IL23A | 5.84818E-05 | 0.0304885 | 10.3444997 | up | mRNA |
| NM_016585 | THEG | 0.234827344 | 0.732399072 | 1.0563042 | up | mRNA |
| NM_016594 | FKBP11 | 0.049416437 | 0.402500646 | 1.1179141 | up | mRNA |
| NM_016598 | ZDHHC3 | 0.458176643 | 0.892646883 | 1.0948527 | up | mRNA |
| NM_016605 | FAM53C | 0.506675468 | 0.911415165 | 1.1425762 | up | mRNA |
| NM_016609 | SLC22A17 | 0.721624154 | 0.966191059 | 1.0177476 | up | mRNA |
| NM_016612 | SLC25A37 | 5.47808E-05 | 0.030102801 | 2.9613397 | up | mRNA |
| NM_016614 | TDP2 | 0.957251774 | 0.996883425 | 1.0057481 | up | mRNA |
| NM_016617 | UFM1 | 0.623653911 | 0.948341211 | 1.0537288 | up | mRNA |
| NM_016639 | TNFRSF12A | 0.917421196 | 0.995349793 | 1.0147392 | up | mRNA |
| NM_016644 | PRR16 | 0.385122771 | 0.855147437 | 1.3932585 | up | mRNA |
| NM_016647 | THEM6 | 0.973155505 | 0.998501532 | 1.0010719 | up | mRNA |
| NM_016734 | PAX5 | 0.238822669 | 0.736834977 | 1.1502122 | up | mRNA |
| NM_016828 | OGG1 | 0.972328752 | 0.998501532 | 1.0044062 | up | mRNA |
| NM_016835 | MAPT | 0.698939281 | 0.962701213 | 1.0271419 | up | mRNA |
| NM_016841 | MAPT | 0.378797895 | 0.852482258 | 1.1508025 | up | mRNA |
| NM_016848 | SHC3 | 0.499787186 | 0.909484746 | 1.2678177 | up | mRNA |
| NM_016937 | POLA1 | 0.841498396 | 0.987096602 | 1.1042316 | up | mRNA |
| NM_016952 | CDON | 0.701279927 | 0.963015537 | 1.1814807 | up | mRNA |
| NM_016953 | PDE11A | 0.523560667 | 0.917239107 | 1.0598247 | up | mRNA |
| NM_017409 | HOXC10 | 0.741889651 | 0.970330526 | 1.0471062 | up | mRNA |
| NM_017413 | APLN | 0.093922775 | 0.513205555 | 1.0810288 | up | mRNA |
| NM_017417 | GALNT8 | 0.53302103 | 0.920530493 | 1.0816388 | up | mRNA |
| NM_017424 | CECR1 | 0.845799072 | 0.987338076 | 1.0330629 | up | mRNA |
| NM_017431 | PRKAG3 | 0.94976323 | 0.996710991 | 1.005417 | up | mRNA |
| NM_017433 | MYO3A | 0.454630165 | 0.89077195 | 1.2329371 | up | mRNA |
| NM_017436 | A4GALT | 0.041990237 | 0.378937196 | 1.4659154 | up | mRNA |
| NM_017444 | CHRAC1 | 0.656661268 | 0.957445617 | 1.0244788 | up | mRNA |
| NM_017446 | MRPL39 | 0.362532962 | 0.843173209 | 1.0414893 | up | mRNA |
| NM_017447 | C21orf91 | 0.758519506 | 0.972072638 | 1.0572933 | up | mRNA |
| NM_017449 | EPHB2 | 0.643379359 | 0.954552622 | 1.0761563 | up | mRNA |
| NM_017450 | BAIAP2 | 0.189362967 | 0.674719659 | 1.1119456 | up | mRNA |
| NM_017453 | STAU1 | 0.125516288 | 0.578441772 | 1.2256056 | up | mRNA |
| NM_017481 | UBQLN3 | 0.290172156 | 0.786468159 | 1.2139444 | up | mRNA |
| NM_017488 | ADD2 | 0.311747755 | 0.806920827 | 1.0661906 | up | mRNA |
| NM_017493 | OTUD4 | 0.000733584 | 0.079656701 | 1.4979569 | up | mRNA |
| NM_017495 | RBM38 | 0.990363791 | 0.999209835 | 1.0013983 | up | mRNA |
| NM_017508 | SOX6 | 0.294437419 | 0.791301949 | 1.0969223 | up | mRNA |
| NM_017512 | ENOSF1 | 0.766658619 | 0.974840715 | 1.0494858 | up | mRNA |
| NM_017523 | XAF1 | 0.811562428 | 0.98118927 | 1.0798898 | up | mRNA |
| NM_017542 | POGK | 0.084859608 | 0.495509924 | 1.2057598 | up | mRNA |
| NM_017548 | CDV3 | 0.010388286 | 0.209736088 | 1.3620572 | up | mRNA |
| NM_017549 | EPDR1 | 0.907007409 | 0.995145546 | 1.0348553 | up | mRNA |
| NM_017554 | PARP14 | 0.034049396 | 0.349898235 | 1.2897434 | up | mRNA |
| NM_017556 | FBLIM1 | 0.909941065 | 0.995342392 | 1.00549 | up | mRNA |
| NM_017566 | KLHDC4 | 0.919126501 | 0.995349793 | 1.0119426 | up | mRNA |
| NM_017575 | SMG6 | 0.763059195 | 0.97349057 | 1.0592656 | up | mRNA |
| NM_017584 | MIOX | 0.007811536 | 0.193678037 | 1.126541 | up | mRNA |
| NM_017585 | SLC2A6 | 0.03354691 | 0.347820389 | 1.6709013 | up | mRNA |
| NM_017590 | ZC3H7B | 0.068877553 | 0.455528425 | 1.2100165 | up | mRNA |
| NM_017602 | OTUD5 | 0.263905559 | 0.764495522 | 1.0772978 | up | mRNA |
| NM_017611 | SLC43A3 | 0.004348881 | 0.15088983 | 1.5502342 | up | mRNA |
| NM_017613 | DONSON | 0.813681797 | 0.98120545 | 1.0271531 | up | mRNA |
| NM_017631 | DDX60 | 0.120545091 | 0.571843862 | 1.4173126 | up | mRNA |
| NM_017636 | TRPM4 | 0.651481808 | 0.956204521 | 1.2219731 | up | mRNA |
| NM_017649 | CNNM2 | 0.602341834 | 0.941406011 | 1.2154686 | up | mRNA |
| NM_017650 | PPP1R9A | 0.320349716 | 0.814552264 | 1.0507232 | up | mRNA |
| NM_017653 | DYM | 0.9520544 | 0.996710991 | 1.0084382 | up | mRNA |
| NM_017656 | ZNF562 | 0.941366536 | 0.996710991 | 1.0130859 | up | mRNA |
| NM_017657 | AFTPH | 0.562761288 | 0.929041598 | 1.1164216 | up | mRNA |
| NM_017659 | QPCTL | 0.096660259 | 0.518684713 | 1.2014895 | up | mRNA |
| NM_017660 | GATAD2A | 0.788474066 | 0.977641695 | 1.0351566 | up | mRNA |
| NM_017665 | ZCCHC10 | 0.252480078 | 0.752621656 | 1.0896373 | up | mRNA |
| NM_017668 | NDE1 | 0.583352615 | 0.934280269 | 1.0640634 | up | mRNA |
| NM_017672 | TRPM7 | 0.523712746 | 0.917239107 | 1.056155 | up | mRNA |
| NM_017675 | CDHR2 | 0.58388362 | 0.934397621 | 1.0903327 | up | mRNA |
| NM_017680 | ASPN | 0.546647581 | 0.924803608 | 1.1187425 | up | mRNA |
| NM_017684 | VPS13C | 0.0498185 | 0.404032605 | 1.2630965 | up | mRNA |
| NM_017688 | BSPRY | 0.242388006 | 0.740622663 | 1.0883266 | up | mRNA |
| NM_017691 | LRRC49 | 0.001798283 | 0.105670067 | 2.4342548 | up | mRNA |
| NM_017702 | DEF8 | 0.955280566 | 0.996883425 | 1.0048947 | up | mRNA |
| NM_017703 | FBXL12 | 0.794467803 | 0.979333052 | 1.0174415 | up | mRNA |
| NM_017707 | ASAP3 | 0.07828672 | 0.480303439 | 1.5022142 | up | mRNA |
| NM_017716 | MS4A12 | 0.381413126 | 0.853514109 | 1.2546905 | up | mRNA |
| NM_017723 | TOR4A | 0.421187164 | 0.872885778 | 1.0701565 | up | mRNA |
| NM_017745 | BCOR | 0.005174326 | 0.16463912 | 1.2531497 | up | mRNA |
| NM_017750 | RETSAT | 0.730125453 | 0.966191059 | 1.1489239 | up | mRNA |
| NM_017760 | NCAPG2 | 0.632971012 | 0.951174475 | 1.1398529 | up | mRNA |
| NM_017761 | PNRC2 | 0.013656406 | 0.236019158 | 1.3986329 | up | mRNA |
| NM_017763 | RNF43 | 0.375028098 | 0.849710156 | 1.0972733 | up | mRNA |
| NM_017769 | G2E3 | 0.775025862 | 0.97589756 | 1.0458834 | up | mRNA |
| NM_017771 | PXK | 0.875462796 | 0.991094934 | 1.0094059 | up | mRNA |
| NM_017773 | LAX1 | 0.596207425 | 0.939114453 | 1.2745018 | up | mRNA |
| NM_017786 | SYBU | 0.8330214 | 0.986434891 | 1.0214442 | up | mRNA |
| NM_017790 | RGS3 | 0.519399819 | 0.916145597 | 1.0186012 | up | mRNA |
| NM_017794 | FOCAD | 0.645600925 | 0.955130468 | 1.0580942 | up | mRNA |
| NM_017802 | HEATR2 | 0.907373713 | 0.995145546 | 1.0164005 | up | mRNA |
| NM_017805 | RASIP1 | 0.484145716 | 0.903250293 | 1.3961452 | up | mRNA |
| NM_017806 | LIME1 | 0.108595383 | 0.545714614 | 1.1163383 | up | mRNA |
| NM_017814 | TMEM161A | 0.208046686 | 0.698793461 | 1.080195 | up | mRNA |
| NM_017828 | COMMD4 | 0.190773977 | 0.676402188 | 1.1663697 | up | mRNA |
| NM_017829 | CECR5 | 0.313242571 | 0.808636478 | 1.2677375 | up | mRNA |
| NM_017831 | RNF125 | 0.060590841 | 0.435971562 | 1.6022013 | up | mRNA |
| NM_017837 | PIGV | 0.064508169 | 0.443826413 | 1.8872544 | up | mRNA |
| NM_017838 | NHP2 | 0.471711959 | 0.898908705 | 1.0786854 | up | mRNA |
| NM_017842 | SLC48A1 | 0.312413411 | 0.807524722 | 1.0741833 | up | mRNA |
| NM_017854 | TMEM160 | 0.576311479 | 0.933656777 | 1.0556137 | up | mRNA |
| NM_017865 | ZNF692 | 0.58467808 | 0.93461341 | 1.0786513 | up | mRNA |
| NM_017870 | TMEM132A | 0.095384682 | 0.516597924 | 1.2366769 | up | mRNA |
| NM_017877 | C2orf18 | 0.501136303 | 0.909916293 | 1.0826933 | up | mRNA |
| NM_017884 | PINX1 | 0.852573548 | 0.987678166 | 1.0308342 | up | mRNA |
| NM_017887 | C1orf123 | 0.929075357 | 0.996456574 | 1.0197845 | up | mRNA |
| NM_017890 | VPS13B | 0.696715731 | 0.962410025 | 1.0705321 | up | mRNA |
| NM_017899 | TESC | 0.30148068 | 0.796581367 | 1.1753099 | up | mRNA |
| NM_017908 | ZNF446 | 0.103223427 | 0.532945334 | 1.1151504 | up | mRNA |
| NM_017911 | FAM118A | 0.921561111 | 0.995349793 | 1.0085841 | up | mRNA |
| NM_017920 | URGCP | 0.23723892 | 0.735183924 | 1.3663437 | up | mRNA |
| NM_017921 | NPLOC4 | 0.857740299 | 0.988512246 | 1.0094998 | up | mRNA |
| NM_017926 | C14orf118 | 0.096522978 | 0.518358476 | 1.0728505 | up | mRNA |
| NM_017929 | PEX26 | 0.420757947 | 0.872676791 | 1.0958709 | up | mRNA |
| NM_017933 | PID1 | 0.000603726 | 0.07439369 | 1.2786318 | up | mRNA |
| NM_017935 | BANK1 | 0.783959722 | 0.977347603 | 1.0170383 | up | mRNA |
| NM_017954 | CADPS2 | 0.044801517 | 0.38849334 | 1.3571326 | up | mRNA |
| NM_017966 | VPS37C | 0.70395566 | 0.963625105 | 1.065346 | up | mRNA |
| NM_017971 | MRPL20 | 0.721015881 | 0.966191059 | 1.0298789 | up | mRNA |
| NM_017975 | ZWILCH | 0.86468326 | 0.989401142 | 1.0452118 | up | mRNA |
| NM_017982 | SUSD4 | 0.473096909 | 0.899184677 | 1.1768465 | up | mRNA |
| NM_017987 | RUFY2 | 0.416414449 | 0.870736736 | 1.0579591 | up | mRNA |
| NM_017999 | RNF31 | 0.61250555 | 0.944159427 | 1.0463806 | up | mRNA |
| NM_018000 | MREG | 0.137397658 | 0.596770033 | 1.2248606 | up | mRNA |
| NM_018014 | BCL11A | 0.984983831 | 0.998971807 | 1.001761 | up | mRNA |
| NM_018027 | FRMD4A | 0.754194593 | 0.970944772 | 1.0280624 | up | mRNA |
| NM_018028 | SAMD4B | 0.278852946 | 0.777783404 | 1.0997793 | up | mRNA |
| NM_018029 | EBLN2 | 0.144702383 | 0.605951408 | 1.1025036 | up | mRNA |
| NM_018031 | WDR6 | 0.955942015 | 0.996883425 | 1.0044685 | up | mRNA |
| NM_018042 | SLFN12 | 0.842275391 | 0.987123192 | 1.1041789 | up | mRNA |
| NM_018043 | ANO1 | 0.120918755 | 0.572400147 | 1.2048311 | up | mRNA |
| NM_018045 | BSDC1 | 0.008381507 | 0.198394583 | 1.3120728 | up | mRNA |
| NM_018047 | RBM22 | 0.448444098 | 0.888104977 | 1.0933768 | up | mRNA |
| NM_018048 | MAGOHB | 0.937561849 | 0.996710991 | 1.0135154 | up | mRNA |
| NM_018050 | MANSC1 | 0.836716971 | 0.986725216 | 1.007336 | up | mRNA |
| NM_018054 | ARHGAP17 | 0.673439088 | 0.959531882 | 1.0143994 | up | mRNA |
| NM_018057 | SLC6A15 | 0.384031577 | 0.854561675 | 1.1557993 | up | mRNA |
| NM_018059 | RADIL | 0.498243226 | 0.909116151 | 1.077993 | up | mRNA |
| NM_018070 | SSBP3 | 0.997742629 | 0.999889342 | 1.0005162 | up | mRNA |
| NM_018071 | ARHGEF40 | 0.963916083 | 0.99793927 | 1.0082989 | up | mRNA |
| NM_018079 | SRBD1 | 0.207078036 | 0.698556218 | 1.1109331 | up | mRNA |
| NM_018083 | ZNF358 | 0.725111463 | 0.966191059 | 1.0378349 | up | mRNA |
| NM_018097 | HAUS2 | 0.641536001 | 0.954332328 | 1.1551071 | up | mRNA |
| NM_018098 | ECT2 | 0.690907845 | 0.961105466 | 1.2085491 | up | mRNA |
| NM_018100 | EFHC1 | 0.682900204 | 0.960111041 | 1.0234623 | up | mRNA |
| NM_018109 | MTPAP | 0.59112455 | 0.937021663 | 1.069955 | up | mRNA |
| NM_018117 | WDR11 | 0.754204528 | 0.970944772 | 1.0640727 | up | mRNA |
| NM_018129 | PNPO | 0.842171786 | 0.987123192 | 1.0111237 | up | mRNA |
| NM_018134 | IQCC | 0.349143916 | 0.834138149 | 1.1437725 | up | mRNA |
| NM_018140 | CEP72 | 0.965280246 | 0.99793927 | 1.004423 | up | mRNA |
| NM_018150 | RNF220 | 0.415058202 | 0.870239791 | 1.0475874 | up | mRNA |
| NM_018151 | RIF1 | 0.626817242 | 0.949203235 | 1.059947 | up | mRNA |
| NM_018154 | ASF1B | 0.739295082 | 0.969564098 | 1.0489224 | up | mRNA |
| NM_018156 | VPS13D | 0.534096748 | 0.920652989 | 1.0869513 | up | mRNA |
| NM_018159 | NUDT11 | 0.039506301 | 0.370322288 | 1.2243423 | up | mRNA |
| NM_018176 | LGI2 | 0.034238036 | 0.350517496 | 1.6761225 | up | mRNA |
| NM_018177 | N4BP2 | 0.803053842 | 0.98118927 | 1.0726807 | up | mRNA |
| NM_018184 | ARL8B | 0.005587078 | 0.169833068 | 1.2095288 | up | mRNA |
| NM_018186 | C1orf112 | 0.91875274 | 0.995349793 | 1.0458117 | up | mRNA |
| NM_018188 | ATAD3A | 0.996465907 | 0.999872237 | 1.0006733 | up | mRNA |
| NM_018192 | LEPREL1 | 0.004724731 | 0.157739293 | 2.0772656 | up | mRNA |
| NM_018198 | DNAJC11 | 0.929622024 | 0.996533591 | 1.0069852 | up | mRNA |
| NM_018199 | EXD2 | 0.392841378 | 0.859694706 | 1.0686174 | up | mRNA |
| NM_018200 | HMG20A | 0.649543171 | 0.95550188 | 1.0508116 | up | mRNA |
| NM_018207 | TRIM62 | 0.307134633 | 0.802113712 | 1.2377814 | up | mRNA |
| NM_018209 | ARFGAP1 | 0.486206594 | 0.903524001 | 1.1921143 | up | mRNA |
| NM_018211 | RAVER2 | 0.952424388 | 0.996710991 | 1.0292111 | up | mRNA |
| NM_018218 | USP40 | 0.125457181 | 0.578441772 | 1.2661513 | up | mRNA |
| NM_018223 | CHFR | 0.865114728 | 0.989401142 | 1.0192974 | up | mRNA |
| NM_018224 | COA1 | 0.703849536 | 0.963625105 | 1.0811988 | up | mRNA |
| NM_018240 | KIRREL | 0.351956807 | 0.835686762 | 1.1082464 | up | mRNA |
| NM_018250 | INTS9 | 0.774672501 | 0.97589756 | 1.0429717 | up | mRNA |
| NM_018262 | IFT122 | 0.002527755 | 0.11946717 | 1.193601 | up | mRNA |
| NM_018264 | TYW1 | 0.138906111 | 0.598648178 | 1.0690822 | up | mRNA |
| NM_018269 | ADI1 | 0.509186871 | 0.911674633 | 1.0597726 | up | mRNA |
| NM_018271 | THNSL2 | 0.196688294 | 0.685161614 | 1.0912856 | up | mRNA |
| NM_018273 | TMEM143 | 0.583396729 | 0.934280269 | 1.097713 | up | mRNA |
| NM_018275 | C7orf43 | 0.370400951 | 0.846215286 | 1.1178997 | up | mRNA |
| NM_018280 | C22orf26 | 0.73749442 | 0.968965205 | 1.0602548 | up | mRNA |
| NM_018284 | GBP3 | 0.021943183 | 0.292173405 | 3.0476432 | up | mRNA |
| NM_018287 | ARHGAP12 | 0.209729894 | 0.700431703 | 1.0537827 | up | mRNA |
| NM_018291 | FGGY | 0.397872306 | 0.863112281 | 1.136389 | up | mRNA |
| NM_018297 | NGLY1 | 0.186478225 | 0.669888134 | 1.1839584 | up | mRNA |
| NM_018306 | TMEM40 | 0.970038698 | 0.998245244 | 1.0106136 | up | mRNA |
| NM_018310 | BRF2 | 0.506326879 | 0.911352523 | 1.1591316 | up | mRNA |
| NM_018324 | OLAH | 0.043643587 | 0.385351177 | 1.152477 | up | mRNA |
| NM_018334 | LRRN3 | 0.725281313 | 0.966191059 | 1.0923617 | up | mRNA |
| NM_018335 | ZNF839 | 0.924526938 | 0.995456159 | 1.0198933 | up | mRNA |
| NM_018344 | SLC29A3 | 0.295459247 | 0.79157694 | 1.2439519 | up | mRNA |
| NM_018362 | LIN7C | 0.768436834 | 0.975142015 | 1.0171306 | up | mRNA |
| NM_018370 | DRAM1 | 0.001430745 | 0.09664422 | 3.3317986 | up | mRNA |
| NM_018378 | FBXL8 | 0.441327087 | 0.883920737 | 1.0974602 | up | mRNA |
| NM_018380 | DDX28 | 0.252944556 | 0.753288068 | 1.0974843 | up | mRNA |
| NM_018381 | C19orf66 | 0.934606319 | 0.996710991 | 1.0113376 | up | mRNA |
| NM_018390 | PLCXD1 | 0.155459084 | 0.623056705 | 1.5310486 | up | mRNA |
| NM_018402 | IL26 | 0.819977449 | 0.982484618 | 1.22797 | up | mRNA |
| NM_018403 | DCP1A | 0.68934685 | 0.96108227 | 1.0368413 | up | mRNA |
| NM_018405 | C17orf79 | 0.831001149 | 0.985710869 | 1.0212647 | up | mRNA |
| NM_018413 | CHST11 | 0.044043539 | 0.385988468 | 1.1411631 | up | mRNA |
| NM_018419 | SOX18 | 0.19575729 | 0.683572955 | 1.0654262 | up | mRNA |
| NM_018425 | PI4K2A | 0.897158926 | 0.994222243 | 1.0065464 | up | mRNA |
| NM_018426 | TMEM63B | 0.106675326 | 0.541344461 | 1.1790481 | up | mRNA |
| NM_018428 | UTP6 | 0.180289248 | 0.663104378 | 1.177046 | up | mRNA |
| NM_018434 | RNF130 | 0.787686656 | 0.977492986 | 1.0345777 | up | mRNA |
| NM_018441 | PECR | 0.1137597 | 0.557825663 | 1.082445 | up | mRNA |
| NM_018462 | BRK1 | 0.69768057 | 0.962410025 | 1.0238538 | up | mRNA |
| NM_018464 | CISD1 | 0.988097631 | 0.999140676 | 1.0027419 | up | mRNA |
| NM_018465 | PLGRKT | 0.118226428 | 0.565462182 | 1.2888117 | up | mRNA |
| NM_018466 | ALG13 | 0.636614069 | 0.952018038 | 1.05469 | up | mRNA |
| NM_018473 | ACOT13 | 0.449030426 | 0.888279993 | 1.0527905 | up | mRNA |
| NM_018475 | TMEM165 | 0.602322531 | 0.941406011 | 1.0764301 | up | mRNA |
| NM_018482 | ASAP1 | 0.000274983 | 0.053149627 | 1.2695124 | up | mRNA |
| NM_018485 | GPR77 | 0.978798077 | 0.998661138 | 1.0078089 | up | mRNA |
| NM_018509 | LRRC59 | 0.70899027 | 0.965462762 | 1.0265377 | up | mRNA |
| NM_018534 | NRP2 | 0.359696537 | 0.841291842 | 1.1464597 | up | mRNA |
| NM_018559 | KIAA1704 | 0.796993011 | 0.980667273 | 1.029127 | up | mRNA |
| NM_018584 | CAMK2N1 | 0.810456346 | 0.98118927 | 1.0207347 | up | mRNA |
| NM_018638 | ETNK1 | 0.807451355 | 0.98118927 | 1.026976 | up | mRNA |
| NM_018646 | TRPV6 | 0.47535513 | 0.899815707 | 1.0963508 | up | mRNA |
| NM_018652 | GOLGA6B | 0.83832339 | 0.986897498 | 1.0226233 | up | mRNA |
| NM_018653 | GPRC5C | 0.486416128 | 0.903524001 | 1.198208 | up | mRNA |
| NM_018664 | BATF3 | 0.936425769 | 0.996710991 | 1.023691 | up | mRNA |
| NM_018666 | SAGE1 | 0.788093213 | 0.977639488 | 1.0224296 | up | mRNA |
| NM_018671 | UNC45A | 0.341823987 | 0.830223283 | 1.1840235 | up | mRNA |
| NM_018672 | ABCA5 | 0.335339283 | 0.825134282 | 1.6072838 | up | mRNA |
| NM_018676 | THSD1 | 0.331326827 | 0.82187807 | 1.3879974 | up | mRNA |
| NM_018683 | RNF114 | 0.844793306 | 0.987338076 | 1.0515225 | up | mRNA |
| NM_018694 | ARL6IP4 | 0.714319632 | 0.966110889 | 1.0472616 | up | mRNA |
| NM_018702 | ADARB2 | 0.143495983 | 0.603870418 | 1.1437771 | up | mRNA |
| NM_018710 | TMEM55A | 0.983915973 | 0.998971807 | 1.0021692 | up | mRNA |
| NM_018712 | ELMOD1 | 0.048867819 | 0.40128453 | 1.3073225 | up | mRNA |
| NM_018724 | IL20 | 0.553844219 | 0.926354119 | 1.0485699 | up | mRNA |
| NM_018725 | IL17RB | 0.286861531 | 0.782942227 | 1.2615852 | up | mRNA |
| NM_018842 | BAIAP2L1 | 0.753427393 | 0.970725371 | 1.0396518 | up | mRNA |
| NM_018844 | BCAP29 | 0.784974844 | 0.977411899 | 1.0137008 | up | mRNA |
| NM_018845 | SLC50A1 | 0.342288623 | 0.830677046 | 1.0799143 | up | mRNA |
| NM_018846 | KLHL7 | 0.020348483 | 0.285032405 | 1.1670791 | up | mRNA |
| NM_018890 | RAC1 | 0.840807589 | 0.987068842 | 1.0150181 | up | mRNA |
| NM_018891 | LAMC2 | 0.023871651 | 0.303389731 | 1.6234218 | up | mRNA |
| NM_018902 | PCDHA11 | 0.930210987 | 0.996649933 | 1.0044397 | up | mRNA |
| NM_018903 | PCDHA12 | 0.151295151 | 0.616500055 | 1.2909355 | up | mRNA |
| NM_018905 | PCDHA2 | 0.64513774 | 0.954828387 | 1.1042753 | up | mRNA |
| NM_018907 | PCDHA4 | 0.282908603 | 0.780627498 | 1.3765022 | up | mRNA |
| NM_018915 | PCDHGA2 | 0.803993828 | 0.98118927 | 1.039606 | up | mRNA |
| NM_018930 | PCDHB10 | 0.909817393 | 0.995342392 | 1.0064297 | up | mRNA |
| NM_018934 | PCDHB14 | 0.883494137 | 0.992766819 | 1.051889 | up | mRNA |
| NM_018941 | CLN8 | 0.670249555 | 0.959531882 | 1.0422822 | up | mRNA |
| NM_018947 | CYCS | 0.028593704 | 0.328520317 | 1.1795829 | up | mRNA |
| NM_018948 | ERRFI1 | 0.137568287 | 0.596789644 | 1.123926 | up | mRNA |
| NM_018950 | HLA-F | 0.237581311 | 0.735771951 | 1.2357945 | up | mRNA |
| NM_018951 | HOXA10 | 0.810668141 | 0.98118927 | 1.0245477 | up | mRNA |
| NM_018953 | HOXC5 | 0.697376109 | 0.962410025 | 1.0285691 | up | mRNA |
| NM_018955 | UBB | 0.204354118 | 0.694397489 | 1.1385142 | up | mRNA |
| NM_018957 | SH3BP1 | 0.967296261 | 0.99793927 | 1.0046343 | up | mRNA |
| NM_018958 | NPAP1 | 0.79354593 | 0.97913499 | 1.0216372 | up | mRNA |
| NM_018960 | GNMT | 0.300689157 | 0.795797471 | 1.2009168 | up | mRNA |
| NM_018971 | GPR27 | 0.780061957 | 0.976938981 | 1.0092427 | up | mRNA |
| NM_018974 | UNC93A | 0.281226963 | 0.779126326 | 1.1080859 | up | mRNA |
| NM_019000 | FAM134B | 0.183495829 | 0.666935501 | 1.7896542 | up | mRNA |
| NM_019012 | PLEKHA5 | 0.369438699 | 0.846072048 | 1.1478306 | up | mRNA |
| NM_019015 | CHPF2 | 0.044556609 | 0.387767262 | 1.1861284 | up | mRNA |
| NM_019020 | TBC1D16 | 0.544176596 | 0.923577434 | 1.0694055 | up | mRNA |
| NM_019021 | C11orf71 | 0.687219965 | 0.960205549 | 1.0533192 | up | mRNA |
| NM_019027 | RBM47 | 0.057062295 | 0.425914308 | 1.0818393 | up | mRNA |
| NM_019032 | ADAMTSL4 | 0.788141484 | 0.97763972 | 1.0265581 | up | mRNA |
| NM_019034 | RHOF | 0.779416445 | 0.976938981 | 1.0989665 | up | mRNA |
| NM_019041 | MTRF1L | 0.686669558 | 0.960205549 | 1.0551543 | up | mRNA |
| NM_019043 | APBB1IP | 0.752347005 | 0.970725371 | 1.1315462 | up | mRNA |
| NM_019044 | CCDC93 | 0.142048654 | 0.60204132 | 1.119937 | up | mRNA |
| NM_019055 | ROBO4 | 0.49600394 | 0.908134987 | 1.0639045 | up | mRNA |
| NM_019056 | NDUFB11 | 0.652429311 | 0.95661646 | 1.046361 | up | mRNA |
| NM_019058 | DDIT4 | 0.005586219 | 0.169833068 | 2.6157627 | up | mRNA |
| NM_019059 | TOMM7 | 0.999393488 | 0.999897336 | 1.0000686 | up | mRNA |
| NM_019060 | CRCT1 | 0.212714087 | 0.704725896 | 2.036231 | up | mRNA |
| NM_019062 | RNF186 | 0.181173431 | 0.664543371 | 1.2833956 | up | mRNA |
| NM_019065 | NECAB2 | 0.067968995 | 0.452403247 | 1.1908454 | up | mRNA |
| NM_019066 | MAGEL2 | 0.508409434 | 0.911674633 | 1.0480639 | up | mRNA |
| NM_019073 | SPATA6 | 0.76232625 | 0.97339948 | 1.0359507 | up | mRNA |
| NM_019076 | UGT1A8 | 0.665343995 | 0.958354006 | 1.0438348 | up | mRNA |
| NM_019084 | CCNJ | 0.278252625 | 0.777440204 | 1.2932135 | up | mRNA |
| NM_019088 | PAF1 | 0.901485634 | 0.994902084 | 1.0097925 | up | mRNA |
| NM_019089 | HES2 | 0.136394493 | 0.595125435 | 1.1354183 | up | mRNA |
| NM_019094 | NUDT4 | 0.233410052 | 0.731130266 | 1.1633376 | up | mRNA |
| NM_019096 | GTPBP2 | 0.130316584 | 0.584331216 | 1.2100699 | up | mRNA |
| NM_019101 | APOM | 0.256392056 | 0.757240077 | 1.3316305 | up | mRNA |
| NM_019107 | C19orf10 | 0.815963918 | 0.98149313 | 1.0169776 | up | mRNA |
| NM_019109 | ALG1 | 0.799041021 | 0.980917337 | 1.0220558 | up | mRNA |
| NM_019111 | HLA-DRA | 0.582374478 | 0.934005566 | 1.1058432 | up | mRNA |
| NM_019114 | EPB41L4B | 0.435237288 | 0.881486657 | 1.1473449 | up | mRNA |
| NM_019116 | UBFD1 | 0.163384514 | 0.635655173 | 1.2005521 | up | mRNA |
| NM_019117 | KLHL4 | 0.986357804 | 0.998971807 | 1.0083207 | up | mRNA |
| NM_019121 | PPP1R37 | 0.897938766 | 0.994326043 | 1.0112756 | up | mRNA |
| NM_019554 | S100A4 | 0.513627362 | 0.91350662 | 1.1776556 | up | mRNA |
| NM_019590 | KIAA1217 | 0.002949419 | 0.129519648 | 1.5270803 | up | mRNA |
| NM_019593 | GPCPD1 | 0.493463478 | 0.90641426 | 1.0435883 | up | mRNA |
| NM_019598 | KLK12 | 0.00501697 | 0.16140036 | 1.3529008 | up | mRNA |
| NM_019605 | SERTAD4 | 0.807751706 | 0.98118927 | 1.1092539 | up | mRNA |
| NM_019610 | RBMXL1 | 0.113915727 | 0.557835051 | 1.5653156 | up | mRNA |
| NM_019617 | GKN1 | 0.311952826 | 0.80699254 | 1.1863162 | up | mRNA |
| NM_019619 | PARD3 | 0.769563521 | 0.975286259 | 1.0173616 | up | mRNA |
| NM_019644 | ANKRD7 | 0.714048514 | 0.966110889 | 1.0570717 | up | mRNA |
| NM_019839 | LTB4R2 | 0.756906003 | 0.971860616 | 1.0402011 | up | mRNA |
| NM_019852 | METTL3 | 0.992436679 | 0.999781402 | 1.0007542 | up | mRNA |
| NM_019856 | MYOM1 | 0.713725557 | 0.966110889 | 1.0343688 | up | mRNA |
| NM_019858 | GPR162 | 0.210171636 | 0.700954461 | 1.1568694 | up | mRNA |
| NM_019887 | DIABLO | 0.323990082 | 0.816480706 | 1.0872237 | up | mRNA |
| NM_019892 | INPP5E | 0.552286264 | 0.926337111 | 1.03903 | up | mRNA |
| NM_019896 | POLE4 | 0.487801921 | 0.903757389 | 1.0580183 | up | mRNA |
| NM_020061 | OPN1LW | 0.01408098 | 0.239157213 | 1.2450538 | up | mRNA |
| NM_020062 | SLC2A4RG | 0.574527465 | 0.933656777 | 1.1562655 | up | mRNA |
| NM_020064 | BARHL1 | 0.850988602 | 0.987678166 | 1.0552191 | up | mRNA |
| NM_020107 | ACRV1 | 0.922687054 | 0.995349793 | 1.0098251 | up | mRNA |
| NM_020108 | ACRV1 | 0.624140873 | 0.948341211 | 1.1037005 | up | mRNA |
| NM_020117 | LARS | 0.002896252 | 0.12797004 | 1.1056473 | up | mRNA |
| NM_020119 | ZC3HAV1 | 0.189007917 | 0.6744312 | 1.167469 | up | mRNA |
| NM_020125 | SLAMF8 | 0.700648486 | 0.962991468 | 1.0256824 | up | mRNA |
| NM_020129 | LGALS14 | 0.159605064 | 0.631463351 | 1.2356677 | up | mRNA |
| NM_020131 | UBQLN4 | 0.29640934 | 0.792868663 | 1.1067886 | up | mRNA |
| NM_020139 | BDH2 | 0.648671145 | 0.95550188 | 1.0272187 | up | mRNA |
| NM_020140 | ANKS1B | 0.073404438 | 0.466102133 | 1.2237004 | up | mRNA |
| NM_020147 | THAP10 | 0.195718002 | 0.683572955 | 1.1582789 | up | mRNA |
| NM_020150 | SAR1A | 0.348731577 | 0.834138149 | 1.0891528 | up | mRNA |
| NM_020151 | STARD7 | 0.290621413 | 0.786596721 | 1.0739372 | up | mRNA |
| NM_020163 | SEMA3G | 0.664113858 | 0.958354006 | 1.0429694 | up | mRNA |
| NM_020170 | NCLN | 0.084606378 | 0.49502644 | 1.2412646 | up | mRNA |
| NM_020178 | CA10 | 0.853393499 | 0.987678166 | 1.0687576 | up | mRNA |
| NM_020180 | CELF4 | 0.667906565 | 0.958354006 | 1.0595301 | up | mRNA |
| NM_020182 | PMEPA1 | 0.219627213 | 0.714276371 | 1.1591683 | up | mRNA |
| NM_020183 | ARNTL2 | 0.162101439 | 0.634236412 | 1.1063484 | up | mRNA |
| NM_020185 | DUSP22 | 0.605599514 | 0.94174007 | 1.1150297 | up | mRNA |
| NM_020189 | ENY2 | 0.475718977 | 0.900105754 | 1.0545496 | up | mRNA |
| NM_020194 | MFF | 0.987762151 | 0.999140676 | 1.0025816 | up | mRNA |
| NM_020198 | CCDC47 | 0.094814044 | 0.516057441 | 1.1268074 | up | mRNA |
| NM_020199 | C5orf15 | 0.850510204 | 0.987678166 | 1.0160004 | up | mRNA |
| NM_020201 | NT5M | 0.129777664 | 0.583907255 | 1.1485255 | up | mRNA |
| NM_020204 | LHX9 | 0.669663218 | 0.959531882 | 1.053081 | up | mRNA |
| NM_020208 | SLC6A20 | 0.744401545 | 0.970330526 | 1.0784945 | up | mRNA |
| NM_020225 | STOX2 | 0.462674336 | 0.894989844 | 1.0175392 | up | mRNA |
| NM_020233 | ADPRM | 0.811939954 | 0.98118927 | 1.0406077 | up | mRNA |
| NM_020235 | BBX | 0.695130493 | 0.962214902 | 1.0560423 | up | mRNA |
| NM_020245 | TULP4 | 0.716521559 | 0.966191059 | 1.0445976 | up | mRNA |
| NM_020246 | SLC12A9 | 0.577312136 | 0.933656777 | 1.0699592 | up | mRNA |
| NM_020251 | ARRB1 | 0.851566738 | 0.987678166 | 1.02266 | up | mRNA |
| NM_020307 | CCNL1 | 0.005169314 | 0.16463912 | 1.4195064 | up | mRNA |
| NM_020311 | CXCR7 | 0.038762476 | 0.367764188 | 1.5734558 | up | mRNA |
| NM_020318 | PAPPA2 | 0.152444369 | 0.618255775 | 1.2403414 | up | mRNA |
| NM_020322 | ASIC3 | 0.497509951 | 0.908789022 | 1.1007537 | up | mRNA |
| NM_020344 | SLC24A2 | 0.249310991 | 0.747319103 | 1.5053264 | up | mRNA |
| NM_020351 | COL8A1 | 0.074491282 | 0.469775665 | 1.1227811 | up | mRNA |
| NM_020354 | ENTPD7 | 0.711832156 | 0.96592341 | 1.0673927 | up | mRNA |
| NM_020365 | EIF2B3 | 0.073904647 | 0.468039773 | 1.0653525 | up | mRNA |
| NM_020369 | FSCN3 | 0.001029818 | 0.0865217 | 1.4415979 | up | mRNA |
| NM_020386 | HRASLS | 0.451268175 | 0.888854908 | 1.1567164 | up | mRNA |
| NM_020393 | PGLYRP4 | 0.263808959 | 0.764495522 | 1.1950448 | up | mRNA |
| NM_020396 | BCL2L10 | 0.367885983 | 0.845181673 | 1.1197516 | up | mRNA |
| NM_020399 | GOPC | 0.839456218 | 0.987032229 | 1.0592712 | up | mRNA |
| NM_020403 | PCDH9 | 0.255591225 | 0.756337058 | 1.1558771 | up | mRNA |
| NM_020407 | RHBG | 0.028219472 | 0.326555662 | 1.1958686 | up | mRNA |
| NM_020410 | ATP13A1 | 0.309479841 | 0.803961878 | 1.1057734 | up | mRNA |
| NM_020422 | TMEM159 | 0.690295803 | 0.961105466 | 1.087093 | up | mRNA |
| NM_020433 | JPH2 | 0.116539959 | 0.562996237 | 1.1076254 | up | mRNA |
| NM_020435 | GJC2 | 0.996147551 | 0.999872237 | 1.0007856 | up | mRNA |
| NM_020459 | PAIP2B | 0.379428289 | 0.852847753 | 1.1020964 | up | mRNA |
| NM_020469 | ABO | 0.912166688 | 0.995342392 | 1.0056465 | up | mRNA |
| NM_020474 | GALNT1 | 0.853655708 | 0.987737756 | 1.0395225 | up | mRNA |
| NM_020476 | ANK1 | 0.385105684 | 0.855147437 | 1.3196075 | up | mRNA |
| NM_020478 | ANK1 | 0.262687596 | 0.763125731 | 1.2331127 | up | mRNA |
| NM_020480 | ANK1 | 0.784820367 | 0.977411899 | 1.0525426 | up | mRNA |
| NM_020485 | RHCE | 0.009557875 | 0.205206664 | 1.1653244 | up | mRNA |
| NM_020529 | NFKBIA | 0.0002616 | 0.052661938 | 11.1731211 | up | mRNA |
| NM_020530 | OSM | 0.502312843 | 0.910335149 | 1.0914224 | up | mRNA |
| NM_020533 | MCOLN1 | 0.311862279 | 0.806920827 | 1.0744084 | up | mRNA |
| NM_020535 | KIR2DL5A | 0.075398637 | 0.471913234 | 1.0979792 | up | mRNA |
| NM_020630 | RET | 0.547274829 | 0.925055514 | 1.0490239 | up | mRNA |
| NM_020632 | ATP6V0A4 | 0.588880994 | 0.935839874 | 1.0941197 | up | mRNA |
| NM_020639 | RIPK4 | 0.68366119 | 0.960111041 | 1.0747454 | up | mRNA |
| NM_020659 | TTYH1 | 0.498812734 | 0.909361611 | 1.136425 | up | mRNA |
| NM_020664 | DECR2 | 0.647850561 | 0.95550188 | 1.0397643 | up | mRNA |
| NM_020673 | RAB22A | 0.67564051 | 0.959531882 | 1.0447754 | up | mRNA |
| NM_020683 | ADORA3 | 0.096631137 | 0.518665334 | 1.0852838 | up | mRNA |
| NM_020685 | C3orf14 | 0.976297212 | 0.998501532 | 1.0063366 | up | mRNA |
| NM_020686 | ABAT | 0.971369546 | 0.998501532 | 1.0059199 | up | mRNA |
| NM_020689 | SLC24A3 | 0.582089127 | 0.934005566 | 1.0983005 | up | mRNA |
| NM_020697 | KCNS2 | 0.619302976 | 0.947444797 | 1.0646626 | up | mRNA |
| NM_020702 | KIAA1161 | 0.963649589 | 0.99793927 | 1.0225686 | up | mRNA |
| NM_020703 | AMIGO1 | 0.736436705 | 0.968717225 | 1.0643161 | up | mRNA |
| NM_020704 | FAM40B | 0.471167538 | 0.898834526 | 1.1563425 | up | mRNA |
| NM_020706 | SCAF4 | 0.680241317 | 0.960111041 | 1.0699505 | up | mRNA |
| NM_020722 | KIAA1211 | 0.940641892 | 0.996710991 | 1.0108536 | up | mRNA |
| NM_020725 | ATXN7L1 | 0.659441169 | 0.958354006 | 1.0669465 | up | mRNA |
| NM_020727 | ZNF295 | 0.026598569 | 0.318484367 | 1.5848043 | up | mRNA |
| NM_020730 | DLG3 | 0.223381158 | 0.71761506 | 1.1104109 | up | mRNA |
| NM_020737 | LRFN2 | 0.967999129 | 0.99793927 | 1.0027141 | up | mRNA |
| NM_020754 | ARHGAP31 | 0.024400352 | 0.304929683 | 2.1590395 | up | mRNA |
| NM_020761 | RPTOR | 0.83214989 | 0.98620142 | 1.0219949 | up | mRNA |
| NM_020765 | UBR4 | 0.13295483 | 0.588930122 | 1.1527047 | up | mRNA |
| NM_020768 | KCTD16 | 0.609668338 | 0.943053241 | 1.2025568 | up | mRNA |
| NM_020772 | NUFIP2 | 0.068667087 | 0.454916653 | 1.0902077 | up | mRNA |
| NM_020773 | TBC1D14 | 0.127488829 | 0.580991132 | 1.2232395 | up | mRNA |
| NM_020775 | KIAA1324 | 0.032719764 | 0.344901399 | 1.3408215 | up | mRNA |
| NM_020782 | KLHDC5 | 0.031200965 | 0.340775376 | 1.2511545 | up | mRNA |
| NM_020783 | SYT4 | 0.592782698 | 0.937820942 | 1.1205393 | up | mRNA |
| NM_020784 | TXNDC16 | 0.972883253 | 0.998501532 | 1.0042808 | up | mRNA |
| NM_020785 | CC2D2A | 0.043393599 | 0.384435145 | 1.2190005 | up | mRNA |
| NM_020796 | SEMA6A | 0.580597353 | 0.93393768 | 1.035912 | up | mRNA |
| NM_020801 | ARRDC3 | 0.005522871 | 0.169833068 | 1.3174584 | up | mRNA |
| NM_020803 | KLHL8 | 0.954947637 | 0.996883425 | 1.0074235 | up | mRNA |
| NM_020806 | GPHN | 0.464239385 | 0.894989844 | 1.0749981 | up | mRNA |
| NM_020809 | ARHGAP20 | 0.282880454 | 0.780627498 | 1.0996387 | up | mRNA |
| NM_020820 | PREX1 | 0.000673405 | 0.076919551 | 1.8143376 | up | mRNA |
| NM_020825 | CRAMP1L | 0.537755287 | 0.923027035 | 1.0515407 | up | mRNA |
| NM_020826 | SYT13 | 0.498204281 | 0.909116151 | 1.0604279 | up | mRNA |
| NM_020828 | ZFP28 | 0.279190083 | 0.777816764 | 1.2885355 | up | mRNA |
| NM_020831 | MKL1 | 0.700840721 | 0.962991468 | 1.0884849 | up | mRNA |
| NM_020840 | FNIP2 | 0.288617392 | 0.784514546 | 1.4785833 | up | mRNA |
| NM_020841 | OSBPL8 | 0.85060569 | 0.987678166 | 1.0532097 | up | mRNA |
| NM_020848 | KIAA1462 | 0.884193577 | 0.992766819 | 1.0250706 | up | mRNA |
| NM_020859 | SHROOM3 | 0.67497731 | 0.959531882 | 1.0550862 | up | mRNA |
| NM_020862 | LRFN1 | 0.084701354 | 0.495154665 | 1.3230753 | up | mRNA |
| NM_020892 | DTX2 | 0.685827116 | 0.960111041 | 1.0413647 | up | mRNA |
| NM_020896 | OSBPL5 | 0.530253601 | 0.91899174 | 1.0481343 | up | mRNA |
| NM_020897 | HCN3 | 0.348703921 | 0.834138149 | 1.1887288 | up | mRNA |
| NM_020898 | CALCOCO1 | 0.409302382 | 0.867591329 | 1.078627 | up | mRNA |
| NM_020902 | CAMSAP3 | 0.262837297 | 0.763125731 | 1.0523276 | up | mRNA |
| NM_020904 | PLEKHA4 | 0.003589105 | 0.139262737 | 1.357923 | up | mRNA |
| NM_020910 | KIAA1549 | 0.838423963 | 0.986906502 | 1.016672 | up | mRNA |
| NM_020924 | ZBTB26 | 0.578668166 | 0.933821303 | 1.0436515 | up | mRNA |
| NM_020925 | CACHD1 | 0.90680257 | 0.995145546 | 1.0243742 | up | mRNA |
| NM_020927 | VAT1L | 0.206295916 | 0.69755672 | 1.3124868 | up | mRNA |
| NM_020933 | ZNF317 | 0.257631706 | 0.7580303 | 1.1654739 | up | mRNA |
| NM_020939 | CPNE5 | 0.953071567 | 0.996710991 | 1.0218679 | up | mRNA |
| NM_020946 | DENND1A | 0.66360022 | 0.958354006 | 1.1141965 | up | mRNA |
| NM_020954 | RNF213 | 0.098292458 | 0.521660729 | 1.0985852 | up | mRNA |
| NM_020956 | PRX | 0.567350422 | 0.931514776 | 1.0498232 | up | mRNA |
| NM_020957 | PCDHB16 | 0.941703122 | 0.996710991 | 1.0051806 | up | mRNA |
| NM_020962 | IGDCC4 | 0.003881125 | 0.143735942 | 1.2094298 | up | mRNA |
| NM_020963 | MOV10 | 0.741334055 | 0.970240145 | 1.0303061 | up | mRNA |
| NM_020967 | NCOA5 | 0.65932963 | 0.958354006 | 1.0336754 | up | mRNA |
| NM_020978 | AMY2B | 0.552765766 | 0.926337111 | 1.1358788 | up | mRNA |
| NM_020979 | SH2B2 | 0.402335555 | 0.864786348 | 1.0902322 | up | mRNA |
| NM_020988 | GNAO1 | 0.94732487 | 0.996710991 | 1.0023713 | up | mRNA |
| NM_020995 | HPR | 0.676736557 | 0.959531882 | 1.0387004 | up | mRNA |
| NM_020996 | FGF6 | 0.117441467 | 0.564147103 | 1.7530165 | up | mRNA |
| NM_020999 | NEUROG3 | 0.017184295 | 0.263492524 | 1.4161391 | up | mRNA |
| NM_021003 | PPM1A | 0.876312635 | 0.991213905 | 1.0155469 | up | mRNA |
| NM_021006 | CCL3L1 | 0.045759424 | 0.391180343 | 6.9881716 | up | mRNA |
| NM_021008 | DEAF1 | 0.245944175 | 0.744459872 | 1.1108678 | up | mRNA |
| NM_021009 | UBC | 0.194480411 | 0.682224934 | 1.112574 | up | mRNA |
| NM_021019 | MYL6 | 0.644608217 | 0.954657712 | 1.008534 | up | mRNA |
| NM_021023 | CFHR3 | 0.099093662 | 0.523009123 | 1.3491299 | up | mRNA |
| NM_021027 | UGT1A9 | 0.00750635 | 0.192457886 | 1.1501383 | up | mRNA |
| NM_021029 | RPL36A | 0.632742509 | 0.951123179 | 1.0193617 | up | mRNA |
| NM_021032 | FGF12 | 0.687274348 | 0.960205549 | 1.0668014 | up | mRNA |
| NM_021034 | IFITM3 | 0.82522699 | 0.98381451 | 1.0627807 | up | mRNA |
| NM_021035 | ZNFX1 | 0.138046359 | 0.596928661 | 1.1771594 | up | mRNA |
| NM_021064 | HIST1H2AG | 0.963654565 | 0.99793927 | 1.033804 | up | mRNA |
| NM_021069 | SORBS2 | 0.671289557 | 0.959531882 | 1.0693002 | up | mRNA |
| NM_021076 | NEFH | 0.694094537 | 0.961951598 | 1.0464502 | up | mRNA |
| NM_021077 | NMB | 0.319200861 | 0.81318029 | 1.5515132 | up | mRNA |
| NM_021078 | KAT2A | 0.943278523 | 0.996710991 | 1.0108244 | up | mRNA |
| NM_021082 | SLC15A2 | 0.467828039 | 0.897093245 | 1.2017557 | up | mRNA |
| NM_021088 | ZNF2 | 0.694771579 | 0.962202557 | 1.0791014 | up | mRNA |
| NM_021096 | CACNA1I | 0.631275441 | 0.950410442 | 1.0780487 | up | mRNA |
| NM_021101 | CLDN1 | 0.007559903 | 0.193257016 | 5.1217047 | up | mRNA |
| NM_021102 | SPINT2 | 0.200503269 | 0.68900633 | 1.1634387 | up | mRNA |
| NM_021103 | TMSB10 | 0.287119709 | 0.782954059 | 1.0588205 | up | mRNA |
| NM_021109 | TMSB4X | 0.711248049 | 0.965746983 | 1.0227898 | up | mRNA |
| NM_021110 | COL14A1 | 0.649849251 | 0.95550188 | 1.1463007 | up | mRNA |
| NM_021114 | SPINK2 | 0.755203724 | 0.971417904 | 1.0280979 | up | mRNA |
| NM_021115 | SEZ6L | 0.27040799 | 0.770234694 | 1.1614372 | up | mRNA |
| NM_021117 | CRY2 | 0.525062248 | 0.917686109 | 1.1256512 | up | mRNA |
| NM_021118 | CYLC1 | 0.144782212 | 0.605951408 | 1.3016587 | up | mRNA |
| NM_021121 | EEF1B2 | 0.821146447 | 0.982756843 | 1.0066968 | up | mRNA |
| NM_021126 | MPST | 0.738949764 | 0.969564098 | 1.0557127 | up | mRNA |
| NM_021127 | PMAIP1 | 0.002193303 | 0.11120755 | 3.4079821 | up | mRNA |
| NM_021128 | POLR2L | 0.908221061 | 0.995302455 | 1.0064852 | up | mRNA |
| NM_021129 | PPA1 | 0.074488383 | 0.469775665 | 1.0878571 | up | mRNA |
| NM_021134 | MRPL23 | 0.450825128 | 0.888798265 | 1.0726294 | up | mRNA |
| NM_021136 | RTN1 | 0.920770889 | 0.995349793 | 1.0172497 | up | mRNA |
| NM_021137 | TNFAIP1 | 0.012903827 | 0.231767331 | 1.3008502 | up | mRNA |
| NM_021138 | TRAF2 | 0.348178294 | 0.834138149 | 1.1485774 | up | mRNA |
| NM_021144 | PSIP1 | 0.898174821 | 0.994360027 | 1.0203807 | up | mRNA |
| NM_021149 | COTL1 | 0.038274564 | 0.365250856 | 1.1251136 | up | mRNA |
| NM_021155 | CD209 | 0.763665397 | 0.973679653 | 1.0179504 | up | mRNA |
| NM_021158 | TRIB3 | 0.125962184 | 0.578441772 | 1.2678819 | up | mRNA |
| NM_021160 | ABHD16A | 0.142546104 | 0.602652383 | 1.0718088 | up | mRNA |
| NM_021170 | HES4 | 0.248854465 | 0.747319103 | 1.3672924 | up | mRNA |
| NM_021174 | KIAA1967 | 0.691153178 | 0.961119378 | 1.1066281 | up | mRNA |
| NM_021181 | SLAMF7 | 0.072720575 | 0.463915802 | 1.3623277 | up | mRNA |
| NM_021183 | RAP2C | 0.001347712 | 0.094163853 | 1.8643452 | up | mRNA |
| NM_021185 | CATSPERG | 0.221202792 | 0.716640124 | 1.0670569 | up | mRNA |
| NM_021186 | ZP4 | 0.361880584 | 0.842812834 | 1.2387453 | up | mRNA |
| NM_021189 | CADM3 | 0.787070749 | 0.977479172 | 1.0320279 | up | mRNA |
| NM_021192 | HOXD11 | 0.164093194 | 0.636706644 | 1.1183996 | up | mRNA |
| NM_021195 | CLDN6 | 0.079569066 | 0.482639336 | 1.0877323 | up | mRNA |
| NM_021199 | SQRDL | 0.005116708 | 0.163643802 | 1.7590596 | up | mRNA |
| NM_021215 | RPRD1B | 0.025204445 | 0.310579867 | 1.1979565 | up | mRNA |
| NM_021219 | JAM2 | 0.306606435 | 0.801653799 | 1.6105823 | up | mRNA |
| NM_021221 | LY6G5B | 0.859543474 | 0.988590875 | 1.017828 | up | mRNA |
| NM_021224 | ZNF462 | 0.069629101 | 0.457632202 | 1.2038675 | up | mRNA |
| NM_021231 | CACTIN | 0.864452517 | 0.989401142 | 1.0311307 | up | mRNA |
| NM_021233 | DNASE2B | 0.687143683 | 0.960205549 | 1.0477146 | up | mRNA |
| NM_021235 | EPS15L1 | 0.209383099 | 0.700367256 | 1.1000752 | up | mRNA |
| NM_021237 | SELK | 0.088359679 | 0.502587299 | 1.1066462 | up | mRNA |
| NM_021253 | TRIM39 | 0.58700751 | 0.93579682 | 1.083817 | up | mRNA |
| NM_021257 | NGB | 0.2955776 | 0.791662837 | 1.2148473 | up | mRNA |
| NM_021259 | TMEM8A | 0.646709396 | 0.95550188 | 1.0338967 | up | mRNA |
| NM_021569 | GRIN1 | 0.84088702 | 0.987068842 | 1.0510324 | up | mRNA |
| NM_021603 | FXYD2 | 0.504943814 | 0.910568383 | 1.0468194 | up | mRNA |
| NM_021619 | PRDM12 | 0.648650365 | 0.95550188 | 1.1600506 | up | mRNA |
| NM_021623 | PLEKHA2 | 0.544104294 | 0.923577434 | 1.0733268 | up | mRNA |
| NM_021625 | TRPV4 | 0.060282052 | 0.435378361 | 1.08418 | up | mRNA |
| NM_021632 | ZNF350 | 0.252119846 | 0.752621656 | 1.0790244 | up | mRNA |
| NM_021636 | LGR6 | 0.19939174 | 0.688290806 | 1.1071911 | up | mRNA |
| NM_021644 | HNRNPH3 | 0.685366236 | 0.960111041 | 1.0376323 | up | mRNA |
| NM_021647 | MFAP3L | 0.9774644 | 0.998501532 | 1.0028455 | up | mRNA |
| NM_021649 | TICAM2 | 0.261137604 | 0.761976144 | 1.2114283 | up | mRNA |
| NM_021706 | LAIR1 | 0.733811859 | 0.967777568 | 1.0687163 | up | mRNA |
| NM_021723 | ADAM22 | 0.938705348 | 0.996710991 | 1.0157185 | up | mRNA |
| NM_021728 | OTX2 | 0.4768407 | 0.900429473 | 1.1409029 | up | mRNA |
| NM_021729 | VPS11 | 0.64794379 | 0.95550188 | 1.1205636 | up | mRNA |
| NM_021738 | SVIL | 0.15449823 | 0.621861274 | 1.7971074 | up | mRNA |
| NM_021783 | EDA2R | 0.520837632 | 0.916605148 | 1.0892044 | up | mRNA |
| NM_021795 | ELK4 | 0.442192386 | 0.88419144 | 1.1365657 | up | mRNA |
| NM_021796 | PLAC1 | 0.632787883 | 0.951123179 | 1.0459918 | up | mRNA |
| NM_021800 | DNAJC12 | 0.207612703 | 0.698556218 | 1.0957078 | up | mRNA |
| NM_021806 | FAM3A | 0.01585012 | 0.253153683 | 1.1537679 | up | mRNA |
| NM_021808 | GALNT9 | 0.71901767 | 0.966191059 | 1.0485001 | up | mRNA |
| NM_021809 | TGIF2 | 0.342202889 | 0.830602529 | 1.0778007 | up | mRNA |
| NM_021818 | SAV1 | 0.002728785 | 0.123567151 | 1.4732616 | up | mRNA |
| NM_021825 | CCDC90B | 0.991374453 | 0.999485564 | 1.0012147 | up | mRNA |
| NM_021826 | FASTKD5 | 0.580726589 | 0.93393768 | 1.0755397 | up | mRNA |
| NM_021833 | UCP1 | 0.38732917 | 0.856773191 | 1.2988112 | up | mRNA |
| NM_021873 | CDC25B | 0.992456033 | 0.999781402 | 1.0015195 | up | mRNA |
| NM_021904 | GABBR1 | 0.156864076 | 0.625751326 | 1.0388996 | up | mRNA |
| NM_021911 | GABRB2 | 0.823022333 | 0.983495984 | 1.0404938 | up | mRNA |
| NM_021913 | AXL | 0.152588939 | 0.618255775 | 1.0595429 | up | mRNA |
| NM_021916 | ZNF70 | 0.258410785 | 0.759296063 | 1.1069792 | up | mRNA |
| NM_021920 | SCT | 0.064433008 | 0.443678541 | 1.5902502 | up | mRNA |
| NM_021926 | ALX4 | 0.914203894 | 0.995342392 | 1.0151737 | up | mRNA |
| NM_021932 | RIC8A | 0.642018086 | 0.954332328 | 1.0398808 | up | mRNA |
| NM_021937 | EEFSEC | 0.785772149 | 0.977479172 | 1.0286458 | up | mRNA |
| NM_021938 | CELF5 | 0.209417797 | 0.700367256 | 1.2035336 | up | mRNA |
| NM_021945 | SLC22A23 | 0.248871299 | 0.747319103 | 1.1319482 | up | mRNA |
| NM_021953 | FOXM1 | 0.773779552 | 0.97589756 | 1.0742677 | up | mRNA |
| NM_021959 | PPP1R11 | 0.339017771 | 0.827579459 | 1.0397259 | up | mRNA |
| NM_021967 | SERF1A | 0.33323395 | 0.82335721 | 1.1669028 | up | mRNA |
| NM_021968 | HIST1H4J | 0.391935941 | 0.859445464 | 1.0658358 | up | mRNA |
| NM_021971 | GMPPB | 0.989920894 | 0.999140676 | 1.0032258 | up | mRNA |
| NM_021973 | HAND2 | 0.991811089 | 0.999548897 | 1.0017649 | up | mRNA |
| NM_021975 | RELA | 0.048103438 | 0.398338064 | 1.1680251 | up | mRNA |
| NM_021979 | HSPA2 | 0.871295297 | 0.98985097 | 1.0322631 | up | mRNA |
| NM_021982 | SEC24A | 0.068189102 | 0.45284301 | 1.1351058 | up | mRNA |
| NM_021991 | JUP | 0.132345031 | 0.587843174 | 1.0940395 | up | mRNA |
| NM_021999 | ITM2B | 0.924492779 | 0.995456159 | 1.0198441 | up | mRNA |
| NM_022036 | GPRC5C | 0.920368305 | 0.995349793 | 1.0087337 | up | mRNA |
| NM_022039 | FBXW4 | 0.353310904 | 0.836312754 | 1.0871922 | up | mRNA |
| NM_022052 | NXF3 | 0.314433092 | 0.809707041 | 1.1186382 | up | mRNA |
| NM_022059 | CXCL16 | 0.99427215 | 0.999872237 | 1.0027099 | up | mRNA |
| NM_022068 | PIEZO2 | 0.618464537 | 0.946956498 | 1.0797819 | up | mRNA |
| NM_022073 | EGLN3 | 0.016258371 | 0.255922225 | 1.5137493 | up | mRNA |
| NM_022074 | FAM111A | 0.917815826 | 0.995349793 | 1.0274172 | up | mRNA |
| NM_022078 | GPATCH3 | 0.908659293 | 0.995333785 | 1.018452 | up | mRNA |
| NM_022080 | NAPB | 0.838667389 | 0.986964481 | 1.0565063 | up | mRNA |
| NM_022082 | SLC17A9 | 0.429207516 | 0.877844001 | 1.306545 | up | mRNA |
| NM_022091 | ASCC3 | 0.004585575 | 0.155029743 | 1.2266864 | up | mRNA |
| NM_022095 | ZNF335 | 0.801439164 | 0.98118927 | 1.0203739 | up | mRNA |
| NM_022100 | MRPS14 | 0.640396643 | 0.953894897 | 1.0373799 | up | mRNA |
| NM_022112 | TP53AIP1 | 0.02131651 | 0.288424269 | 1.4206344 | up | mRNA |
| NM_022121 | PERP | 0.481320009 | 0.901538317 | 1.092464 | up | mRNA |
| NM_022128 | RBKS | 0.54108966 | 0.923577434 | 1.038052 | up | mRNA |
| NM_022132 | MCCC2 | 0.819808173 | 0.982397582 | 1.0202635 | up | mRNA |
| NM_022135 | POPDC2 | 0.026749595 | 0.318484367 | 2.4163164 | up | mRNA |
| NM_022140 | EPB41L4A | 0.594827303 | 0.938655001 | 1.3039829 | up | mRNA |
| NM_022145 | CENPK | 0.755229479 | 0.971417904 | 1.0696962 | up | mRNA |
| NM_022146 | NPFFR1 | 0.543382437 | 0.923577434 | 1.0555391 | up | mRNA |
| NM_022150 | NPVF | 0.274672344 | 0.774767693 | 1.1123055 | up | mRNA |
| NM_022152 | TMBIM1 | 0.894799643 | 0.993978977 | 1.0153932 | up | mRNA |
| NM_022153 | C10orf54 | 0.777201482 | 0.97688307 | 1.02804 | up | mRNA |
| NM_022157 | RRAGC | 0.975777066 | 0.998501532 | 1.0030846 | up | mRNA |
| NM_022159 | ELTD1 | 0.905208473 | 0.99508468 | 1.0452896 | up | mRNA |
| NM_022166 | XYLT1 | 0.708234255 | 0.965462762 | 1.0442595 | up | mRNA |
| NM_022168 | IFIH1 | 0.001051908 | 0.086588663 | 2.072798 | up | mRNA |
| NM_022170 | EIF4H | 0.333057363 | 0.82335721 | 1.0872442 | up | mRNA |
| NM_022171 | TCTA | 0.652598839 | 0.95670626 | 1.0573557 | up | mRNA |
| NM_022172 | PC | 0.343641501 | 0.831536584 | 1.3089745 | up | mRNA |
| NM_022307 | ICA1 | 0.41218868 | 0.869144722 | 1.2497251 | up | mRNA |
| NM_022337 | RAB38 | 0.514955643 | 0.914177782 | 1.1339258 | up | mRNA |
| NM_022343 | GLIPR2 | 0.129838559 | 0.583907255 | 1.23544 | up | mRNA |
| NM_022344 | C17orf75 | 0.520724397 | 0.916605148 | 1.0870389 | up | mRNA |
| NM_022347 | TOR1AIP2 | 0.095421005 | 0.516597924 | 1.0729997 | up | mRNA |
| NM_022349 | MS4A6A | 0.127636506 | 0.580991132 | 1.1713432 | up | mRNA |
| NM_022351 | NECAB1 | 0.125795417 | 0.578441772 | 1.1013033 | up | mRNA |
| NM_022355 | DPEP2 | 0.813158778 | 0.98120545 | 1.0505503 | up | mRNA |
| NM_022358 | KCNK15 | 0.746903657 | 0.970432947 | 1.0631217 | up | mRNA |
| NM_022361 | POPDC3 | 0.936318108 | 0.996710991 | 1.0665246 | up | mRNA |
| NM_022362 | MMS19 | 0.514531782 | 0.913824266 | 1.1764678 | up | mRNA |
| NM_022365 | DNAJC1 | 0.395622938 | 0.861590143 | 1.1202264 | up | mRNA |
| NM_022369 | STRA6 | 0.498506245 | 0.909361611 | 1.0361482 | up | mRNA |
| NM_022372 | MLST8 | 0.184944592 | 0.668140272 | 1.0324241 | up | mRNA |
| NM_022377 | ICAM4 | 6.26642E-05 | 0.031075339 | 5.3754099 | up | mRNA |
| NM_022405 | SLC6A20 | 0.354254774 | 0.837147328 | 1.0763299 | up | mRNA |
| NM_022438 | MAL | 0.090174008 | 0.507386748 | 1.2246878 | up | mRNA |
| NM_022439 | MAL | 0.871303541 | 0.98985097 | 1.0152203 | up | mRNA |
| NM_022440 | MAL | 0.39003345 | 0.858893111 | 1.1338221 | up | mRNA |
| NM_022449 | RAB17 | 0.905042847 | 0.99508468 | 1.0288922 | up | mRNA |
| NM_022451 | NOC3L | 0.10512099 | 0.536745347 | 1.1395589 | up | mRNA |
| NM_022459 | XPO4 | 0.350457754 | 0.8348573 | 1.0957597 | up | mRNA |
| NM_022460 | HS1BP3 | 0.098051843 | 0.520720649 | 1.1360673 | up | mRNA |
| NM_022462 | HIF3A | 0.601479997 | 0.941048131 | 1.126953 | up | mRNA |
| NM_022463 | NXN | 0.556362389 | 0.926833616 | 1.0490796 | up | mRNA |
| NM_022464 | SIL1 | 0.347182158 | 0.833588015 | 1.1044536 | up | mRNA |
| NM_022477 | NDRG3 | 0.597019661 | 0.939714862 | 1.0441866 | up | mRNA |
| NM_022480 | KLHL25 | 0.010814198 | 0.213674567 | 1.2557689 | up | mRNA |
| NM_022481 | ARAP3 | 0.248580655 | 0.7470442 | 1.0827821 | up | mRNA |
| NM_022489 | INF2 | 0.507831499 | 0.911674633 | 1.0398816 | up | mRNA |
| NM_022497 | MRPS25 | 0.532058751 | 0.920228311 | 1.0552289 | up | mRNA |
| NM_022549 | FEZ1 | 0.67802452 | 0.959531882 | 1.0555847 | up | mRNA |
| NM_022550 | XRCC4 | 0.823584846 | 0.983498765 | 1.0288331 | up | mRNA |
| NM_022551 | RPS18 | 0.704052883 | 0.963625105 | 1.0641685 | up | mRNA |
| NM_022553 | VPS52 | 0.618877996 | 0.947160514 | 1.1169423 | up | mRNA |
| NM_022560 | GH1 | 0.228893174 | 0.725125586 | 1.1541614 | up | mRNA |
| NM_022566 | MESDC1 | 0.004457801 | 0.153101361 | 1.944448 | up | mRNA |
| NM_022570 | CLEC7A | 0.614834032 | 0.944954685 | 1.1077439 | up | mRNA |
| NM_022574 | GIGYF1 | 0.499208616 | 0.909361611 | 1.1059233 | up | mRNA |
| NM_022575 | VPS16 | 0.463226554 | 0.894989844 | 1.0605562 | up | mRNA |
| NM_022579 | CSHL1 | 0.037411018 | 0.361408713 | 1.4402048 | up | mRNA |
| NM_022644 | CSH2 | 0.904825351 | 0.99508468 | 1.0192537 | up | mRNA |
| NM_022652 | DUSP6 | 0.092180762 | 0.511207518 | 2.1259035 | up | mRNA |
| NM_022659 | EBF2 | 0.965027283 | 0.99793927 | 1.0545928 | up | mRNA |
| NM_022661 | SPANXC | 0.419898754 | 0.872365335 | 1.115208 | up | mRNA |
| NM_022716 | PRRX1 | 0.001288564 | 0.093126586 | 1.2297107 | up | mRNA |
| NM_022720 | DGCR8 | 0.781655743 | 0.976938981 | 1.0276274 | up | mRNA |
| NM_022733 | SMAP2 | 0.686374485 | 0.960205549 | 1.045382 | up | mRNA |
| NM_022739 | SMURF2 | 0.425000336 | 0.874871604 | 1.0560804 | up | mRNA |
| NM_022740 | HIPK2 | 0.003093607 | 0.131960624 | 1.2544487 | up | mRNA |
| NM_022742 | CCDC136 | 0.997852415 | 0.999889342 | 1.001676 | up | mRNA |
| NM_022744 | C16orf58 | 0.537058671 | 0.922702335 | 1.0814782 | up | mRNA |
| NM_022745 | ATPAF1 | 0.972197687 | 0.998501532 | 1.0029551 | up | mRNA |
| NM_022749 | FAM160B2 | 0.204953948 | 0.694868044 | 1.0405664 | up | mRNA |
| NM_022750 | PARP12 | 0.293751615 | 0.791146775 | 1.145262 | up | mRNA |
| NM_022757 | CCDC14 | 0.140483808 | 0.600490836 | 1.5538693 | up | mRNA |
| NM_022761 | C11orf1 | 0.835088977 | 0.986575022 | 1.0130601 | up | mRNA |
| NM_022769 | CRTC3 | 0.588379095 | 0.93579682 | 1.0761283 | up | mRNA |
| NM_022772 | EPS8L2 | 0.412480818 | 0.869219033 | 1.0693492 | up | mRNA |
| NM_022784 | IQCH | 0.676629767 | 0.959531882 | 1.1261646 | up | mRNA |
| NM_022817 | PER2 | 0.607895293 | 0.942753028 | 1.1270352 | up | mRNA |
| NM_022826 | MARCH7 | 0.183020838 | 0.666166668 | 1.1128345 | up | mRNA |
| NM_022828 | YTHDC2 | 0.802135643 | 0.98118927 | 1.0206251 | up | mRNA |
| NM_022839 | MRPS11 | 0.902978698 | 0.99508468 | 1.01578 | up | mRNA |
| NM_022840 | METTL4 | 0.000439715 | 0.06478466 | 1.0963631 | up | mRNA |
| NM_022843 | PCDH20 | 0.25474036 | 0.755011807 | 1.152174 | up | mRNA |
| NM_022872 | IFI6 | 0.340466359 | 0.828628442 | 1.094411 | up | mRNA |
| NM_022873 | IFI6 | 0.437825981 | 0.882682266 | 1.1346758 | up | mRNA |
| NM_022893 | BCL11A | 0.845958718 | 0.987338076 | 1.0275077 | up | mRNA |
| NM_022908 | NT5DC2 | 0.864855557 | 0.989401142 | 1.0183805 | up | mRNA |
| NM_022910 | NDRG4 | 0.810113003 | 0.98118927 | 1.0389866 | up | mRNA |
| NM_022911 | SLC26A6 | 0.734525507 | 0.967777568 | 1.0179093 | up | mRNA |
| NM_022912 | REEP1 | 0.128719877 | 0.58263855 | 1.269755 | up | mRNA |
| NM_022917 | NOL6 | 0.290126892 | 0.786468159 | 1.0885305 | up | mRNA |
| NM_022977 | ACSL4 | 0.004699193 | 0.15714472 | 2.144297 | up | mRNA |
| NM_022978 | SERF1B | 0.792992525 | 0.979003983 | 1.0214623 | up | mRNA |
| NM_023004 | RTN4R | 0.024125855 | 0.303741948 | 1.1619964 | up | mRNA |
| NM_023009 | MARCKSL1 | 0.084286485 | 0.494150176 | 1.4356547 | up | mRNA |
| NM_023015 | INTS3 | 0.125891885 | 0.578441772 | 1.0916468 | up | mRNA |
| NM_023033 | METTL1 | 0.619517488 | 0.947444797 | 1.0265508 | up | mRNA |
| NM_023037 | FRY | 0.124367094 | 0.578369571 | 1.5812973 | up | mRNA |
| NM_023039 | ANKRA2 | 0.851841297 | 0.987678166 | 1.0373043 | up | mRNA |
| NM_023085 | CAPN10 | 0.561500204 | 0.928513767 | 1.0439766 | up | mRNA |
| NM_023923 | PHACTR4 | 0.102756295 | 0.531998421 | 1.2282374 | up | mRNA |
| NM_023927 | GRAMD3 | 0.089625376 | 0.506107454 | 1.1423639 | up | mRNA |
| NM_023929 | ZBTB10 | 0.731971113 | 0.96727133 | 1.0946353 | up | mRNA |
| NM_023931 | ZNF747 | 0.723043729 | 0.966191059 | 1.0315224 | up | mRNA |
| NM_023932 | DLK2 | 0.413563747 | 0.86961884 | 1.045927 | up | mRNA |
| NM_023934 | FUNDC2 | 0.865041092 | 0.989401142 | 1.0276695 | up | mRNA |
| NM_023935 | DDRGK1 | 0.699783063 | 0.962977276 | 1.0339808 | up | mRNA |
| NM_023942 | LRRC61 | 0.46833968 | 0.897228151 | 1.0715061 | up | mRNA |
| NM_023946 | LYNX1 | 0.263834038 | 0.764495522 | 1.1367375 | up | mRNA |
| NM_024006 | VKORC1 | 0.385260961 | 0.85515381 | 1.0951176 | up | mRNA |
| NM_024007 | EBF1 | 0.719337412 | 0.966191059 | 1.0827621 | up | mRNA |
| NM_024014 | HOXA6 | 0.307163946 | 0.802113712 | 1.083355 | up | mRNA |
| NM_024015 | HOXB4 | 0.205317193 | 0.695403826 | 1.2914642 | up | mRNA |
| NM_024017 | HOXB9 | 0.774796014 | 0.97589756 | 1.067157 | up | mRNA |
| NM_024021 | MS4A4A | 0.29915747 | 0.79551003 | 1.1713148 | up | mRNA |
| NM_024025 | DUSP26 | 0.096232171 | 0.51782059 | 1.126226 | up | mRNA |
| NM_024031 | PRR14 | 0.033437518 | 0.347572397 | 1.0967311 | up | mRNA |
| NM_024040 | CUEDC2 | 0.594849634 | 0.938655001 | 1.0192361 | up | mRNA |
| NM_024042 | METRN | 0.542039478 | 0.923577434 | 1.084302 | up | mRNA |
| NM_024046 | CAMKV | 0.724450726 | 0.966191059 | 1.0481066 | up | mRNA |
| NM_024050 | DDA1 | 0.451904739 | 0.888854908 | 1.0953906 | up | mRNA |
| NM_024054 | C7orf25 | 0.237070881 | 0.735183924 | 1.1971342 | up | mRNA |
| NM_024059 | C20orf195 | 0.004689632 | 0.15714472 | 1.4275751 | up | mRNA |
| NM_024060 | AHNAK | 0.24717097 | 0.745152522 | 1.0651023 | up | mRNA |
| NM_024063 | SPATA5L1 | 0.78623245 | 0.977479172 | 1.0298457 | up | mRNA |
| NM_024070 | PVRIG | 0.366857258 | 0.845181673 | 1.0498632 | up | mRNA |
| NM_024081 | PRRG4 | 0.047254308 | 0.396850304 | 1.5269982 | up | mRNA |
| NM_024085 | ATG9A | 0.748939779 | 0.970725371 | 1.0357195 | up | mRNA |
| NM_024087 | ASB9 | 0.659700968 | 0.958354006 | 1.2831126 | up | mRNA |
| NM_024122 | APOO | 0.440914477 | 0.883920737 | 1.0436112 | up | mRNA |
| NM_024293 | FAM134A | 0.047646801 | 0.397835916 | 1.1242005 | up | mRNA |
| NM_024296 | CCDC28B | 0.580748283 | 0.93393768 | 1.0506174 | up | mRNA |
| NM_024300 | CHCHD7 | 0.708970644 | 0.965462762 | 1.1122813 | up | mRNA |
| NM_024306 | FA2H | 0.968783957 | 0.998196842 | 1.0019976 | up | mRNA |
| NM_024308 | DHRS11 | 0.955222383 | 0.996883425 | 1.004361 | up | mRNA |
| NM_024311 | MFSD11 | 0.520636198 | 0.916605148 | 1.1822031 | up | mRNA |
| NM_024312 | GNPTAB | 0.75871997 | 0.972154041 | 1.049363 | up | mRNA |
| NM_024316 | LENG1 | 0.722408612 | 0.966191059 | 1.0693463 | up | mRNA |
| NM_024318 | LILRA6 | 0.374324927 | 0.849321997 | 1.0792054 | up | mRNA |
| NM_024321 | RBM42 | 0.869298098 | 0.989592579 | 1.0212403 | up | mRNA |
| NM_024328 | THTPA | 0.192466861 | 0.678822446 | 1.1434702 | up | mRNA |
| NM_024330 | SLC27A3 | 0.31788837 | 0.811908136 | 1.2868873 | up | mRNA |
| NM_024331 | TTPAL | 0.781762035 | 0.976938981 | 1.0766533 | up | mRNA |
| NM_024407 | NDUFS7 | 0.786498851 | 0.977479172 | 1.0142389 | up | mRNA |
| NM_024408 | NOTCH2 | 0.017886527 | 0.268985846 | 1.5064586 | up | mRNA |
| NM_024420 | PLA2G4A | 0.611396505 | 0.943894921 | 1.064314 | up | mRNA |
| NM_024426 | WT1 | 0.265689822 | 0.766537754 | 1.1209493 | up | mRNA |
| NM_024429 | PRKAG2 | 0.286836215 | 0.782942227 | 1.1391802 | up | mRNA |
| NM_024430 | PSTPIP2 | 0.446796342 | 0.886875255 | 1.1161135 | up | mRNA |
| NM_024482 | GMEB1 | 0.419449813 | 0.872365335 | 1.1064694 | up | mRNA |
| NM_024494 | WNT2B | 0.020093327 | 0.284065167 | 1.1574581 | up | mRNA |
| NM_024503 | HIVEP3 | 0.014925784 | 0.245074119 | 2.7247907 | up | mRNA |
| NM_024514 | CYP2R1 | 0.61242087 | 0.944159427 | 1.0403983 | up | mRNA |
| NM_024519 | FAM65A | 0.277865956 | 0.777287651 | 1.1085186 | up | mRNA |
| NM_024522 | NKAIN1 | 0.874278298 | 0.990793509 | 1.0177065 | up | mRNA |
| NM_024524 | ATP13A3 | 0.275107166 | 0.774827385 | 1.4312615 | up | mRNA |
| NM_024526 | EPS8L3 | 0.88629044 | 0.992981836 | 1.0106936 | up | mRNA |
| NM_024535 | CORO7 | 0.840257149 | 0.987068842 | 1.0199698 | up | mRNA |
| NM_024546 | RNF219 | 0.253546222 | 0.753559682 | 1.1131555 | up | mRNA |
| NM_024551 | ADIPOR2 | 0.833589945 | 0.986434891 | 1.0189006 | up | mRNA |
| NM_024560 | ACSS3 | 0.413835614 | 0.86961884 | 1.1476258 | up | mRNA |
| NM_024562 | TMCO7 | 0.661214221 | 0.958354006 | 1.022635 | up | mRNA |
| NM_024569 | MPZL1 | 0.001135517 | 0.087784542 | 1.3050205 | up | mRNA |
| NM_024572 | GALNT14 | 0.898953001 | 0.99453218 | 1.0174096 | up | mRNA |
| NM_024576 | OGFRL1 | 0.446791754 | 0.886875255 | 1.2490718 | up | mRNA |
| NM_024578 | OCEL1 | 0.50853494 | 0.911674633 | 1.0325895 | up | mRNA |
| NM_024581 | FAM184A | 0.533404789 | 0.920530493 | 1.0642418 | up | mRNA |
| NM_024589 | ROGDI | 0.41558763 | 0.870382888 | 1.1730917 | up | mRNA |
| NM_024594 | PANK3 | 0.873413183 | 0.990580595 | 1.0106195 | up | mRNA |
| NM_024595 | AKIRIN1 | 0.317478107 | 0.811824899 | 1.1153911 | up | mRNA |
| NM_024616 | C3orf52 | 0.176131211 | 0.6551228 | 1.8726674 | up | mRNA |
| NM_024619 | FN3KRP | 0.820773177 | 0.982756843 | 1.0608351 | up | mRNA |
| NM_024621 | VEPH1 | 0.465796797 | 0.895570502 | 1.0250983 | up | mRNA |
| NM_024623 | OGFOD2 | 0.947659199 | 0.996710991 | 1.0068011 | up | mRNA |
| NM_024628 | SLC12A8 | 0.304424789 | 0.800038203 | 1.2094238 | up | mRNA |
| NM_024637 | GAL3ST4 | 0.209382163 | 0.700367256 | 1.2538911 | up | mRNA |
| NM_024640 | YRDC | 0.665603288 | 0.958354006 | 1.0330159 | up | mRNA |
| NM_024649 | BBS1 | 0.004893878 | 0.158753988 | 1.179134 | up | mRNA |
| NM_024652 | LRRK1 | 0.351521671 | 0.835247013 | 1.09901 | up | mRNA |
| NM_024653 | PRKRIP1 | 0.78705538 | 0.977479172 | 1.0240594 | up | mRNA |
| NM_024660 | IGFLR1 | 0.597841995 | 0.940150767 | 1.1597788 | up | mRNA |
| NM_024662 | NAT10 | 0.436656831 | 0.882102748 | 1.1581701 | up | mRNA |
| NM_024665 | TBL1XR1 | 0.936454062 | 0.996710991 | 1.0092817 | up | mRNA |
| NM_024667 | VPS37B | 0.932990176 | 0.996710991 | 1.013118 | up | mRNA |
| NM_024668 | ANKHD1 | 0.549790432 | 0.925533435 | 1.051247 | up | mRNA |
| NM_024671 | ZNF768 | 0.048061977 | 0.398338064 | 1.4441721 | up | mRNA |
| NM_024680 | E2F8 | 0.904051113 | 0.99508468 | 1.0074256 | up | mRNA |
| NM_024682 | TBC1D17 | 0.021288502 | 0.288424269 | 1.3049333 | up | mRNA |
| NM_024684 | C11orf67 | 0.757038135 | 0.971960783 | 1.0338919 | up | mRNA |
| NM_024686 | TTLL7 | 0.360800397 | 0.841780454 | 1.1210291 | up | mRNA |
| NM_024700 | SNIP1 | 0.107168857 | 0.542569024 | 1.0574494 | up | mRNA |
| NM_024701 | ASB13 | 0.478280829 | 0.901271944 | 1.0828926 | up | mRNA |
| NM_024715 | TXNDC15 | 0.125819985 | 0.578441772 | 1.2183055 | up | mRNA |
| NM_024717 | MCTP1 | 0.015891353 | 0.253613015 | 1.1082061 | up | mRNA |
| NM_024719 | GRTP1 | 0.900580558 | 0.994789506 | 1.0282154 | up | mRNA |
| NM_024722 | ACBD4 | 0.581605089 | 0.934005566 | 1.1849165 | up | mRNA |
| NM_024727 | LRRC31 | 0.292255074 | 0.788954556 | 1.0950717 | up | mRNA |
| NM_024735 | FBXO31 | 0.363632392 | 0.843238468 | 1.086506 | up | mRNA |
| NM_024742 | ARMC5 | 0.337115508 | 0.826919371 | 1.1058175 | up | mRNA |
| NM_024747 | HPS6 | 0.495112126 | 0.907415174 | 1.0979037 | up | mRNA |
| NM_024749 | VASH2 | 0.039163468 | 0.369499601 | 1.1186516 | up | mRNA |
| NM_024761 | MOB3B | 0.086849553 | 0.499950487 | 1.0906261 | up | mRNA |
| NM_024767 | DLC1 | 0.030174595 | 0.337093329 | 1.7185263 | up | mRNA |
| NM_024770 | METTL8 | 0.011860942 | 0.221526283 | 1.4640821 | up | mRNA |
| NM_024776 | PEAK1 | 0.74310558 | 0.970330526 | 1.0239672 | up | mRNA |
| NM_024780 | TMC5 | 0.223098511 | 0.71761506 | 1.1565497 | up | mRNA |
| NM_024791 | PDZD3 | 0.096913214 | 0.519207575 | 1.1275354 | up | mRNA |
| NM_024804 | ZNF669 | 0.723457513 | 0.966191059 | 1.0371576 | up | mRNA |
| NM_024809 | TCTN2 | 0.600143336 | 0.940648177 | 1.235682 | up | mRNA |
| NM_024812 | BAALC | 0.778176001 | 0.976938981 | 1.0389613 | up | mRNA |
| NM_024815 | NUDT18 | 0.730932193 | 0.966589486 | 1.0669357 | up | mRNA |
| NM_024820 | DENND1A | 0.342113753 | 0.830550068 | 1.140358 | up | mRNA |
| NM_024821 | CCDC134 | 0.939607641 | 0.996710991 | 1.0238942 | up | mRNA |
| NM_024828 | CAAP1 | 0.528333346 | 0.918883792 | 1.0539973 | up | mRNA |
| NM_024829 | PLBD1 | 0.806409599 | 0.98118927 | 1.1055806 | up | mRNA |
| NM_024831 | TGS1 | 0.841404585 | 0.987068842 | 1.008353 | up | mRNA |
| NM_024844 | NUP85 | 0.924518471 | 0.995456159 | 1.0252539 | up | mRNA |
| NM_024857 | ATAD5 | 0.865821097 | 0.989426402 | 1.0310963 | up | mRNA |
| NM_024859 | MAGIX | 0.049135805 | 0.402023817 | 1.0985318 | up | mRNA |
| NM_024864 | MRM1 | 0.823125209 | 0.983498765 | 1.0281073 | up | mRNA |
| NM_024865 | NANOG | 0.599603455 | 0.940648177 | 1.1616056 | up | mRNA |
| NM_024867 | SPEF2 | 0.697824493 | 0.962410025 | 1.1453305 | up | mRNA |
| NM_024875 | SYNPO2L | 0.631755553 | 0.950595536 | 1.0646938 | up | mRNA |
| NM_024896 | ERMP1 | 0.684227551 | 0.960111041 | 1.2253761 | up | mRNA |
| NM_024901 | DENND2D | 0.434722566 | 0.881074297 | 1.1701575 | up | mRNA |
| NM_024909 | ATAT1 | 0.675730768 | 0.959531882 | 1.0518019 | up | mRNA |
| NM_024913 | CPED1 | 0.472798677 | 0.899184677 | 1.069694 | up | mRNA |
| NM_024915 | GRHL2 | 0.84805806 | 0.987678166 | 1.0778241 | up | mRNA |
| NM_024919 | FRMD1 | 0.740936667 | 0.970165142 | 1.0392439 | up | mRNA |
| NM_024922 | CES3 | 0.239812972 | 0.737986583 | 1.1224265 | up | mRNA |
| NM_024927 | PLEKHH3 | 0.011489031 | 0.218926869 | 1.2413863 | up | mRNA |
| NM_024936 | ZCCHC4 | 0.989693482 | 0.999140676 | 1.0021169 | up | mRNA |
| NM_024943 | TMEM156 | 0.002206446 | 0.111580485 | 2.1601448 | up | mRNA |
| NM_024955 | FOXRED2 | 0.707885722 | 0.965436846 | 1.038895 | up | mRNA |
| NM_024958 | NRSN2 | 0.780817461 | 0.976938981 | 1.029114 | up | mRNA |
| NM_024959 | SLC24A6 | 0.393023425 | 0.859795559 | 1.1370191 | up | mRNA |
| NM_024966 | SEMA6D | 0.49805286 | 0.909116151 | 1.1258375 | up | mRNA |
| NM_024996 | GFM1 | 0.199746793 | 0.68900633 | 1.2999032 | up | mRNA |
| NM_025008 | ADAMTSL4 | 0.366363008 | 0.845181673 | 1.0630165 | up | mRNA |
| NM_025009 | CEP135 | 0.168721262 | 0.644327097 | 1.1348957 | up | mRNA |
| NM_025045 | BAIAP2L2 | 0.98760791 | 0.999140676 | 1.0027445 | up | mRNA |
| NM_025048 | GPR110 | 0.06722793 | 0.45156203 | 1.2288506 | up | mRNA |
| NM_025052 | YSK4 | 0.116470981 | 0.562996237 | 1.2110512 | up | mRNA |
| NM_025069 | ZNF703 | 0.191375523 | 0.676939305 | 1.0637291 | up | mRNA |
| NM_025073 | SIKE1 | 0.6892956 | 0.96108227 | 1.025303 | up | mRNA |
| NM_025076 | UXS1 | 0.000771391 | 0.081471496 | 1.6211708 | up | mRNA |
| NM_025078 | PQLC1 | 0.75043804 | 0.970725371 | 1.0111049 | up | mRNA |
| NM_025079 | ZC3H12A | 2.07771E-06 | 0.008848943 | 26.0019647 | up | mRNA |
| NM_025099 | CTC1 | 0.526733463 | 0.917975454 | 1.0688702 | up | mRNA |
| NM_025106 | SPSB1 | 0.007544938 | 0.193203621 | 3.623436 | up | mRNA |
| NM_025124 | TMEM134 | 0.321563821 | 0.815271211 | 1.0572876 | up | mRNA |
| NM_025140 | CCDC92 | 0.826363903 | 0.984423205 | 1.0598481 | up | mRNA |
| NM_025151 | RAB11FIP1 | 0.749823421 | 0.970725371 | 1.0684986 | up | mRNA |
| NM_025152 | NUBPL | 0.490366273 | 0.905264563 | 1.1638235 | up | mRNA |
| NM_025157 | PXN | 0.877223778 | 0.991258481 | 1.0090428 | up | mRNA |
| NM_025164 | SIK3 | 0.385426555 | 0.85515381 | 1.0499966 | up | mRNA |
| NM_025180 | CEP63 | 0.841027041 | 0.987068842 | 1.0165883 | up | mRNA |
| NM_025188 | TRIM45 | 0.639634055 | 0.953588474 | 1.2137587 | up | mRNA |
| NM_025194 | ITPKC | 0.152563358 | 0.618255775 | 1.2682475 | up | mRNA |
| NM_025195 | TRIB1 | 0.019947537 | 0.283521851 | 2.0031455 | up | mRNA |
| NM_025202 | EFHD1 | 0.859381392 | 0.988512246 | 1.0261458 | up | mRNA |
| NM_025208 | PDGFD | 0.861130958 | 0.989013989 | 1.1661653 | up | mRNA |
| NM_025218 | ULBP1 | 0.327852596 | 0.820730147 | 1.1326862 | up | mRNA |
| NM_025221 | KCNIP4 | 0.478938047 | 0.901271944 | 1.0198378 | up | mRNA |
| NM_025227 | BPIFB2 | 0.516360973 | 0.914676016 | 1.1036765 | up | mRNA |
| NM_025239 | PDCD1LG2 | 0.358817103 | 0.840767187 | 1.4251892 | up | mRNA |
| NM_025240 | CD276 | 0.234653045 | 0.732080054 | 1.0721732 | up | mRNA |
| NM_025241 | UBXN6 | 0.095360022 | 0.516597924 | 1.2548952 | up | mRNA |
| NM_025243 | SLC19A3 | 0.434989597 | 0.881331247 | 1.0523324 | up | mRNA |
| NM_025249 | KIAA1683 | 0.856593771 | 0.988448602 | 1.0302871 | up | mRNA |
| NM_025250 | TTYH3 | 0.140578727 | 0.600724394 | 1.1461726 | up | mRNA |
| NM_025261 | LY6G6C | 0.470338563 | 0.898180112 | 1.0767615 | up | mRNA |
| NM_030568 | KHDC1 | 0.418777895 | 0.872328878 | 1.0975302 | up | mRNA |
| NM_030569 | ITIH5 | 0.791870494 | 0.978996327 | 1.0308205 | up | mRNA |
| NM_030570 | UPK3B | 0.249116444 | 0.747319103 | 1.1446825 | up | mRNA |
| NM_030624 | KLHL15 | 0.530122426 | 0.91899174 | 1.1170723 | up | mRNA |
| NM_030627 | CPEB4 | 0.071633346 | 0.462218089 | 1.2892657 | up | mRNA |
| NM_030630 | C17orf28 | 0.547672854 | 0.92514258 | 1.1310741 | up | mRNA |
| NM_030631 | SLC25A21 | 0.412019195 | 0.869001481 | 1.6785105 | up | mRNA |
| NM_030632 | ASXL3 | 0.355656829 | 0.838401697 | 1.1614152 | up | mRNA |
| NM_030636 | EEPD1 | 0.589449599 | 0.936090876 | 1.1025419 | up | mRNA |
| NM_030639 | BHLHB9 | 0.424939663 | 0.874854499 | 1.1454311 | up | mRNA |
| NM_030640 | DUSP16 | 0.164715073 | 0.638119216 | 1.1928754 | up | mRNA |
| NM_030641 | APOL6 | 0.788479663 | 0.977641695 | 1.0667703 | up | mRNA |
| NM_030647 | JHDM1D | 0.459481732 | 0.892904132 | 1.2796596 | up | mRNA |
| NM_030650 | KIAA1715 | 0.544249115 | 0.923577434 | 1.0466052 | up | mRNA |
| NM_030651 | PRRT1 | 0.905941884 | 0.99508468 | 1.0178427 | up | mRNA |
| NM_030662 | MAP2K2 | 0.268826497 | 0.769503074 | 1.1669122 | up | mRNA |
| NM_030667 | PTPRO | 0.956094478 | 0.996883425 | 1.0143754 | up | mRNA |
| NM_030764 | FCRL2 | 0.932926921 | 0.996710991 | 1.0306569 | up | mRNA |
| NM_030765 | B3GNT4 | 0.277959581 | 0.777290204 | 1.1605691 | up | mRNA |
| NM_030766 | BCL2L14 | 0.084801237 | 0.495311334 | 1.16022 | up | mRNA |
| NM_030767 | AKNA | 0.723532377 | 0.966191059 | 1.0456336 | up | mRNA |
| NM_030768 | ILKAP | 0.638805906 | 0.953122601 | 1.0161115 | up | mRNA |
| NM_030769 | NPL | 0.238606809 | 0.736616102 | 1.3818948 | up | mRNA |
| NM_030772 | GJA9 | 0.022957647 | 0.298169644 | 1.286463 | up | mRNA |
| NM_030774 | OR51E2 | 0.133006099 | 0.588930122 | 1.5126353 | up | mRNA |
| NM_030779 | KCNH6 | 0.857205549 | 0.988512246 | 1.0407873 | up | mRNA |
| NM_030780 | SLC25A32 | 0.433478795 | 0.880871258 | 1.1169351 | up | mRNA |
| NM_030784 | GPR63 | 0.947436775 | 0.996710991 | 1.0169969 | up | mRNA |
| NM_030788 | DCSTAMP | 0.363533415 | 0.843238468 | 2.1507101 | up | mRNA |
| NM_030792 | GDPD5 | 0.26936805 | 0.769752802 | 1.052598 | up | mRNA |
| NM_030797 | FAM49A | 0.288516265 | 0.784514546 | 1.4140544 | up | mRNA |
| NM_030798 | WBSCR16 | 0.891266452 | 0.9937521 | 1.0096473 | up | mRNA |
| NM_030809 | CSRNP2 | 0.670857063 | 0.959531882 | 1.0413632 | up | mRNA |
| NM_030820 | COL21A1 | 0.476452695 | 0.900415348 | 1.2255762 | up | mRNA |
| NM_030824 | ZNF442 | 0.997354394 | 0.999889342 | 1.000713 | up | mRNA |
| NM_030885 | MAP4 | 0.761923962 | 0.97339948 | 1.0500014 | up | mRNA |
| NM_030891 | LRRC3 | 0.366395704 | 0.845181673 | 1.0951418 | up | mRNA |
| NM_030893 | CD1E | 0.998076163 | 0.999889342 | 1.0006617 | up | mRNA |
| NM_030901 | OR7A17 | 0.92755317 | 0.995829678 | 1.0190011 | up | mRNA |
| NM_030907 | RSG1 | 0.831230742 | 0.985710869 | 1.0367186 | up | mRNA |
| NM_030912 | TRIM8 | 0.732772263 | 0.967703318 | 1.0628284 | up | mRNA |
| NM_030916 | PVRL4 | 0.685039396 | 0.960111041 | 1.0488238 | up | mRNA |
| NM_030930 | UNC93B1 | 0.760286347 | 0.972883253 | 1.0287655 | up | mRNA |
| NM_030933 | SHCBP1L | 0.864631604 | 0.989401142 | 1.0496313 | up | mRNA |
| NM_030939 | C6orf62 | 0.483897607 | 0.903204457 | 1.0455391 | up | mRNA |
| NM_030945 | C1QTNF3 | 0.077022957 | 0.477122268 | 1.0876958 | up | mRNA |
| NM_030948 | PHACTR1 | 0.019381998 | 0.280114993 | 1.9379138 | up | mRNA |
| NM_030952 | NUAK2 | 0.0008213 | 0.083087642 | 8.2313411 | up | mRNA |
| NM_030961 | TRIM56 | 0.849726796 | 0.987678166 | 1.0325804 | up | mRNA |
| NM_030964 | SPRY4 | 0.390919737 | 0.858920947 | 1.1358136 | up | mRNA |
| NM_030981 | RAB1B | 0.941866686 | 0.996710991 | 1.0091328 | up | mRNA |
| NM_031212 | SLC25A28 | 0.036571032 | 0.359035355 | 1.6444166 | up | mRNA |
| NM_031215 | CABLES2 | 0.528960513 | 0.918932853 | 1.0513226 | up | mRNA |
| NM_031229 | RBCK1 | 0.639137001 | 0.953266836 | 1.0418685 | up | mRNA |
| NM_031243 | HNRNPA2B1 | 0.476584024 | 0.900429473 | 1.0790449 | up | mRNA |
| NM_031262 | HNRNPK | 0.509576994 | 0.911674633 | 1.1158388 | up | mRNA |
| NM_031263 | HNRNPK | 0.384591361 | 0.85485898 | 1.1959394 | up | mRNA |
| NM_031264 | CDHR5 | 0.837474701 | 0.986775289 | 1.0255229 | up | mRNA |
| NM_031283 | TCF7L1 | 0.304575011 | 0.800038203 | 1.1145885 | up | mRNA |
| NM_031290 | CCDC70 | 0.753682408 | 0.970725371 | 1.0209975 | up | mRNA |
| NM_031296 | RAB33B | 0.688281108 | 0.96055024 | 1.041917 | up | mRNA |
| NM_031297 | RNF208 | 0.142571216 | 0.602652383 | 1.0785694 | up | mRNA |
| NM_031301 | APH1B | 0.798539093 | 0.980749101 | 1.0612765 | up | mRNA |
| NM_031303 | KATNAL2 | 0.64910083 | 0.95550188 | 1.1209871 | up | mRNA |
| NM_031309 | SCRT1 | 0.070816644 | 0.460237837 | 1.1237051 | up | mRNA |
| NM_031313 | ALPPL2 | 0.844718161 | 0.987338076 | 1.0258795 | up | mRNA |
| NM_031314 | HNRNPC | 0.042855255 | 0.382411463 | 1.2206399 | up | mRNA |
| NM_031371 | ARID4B | 0.370513971 | 0.846215286 | 1.104939 | up | mRNA |
| NM_031407 | HUWE1 | 0.456858581 | 0.891568474 | 1.05054 | up | mRNA |
| NM_031412 | GABARAPL1 | 0.805335854 | 0.98118927 | 1.0722188 | up | mRNA |
| NM_031413 | CECR2 | 0.249314651 | 0.747319103 | 1.1520911 | up | mRNA |
| NM_031417 | MARK4 | 0.922014226 | 0.995349793 | 1.0242355 | up | mRNA |
| NM_031419 | NFKBIZ | 6.91148E-05 | 0.031937303 | 12.6255699 | up | mRNA |
| NM_031426 | AIF1L | 0.45918326 | 0.892904132 | 1.1469903 | up | mRNA |
| NM_031435 | THAP2 | 0.743190363 | 0.970330526 | 1.0397562 | up | mRNA |
| NM_031444 | C22orf13 | 0.754637791 | 0.971217595 | 1.0178662 | up | mRNA |
| NM_031445 | AMMECR1L | 0.447827433 | 0.887708626 | 1.1089583 | up | mRNA |
| NM_031448 | C19orf12 | 0.431699671 | 0.880121832 | 1.0958414 | up | mRNA |
| NM_031449 | ZMIZ2 | 0.305942342 | 0.801497191 | 1.2310101 | up | mRNA |
| NM_031450 | C11orf68 | 0.382164863 | 0.853514109 | 1.0603001 | up | mRNA |
| NM_031451 | TEX101 | 0.372198413 | 0.847333796 | 1.1199223 | up | mRNA |
| NM_031455 | CCDC3 | 0.413749007 | 0.86961884 | 1.0415881 | up | mRNA |
| NM_031462 | CD99L2 | 0.505784685 | 0.911021812 | 1.0624649 | up | mRNA |
| NM_031468 | CALN1 | 0.656191526 | 0.957440295 | 1.0469408 | up | mRNA |
| NM_031469 | SH3BGRL2 | 0.46398401 | 0.894989844 | 1.0641931 | up | mRNA |
| NM_031471 | FERMT3 | 0.144352371 | 0.605523499 | 1.2676722 | up | mRNA |
| NM_031476 | CRISPLD2 | 0.763686279 | 0.973679653 | 1.0641964 | up | mRNA |
| NM_031479 | INHBE | 0.440422476 | 0.883544565 | 1.0725874 | up | mRNA |
| NM_031484 | MARVELD1 | 0.612239582 | 0.944159427 | 1.0694693 | up | mRNA |
| NM_031498 | GNGT2 | 0.596148542 | 0.939108209 | 1.1059969 | up | mRNA |
| NM_031844 | HNRNPU | 0.445819764 | 0.886234595 | 1.0879419 | up | mRNA |
| NM_031857 | PCDHA9 | 0.500470129 | 0.909680616 | 1.3925246 | up | mRNA |
| NM_031860 | PCDHA10 | 0.105543956 | 0.537720406 | 1.1354861 | up | mRNA |
| NM_031862 | NBR1 | 0.433871719 | 0.880923255 | 1.080651 | up | mRNA |
| NM_031890 | CECR6 | 0.263011298 | 0.763125731 | 1.0775731 | up | mRNA |
| NM_031892 | SH3KBP1 | 0.025037035 | 0.309266703 | 1.3211528 | up | mRNA |
| NM_031894 | FTHL17 | 0.02770264 | 0.32381669 | 1.373107 | up | mRNA |
| NM_031895 | CACNG8 | 0.181509223 | 0.664894785 | 1.0999429 | up | mRNA |
| NM_031900 | AGXT2 | 0.383175607 | 0.853514109 | 1.2543793 | up | mRNA |
| NM_031903 | MRPL32 | 0.051752916 | 0.41031217 | 1.2375983 | up | mRNA |
| NM_031904 | FRMD8 | 0.28913566 | 0.7849788 | 1.4129328 | up | mRNA |
| NM_031916 | ROPN1L | 0.917998457 | 0.995349793 | 1.0089706 | up | mRNA |
| NM_031919 | FSD1L | 0.603802492 | 0.941514447 | 1.0541288 | up | mRNA |
| NM_031937 | TBC1D10A | 0.242805759 | 0.741106522 | 1.2341442 | up | mRNA |
| NM_031938 | BCO2 | 0.698872992 | 0.962701213 | 1.0189957 | up | mRNA |
| NM_031948 | PRSS27 | 0.575955916 | 0.933656777 | 1.1528213 | up | mRNA |
| NM_031950 | FGFBP2 | 0.920926859 | 0.995349793 | 1.0401489 | up | mRNA |
| NM_031951 | WDR87 | 0.944324944 | 0.996710991 | 1.0102409 | up | mRNA |
| NM_031954 | KCTD10 | 0.418215758 | 0.8719404 | 1.0601744 | up | mRNA |
| NM_031958 | KRTAP3-1 | 0.662808763 | 0.958354006 | 1.0460929 | up | mRNA |
| NM_031965 | GSG2 | 0.382342891 | 0.853514109 | 1.2928293 | up | mRNA |
| NM_032014 | MRPS24 | 0.000997991 | 0.085705167 | 1.5681443 | up | mRNA |
| NM_032019 | HDAC10 | 0.810211218 | 0.98118927 | 1.0123043 | up | mRNA |
| NM_032021 | TMEM133 | 0.309041396 | 0.803961878 | 1.2061425 | up | mRNA |
| NM_032023 | RASSF4 | 0.765865785 | 0.974685975 | 1.0668225 | up | mRNA |
| NM_032036 | IFI27L2 | 0.054829916 | 0.419413793 | 1.105536 | up | mRNA |
| NM_032045 | KREMEN1 | 0.98501615 | 0.998971807 | 1.0024713 | up | mRNA |
| NM_032048 | EMILIN2 | 0.419495182 | 0.872365335 | 1.1315361 | up | mRNA |
| NM_032092 | PCDHGA11 | 0.944810049 | 0.996710991 | 1.0080245 | up | mRNA |
| NM_032116 | KATNAL1 | 0.787014393 | 0.977479172 | 1.0406482 | up | mRNA |
| NM_032118 | WDR54 | 0.671799475 | 0.959531882 | 1.0628758 | up | mRNA |
| NM_032119 | GPR98 | 0.342713975 | 0.8313124 | 1.133797 | up | mRNA |
| NM_032130 | FAM186B | 0.521576503 | 0.916644975 | 1.0643146 | up | mRNA |
| NM_032133 | MYCBPAP | 0.310044297 | 0.804899469 | 1.1267087 | up | mRNA |
| NM_032135 | FSCB | 0.615441058 | 0.945458828 | 1.0730155 | up | mRNA |
| NM_032138 | KBTBD7 | 0.033538923 | 0.347820389 | 1.568486 | up | mRNA |
| NM_032140 | C16orf48 | 0.337251119 | 0.827040134 | 1.1564046 | up | mRNA |
| NM_032141 | NSRP1 | 0.836115181 | 0.986575022 | 1.0221493 | up | mRNA |
| NM_032143 | ZRANB3 | 0.533303123 | 0.920530493 | 1.1782989 | up | mRNA |
| NM_032148 | SLC41A2 | 0.006343631 | 0.180328763 | 1.4175718 | up | mRNA |
| NM_032152 | PRAM1 | 0.782613109 | 0.977106756 | 1.0297457 | up | mRNA |
| NM_032160 | DSEL | 0.978499883 | 0.998661138 | 1.0046899 | up | mRNA |
| NM_032164 | ZNF394 | 0.00041014 | 0.062231059 | 1.2611472 | up | mRNA |
| NM_032167 | SNX29 | 0.734041239 | 0.967777568 | 1.031476 | up | mRNA |
| NM_032172 | USP42 | 0.139557285 | 0.598750522 | 1.2474263 | up | mRNA |
| NM_032194 | RPF2 | 0.919371617 | 0.995349793 | 1.0164829 | up | mRNA |
| NM_032204 | ASCC2 | 0.304351595 | 0.800038203 | 1.1344494 | up | mRNA |
| NM_032205 | PHF20L1 | 0.93903939 | 0.996710991 | 1.0196563 | up | mRNA |
| NM_032206 | NLRC5 | 0.308280546 | 0.803275671 | 1.0807977 | up | mRNA |
| NM_032221 | CHD6 | 0.354982463 | 0.837818031 | 1.0905083 | up | mRNA |
| NM_032223 | PCNXL3 | 0.940631984 | 0.996710991 | 1.0101369 | up | mRNA |
| NM_032227 | TMEM164 | 0.428295415 | 0.877211962 | 1.1759901 | up | mRNA |
| NM_032251 | CCDC88B | 0.423138895 | 0.873989281 | 1.207844 | up | mRNA |
| NM_032258 | TBC1D3F | 0.792799345 | 0.979003983 | 1.0227203 | up | mRNA |
| NM_032268 | ZNRF1 | 0.098986384 | 0.523009123 | 1.1768562 | up | mRNA |
| NM_032269 | CCDC135 | 0.878774478 | 0.991908215 | 1.0236186 | up | mRNA |
| NM_032270 | LRRC8C | 0.004869838 | 0.158753988 | 2.0551895 | up | mRNA |
| NM_032271 | TRAF7 | 0.597126279 | 0.939714862 | 1.0350367 | up | mRNA |
| NM_032288 | FYTTD1 | 0.306088382 | 0.801520161 | 1.1021399 | up | mRNA |
| NM_032290 | ANKRD32 | 0.017592578 | 0.267267899 | 1.302446 | up | mRNA |
| NM_032291 | SGIP1 | 0.837052515 | 0.986775289 | 1.0165024 | up | mRNA |
| NM_032301 | FBXW9 | 0.876453058 | 0.991213905 | 1.0285813 | up | mRNA |
| NM_032302 | PSMG3 | 0.5045888 | 0.910568383 | 1.0852668 | up | mRNA |
| NM_032309 | CHCHD5 | 0.180061863 | 0.662747609 | 1.0416787 | up | mRNA |
| NM_032311 | POLDIP3 | 0.241984521 | 0.740522167 | 1.0653686 | up | mRNA |
| NM_032316 | NICN1 | 0.385326033 | 0.85515381 | 1.1317037 | up | mRNA |
| NM_032326 | TMEM175 | 0.883504803 | 0.992766819 | 1.0075111 | up | mRNA |
| NM_032331 | ECE2 | 0.645578564 | 0.955130468 | 1.0138073 | up | mRNA |
| NM_032333 | FAM213A | 0.66073944 | 0.958354006 | 1.0945384 | up | mRNA |
| NM_032339 | MIEN1 | 0.521449958 | 0.916644975 | 1.0616471 | up | mRNA |
| NM_032340 | MNF1 | 0.190486603 | 0.676402188 | 1.0967216 | up | mRNA |
| NM_032342 | TMEM246 | 0.626704836 | 0.949203235 | 1.0893921 | up | mRNA |
| NM_032349 | NUDT16L1 | 0.330530363 | 0.821853462 | 1.0607037 | up | mRNA |
| NM_032354 | TMEM107 | 0.935075777 | 0.996710991 | 1.0073291 | up | mRNA |
| NM_032359 | C3orf26 | 0.609422431 | 0.943053241 | 1.0972561 | up | mRNA |
| NM_032366 | C16orf13 | 0.909801766 | 0.995342392 | 1.0069995 | up | mRNA |
| NM_032370 | ZNF414 | 0.722647075 | 0.966191059 | 1.0224328 | up | mRNA |
| NM_032372 | DYDC2 | 0.224666382 | 0.719349659 | 1.2567242 | up | mRNA |
| NM_032380 | GFM2 | 0.94848741 | 0.996710991 | 1.0052142 | up | mRNA |
| NM_032382 | COG8 | 0.686653047 | 0.960205549 | 1.0202495 | up | mRNA |
| NM_032389 | ARFGAP2 | 0.138516244 | 0.597943156 | 1.0583668 | up | mRNA |
| NM_032390 | MKI67IP | 0.196035825 | 0.683905351 | 1.1711812 | up | mRNA |
| NM_032391 | PRAC | 0.279389745 | 0.777944714 | 1.2001747 | up | mRNA |
| NM_032411 | C2orf40 | 0.866663974 | 0.989481868 | 1.0194495 | up | mRNA |
| NM_032412 | CYSTM1 | 0.427301011 | 0.876836544 | 1.0639778 | up | mRNA |
| NM_032413 | C15orf48 | 0.000117034 | 0.03965902 | 33.4188419 | up | mRNA |
| NM_032420 | PCDH1 | 0.205529793 | 0.695756183 | 1.189733 | up | mRNA |
| NM_032421 | CLIP2 | 0.080297093 | 0.483876854 | 1.6451174 | up | mRNA |
| NM_032427 | MAML2 | 0.208277962 | 0.698911952 | 1.2752493 | up | mRNA |
| NM_032450 | HEATR7A | 0.548507738 | 0.92514258 | 1.0688901 | up | mRNA |
| NM_032454 | STK19 | 0.744009326 | 0.970330526 | 1.0225456 | up | mRNA |
| NM_032459 | EFS | 0.406001753 | 0.866541972 | 1.0600808 | up | mRNA |
| NM_032470 | TNXB | 0.607930108 | 0.942753028 | 1.0649529 | up | mRNA |
| NM_032477 | MRPL41 | 0.892077232 | 0.993792904 | 1.0106686 | up | mRNA |
| NM_032483 | PPAPDC1B | 0.681378756 | 0.960111041 | 1.0270426 | up | mRNA |
| NM_032484 | GHDC | 0.901033427 | 0.994789506 | 1.0048975 | up | mRNA |
| NM_032488 | CNFN | 0.554287306 | 0.926549129 | 1.0314629 | up | mRNA |
| NM_032489 | ACRBP | 0.587304299 | 0.93579682 | 1.0513627 | up | mRNA |
| NM_032492 | JAGN1 | 0.358296437 | 0.840546205 | 1.0426563 | up | mRNA |
| NM_032505 | KBTBD8 | 0.408936918 | 0.867591329 | 1.0770004 | up | mRNA |
| NM_032507 | PGBD1 | 0.155645296 | 0.623194202 | 1.3826282 | up | mRNA |
| NM_032508 | TMEM185A | 0.662899453 | 0.958354006 | 1.0686224 | up | mRNA |
| NM_032510 | PARD6G | 0.927651687 | 0.995829678 | 1.0162453 | up | mRNA |
| NM_032512 | PDZD4 | 0.869578647 | 0.989592579 | 1.0169908 | up | mRNA |
| NM_032513 | SLC30A2 | 0.956236529 | 0.996883425 | 1.0081582 | up | mRNA |
| NM_032514 | MAP1LC3A | 0.801284443 | 0.98118927 | 1.0351792 | up | mRNA |
| NM_032515 | BOK | 0.061262733 | 0.437893235 | 1.2530879 | up | mRNA |
| NM_032518 | COL25A1 | 0.542230424 | 0.923577434 | 1.0502864 | up | mRNA |
| NM_032523 | OSBPL6 | 0.638213541 | 0.952852353 | 1.0890443 | up | mRNA |
| NM_032527 | ZGPAT | 0.420971486 | 0.872788744 | 1.1778156 | up | mRNA |
| NM_032532 | FNDC1 | 0.393739816 | 0.860623301 | 1.0726994 | up | mRNA |
| NM_032538 | TTBK1 | 0.277570209 | 0.777234326 | 1.0722721 | up | mRNA |
| NM_032546 | TRIM54 | 0.002761933 | 0.124513589 | 1.3265104 | up | mRNA |
| NM_032551 | KISS1R | 0.09126105 | 0.50968981 | 1.4111742 | up | mRNA |
| NM_032563 | LCE3D | 0.077583388 | 0.478499021 | 1.1160859 | up | mRNA |
| NM_032564 | DGAT2 | 0.781030442 | 0.976938981 | 1.0459422 | up | mRNA |
| NM_032569 | GLYR1 | 0.464859327 | 0.894989844 | 1.0883115 | up | mRNA |
| NM_032571 | EMR3 | 0.613206003 | 0.944565792 | 1.0532679 | up | mRNA |
| NM_032574 | DPY30 | 0.451479691 | 0.888854908 | 1.1793761 | up | mRNA |
| NM_032579 | RETNLB | 0.48569853 | 0.903250293 | 1.1110535 | up | mRNA |
| NM_032580 | HES7 | 0.373090251 | 0.848112577 | 1.1410025 | up | mRNA |
| NM_032581 | FAM126A | 0.697943787 | 0.962410025 | 1.0428136 | up | mRNA |
| NM_032587 | CARD6 | 0.18284083 | 0.666166668 | 1.7983107 | up | mRNA |
| NM_032590 | KDM2B | 0.33918741 | 0.827695551 | 1.0453321 | up | mRNA |
| NM_032596 | C9orf24 | 0.25934705 | 0.759808377 | 1.0609599 | up | mRNA |
| NM_032599 | FAM71F1 | 0.61965152 | 0.947444797 | 1.0620069 | up | mRNA |
| NM_032607 | CREB3L3 | 0.190948651 | 0.676402188 | 1.3510868 | up | mRNA |
| NM_032620 | GTPBP3 | 0.750007285 | 0.970725371 | 1.0583707 | up | mRNA |
| NM_032623 | MGARP | 0.787651675 | 0.977492986 | 1.0284992 | up | mRNA |
| NM_032626 | RBBP6 | 0.056746254 | 0.424322651 | 1.8291275 | up | mRNA |
| NM_032637 | SKP2 | 0.715170461 | 0.966191059 | 1.0214734 | up | mRNA |
| NM_032646 | TTYH2 | 0.093134374 | 0.512201269 | 1.1495809 | up | mRNA |
| NM_032648 | FAM167B | 0.43125712 | 0.879911667 | 1.0966596 | up | mRNA |
| NM_032649 | CNDP1 | 0.46584466 | 0.895570502 | 1.1641215 | up | mRNA |
| NM_032656 | DHX37 | 0.388558221 | 0.857898453 | 1.1513781 | up | mRNA |
| NM_032667 | BSCL2 | 0.454434185 | 0.890473771 | 1.0268604 | up | mRNA |
| NM_032681 | TRIM51 | 0.018305481 | 0.27048476 | 1.3559646 | up | mRNA |
| NM_032682 | FOXP1 | 0.252985626 | 0.753288068 | 1.1986307 | up | mRNA |
| NM_032711 | MAFG | 0.570329791 | 0.932906321 | 1.0406986 | up | mRNA |
| NM_032714 | INF2 | 0.49266908 | 0.906047853 | 1.053044 | up | mRNA |
| NM_032725 | BUD13 | 0.526759925 | 0.917975454 | 1.1379084 | up | mRNA |
| NM_032732 | IL17RC | 0.060697472 | 0.436078091 | 1.1118481 | up | mRNA |
| NM_032738 | FCRLA | 0.305645066 | 0.801330686 | 1.1680346 | up | mRNA |
| NM_032747 | USMG5 | 0.365576702 | 0.845008704 | 1.0513332 | up | mRNA |
| NM_032752 | ZNF496 | 0.209175149 | 0.700304482 | 1.0942437 | up | mRNA |
| NM_032753 | RAX2 | 0.162377131 | 0.634650487 | 1.2874777 | up | mRNA |
| NM_032772 | ZNF503 | 0.763502691 | 0.973567683 | 1.0330412 | up | mRNA |
| NM_032777 | GPR124 | 0.119280718 | 0.568456822 | 1.0493287 | up | mRNA |
| NM_032780 | TMEM25 | 0.792731151 | 0.979003983 | 1.0322981 | up | mRNA |
| NM_032781 | PTPN5 | 0.167967178 | 0.642921845 | 1.063199 | up | mRNA |
| NM_032782 | HAVCR2 | 0.591720627 | 0.937303262 | 1.0502659 | up | mRNA |
| NM_032788 | ZNF514 | 0.008608766 | 0.199898185 | 1.2603079 | up | mRNA |
| NM_032792 | ZBTB45 | 0.089251051 | 0.50505215 | 1.2197858 | up | mRNA |
| NM_032793 | MFSD2A | 0.027895507 | 0.324654525 | 1.1399388 | up | mRNA |
| NM_032800 | C1orf198 | 0.492431178 | 0.906047853 | 1.0598806 | up | mRNA |
| NM_032802 | SPPL2A | 0.264923866 | 0.76613535 | 1.0768779 | up | mRNA |
| NM_032810 | ATAD1 | 0.831905384 | 0.986141573 | 1.0122862 | up | mRNA |
| NM_032815 | NFATC2IP | 0.62109357 | 0.947926299 | 1.0627378 | up | mRNA |
| NM_032817 | ITIH5 | 0.098562631 | 0.522412778 | 1.2845362 | up | mRNA |
| NM_032823 | C9orf3 | 0.630683206 | 0.950410442 | 1.0755866 | up | mRNA |
| NM_032826 | SLC35B4 | 0.719753564 | 0.966191059 | 1.0717497 | up | mRNA |
| NM_032829 | FAM222A | 0.975788877 | 0.998501532 | 1.0034217 | up | mRNA |
| NM_032833 | PPP1R15B | 0.010832225 | 0.213674567 | 1.4910261 | up | mRNA |
| NM_032868 | MPND | 0.726430573 | 0.966191059 | 1.0220046 | up | mRNA |
| NM_032869 | NUDCD1 | 0.104375997 | 0.535011073 | 1.1051589 | up | mRNA |
| NM_032884 | C1orf94 | 0.745242905 | 0.970378122 | 1.1035629 | up | mRNA |
| NM_032888 | COL27A1 | 0.294070574 | 0.791146775 | 4.4239549 | up | mRNA |
| NM_032898 | CEP19 | 0.979324669 | 0.998935223 | 1.0065555 | up | mRNA |
| NM_032899 | FAM83A | 0.701024824 | 0.962991468 | 1.0391386 | up | mRNA |
| NM_032901 | COX14 | 0.294797637 | 0.791301949 | 1.0686633 | up | mRNA |
| NM_032918 | RERG | 0.803792768 | 0.98118927 | 1.0648878 | up | mRNA |
| NM_032928 | TMEM141 | 0.481058113 | 0.901538317 | 1.0650361 | up | mRNA |
| NM_032933 | TMEM241 | 0.950417535 | 0.996710991 | 1.0070446 | up | mRNA |
| NM_032949 | PCDH8 | 0.447108963 | 0.886972498 | 1.0594088 | up | mRNA |
| NM_032960 | MAPKAPK2 | 0.464696642 | 0.894989844 | 1.0617373 | up | mRNA |
| NM_032968 | PCDH11X | 0.356134626 | 0.838847221 | 1.220095 | up | mRNA |
| NM_032969 | PCDH11X | 0.915781844 | 0.995349793 | 1.0090383 | up | mRNA |
| NM_032983 | CASP2 | 0.499718468 | 0.909460484 | 1.0977146 | up | mRNA |
| NM_033011 | PLAT | 0.317486828 | 0.811824899 | 1.1969986 | up | mRNA |
| NM_033016 | PDGFB | 0.853911713 | 0.987855862 | 1.0441684 | up | mRNA |
| NM_033018 | CDK16 | 0.222190352 | 0.717189116 | 1.0991284 | up | mRNA |
| NM_033022 | RPS24 | 0.525483682 | 0.917686109 | 1.0591441 | up | mRNA |
| NM_033023 | PDGFA | 0.999197752 | 0.999897336 | 1.0003395 | up | mRNA |
| NM_033027 | CSRNP1 | 0.234362456 | 0.731734866 | 1.0951333 | up | mRNA |
| NM_033028 | BBS4 | 0.960710452 | 0.997798988 | 1.003344 | up | mRNA |
| NM_033031 | CCNB3 | 0.181525475 | 0.664894785 | 1.3582119 | up | mRNA |
| NM_033035 | TSLP | 0.01399321 | 0.238634683 | 1.5830138 | up | mRNA |
| NM_033045 | KRT84 | 0.009756027 | 0.206194951 | 1.415876 | up | mRNA |
| NM_033054 | MYO1G | 0.00186869 | 0.105670067 | 1.9365268 | up | mRNA |
| NM_033060 | KRTAP4-1 | 0.389080526 | 0.85800274 | 1.0912293 | up | mRNA |
| NM_033070 | CECR5 | 0.348175636 | 0.834138149 | 1.0329812 | up | mRNA |
| NM_033082 | SARNP | 0.597152562 | 0.939714862 | 1.0386177 | up | mRNA |
| NM_033083 | EAF1 | 0.227883376 | 0.723854154 | 1.1012921 | up | mRNA |
| NM_033084 | FANCD2 | 0.974210769 | 0.998501532 | 1.0246424 | up | mRNA |
| NM_033085 | FATE1 | 0.840096418 | 0.987068842 | 1.0102249 | up | mRNA |
| NM_033092 | TRIM5 | 0.862903256 | 0.989401142 | 1.0387692 | up | mRNA |
| NM_033102 | SLC45A3 | 0.652959238 | 0.956757512 | 1.0470433 | up | mRNA |
| NM_033115 | TBCK | 0.921466838 | 0.995349793 | 1.0343389 | up | mRNA |
| NM_033118 | MYLK2 | 0.502714729 | 0.910354901 | 1.1683608 | up | mRNA |
| NM_033122 | CABS1 | 0.763005387 | 0.97349057 | 1.0260585 | up | mRNA |
| NM_033125 | SLC22A16 | 0.514720665 | 0.913840427 | 1.1434034 | up | mRNA |
| NM_033131 | WNT3A | 0.22526156 | 0.720245224 | 1.0572331 | up | mRNA |
| NM_033136 | FGF1 | 0.127912818 | 0.581220503 | 1.2136095 | up | mRNA |
| NM_033143 | FGF5 | 0.075104186 | 0.470853901 | 1.9035397 | up | mRNA |
| NM_033148 | DTNB | 0.602340226 | 0.941406011 | 1.1239352 | up | mRNA |
| NM_033157 | CALD1 | 0.954356166 | 0.996710991 | 1.015867 | up | mRNA |
| NM_033165 | FGF8 | 0.072458844 | 0.463255488 | 1.1771475 | up | mRNA |
| NM_033173 | B3GALT5 | 0.499600049 | 0.909460484 | 1.1664984 | up | mRNA |
| NM_033176 | NKX2-4 | 0.464276556 | 0.894989844 | 1.1540732 | up | mRNA |
| NM_033178 | DUX4 | 0.672108617 | 0.959531882 | 1.0813263 | up | mRNA |
| NM_033199 | UCN2 | 0.077494254 | 0.478473478 | 1.1379823 | up | mRNA |
| NM_033200 | LMF2 | 0.612387035 | 0.944159427 | 1.060609 | up | mRNA |
| NM_033201 | C16orf45 | 0.629783133 | 0.950410442 | 1.1019087 | up | mRNA |
| NM_033207 | OPALIN | 0.256501044 | 0.757240077 | 1.3304267 | up | mRNA |
| NM_033213 | ZNF670 | 0.018102297 | 0.269173581 | 2.7577485 | up | mRNA |
| NM_033215 | PPP1R3F | 0.663526573 | 0.958354006 | 1.1377549 | up | mRNA |
| NM_033224 | PURB | 0.046100432 | 0.391970965 | 1.1775232 | up | mRNA |
| NM_033238 | PML | 0.136712761 | 0.595655761 | 1.2023425 | up | mRNA |
| NM_033239 | PML | 0.006701859 | 0.183552613 | 1.7951544 | up | mRNA |
| NM_033240 | PML | 0.353013551 | 0.83624275 | 1.0977173 | up | mRNA |
| NM_033244 | PML | 0.05289207 | 0.413038171 | 1.3317504 | up | mRNA |
| NM_033246 | PML | 0.0804596 | 0.484568897 | 1.2912321 | up | mRNA |
| NM_033247 | PML | 0.630758867 | 0.950410442 | 1.034072 | up | mRNA |
| NM_033250 | PML | 0.110649195 | 0.550457408 | 1.2435331 | up | mRNA |
| NM_033280 | SEC11C | 0.933688707 | 0.996710991 | 1.0132812 | up | mRNA |
| NM_033281 | MRPS36 | 0.925501149 | 0.995465766 | 1.0194229 | up | mRNA |
| NM_033292 | CASP1 | 0.407971952 | 0.867591329 | 1.0602599 | up | mRNA |
| NM_033301 | RPL8 | 0.186563681 | 0.669953595 | 1.116286 | up | mRNA |
| NM_033305 | VPS13A | 0.796657407 | 0.980665598 | 1.0180074 | up | mRNA |
| NM_033306 | CASP4 | 0.415102525 | 0.870239791 | 1.1695896 | up | mRNA |
| NM_033334 | NR6A1 | 0.188329057 | 0.673545538 | 1.2073804 | up | mRNA |
| NM_033340 | CASP7 | 0.547342972 | 0.925055514 | 1.2423576 | up | mRNA |
| NM_033341 | BIRC8 | 0.24575435 | 0.743996045 | 1.1002567 | up | mRNA |
| NM_033345 | RGS8 | 0.921760033 | 0.995349793 | 1.0180061 | up | mRNA |
| NM_033358 | CASP8 | 0.495326726 | 0.907415174 | 1.0840147 | up | mRNA |
| NM_033375 | MYO1C | 0.482926232 | 0.902654749 | 1.0988931 | up | mRNA |
| NM_033377 | CGB1 | 0.120622071 | 0.572011066 | 1.1923119 | up | mRNA |
| NM_033382 | SEC14L2 | 0.539881998 | 0.923577434 | 1.0627412 | up | mRNA |
| NM_033386 | MICALL1 | 0.799654678 | 0.981146515 | 1.0179718 | up | mRNA |
| NM_033394 | TANC1 | 0.480418419 | 0.901538317 | 1.2810084 | up | mRNA |
| NM_033397 | ITPRIP | 0.785361902 | 0.977411899 | 1.0615946 | up | mRNA |
| NM_033409 | SLC52A3 | 0.144603123 | 0.605950267 | 1.2498531 | up | mRNA |
| NM_033410 | ZNF764 | 0.682773443 | 0.960111041 | 1.0869676 | up | mRNA |
| NM_033412 | SLC25A51 | 0.000154683 | 0.044621794 | 1.3144547 | up | mRNA |
| NM_033416 | IMP4 | 0.864399718 | 0.989401142 | 1.0396962 | up | mRNA |
| NM_033419 | PGAP3 | 0.25922065 | 0.759762759 | 1.1375258 | up | mRNA |
| NM_033425 | DIXDC1 | 0.447345955 | 0.88723888 | 1.0763346 | up | mRNA |
| NM_033426 | KIAA1737 | 0.413825802 | 0.86961884 | 1.1262559 | up | mRNA |
| NM_033427 | CTTNBP2 | 0.111126672 | 0.551887519 | 1.1749894 | up | mRNA |
| NM_033428 | C9orf123 | 0.947967968 | 0.996710991 | 1.0070858 | up | mRNA |
| NM_033429 | CALML4 | 0.030866266 | 0.340185047 | 1.25485 | up | mRNA |
| NM_033430 | PDE5A | 0.859120603 | 0.988512246 | 1.0649408 | up | mRNA |
| NM_033482 | POM121L2 | 0.815744464 | 0.981459009 | 1.0481315 | up | mRNA |
| NM_033495 | KLHL13 | 0.388667614 | 0.857898453 | 1.0894431 | up | mRNA |
| NM_033505 | EPT1 | 0.999639715 | 0.999940005 | 1.0000878 | up | mRNA |
| NM_033510 | DISP2 | 0.896604641 | 0.994222243 | 1.0432853 | up | mRNA |
| NM_033529 | CDK11A | 0.722520553 | 0.966191059 | 1.0315529 | up | mRNA |
| NM_033542 | SYS1 | 0.286327371 | 0.782895119 | 1.0321507 | up | mRNA |
| NM_033543 | CEACAM21 | 0.27335885 | 0.773331313 | 1.1404928 | up | mRNA |
| NM_033553 | GUCA2A | 0.159570564 | 0.631449728 | 1.2644146 | up | mRNA |
| NM_033554 | HLA-DPA1 | 0.461682381 | 0.894667841 | 1.0664887 | up | mRNA |
| NM_033629 | TREX1 | 0.543190512 | 0.923577434 | 1.0218555 | up | mRNA |
| NM_033644 | FBXW11 | 0.172015013 | 0.649834644 | 1.1225629 | up | mRNA |
| NM_033645 | FBXW11 | 0.822382743 | 0.983107122 | 1.0153243 | up | mRNA |
| NM_033663 | DRD3 | 0.184248624 | 0.667764384 | 1.2777881 | up | mRNA |
| NM_033670 | CCNB3 | 0.195897094 | 0.683773342 | 1.3143446 | up | mRNA |
| NM_044472 | CDC42 | 0.942418653 | 0.996710991 | 1.0068295 | up | mRNA |
| NM_052817 | MID2 | 0.814896711 | 0.981277402 | 1.1284935 | up | mRNA |
| NM_052818 | N4BP2L1 | 0.302862398 | 0.798262675 | 1.4936909 | up | mRNA |
| NM_052827 | CDK2 | 0.170909303 | 0.647703251 | 1.2285815 | up | mRNA |
| NM_052828 | TRIM10 | 0.505486045 | 0.910772598 | 1.053163 | up | mRNA |
| NM_052836 | CDH23 | 0.956830789 | 0.996883425 | 1.0036778 | up | mRNA |
| NM_052841 | TSSK3 | 0.061326115 | 0.437893235 | 1.4645298 | up | mRNA |
| NM_052848 | CCDC97 | 0.816634846 | 0.981692737 | 1.0134896 | up | mRNA |
| NM_052849 | C15orf57 | 0.317077261 | 0.811824899 | 1.1951119 | up | mRNA |
| NM_052851 | STARD13 | 0.029103173 | 0.331684822 | 1.5489245 | up | mRNA |
| NM_052854 | CREB3L1 | 0.817203098 | 0.981692737 | 1.0094195 | up | mRNA |
| NM_052862 | RCSD1 | 0.008541087 | 0.19914837 | 2.1520638 | up | mRNA |
| NM_052864 | TIFA | 2.36384E-05 | 0.020025668 | 8.1993575 | up | mRNA |
| NM_052869 | TTYH2 | 0.51543443 | 0.914319064 | 1.0515098 | up | mRNA |
| NM_052870 | SNX18 | 0.739563775 | 0.969564098 | 1.0451036 | up | mRNA |
| NM_052874 | STX1B | 0.464285055 | 0.894989844 | 1.1280125 | up | mRNA |
| NM_052876 | NACC1 | 0.190671009 | 0.676402188 | 1.0719362 | up | mRNA |
| NM_052879 | LARP4 | 0.073356385 | 0.466088132 | 1.074665 | up | mRNA |
| NM_052884 | SIGLEC11 | 0.83010749 | 0.985549562 | 1.0123014 | up | mRNA |
| NM_052889 | CARD16 | 0.995305689 | 0.999872237 | 1.0016801 | up | mRNA |
| NM_052898 | XKR4 | 0.785914249 | 0.977479172 | 1.0913766 | up | mRNA |
| NM_052901 | SLC25A25 | 0.583083013 | 0.934269626 | 1.077705 | up | mRNA |
| NM_052924 | RHPN1 | 0.186085759 | 0.669763792 | 1.1106415 | up | mRNA |
| NM_052925 | LENG8 | 0.119688308 | 0.56964014 | 1.1491653 | up | mRNA |
| NM_052932 | TMEM123 | 0.924673904 | 0.995456159 | 1.0022789 | up | mRNA |
| NM_052934 | SLC26A9 | 0.160635712 | 0.632017029 | 1.19958 | up | mRNA |
| NM_052937 | PCMTD1 | 0.912698332 | 0.995342392 | 1.0178951 | up | mRNA |
| NM_052941 | GBP4 | 0.695158412 | 0.962214902 | 1.0452225 | up | mRNA |
| NM_052944 | SLC5A11 | 0.217220098 | 0.711339274 | 1.263591 | up | mRNA |
| NM_052945 | TNFRSF13C | 0.532090143 | 0.920228311 | 1.0287447 | up | mRNA |
| NM_052947 | ALPK2 | 0.822368855 | 0.983107122 | 1.0998565 | up | mRNA |
| NM_052952 | DIRC1 | 0.428518297 | 0.877316889 | 1.1323669 | up | mRNA |
| NM_052957 | ACRC | 0.114268623 | 0.558219521 | 1.1807849 | up | mRNA |
| NM_052958 | C8orf34 | 0.464989999 | 0.895027612 | 1.1893351 | up | mRNA |
| NM_052963 | TOP1MT | 0.903181943 | 0.99508468 | 1.0133312 | up | mRNA |
| NM_052966 | FAM129A | 0.125452833 | 0.578441772 | 1.8781857 | up | mRNA |
| NM_052968 | APOA5 | 0.00016742 | 0.044621794 | 1.4591174 | up | mRNA |
| NM_052969 | RPL39L | 0.729783811 | 0.966191059 | 1.0711268 | up | mRNA |
| NM_052970 | HSPA12B | 0.062091961 | 0.439379649 | 1.22458 | up | mRNA |
| NM_052971 | LEAP2 | 0.861901244 | 0.989401142 | 1.0166049 | up | mRNA |
| NM_052985 | IFT122 | 0.563336733 | 0.929041598 | 1.060845 | up | mRNA |
| NM_053003 | SIGLEC12 | 0.132291285 | 0.587843174 | 1.2211993 | up | mRNA |
| NM_053023 | ZFP91 | 0.1160414 | 0.562308327 | 1.1960266 | up | mRNA |
| NM_053024 | PFN2 | 0.982500839 | 0.998971807 | 1.0041369 | up | mRNA |
| NM_053025 | MYLK | 0.250608163 | 0.749980154 | 1.4475321 | up | mRNA |
| NM_053026 | MYLK | 0.974324793 | 0.998501532 | 1.0020146 | up | mRNA |
| NM_053027 | MYLK | 0.939846126 | 0.996710991 | 1.033859 | up | mRNA |
| NM_053032 | MYLK | 0.796465584 | 0.980606652 | 1.118189 | up | mRNA |
| NM_053034 | ANTXR1 | 0.051699353 | 0.41031217 | 1.094626 | up | mRNA |
| NM_053049 | UCN3 | 0.600588228 | 0.940757262 | 1.0599835 | up | mRNA |
| NM_053064 | GNG2 | 0.850798915 | 0.987678166 | 1.0599726 | up | mRNA |
| NM_053275 | RPLP0 | 0.700988889 | 0.962991468 | 1.0195063 | up | mRNA |
| NM_053276 | VIT | 0.943759654 | 0.996710991 | 1.0128162 | up | mRNA |
| NM_053277 | CLIC6 | 0.283034572 | 0.780627498 | 1.0780061 | up | mRNA |
| NM_053280 | ODF3 | 0.09098259 | 0.50932214 | 1.1440387 | up | mRNA |
| NM_054012 | ASS1 | 0.119249789 | 0.568456822 | 1.1008773 | up | mRNA |
| NM_054013 | MGAT4B | 0.934882261 | 0.996710991 | 1.018183 | up | mRNA |
| NM_054014 | FKBP1A | 0.23196506 | 0.729740617 | 1.1575975 | up | mRNA |
| NM_054022 | GOSR2 | 0.65125166 | 0.956204521 | 1.0258847 | up | mRNA |
| NM_054023 | SCGB3A2 | 0.680704878 | 0.960111041 | 1.074642 | up | mRNA |
| NM_054028 | SLC35G5 | 0.001851394 | 0.105670067 | 1.3506172 | up | mRNA |
| NM_054108 | HRASLS5 | 0.350715086 | 0.834911159 | 1.0970328 | up | mRNA |
| NM_054114 | TAGAP | 0.378304756 | 0.851828819 | 1.0477048 | up | mRNA |
| NM_057091 | ARTN | 0.005606824 | 0.16989262 | 1.1328556 | up | mRNA |
| NM_057093 | CRYBA2 | 0.75057451 | 0.970725371 | 1.0340379 | up | mRNA |
| NM_057174 | PEX16 | 0.524877861 | 0.917632491 | 1.0555025 | up | mRNA |
| NM_057176 | BSND | 0.305244902 | 0.801056074 | 1.090943 | up | mRNA |
| NM_057177 | PARD3B | 0.977083441 | 0.998501532 | 1.0104607 | up | mRNA |
| NM_057180 | VPS29 | 0.481476104 | 0.901682656 | 1.0370351 | up | mRNA |
| NM_058164 | OLFM2 | 0.012114818 | 0.222675671 | 1.1414114 | up | mRNA |
| NM_058172 | ANTXR2 | 0.959220712 | 0.997466408 | 1.0180763 | up | mRNA |
| NM_058173 | MUCL1 | 0.880053503 | 0.992197209 | 1.0136427 | up | mRNA |
| NM_058181 | YBEY | 0.745516301 | 0.970394407 | 1.0328758 | up | mRNA |
| NM_058182 | FAM165B | 0.883401266 | 0.992766819 | 1.0202127 | up | mRNA |
| NM_058190 | FAM207A | 0.73389051 | 0.967777568 | 1.050571 | up | mRNA |
| NM_058203 | SPAG11B | 0.491678878 | 0.905806259 | 1.1546918 | up | mRNA |
| NM_058238 | WNT7B | 0.272518486 | 0.772493443 | 1.2668764 | up | mRNA |
| NM_078481 | CD97 | 0.544725371 | 0.924012779 | 1.0659724 | up | mRNA |
| NM_078485 | COL9A1 | 0.418851346 | 0.872328878 | 1.0999498 | up | mRNA |
| NM_078487 | CDKN2B | 0.009358619 | 0.204533521 | 2.0901687 | up | mRNA |
| NM_079421 | CDKN2D | 0.162606943 | 0.63494386 | 1.0700295 | up | mRNA |
| NM_079422 | MYL1 | 0.060661202 | 0.435971562 | 1.1774248 | up | mRNA |
| NM_079423 | MYL6 | 0.66880635 | 0.959036237 | 1.020922 | up | mRNA |
| NM_079837 | BANP | 0.866427752 | 0.989481868 | 1.0270038 | up | mRNA |
| NM_080386 | TUBA3D | 0.719792403 | 0.966191059 | 1.0720241 | up | mRNA |
| NM_080415 | SEPT4 | 0.179817915 | 0.662598807 | 1.1314709 | up | mRNA |
| NM_080422 | PTPN2 | 0.562371082 | 0.929041598 | 1.0551648 | up | mRNA |
| NM_080423 | PTPN2 | 0.547992741 | 0.92514258 | 1.0712333 | up | mRNA |
| NM_080430 | SELM | 0.69158976 | 0.961451327 | 1.052518 | up | mRNA |
| NM_080489 | SDCBP2 | 0.569037272 | 0.93232103 | 1.0690092 | up | mRNA |
| NM_080604 | TJAP1 | 0.520060668 | 0.916394575 | 1.0583713 | up | mRNA |
| NM_080607 | VSTM2L | 0.718739089 | 0.966191059 | 1.0185373 | up | mRNA |
| NM_080608 | SPATA25 | 0.300190523 | 0.795797471 | 1.0930926 | up | mRNA |
| NM_080611 | DUSP15 | 0.775535265 | 0.97589756 | 1.0120713 | up | mRNA |
| NM_080625 | C20orf160 | 0.998496536 | 0.999889342 | 1.0004453 | up | mRNA |
| NM_080626 | BRI3BP | 0.762617696 | 0.973443358 | 1.0487179 | up | mRNA |
| NM_080627 | SOGA1 | 0.403858495 | 0.865367868 | 1.0848185 | up | mRNA |
| NM_080630 | COL11A1 | 0.922044259 | 0.995349793 | 1.0926631 | up | mRNA |
| NM_080632 | UPF3B | 0.470270646 | 0.898180112 | 1.1281975 | up | mRNA |
| NM_080651 | MED30 | 0.857870055 | 0.988512246 | 1.0268796 | up | mRNA |
| NM_080655 | MSANTD3 | 0.883108762 | 0.992766819 | 1.0112798 | up | mRNA |
| NM_080656 | CDKN2AIPNL | 0.997016287 | 0.999889342 | 1.000438 | up | mRNA |
| NM_080662 | PEX11G | 0.654914173 | 0.957205183 | 1.0537263 | up | mRNA |
| NM_080668 | CDCA5 | 0.823430811 | 0.983498765 | 1.0257622 | up | mRNA |
| NM_080669 | SLC46A1 | 0.916511431 | 0.995349793 | 1.013064 | up | mRNA |
| NM_080670 | SLC35A4 | 0.024148586 | 0.303829853 | 1.1231817 | up | mRNA |
| NM_080671 | KCNE4 | 0.15327011 | 0.61991006 | 1.3816121 | up | mRNA |
| NM_080676 | MACROD2 | 0.5368485 | 0.922650285 | 1.2424288 | up | mRNA |
| NM_080682 | VCAM1 | 0.008425661 | 0.198394583 | 10.3018586 | up | mRNA |
| NM_080686 | PRRC2A | 0.472659579 | 0.899184677 | 1.0765403 | up | mRNA |
| NM_080687 | UPF3A | 0.46983994 | 0.897941557 | 1.0762297 | up | mRNA |
| NM_080706 | TRPV1 | 0.133751493 | 0.590539708 | 1.4327333 | up | mRNA |
| NM_080722 | ADAMTS14 | 0.360927023 | 0.841780454 | 1.0678847 | up | mRNA |
| NM_080730 | IFFO1 | 0.197242937 | 0.685177414 | 1.2337049 | up | mRNA |
| NM_080739 | C20orf141 | 0.586802187 | 0.93579682 | 1.07094 | up | mRNA |
| NM_080741 | NEU4 | 0.495356626 | 0.907415174 | 1.051307 | up | mRNA |
| NM_080743 | SRSF12 | 0.000821165 | 0.083087642 | 6.0447818 | up | mRNA |
| NM_080746 | RPL10L | 0.000670589 | 0.076919551 | 1.5728327 | up | mRNA |
| NM_080748 | ROMO1 | 0.44873986 | 0.888279993 | 1.047072 | up | mRNA |
| NM_080753 | WFDC10A | 0.880660791 | 0.992287164 | 1.0277366 | up | mRNA |
| NM_080794 | MRPL39 | 0.043857103 | 0.385351177 | 2.2058009 | up | mRNA |
| NM_080800 | COL13A1 | 0.39292362 | 0.859763564 | 1.0448909 | up | mRNA |
| NM_080802 | COL13A1 | 0.364650778 | 0.844043673 | 1.0854031 | up | mRNA |
| NM_080816 | SIRPG | 0.993979727 | 0.999872237 | 1.0003953 | up | mRNA |
| NM_080825 | C20orf144 | 0.160646469 | 0.632017029 | 1.0811269 | up | mRNA |
| NM_080829 | FAM65C | 0.547925103 | 0.92514258 | 1.0525537 | up | mRNA |
| NM_080830 | CST11 | 0.86534527 | 0.989401142 | 1.0796488 | up | mRNA |
| NM_080832 | PABPC5 | 0.777027518 | 0.976821563 | 1.0645177 | up | mRNA |
| NM_080838 | WISP1 | 0.099598727 | 0.524350421 | 1.2938536 | up | mRNA |
| NM_080841 | PTPRA | 0.370461725 | 0.846215286 | 1.0749908 | up | mRNA |
| NM_080862 | SPSB4 | 0.947448487 | 0.996710991 | 1.0056187 | up | mRNA |
| NM_080878 | ITLN2 | 0.370691779 | 0.846330273 | 1.1920938 | up | mRNA |
| NM_080911 | UNG | 0.977604646 | 0.998501532 | 1.0011563 | up | mRNA |
| NM_080913 | ASGR2 | 0.017949083 | 0.269173581 | 1.1418661 | up | mRNA |
| NM_130384 | ATRIP | 0.923366582 | 0.995456159 | 1.0364906 | up | mRNA |
| NM_130386 | COLEC12 | 0.949055487 | 0.996710991 | 1.0179686 | up | mRNA |
| NM_130398 | EXO1 | 0.862093724 | 0.989401142 | 1.0872369 | up | mRNA |
| NM_130445 | COL18A1 | 0.396557466 | 0.861995469 | 1.0611548 | up | mRNA |
| NM_130466 | UBE3B | 0.891460902 | 0.9937521 | 1.0056173 | up | mRNA |
| NM_130468 | CHST14 | 0.951747425 | 0.996710991 | 1.0095864 | up | mRNA |
| NM_130469 | JDP2 | 0.677535268 | 0.959531882 | 1.0351088 | up | mRNA |
| NM_130762 | MADCAM1 | 0.004893567 | 0.158753988 | 1.2299373 | up | mRNA |
| NM_130766 | INPP5K | 0.860446823 | 0.988975154 | 1.0155617 | up | mRNA |
| NM_130771 | OSCAR | 0.701309168 | 0.963015537 | 1.028532 | up | mRNA |
| NM_130785 | TPTE2 | 0.488936757 | 0.90439066 | 1.2048616 | up | mRNA |
| NM_130791 | WWOX | 0.840182794 | 0.987068842 | 1.0593042 | up | mRNA |
| NM_130794 | CST11 | 0.245454032 | 0.743640499 | 1.0816452 | up | mRNA |
| NM_130798 | SNAP23 | 0.793180118 | 0.979003983 | 1.0119629 | up | mRNA |
| NM_130799 | MEN1 | 0.227649727 | 0.723780179 | 1.0653922 | up | mRNA |
| NM_130802 | MEN1 | 0.805444156 | 0.98118927 | 1.024919 | up | mRNA |
| NM_130807 | MOB3A | 0.396278666 | 0.861995469 | 1.1432922 | up | mRNA |
| NM_130847 | AMOTL1 | 0.233847716 | 0.73118887 | 1.0972699 | up | mRNA |
| NM_130851 | BMP4 | 0.024306603 | 0.304521009 | 1.3605602 | up | mRNA |
| NM_130898 | CREB3L4 | 0.535721168 | 0.921980938 | 1.0952305 | up | mRNA |
| NM_133168 | OSCAR | 0.885097434 | 0.992981836 | 1.0265114 | up | mRNA |
| NM_133169 | OSCAR | 0.182511252 | 0.66575662 | 1.1812811 | up | mRNA |
| NM_133172 | APBB3 | 0.741709747 | 0.970240145 | 1.049529 | up | mRNA |
| NM_133181 | EPS8L3 | 0.206592002 | 0.698066071 | 1.7191436 | up | mRNA |
| NM_133263 | PPARGC1B | 0.985464341 | 0.998971807 | 1.0015486 | up | mRNA |
| NM_133277 | FCAR | 0.226308973 | 0.721667695 | 1.1260118 | up | mRNA |
| NM_133279 | FCAR | 0.257131968 | 0.757669958 | 1.1762864 | up | mRNA |
| NM_133337 | MYOF | 0.621618533 | 0.947997886 | 1.0536212 | up | mRNA |
| NM_133369 | UNC5A | 0.06997256 | 0.457987595 | 1.3697728 | up | mRNA |
| NM_133373 | PLCD3 | 0.915255893 | 0.995349793 | 1.0128788 | up | mRNA |
| NM_133374 | ZNF618 | 0.853166787 | 0.987678166 | 1.0223277 | up | mRNA |
| NM_133443 | GPT2 | 0.095623852 | 0.51694341 | 1.6570432 | up | mRNA |
| NM_133445 | GRIN3A | 0.281392973 | 0.779126326 | 1.7420518 | up | mRNA |
| NM_133455 | EMID1 | 0.995187947 | 0.999872237 | 1.0004682 | up | mRNA |
| NM_133463 | AMZ1 | 0.073310755 | 0.465943817 | 1.11067 | up | mRNA |
| NM_133467 | CITED4 | 0.003755554 | 0.142058481 | 6.206387 | up | mRNA |
| NM_133486 | MBNL3 | 0.726923535 | 0.966191059 | 1.0222488 | up | mRNA |
| NM_133497 | KCNV2 | 0.838262293 | 0.986897498 | 1.0194997 | up | mRNA |
| NM_133498 | SPACA4 | 0.210265653 | 0.700954461 | 1.1179109 | up | mRNA |
| NM_133504 | DCN | 0.964323908 | 0.99793927 | 1.0144384 | up | mRNA |
| NM_133507 | DCN | 0.950741592 | 0.996710991 | 1.0222066 | up | mRNA |
| NM_133509 | RAD51B | 0.965002164 | 0.99793927 | 1.0067604 | up | mRNA |
| NM_133510 | RAD51B | 0.991829376 | 0.999548897 | 1.0034591 | up | mRNA |
| NM_133631 | ROBO1 | 0.274005523 | 0.774088254 | 1.2722774 | up | mRNA |
| NM_133635 | POFUT2 | 0.85591391 | 0.988315074 | 1.0103162 | up | mRNA |
| NM_133637 | DQX1 | 0.200878822 | 0.68900633 | 1.152646 | up | mRNA |
| NM_133644 | GTPBP3 | 0.525882625 | 0.917868103 | 1.0576861 | up | mRNA |
| NM_134260 | RORA | 0.154517173 | 0.621861274 | 1.1219624 | up | mRNA |
| NM_134261 | RORA | 0.90588837 | 0.99508468 | 1.0289302 | up | mRNA |
| NM_134262 | RORA | 0.953129475 | 0.996710991 | 1.0109846 | up | mRNA |
| NM_134268 | CYGB | 0.288594985 | 0.784514546 | 1.1824074 | up | mRNA |
| NM_134325 | SLC26A9 | 0.207672217 | 0.698556218 | 1.1196747 | up | mRNA |
| NM_134425 | SLC26A1 | 0.193429963 | 0.680621781 | 1.4031563 | up | mRNA |
| NM_134426 | SLC26A6 | 0.139407708 | 0.598648178 | 1.0730292 | up | mRNA |
| NM_134441 | RLN2 | 0.724263059 | 0.966191059 | 1.1821 | up | mRNA |
| NM_134444 | NLRP4 | 0.162694919 | 0.63503803 | 1.3060384 | up | mRNA |
| NM_134445 | CD99L2 | 0.271326763 | 0.77133889 | 1.0565965 | up | mRNA |
| NM_134446 | CD99L2 | 0.200736436 | 0.68900633 | 1.1020795 | up | mRNA |
| NM_138274 | C6orf25 | 0.894823196 | 0.993978977 | 1.0175655 | up | mRNA |
| NM_138281 | DLX4 | 0.839406674 | 0.987032229 | 1.0240192 | up | mRNA |
| NM_138287 | DTX3L | 0.046229757 | 0.392057225 | 1.3404879 | up | mRNA |
| NM_138288 | SPTSSA | 0.711059566 | 0.96568622 | 1.0465181 | up | mRNA |
| NM_138295 | PKD1L1 | 0.022706312 | 0.2968905 | 2.1476732 | up | mRNA |
| NM_138296 | PTCRA | 0.131586435 | 0.586786184 | 1.2095198 | up | mRNA |
| NM_138321 | PCSK6 | 0.720115636 | 0.966191059 | 1.0442594 | up | mRNA |
| NM_138335 | GNPDA2 | 0.986201675 | 0.998971807 | 1.0008504 | up | mRNA |
| NM_138344 | FAM181A | 0.818983429 | 0.982103868 | 1.1048775 | up | mRNA |
| NM_138350 | THAP3 | 0.277816327 | 0.777287651 | 1.2219377 | up | mRNA |
| NM_138360 | LRRC16B | 0.924656728 | 0.995456159 | 1.0280324 | up | mRNA |
| NM_138371 | PCED1B | 0.567167315 | 0.931514776 | 1.5475699 | up | mRNA |
| NM_138375 | CABLES1 | 0.606140319 | 0.94174007 | 1.0870732 | up | mRNA |
| NM_138384 | MTG1 | 0.781103871 | 0.976938981 | 1.0782785 | up | mRNA |
| NM_138385 | TMEM129 | 0.556086975 | 0.926833616 | 1.2187021 | up | mRNA |
| NM_138387 | G6PC3 | 0.698772703 | 0.962701213 | 1.0216357 | up | mRNA |
| NM_138389 | FAM114A1 | 0.107083698 | 0.54246118 | 1.0959523 | up | mRNA |
| NM_138392 | SHKBP1 | 0.302434112 | 0.797857839 | 1.2750815 | up | mRNA |
| NM_138409 | MRAP2 | 0.593997095 | 0.938299318 | 1.0802133 | up | mRNA |
| NM_138410 | CMTM7 | 0.829572641 | 0.985549562 | 1.0132012 | up | mRNA |
| NM_138413 | HOGA1 | 0.116613033 | 0.562996237 | 1.3662659 | up | mRNA |
| NM_138420 | AHNAK2 | 0.835971274 | 0.986575022 | 1.0100039 | up | mRNA |
| NM_138423 | CASC4 | 0.55364779 | 0.926337111 | 1.0558438 | up | mRNA |
| NM_138425 | C12orf57 | 0.091102303 | 0.509602046 | 1.0890327 | up | mRNA |
| NM_138426 | GLCCI1 | 0.217088029 | 0.711335019 | 1.1860124 | up | mRNA |
| NM_138430 | ADPRHL1 | 0.50079569 | 0.909680616 | 1.2083435 | up | mRNA |
| NM_138433 | KLHDC7B | 0.368160539 | 0.845312226 | 1.1851317 | up | mRNA |
| NM_138434 | C7orf29 | 0.552722899 | 0.926337111 | 1.3216836 | up | mRNA |
| NM_138441 | MB21D1 | 0.062517176 | 0.439458294 | 1.3789025 | up | mRNA |
| NM_138448 | ACYP2 | 0.578791782 | 0.933821303 | 1.0988207 | up | mRNA |
| NM_138453 | RAB3C | 0.735223878 | 0.96794858 | 1.0452888 | up | mRNA |
| NM_138454 | NXNL1 | 0.401027778 | 0.864768873 | 1.1893247 | up | mRNA |
| NM_138455 | CTHRC1 | 0.379747304 | 0.853151623 | 1.3742349 | up | mRNA |
| NM_138458 | WDR92 | 0.583765764 | 0.934397621 | 1.0167647 | up | mRNA |
| NM_138463 | TLCD1 | 0.189356993 | 0.674719659 | 1.1606418 | up | mRNA |
| NM_138465 | GLI4 | 0.921428041 | 0.995349793 | 1.0470579 | up | mRNA |
| NM_138466 | ZNF837 | 0.948794398 | 0.996710991 | 1.0076908 | up | mRNA |
| NM_138468 | ICA1L | 0.021789757 | 0.290701669 | 1.1154079 | up | mRNA |
| NM_138471 | C11orf84 | 0.335107798 | 0.824952235 | 1.061853 | up | mRNA |
| NM_138485 | GTPBP8 | 0.888384317 | 0.993440915 | 1.0414438 | up | mRNA |
| NM_138492 | PRELID2 | 0.012640797 | 0.229680687 | 1.2776373 | up | mRNA |
| NM_138493 | CCDC167 | 0.891717746 | 0.9937521 | 1.0136426 | up | mRNA |
| NM_138554 | TLR4 | 0.259761653 | 0.760388386 | 1.3026782 | up | mRNA |
| NM_138559 | BCL11A | 0.730184001 | 0.966191059 | 1.0619362 | up | mRNA |
| NM_138567 | SYT8 | 0.061577355 | 0.437989276 | 1.3695782 | up | mRNA |
| NM_138572 | TAF8 | 0.506663188 | 0.911415165 | 1.1603406 | up | mRNA |
| NM_138612 | HAS3 | 0.619830213 | 0.947444797 | 1.1018785 | up | mRNA |
| NM_138615 | DHX30 | 0.455411917 | 0.891148383 | 1.1095795 | up | mRNA |
| NM_138621 | BCL2L11 | 0.007839774 | 0.193915182 | 2.035471 | up | mRNA |
| NM_138635 | H2AFV | 0.967415153 | 0.99793927 | 1.0064615 | up | mRNA |
| NM_138720 | HIST1H2BD | 0.022568325 | 0.295888941 | 1.2421031 | up | mRNA |
| NM_138737 | HEPH | 0.991146823 | 0.999376923 | 1.001333 | up | mRNA |
| NM_138761 | BAX | 0.191024069 | 0.676402188 | 1.3444925 | up | mRNA |
| NM_138763 | BAX | 0.148673248 | 0.61203168 | 1.3849037 | up | mRNA |
| NM_138764 | BAX | 0.174071991 | 0.652513223 | 1.452471 | up | mRNA |
| NM_138766 | PAM | 0.869536696 | 0.989592579 | 1.0236333 | up | mRNA |
| NM_138770 | CCDC74A | 0.868304012 | 0.989481868 | 1.0145317 | up | mRNA |
| NM_138777 | MRRF | 0.812181502 | 0.98118927 | 1.0593375 | up | mRNA |
| NM_138787 | C11orf74 | 0.055046191 | 0.419520587 | 1.0667056 | up | mRNA |
| NM_138790 | PLD4 | 0.905549039 | 0.99508468 | 1.0095852 | up | mRNA |
| NM_138793 | CANT1 | 0.007781135 | 0.19345927 | 1.0791462 | up | mRNA |
| NM_138809 | CMBL | 0.428763666 | 0.877386753 | 1.0295368 | up | mRNA |
| NM_138811 | C7orf31 | 0.850075143 | 0.987678166 | 1.0130912 | up | mRNA |
| NM_138820 | HIGD2A | 0.712658542 | 0.966110889 | 1.0413175 | up | mRNA |
| NM_138821 | PAM | 0.162897493 | 0.635098861 | 1.1650226 | up | mRNA |
| NM_138929 | DIABLO | 0.69255853 | 0.961584652 | 1.0412526 | up | mRNA |
| NM_138960 | TGIF2LX | 0.366903944 | 0.845181673 | 1.1836555 | up | mRNA |
| NM_138971 | BACE1 | 0.115452581 | 0.561007186 | 1.082551 | up | mRNA |
| NM_138972 | BACE1 | 0.059438865 | 0.432586127 | 1.0855386 | up | mRNA |
| NM_138973 | BACE1 | 0.176265154 | 0.655417542 | 1.0879181 | up | mRNA |
| NM_138982 | MAPK10 | 0.062393943 | 0.439458294 | 1.1409386 | up | mRNA |
| NM_138995 | MYO3B | 0.201021708 | 0.689092046 | 1.1633115 | up | mRNA |
| NM_139009 | HFE | 0.787036714 | 0.977479172 | 1.1407152 | up | mRNA |
| NM_139010 | HFE | 0.492018706 | 0.905806259 | 1.1954162 | up | mRNA |
| NM_139011 | HFE | 0.653373315 | 0.956860606 | 1.0423148 | up | mRNA |
| NM_139017 | IL31RA | 0.269845709 | 0.770082629 | 1.1325336 | up | mRNA |
| NM_139053 | EPS8L3 | 0.07655291 | 0.475659052 | 1.2364274 | up | mRNA |
| NM_139067 | SMARCC2 | 0.716893743 | 0.966191059 | 1.0345581 | up | mRNA |
| NM_139069 | MAPK9 | 0.819972168 | 0.982484618 | 1.0521214 | up | mRNA |
| NM_139076 | FAM175A | 0.403324686 | 0.864786348 | 1.2219275 | up | mRNA |
| NM_139119 | YY1AP1 | 0.288211846 | 0.784355942 | 1.0639006 | up | mRNA |
| NM_139131 | NUP98 | 0.958044887 | 0.996933183 | 1.0078469 | up | mRNA |
| NM_139135 | ARID1A | 0.798078363 | 0.980749101 | 1.0190958 | up | mRNA |
| NM_139155 | ADAMTS14 | 0.361257569 | 0.842165256 | 1.1150556 | up | mRNA |
| NM_139162 | SMCR7 | 0.419803066 | 0.872365335 | 1.1321562 | up | mRNA |
| NM_139208 | MASP2 | 0.351133045 | 0.835187247 | 1.064629 | up | mRNA |
| NM_139211 | HOPX | 0.836002261 | 0.986575022 | 1.0292066 | up | mRNA |
| NM_139215 | TAF15 | 0.822790229 | 0.983305175 | 1.0246047 | up | mRNA |
| NM_139245 | PPM1L | 0.905439892 | 0.99508468 | 1.0413692 | up | mRNA |
| NM_139276 | STAT3 | 0.050410666 | 0.406242436 | 1.3381479 | up | mRNA |
| NM_139278 | LGI3 | 0.285488047 | 0.782178004 | 1.2991047 | up | mRNA |
| NM_139284 | LGI4 | 0.635893608 | 0.951814727 | 1.1311039 | up | mRNA |
| NM_139312 | YME1L1 | 0.31657464 | 0.811181435 | 1.0469997 | up | mRNA |
| NM_139322 | ATRN | 0.949927511 | 0.996710991 | 1.0045827 | up | mRNA |
| NM_139343 | BIN1 | 0.916040376 | 0.995349793 | 1.0124086 | up | mRNA |
| NM_139345 | BIN1 | 0.311336485 | 0.806503245 | 1.0708974 | up | mRNA |
| NM_139349 | BIN1 | 0.083887991 | 0.493179896 | 1.1027093 | up | mRNA |
| NM_144492 | CLDN14 | 0.091298952 | 0.50968981 | 1.2249312 | up | mRNA |
| NM_144495 | PQBP1 | 0.091718152 | 0.510767863 | 1.1339484 | up | mRNA |
| NM_144497 | AKAP12 | 0.860206811 | 0.988947627 | 1.1113154 | up | mRNA |
| NM_144499 | GNAT1 | 0.981852262 | 0.998971807 | 1.0037246 | up | mRNA |
| NM_144565 | DUOXA1 | 0.709627333 | 0.965462762 | 1.042018 | up | mRNA |
| NM_144568 | TMEM55B | 0.757472889 | 0.972045462 | 1.0557057 | up | mRNA |
| NM_144570 | HN1L | 0.280626852 | 0.779126326 | 1.2678631 | up | mRNA |
| NM_144576 | COQ10A | 0.562812035 | 0.929041598 | 1.1004973 | up | mRNA |
| NM_144582 | TEX261 | 0.540411647 | 0.923577434 | 1.0371634 | up | mRNA |
| NM_144598 | LRRC28 | 0.886314799 | 0.992981836 | 1.0306819 | up | mRNA |
| NM_144599 | NIPA1 | 0.031594909 | 0.341807319 | 1.1307654 | up | mRNA |
| NM_144604 | ZC3H18 | 0.951534219 | 0.996710991 | 1.0085511 | up | mRNA |
| NM_144605 | SEPT12 | 0.414126538 | 0.86961884 | 1.0979754 | up | mRNA |
| NM_144606 | FLCN | 0.654831854 | 0.957205183 | 1.0365755 | up | mRNA |
| NM_144607 | CYB5D1 | 0.016617816 | 0.257525485 | 1.2116051 | up | mRNA |
| NM_144608 | HEXIM2 | 0.584509183 | 0.93461341 | 1.1849848 | up | mRNA |
| NM_144622 | DCST2 | 0.93441576 | 0.996710991 | 1.0125298 | up | mRNA |
| NM_144627 | C1orf182 | 0.744275854 | 0.970330526 | 1.1495321 | up | mRNA |
| NM_144629 | RFTN2 | 0.14535077 | 0.606832003 | 1.1111071 | up | mRNA |
| NM_144634 | LYZL4 | 0.714735594 | 0.966110889 | 1.0431192 | up | mRNA |
| NM_144646 | IGJ | 0.694331501 | 0.962043956 | 1.2530153 | up | mRNA |
| NM_144666 | DNHD1 | 0.258244942 | 0.759091536 | 1.1690712 | up | mRNA |
| NM_144671 | FAM109A | 0.953555129 | 0.996710991 | 1.0071063 | up | mRNA |
| NM_144676 | TMED6 | 0.061790822 | 0.438663056 | 1.2734231 | up | mRNA |
| NM_144695 | BROX | 0.940338327 | 0.996710991 | 1.0097589 | up | mRNA |
| NM_144698 | ANKRD35 | 0.557307331 | 0.926833616 | 1.1271363 | up | mRNA |
| NM_144699 | ATP1A4 | 0.942435653 | 0.996710991 | 1.0259645 | up | mRNA |
| NM_144701 | IL23R | 0.896721673 | 0.994222243 | 1.038189 | up | mRNA |
| NM_144710 | SEPT10 | 0.917034086 | 0.995349793 | 1.0093791 | up | mRNA |
| NM_144715 | EFHB | 0.235353793 | 0.732804491 | 1.3830271 | up | mRNA |
| NM_144723 | ZMAT2 | 0.187246452 | 0.671419231 | 1.1178845 | up | mRNA |
| NM_144769 | FOXI1 | 0.031007892 | 0.340185047 | 1.5304644 | up | mRNA |
| NM_144772 | APOA1BP | 0.957241275 | 0.996883425 | 1.0057398 | up | mRNA |
| NM_144778 | MBNL2 | 0.679309189 | 0.959969193 | 1.0451503 | up | mRNA |
| NM_144779 | FXYD5 | 0.791713495 | 0.978905301 | 1.029654 | up | mRNA |
| NM_144965 | TTC16 | 0.958880969 | 0.99728722 | 1.0090161 | up | mRNA |
| NM_144968 | RIBC1 | 0.649053088 | 0.95550188 | 1.0207267 | up | mRNA |
| NM_144974 | CCDC122 | 0.252490277 | 0.752621656 | 1.2071199 | up | mRNA |
| NM_144975 | SLFN5 | 0.003362205 | 0.137189565 | 1.6388501 | up | mRNA |
| NM_144977 | DENND1B | 0.284211562 | 0.78179061 | 1.1880057 | up | mRNA |
| NM_144979 | RBM46 | 0.275335596 | 0.775184524 | 1.3278335 | up | mRNA |
| NM_144982 | ZFC3H1 | 0.92406566 | 0.995456159 | 1.0222262 | up | mRNA |
| NM_144984 | VSTM4 | 0.052263392 | 0.412347416 | 1.5943053 | up | mRNA |
| NM_144989 | DNAH14 | 0.722673719 | 0.966191059 | 1.0387174 | up | mRNA |
| NM_144993 | TET3 | 0.012108035 | 0.222675671 | 2.1656595 | up | mRNA |
| NM_144997 | FLCN | 0.330106781 | 0.821769369 | 1.1087129 | up | mRNA |
| NM_144998 | STRA13 | 0.970183078 | 0.998245244 | 1.0160421 | up | mRNA |
| NM_145001 | STK32A | 0.924397025 | 0.995456159 | 1.0045033 | up | mRNA |
| NM_145003 | TSNARE1 | 0.760596251 | 0.972931992 | 1.0210491 | up | mRNA |
| NM_145006 | SUSD3 | 0.682757887 | 0.960111041 | 1.0382582 | up | mRNA |
| NM_145019 | FAM124A | 0.476513336 | 0.900415348 | 1.1545801 | up | mRNA |
| NM_145029 | C6orf136 | 0.658684317 | 0.958354006 | 1.0509405 | up | mRNA |
| NM_145036 | CEP112 | 0.413543136 | 0.86961884 | 1.1745025 | up | mRNA |
| NM_145047 | OSCP1 | 0.202673602 | 0.691983154 | 1.0782233 | up | mRNA |
| NM_145051 | RNF183 | 0.732592385 | 0.967703318 | 1.0324238 | up | mRNA |
| NM_145055 | C18orf25 | 0.837510433 | 0.986775289 | 1.0191039 | up | mRNA |
| NM_145064 | STAC3 | 0.676262371 | 0.959531882 | 1.0535921 | up | mRNA |
| NM_145102 | ZKSCAN5 | 0.427143287 | 0.876731038 | 1.1532691 | up | mRNA |
| NM_145117 | NAV2 | 0.43802903 | 0.882682266 | 1.3708469 | up | mRNA |
| NM_145119 | PJA1 | 0.923293685 | 0.995456159 | 1.0104473 | up | mRNA |
| NM_145175 | FAM84A | 0.170466193 | 0.64730412 | 1.1572885 | up | mRNA |
| NM_145177 | DHRSX | 0.97643416 | 0.998501532 | 1.0021061 | up | mRNA |
| NM_145178 | ATOH7 | 0.000866424 | 0.083094924 | 3.3949236 | up | mRNA |
| NM_145185 | MAP2K7 | 0.656312941 | 0.957440295 | 1.0312037 | up | mRNA |
| NM_145186 | ABCC11 | 0.86925731 | 0.989592579 | 1.039063 | up | mRNA |
| NM_145200 | CABP4 | 0.157026254 | 0.625751326 | 1.1484282 | up | mRNA |
| NM_145204 | SENP8 | 0.706581726 | 0.965401045 | 1.0668281 | up | mRNA |
| NM_145230 | ATP6V0E2 | 0.911112153 | 0.995342392 | 1.008445 | up | mRNA |
| NM_145231 | EFCAB11 | 0.496979116 | 0.908766921 | 1.071622 | up | mRNA |
| NM_145234 | CHRDL1 | 0.918326267 | 0.995349793 | 1.0490888 | up | mRNA |
| NM_145239 | PRRT2 | 0.859546995 | 0.988590875 | 1.0625737 | up | mRNA |
| NM_145241 | WDR31 | 0.177183794 | 0.65715084 | 1.1709706 | up | mRNA |
| NM_145245 | EVI5L | 0.617957534 | 0.946754755 | 1.0663275 | up | mRNA |
| NM_145248 | SPACA7 | 0.174253828 | 0.652872869 | 1.6363966 | up | mRNA |
| NM_145249 | IFI27L1 | 0.663966748 | 0.958354006 | 1.0857148 | up | mRNA |
| NM_145259 | ACVR1C | 0.158763538 | 0.630341781 | 1.3786881 | up | mRNA |
| NM_145262 | GLYCTK | 0.711888011 | 0.96592341 | 1.0805981 | up | mRNA |
| NM_145266 | NUDCD2 | 0.695510917 | 0.96236244 | 1.0313 | up | mRNA |
| NM_145269 | FAM92A1 | 0.601970754 | 0.941406011 | 1.0561811 | up | mRNA |
| NM_145273 | CD300LG | 0.929874993 | 0.996643872 | 1.0095124 | up | mRNA |
| NM_145277 | HFE2 | 0.425386936 | 0.875271081 | 1.1417929 | up | mRNA |
| NM_145288 | ZNF296 | 0.609605802 | 0.943053241 | 1.0807749 | up | mRNA |
| NM_145297 | ZNF626 | 0.929618068 | 0.996533591 | 1.0169581 | up | mRNA |
| NM_145298 | APOBEC3F | 0.847136149 | 0.987678166 | 1.0535264 | up | mRNA |
| NM_145309 | LRTOMT | 0.800320729 | 0.98118927 | 1.0205835 | up | mRNA |
| NM_145312 | ZNF485 | 0.847824653 | 0.987678166 | 1.0191444 | up | mRNA |
| NM_145314 | UCMA | 0.348297022 | 0.834138149 | 1.2708236 | up | mRNA |
| NM_145316 | TMEM217 | 0.820940417 | 0.982756843 | 1.0614948 | up | mRNA |
| NM_145331 | MAP3K7 | 0.545346726 | 0.924012779 | 1.1137551 | up | mRNA |
| NM_145345 | UBXN11 | 0.039889759 | 0.371524769 | 1.1964365 | up | mRNA |
| NM_145350 | SCARF1 | 0.132963289 | 0.588930122 | 1.1712436 | up | mRNA |
| NM_145637 | APOL2 | 4.1519E-05 | 0.027231107 | 2.8015536 | up | mRNA |
| NM_145641 | APOL3 | 0.471524745 | 0.898908705 | 1.155247 | up | mRNA |
| NM_145663 | DBF4B | 0.066942235 | 0.451282995 | 1.2745588 | up | mRNA |
| NM_145686 | MAP4K4 | 0.189366856 | 0.674719659 | 1.1522489 | up | mRNA |
| NM_145687 | MAP4K4 | 0.987345155 | 0.999140676 | 1.0010593 | up | mRNA |
| NM_145698 | ACBD5 | 0.050842345 | 0.407751844 | 1.1404264 | up | mRNA |
| NM_145716 | SSBP3 | 0.322895564 | 0.815766149 | 1.2072306 | up | mRNA |
| NM_145726 | TRAF3 | 0.037044065 | 0.359628653 | 1.3158577 | up | mRNA |
| NM_145731 | SYNGR1 | 0.803629795 | 0.98118927 | 1.0220048 | up | mRNA |
| NM_145733 | SEPT3 | 0.074637113 | 0.469867247 | 1.1275754 | up | mRNA |
| NM_145740 | GSTA1 | 0.536586237 | 0.922509728 | 1.3087731 | up | mRNA |
| NM_145753 | PHLDB2 | 0.001069168 | 0.086778252 | 2.7293352 | up | mRNA |
| NM_145762 | GFRA4 | 0.572630349 | 0.933460721 | 1.0665078 | up | mRNA |
| NM_145798 | OSBPL7 | 0.616501633 | 0.945912508 | 1.1026627 | up | mRNA |
| NM_145800 | SEPT6 | 0.277528061 | 0.777234326 | 1.3229387 | up | mRNA |
| NM_145804 | ABTB2 | 0.007783282 | 0.19345927 | 2.3670857 | up | mRNA |
| NM_145807 | NTN5 | 0.611788894 | 0.943913476 | 1.1137308 | up | mRNA |
| NM_145811 | CACNG5 | 0.905254617 | 0.99508468 | 1.0229461 | up | mRNA |
| NM_145812 | AIFM1 | 0.532690695 | 0.92042723 | 1.0485886 | up | mRNA |
| NM_145867 | LTC4S | 0.9154749 | 0.995349793 | 1.0077349 | up | mRNA |
| NM_145871 | GSTZ1 | 0.782492867 | 0.977106756 | 1.0208794 | up | mRNA |
| NM_145897 | PFDN5 | 0.355450569 | 0.838369858 | 1.0888263 | up | mRNA |
| NM_145899 | HMGA1 | 0.232575326 | 0.730348439 | 1.3431614 | up | mRNA |
| NM_145905 | HMGA1 | 0.227832787 | 0.723854154 | 1.1060506 | up | mRNA |
| NM_145911 | ZNF23 | 0.554578564 | 0.92659145 | 1.1255381 | up | mRNA |
| NM_145913 | SLC5A8 | 0.844220444 | 0.987338076 | 1.048203 | up | mRNA |
| NM_146421 | GSTM1 | 0.835138942 | 0.986575022 | 1.1117442 | up | mRNA |
| NM_147152 | ITSN2 | 0.474150652 | 0.899418411 | 1.2107777 | up | mRNA |
| NM_147161 | ACOT11 | 0.816039454 | 0.981525922 | 1.0176313 | up | mRNA |
| NM_147169 | C9orf24 | 0.437523625 | 0.882425389 | 1.1881243 | up | mRNA |
| NM_147175 | HS6ST2 | 0.695443626 | 0.96236244 | 1.0292354 | up | mRNA |
| NM_147187 | TNFRSF10B | 0.004046841 | 0.145628971 | 2.008309 | up | mRNA |
| NM_147195 | ANKRD18A | 0.981249903 | 0.998971807 | 1.0023118 | up | mRNA |
| NM_147196 | TMIE | 0.463735164 | 0.894989844 | 1.1019951 | up | mRNA |
| NM_147686 | TRAF3IP2 | 0.012654007 | 0.229715413 | 1.8218587 | up | mRNA |
| NM_148170 | CTSC | 0.147809774 | 0.610583444 | 1.0989758 | up | mRNA |
| NM_148172 | PEMT | 0.414148494 | 0.86961884 | 1.0552439 | up | mRNA |
| NM_148174 | AZIN1 | 0.229798113 | 0.726859868 | 1.0920117 | up | mRNA |
| NM_148175 | PPIL2 | 0.870683764 | 0.989592579 | 1.0179813 | up | mRNA |
| NM_148416 | ATXN2L | 0.792332928 | 0.978996327 | 1.0277197 | up | mRNA |
| NM_148893 | SVIP | 0.36800048 | 0.845264883 | 1.1132552 | up | mRNA |
| NM_148896 | NPB | 0.97406479 | 0.998501532 | 1.0053434 | up | mRNA |
| NM_148913 | ABHD11 | 0.32880136 | 0.821644684 | 1.1724451 | up | mRNA |
| NM_148919 | PSMB8 | 0.344577283 | 0.831973758 | 1.0940296 | up | mRNA |
| NM_148920 | PIGQ | 0.332206451 | 0.822707947 | 1.1686125 | up | mRNA |
| NM_148923 | CYB5A | 0.112835882 | 0.555760453 | 1.1940225 | up | mRNA |
| NM_148959 | HUS1B | 0.102497699 | 0.531357269 | 1.1236669 | up | mRNA |
| NM_148976 | PSMA1 | 0.593344848 | 0.938165712 | 1.154866 | up | mRNA |
| NM_148977 | PANK1 | 0.060210621 | 0.435194581 | 1.1311762 | up | mRNA |
| NM_152132 | PSMA3 | 0.274704913 | 0.774767693 | 1.073403 | up | mRNA |
| NM_152233 | SNX6 | 0.325856784 | 0.818530154 | 1.4619861 | up | mRNA |
| NM_152260 | RPUSD2 | 0.879615323 | 0.992178089 | 1.0251156 | up | mRNA |
| NM_152263 | TPM3 | 0.588474829 | 0.93579682 | 1.0868135 | up | mRNA |
| NM_152264 | SLC39A13 | 0.685073912 | 0.960111041 | 1.073523 | up | mRNA |
| NM_152266 | C19orf40 | 0.809258874 | 0.98118927 | 1.0556376 | up | mRNA |
| NM_152267 | RNF185 | 0.981571372 | 0.998971807 | 1.0056001 | up | mRNA |
| NM_152268 | PARS2 | 0.969233217 | 0.998245244 | 1.0045734 | up | mRNA |
| NM_152270 | SLFN11 | 0.021765637 | 0.290701669 | 1.2020016 | up | mRNA |
| NM_152271 | LONRF1 | 0.56298854 | 0.929041598 | 1.0672606 | up | mRNA |
| NM_152280 | SYT11 | 0.502782875 | 0.910354901 | 1.0580567 | up | mRNA |
| NM_152281 | GORAB | 0.001267927 | 0.092399617 | 1.5773547 | up | mRNA |
| NM_152285 | ARRDC1 | 0.752776351 | 0.970725371 | 1.0433349 | up | mRNA |
| NM_152286 | PNPLA7 | 0.237740577 | 0.735771951 | 1.2283742 | up | mRNA |
| NM_152287 | ZNF276 | 0.888567426 | 0.993440915 | 1.0208116 | up | mRNA |
| NM_152293 | TADA2B | 0.124607807 | 0.578441772 | 1.2200078 | up | mRNA |
| NM_152304 | RAB42 | 0.966367319 | 0.99793927 | 1.0042861 | up | mRNA |
| NM_152313 | SLC36A4 | 0.476438202 | 0.900415348 | 1.0466459 | up | mRNA |
| NM_152319 | C12orf54 | 0.269497132 | 0.769905254 | 1.167805 | up | mRNA |
| NM_152324 | TEX29 | 0.17030644 | 0.647247575 | 1.2475292 | up | mRNA |
| NM_152330 | FRMD6 | 0.349420319 | 0.834138149 | 1.2862972 | up | mRNA |
| NM_152336 | AGBL1 | 0.868384299 | 0.989481868 | 1.0283412 | up | mRNA |
| NM_152354 | ZNF285 | 0.454993393 | 0.891140128 | 1.2126172 | up | mRNA |
| NM_152375 | KLHDC7A | 0.454199036 | 0.890270395 | 1.1254586 | up | mRNA |
| NM_152376 | UBXN10 | 0.516673719 | 0.914937059 | 1.4478994 | up | mRNA |
| NM_152383 | DIS3L2 | 0.548342208 | 0.92514258 | 1.0625413 | up | mRNA |
| NM_152388 | TMEM237 | 0.719598298 | 0.966191059 | 1.0940843 | up | mRNA |
| NM_152391 | PQLC3 | 0.952611452 | 0.996710991 | 1.0119799 | up | mRNA |
| NM_152396 | METTL6 | 0.737801238 | 0.969060091 | 1.0270363 | up | mRNA |
| NM_152398 | OCIAD2 | 0.699226292 | 0.962701213 | 1.0163719 | up | mRNA |
| NM_152407 | GRPEL2 | 0.889776558 | 0.9937521 | 1.0244514 | up | mRNA |
| NM_152409 | C5orf24 | 0.196575766 | 0.685083729 | 1.116979 | up | mRNA |
| NM_152410 | PACRG | 0.671881225 | 0.959531882 | 1.0941312 | up | mRNA |
| NM_152411 | ZNF786 | 0.077737812 | 0.479016418 | 1.1872794 | up | mRNA |
| NM_152430 | OR51E1 | 0.88743526 | 0.993247479 | 1.028331 | up | mRNA |
| NM_152473 | ERVV-1 | 0.814585711 | 0.981277402 | 1.0302946 | up | mRNA |
| NM_152491 | PM20D1 | 0.994232775 | 0.999872237 | 1.0006652 | up | mRNA |
| NM_152494 | DCST1 | 0.205998913 | 0.697016126 | 1.1959526 | up | mRNA |
| NM_152496 | MANEAL | 0.472741753 | 0.899184677 | 1.0445091 | up | mRNA |
| NM_152499 | CCDC24 | 0.343604675 | 0.831536584 | 1.1148743 | up | mRNA |
| NM_152501 | PYHIN1 | 0.414156997 | 0.86961884 | 1.1446033 | up | mRNA |
| NM_152515 | CKAP2L | 0.926341662 | 0.99579035 | 1.067757 | up | mRNA |
| NM_152516 | COMMD1 | 0.256682382 | 0.757240077 | 1.0860253 | up | mRNA |
| NM_152536 | FGD5 | 0.080093201 | 0.483876854 | 1.0958946 | up | mRNA |
| NM_152542 | PPM1K | 0.374110016 | 0.849308269 | 1.4111862 | up | mRNA |
| NM_152546 | SRFBP1 | 0.121546442 | 0.573155624 | 1.2136006 | up | mRNA |
| NM_152548 | FAM81B | 0.477093826 | 0.900429473 | 1.1168843 | up | mRNA |
| NM_152554 | C6orf195 | 0.726914666 | 0.966191059 | 1.0397954 | up | mRNA |
| NM_152568 | NKX6-3 | 0.20165067 | 0.689996873 | 1.3314229 | up | mRNA |
| NM_152570 | LINGO2 | 0.862993283 | 0.989401142 | 1.0551216 | up | mRNA |
| NM_152572 | AK8 | 0.471800267 | 0.898908705 | 1.078988 | up | mRNA |
| NM_152574 | TTC39B | 0.135738884 | 0.593770008 | 1.1601528 | up | mRNA |
| NM_152584 | HSFY1 | 0.070297704 | 0.458686035 | 1.3352609 | up | mRNA |
| NM_152586 | USP54 | 0.012259371 | 0.224556341 | 2.5390975 | up | mRNA |
| NM_152588 | TMTC2 | 0.238408454 | 0.736444734 | 1.2009806 | up | mRNA |
| NM_152591 | CCDC63 | 0.436219658 | 0.881719662 | 1.1273401 | up | mRNA |
| NM_152592 | SYNE3 | 0.538939567 | 0.923577434 | 1.0683512 | up | mRNA |
| NM_152597 | FSIP1 | 0.883745359 | 0.992766819 | 1.0611643 | up | mRNA |
| NM_152600 | ZNF579 | 0.614655254 | 0.944827645 | 1.058389 | up | mRNA |
| NM_152616 | TRIM42 | 0.362931685 | 0.843238468 | 1.1523187 | up | mRNA |
| NM_152620 | TRIM60 | 0.503097096 | 0.910475258 | 1.1902293 | up | mRNA |
| NM_152621 | SGMS2 | 0.025658288 | 0.314576454 | 1.1983168 | up | mRNA |
| NM_152624 | DCP2 | 0.698867864 | 0.962701213 | 1.0595061 | up | mRNA |
| NM_152629 | GLIS3 | 0.463135141 | 0.894989844 | 1.1109381 | up | mRNA |
| NM_152632 | CXorf22 | 0.413900614 | 0.86961884 | 1.3066092 | up | mRNA |
| NM_152638 | CCER1 | 0.985755024 | 0.998971807 | 1.0018322 | up | mRNA |
| NM_152653 | UBE2E2 | 0.857240548 | 0.988512246 | 1.0424419 | up | mRNA |
| NM_152660 | FAM76A | 0.824051383 | 0.983568283 | 1.017581 | up | mRNA |
| NM_152663 | RALGPS2 | 0.515907065 | 0.914429643 | 1.0575249 | up | mRNA |
| NM_152666 | PLD5 | 0.473515131 | 0.899184677 | 1.2035778 | up | mRNA |
| NM_152683 | CCDC111 | 0.907698441 | 0.995145546 | 1.009921 | up | mRNA |
| NM_152686 | DNAJC18 | 0.945223475 | 0.996710991 | 1.0061754 | up | mRNA |
| NM_152699 | SENP5 | 0.655902443 | 0.957336187 | 1.0312743 | up | mRNA |
| NM_152703 | SAMD9L | 0.117941376 | 0.564630104 | 1.492786 | up | mRNA |
| NM_152710 | TBATA | 0.139950455 | 0.599804521 | 1.1644427 | up | mRNA |
| NM_152716 | PATL1 | 0.065011621 | 0.445228144 | 1.1323983 | up | mRNA |
| NM_152727 | CPNE2 | 0.463733952 | 0.894989844 | 1.0905806 | up | mRNA |
| NM_152748 | KIAA1324L | 0.084917808 | 0.495565119 | 1.5138616 | up | mRNA |
| NM_152750 | CDHR3 | 0.567282793 | 0.931514776 | 1.1569832 | up | mRNA |
| NM_152755 | CNPY4 | 0.548706196 | 0.92514258 | 1.1024644 | up | mRNA |
| NM_152762 | TSGA10IP | 0.281867004 | 0.779398875 | 1.0491789 | up | mRNA |
| NM_152765 | C8orf46 | 0.18985674 | 0.675909261 | 1.1050563 | up | mRNA |
| NM_152773 | TCTEX1D2 | 0.876087969 | 0.991213905 | 1.0087648 | up | mRNA |
| NM_152781 | C17orf66 | 0.519488618 | 0.916145597 | 1.0982844 | up | mRNA |
| NM_152783 | D2HGDH | 0.101685489 | 0.529590777 | 1.1198447 | up | mRNA |
| NM_152784 | CATSPERD | 0.189487467 | 0.674957811 | 1.2343472 | up | mRNA |
| NM_152787 | TAB3 | 0.097451833 | 0.520323178 | 1.210263 | up | mRNA |
| NM_152789 | FAM133B | 0.338695919 | 0.827403747 | 1.0560089 | up | mRNA |
| NM_152793 | C7orf41 | 0.023593056 | 0.301884214 | 1.1696585 | up | mRNA |
| NM_152833 | C9orf69 | 0.995287425 | 0.999872237 | 1.0006856 | up | mRNA |
| NM_152834 | TMEM18 | 0.366812385 | 0.845181673 | 1.0787999 | up | mRNA |
| NM_152850 | PIGO | 0.351648359 | 0.835247013 | 1.0737812 | up | mRNA |
| NM_152851 | MS4A6A | 0.465955262 | 0.895613764 | 1.3713492 | up | mRNA |
| NM_152852 | MS4A6A | 0.497404796 | 0.908789022 | 1.159006 | up | mRNA |
| NM_152858 | WTAP | 0.003954874 | 0.144623204 | 2.710681 | up | mRNA |
| NM_152860 | SP7 | 0.490023769 | 0.905248343 | 1.0887369 | up | mRNA |
| NM_152871 | FAS | 0.33013977 | 0.821769369 | 1.1133027 | up | mRNA |
| NM_152889 | CHST13 | 0.965058001 | 0.99793927 | 1.0026538 | up | mRNA |
| NM_152897 | SNX21 | 0.990034112 | 0.999140676 | 1.0016511 | up | mRNA |
| NM_152899 | IL4I1 | 0.054147777 | 0.417336086 | 1.120697 | up | mRNA |
| NM_152902 | TIPRL | 0.301753869 | 0.796890463 | 1.1100833 | up | mRNA |
| NM_152904 | SPECC1 | 0.959406644 | 0.997578802 | 1.0058086 | up | mRNA |
| NM_152905 | NEDD1 | 0.001009988 | 0.085705167 | 4.5874641 | up | mRNA |
| NM_152906 | C22orf25 | 0.764920417 | 0.97415819 | 1.0349148 | up | mRNA |
| NM_152910 | DGKH | 0.011698481 | 0.220344574 | 1.366607 | up | mRNA |
| NM_152911 | PAOX | 0.325605905 | 0.818119039 | 1.0785694 | up | mRNA |
| NM_152913 | TMEM130 | 0.092112263 | 0.511207518 | 1.0994959 | up | mRNA |
| NM_152914 | C17orf103 | 0.775280817 | 0.97589756 | 1.0967076 | up | mRNA |
| NM_152942 | TNFRSF8 | 0.570709672 | 0.93296476 | 1.0462634 | up | mRNA |
| NM_152988 | SPPL2B | 0.275864196 | 0.775741613 | 1.1479204 | up | mRNA |
| NM_152989 | SOX5 | 0.242601607 | 0.740802161 | 1.2095353 | up | mRNA |
| NM_152998 | EZH2 | 0.278836873 | 0.777783404 | 1.6851666 | up | mRNA |
| NM_153001 | PSMC4 | 0.976508829 | 0.998501532 | 1.0009933 | up | mRNA |
| NM_153007 | ODF4 | 0.068106915 | 0.452817212 | 1.3795769 | up | mRNA |
| NM_153013 | NADKD1 | 0.939499383 | 0.996710991 | 1.0100385 | up | mRNA |
| NM_153015 | TMEM74 | 0.64629056 | 0.95550188 | 1.1828393 | up | mRNA |
| NM_153020 | RBM24 | 0.048116897 | 0.398338064 | 1.0987803 | up | mRNA |
| NM_153021 | PLB1 | 0.914112046 | 0.995342392 | 1.0032135 | up | mRNA |
| NM_153022 | C12orf59 | 0.88375031 | 0.992766819 | 1.0249149 | up | mRNA |
| NM_153023 | SPATA13 | 0.405282524 | 0.866165103 | 1.1916728 | up | mRNA |
| NM_153033 | KCTD7 | 0.661236341 | 0.958354006 | 1.0915226 | up | mRNA |
| NM_153042 | KDM1B | 0.876856981 | 0.991255275 | 1.009867 | up | mRNA |
| NM_153047 | FYN | 0.049886568 | 0.404101075 | 1.2460507 | up | mRNA |
| NM_153048 | FYN | 0.003423883 | 0.137823993 | 1.263707 | up | mRNA |
| NM_153184 | CADM2 | 0.418916937 | 0.872328878 | 1.5398875 | up | mRNA |
| NM_153186 | KANK1 | 0.00381279 | 0.142765478 | 1.6462681 | up | mRNA |
| NM_153188 | TNPO1 | 0.969034491 | 0.998196842 | 1.001729 | up | mRNA |
| NM_153208 | IQCK | 0.396576706 | 0.861995469 | 1.1696101 | up | mRNA |
| NM_153218 | LACC1 | 0.211632513 | 0.702997934 | 1.3397399 | up | mRNA |
| NM_153225 | SBSPON | 0.12305404 | 0.575686778 | 1.1746442 | up | mRNA |
| NM_153226 | SLC35G1 | 0.011027465 | 0.215173155 | 2.7400188 | up | mRNA |
| NM_153229 | TMEM92 | 0.609727826 | 0.943053241 | 1.0270862 | up | mRNA |
| NM_153253 | SIPA1 | 0.405220822 | 0.866165103 | 1.1051314 | up | mRNA |
| NM_153254 | TTLL10 | 0.601342274 | 0.941048131 | 1.039584 | up | mRNA |
| NM_153260 | LRRC57 | 0.802684985 | 0.98118927 | 1.0404796 | up | mRNA |
| NM_153265 | EML3 | 0.572378783 | 0.933397932 | 1.0559235 | up | mRNA |
| NM_153271 | SNX33 | 0.021373353 | 0.288424269 | 1.2122782 | up | mRNA |
| NM_153278 | SLC22A6 | 0.698060276 | 0.962430429 | 1.0329688 | up | mRNA |
| NM_153281 | HYAL1 | 0.76832924 | 0.975142015 | 1.0609124 | up | mRNA |
| NM_153324 | DEFB123 | 0.576806794 | 0.933656777 | 1.1471178 | up | mRNA |
| NM_153325 | DEFB125 | 0.096490092 | 0.518318773 | 1.3825993 | up | mRNA |
| NM_153336 | PSTK | 0.884834867 | 0.992981836 | 1.023294 | up | mRNA |
| NM_153341 | RNF19B | 0.003674492 | 0.140696375 | 2.716025 | up | mRNA |
| NM_153345 | TMEM139 | 0.579009747 | 0.933821303 | 1.062364 | up | mRNA |
| NM_153347 | TMEM86A | 0.941752316 | 0.996710991 | 1.0079187 | up | mRNA |
| NM_153350 | FBXL16 | 0.661279575 | 0.958354006 | 1.0216032 | up | mRNA |
| NM_153360 | APCDD1L | 0.695954599 | 0.962410025 | 1.0399051 | up | mRNA |
| NM_153437 | ODF2 | 0.909942079 | 0.995342392 | 1.0070613 | up | mRNA |
| NM_153446 | B4GALNT2 | 0.738870722 | 0.969564098 | 1.0936751 | up | mRNA |
| NM_153450 | MED19 | 0.304086307 | 0.799856028 | 1.072182 | up | mRNA |
| NM_153461 | IL17RC | 0.27494145 | 0.774827385 | 1.12814 | up | mRNA |
| NM_153477 | UXT | 0.697616316 | 0.962410025 | 1.0293871 | up | mRNA |
| NM_153480 | IL17RE | 0.634335356 | 0.951260652 | 1.0824379 | up | mRNA |
| NM_153485 | NUP155 | 0.721731499 | 0.966191059 | 1.0635825 | up | mRNA |
| NM_153490 | KRT13 | 0.330764908 | 0.821853462 | 1.1523155 | up | mRNA |
| NM_153497 | TAB1 | 0.072092949 | 0.462687451 | 1.1950398 | up | mRNA |
| NM_153499 | CAMKK2 | 0.323321012 | 0.816114734 | 1.1650035 | up | mRNA |
| NM_153500 | CAMKK2 | 0.892588857 | 0.993978977 | 1.021482 | up | mRNA |
| NM_153610 | CMYA5 | 0.08229348 | 0.48981002 | 1.257447 | up | mRNA |
| NM_153613 | LPCAT4 | 0.768661043 | 0.975191334 | 1.0216638 | up | mRNA |
| NM_153620 | HOXA1 | 0.809587361 | 0.98118927 | 1.0261412 | up | mRNA |
| NM_153634 | CPNE8 | 0.409765582 | 0.86801103 | 1.5553117 | up | mRNA |
| NM_153636 | CPNE7 | 0.500603292 | 0.909680616 | 1.0612874 | up | mRNA |
| NM_153646 | SLC24A4 | 0.013240523 | 0.234704726 | 1.1721333 | up | mRNA |
| NM_153647 | SLC24A4 | 0.589490095 | 0.936090876 | 1.086007 | up | mRNA |
| NM_153681 | PIGP | 0.643734369 | 0.954605197 | 1.041401 | up | mRNA |
| NM_153682 | PIGP | 0.559476189 | 0.927203296 | 1.0928796 | up | mRNA |
| NM_153685 | C12orf53 | 0.047426983 | 0.397265185 | 1.280125 | up | mRNA |
| NM_153690 | FAM43A | 0.599356261 | 0.940648177 | 1.1848898 | up | mRNA |
| NM_153695 | ZNF367 | 0.979977614 | 0.998971807 | 1.0053896 | up | mRNA |
| NM_153697 | ANKRD44 | 0.999558406 | 0.999940005 | 1.000105 | up | mRNA |
| NM_153705 | KDELC2 | 0.967154007 | 0.99793927 | 1.0057383 | up | mRNA |
| NM_153706 | SETD9 | 0.987871747 | 0.999140676 | 1.0046128 | up | mRNA |
| NM_153712 | TTL | 0.508526711 | 0.911674633 | 1.0707916 | up | mRNA |
| NM_153719 | NUP62 | 0.904661747 | 0.99508468 | 1.0372852 | up | mRNA |
| NM_153746 | ZDHHC14 | 0.523648342 | 0.917239107 | 1.0579128 | up | mRNA |
| NM_153758 | IL19 | 0.607245145 | 0.942627675 | 1.1016787 | up | mRNA |
| NM_153769 | CABYR | 0.525507543 | 0.917686109 | 1.2299293 | up | mRNA |
| NM_153770 | CABYR | 0.712438342 | 0.966072854 | 1.1480534 | up | mRNA |
| NM_153813 | ZFPM1 | 0.487229636 | 0.903705199 | 1.1152678 | up | mRNA |
| NM_153823 | GSG1 | 0.265467162 | 0.766537754 | 1.4816286 | up | mRNA |
| NM_153827 | MINK1 | 0.389340129 | 0.858189054 | 1.0735488 | up | mRNA |
| NM_153831 | PTK2 | 0.018628645 | 0.273264013 | 1.2110835 | up | mRNA |
| NM_153832 | GPR161 | 0.585364845 | 0.934708084 | 1.0403539 | up | mRNA |
| NM_170601 | SIAE | 0.728380258 | 0.966191059 | 1.1284829 | up | mRNA |
| NM_170604 | RASGRP4 | 0.712181554 | 0.966046791 | 1.0252068 | up | mRNA |
| NM_170607 | MLX | 0.522541918 | 0.917200998 | 1.1099932 | up | mRNA |
| NM_170609 | CRISP1 | 0.128117805 | 0.581578746 | 1.5883619 | up | mRNA |
| NM_170679 | SKP1 | 0.914419909 | 0.995342392 | 1.0102439 | up | mRNA |
| NM_170681 | GFM2 | 0.52801437 | 0.918844141 | 1.1018898 | up | mRNA |
| NM_170683 | P2RX2 | 0.843087837 | 0.987338076 | 1.03804 | up | mRNA |
| NM_170714 | RASSF1 | 0.433590377 | 0.880871258 | 1.1236492 | up | mRNA |
| NM_170736 | KCNJ15 | 0.088518843 | 0.502587299 | 2.3295649 | up | mRNA |
| NM_170738 | MRPL11 | 0.804965129 | 0.98118927 | 1.0201818 | up | mRNA |
| NM_170744 | UNC5B | 0.244648107 | 0.743303244 | 1.322539 | up | mRNA |
| NM_170746 | C11orf31 | 0.221535697 | 0.716734257 | 1.068282 | up | mRNA |
| NM_170750 | PSMD10 | 0.853439224 | 0.987678166 | 1.0083368 | up | mRNA |
| NM_170783 | ZNRD1 | 0.094673719 | 0.516031114 | 1.096271 | up | mRNA |
| NM_171825 | CAMK2A | 0.020356166 | 0.285032405 | 1.4654782 | up | mRNA |
| NM_171827 | CD8A | 0.263979151 | 0.764561839 | 1.155982 | up | mRNA |
| NM_171830 | KCNMB3 | 0.441420112 | 0.883920737 | 1.0938117 | up | mRNA |
| NM_171997 | USP2 | 0.523229333 | 0.917239107 | 1.0935132 | up | mRNA |
| NM_171999 | SALL3 | 0.355361147 | 0.838369858 | 1.1201566 | up | mRNA |
| NM_172016 | TRIM39 | 0.066831803 | 0.450987126 | 1.1715835 | up | mRNA |
| NM_172027 | ABTB1 | 0.757498552 | 0.972045462 | 1.0603746 | up | mRNA |
| NM_172037 | RDH10 | 0.849368097 | 0.987678166 | 1.0464916 | up | mRNA |
| NM_172056 | KCNH2 | 0.64244436 | 0.954350564 | 1.0388236 | up | mRNA |
| NM_172057 | KCNH2 | 0.319753773 | 0.813912372 | 1.1706977 | up | mRNA |
| NM_172080 | CAMK2B | 0.468157711 | 0.897203143 | 1.1234895 | up | mRNA |
| NM_172082 | CAMK2B | 0.948960494 | 0.996710991 | 1.0124548 | up | mRNA |
| NM_172087 | TNFSF13 | 0.055571947 | 0.420971993 | 1.2848575 | up | mRNA |
| NM_172088 | TNFSF13 | 0.946543087 | 0.996710991 | 1.0073768 | up | mRNA |
| NM_172102 | CD8B | 0.284583335 | 0.78179061 | 1.4478836 | up | mRNA |
| NM_172105 | EYA4 | 0.982385126 | 0.998971807 | 1.0016165 | up | mRNA |
| NM_172106 | KCNQ2 | 0.752721329 | 0.970725371 | 1.0248525 | up | mRNA |
| NM_172107 | KCNQ2 | 0.750024223 | 0.970725371 | 1.0269482 | up | mRNA |
| NM_172109 | KCNQ2 | 0.127588386 | 0.580991132 | 1.3359883 | up | mRNA |
| NM_172130 | KCNAB2 | 0.937811694 | 0.996710991 | 1.0119191 | up | mRNA |
| NM_172139 | IL28B | 0.831299639 | 0.985710869 | 1.0352159 | up | mRNA |
| NM_172168 | NOXO1 | 0.922952926 | 0.995349793 | 1.0080853 | up | mRNA |
| NM_172197 | AGER | 0.517434541 | 0.91546111 | 1.066955 | up | mRNA |
| NM_172198 | KCND3 | 0.710248876 | 0.965462762 | 1.1961987 | up | mRNA |
| NM_172200 | IL15RA | 0.368828061 | 0.845559574 | 1.1112922 | up | mRNA |
| NM_172208 | TAPBP | 0.002488247 | 0.118591515 | 1.4514656 | up | mRNA |
| NM_172209 | TAPBP | 0.572126462 | 0.933397932 | 1.0625438 | up | mRNA |
| NM_172210 | CSF1 | 3.44681E-06 | 0.009515876 | 5.1919384 | up | mRNA |
| NM_172211 | CSF1 | 8.83065E-06 | 0.014870836 | 8.227251 | up | mRNA |
| NM_172212 | CSF1 | 0.000107085 | 0.037538791 | 9.7385839 | up | mRNA |
| NM_172215 | CAMKK2 | 0.79597913 | 0.980245165 | 1.0446548 | up | mRNA |
| NM_172216 | CAMKK2 | 0.642238961 | 0.954332328 | 1.087503 | up | mRNA |
| NM_172217 | IL16 | 0.049285382 | 0.402199077 | 1.1152331 | up | mRNA |
| NM_172220 | CSF3 | 0.243989567 | 0.742391426 | 1.2120939 | up | mRNA |
| NM_172231 | SUGP1 | 0.669828384 | 0.959531882 | 1.0948044 | up | mRNA |
| NM_172241 | CTAGE1 | 0.788312911 | 0.977641695 | 1.052768 | up | mRNA |
| NM_172244 | SGCD | 0.749619695 | 0.970725371 | 1.0645414 | up | mRNA |
| NM_172250 | MMAA | 0.599197278 | 0.940648177 | 1.0405503 | up | mRNA |
| NM_172251 | MRPL54 | 0.85902682 | 0.988512246 | 1.0088686 | up | mRNA |
| NM_172312 | SPAG8 | 0.270164239 | 0.770135843 | 1.1640622 | up | mRNA |
| NM_172337 | OTX2 | 0.989265117 | 0.999140676 | 1.0019404 | up | mRNA |
| NM_172347 | KCNG4 | 0.051567296 | 0.41031217 | 1.1379839 | up | mRNA |
| NM_172359 | CD46 | 0.677558251 | 0.959531882 | 1.0643578 | up | mRNA |
| NM_172361 | CD46 | 0.748190988 | 0.970725371 | 1.0211406 | up | mRNA |
| NM_172369 | C1QC | 0.189183358 | 0.674719659 | 1.2586772 | up | mRNA |
| NM_172373 | ELF1 | 0.186599804 | 0.669953595 | 1.0570122 | up | mRNA |
| NM_172387 | NFATC1 | 0.005596506 | 0.169833068 | 1.4083075 | up | mRNA |
| NM_172388 | NFATC1 | 0.006674395 | 0.183552613 | 1.297385 | up | mRNA |
| NM_173059 | ZAN | 0.117387075 | 0.564147103 | 1.2195649 | up | mRNA |
| NM_173060 | CAST | 0.259057156 | 0.759547173 | 1.138706 | up | mRNA |
| NM_173065 | IL28RA | 0.239491744 | 0.73766795 | 1.2020795 | up | mRNA |
| NM_173074 | PIGF | 0.409244836 | 0.867591329 | 1.0287334 | up | mRNA |
| NM_173075 | APBB2 | 0.765005178 | 0.974183719 | 1.0154315 | up | mRNA |
| NM_173080 | SPRR4 | 0.683264153 | 0.960111041 | 1.0644117 | up | mRNA |
| NM_173090 | CAPN3 | 0.63205502 | 0.950683967 | 1.1560655 | up | mRNA |
| NM_173160 | FXYD4 | 0.343578842 | 0.831536584 | 1.1670645 | up | mRNA |
| NM_173167 | UNC45B | 0.599902946 | 0.940648177 | 1.1055901 | up | mRNA |
| NM_173193 | KCNIP2 | 0.518625713 | 0.916145597 | 1.0716846 | up | mRNA |
| NM_173197 | KCNIP2 | 0.600713826 | 0.940757262 | 1.1087758 | up | mRNA |
| NM_173200 | NR4A3 | 0.00853605 | 0.19914837 | 2.8217109 | up | mRNA |
| NM_173204 | IL37 | 0.585054924 | 0.934708084 | 1.1899857 | up | mRNA |
| NM_173207 | TGIF1 | 0.443705749 | 0.884887229 | 1.1401881 | up | mRNA |
| NM_173208 | TGIF1 | 0.035062951 | 0.352396712 | 1.58822 | up | mRNA |
| NM_173344 | ST3GAL1 | 0.040151505 | 0.371915487 | 1.2692756 | up | mRNA |
| NM_173354 | SIK1 | 0.451976492 | 0.888854908 | 1.2093078 | up | mRNA |
| NM_173454 | PDE8A | 0.254143096 | 0.754561542 | 1.1362321 | up | mRNA |
| NM_173470 | MMGT1 | 0.557494226 | 0.926833616 | 1.0471407 | up | mRNA |
| NM_173473 | ANAPC16 | 0.740387843 | 0.969794316 | 1.0352267 | up | mRNA |
| NM_173475 | DCUN1D3 | 0.003728271 | 0.141753453 | 2.0617876 | up | mRNA |
| NM_173478 | CNTD1 | 0.366609888 | 0.845181673 | 1.0958178 | up | mRNA |
| NM_173479 | WDR88 | 0.187938645 | 0.67262252 | 1.3224879 | up | mRNA |
| NM_173484 | KLF17 | 0.546837483 | 0.924803608 | 1.1034587 | up | mRNA |
| NM_173489 | HEATR7B2 | 0.034592089 | 0.35197585 | 1.4444351 | up | mRNA |
| NM_173490 | TMEM171 | 0.402864403 | 0.864786348 | 1.3651516 | up | mRNA |
| NM_173495 | PTCHD1 | 0.238515078 | 0.736444734 | 1.3752197 | up | mRNA |
| NM_173496 | MPP7 | 0.303239934 | 0.798533134 | 1.1638713 | up | mRNA |
| NM_173505 | ANKRD29 | 0.973786572 | 0.998501532 | 1.0055862 | up | mRNA |
| NM_173520 | C9orf62 | 0.200856686 | 0.68900633 | 1.3901014 | up | mRNA |
| NM_173528 | C15orf26 | 0.061296149 | 0.437893235 | 1.1748428 | up | mRNA |
| NM_173535 | CLEC4F | 0.22178043 | 0.716734257 | 1.1666229 | up | mRNA |
| NM_173537 | GTF2IRD2 | 0.034998688 | 0.352396712 | 1.0927525 | up | mRNA |
| NM_173541 | C10orf91 | 0.870373385 | 0.989592579 | 1.076019 | up | mRNA |
| NM_173542 | PLBD2 | 0.857666278 | 0.988512246 | 1.0220985 | up | mRNA |
| NM_173544 | FAM129C | 0.713122443 | 0.966110889 | 1.0932672 | up | mRNA |
| NM_173545 | APLF | 0.1391671 | 0.598648178 | 1.1889959 | up | mRNA |
| NM_173548 | ZNF584 | 0.225770607 | 0.721075711 | 1.1920769 | up | mRNA |
| NM_173552 | C3orf58 | 0.499008581 | 0.909361611 | 1.1932139 | up | mRNA |
| NM_173556 | CCDC83 | 0.187364778 | 0.671419231 | 1.1444787 | up | mRNA |
| NM_173557 | RNF152 | 0.866265587 | 0.989481868 | 1.0151658 | up | mRNA |
| NM_173558 | FGD2 | 0.743333801 | 0.970330526 | 1.006395 | up | mRNA |
| NM_173567 | EPHX4 | 0.624531053 | 0.948341211 | 1.2595379 | up | mRNA |
| NM_173573 | C11orf35 | 0.187024779 | 0.670887051 | 1.2205968 | up | mRNA |
| NM_173582 | PGM2L1 | 0.060897875 | 0.436633496 | 2.0040983 | up | mRNA |
| NM_173607 | FAM177A1 | 0.062914256 | 0.439491843 | 1.1199283 | up | mRNA |
| NM_173611 | FAM98B | 0.256571194 | 0.757240077 | 1.0614309 | up | mRNA |
| NM_173614 | NOMO2 | 0.204320506 | 0.694397489 | 1.0350164 | up | mRNA |
| NM_173618 | INO80E | 0.882903524 | 0.992766819 | 1.0126659 | up | mRNA |
| NM_173655 | EPHA6 | 0.548477158 | 0.92514258 | 1.0872626 | up | mRNA |
| NM_173658 | ZNF660 | 0.713384267 | 0.966110889 | 1.1290884 | up | mRNA |
| NM_173663 | FAM153A | 0.382335548 | 0.853514109 | 1.0541295 | up | mRNA |
| NM_173666 | DTWD2 | 0.414099861 | 0.86961884 | 1.0869033 | up | mRNA |
| NM_173672 | PPIL6 | 0.90802833 | 0.995150496 | 1.0205315 | up | mRNA |
| NM_173676 | PNPLA1 | 0.501717698 | 0.910146701 | 1.2009782 | up | mRNA |
| NM_173680 | ZNF775 | 0.479362442 | 0.901538317 | 1.0551512 | up | mRNA |
| NM_173681 | ATG9B | 0.302609404 | 0.79797107 | 1.0772216 | up | mRNA |
| NM_173685 | NSMCE2 | 0.803262648 | 0.98118927 | 1.0254328 | up | mRNA |
| NM_173690 | SCAI | 0.835991745 | 0.986575022 | 1.0373363 | up | mRNA |
| NM_173694 | ATP11C | 0.126049592 | 0.578441772 | 1.2284329 | up | mRNA |
| NM_173698 | FAM133A | 0.394768953 | 0.861511465 | 1.3322405 | up | mRNA |
| NM_173699 | MAGEB18 | 0.449459212 | 0.888279993 | 1.1914059 | up | mRNA |
| NM_173701 | WARS | 0.750150953 | 0.970725371 | 1.0586238 | up | mRNA |
| NM_173803 | MPV17L | 0.95341555 | 0.996710991 | 1.0103025 | up | mRNA |
| NM_173809 | BLOC1S2 | 0.849113954 | 0.987678166 | 1.0355667 | up | mRNA |
| NM_173829 | SREK1IP1 | 0.934507629 | 0.996710991 | 1.0059559 | up | mRNA |
| NM_173833 | SCARA5 | 0.502020927 | 0.910209514 | 1.242024 | up | mRNA |
| NM_173847 | SPACA3 | 0.246922488 | 0.745152522 | 1.1887249 | up | mRNA |
| NM_173852 | KRTCAP2 | 0.039052238 | 0.368792428 | 1.2238618 | up | mRNA |
| NM_173857 | VN1R4 | 0.769744067 | 0.975286259 | 1.1068105 | up | mRNA |
| NM_173860 | HOXC12 | 0.321553192 | 0.815271211 | 1.1531672 | up | mRNA |
| NM_174858 | AK5 | 0.587658131 | 0.93579682 | 1.2547224 | up | mRNA |
| NM_174872 | P2RX2 | 0.705157301 | 0.96462748 | 1.0688686 | up | mRNA |
| NM_174873 | P2RX2 | 0.609085544 | 0.943053241 | 1.149738 | up | mRNA |
| NM_174881 | CRB3 | 0.204659154 | 0.694679453 | 1.4779856 | up | mRNA |
| NM_174886 | TGIF1 | 0.021813174 | 0.29082325 | 1.5406224 | up | mRNA |
| NM_174887 | IFT20 | 0.087040528 | 0.500185639 | 1.0664198 | up | mRNA |
| NM_174889 | NDUFAF2 | 0.221322257 | 0.716734257 | 1.2720754 | up | mRNA |
| NM_174891 | C14orf79 | 0.274419482 | 0.774561497 | 1.1075361 | up | mRNA |
| NM_174893 | C17orf49 | 0.671140846 | 0.959531882 | 1.1079388 | up | mRNA |
| NM_174896 | C1orf162 | 0.670992007 | 0.959531882 | 1.0794683 | up | mRNA |
| NM_174899 | FBXO36 | 0.413501457 | 0.86961884 | 1.2834841 | up | mRNA |
| NM_174902 | LDLRAD3 | 0.387817075 | 0.857110401 | 1.2269916 | up | mRNA |
| NM_174908 | CCDC50 | 0.453280742 | 0.889563297 | 1.1711389 | up | mRNA |
| NM_174909 | TMEM167A | 0.90894219 | 0.995333785 | 1.0088094 | up | mRNA |
| NM_174912 | FAAH2 | 0.077057586 | 0.477122268 | 1.5528432 | up | mRNA |
| NM_174913 | NOP9 | 0.260928134 | 0.761583524 | 1.3323364 | up | mRNA |
| NM_174926 | TMEM136 | 0.653500215 | 0.956860606 | 1.231959 | up | mRNA |
| NM_174928 | N6AMT2 | 0.84226107 | 0.987123192 | 1.0480833 | up | mRNA |
| NM_174929 | ZMIZ2 | 0.675681457 | 0.959531882 | 1.0619111 | up | mRNA |
| NM_174936 | PCSK9 | 0.262734153 | 0.763125731 | 1.1303149 | up | mRNA |
| NM_174942 | GAS2L3 | 0.470978848 | 0.898643199 | 1.1900025 | up | mRNA |
| NM_174953 | ATP2A3 | 0.870086824 | 0.989592579 | 1.0341404 | up | mRNA |
| NM_174964 | ST3GAL3 | 0.468032837 | 0.89714751 | 1.0514861 | up | mRNA |
| NM_174969 | ST3GAL3 | 0.462257618 | 0.894765983 | 1.0356423 | up | mRNA |
| NM_174971 | ST3GAL3 | 0.973805507 | 0.998501532 | 1.0031324 | up | mRNA |
| NM_174975 | SEC14L3 | 0.68386269 | 0.960111041 | 1.064199 | up | mRNA |
| NM_174977 | SEC14L4 | 0.179297944 | 0.661492613 | 1.1999991 | up | mRNA |
| NM_174983 | MFSD12 | 0.027087115 | 0.320017762 | 1.104289 | up | mRNA |
| NM_175054 | HIST4H4 | 0.923886598 | 0.995456159 | 1.0323888 | up | mRNA |
| NM_175060 | CLEC14A | 0.094521839 | 0.51550913 | 1.2278792 | up | mRNA |
| NM_175064 | SPDYE1 | 0.001263449 | 0.092399617 | 1.8299971 | up | mRNA |
| NM_175077 | SLA2 | 0.878121111 | 0.991445937 | 1.0175105 | up | mRNA |
| NM_175569 | XG | 0.674900906 | 0.959531882 | 1.0544162 | up | mRNA |
| NM_175573 | ADRM1 | 0.914533473 | 0.995342392 | 1.018599 | up | mRNA |
| NM_175575 | WFIKKN2 | 0.576795178 | 0.933656777 | 1.0670661 | up | mRNA |
| NM_175605 | IFT88 | 0.896106639 | 0.99418353 | 1.0116645 | up | mRNA |
| NM_175613 | CNTN4 | 0.251258754 | 0.75104278 | 1.3390765 | up | mRNA |
| NM_175617 | MT1E | 0.103557394 | 0.533123263 | 1.9796627 | up | mRNA |
| NM_175619 | ZAR1 | 0.202269352 | 0.69131473 | 1.3134051 | up | mRNA |
| NM_175623 | RAB3IP | 0.225614305 | 0.720696169 | 1.9774055 | up | mRNA |
| NM_175624 | RAB3IP | 0.207665877 | 0.698556218 | 1.1373008 | up | mRNA |
| NM_175727 | IL5RA | 0.719763893 | 0.966191059 | 1.1920502 | up | mRNA |
| NM_175728 | IL5RA | 0.746740588 | 0.970432947 | 1.0363318 | up | mRNA |
| NM_175732 | PTPMT1 | 0.178116676 | 0.658927996 | 1.0586321 | up | mRNA |
| NM_175733 | SYT9 | 0.87729161 | 0.991280039 | 1.0368964 | up | mRNA |
| NM_175735 | LYG2 | 0.784254582 | 0.977347603 | 1.0309909 | up | mRNA |
| NM_175737 | KLB | 0.360643606 | 0.841780454 | 1.1104927 | up | mRNA |
| NM_175739 | SERPINA9 | 0.018242287 | 0.26991728 | 1.524921 | up | mRNA |
| NM_175767 | IL6ST | 0.190994759 | 0.676402188 | 1.0849646 | up | mRNA |
| NM_175769 | TCF23 | 0.774820213 | 0.97589756 | 1.0547357 | up | mRNA |
| NM_175841 | SMOX | 0.058486273 | 0.429914285 | 2.3572476 | up | mRNA |
| NM_175852 | TXLNA | 0.134448291 | 0.591048927 | 1.1028213 | up | mRNA |
| NM_175856 | CHSY3 | 0.955700839 | 0.996883425 | 1.008321 | up | mRNA |
| NM_175858 | KRTAP11-1 | 0.070379308 | 0.458686035 | 1.5567612 | up | mRNA |
| NM_175866 | UHMK1 | 0.85414728 | 0.98790708 | 1.0317644 | up | mRNA |
| NM_175876 | EXOC8 | 0.802949046 | 0.98118927 | 1.024826 | up | mRNA |
| NM_175881 | ODF3L1 | 0.935897098 | 0.996710991 | 1.0152542 | up | mRNA |
| NM_175907 | ZADH2 | 0.776029202 | 0.976080759 | 1.0336944 | up | mRNA |
| NM_175911 | OR2L13 | 0.976005595 | 0.998501532 | 1.0023808 | up | mRNA |
| NM_175913 | JPH2 | 0.708458103 | 0.965462762 | 1.3094101 | up | mRNA |
| NM_175914 | HNF4A | 0.137430758 | 0.596770033 | 1.1439317 | up | mRNA |
| NM_175924 | ILDR1 | 0.314300411 | 0.809623206 | 1.2432858 | up | mRNA |
| NM_175932 | PSMD13 | 0.86000438 | 0.988781331 | 1.0359868 | up | mRNA |
| NM_176096 | CDK5RAP3 | 0.702134984 | 0.963247543 | 1.0658193 | up | mRNA |
| NM_176783 | PSME1 | 0.179258317 | 0.661466445 | 1.1327643 | up | mRNA |
| NM_176793 | MRPL43 | 0.940047676 | 0.996710991 | 1.0152753 | up | mRNA |
| NM_176801 | ADD1 | 0.438208405 | 0.882682266 | 1.0477426 | up | mRNA |
| NM_176812 | CHMP4B | 0.15330751 | 0.619938007 | 1.1630557 | up | mRNA |
| NM_176818 | GATC | 0.258083483 | 0.758836353 | 1.0529702 | up | mRNA |
| NM_176822 | NLRP14 | 0.359165389 | 0.841017008 | 1.1230249 | up | mRNA |
| NM_176823 | S100A7A | 0.006489867 | 0.182002737 | 1.1341215 | up | mRNA |
| NM_176870 | MT1M | 0.100722695 | 0.527535765 | 1.1884582 | up | mRNA |
| NM_176891 | IFNE | 0.406898607 | 0.867591329 | 1.1541995 | up | mRNA |
| NM_176895 | PPAP2A | 0.466229279 | 0.895886371 | 1.0951147 | up | mRNA |
| NM_177400 | NKX6-2 | 0.407814196 | 0.867591329 | 1.0895357 | up | mRNA |
| NM_177435 | PPARD | 0.002221722 | 0.111580485 | 1.7653174 | up | mRNA |
| NM_177458 | LYNX1 | 0.077087677 | 0.477122268 | 1.0937293 | up | mRNA |
| NM_177476 | LYNX1 | 0.000629685 | 0.075756001 | 1.297625 | up | mRNA |
| NM_177478 | FTMT | 0.221480465 | 0.716734257 | 1.0546395 | up | mRNA |
| NM_177525 | MEST | 0.891551658 | 0.9937521 | 1.0105069 | up | mRNA |
| NM_177529 | SULT1A1 | 0.856440723 | 0.988372845 | 1.0247719 | up | mRNA |
| NM_177533 | NUDT14 | 0.011008703 | 0.215013404 | 1.1505096 | up | mRNA |
| NM_177543 | PPAP2C | 0.829254652 | 0.985532347 | 1.0350934 | up | mRNA |
| NM_177551 | HCAR2 | 0.280851499 | 0.779126326 | 1.1688657 | up | mRNA |
| NM_177559 | CSNK2A1 | 0.217311719 | 0.711339274 | 1.1766173 | up | mRNA |
| NM_177924 | ASAH1 | 0.839129832 | 0.987032229 | 1.0218508 | up | mRNA |
| NM_177925 | H2AFJ | 0.588464443 | 0.93579682 | 1.0844508 | up | mRNA |
| NM_177938 | P4HTM | 0.512364004 | 0.91317044 | 1.0545163 | up | mRNA |
| NM_177967 | UBAC2 | 0.919772193 | 0.995349793 | 1.0086183 | up | mRNA |
| NM_177972 | TUB | 0.636500322 | 0.951995735 | 1.040088 | up | mRNA |
| NM_177991 | DUSP15 | 0.798593107 | 0.980749101 | 1.0936678 | up | mRNA |
| NM_177996 | EPB41L1 | 0.799294096 | 0.980917337 | 1.0722685 | up | mRNA |
| NM_178006 | STARD13 | 0.174988312 | 0.653821873 | 1.199223 | up | mRNA |
| NM_178007 | STARD13 | 0.379457865 | 0.852847753 | 1.2965825 | up | mRNA |
| NM_178009 | DGKH | 0.009320489 | 0.204533521 | 1.4989422 | up | mRNA |
| NM_178010 | SOX5 | 0.642715766 | 0.95447352 | 1.2578781 | up | mRNA |
| NM_178013 | PRIMA1 | 0.416252379 | 0.870736736 | 1.0938456 | up | mRNA |
| NM_178031 | TMEM132A | 0.541547211 | 0.923577434 | 1.0307467 | up | mRNA |
| NM_178039 | ERC1 | 0.08025752 | 0.483876854 | 1.0620766 | up | mRNA |
| NM_178126 | FAM134C | 0.751655612 | 0.970725371 | 1.0341126 | up | mRNA |
| NM_178150 | FBXO18 | 0.157623034 | 0.627038062 | 1.1629715 | up | mRNA |
| NM_178151 | DCX | 0.498609635 | 0.909361611 | 1.0817433 | up | mRNA |
| NM_178161 | PTF1A | 0.10924998 | 0.547380631 | 1.5242185 | up | mRNA |
| NM_178167 | ZNF598 | 0.656486535 | 0.957445617 | 1.0866653 | up | mRNA |
| NM_178168 | OR10A5 | 0.538621874 | 0.923577434 | 1.0765465 | up | mRNA |
| NM_178177 | NMNAT3 | 0.529475249 | 0.91899174 | 1.098457 | up | mRNA |
| NM_178181 | CDCP1 | 0.824613605 | 0.983779567 | 1.0129779 | up | mRNA |
| NM_178191 | ATPIF1 | 0.388793569 | 0.857898453 | 1.0733284 | up | mRNA |
| NM_178229 | IQGAP3 | 0.89557344 | 0.993978977 | 1.0710259 | up | mRNA |
| NM_178273 | PILRA | 0.491806749 | 0.905806259 | 1.1727843 | up | mRNA |
| NM_178324 | SPTLC1 | 0.380738948 | 0.853514109 | 1.1227462 | up | mRNA |
| NM_178331 | GNRH2 | 0.039922773 | 0.371662008 | 1.1511491 | up | mRNA |
| NM_178335 | CCDC50 | 0.303074595 | 0.798533134 | 1.7176235 | up | mRNA |
| NM_178339 | C3orf35 | 0.955721198 | 0.996883425 | 1.0053865 | up | mRNA |
| NM_178423 | HDAC9 | 0.557108173 | 0.926833616 | 1.07853 | up | mRNA |
| NM_178429 | LCE2C | 0.302647918 | 0.79797107 | 1.2720447 | up | mRNA |
| NM_178443 | FERMT3 | 0.732665668 | 0.967703318 | 1.0479708 | up | mRNA |
| NM_178450 | MARCH3 | 0.003492711 | 0.138159138 | 1.4628603 | up | mRNA |
| NM_178456 | C20orf85 | 0.947329554 | 0.996710991 | 1.0033899 | up | mRNA |
| NM_178468 | FAM83C | 0.049585878 | 0.402973698 | 1.1777495 | up | mRNA |
| NM_178493 | NOTUM | 0.146108463 | 0.608122555 | 1.5822897 | up | mRNA |
| NM_178495 | ITPRIPL1 | 0.052727837 | 0.413038171 | 1.1034228 | up | mRNA |
| NM_178497 | C4orf26 | 0.170134961 | 0.646939224 | 1.1620853 | up | mRNA |
| NM_178498 | SLC5A12 | 0.870875517 | 0.989640119 | 1.0154986 | up | mRNA |
| NM_178500 | PHOSPHO1 | 0.625596632 | 0.948851902 | 1.0390885 | up | mRNA |
| NM_178505 | TMEM26 | 0.543379509 | 0.923577434 | 1.2349943 | up | mRNA |
| NM_178507 | OAF | 0.031917202 | 0.342121122 | 1.1108964 | up | mRNA |
| NM_178511 | PRR24 | 0.672752542 | 0.959531882 | 1.0362822 | up | mRNA |
| NM_178520 | TMEM105 | 0.214571735 | 0.707672362 | 1.2340408 | up | mRNA |
| NM_178526 | SLC25A42 | 0.493794544 | 0.906630188 | 1.1372971 | up | mRNA |
| NM_178532 | RNF180 | 0.396025229 | 0.861849835 | 1.3632066 | up | mRNA |
| NM_178543 | ENPP7 | 0.396399203 | 0.861995469 | 1.0469822 | up | mRNA |
| NM_178545 | TMEM52 | 0.056170406 | 0.422065984 | 1.5501961 | up | mRNA |
| NM_178549 | ZNF678 | 0.769242014 | 0.975270932 | 1.0506904 | up | mRNA |
| NM_178553 | C2orf53 | 0.887535491 | 0.993263732 | 1.015493 | up | mRNA |
| NM_178557 | NAT8L | 0.174876759 | 0.653821873 | 1.3166705 | up | mRNA |
| NM_178562 | TSPAN33 | 0.573895566 | 0.933656777 | 1.1065147 | up | mRNA |
| NM_178564 | NRBP2 | 0.220582131 | 0.715799221 | 1.1170798 | up | mRNA |
| NM_178568 | RTN4RL1 | 0.073253452 | 0.4657252 | 1.1925422 | up | mRNA |
| NM_178570 | RTN4RL2 | 0.234082547 | 0.731403918 | 1.1049371 | up | mRNA |
| NM_178580 | HM13 | 0.80992115 | 0.98118927 | 1.0380563 | up | mRNA |
| NM_178584 | SEPT10 | 0.416604764 | 0.870736736 | 1.0638498 | up | mRNA |
| NM_178815 | ARL5B | 0.080090367 | 0.483876854 | 1.6046694 | up | mRNA |
| NM_178817 | MRAP | 0.845609373 | 0.987338076 | 1.0117501 | up | mRNA |
| NM_178820 | FBXO27 | 0.778691254 | 0.976938981 | 1.1187987 | up | mRNA |
| NM_178826 | ANO4 | 0.296217611 | 0.79250767 | 1.4213653 | up | mRNA |
| NM_178832 | MORN4 | 0.981443042 | 0.998971807 | 1.0032571 | up | mRNA |
| NM_178833 | SLC9B2 | 0.566818798 | 0.931401262 | 1.1722188 | up | mRNA |
| NM_178834 | LAYN | 0.795747301 | 0.980137767 | 1.0253524 | up | mRNA |
| NM_178836 | PLD6 | 0.890238411 | 0.9937521 | 1.0184328 | up | mRNA |
| NM_178839 | LRRTM1 | 0.643683072 | 0.954605197 | 1.1168379 | up | mRNA |
| NM_178844 | NLRC3 | 0.946464798 | 0.996710991 | 1.0074209 | up | mRNA |
| NM_178850 | HNF4A | 0.355814566 | 0.838676299 | 1.2128769 | up | mRNA |
| NM_178859 | SLC51B | 0.595368319 | 0.938738167 | 1.131671 | up | mRNA |
| NM_178863 | KCTD13 | 0.392292778 | 0.859493185 | 1.0927046 | up | mRNA |
| NM_178868 | CMTM8 | 0.037564894 | 0.362147666 | 1.320552 | up | mRNA |
| NM_180977 | PPP2R5D | 0.333822469 | 0.824079937 | 1.1160181 | up | mRNA |
| NM_180990 | ZACN | 0.837400606 | 0.986775289 | 1.0205683 | up | mRNA |
| NM_181283 | CMTM1 | 0.556000435 | 0.926833616 | 1.1452824 | up | mRNA |
| NM_181304 | MRPL52 | 0.466588055 | 0.89599965 | 1.1531051 | up | mRNA |
| NM_181335 | ARHGAP8 | 0.931986158 | 0.996710991 | 1.0067419 | up | mRNA |
| NM_181340 | DCAF4 | 0.706772241 | 0.965401045 | 1.0320677 | up | mRNA |
| NM_181349 | SMURF1 | 0.150886567 | 0.616029255 | 1.1448353 | up | mRNA |
| NM_181359 | IL6R | 0.268660667 | 0.76931062 | 1.47683 | up | mRNA |
| NM_181361 | KCNMB2 | 0.48057744 | 0.901538317 | 1.0871174 | up | mRNA |
| NM_181427 | GABPB1 | 0.067511199 | 0.451808679 | 1.3205548 | up | mRNA |
| NM_181449 | CD300E | 0.282751096 | 0.780627498 | 1.1113568 | up | mRNA |
| NM_181453 | GCC2 | 0.65914859 | 0.958354006 | 1.0303996 | up | mRNA |
| NM_181460 | PAX3 | 0.053490244 | 0.416053423 | 1.2038936 | up | mRNA |
| NM_181462 | MRPL55 | 0.859116269 | 0.988512246 | 1.0142939 | up | mRNA |
| NM_181468 | EIF6 | 0.746219266 | 0.970394407 | 1.0306768 | up | mRNA |
| NM_181471 | RFC2 | 0.807974012 | 0.98118927 | 1.0680253 | up | mRNA |
| NM_181472 | CMTM7 | 0.521152278 | 0.916644975 | 1.0654351 | up | mRNA |
| NM_181486 | TBX5 | 0.254683288 | 0.755011807 | 1.0851647 | up | mRNA |
| NM_181500 | CUX1 | 0.378889093 | 0.852482258 | 1.0588701 | up | mRNA |
| NM_181507 | HPS5 | 0.385643581 | 0.85515381 | 1.1486949 | up | mRNA |
| NM_181523 | PIK3R1 | 0.004619146 | 0.155233834 | 1.5701534 | up | mRNA |
| NM_181524 | PIK3R1 | 0.254632418 | 0.755011807 | 1.2951165 | up | mRNA |
| NM_181527 | NAA20 | 0.222438512 | 0.71737156 | 1.147897 | up | mRNA |
| NM_181531 | BTN2A2 | 0.84444547 | 0.987338076 | 1.0251996 | up | mRNA |
| NM_181539 | KRT26 | 0.322089806 | 0.815372756 | 1.3727617 | up | mRNA |
| NM_181602 | KRTAP6-1 | 0.786965227 | 0.977479172 | 1.0356424 | up | mRNA |
| NM_181604 | KRTAP6-2 | 0.270221774 | 0.770135843 | 1.1261214 | up | mRNA |
| NM_181606 | KRTAP7-1 | 0.600573464 | 0.940757262 | 1.1580614 | up | mRNA |
| NM_181608 | KRTAP19-2 | 0.514419076 | 0.913819457 | 1.2463292 | up | mRNA |
| NM_181611 | KRTAP19-5 | 0.22526261 | 0.720245224 | 1.749666 | up | mRNA |
| NM_181615 | KRTAP20-1 | 0.049207829 | 0.402199077 | 1.3789425 | up | mRNA |
| NM_181619 | KRTAP21-1 | 0.34554118 | 0.832533915 | 1.0850801 | up | mRNA |
| NM_181620 | KRTAP22-1 | 0.582527377 | 0.934005566 | 1.1084536 | up | mRNA |
| NM_181641 | CKLF | 0.792160964 | 0.978996327 | 1.0232587 | up | mRNA |
| NM_181642 | SPINT1 | 0.316907916 | 0.811712238 | 1.1944671 | up | mRNA |
| NM_181655 | C17orf58 | 0.650266463 | 0.955894361 | 1.0265437 | up | mRNA |
| NM_181657 | LTB4R | 0.170635454 | 0.647381721 | 1.2104527 | up | mRNA |
| NM_181672 | OGT | 0.509512934 | 0.911674633 | 1.1516695 | up | mRNA |
| NM_181673 | OGT | 0.801793817 | 0.98118927 | 1.0307633 | up | mRNA |
| NM_181688 | KRTAP10-10 | 0.628995223 | 0.950128593 | 1.0863043 | up | mRNA |
| NM_181698 | CCNY | 0.122304636 | 0.57455499 | 1.0433227 | up | mRNA |
| NM_181699 | PPP2R1B | 0.120764999 | 0.572339921 | 1.1304022 | up | mRNA |
| NM_181701 | QSOX2 | 0.935020473 | 0.996710991 | 1.0126304 | up | mRNA |
| NM_181711 | GRASP | 0.714330929 | 0.966110889 | 1.0896104 | up | mRNA |
| NM_181720 | ARHGAP30 | 0.887377312 | 0.993247479 | 1.015878 | up | mRNA |
| NM_181723 | EFHA2 | 0.857284944 | 0.988512246 | 1.027729 | up | mRNA |
| NM_181724 | TMEM119 | 0.19749743 | 0.685827115 | 1.10784 | up | mRNA |
| NM_181726 | ANKRD37 | 0.540960173 | 0.923577434 | 1.0494012 | up | mRNA |
| NM_181744 | OPN5 | 0.234129802 | 0.731403918 | 1.3276934 | up | mRNA |
| NM_181786 | HKR1 | 0.168750826 | 0.644327097 | 1.1707159 | up | mRNA |
| NM_181790 | GPR142 | 0.538063825 | 0.923277381 | 1.0575731 | up | mRNA |
| NM_181809 | BMP8A | 0.821010188 | 0.982756843 | 1.02122 | up | mRNA |
| NM_181829 | NF2 | 0.161734824 | 0.633721912 | 1.0828845 | up | mRNA |
| NM_181839 | PKIA | 0.341629258 | 0.830166855 | 1.2406045 | up | mRNA |
| NM_181842 | ZBTB12 | 0.053822423 | 0.416724103 | 1.3507531 | up | mRNA |
| NM_181844 | BCL6B | 0.910642503 | 0.995342392 | 1.0136712 | up | mRNA |
| NM_181847 | AMIGO2 | 0.027751733 | 0.32381669 | 2.6870981 | up | mRNA |
| NM_181868 | APAF1 | 0.982970336 | 0.998971807 | 1.0035302 | up | mRNA |
| NM_181872 | DMRT2 | 0.872416149 | 0.990534615 | 1.0288439 | up | mRNA |
| NM_181882 | PRX | 0.697522057 | 0.962410025 | 1.0733134 | up | mRNA |
| NM_181897 | PPP2R3A | 0.024287764 | 0.304521009 | 1.3338298 | up | mRNA |
| NM_182470 | PKM | 0.518934906 | 0.916145597 | 1.1541917 | up | mRNA |
| NM_182480 | COQ6 | 0.848883955 | 0.987678166 | 1.0320622 | up | mRNA |
| NM_182487 | OLFML2A | 0.282388831 | 0.780042042 | 1.2609723 | up | mRNA |
| NM_182488 | USP12 | 0.000509015 | 0.068538322 | 1.8207637 | up | mRNA |
| NM_182491 | ZFAND2A | 0.129223997 | 0.583473751 | 1.2763392 | up | mRNA |
| NM_182498 | ZNF428 | 0.984375783 | 0.998971807 | 1.0016818 | up | mRNA |
| NM_182505 | C9orf85 | 0.421845075 | 0.873398499 | 1.0507814 | up | mRNA |
| NM_182509 | ISM2 | 0.525815488 | 0.917868103 | 1.0677083 | up | mRNA |
| NM_182521 | ZSWIM2 | 0.008779067 | 0.202329498 | 1.4954418 | up | mRNA |
| NM_182528 | C1QL2 | 0.131138819 | 0.586131998 | 1.1059684 | up | mRNA |
| NM_182538 | SPNS3 | 0.037291267 | 0.36076463 | 1.2096689 | up | mRNA |
| NM_182547 | TMED4 | 0.149058778 | 0.612750316 | 1.1016535 | up | mRNA |
| NM_182553 | CNIH2 | 0.006394083 | 0.180561804 | 1.904745 | up | mRNA |
| NM_182554 | C10orf53 | 0.529289112 | 0.91899174 | 1.0968563 | up | mRNA |
| NM_182557 | BCL9L | 0.543201411 | 0.923577434 | 1.0528759 | up | mRNA |
| NM_182573 | LYPD5 | 0.044698808 | 0.388217071 | 1.3637637 | up | mRNA |
| NM_182577 | ODF3L2 | 0.089692465 | 0.506107454 | 1.1480563 | up | mRNA |
| NM_182580 | CYB561D1 | 0.427111316 | 0.876731038 | 1.0707839 | up | mRNA |
| NM_182581 | C1orf111 | 0.017551992 | 0.267116092 | 1.1855125 | up | mRNA |
| NM_182595 | POM121L12 | 0.612351566 | 0.944159427 | 1.0717482 | up | mRNA |
| NM_182612 | PDDC1 | 0.889929413 | 0.9937521 | 1.0138144 | up | mRNA |
| NM_182616 | C15orf38 | 0.64214974 | 0.954332328 | 1.0497093 | up | mRNA |
| NM_182620 | SKA2 | 0.707176374 | 0.965401045 | 1.0425202 | up | mRNA |
| NM_182646 | CPEB2 | 0.006811396 | 0.183672804 | 1.3856677 | up | mRNA |
| NM_182659 | UTY | 0.217567403 | 0.711744627 | 1.3255684 | up | mRNA |
| NM_182663 | RASSF5 | 0.268373375 | 0.768988803 | 1.3455559 | up | mRNA |
| NM_182664 | RASSF5 | 0.000849877 | 0.083093876 | 1.5600056 | up | mRNA |
| NM_182666 | UBE2E1 | 0.217598102 | 0.711744627 | 1.1036923 | up | mRNA |
| NM_182676 | PLTP | 0.278614386 | 0.777783404 | 1.1949147 | up | mRNA |
| NM_182685 | EFNA1 | 0.106691758 | 0.541344461 | 1.2953188 | up | mRNA |
| NM_182687 | PKMYT1 | 0.93359466 | 0.996710991 | 1.0135018 | up | mRNA |
| NM_182688 | UBE2G2 | 0.405056683 | 0.866165103 | 1.0905013 | up | mRNA |
| NM_182697 | UBE2H | 0.209011688 | 0.700218428 | 1.1637649 | up | mRNA |
| NM_182705 | FAM101B | 0.999905084 | 0.999954265 | 1.0000275 | up | mRNA |
| NM_182715 | SYPL1 | 0.790795517 | 0.978565927 | 1.0443564 | up | mRNA |
| NM_182728 | SLC7A8 | 0.853390762 | 0.987678166 | 1.061439 | up | mRNA |
| NM_182761 | FAM170A | 0.26556455 | 0.766537754 | 1.4445717 | up | mRNA |
| NM_182763 | MCL1 | 0.247628565 | 0.745868458 | 1.304835 | up | mRNA |
| NM_182764 | ELMO2 | 0.434357026 | 0.880931467 | 1.0552793 | up | mRNA |
| NM_182771 | CREM | 0.895088422 | 0.993978977 | 1.0128157 | up | mRNA |
| NM_182775 | ALS2CL | 0.045230657 | 0.389550017 | 1.2248351 | up | mRNA |
| NM_182789 | PAIP1 | 0.889771017 | 0.9937521 | 1.0170492 | up | mRNA |
| NM_182791 | CCDC33 | 0.573892673 | 0.933656777 | 1.0339638 | up | mRNA |
| NM_182798 | EGFLAM | 0.932147351 | 0.996710991 | 1.012115 | up | mRNA |
| NM_182830 | MDGA2 | 0.63044383 | 0.950410442 | 1.1173239 | up | mRNA |
| NM_182832 | PLAC4 | 0.006224316 | 0.178999708 | 2.0344929 | up | mRNA |
| NM_182838 | SLC35E2 | 0.114041319 | 0.557835051 | 1.1396387 | up | mRNA |
| NM_182847 | ASIC4 | 0.429661815 | 0.878226842 | 1.1299662 | up | mRNA |
| NM_182895 | SCARF2 | 0.404822427 | 0.866165103 | 1.0933354 | up | mRNA |
| NM_182917 | EIF4G1 | 0.491396736 | 0.905806259 | 1.0854801 | up | mRNA |
| NM_182919 | TICAM1 | 0.033966242 | 0.349511805 | 1.3857992 | up | mRNA |
| NM_182962 | BIRC3 | 0.005522188 | 0.169833068 | 8.3671838 | up | mRNA |
| NM_182964 | NAV2 | 0.178534148 | 0.65979922 | 1.5008011 | up | mRNA |
| NM_182966 | NEDD9 | 0.09908407 | 0.523009123 | 1.6960884 | up | mRNA |
| NM_182969 | XRRA1 | 0.05842004 | 0.429582734 | 1.2285161 | up | mRNA |
| NM_182970 | RIMS4 | 0.866891387 | 0.989481868 | 1.0382407 | up | mRNA |
| NM_182976 | ZNF326 | 0.537916245 | 0.923138873 | 1.1013777 | up | mRNA |
| NM_183013 | CREM | 0.551908224 | 0.926316494 | 1.0373821 | up | mRNA |
| NM_183050 | BCKDHB | 0.853390689 | 0.987678166 | 1.0108788 | up | mRNA |
| NM_183065 | TMEM107 | 0.727459731 | 0.966191059 | 1.1078008 | up | mRNA |
| NM_183232 | IKZF3 | 0.086684059 | 0.499950487 | 1.4509693 | up | mRNA |
| NM_183234 | RAB27A | 0.151256662 | 0.616500055 | 1.2405083 | up | mRNA |
| NM_183337 | RGS11 | 0.911813695 | 0.995342392 | 1.0283176 | up | mRNA |
| NM_183360 | DTNB | 0.513919427 | 0.91350662 | 1.0701614 | up | mRNA |
| NM_183375 | PRSS48 | 0.29675336 | 0.793167205 | 1.1983057 | up | mRNA |
| NM_183377 | ASIC2 | 0.690975941 | 0.961105466 | 1.0523448 | up | mRNA |
| NM_183394 | CADPS | 0.942695492 | 0.996710991 | 1.0134713 | up | mRNA |
| NM_183395 | NLRP3 | 0.129408972 | 0.583907255 | 1.1690952 | up | mRNA |
| NM_183416 | KIF1B | 0.03897319 | 0.368388142 | 1.3071391 | up | mRNA |
| NM_183419 | RNF19A | 0.978798783 | 0.998661138 | 1.0044979 | up | mRNA |
| NM_183420 | FBXO25 | 0.200941637 | 0.689078321 | 1.0681269 | up | mRNA |
| NM_183421 | FBXO25 | 0.780210381 | 0.976938981 | 1.0194093 | up | mRNA |
| NM_184085 | TRIM55 | 0.680906551 | 0.960111041 | 1.0400987 | up | mRNA |
| NM_184086 | TRIM55 | 0.931895415 | 0.996710991 | 1.0099172 | up | mRNA |
| NM_184087 | TRIM55 | 0.812618204 | 0.98118927 | 1.0252272 | up | mRNA |
| NM_184231 | NCKIPSD | 0.915968873 | 0.995349793 | 1.0208187 | up | mRNA |
| NM_194250 | ZNF804A | 0.92915626 | 0.996456574 | 1.013778 | up | mRNA |
| NM_194255 | SLC19A1 | 0.09635187 | 0.517986839 | 1.0944424 | up | mRNA |
| NM_194272 | RBPMS2 | 0.307648377 | 0.802348229 | 1.0523279 | up | mRNA |
| NM_194281 | INO80C | 0.268099594 | 0.768907819 | 1.0372821 | up | mRNA |
| NM_194293 | XIRP1 | 0.053439505 | 0.415824909 | 1.4072374 | up | mRNA |
| NM_194296 | SPATA24 | 0.029767925 | 0.334953885 | 1.2161495 | up | mRNA |
| NM_194435 | VIP | 0.089883534 | 0.506516635 | 1.1350573 | up | mRNA |
| NM_194442 | LBR | 0.580646816 | 0.93393768 | 1.0525955 | up | mRNA |
| NM_194447 | CLEC4A | 0.005450845 | 0.168942963 | 1.5906557 | up | mRNA |
| NM_194460 | RNF126 | 0.767014601 | 0.975033576 | 1.0291802 | up | mRNA |
| NM_197948 | CLEC7A | 0.433838831 | 0.880923255 | 1.1816523 | up | mRNA |
| NM_197949 | CLEC7A | 0.171683192 | 0.649425612 | 1.0996189 | up | mRNA |
| NM_197950 | CLEC7A | 0.034398287 | 0.35145024 | 1.2144828 | up | mRNA |
| NM_197958 | LARP6 | 0.480219666 | 0.901538317 | 1.2894625 | up | mRNA |
| NM_197962 | GLRX2 | 0.780331286 | 0.976938981 | 1.0430115 | up | mRNA |
| NM_197966 | BID | 0.012056918 | 0.222675671 | 2.7738965 | up | mRNA |
| NM_197967 | BID | 0.002289302 | 0.113527028 | 2.4897692 | up | mRNA |
| NM_197970 | BOLL | 0.681561049 | 0.960111041 | 1.0341653 | up | mRNA |
| NM_197974 | BTN3A3 | 0.978209845 | 0.998641374 | 1.0027491 | up | mRNA |
| NM_197977 | ZNF189 | 0.215017227 | 0.70866109 | 1.2285042 | up | mRNA |
| NM_198041 | NUDT6 | 0.444308326 | 0.885088246 | 1.0546612 | up | mRNA |
| NM_198045 | ZDHHC16 | 0.843672622 | 0.987338076 | 1.017918 | up | mRNA |
| NM_198057 | TSC22D3 | 0.831184842 | 0.985710869 | 1.020147 | up | mRNA |
| NM_198080 | MSRB3 | 0.965787405 | 0.99793927 | 1.0036693 | up | mRNA |
| NM_198081 | SCML4 | 0.503598802 | 0.910553209 | 1.1188841 | up | mRNA |
| NM_198082 | CCDC57 | 0.855430922 | 0.988178307 | 1.0393206 | up | mRNA |
| NM_198085 | RNF148 | 0.507576564 | 0.911674633 | 1.2913524 | up | mRNA |
| NM_198097 | CCZ1B | 0.290743537 | 0.786822522 | 1.145105 | up | mRNA |
| NM_198098 | AQP1 | 0.692852006 | 0.961584652 | 1.0677879 | up | mRNA |
| NM_198123 | CSMD3 | 0.62371031 | 0.948341211 | 1.1154525 | up | mRNA |
| NM_198124 | CSMD3 | 0.94771005 | 0.996710991 | 1.0124596 | up | mRNA |
| NM_198125 | TYROBP | 0.48128397 | 0.901538317 | 1.5746927 | up | mRNA |
| NM_198129 | LAMA3 | 0.652769537 | 0.956714149 | 1.2359338 | up | mRNA |
| NM_198141 | GANC | 0.259739476 | 0.760388386 | 1.1749537 | up | mRNA |
| NM_198149 | SHISA4 | 0.503362212 | 0.91053927 | 1.0667566 | up | mRNA |
| NM_198150 | ARSK | 0.801055006 | 0.98118927 | 1.0104398 | up | mRNA |
| NM_198151 | HEPACAM2 | 0.405933443 | 0.866541972 | 1.1498874 | up | mRNA |
| NM_198156 | VHL | 0.182982737 | 0.666166668 | 1.1616889 | up | mRNA |
| NM_198174 | GRHL3 | 0.411661347 | 0.869001481 | 1.1103383 | up | mRNA |
| NM_198178 | MITF | 0.952354668 | 0.996710991 | 1.0349726 | up | mRNA |
| NM_198181 | GOLGA6L9 | 0.993828374 | 0.999872237 | 1.0010286 | up | mRNA |
| NM_198182 | GRHL1 | 0.85232679 | 0.987678166 | 1.0182972 | up | mRNA |
| NM_198183 | UBE2L6 | 0.869651717 | 0.989592579 | 1.0221766 | up | mRNA |
| NM_198188 | ASTN2 | 0.654900588 | 0.957205183 | 1.0331409 | up | mRNA |
| NM_198196 | CD96 | 0.131635321 | 0.586803191 | 1.2838299 | up | mRNA |
| NM_198205 | MLX | 0.647926397 | 0.95550188 | 1.0613473 | up | mRNA |
| NM_198207 | CERS1 | 0.864093399 | 0.989401142 | 1.0229524 | up | mRNA |
| NM_198212 | CAV2 | 0.677700744 | 0.959531882 | 1.0209315 | up | mRNA |
| NM_198213 | OASL | 0.699935883 | 0.962991468 | 1.0538434 | up | mRNA |
| NM_198216 | SNRPB | 0.471825384 | 0.898908705 | 1.0390589 | up | mRNA |
| NM_198218 | ING1 | 0.003289737 | 0.135125133 | 1.3071289 | up | mRNA |
| NM_198219 | ING1 | 0.022230654 | 0.293936579 | 1.3522653 | up | mRNA |
| NM_198220 | SNRPB2 | 0.787998049 | 0.97758108 | 1.0367538 | up | mRNA |
| NM_198240 | CLIP1 | 0.952342193 | 0.996710991 | 1.0060521 | up | mRNA |
| NM_198244 | EIF4G1 | 0.67667353 | 0.959531882 | 1.0395667 | up | mRNA |
| NM_198253 | TERT | 0.021699465 | 0.29025889 | 1.0832079 | up | mRNA |
| NM_198265 | SPO11 | 0.449456827 | 0.888279993 | 1.2523915 | up | mRNA |
| NM_198269 | HIPK1 | 0.108713428 | 0.546082252 | 1.0342313 | up | mRNA |
| NM_198270 | NHS | 0.729859544 | 0.966191059 | 1.0949767 | up | mRNA |
| NM_198282 | TMEM173 | 0.001488395 | 0.098573423 | 1.3717726 | up | mRNA |
| NM_198315 | VWA5A | 0.86954236 | 0.989592579 | 1.0205988 | up | mRNA |
| NM_198335 | GANAB | 0.725266546 | 0.966191059 | 1.0465177 | up | mRNA |
| NM_198337 | INSIG1 | 0.726421685 | 0.966191059 | 1.1067303 | up | mRNA |
| NM_198380 | CACNA1G | 0.793293925 | 0.979053303 | 1.0282122 | up | mRNA |
| NM_198382 | CACNA1G | 0.866994726 | 0.989481868 | 1.0212759 | up | mRNA |
| NM_198386 | CACNA1G | 0.576952822 | 0.933656777 | 1.1264678 | up | mRNA |
| NM_198388 | CACNA1G | 0.13428533 | 0.590871682 | 1.2053848 | up | mRNA |
| NM_198389 | PDPN | 0.955722167 | 0.996883425 | 1.0043694 | up | mRNA |
| NM_198390 | CMIP | 0.33825708 | 0.827403747 | 1.0532176 | up | mRNA |
| NM_198392 | TCF21 | 0.510034648 | 0.911891001 | 1.081619 | up | mRNA |
| NM_198395 | G3BP1 | 0.699279979 | 0.962701213 | 1.0672088 | up | mRNA |
| NM_198396 | CACNA1G | 0.960723399 | 0.997798988 | 1.0099991 | up | mRNA |
| NM_198426 | CHMP2A | 0.504496339 | 0.910568383 | 1.0498962 | up | mRNA |
| NM_198427 | BCAN | 0.297848762 | 0.794295449 | 1.2319944 | up | mRNA |
| NM_198443 | NRN1L | 0.582954014 | 0.934269626 | 1.0594476 | up | mRNA |
| NM_198445 | RINL | 0.636111867 | 0.951827088 | 1.0683159 | up | mRNA |
| NM_198446 | C1orf122 | 0.879745553 | 0.992178089 | 1.0119154 | up | mRNA |
| NM_198449 | EMB | 0.606596294 | 0.942193725 | 1.1327339 | up | mRNA |
| NM_198450 | APOOL | 0.006408547 | 0.180700212 | 1.2897243 | up | mRNA |
| NM_198455 | SSPO | 0.091557512 | 0.510712576 | 1.6492354 | up | mRNA |
| NM_198471 | KANK3 | 0.201488469 | 0.689610625 | 1.4756949 | up | mRNA |
| NM_198479 | TPRX1 | 0.221631927 | 0.716734257 | 1.122849 | up | mRNA |
| NM_198492 | CLEC4G | 0.207706058 | 0.698556218 | 1.1833736 | up | mRNA |
| NM_198526 | ZNF710 | 0.024620318 | 0.306728123 | 1.8762551 | up | mRNA |
| NM_198532 | C19orf35 | 0.762222198 | 0.97339948 | 1.0386569 | up | mRNA |
| NM_198544 | APITD1-CORT | 0.968689289 | 0.998196842 | 1.0084822 | up | mRNA |
| NM_198545 | C1orf187 | 0.930950858 | 0.996710991 | 1.0206027 | up | mRNA |
| NM_198557 | RBM43 | 0.076980599 | 0.477122268 | 1.3577169 | up | mRNA |
| NM_198563 | TMEM110 | 0.119633338 | 0.569511831 | 1.1525226 | up | mRNA |
| NM_198564 | DNAH12 | 0.480278254 | 0.901538317 | 1.1496249 | up | mRNA |
| NM_198573 | ENHO | 0.045469474 | 0.3905726 | 1.1729067 | up | mRNA |
| NM_198576 | AGRN | 0.269243677 | 0.76973577 | 1.3740928 | up | mRNA |
| NM_198584 | CA13 | 0.223605434 | 0.717768501 | 1.0780638 | up | mRNA |
| NM_198589 | BSG | 0.124480089 | 0.578441772 | 1.102875 | up | mRNA |
| NM_198591 | BSG | 0.521992571 | 0.916999248 | 1.0192691 | up | mRNA |
| NM_198593 | C1QTNF1 | 0.308332559 | 0.803308251 | 1.1867711 | up | mRNA |
| NM_198594 | C1QTNF1 | 0.002119422 | 0.110028846 | 2.7057485 | up | mRNA |
| NM_198595 | AFAP1 | 0.906163139 | 0.99508468 | 1.0072096 | up | mRNA |
| NM_198682 | GYPE | 0.197622912 | 0.686145671 | 1.2819384 | up | mRNA |
| NM_198689 | KRTAP10-7 | 0.521958487 | 0.916999248 | 1.0318013 | up | mRNA |
| NM_198694 | KRTAP10-5 | 0.60704143 | 0.942525113 | 1.0516853 | up | mRNA |
| NM_198696 | KRTAP10-3 | 0.907676115 | 0.995145546 | 1.0042731 | up | mRNA |
| NM_198700 | CELF1 | 0.395537947 | 0.861590143 | 1.0821571 | up | mRNA |
| NM_198704 | HSD11B1L | 0.85347708 | 0.987678166 | 1.0165108 | up | mRNA |
| NM_198705 | HSD11B1L | 0.181490572 | 0.664894785 | 1.1130967 | up | mRNA |
| NM_198706 | HSD11B1L | 0.747160588 | 0.970502081 | 1.0979231 | up | mRNA |
| NM_198707 | HSD11B1L | 0.526153574 | 0.917975454 | 1.1275341 | up | mRNA |
| NM_198709 | ARSB | 0.749874982 | 0.970725371 | 1.0626205 | up | mRNA |
| NM_198716 | PTGER3 | 0.968813012 | 0.998196842 | 1.0140626 | up | mRNA |
| NM_198719 | PTGER3 | 0.814570667 | 0.981277402 | 1.0456941 | up | mRNA |
| NM_198721 | COL25A1 | 0.980463718 | 0.998971807 | 1.0117504 | up | mRNA |
| NM_198828 | MAST4 | 0.25537493 | 0.756286855 | 1.1783689 | up | mRNA |
| NM_198833 | SERPINB8 | 0.027680301 | 0.32381669 | 1.6924793 | up | mRNA |
| NM_198834 | ACACA | 0.580047659 | 0.933893479 | 1.0861928 | up | mRNA |
| NM_198836 | ACACA | 0.179998287 | 0.662747609 | 1.159054 | up | mRNA |
| NM_198841 | FAM120AOS | 0.744106509 | 0.970330526 | 1.05182 | up | mRNA |
| NM_198846 | SIGLEC6 | 0.087844207 | 0.501417299 | 1.3190008 | up | mRNA |
| NM_198850 | PHLDB3 | 0.284446461 | 0.78179061 | 1.1307941 | up | mRNA |
| NM_198859 | PRICKLE2 | 0.334255126 | 0.824174306 | 1.5139836 | up | mRNA |
| NM_198868 | TBC1D9B | 0.56587941 | 0.930458047 | 1.0712308 | up | mRNA |
| NM_198882 | SYNRG | 0.883714423 | 0.992766819 | 1.0166893 | up | mRNA |
| NM_198892 | BMP2K | 0.465412191 | 0.895416414 | 1.0824422 | up | mRNA |
| NM_198897 | FIBP | 0.542490107 | 0.923577434 | 1.0409591 | up | mRNA |
| NM_198900 | FMNL3 | 0.432321207 | 0.880427272 | 1.2233141 | up | mRNA |
| NM_198903 | GABRG2 | 0.411890219 | 0.869001481 | 1.0868838 | up | mRNA |
| NM_198924 | TRIM73 | 0.880867157 | 0.992287164 | 1.0232158 | up | mRNA |
| NM_198925 | SEMA4B | 0.885441576 | 0.992981836 | 1.0240345 | up | mRNA |
| NM_198947 | FAM111B | 0.914482133 | 0.995342392 | 1.0679547 | up | mRNA |
| NM_198955 | MGAT5B | 0.655478418 | 0.957222998 | 1.0293233 | up | mRNA |
| NM_198966 | PTHLH | 0.024550809 | 0.306334086 | 2.7549517 | up | mRNA |
| NM_198976 | TH1L | 0.232965786 | 0.731100552 | 1.4508901 | up | mRNA |
| NM_198977 | ARHGEF1 | 0.713654909 | 0.966110889 | 1.0233718 | up | mRNA |
| NM_198988 | LENG9 | 0.262793364 | 0.763125731 | 1.2449033 | up | mRNA |
| NM_198992 | SYT10 | 0.432126875 | 0.880427272 | 1.081174 | up | mRNA |
| NM_198999 | SLC26A5 | 0.546958656 | 0.924803608 | 1.0841745 | up | mRNA |
| NM_199001 | C9orf169 | 0.516761075 | 0.91493866 | 1.0697643 | up | mRNA |
| NM_199002 | ARHGEF1 | 0.359797026 | 0.841333311 | 1.1601312 | up | mRNA |
| NM_199004 | ARRB2 | 0.434514839 | 0.880988803 | 1.0454528 | up | mRNA |
| NM_199039 | KLHL5 | 0.29767485 | 0.794023716 | 1.0578773 | up | mRNA |
| NM_199050 | C2CD2 | 0.085940659 | 0.498103045 | 1.4101079 | up | mRNA |
| NM_199054 | MKNK2 | 0.97673065 | 0.998501532 | 1.0066562 | up | mRNA |
| NM_199073 | NDUFAF3 | 0.948468209 | 0.996710991 | 1.0038609 | up | mRNA |
| NM_199076 | CNNM2 | 0.152636348 | 0.618255775 | 1.1430621 | up | mRNA |
| NM_199123 | SETD3 | 0.724670561 | 0.966191059 | 1.0428137 | up | mRNA |
| NM_199124 | C11orf63 | 0.998692837 | 0.999889342 | 1.0002115 | up | mRNA |
| NM_199135 | FOXD4L3 | 0.199105118 | 0.687651648 | 1.9098237 | up | mRNA |
| NM_199161 | SAA1 | 0.439678963 | 0.883054028 | 1.1147669 | up | mRNA |
| NM_199165 | ADSSL1 | 0.768184782 | 0.975142015 | 1.0398357 | up | mRNA |
| NM_199166 | ALAS1 | 0.113601314 | 0.557454702 | 1.2859923 | up | mRNA |
| NM_199169 | PMEPA1 | 0.500002445 | 0.909550837 | 1.0688545 | up | mRNA |
| NM_199170 | PMEPA1 | 0.32305798 | 0.815915892 | 1.1341962 | up | mRNA |
| NM_199183 | PRSS45 | 0.122770306 | 0.574983504 | 1.1311384 | up | mRNA |
| NM_199188 | LARP4 | 0.324072227 | 0.816485317 | 1.0391514 | up | mRNA |
| NM_199192 | BRE | 0.605922753 | 0.94174007 | 1.0276806 | up | mRNA |
| NM_199203 | TMEM189-UBE2V1 | 0.753606587 | 0.970725371 | 1.0172834 | up | mRNA |
| NM_199227 | METAP1D | 0.823046634 | 0.983495984 | 1.0335706 | up | mRNA |
| NM_199242 | UNC13D | 0.891429103 | 0.9937521 | 1.017837 | up | mRNA |
| NM_199245 | VAMP1 | 0.00347594 | 0.138138947 | 1.2916414 | up | mRNA |
| NM_199259 | TPTE | 0.382761544 | 0.853514109 | 1.0833012 | up | mRNA |
| NM_199260 | TPTE | 0.798554405 | 0.980749101 | 1.1818293 | up | mRNA |
| NM_199262 | SP6 | 0.219031898 | 0.713450263 | 1.0754036 | up | mRNA |
| NM_199263 | THSD1 | 0.252302057 | 0.752621656 | 1.4003485 | up | mRNA |
| NM_199282 | ARHGAP27 | 0.117039817 | 0.56398437 | 1.1721081 | up | mRNA |
| NM_199294 | APITD1 | 0.908998957 | 0.995333785 | 1.0204317 | up | mRNA |
| NM_199328 | CLDN8 | 0.753158675 | 0.970725371 | 1.0537879 | up | mRNA |
| NM_199335 | FYB | 0.120790409 | 0.572339921 | 1.0956753 | up | mRNA |
| NM_199337 | TMEM179B | 0.226714221 | 0.722135151 | 1.0961814 | up | mRNA |
| NM_199342 | CCDC23 | 0.707571088 | 0.965401045 | 1.0999145 | up | mRNA |
| NM_199344 | SFT2D2 | 0.607517877 | 0.942627675 | 1.0201788 | up | mRNA |
| NM_199346 | PFN4 | 0.497826505 | 0.909116151 | 1.0808326 | up | mRNA |
| NM_199347 | NEK10 | 0.648434365 | 0.95550188 | 1.2730297 | up | mRNA |
| NM_199352 | SLC22A25 | 0.092568024 | 0.511338764 | 1.3051918 | up | mRNA |
| NM_199367 | SPG7 | 0.182402023 | 0.66575662 | 1.1052184 | up | mRNA |
| NM_199418 | PRCP | 0.0259164 | 0.315604326 | 1.4205915 | up | mRNA |
| NM_199456 | TEPP | 0.798482736 | 0.980749101 | 1.0240724 | up | mRNA |
| NM_199478 | PLP1 | 0.584607248 | 0.93461341 | 1.2816639 | up | mRNA |
| NM_199511 | CCDC80 | 0.659180332 | 0.958354006 | 1.1092243 | up | mRNA |
| NM_201224 | DDX47 | 0.493509932 | 0.906417701 | 1.0635256 | up | mRNA |
| NM_201262 | DNAJC12 | 0.122633506 | 0.574983504 | 1.1278727 | up | mRNA |
| NM_201264 | NRP2 | 0.002057162 | 0.109514633 | 1.453652 | up | mRNA |
| NM_201267 | NRP2 | 0.119497573 | 0.569132032 | 1.3471218 | up | mRNA |
| NM_201279 | NRP2 | 0.002504024 | 0.118675568 | 2.1982189 | up | mRNA |
| NM_201283 | EGFR | 0.176153666 | 0.6551228 | 1.123886 | up | mRNA |
| NM_201284 | EGFR | 0.570688223 | 0.93296476 | 1.5164751 | up | mRNA |
| NM_201348 | PRELP | 0.913108068 | 0.995342392 | 1.0640766 | up | mRNA |
| NM_201397 | GPX1 | 0.047294459 | 0.396911378 | 1.1934937 | up | mRNA |
| NM_201402 | USP17L2 | 0.098992573 | 0.523009123 | 1.2806348 | up | mRNA |
| NM_201413 | APP | 0.783661694 | 0.977275912 | 1.0387626 | up | mRNA |
| NM_201414 | APP | 0.929438053 | 0.996533591 | 1.0118537 | up | mRNA |
| NM_201433 | GAS7 | 0.906350401 | 0.99508468 | 1.0161403 | up | mRNA |
| NM_201439 | PPHLN1 | 0.68505108 | 0.960111041 | 1.045724 | up | mRNA |
| NM_201515 | PPHLN1 | 0.61561015 | 0.945575702 | 1.0328028 | up | mRNA |
| NM_201533 | DGKZ | 0.783761685 | 0.977334718 | 1.0142616 | up | mRNA |
| NM_201548 | CERKL | 0.532667446 | 0.92042723 | 1.1121787 | up | mRNA |
| NM_201550 | LRRC10 | 0.247748995 | 0.745868458 | 1.2000504 | up | mRNA |
| NM_201563 | FCGR2C | 0.707493171 | 0.965401045 | 1.2666687 | up | mRNA |
| NM_201589 | MAFA | 0.983447482 | 0.998971807 | 1.003245 | up | mRNA |
| NM_201596 | CACNB2 | 0.894930559 | 0.993978977 | 1.0266245 | up | mRNA |
| NM_201599 | ZMYM3 | 0.600232739 | 0.940648177 | 1.0658549 | up | mRNA |
| NM_201629 | TJP2 | 0.001887847 | 0.105670067 | 1.6085201 | up | mRNA |
| NM_201630 | LRRN2 | 0.595028455 | 0.938655001 | 1.1001378 | up | mRNA |
| NM_201647 | STAMBP | 0.942109032 | 0.996710991 | 1.0074426 | up | mRNA |
| NM_201649 | SLC6A9 | 0.249691419 | 0.748007356 | 1.1056619 | up | mRNA |
| NM_201651 | SLC28A1 | 0.849759967 | 0.987678166 | 1.0481335 | up | mRNA |
| NM_201995 | SF1 | 0.325576639 | 0.818119039 | 1.1258639 | up | mRNA |
| NM_201998 | SF1 | 0.413835579 | 0.86961884 | 1.0762126 | up | mRNA |
| NM_202000 | ACSM3 | 0.456015091 | 0.891365829 | 1.122405 | up | mRNA |
| NM_203284 | RBPJ | 0.902176892 | 0.995003485 | 1.0058229 | up | mRNA |
| NM_203286 | PVRL1 | 0.875407025 | 0.991094934 | 1.0376994 | up | mRNA |
| NM_203292 | RBBP8 | 0.40885881 | 0.867591329 | 1.1779448 | up | mRNA |
| NM_203297 | TRIM7 | 0.881899673 | 0.992766819 | 1.0269638 | up | mRNA |
| NM_203303 | ZCCHC13 | 0.97851795 | 0.998661138 | 1.004133 | up | mRNA |
| NM_203350 | ZRANB2 | 0.882944487 | 0.992766819 | 1.012139 | up | mRNA |
| NM_203351 | MAP3K3 | 0.176624912 | 0.65602809 | 1.0994471 | up | mRNA |
| NM_203371 | FIBIN | 0.044529849 | 0.387743424 | 1.4713109 | up | mRNA |
| NM_203391 | GK | 0.006557349 | 0.182886161 | 1.25615 | up | mRNA |
| NM_203405 | KRTAP26-1 | 0.72475851 | 0.966191059 | 1.0287182 | up | mRNA |
| NM_203411 | TMEM88 | 0.067618833 | 0.451808679 | 1.1442277 | up | mRNA |
| NM_203417 | RCAN1 | 0.003344157 | 0.136839396 | 1.8925407 | up | mRNA |
| NM_203418 | RCAN1 | 0.000269024 | 0.053149627 | 2.2710556 | up | mRNA |
| NM_203437 | AFTPH | 0.117565183 | 0.564147103 | 1.4970693 | up | mRNA |
| NM_203446 | SYNJ1 | 0.849346337 | 0.987678166 | 1.0286007 | up | mRNA |
| NM_203458 | NOTCH2NL | 0.580123996 | 0.93389399 | 1.1218598 | up | mRNA |
| NM_203471 | LGALS14 | 0.566123263 | 0.930661237 | 1.0551683 | up | mRNA |
| NM_203475 | PORCN | 0.591237995 | 0.937021663 | 1.1345092 | up | mRNA |
| NM_203487 | PCDH9 | 0.483875436 | 0.903204457 | 1.1047737 | up | mRNA |
| NM_205545 | LYPD2 | 0.131002728 | 0.585910135 | 1.2367639 | up | mRNA |
| NM_205548 | FAM151B | 0.066070493 | 0.449693785 | 1.1475615 | up | mRNA |
| NM_205833 | IGSF1 | 0.143592254 | 0.603870418 | 1.0389043 | up | mRNA |
| NM_205834 | LSR | 0.66109448 | 0.958354006 | 1.0338691 | up | mRNA |
| NM_205835 | LSR | 0.83546617 | 0.986575022 | 1.0308942 | up | mRNA |
| NM_205837 | LST1 | 0.051537458 | 0.41031217 | 1.1205492 | up | mRNA |
| NM_205838 | LST1 | 0.696257924 | 0.962410025 | 1.5669147 | up | mRNA |
| NM_205840 | LST1 | 0.120481144 | 0.571691731 | 1.1924716 | up | mRNA |
| NM_205843 | NFIC | 0.357087071 | 0.839540876 | 1.0211319 | up | mRNA |
| NM_205848 | SYT6 | 0.967878631 | 0.99793927 | 1.0037529 | up | mRNA |
| NM_205850 | SLC24A5 | 0.24392427 | 0.742391426 | 1.0785447 | up | mRNA |
| NM_205853 | MUSTN1 | 0.797032004 | 0.980667273 | 1.0157028 | up | mRNA |
| NM_205858 | NMB | 0.282208087 | 0.779872438 | 1.5960795 | up | mRNA |
| NM_206808 | CLYBL | 0.732107209 | 0.967388301 | 1.1301902 | up | mRNA |
| NM_206814 | MOG | 0.14419516 | 0.605238644 | 1.2265264 | up | mRNA |
| NM_206818 | OSCAR | 0.60980028 | 0.943063763 | 1.0813029 | up | mRNA |
| NM_206819 | MYBPC1 | 0.357428026 | 0.839643099 | 1.0726333 | up | mRNA |
| NM_206824 | VKORC1 | 0.516418359 | 0.914697976 | 1.0554913 | up | mRNA |
| NM_206837 | OSCP1 | 0.855516247 | 0.988178307 | 1.0209919 | up | mRNA |
| NM_206839 | MORF4L1 | 0.859327102 | 0.988512246 | 1.0128629 | up | mRNA |
| NM_206853 | QKI | 0.448897747 | 0.888279993 | 1.1606121 | up | mRNA |
| NM_206854 | QKI | 0.490273529 | 0.905264563 | 1.1607223 | up | mRNA |
| NM_206855 | QKI | 0.110450083 | 0.549968727 | 1.7609074 | up | mRNA |
| NM_206899 | OR10P1 | 0.191249626 | 0.676939305 | 1.2113334 | up | mRNA |
| NM_206901 | RTN2 | 0.370247771 | 0.846215286 | 1.1368979 | up | mRNA |
| NM_206909 | PSD3 | 0.195648497 | 0.683572955 | 1.1983289 | up | mRNA |
| NM_206917 | NGFRAP1 | 0.821296227 | 0.982756843 | 1.0293741 | up | mRNA |
| NM_206918 | DEGS2 | 0.018652218 | 0.273264013 | 1.3077806 | up | mRNA |
| NM_206920 | MAMDC4 | 0.678207991 | 0.959724726 | 1.0583631 | up | mRNA |
| NM_206921 | CEP85L | 0.497617495 | 0.908871623 | 1.0492925 | up | mRNA |
| NM_206922 | CRIP3 | 0.200798002 | 0.68900633 | 1.0961209 | up | mRNA |
| NM_206939 | MS4A7 | 0.583014249 | 0.934269626 | 1.3660748 | up | mRNA |
| NM_206956 | PRAME | 0.932415166 | 0.996710991 | 1.0172403 | up | mRNA |
| NM_206961 | LTK | 0.382541454 | 0.853514109 | 1.1351953 | up | mRNA |
| NM_206963 | RARRES1 | 0.001977808 | 0.107570744 | 2.5868376 | up | mRNA |
| NM_206964 | FAM3B | 0.500474764 | 0.909680616 | 1.0914918 | up | mRNA |
| NM_206966 | C5orf46 | 0.381896923 | 0.853514109 | 1.1678718 | up | mRNA |
| NM_206967 | C16orf74 | 0.236219548 | 0.734460585 | 1.129327 | up | mRNA |
| NM_206996 | SPAG17 | 0.664570623 | 0.958354006 | 1.1546426 | up | mRNA |
| NM_207005 | USF1 | 0.718697063 | 0.966191059 | 1.0211961 | up | mRNA |
| NM_207015 | NAALADL2 | 0.827210766 | 0.984825466 | 1.0511125 | up | mRNA |
| NM_207033 | EDN3 | 0.352879964 | 0.836121132 | 1.2397949 | up | mRNA |
| NM_207042 | ENSA | 0.90842369 | 0.995329331 | 1.0156444 | up | mRNA |
| NM_207043 | ENSA | 0.081768714 | 0.488477232 | 1.0714838 | up | mRNA |
| NM_207045 | ENSA | 0.991497474 | 0.999495983 | 1.0026258 | up | mRNA |
| NM_207047 | ENSA | 0.833270009 | 0.986434891 | 1.0226922 | up | mRNA |
| NM_207118 | GTF2H5 | 0.70910137 | 0.965462762 | 1.052113 | up | mRNA |
| NM_207168 | ENSA | 0.812861585 | 0.98120545 | 1.0227295 | up | mRNA |
| NM_207170 | SYF2 | 0.381907518 | 0.853514109 | 1.0581868 | up | mRNA |
| NM_207171 | POGZ | 0.480350645 | 0.901538317 | 1.1635007 | up | mRNA |
| NM_207195 | ADAM15 | 0.515461382 | 0.914319064 | 1.1592933 | up | mRNA |
| NM_207196 | ADAM15 | 0.440325326 | 0.883544565 | 1.1819601 | up | mRNA |
| NM_207305 | FOXD4 | 0.296738333 | 0.793167205 | 1.6326421 | up | mRNA |
| NM_207307 | EFCAB12 | 0.389348743 | 0.858189054 | 1.1279529 | up | mRNA |
| NM_207310 | CCDC74B | 0.543835869 | 0.923577434 | 1.0614463 | up | mRNA |
| NM_207317 | ZNF474 | 0.613580223 | 0.944706254 | 1.1696115 | up | mRNA |
| NM_207321 | C10orf129 | 0.967103709 | 0.99793927 | 1.0060982 | up | mRNA |
| NM_207328 | GPAT2 | 0.291580137 | 0.787986325 | 1.1427518 | up | mRNA |
| NM_207334 | FAM43B | 0.249909978 | 0.748423466 | 1.2724306 | up | mRNA |
| NM_207336 | ZNF467 | 0.333976917 | 0.824079937 | 1.1699044 | up | mRNA |
| NM_207348 | SLC25A34 | 0.123668007 | 0.577726888 | 1.3837876 | up | mRNA |
| NM_207361 | FREM2 | 0.435535336 | 0.881627543 | 1.1200399 | up | mRNA |
| NM_207381 | TNFAIP8L3 | 0.693815116 | 0.961812388 | 1.153135 | up | mRNA |
| NM_207387 | FAM211A | 0.690484374 | 0.961105466 | 1.0379838 | up | mRNA |
| NM_207392 | KRTDAP | 0.63207485 | 0.950683967 | 1.053802 | up | mRNA |
| NM_207413 | FAM150A | 0.275410766 | 0.775184524 | 1.1183118 | up | mRNA |
| NM_207414 | FLJ43860 | 0.664410741 | 0.958354006 | 1.0393393 | up | mRNA |
| NM_207416 | FAM75D3 | 0.008494707 | 0.198979698 | 1.1252555 | up | mRNA |
| NM_207421 | PADI6 | 0.918792716 | 0.995349793 | 1.003584 | up | mRNA |
| NM_207422 | FLJ44635 | 0.723450767 | 0.966191059 | 1.052356 | up | mRNA |
| NM_207426 | FOXI2 | 0.182941107 | 0.666166668 | 1.1213977 | up | mRNA |
| NM_207446 | FAM174B | 0.676244131 | 0.959531882 | 1.030943 | up | mRNA |
| NM_207506 | SAMD12 | 0.594132147 | 0.938369994 | 1.1007209 | up | mRNA |
| NM_207510 | LCNL1 | 0.038279858 | 0.365250856 | 1.1897219 | up | mRNA |
| NM_207519 | ZAP70 | 0.578672818 | 0.933821303 | 1.06902 | up | mRNA |
| NM_207581 | DUOXA2 | 0.369950731 | 0.846215286 | 1.1730029 | up | mRNA |
| NM_207582 | ERVFRD-1 | 0.268097533 | 0.768907819 | 1.0740419 | up | mRNA |
| NM_207585 | IFNAR2 | 0.000571146 | 0.072578428 | 2.49032 | up | mRNA |
| NM_207645 | C11orf87 | 0.71690873 | 0.966191059 | 1.0289734 | up | mRNA |
| NM_207647 | FSD1L | 0.480930248 | 0.901538317 | 1.1055237 | up | mRNA |
| NM_212478 | FN1 | 0.95724301 | 0.996883425 | 1.0116919 | up | mRNA |
| NM_212482 | FN1 | 0.774929204 | 0.97589756 | 1.0347688 | up | mRNA |
| NM_212492 | GPS1 | 0.141546062 | 0.601858131 | 1.1671096 | up | mRNA |
| NM_212535 | PRKCB | 0.057523002 | 0.42700171 | 1.4872465 | up | mRNA |
| NM_212539 | PRKCD | 0.358433254 | 0.840546205 | 1.0648388 | up | mRNA |
| NM_212543 | B4GALT4 | 0.894043601 | 0.993978977 | 1.0424865 | up | mRNA |
| NM_212552 | BOLA3 | 0.860562091 | 0.988975154 | 1.049324 | up | mRNA |
| NM_213560 | PKN1 | 0.000946106 | 0.084526081 | 1.6306227 | up | mRNA |
| NM_213600 | PLA2G4F | 0.430800182 | 0.879381002 | 1.0729155 | up | mRNA |
| NM_213601 | TMED8 | 0.255677069 | 0.75646481 | 1.0452448 | up | mRNA |
| NM_213607 | CCDC103 | 0.516688339 | 0.914937059 | 1.1385503 | up | mRNA |
| NM_213631 | C20orf132 | 0.067524698 | 0.451808679 | 1.1396732 | up | mRNA |
| NM_213645 | WARS | 0.791113926 | 0.978610795 | 1.0481265 | up | mRNA |
| NM_213649 | SFXN4 | 0.928060513 | 0.995953043 | 1.0058353 | up | mRNA |
| NM_213658 | KLRC1 | 0.505763406 | 0.911021812 | 1.2313945 | up | mRNA |
| NM_213720 | CHCHD10 | 0.919855902 | 0.995349793 | 1.0131232 | up | mRNA |
| NM_213725 | RPLP1 | 0.19846371 | 0.686836451 | 1.0440938 | up | mRNA |
| NM_214710 | PRSS57 | 0.446958703 | 0.88691918 | 1.1345292 | up | mRNA |
| NM_214711 | C4orf40 | 0.887596338 | 0.993263732 | 1.0237223 | up | mRNA |
| uc011jsg.2 | HLA-DRB4 | 0.091054556 | 0.509584704 | 1.0978229 | up | mRNA |
| ENST00000013070 | UBR7 | 0.922355347 | 0.995349793 | 1.0143106 | down | mRNA |
| ENST00000020673 | PSD | 0.179993574 | 0.662747609 | 1.1124521 | down | mRNA |
| ENST00000040663 | MRI1 | 0.093034089 | 0.511788177 | 1.3089055 | down | mRNA |
| ENST00000040877 | TARBP1 | 0.508760811 | 0.911674633 | 1.1107633 | down | mRNA |
| ENST00000053243 | TNFRSF17 | 0.304801049 | 0.800466924 | 1.1952532 | down | mRNA |
| ENST00000053867 | GRN | 0.846857306 | 0.987678166 | 1.045715 | down | mRNA |
| ENST00000054666 | VAMP3 | 0.514069809 | 0.91350662 | 1.0421545 | down | mRNA |
| ENST00000072516 | IL1RAP | 0.000175059 | 0.044621794 | 1.9155526 | down | mRNA |
| ENST00000074304 | INPP4A | 0.866715526 | 0.989481868 | 1.021005 | down | mRNA |
| ENST00000156825 | MBD3 | 0.331982697 | 0.822453051 | 1.1101077 | down | mRNA |
| ENST00000158149 | C17orf85 | 0.071188125 | 0.46104346 | 1.3616383 | down | mRNA |
| ENST00000159087 | ANO8 | 0.87691406 | 0.991255275 | 1.0282273 | down | mRNA |
| ENST00000161006 | PRSS22 | 0.712552502 | 0.966110889 | 1.0399666 | down | mRNA |
| ENST00000166139 | FSTL3 | 0.286313563 | 0.782895119 | 1.0455108 | down | mRNA |
| ENST00000193322 | OSTM1 | 0.41829848 | 0.872023448 | 1.1711743 | down | mRNA |
| ENST00000196548 | ZNF8 | 0.1816985 | 0.664924727 | 1.2142606 | down | mRNA |
| ENST00000200181 | ITGB4 | 0.577130812 | 0.933656777 | 1.0669803 | down | mRNA |
| ENST00000201963 | DNMT3B | 0.939147408 | 0.996710991 | 1.0157026 | down | mRNA |
| ENST00000202816 | ESF1 | 0.65054287 | 0.955894361 | 1.0287892 | down | mRNA |
| ENST00000204549 | PDCD7 | 0.001408135 | 0.096194096 | 1.2602208 | down | mRNA |
| ENST00000215376 | C19orf26 | 0.042233084 | 0.379780211 | 1.3402661 | down | mRNA |
| ENST00000215479 | AMELY | 0.164386023 | 0.637590354 | 1.2281548 | down | mRNA |
| ENST00000215582 | C19orf21 | 0.388630823 | 0.857898453 | 1.2455574 | down | mRNA |
| ENST00000215587 | POLR2E | 0.334181855 | 0.824174306 | 1.07212 | down | mRNA |
| ENST00000215591 | POLRMT | 0.02566374 | 0.314576454 | 1.2850717 | down | mRNA |
| ENST00000216180 | PNPLA3 | 0.034845835 | 0.352338753 | 1.799632 | down | mRNA |
| ENST00000216254 | ACO2 | 0.158070932 | 0.628450954 | 1.1351554 | down | mRNA |
| ENST00000216294 | SNAPC1 | 0.928299592 | 0.996053205 | 1.0075467 | down | mRNA |
| ENST00000216297 | SUPT16H | 0.377196394 | 0.850710713 | 1.143201 | down | mRNA |
| ENST00000217428 | SPINT3 | 0.851173406 | 0.987678166 | 1.022409 | down | mRNA |
| ENST00000217429 | FAM83D | 0.183172159 | 0.66623548 | 1.4554382 | down | mRNA |
| ENST00000217652 | MYL12A | 0.954187726 | 0.996710991 | 1.0113635 | down | mRNA |
| ENST00000217901 | IDH3G | 0.593904066 | 0.938299318 | 1.0233079 | down | mRNA |
| ENST00000218249 | RAB40AL | 0.078894436 | 0.481483348 | 1.2020793 | down | mRNA |
| ENST00000218721 | MLNR | 0.071993945 | 0.462605549 | 1.5373267 | down | mRNA |
| ENST00000219097 | ORC6 | 0.624593604 | 0.948341211 | 1.2048628 | down | mRNA |
| ENST00000219313 | PSMD7 | 0.68993447 | 0.961105466 | 1.0328087 | down | mRNA |
| ENST00000219593 | ZP2 | 0.48510695 | 0.903250293 | 1.1854554 | down | mRNA |
| ENST00000219611 | SOLH | 0.289837034 | 0.786462908 | 1.2094131 | down | mRNA |
| ENST00000219905 | MGA | 0.054453328 | 0.418738677 | 1.4459908 | down | mRNA |
| ENST00000220509 | VPS18 | 0.002111837 | 0.110028846 | 1.5132893 | down | mRNA |
| ENST00000220514 | OIP5 | 0.946768681 | 0.996710991 | 1.0436846 | down | mRNA |
| ENST00000220966 | PYCRL | 0.753485059 | 0.970725371 | 1.1238028 | down | mRNA |
| ENST00000221222 | HOMER3 | 0.220633175 | 0.715799221 | 1.1463387 | down | mRNA |
| ENST00000221413 | RUVBL2 | 0.125128894 | 0.578441772 | 1.195302 | down | mRNA |
| ENST00000221421 | LHB | 0.645161931 | 0.954828387 | 1.0380655 | down | mRNA |
| ENST00000221444 | KCNA7 | 0.702861479 | 0.963625105 | 1.0226955 | down | mRNA |
| ENST00000221515 | RETN | 0.571225824 | 0.933029103 | 1.0412425 | down | mRNA |
| ENST00000221566 | SGTA | 0.889051217 | 0.993470148 | 1.0238271 | down | mRNA |
| ENST00000221671 | C19orf44 | 0.725309113 | 0.966191059 | 1.0761141 | down | mRNA |
| ENST00000221770 | POP4 | 0.858093499 | 0.988512246 | 1.0215773 | down | mRNA |
| ENST00000221957 | PLIN3 | 0.092600002 | 0.511338764 | 1.2247568 | down | mRNA |
| ENST00000222286 | GAPDHS | 0.41676277 | 0.870736736 | 1.0909804 | down | mRNA |
| ENST00000222305 | USF2 | 0.578666196 | 0.933821303 | 1.0524915 | down | mRNA |
| ENST00000222308 | FKBP8 | 0.508955909 | 0.911674633 | 1.2213569 | down | mRNA |
| ENST00000222329 | ERF | 0.006764205 | 0.183552613 | 1.3611516 | down | mRNA |
| ENST00000222574 | HBP1 | 0.724846445 | 0.966191059 | 1.0333409 | down | mRNA |
| ENST00000222990 | SNX8 | 0.452474526 | 0.889032863 | 1.1144338 | down | mRNA |
| ENST00000223051 | TFR2 | 0.632698447 | 0.951123179 | 1.1787394 | down | mRNA |
| ENST00000223095 | SERPINE1 | 0.130029931 | 0.584037049 | 1.3723882 | down | mRNA |
| ENST00000223398 | CLIP2 | 0.796706391 | 0.980665598 | 1.0524411 | down | mRNA |
| ENST00000223428 | ZNF510 | 0.418617447 | 0.872328878 | 1.0968078 | down | mRNA |
| ENST00000223528 | FKTN | 0.190018987 | 0.676022054 | 1.1048246 | down | mRNA |
| ENST00000224073 | EDF1 | 0.949218536 | 0.996710991 | 1.0037672 | down | mRNA |
| ENST00000224862 | FBXL15 | 0.981015901 | 0.998971807 | 1.0010259 | down | mRNA |
| ENST00000225296 | DHX33 | 0.001255557 | 0.092399617 | 1.274365 | down | mRNA |
| ENST00000225519 | SHPK | 0.599736072 | 0.940648177 | 1.1594232 | down | mRNA |
| ENST00000225614 | GALK1 | 0.397711935 | 0.863112281 | 1.0607963 | down | mRNA |
| ENST00000225983 | HDAC5 | 0.375885787 | 0.849862269 | 1.2344534 | down | mRNA |
| ENST00000225992 | PPY | 0.905092831 | 0.99508468 | 1.0229297 | down | mRNA |
| ENST00000226319 | PHF17 | 0.006416759 | 0.180700212 | 1.6132977 | down | mRNA |
| ENST00000229264 | GNB3 | 0.61236801 | 0.944159427 | 1.0297373 | down | mRNA |
| ENST00000229277 | ENO2 | 0.876220108 | 0.991213905 | 1.0330342 | down | mRNA |
| ENST00000229340 | RAB35 | 0.721894478 | 0.966191059 | 1.0395967 | down | mRNA |
| ENST00000229812 | STK38 | 0.047472927 | 0.397373218 | 1.3246574 | down | mRNA |
| ENST00000229971 | FBXL4 | 0.265790587 | 0.766569994 | 1.1999192 | down | mRNA |
| ENST00000230859 | PAPD7 | 0.010379208 | 0.209736088 | 1.4067756 | down | mRNA |
| ENST00000231121 | HAND1 | 0.818811928 | 0.982093332 | 1.0171716 | down | mRNA |
| ENST00000231130 | PCDHB3 | 0.955065014 | 0.996883425 | 1.0737047 | down | mRNA |
| ENST00000231229 | BTNL8 | 0.715887801 | 0.966191059 | 1.0557738 | down | mRNA |
| ENST00000231357 | IRX4 | 0.879577232 | 0.992178089 | 1.0218772 | down | mRNA |
| ENST00000233331 | INO80B | 0.328771315 | 0.821644684 | 1.0918394 | down | mRNA |
| ENST00000233609 | RPS15 | 0.500257346 | 0.909680616 | 1.1168902 | down | mRNA |
| ENST00000233616 | MOGS | 0.971951189 | 0.998501532 | 1.0104076 | down | mRNA |
| ENST00000233892 | MOB4 | 0.010449923 | 0.209736088 | 1.1967706 | down | mRNA |
| ENST00000233946 | IL1R1 | 0.291599897 | 0.787986325 | 1.1270228 | down | mRNA |
| ENST00000234040 | PASK | 0.734781739 | 0.967777568 | 1.1412044 | down | mRNA |
| ENST00000234827 | TCEANC2 | 0.233846896 | 0.73118887 | 1.5480338 | down | mRNA |
| ENST00000234831 | TMEM59 | 0.630827975 | 0.950410442 | 1.1388185 | down | mRNA |
| ENST00000236671 | CTSD | 0.705358907 | 0.964773448 | 1.0761459 | down | mRNA |
| ENST00000237201 | SPACA1 | 0.439595996 | 0.883054028 | 1.0959005 | down | mRNA |
| ENST00000237853 | ELL2 | 0.822778663 | 0.983305175 | 1.0141673 | down | mRNA |
| ENST00000238256 | FKBP15 | 0.003146819 | 0.132624561 | 1.1445318 | down | mRNA |
| ENST00000238341 | CNTRL | 0.129854945 | 0.583907255 | 1.1701135 | down | mRNA |
| ENST00000238936 | TMEM180 | 0.618336381 | 0.946956498 | 1.0706639 | down | mRNA |
| ENST00000239032 | PRLHR | 0.842089279 | 0.987123192 | 1.0428802 | down | mRNA |
| ENST00000239125 | C10orf95 | 0.951698983 | 0.996710991 | 1.0236225 | down | mRNA |
| ENST00000241463 | RASL11A | 0.561981059 | 0.928808234 | 1.0930665 | down | mRNA |
| ENST00000242257 | FTSJ2 | 0.005326096 | 0.167114495 | 1.4392277 | down | mRNA |
| ENST00000242770 | STX10 | 0.142087918 | 0.60204132 | 1.1038578 | down | mRNA |
| ENST00000244137 | PEPD | 0.108802833 | 0.546082252 | 1.1725082 | down | mRNA |
| ENST00000244204 | NAGK | 0.244112225 | 0.742391426 | 1.3879783 | down | mRNA |
| ENST00000244296 | PSG1 | 0.751271456 | 0.970725371 | 1.13726 | down | mRNA |
| ENST00000245543 | ARMC7 | 0.502080205 | 0.910235821 | 1.0880795 | down | mRNA |
| ENST00000245812 | ALKBH7 | 0.899285944 | 0.994575817 | 1.0326859 | down | mRNA |
| ENST00000245816 | CLPP | 0.300126935 | 0.795797471 | 1.0869872 | down | mRNA |
| ENST00000246794 | PRRG2 | 0.22356132 | 0.717768501 | 1.1344554 | down | mRNA |
| ENST00000246957 | TRAP1 | 0.381713288 | 0.853514109 | 1.1371214 | down | mRNA |
| ENST00000247003 | DDX49 | 0.767423281 | 0.975033576 | 1.0458468 | down | mRNA |
| ENST00000247883 | TAS2R5 | 0.375206462 | 0.849710156 | 1.5151075 | down | mRNA |
| ENST00000247992 | PLA2G2C | 0.628741616 | 0.950128593 | 1.0730342 | down | mRNA |
| ENST00000248121 | SYNGR3 | 0.599923942 | 0.940648177 | 1.0440332 | down | mRNA |
| ENST00000249064 | CCDC117 | 0.215547715 | 0.709834167 | 1.2481104 | down | mRNA |
| ENST00000249714 | SERINC4 | 0.910760482 | 0.995342392 | 1.0143173 | down | mRNA |
| ENST00000249760 | IVD | 0.047070263 | 0.395610183 | 1.3692167 | down | mRNA |
| ENST00000249806 | CLN6 | 0.437255496 | 0.882234667 | 1.0970188 | down | mRNA |
| ENST00000249910 | MBD4 | 0.001642306 | 0.104022951 | 1.5302556 | down | mRNA |
| ENST00000250241 | ILF3 | 0.037628782 | 0.362248289 | 1.1211329 | down | mRNA |
| ENST00000250263 | ERI1 | 0.458177399 | 0.892646883 | 1.1906556 | down | mRNA |
| ENST00000250699 | CHRNA10 | 0.493582105 | 0.906462119 | 1.0948186 | down | mRNA |
| ENST00000251038 | ZC3H14 | 0.112220051 | 0.554473407 | 1.2524917 | down | mRNA |
| ENST00000251269 | ZNF221 | 0.216880058 | 0.711074992 | 1.1671804 | down | mRNA |
| ENST00000251289 | WDR18 | 0.156989423 | 0.625751326 | 1.0700002 | down | mRNA |
| ENST00000251535 | ALOX12 | 0.521599793 | 0.916644975 | 1.0722242 | down | mRNA |
| ENST00000251582 | ADAMTS2 | 0.530056515 | 0.91899174 | 1.0714615 | down | mRNA |
| ENST00000251595 | HBA2 | 0.930168269 | 0.996649933 | 1.0086008 | down | mRNA |
| ENST00000251646 | KRT33B | 0.204082306 | 0.694228116 | 1.2338867 | down | mRNA |
| ENST00000251864 | TDRD1 | 0.039472724 | 0.370322288 | 1.3332029 | down | mRNA |
| ENST00000252087 | PCDHGC5 | 0.885296146 | 0.992981836 | 1.0155519 | down | mRNA |
| ENST00000252445 | ELOF1 | 0.23070044 | 0.728071705 | 1.0837012 | down | mRNA |
| ENST00000252456 | CNN1 | 0.779532524 | 0.976938981 | 1.0956456 | down | mRNA |
| ENST00000252483 | PVRL2 | 0.3242356 | 0.816593362 | 1.1939346 | down | mRNA |
| ENST00000252602 | MRPL34 | 0.19404236 | 0.681590151 | 1.0378312 | down | mRNA |
| ENST00000252711 | NDUFA10 | 0.648865145 | 0.95550188 | 1.0699275 | down | mRNA |
| ENST00000252809 | GDF15 | 0.902031205 | 0.994906068 | 1.0522834 | down | mRNA |
| ENST00000252992 | CEP85 | 0.165532612 | 0.639363423 | 2.2105247 | down | mRNA |
| ENST00000253024 | TRIM28 | 0.97331384 | 0.998501532 | 1.0069726 | down | mRNA |
| ENST00000253108 | EIF3G | 0.306695873 | 0.801715156 | 1.0574311 | down | mRNA |
| ENST00000253237 | GRWD1 | 0.175540168 | 0.653962789 | 1.1071114 | down | mRNA |
| ENST00000253699 | ZFYVE20 | 0.315486157 | 0.810346887 | 1.2003153 | down | mRNA |
| ENST00000253812 | PCDHGA3 | 0.567956359 | 0.931705674 | 1.0286407 | down | mRNA |
| ENST00000254190 | CHSY1 | 0.000838622 | 0.083093876 | 1.7761099 | down | mRNA |
| ENST00000254321 | ZNF700 | 0.022586053 | 0.295888941 | 1.4126712 | down | mRNA |
| ENST00000254322 | DNAJB1 | 0.174380719 | 0.652930084 | 1.2704852 | down | mRNA |
| ENST00000254630 | PTCD3 | 0.04317582 | 0.383508423 | 1.2440697 | down | mRNA |
| ENST00000254770 | LANCL2 | 0.660681172 | 0.958354006 | 1.1505716 | down | mRNA |
| ENST00000255882 | PI4KA | 0.711315521 | 0.965774102 | 1.0486391 | down | mRNA |
| ENST00000256190 | SBF2 | 0.009291808 | 0.204533521 | 1.2301544 | down | mRNA |
| ENST00000256367 | TTC9 | 0.835106479 | 0.986575022 | 1.1433467 | down | mRNA |
| ENST00000256997 | ACP2 | 9.99974E-05 | 0.036966313 | 1.0465981 | down | mRNA |
| ENST00000256999 | FOLH1 | 0.440321595 | 0.883544565 | 1.2430556 | down | mRNA |
| ENST00000257527 | ADAM19 | 0.230253691 | 0.727508632 | 1.3665412 | down | mRNA |
| ENST00000257700 | RINT1 | 0.014733488 | 0.24380986 | 1.2013982 | down | mRNA |
| ENST00000258083 | PRADC1 | 0.602152032 | 0.941406011 | 1.0685091 | down | mRNA |
| ENST00000258098 | RAB11FIP5 | 0.233083024 | 0.731100552 | 1.255436 | down | mRNA |
| ENST00000258229 | PCNXL2 | 0.061589451 | 0.437989276 | 1.2581205 | down | mRNA |
| ENST00000258243 | URB2 | 0.200238074 | 0.68900633 | 1.1369933 | down | mRNA |
| ENST00000258383 | MRPL44 | 0.014791884 | 0.244114105 | 1.1988845 | down | mRNA |
| ENST00000258399 | USP37 | 0.087431871 | 0.500769598 | 1.2539254 | down | mRNA |
| ENST00000258947 | CALCOCO2 | 0.886249668 | 0.992981836 | 1.0182724 | down | mRNA |
| ENST00000258975 | TACO1 | 0.065819108 | 0.448620218 | 1.1757895 | down | mRNA |
| ENST00000259339 | TOR1B | 0.31627594 | 0.810808526 | 1.0993396 | down | mRNA |
| ENST00000259727 | GMPR | 0.931624618 | 0.996710991 | 1.0154831 | down | mRNA |
| ENST00000259806 | FOXF2 | 0.615054586 | 0.94515077 | 1.0784723 | down | mRNA |
| ENST00000259953 | NRM | 0.016836069 | 0.260510618 | 1.053568 | down | mRNA |
| ENST00000260008 | FHDC1 | 0.653408935 | 0.956860606 | 1.0898693 | down | mRNA |
| ENST00000260453 | MNS1 | 0.708440442 | 0.965462762 | 1.1317418 | down | mRNA |
| ENST00000260605 | DYNC2LI1 | 0.571507061 | 0.933223365 | 1.0627281 | down | mRNA |
| ENST00000261015 | WDR12 | 0.102092681 | 0.530503273 | 1.1077355 | down | mRNA |
| ENST00000261296 | TGDS | 0.013546468 | 0.235810597 | 1.2664312 | down | mRNA |
| ENST00000261332 | ZNF24 | 0.408782244 | 0.867591329 | 1.1084673 | down | mRNA |
| ENST00000261416 | HEXB | 0.734018457 | 0.967777568 | 1.0855757 | down | mRNA |
| ENST00000261520 | NARG2 | 0.191636692 | 0.677509517 | 1.1169671 | down | mRNA |
| ENST00000261560 | ZNF430 | 0.1712172 | 0.648628305 | 1.2287981 | down | mRNA |
| ENST00000261593 | RNF138 | 0.469394406 | 0.897810636 | 1.1585923 | down | mRNA |
| ENST00000261596 | LPIN2 | 0.221995972 | 0.716912415 | 1.1191819 | down | mRNA |
| ENST00000261597 | NDC80 | 0.805880367 | 0.98118927 | 1.1611223 | down | mRNA |
| ENST00000261749 | ZFAND6 | 0.526576172 | 0.917975454 | 1.0934101 | down | mRNA |
| ENST00000261797 | NDST1 | 0.925289223 | 0.995465766 | 1.0103988 | down | mRNA |
| ENST00000261844 | FAM214A | 0.571770766 | 0.933299883 | 1.1210721 | down | mRNA |
| ENST00000261884 | TRIP4 | 0.430258266 | 0.87878612 | 1.0858757 | down | mRNA |
| ENST00000261892 | SLC24A1 | 0.605098339 | 0.94174007 | 1.161436 | down | mRNA |
| ENST00000261893 | LACTB | 0.640420117 | 0.953894897 | 1.2193829 | down | mRNA |
| ENST00000261908 | NEO1 | 0.957398375 | 0.996883425 | 1.0120523 | down | mRNA |
| ENST00000261917 | HCN4 | 0.081781256 | 0.488477232 | 1.4414874 | down | mRNA |
| ENST00000261951 | CNOT6 | 0.054005111 | 0.417146607 | 1.1694042 | down | mRNA |
| ENST00000262061 | COPZ1 | 0.978062008 | 0.998641374 | 1.0017621 | down | mRNA |
| ENST00000262187 | RHEB | 0.534360905 | 0.920652989 | 1.0322228 | down | mRNA |
| ENST00000262189 | MLL3 | 0.086501973 | 0.49936346 | 1.168544 | down | mRNA |
| ENST00000262215 | ARFGEF1 | 0.889908714 | 0.9937521 | 1.0103026 | down | mRNA |
| ENST00000262304 | PKD1 | 0.461049489 | 0.894556562 | 1.0980477 | down | mRNA |
| ENST00000262345 | IL12RB2 | 0.893960685 | 0.993978977 | 1.0191816 | down | mRNA |
| ENST00000262384 | N4BP1 | 0.030507943 | 0.338388833 | 1.2080257 | down | mRNA |
| ENST00000262415 | DHX8 | 0.07955658 | 0.482639336 | 1.134717 | down | mRNA |
| ENST00000262432 | METTL2B | 0.212208848 | 0.703969702 | 1.0916565 | down | mRNA |
| ENST00000262487 | ISM1 | 0.95295007 | 0.996710991 | 1.0572618 | down | mRNA |
| ENST00000262518 | SRCAP | 0.714659178 | 0.966110889 | 1.0371079 | down | mRNA |
| ENST00000262625 | KIRREL2 | 0.558210793 | 0.926833616 | 1.0591273 | down | mRNA |
| ENST00000262738 | CELSR1 | 0.30032665 | 0.795797471 | 1.0159494 | down | mRNA |
| ENST00000262795 | SHANK3 | 0.217333456 | 0.711339274 | 1.2336613 | down | mRNA |
| ENST00000262811 | MAST3 | 0.004695906 | 0.15714472 | 1.2878122 | down | mRNA |
| ENST00000262812 | COPE | 0.251172374 | 0.750894971 | 1.1036391 | down | mRNA |
| ENST00000262887 | XRCC1 | 0.556714769 | 0.926833616 | 1.2401321 | down | mRNA |
| ENST00000262888 | KCNN4 | 0.409171602 | 0.867591329 | 1.1796539 | down | mRNA |
| ENST00000262890 | PAFAH1B3 | 0.76040343 | 0.972923327 | 1.0308547 | down | mRNA |
| ENST00000262903 | HACE1 | 0.953214357 | 0.996710991 | 1.0095085 | down | mRNA |
| ENST00000262968 | TJP3 | 0.766309755 | 0.974737263 | 1.0669042 | down | mRNA |
| ENST00000263062 | EPC1 | 0.605839141 | 0.94174007 | 1.1488221 | down | mRNA |
| ENST00000263071 | SCARF1 | 0.81846507 | 0.981973932 | 1.0369575 | down | mRNA |
| ENST00000263084 | OVCA2 | 0.007794335 | 0.19349746 | 1.1343605 | down | mRNA |
| ENST00000263119 | CABIN1 | 0.509399435 | 0.911674633 | 1.0684041 | down | mRNA |
| ENST00000263202 | UFD1L | 0.376474312 | 0.850025065 | 1.0636858 | down | mRNA |
| ENST00000263266 | FAM83E | 0.22508523 | 0.720131063 | 1.1756842 | down | mRNA |
| ENST00000263270 | AP2S1 | 0.093863978 | 0.513134818 | 1.0931843 | down | mRNA |
| ENST00000263274 | LIG1 | 0.944335519 | 0.996710991 | 1.0316211 | down | mRNA |
| ENST00000263284 | CCDC61 | 0.909693895 | 0.995342392 | 1.0214609 | down | mRNA |
| ENST00000263377 | BRD4 | 0.090546085 | 0.508246703 | 1.140907 | down | mRNA |
| ENST00000263390 | MED26 | 0.254186883 | 0.754581357 | 1.36617 | down | mRNA |
| ENST00000263579 | DCPS | 0.329482576 | 0.821768501 | 1.0693296 | down | mRNA |
| ENST00000263629 | MTIF2 | 0.350672433 | 0.834911159 | 1.0742869 | down | mRNA |
| ENST00000263640 | ACVR1 | 0.388822263 | 0.857898453 | 1.2396361 | down | mRNA |
| ENST00000263805 | ZFP106 | 0.936191352 | 0.996710991 | 1.0143835 | down | mRNA |
| ENST00000263847 | OSBP | 0.476697987 | 0.900429473 | 1.0380321 | down | mRNA |
| ENST00000263881 | MAP4K3 | 0.050869934 | 0.407751844 | 1.5189948 | down | mRNA |
| ENST00000263904 | STAM2 | 0.15970864 | 0.631516339 | 1.087212 | down | mRNA |
| ENST00000263932 | TNFRSF8 | 0.894060802 | 0.993978977 | 1.023089 | down | mRNA |
| ENST00000264021 | IFT46 | 0.804444722 | 0.98118927 | 1.0394296 | down | mRNA |
| ENST00000264093 | DGUOK | 0.15361151 | 0.620667112 | 1.0849908 | down | mRNA |
| ENST00000264110 | ATF2 | 0.290850208 | 0.787006447 | 1.1420032 | down | mRNA |
| ENST00000264221 | PAICS | 0.303935073 | 0.799797576 | 1.1280734 | down | mRNA |
| ENST00000264380 | PIKFYVE | 0.089921416 | 0.506589703 | 1.3438893 | down | mRNA |
| ENST00000264538 | IFT57 | 0.744713122 | 0.970330526 | 1.0333395 | down | mRNA |
| ENST00000264651 | KRT24 | 0.921620235 | 0.995349793 | 1.0262142 | down | mRNA |
| ENST00000264711 | DNAJC27 | 0.733260741 | 0.967777568 | 1.1063988 | down | mRNA |
| ENST00000264819 | MIER2 | 0.747703826 | 0.970660679 | 1.0860234 | down | mRNA |
| ENST00000264827 | HOOK2 | 0.033220159 | 0.346375523 | 1.2977729 | down | mRNA |
| ENST00000264828 | COL5A3 | 0.090212625 | 0.507386748 | 1.1798225 | down | mRNA |
| ENST00000264933 | PDCD6 | 0.069222529 | 0.456727607 | 1.0750551 | down | mRNA |
| ENST00000265000 | GALNT7 | 0.181790008 | 0.664924727 | 1.1449204 | down | mRNA |
| ENST00000265016 | BST1 | 0.966376877 | 0.99793927 | 1.0156371 | down | mRNA |
| ENST00000265148 | CENPE | 0.970554396 | 0.998245244 | 1.0132352 | down | mRNA |
| ENST00000265245 | LSG1 | 0.019760974 | 0.282883797 | 1.3365288 | down | mRNA |
| ENST00000265362 | SEMA3A | 0.745245967 | 0.970378122 | 1.1092745 | down | mRNA |
| ENST00000265460 | C11orf9 | 0.665360062 | 0.958354006 | 1.0383318 | down | mRNA |
| ENST00000265495 | ELF2 | 0.136185373 | 0.594906342 | 1.3419541 | down | mRNA |
| ENST00000265713 | KAT6A | 0.6821592 | 0.960111041 | 1.0466682 | down | mRNA |
| ENST00000265728 | DBF4 | 0.095859329 | 0.517253681 | 1.7438059 | down | mRNA |
| ENST00000265741 | CDK14 | 0.917337664 | 0.995349793 | 1.0071362 | down | mRNA |
| ENST00000265742 | ANKIB1 | 0.255320364 | 0.756286855 | 1.1542996 | down | mRNA |
| ENST00000265960 | MAPKAP1 | 0.299680791 | 0.795756183 | 1.0886358 | down | mRNA |
| ENST00000265993 | TCTN3 | 0.37875196 | 0.852482258 | 1.1282878 | down | mRNA |
| ENST00000266110 | RAPGEF1 | 0.938070705 | 0.996710991 | 1.0044412 | down | mRNA |
| ENST00000266712 | TMTC3 | 0.93652558 | 0.996710991 | 1.0130168 | down | mRNA |
| ENST00000267017 | NPFF | 0.769498546 | 0.975286259 | 1.0539516 | down | mRNA |
| ENST00000267119 | KRT71 | 0.38856198 | 0.857898453 | 1.0897846 | down | mRNA |
| ENST00000267176 | SBNO1 | 0.074692966 | 0.469867247 | 1.2503292 | down | mRNA |
| ENST00000267197 | SETD1B | 0.202178381 | 0.69131473 | 1.479387 | down | mRNA |
| ENST00000267339 | ANKRD10 | 0.623968935 | 0.948341211 | 1.0965032 | down | mRNA |
| ENST00000268130 | WDR93 | 0.679452798 | 0.959969193 | 1.028068 | down | mRNA |
| ENST00000268383 | CDR2 | 0.092321554 | 0.511338764 | 1.4845396 | down | mRNA |
| ENST00000268720 | CPNE7 | 0.299719695 | 0.795756183 | 1.263121 | down | mRNA |
| ENST00000268919 | KIF2B | 0.665719255 | 0.958354006 | 1.0785374 | down | mRNA |
| ENST00000269349 | EIF4A3 | 0.094693689 | 0.516031114 | 1.1541173 | down | mRNA |
| ENST00000269439 | RNF165 | 0.277231387 | 0.777205298 | 1.0966719 | down | mRNA |
| ENST00000269856 | FEM1A | 0.063025865 | 0.439769486 | 1.1896246 | down | mRNA |
| ENST00000269919 | PGPEP1 | 0.84908745 | 0.987678166 | 1.0181716 | down | mRNA |
| ENST00000269973 | ZNF45 | 0.206415066 | 0.697843553 | 1.2461052 | down | mRNA |
| ENST00000270066 | SMG9 | 0.014100885 | 0.239157213 | 1.2212298 | down | mRNA |
| ENST00000270593 | ACPT | 0.317818126 | 0.811908136 | 1.2954202 | down | mRNA |
| ENST00000270649 | ZNF614 | 0.26283532 | 0.763125731 | 1.1312915 | down | mRNA |
| ENST00000270747 | ARHGEF19 | 0.810422631 | 0.98118927 | 1.0356399 | down | mRNA |
| ENST00000270824 | FAM176B | 0.96555102 | 0.99793927 | 1.0038163 | down | mRNA |
| ENST00000271638 | S100A11 | 0.591536228 | 0.937275139 | 1.0824466 | down | mRNA |
| ENST00000271715 | POGZ | 0.806440916 | 0.98118927 | 1.0239881 | down | mRNA |
| ENST00000272198 | PPFIA4 | 0.853372252 | 0.987678166 | 1.0443968 | down | mRNA |
| ENST00000272298 | CALM2 | 0.162869946 | 0.635098861 | 1.1894965 | down | mRNA |
| ENST00000272433 | SFXN5 | 0.504141935 | 0.910568383 | 1.1254775 | down | mRNA |
| ENST00000272852 | CPO | 0.669220458 | 0.95930319 | 1.0709774 | down | mRNA |
| ENST00000273038 | VGLL4 | 0.452252804 | 0.888854908 | 1.2214642 | down | mRNA |
| ENST00000273130 | DYNC1LI1 | 0.21022497 | 0.700954461 | 1.1281146 | down | mRNA |
| ENST00000273375 | RABL3 | 0.303139405 | 0.798533134 | 1.2737748 | down | mRNA |
| ENST00000273512 | NCEH1 | 0.921402579 | 0.995349793 | 1.0337072 | down | mRNA |
| ENST00000274532 | TIMD4 | 0.519641673 | 0.916187634 | 1.1201075 | down | mRNA |
| ENST00000274853 | PPP1R18 | 0.813358133 | 0.98120545 | 1.0496126 | down | mRNA |
| ENST00000274897 | MLIP | 0.111032549 | 0.5515548 | 1.1334165 | down | mRNA |
| ENST00000274979 | ANO7 | 0.000565832 | 0.072355298 | 1.3039594 | down | mRNA |
| ENST00000275053 | MMS22L | 0.538808841 | 0.923577434 | 1.2465007 | down | mRNA |
| ENST00000275200 | TAAR8 | 0.977196491 | 0.998501532 | 1.0040024 | down | mRNA |
| ENST00000275766 | ZC3HAV1L | 0.215479277 | 0.709723741 | 1.2251327 | down | mRNA |
| ENST00000275815 | EPHA1 | 0.587756488 | 0.93579682 | 1.1130881 | down | mRNA |
| ENST00000275820 | NOM1 | 0.046839797 | 0.394183256 | 1.2503536 | down | mRNA |
| ENST00000275838 | ASB10 | 0.904691079 | 0.99508468 | 1.0165029 | down | mRNA |
| ENST00000275954 | TMEM47 | 0.995348941 | 0.999872237 | 1.0015941 | down | mRNA |
| ENST00000276594 | PRDM14 | 0.089987007 | 0.506818788 | 1.4001885 | down | mRNA |
| ENST00000276704 | C8orf76 | 0.018468949 | 0.271518921 | 1.2349579 | down | mRNA |
| ENST00000277540 | WDR85 | 0.04275411 | 0.38209959 | 1.337677 | down | mRNA |
| ENST00000277570 | C10orf47 | 0.880390646 | 0.992245156 | 1.0517276 | down | mRNA |
| ENST00000277905 | VAX1 | 0.590201895 | 0.936215326 | 1.2462304 | down | mRNA |
| ENST00000278200 | IMMP1L | 0.136500142 | 0.595179258 | 1.3402584 | down | mRNA |
| ENST00000278409 | OR5F1 | 0.41811209 | 0.871927185 | 1.0888159 | down | mRNA |
| ENST00000278412 | SSRP1 | 0.06289599 | 0.439491843 | 1.1341081 | down | mRNA |
| ENST00000278590 | ZC3H12C | 0.743438837 | 0.970330526 | 1.0626365 | down | mRNA |
| ENST00000278616 | ATM | 0.095779456 | 0.517208636 | 1.3033948 | down | mRNA |
| ENST00000279068 | LSM14B | 0.024967845 | 0.308874358 | 1.1461443 | down | mRNA |
| ENST00000279396 | TMEM219 | 0.529676512 | 0.91899174 | 1.0715442 | down | mRNA |
| ENST00000279804 | CTF1 | 0.116156628 | 0.562521166 | 1.2746924 | down | mRNA |
| ENST00000280871 | SLC2A13 | 0.349787469 | 0.834140137 | 1.0544785 | down | mRNA |
| ENST00000281471 | AMN1 | 0.66051608 | 0.958354006 | 1.1111643 | down | mRNA |
| ENST00000281806 | MCHR2 | 0.334917238 | 0.824836547 | 1.1938586 | down | mRNA |
| ENST00000282018 | CYSLTR2 | 0.329379826 | 0.821768501 | 1.0886761 | down | mRNA |
| ENST00000282030 | SETBP1 | 0.296040777 | 0.792404038 | 1.22759 | down | mRNA |
| ENST00000282050 | ATP5A1 | 0.036722189 | 0.359628653 | 1.1448071 | down | mRNA |
| ENST00000282441 | YAP1 | 0.012781013 | 0.23037549 | 1.3107553 | down | mRNA |
| ENST00000282892 | MED21 | 0.630700939 | 0.950410442 | 1.0607703 | down | mRNA |
| ENST00000283006 | CENPH | 0.956121277 | 0.996883425 | 1.0246293 | down | mRNA |
| ENST00000283195 | RANBP2 | 0.650100026 | 0.955736351 | 1.0244711 | down | mRNA |
| ENST00000284629 | ASZ1 | 0.872733934 | 0.990534615 | 1.0083846 | down | mRNA |
| ENST00000284669 | KBTBD10 | 0.315484762 | 0.810346887 | 1.271232 | down | mRNA |
| ENST00000284690 | DHX32 | 0.038420616 | 0.366145077 | 1.2693379 | down | mRNA |
| ENST00000284984 | ADAMTS1 | 0.001935106 | 0.107205943 | 1.7367513 | down | mRNA |
| ENST00000285023 | C17orf50 | 0.340819479 | 0.828854153 | 1.2806366 | down | mRNA |
| ENST00000285398 | ERCC3 | 0.032131954 | 0.3425639 | 1.3702537 | down | mRNA |
| ENST00000285420 | OTUD6B | 0.135052158 | 0.592787801 | 1.2838083 | down | mRNA |
| ENST00000286380 | RAET1L | 0.543329353 | 0.923577434 | 1.0484313 | down | mRNA |
| ENST00000287020 | GDF6 | 0.009518207 | 0.204959264 | 2.2590084 | down | mRNA |
| ENST00000287468 | FBRS | 0.84442089 | 0.987338076 | 1.0249686 | down | mRNA |
| ENST00000287490 | COX6A2 | 0.453120847 | 0.889563297 | 1.0634984 | down | mRNA |
| ENST00000287996 | IPPK | 0.019979008 | 0.283521851 | 1.3945572 | down | mRNA |
| ENST00000288670 | FMNL2 | 0.945726274 | 0.996710991 | 1.0091098 | down | mRNA |
| ENST00000288709 | MMEL1 | 0.987363927 | 0.999140676 | 1.0027475 | down | mRNA |
| ENST00000288986 | NCK1 | 0.374814 | 0.849578399 | 1.2092049 | down | mRNA |
| ENST00000289753 | HTR6 | 0.599353501 | 0.940648177 | 1.030916 | down | mRNA |
| ENST00000289820 | NPM2 | 0.31840071 | 0.812422372 | 1.0595081 | down | mRNA |
| ENST00000289877 | SLC45A1 | 0.605860811 | 0.94174007 | 1.0854688 | down | mRNA |
| ENST00000289902 | FCER1G | 0.26005685 | 0.760388386 | 1.1569733 | down | mRNA |
| ENST00000290438 | GOLGA6A | 0.959564195 | 0.99758957 | 1.0169599 | down | mRNA |
| ENST00000290894 | SHF | 0.656699981 | 0.957445617 | 1.0748127 | down | mRNA |
| ENST00000291439 | AP1M1 | 0.09557941 | 0.51694341 | 1.1322238 | down | mRNA |
| ENST00000291442 | NR2F6 | 0.078731131 | 0.480853518 | 1.5122174 | down | mRNA |
| ENST00000291478 | KALRN | 0.60757084 | 0.942627675 | 1.1145609 | down | mRNA |
| ENST00000291495 | CILP2 | 0.550642796 | 0.925928432 | 1.1208262 | down | mRNA |
| ENST00000291670 | FTCD | 0.102098136 | 0.530503273 | 1.5747409 | down | mRNA |
| ENST00000291688 | MCM3AP | 0.013146989 | 0.2344777 | 1.2769018 | down | mRNA |
| ENST00000291715 | CLDND2 | 0.643127842 | 0.954522001 | 1.0739713 | down | mRNA |
| ENST00000291726 | KLK8 | 0.478767314 | 0.901271944 | 1.1047297 | down | mRNA |
| ENST00000291764 | CYP2A7 | 0.317807281 | 0.811908136 | 1.1342564 | down | mRNA |
| ENST00000292079 | FXYD2 | 0.306120766 | 0.801520161 | 1.3656198 | down | mRNA |
| ENST00000292357 | PEAR1 | 0.403385487 | 0.864786348 | 1.2019912 | down | mRNA |
| ENST00000292510 | VPS28 | 0.630771009 | 0.950410442 | 1.0227066 | down | mRNA |
| ENST00000292579 | ZNF250 | 0.262980666 | 0.763125731 | 1.1274328 | down | mRNA |
| ENST00000293330 | HCRT | 0.938486759 | 0.996710991 | 1.009009 | down | mRNA |
| ENST00000293889 | CCDC78 | 0.247166761 | 0.745152522 | 1.6622494 | down | mRNA |
| ENST00000293894 | SOX8 | 0.419997541 | 0.872365335 | 1.0749374 | down | mRNA |
| ENST00000294172 | NXF1 | 0.433225168 | 0.880722826 | 1.1127589 | down | mRNA |
| ENST00000294189 | RPL29 | 0.759699861 | 0.972806246 | 1.0089887 | down | mRNA |
| ENST00000294517 | ADC | 0.918200986 | 0.995349793 | 1.0184735 | down | mRNA |
| ENST00000294613 | TM2D1 | 0.326528644 | 0.819425644 | 1.5380438 | down | mRNA |
| ENST00000294638 | LHX8 | 0.806536699 | 0.98118927 | 1.0680802 | down | mRNA |
| ENST00000294794 | OLFML2B | 0.678347215 | 0.959825429 | 1.0586731 | down | mRNA |
| ENST00000295031 | KIAA1841 | 0.213149627 | 0.705479118 | 1.1991499 | down | mRNA |
| ENST00000295119 | NUP35 | 0.079949983 | 0.483876854 | 1.3021806 | down | mRNA |
| ENST00000295641 | STK11IP | 0.849309085 | 0.987678166 | 1.0154806 | down | mRNA |
| ENST00000295962 | ABHD6 | 0.281535755 | 0.779126326 | 1.2031911 | down | mRNA |
| ENST00000295974 | APBB2 | 0.463658157 | 0.894989844 | 1.0682655 | down | mRNA |
| ENST00000296102 | MRPL33 | 0.983360838 | 0.998971807 | 1.0013012 | down | mRNA |
| ENST00000296218 | DNALI1 | 0.060166579 | 0.435030895 | 1.3288875 | down | mRNA |
| ENST00000296223 | POLR2H | 0.101489236 | 0.529356363 | 1.2276594 | down | mRNA |
| ENST00000296318 | IL17RD | 0.589373876 | 0.936090876 | 1.1056833 | down | mRNA |
| ENST00000296402 | CAMK2D | 0.207559466 | 0.698556218 | 1.0712031 | down | mRNA |
| ENST00000296595 | TMEM161B | 0.022326433 | 0.294616576 | 1.2255174 | down | mRNA |
| ENST00000296662 | C5orf63 | 0.677791716 | 0.959531882 | 1.083777 | down | mRNA |
| ENST00000296777 | CARTPT | 0.122512194 | 0.574983504 | 1.1116794 | down | mRNA |
| ENST00000296875 | GDF9 | 0.480270782 | 0.901538317 | 1.0683689 | down | mRNA |
| ENST00000297161 | BMPER | 0.773490097 | 0.97589756 | 1.0416455 | down | mRNA |
| ENST00000297431 | ORC5 | 0.306534238 | 0.801653799 | 1.3193402 | down | mRNA |
| ENST00000297468 | GPR146 | 0.529933836 | 0.91899174 | 1.1493833 | down | mRNA |
| ENST00000297534 | C7orf55 | 0.967957383 | 0.99793927 | 1.009715 | down | mRNA |
| ENST00000297988 | AQP7 | 0.970055909 | 0.998245244 | 1.0035791 | down | mRNA |
| ENST00000298231 | PHOX2A | 0.988326849 | 0.999140676 | 1.0036803 | down | mRNA |
| ENST00000298288 | LRR1 | 0.25998424 | 0.760388386 | 1.4349004 | down | mRNA |
| ENST00000298292 | DNAAF2 | 0.000860526 | 0.083093876 | 1.7675928 | down | mRNA |
| ENST00000298767 | WAPAL | 0.065526697 | 0.447226857 | 1.1378795 | down | mRNA |
| ENST00000299001 | PIWIL4 | 0.140125271 | 0.600174217 | 1.2615795 | down | mRNA |
| ENST00000299204 | BAG5 | 0.261710816 | 0.762445093 | 1.1625917 | down | mRNA |
| ENST00000299505 | KIAA0355 | 0.102598752 | 0.531600463 | 1.3865804 | down | mRNA |
| ENST00000299543 | CTDP1 | 0.255468135 | 0.756286855 | 1.2359839 | down | mRNA |
| ENST00000299563 | RNF169 | 0.723204866 | 0.966191059 | 1.0416575 | down | mRNA |
| ENST00000299697 | TK2 | 0.59792886 | 0.940150767 | 1.0609203 | down | mRNA |
| ENST00000300131 | NAB2 | 0.004849776 | 0.158753988 | 1.226887 | down | mRNA |
| ENST00000300151 | MRPL16 | 0.090582005 | 0.508246703 | 1.1107058 | down | mRNA |
| ENST00000300283 | CKMT1B | 0.849639391 | 0.987678166 | 1.066816 | down | mRNA |
| ENST00000300432 | PIP5KL1 | 0.410635813 | 0.868335658 | 1.2026887 | down | mRNA |
| ENST00000300575 | C16orf92 | 0.732758621 | 0.967703318 | 1.1191848 | down | mRNA |
| ENST00000300747 | TRIM68 | 0.08648313 | 0.49936346 | 1.4461937 | down | mRNA |
| ENST00000300853 | ERCC1 | 0.81704012 | 0.981692737 | 1.0220799 | down | mRNA |
| ENST00000300873 | GNG8 | 0.374663381 | 0.849426389 | 1.1371796 | down | mRNA |
| ENST00000300896 | USP32 | 0.013253574 | 0.234731416 | 1.4185129 | down | mRNA |
| ENST00000300954 | PCSK4 | 0.850334922 | 0.987678166 | 1.0473979 | down | mRNA |
| ENST00000300976 | KLHL26 | 0.501981001 | 0.910209514 | 1.1001522 | down | mRNA |
| ENST00000301031 | C16orf55 | 0.926664005 | 0.99582096 | 1.0149318 | down | mRNA |
| ENST00000301067 | MLL2 | 0.060136085 | 0.435030895 | 1.5651985 | down | mRNA |
| ENST00000301073 | ZNF524 | 0.860369839 | 0.988975154 | 1.0107674 | down | mRNA |
| ENST00000301215 | ZNF526 | 0.965529766 | 0.99793927 | 1.0036305 | down | mRNA |
| ENST00000301264 | DAPK3 | 0.555707808 | 0.926833616 | 1.1331821 | down | mRNA |
| ENST00000301310 | ZNF582 | 0.597271766 | 0.939714862 | 1.0935208 | down | mRNA |
| ENST00000301420 | KLK1 | 0.522630484 | 0.917200998 | 1.1151243 | down | mRNA |
| ENST00000301458 | CD320 | 0.349613981 | 0.834138149 | 1.0503919 | down | mRNA |
| ENST00000301475 | ZNF558 | 0.015359927 | 0.248644932 | 1.4732029 | down | mRNA |
| ENST00000301488 | CDK2AP2 | 0.341722213 | 0.830223283 | 1.1700442 | down | mRNA |
| ENST00000301727 | E4F1 | 0.013385855 | 0.23544335 | 1.4339878 | down | mRNA |
| ENST00000301740 | SRRM2 | 0.845197946 | 0.987338076 | 1.0267665 | down | mRNA |
| ENST00000302054 | BRPF1 | 0.06872288 | 0.455137976 | 1.3644416 | down | mRNA |
| ENST00000302165 | IRF2BP1 | 0.003983194 | 0.14487712 | 1.5972842 | down | mRNA |
| ENST00000302303 | RFX3 | 0.529953763 | 0.91899174 | 1.100052 | down | mRNA |
| ENST00000302326 | MN1 | 0.301990147 | 0.797262221 | 1.3321266 | down | mRNA |
| ENST00000302746 | PDHB | 0.451335203 | 0.888854908 | 1.0669826 | down | mRNA |
| ENST00000302787 | LETM1 | 0.847831852 | 0.987678166 | 1.028101 | down | mRNA |
| ENST00000302850 | INSR | 0.963998913 | 0.99793927 | 1.0088244 | down | mRNA |
| ENST00000302926 | NLGN2 | 0.474157018 | 0.899418411 | 1.067895 | down | mRNA |
| ENST00000302981 | OR5AP2 | 0.90143778 | 0.994902084 | 1.04892 | down | mRNA |
| ENST00000303015 | KCNK9 | 0.097636945 | 0.520535504 | 1.0980307 | down | mRNA |
| ENST00000303415 | SIRPG | 0.786102224 | 0.977479172 | 1.0228245 | down | mRNA |
| ENST00000303538 | PDS5A | 0.011778023 | 0.221117975 | 1.1919054 | down | mRNA |
| ENST00000303790 | KCNAB3 | 0.093615213 | 0.512619241 | 1.348367 | down | mRNA |
| ENST00000303798 | C2orf27B | 0.728760335 | 0.966191059 | 1.0848795 | down | mRNA |
| ENST00000303941 | ANKK1 | 0.318703361 | 0.812829495 | 1.8591607 | down | mRNA |
| ENST00000304270 | SPACA5B | 0.391960676 | 0.859445464 | 1.2337705 | down | mRNA |
| ENST00000304477 | UTF1 | 0.832529039 | 0.98623676 | 1.0374825 | down | mRNA |
| ENST00000304527 | SH3D19 | 0.075139954 | 0.470853901 | 1.4258392 | down | mRNA |
| ENST00000304677 | RNASE6 | 0.974038923 | 0.998501532 | 1.0062181 | down | mRNA |
| ENST00000304735 | VASN | 1.42198E-05 | 0.017006855 | 1.5888049 | down | mRNA |
| ENST00000304880 | OR1N1 | 0.555497931 | 0.926833616 | 1.0611731 | down | mRNA |
| ENST00000304920 | IL17D | 0.832263878 | 0.986211576 | 1.036118 | down | mRNA |
| ENST00000304992 | PRPF8 | 0.14562943 | 0.607496425 | 1.1552483 | down | mRNA |
| ENST00000305107 | UGT2B4 | 0.127612187 | 0.580991132 | 1.2342603 | down | mRNA |
| ENST00000305135 | YEATS2 | 0.161143352 | 0.632747516 | 1.2997754 | down | mRNA |
| ENST00000305425 | DACH1 | 0.384627678 | 0.85485898 | 1.0647089 | down | mRNA |
| ENST00000305456 | OR7G2 | 0.836080129 | 0.986575022 | 1.0576538 | down | mRNA |
| ENST00000305596 | CD2BP2 | 0.124865064 | 0.578441772 | 1.1548595 | down | mRNA |
| ENST00000305748 | KRT73 | 0.06310623 | 0.439861454 | 1.192516 | down | mRNA |
| ENST00000305892 | OR10H3 | 0.731582416 | 0.967176866 | 1.0430576 | down | mRNA |
| ENST00000306051 | PTGDR | 0.534904908 | 0.921043919 | 1.3370224 | down | mRNA |
| ENST00000306065 | ANKRD27 | 0.473084804 | 0.899184677 | 1.1193807 | down | mRNA |
| ENST00000306078 | KCNG3 | 0.595969489 | 0.93896389 | 1.0452176 | down | mRNA |
| ENST00000306096 | RAB31 | 0.466097767 | 0.895718317 | 1.0709917 | down | mRNA |
| ENST00000306271 | KRTAP1-1 | 0.242239143 | 0.740610726 | 2.3367133 | down | mRNA |
| ENST00000306322 | PSG11 | 0.305218276 | 0.801056074 | 1.2484264 | down | mRNA |
| ENST00000306448 | ATP6V1E2 | 0.73681285 | 0.968834005 | 1.0199079 | down | mRNA |
| ENST00000306511 | PSG8 | 0.375035718 | 0.849710156 | 1.3920606 | down | mRNA |
| ENST00000306739 | ASPSCR1 | 0.817115863 | 0.981692737 | 1.0158095 | down | mRNA |
| ENST00000306908 | ZNF57 | 0.4885124 | 0.904099228 | 1.370325 | down | mRNA |
| ENST00000306985 | MAP1LC3B2 | 0.999859183 | 0.999954265 | 1.0000203 | down | mRNA |
| ENST00000306999 | ANKMY2 | 0.056106124 | 0.422065984 | 1.3865648 | down | mRNA |
| ENST00000307050 | OTUD7A | 0.442349168 | 0.88419144 | 1.1028431 | down | mRNA |
| ENST00000307142 | SYNPO2 | 0.031891602 | 0.342121122 | 1.1802074 | down | mRNA |
| ENST00000307266 | MYEOV2 | 0.914927524 | 0.995349793 | 1.0068885 | down | mRNA |
| ENST00000307401 | OR2AG1 | 0.985856676 | 0.998971807 | 1.0054271 | down | mRNA |
| ENST00000307471 | CCDC126 | 0.76829598 | 0.975142015 | 1.0514188 | down | mRNA |
| ENST00000307658 | ZNF17 | 0.132481247 | 0.588108866 | 1.3577517 | down | mRNA |
| ENST00000307823 | CARNS1 | 0.929929712 | 0.996643872 | 1.0176652 | down | mRNA |
| ENST00000307892 | CYB5B | 0.200807776 | 0.68900633 | 1.1000664 | down | mRNA |
| ENST00000308009 | SLC16A11 | 0.86716249 | 0.989481868 | 1.020614 | down | mRNA |
| ENST00000308020 | PTDSS2 | 0.221740906 | 0.716734257 | 1.1084685 | down | mRNA |
| ENST00000308234 | RGMB | 0.370254862 | 0.846215286 | 1.2814389 | down | mRNA |
| ENST00000308304 | PROP1 | 0.062795599 | 0.439458294 | 1.6289205 | down | mRNA |
| ENST00000308344 | CLDN15 | 0.299798107 | 0.795797471 | 1.1061782 | down | mRNA |
| ENST00000308436 | TUT1 | 0.094838485 | 0.516057441 | 1.3090592 | down | mRNA |
| ENST00000308528 | LYG1 | 0.237296392 | 0.735249961 | 1.3601478 | down | mRNA |
| ENST00000308906 | C20orf201 | 0.993892706 | 0.999872237 | 1.0013286 | down | mRNA |
| ENST00000308919 | CYP2W1 | 0.330192068 | 0.821769369 | 1.1468302 | down | mRNA |
| ENST00000309052 | LCLAT1 | 0.075289882 | 0.471448685 | 1.4932165 | down | mRNA |
| ENST00000309137 | FAM192A | 0.725247886 | 0.966191059 | 1.0301329 | down | mRNA |
| ENST00000309212 | MXRA8 | 0.537493795 | 0.922702335 | 1.1177018 | down | mRNA |
| ENST00000309305 | ZNF519 | 0.504558371 | 0.910568383 | 1.0970537 | down | mRNA |
| ENST00000309424 | CD3EAP | 0.215122141 | 0.708891956 | 1.2614289 | down | mRNA |
| ENST00000309777 | TMEM234 | 0.708645896 | 0.965462762 | 1.0191275 | down | mRNA |
| ENST00000309859 | GGA2 | 0.886477882 | 0.992981836 | 1.0163526 | down | mRNA |
| ENST00000310125 | TFDP3 | 0.989360275 | 0.999140676 | 1.002119 | down | mRNA |
| ENST00000310447 | CASP2 | 0.621756195 | 0.948047106 | 1.0547746 | down | mRNA |
| ENST00000310483 | SLC39A1 | 0.204371167 | 0.694397489 | 1.1280745 | down | mRNA |
| ENST00000310496 | RFNG | 0.90071853 | 0.994789506 | 1.0118767 | down | mRNA |
| ENST00000310597 | ZNHIT2 | 0.000740464 | 0.079656701 | 1.4984459 | down | mRNA |
| ENST00000310645 | COL6A2 | 0.021654241 | 0.29025889 | 1.3951567 | down | mRNA |
| ENST00000310791 | REP15 | 0.012735291 | 0.230163503 | 1.7079454 | down | mRNA |
| ENST00000310806 | MIS18BP1 | 0.076084241 | 0.473941421 | 1.5567075 | down | mRNA |
| ENST00000310864 | IQCB1 | 0.002403542 | 0.117191396 | 1.407928 | down | mRNA |
| ENST00000310926 | SLCO2A1 | 0.807223109 | 0.98118927 | 1.0457817 | down | mRNA |
| ENST00000311048 | ZNF431 | 0.14030951 | 0.600457367 | 1.5055669 | down | mRNA |
| ENST00000311051 | APBB1 | 0.528862344 | 0.918932853 | 1.0217085 | down | mRNA |
| ENST00000311330 | CD248 | 0.375184867 | 0.849710156 | 1.1642681 | down | mRNA |
| ENST00000311469 | COQ2 | 0.670726664 | 0.959531882 | 1.0480334 | down | mRNA |
| ENST00000311966 | GLS2 | 0.101792184 | 0.529641203 | 1.2371556 | down | mRNA |
| ENST00000312026 | ZNF417 | 0.051836448 | 0.41031217 | 1.2108576 | down | mRNA |
| ENST00000312051 | URI1 | 0.167260302 | 0.642184223 | 1.1479982 | down | mRNA |
| ENST00000312352 | PFKM | 0.910151399 | 0.995342392 | 1.0156922 | down | mRNA |
| ENST00000312413 | CLCN6 | 0.789173573 | 0.977807732 | 1.0524683 | down | mRNA |
| ENST00000312513 | MRPL45 | 0.770510811 | 0.975347132 | 1.0097777 | down | mRNA |
| ENST00000312584 | TNFRSF10D | 0.941966044 | 0.996710991 | 1.0061588 | down | mRNA |
| ENST00000312675 | LPP | 0.867531437 | 0.989481868 | 1.0227771 | down | mRNA |
| ENST00000312942 | KCNRG | 0.855206053 | 0.988178307 | 1.0318781 | down | mRNA |
| ENST00000313117 | SRSF7 | 0.151980095 | 0.617517843 | 1.1574825 | down | mRNA |
| ENST00000313234 | KRT80 | 0.953907739 | 0.996710991 | 1.0064345 | down | mRNA |
| ENST00000313503 | OR8H2 | 0.693312248 | 0.961584652 | 1.0889192 | down | mRNA |
| ENST00000313511 | TUFM | 0.172359106 | 0.6499464 | 1.0978746 | down | mRNA |
| ENST00000313525 | DNAJC25 | 0.778850796 | 0.976938981 | 1.0333145 | down | mRNA |
| ENST00000313860 | LIMCH1 | 0.378338999 | 0.851828819 | 1.1188418 | down | mRNA |
| ENST00000314133 | COX8A | 0.64729495 | 0.95550188 | 1.0277193 | down | mRNA |
| ENST00000314136 | ANP32E | 0.208506194 | 0.699446946 | 1.2230728 | down | mRNA |
| ENST00000314218 | BBS12 | 0.026674818 | 0.318484367 | 1.2489784 | down | mRNA |
| ENST00000314349 | TIMM50 | 0.797156199 | 0.980667273 | 1.0214147 | down | mRNA |
| ENST00000314358 | KDM3B | 0.017062652 | 0.26281654 | 1.1809175 | down | mRNA |
| ENST00000314571 | TSEN2 | 0.010139523 | 0.208872123 | 1.5647357 | down | mRNA |
| ENST00000314595 | POLR2B | 0.332730181 | 0.823201514 | 1.0517387 | down | mRNA |
| ENST00000314606 | FARSA | 0.159374265 | 0.631041393 | 1.0996814 | down | mRNA |
| ENST00000314607 | ZMYM4 | 0.0922135 | 0.511207518 | 1.151708 | down | mRNA |
| ENST00000314636 | DHFRL1 | 0.631115561 | 0.950410442 | 1.0507801 | down | mRNA |
| ENST00000314644 | OR4C15 | 0.721067207 | 0.966191059 | 1.0515674 | down | mRNA |
| ENST00000314720 | TCEANC | 0.560305523 | 0.92782421 | 1.0957249 | down | mRNA |
| ENST00000314759 | SKA3 | 0.749995038 | 0.970725371 | 1.1706679 | down | mRNA |
| ENST00000315204 | STK33 | 0.539646358 | 0.923577434 | 1.0552688 | down | mRNA |
| ENST00000315286 | CCDC43 | 0.584011912 | 0.934461268 | 1.0670115 | down | mRNA |
| ENST00000315456 | DTNA | 0.958478829 | 0.997072988 | 1.0074258 | down | mRNA |
| ENST00000315576 | EMR2 | 0.305531609 | 0.801185951 | 1.123259 | down | mRNA |
| ENST00000315732 | LSM10 | 0.658713391 | 0.958354006 | 1.0178722 | down | mRNA |
| ENST00000315927 | YOD1 | 0.25105055 | 0.750861986 | 1.1471442 | down | mRNA |
| ENST00000315939 | WNK1 | 0.032089834 | 0.342493703 | 1.1577395 | down | mRNA |
| ENST00000316048 | C12orf69 | 0.638535522 | 0.952998916 | 1.059105 | down | mRNA |
| ENST00000316144 | LCN15 | 0.495584037 | 0.907685311 | 1.0452479 | down | mRNA |
| ENST00000316157 | LARP4B | 0.080817564 | 0.486004945 | 1.2915826 | down | mRNA |
| ENST00000316163 | RGS11 | 0.400292577 | 0.864237287 | 1.4151648 | down | mRNA |
| ENST00000316409 | FAM89B | 0.053634896 | 0.416065637 | 1.1482332 | down | mRNA |
| ENST00000316515 | LRRC34 | 0.771426773 | 0.975347132 | 1.0247975 | down | mRNA |
| ENST00000316554 | C12orf68 | 0.556245183 | 0.926833616 | 1.1346372 | down | mRNA |
| ENST00000316562 | PANK2 | 0.167344882 | 0.642215201 | 1.1017307 | down | mRNA |
| ENST00000316610 | MBLAC2 | 0.052716424 | 0.413038171 | 1.2419126 | down | mRNA |
| ENST00000316615 | GRB2 | 0.654208256 | 0.957205183 | 1.022437 | down | mRNA |
| ENST00000316650 | OR56A1 | 0.977424782 | 0.998501532 | 1.0028547 | down | mRNA |
| ENST00000316963 | LYPLA1 | 0.631546505 | 0.950475493 | 1.0308608 | down | mRNA |
| ENST00000316985 | WHSC1L1 | 0.030724937 | 0.339510558 | 1.2044747 | down | mRNA |
| ENST00000317178 | ZNF776 | 0.001363903 | 0.094832054 | 1.3613565 | down | mRNA |
| ENST00000317243 | ZNF554 | 0.038373014 | 0.365947526 | 1.227026 | down | mRNA |
| ENST00000317449 | LRRN4CL | 0.886290604 | 0.992981836 | 1.0452624 | down | mRNA |
| ENST00000317506 | RANBP10 | 0.097878973 | 0.520535504 | 1.5205054 | down | mRNA |
| ENST00000317643 | SIGLEC7 | 0.343398295 | 0.831536584 | 1.0844364 | down | mRNA |
| ENST00000317676 | DEFB124 | 0.083724893 | 0.493003289 | 1.3184749 | down | mRNA |
| ENST00000317683 | FBXO46 | 0.89416343 | 0.993978977 | 1.007311 | down | mRNA |
| ENST00000317861 | OR14C36 | 0.759089967 | 0.972369912 | 1.0602982 | down | mRNA |
| ENST00000317996 | OR2T12 | 0.818214472 | 0.981818735 | 1.0453764 | down | mRNA |
| ENST00000318008 | LASP1 | 0.197429446 | 0.685708149 | 1.0936588 | down | mRNA |
| ENST00000318024 | USH1C | 0.4619366 | 0.894754673 | 1.1215721 | down | mRNA |
| ENST00000318430 | TMC8 | 0.327400331 | 0.820319134 | 1.0833605 | down | mRNA |
| ENST00000318438 | SYCN | 0.725544643 | 0.966191059 | 1.039341 | down | mRNA |
| ENST00000318622 | RHBDD2 | 0.438934807 | 0.882895421 | 1.0149871 | down | mRNA |
| ENST00000318948 | NRIP1 | 0.828692718 | 0.985493382 | 1.0179235 | down | mRNA |
| ENST00000319129 | FBF1 | 0.842297868 | 0.987123192 | 1.0396947 | down | mRNA |
| ENST00000319234 | SHISA3 | 0.095260394 | 0.516597924 | 1.851647 | down | mRNA |
| ENST00000319545 | MRI1 | 0.336662592 | 0.826506012 | 1.3125062 | down | mRNA |
| ENST00000319694 | PRR15 | 0.662891838 | 0.958354006 | 1.0235238 | down | mRNA |
| ENST00000319760 | OR4A5 | 0.486750378 | 0.903524001 | 1.1528718 | down | mRNA |
| ENST00000319822 | U2SURP | 0.550100633 | 0.925651417 | 1.0478666 | down | mRNA |
| ENST00000319836 | MARCH8 | 0.273959039 | 0.774088254 | 1.1144121 | down | mRNA |
| ENST00000319942 | RABGGTB | 0.095800104 | 0.517208636 | 1.1286005 | down | mRNA |
| ENST00000319974 | C2orf69 | 0.906559197 | 0.99508468 | 1.0115865 | down | mRNA |
| ENST00000320152 | PRTFDC1 | 0.943790556 | 0.996710991 | 1.0093422 | down | mRNA |
| ENST00000320254 | LRRC37A | 0.041308605 | 0.376630745 | 1.2676812 | down | mRNA |
| ENST00000320316 | PHF23 | 0.007689599 | 0.193257016 | 1.3641974 | down | mRNA |
| ENST00000320451 | ZNF121 | 0.520750779 | 0.916605148 | 1.0392816 | down | mRNA |
| ENST00000320574 | POLE | 0.990736578 | 0.999376923 | 1.0028779 | down | mRNA |
| ENST00000320936 | CIRBP | 0.44039249 | 0.883544565 | 1.0752387 | down | mRNA |
| ENST00000321063 | PLXNA4 | 0.441229124 | 0.883920737 | 1.086077 | down | mRNA |
| ENST00000321149 | DYX1C1 | 0.54247876 | 0.923577434 | 1.1232003 | down | mRNA |
| ENST00000321322 | DSCAML1 | 0.315447499 | 0.810346887 | 2.7918812 | down | mRNA |
| ENST00000321367 | SEPT1 | 0.437372036 | 0.882322896 | 1.200122 | down | mRNA |
| ENST00000321543 | OR51L1 | 0.168732865 | 0.644327097 | 1.2853283 | down | mRNA |
| ENST00000321545 | ZNF543 | 0.921191602 | 0.995349793 | 1.0119214 | down | mRNA |
| ENST00000321556 | PINK1 | 0.428363672 | 0.877211962 | 1.0991207 | down | mRNA |
| ENST00000321600 | C18orf26 | 0.91564155 | 0.995349793 | 1.0617552 | down | mRNA |
| ENST00000321662 | GPR137C | 0.28332632 | 0.780779444 | 1.2272391 | down | mRNA |
| ENST00000321925 | SLC14A1 | 0.764241798 | 0.973899357 | 1.2165341 | down | mRNA |
| ENST00000322067 | BPNT1 | 0.337528553 | 0.827187565 | 1.0651055 | down | mRNA |
| ENST00000322146 | ZNF836 | 0.010328849 | 0.209736088 | 1.4056249 | down | mRNA |
| ENST00000322238 | XPNPEP1 | 0.046460555 | 0.392453681 | 1.2587042 | down | mRNA |
| ENST00000322716 | EPM2AIP1 | 0.015932241 | 0.25406613 | 1.7825731 | down | mRNA |
| ENST00000322748 | ZNF18 | 0.110090068 | 0.549130968 | 1.4051287 | down | mRNA |
| ENST00000322810 | PLEC | 0.326923217 | 0.819909073 | 1.0879202 | down | mRNA |
| ENST00000322813 | FAM214B | 0.134013609 | 0.590871682 | 1.1640993 | down | mRNA |
| ENST00000323084 | TSPEAR | 0.739278704 | 0.969564098 | 1.0237934 | down | mRNA |
| ENST00000323205 | GCAT | 0.77981949 | 0.976938981 | 1.0544864 | down | mRNA |
| ENST00000323319 | CLDN22 | 0.190950989 | 0.676402188 | 1.4762407 | down | mRNA |
| ENST00000323570 | ELMOD2 | 0.805239737 | 0.98118927 | 1.0258646 | down | mRNA |
| ENST00000323830 | CPN2 | 0.472270181 | 0.899166338 | 1.1713076 | down | mRNA |
| ENST00000324109 | DNMBP | 0.060261397 | 0.435378361 | 1.3669159 | down | mRNA |
| ENST00000324436 | ACTL9 | 0.418006846 | 0.871927185 | 1.0453837 | down | mRNA |
| ENST00000324464 | ADCK4 | 0.556880771 | 0.926833616 | 1.0862967 | down | mRNA |
| ENST00000324932 | NUP98 | 0.157053365 | 0.625751326 | 1.2732036 | down | mRNA |
| ENST00000324978 | AQP10 | 0.886581848 | 0.992981836 | 1.0032858 | down | mRNA |
| ENST00000325167 | CHMP6 | 0.130968394 | 0.585885453 | 1.1094979 | down | mRNA |
| ENST00000325366 | C5orf22 | 0.508017138 | 0.911674633 | 1.0832155 | down | mRNA |
| ENST00000325437 | UBE2I | 0.159797855 | 0.63153842 | 1.2007705 | down | mRNA |
| ENST00000325542 | CEP57 | 0.448308655 | 0.887969953 | 1.0708792 | down | mRNA |
| ENST00000325748 | PRKRA | 0.621882183 | 0.948047106 | 1.0995587 | down | mRNA |
| ENST00000326000 | WFDC9 | 0.663868476 | 0.958354006 | 1.0689597 | down | mRNA |
| ENST00000326003 | KLK3 | 0.443953701 | 0.885088246 | 1.09492 | down | mRNA |
| ENST00000326219 | TAC4 | 0.695712263 | 0.962390919 | 1.0781496 | down | mRNA |
| ENST00000326233 | LCE1D | 0.915146518 | 0.995349793 | 1.0109239 | down | mRNA |
| ENST00000326282 | EID2B | 0.231414296 | 0.729136134 | 1.067671 | down | mRNA |
| ENST00000326524 | GDNF | 0.342217066 | 0.830602529 | 1.3673062 | down | mRNA |
| ENST00000326665 | C1orf173 | 0.697944945 | 0.962410025 | 1.0487121 | down | mRNA |
| ENST00000326783 | EDDM3B | 0.499102259 | 0.909361611 | 1.0639365 | down | mRNA |
| ENST00000326856 | KLK15 | 0.245119772 | 0.743589595 | 1.1043887 | down | mRNA |
| ENST00000326873 | STK11 | 0.149816327 | 0.614198134 | 1.1388698 | down | mRNA |
| ENST00000327087 | ANO2 | 0.192475477 | 0.678822446 | 1.3256854 | down | mRNA |
| ENST00000327134 | PAK2 | 0.30618764 | 0.801520161 | 1.069818 | down | mRNA |
| ENST00000327365 | GGT5 | 0.157222638 | 0.625935125 | 1.2401273 | down | mRNA |
| ENST00000327470 | FANCF | 0.03982097 | 0.371365115 | 1.5968157 | down | mRNA |
| ENST00000327554 | CCDC60 | 0.777780363 | 0.976938981 | 1.0566366 | down | mRNA |
| ENST00000327619 | EYA2 | 0.018456526 | 0.271518921 | 1.2993014 | down | mRNA |
| ENST00000327835 | ZNF704 | 0.66137705 | 0.958354006 | 1.0519245 | down | mRNA |
| ENST00000328090 | FAM208B | 0.68395782 | 0.960111041 | 1.0395497 | down | mRNA |
| ENST00000328111 | DNMT3B | 0.62276199 | 0.948341211 | 1.1850826 | down | mRNA |
| ENST00000328119 | KRT36 | 0.751981897 | 0.970725371 | 1.0324029 | down | mRNA |
| ENST00000328178 | ZNF85 | 0.006782195 | 0.183552613 | 1.2122676 | down | mRNA |
| ENST00000328396 | PIGW | 0.521513857 | 0.916644975 | 1.1338196 | down | mRNA |
| ENST00000328488 | HIST1H3I | 0.800073703 | 0.981186955 | 1.1757518 | down | mRNA |
| ENST00000328526 | XKRX | 0.58428864 | 0.93461341 | 1.1412537 | down | mRNA |
| ENST00000328771 | DHX34 | 0.630029496 | 0.950410442 | 1.1188844 | down | mRNA |
| ENST00000328834 | CNOT10 | 0.114158039 | 0.558081569 | 1.2136625 | down | mRNA |
| ENST00000328867 | CAPN12 | 0.870402407 | 0.989592579 | 1.00604 | down | mRNA |
| ENST00000328879 | KLHL22 | 0.226464278 | 0.721817166 | 1.2333224 | down | mRNA |
| ENST00000328895 | GKN2 | 0.542680919 | 0.923577434 | 1.2751747 | down | mRNA |
| ENST00000329365 | OR2V1 | 0.94417701 | 0.996710991 | 1.0055953 | down | mRNA |
| ENST00000329446 | C10orf120 | 0.507538292 | 0.911674633 | 1.0879517 | down | mRNA |
| ENST00000329665 | ZSCAN22 | 0.67270368 | 0.959531882 | 1.0743829 | down | mRNA |
| ENST00000330029 | UQCR10 | 0.337626658 | 0.827187565 | 1.0871796 | down | mRNA |
| ENST00000330180 | HIST1H2AK | 0.315765137 | 0.810520991 | 1.8861247 | down | mRNA |
| ENST00000330271 | ACTL10 | 0.024673587 | 0.306827745 | 1.581169 | down | mRNA |
| ENST00000330333 | BACE2 | 0.927381121 | 0.995829678 | 1.0215756 | down | mRNA |
| ENST00000330501 | ZNF397 | 0.010247676 | 0.20936306 | 1.3972056 | down | mRNA |
| ENST00000330687 | CMTM4 | 0.215954352 | 0.710711878 | 1.4087773 | down | mRNA |
| ENST00000330720 | KDELR1 | 0.475080345 | 0.89971438 | 1.0488612 | down | mRNA |
| ENST00000330767 | TMEM95 | 0.721843814 | 0.966191059 | 1.0480106 | down | mRNA |
| ENST00000330793 | CD300C | 0.476411008 | 0.900415348 | 1.0934314 | down | mRNA |
| ENST00000330807 | ALAS2 | 0.734402503 | 0.967777568 | 1.0350619 | down | mRNA |
| ENST00000331056 | PDE6G | 0.990048128 | 0.999140676 | 1.0036694 | down | mRNA |
| ENST00000331128 | HIST2H2AB | 0.911577943 | 0.995342392 | 1.0778439 | down | mRNA |
| ENST00000331203 | FBLN7 | 0.526844252 | 0.917975454 | 1.144224 | down | mRNA |
| ENST00000331563 | PKP3 | 0.055799895 | 0.421575513 | 1.1417899 | down | mRNA |
| ENST00000332112 | EPGN | 0.974294611 | 0.998501532 | 1.0145342 | down | mRNA |
| ENST00000332160 | METTL7A | 0.489870303 | 0.905047069 | 1.2032882 | down | mRNA |
| ENST00000332217 | OR10J3 | 0.830564727 | 0.985549562 | 1.1010785 | down | mRNA |
| ENST00000332235 | C2CD4C | 0.297279038 | 0.79373308 | 1.2764108 | down | mRNA |
| ENST00000332314 | MRGPRG | 0.917098577 | 0.995349793 | 1.0121913 | down | mRNA |
| ENST00000332323 | ZNF534 | 0.155476914 | 0.623056705 | 1.0919348 | down | mRNA |
| ENST00000332372 | FIGLA | 0.016420578 | 0.255922225 | 1.337969 | down | mRNA |
| ENST00000332499 | HEXIM1 | 0.001099504 | 0.086778252 | 1.8030043 | down | mRNA |
| ENST00000332663 | OR4M2 | 0.95320879 | 0.996710991 | 1.0170722 | down | mRNA |
| ENST00000332690 | TAS2R60 | 0.977613442 | 0.998501532 | 1.008392 | down | mRNA |
| ENST00000332715 | PRHOXNB | 0.767700769 | 0.975033576 | 1.0669521 | down | mRNA |
| ENST00000332797 | CCDC28A | 0.448096422 | 0.887964572 | 1.1280271 | down | mRNA |
| ENST00000332822 | NOG | 0.61435754 | 0.944827645 | 1.2380255 | down | mRNA |
| ENST00000332854 | ZNF530 | 0.00082738 | 0.083087642 | 1.5099301 | down | mRNA |
| ENST00000333050 | TMEM186 | 0.630507891 | 0.950410442 | 1.0612306 | down | mRNA |
| ENST00000333175 | C19orf6 | 0.109749063 | 0.54819304 | 1.0819553 | down | mRNA |
| ENST00000333206 | ZSCAN30 | 0.631285315 | 0.950410442 | 1.0516377 | down | mRNA |
| ENST00000333209 | GPRIN3 | 0.396038904 | 0.861849835 | 1.2039755 | down | mRNA |
| ENST00000333274 | EFNA5 | 0.982165336 | 0.998971807 | 1.0016452 | down | mRNA |
| ENST00000333305 | IGIP | 0.198206329 | 0.686763988 | 1.2701184 | down | mRNA |
| ENST00000333625 | IL29 | 0.903597695 | 0.99508468 | 1.0101538 | down | mRNA |
| ENST00000333681 | BCL2 | 0.379930795 | 0.853273195 | 1.161533 | down | mRNA |
| ENST00000333743 | GABRG3 | 0.445556449 | 0.885884384 | 1.0669289 | down | mRNA |
| ENST00000333775 | LSMD1 | 0.528033433 | 0.918844141 | 1.1092481 | down | mRNA |
| ENST00000333861 | CCDC87 | 0.096243529 | 0.51782059 | 1.4716128 | down | mRNA |
| ENST00000333881 | LCE3C | 0.38132791 | 0.853514109 | 1.135399 | down | mRNA |
| ENST00000334163 | POLR3C | 0.072070249 | 0.462687451 | 1.1753073 | down | mRNA |
| ENST00000334241 | ZNF555 | 0.013397991 | 0.23544335 | 1.7660097 | down | mRNA |
| ENST00000334256 | KPNA4 | 0.294711232 | 0.791301949 | 1.0946629 | down | mRNA |
| ENST00000334269 | LCE5A | 0.050741159 | 0.407674476 | 1.3065347 | down | mRNA |
| ENST00000334289 | ANGPTL5 | 0.988307155 | 0.999140676 | 1.0096217 | down | mRNA |
| ENST00000334418 | CCDC84 | 0.997842902 | 0.999889342 | 1.0004173 | down | mRNA |
| ENST00000334528 | FMN1 | 0.863012015 | 0.989401142 | 1.0983969 | down | mRNA |
| ENST00000334568 | TAC4 | 0.139320031 | 0.598648178 | 1.7142878 | down | mRNA |
| ENST00000334678 | RPS19BP1 | 0.630831183 | 0.950410442 | 1.1213687 | down | mRNA |
| ENST00000334793 | FOXD2 | 0.933745162 | 0.996710991 | 1.0093641 | down | mRNA |
| ENST00000334810 | GPR144 | 0.403768024 | 0.865367868 | 1.1996064 | down | mRNA |
| ENST00000334859 | LRCH3 | 0.897279441 | 0.994222243 | 1.0293801 | down | mRNA |
| ENST00000334879 | DEFB131 | 0.740144885 | 0.969794316 | 1.0423851 | down | mRNA |
| ENST00000335144 | SPIN4 | 0.461722883 | 0.894667841 | 1.1402206 | down | mRNA |
| ENST00000335221 | BPTF | 0.314270632 | 0.809623206 | 1.1730337 | down | mRNA |
| ENST00000335351 | TCFL5 | 0.056613213 | 0.424009517 | 1.2148226 | down | mRNA |
| ENST00000335426 | SELV | 0.715991138 | 0.966191059 | 1.0692331 | down | mRNA |
| ENST00000335475 | KCNQ1 | 0.651566303 | 0.956204521 | 1.0357004 | down | mRNA |
| ENST00000335503 | CCT6A | 0.371534578 | 0.846675738 | 1.1422598 | down | mRNA |
| ENST00000335568 | UGT2B28 | 0.318831495 | 0.812850403 | 1.3182357 | down | mRNA |
| ENST00000335633 | LCE3B | 0.663141818 | 0.958354006 | 1.0998994 | down | mRNA |
| ENST00000335674 | LCE3A | 0.920269423 | 0.995349793 | 1.0377131 | down | mRNA |
| ENST00000335757 | SLC44A2 | 0.222473231 | 0.71737156 | 1.1043495 | down | mRNA |
| ENST00000335791 | ST6GALNAC4 | 0.542364636 | 0.923577434 | 1.1767062 | down | mRNA |
| ENST00000335870 | ZNF212 | 0.064591892 | 0.443826413 | 1.5580584 | down | mRNA |
| ENST00000336596 | EPHA3 | 0.858262035 | 0.988512246 | 1.2083139 | down | mRNA |
| ENST00000336614 | ZNF324B | 0.779340377 | 0.976938981 | 1.0196002 | down | mRNA |
| ENST00000337018 | SUGP2 | 0.03112071 | 0.34055236 | 1.2505702 | down | mRNA |
| ENST00000337303 | PBRM1 | 0.049291777 | 0.402199077 | 1.1569533 | down | mRNA |
| ENST00000337491 | SIRT6 | 0.054965058 | 0.419520587 | 1.2424845 | down | mRNA |
| ENST00000337679 | YIF1B | 0.817240373 | 0.981692737 | 1.0406451 | down | mRNA |
| ENST00000337817 | STIL | 0.311854389 | 0.806920827 | 1.555888 | down | mRNA |
| ENST00000337894 | ADORA1 | 0.721674137 | 0.966191059 | 1.0679648 | down | mRNA |
| ENST00000338134 | KPTN | 0.437942386 | 0.882682266 | 1.0583513 | down | mRNA |
| ENST00000338222 | UBQLN2 | 0.140050204 | 0.600105533 | 1.1050901 | down | mRNA |
| ENST00000338376 | FAM82A2 | 0.257275209 | 0.757735833 | 1.173411 | down | mRNA |
| ENST00000338458 | ARHGEF3 | 0.125622513 | 0.578441772 | 1.6305554 | down | mRNA |
| ENST00000338832 | C11orf34 | 0.446844127 | 0.886883521 | 1.1523157 | down | mRNA |
| ENST00000338875 | HELT | 0.7284723 | 0.966191059 | 1.0299523 | down | mRNA |
| ENST00000338961 | PTGES2 | 0.234764196 | 0.732314458 | 1.0558786 | down | mRNA |
| ENST00000339276 | SFN | 0.711673978 | 0.96592341 | 1.2719188 | down | mRNA |
| ENST00000339503 | ZNF573 | 0.001978726 | 0.107570744 | 1.672109 | down | mRNA |
| ENST00000339560 | PODNL1 | 0.46977537 | 0.897941557 | 1.1797644 | down | mRNA |
| ENST00000339697 | SERINC1 | 0.652062852 | 0.956271055 | 1.06465 | down | mRNA |
| ENST00000339818 | C21orf2 | 0.425742889 | 0.87545849 | 1.1185275 | down | mRNA |
| ENST00000340107 | FGFR3 | 0.291998486 | 0.788748934 | 1.2717722 | down | mRNA |
| ENST00000340189 | NCOA3 | 0.135103498 | 0.592787801 | 1.4645139 | down | mRNA |
| ENST00000340192 | ERCC1 | 0.533369706 | 0.920530493 | 1.0471354 | down | mRNA |
| ENST00000340313 | KCNK7 | 0.813195361 | 0.98120545 | 1.0321236 | down | mRNA |
| ENST00000340326 | SH2D6 | 0.650495492 | 0.955894361 | 1.0276271 | down | mRNA |
| ENST00000340363 | C17orf99 | 0.52325194 | 0.917239107 | 1.0646584 | down | mRNA |
| ENST00000340394 | ATXN2L | 0.284527361 | 0.78179061 | 1.0885587 | down | mRNA |
| ENST00000340417 | LIN54 | 0.26089508 | 0.761583524 | 1.3571563 | down | mRNA |
| ENST00000340465 | GFRAL | 0.713329928 | 0.966110889 | 1.0453867 | down | mRNA |
| ENST00000340556 | MORN2 | 0.863165043 | 0.989401142 | 1.0124666 | down | mRNA |
| ENST00000340595 | SLC4A4 | 0.438135301 | 0.882682266 | 1.12471 | down | mRNA |
| ENST00000340650 | NHLRC1 | 0.000708654 | 0.078885568 | 4.187437 | down | mRNA |
| ENST00000340684 | ZNF124 | 0.000903516 | 0.083882581 | 1.3053554 | down | mRNA |
| ENST00000340750 | OR1J4 | 0.845203276 | 0.987338076 | 1.0144949 | down | mRNA |
| ENST00000341228 | SERPINA12 | 0.090819413 | 0.508828962 | 1.1766548 | down | mRNA |
| ENST00000341233 | SLC16A12 | 0.442574763 | 0.884228603 | 1.1939052 | down | mRNA |
| ENST00000341259 | SH2B3 | 0.230889931 | 0.728071705 | 1.1340678 | down | mRNA |
| ENST00000341495 | TREML4 | 0.647503249 | 0.95550188 | 1.0516153 | down | mRNA |
| ENST00000341785 | FAM163A | 0.06482913 | 0.4445551 | 2.0037716 | down | mRNA |
| ENST00000341959 | OR2S2 | 0.363464094 | 0.843238468 | 1.1515842 | down | mRNA |
| ENST00000342071 | MRPL43 | 0.823280711 | 0.983498765 | 1.0461471 | down | mRNA |
| ENST00000342192 | UBE2L3 | 0.391359022 | 0.85910136 | 1.1514014 | down | mRNA |
| ENST00000342303 | METTL15 | 0.947100994 | 0.996710991 | 1.0227041 | down | mRNA |
| ENST00000342704 | CBFA2T2 | 0.011564281 | 0.219901175 | 1.5106516 | down | mRNA |
| ENST00000342784 | SYDE1 | 0.947321962 | 0.996710991 | 1.0118739 | down | mRNA |
| ENST00000342785 | VCAN | 0.099112441 | 0.523009123 | 1.3630121 | down | mRNA |
| ENST00000342907 | ST3GAL2 | 0.852950173 | 0.987678166 | 1.0367149 | down | mRNA |
| ENST00000342922 | MADD | 0.031181202 | 0.340775376 | 1.3372277 | down | mRNA |
| ENST00000343131 | SYCE1 | 0.488069824 | 0.903757389 | 1.062159 | down | mRNA |
| ENST00000343280 | ZNF772 | 0.368973451 | 0.84565745 | 1.0721006 | down | mRNA |
| ENST00000343450 | TADA3 | 0.882590241 | 0.992766819 | 1.018902 | down | mRNA |
| ENST00000343550 | USF2 | 0.076601239 | 0.475659052 | 1.2475022 | down | mRNA |
| ENST00000343625 | RASAL3 | 0.941919129 | 0.996710991 | 1.0189183 | down | mRNA |
| ENST00000343730 | MED22 | 0.01986435 | 0.283521851 | 1.1952021 | down | mRNA |
| ENST00000343769 | ZNF93 | 0.179491717 | 0.661888586 | 1.7010461 | down | mRNA |
| ENST00000343796 | NR3C1 | 0.198734342 | 0.687087939 | 1.3268285 | down | mRNA |
| ENST00000343939 | OSGIN1 | 0.749781097 | 0.970725371 | 1.0423993 | down | mRNA |
| ENST00000343958 | FGD6 | 0.775606006 | 0.97589756 | 1.0504741 | down | mRNA |
| ENST00000344035 | PTP4A2 | 0.115813521 | 0.561697782 | 1.1458493 | down | mRNA |
| ENST00000344517 | ABCB10 | 0.42565403 | 0.87545849 | 1.1390543 | down | mRNA |
| ENST00000344575 | FZD9 | 0.158312065 | 0.628995854 | 1.1782845 | down | mRNA |
| ENST00000344622 | GDNF | 0.464697934 | 0.894989844 | 1.4283996 | down | mRNA |
| ENST00000344642 | LDLRAD2 | 0.298202301 | 0.794528788 | 1.1904119 | down | mRNA |
| ENST00000344762 | CCDC15 | 0.955379916 | 0.996883425 | 1.0218719 | down | mRNA |
| ENST00000344795 | C20orf24 | 0.729329128 | 0.966191059 | 1.0276191 | down | mRNA |
| ENST00000344824 | TPM4 | 0.165611347 | 0.63954604 | 1.1435697 | down | mRNA |
| ENST00000344836 | USP7 | 0.088718702 | 0.503160013 | 1.2067593 | down | mRNA |
| ENST00000345034 | RSC1A1 | 0.046010276 | 0.391970965 | 1.18931 | down | mRNA |
| ENST00000345122 | ARHGAP5 | 0.060841646 | 0.436633496 | 1.1165389 | down | mRNA |
| ENST00000345136 | PLEC | 0.997815796 | 0.999889342 | 1.0008216 | down | mRNA |
| ENST00000345148 | ABCC1 | 0.990107009 | 0.999140676 | 1.0050142 | down | mRNA |
| ENST00000345617 | HDAC4 | 0.104014456 | 0.533896783 | 1.2629077 | down | mRNA |
| ENST00000346057 | EMR2 | 0.53336919 | 0.920530493 | 1.1653858 | down | mRNA |
| ENST00000346370 | ABCC1 | 0.643462057 | 0.954605197 | 1.2025436 | down | mRNA |
| ENST00000346541 | CBFA2T2 | 0.914156061 | 0.995342392 | 1.0221221 | down | mRNA |
| ENST00000346736 | C19orf57 | 0.542017747 | 0.923577434 | 1.1591502 | down | mRNA |
| ENST00000346964 | DLG1 | 0.725760764 | 0.966191059 | 1.0418176 | down | mRNA |
| ENST00000347055 | KRCC1 | 0.085644269 | 0.497540559 | 1.1983002 | down | mRNA |
| ENST00000347077 | ZNF546 | 0.094503202 | 0.51550913 | 1.0907413 | down | mRNA |
| ENST00000347132 | KCNQ4 | 0.553036927 | 0.926337111 | 1.0458132 | down | mRNA |
| ENST00000347230 | ZFYVE26 | 0.46690474 | 0.896167958 | 1.0705771 | down | mRNA |
| ENST00000347667 | BACE2 | 0.772603024 | 0.975566059 | 1.0620242 | down | mRNA |
| ENST00000347970 | TM2D3 | 0.203952669 | 0.693939432 | 1.3409329 | down | mRNA |
| ENST00000348222 | DGUOK | 0.100142328 | 0.525850677 | 1.0709383 | down | mRNA |
| ENST00000348295 | CHEK2 | 0.510639865 | 0.912251098 | 1.0769662 | down | mRNA |
| ENST00000348459 | HELLS | 0.962016344 | 0.99793927 | 1.0292639 | down | mRNA |
| ENST00000348518 | DYX1C1 | 0.575361414 | 0.933656777 | 1.1473637 | down | mRNA |
| ENST00000349029 | ABCC1 | 0.875258674 | 0.991094934 | 1.0626844 | down | mRNA |
| ENST00000349339 | C20orf152 | 0.016269401 | 0.255922225 | 1.1378623 | down | mRNA |
| ENST00000349841 | MYNN | 0.020695963 | 0.286034385 | 1.333434 | down | mRNA |
| ENST00000349893 | COPE | 0.172015053 | 0.649834644 | 1.1769399 | down | mRNA |
| ENST00000350941 | BBS9 | 0.668314434 | 0.958736264 | 1.1182434 | down | mRNA |
| ENST00000351079 | COPE | 0.179023939 | 0.660961453 | 1.1998268 | down | mRNA |
| ENST00000351154 | ABCC1 | 0.52321673 | 0.917239107 | 1.137943 | down | mRNA |
| ENST00000352203 | AP2S1 | 0.244417357 | 0.742713152 | 1.0880908 | down | mRNA |
| ENST00000352665 | WDR16 | 0.667548999 | 0.958354006 | 1.175209 | down | mRNA |
| ENST00000352793 | TAC4 | 0.694572467 | 0.962123409 | 1.1014965 | down | mRNA |
| ENST00000353130 | ABHD12B | 0.135072591 | 0.592787801 | 1.5105384 | down | mRNA |
| ENST00000353145 | RFXANK | 0.404765848 | 0.866165103 | 1.0554857 | down | mRNA |
| ENST00000353284 | PTPRS | 0.387500237 | 0.856773191 | 1.0356104 | down | mRNA |
| ENST00000353379 | CDK10 | 0.694717766 | 0.962202557 | 1.0516569 | down | mRNA |
| ENST00000353414 | COL11A1 | 0.969980619 | 0.998245244 | 1.0495597 | down | mRNA |
| ENST00000353578 | COL6A3 | 0.214578011 | 0.707672362 | 1.100822 | down | mRNA |
| ENST00000353836 | SIGLEC10 | 0.447423662 | 0.887254257 | 1.0668232 | down | mRNA |
| ENST00000354105 | CWF19L1 | 0.41275572 | 0.869353641 | 1.2325323 | down | mRNA |
| ENST00000354190 | PLEKHB1 | 0.833506404 | 0.986434891 | 1.0382083 | down | mRNA |
| ENST00000354249 | NOXO1 | 0.734256942 | 0.967777568 | 1.155687 | down | mRNA |
| ENST00000354308 | SYT5 | 0.904572406 | 0.99508468 | 1.020399 | down | mRNA |
| ENST00000354309 | ZNF805 | 0.098404284 | 0.521845565 | 1.1504596 | down | mRNA |
| ENST00000354313 | ITPK1 | 0.214151903 | 0.706760409 | 1.1845631 | down | mRNA |
| ENST00000354323 | HRCT1 | 0.362434356 | 0.843173209 | 1.7924464 | down | mRNA |
| ENST00000354336 | CRKL | 0.489692244 | 0.904964798 | 1.1126995 | down | mRNA |
| ENST00000354452 | TCF4 | 0.348378133 | 0.834138149 | 1.1030781 | down | mRNA |
| ENST00000354484 | ADAMTSL2 | 0.726368454 | 0.966191059 | 1.0173238 | down | mRNA |
| ENST00000354636 | HMGA2 | 0.995940205 | 0.999872237 | 1.0012963 | down | mRNA |
| ENST00000354958 | PLEC | 0.42681318 | 0.876385133 | 1.151441 | down | mRNA |
| ENST00000355016 | H2BFM | 0.202411939 | 0.691421917 | 1.455531 | down | mRNA |
| ENST00000355059 | NEIL1 | 0.886176611 | 0.992981836 | 1.0086555 | down | mRNA |
| ENST00000355070 | BBS9 | 0.623023044 | 0.948341211 | 1.1538212 | down | mRNA |
| ENST00000355155 | VPS13B | 0.757483568 | 0.972045462 | 1.0475602 | down | mRNA |
| ENST00000355202 | ZNF607 | 0.941316249 | 0.996710991 | 1.0060353 | down | mRNA |
| ENST00000355522 | C2CD4A | 0.420283279 | 0.872365335 | 1.0867715 | down | mRNA |
| ENST00000355537 | ZNF536 | 0.543880408 | 0.923577434 | 1.3526792 | down | mRNA |
| ENST00000355540 | TRRAP | 0.344403085 | 0.831959196 | 1.1640035 | down | mRNA |
| ENST00000355610 | MORC4 | 0.07819726 | 0.480303439 | 1.1175675 | down | mRNA |
| ENST00000355624 | RAB11FIP2 | 0.158399726 | 0.629143041 | 1.1016971 | down | mRNA |
| ENST00000355667 | DNM2 | 0.408898783 | 0.867591329 | 1.0657954 | down | mRNA |
| ENST00000355683 | STRN3 | 0.392692193 | 0.859694706 | 1.1272886 | down | mRNA |
| ENST00000355689 | TTC30A | 0.000520902 | 0.069222072 | 1.3744172 | down | mRNA |
| ENST00000355765 | PRPF39 | 0.644030181 | 0.954605197 | 1.0542054 | down | mRNA |
| ENST00000355774 | MLH3 | 0.06470256 | 0.444345911 | 1.9802479 | down | mRNA |
| ENST00000355977 | MIER1 | 0.263012744 | 0.763125731 | 1.1484817 | down | mRNA |
| ENST00000355981 | HIST1H4L | 0.770531249 | 0.975347132 | 1.3428737 | down | mRNA |
| ENST00000356049 | C13orf35 | 0.05053938 | 0.406628421 | 1.8824004 | down | mRNA |
| ENST00000356178 | ZNF527 | 0.865802435 | 0.989426402 | 1.0236718 | down | mRNA |
| ENST00000356272 | PHLPP2 | 0.357107346 | 0.839540876 | 1.241151 | down | mRNA |
| ENST00000356317 | GALNT6 | 0.601175889 | 0.941028182 | 1.140221 | down | mRNA |
| ENST00000356350 | HIST1H2BH | 0.736922385 | 0.968834005 | 1.3481533 | down | mRNA |
| ENST00000356443 | MYOM1 | 0.621659629 | 0.947997886 | 1.0776054 | down | mRNA |
| ENST00000356487 | GPI | 0.392512026 | 0.859680391 | 1.1104833 | down | mRNA |
| ENST00000356663 | C19orf6 | 0.470670347 | 0.898445976 | 1.0240493 | down | mRNA |
| ENST00000356713 | CNNM1 | 0.069672112 | 0.457632202 | 1.1757807 | down | mRNA |
| ENST00000356725 | ZNF790 | 0.017517348 | 0.266988538 | 1.4481105 | down | mRNA |
| ENST00000356751 | MAMSTR | 0.57534889 | 0.933656777 | 1.0527447 | down | mRNA |
| ENST00000356770 | PBRM1 | 0.139399196 | 0.598648178 | 1.1419003 | down | mRNA |
| ENST00000356818 | PPP1R26 | 0.54288732 | 0.923577434 | 1.0728814 | down | mRNA |
| ENST00000356861 | TNPO2 | 0.194320079 | 0.682224934 | 1.3819309 | down | mRNA |
| ENST00000356926 | AP3D1 | 0.618561579 | 0.946956498 | 1.0402048 | down | mRNA |
| ENST00000356929 | ZNF708 | 0.02552064 | 0.313526074 | 1.1638144 | down | mRNA |
| ENST00000356951 | PRDX3 | 0.046509852 | 0.392706942 | 1.0676085 | down | mRNA |
| ENST00000356972 | SPINK14 | 0.294132829 | 0.791146775 | 1.2372112 | down | mRNA |
| ENST00000357003 | ADPRH | 0.634264429 | 0.951249707 | 1.1516942 | down | mRNA |
| ENST00000357155 | MYBPHL | 0.584849432 | 0.934705544 | 1.1155112 | down | mRNA |
| ENST00000357183 | RAET1E | 0.912743246 | 0.995342392 | 1.0495344 | down | mRNA |
| ENST00000357232 | DCHS2 | 0.215240074 | 0.709072014 | 1.3508409 | down | mRNA |
| ENST00000357244 | RC3H2 | 0.076533368 | 0.475659052 | 1.2073248 | down | mRNA |
| ENST00000357260 | FAM212B | 0.128296689 | 0.581871132 | 1.1899326 | down | mRNA |
| ENST00000357309 | ZNF573 | 0.067102841 | 0.451445852 | 1.2908182 | down | mRNA |
| ENST00000357332 | ASNA1 | 0.71790391 | 0.966191059 | 1.0266053 | down | mRNA |
| ENST00000357368 | PTPRS | 0.955833121 | 0.996883425 | 1.0054794 | down | mRNA |
| ENST00000357410 | EYA2 | 0.008230203 | 0.197897997 | 1.4036335 | down | mRNA |
| ENST00000357574 | WDR7 | 0.342987738 | 0.831521302 | 1.1428674 | down | mRNA |
| ENST00000357649 | PLEC | 0.115113292 | 0.560460597 | 1.1838685 | down | mRNA |
| ENST00000357702 | SAP130 | 0.014261311 | 0.240831369 | 1.5720545 | down | mRNA |
| ENST00000357743 | GOLGA7 | 0.233342697 | 0.731130266 | 1.1191112 | down | mRNA |
| ENST00000357749 | CHM | 0.883952904 | 0.992766819 | 1.0218688 | down | mRNA |
| ENST00000357774 | ZNF98 | 0.008008626 | 0.195524479 | 1.3035533 | down | mRNA |
| ENST00000357814 | NAT9 | 0.000156126 | 0.044621794 | 1.5628693 | down | mRNA |
| ENST00000357838 | COL6A2 | 0.611544747 | 0.943894921 | 1.0811487 | down | mRNA |
| ENST00000358011 | C1orf109 | 0.011444804 | 0.218702965 | 1.4625742 | down | mRNA |
| ENST00000358105 | C4orf22 | 0.907834904 | 0.995145546 | 1.1314304 | down | mRNA |
| ENST00000358204 | TES | 0.73195333 | 0.96727133 | 1.1216594 | down | mRNA |
| ENST00000358301 | PAK4 | 0.531734652 | 0.920228311 | 1.2065528 | down | mRNA |
| ENST00000358350 | FRYL | 0.074489565 | 0.469775665 | 1.1875783 | down | mRNA |
| ENST00000358432 | EPHA2 | 0.11753085 | 0.564147103 | 1.2403391 | down | mRNA |
| ENST00000358465 | TRIM33 | 0.007627771 | 0.193257016 | 1.525301 | down | mRNA |
| ENST00000358491 | ZNF429 | 0.002698957 | 0.122795128 | 1.3668355 | down | mRNA |
| ENST00000358582 | ZNF781 | 0.001050537 | 0.086588663 | 1.3973199 | down | mRNA |
| ENST00000358595 | ARNT | 0.558906661 | 0.927153474 | 1.0356502 | down | mRNA |
| ENST00000358738 | ZBTB1 | 0.043971768 | 0.385691973 | 1.4724861 | down | mRNA |
| ENST00000358769 | PBLD | 0.907711711 | 0.995145546 | 1.0116489 | down | mRNA |
| ENST00000358864 | TMC2 | 0.748634559 | 0.970725371 | 1.0875628 | down | mRNA |
| ENST00000358935 | MARCH5 | 0.574118706 | 0.933656777 | 1.0957363 | down | mRNA |
| ENST00000359204 | FRA10AC1 | 0.20777773 | 0.698556218 | 1.0599508 | down | mRNA |
| ENST00000359332 | KLHDC1 | 0.321808375 | 0.815271211 | 1.1405118 | down | mRNA |
| ENST00000359435 | BABAM1 | 0.544371039 | 0.923577434 | 1.0639759 | down | mRNA |
| ENST00000359465 | HIST1H2BM | 0.876758716 | 0.991255275 | 1.1067932 | down | mRNA |
| ENST00000359568 | PCNT | 0.408895131 | 0.867591329 | 1.234149 | down | mRNA |
| ENST00000359596 | RYR1 | 0.020410201 | 0.285405913 | 1.5237871 | down | mRNA |
| ENST00000359678 | HIBCH | 0.542762478 | 0.923577434 | 1.0455862 | down | mRNA |
| ENST00000359788 | ZNF675 | 0.092755566 | 0.511370653 | 1.7206533 | down | mRNA |
| ENST00000359867 | CSNK1E | 0.191349111 | 0.676939305 | 1.1134098 | down | mRNA |
| ENST00000359907 | HIST1H4A | 0.745773458 | 0.970394407 | 1.1156735 | down | mRNA |
| ENST00000360057 | TPK1 | 0.537384141 | 0.922702335 | 1.0764459 | down | mRNA |
| ENST00000360105 | NFIX | 0.941493986 | 0.996710991 | 1.0089264 | down | mRNA |
| ENST00000360127 | C17orf97 | 0.116240213 | 0.562521166 | 1.4844429 | down | mRNA |
| ENST00000360213 | OR52M1 | 0.98772197 | 0.999140676 | 1.0026924 | down | mRNA |
| ENST00000360338 | ZNF460 | 0.559120168 | 0.927203296 | 1.0451912 | down | mRNA |
| ENST00000360358 | OR2W3 | 0.729871528 | 0.966191059 | 1.0802546 | down | mRNA |
| ENST00000360383 | CDC16 | 0.114658017 | 0.558779196 | 1.1725276 | down | mRNA |
| ENST00000360465 | ZNF528 | 0.339699841 | 0.828117387 | 1.0702831 | down | mRNA |
| ENST00000360614 | LONP1 | 0.227978041 | 0.723854154 | 1.4109891 | down | mRNA |
| ENST00000360689 | FUT8 | 0.4452195 | 0.885734136 | 1.1300738 | down | mRNA |
| ENST00000360797 | PCGF2 | 0.707012425 | 0.965401045 | 1.03576 | down | mRNA |
| ENST00000360839 | ANKHD1 | 0.055957759 | 0.421695017 | 1.2635105 | down | mRNA |
| ENST00000360962 | C11orf80 | 0.475854911 | 0.900175108 | 1.3150041 | down | mRNA |
| ENST00000360971 | ENPP1 | 0.100183949 | 0.525933396 | 1.7863796 | down | mRNA |
| ENST00000361113 | ZNF627 | 0.069872083 | 0.457827646 | 1.3299564 | down | mRNA |
| ENST00000361256 | C9orf114 | 0.496817038 | 0.908665955 | 1.1292516 | down | mRNA |
| ENST00000361371 | LTN1 | 0.033772785 | 0.348587681 | 1.2740252 | down | mRNA |
| ENST00000361421 | TOX | 0.781294144 | 0.976938981 | 1.180903 | down | mRNA |
| ENST00000361428 | ZNF318 | 0.000288756 | 0.054868955 | 1.3165277 | down | mRNA |
| ENST00000361523 | BECN1 | 0.313362551 | 0.808636478 | 1.1278872 | down | mRNA |
| ENST00000361547 | SEPN1 | 0.48603015 | 0.903370052 | 1.0680723 | down | mRNA |
| ENST00000361611 | PSMB5 | 0.657038131 | 0.957445617 | 1.0250605 | down | mRNA |
| ENST00000361677 | RUNDC1 | 0.000948062 | 0.084526081 | 1.5345993 | down | mRNA |
| ENST00000361773 | TGFB1I1 | 0.830243471 | 0.985549562 | 1.0287067 | down | mRNA |
| ENST00000361874 | DCTN1 | 0.219636822 | 0.714276371 | 1.0643731 | down | mRNA |
| ENST00000361908 | OPA1 | 0.352039008 | 0.835686762 | 1.0668352 | down | mRNA |
| ENST00000361919 | PRC1 | 0.556311066 | 0.926833616 | 1.2569014 | down | mRNA |
| ENST00000361923 | PER3 | 0.020227647 | 0.28481199 | 1.4449165 | down | mRNA |
| ENST00000361952 | ZNF830 | 0.356294655 | 0.838932592 | 1.0817812 | down | mRNA |
| ENST00000366618 | SLC35F3 | 0.035946784 | 0.356229271 | 1.22271 | down | mRNA |
| ENST00000366621 | KCNK1 | 0.279440352 | 0.777968199 | 1.3862639 | down | mRNA |
| ENST00000366691 | RHOU | 0.934016499 | 0.996710991 | 1.0130329 | down | mRNA |
| ENST00000366758 | JMJD4 | 0.727582488 | 0.966191059 | 1.0697111 | down | mRNA |
| ENST00000366795 | DACT2 | 0.055195034 | 0.419634622 | 1.4586869 | down | mRNA |
| ENST00000366862 | FBXO28 | 0.118595233 | 0.566426656 | 1.2070302 | down | mRNA |
| ENST00000366882 | PDE10A | 0.323723481 | 0.816213518 | 1.118407 | down | mRNA |
| ENST00000366962 | ANGEL2 | 0.602536958 | 0.941464987 | 1.0430568 | down | mRNA |
| ENST00000366971 | FLVCR1 | 0.327166569 | 0.820196554 | 1.0992862 | down | mRNA |
| ENST00000367165 | CLDN20 | 0.438770828 | 0.882895421 | 1.1805725 | down | mRNA |
| ENST00000367255 | SYNE1 | 0.003126952 | 0.132452472 | 1.5457166 | down | mRNA |
| ENST00000367279 | PTPN7 | 0.943524113 | 0.996710991 | 1.0128844 | down | mRNA |
| ENST00000367287 | TIMM17A | 0.312387972 | 0.807524722 | 1.0705317 | down | mRNA |
| ENST00000367324 | PKP1 | 0.006287729 | 0.179806047 | 2.3917709 | down | mRNA |
| ENST00000367565 | TEDDM1 | 0.940502611 | 0.996710991 | 1.0116886 | down | mRNA |
| ENST00000367569 | SF3B5 | 0.409092358 | 0.867591329 | 1.0761976 | down | mRNA |
| ENST00000367685 | GPR52 | 0.445470492 | 0.885807324 | 1.0959573 | down | mRNA |
| ENST00000367755 | FMO3 | 0.230847458 | 0.728071705 | 1.7638902 | down | mRNA |
| ENST00000368046 | CD48 | 0.562526868 | 0.929041598 | 1.0501571 | down | mRNA |
| ENST00000368090 | PIGM | 0.492737303 | 0.906047853 | 1.1022366 | down | mRNA |
| ENST00000368100 | VSIG8 | 0.68407963 | 0.960111041 | 1.0801672 | down | mRNA |
| ENST00000368102 | C1orf204 | 0.196196871 | 0.684309956 | 1.3730193 | down | mRNA |
| ENST00000368226 | PTPRK | 0.468813163 | 0.897560548 | 1.1314014 | down | mRNA |
| ENST00000368236 | TTC24 | 0.636265881 | 0.951847392 | 1.0206123 | down | mRNA |
| ENST00000368318 | RXFP4 | 0.744359146 | 0.970330526 | 1.0284713 | down | mRNA |
| ENST00000368399 | DPM3 | 0.819252581 | 0.98219491 | 1.0397103 | down | mRNA |
| ENST00000368427 | LENEP | 0.451980198 | 0.888854908 | 1.0901402 | down | mRNA |
| ENST00000368525 | C1orf189 | 0.991830563 | 0.999548897 | 1.0008247 | down | mRNA |
| ENST00000368581 | RSPH4A | 0.64329043 | 0.954522001 | 1.0393323 | down | mRNA |
| ENST00000368605 | FAM26F | 0.676769122 | 0.959531882 | 1.0606429 | down | mRNA |
| ENST00000368779 | LCE2A | 0.8074025 | 0.98118927 | 1.0266868 | down | mRNA |
| ENST00000368799 | FLG | 0.791384953 | 0.978710629 | 1.099418 | down | mRNA |
| ENST00000368849 | TUFT1 | 0.906597905 | 0.99508468 | 1.0202559 | down | mRNA |
| ENST00000368954 | CERS2 | 0.161441303 | 0.633496168 | 1.1323283 | down | mRNA |
| ENST00000368984 | HTRA1 | 0.268336313 | 0.768988803 | 1.0997198 | down | mRNA |
| ENST00000369390 | FAM72B | 0.791394619 | 0.978710629 | 1.0785947 | down | mRNA |
| ENST00000369405 | ZDHHC6 | 0.074596875 | 0.469867247 | 1.1962329 | down | mRNA |
| ENST00000369519 | RBM20 | 0.487243527 | 0.903705199 | 1.1079078 | down | mRNA |
| ENST00000369550 | DKC1 | 0.04615205 | 0.391970965 | 1.1470519 | down | mRNA |
| ENST00000369601 | IKBKG | 0.718117963 | 0.966191059 | 1.0118959 | down | mRNA |
| ENST00000369762 | ATP6AP1 | 0.506240841 | 0.911278334 | 1.0821306 | down | mRNA |
| ENST00000369798 | TTK | 0.429651702 | 0.878226842 | 1.3963962 | down | mRNA |
| ENST00000369809 | DNASE1L1 | 0.040238201 | 0.371915487 | 1.1762709 | down | mRNA |
| ENST00000369839 | TAF5 | 0.121399584 | 0.573089471 | 1.2707989 | down | mRNA |
| ENST00000369847 | PCGF6 | 0.04031012 | 0.371915487 | 1.3772329 | down | mRNA |
| ENST00000369888 | ZNF512B | 0.800372688 | 0.98118927 | 1.0192876 | down | mRNA |
| ENST00000369940 | IRAK1BP1 | 0.468628443 | 0.89739427 | 1.0702317 | down | mRNA |
| ENST00000370053 | STMN3 | 0.941090597 | 0.996710991 | 1.0143717 | down | mRNA |
| ENST00000370074 | NTNG1 | 0.145604491 | 0.607496425 | 1.1020871 | down | mRNA |
| ENST00000370202 | RWDD3 | 0.120484904 | 0.571691731 | 1.1974779 | down | mRNA |
| ENST00000370278 | MAGEA3 | 0.854483577 | 0.988044084 | 1.0168538 | down | mRNA |
| ENST00000370316 | NKAIN4 | 0.75029842 | 0.970725371 | 1.0167146 | down | mRNA |
| ENST00000370331 | EVI5 | 0.159284599 | 0.631041393 | 1.2989062 | down | mRNA |
| ENST00000370346 | BHLHE23 | 0.693757621 | 0.961812388 | 1.0178667 | down | mRNA |
| ENST00000370487 | C20orf20 | 0.059032559 | 0.431899958 | 1.1360429 | down | mRNA |
| ENST00000370498 | SPANXN2 | 0.723079318 | 0.966191059 | 1.0813523 | down | mRNA |
| ENST00000370527 | C20orf166 | 0.542354178 | 0.923577434 | 1.1502094 | down | mRNA |
| ENST00000370532 | CDR1 | 0.092335186 | 0.511338764 | 1.3547275 | down | mRNA |
| ENST00000370597 | CRTAC1 | 0.744369604 | 0.970330526 | 1.0454642 | down | mRNA |
| ENST00000370655 | ANKRD2 | 0.160401635 | 0.631980676 | 1.3159149 | down | mRNA |
| ENST00000370658 | LGSN | 0.964622238 | 0.99793927 | 1.0112984 | down | mRNA |
| ENST00000370689 | PRKACB | 0.074687536 | 0.469867247 | 1.1133543 | down | mRNA |
| ENST00000370888 | LRRC1 | 0.681652439 | 0.960111041 | 1.083043 | down | mRNA |
| ENST00000370932 | PTGER3 | 0.592096344 | 0.937519382 | 1.0358543 | down | mRNA |
| ENST00000371015 | PHACTR3 | 0.619793777 | 0.947444797 | 1.1340554 | down | mRNA |
| ENST00000371018 | MIER1 | 0.483326727 | 0.902884878 | 1.2012281 | down | mRNA |
| ENST00000371060 | LEPR | 0.801955116 | 0.98118927 | 1.1246739 | down | mRNA |
| ENST00000371084 | PGM1 | 0.235762322 | 0.73362711 | 1.0672087 | down | mRNA |
| ENST00000371108 | ALG6 | 0.367846563 | 0.845181673 | 1.1134865 | down | mRNA |
| ENST00000371201 | C1orf87 | 0.943261412 | 0.996710991 | 1.0168475 | down | mRNA |
| ENST00000371325 | FAM209B | 0.814233227 | 0.98120545 | 1.0598188 | down | mRNA |
| ENST00000371486 | CPT2 | 0.000212059 | 0.048871331 | 1.7507057 | down | mRNA |
| ENST00000371527 | NKRF | 0.064576096 | 0.443826413 | 1.3652083 | down | mRNA |
| ENST00000371581 | IDE | 0.024046003 | 0.303619251 | 1.199448 | down | mRNA |
| ENST00000371655 | RAB3B | 0.218728858 | 0.712577334 | 1.223868 | down | mRNA |
| ENST00000371663 | C9orf86 | 0.236415131 | 0.734520115 | 1.0905561 | down | mRNA |
| ENST00000371732 | CARD9 | 0.907754729 | 0.995145546 | 1.0190661 | down | mRNA |
| ENST00000371738 | DNLZ | 0.241219518 | 0.73945497 | 1.0780021 | down | mRNA |
| ENST00000371756 | UBAC1 | 0.040134098 | 0.371915487 | 1.1385121 | down | mRNA |
| ENST00000371763 | GLT6D1 | 0.50870251 | 0.911674633 | 1.2328562 | down | mRNA |
| ENST00000371873 | CMPK1 | 0.161598775 | 0.633561373 | 1.1108633 | down | mRNA |
| ENST00000371929 | ADAMTS13 | 0.783159761 | 0.977275912 | 1.0336899 | down | mRNA |
| ENST00000371933 | KIAA0494 | 0.347991702 | 0.834138149 | 1.0696907 | down | mRNA |
| ENST00000371942 | REXO4 | 0.09544749 | 0.516597924 | 1.2752345 | down | mRNA |
| ENST00000371959 | AMOT | 0.495180748 | 0.907415174 | 1.1605044 | down | mRNA |
| ENST00000372398 | NCS1 | 0.623200239 | 0.948341211 | 1.1095009 | down | mRNA |
| ENST00000372410 | GPR107 | 0.754234789 | 0.970944772 | 1.036629 | down | mRNA |
| ENST00000372422 | YIPF3 | 0.600179471 | 0.940648177 | 1.0252777 | down | mRNA |
| ENST00000372441 | LRRC73 | 0.849220674 | 0.987678166 | 1.0036512 | down | mRNA |
| ENST00000372445 | TJAP1 | 0.248598321 | 0.7470442 | 1.5757799 | down | mRNA |
| ENST00000372581 | GUCA2B | 0.133813454 | 0.590685007 | 1.3290497 | down | mRNA |
| ENST00000372622 | DNTTIP1 | 0.797207238 | 0.980667273 | 1.0280695 | down | mRNA |
| ENST00000372627 | TCEAL3 | 0.734491756 | 0.967777568 | 1.0618228 | down | mRNA |
| ENST00000372677 | BEX2 | 0.712436284 | 0.966072854 | 1.2782995 | down | mRNA |
| ENST00000372699 | ZNF684 | 0.038712834 | 0.367636309 | 2.1947917 | down | mRNA |
| ENST00000372717 | DBNDD2 | 0.495391916 | 0.907415174 | 1.0940777 | down | mRNA |
| ENST00000372789 | WFDC5 | 0.002891824 | 0.12797004 | 1.493216 | down | mRNA |
| ENST00000372966 | NOX1 | 0.549353635 | 0.925481755 | 1.0631462 | down | mRNA |
| ENST00000373006 | USP49 | 0.950292955 | 0.996710991 | 1.0058897 | down | mRNA |
| ENST00000373012 | POU3F1 | 0.967212984 | 0.99793927 | 1.0028298 | down | mRNA |
| ENST00000373053 | MCU | 0.037027232 | 0.359628653 | 1.2574261 | down | mRNA |
| ENST00000373063 | FOXP4 | 0.023863922 | 0.303389731 | 1.2435263 | down | mRNA |
| ENST00000373257 | LPIN3 | 0.859375045 | 0.988512246 | 1.0410608 | down | mRNA |
| ENST00000373263 | ZHX3 | 0.003758974 | 0.142058481 | 1.163947 | down | mRNA |
| ENST00000373359 | MAGEE2 | 0.627045257 | 0.949218083 | 1.0278668 | down | mRNA |
| ENST00000373366 | GJB3 | 0.549567456 | 0.925533435 | 1.1083958 | down | mRNA |
| ENST00000373479 | RNF8 | 0.010633046 | 0.210507384 | 1.4160662 | down | mRNA |
| ENST00000373484 | SYNC | 0.310051773 | 0.804899469 | 1.1605972 | down | mRNA |
| ENST00000373573 | HDAC8 | 0.415144069 | 0.870239791 | 1.1427273 | down | mRNA |
| ENST00000373585 | DDX50 | 0.008450212 | 0.198394583 | 1.226265 | down | mRNA |
| ENST00000373674 | PI16 | 0.975955213 | 0.998501532 | 1.0173929 | down | mRNA |
| ENST00000373693 | CXCR3 | 0.864515337 | 0.989401142 | 1.0249957 | down | mRNA |
| ENST00000373758 | REEP3 | 0.227968531 | 0.723854154 | 1.0602368 | down | mRNA |
| ENST00000373789 | RTKN2 | 0.730655169 | 0.966329458 | 1.1323176 | down | mRNA |
| ENST00000373790 | TAF1 | 0.055724399 | 0.421341944 | 1.4605153 | down | mRNA |
| ENST00000373960 | DES | 0.774067384 | 0.97589756 | 1.10827 | down | mRNA |
| ENST00000373968 | MBL2 | 0.873367233 | 0.990580595 | 1.0477614 | down | mRNA |
| ENST00000374006 | ASAH2B | 0.48348659 | 0.903010541 | 1.3124905 | down | mRNA |
| ENST00000374056 | AGAP6 | 0.395707549 | 0.861639069 | 1.0679357 | down | mRNA |
| ENST00000374095 | AGAP7 | 0.137932075 | 0.596815269 | 1.1182419 | down | mRNA |
| ENST00000374282 | PAFAH2 | 0.584570181 | 0.93461341 | 1.0321151 | down | mRNA |
| ENST00000374325 | SYT15 | 0.915915563 | 0.995349793 | 1.0266054 | down | mRNA |
| ENST00000374550 | RPL11 | 0.040074653 | 0.371915487 | 1.0764888 | down | mRNA |
| ENST00000374694 | FZD8 | 0.002098937 | 0.110028846 | 1.6247136 | down | mRNA |
| ENST00000374762 | NIPSNAP3B | 0.782531365 | 0.977106756 | 1.0467664 | down | mRNA |
| ENST00000374806 | PPP3R2 | 0.414193825 | 0.86961884 | 1.1508903 | down | mRNA |
| ENST00000374864 | ITCH | 0.159066839 | 0.630863036 | 1.1069658 | down | mRNA |
| ENST00000374928 | KLF8 | 0.622981234 | 0.948341211 | 1.0384406 | down | mRNA |
| ENST00000375031 | SH2D5 | 0.585287727 | 0.934708084 | 1.0542936 | down | mRNA |
| ENST00000375135 | FGD1 | 0.099347305 | 0.523708821 | 1.2863037 | down | mRNA |
| ENST00000375241 | CDC14B | 0.843442222 | 0.987338076 | 1.0345229 | down | mRNA |
| ENST00000375360 | PTPDC1 | 0.659743333 | 0.958354006 | 1.1104207 | down | mRNA |
| ENST00000375375 | PAX7 | 0.993895074 | 0.999872237 | 1.0008939 | down | mRNA |
| ENST00000375406 | ACTL8 | 0.465820772 | 0.895570502 | 1.0545196 | down | mRNA |
| ENST00000375419 | C9orf129 | 0.023630362 | 0.302131789 | 1.3584252 | down | mRNA |
| ENST00000375571 | MAPRE1 | 0.723385574 | 0.966191059 | 1.0254699 | down | mRNA |
| ENST00000375730 | POFUT1 | 0.080904075 | 0.486246998 | 1.2359756 | down | mRNA |
| ENST00000375735 | DYNC2H1 | 0.919962957 | 0.995349793 | 1.0106955 | down | mRNA |
| ENST00000375739 | ARHGEF7 | 0.828242533 | 0.985325387 | 1.0390225 | down | mRNA |
| ENST00000375741 | ARHGEF7 | 0.826202254 | 0.984374112 | 1.0226661 | down | mRNA |
| ENST00000375854 | NXNL2 | 0.911374272 | 0.995342392 | 1.0205415 | down | mRNA |
| ENST00000375858 | LY6G5C | 0.853021135 | 0.987678166 | 1.0304841 | down | mRNA |
| ENST00000375910 | CELA2B | 0.348738521 | 0.834138149 | 1.2698523 | down | mRNA |
| ENST00000375978 | FOXS1 | 0.016203531 | 0.255838153 | 4.0091935 | down | mRNA |
| ENST00000376180 | ITGBL1 | 0.992913078 | 0.999798141 | 1.0016265 | down | mRNA |
| ENST00000376207 | FOXP3 | 0.471099994 | 0.898790004 | 1.0865154 | down | mRNA |
| ENST00000376227 | CCDC22 | 0.16383643 | 0.63628397 | 1.1491903 | down | mRNA |
| ENST00000376266 | CCHCR1 | 0.245429507 | 0.743640499 | 1.2697572 | down | mRNA |
| ENST00000376325 | SHISA7 | 0.170787105 | 0.647381721 | 1.0759638 | down | mRNA |
| ENST00000376490 | CLCN6 | 0.52084021 | 0.916605148 | 1.0722736 | down | mRNA |
| ENST00000376521 | SLC35A2 | 0.974466684 | 0.998501532 | 1.0036262 | down | mRNA |
| ENST00000376582 | TIMM17B | 0.574039817 | 0.933656777 | 1.053427 | down | mRNA |
| ENST00000376588 | PSAT1 | 0.914478473 | 0.995342392 | 1.0218446 | down | mRNA |
| ENST00000376741 | SP2 | 0.221504711 | 0.716734257 | 1.0880137 | down | mRNA |
| ENST00000376819 | ANGPTL7 | 0.530107774 | 0.91899174 | 1.0820806 | down | mRNA |
| ENST00000376917 | MOG | 0.695159094 | 0.962214902 | 1.042621 | down | mRNA |
| ENST00000377004 | C1orf127 | 0.634376509 | 0.951260652 | 1.0325432 | down | mRNA |
| ENST00000377075 | CNNM4 | 0.787102389 | 0.977479172 | 1.064466 | down | mRNA |
| ENST00000377205 | NMNAT1 | 0.183226624 | 0.666292349 | 1.1205865 | down | mRNA |
| ENST00000377280 | CGB7 | 0.066465152 | 0.450298982 | 1.6252641 | down | mRNA |
| ENST00000377348 | VPS28 | 0.659626062 | 0.958354006 | 1.0451522 | down | mRNA |
| ENST00000377364 | HIST1H4B | 0.919125146 | 0.995349793 | 1.0746052 | down | mRNA |
| ENST00000377457 | CBWD6 | 0.456947518 | 0.891568474 | 1.1374513 | down | mRNA |
| ENST00000377574 | SLC22A12 | 0.756768983 | 0.971824502 | 1.0743266 | down | mRNA |
| ENST00000377617 | ATXN2 | 0.56790734 | 0.931705674 | 1.107885 | down | mRNA |
| ENST00000377621 | FAM75A5 | 0.506410203 | 0.911362118 | 1.0819544 | down | mRNA |
| ENST00000377671 | ZNF133 | 0.083053318 | 0.491579514 | 1.5675575 | down | mRNA |
| ENST00000377843 | CSNK1A1 | 0.380448859 | 0.853407568 | 1.0991516 | down | mRNA |
| ENST00000378103 | GBA2 | 0.016910656 | 0.260870611 | 1.5622424 | down | mRNA |
| ENST00000378226 | BTBD3 | 0.013377977 | 0.23544335 | 1.5905182 | down | mRNA |
| ENST00000378251 | LRRC47 | 0.529509344 | 0.91899174 | 1.1183281 | down | mRNA |
| ENST00000378288 | TP73 | 0.490586522 | 0.905300886 | 1.067643 | down | mRNA |
| ENST00000378486 | PLCH2 | 0.564549222 | 0.929793142 | 1.1309384 | down | mRNA |
| ENST00000378526 | LIG3 | 0.034851894 | 0.352338753 | 1.1578942 | down | mRNA |
| ENST00000378560 | TCF7 | 0.820423927 | 0.982672123 | 1.0498388 | down | mRNA |
| ENST00000378643 | FANCG | 0.687487517 | 0.960205549 | 1.0962467 | down | mRNA |
| ENST00000378654 | SUCLA2 | 0.906539237 | 0.99508468 | 1.0233258 | down | mRNA |
| ENST00000378679 | SHROOM1 | 0.454308082 | 0.890398297 | 1.0748567 | down | mRNA |
| ENST00000378714 | MCM10 | 0.967356079 | 0.99793927 | 1.0311017 | down | mRNA |
| ENST00000378720 | ESD | 0.663945849 | 0.958354006 | 1.0370952 | down | mRNA |
| ENST00000378821 | TMEM88B | 0.936594199 | 0.996710991 | 1.0128098 | down | mRNA |
| ENST00000378823 | RAD50 | 0.029824596 | 0.335098261 | 1.1812301 | down | mRNA |
| ENST00000378887 | SDHAF1 | 0.009930555 | 0.207085174 | 1.3280643 | down | mRNA |
| ENST00000378944 | ARHGAP33 | 0.698749001 | 0.962701213 | 1.0465752 | down | mRNA |
| ENST00000379406 | NAA16 | 0.04078015 | 0.374838156 | 1.6431241 | down | mRNA |
| ENST00000379426 | TMEM170B | 0.18589698 | 0.669321303 | 1.9909443 | down | mRNA |
| ENST00000379499 | KLHL34 | 0.035144515 | 0.352396712 | 3.1453759 | down | mRNA |
| ENST00000379554 | INSC | 0.091612186 | 0.510719066 | 1.1331034 | down | mRNA |
| ENST00000379751 | CENPB | 0.162120726 | 0.634236412 | 1.2002648 | down | mRNA |
| ENST00000379826 | SMAD9 | 0.172516837 | 0.65011298 | 1.2408905 | down | mRNA |
| ENST00000379881 | SOHLH2 | 0.019594028 | 0.281743828 | 1.1361783 | down | mRNA |
| ENST00000379906 | SLC6A2 | 0.381284569 | 0.853514109 | 9.8897864 | down | mRNA |
| ENST00000379919 | MAB21L1 | 0.235476879 | 0.732851049 | 2.1773372 | down | mRNA |
| ENST00000380191 | GDI2 | 0.147071123 | 0.609351845 | 1.1492511 | down | mRNA |
| ENST00000380332 | CALML5 | 0.356075106 | 0.838847221 | 1.1202319 | down | mRNA |
| ENST00000380516 | SLTM | 0.143080649 | 0.603147721 | 1.1245851 | down | mRNA |
| ENST00000380671 | LCA5L | 0.655046111 | 0.957205183 | 1.1356812 | down | mRNA |
| ENST00000380712 | AMELX | 0.99014782 | 0.999140676 | 1.0050108 | down | mRNA |
| ENST00000380718 | ARHGAP6 | 0.704092788 | 0.963625105 | 1.2150629 | down | mRNA |
| ENST00000380794 | POMC | 0.748704198 | 0.970725371 | 1.0238994 | down | mRNA |
| ENST00000380843 | IDH3B | 0.236968984 | 0.735131735 | 1.06436 | down | mRNA |
| ENST00000380851 | IDH3B | 0.499709832 | 0.909460484 | 1.0351603 | down | mRNA |
| ENST00000380985 | NLN | 0.943646125 | 0.996710991 | 1.0146138 | down | mRNA |
| ENST00000381031 | TTC38 | 0.135547922 | 0.593260206 | 1.2515783 | down | mRNA |
| ENST00000381077 | HDHD1 | 0.598779057 | 0.940648177 | 1.0867623 | down | mRNA |
| ENST00000381951 | OR1A2 | 0.684934367 | 0.960111041 | 1.092423 | down | mRNA |
| ENST00000382021 | DNAJC21 | 0.323400858 | 0.816209812 | 1.3912303 | down | mRNA |
| ENST00000382058 | FBXO7 | 0.735350936 | 0.968017545 | 1.029005 | down | mRNA |
| ENST00000382119 | NANOGNB | 0.044923025 | 0.388835651 | 1.2366477 | down | mRNA |
| ENST00000382159 | GNG7 | 0.381832263 | 0.853514109 | 1.2421449 | down | mRNA |
| ENST00000382160 | KRTAP5-6 | 0.588881337 | 0.935839874 | 1.08119 | down | mRNA |
| ENST00000382171 | KRTAP5-1 | 0.926639073 | 0.99582096 | 1.0190084 | down | mRNA |
| ENST00000382247 | DCAF16 | 0.360283882 | 0.841780454 | 1.0604641 | down | mRNA |
| ENST00000382298 | SACS | 0.023427439 | 0.301091463 | 1.300395 | down | mRNA |
| ENST00000382492 | TAS2R1 | 0.390256983 | 0.858920947 | 1.0784965 | down | mRNA |
| ENST00000382554 | NYNRIN | 0.621261945 | 0.947954811 | 1.2399801 | down | mRNA |
| ENST00000382744 | TMEM211 | 0.644006974 | 0.954605197 | 1.1194621 | down | mRNA |
| ENST00000382865 | POLN | 0.149336176 | 0.61339457 | 1.1915839 | down | mRNA |
| ENST00000382869 | ZMYM2 | 0.026654299 | 0.318484367 | 1.3517237 | down | mRNA |
| ENST00000382932 | SLC7A4 | 0.781143316 | 0.976938981 | 1.0427323 | down | mRNA |
| ENST00000383746 | C3orf23 | 0.32167666 | 0.815271211 | 1.0936499 | down | mRNA |
| ENST00000383759 | VILL | 0.661466065 | 0.958354006 | 1.0896746 | down | mRNA |
| ENST00000383763 | TRIM71 | 0.732975716 | 0.967703318 | 1.1390989 | down | mRNA |
| ENST00000383781 | COLQ | 0.536737524 | 0.922636737 | 1.1196825 | down | mRNA |
| ENST00000383811 | CRELD1 | 0.601812154 | 0.941306617 | 1.0429449 | down | mRNA |
| ENST00000388767 | CORO6 | 0.577359171 | 0.933656777 | 1.1558652 | down | mRNA |
| ENST00000388866 | NOX5 | 0.607362349 | 0.942627675 | 1.1079108 | down | mRNA |
| ENST00000389328 | TRAPPC9 | 0.750014692 | 0.970725371 | 1.0321254 | down | mRNA |
| ENST00000389395 | MFSD12 | 0.19688479 | 0.685161614 | 1.0704033 | down | mRNA |
| ENST00000389474 | SIGLEC15 | 0.805170521 | 0.98118927 | 1.0206347 | down | mRNA |
| ENST00000389534 | ZNF841 | 0.074629348 | 0.469867247 | 1.2697848 | down | mRNA |
| ENST00000389623 | SSC5D | 0.716208172 | 0.966191059 | 1.0335348 | down | mRNA |
| ENST00000389629 | RTF1 | 0.726306969 | 0.966191059 | 1.0280701 | down | mRNA |
| ENST00000389658 | LAMA1 | 0.384875527 | 0.855147437 | 1.0730266 | down | mRNA |
| ENST00000389698 | RALGAPA1 | 0.093300149 | 0.512441525 | 1.2021435 | down | mRNA |
| ENST00000389709 | ZXDC | 0.032776904 | 0.34498156 | 1.3060009 | down | mRNA |
| ENST00000389740 | GPR149 | 0.574750236 | 0.933656777 | 1.0615413 | down | mRNA |
| ENST00000390675 | TAS2R31 | 0.043498941 | 0.384598968 | 1.2478829 | down | mRNA |
| ENST00000391417 | KRTAP4-7 | 0.000517238 | 0.069187399 | 6.2173398 | down | mRNA |
| ENST00000391701 | ZNF552 | 0.192233777 | 0.678323006 | 1.1127307 | down | mRNA |
| ENST00000391718 | ZNF628 | 0.037091763 | 0.359628653 | 1.4814497 | down | mRNA |
| ENST00000391759 | TFPT | 0.18424937 | 0.667764384 | 1.263955 | down | mRNA |
| ENST00000391785 | ZNF701 | 0.575444007 | 0.933656777 | 1.1118186 | down | mRNA |
| ENST00000391916 | DACT3 | 0.602310066 | 0.941406011 | 1.1236051 | down | mRNA |
| ENST00000391959 | PPP1R12B | 0.009016619 | 0.202329498 | 1.1503475 | down | mRNA |
| ENST00000391980 | MTERFD2 | 0.089703817 | 0.506107454 | 1.1588274 | down | mRNA |
| ENST00000392003 | COL6A3 | 0.606508087 | 0.94212869 | 1.1002764 | down | mRNA |
| ENST00000392006 | HNRNPUL1 | 0.581936485 | 0.934005566 | 1.1323268 | down | mRNA |
| ENST00000392017 | ATG16L1 | 0.336712143 | 0.826515911 | 1.0483066 | down | mRNA |
| ENST00000392023 | SPTBN4 | 0.266766331 | 0.767278687 | 1.1348823 | down | mRNA |
| ENST00000392035 | C19orf47 | 0.320963742 | 0.814914437 | 1.6437783 | down | mRNA |
| ENST00000392038 | AKT2 | 0.602572659 | 0.941464987 | 1.0317847 | down | mRNA |
| ENST00000392040 | GPR55 | 0.363763087 | 0.843238468 | 1.1440646 | down | mRNA |
| ENST00000392122 | CD247 | 0.488230572 | 0.903785537 | 1.0731919 | down | mRNA |
| ENST00000392325 | WDR45L | 0.358629388 | 0.840603483 | 1.0907915 | down | mRNA |
| ENST00000392351 | HOMER3 | 0.38516253 | 0.855147437 | 1.0949942 | down | mRNA |
| ENST00000392379 | BDH1 | 0.579494357 | 0.933821303 | 1.0737622 | down | mRNA |
| ENST00000392533 | TAOK3 | 0.515839573 | 0.914429643 | 1.1722446 | down | mRNA |
| ENST00000392607 | GPR123 | 0.641584885 | 0.954332328 | 1.0413625 | down | mRNA |
| ENST00000392736 | MECOM | 0.92250325 | 0.995349793 | 1.0072155 | down | mRNA |
| ENST00000392841 | HMBS | 0.102896807 | 0.532205007 | 1.1895927 | down | mRNA |
| ENST00000392945 | GPSM1 | 0.763862297 | 0.973773636 | 1.064742 | down | mRNA |
| ENST00000392953 | ARHGEF4 | 0.175140725 | 0.653821873 | 1.6090642 | down | mRNA |
| ENST00000393197 | IL1F10 | 0.775380263 | 0.97589756 | 1.0232909 | down | mRNA |
| ENST00000393207 | BANP | 0.125504856 | 0.578441772 | 1.3069879 | down | mRNA |
| ENST00000393306 | OSGIN1 | 0.135425172 | 0.593245913 | 1.4506364 | down | mRNA |
| ENST00000393357 | PHTF1 | 0.352859486 | 0.836121132 | 1.1686275 | down | mRNA |
| ENST00000393387 | PHF3 | 0.292355426 | 0.788954556 | 1.1914529 | down | mRNA |
| ENST00000393408 | CBX1 | 0.186388148 | 0.669888134 | 1.0626343 | down | mRNA |
| ENST00000393409 | PLXNA1 | 0.125252615 | 0.578441772 | 1.0864055 | down | mRNA |
| ENST00000393600 | FAM50A | 0.319905096 | 0.814056373 | 1.039935 | down | mRNA |
| ENST00000393622 | HDAC5 | 0.240797194 | 0.739113608 | 1.3114876 | down | mRNA |
| ENST00000393651 | SRPK2 | 0.570102861 | 0.932828856 | 1.0376621 | down | mRNA |
| ENST00000393733 | MRPL4 | 0.401247671 | 0.864768873 | 1.0888269 | down | mRNA |
| ENST00000393752 | DRD1 | 0.81699387 | 0.981692737 | 1.0584037 | down | mRNA |
| ENST00000393845 | WDR52 | 0.463388889 | 0.894989844 | 1.0789964 | down | mRNA |
| ENST00000393872 | MAGEA2 | 0.172693452 | 0.650463738 | 1.2425842 | down | mRNA |
| ENST00000394003 | DCTN1 | 0.265998142 | 0.766913531 | 1.1357956 | down | mRNA |
| ENST00000394063 | MAPKAP1 | 0.669268654 | 0.95930319 | 1.0216056 | down | mRNA |
| ENST00000394152 | ZNF498 | 0.144709955 | 0.605951408 | 1.1113507 | down | mRNA |
| ENST00000394155 | FHL1 | 0.569173231 | 0.93232103 | 1.1012955 | down | mRNA |
| ENST00000394175 | GSDMB | 0.185280583 | 0.668759953 | 1.3064095 | down | mRNA |
| ENST00000394217 | KCNK7 | 0.633862095 | 0.951174475 | 1.2193715 | down | mRNA |
| ENST00000394264 | FAM122A | 0.073877762 | 0.468039773 | 1.314993 | down | mRNA |
| ENST00000394303 | CACNB1 | 0.956556258 | 0.996883425 | 1.0058291 | down | mRNA |
| ENST00000394353 | GSN | 0.063348086 | 0.440687083 | 1.5984963 | down | mRNA |
| ENST00000394507 | KRIT1 | 0.009929789 | 0.207085174 | 1.2840909 | down | mRNA |
| ENST00000394601 | PTBP1 | 0.963024363 | 0.99793927 | 1.0028411 | down | mRNA |
| ENST00000394703 | DMTF1 | 0.426704537 | 0.876250545 | 1.0233463 | down | mRNA |
| ENST00000394730 | GSTCD | 0.94873694 | 0.996710991 | 1.027314 | down | mRNA |
| ENST00000394755 | EVI2A | 0.994502815 | 0.999872237 | 1.0015426 | down | mRNA |
| ENST00000395003 | PHF15 | 0.025161217 | 0.310235208 | 1.3740975 | down | mRNA |
| ENST00000395095 | FZR1 | 0.856780649 | 0.988512246 | 1.0188326 | down | mRNA |
| ENST00000395146 | AFF1 | 0.075951233 | 0.473352674 | 1.2274099 | down | mRNA |
| ENST00000395172 | C8orf31 | 0.485174585 | 0.903250293 | 1.077492 | down | mRNA |
| ENST00000395251 | ZNF365 | 0.564819724 | 0.929820378 | 1.0880525 | down | mRNA |
| ENST00000395284 | CDK1 | 0.834760338 | 0.986575022 | 1.1547714 | down | mRNA |
| ENST00000395338 | AMT | 0.51021051 | 0.912097614 | 1.1231938 | down | mRNA |
| ENST00000395461 | LAT | 0.127053625 | 0.580406758 | 1.1980025 | down | mRNA |
| ENST00000395510 | TWF1 | 0.264691589 | 0.766079628 | 1.1292974 | down | mRNA |
| ENST00000395562 | CHAT | 0.155618931 | 0.623194202 | 1.09501 | down | mRNA |
| ENST00000395636 | PHACTR3 | 0.757848398 | 0.972072638 | 1.0391229 | down | mRNA |
| ENST00000395644 | C15orf44 | 0.11245981 | 0.554849033 | 1.3382005 | down | mRNA |
| ENST00000395647 | SLC5A10 | 0.364953926 | 0.844553064 | 1.130293 | down | mRNA |
| ENST00000395706 | SMCR7 | 0.745498493 | 0.970394407 | 1.0282268 | down | mRNA |
| ENST00000395715 | TRPS1 | 0.008396881 | 0.198394583 | 1.2739771 | down | mRNA |
| ENST00000395822 | ZBP1 | 0.895239932 | 0.993978977 | 1.0070734 | down | mRNA |
| ENST00000396060 | GTF2A2 | 0.090476617 | 0.508168667 | 1.0692029 | down | mRNA |
| ENST00000396115 | GAS7 | 0.6267376 | 0.949203235 | 1.0522664 | down | mRNA |
| ENST00000396127 | BBS9 | 0.612931743 | 0.944423643 | 1.1591085 | down | mRNA |
| ENST00000396147 | ZNF418 | 0.246633766 | 0.745152522 | 1.22781 | down | mRNA |
| ENST00000396265 | TSPO | 0.344119593 | 0.831843962 | 1.0218157 | down | mRNA |
| ENST00000396602 | ZNF143 | 0.0016068 | 0.103549765 | 1.4293965 | down | mRNA |
| ENST00000396893 | ZNF461 | 0.148070212 | 0.611080968 | 1.1622216 | down | mRNA |
| ENST00000396912 | RBAK | 0.029795999 | 0.33507315 | 1.5677844 | down | mRNA |
| ENST00000396980 | HIST1H2BN | 0.897035226 | 0.994222243 | 1.0841999 | down | mRNA |
| ENST00000397032 | PEPD | 0.636221596 | 0.951847392 | 1.0769289 | down | mRNA |
| ENST00000397054 | LEPRE1 | 0.433072288 | 0.880677205 | 1.0845511 | down | mRNA |
| ENST00000397109 | SEC13 | 0.889520666 | 0.9937521 | 1.0103508 | down | mRNA |
| ENST00000397121 | ZNF676 | 0.087755369 | 0.501332443 | 1.1673684 | down | mRNA |
| ENST00000397126 | ZNF208 | 0.020957649 | 0.287697675 | 1.193717 | down | mRNA |
| ENST00000397162 | ZNF682 | 0.376746679 | 0.85040519 | 1.0794086 | down | mRNA |
| ENST00000397165 | ZNF682 | 0.577844018 | 0.933821303 | 1.0333226 | down | mRNA |
| ENST00000397396 | TSPAN4 | 0.279169618 | 0.777816764 | 1.1055282 | down | mRNA |
| ENST00000397410 | AKAP8L | 0.009478325 | 0.204959264 | 1.2500957 | down | mRNA |
| ENST00000397541 | WEE2 | 0.318155332 | 0.812165395 | 1.2319349 | down | mRNA |
| ENST00000397542 | CDHR5 | 0.944509395 | 0.996710991 | 1.0091651 | down | mRNA |
| ENST00000397547 | SSTR5 | 0.790980808 | 0.978594487 | 1.0399465 | down | mRNA |
| ENST00000397570 | IRF7 | 0.734464857 | 0.967777568 | 1.0651393 | down | mRNA |
| ENST00000397622 | TMEM209 | 0.032444796 | 0.343869895 | 1.1612209 | down | mRNA |
| ENST00000397668 | HOOK2 | 0.022779655 | 0.297426317 | 1.2372111 | down | mRNA |
| ENST00000397762 | RAB2B | 0.234151429 | 0.731403918 | 1.0511085 | down | mRNA |
| ENST00000397766 | PGBD4 | 0.477841982 | 0.901000016 | 1.1207426 | down | mRNA |
| ENST00000397786 | MED13 | 0.467334198 | 0.896646168 | 1.0466712 | down | mRNA |
| ENST00000397907 | KRTAP12-3 | 0.704188431 | 0.963691133 | 1.0721191 | down | mRNA |
| ENST00000398014 | NIPA2 | 0.058049424 | 0.42918578 | 1.1616561 | down | mRNA |
| ENST00000398035 | C11orf93 | 0.402314811 | 0.864786348 | 1.1654204 | down | mRNA |
| ENST00000398039 | HNRNPA1L2 | 0.00177734 | 0.105670067 | 1.5614701 | down | mRNA |
| ENST00000398153 | HDDC2 | 0.436112074 | 0.881677506 | 1.0770726 | down | mRNA |
| ENST00000398243 | S100PBP | 0.054989533 | 0.419520587 | 1.0862431 | down | mRNA |
| ENST00000398250 | PPAPDC1A | 0.22951536 | 0.726627973 | 1.5253454 | down | mRNA |
| ENST00000398253 | ZFP90 | 0.372526095 | 0.84763089 | 1.1220021 | down | mRNA |
| ENST00000398261 | WHSC1 | 0.685847568 | 0.960111041 | 1.0423422 | down | mRNA |
| ENST00000398283 | HDAC2 | 0.170485617 | 0.64730412 | 1.0685581 | down | mRNA |
| ENST00000398534 | KRTAP5-8 | 0.478933785 | 0.901271944 | 1.1629947 | down | mRNA |
| ENST00000398665 | DOT1L | 0.370377792 | 0.846215286 | 1.0675971 | down | mRNA |
| ENST00000398733 | UBE2D2 | 0.254727675 | 0.755011807 | 1.0630013 | down | mRNA |
| ENST00000398793 | TOP3B | 0.435510828 | 0.881627543 | 1.0546607 | down | mRNA |
| ENST00000398802 | SLC25A45 | 0.401646768 | 0.864768873 | 1.0872668 | down | mRNA |
| ENST00000398841 | YPEL3 | 0.787012189 | 0.977479172 | 1.0421416 | down | mRNA |
| ENST00000398958 | RCC1 | 0.153005792 | 0.619205018 | 1.6202283 | down | mRNA |
| ENST00000399107 | POLR3G | 0.072477001 | 0.463255488 | 1.1355719 | down | mRNA |
| ENST00000399231 | MYO5A | 0.136424984 | 0.595125435 | 1.2370057 | down | mRNA |
| ENST00000399332 | HECTD1 | 0.01799553 | 0.269173581 | 1.1613152 | down | mRNA |
| ENST00000399396 | N4BP2L2 | 0.224568517 | 0.719270177 | 1.1839545 | down | mRNA |
| ENST00000399448 | PTPN6 | 0.794036065 | 0.97921643 | 1.0291914 | down | mRNA |
| ENST00000399810 | C5orf49 | 0.673225664 | 0.959531882 | 1.099862 | down | mRNA |
| ENST00000399820 | WDR19 | 0.530854593 | 0.91936419 | 1.1402288 | down | mRNA |
| ENST00000399847 | C18orf1 | 0.678732902 | 0.959891867 | 1.0755404 | down | mRNA |
| ENST00000400335 | KREMEN1 | 0.004518649 | 0.154319835 | 1.2481861 | down | mRNA |
| ENST00000400889 | FAM72D | 0.490361436 | 0.905264563 | 1.31956 | down | mRNA |
| ENST00000400913 | PLEKHG5 | 0.394091487 | 0.861008404 | 1.0656406 | down | mRNA |
| ENST00000400926 | CCR6 | 0.81535687 | 0.981459009 | 1.0572061 | down | mRNA |
| ENST00000401084 | CAPZB | 0.149959538 | 0.614295311 | 1.1091232 | down | mRNA |
| ENST00000401450 | RASL10A | 0.875535838 | 0.991094934 | 1.0133065 | down | mRNA |
| ENST00000401500 | WDR62 | 0.839175518 | 0.987032229 | 1.0487072 | down | mRNA |
| ENST00000401743 | CD14 | 0.400568831 | 0.864582322 | 1.1091098 | down | mRNA |
| ENST00000402077 | KCTD17 | 0.707622063 | 0.965401045 | 1.0575192 | down | mRNA |
| ENST00000402198 | SETD5 | 0.001843737 | 0.105670067 | 1.3002665 | down | mRNA |
| ENST00000402377 | ZNF681 | 0.17283429 | 0.650632622 | 1.0889067 | down | mRNA |
| ENST00000402521 | EFTUD2 | 0.064240826 | 0.443211564 | 1.1264599 | down | mRNA |
| ENST00000402764 | ETV2 | 0.943973827 | 0.996710991 | 1.0078672 | down | mRNA |
| ENST00000403359 | FBXO11 | 0.333373691 | 0.823381801 | 1.8718721 | down | mRNA |
| ENST00000403528 | HCRTR1 | 0.50709179 | 0.911674633 | 1.1333489 | down | mRNA |
| ENST00000404125 | PSME4 | 0.120085955 | 0.570864541 | 1.2715897 | down | mRNA |
| ENST00000404138 | ZBTB40 | 0.534350826 | 0.920652989 | 1.0570582 | down | mRNA |
| ENST00000404279 | LINGO3 | 0.346218538 | 0.833163669 | 1.1336289 | down | mRNA |
| ENST00000404702 | VWA1 | 0.952689362 | 0.996710991 | 1.0148702 | down | mRNA |
| ENST00000404875 | POMT1 | 0.007490794 | 0.192301549 | 1.1292441 | down | mRNA |
| ENST00000405535 | TMEM79 | 0.767660135 | 0.975033576 | 1.0675578 | down | mRNA |
| ENST00000405957 | LSP1 | 0.358468235 | 0.840546205 | 1.3238496 | down | mRNA |
| ENST00000405990 | IDNK | 0.356789536 | 0.839417363 | 1.0727414 | down | mRNA |
| ENST00000406043 | DNAAF2 | 0.000361666 | 0.057900793 | 1.8948995 | down | mRNA |
| ENST00000406242 | FXYD3 | 0.481198471 | 0.901538317 | 1.3099434 | down | mRNA |
| ENST00000406936 | COMMD6 | 0.872172799 | 0.990339403 | 1.0226791 | down | mRNA |
| ENST00000407004 | ILF3 | 0.285131432 | 0.781963593 | 1.1272541 | down | mRNA |
| ENST00000407071 | KIF26B | 0.37608695 | 0.849862269 | 1.3985357 | down | mRNA |
| ENST00000407578 | MYCBP2 | 0.647728946 | 0.95550188 | 1.0568137 | down | mRNA |
| ENST00000407793 | DDX11 | 0.276121556 | 0.775838072 | 1.1804184 | down | mRNA |
| ENST00000407967 | RNFT2 | 0.232396109 | 0.730317988 | 1.2370053 | down | mRNA |
| ENST00000408893 | C1orf229 | 0.04502296 | 0.389144768 | 1.7714702 | down | mRNA |
| ENST00000408934 | PRR21 | 0.996258808 | 0.999872237 | 1.0007023 | down | mRNA |
| ENST00000408941 | SCP2 | 0.556968798 | 0.926833616 | 1.0727533 | down | mRNA |
| ENST00000408946 | WIBG | 0.045235471 | 0.389550017 | 1.3456161 | down | mRNA |
| ENST00000408959 | C3orf71 | 0.194879275 | 0.68256424 | 1.17542 | down | mRNA |
| ENST00000408974 | DNM2 | 0.278462243 | 0.777783404 | 1.2285243 | down | mRNA |
| ENST00000408989 | UMODL1 | 0.203896153 | 0.693939432 | 1.0685076 | down | mRNA |
| ENST00000409703 | FAM115C | 0.67933525 | 0.959969193 | 1.0506262 | down | mRNA |
| ENST00000409745 | MYADML2 | 0.380423535 | 0.853407568 | 1.2604217 | down | mRNA |
| ENST00000409749 | ASB18 | 0.057765207 | 0.428018294 | 1.108156 | down | mRNA |
| ENST00000409877 | C2orf76 | 0.465675561 | 0.895570502 | 1.185041 | down | mRNA |
| ENST00000409895 | PPM1B | 0.373547447 | 0.848504826 | 1.0628691 | down | mRNA |
| ENST00000410061 | RAB6C | 0.285865988 | 0.782635081 | 1.1242579 | down | mRNA |
| ENST00000410062 | UBE2E3 | 0.742921705 | 0.970330526 | 1.0306304 | down | mRNA |
| ENST00000411486 | HJURP | 0.483016549 | 0.902654749 | 1.9372717 | down | mRNA |
| ENST00000411826 | HS3ST5 | 0.630244267 | 0.950410442 | 1.0713123 | down | mRNA |
| ENST00000411986 | CTH | 0.701695537 | 0.963236071 | 1.110588 | down | mRNA |
| ENST00000412055 | PRRT3 | 0.293759685 | 0.791146775 | 1.2193958 | down | mRNA |
| ENST00000412145 | PTPN3 | 0.639544577 | 0.953550763 | 1.1894963 | down | mRNA |
| ENST00000412532 | SLC1A5 | 0.229643791 | 0.726710905 | 1.2826055 | down | mRNA |
| ENST00000412676 | CMKLR1 | 7.77253E-05 | 0.033623631 | 3.117087 | down | mRNA |
| ENST00000412718 | FAM22D | 0.932010566 | 0.996710991 | 1.0064183 | down | mRNA |
| ENST00000413199 | PRR23C | 0.647967997 | 0.95550188 | 1.05274 | down | mRNA |
| ENST00000413330 | RPP30 | 0.029515759 | 0.333953485 | 1.1348652 | down | mRNA |
| ENST00000414350 | GGA1 | 0.359929473 | 0.841449472 | 1.1405209 | down | mRNA |
| ENST00000414423 | WDR26 | 0.132291987 | 0.587843174 | 1.2896202 | down | mRNA |
| ENST00000414468 | ZNF805 | 0.561949463 | 0.928808234 | 1.0388008 | down | mRNA |
| ENST00000414982 | PNPLA6 | 0.411452804 | 0.86887062 | 1.1716607 | down | mRNA |
| ENST00000415331 | CLCC1 | 0.519024906 | 0.916145597 | 1.0308589 | down | mRNA |
| ENST00000416044 | KRTAP25-1 | 0.154945569 | 0.62235348 | 1.2677708 | down | mRNA |
| ENST00000416184 | KLK15 | 0.418518225 | 0.87232061 | 1.2267826 | down | mRNA |
| ENST00000416651 | NIF3L1 | 0.00187865 | 0.105670067 | 1.3171694 | down | mRNA |
| ENST00000416691 | COG6 | 0.328694397 | 0.821614764 | 1.1709708 | down | mRNA |
| ENST00000416983 | MFNG | 0.306632972 | 0.801653799 | 1.2112565 | down | mRNA |
| ENST00000417098 | SATB2 | 0.763379313 | 0.973567683 | 1.1016101 | down | mRNA |
| ENST00000417170 | TADA2A | 0.697495673 | 0.962410025 | 1.049093 | down | mRNA |
| ENST00000417626 | IFRD2 | 0.574030053 | 0.933656777 | 1.2485709 | down | mRNA |
| ENST00000418210 | TMEM64 | 0.827428512 | 0.98496935 | 1.0241393 | down | mRNA |
| ENST00000418263 | CCNA1 | 0.477096533 | 0.900429473 | 1.294819 | down | mRNA |
| ENST00000418425 | LETMD1 | 5.73321E-05 | 0.0304885 | 1.4391855 | down | mRNA |
| ENST00000418587 | ZNF300 | 0.072573946 | 0.463579474 | 1.5549456 | down | mRNA |
| ENST00000420083 | ILF3 | 0.03988872 | 0.371524769 | 1.193226 | down | mRNA |
| ENST00000420846 | CD63 | 0.798300571 | 0.980749101 | 1.0206777 | down | mRNA |
| ENST00000420886 | SBNO1 | 0.334749558 | 0.824597423 | 1.0779843 | down | mRNA |
| ENST00000421025 | ZNF679 | 0.022347616 | 0.2946639 | 1.1634023 | down | mRNA |
| ENST00000421063 | PHYHD1 | 0.912013617 | 0.995342392 | 1.0496016 | down | mRNA |
| ENST00000421176 | ZNF284 | 0.04206109 | 0.379087707 | 1.9463788 | down | mRNA |
| ENST00000421699 | GOLGA2 | 0.66842477 | 0.958759255 | 1.0260119 | down | mRNA |
| ENST00000421832 | CTU1 | 0.062471124 | 0.439458294 | 1.2565363 | down | mRNA |
| ENST00000421852 | TMEM41A | 0.001520174 | 0.100026467 | 1.4503057 | down | mRNA |
| ENST00000422024 | ASB10 | 0.807655809 | 0.98118927 | 1.023055 | down | mRNA |
| ENST00000422325 | ZNF717 | 0.848739555 | 0.987678166 | 1.0400746 | down | mRNA |
| ENST00000422387 | KAZN | 0.703370158 | 0.963625105 | 1.0347003 | down | mRNA |
| ENST00000422611 | SLC12A4 | 0.00509145 | 0.163279737 | 1.1213647 | down | mRNA |
| ENST00000423007 | POMT1 | 0.714265178 | 0.966110889 | 1.0552314 | down | mRNA |
| ENST00000423327 | SPC24 | 0.995547671 | 0.999872237 | 1.0012736 | down | mRNA |
| ENST00000423932 | OR10J1 | 0.95660611 | 0.996883425 | 1.0333004 | down | mRNA |
| ENST00000424082 | RALGPS1 | 0.265717002 | 0.766537754 | 1.0764792 | down | mRNA |
| ENST00000424325 | RPL10 | 0.527756348 | 0.918772333 | 1.0690297 | down | mRNA |
| ENST00000424542 | SLC9A3R2 | 0.00027206 | 0.053149627 | 1.2236329 | down | mRNA |
| ENST00000425133 | FAM104B | 0.992360148 | 0.999781402 | 1.0006418 | down | mRNA |
| ENST00000425175 | SLC4A4 | 0.474255337 | 0.899418411 | 1.080843 | down | mRNA |
| ENST00000425217 | KSR2 | 0.700291477 | 0.962991468 | 1.0758356 | down | mRNA |
| ENST00000425232 | C5orf42 | 0.070442496 | 0.45875619 | 1.1791579 | down | mRNA |
| ENST00000425583 | OCIAD1 | 0.299641503 | 0.795756183 | 1.0899384 | down | mRNA |
| ENST00000425699 | NANOS1 | 0.692714566 | 0.961584652 | 1.0859126 | down | mRNA |
| ENST00000425933 | RGMA | 0.511863661 | 0.912870256 | 1.2061443 | down | mRNA |
| ENST00000426333 | EFTUD2 | 0.748197227 | 0.970725371 | 1.0406529 | down | mRNA |
| ENST00000426426 | TMEM233 | 0.545010773 | 0.924012779 | 1.1320768 | down | mRNA |
| ENST00000426588 | DNAJC7 | 0.066634217 | 0.450526863 | 1.2013311 | down | mRNA |
| ENST00000426744 | ZBTB10 | 0.749728985 | 0.970725371 | 1.0685005 | down | mRNA |
| ENST00000426954 | ZNF419 | 0.010520014 | 0.210032734 | 1.4168546 | down | mRNA |
| ENST00000427047 | NDOR1 | 0.921972553 | 0.995349793 | 1.0077632 | down | mRNA |
| ENST00000427159 | FTSJ3 | 0.086779224 | 0.499950487 | 1.3072392 | down | mRNA |
| ENST00000427266 | LARP1B | 0.004134373 | 0.147175197 | 1.3192937 | down | mRNA |
| ENST00000427304 | ZBTB37 | 0.175552277 | 0.653962789 | 1.271235 | down | mRNA |
| ENST00000427358 | FAHD1 | 0.573772346 | 0.933656777 | 1.0751473 | down | mRNA |
| ENST00000427587 | FAM86A | 0.525427531 | 0.917686109 | 1.0887435 | down | mRNA |
| ENST00000427715 | SLC30A8 | 0.571993093 | 0.933338778 | 1.0748781 | down | mRNA |
| ENST00000428024 | GBA | 0.64844243 | 0.95550188 | 1.0254687 | down | mRNA |
| ENST00000428041 | ARHGAP11B | 0.446363722 | 0.886582845 | 1.4206764 | down | mRNA |
| ENST00000428046 | ADAL | 0.114497895 | 0.558534358 | 1.1207189 | down | mRNA |
| ENST00000428113 | ASB4 | 0.918542904 | 0.995349793 | 1.024154 | down | mRNA |
| ENST00000428701 | HLA-G | 0.976805438 | 0.998501532 | 1.0025164 | down | mRNA |
| ENST00000428876 | MDN1 | 0.000277093 | 0.053149627 | 1.2656571 | down | mRNA |
| ENST00000429154 | ZNF230 | 0.242854808 | 0.741106522 | 1.3091561 | down | mRNA |
| ENST00000429455 | GPSM1 | 0.81255011 | 0.98118927 | 1.0340697 | down | mRNA |
| ENST00000429604 | ZNF160 | 0.113426479 | 0.556915522 | 1.2111303 | down | mRNA |
| ENST00000430026 | IL12RB1 | 0.451865662 | 0.888854908 | 1.0519473 | down | mRNA |
| ENST00000430056 | SIM2 | 0.587100181 | 0.93579682 | 1.144471 | down | mRNA |
| ENST00000430368 | TCTN3 | 0.127879827 | 0.581220503 | 1.1390714 | down | mRNA |
| ENST00000430658 | KCNQ2 | 0.502406563 | 0.910335149 | 1.2450769 | down | mRNA |
| ENST00000430935 | TTC26 | 0.753521494 | 0.970725371 | 1.037456 | down | mRNA |
| ENST00000431261 | C15orf44 | 0.760275627 | 0.972883253 | 1.0504109 | down | mRNA |
| ENST00000431522 | ABCC4 | 0.273622314 | 0.773861301 | 1.0535254 | down | mRNA |
| ENST00000431532 | ZNHIT6 | 0.06064602 | 0.435971562 | 1.2115341 | down | mRNA |
| ENST00000431837 | IRAK4 | 0.140399313 | 0.600490836 | 1.1972251 | down | mRNA |
| ENST00000432196 | KBTBD13 | 0.931141858 | 0.996710991 | 1.0188783 | down | mRNA |
| ENST00000432347 | LARP1B | 0.514511647 | 0.913824266 | 1.2267285 | down | mRNA |
| ENST00000432496 | TBCK | 0.310459631 | 0.805412658 | 1.2274655 | down | mRNA |
| ENST00000432887 | TCP11L1 | 0.809752624 | 0.98118927 | 1.03315 | down | mRNA |
| ENST00000432921 | ARHGAP5 | 0.144905176 | 0.606253761 | 1.2410211 | down | mRNA |
| ENST00000432999 | ZBED5 | 0.000823839 | 0.083087642 | 1.7075706 | down | mRNA |
| ENST00000433218 | HOMER3 | 0.01013702 | 0.208872123 | 1.1395338 | down | mRNA |
| ENST00000433375 | GLIS2 | 0.002185513 | 0.11120755 | 1.4049395 | down | mRNA |
| ENST00000433627 | LSM14A | 0.284175519 | 0.78179061 | 1.1806167 | down | mRNA |
| ENST00000433688 | C11orf95 | 0.780063876 | 0.976938981 | 1.0354934 | down | mRNA |
| ENST00000433756 | EFCAB1 | 0.84550484 | 0.987338076 | 1.050153 | down | mRNA |
| ENST00000433765 | CHRM4 | 0.719762495 | 0.966191059 | 1.0430514 | down | mRNA |
| ENST00000434396 | ANKRD63 | 0.267292265 | 0.768030857 | 1.1554141 | down | mRNA |
| ENST00000434425 | RFC3 | 0.917577574 | 0.995349793 | 1.0806019 | down | mRNA |
| ENST00000434704 | DLX3 | 0.224934016 | 0.719865752 | 1.3385558 | down | mRNA |
| ENST00000434726 | SLC1A5 | 0.310262865 | 0.805036317 | 1.2836581 | down | mRNA |
| ENST00000434752 | C5orf65 | 0.228613997 | 0.724692827 | 1.1048269 | down | mRNA |
| ENST00000434772 | ZNF223 | 0.020012425 | 0.283521851 | 1.7225204 | down | mRNA |
| ENST00000435152 | KLK3 | 0.858268878 | 0.988512246 | 1.0103949 | down | mRNA |
| ENST00000435359 | KIAA0664 | 0.924780142 | 0.995456159 | 1.0100914 | down | mRNA |
| ENST00000435364 | CSRP2BP | 0.109061206 | 0.546839071 | 2.4809408 | down | mRNA |
| ENST00000435634 | EXOSC6 | 0.542049246 | 0.923577434 | 1.0247294 | down | mRNA |
| ENST00000435680 | MTMR10 | 0.721425855 | 0.966191059 | 1.0337137 | down | mRNA |
| ENST00000435703 | PRDX2 | 0.412315214 | 0.869163012 | 1.2052435 | down | mRNA |
| ENST00000435817 | FCHSD1 | 0.518436926 | 0.916145597 | 1.1005365 | down | mRNA |
| ENST00000435989 | ZNF814 | 0.026426186 | 0.318338323 | 1.1749348 | down | mRNA |
| ENST00000436370 | PEPD | 0.409205125 | 0.867591329 | 1.040985 | down | mRNA |
| ENST00000436587 | C1GALT1 | 0.48458772 | 0.903250293 | 1.0851627 | down | mRNA |
| ENST00000436615 | RAPGEFL1 | 0.721368888 | 0.966191059 | 1.0976325 | down | mRNA |
| ENST00000436816 | MFAP3 | 0.829295066 | 0.985532347 | 1.0321757 | down | mRNA |
| ENST00000436874 | VEZT | 0.810249172 | 0.98118927 | 1.0281105 | down | mRNA |
| ENST00000437048 | VPS53 | 0.493967951 | 0.906630188 | 1.1144248 | down | mRNA |
| ENST00000437180 | RUNX1 | 0.471559935 | 0.898908705 | 1.1673713 | down | mRNA |
| ENST00000437720 | DPF1 | 0.84990978 | 0.987678166 | 1.0249463 | down | mRNA |
| ENST00000438097 | UBE3A | 0.169632638 | 0.646121878 | 1.1398653 | down | mRNA |
| ENST00000438272 | ULBP3 | 0.323807382 | 0.816222625 | 1.2512359 | down | mRNA |
| ENST00000438926 | SLC12A3 | 0.622588378 | 0.948341211 | 1.1992787 | down | mRNA |
| ENST00000439096 | BBC3 | 0.951708342 | 0.996710991 | 1.0154512 | down | mRNA |
| ENST00000439461 | ZNF766 | 0.02220341 | 0.293936579 | 1.4264495 | down | mRNA |
| ENST00000439576 | ACAN | 0.760505323 | 0.972931992 | 1.0815642 | down | mRNA |
| ENST00000439632 | NARG2 | 0.317443401 | 0.811824899 | 1.1849438 | down | mRNA |
| ENST00000439858 | TMEM125 | 0.757918535 | 0.972072638 | 1.0322993 | down | mRNA |
| ENST00000440549 | SLC39A6 | 0.926146649 | 0.995699344 | 1.0122935 | down | mRNA |
| ENST00000440823 | GALP | 0.593820317 | 0.938299318 | 1.0306695 | down | mRNA |
| ENST00000440944 | GPSM1 | 0.04042634 | 0.372623133 | 1.2107021 | down | mRNA |
| ENST00000441594 | HHATL | 0.932713757 | 0.996710991 | 1.0200674 | down | mRNA |
| ENST00000441599 | SHBG | 0.981593342 | 0.998971807 | 1.0017297 | down | mRNA |
| ENST00000441611 | BLOC1S2 | 0.015804626 | 0.25282428 | 1.2440703 | down | mRNA |
| ENST00000441788 | FZR1 | 0.725554275 | 0.966191059 | 1.0458742 | down | mRNA |
| ENST00000442253 | PAQR8 | 0.564880057 | 0.929820378 | 1.0766975 | down | mRNA |
| ENST00000442919 | TLK1 | 0.142735572 | 0.602781779 | 1.21009 | down | mRNA |
| ENST00000443585 | C12orf75 | 0.797984084 | 0.980749101 | 1.0677228 | down | mRNA |
| ENST00000444444 | SIRPB2 | 0.033766846 | 0.348587681 | 1.4551811 | down | mRNA |
| ENST00000444449 | AP1M1 | 0.08367241 | 0.493003289 | 1.1623154 | down | mRNA |
| ENST00000444723 | C20orf173 | 0.585926633 | 0.93505319 | 1.0978463 | down | mRNA |
| ENST00000444736 | PNPLA4 | 0.968729366 | 0.998196842 | 1.0106184 | down | mRNA |
| ENST00000444864 | TSEN2 | 0.896991413 | 0.994222243 | 1.0129704 | down | mRNA |
| ENST00000445369 | CLDN9 | 0.8324518 | 0.986218684 | 1.0318326 | down | mRNA |
| ENST00000445590 | FAM177B | 0.937067545 | 0.996710991 | 1.0240322 | down | mRNA |
| ENST00000445927 | C2orf61 | 0.62074213 | 0.947874051 | 1.0871986 | down | mRNA |
| ENST00000446148 | ZNF300 | 0.441525625 | 0.883920737 | 1.0770226 | down | mRNA |
| ENST00000447098 | PTCH2 | 0.98787213 | 0.999140676 | 1.0023058 | down | mRNA |
| ENST00000447275 | PDE4C | 0.462081905 | 0.894754673 | 1.1860029 | down | mRNA |
| ENST00000447306 | PNMA3 | 0.79761979 | 0.980721189 | 1.1002372 | down | mRNA |
| ENST00000447716 | RAB34 | 0.12801492 | 0.581241479 | 1.1262867 | down | mRNA |
| ENST00000447866 | FDPS | 0.971977385 | 0.998501532 | 1.0034465 | down | mRNA |
| ENST00000448110 | DSN1 | 0.942937706 | 0.996710991 | 1.0312929 | down | mRNA |
| ENST00000448116 | SHC1 | 0.151569242 | 0.616711191 | 1.5576233 | down | mRNA |
| ENST00000448616 | FIBCD1 | 0.689560138 | 0.96108227 | 1.0712169 | down | mRNA |
| ENST00000449624 | COASY | 0.031258414 | 0.340775376 | 1.1773948 | down | mRNA |
| ENST00000449653 | COMT | 0.948899122 | 0.996710991 | 1.0102453 | down | mRNA |
| ENST00000449934 | MICA | 0.518419308 | 0.916145597 | 1.0627389 | down | mRNA |
| ENST00000450222 | ATXN1 | 0.907091883 | 0.995145546 | 1.0072242 | down | mRNA |
| ENST00000450318 | NUSAP1 | 0.84963855 | 0.987678166 | 1.1146627 | down | mRNA |
| ENST00000450764 | TNPO2 | 0.698278209 | 0.962600349 | 1.0917081 | down | mRNA |
| ENST00000451127 | TEX22 | 0.929095345 | 0.996456574 | 1.0251788 | down | mRNA |
| ENST00000451237 | B3GALT4 | 0.983197084 | 0.998971807 | 1.0045328 | down | mRNA |
| ENST00000452194 | HSF2 | 0.010763059 | 0.21287405 | 1.3879128 | down | mRNA |
| ENST00000452215 | ARL5A | 0.062551629 | 0.439458294 | 1.1614608 | down | mRNA |
| ENST00000452318 | CGREF1 | 0.678606761 | 0.959891867 | 1.0565454 | down | mRNA |
| ENST00000452723 | CDK5RAP1 | 0.006175532 | 0.178714838 | 1.2635032 | down | mRNA |
| ENST00000452825 | KDM5C | 0.17602252 | 0.655114384 | 1.0946355 | down | mRNA |
| ENST00000453009 | IFT27 | 0.80407855 | 0.98118927 | 1.0118806 | down | mRNA |
| ENST00000453514 | PAPD4 | 0.25870427 | 0.759358563 | 1.1580809 | down | mRNA |
| ENST00000453741 | ZNF611 | 0.001260088 | 0.092399617 | 1.4709957 | down | mRNA |
| ENST00000453908 | SH3D21 | 0.426585177 | 0.876202345 | 1.4314085 | down | mRNA |
| ENST00000454036 | SLC12A5 | 0.276004074 | 0.775741613 | 1.0845303 | down | mRNA |
| ENST00000454319 | ZNF566 | 0.008243815 | 0.197897997 | 1.2679947 | down | mRNA |
| ENST00000454366 | GTSE1 | 0.781101586 | 0.976938981 | 1.1963334 | down | mRNA |
| ENST00000454822 | RPE | 0.115680767 | 0.561475615 | 1.0968997 | down | mRNA |
| ENST00000454903 | FXYD3 | 0.293414016 | 0.790681746 | 1.0926241 | down | mRNA |
| ENST00000455511 | ANAPC7 | 0.024060355 | 0.303619251 | 1.2211465 | down | mRNA |
| ENST00000455600 | RUSC2 | 0.085877583 | 0.498103045 | 1.2867577 | down | mRNA |
| ENST00000455780 | PPCS | 0.542968326 | 0.923577434 | 1.0790967 | down | mRNA |
| ENST00000456098 | IGF1 | 0.163897992 | 0.636313534 | 1.7293385 | down | mRNA |
| ENST00000456783 | ZNF492 | 0.500879131 | 0.909680616 | 1.4893219 | down | mRNA |
| ENST00000456984 | AGAP9 | 0.050275945 | 0.405800127 | 1.0717693 | down | mRNA |
| ENST00000457167 | DNAJC7 | 0.02001022 | 0.283521851 | 1.3077888 | down | mRNA |
| ENST00000457222 | TSPY3 | 0.833432435 | 0.986434891 | 1.0360795 | down | mRNA |
| ENST00000457422 | CCDC43 | 0.089858178 | 0.506514131 | 1.2041683 | down | mRNA |
| ENST00000457842 | ZBTB47 | 0.208959652 | 0.700205031 | 1.245783 | down | mRNA |
| ENST00000458035 | ZNF845 | 0.00110116 | 0.086778252 | 1.5085407 | down | mRNA |
| ENST00000458469 | NCS1 | 0.8409843 | 0.987068842 | 1.0329367 | down | mRNA |
| ENST00000460041 | HOXB-AS3 | 0.13588183 | 0.594011904 | 1.275577 | down | mRNA |
| ENST00000460270 | CREM | 0.052292262 | 0.412415155 | 1.239412 | down | mRNA |
| ENST00000465301 | RGAG1 | 0.078431428 | 0.480427915 | 1.3320871 | down | mRNA |
| ENST00000465846 | HOXB-AS3 | 0.789140268 | 0.977807732 | 1.1366586 | down | mRNA |
| ENST00000466380 | NR1I2 | 0.258463902 | 0.759296063 | 1.2395929 | down | mRNA |
| ENST00000467087 | STIM2 | 0.273084537 | 0.772770327 | 1.0656197 | down | mRNA |
| ENST00000467773 | NOBOX | 0.174593718 | 0.653144337 | 1.0894709 | down | mRNA |
| ENST00000469243 | DZIP1L | 0.058737427 | 0.430953947 | 1.1321889 | down | mRNA |
| ENST00000470540 | C1orf198 | 0.403092089 | 0.864786348 | 1.0930592 | down | mRNA |
| ENST00000473414 | COMMD2 | 0.117028333 | 0.56398437 | 1.1299503 | down | mRNA |
| ENST00000473942 | NUDT9 | 0.85325531 | 0.987678166 | 1.0264392 | down | mRNA |
| ENST00000474362 | CREM | 0.579039175 | 0.933821303 | 1.1535485 | down | mRNA |
| ENST00000477084 | FAM120C | 0.073083382 | 0.464789281 | 1.307746 | down | mRNA |
| ENST00000477616 | KCNC3 | 0.960747137 | 0.997798988 | 1.0064338 | down | mRNA |
| ENST00000482061 | C3orf33 | 0.409221441 | 0.867591329 | 1.435872 | down | mRNA |
| ENST00000484524 | NOS3 | 0.871981747 | 0.990243379 | 1.0123085 | down | mRNA |
| ENST00000485286 | MCM7 | 0.845760424 | 0.987338076 | 1.0901561 | down | mRNA |
| ENST00000491143 | ONECUT2 | 0.485610461 | 0.903250293 | 1.1385167 | down | mRNA |
| ENST00000502935 | XPNPEP1 | 0.000921817 | 0.084526081 | 1.2719468 | down | mRNA |
| ENST00000502986 | IDO2 | 0.443837732 | 0.88506363 | 1.1222002 | down | mRNA |
| ENST00000504548 | TIGD5 | 9.56252E-05 | 0.036683999 | 1.8539234 | down | mRNA |
| ENST00000504595 | FBXL7 | 0.900032689 | 0.994752372 | 1.0317278 | down | mRNA |
| ENST00000504681 | B4GALNT2 | 0.029505803 | 0.333953485 | 1.2897937 | down | mRNA |
| ENST00000504924 | ARL15 | 0.324288567 | 0.816625605 | 1.1256797 | down | mRNA |
| ENST00000506868 | TAS2R50 | 0.539757755 | 0.923577434 | 1.0720423 | down | mRNA |
| ENST00000509018 | RAPGEF6 | 0.140285201 | 0.600457367 | 1.1014629 | down | mRNA |
| ENST00000517417 | PCDHGA1 | 0.775709613 | 0.97589756 | 1.0460984 | down | mRNA |
| ENST00000517434 | PCDHGA6 | 0.884289166 | 0.992766819 | 1.0234178 | down | mRNA |
| ENST00000518325 | PCDHGA7 | 0.280266856 | 0.778821055 | 1.2376406 | down | mRNA |
| ENST00000519479 | PCDHGB4 | 0.950089706 | 0.996710991 | 1.010656 | down | mRNA |
| ENST00000520272 | DDHD2 | 0.001036519 | 0.086588663 | 1.3257351 | down | mRNA |
| ENST00000520296 | ANKRD65 | 0.707385998 | 0.965401045 | 1.0766915 | down | mRNA |
| ENST00000521271 | CPNE3 | 0.391252178 | 0.859096647 | 1.0817597 | down | mRNA |
| ENST00000521764 | NACA2 | 0.443001241 | 0.884304813 | 1.0683253 | down | mRNA |
| ENST00000522917 | FER1L6 | 0.860246298 | 0.988947627 | 1.0190778 | down | mRNA |
| ENST00000523500 | CLU | 0.451495644 | 0.888854908 | 1.1499414 | down | mRNA |
| ENST00000525354 | ZNF726 | 0.843491283 | 0.987338076 | 1.0259654 | down | mRNA |
| ENST00000528880 | HCAR3 | 0.555981804 | 0.926833616 | 1.0763118 | down | mRNA |
| ENST00000528956 | WDR65 | 0.24156518 | 0.739795638 | 1.1860441 | down | mRNA |
| ENST00000529221 | TTC12 | 0.682622971 | 0.960111041 | 1.1059526 | down | mRNA |
| ENST00000529803 | OMP | 0.619467193 | 0.947444797 | 1.0302288 | down | mRNA |
| ENST00000531224 | BCLAF1 | 0.338676714 | 0.827403747 | 1.0388814 | down | mRNA |
| ENST00000531526 | GYLTL1B | 0.720753318 | 0.966191059 | 1.0132824 | down | mRNA |
| ENST00000531678 | TAS2R43 | 0.146099887 | 0.608122555 | 1.3182657 | down | mRNA |
| ENST00000533357 | MPZ | 0.495185078 | 0.907415174 | 1.0386049 | down | mRNA |
| ENST00000533467 | TAS2R46 | 0.024734386 | 0.307244129 | 1.1498372 | down | mRNA |
| ENST00000534940 | ASMTL | 0.032669398 | 0.344901399 | 1.4739139 | down | mRNA |
| ENST00000535358 | C1orf228 | 0.224966899 | 0.719865752 | 1.165204 | down | mRNA |
| ENST00000535381 | C5orf63 | 0.775531876 | 0.97589756 | 1.0410034 | down | mRNA |
| ENST00000535737 | FHL1 | 0.071147625 | 0.46104346 | 1.1782082 | down | mRNA |
| ENST00000535842 | TTLL9 | 0.677812023 | 0.959531882 | 1.0225707 | down | mRNA |
| ENST00000536055 | ATAD3A | 0.707684543 | 0.965421506 | 1.1999255 | down | mRNA |
| ENST00000536252 | UBAP1 | 0.271617576 | 0.771734007 | 1.1754746 | down | mRNA |
| ENST00000536359 | TMEM185A | 0.279300186 | 0.777816764 | 1.1055503 | down | mRNA |
| ENST00000537045 | DGCR2 | 0.276723483 | 0.777011718 | 1.5857516 | down | mRNA |
| ENST00000537687 | WNK1 | 0.490130688 | 0.905264563 | 1.0471726 | down | mRNA |
| ENST00000537716 | CD320 | 0.384452294 | 0.85485898 | 1.0495025 | down | mRNA |
| ENST00000537763 | RAB19 | 0.400196449 | 0.864237287 | 1.0547676 | down | mRNA |
| ENST00000537971 | TSPAN9 | 0.853189941 | 0.987678166 | 1.0103364 | down | mRNA |
| ENST00000538396 | FAM195B | 0.895680032 | 0.993978977 | 1.0115528 | down | mRNA |
| ENST00000538489 | UHMK1 | 0.041007053 | 0.375308608 | 1.2104594 | down | mRNA |
| ENST00000538906 | ZNF668 | 0.649361726 | 0.95550188 | 1.0565836 | down | mRNA |
| ENST00000539002 | NDUFC1 | 0.36987149 | 0.846215286 | 1.0725808 | down | mRNA |
| ENST00000539068 | FKBP5 | 0.94303362 | 0.996710991 | 1.0123231 | down | mRNA |
| ENST00000539173 | ADARB1 | 0.358898388 | 0.840767187 | 1.3460432 | down | mRNA |
| ENST00000539214 | OAT | 0.804249076 | 0.98118927 | 1.0409636 | down | mRNA |
| ENST00000539225 | TJP2 | 0.689929301 | 0.961105466 | 1.0673295 | down | mRNA |
| ENST00000539286 | BRI3 | 0.951034591 | 0.996710991 | 1.0041156 | down | mRNA |
| ENST00000539719 | ZNF362 | 0.231122974 | 0.728531274 | 1.1295227 | down | mRNA |
| ENST00000540122 | HDHD1 | 0.435860293 | 0.881677506 | 1.0527244 | down | mRNA |
| ENST00000540331 | ZNF701 | 0.533866098 | 0.92058057 | 1.2419223 | down | mRNA |
| ENST00000541284 | CNOT4 | 0.60014944 | 0.940648177 | 1.1241099 | down | mRNA |
| ENST00000541685 | DPF3 | 0.893663469 | 0.993978977 | 1.0384658 | down | mRNA |
| ENST00000541709 | DRP2 | 0.526619796 | 0.917975454 | 1.0737844 | down | mRNA |
| ENST00000541722 | GLRB | 0.729609529 | 0.966191059 | 1.0734914 | down | mRNA |
| ENST00000541742 | SMYD3 | 0.126036538 | 0.578441772 | 1.2388528 | down | mRNA |
| ENST00000542322 | GRAMD3 | 0.933402901 | 0.996710991 | 1.0074261 | down | mRNA |
| ENST00000542575 | SLC1A5 | 0.340885226 | 0.828854153 | 1.2023617 | down | mRNA |
| ENST00000542887 | ITPRIPL1 | 0.307277417 | 0.802168809 | 1.1370596 | down | mRNA |
| ENST00000543067 | CNKSR2 | 0.455437581 | 0.891148383 | 1.1288744 | down | mRNA |
| ENST00000543500 | MTRNR2L3 | 0.799323158 | 0.980917337 | 1.0483475 | down | mRNA |
| ENST00000543571 | LATS1 | 0.343930288 | 0.831629639 | 1.0756338 | down | mRNA |
| ENST00000543753 | KRTCAP3 | 0.032739509 | 0.344901399 | 1.2383562 | down | mRNA |
| ENST00000543825 | OR5V1 | 0.95418907 | 0.996710991 | 1.0103656 | down | mRNA |
| ENST00000543944 | UBAP1 | 0.027649367 | 0.32381669 | 1.1790736 | down | mRNA |
| ENST00000544106 | MYNN | 0.006089877 | 0.177646158 | 1.3154296 | down | mRNA |
| ENST00000544216 | LSM14A | 0.248959139 | 0.747319103 | 1.2271394 | down | mRNA |
| ENST00000544396 | GRAMD3 | 0.762349677 | 0.97339948 | 1.0202097 | down | mRNA |
| ENST00000544766 | MAGEC3 | 0.216262148 | 0.71103525 | 1.200016 | down | mRNA |
| ENST00000544824 | MTRNR2L7 | 0.938749013 | 0.996710991 | 1.0111947 | down | mRNA |
| ENST00000544865 | SMG6 | 0.676263973 | 0.959531882 | 1.0786583 | down | mRNA |
| ENST00000545075 | MTRNR2L10 | 0.834091333 | 0.986475536 | 1.0367428 | down | mRNA |
| ENST00000545221 | OR4D2 | 0.938471806 | 0.996710991 | 1.0300059 | down | mRNA |
| ENST00000545260 | PARD3 | 0.419521694 | 0.872365335 | 1.0667613 | down | mRNA |
| ENST00000545693 | PARD3 | 0.602284577 | 0.941406011 | 1.062736 | down | mRNA |
| ENST00000545875 | GOLGA3 | 0.43298662 | 0.88061258 | 1.1876944 | down | mRNA |
| ENST00000546954 | TRIAP1 | 0.134230209 | 0.590871682 | 1.1333536 | down | mRNA |
| ENST00000551505 | RP11-144F15.1 | 0.529250935 | 0.91899174 | 1.0525037 | down | mRNA |
| ENST00000555147 | MC1R | 0.005563454 | 0.169833068 | 2.0702567 | down | mRNA |
| ENST00000555248 | YAF2 | 0.497189105 | 0.908789022 | 1.0894427 | down | mRNA |
| ENST00000555709 | MTHFD1 | 0.640832946 | 0.953894897 | 1.0830766 | down | mRNA |
| ENST00000557342 | SLIRP | 0.959496668 | 0.99758736 | 1.0072964 | down | mRNA |
| ENST00000557640 | RD3L | 0.983963141 | 0.998971807 | 1.0081121 | down | mRNA |
| ENST00000558318 | SHISA9 | 0.656001361 | 0.957343034 | 1.1333728 | down | mRNA |
| ENST00000560104 | BLID | 0.916371729 | 0.995349793 | 1.010844 | down | mRNA |
| ENST00000561100 | GPR176 | 0.988099182 | 0.999140676 | 1.001642 | down | mRNA |
| ENST00000561626 | ELFN1 | 0.588588145 | 0.93579682 | 1.0396952 | down | mRNA |
| ENST00000562787 | CENPT | 0.706685925 | 0.965401045 | 1.1471319 | down | mRNA |
| ENST00000564831 | APOBR | 0.262462757 | 0.763125731 | 1.1485202 | down | mRNA |
| ENST00000575751 | OR1D5 | 0.639984025 | 0.953734314 | 1.0582868 | down | mRNA |
| ENST00000576860 | AC103702.1 | 0.651092226 | 0.956204521 | 1.1435248 | down | mRNA |
| ENST00000584306 | LRRC37A3 | 0.569874913 | 0.932738884 | 1.0629528 | down | mRNA |
| NM_000015 | NAT2 | 0.188515741 | 0.673885712 | 1.4360884 | down | mRNA |
| NM_000016 | ACADM | 0.009514582 | 0.204959264 | 1.2944087 | down | mRNA |
| NM_000017 | ACADS | 0.790861465 | 0.978565927 | 1.0213082 | down | mRNA |
| NM_000018 | ACADVL | 0.2709823 | 0.770905786 | 1.1297008 | down | mRNA |
| NM_000019 | ACAT1 | 0.759469959 | 0.972695635 | 1.0283402 | down | mRNA |
| NM_000020 | ACVRL1 | 0.491312347 | 0.905806259 | 1.0611398 | down | mRNA |
| NM_000022 | ADA | 0.019134112 | 0.278080601 | 1.1321049 | down | mRNA |
| NM_000024 | ADRB2 | 0.842468113 | 0.987182488 | 1.1454513 | down | mRNA |
| NM_000026 | ADSL | 0.63499053 | 0.951399945 | 1.0456281 | down | mRNA |
| NM_000027 | AGA | 0.023953999 | 0.303389731 | 1.2347172 | down | mRNA |
| NM_000030 | AGXT | 0.920005987 | 0.995349793 | 1.0220588 | down | mRNA |
| NM_000031 | ALAD | 0.683225324 | 0.960111041 | 1.0624579 | down | mRNA |
| NM_000037 | ANK1 | 0.352007704 | 0.835686762 | 1.2287805 | down | mRNA |
| NM_000040 | APOC3 | 0.088407105 | 0.502587299 | 1.253228 | down | mRNA |
| NM_000048 | ASL | 0.190735424 | 0.676402188 | 1.443825 | down | mRNA |
| NM_000057 | BLM | 0.811316857 | 0.98118927 | 1.160942 | down | mRNA |
| NM_000060 | BTD | 0.150300188 | 0.615241279 | 1.1956942 | down | mRNA |
| NM_000062 | SERPING1 | 0.439581202 | 0.883054028 | 1.3444139 | down | mRNA |
| NM_000067 | CA2 | 0.298723191 | 0.795233272 | 1.2355608 | down | mRNA |
| NM_000071 | CBS | 0.88357064 | 0.992766819 | 1.0617147 | down | mRNA |
| NM_000075 | CDK4 | 0.121364157 | 0.573055281 | 1.0894165 | down | mRNA |
| NM_000076 | CDKN1C | 0.636001651 | 0.951814727 | 1.2305259 | down | mRNA |
| NM_000080 | CHRNE | 0.349136457 | 0.834138149 | 1.2482019 | down | mRNA |
| NM_000082 | ERCC8 | 0.086810231 | 0.499950487 | 1.1303182 | down | mRNA |
| NM_000088 | COL1A1 | 0.279104218 | 0.777816764 | 1.1307064 | down | mRNA |
| NM_000090 | COL3A1 | 0.844967269 | 0.987338076 | 1.0930732 | down | mRNA |
| NM_000092 | COL4A4 | 0.461293767 | 0.89460367 | 1.3143545 | down | mRNA |
| NM_000093 | COL5A1 | 0.537460233 | 0.922702335 | 1.1061367 | down | mRNA |
| NM_000095 | COMP | 0.432894509 | 0.88061258 | 1.1261525 | down | mRNA |
| NM_000097 | CPOX | 0.174457095 | 0.652930084 | 1.240677 | down | mRNA |
| NM_000100 | CSTB | 0.01052733 | 0.210032734 | 1.046019 | down | mRNA |
| NM_000101 | CYBA | 0.845902056 | 0.987338076 | 1.0835154 | down | mRNA |
| NM_000104 | CYP1B1 | 0.833819789 | 0.986475536 | 1.0539093 | down | mRNA |
| NM_000106 | CYP2D6 | 0.796444083 | 0.980606652 | 1.0124039 | down | mRNA |
| NM_000107 | DDB2 | 0.955845737 | 0.996883425 | 1.0052102 | down | mRNA |
| NM_000108 | DLD | 0.097127065 | 0.519544195 | 1.0948489 | down | mRNA |
| NM_000110 | DPYD | 0.299923393 | 0.795797471 | 1.208797 | down | mRNA |
| NM_000112 | SLC26A2 | 0.007924915 | 0.194835995 | 1.4260884 | down | mRNA |
| NM_000113 | TOR1A | 0.438050326 | 0.882682266 | 1.0630826 | down | mRNA |
| NM_000116 | TAZ | 0.515368014 | 0.914319064 | 1.1416582 | down | mRNA |
| NM_000117 | EMD | 0.625930227 | 0.948882596 | 1.0983492 | down | mRNA |
| NM_000118 | ENG | 0.037092184 | 0.359628653 | 1.1950819 | down | mRNA |
| NM_000124 | ERCC6 | 0.806275186 | 0.98118927 | 1.0805612 | down | mRNA |
| NM_000134 | FABP2 | 0.480228381 | 0.901538317 | 1.0708054 | down | mRNA |
| NM_000135 | FANCA | 0.635995694 | 0.951814727 | 1.3017198 | down | mRNA |
| NM_000136 | FANCC | 0.624484742 | 0.948341211 | 1.1421677 | down | mRNA |
| NM_000138 | FBN1 | 0.102017135 | 0.530353463 | 1.0956014 | down | mRNA |
| NM_000140 | FECH | 0.581497942 | 0.934005566 | 1.0733434 | down | mRNA |
| NM_000147 | FUCA1 | 0.845101237 | 0.987338076 | 1.0333185 | down | mRNA |
| NM_000155 | GALT | 0.973092484 | 0.998501532 | 1.0033153 | down | mRNA |
| NM_000156 | GAMT | 0.26034436 | 0.760753309 | 1.0645497 | down | mRNA |
| NM_000158 | GBE1 | 0.349531511 | 0.834138149 | 1.0941448 | down | mRNA |
| NM_000159 | GCDH | 0.314736934 | 0.809824265 | 1.0294882 | down | mRNA |
| NM_000165 | GJA1 | 0.940726782 | 0.996710991 | 1.0127274 | down | mRNA |
| NM_000171 | GLRA1 | 0.583626827 | 0.934280817 | 1.0755433 | down | mRNA |
| NM_000174 | GP9 | 0.082541967 | 0.490238261 | 1.4380097 | down | mRNA |
| NM_000177 | GSN | 0.553249051 | 0.926337111 | 1.0949586 | down | mRNA |
| NM_000178 | GSS | 0.933031864 | 0.996710991 | 1.0064579 | down | mRNA |
| NM_000183 | HADHB | 0.724430873 | 0.966191059 | 1.0502931 | down | mRNA |
| NM_000187 | HGD | 0.901887216 | 0.994906068 | 1.0164066 | down | mRNA |
| NM_000189 | HK2 | 0.016602231 | 0.257480213 | 1.1893464 | down | mRNA |
| NM_000190 | HMBS | 0.762438417 | 0.973431579 | 1.0492325 | down | mRNA |
| NM_000191 | HMGCL | 0.541454997 | 0.923577434 | 1.0734587 | down | mRNA |
| NM_000193 | SHH | 0.583449654 | 0.934280817 | 1.1273042 | down | mRNA |
| NM_000194 | HPRT1 | 0.957820847 | 0.996933183 | 1.0104388 | down | mRNA |
| NM_000195 | HPS1 | 0.929556427 | 0.996533591 | 1.010685 | down | mRNA |
| NM_000199 | SGSH | 0.528113349 | 0.918844141 | 1.0847235 | down | mRNA |
| NM_000204 | CFI | 0.890327169 | 0.9937521 | 1.0488449 | down | mRNA |
| NM_000210 | ITGA6 | 0.920862232 | 0.995349793 | 1.0274622 | down | mRNA |
| NM_000221 | KHK | 0.345041589 | 0.832489094 | 1.2618237 | down | mRNA |
| NM_000224 | KRT18 | 0.349675258 | 0.834138149 | 1.12122 | down | mRNA |
| NM_000229 | LCAT | 0.140246475 | 0.600440374 | 1.1015809 | down | mRNA |
| NM_000232 | SGCB | 0.433962587 | 0.880923255 | 1.0428081 | down | mRNA |
| NM_000233 | LHCGR | 0.338788227 | 0.827403747 | 1.3470087 | down | mRNA |
| NM_000245 | MET | 0.713644417 | 0.966110889 | 1.116732 | down | mRNA |
| NM_000246 | CIITA | 0.664477624 | 0.958354006 | 1.1215308 | down | mRNA |
| NM_000247 | MICA | 0.473770313 | 0.899411578 | 1.0508291 | down | mRNA |
| NM_000248 | MITF | 0.648633435 | 0.95550188 | 1.1532893 | down | mRNA |
| NM_000249 | MLH1 | 0.569894549 | 0.932738884 | 1.0868217 | down | mRNA |
| NM_000254 | MTR | 0.193391279 | 0.680621781 | 1.1382148 | down | mRNA |
| NM_000255 | MUT | 0.124057291 | 0.577766088 | 1.2089724 | down | mRNA |
| NM_000261 | MYOC | 0.022329537 | 0.294616576 | 1.3327373 | down | mRNA |
| NM_000262 | NAGA | 0.349376565 | 0.834138149 | 1.1001116 | down | mRNA |
| NM_000268 | NF2 | 0.284018867 | 0.78179061 | 1.1014726 | down | mRNA |
| NM_000270 | PNP | 0.337432972 | 0.827187565 | 1.1125148 | down | mRNA |
| NM_000272 | NPHP1 | 0.088636542 | 0.502998249 | 1.2325153 | down | mRNA |
| NM_000274 | OAT | 0.297167495 | 0.793643704 | 1.0860566 | down | mRNA |
| NM_000275 | OCA2 | 0.238469852 | 0.736444734 | 1.0896351 | down | mRNA |
| NM_000276 | OCRL | 0.063532665 | 0.440919074 | 1.2366288 | down | mRNA |
| NM_000277 | PAH | 0.094978358 | 0.516214612 | 1.2085343 | down | mRNA |
| NM_000282 | PCCA | 0.222829298 | 0.71761506 | 1.2153068 | down | mRNA |
| NM_000284 | PDHA1 | 0.326375948 | 0.819345076 | 1.0755679 | down | mRNA |
| NM_000287 | PEX6 | 0.5195937 | 0.916187634 | 1.1305329 | down | mRNA |
| NM_000291 | PGK1 | 0.349560866 | 0.834138149 | 1.0867981 | down | mRNA |
| NM_000292 | PHKA2 | 0.511317307 | 0.912481293 | 1.2626235 | down | mRNA |
| NM_000293 | PHKB | 0.572315016 | 0.933397932 | 1.0858693 | down | mRNA |
| NM_000294 | PHKG2 | 0.346799805 | 0.833567116 | 1.074006 | down | mRNA |
| NM_000297 | PKD2 | 0.29182754 | 0.788496683 | 1.3665608 | down | mRNA |
| NM_000302 | PLOD1 | 0.104543206 | 0.535513021 | 1.1647528 | down | mRNA |
| NM_000305 | PON2 | 0.268382085 | 0.768988803 | 1.0783713 | down | mRNA |
| NM_000308 | CTSA | 0.87967684 | 0.992178089 | 1.0235557 | down | mRNA |
| NM_000309 | PPOX | 0.09027715 | 0.507468901 | 1.4283519 | down | mRNA |
| NM_000310 | PPT1 | 0.150408886 | 0.615491439 | 1.2259944 | down | mRNA |
| NM_000312 | PROC | 0.752925282 | 0.970725371 | 1.0647347 | down | mRNA |
| NM_000313 | PROS1 | 0.151288003 | 0.616500055 | 1.2080441 | down | mRNA |
| NM_000314 | PTEN | 0.435111912 | 0.881404713 | 1.1049234 | down | mRNA |
| NM_000317 | PTS | 0.60762359 | 0.942627675 | 1.0322812 | down | mRNA |
| NM_000321 | RB1 | 0.363804167 | 0.843238468 | 1.0648672 | down | mRNA |
| NM_000329 | RPE65 | 0.478429075 | 0.901271944 | 1.0529622 | down | mRNA |
| NM_000333 | ATXN7 | 0.12109924 | 0.572400147 | 1.3783952 | down | mRNA |
| NM_000342 | SLC4A1 | 0.071662779 | 0.462237305 | 1.2759962 | down | mRNA |
| NM_000346 | SOX9 | 0.723002141 | 0.966191059 | 1.0409473 | down | mRNA |
| NM_000348 | SRD5A2 | 0.616272941 | 0.945912508 | 1.1414697 | down | mRNA |
| NM_000351 | STS | 0.739412642 | 0.969564098 | 1.0188359 | down | mRNA |
| NM_000355 | TCN2 | 0.080043146 | 0.483876854 | 2.1363561 | down | mRNA |
| NM_000356 | TCOF1 | 0.949530348 | 0.996710991 | 1.0051738 | down | mRNA |
| NM_000358 | TGFBI | 0.593157655 | 0.938046215 | 1.1090125 | down | mRNA |
| NM_000360 | TH | 0.711957033 | 0.96592341 | 1.1212427 | down | mRNA |
| NM_000361 | THBD | 0.280010717 | 0.778697234 | 1.2981648 | down | mRNA |
| NM_000362 | TIMP3 | 0.282167363 | 0.779872438 | 1.4252856 | down | mRNA |
| NM_000365 | TPI1 | 0.511678334 | 0.912662856 | 1.1073639 | down | mRNA |
| NM_000366 | TPM1 | 0.379629747 | 0.852981768 | 1.1546234 | down | mRNA |
| NM_000368 | TSC1 | 0.000355771 | 0.057900793 | 1.4606368 | down | mRNA |
| NM_000373 | UMPS | 0.613872746 | 0.944827645 | 1.0577684 | down | mRNA |
| NM_000376 | VDR | 0.141592718 | 0.601858131 | 1.8877413 | down | mRNA |
| NM_000380 | XPA | 0.017148327 | 0.263302002 | 1.2028666 | down | mRNA |
| NM_000382 | ALDH3A2 | 0.712362622 | 0.966072854 | 1.0519 | down | mRNA |
| NM_000383 | AIRE | 0.244366084 | 0.742705433 | 1.1480918 | down | mRNA |
| NM_000386 | BLMH | 0.014473002 | 0.242674797 | 1.2068645 | down | mRNA |
| NM_000387 | SLC25A20 | 0.066165715 | 0.449693785 | 1.2519719 | down | mRNA |
| NM_000391 | TPP1 | 0.207365839 | 0.698556218 | 1.0879368 | down | mRNA |
| NM_000394 | CRYAA | 0.39126672 | 0.859096647 | 1.0507935 | down | mRNA |
| NM_000400 | ERCC2 | 0.005410667 | 0.16872651 | 1.1634127 | down | mRNA |
| NM_000401 | EXT2 | 0.53605096 | 0.922302595 | 1.0378112 | down | mRNA |
| NM_000404 | GLB1 | 0.427705108 | 0.876992614 | 1.2568106 | down | mRNA |
| NM_000409 | GUCA1A | 0.487544467 | 0.903757389 | 1.2738229 | down | mRNA |
| NM_000410 | HFE | 0.001774665 | 0.105670067 | 1.3096312 | down | mRNA |
| NM_000411 | HLCS | 0.57710066 | 0.933656777 | 1.049005 | down | mRNA |
| NM_000413 | HSD17B1 | 0.009515742 | 0.204959264 | 2.1118058 | down | mRNA |
| NM_000416 | IFNGR1 | 0.737484139 | 0.968965205 | 1.0571629 | down | mRNA |
| NM_000420 | KEL | 0.781913021 | 0.976963215 | 1.0394778 | down | mRNA |
| NM_000422 | KRT17 | 0.603352467 | 0.941484604 | 1.1462308 | down | mRNA |
| NM_000424 | KRT5 | 0.513408277 | 0.91350662 | 1.0773738 | down | mRNA |
| NM_000427 | LOR | 0.849871429 | 0.987678166 | 1.02591 | down | mRNA |
| NM_000430 | PAFAH1B1 | 0.433377122 | 0.880817674 | 1.0822662 | down | mRNA |
| NM_000432 | MYL2 | 0.839876332 | 0.987068842 | 1.0202877 | down | mRNA |
| NM_000434 | NEU1 | 0.400941498 | 0.864768873 | 1.1303309 | down | mRNA |
| NM_000442 | PECAM1 | 0.630861256 | 0.950410442 | 1.3325744 | down | mRNA |
| NM_000445 | PLEC | 0.064018031 | 0.442124523 | 1.3746527 | down | mRNA |
| NM_000447 | PSEN2 | 0.45938308 | 0.892904132 | 1.0906367 | down | mRNA |
| NM_000453 | SLC5A5 | 0.21332181 | 0.705704366 | 1.120029 | down | mRNA |
| NM_000457 | HNF4A | 0.848675643 | 0.987678166 | 1.034378 | down | mRNA |
| NM_000459 | TEK | 0.560855182 | 0.928003546 | 1.1837435 | down | mRNA |
| NM_000462 | UBE3A | 0.763417002 | 0.973567683 | 1.0371862 | down | mRNA |
| NM_000465 | BARD1 | 0.251096159 | 0.750876415 | 1.2836415 | down | mRNA |
| NM_000480 | AMPD3 | 0.906686768 | 0.995128494 | 1.0106092 | down | mRNA |
| NM_000482 | APOA4 | 0.240038392 | 0.73834502 | 1.0599147 | down | mRNA |
| NM_000484 | APP | 0.858381661 | 0.988512246 | 1.0316437 | down | mRNA |
| NM_000485 | APRT | 0.74585506 | 0.970394407 | 1.0433795 | down | mRNA |
| NM_000489 | ATRX | 0.278110731 | 0.777440204 | 1.1511134 | down | mRNA |
| NM_000497 | CYP11B1 | 0.70954141 | 0.965462762 | 1.0437295 | down | mRNA |
| NM_000501 | ELN | 0.455655055 | 0.891148383 | 1.2816789 | down | mRNA |
| NM_000504 | F10 | 0.384231444 | 0.854819314 | 1.3311234 | down | mRNA |
| NM_000510 | FSHB | 0.886842376 | 0.993077367 | 1.0344653 | down | mRNA |
| NM_000516 | GNAS | 0.358360746 | 0.840546205 | 1.1222257 | down | mRNA |
| NM_000518 | HBB | 0.415397533 | 0.870382888 | 1.4674529 | down | mRNA |
| NM_000520 | HEXA | 0.704593848 | 0.964116167 | 1.043018 | down | mRNA |
| NM_000524 | HTR1A | 0.180007704 | 0.662747609 | 1.6909014 | down | mRNA |
| NM_000526 | KRT14 | 0.745854551 | 0.970394407 | 1.2047101 | down | mRNA |
| NM_000527 | LDLR | 0.155549667 | 0.623056705 | 1.1193798 | down | mRNA |
| NM_000532 | PCCB | 0.739669728 | 0.969564098 | 1.0444491 | down | mRNA |
| NM_000534 | PMS1 | 0.450604051 | 0.888707108 | 1.1525798 | down | mRNA |
| NM_000535 | PMS2 | 0.076221275 | 0.474504275 | 1.367697 | down | mRNA |
| NM_000538 | RFXAP | 0.265038032 | 0.766318724 | 1.2307518 | down | mRNA |
| NM_000543 | SMPD1 | 0.38664547 | 0.856062164 | 1.1403402 | down | mRNA |
| NM_000548 | TSC2 | 0.132179274 | 0.587843174 | 1.1557697 | down | mRNA |
| NM_000553 | WRN | 0.631666993 | 0.95056275 | 1.0319436 | down | mRNA |
| NM_000557 | GDF5 | 0.096119943 | 0.51782059 | 1.5748117 | down | mRNA |
| NM_000582 | SPP1 | 0.83022468 | 0.985549562 | 1.028381 | down | mRNA |
| NM_000596 | IGFBP1 | 0.88258913 | 0.992766819 | 1.0915513 | down | mRNA |
| NM_000609 | CXCL12 | 0.515437934 | 0.914319064 | 1.2934712 | down | mRNA |
| NM_000612 | IGF2 | 0.189787282 | 0.675788971 | 1.3538616 | down | mRNA |
| NM_000613 | HPX | 0.044623472 | 0.387894155 | 1.3483301 | down | mRNA |
| NM_000618 | IGF1 | 0.63463496 | 0.951279916 | 1.3364509 | down | mRNA |
| NM_000620 | NOS1 | 0.626417195 | 0.949203235 | 1.3044927 | down | mRNA |
| NM_000627 | LTBP1 | 0.78776787 | 0.977534106 | 1.0957914 | down | mRNA |
| NM_000637 | GSR | 0.052892251 | 0.413038171 | 1.1276908 | down | mRNA |
| NM_000638 | VTN | 0.848422039 | 0.987678166 | 1.0261764 | down | mRNA |
| NM_000639 | FASLG | 0.678751002 | 0.959891867 | 1.0185563 | down | mRNA |
| NM_000641 | IL11 | 0.65812359 | 0.958354006 | 1.0301518 | down | mRNA |
| NM_000645 | AGL | 0.008520362 | 0.199121848 | 1.2173505 | down | mRNA |
| NM_000646 | AGL | 0.252486362 | 0.752621656 | 1.1093981 | down | mRNA |
| NM_000660 | TGFB1 | 0.143016035 | 0.603147721 | 1.2294312 | down | mRNA |
| NM_000666 | ACY1 | 0.335141917 | 0.824952235 | 1.3222174 | down | mRNA |
| NM_000671 | ADH5 | 0.434922458 | 0.881287963 | 1.169492 | down | mRNA |
| NM_000675 | ADORA2A | 0.165810166 | 0.639669852 | 1.1472748 | down | mRNA |
| NM_000677 | ADORA3 | 0.11910227 | 0.568314329 | 1.4008764 | down | mRNA |
| NM_000679 | ADRA1B | 0.167717585 | 0.642432918 | 1.1133991 | down | mRNA |
| NM_000681 | ADRA2A | 0.315222524 | 0.810240865 | 1.4413021 | down | mRNA |
| NM_000683 | ADRA2C | 1.76579E-05 | 0.018895843 | 1.8374003 | down | mRNA |
| NM_000694 | ALDH3B1 | 0.094373952 | 0.51525542 | 1.2559715 | down | mRNA |
| NM_000696 | ALDH9A1 | 0.391898497 | 0.859445464 | 1.1342908 | down | mRNA |
| NM_000698 | ALOX5 | 0.669465744 | 0.959440157 | 1.1055625 | down | mRNA |
| NM_000700 | ANXA1 | 0.402505682 | 0.864786348 | 1.0496066 | down | mRNA |
| NM_000702 | ATP1A2 | 0.895131414 | 0.993978977 | 1.0155489 | down | mRNA |
| NM_000705 | ATP4B | 0.627112815 | 0.949218083 | 1.0437663 | down | mRNA |
| NM_000718 | CACNA1B | 0.880795417 | 0.992287164 | 1.0126901 | down | mRNA |
| NM_000722 | CACNA2D1 | 0.605919428 | 0.94174007 | 1.0555415 | down | mRNA |
| NM_000724 | CACNB2 | 0.778898491 | 0.976938981 | 1.0230068 | down | mRNA |
| NM_000737 | CGB | 0.330385949 | 0.821797487 | 1.1916363 | down | mRNA |
| NM_000738 | CHRM1 | 0.490311205 | 0.905264563 | 1.0682021 | down | mRNA |
| NM_000740 | CHRM3 | 0.994404697 | 0.999872237 | 1.0053387 | down | mRNA |
| NM_000755 | CRAT | 0.64476483 | 0.954657712 | 1.0376 | down | mRNA |
| NM_000759 | CSF3 | 0.936192371 | 0.996710991 | 1.0072747 | down | mRNA |
| NM_000760 | CSF3R | 0.984112701 | 0.998971807 | 1.0019442 | down | mRNA |
| NM_000766 | CYP2A13 | 0.434036274 | 0.880931467 | 1.0457115 | down | mRNA |
| NM_000770 | CYP2C8 | 0.817636667 | 0.981727075 | 1.1017369 | down | mRNA |
| NM_000775 | CYP2J2 | 0.141334882 | 0.601679402 | 1.2052772 | down | mRNA |
| NM_000777 | CYP3A5 | 0.336365493 | 0.826506012 | 1.18187 | down | mRNA |
| NM_000783 | CYP26A1 | 0.975544722 | 0.998501532 | 1.0044631 | down | mRNA |
| NM_000784 | CYP27A1 | 0.791138385 | 0.978610795 | 1.0300587 | down | mRNA |
| NM_000786 | CYP51A1 | 0.315073585 | 0.810240865 | 1.1517821 | down | mRNA |
| NM_000788 | DCK | 0.070211895 | 0.458429109 | 1.2021431 | down | mRNA |
| NM_000791 | DHFR | 0.673861289 | 0.959531882 | 1.0793427 | down | mRNA |
| NM_000813 | GABRB2 | 0.838141527 | 0.986897498 | 1.0317415 | down | mRNA |
| NM_000820 | GAS6 | 0.945228826 | 0.996710991 | 1.0169986 | down | mRNA |
| NM_000825 | GNRH1 | 0.930412143 | 0.996710991 | 1.0097079 | down | mRNA |
| NM_000830 | GRIK1 | 0.489402487 | 0.904676004 | 1.1251028 | down | mRNA |
| NM_000831 | GRIK3 | 0.253083695 | 0.753288068 | 1.3535949 | down | mRNA |
| NM_000835 | GRIN2C | 0.824074452 | 0.983568283 | 1.0226175 | down | mRNA |
| NM_000836 | GRIN2D | 0.31019988 | 0.805036317 | 1.4024574 | down | mRNA |
| NM_000841 | GRM4 | 0.877851031 | 0.991306146 | 1.0229218 | down | mRNA |
| NM_000849 | GSTM3 | 0.863645193 | 0.989401142 | 1.0253137 | down | mRNA |
| NM_000850 | GSTM4 | 0.914309951 | 0.995342392 | 1.0448771 | down | mRNA |
| NM_000852 | GSTP1 | 0.55574183 | 0.926833616 | 1.1163283 | down | mRNA |
| NM_000854 | GSTT2 | 0.965823505 | 0.99793927 | 1.0014932 | down | mRNA |
| NM_000859 | HMGCR | 0.285750108 | 0.782579634 | 1.3068808 | down | mRNA |
| NM_000867 | HTR2B | 0.996540707 | 0.999872237 | 1.0005195 | down | mRNA |
| NM_000875 | IGF1R | 0.213501542 | 0.705954358 | 1.1828619 | down | mRNA |
| NM_000883 | IMPDH1 | 0.366547961 | 0.845181673 | 1.3151746 | down | mRNA |
| NM_000884 | IMPDH2 | 0.12086856 | 0.572400147 | 1.1010403 | down | mRNA |
| NM_000885 | ITGA4 | 0.876381239 | 0.991213905 | 1.1218417 | down | mRNA |
| NM_000895 | LTA4H | 0.660656421 | 0.958354006 | 1.024288 | down | mRNA |
| NM_000896 | CYP4F3 | 0.940249786 | 0.996710991 | 1.0206814 | down | mRNA |
| NM_000899 | KITLG | 0.371234424 | 0.84646611 | 1.2940867 | down | mRNA |
| NM_000903 | NQO1 | 0.764374074 | 0.973968787 | 1.110792 | down | mRNA |
| NM_000904 | NQO2 | 0.999253275 | 0.999897336 | 1.0003108 | down | mRNA |
| NM_000906 | NPR1 | 0.164301922 | 0.637394902 | 1.6071166 | down | mRNA |
| NM_000908 | NPR3 | 0.339187455 | 0.827695551 | 1.1568705 | down | mRNA |
| NM_000909 | NPY1R | 0.3547642 | 0.83766056 | 1.1868709 | down | mRNA |
| NM_000915 | OXT | 0.998523363 | 0.999889342 | 1.0005935 | down | mRNA |
| NM_000916 | OXTR | 0.894190947 | 0.993978977 | 1.1397247 | down | mRNA |
| NM_000917 | P4HA1 | 0.120961052 | 0.572400147 | 1.1144586 | down | mRNA |
| NM_000919 | PAM | 0.543857133 | 0.923577434 | 1.0522455 | down | mRNA |
| NM_000921 | PDE3A | 0.580629801 | 0.93393768 | 1.49252 | down | mRNA |
| NM_000926 | PGR | 0.907558654 | 0.995145546 | 1.0462093 | down | mRNA |
| NM_000932 | PLCB3 | 0.126818079 | 0.580406758 | 1.3799097 | down | mRNA |
| NM_000933 | PLCB4 | 0.834588273 | 0.986575022 | 1.0317533 | down | mRNA |
| NM_000935 | PLOD2 | 0.70760928 | 0.965401045 | 1.0206713 | down | mRNA |
| NM_000937 | POLR2A | 0.142500498 | 0.602652383 | 1.1635225 | down | mRNA |
| NM_000944 | PPP3CA | 0.112990843 | 0.556119542 | 1.0885799 | down | mRNA |
| NM_000945 | PPP3R1 | 0.452806335 | 0.889490855 | 1.3789903 | down | mRNA |
| NM_000946 | PRIM1 | 0.995227816 | 0.999872237 | 1.0032402 | down | mRNA |
| NM_000947 | PRIM2 | 0.520811969 | 0.916605148 | 1.1278995 | down | mRNA |
| NM_000962 | PTGS1 | 0.75115214 | 0.970725371 | 1.0712692 | down | mRNA |
| NM_000964 | RARA | 0.587444847 | 0.93579682 | 1.0525735 | down | mRNA |
| NM_000969 | RPL5 | 0.786775384 | 0.977479172 | 1.0159443 | down | mRNA |
| NM_000971 | RPL7 | 0.936272031 | 0.996710991 | 1.0024806 | down | mRNA |
| NM_000972 | RPL7A | 0.741779341 | 0.970268758 | 1.0365967 | down | mRNA |
| NM_000976 | RPL12 | 0.519075473 | 0.916145597 | 1.0360597 | down | mRNA |
| NM_000985 | RPL17 | 0.879137471 | 0.992149178 | 1.0167951 | down | mRNA |
| NM_000988 | RPL27 | 0.581816053 | 0.934005566 | 1.0064855 | down | mRNA |
| NM_000989 | RPL30 | 0.13863273 | 0.598141596 | 1.0351755 | down | mRNA |
| NM_000991 | RPL28 | 0.18395548 | 0.667532183 | 1.0593221 | down | mRNA |
| NM_001001 | RPL36AL | 0.375854187 | 0.849862269 | 1.0568657 | down | mRNA |
| NM_001001132 | ITSN1 | 0.477988942 | 0.901130939 | 1.1270669 | down | mRNA |
| NM_001001290 | SLC2A9 | 0.118402446 | 0.565904687 | 1.1490382 | down | mRNA |
| NM_001001329 | PRKCSH | 0.271258526 | 0.77133889 | 1.4410644 | down | mRNA |
| NM_001001343 | FNDC9 | 0.036718794 | 0.359628653 | 1.3454394 | down | mRNA |
| NM_001001395 | LMO3 | 0.364893741 | 0.844509908 | 1.0729608 | down | mRNA |
| NM_001001410 | TSR3 | 0.368801002 | 0.845559574 | 1.0255996 | down | mRNA |
| NM_001001419 | SMAD5 | 0.000192181 | 0.047651492 | 1.3080651 | down | mRNA |
| NM_001001420 | SMAD5 | 0.032621182 | 0.344901399 | 1.1834248 | down | mRNA |
| NM_001001430 | TNNT2 | 0.238258966 | 0.736436805 | 1.327027 | down | mRNA |
| NM_001001436 | C16orf87 | 0.246898623 | 0.745152522 | 1.1205265 | down | mRNA |
| NM_001001479 | SLC35E4 | 0.695028906 | 0.962214902 | 1.0320524 | down | mRNA |
| NM_001001481 | UBE2W | 0.059722468 | 0.43351561 | 1.5309344 | down | mRNA |
| NM_001001483 | ZDHHC13 | 0.722956242 | 0.966191059 | 1.0339409 | down | mRNA |
| NM_001001485 | ATP2C1 | 0.68406593 | 0.960111041 | 1.0428734 | down | mRNA |
| NM_001001486 | ATP2C1 | 0.456102658 | 0.891365829 | 1.0966679 | down | mRNA |
| NM_001001487 | ATP2C1 | 0.800871317 | 0.98118927 | 1.0330027 | down | mRNA |
| NM_001001503 | NDUFV3 | 0.038155569 | 0.365047918 | 1.1024145 | down | mRNA |
| NM_001001551 | IDNK | 0.313831225 | 0.808965923 | 2.0970871 | down | mRNA |
| NM_001001556 | GALK2 | 0.103184576 | 0.532945334 | 1.1147183 | down | mRNA |
| NM_001001571 | PDE9A | 0.409287321 | 0.867591329 | 1.264332 | down | mRNA |
| NM_001001574 | PDE9A | 0.412352273 | 0.869163012 | 1.856631 | down | mRNA |
| NM_001001581 | PDE9A | 0.295928189 | 0.792311289 | 1.1732446 | down | mRNA |
| NM_001001583 | PDE9A | 0.626522907 | 0.949203235 | 1.1742842 | down | mRNA |
| NM_001001657 | OR2Y1 | 0.521261038 | 0.916644975 | 1.1797601 | down | mRNA |
| NM_001001660 | LYRM5 | 0.84664503 | 0.987644719 | 1.0257038 | down | mRNA |
| NM_001001662 | ZNF782 | 0.050266462 | 0.405800127 | 1.3753897 | down | mRNA |
| NM_001001664 | SPOPL | 0.003465721 | 0.138138947 | 1.2016471 | down | mRNA |
| NM_001001665 | CYP27C1 | 0.062545482 | 0.439458294 | 1.2759358 | down | mRNA |
| NM_001001668 | ZNF470 | 0.045460849 | 0.3905726 | 1.3235148 | down | mRNA |
| NM_001001669 | ARHGEF37 | 0.577566364 | 0.933787388 | 1.1632014 | down | mRNA |
| NM_001001712 | LCN10 | 0.435921433 | 0.881677506 | 1.0702932 | down | mRNA |
| NM_001001722 | CDY2B | 0.134135836 | 0.590871682 | 1.1636599 | down | mRNA |
| NM_001001740 | RFWD2 | 0.170000242 | 0.646789844 | 1.1877828 | down | mRNA |
| NM_001001790 | TOMM5 | 0.812603448 | 0.98118927 | 1.0107812 | down | mRNA |
| NM_001001795 | C8orf82 | 0.72796662 | 0.966191059 | 1.0362465 | down | mRNA |
| NM_001001875 | TPD52L3 | 0.120953995 | 0.572400147 | 1.3410547 | down | mRNA |
| NM_001001895 | UBASH3A | 0.660398943 | 0.958354006 | 1.0802883 | down | mRNA |
| NM_001001923 | OR5C1 | 0.725239605 | 0.966191059 | 1.0327974 | down | mRNA |
| NM_001001973 | ATP5C1 | 0.933173404 | 0.996710991 | 1.0078102 | down | mRNA |
| NM_001001974 | PLEKHA1 | 0.88389942 | 0.992766819 | 1.0119356 | down | mRNA |
| NM_001001976 | ATE1 | 0.008253861 | 0.197897997 | 1.3831887 | down | mRNA |
| NM_001001991 | PAMR1 | 0.552156402 | 0.926337111 | 1.1829054 | down | mRNA |
| NM_001001994 | GPM6B | 0.747011905 | 0.970432947 | 1.0329342 | down | mRNA |
| NM_001001998 | EXOSC10 | 0.008298352 | 0.198160569 | 1.2582367 | down | mRNA |
| NM_001002000 | GMPR2 | 0.766570339 | 0.974840715 | 1.053051 | down | mRNA |
| NM_001002010 | NT5C3 | 0.180535522 | 0.663530048 | 1.1605551 | down | mRNA |
| NM_001002030 | ECHDC1 | 0.687791709 | 0.960254139 | 1.0612189 | down | mRNA |
| NM_001002031 | ATP5G2 | 0.370883471 | 0.846330273 | 1.1664139 | down | mRNA |
| NM_001002032 | HN1 | 0.860303093 | 0.988957001 | 1.050653 | down | mRNA |
| NM_001002033 | HN1 | 0.879231569 | 0.992149178 | 1.0431515 | down | mRNA |
| NM_001002034 | FAM109B | 0.015139984 | 0.247051486 | 1.5390947 | down | mRNA |
| NM_001002231 | KLK2 | 0.037021436 | 0.359628653 | 1.2445449 | down | mRNA |
| NM_001002244 | ANAPC11 | 0.600069838 | 0.940648177 | 1.1177749 | down | mRNA |
| NM_001002251 | ARL6IP4 | 0.442538028 | 0.884228603 | 1.2428942 | down | mRNA |
| NM_001002252 | ARL6IP4 | 0.055659162 | 0.42131872 | 1.0969228 | down | mRNA |
| NM_001002257 | LCLAT1 | 0.452838867 | 0.889490855 | 1.2101948 | down | mRNA |
| NM_001002258 | ATP5G3 | 0.479275892 | 0.901538317 | 1.072279 | down | mRNA |
| NM_001002261 | ZFYVE27 | 0.049807417 | 0.404032605 | 1.1897372 | down | mRNA |
| NM_001002262 | ZFYVE27 | 0.423519416 | 0.874474551 | 1.1781712 | down | mRNA |
| NM_001002269 | EXOSC3 | 0.055209704 | 0.419634622 | 1.2064577 | down | mRNA |
| NM_001002292 | WLS | 0.629522745 | 0.950410442 | 1.1468957 | down | mRNA |
| NM_001002756 | NFU1 | 0.932636888 | 0.996710991 | 1.0085643 | down | mRNA |
| NM_001002796 | MCTP1 | 0.467295687 | 0.896646168 | 1.1538792 | down | mRNA |
| NM_001002810 | PDE4DIP | 0.987320705 | 0.999140676 | 1.0007332 | down | mRNA |
| NM_001002811 | PDE4DIP | 0.920831084 | 0.995349793 | 1.018421 | down | mRNA |
| NM_001002812 | PDE4DIP | 0.831863937 | 0.986141573 | 1.2916284 | down | mRNA |
| NM_001002814 | RAB11FIP1 | 0.836187697 | 0.986575022 | 1.0138975 | down | mRNA |
| NM_001002837 | INPP5J | 0.138123095 | 0.597133482 | 1.2243804 | down | mRNA |
| NM_001002843 | ZNF280D | 0.367409654 | 0.845181673 | 1.1771194 | down | mRNA |
| NM_001002858 | ANXA2 | 0.281611198 | 0.779126326 | 1.0555288 | down | mRNA |
| NM_001002860 | BTBD7 | 0.057666596 | 0.427646201 | 1.2033033 | down | mRNA |
| NM_001002876 | CENPM | 0.923504113 | 0.995456159 | 1.0645789 | down | mRNA |
| NM_001002880 | CBY1 | 0.056500713 | 0.423588675 | 1.1084874 | down | mRNA |
| NM_001002901 | FCRLB | 0.031487325 | 0.341322176 | 1.1985104 | down | mRNA |
| NM_001002909 | GPATCH8 | 0.127103411 | 0.580406758 | 1.1919412 | down | mRNA |
| NM_001003397 | TPD52L1 | 0.85590758 | 0.988315074 | 1.02793 | down | mRNA |
| NM_001003398 | BICD1 | 0.177179363 | 0.65715084 | 1.4610854 | down | mRNA |
| NM_001003406 | CACNA1I | 0.790236559 | 0.978438986 | 1.01756 | down | mRNA |
| NM_001003676 | C11orf49 | 0.087916337 | 0.501543709 | 1.2425691 | down | mRNA |
| NM_001003677 | C11orf49 | 0.737087245 | 0.968834005 | 1.0201298 | down | mRNA |
| NM_001003681 | HMGXB4 | 0.642792978 | 0.954490115 | 1.0756199 | down | mRNA |
| NM_001003690 | MAD2L1BP | 0.009238332 | 0.204167136 | 1.1404244 | down | mRNA |
| NM_001003692 | ZMAT5 | 0.452370799 | 0.888914959 | 1.060087 | down | mRNA |
| NM_001003700 | RREB1 | 0.016300376 | 0.255922225 | 2.4228651 | down | mRNA |
| NM_001003702 | ARHGEF35 | 0.690627004 | 0.961105466 | 1.1780648 | down | mRNA |
| NM_001003713 | ATP5J2 | 0.295414582 | 0.79157694 | 1.0469153 | down | mRNA |
| NM_001003715 | RECQL5 | 0.035084215 | 0.352396712 | 1.2465759 | down | mRNA |
| NM_001003722 | GLE1 | 0.021104769 | 0.288375117 | 1.2450108 | down | mRNA |
| NM_001003787 | STRADA | 0.025412839 | 0.312768665 | 1.6419915 | down | mRNA |
| NM_001003790 | ERLIN2 | 0.147426882 | 0.609973207 | 1.2486063 | down | mRNA |
| NM_001003792 | RBMS3 | 0.620885973 | 0.947926299 | 1.166872 | down | mRNA |
| NM_001003795 | GTF2IRD2B | 0.55552047 | 0.926833616 | 1.1608092 | down | mRNA |
| NM_001003800 | BICD2 | 0.78109017 | 0.976938981 | 1.0487033 | down | mRNA |
| NM_001003801 | SMARCD3 | 0.046295205 | 0.392057225 | 1.3217341 | down | mRNA |
| NM_001003803 | ATP5S | 0.518863208 | 0.916145597 | 1.060148 | down | mRNA |
| NM_001003805 | ATP5S | 0.590121015 | 0.936215326 | 1.0586854 | down | mRNA |
| NM_001003827 | TRIM34 | 0.175973814 | 0.655114384 | 1.1069786 | down | mRNA |
| NM_001003828 | PARVB | 0.014730397 | 0.24380986 | 1.4933603 | down | mRNA |
| NM_001003845 | SP5 | 0.405265202 | 0.866165103 | 1.7176198 | down | mRNA |
| NM_001003927 | EVI2A | 0.051706798 | 0.41031217 | 1.1066961 | down | mRNA |
| NM_001003941 | OGDH | 0.554471704 | 0.926549129 | 1.1314151 | down | mRNA |
| NM_001003942 | BMF | 0.014489829 | 0.242674797 | 1.2262041 | down | mRNA |
| NM_001003943 | BMF | 0.937394335 | 0.996710991 | 1.0020069 | down | mRNA |
| NM_001003954 | ANXA13 | 0.088476198 | 0.502587299 | 1.6391085 | down | mRNA |
| NM_001003962 | CAPNS1 | 0.54943254 | 0.925522983 | 1.1158522 | down | mRNA |
| NM_001004 | RPLP2 | 0.590607438 | 0.936533336 | 1.0752715 | down | mRNA |
| NM_001004055 | LRRC29 | 0.363100062 | 0.843238468 | 1.1602275 | down | mRNA |
| NM_001004056 | GRK4 | 0.752986583 | 0.970725371 | 1.1352456 | down | mRNA |
| NM_001004060 | NOMO2 | 0.814706197 | 0.981277402 | 1.0242356 | down | mRNA |
| NM_001004065 | AKAP2 | 0.808172159 | 0.98118927 | 1.0407931 | down | mRNA |
| NM_001004105 | GRK6 | 0.028426397 | 0.327830694 | 1.1749431 | down | mRNA |
| NM_001004106 | GRK6 | 0.04988401 | 0.404101075 | 1.164643 | down | mRNA |
| NM_001004127 | ALG11 | 0.216233298 | 0.71103525 | 1.0889722 | down | mRNA |
| NM_001004128 | QSOX1 | 0.222370002 | 0.71737156 | 1.1338075 | down | mRNA |
| NM_001004301 | ZNF813 | 0.271230641 | 0.77133889 | 1.1172337 | down | mRNA |
| NM_001004304 | ZNF740 | 0.276267833 | 0.77594662 | 1.2473937 | down | mRNA |
| NM_001004309 | ZNF774 | 0.010244717 | 0.20936306 | 1.2394705 | down | mRNA |
| NM_001004325 | KRTAP5-2 | 0.363239186 | 0.843238468 | 1.1796453 | down | mRNA |
| NM_001004326 | SLC22A20 | 0.708498731 | 0.965462762 | 1.1244712 | down | mRNA |
| NM_001004333 | RNASEK | 0.793763167 | 0.97921643 | 1.0295226 | down | mRNA |
| NM_001004340 | FCGR1B | 0.43923309 | 0.882895421 | 1.0991018 | down | mRNA |
| NM_001004356 | FGFRL1 | 0.478944309 | 0.901271944 | 1.189646 | down | mRNA |
| NM_001004360 | DPP10 | 0.48571356 | 0.903250293 | 1.2889596 | down | mRNA |
| NM_001004431 | METRNL | 0.470323439 | 0.898180112 | 1.0744758 | down | mRNA |
| NM_001004434 | SLC30A2 | 0.66789739 | 0.958354006 | 1.0789039 | down | mRNA |
| NM_001004439 | ITGA11 | 0.068048307 | 0.452735007 | 1.4850173 | down | mRNA |
| NM_001004685 | OR2F2 | 0.774057348 | 0.97589756 | 1.047395 | down | mRNA |
| NM_001004689 | OR2M3 | 0.281315302 | 0.779126326 | 1.094031 | down | mRNA |
| NM_001004708 | OR4D6 | 0.680859965 | 0.960111041 | 1.0416849 | down | mRNA |
| NM_001004713 | OR1I1 | 0.624580025 | 0.948341211 | 1.1376339 | down | mRNA |
| NM_001004728 | OR5A1 | 0.865934454 | 0.989454945 | 1.0538648 | down | mRNA |
| NM_001005 | RPS3 | 0.828195287 | 0.985325387 | 1.0195006 | down | mRNA |
| NM_001005207 | TRIM37 | 0.855641044 | 0.988179137 | 1.0144567 | down | mRNA |
| NM_001005213 | OR9G1 | 0.744581135 | 0.970330526 | 1.1462816 | down | mRNA |
| NM_001005238 | OR51G2 | 0.849052469 | 0.987678166 | 1.0318165 | down | mRNA |
| NM_001005287 | OR2A1 | 0.799949222 | 0.981152656 | 1.0939154 | down | mRNA |
| NM_001005290 | PSRC1 | 0.951583884 | 0.996710991 | 1.0099637 | down | mRNA |
| NM_001005340 | GPNMB | 0.491068794 | 0.90553335 | 1.1278369 | down | mRNA |
| NM_001005354 | PRR13 | 0.835242243 | 0.986575022 | 1.0117365 | down | mRNA |
| NM_001005360 | DNM2 | 0.736506414 | 0.968717225 | 1.0417267 | down | mRNA |
| NM_001005361 | DNM2 | 0.035149544 | 0.352396712 | 1.2584511 | down | mRNA |
| NM_001005368 | ZNF32 | 0.51061413 | 0.912251098 | 1.1999884 | down | mRNA |
| NM_001005389 | NFASC | 0.757246779 | 0.971960783 | 1.0132379 | down | mRNA |
| NM_001005407 | CACNA1H | 0.858685173 | 0.988512246 | 1.0317662 | down | mRNA |
| NM_001005409 | SF3A1 | 0.29949619 | 0.795720295 | 1.0809297 | down | mRNA |
| NM_001005417 | B4GALT2 | 0.021348271 | 0.288424269 | 1.1790433 | down | mRNA |
| NM_001005466 | OR10G2 | 0.135251948 | 0.592787801 | 1.0846691 | down | mRNA |
| NM_001005468 | OR8B2 | 0.420339547 | 0.872365335 | 1.2999446 | down | mRNA |
| NM_001005473 | PLCXD3 | 0.974190545 | 0.998501532 | 1.0043311 | down | mRNA |
| NM_001005476 | PKP4 | 0.58516989 | 0.934708084 | 1.1216439 | down | mRNA |
| NM_001005480 | OR2A2 | 0.299407229 | 0.795703014 | 1.0645427 | down | mRNA |
| NM_001005514 | OR5H14 | 0.870012322 | 0.989592579 | 1.0143256 | down | mRNA |
| NM_001005610 | EDA | 0.489820142 | 0.905036635 | 1.0467972 | down | mRNA |
| NM_001005613 | EDA | 0.509495351 | 0.911674633 | 1.1452447 | down | mRNA |
| NM_001005735 | CHEK2 | 0.811502295 | 0.98118927 | 1.0510374 | down | mRNA |
| NM_001005739 | VPS54 | 0.154700336 | 0.622213869 | 1.1472731 | down | mRNA |
| NM_001005753 | CHMP3 | 0.685761102 | 0.960111041 | 1.0381047 | down | mRNA |
| NM_001005782 | SUMO1 | 0.508234642 | 0.911674633 | 1.0587287 | down | mRNA |
| NM_001005849 | SUMO2 | 0.729581309 | 0.966191059 | 1.0213795 | down | mRNA |
| NM_001005850 | ZNF835 | 0.563344662 | 0.929041598 | 1.1891123 | down | mRNA |
| NM_001005851 | ZNF780B | 0.81413395 | 0.98120545 | 1.0477437 | down | mRNA |
| NM_001005855 | ATP8B2 | 0.080990251 | 0.486318878 | 1.255136 | down | mRNA |
| NM_001006109 | C3orf37 | 0.002246962 | 0.112066979 | 1.4315949 | down | mRNA |
| NM_001006121 | RBMY1B | 0.253225687 | 0.753288068 | 1.0908319 | down | mRNA |
| NM_001006600 | ERBB2IP | 0.084518771 | 0.494805206 | 1.2345107 | down | mRNA |
| NM_001006605 | FAM69A | 0.826437128 | 0.984423205 | 1.1104492 | down | mRNA |
| NM_001006607 | LRRC37A2 | 0.178579961 | 0.65979922 | 1.2216906 | down | mRNA |
| NM_001006610 | SIAH1 | 0.288738273 | 0.784633328 | 1.144601 | down | mRNA |
| NM_001006613 | WBP5 | 0.073643114 | 0.467143085 | 1.1748946 | down | mRNA |
| NM_001006616 | TSPAN17 | 0.577012745 | 0.933656777 | 1.0614753 | down | mRNA |
| NM_001006619 | MAPKAP1 | 0.868776412 | 0.989481868 | 1.012621 | down | mRNA |
| NM_001006622 | WDR33 | 0.186402978 | 0.669888134 | 1.1776433 | down | mRNA |
| NM_001006623 | WDR33 | 0.012123843 | 0.222675671 | 1.432915 | down | mRNA |
| NM_001006638 | RAB37 | 0.811937946 | 0.98118927 | 1.0269863 | down | mRNA |
| NM_001006681 | SPIN2B | 0.193215565 | 0.680488286 | 1.0980753 | down | mRNA |
| NM_001006935 | TCEAL4 | 0.967329311 | 0.99793927 | 1.0067279 | down | mRNA |
| NM_001006941 | ALG3 | 0.438790225 | 0.882895421 | 1.2557254 | down | mRNA |
| NM_001006945 | LRIF1 | 0.062708852 | 0.439458294 | 1.0724965 | down | mRNA |
| NM_001007 | RPS4X | 0.790656488 | 0.978509111 | 1.0128082 | down | mRNA |
| NM_001007022 | ODF2L | 0.488719984 | 0.904236483 | 1.1207971 | down | mRNA |
| NM_001007024 | GOSR1 | 0.051714362 | 0.41031217 | 1.1588187 | down | mRNA |
| NM_001007025 | GOSR1 | 0.168608752 | 0.644268587 | 1.1731607 | down | mRNA |
| NM_001007026 | ATN1 | 0.735212567 | 0.96794858 | 1.0195958 | down | mRNA |
| NM_001007027 | ALG8 | 0.21406713 | 0.706760409 | 1.0633899 | down | mRNA |
| NM_001007072 | ZSCAN2 | 0.438790382 | 0.882895421 | 1.0469281 | down | mRNA |
| NM_001007088 | ZNF182 | 0.982687637 | 0.998971807 | 1.0023146 | down | mRNA |
| NM_001007094 | ZNF37A | 0.010190456 | 0.20936306 | 1.3489736 | down | mRNA |
| NM_001007098 | SCP2 | 0.584608287 | 0.93461341 | 1.0444171 | down | mRNA |
| NM_001007099 | SCP2 | 0.614119426 | 0.944827645 | 1.0632189 | down | mRNA |
| NM_001007100 | SCP2 | 0.469282106 | 0.897810636 | 1.1046778 | down | mRNA |
| NM_001007101 | ZNF484 | 0.002962599 | 0.129818007 | 1.7755192 | down | mRNA |
| NM_001007214 | CACYBP | 0.680694831 | 0.960111041 | 1.0599118 | down | mRNA |
| NM_001007225 | IGF2BP2 | 0.544907119 | 0.924012779 | 1.1074061 | down | mRNA |
| NM_001007228 | SPOP | 0.040004001 | 0.371915487 | 1.3459377 | down | mRNA |
| NM_001007231 | ARHGAP25 | 0.400501719 | 0.864529245 | 1.9006467 | down | mRNA |
| NM_001007237 | IGSF3 | 0.617960966 | 0.946754755 | 1.1236881 | down | mRNA |
| NM_001007239 | METTL13 | 0.015538603 | 0.250096132 | 1.2911863 | down | mRNA |
| NM_001007246 | BRWD1 | 0.964790581 | 0.99793927 | 1.0056558 | down | mRNA |
| NM_001007259 | SETD4 | 0.478000058 | 0.901130939 | 1.2035626 | down | mRNA |
| NM_001007271 | DUSP13 | 0.459314501 | 0.892904132 | 1.0519244 | down | mRNA |
| NM_001007277 | EI24 | 0.023952165 | 0.303389731 | 1.1606424 | down | mRNA |
| NM_001007278 | TRIM13 | 0.160030073 | 0.631816652 | 1.2600093 | down | mRNA |
| NM_001007467 | SFI1 | 0.259299333 | 0.759808377 | 3.3013474 | down | mRNA |
| NM_001007527 | LMBRD2 | 0.016219525 | 0.255838153 | 1.1810691 | down | mRNA |
| NM_001007533 | PPP1R27 | 0.818779362 | 0.982093332 | 1.0389713 | down | mRNA |
| NM_001007561 | IRGQ | 0.418900986 | 0.872328878 | 1.3776557 | down | mRNA |
| NM_001007595 | C2CD4B | 0.527222087 | 0.918238774 | 1.0698928 | down | mRNA |
| NM_001007793 | BUB3 | 0.33996534 | 0.828118322 | 1.1816824 | down | mRNA |
| NM_001008 | RPS4Y1 | 0.212499722 | 0.704360017 | 1.2301477 | down | mRNA |
| NM_001008215 | COA5 | 0.514439185 | 0.913819457 | 1.0672696 | down | mRNA |
| NM_001008216 | GALE | 0.394361373 | 0.86105621 | 1.1706438 | down | mRNA |
| NM_001008228 | MOG | 0.99484833 | 0.999872237 | 1.0026054 | down | mRNA |
| NM_001008229 | MOG | 0.722610271 | 0.966191059 | 1.0486483 | down | mRNA |
| NM_001008239 | C18orf25 | 0.989899991 | 0.999140676 | 1.0012769 | down | mRNA |
| NM_001008269 | TMEM89 | 0.604959633 | 0.94174007 | 1.0621756 | down | mRNA |
| NM_001008271 | SCXA | 0.000215179 | 0.048871331 | 2.0242288 | down | mRNA |
| NM_001008392 | CTDSPL | 0.277605532 | 0.777234326 | 1.190721 | down | mRNA |
| NM_001008396 | WDHD1 | 0.281754386 | 0.779299439 | 1.3593179 | down | mRNA |
| NM_001008397 | GPX8 | 0.571234816 | 0.933029103 | 1.0840255 | down | mRNA |
| NM_001008401 | ZNF761 | 0.00690755 | 0.18551293 | 1.4435806 | down | mRNA |
| NM_001008404 | C14orf180 | 0.082086173 | 0.489148905 | 1.3175945 | down | mRNA |
| NM_001008485 | SLC41A3 | 0.476257804 | 0.900415348 | 1.0811347 | down | mRNA |
| NM_001008487 | SLC41A3 | 0.3799831 | 0.853273195 | 1.0764614 | down | mRNA |
| NM_001008489 | PHOSPHO2 | 0.391494742 | 0.859202394 | 1.126249 | down | mRNA |
| NM_001008493 | ENAH | 0.00124091 | 0.092399617 | 1.3473911 | down | mRNA |
| NM_001008494 | ISX | 0.389073738 | 0.85800274 | 2.2864139 | down | mRNA |
| NM_001008495 | TMEM64 | 0.13406682 | 0.590871682 | 1.2668015 | down | mRNA |
| NM_001008529 | MXRA7 | 0.449115905 | 0.888279993 | 1.1123504 | down | mRNA |
| NM_001008660 | PICALM | 0.04517794 | 0.389384436 | 1.1768268 | down | mRNA |
| NM_001008662 | CCBL2 | 0.967459825 | 0.99793927 | 1.017142 | down | mRNA |
| NM_001008693 | CST9 | 0.879246747 | 0.992149178 | 1.0420309 | down | mRNA |
| NM_001008697 | TFIP11 | 0.002690351 | 0.122795128 | 1.3704946 | down | mRNA |
| NM_001008704 | C6orf1 | 0.764916837 | 0.97415819 | 1.0180303 | down | mRNA |
| NM_001008708 | CHAC2 | 0.948840587 | 0.996710991 | 1.024514 | down | mRNA |
| NM_001008709 | PPP1CA | 0.736305573 | 0.968717225 | 1.0487038 | down | mRNA |
| NM_001008712 | RBPMS | 0.370252118 | 0.846215286 | 1.1014598 | down | mRNA |
| NM_001008738 | FNIP1 | 0.146099098 | 0.608122555 | 1.3003874 | down | mRNA |
| NM_001008747 | CTAGE15P | 0.535914164 | 0.922156973 | 1.1387428 | down | mRNA |
| NM_001008783 | SLC35D3 | 0.870567967 | 0.989592579 | 1.0159622 | down | mRNA |
| NM_001008844 | DSP | 0.51936205 | 0.916145597 | 1.2830218 | down | mRNA |
| NM_001008894 | NIPA2 | 0.014075826 | 0.239157213 | 1.1970053 | down | mRNA |
| NM_001008897 | TCP1 | 0.678420777 | 0.959825429 | 1.0458247 | down | mRNA |
| NM_001008938 | CKAP5 | 0.065154601 | 0.445966419 | 1.1500312 | down | mRNA |
| NM_001009 | RPS5 | 0.513658615 | 0.91350662 | 1.0358504 | down | mRNA |
| NM_001009182 | GEMIN2 | 0.127842751 | 0.581220503 | 1.2827197 | down | mRNA |
| NM_001009183 | GEMIN2 | 0.065968608 | 0.449488516 | 1.4200664 | down | mRNA |
| NM_001009568 | SMPDL3B | 0.92467187 | 0.995456159 | 1.0088787 | down | mRNA |
| NM_001009570 | CCT7 | 0.180780548 | 0.663780602 | 1.1390041 | down | mRNA |
| NM_001009571 | CADPS2 | 0.481246762 | 0.901538317 | 1.0803024 | down | mRNA |
| NM_001009584 | ARMCX6 | 0.009013746 | 0.202329498 | 1.2098181 | down | mRNA |
| NM_001009606 | HS3ST6 | 0.021088529 | 0.288346982 | 1.3302123 | down | mRNA |
| NM_001009811 | AZI1 | 0.304503127 | 0.800038203 | 1.3094662 | down | mRNA |
| NM_001009813 | MEIS3 | 0.608282728 | 0.942940258 | 1.079622 | down | mRNA |
| NM_001009814 | VWA8 | 0.411105182 | 0.868434057 | 1.079004 | down | mRNA |
| NM_001009877 | BRD9 | 0.029750851 | 0.334953885 | 1.2715241 | down | mRNA |
| NM_001009881 | ZCCHC11 | 0.837584494 | 0.986775289 | 1.0305745 | down | mRNA |
| NM_001009894 | C12orf29 | 0.557333998 | 0.926833616 | 1.1076673 | down | mRNA |
| NM_001009899 | KIAA2018 | 0.149551391 | 0.61391883 | 1.1415666 | down | mRNA |
| NM_001009921 | VPS8 | 0.421523905 | 0.873398499 | 1.1516979 | down | mRNA |
| NM_001009922 | RCHY1 | 0.005681121 | 0.170618233 | 1.3642957 | down | mRNA |
| NM_001009943 | ANKRD16 | 0.371576216 | 0.846675738 | 1.2957477 | down | mRNA |
| NM_001009993 | FAM168B | 0.428349238 | 0.877211962 | 1.1137468 | down | mRNA |
| NM_001009996 | DALRD3 | 0.648287314 | 0.95550188 | 1.048692 | down | mRNA |
| NM_001010000 | ARHGAP28 | 0.5809466 | 0.934005566 | 1.0757914 | down | mRNA |
| NM_001010848 | NRG3 | 0.665561713 | 0.958354006 | 1.0435118 | down | mRNA |
| NM_001010854 | TTC7B | 0.018753297 | 0.274113616 | 1.1466143 | down | mRNA |
| NM_001010860 | SAMD15 | 0.697800172 | 0.962410025 | 1.1079723 | down | mRNA |
| NM_001010862 | SPIN3 | 0.439162363 | 0.882895421 | 1.2848695 | down | mRNA |
| NM_001010866 | TMEM201 | 0.239673964 | 0.737916177 | 1.7097683 | down | mRNA |
| NM_001010872 | FAM83B | 0.786235776 | 0.977479172 | 1.0214375 | down | mRNA |
| NM_001010875 | SLC25A30 | 0.138994105 | 0.598648178 | 1.6018245 | down | mRNA |
| NM_001010887 | ACER2 | 0.991015146 | 0.999376923 | 1.0027482 | down | mRNA |
| NM_001010889 | PRAMEF6 | 0.386358653 | 0.855747763 | 1.405353 | down | mRNA |
| NM_001010891 | MTX3 | 0.576967839 | 0.933656777 | 1.2489121 | down | mRNA |
| NM_001010924 | FAM171A1 | 0.819197168 | 0.98219491 | 1.0316993 | down | mRNA |
| NM_001010932 | HGF | 0.926077025 | 0.995699344 | 1.0759719 | down | mRNA |
| NM_001010933 | HGF | 0.831134445 | 0.985710869 | 1.1360403 | down | mRNA |
| NM_001010938 | TNK2 | 0.003397846 | 0.137400229 | 1.5650199 | down | mRNA |
| NM_001010969 | CYP4A22 | 0.707582752 | 0.965401045 | 1.1009008 | down | mRNA |
| NM_001010980 | NCMAP | 0.876411219 | 0.991213905 | 1.0443764 | down | mRNA |
| NM_001010984 | SPRTN | 0.218702706 | 0.712577334 | 1.1627807 | down | mRNA |
| NM_001010989 | HERPUD1 | 0.253170618 | 0.753288068 | 1.5763665 | down | mRNA |
| NM_001010990 | HERPUD1 | 0.440871716 | 0.883920737 | 1.4036348 | down | mRNA |
| NM_001011513 | PDLIM5 | 0.749034075 | 0.970725371 | 1.0404151 | down | mRNA |
| NM_001011515 | PDLIM5 | 0.770804018 | 0.975347132 | 1.0318825 | down | mRNA |
| NM_001011516 | PDLIM5 | 0.949078338 | 0.996710991 | 1.0105623 | down | mRNA |
| NM_001011550 | MAGEA4 | 0.366392837 | 0.845181673 | 1.5337718 | down | mRNA |
| NM_001011553 | SEPT7 | 0.297018427 | 0.793620415 | 1.0441821 | down | mRNA |
| NM_001011645 | AR | 0.691027905 | 0.961105466 | 1.0697099 | down | mRNA |
| NM_001011649 | CDK5RAP2 | 0.145773546 | 0.607759658 | 1.0985185 | down | mRNA |
| NM_001011655 | TMEM44 | 0.211639305 | 0.702997934 | 1.2661524 | down | mRNA |
| NM_001011658 | TRAPPC2 | 0.137024704 | 0.595932894 | 1.0851565 | down | mRNA |
| NM_001011699 | HAUS8 | 0.904714249 | 0.99508468 | 1.0275433 | down | mRNA |
| NM_001011700 | MCCD1 | 0.4016094 | 0.864768873 | 1.2653517 | down | mRNA |
| NM_001011703 | FAM125B | 0.962582989 | 0.99793927 | 1.0033242 | down | mRNA |
| NM_001011708 | OLA1 | 0.234984667 | 0.732440325 | 1.0711431 | down | mRNA |
| NM_001011709 | PNLIPRP3 | 0.103555518 | 0.533123263 | 1.1370392 | down | mRNA |
| NM_001011713 | NAA30 | 0.275072893 | 0.774827385 | 1.1022924 | down | mRNA |
| NM_001011880 | CLEC18B | 0.493312909 | 0.906383343 | 1.0318928 | down | mRNA |
| NM_001011885 | BTBD1 | 0.427804927 | 0.876992614 | 1.0892459 | down | mRNA |
| NM_001012241 | MSL1 | 0.053062463 | 0.413834293 | 1.3205158 | down | mRNA |
| NM_001012270 | BIRC5 | 0.8083456 | 0.98118927 | 1.1189123 | down | mRNA |
| NM_001012271 | BIRC5 | 0.898137717 | 0.994360027 | 1.0634298 | down | mRNA |
| NM_001012300 | MCRS1 | 0.405470417 | 0.866165103 | 1.0445074 | down | mRNA |
| NM_001012301 | ARSI | 0.284325326 | 0.78179061 | 1.1977058 | down | mRNA |
| NM_001012302 | ANO9 | 0.119131249 | 0.568319228 | 1.7708665 | down | mRNA |
| NM_001012331 | NTRK1 | 0.225444184 | 0.720462581 | 1.758005 | down | mRNA |
| NM_001012339 | DNAJC21 | 0.275457143 | 0.775184524 | 1.073611 | down | mRNA |
| NM_001012398 | AKTIP | 0.66574473 | 0.958354006 | 1.0562875 | down | mRNA |
| NM_001012410 | SGOL1 | 0.761308829 | 0.973219662 | 1.0800043 | down | mRNA |
| NM_001012411 | SGOL1 | 0.721278807 | 0.966191059 | 1.2694661 | down | mRNA |
| NM_001012412 | SGOL1 | 0.873560955 | 0.990580595 | 1.0779926 | down | mRNA |
| NM_001012413 | SGOL1 | 0.730449085 | 0.966329458 | 1.2568208 | down | mRNA |
| NM_001012414 | TRIM61 | 0.031804113 | 0.341957288 | 1.3549448 | down | mRNA |
| NM_001012415 | SOHLH1 | 0.038429746 | 0.366145077 | 1.1973257 | down | mRNA |
| NM_001012426 | FOXP4 | 0.153999753 | 0.62125456 | 1.1789256 | down | mRNA |
| NM_001012427 | FOXP4 | 0.066988121 | 0.451412357 | 1.2345568 | down | mRNA |
| NM_001012455 | ZSCAN23 | 0.340876666 | 0.828854153 | 1.3045035 | down | mRNA |
| NM_001012506 | CCDC66 | 0.009429939 | 0.204717305 | 1.6241201 | down | mRNA |
| NM_001012507 | CENPW | 0.983431599 | 0.998971807 | 1.0082958 | down | mRNA |
| NM_001012508 | SPRN | 0.981408769 | 0.998971807 | 1.0021747 | down | mRNA |
| NM_001012515 | FECH | 0.897157409 | 0.994222243 | 1.0223553 | down | mRNA |
| NM_001012614 | CTBP1 | 0.668364351 | 0.958740228 | 1.0495144 | down | mRNA |
| NM_001012643 | MYPOP | 0.657658821 | 0.958051096 | 1.1227229 | down | mRNA |
| NM_001012662 | SLC3A2 | 0.989797275 | 0.999140676 | 1.0022356 | down | mRNA |
| NM_001012664 | SLC3A2 | 0.0714613 | 0.461977497 | 1.1698987 | down | mRNA |
| NM_001012709 | KRTAP5-4 | 0.658637494 | 0.958354006 | 1.0546681 | down | mRNA |
| NM_001012715 | C9orf106 | 0.623900855 | 0.948341211 | 1.1484191 | down | mRNA |
| NM_001012722 | RGR | 0.813941424 | 0.98120545 | 1.0139172 | down | mRNA |
| NM_001012727 | AGPAT2 | 0.235131902 | 0.73249698 | 1.0567257 | down | mRNA |
| NM_001012729 | DUXA | 0.868834755 | 0.989481868 | 1.0235756 | down | mRNA |
| NM_001012732 | DCTD | 0.655491424 | 0.957222998 | 1.0654248 | down | mRNA |
| NM_001012753 | ZNF763 | 0.062810634 | 0.439458294 | 1.0683233 | down | mRNA |
| NM_001012758 | NUDT17 | 0.888812485 | 0.993440915 | 1.0211255 | down | mRNA |
| NM_001012759 | CTU2 | 0.067822585 | 0.45182575 | 1.1218323 | down | mRNA |
| NM_001012762 | CTU2 | 0.781379601 | 0.976938981 | 1.0142437 | down | mRNA |
| NM_001012964 | KLK6 | 0.665030389 | 0.958354006 | 1.0293417 | down | mRNA |
| NM_001012967 | DDX60L | 0.87845895 | 0.991717233 | 1.031117 | down | mRNA |
| NM_001012973 | PLAC9 | 0.932534314 | 0.996710991 | 1.0210529 | down | mRNA |
| NM_001012985 | C1orf31 | 0.947855262 | 0.996710991 | 1.0094574 | down | mRNA |
| NM_001012989 | UBE2NL | 0.472968974 | 0.899184677 | 1.0335433 | down | mRNA |
| NM_001012991 | C16orf88 | 0.015474558 | 0.249507308 | 1.205121 | down | mRNA |
| NM_001013251 | SLC3A2 | 0.004076081 | 0.146422035 | 1.2025173 | down | mRNA |
| NM_001013257 | BCAM | 0.459321916 | 0.892904132 | 1.1062802 | down | mRNA |
| NM_001013258 | ZNF789 | 0.064051226 | 0.442157878 | 1.1899229 | down | mRNA |
| NM_001013398 | IGFBP3 | 0.945386873 | 0.996710991 | 1.0108808 | down | mRNA |
| NM_001013406 | KRIT1 | 0.017081334 | 0.262894868 | 1.2419171 | down | mRNA |
| NM_001013437 | SEH1L | 0.051548141 | 0.41031217 | 1.133906 | down | mRNA |
| NM_001013438 | FXR1 | 0.762254009 | 0.97339948 | 1.0465838 | down | mRNA |
| NM_001013579 | AWAT1 | 0.020303195 | 0.285032405 | 2.3409794 | down | mRNA |
| NM_001013615 | LURAP1 | 0.237105824 | 0.735183924 | 1.2436933 | down | mRNA |
| NM_001013620 | ALG10B | 0.881655413 | 0.992766819 | 1.0295369 | down | mRNA |
| NM_001013657 | GDPGP1 | 0.060132446 | 0.435030895 | 1.1267952 | down | mRNA |
| NM_001013663 | PTRHD1 | 0.970484912 | 0.998245244 | 1.0044031 | down | mRNA |
| NM_001013694 | SRRD | 0.734581626 | 0.967777568 | 1.0333777 | down | mRNA |
| NM_001013703 | EIF2AK4 | 0.011813262 | 0.221393704 | 1.3043243 | down | mRNA |
| NM_001013746 | ZNF107 | 0.181835298 | 0.664924727 | 1.1213238 | down | mRNA |
| NM_001013839 | EXOC7 | 0.039959129 | 0.37183021 | 1.1141621 | down | mRNA |
| NM_001013842 | C8orf58 | 0.381477123 | 0.853514109 | 1.1036426 | down | mRNA |
| NM_001013845 | CXorf40B | 0.961456268 | 0.99793927 | 1.0080024 | down | mRNA |
| NM_001014283 | DCUN1D2 | 0.071829038 | 0.462266629 | 1.3263018 | down | mRNA |
| NM_001014286 | FAM48A | 0.113840768 | 0.557835051 | 1.1518532 | down | mRNA |
| NM_001014431 | AKT1 | 0.778213569 | 0.976938981 | 1.0504305 | down | mRNA |
| NM_001014436 | DBNL | 0.551366504 | 0.926149092 | 1.0779922 | down | mRNA |
| NM_001014437 | CARS | 0.592761543 | 0.937820942 | 1.036101 | down | mRNA |
| NM_001014442 | C1QTNF9B-AS1 | 0.877052906 | 0.991255275 | 1.0513949 | down | mRNA |
| NM_001014445 | NLE1 | 0.154441923 | 0.621861274 | 1.1675009 | down | mRNA |
| NM_001014448 | CPZ | 0.747928089 | 0.970692832 | 1.0223382 | down | mRNA |
| NM_001014449 | DDX19B | 0.540025617 | 0.923577434 | 1.0665365 | down | mRNA |
| NM_001014450 | SPRR2F | 0.149718441 | 0.614198134 | 1.115015 | down | mRNA |
| NM_001014794 | ILK | 0.35638875 | 0.839056978 | 1.0650905 | down | mRNA |
| NM_001014811 | ME3 | 0.397845515 | 0.863112281 | 1.1191463 | down | mRNA |
| NM_001014812 | FAM96A | 0.385615748 | 0.85515381 | 1.1163428 | down | mRNA |
| NM_001014840 | CUTA | 0.957492397 | 0.996883425 | 1.0046612 | down | mRNA |
| NM_001014979 | C16orf93 | 0.762703343 | 0.973443358 | 1.0552586 | down | mRNA |
| NM_001014980 | FAM132A | 0.752295612 | 0.970725371 | 1.1074988 | down | mRNA |
| NM_001014987 | LAT | 0.188398926 | 0.673676919 | 1.2667903 | down | mRNA |
| NM_001014988 | LAT | 0.133550569 | 0.590036976 | 1.1035123 | down | mRNA |
| NM_001014990 | MRPL22 | 0.211789271 | 0.703266283 | 1.0671796 | down | mRNA |
| NM_001015000 | SLX1A | 0.518848924 | 0.916145597 | 1.0728202 | down | mRNA |
| NM_001015002 | LLGL2 | 0.780098159 | 0.976938981 | 1.0262028 | down | mRNA |
| NM_001015045 | FAM13A | 0.50233716 | 0.910335149 | 1.1266504 | down | mRNA |
| NM_001015050 | ALG1L | 0.187404296 | 0.671419231 | 1.132369 | down | mRNA |
| NM_001015051 | RUNX2 | 0.097978033 | 0.520535504 | 1.1095172 | down | mRNA |
| NM_001015052 | MPG | 0.736638649 | 0.968717225 | 1.0260848 | down | mRNA |
| NM_001015054 | MPG | 0.757528052 | 0.972045462 | 1.0244548 | down | mRNA |
| NM_001015055 | RTKN | 0.441327142 | 0.883920737 | 1.1949799 | down | mRNA |
| NM_001015879 | AURKC | 0.40758615 | 0.867591329 | 1.2106911 | down | mRNA |
| NM_001015881 | TSC22D3 | 0.023480152 | 0.301578303 | 5.8750112 | down | mRNA |
| NM_001015885 | RAE1 | 0.03350152 | 0.347704395 | 1.2483967 | down | mRNA |
| NM_001017363 | ARID3C | 0.238790286 | 0.736834977 | 1.2115742 | down | mRNA |
| NM_001017369 | MSMO1 | 0.595461276 | 0.938746918 | 1.0854577 | down | mRNA |
| NM_001017370 | NHLRC3 | 0.231644484 | 0.729296323 | 1.4031019 | down | mRNA |
| NM_001017371 | SP3 | 0.996433468 | 0.999872237 | 1.0002778 | down | mRNA |
| NM_001017395 | TMCC1 | 0.585183063 | 0.934708084 | 1.1572343 | down | mRNA |
| NM_001017405 | MAEA | 0.012055788 | 0.222675671 | 1.2494104 | down | mRNA |
| NM_001017408 | GOPC | 0.755459458 | 0.971497464 | 1.0227532 | down | mRNA |
| NM_001017423 | ALDH18A1 | 0.012435109 | 0.226847373 | 1.2580949 | down | mRNA |
| NM_001017424 | KCNK2 | 0.284629676 | 0.78179061 | 1.4359208 | down | mRNA |
| NM_001017425 | KCNK2 | 0.554981521 | 0.926833616 | 1.0759022 | down | mRNA |
| NM_001017434 | PGBD2 | 0.00025185 | 0.052661938 | 2.0436834 | down | mRNA |
| NM_001017437 | CCDC157 | 0.049966692 | 0.40410612 | 1.2524261 | down | mRNA |
| NM_001017524 | SIRT3 | 0.027729999 | 0.32381669 | 1.1405924 | down | mRNA |
| NM_001017528 | PRR5 | 0.91947859 | 0.995349793 | 1.0143144 | down | mRNA |
| NM_001017534 | CARD16 | 0.019660227 | 0.282298294 | 1.0947881 | down | mRNA |
| NM_001017917 | CYB561 | 0.721254095 | 0.966191059 | 1.0268792 | down | mRNA |
| NM_001017919 | RCCD1 | 0.365607986 | 0.845008704 | 1.5504153 | down | mRNA |
| NM_001017921 | VMAC | 0.109434239 | 0.547509603 | 1.0893426 | down | mRNA |
| NM_001017922 | ERMAP | 0.516828522 | 0.91493866 | 1.2294199 | down | mRNA |
| NM_001017923 | C14orf28 | 0.784597267 | 0.977411899 | 1.035341 | down | mRNA |
| NM_001017926 | ZHX1 | 0.002462843 | 0.118591515 | 1.3371438 | down | mRNA |
| NM_001017928 | CCDC58 | 0.865043726 | 0.989401142 | 1.0422317 | down | mRNA |
| NM_001017956 | OS9 | 0.546584751 | 0.924803608 | 1.0758683 | down | mRNA |
| NM_001017957 | OS9 | 0.96256958 | 0.99793927 | 1.0057691 | down | mRNA |
| NM_001017958 | OS9 | 0.673253705 | 0.959531882 | 1.0994937 | down | mRNA |
| NM_001017962 | P4HA1 | 0.30374761 | 0.799455845 | 1.0563823 | down | mRNA |
| NM_001017964 | YDJC | 0.083074327 | 0.491579514 | 1.1562141 | down | mRNA |
| NM_001017969 | KIAA2026 | 0.06771238 | 0.451808679 | 1.2976626 | down | mRNA |
| NM_001017970 | TMEM30B | 0.850324724 | 0.987678166 | 1.0122956 | down | mRNA |
| NM_001017974 | P4HA2 | 0.851327823 | 0.987678166 | 1.063775 | down | mRNA |
| NM_001017977 | DCAF6 | 0.217003116 | 0.7111714 | 1.116878 | down | mRNA |
| NM_001017979 | RAB28 | 0.574247583 | 0.933656777 | 1.141509 | down | mRNA |
| NM_001017987 | C5orf45 | 0.359089651 | 0.840936511 | 1.3149209 | down | mRNA |
| NM_001017990 | H2AFB1 | 0.190496673 | 0.676402188 | 1.2193219 | down | mRNA |
| NM_001017998 | GNG10 | 0.667465261 | 0.958354006 | 1.0361563 | down | mRNA |
| NM_001017999 | KAZN | 0.313400327 | 0.808636478 | 1.2550086 | down | mRNA |
| NM_001018003 | SORBS3 | 0.245173576 | 0.743589595 | 1.0948744 | down | mRNA |
| NM_001018005 | TPM1 | 0.266374074 | 0.767064488 | 1.1696151 | down | mRNA |
| NM_001018006 | TPM1 | 0.136933085 | 0.595872872 | 1.3191473 | down | mRNA |
| NM_001018007 | TPM1 | 0.772881697 | 0.975793214 | 1.056592 | down | mRNA |
| NM_001018008 | TPM1 | 0.051509084 | 0.41031217 | 2.3770117 | down | mRNA |
| NM_001018009 | SH3BP5 | 0.154803948 | 0.622213869 | 1.4074611 | down | mRNA |
| NM_001018024 | MTCP1NB | 0.780249113 | 0.976938981 | 1.0182882 | down | mRNA |
| NM_001018052 | POLR3H | 0.234457085 | 0.731905307 | 1.2701434 | down | mRNA |
| NM_001018053 | PFKFB2 | 0.187784618 | 0.672189586 | 1.1479194 | down | mRNA |
| NM_001018054 | LRP8 | 0.061489839 | 0.437893235 | 1.3005724 | down | mRNA |
| NM_001018055 | BRCC3 | 0.193286151 | 0.68050113 | 1.1470551 | down | mRNA |
| NM_001018056 | VLDLR | 0.105504887 | 0.537720406 | 1.190203 | down | mRNA |
| NM_001018060 | AIFM3 | 0.473566472 | 0.89919252 | 1.1270753 | down | mRNA |
| NM_001018065 | NTRK2 | 0.595084742 | 0.938655001 | 1.1155641 | down | mRNA |
| NM_001018067 | SERBP1 | 0.220559144 | 0.715799221 | 1.0968654 | down | mRNA |
| NM_001018068 | SERBP1 | 0.442134876 | 0.88419144 | 1.0928532 | down | mRNA |
| NM_001018069 | SERBP1 | 0.053131551 | 0.414055462 | 1.1426242 | down | mRNA |
| NM_001018072 | BTBD11 | 0.643053894 | 0.954522001 | 1.0746482 | down | mRNA |
| NM_001018073 | PCK2 | 0.814666789 | 0.981277402 | 1.1224497 | down | mRNA |
| NM_001018090 | GCOM1 | 0.385775724 | 0.855353547 | 1.08947 | down | mRNA |
| NM_001018091 | GCOM1 | 0.01821167 | 0.26991728 | 1.2407307 | down | mRNA |
| NM_001018100 | MYZAP | 0.911363438 | 0.995342392 | 1.0600693 | down | mRNA |
| NM_001018102 | POLR2M | 0.047915896 | 0.398293541 | 1.2322266 | down | mRNA |
| NM_001018104 | FAHD1 | 0.838179944 | 0.986897498 | 1.0357765 | down | mRNA |
| NM_001018109 | PIR | 0.982657638 | 0.998971807 | 1.0063813 | down | mRNA |
| NM_001018111 | PODXL | 0.0397205 | 0.370797613 | 1.8141999 | down | mRNA |
| NM_001018112 | FANCA | 0.998512318 | 0.999889342 | 1.000206 | down | mRNA |
| NM_001018116 | MURC | 0.003808942 | 0.142765478 | 1.4340682 | down | mRNA |
| NM_001018160 | NAE1 | 0.167299631 | 0.642184223 | 1.1595315 | down | mRNA |
| NM_001018161 | PON2 | 0.824961901 | 0.98381451 | 1.0206765 | down | mRNA |
| NM_001018837 | HAX1 | 0.965225322 | 0.99793927 | 1.0043502 | down | mRNA |
| NM_001020658 | PUM1 | 0.01035218 | 0.209736088 | 1.3477096 | down | mRNA |
| NM_001020819 | MYADM | 0.066258355 | 0.449693785 | 1.1641218 | down | mRNA |
| NM_001020825 | NR3C1 | 0.155915558 | 0.623662233 | 1.3166074 | down | mRNA |
| NM_001021 | RPS17 | 0.562314389 | 0.929041598 | 1.017184 | down | mRNA |
| NM_001022 | RPS19 | 0.794225852 | 0.979253685 | 1.0086162 | down | mRNA |
| NM_001023560 | ZNF187 | 0.055154618 | 0.419634622 | 1.8160855 | down | mRNA |
| NM_001023561 | ZNF749 | 0.640860218 | 0.953894897 | 1.0607383 | down | mRNA |
| NM_001023571 | IQCB1 | 0.042124558 | 0.379087707 | 1.3011965 | down | mRNA |
| NM_001024094 | NR3C1 | 0.32416774 | 0.816523598 | 1.277257 | down | mRNA |
| NM_001024215 | FBLIM1 | 0.765039909 | 0.974183719 | 1.1003792 | down | mRNA |
| NM_001024218 | GPHN | 0.774687841 | 0.97589756 | 1.0252063 | down | mRNA |
| NM_001024593 | MSS51 | 0.543368318 | 0.923577434 | 1.1094972 | down | mRNA |
| NM_001024599 | HIST2H2BF | 0.265158341 | 0.766448591 | 1.263597 | down | mRNA |
| NM_001024649 | CANX | 0.676007995 | 0.959531882 | 1.0585015 | down | mRNA |
| NM_001024656 | ASPDH | 0.33846445 | 0.827403747 | 1.5527035 | down | mRNA |
| NM_001024674 | LIN52 | 0.125216078 | 0.578441772 | 1.352188 | down | mRNA |
| NM_001024678 | LRRC24 | 0.486443058 | 0.903524001 | 1.1033784 | down | mRNA |
| NM_001024732 | MECR | 0.314730081 | 0.809824265 | 1.0621447 | down | mRNA |
| NM_001024809 | RARA | 0.127421893 | 0.580991132 | 1.1574024 | down | mRNA |
| NM_001024855 | ZNF197 | 0.033315211 | 0.346667968 | 1.2859415 | down | mRNA |
| NM_001024912 | CEACAM1 | 0.093396064 | 0.512497334 | 1.2315176 | down | mRNA |
| NM_001024924 | EXOC1 | 0.216799195 | 0.711074992 | 1.0974392 | down | mRNA |
| NM_001024939 | SLC2A11 | 0.996580671 | 0.999872237 | 1.000529 | down | mRNA |
| NM_001024944 | ASL | 0.459443132 | 0.892904132 | 1.0507451 | down | mRNA |
| NM_001024946 | ASL | 0.977727124 | 0.998501954 | 1.0019848 | down | mRNA |
| NM_001024947 | YIPF5 | 0.098064104 | 0.520720649 | 1.1322748 | down | mRNA |
| NM_001024956 | SC5DL | 0.585571754 | 0.934869412 | 1.063753 | down | mRNA |
| NM_001024957 | BRMS1 | 0.04796053 | 0.398338064 | 1.225114 | down | mRNA |
| NM_001024959 | ARPC4 | 0.685565925 | 0.960111041 | 1.0279666 | down | mRNA |
| NM_001025076 | CELF2 | 0.759040286 | 0.972369912 | 1.1685679 | down | mRNA |
| NM_001025087 | CELF4 | 0.969119042 | 0.998233363 | 1.0048539 | down | mRNA |
| NM_001025089 | CELF4 | 0.770175364 | 0.975286259 | 1.0356453 | down | mRNA |
| NM_001025096 | PSMA8 | 0.754310391 | 0.970980619 | 1.0542103 | down | mRNA |
| NM_001025100 | MBP | 0.472872057 | 0.899184677 | 1.2813616 | down | mRNA |
| NM_001025109 | CD34 | 0.236712724 | 0.73465251 | 1.5378117 | down | mRNA |
| NM_001025158 | CD74 | 0.89636629 | 0.994213049 | 1.023656 | down | mRNA |
| NM_001025159 | CD74 | 0.485996054 | 0.903370052 | 1.2398897 | down | mRNA |
| NM_001025200 | CTRB2 | 0.641064404 | 0.954041974 | 1.0388817 | down | mRNA |
| NM_001025203 | U2AF1 | 0.685254976 | 0.960111041 | 1.0396284 | down | mRNA |
| NM_001025204 | U2AF1 | 0.833085054 | 0.986434891 | 1.012106 | down | mRNA |
| NM_001025205 | AP2M1 | 0.020888845 | 0.287161595 | 1.1550223 | down | mRNA |
| NM_001025238 | TSPAN4 | 0.008989767 | 0.202329498 | 1.1569592 | down | mRNA |
| NM_001025242 | IRAK1 | 0.44967799 | 0.888279993 | 1.0624689 | down | mRNA |
| NM_001025248 | DUT | 0.988858236 | 0.999140676 | 1.0007919 | down | mRNA |
| NM_001025249 | DUT | 0.484683573 | 0.903250293 | 1.0317095 | down | mRNA |
| NM_001025265 | CXorf65 | 0.436277669 | 0.881749261 | 1.5285497 | down | mRNA |
| NM_001025300 | RAB12 | 0.026720917 | 0.318484367 | 1.1244188 | down | mRNA |
| NM_001025357 | AARD | 0.347734267 | 0.834082222 | 1.059941 | down | mRNA |
| NM_001025433 | NQO1 | 0.992913833 | 0.999798141 | 1.0025676 | down | mRNA |
| NM_001025434 | NQO1 | 0.872978053 | 0.990534615 | 1.0593373 | down | mRNA |
| NM_001025436 | SPAG16 | 0.394265982 | 0.86105621 | 1.1134309 | down | mRNA |
| NM_001025579 | NDEL1 | 0.219135197 | 0.71361728 | 1.3102861 | down | mRNA |
| NM_001025580 | AMMECR1 | 0.803514857 | 0.98118927 | 1.0201038 | down | mRNA |
| NM_001025595 | ARFIP1 | 0.963465241 | 0.99793927 | 1.0026334 | down | mRNA |
| NM_001025780 | FAM108B1 | 0.110034491 | 0.549011353 | 1.7940873 | down | mRNA |
| NM_001026 | RPS24 | 0.895465279 | 0.993978977 | 1.0075411 | down | mRNA |
| NM_001026383 | CCDC7 | 0.816498875 | 0.981692737 | 1.0319549 | down | mRNA |
| NM_001028 | RPS25 | 0.847862928 | 0.987678166 | 1.0036287 | down | mRNA |
| NM_001029859 | KCTD21 | 0.026865929 | 0.319244809 | 1.4194579 | down | mRNA |
| NM_001029864 | KIAA1755 | 0.345947704 | 0.833093536 | 1.0897169 | down | mRNA |
| NM_001029865 | DBX1 | 0.31637992 | 0.810870607 | 1.3454215 | down | mRNA |
| NM_001029869 | PLAC8L1 | 0.096819501 | 0.518991327 | 1.2939774 | down | mRNA |
| NM_001029880 | SFMBT2 | 0.363051891 | 0.843238468 | 1.1513462 | down | mRNA |
| NM_001029882 | AHDC1 | 0.005246234 | 0.165444121 | 1.7043785 | down | mRNA |
| NM_001029885 | GLTPD1 | 0.450737107 | 0.888732431 | 1.1661532 | down | mRNA |
| NM_001029991 | METTL17 | 0.05247699 | 0.412637354 | 1.4368989 | down | mRNA |
| NM_001029997 | ZNF181 | 0.000829569 | 0.083087642 | 1.4081864 | down | mRNA |
| NM_001029998 | SLC10A7 | 0.047943987 | 0.398338064 | 1.3567111 | down | mRNA |
| NM_001030001 | RPS29 | 0.242658981 | 0.740802161 | 1.123765 | down | mRNA |
| NM_001030005 | CPLX3 | 0.436109226 | 0.881677506 | 1.3916662 | down | mRNA |
| NM_001030006 | AP2B1 | 0.580077747 | 0.933893479 | 1.1097284 | down | mRNA |
| NM_001030007 | AP1G1 | 0.622773099 | 0.948341211 | 1.0637689 | down | mRNA |
| NM_001030273 | ARNTL | 0.327754249 | 0.820730147 | 1.4495676 | down | mRNA |
| NM_001030288 | SPN | 0.677319927 | 0.959531882 | 1.0444128 | down | mRNA |
| NM_001031623 | ZNF451 | 0.012020803 | 0.222675671 | 1.5652348 | down | mRNA |
| NM_001031665 | ZNF816 | 0.217344862 | 0.711339274 | 1.6392384 | down | mRNA |
| NM_001031683 | IFIT3 | 0.988272491 | 0.999140676 | 1.0026996 | down | mRNA |
| NM_001031689 | PLAA | 0.036613059 | 0.359248842 | 1.1868114 | down | mRNA |
| NM_001031692 | LRRC17 | 0.983279007 | 0.998971807 | 1.0139072 | down | mRNA |
| NM_001031693 | HHLA3 | 0.587503672 | 0.93579682 | 1.1469722 | down | mRNA |
| NM_001031695 | RBFOX2 | 0.467652322 | 0.896909118 | 1.2519553 | down | mRNA |
| NM_001031701 | NT5DC3 | 0.001309403 | 0.093126586 | 1.5425106 | down | mRNA |
| NM_001031703 | ELP6 | 0.083185137 | 0.491805818 | 1.1935803 | down | mRNA |
| NM_001031712 | TRMT11 | 0.235436951 | 0.732838961 | 1.1425199 | down | mRNA |
| NM_001031717 | CRELD1 | 0.720850338 | 0.966191059 | 1.0307498 | down | mRNA |
| NM_001031721 | ZNF613 | 0.0080457 | 0.195676039 | 1.545087 | down | mRNA |
| NM_001031722 | ATAT1 | 0.571361869 | 0.933118573 | 1.0495227 | down | mRNA |
| NM_001031723 | DNAJB14 | 0.032249532 | 0.343117475 | 1.2507355 | down | mRNA |
| NM_001031725 | DDX59 | 0.642339067 | 0.954332328 | 1.0628316 | down | mRNA |
| NM_001031726 | C19orf12 | 0.266925107 | 0.767456064 | 1.079504 | down | mRNA |
| NM_001031732 | YTHDC1 | 0.167037821 | 0.642006233 | 1.182923 | down | mRNA |
| NM_001031733 | CALML4 | 0.201142007 | 0.689184243 | 1.3834983 | down | mRNA |
| NM_001031734 | FDX1L | 0.45395704 | 0.890139314 | 1.2291186 | down | mRNA |
| NM_001031806 | ALDH3A2 | 0.907233263 | 0.995145546 | 1.0170277 | down | mRNA |
| NM_001031812 | CSNK1G3 | 0.315734484 | 0.810520991 | 1.1619336 | down | mRNA |
| NM_001031827 | BOLA2 | 0.739328089 | 0.969564098 | 1.0605371 | down | mRNA |
| NM_001031835 | PHKB | 0.51127311 | 0.912481293 | 1.1128503 | down | mRNA |
| NM_001031836 | KCNU1 | 0.26303291 | 0.763125731 | 1.2653316 | down | mRNA |
| NM_001031847 | CPT1A | 0.948681544 | 0.996710991 | 1.0174359 | down | mRNA |
| NM_001031849 | MASP1 | 0.739261379 | 0.969564098 | 1.0410988 | down | mRNA |
| NM_001032221 | STXBP1 | 0.672765693 | 0.959531882 | 1.0259203 | down | mRNA |
| NM_001032283 | TMPO | 0.673377412 | 0.959531882 | 1.2221859 | down | mRNA |
| NM_001032284 | TMPO | 0.720663543 | 0.966191059 | 1.1646719 | down | mRNA |
| NM_001032287 | NR2C1 | 0.034480145 | 0.351579889 | 1.2352424 | down | mRNA |
| NM_001032292 | ZNF174 | 0.014971737 | 0.24511734 | 1.2282884 | down | mRNA |
| NM_001032296 | STK24 | 0.34064973 | 0.828777111 | 1.0970394 | down | mRNA |
| NM_001032363 | MINOS1 | 0.610861277 | 0.943627981 | 1.0350601 | down | mRNA |
| NM_001032372 | ZNF226 | 0.29455088 | 0.791301949 | 1.1334167 | down | mRNA |
| NM_001032391 | LCMT1 | 0.204536144 | 0.694609801 | 1.0647116 | down | mRNA |
| NM_001032392 | PLGLB1 | 0.673764597 | 0.959531882 | 1.0414235 | down | mRNA |
| NM_001032394 | GPR126 | 0.563253646 | 0.929041598 | 1.2992393 | down | mRNA |
| NM_001032395 | GPR126 | 0.458962857 | 0.892904132 | 1.1194721 | down | mRNA |
| NM_001032396 | PJA1 | 0.082511947 | 0.490238261 | 1.2287538 | down | mRNA |
| NM_001032730 | USP48 | 0.340012382 | 0.828118322 | 1.0733854 | down | mRNA |
| NM_001032731 | OAS2 | 0.186220362 | 0.669888134 | 1.3655208 | down | mRNA |
| NM_001033 | RRM1 | 0.891839263 | 0.9937521 | 1.0419234 | down | mRNA |
| NM_001033032 | FAIM | 0.567035714 | 0.931483408 | 1.066991 | down | mRNA |
| NM_001033045 | GPR155 | 0.327490183 | 0.820319134 | 1.4449482 | down | mRNA |
| NM_001033050 | MTERFD3 | 0.293966351 | 0.791146775 | 1.1181054 | down | mRNA |
| NM_001033053 | NLRP1 | 0.49228186 | 0.906044608 | 1.2427254 | down | mRNA |
| NM_001033055 | AIPL1 | 0.989344533 | 0.999140676 | 1.0010369 | down | mRNA |
| NM_001033057 | MAGI1 | 0.760173593 | 0.972883253 | 1.1012286 | down | mRNA |
| NM_001033059 | AMD1 | 0.003518414 | 0.13849526 | 1.3176765 | down | mRNA |
| NM_001033088 | NGRN | 0.566848279 | 0.931401262 | 1.136884 | down | mRNA |
| NM_001033112 | PAIP2 | 0.941499229 | 0.996710991 | 1.0085754 | down | mRNA |
| NM_001033505 | CSTF3 | 0.840673616 | 0.987068842 | 1.026287 | down | mRNA |
| NM_001033506 | CSTF3 | 0.122805833 | 0.574983504 | 1.2849985 | down | mRNA |
| NM_001033518 | WIPI2 | 0.347317817 | 0.83359566 | 1.0429623 | down | mRNA |
| NM_001033557 | PPM1B | 0.722990501 | 0.966191059 | 1.0786702 | down | mRNA |
| NM_001033561 | PHF12 | 0.297041676 | 0.793620415 | 1.4161862 | down | mRNA |
| NM_001033566 | RHOT1 | 0.051768161 | 0.41031217 | 1.2102869 | down | mRNA |
| NM_001033568 | RHOT1 | 0.065303343 | 0.446303313 | 1.2019701 | down | mRNA |
| NM_001033569 | AMZ2 | 0.591775089 | 0.937303262 | 1.1022945 | down | mRNA |
| NM_001033574 | AMZ2 | 0.420697694 | 0.872676791 | 1.1597454 | down | mRNA |
| NM_001033678 | TRPT1 | 0.912289348 | 0.995342392 | 1.02399 | down | mRNA |
| NM_001033714 | NOP2 | 0.473136415 | 0.899184677 | 1.1612632 | down | mRNA |
| NM_001033719 | ZNF404 | 0.183575066 | 0.667104243 | 1.2558562 | down | mRNA |
| NM_001033855 | DCLRE1C | 0.47008984 | 0.898057982 | 1.1371573 | down | mRNA |
| NM_001033859 | ACADVL | 0.837344266 | 0.986775289 | 1.0181747 | down | mRNA |
| NM_001033925 | TIAL1 | 0.003917549 | 0.144296388 | 1.3071121 | down | mRNA |
| NM_001034 | RRM2 | 0.953183275 | 0.996710991 | 1.0424508 | down | mRNA |
| NM_001034024 | DMAP1 | 0.158331732 | 0.628995854 | 1.2057617 | down | mRNA |
| NM_001034077 | HIST2H4B | 0.964392343 | 0.99793927 | 1.0216228 | down | mRNA |
| NM_001034116 | EIF2B4 | 0.227234249 | 0.72290573 | 1.1054893 | down | mRNA |
| NM_001034173 | ALDH1L2 | 0.146388365 | 0.608122555 | 1.1348346 | down | mRNA |
| NM_001034194 | EXOSC9 | 0.598790385 | 0.940648177 | 1.0881544 | down | mRNA |
| NM_001034833 | NHP2 | 0.609532787 | 0.943053241 | 1.0499702 | down | mRNA |
| NM_001034838 | KCNIP1 | 0.347808927 | 0.834082222 | 1.1913605 | down | mRNA |
| NM_001034841 | ITPRIPL2 | 0.806311499 | 0.98118927 | 1.0205683 | down | mRNA |
| NM_001034853 | RPGR | 0.952772182 | 0.996710991 | 1.0097562 | down | mRNA |
| NM_001034996 | RPL14 | 0.662681943 | 0.958354006 | 1.0568163 | down | mRNA |
| NM_001035235 | SRA1 | 0.864546266 | 0.989401142 | 1.0118211 | down | mRNA |
| NM_001035254 | FAM102A | 0.504411891 | 0.910568383 | 1.040118 | down | mRNA |
| NM_001035260 | VPS26A | 0.147726365 | 0.610482204 | 1.0921239 | down | mRNA |
| NM_001035507 | AGBL5 | 0.713527156 | 0.966110889 | 1.0596545 | down | mRNA |
| NM_001037126 | EXOC4 | 0.043754291 | 0.385351177 | 1.2191764 | down | mRNA |
| NM_001037131 | AGAP1 | 0.265406448 | 0.766537754 | 1.1347315 | down | mRNA |
| NM_001037144 | CNTROB | 0.160906211 | 0.632304808 | 1.1608117 | down | mRNA |
| NM_001037161 | ACOT1 | 0.692448815 | 0.961584652 | 1.1383777 | down | mRNA |
| NM_001037163 | FAM220A | 0.080573715 | 0.484779682 | 1.3742283 | down | mRNA |
| NM_001037165 | FOXK1 | 0.233528399 | 0.73118887 | 1.1647516 | down | mRNA |
| NM_001037277 | GGPS1 | 0.283798793 | 0.781657689 | 1.1654438 | down | mRNA |
| NM_001037288 | CMTM5 | 0.992786236 | 0.999798141 | 1.0012581 | down | mRNA |
| NM_001037305 | THYN1 | 0.932174766 | 0.996710991 | 1.0080544 | down | mRNA |
| NM_001037333 | CYFIP2 | 0.334729156 | 0.824597423 | 1.0873831 | down | mRNA |
| NM_001037334 | USP14 | 0.57683087 | 0.933656777 | 1.0414155 | down | mRNA |
| NM_001037442 | RUFY3 | 0.132537812 | 0.588118462 | 1.2415125 | down | mRNA |
| NM_001037494 | DYNLL1 | 0.014079998 | 0.239157213 | 1.1682439 | down | mRNA |
| NM_001037533 | GON4L | 0.052482796 | 0.412637354 | 1.2544303 | down | mRNA |
| NM_001037537 | PHYH | 0.875616678 | 0.991094934 | 1.0222568 | down | mRNA |
| NM_001037637 | BTF3 | 0.495734923 | 0.907879882 | 1.0474332 | down | mRNA |
| NM_001037639 | PARL | 0.051212414 | 0.409457648 | 1.1536823 | down | mRNA |
| NM_001037671 | C12orf74 | 0.667296123 | 0.958354006 | 1.0427515 | down | mRNA |
| NM_001037732 | DEFB128 | 0.921547048 | 0.995349793 | 1.0139439 | down | mRNA |
| NM_001037738 | NPM1 | 0.947524952 | 0.996710991 | 1.0049523 | down | mRNA |
| NM_001037763 | COL28A1 | 0.161611954 | 0.633561373 | 1.2993122 | down | mRNA |
| NM_001037804 | DEFB130 | 0.021356842 | 0.288424269 | 1.2791639 | down | mRNA |
| NM_001037808 | EIF3C | 0.087378294 | 0.500725893 | 1.1249086 | down | mRNA |
| NM_001037811 | HSD17B10 | 0.68360132 | 0.960111041 | 1.0477312 | down | mRNA |
| NM_001037872 | REV1 | 0.005591666 | 0.169833068 | 1.4963911 | down | mRNA |
| NM_001037968 | ALAS2 | 0.418134035 | 0.871927185 | 1.3111449 | down | mRNA |
| NM_001037984 | SLC38A10 | 0.578677612 | 0.933821303 | 1.0350918 | down | mRNA |
| NM_001038 | SCNN1A | 0.421922096 | 0.873398499 | 1.0749136 | down | mRNA |
| NM_001038493 | DLX1 | 0.553590694 | 0.926337111 | 1.1920061 | down | mRNA |
| NM_001038603 | MARVELD2 | 0.397169366 | 0.862344286 | 1.1100737 | down | mRNA |
| NM_001038618 | NARF | 0.155448214 | 0.623056705 | 1.162273 | down | mRNA |
| NM_001039091 | PRPS2 | 0.087639991 | 0.501313828 | 1.2738474 | down | mRNA |
| NM_001039127 | ZNF718 | 0.105994319 | 0.53890385 | 1.1383365 | down | mRNA |
| NM_001039131 | ALOX15B | 0.173950323 | 0.652177388 | 1.1973077 | down | mRNA |
| NM_001039165 | MRGPRE | 0.865943404 | 0.989454945 | 1.0456943 | down | mRNA |
| NM_001039213 | CEACAM16 | 0.100660675 | 0.527482692 | 1.3011949 | down | mRNA |
| NM_001039348 | EFEMP1 | 0.870419391 | 0.989592579 | 1.0318424 | down | mRNA |
| NM_001039360 | ZBTB7C | 0.368813816 | 0.845559574 | 1.1362225 | down | mRNA |
| NM_001039361 | PRAMEF10 | 0.921388103 | 0.995349793 | 1.0152025 | down | mRNA |
| NM_001039366 | ATP6V1E1 | 0.554370341 | 0.926549129 | 1.0389045 | down | mRNA |
| NM_001039374 | KIAA1984 | 0.869959909 | 0.989592579 | 1.0087947 | down | mRNA |
| NM_001039375 | NDUFAF5 | 0.146908849 | 0.609050728 | 1.1483908 | down | mRNA |
| NM_001039457 | ATP6V0B | 0.182067331 | 0.665333517 | 1.0852187 | down | mRNA |
| NM_001039467 | RGS19 | 0.032848923 | 0.34498156 | 1.2074827 | down | mRNA |
| NM_001039476 | NPRL3 | 0.163199921 | 0.63542336 | 1.1076144 | down | mRNA |
| NM_001039477 | THEMIS2 | 0.331847137 | 0.822255387 | 1.0849785 | down | mRNA |
| NM_001039479 | KIAA0317 | 0.890783385 | 0.9937521 | 1.0151139 | down | mRNA |
| NM_001039490 | CD151 | 0.530341769 | 0.919024021 | 1.043848 | down | mRNA |
| NM_001039503 | PRSS53 | 0.347260008 | 0.833588015 | 1.1225008 | down | mRNA |
| NM_001039535 | SKA1 | 0.886602266 | 0.992981836 | 1.1398584 | down | mRNA |
| NM_001039550 | DNAJB2 | 0.295561022 | 0.791662837 | 1.2661821 | down | mRNA |
| NM_001039569 | AP1S3 | 0.960324493 | 0.997738713 | 1.0268239 | down | mRNA |
| NM_001039573 | SEC14L1 | 0.010882552 | 0.213674567 | 1.2255852 | down | mRNA |
| NM_001039574 | KCNC4 | 0.545141434 | 0.924012779 | 1.1420636 | down | mRNA |
| NM_001039580 | MAP9 | 0.742431369 | 0.970330526 | 1.2438835 | down | mRNA |
| NM_001039582 | PNCK | 0.609582358 | 0.943053241 | 1.093515 | down | mRNA |
| NM_001039584 | PICK1 | 0.33834787 | 0.827403747 | 1.2767767 | down | mRNA |
| NM_001039589 | DPH2 | 0.684944328 | 0.960111041 | 1.0785502 | down | mRNA |
| NM_001039590 | USP9X | 0.506196675 | 0.911278334 | 1.1656546 | down | mRNA |
| NM_001039591 | USP9X | 0.703271209 | 0.963625105 | 1.0944259 | down | mRNA |
| NM_001039613 | IAH1 | 0.595302685 | 0.938707475 | 1.0915592 | down | mRNA |
| NM_001039614 | C15orf59 | 0.038816038 | 0.368040971 | 1.1132233 | down | mRNA |
| NM_001039617 | ZDHHC19 | 0.103538132 | 0.533123263 | 1.4019115 | down | mRNA |
| NM_001039649 | ZMYM5 | 0.362373051 | 0.843173209 | 1.0714033 | down | mRNA |
| NM_001039650 | ZMYM5 | 0.970129099 | 0.998245244 | 1.0036459 | down | mRNA |
| NM_001039651 | SAPCD1 | 0.468822202 | 0.897560548 | 1.2330214 | down | mRNA |
| NM_001039654 | ZNF550 | 0.03468817 | 0.352074963 | 1.3107525 | down | mRNA |
| NM_001039670 | IFFO1 | 0.36636227 | 0.845181673 | 1.1222145 | down | mRNA |
| NM_001039693 | TYW5 | 0.812234819 | 0.98118927 | 1.0247716 | down | mRNA |
| NM_001039697 | SNAPC3 | 0.099212675 | 0.523402206 | 1.1067209 | down | mRNA |
| NM_001039705 | TRO | 0.339461442 | 0.827966656 | 1.2123885 | down | mRNA |
| NM_001039707 | SDCCAG3 | 0.916041332 | 0.995349793 | 1.0172473 | down | mRNA |
| NM_001039708 | SDCCAG3 | 0.274113084 | 0.774279969 | 1.2222486 | down | mRNA |
| NM_001039711 | DEDD | 0.188825843 | 0.674422532 | 1.1628179 | down | mRNA |
| NM_001039715 | DIO1 | 0.940967778 | 0.996710991 | 1.0241635 | down | mRNA |
| NM_001039717 | C4orf29 | 0.41754936 | 0.871416436 | 1.0940275 | down | mRNA |
| NM_001039753 | EML6 | 0.773646008 | 0.97589756 | 1.1649117 | down | mRNA |
| NM_001039770 | TMPPE | 0.188069336 | 0.672871083 | 1.2909824 | down | mRNA |
| NM_001039802 | CDC42 | 0.955640228 | 0.996883425 | 1.004644 | down | mRNA |
| NM_001039840 | CHIC1 | 0.079490547 | 0.482639336 | 1.1050918 | down | mRNA |
| NM_001039886 | ZNF808 | 0.022916649 | 0.298107041 | 1.4193845 | down | mRNA |
| NM_001039888 | ANKRD34A | 0.424411923 | 0.874854499 | 1.1066515 | down | mRNA |
| NM_001039891 | ZNF674 | 0.228861429 | 0.725125586 | 1.1389613 | down | mRNA |
| NM_001039905 | C15orf56 | 0.284295886 | 0.78179061 | 1.2684835 | down | mRNA |
| NM_001039960 | SLC4A8 | 0.241487641 | 0.739781033 | 1.33687 | down | mRNA |
| NM_001039999 | FAM83G | 0.682456199 | 0.960111041 | 1.032052 | down | mRNA |
| NM_001040 | SHBG | 0.173441241 | 0.65174838 | 1.5414729 | down | mRNA |
| NM_001040000 | MLLT4 | 0.410160855 | 0.86807846 | 1.1551608 | down | mRNA |
| NM_001040002 | MEOX1 | 0.316325988 | 0.810834592 | 1.4057373 | down | mRNA |
| NM_001040006 | TCERG1 | 0.786025382 | 0.977479172 | 1.009656 | down | mRNA |
| NM_001040023 | SIRPA | 0.767856795 | 0.975083653 | 1.0501863 | down | mRNA |
| NM_001040056 | MAPK3 | 0.91963215 | 0.995349793 | 1.0155573 | down | mRNA |
| NM_001040078 | LGALS9C | 0.781727169 | 0.976938981 | 1.0289938 | down | mRNA |
| NM_001040084 | ANXA8 | 0.667332226 | 0.958354006 | 1.0676954 | down | mRNA |
| NM_001040092 | ENPP2 | 0.344254272 | 0.831959196 | 1.1652434 | down | mRNA |
| NM_001040099 | MOSPD3 | 0.243682794 | 0.742130994 | 1.2637734 | down | mRNA |
| NM_001040101 | D4S234E | 0.710679848 | 0.965555808 | 1.0884516 | down | mRNA |
| NM_001040103 | OPALIN | 0.266127169 | 0.767064488 | 1.1290305 | down | mRNA |
| NM_001040118 | ARAP1 | 0.044225861 | 0.386586501 | 1.236576 | down | mRNA |
| NM_001040126 | PQLC2 | 0.02486714 | 0.307916375 | 1.8661983 | down | mRNA |
| NM_001040135 | ACTR3B | 0.034140852 | 0.349933975 | 1.4585581 | down | mRNA |
| NM_001040152 | PEG10 | 0.545380239 | 0.924012779 | 1.1979741 | down | mRNA |
| NM_001040153 | SLAIN1 | 0.973129675 | 0.998501532 | 1.0019616 | down | mRNA |
| NM_001040157 | CEP44 | 0.97181537 | 0.998501532 | 1.0027154 | down | mRNA |
| NM_001040158 | H2AFY | 0.683949334 | 0.960111041 | 1.0764848 | down | mRNA |
| NM_001040161 | C16orf13 | 0.479755131 | 0.901538317 | 1.0570901 | down | mRNA |
| NM_001040165 | C16orf13 | 0.308942855 | 0.803961878 | 1.0656337 | down | mRNA |
| NM_001040168 | LFNG | 0.433570253 | 0.880871258 | 1.3402181 | down | mRNA |
| NM_001040172 | HTR4 | 0.12988824 | 0.583907255 | 1.5500738 | down | mRNA |
| NM_001040183 | CLDND1 | 0.375422055 | 0.849862269 | 1.1091809 | down | mRNA |
| NM_001040185 | ZNF765 | 0.00154475 | 0.100989914 | 1.3827339 | down | mRNA |
| NM_001040194 | AGTRAP | 0.959249493 | 0.997466408 | 1.0034848 | down | mRNA |
| NM_001040196 | AGTRAP | 0.781422653 | 0.976938981 | 1.0379746 | down | mRNA |
| NM_001040200 | CLDND1 | 0.578341969 | 0.933821303 | 1.056317 | down | mRNA |
| NM_001040202 | PAQR3 | 0.1401668 | 0.60022565 | 1.1300841 | down | mRNA |
| NM_001040214 | NKAIN2 | 0.34989236 | 0.834214008 | 1.2709998 | down | mRNA |
| NM_001040260 | DCLK2 | 0.001386176 | 0.095442746 | 2.2561699 | down | mRNA |
| NM_001040272 | ADAMTSL1 | 0.392624212 | 0.859694706 | 1.0540395 | down | mRNA |
| NM_001040282 | TBC1D3G | 0.242250953 | 0.740610726 | 1.111055 | down | mRNA |
| NM_001040284 | PAPD5 | 0.002460423 | 0.118591515 | 1.6991281 | down | mRNA |
| NM_001040402 | DCUN1D4 | 0.063777963 | 0.441366077 | 1.2277233 | down | mRNA |
| NM_001040412 | C9orf131 | 0.092593546 | 0.511338764 | 1.160263 | down | mRNA |
| NM_001040425 | U2AF1L4 | 0.446507784 | 0.886701288 | 1.2358837 | down | mRNA |
| NM_001040428 | SPATA7 | 0.218148936 | 0.712269655 | 1.4685727 | down | mRNA |
| NM_001040431 | COA3 | 0.206686885 | 0.698066071 | 1.0986552 | down | mRNA |
| NM_001040438 | C6orf48 | 0.557283111 | 0.926833616 | 1.1310212 | down | mRNA |
| NM_001040440 | CCDC112 | 0.841327865 | 0.987068842 | 1.0294997 | down | mRNA |
| NM_001040441 | ZBTB8A | 0.001828779 | 0.105670067 | 1.1902561 | down | mRNA |
| NM_001040446 | MTMR12 | 0.050177221 | 0.40548619 | 1.1963102 | down | mRNA |
| NM_001040450 | FAM63B | 0.143841146 | 0.604279163 | 1.2536567 | down | mRNA |
| NM_001040453 | FAM63B | 0.074704589 | 0.469867247 | 1.265 | down | mRNA |
| NM_001040454 | SLC26A6 | 0.612048613 | 0.944027644 | 1.0929236 | down | mRNA |
| NM_001040455 | SIDT2 | 0.129549682 | 0.583907255 | 1.3797023 | down | mRNA |
| NM_001040456 | RHBDD2 | 0.179199418 | 0.661369134 | 1.1099363 | down | mRNA |
| NM_001040462 | BTNL8 | 0.300895478 | 0.795950279 | 1.183991 | down | mRNA |
| NM_001040610 | EFTUD1 | 0.337900292 | 0.827394958 | 1.1638405 | down | mRNA |
| NM_001040613 | TMEM70 | 0.004903477 | 0.158753988 | 1.2428409 | down | mRNA |
| NM_001040616 | LINS | 0.013524011 | 0.235723775 | 1.4736888 | down | mRNA |
| NM_001040633 | PRKAG2 | 0.74512224 | 0.970378122 | 1.0643462 | down | mRNA |
| NM_001040647 | CRCP | 0.035577893 | 0.354419264 | 1.2555786 | down | mRNA |
| NM_001040648 | CRCP | 0.531559104 | 0.920228311 | 1.0779329 | down | mRNA |
| NM_001040649 | ACP1 | 0.093422777 | 0.512497334 | 1.0758294 | down | mRNA |
| NM_001040653 | ZXDC | 0.021132982 | 0.288424269 | 1.2384099 | down | mRNA |
| NM_001040694 | INCENP | 0.958029574 | 0.996933183 | 1.0358534 | down | mRNA |
| NM_001040697 | UEVLD | 0.389466357 | 0.858209114 | 1.0653435 | down | mRNA |
| NM_001040701 | FUT6 | 0.952618696 | 0.996710991 | 1.0103153 | down | mRNA |
| NM_001040715 | KIAA0895L | 0.725643726 | 0.966191059 | 1.0428206 | down | mRNA |
| NM_001040876 | ABCE1 | 0.013813733 | 0.236813499 | 1.111691 | down | mRNA |
| NM_001042351 | G6PD | 0.974492262 | 0.998501532 | 1.0023379 | down | mRNA |
| NM_001042361 | PTPN20B | 0.560495548 | 0.92782421 | 1.0410505 | down | mRNA |
| NM_001042369 | TROVE2 | 0.549362547 | 0.925481755 | 1.0394043 | down | mRNA |
| NM_001042370 | TROVE2 | 0.016333124 | 0.255922225 | 1.0852015 | down | mRNA |
| NM_001042371 | PGP | 0.575105811 | 0.933656777 | 1.0797141 | down | mRNA |
| NM_001042376 | INS-IGF2 | 0.751866315 | 0.970725371 | 1.036395 | down | mRNA |
| NM_001042383 | CEP63 | 0.695017686 | 0.962214902 | 1.0116942 | down | mRNA |
| NM_001042384 | CEP63 | 0.576344466 | 0.933656777 | 1.0796236 | down | mRNA |
| NM_001042386 | PREPL | 0.520170656 | 0.916394575 | 1.1174364 | down | mRNA |
| NM_001042396 | PTPN20A | 0.203963311 | 0.693939432 | 1.0926174 | down | mRNA |
| NM_001042400 | CEP63 | 0.328827602 | 0.821644684 | 1.1091756 | down | mRNA |
| NM_001042402 | NAAA | 0.320927459 | 0.814914437 | 1.4757405 | down | mRNA |
| NM_001042404 | CEP170 | 0.096899136 | 0.519207575 | 1.314314 | down | mRNA |
| NM_001042405 | CEP170 | 0.016156401 | 0.255635752 | 1.2267425 | down | mRNA |
| NM_001042410 | ANKZF1 | 0.831889536 | 0.986141573 | 1.0319785 | down | mRNA |
| NM_001042416 | ZNF596 | 0.001262821 | 0.092399617 | 1.6411497 | down | mRNA |
| NM_001042423 | SLC16A3 | 0.362518176 | 0.843173209 | 1.0659189 | down | mRNA |
| NM_001042426 | CENPA | 0.518723553 | 0.916145597 | 1.2591131 | down | mRNA |
| NM_001042428 | ZNF205 | 0.810705604 | 0.98118927 | 1.0420306 | down | mRNA |
| NM_001042429 | FAM98B | 0.239781131 | 0.737986583 | 1.6234535 | down | mRNA |
| NM_001042430 | ZC2HC1C | 0.7415207 | 0.970240145 | 1.0775231 | down | mRNA |
| NM_001042437 | ST3GAL5 | 0.494458342 | 0.906930718 | 1.3335654 | down | mRNA |
| NM_001042469 | SUMF2 | 0.896953687 | 0.994222243 | 1.0191787 | down | mRNA |
| NM_001042470 | SUMF2 | 0.673282459 | 0.959531882 | 1.0672562 | down | mRNA |
| NM_001042472 | ABHD12 | 0.646044922 | 0.95550188 | 1.0140491 | down | mRNA |
| NM_001042473 | ACBD5 | 0.717875515 | 0.966191059 | 1.0408716 | down | mRNA |
| NM_001042475 | CEP85L | 0.013009584 | 0.233238 | 1.5883914 | down | mRNA |
| NM_001042482 | TPK1 | 0.507759346 | 0.911674633 | 1.2218655 | down | mRNA |
| NM_001042483 | NUPR1 | 0.76664571 | 0.974840715 | 1.0655783 | down | mRNA |
| NM_001042495 | SLC12A6 | 0.547382446 | 0.925055514 | 1.0941553 | down | mRNA |
| NM_001042496 | SLC12A6 | 0.730194214 | 0.966191059 | 1.0612706 | down | mRNA |
| NM_001042497 | SLC12A6 | 0.298036028 | 0.794295449 | 1.1430201 | down | mRNA |
| NM_001042518 | SERPINA9 | 0.614658433 | 0.944827645 | 1.1053959 | down | mRNA |
| NM_001042520 | C2orf88 | 0.914312376 | 0.995342392 | 1.0232944 | down | mRNA |
| NM_001042532 | COASY | 0.515477386 | 0.914319064 | 1.043375 | down | mRNA |
| NM_001042535 | AGAP3 | 0.029542646 | 0.334071792 | 1.2440762 | down | mRNA |
| NM_001042537 | SLC9A6 | 0.021668994 | 0.29025889 | 1.3662927 | down | mRNA |
| NM_001042539 | MAZ | 0.948833625 | 0.996710991 | 1.0072334 | down | mRNA |
| NM_001042545 | LTBP4 | 0.679746119 | 0.960007989 | 1.0757638 | down | mRNA |
| NM_001042546 | ATPAF1 | 0.616554902 | 0.945912508 | 1.0491103 | down | mRNA |
| NM_001042549 | NSL1 | 0.103770616 | 0.533622448 | 1.157466 | down | mRNA |
| NM_001042550 | SMC2 | 0.747827785 | 0.970686576 | 1.1116719 | down | mRNA |
| NM_001042552 | TATDN3 | 0.554419107 | 0.926549129 | 1.0646316 | down | mRNA |
| NM_001042553 | TATDN3 | 0.512443525 | 0.91317044 | 1.0621948 | down | mRNA |
| NM_001042559 | EIF4G2 | 0.062049102 | 0.439269616 | 1.1672912 | down | mRNA |
| NM_001042574 | CRTC3 | 0.066289811 | 0.449693785 | 1.2457521 | down | mRNA |
| NM_001042590 | TMEM8B | 0.841837846 | 0.987123192 | 1.0201601 | down | mRNA |
| NM_001042595 | TMEM91 | 0.314557687 | 0.809772968 | 1.1330287 | down | mRNA |
| NM_001042601 | TTC14 | 0.350835247 | 0.834973926 | 1.039018 | down | mRNA |
| NM_001042603 | KDM5A | 0.210579848 | 0.701214058 | 1.2135421 | down | mRNA |
| NM_001042610 | DBNDD1 | 0.586837553 | 0.93579682 | 1.0934104 | down | mRNA |
| NM_001042616 | PIGY | 0.445474124 | 0.885807324 | 1.1173212 | down | mRNA |
| NM_001042618 | PARP2 | 0.865307977 | 0.989401142 | 1.0407949 | down | mRNA |
| NM_001042633 | SNX21 | 0.89361695 | 0.993978977 | 1.017098 | down | mRNA |
| NM_001042635 | NGDN | 0.416767292 | 0.870736736 | 1.1127057 | down | mRNA |
| NM_001042646 | TRAK1 | 0.032052488 | 0.342454646 | 1.1936136 | down | mRNA |
| NM_001042683 | SHPRH | 0.008844446 | 0.202329498 | 1.440871 | down | mRNA |
| NM_001042697 | ZSWIM7 | 0.124041654 | 0.577766088 | 1.1036813 | down | mRNA |
| NM_001042706 | IQCJ | 0.600941406 | 0.941023551 | 1.1560153 | down | mRNA |
| NM_001042717 | GTPBP10 | 0.062256905 | 0.439379649 | 1.3811098 | down | mRNA |
| NM_001042734 | SEC24B | 0.092775592 | 0.511370653 | 1.0993309 | down | mRNA |
| NM_001042750 | STAG2 | 0.710112744 | 0.965462762 | 1.0212981 | down | mRNA |
| NM_001042751 | STAG2 | 0.113297807 | 0.556552552 | 1.077515 | down | mRNA |
| NM_001042758 | SRGAP2 | 0.000106554 | 0.037538791 | 1.0974695 | down | mRNA |
| NM_001042780 | TNNT3 | 0.749178816 | 0.970725371 | 1.1665791 | down | mRNA |
| NM_001042781 | TNNT3 | 0.557088149 | 0.926833616 | 1.2211823 | down | mRNA |
| NM_001042782 | TNNT3 | 0.204304839 | 0.694397489 | 1.4508414 | down | mRNA |
| NM_001043229 | METTL12 | 0.09892298 | 0.522959445 | 1.2540978 | down | mRNA |
| NM_001043351 | TPM3 | 0.150497327 | 0.615491439 | 1.1926296 | down | mRNA |
| NM_001043352 | TPM3 | 0.317021548 | 0.811824899 | 1.1342288 | down | mRNA |
| NM_001044388 | ZNF557 | 0.027067075 | 0.320017762 | 2.8994628 | down | mRNA |
| NM_001045556 | SLA | 0.508045023 | 0.911674633 | 1.178833 | down | mRNA |
| NM_001046 | SLC12A2 | 0.525602037 | 0.917693484 | 1.0786332 | down | mRNA |
| NM_001047 | SRD5A1 | 0.579124767 | 0.933821303 | 1.1253361 | down | mRNA |
| NM_001048183 | PHACTR4 | 0.092626847 | 0.511338764 | 1.2992164 | down | mRNA |
| NM_001048194 | RCC1 | 0.810005556 | 0.98118927 | 1.0249574 | down | mRNA |
| NM_001048200 | HIPK3 | 0.077592528 | 0.478499021 | 1.4470382 | down | mRNA |
| NM_001048209 | NTM | 0.286681238 | 0.782942227 | 1.1950813 | down | mRNA |
| NM_001048218 | SCYL1 | 0.149406491 | 0.613559438 | 1.1338737 | down | mRNA |
| NM_001048221 | DBNDD2 | 0.902751425 | 0.99508468 | 1.0697266 | down | mRNA |
| NM_001048249 | C5orf43 | 0.080259383 | 0.483876854 | 1.2244299 | down | mRNA |
| NM_001048265 | C9orf116 | 0.878510544 | 0.991720414 | 1.0352669 | down | mRNA |
| NM_001065 | TNFRSF1A | 0.096124409 | 0.51782059 | 1.3040893 | down | mRNA |
| NM_001067 | TOP2A | 0.72147694 | 0.966191059 | 1.267428 | down | mRNA |
| NM_001070 | TUBG1 | 0.967310141 | 0.99793927 | 1.0069183 | down | mRNA |
| NM_001071 | TYMS | 0.957042522 | 0.996883425 | 1.0253342 | down | mRNA |
| NM_001071775 | MZT1 | 0.365277208 | 0.844953778 | 1.1502385 | down | mRNA |
| NM_001075098 | MOCS1 | 0.563684105 | 0.929146073 | 1.0382674 | down | mRNA |
| NM_001076552 | ACSS2 | 0.708253647 | 0.965462762 | 1.0651616 | down | mRNA |
| NM_001076674 | TMUB2 | 0.167986308 | 0.642921845 | 1.2805074 | down | mRNA |
| NM_001076675 | ZNF626 | 0.006709337 | 0.183552613 | 1.2119864 | down | mRNA |
| NM_001076677 | CLTA | 0.018594594 | 0.272971322 | 1.0978914 | down | mRNA |
| NM_001076678 | ZNF493 | 0.040874094 | 0.375308608 | 1.3176694 | down | mRNA |
| NM_001076680 | C17orf108 | 0.604194105 | 0.94174007 | 1.0532737 | down | mRNA |
| NM_001076683 | UBTF | 0.09190805 | 0.511125402 | 1.3221111 | down | mRNA |
| NM_001076786 | QSER1 | 0.030110399 | 0.336931558 | 1.2109868 | down | mRNA |
| NM_001077180 | METTL9 | 0.349708365 | 0.834138149 | 1.0714682 | down | mRNA |
| NM_001077199 | SREK1 | 0.008365421 | 0.198394583 | 1.1420412 | down | mRNA |
| NM_001077203 | SENP7 | 0.110435676 | 0.549968727 | 1.2418258 | down | mRNA |
| NM_001077206 | SEC31A | 0.710367734 | 0.965462762 | 1.0567029 | down | mRNA |
| NM_001077207 | SEC31A | 0.706468292 | 0.965401045 | 1.0600632 | down | mRNA |
| NM_001077208 | SEC31A | 0.755529705 | 0.971497464 | 1.0493566 | down | mRNA |
| NM_001077238 | SPPL2B | 0.561970551 | 0.928808234 | 1.1470707 | down | mRNA |
| NM_001077262 | UBXN11 | 0.380831459 | 0.853514109 | 1.0953399 | down | mRNA |
| NM_001077350 | NPRL3 | 0.925795923 | 0.995677477 | 1.0163087 | down | mRNA |
| NM_001077351 | RBM23 | 0.510025241 | 0.911891001 | 1.0658017 | down | mRNA |
| NM_001077352 | RBM23 | 0.412507105 | 0.869219033 | 1.1181734 | down | mRNA |
| NM_001077366 | POMT1 | 0.010898118 | 0.213674567 | 1.419143 | down | mRNA |
| NM_001077394 | DPH5 | 0.995533944 | 0.999872237 | 1.0006367 | down | mRNA |
| NM_001077416 | TMEM231 | 0.933386557 | 0.996710991 | 1.0052755 | down | mRNA |
| NM_001077427 | LYPD1 | 0.620944742 | 0.947926299 | 1.2211832 | down | mRNA |
| NM_001077441 | BCLAF1 | 0.413243485 | 0.869509111 | 1.0359679 | down | mRNA |
| NM_001077488 | GNAS | 0.212365785 | 0.704145654 | 1.1488704 | down | mRNA |
| NM_001077489 | GNAS | 0.076379433 | 0.475343325 | 1.2493944 | down | mRNA |
| NM_001077497 | PRR3 | 0.598849324 | 0.940648177 | 1.1370409 | down | mRNA |
| NM_001077525 | MTMR14 | 0.4150623 | 0.870239791 | 1.0769886 | down | mRNA |
| NM_001077526 | MTMR14 | 0.258655272 | 0.759358563 | 1.1024097 | down | mRNA |
| NM_001077527 | JRK | 0.001855897 | 0.105670067 | 1.6781475 | down | mRNA |
| NM_001077686 | AGAP8 | 0.029100178 | 0.331684822 | 1.1669681 | down | mRNA |
| NM_001077691 | ALG9 | 0.309285784 | 0.803961878 | 1.0410182 | down | mRNA |
| NM_001077692 | ALG9 | 0.103136896 | 0.532943442 | 1.1739157 | down | mRNA |
| NM_001077700 | MIER1 | 0.058809809 | 0.431078857 | 1.1518654 | down | mRNA |
| NM_001077701 | MIER1 | 0.367800909 | 0.845181673 | 1.0864874 | down | mRNA |
| NM_001077702 | MIER1 | 0.382951851 | 0.853514109 | 1.2200873 | down | mRNA |
| NM_001077703 | MIER1 | 0.737578103 | 0.969012535 | 1.0740839 | down | mRNA |
| NM_001077710 | FAM110C | 0.69666855 | 0.962410025 | 1.0583011 | down | mRNA |
| NM_001078171 | FAM127A | 0.020115714 | 0.284065167 | 1.2612157 | down | mRNA |
| NM_001078173 | FAM127C | 0.358324707 | 0.840546205 | 1.0998115 | down | mRNA |
| NM_001078650 | TMEM134 | 0.623528362 | 0.948341211 | 1.0388183 | down | mRNA |
| NM_001079 | ZAP70 | 0.812719285 | 0.98118927 | 1.0187846 | down | mRNA |
| NM_001079515 | TBCE | 0.963829277 | 0.99793927 | 1.0069857 | down | mRNA |
| NM_001079518 | MED24 | 0.534564847 | 0.920848299 | 1.1542326 | down | mRNA |
| NM_001079520 | DACT1 | 0.000721656 | 0.078885568 | 2.0319922 | down | mRNA |
| NM_001079521 | KTN1 | 0.546879073 | 0.924803608 | 1.0411614 | down | mRNA |
| NM_001079522 | KTN1 | 0.261656339 | 0.762395628 | 1.0299234 | down | mRNA |
| NM_001079535 | CPEB1 | 0.609273346 | 0.943053241 | 1.1192988 | down | mRNA |
| NM_001079537 | TRAPPC6B | 0.39074966 | 0.858920947 | 1.0543427 | down | mRNA |
| NM_001079559 | HNRNPUL2 | 0.232203288 | 0.730136725 | 1.2466422 | down | mRNA |
| NM_001079668 | NKX2-1 | 0.332409295 | 0.822707947 | 1.1662957 | down | mRNA |
| NM_001079669 | TMTC4 | 0.270840999 | 0.770820157 | 1.2208364 | down | mRNA |
| NM_001079673 | FNDC3A | 0.247663743 | 0.745868458 | 1.1020806 | down | mRNA |
| NM_001079675 | ETV4 | 0.536881542 | 0.922650285 | 1.0881723 | down | mRNA |
| NM_001079685 | KIAA1191 | 0.862331658 | 0.989401142 | 1.0204083 | down | mRNA |
| NM_001079807 | PGA3 | 0.382915221 | 0.853514109 | 1.1578944 | down | mRNA |
| NM_001079810 | XIRP2 | 0.77976575 | 0.976938981 | 1.0490834 | down | mRNA |
| NM_001079839 | OCIAD1 | 0.393399405 | 0.860064163 | 1.1058221 | down | mRNA |
| NM_001079841 | OCIAD1 | 0.629071343 | 0.950172985 | 1.0302484 | down | mRNA |
| NM_001079843 | CASZ1 | 0.32378048 | 0.816222625 | 1.0832835 | down | mRNA |
| NM_001079855 | GYG2 | 0.199876526 | 0.68900633 | 1.1316188 | down | mRNA |
| NM_001079877 | RASA4 | 0.784138775 | 0.977347603 | 1.0451051 | down | mRNA |
| NM_001079878 | CNGA3 | 0.413418001 | 0.86961884 | 1.5783823 | down | mRNA |
| NM_001079907 | ZNF331 | 3.7442E-06 | 0.009515876 | 1.7973432 | down | mRNA |
| NM_001079935 | OR7E24 | 0.559885434 | 0.927606799 | 1.189708 | down | mRNA |
| NM_001080114 | LDB3 | 0.902461326 | 0.99508468 | 1.0345288 | down | mRNA |
| NM_001080391 | SP100 | 0.610241405 | 0.943169861 | 1.0852666 | down | mRNA |
| NM_001080393 | GXYLT2 | 0.554834004 | 0.926833616 | 1.1695654 | down | mRNA |
| NM_001080394 | KIAA0146 | 0.363397637 | 0.843238468 | 1.0527007 | down | mRNA |
| NM_001080401 | PPM1N | 0.676927006 | 0.959531882 | 1.1359989 | down | mRNA |
| NM_001080409 | ZNF99 | 0.677893887 | 0.959531882 | 1.1021377 | down | mRNA |
| NM_001080410 | FBXO41 | 0.862015724 | 0.989401142 | 1.0190972 | down | mRNA |
| NM_001080411 | ZNF433 | 0.003468007 | 0.138138947 | 1.2362047 | down | mRNA |
| NM_001080416 | MYBL1 | 0.852593967 | 0.987678166 | 1.0979519 | down | mRNA |
| NM_001080419 | UNK | 0.442592686 | 0.884228603 | 1.075847 | down | mRNA |
| NM_001080423 | GRIP2 | 0.765290102 | 0.974380259 | 1.0455739 | down | mRNA |
| NM_001080425 | BEX4 | 0.891820009 | 0.9937521 | 1.0771653 | down | mRNA |
| NM_001080432 | FTO | 0.68387406 | 0.960111041 | 1.0588184 | down | mRNA |
| NM_001080435 | WHAMM | 0.034141662 | 0.349933975 | 1.1982866 | down | mRNA |
| NM_001080436 | WTIP | 0.211533301 | 0.702875483 | 1.2629086 | down | mRNA |
| NM_001080437 | SNED1 | 0.044853509 | 0.38849334 | 1.1179539 | down | mRNA |
| NM_001080446 | C11orf94 | 0.798293256 | 0.980749101 | 1.035347 | down | mRNA |
| NM_001080449 | DNA2 | 0.940258506 | 0.996710991 | 1.0075613 | down | mRNA |
| NM_001080452 | GPR108 | 0.641321924 | 0.954285521 | 1.095455 | down | mRNA |
| NM_001080463 | DYNC2H1 | 0.8930024 | 0.993978977 | 1.0349271 | down | mRNA |
| NM_001080465 | C17orf98 | 0.143046739 | 0.603147721 | 1.1407363 | down | mRNA |
| NM_001080466 | BTBD17 | 0.853257703 | 0.987678166 | 1.0233462 | down | mRNA |
| NM_001080475 | PLEKHM3 | 0.156656718 | 0.625273733 | 1.1051169 | down | mRNA |
| NM_001080477 | ODZ3 | 0.759119989 | 0.972369912 | 1.0517778 | down | mRNA |
| NM_001080479 | ARHGEF28 | 0.112071974 | 0.554156842 | 1.1240203 | down | mRNA |
| NM_001080481 | USP45 | 0.753576332 | 0.970725371 | 1.0405882 | down | mRNA |
| NM_001080485 | ZNF275 | 0.182143659 | 0.665351217 | 1.0753256 | down | mRNA |
| NM_001080493 | ZNF823 | 0.114207034 | 0.558096391 | 1.3101302 | down | mRNA |
| NM_001080494 | TTC39A | 0.449502193 | 0.888279993 | 1.1423213 | down | mRNA |
| NM_001080495 | TNRC18 | 0.135553133 | 0.593260206 | 1.4655465 | down | mRNA |
| NM_001080497 | MEGF9 | 0.611495779 | 0.943894921 | 1.0733772 | down | mRNA |
| NM_001080508 | TBX18 | 0.009045717 | 0.202329498 | 1.500963 | down | mRNA |
| NM_001080519 | BAHCC1 | 0.907876979 | 0.995145546 | 1.0109449 | down | mRNA |
| NM_001080521 | RASSF10 | 0.978866703 | 0.998661138 | 1.0074531 | down | mRNA |
| NM_001080522 | CC2D2A | 0.246218842 | 0.744847716 | 1.1423663 | down | mRNA |
| NM_001080524 | C16orf90 | 0.273769344 | 0.774061785 | 1.1465012 | down | mRNA |
| NM_001080528 | LOC646498 | 0.247742311 | 0.745868458 | 1.17168 | down | mRNA |
| NM_001080533 | UNC119B | 0.903232618 | 0.99508468 | 1.0134769 | down | mRNA |
| NM_001080541 | MGA | 0.236079638 | 0.734389717 | 1.3491437 | down | mRNA |
| NM_001080547 | SPI1 | 0.171558961 | 0.649286717 | 1.3160957 | down | mRNA |
| NM_001080790 | MAGEA9B | 0.240907085 | 0.739116169 | 1.2910499 | down | mRNA |
| NM_001080848 | CSAG2 | 0.533818684 | 0.92058057 | 1.098474 | down | mRNA |
| NM_001080950 | MYO1C | 0.768481592 | 0.975142015 | 1.0738345 | down | mRNA |
| NM_001080953 | PLAGL1 | 0.81714497 | 0.981692737 | 1.0228045 | down | mRNA |
| NM_001081550 | THOC2 | 0.322410022 | 0.815372756 | 1.0549627 | down | mRNA |
| NM_001081559 | CPSF4 | 0.035021819 | 0.352396712 | 1.3746034 | down | mRNA |
| NM_001081562 | DMPK | 0.003863994 | 0.143624712 | 1.4009368 | down | mRNA |
| NM_001081640 | PRKDC | 0.11211212 | 0.554209487 | 1.1322051 | down | mRNA |
| NM_001081755 | ELN | 0.925460283 | 0.995465766 | 1.0694356 | down | mRNA |
| NM_001081976 | ADORA3 | 0.185705869 | 0.669321303 | 1.3967134 | down | mRNA |
| NM_001082486 | ACD | 0.484821625 | 0.903250293 | 1.1122984 | down | mRNA |
| NM_001082576 | RBFOX2 | 0.018001908 | 0.269173581 | 1.1438969 | down | mRNA |
| NM_001082577 | RBFOX2 | 0.020679885 | 0.286034385 | 1.1579732 | down | mRNA |
| NM_001082967 | FAM19A5 | 0.745147388 | 0.970378122 | 1.0213513 | down | mRNA |
| NM_001082968 | TOM1L2 | 0.730473455 | 0.966329458 | 1.068507 | down | mRNA |
| NM_001082969 | C11orf57 | 0.020344475 | 0.285032405 | 1.1188686 | down | mRNA |
| NM_001082970 | C11orf57 | 0.010211561 | 0.20936306 | 1.2334217 | down | mRNA |
| NM_001082971 | DDC | 0.10775677 | 0.544052509 | 1.2912685 | down | mRNA |
| NM_001083112 | GPD2 | 0.284117599 | 0.78179061 | 1.2633882 | down | mRNA |
| NM_001083537 | FAM86B1 | 0.676375076 | 0.959531882 | 1.0255314 | down | mRNA |
| NM_001083585 | RABEP1 | 0.269937975 | 0.770082629 | 1.0999511 | down | mRNA |
| NM_001083588 | E2F5 | 0.125785702 | 0.578441772 | 1.2065937 | down | mRNA |
| NM_001083589 | E2F5 | 0.014489055 | 0.242674797 | 1.2118559 | down | mRNA |
| NM_001083590 | TMEM214 | 0.291127702 | 0.787547689 | 1.0917113 | down | mRNA |
| NM_001083591 | CELF2 | 0.543789641 | 0.923577434 | 1.3218924 | down | mRNA |
| NM_001083592 | ROR1 | 0.21283712 | 0.704903783 | 1.1988396 | down | mRNA |
| NM_001083607 | PTCH1 | 0.046565561 | 0.392866345 | 1.247464 | down | mRNA |
| NM_001083608 | NARF | 0.347251033 | 0.833588015 | 1.1014133 | down | mRNA |
| NM_001083612 | AGPHD1 | 0.381388634 | 0.853514109 | 1.2725278 | down | mRNA |
| NM_001083614 | EARS2 | 0.021006158 | 0.287801352 | 1.2778318 | down | mRNA |
| NM_001083617 | RB1CC1 | 0.723265211 | 0.966191059 | 1.0145003 | down | mRNA |
| NM_001083625 | ANKRD12 | 0.394170088 | 0.861008404 | 1.1251303 | down | mRNA |
| NM_001083893 | STRN3 | 0.698803801 | 0.962701213 | 1.0338389 | down | mRNA |
| NM_001083914 | CTBP2 | 0.002129061 | 0.110028846 | 1.3756821 | down | mRNA |
| NM_001083946 | C2orf56 | 0.073039886 | 0.464657997 | 1.2364077 | down | mRNA |
| NM_001084 | PLOD3 | 0.419492064 | 0.872365335 | 1.1221325 | down | mRNA |
| NM_001085354 | MORC4 | 0.043175244 | 0.383508423 | 1.1213329 | down | mRNA |
| NM_001085365 | MZT2A | 0.913598576 | 0.995342392 | 1.011974 | down | mRNA |
| NM_001085400 | RELL1 | 0.918964915 | 0.995349793 | 1.0137833 | down | mRNA |
| NM_001085411 | NADKD1 | 0.104003948 | 0.533896783 | 1.1284746 | down | mRNA |
| NM_001085423 | MILR1 | 0.867081141 | 0.989481868 | 1.0621607 | down | mRNA |
| NM_001085428 | ARSA | 0.465902394 | 0.895596812 | 1.1784011 | down | mRNA |
| NM_001085429 | TMEM213 | 0.611879079 | 0.943964604 | 1.0950681 | down | mRNA |
| NM_001085430 | C17orf67 | 0.497968066 | 0.909116151 | 1.081003 | down | mRNA |
| NM_001085451 | LNP1 | 0.724714993 | 0.966191059 | 1.0347721 | down | mRNA |
| NM_001085454 | GIT1 | 0.090337117 | 0.507665631 | 1.3501119 | down | mRNA |
| NM_001085458 | CTNND1 | 0.785273185 | 0.977411899 | 1.0224147 | down | mRNA |
| NM_001085461 | CTNND1 | 0.078905298 | 0.481483348 | 1.2601463 | down | mRNA |
| NM_001085463 | CTNND1 | 0.309435179 | 0.803961878 | 1.0974681 | down | mRNA |
| NM_001085465 | CTNND1 | 0.501812104 | 0.910194076 | 1.0848989 | down | mRNA |
| NM_001085466 | CTNND1 | 0.505020944 | 0.910568383 | 1.046832 | down | mRNA |
| NM_001085469 | CTNND1 | 0.666466878 | 0.958354006 | 1.0375891 | down | mRNA |
| NM_001085487 | MYSM1 | 0.09862512 | 0.52242281 | 1.2609731 | down | mRNA |
| NM_001086521 | C17orf89 | 0.817650895 | 0.981727075 | 1.0197706 | down | mRNA |
| NM_001087 | AAMP | 0.758562538 | 0.972072638 | 1.0497369 | down | mRNA |
| NM_001089591 | UQCRHL | 0.384559883 | 0.85485898 | 1.0561595 | down | mRNA |
| NM_001091 | ABP1 | 0.392547351 | 0.859680391 | 1.1750142 | down | mRNA |
| NM_001093728 | TCP11 | 0.769332777 | 0.975286259 | 1.0449353 | down | mRNA |
| NM_001093755 | C5orf44 | 0.082403357 | 0.489943729 | 1.2541155 | down | mRNA |
| NM_001093756 | C5orf44 | 0.166900537 | 0.641842579 | 1.1075355 | down | mRNA |
| NM_001093770 | SFTPA1 | 0.511417327 | 0.912481293 | 1.3369735 | down | mRNA |
| NM_001093771 | TXNRD1 | 0.87147617 | 0.989880083 | 1.0423214 | down | mRNA |
| NM_001093772 | KIT | 0.477373967 | 0.900451573 | 1.0223515 | down | mRNA |
| NM_001095 | ASIC1 | 0.390933231 | 0.858920947 | 1.3411334 | down | mRNA |
| NM_001096 | ACLY | 0.924958549 | 0.995456159 | 1.0054588 | down | mRNA |
| NM_001097599 | SLC35G2 | 0.10459382 | 0.535533 | 1.1571517 | down | mRNA |
| NM_001097613 | GPR89A | 0.014406999 | 0.242285449 | 1.2106581 | down | mRNA |
| NM_001097616 | GPR89C | 0.006060155 | 0.177287877 | 1.2557528 | down | mRNA |
| NM_001097641 | FUT3 | 0.724341168 | 0.966191059 | 1.0514072 | down | mRNA |
| NM_001097643 | TAS2R30 | 0.070540337 | 0.459099275 | 1.6198485 | down | mRNA |
| NM_001098169 | BSX | 0.725732415 | 0.966191059 | 1.1534855 | down | mRNA |
| NM_001098199 | GPR1 | 0.356980335 | 0.839477697 | 1.1002148 | down | mRNA |
| NM_001098202 | HIC1 | 0.424445214 | 0.874854499 | 1.3265941 | down | mRNA |
| NM_001098205 | HNRNPF | 0.13292573 | 0.588930122 | 1.178586 | down | mRNA |
| NM_001098268 | LIG4 | 0.591984681 | 0.9374743 | 1.0658169 | down | mRNA |
| NM_001098376 | PRAMEF15 | 0.364240283 | 0.84362654 | 1.1273862 | down | mRNA |
| NM_001098416 | HDAC7 | 0.097867161 | 0.520535504 | 1.3546513 | down | mRNA |
| NM_001098477 | GRSF1 | 0.021526041 | 0.289533061 | 1.2606055 | down | mRNA |
| NM_001098478 | HLA-F | 0.339884925 | 0.828118322 | 1.0952158 | down | mRNA |
| NM_001098483 | FUOM | 0.310730542 | 0.805427509 | 1.0681419 | down | mRNA |
| NM_001098493 | ZNF419 | 0.020641042 | 0.286034385 | 1.3375413 | down | mRNA |
| NM_001098494 | ZNF419 | 0.766315999 | 0.974737263 | 1.020001 | down | mRNA |
| NM_001098496 | ZNF419 | 0.006843655 | 0.184054476 | 1.6020451 | down | mRNA |
| NM_001098502 | CHCHD4 | 0.0073813 | 0.190938402 | 1.1861572 | down | mRNA |
| NM_001098509 | SGSM2 | 0.9248247 | 0.995456159 | 1.0137533 | down | mRNA |
| NM_001098510 | PELI3 | 0.840633105 | 0.987068842 | 1.0182376 | down | mRNA |
| NM_001098515 | MRGPRF | 0.088287985 | 0.502399895 | 1.2172888 | down | mRNA |
| NM_001098516 | MUC20 | 0.820700448 | 0.982756843 | 1.0153299 | down | mRNA |
| NM_001098517 | CADM1 | 0.651624308 | 0.956204521 | 1.1022384 | down | mRNA |
| NM_001098521 | HTATIP2 | 0.071039907 | 0.460875363 | 1.1823269 | down | mRNA |
| NM_001098523 | HTATIP2 | 0.661752788 | 0.958354006 | 1.0518008 | down | mRNA |
| NM_001098525 | CKAP2 | 0.453696168 | 0.890025645 | 1.3628214 | down | mRNA |
| NM_001098526 | AMICA1 | 0.477336205 | 0.900451573 | 1.2737963 | down | mRNA |
| NM_001098536 | USP5 | 0.44174345 | 0.884182697 | 1.0940726 | down | mRNA |
| NM_001098577 | RPL31 | 0.885992905 | 0.992981836 | 1.0809289 | down | mRNA |
| NM_001098612 | SIGLEC14 | 0.524113894 | 0.917428132 | 1.1180631 | down | mRNA |
| NM_001098614 | PUS7L | 0.087940356 | 0.501543709 | 1.3489178 | down | mRNA |
| NM_001098616 | C1orf43 | 0.290497805 | 0.786596721 | 1.091161 | down | mRNA |
| NM_001098621 | TMEM251 | 0.713858143 | 0.966110889 | 1.0407175 | down | mRNA |
| NM_001098625 | JKAMP | 0.197148873 | 0.685161614 | 1.0725153 | down | mRNA |
| NM_001098629 | IRF5 | 0.256500929 | 0.757240077 | 1.0480885 | down | mRNA |
| NM_001098637 | PWWP2B | 0.077459854 | 0.478473478 | 1.3308179 | down | mRNA |
| NM_001098668 | SFTPA2 | 0.533865233 | 0.92058057 | 1.0505904 | down | mRNA |
| NM_001098673 | C12orf44 | 0.051332827 | 0.410259053 | 1.3509348 | down | mRNA |
| NM_001098728 | GTF2H2C | 0.227954643 | 0.723854154 | 1.3670489 | down | mRNA |
| NM_001098784 | FAM89B | 0.07785124 | 0.479367476 | 1.0865615 | down | mRNA |
| NM_001098785 | FAM89B | 0.101360267 | 0.529124784 | 1.1168028 | down | mRNA |
| NM_001098786 | HILPDA | 0.13926201 | 0.598648178 | 1.2489315 | down | mRNA |
| NM_001098787 | BET1L | 0.786958456 | 0.977479172 | 1.0536893 | down | mRNA |
| NM_001098794 | FAM160A2 | 0.200749794 | 0.68900633 | 1.1243935 | down | mRNA |
| NM_001098796 | TOX2 | 0.432332574 | 0.880427272 | 1.2610265 | down | mRNA |
| NM_001098798 | TOX2 | 0.908870172 | 0.995333785 | 1.0178876 | down | mRNA |
| NM_001098801 | FAM210A | 0.025992699 | 0.315604326 | 1.3193885 | down | mRNA |
| NM_001098802 | CEP78 | 0.052504454 | 0.412648073 | 1.1139151 | down | mRNA |
| NM_001098812 | SEPT8 | 0.629751599 | 0.950410442 | 1.0361803 | down | mRNA |
| NM_001098823 | TMEM91 | 0.35457868 | 0.83766056 | 1.066484 | down | mRNA |
| NM_001098824 | TMEM91 | 0.116630934 | 0.562996237 | 1.1695498 | down | mRNA |
| NM_001098825 | TMEM91 | 0.175477754 | 0.653962789 | 1.0688819 | down | mRNA |
| NM_001098832 | FAM104A | 0.012041141 | 0.222675671 | 1.1642471 | down | mRNA |
| NM_001098833 | ATXN7L3 | 0.680827451 | 0.960111041 | 1.0251533 | down | mRNA |
| NM_001098835 | MS4A15 | 0.324952322 | 0.817191271 | 1.145425 | down | mRNA |
| NM_001099218 | RAD51AP2 | 0.753282509 | 0.970725371 | 1.102862 | down | mRNA |
| NM_001099224 | IFT74 | 0.839788018 | 0.987068842 | 1.0256958 | down | mRNA |
| NM_001099269 | ZNF506 | 0.071031084 | 0.460875363 | 1.1823518 | down | mRNA |
| NM_001099271 | POC5 | 0.034640967 | 0.351984074 | 2.6488292 | down | mRNA |
| NM_001099272 | BTBD9 | 0.102357101 | 0.53117013 | 1.2685194 | down | mRNA |
| NM_001099284 | ZNF239 | 0.166132203 | 0.640243452 | 1.5686114 | down | mRNA |
| NM_001099285 | PTMA | 0.755073135 | 0.971417904 | 1.0258504 | down | mRNA |
| NM_001099287 | NIPAL4 | 0.709383724 | 0.965462762 | 1.0699549 | down | mRNA |
| NM_001099289 | SH3RF3 | 0.302673268 | 0.79797107 | 1.1258599 | down | mRNA |
| NM_001099294 | KIAA1644 | 0.88742904 | 0.993247479 | 1.054379 | down | mRNA |
| NM_001099339 | COMMD7 | 0.779996843 | 0.976938981 | 1.0628194 | down | mRNA |
| NM_001099401 | SGCE | 0.977398656 | 0.998501532 | 1.0050486 | down | mRNA |
| NM_001099409 | EHBP1L1 | 0.090947905 | 0.509268194 | 1.0806394 | down | mRNA |
| NM_001099411 | GPRASP1 | 0.468910173 | 0.897644444 | 1.2585509 | down | mRNA |
| NM_001099415 | POM121C | 0.809554563 | 0.98118927 | 1.0301425 | down | mRNA |
| NM_001099432 | BCAS3 | 0.711028721 | 0.96568622 | 1.054019 | down | mRNA |
| NM_001099435 | SPDYE5 | 0.350151158 | 0.834344847 | 1.0713951 | down | mRNA |
| NM_001099455 | CPPED1 | 0.897117797 | 0.994222243 | 1.0211315 | down | mRNA |
| NM_001099627 | FAM54B | 0.008758348 | 0.202329498 | 1.2721746 | down | mRNA |
| NM_001099640 | TMEM100 | 0.337321083 | 0.827111947 | 1.7775227 | down | mRNA |
| NM_001099642 | FTSJD1 | 0.000707564 | 0.078885568 | 1.8994253 | down | mRNA |
| NM_001099650 | GXYLT1 | 0.065014871 | 0.445228144 | 1.2162575 | down | mRNA |
| NM_001099653 | FAM86C1 | 0.901118479 | 0.994807691 | 1.0178325 | down | mRNA |
| NM_001099661 | EIF3CL | 0.071495195 | 0.461977497 | 1.1096761 | down | mRNA |
| NM_001099666 | PTAR1 | 0.073916767 | 0.468039773 | 1.1020198 | down | mRNA |
| NM_001099668 | HIGD1A | 0.438034162 | 0.882682266 | 1.372396 | down | mRNA |
| NM_001099669 | HIGD1A | 0.794378871 | 0.979333052 | 1.0178341 | down | mRNA |
| NM_001099672 | C8orf59 | 0.703199182 | 0.963625105 | 1.0168907 | down | mRNA |
| NM_001099678 | LRRC58 | 0.033945806 | 0.349511805 | 1.209438 | down | mRNA |
| NM_001099679 | TRIM32 | 0.018074583 | 0.269173581 | 1.7346943 | down | mRNA |
| NM_001099681 | MAGIX | 0.419982032 | 0.872365335 | 1.2844486 | down | mRNA |
| NM_001099684 | FAM156B | 0.01126165 | 0.21765386 | 1.5648048 | down | mRNA |
| NM_001099685 | RHOXF2B | 0.368526198 | 0.845559574 | 1.2162196 | down | mRNA |
| NM_001099691 | TGFA | 0.836664745 | 0.986725216 | 1.0179648 | down | mRNA |
| NM_001099754 | SYBU | 0.300676034 | 0.795797471 | 1.1885623 | down | mRNA |
| NM_001099781 | GGT5 | 0.424763403 | 0.874854499 | 1.3141233 | down | mRNA |
| NM_001099782 | GGT5 | 0.063545184 | 0.440919074 | 1.4652123 | down | mRNA |
| NM_001100 | ACTA1 | 0.792456772 | 0.978996327 | 1.0645391 | down | mRNA |
| NM_001100167 | MRVI1 | 0.973173883 | 0.998501532 | 1.0067131 | down | mRNA |
| NM_001100168 | CCR5 | 0.464080197 | 0.894989844 | 2.0394703 | down | mRNA |
| NM_001100170 | IMMT | 0.002637539 | 0.122061602 | 1.2083794 | down | mRNA |
| NM_001100389 | TMEM192 | 0.584974027 | 0.934708084 | 1.2069004 | down | mRNA |
| NM_001100398 | RAP1GAP2 | 0.095192607 | 0.516597924 | 1.1738534 | down | mRNA |
| NM_001100408 | C17orf62 | 0.888008703 | 0.993440915 | 1.0142561 | down | mRNA |
| NM_001100409 | SENP6 | 0.031472437 | 0.341322176 | 1.2237286 | down | mRNA |
| NM_001100412 | DROSHA | 0.411965602 | 0.869001481 | 1.1869291 | down | mRNA |
| NM_001100418 | C19orf60 | 0.939054459 | 0.996710991 | 1.002907 | down | mRNA |
| NM_001100419 | C19orf60 | 0.188987375 | 0.6744312 | 1.1382598 | down | mRNA |
| NM_001100424 | SPATS2L | 0.174472219 | 0.652930084 | 1.0963427 | down | mRNA |
| NM_001100426 | RAP1GDS1 | 0.188658572 | 0.674131123 | 1.1460309 | down | mRNA |
| NM_001100427 | RAP1GDS1 | 0.060893772 | 0.436633496 | 1.246059 | down | mRNA |
| NM_001100428 | RAP1GDS1 | 0.086933444 | 0.500185639 | 1.2008733 | down | mRNA |
| NM_001100429 | RAP1GDS1 | 0.137601327 | 0.596789644 | 1.1630744 | down | mRNA |
| NM_001100430 | RAP1GDS1 | 0.137057893 | 0.595949762 | 1.1876294 | down | mRNA |
| NM_001100590 | KIAA0232 | 0.141065297 | 0.601413215 | 1.1823298 | down | mRNA |
| NM_001100592 | ATP6V0E2 | 0.398604374 | 0.863643939 | 1.2376468 | down | mRNA |
| NM_001100598 | ZNF707 | 0.039666695 | 0.370777582 | 1.1495303 | down | mRNA |
| NM_001100603 | KDELR2 | 0.357878874 | 0.840133155 | 1.101873 | down | mRNA |
| NM_001100621 | C17orf80 | 0.00131267 | 0.093126586 | 2.0196617 | down | mRNA |
| NM_001100622 | C17orf80 | 0.200583074 | 0.68900633 | 1.6432859 | down | mRNA |
| NM_001100623 | PLEKHB2 | 0.0976634 | 0.520535504 | 1.1594329 | down | mRNA |
| NM_001100624 | CENPN | 0.473393716 | 0.899184677 | 1.1534022 | down | mRNA |
| NM_001100816 | MED7 | 0.058230581 | 0.429558128 | 1.3355775 | down | mRNA |
| NM_001100819 | MOB4 | 0.065219392 | 0.44602781 | 1.212373 | down | mRNA |
| NM_001100873 | C16orf46 | 0.93018313 | 0.996649933 | 1.0118607 | down | mRNA |
| NM_001100874 | SLC9B1 | 0.502612161 | 0.910335149 | 1.0746083 | down | mRNA |
| NM_001100912 | BEND7 | 0.507463221 | 0.911674633 | 1.1702311 | down | mRNA |
| NM_001100913 | PACS2 | 0.819760683 | 0.982397582 | 1.0584745 | down | mRNA |
| NM_001101314 | TMEM176B | 0.002393092 | 0.117191396 | 1.3092019 | down | mRNA |
| NM_001101338 | ZNF883 | 0.753068502 | 0.970725371 | 1.0722748 | down | mRNA |
| NM_001101389 | CLDN25 | 0.964512107 | 0.99793927 | 1.0173458 | down | mRNA |
| NM_001101417 | ISPD | 0.611763194 | 0.943913476 | 1.0352005 | down | mRNA |
| NM_001101648 | NPC1L1 | 0.577965061 | 0.933821303 | 1.0491733 | down | mRNA |
| NM_001101654 | CXXC1 | 0.137667865 | 0.596789644 | 1.2364103 | down | mRNA |
| NM_001101662 | NRD1 | 0.112009564 | 0.554156842 | 1.3040379 | down | mRNA |
| NM_001101667 | ACOX3 | 0.065518996 | 0.447226857 | 1.2508724 | down | mRNA |
| NM_001101676 | SAMD12 | 0.982570127 | 0.998971807 | 1.0061849 | down | mRNA |
| NM_001101677 | SOHLH1 | 0.737853117 | 0.969060757 | 1.0720604 | down | mRNA |
| NM_001101800 | FAM13B | 0.192729469 | 0.679364694 | 1.2512815 | down | mRNA |
| NM_001101802 | PHF21A | 0.048270368 | 0.398851601 | 1.2274687 | down | mRNA |
| NM_001102369 | C14orf159 | 0.369856418 | 0.846215286 | 1.1180484 | down | mRNA |
| NM_001102396 | SIKE1 | 0.665246873 | 0.958354006 | 1.0376096 | down | mRNA |
| NM_001102398 | HNRNPR | 0.304568558 | 0.800038203 | 1.0561377 | down | mRNA |
| NM_001102401 | TTI2 | 0.008029851 | 0.195524479 | 1.2641783 | down | mRNA |
| NM_001102402 | PCTP | 0.002583001 | 0.120730042 | 1.7359754 | down | mRNA |
| NM_001102454 | ZNF219 | 0.395518369 | 0.861590143 | 1.361013 | down | mRNA |
| NM_001102559 | PPAPDC1B | 0.302634955 | 0.79797107 | 1.0274505 | down | mRNA |
| NM_001102575 | SNX18 | 0.101262259 | 0.528999035 | 1.3686569 | down | mRNA |
| NM_001102592 | HENMT1 | 0.590462982 | 0.936429414 | 1.1567459 | down | mRNA |
| NM_001102599 | CEACAM20 | 0.63362311 | 0.951174475 | 1.0815441 | down | mRNA |
| NM_001102605 | IMPDH1 | 0.649725764 | 0.95550188 | 1.1464447 | down | mRNA |
| NM_001102610 | TUBGCP5 | 0.012564576 | 0.228499968 | 1.2551159 | down | mRNA |
| NM_001102664 | EPN2 | 0.156317451 | 0.62465535 | 1.1576371 | down | mRNA |
| NM_001102668 | PSMA4 | 0.970254201 | 0.998245244 | 1.0033326 | down | mRNA |
| NM_001102669 | RRAS2 | 0.662759392 | 0.958354006 | 1.0605583 | down | mRNA |
| NM_001103148 | GIGYF2 | 0.324535236 | 0.816842093 | 1.1172888 | down | mRNA |
| NM_001103161 | SH2D5 | 0.783608159 | 0.977275912 | 1.0346218 | down | mRNA |
| NM_001103167 | ZGLP1 | 0.56388972 | 0.92931878 | 1.0559785 | down | mRNA |
| NM_001104586 | ALS2CR8 | 0.496300527 | 0.908342993 | 1.1638588 | down | mRNA |
| NM_001104631 | PDE4D | 0.689550721 | 0.96108227 | 1.0296625 | down | mRNA |
| NM_001104647 | SLC25A36 | 0.029305414 | 0.33342903 | 1.2256569 | down | mRNA |
| NM_001104925 | PCMTD2 | 0.08558944 | 0.49748556 | 1.1381909 | down | mRNA |
| NM_001105193 | FAM123C | 0.898918819 | 0.99453218 | 1.0208705 | down | mRNA |
| NM_001105203 | RUSC1 | 0.901738308 | 0.994906068 | 1.0398708 | down | mRNA |
| NM_001105204 | RUSC1 | 0.430739365 | 0.879381002 | 1.2720002 | down | mRNA |
| NM_001105205 | RUSC1 | 0.886941368 | 0.993077367 | 1.0185579 | down | mRNA |
| NM_001105206 | LAMA4 | 0.371636117 | 0.846717338 | 7.1239135 | down | mRNA |
| NM_001105214 | ASH2L | 0.68419197 | 0.960111041 | 1.0527361 | down | mRNA |
| NM_001105247 | ARMC5 | 0.330251698 | 0.821769369 | 1.0998227 | down | mRNA |
| NM_001105520 | C17orf100 | 0.146451791 | 0.608122555 | 1.1567316 | down | mRNA |
| NM_001105521 | JAKMIP3 | 0.593157597 | 0.938046215 | 1.1063953 | down | mRNA |
| NM_001105530 | CAP1 | 0.17785196 | 0.658188638 | 1.1422337 | down | mRNA |
| NM_001105531 | FAM135A | 0.00048395 | 0.066965341 | 1.4546029 | down | mRNA |
| NM_001105538 | MYBBP1A | 0.318468382 | 0.812422372 | 1.1055352 | down | mRNA |
| NM_001105541 | NEFM | 0.365504651 | 0.845008704 | 1.0991323 | down | mRNA |
| NM_001105544 | ECHDC1 | 0.281957437 | 0.779542917 | 1.1051187 | down | mRNA |
| NM_001105545 | ECHDC1 | 0.280787242 | 0.779126326 | 1.0733493 | down | mRNA |
| NM_001105552 | ZNF83 | 0.409716733 | 0.86801103 | 1.1369128 | down | mRNA |
| NM_001105553 | ZNF83 | 0.341856646 | 0.830223283 | 1.1495717 | down | mRNA |
| NM_001105562 | UBE4B | 0.028920353 | 0.331085937 | 1.2074695 | down | mRNA |
| NM_001105563 | CCHCR1 | 0.685622201 | 0.960111041 | 1.1254559 | down | mRNA |
| NM_001105566 | KIF13A | 0.030654366 | 0.338914937 | 1.1675735 | down | mRNA |
| NM_001105567 | KIF13A | 0.939281585 | 0.996710991 | 1.0108788 | down | mRNA |
| NM_001105568 | KIF13A | 0.008926615 | 0.202329498 | 1.3371455 | down | mRNA |
| NM_001105570 | NUDT19 | 0.206908319 | 0.698502807 | 1.1383652 | down | mRNA |
| NM_001105573 | FBXO45 | 0.030076789 | 0.336740788 | 1.2005592 | down | mRNA |
| NM_001106 | ACVR2B | 0.097867965 | 0.520535504 | 1.2512086 | down | mRNA |
| NM_001109 | ADAM8 | 0.870736339 | 0.989592579 | 1.0729594 | down | mRNA |
| NM_001109619 | HIGD1C | 0.53185319 | 0.920228311 | 1.0984222 | down | mRNA |
| NM_001109662 | C12orf51 | 0.123817816 | 0.577744894 | 1.1479927 | down | mRNA |
| NM_001109754 | PTPRB | 0.074330815 | 0.469637706 | 1.5983377 | down | mRNA |
| NM_001109891 | MAPK3 | 0.349566838 | 0.834138149 | 1.0546929 | down | mRNA |
| NM_001109938 | C6orf136 | 0.156614036 | 0.625226111 | 1.1551663 | down | mRNA |
| NM_001110 | ADAM10 | 0.236742241 | 0.73465251 | 1.1073076 | down | mRNA |
| NM_001110514 | EBF4 | 0.269138092 | 0.76973577 | 1.1609539 | down | mRNA |
| NM_001110781 | SLC35E2B | 0.093205229 | 0.512313794 | 1.2761763 | down | mRNA |
| NM_001110798 | NAA16 | 0.775615966 | 0.97589756 | 1.0847443 | down | mRNA |
| NM_001111018 | NAV2 | 0.989103707 | 0.999140676 | 1.0068956 | down | mRNA |
| NM_001111077 | EZR | 0.987386616 | 0.999140676 | 1.0031866 | down | mRNA |
| NM_001111112 | UBE2K | 0.626934358 | 0.949203235 | 1.0407168 | down | mRNA |
| NM_001111113 | UBE2K | 0.351098635 | 0.835187247 | 1.0784583 | down | mRNA |
| NM_001111285 | IGF1 | 0.416726821 | 0.870736736 | 1.5558467 | down | mRNA |
| NM_001111322 | DDX54 | 0.003089129 | 0.131960624 | 1.1708434 | down | mRNA |
| NM_001112 | ADARB1 | 0.663710796 | 0.958354006 | 1.10657 | down | mRNA |
| NM_001112717 | LIMCH1 | 0.877028223 | 0.991255275 | 1.0298617 | down | mRNA |
| NM_001112726 | KIAA0284 | 0.003035352 | 0.130475228 | 1.3233179 | down | mRNA |
| NM_001112736 | FAM208A | 0.03935306 | 0.36991513 | 1.1268724 | down | mRNA |
| NM_001112802 | SLC8A1 | 0.787022242 | 0.977479172 | 1.0345518 | down | mRNA |
| NM_001113202 | NACA | 0.487957865 | 0.903757389 | 1.0705081 | down | mRNA |
| NM_001113203 | NACA | 0.892990517 | 0.993978977 | 1.0183989 | down | mRNA |
| NM_001113206 | TSTD1 | 0.997620896 | 0.999889342 | 1.0019254 | down | mRNA |
| NM_001113378 | FANCI | 0.799563668 | 0.981094055 | 1.166495 | down | mRNA |
| NM_001113380 | RGS4 | 0.136420584 | 0.595125435 | 1.7549848 | down | mRNA |
| NM_001113381 | RGS4 | 0.091223889 | 0.50968981 | 1.900194 | down | mRNA |
| NM_001113407 | LDB1 | 0.253187097 | 0.753288068 | 1.07565 | down | mRNA |
| NM_001113411 | FGGY | 0.939739461 | 0.996710991 | 1.0128172 | down | mRNA |
| NM_001113434 | C17orf51 | 0.816564684 | 0.981692737 | 1.0367552 | down | mRNA |
| NM_001113482 | MANEAL | 0.511198595 | 0.912481293 | 1.0838046 | down | mRNA |
| NM_001113498 | MDGA2 | 0.629867817 | 0.950410442 | 1.0625471 | down | mRNA |
| NM_001113528 | METTL15 | 0.415658384 | 0.870382888 | 1.0860497 | down | mRNA |
| NM_001113575 | CDKL3 | 0.895738843 | 0.993978977 | 1.025981 | down | mRNA |
| NM_001113738 | ARL17A | 0.003541549 | 0.138758659 | 1.4621589 | down | mRNA |
| NM_001114 | ADCY7 | 0.107836495 | 0.544052509 | 1.188706 | down | mRNA |
| NM_001114089 | ENTPD6 | 0.282406525 | 0.780042042 | 1.1089956 | down | mRNA |
| NM_001114090 | NGEF | 0.387467058 | 0.856773191 | 1.1058216 | down | mRNA |
| NM_001114092 | THUMPD3 | 0.040102651 | 0.371915487 | 1.2397611 | down | mRNA |
| NM_001114094 | BLNK | 0.68161578 | 0.960111041 | 1.0213751 | down | mRNA |
| NM_001114108 | TTC22 | 0.444024519 | 0.885088246 | 1.1437048 | down | mRNA |
| NM_001114118 | C17orf85 | 0.015433191 | 0.249037809 | 1.3508327 | down | mRNA |
| NM_001114132 | NBEAL1 | 0.93129839 | 0.996710991 | 1.0070466 | down | mRNA |
| NM_001114133 | SYNPO2L | 0.585994873 | 0.93505319 | 1.1029586 | down | mRNA |
| NM_001114176 | AEBP2 | 0.206489536 | 0.69797926 | 1.361999 | down | mRNA |
| NM_001114184 | MTRF1L | 0.336868798 | 0.826617309 | 1.1264949 | down | mRNA |
| NM_001114357 | C4orf47 | 0.047711398 | 0.397835916 | 1.3003551 | down | mRNA |
| NM_001114377 | FOXP3 | 0.597887127 | 0.940150767 | 1.0497413 | down | mRNA |
| NM_001114382 | TSC2 | 0.969950908 | 0.998245244 | 1.0036665 | down | mRNA |
| NM_001114395 | CNTLN | 0.971015158 | 0.998441289 | 1.0028343 | down | mRNA |
| NM_001114397 | DHX36 | 0.527140237 | 0.918238774 | 1.0574943 | down | mRNA |
| NM_001114600 | SZRD1 | 0.989368225 | 0.999140676 | 1.0011773 | down | mRNA |
| NM_001114617 | MGAT1 | 0.92224123 | 0.995349793 | 1.0083641 | down | mRNA |
| NM_001114632 | JMJD7 | 0.733011942 | 0.967703318 | 1.1138838 | down | mRNA |
| NM_001114633 | PLA2G4B | 0.533233016 | 0.920530493 | 1.1015802 | down | mRNA |
| NM_001114636 | FANCL | 0.54667822 | 0.924803608 | 1.0456743 | down | mRNA |
| NM_001114748 | TMEM240 | 0.434662518 | 0.881074297 | 1.2472374 | down | mRNA |
| NM_001114753 | ENG | 0.702335758 | 0.963378163 | 1.0364878 | down | mRNA |
| NM_001114938 | CCDC17 | 0.356714257 | 0.839337376 | 1.2490726 | down | mRNA |
| NM_001114979 | TP63 | 0.067144022 | 0.451445852 | 1.2055774 | down | mRNA |
| NM_001115007 | LIN54 | 0.136561354 | 0.595318495 | 1.470399 | down | mRNA |
| NM_001115156 | GDI2 | 0.061716794 | 0.438481196 | 1.2588558 | down | mRNA |
| NM_001116 | ADCY9 | 0.693035273 | 0.961584652 | 1.0861329 | down | mRNA |
| NM_001120 | MFSD10 | 0.456962079 | 0.891568474 | 1.0492292 | down | mRNA |
| NM_001122631 | CDKN1C | 0.829930584 | 0.985549562 | 1.0518395 | down | mRNA |
| NM_001122634 | CPS1 | 0.790259276 | 0.978438986 | 1.1055317 | down | mRNA |
| NM_001122646 | FAM178B | 0.944516663 | 0.996710991 | 1.0105502 | down | mRNA |
| NM_001122670 | PHKA1 | 0.611539418 | 0.943894921 | 1.0296598 | down | mRNA |
| NM_001122671 | CCBL1 | 0.836360161 | 0.986575022 | 1.0616155 | down | mRNA |
| NM_001122672 | CCBL1 | 0.188967363 | 0.6744312 | 1.2262689 | down | mRNA |
| NM_001122679 | ODZ2 | 0.625791986 | 0.948851902 | 1.0596487 | down | mRNA |
| NM_001122769 | LCA5 | 0.032300484 | 0.343300278 | 1.670633 | down | mRNA |
| NM_001122823 | GTF3C5 | 0.058919547 | 0.431383592 | 1.1341043 | down | mRNA |
| NM_001122870 | PPM1M | 0.624640606 | 0.948341756 | 1.1242969 | down | mRNA |
| NM_001122898 | CD99 | 0.950566539 | 0.996710991 | 1.0199895 | down | mRNA |
| NM_001122951 | DARC | 0.386317155 | 0.855747763 | 1.0715604 | down | mRNA |
| NM_001122955 | BSCL2 | 0.035688571 | 0.354652995 | 1.1277395 | down | mRNA |
| NM_001122956 | DBNL | 0.438258778 | 0.882682266 | 1.0484309 | down | mRNA |
| NM_001122957 | BCKDK | 0.001891791 | 0.105670067 | 1.1614567 | down | mRNA |
| NM_001122964 | SMEK2 | 0.041228537 | 0.376238157 | 1.1516102 | down | mRNA |
| NM_001123 | ADK | 0.58290118 | 0.934269626 | 1.0698065 | down | mRNA |
| NM_001123168 | FAM72A | 0.75306329 | 0.970725371 | 1.0838506 | down | mRNA |
| NM_001123226 | MTO1 | 0.205293679 | 0.695403826 | 1.1587123 | down | mRNA |
| NM_001123329 | ZBTB1 | 0.010981289 | 0.214890824 | 1.2783829 | down | mRNA |
| NM_001123364 | METTL24 | 0.974377608 | 0.998501532 | 1.0052241 | down | mRNA |
| NM_001123369 | PPP6C | 0.036893856 | 0.359628653 | 1.110348 | down | mRNA |
| NM_001123375 | HIST2H3D | 0.543596123 | 0.923577434 | 1.4988839 | down | mRNA |
| NM_001123376 | TMEM72 | 0.753523878 | 0.970725371 | 1.0288452 | down | mRNA |
| NM_001123378 | ADSL | 0.272582877 | 0.772533462 | 1.0967952 | down | mRNA |
| NM_001124767 | C3orf78 | 0.008170078 | 0.197051047 | 1.0929474 | down | mRNA |
| NM_001126 | ADSS | 0.672057821 | 0.959531882 | 1.0238441 | down | mRNA |
| NM_001126051 | HDGF | 0.692867733 | 0.961584652 | 1.1264902 | down | mRNA |
| NM_001126054 | CASK | 0.093356142 | 0.512497334 | 1.2027034 | down | mRNA |
| NM_001126055 | CASK | 0.411419633 | 0.86887062 | 1.0411142 | down | mRNA |
| NM_001126056 | DMKN | 0.223112344 | 0.71761506 | 1.1563354 | down | mRNA |
| NM_001126059 | DMKN | 0.044973059 | 0.389103076 | 2.0056976 | down | mRNA |
| NM_001126063 | KHDC1L | 0.846063614 | 0.987338076 | 1.012392 | down | mRNA |
| NM_001126107 | SLC12A3 | 0.859937064 | 0.988781331 | 1.0202826 | down | mRNA |
| NM_001126111 | OSGIN2 | 0.919083392 | 0.995349793 | 1.0113719 | down | mRNA |
| NM_001126122 | SLC25A19 | 0.157291327 | 0.626085994 | 1.6186168 | down | mRNA |
| NM_001126129 | GINS3 | 0.181883987 | 0.664924727 | 1.0957174 | down | mRNA |
| NM_001126241 | TP73 | 0.036747018 | 0.359628653 | 1.1564252 | down | mRNA |
| NM_001126336 | VCAN | 0.184761986 | 0.668017166 | 1.1293469 | down | mRNA |
| NM_001127 | AP1B1 | 0.485158621 | 0.903250293 | 1.0574299 | down | mRNA |
| NM_001127174 | MRO | 0.891448163 | 0.9937521 | 1.0194324 | down | mRNA |
| NM_001127178 | PIGG | 0.152018673 | 0.617551179 | 1.1564744 | down | mRNA |
| NM_001127179 | MYO7A | 0.461145517 | 0.89460367 | 1.2477679 | down | mRNA |
| NM_001127180 | MYO7A | 0.675045534 | 0.959531882 | 1.2209253 | down | mRNA |
| NM_001127181 | CENPL | 0.527598877 | 0.918678174 | 1.3395711 | down | mRNA |
| NM_001127182 | CEP55 | 0.995759607 | 0.999872237 | 1.0022226 | down | mRNA |
| NM_001127190 | CSK | 0.677594734 | 0.959531882 | 1.0783923 | down | mRNA |
| NM_001127192 | CNBP | 0.21048669 | 0.701214058 | 1.0433653 | down | mRNA |
| NM_001127193 | CNBP | 0.138993087 | 0.598648178 | 1.1484744 | down | mRNA |
| NM_001127194 | CNBP | 0.070333941 | 0.458686035 | 1.1604706 | down | mRNA |
| NM_001127196 | CNBP | 0.039447226 | 0.370286706 | 1.1781038 | down | mRNA |
| NM_001127211 | KIAA1598 | 0.348692804 | 0.834138149 | 1.0961962 | down | mRNA |
| NM_001127212 | GAGE2A | 0.599499614 | 0.940648177 | 1.0897932 | down | mRNA |
| NM_001127213 | LY6E | 0.780868959 | 0.976938981 | 1.0344807 | down | mRNA |
| NM_001127214 | ACSF3 | 0.326650113 | 0.819628544 | 1.0580134 | down | mRNA |
| NM_001127218 | POLD2 | 0.623599529 | 0.948341211 | 1.0793048 | down | mRNA |
| NM_001127235 | GPBP1 | 0.320544346 | 0.814552264 | 1.1497974 | down | mRNA |
| NM_001127236 | GPBP1 | 0.851862728 | 0.987678166 | 1.0204725 | down | mRNA |
| NM_001127240 | BBC3 | 0.537211988 | 0.922702335 | 1.1709506 | down | mRNA |
| NM_001127266 | TMEM129 | 0.386931498 | 0.856421862 | 1.3185036 | down | mRNA |
| NM_001127325 | MAD2L2 | 0.627852922 | 0.949611308 | 1.0148243 | down | mRNA |
| NM_001127328 | ACADM | 0.115226583 | 0.560621161 | 1.3515826 | down | mRNA |
| NM_001127357 | PHTF2 | 0.061530985 | 0.437893235 | 1.274905 | down | mRNA |
| NM_001127358 | PHTF2 | 0.222459643 | 0.71737156 | 1.1890334 | down | mRNA |
| NM_001127359 | PHTF2 | 0.013414569 | 0.235531104 | 1.2526904 | down | mRNA |
| NM_001127362 | NR2C1 | 0.282320633 | 0.780016729 | 1.1363458 | down | mRNA |
| NM_001127364 | FAM221A | 0.720215032 | 0.966191059 | 1.174263 | down | mRNA |
| NM_001127370 | CDCA7L | 0.265841286 | 0.766569994 | 1.2916532 | down | mRNA |
| NM_001127371 | CDCA7L | 0.184364332 | 0.66791858 | 1.3936112 | down | mRNA |
| NM_001127383 | CYBRD1 | 0.891188077 | 0.9937521 | 1.0165346 | down | mRNA |
| NM_001127386 | DUX4L2 | 0.825067401 | 0.98381451 | 1.053198 | down | mRNA |
| NM_001127388 | DUX4L6 | 0.127117416 | 0.580406758 | 1.0818545 | down | mRNA |
| NM_001127391 | ALS2CR12 | 0.710189925 | 0.965462762 | 1.1288527 | down | mRNA |
| NM_001127394 | TSEN15 | 0.287157604 | 0.782954059 | 1.1213976 | down | mRNA |
| NM_001127396 | STXBP2 | 0.552619575 | 0.926337111 | 1.1339846 | down | mRNA |
| NM_001127397 | ERLEC1 | 0.093440272 | 0.512497334 | 1.1325829 | down | mRNA |
| NM_001127398 | ERLEC1 | 0.438407319 | 0.882830305 | 1.0734016 | down | mRNA |
| NM_001127482 | SPRYD7 | 0.010054398 | 0.207961374 | 1.2157956 | down | mRNA |
| NM_001127487 | FLNC | 0.724053172 | 0.966191059 | 1.0525092 | down | mRNA |
| NM_001127500 | MET | 0.648822106 | 0.95550188 | 1.1494977 | down | mRNA |
| NM_001127501 | ALPL | 0.480027042 | 0.901538317 | 1.2989449 | down | mRNA |
| NM_001127505 | ASAH1 | 0.953530831 | 0.996710991 | 1.0057527 | down | mRNA |
| NM_001127507 | BANK1 | 0.89958491 | 0.994708735 | 1.0270248 | down | mRNA |
| NM_001127510 | APC | 0.000671796 | 0.076919551 | 1.3328225 | down | mRNA |
| NM_001127582 | ING4 | 0.356528613 | 0.839204431 | 1.2199382 | down | mRNA |
| NM_001127584 | ING4 | 0.397691603 | 0.863112281 | 1.1243537 | down | mRNA |
| NM_001127585 | ING4 | 0.31276934 | 0.808086566 | 1.2334987 | down | mRNA |
| NM_001127586 | ING4 | 0.318821962 | 0.812850403 | 1.203072 | down | mRNA |
| NM_001127603 | NMRK1 | 0.830431175 | 0.985549562 | 1.0413384 | down | mRNA |
| NM_001127617 | ALDOA | 0.237210215 | 0.735183924 | 1.0451413 | down | mRNA |
| NM_001127645 | GABRA1 | 0.780616878 | 0.976938981 | 1.0412583 | down | mRNA |
| NM_001127671 | LIFR | 0.609020895 | 0.943053241 | 1.1038364 | down | mRNA |
| NM_001127696 | ATXN3 | 0.498765482 | 0.909361611 | 1.0669824 | down | mRNA |
| NM_001127709 | PRG4 | 0.990625817 | 0.999375018 | 1.0100288 | down | mRNA |
| NM_001127713 | ATL1 | 0.313822326 | 0.808965923 | 1.0995791 | down | mRNA |
| NM_001127715 | STXBP5 | 0.612934674 | 0.944423643 | 1.1120814 | down | mRNA |
| NM_001127889 | SPG21 | 0.254517759 | 0.754902272 | 1.2420534 | down | mRNA |
| NM_001127890 | SPG21 | 0.261636103 | 0.762395628 | 1.2234287 | down | mRNA |
| NM_001127893 | CEACAM19 | 0.940045033 | 0.996710991 | 1.0093893 | down | mRNA |
| NM_001127897 | RPGRIP1L | 0.197820536 | 0.686504248 | 1.4170616 | down | mRNA |
| NM_001128 | AP1G1 | 0.19047932 | 0.676402188 | 1.176098 | down | mRNA |
| NM_001128126 | AP4S1 | 0.67254424 | 0.959531882 | 1.0485481 | down | mRNA |
| NM_001128142 | RWDD3 | 0.348684465 | 0.834138149 | 1.186573 | down | mRNA |
| NM_001128143 | PMS1 | 0.00315294 | 0.132624561 | 1.7295308 | down | mRNA |
| NM_001128144 | PMS1 | 0.367533554 | 0.845181673 | 1.1880277 | down | mRNA |
| NM_001128147 | NF1 | 0.912865886 | 0.995342392 | 1.023747 | down | mRNA |
| NM_001128149 | ATXN7 | 0.601508042 | 0.941048131 | 1.1004377 | down | mRNA |
| NM_001128165 | FBLN7 | 0.172350263 | 0.6499464 | 1.1406391 | down | mRNA |
| NM_001128214 | KCTD6 | 0.486556687 | 0.903524001 | 1.3589167 | down | mRNA |
| NM_001128217 | PSIP1 | 0.524707441 | 0.917632259 | 1.0620362 | down | mRNA |
| NM_001128219 | VGLL4 | 0.002430573 | 0.117962433 | 2.1557624 | down | mRNA |
| NM_001128220 | VGLL4 | 0.007409522 | 0.190938402 | 2.0166774 | down | mRNA |
| NM_001128221 | VGLL4 | 0.017679399 | 0.267776588 | 1.8718112 | down | mRNA |
| NM_001128225 | SLC39A13 | 0.833637236 | 0.986434891 | 1.0138569 | down | mRNA |
| NM_001128226 | DIS3 | 0.665447033 | 0.958354006 | 1.0433966 | down | mRNA |
| NM_001128227 | GNE | 0.770098252 | 0.975286259 | 1.0291244 | down | mRNA |
| NM_001128324 | ZFAND4 | 0.453280712 | 0.889563297 | 1.0614173 | down | mRNA |
| NM_001128325 | SPON2 | 0.809728257 | 0.98118927 | 1.0163582 | down | mRNA |
| NM_001128426 | AP1AR | 0.582387666 | 0.934005566 | 1.0839565 | down | mRNA |
| NM_001128429 | SMARCAD1 | 0.328355002 | 0.821170223 | 1.0224944 | down | mRNA |
| NM_001128610 | USP8 | 0.186812231 | 0.670479485 | 1.1131063 | down | mRNA |
| NM_001128612 | NUDT22 | 0.123436163 | 0.577076127 | 1.1168139 | down | mRNA |
| NM_001128617 | REPS1 | 0.137854048 | 0.596789644 | 1.2746661 | down | mRNA |
| NM_001128620 | PAK1 | 0.027791665 | 0.32381669 | 1.2207233 | down | mRNA |
| NM_001128626 | SPIRE1 | 0.050853299 | 0.407751844 | 1.1582625 | down | mRNA |
| NM_001128629 | PAK6 | 0.009639853 | 0.205650016 | 1.2580706 | down | mRNA |
| NM_001128633 | RIMBP3C | 0.001891017 | 0.105670067 | 2.1702484 | down | mRNA |
| NM_001128635 | RIMBP3B | 0.264565136 | 0.765822658 | 1.2874308 | down | mRNA |
| NM_001128827 | DLG4 | 0.440289853 | 0.883544565 | 1.0469624 | down | mRNA |
| NM_001128833 | ZBTB4 | 0.009922698 | 0.207085174 | 1.3183513 | down | mRNA |
| NM_001128835 | TRAPPC2 | 0.009728877 | 0.206100289 | 1.1536098 | down | mRNA |
| NM_001128845 | SMARCA4 | 0.090257346 | 0.507468901 | 1.1981012 | down | mRNA |
| NM_001128846 | SMARCA4 | 0.006555381 | 0.182886161 | 1.298261 | down | mRNA |
| NM_001128847 | SMARCA4 | 0.060002456 | 0.434771891 | 1.1401082 | down | mRNA |
| NM_001128849 | SMARCA4 | 0.072153 | 0.462926723 | 1.1712984 | down | mRNA |
| NM_001128854 | SRRT | 0.9690192 | 0.998196842 | 1.0017908 | down | mRNA |
| NM_001128911 | PCBP2 | 0.204580861 | 0.694645636 | 1.1704465 | down | mRNA |
| NM_001128913 | PCBP2 | 0.265242369 | 0.766494847 | 1.0571483 | down | mRNA |
| NM_001128914 | PCBP2 | 0.927066021 | 0.995829678 | 1.0114152 | down | mRNA |
| NM_001128928 | INPP1 | 0.22060848 | 0.715799221 | 1.2696363 | down | mRNA |
| NM_001128930 | ENTPD4 | 0.490498795 | 0.905300886 | 1.1649816 | down | mRNA |
| NM_001128933 | SYNPO2 | 0.920550406 | 0.995349793 | 1.1231276 | down | mRNA |
| NM_001129831 | CACNA1C | 0.286566296 | 0.782942227 | 1.1183751 | down | mRNA |
| NM_001129832 | CACNA1C | 0.383113979 | 0.853514109 | 1.0812907 | down | mRNA |
| NM_001129833 | CACNA1C | 0.622170799 | 0.948131965 | 1.033578 | down | mRNA |
| NM_001129834 | CACNA1C | 0.927273113 | 0.995829678 | 1.0265902 | down | mRNA |
| NM_001129837 | CACNA1C | 0.138493858 | 0.597943156 | 1.1198428 | down | mRNA |
| NM_001129838 | CACNA1C | 0.553258063 | 0.926337111 | 1.0738882 | down | mRNA |
| NM_001129839 | CACNA1C | 0.167519518 | 0.642346587 | 1.1366715 | down | mRNA |
| NM_001129841 | CACNA1C | 0.029089015 | 0.331684822 | 1.0971535 | down | mRNA |
| NM_001129887 | DNAJC2 | 0.04209735 | 0.379087707 | 1.1608666 | down | mRNA |
| NM_001129891 | FAM196B | 0.561718925 | 0.928676955 | 1.0844453 | down | mRNA |
| NM_001129898 | ZNF673 | 0.068127476 | 0.452817212 | 1.3229364 | down | mRNA |
| NM_001129908 | FAM198A | 0.006447359 | 0.18106035 | 1.6195083 | down | mRNA |
| NM_001129981 | ANKRD2 | 0.660202795 | 0.958354006 | 1.151412 | down | mRNA |
| NM_001129993 | KIAA1841 | 0.442660661 | 0.884277516 | 1.2520269 | down | mRNA |
| NM_001129994 | KCTD15 | 0.2307487 | 0.728071705 | 1.2418642 | down | mRNA |
| NM_001129996 | ZNF222 | 0.190628444 | 0.676402188 | 1.202638 | down | mRNA |
| NM_001130005 | ACTN1 | 0.956271285 | 0.996883425 | 1.0126277 | down | mRNA |
| NM_001130007 | GSPT1 | 0.16997022 | 0.646789844 | 1.0847977 | down | mRNA |
| NM_001130022 | ZNF680 | 0.750006823 | 0.970725371 | 1.0316375 | down | mRNA |
| NM_001130028 | CLK3 | 0.772926899 | 0.975793214 | 1.0430844 | down | mRNA |
| NM_001130029 | RELL2 | 0.094106233 | 0.514069835 | 1.521484 | down | mRNA |
| NM_001130032 | ZNF562 | 0.335290055 | 0.82511709 | 1.0228994 | down | mRNA |
| NM_001130034 | PLEKHB1 | 0.71850601 | 0.966191059 | 1.0870003 | down | mRNA |
| NM_001130041 | SHC1 | 0.025841113 | 0.315177872 | 1.1011495 | down | mRNA |
| NM_001130042 | CRYZ | 0.654387927 | 0.957205183 | 1.2036015 | down | mRNA |
| NM_001130043 | CRYZ | 0.967009311 | 0.99793927 | 1.0163594 | down | mRNA |
| NM_001130047 | TBCEL | 0.00030379 | 0.055508758 | 1.2475574 | down | mRNA |
| NM_001130048 | DOCK9 | 0.425687384 | 0.87545849 | 1.0744003 | down | mRNA |
| NM_001130053 | EEF1D | 0.489234458 | 0.904620847 | 1.1292336 | down | mRNA |
| NM_001130057 | EEF1D | 0.197040279 | 0.685161614 | 1.0640124 | down | mRNA |
| NM_001130065 | MYO9B | 0.888882951 | 0.993445919 | 1.0177061 | down | mRNA |
| NM_001130067 | TRIM2 | 0.42039325 | 0.872365335 | 1.0465631 | down | mRNA |
| NM_001130086 | ABLIM2 | 0.873421894 | 0.990580595 | 1.0386541 | down | mRNA |
| NM_001130087 | ABLIM2 | 0.977629844 | 0.998501532 | 1.0053564 | down | mRNA |
| NM_001130090 | LRRC48 | 0.121967452 | 0.574170462 | 1.3257852 | down | mRNA |
| NM_001130099 | KIFC3 | 0.280262171 | 0.778821055 | 1.2404755 | down | mRNA |
| NM_001130100 | KIFC3 | 0.122804212 | 0.574983504 | 1.2014695 | down | mRNA |
| NM_001130101 | NR1H3 | 0.334734532 | 0.824597423 | 1.0833046 | down | mRNA |
| NM_001130102 | NR1H3 | 0.160307572 | 0.631980676 | 1.2282393 | down | mRNA |
| NM_001130105 | COL4A3BP | 0.391067884 | 0.858960277 | 1.3305554 | down | mRNA |
| NM_001130106 | HSPBP1 | 0.006981996 | 0.186688411 | 1.1535023 | down | mRNA |
| NM_001130107 | KLC1 | 0.436090744 | 0.881677506 | 1.0951362 | down | mRNA |
| NM_001130110 | SETBP1 | 0.805218776 | 0.98118927 | 1.1645791 | down | mRNA |
| NM_001130144 | LTBP3 | 0.928456218 | 0.996066203 | 1.0113468 | down | mRNA |
| NM_001130148 | CCDC77 | 0.140789184 | 0.60102484 | 1.1341719 | down | mRNA |
| NM_001130157 | MLF1 | 0.670281402 | 0.959531882 | 1.2053231 | down | mRNA |
| NM_001130158 | MYO1B | 0.405519655 | 0.866165103 | 1.0991178 | down | mRNA |
| NM_001130162 | STOX1 | 0.849825575 | 0.987678166 | 1.0750892 | down | mRNA |
| NM_001130167 | PSG8 | 0.84898999 | 0.987678166 | 1.1386752 | down | mRNA |
| NM_001130415 | APOLD1 | 0.856105329 | 0.988315074 | 1.0463741 | down | mRNA |
| NM_001130438 | SPTAN1 | 0.804554779 | 0.98118927 | 1.0286074 | down | mRNA |
| NM_001130440 | SRP9 | 0.482858507 | 0.902654749 | 1.0479059 | down | mRNA |
| NM_001130445 | SSFA2 | 0.292422776 | 0.788954556 | 1.2521936 | down | mRNA |
| NM_001130481 | VRK2 | 0.243452104 | 0.741870658 | 1.3569963 | down | mRNA |
| NM_001130482 | VRK2 | 0.078674627 | 0.480652799 | 1.4587046 | down | mRNA |
| NM_001130483 | VRK2 | 0.995785872 | 0.999872237 | 1.0009708 | down | mRNA |
| NM_001130517 | MPST | 0.31070826 | 0.805427509 | 1.2132451 | down | mRNA |
| NM_001130519 | ZNF195 | 0.452159516 | 0.888854908 | 1.0440904 | down | mRNA |
| NM_001130674 | CALU | 0.601444666 | 0.941048131 | 1.073516 | down | mRNA |
| NM_001130689 | HMGB2 | 0.66798811 | 0.958403376 | 1.17982 | down | mRNA |
| NM_001130691 | PPP3CA | 0.269248515 | 0.76973577 | 1.099513 | down | mRNA |
| NM_001130692 | PPP3CA | 0.023586256 | 0.301884214 | 1.1694523 | down | mRNA |
| NM_001130701 | STYX | 0.481218552 | 0.901538317 | 1.0714012 | down | mRNA |
| NM_001130706 | C14orf93 | 0.062618812 | 0.439458294 | 1.0758742 | down | mRNA |
| NM_001130709 | RUFY3 | 0.001148245 | 0.088098571 | 1.459854 | down | mRNA |
| NM_001130721 | ELOVL6 | 0.471982318 | 0.899039207 | 1.08702 | down | mRNA |
| NM_001130727 | PACRGL | 0.012440233 | 0.226847373 | 1.3626625 | down | mRNA |
| NM_001130822 | RABL5 | 0.268889817 | 0.769564942 | 1.1046898 | down | mRNA |
| NM_001130825 | SUPT5H | 0.388017482 | 0.857332258 | 1.0813925 | down | mRNA |
| NM_001130834 | LDB2 | 0.783069475 | 0.977275912 | 1.1076718 | down | mRNA |
| NM_001130841 | RTCA | 0.269065182 | 0.769715598 | 1.114985 | down | mRNA |
| NM_001130842 | ZNF286A | 0.228955132 | 0.725208872 | 1.1790212 | down | mRNA |
| NM_001130846 | AIFM1 | 0.263420076 | 0.763813032 | 1.13105 | down | mRNA |
| NM_001130848 | PITPNM1 | 0.803297799 | 0.98118927 | 1.0316017 | down | mRNA |
| NM_001130852 | CBLC | 0.121614867 | 0.573155624 | 1.1221534 | down | mRNA |
| NM_001130858 | PPIP5K1 | 0.336913966 | 0.826617309 | 1.0716549 | down | mRNA |
| NM_001130862 | RAD51AP1 | 0.880695927 | 0.992287164 | 1.0964418 | down | mRNA |
| NM_001130863 | ENPP2 | 0.687872966 | 0.960301644 | 1.0727698 | down | mRNA |
| NM_001130864 | PWWP2A | 0.143084731 | 0.603147721 | 1.195425 | down | mRNA |
| NM_001130867 | ERCC2 | 0.052889412 | 0.413038171 | 1.4366153 | down | mRNA |
| NM_001130912 | SUGT1 | 0.623046591 | 0.948341211 | 1.0761521 | down | mRNA |
| NM_001130917 | LILRA2 | 0.100082528 | 0.52577303 | 1.1950749 | down | mRNA |
| NM_001130924 | TMEM201 | 0.419781384 | 0.872365335 | 1.0737175 | down | mRNA |
| NM_001130926 | MEF2A | 0.009815302 | 0.206905383 | 1.2620474 | down | mRNA |
| NM_001130927 | MEF2A | 0.000809589 | 0.083087642 | 1.4948759 | down | mRNA |
| NM_001130957 | C1orf101 | 0.772027581 | 0.975347132 | 1.1273233 | down | mRNA |
| NM_001130963 | TMEM194A | 0.798875335 | 0.980813744 | 1.0770828 | down | mRNA |
| NM_001130967 | ZNF385A | 0.125365371 | 0.578441772 | 1.1254054 | down | mRNA |
| NM_001130968 | ZNF385A | 0.223104652 | 0.71761506 | 1.2173127 | down | mRNA |
| NM_001130969 | NELF | 0.74503548 | 0.970378122 | 1.0497959 | down | mRNA |
| NM_001130971 | NELF | 0.58332337 | 0.934280269 | 1.0777642 | down | mRNA |
| NM_001130981 | DYSF | 0.411919887 | 0.869001481 | 1.4083093 | down | mRNA |
| NM_001130991 | HYOU1 | 0.518995998 | 0.916145597 | 1.1388211 | down | mRNA |
| NM_001130996 | HMGCR | 0.496363322 | 0.908376152 | 1.1677507 | down | mRNA |
| NM_001130997 | FAM58A | 0.023314779 | 0.300022844 | 1.1725377 | down | mRNA |
| NM_001131007 | KIAA0922 | 0.579764436 | 0.933893479 | 1.081415 | down | mRNA |
| NM_001131016 | CIZ1 | 0.379836181 | 0.853257014 | 1.1548824 | down | mRNA |
| NM_001131018 | CIZ1 | 0.293164455 | 0.790364658 | 1.1563006 | down | mRNA |
| NM_001131024 | PEX5 | 0.104513178 | 0.535513021 | 1.2564867 | down | mRNA |
| NM_001131025 | PEX5 | 0.716692177 | 0.966191059 | 1.0509527 | down | mRNA |
| NM_001131028 | ATG10 | 0.066173482 | 0.449693785 | 1.1426635 | down | mRNA |
| NM_001131062 | SAP30L | 0.386642876 | 0.856062164 | 1.0688872 | down | mRNA |
| NM_001131063 | SAP30L | 0.262358284 | 0.763125731 | 1.0933078 | down | mRNA |
| NM_001131064 | ACP2 | 0.105615565 | 0.537919754 | 1.0958763 | down | mRNA |
| NM_001131065 | RFESD | 0.565237868 | 0.930020145 | 1.1166964 | down | mRNA |
| NM_001134194 | ACPP | 0.524631625 | 0.917632259 | 1.1321426 | down | mRNA |
| NM_001134223 | USP46 | 0.674971562 | 0.959531882 | 1.0643298 | down | mRNA |
| NM_001134225 | INPP4A | 0.358967148 | 0.840767187 | 1.151013 | down | mRNA |
| NM_001134231 | NT5DC2 | 0.690140728 | 0.961105466 | 1.0821569 | down | mRNA |
| NM_001134232 | TMEM106B | 0.428791858 | 0.877386753 | 1.0962469 | down | mRNA |
| NM_001134296 | AP3M2 | 0.21955442 | 0.714276371 | 1.1922122 | down | mRNA |
| NM_001134336 | FAM40B | 0.130143101 | 0.584037049 | 1.0509465 | down | mRNA |
| NM_001134338 | RNF24 | 0.44737227 | 0.88723888 | 1.0408286 | down | mRNA |
| NM_001134368 | SLC6A6 | 0.974064231 | 0.998501532 | 1.0048223 | down | mRNA |
| NM_001134369 | LRRFIP2 | 0.063411562 | 0.440687083 | 1.2192542 | down | mRNA |
| NM_001134373 | NT5C2 | 0.385339004 | 0.85515381 | 1.0942706 | down | mRNA |
| NM_001134375 | CCNJ | 0.267053958 | 0.76755337 | 1.2426884 | down | mRNA |
| NM_001134376 | CCNJ | 0.720081692 | 0.966191059 | 1.0363322 | down | mRNA |
| NM_001134380 | TBC1D5 | 0.944850527 | 0.996710991 | 1.01141 | down | mRNA |
| NM_001134388 | ZDHHC4 | 0.81125536 | 0.98118927 | 1.0399415 | down | mRNA |
| NM_001134398 | VAV2 | 0.511441905 | 0.912481293 | 1.1583296 | down | mRNA |
| NM_001134400 | CYB561D1 | 0.258848816 | 0.759358563 | 1.2902185 | down | mRNA |
| NM_001134403 | CYB561D1 | 0.99945473 | 0.999897336 | 1.0000933 | down | mRNA |
| NM_001134404 | CYB561D1 | 0.85987389 | 0.988781331 | 1.0078872 | down | mRNA |
| NM_001134419 | CDC7 | 0.722137516 | 0.966191059 | 1.1637729 | down | mRNA |
| NM_001134430 | TOR2A | 0.299280868 | 0.79562655 | 1.2771904 | down | mRNA |
| NM_001134445 | DDAH1 | 0.660282587 | 0.958354006 | 1.0301123 | down | mRNA |
| NM_001134451 | TMEM130 | 0.946556633 | 0.996710991 | 1.0154834 | down | mRNA |
| NM_001134456 | NXPE3 | 0.014432465 | 0.242513124 | 1.3097462 | down | mRNA |
| NM_001134470 | C3orf58 | 0.216063683 | 0.710711878 | 1.3001108 | down | mRNA |
| NM_001134473 | KIAA0182 | 0.032843472 | 0.34498156 | 1.5134132 | down | mRNA |
| NM_001134477 | PARN | 0.000936978 | 0.084526081 | 1.2189687 | down | mRNA |
| NM_001134484 | TOMM5 | 0.247766849 | 0.745868458 | 1.0615983 | down | mRNA |
| NM_001134485 | TOMM5 | 0.117820969 | 0.564437853 | 1.1365974 | down | mRNA |
| NM_001134493 | TOMM6 | 0.826982044 | 0.984668477 | 1.0265559 | down | mRNA |
| NM_001134663 | SAMD13 | 0.663990892 | 0.958354006 | 1.0672188 | down | mRNA |
| NM_001134671 | DERL1 | 0.013739857 | 0.236215185 | 1.1534709 | down | mRNA |
| NM_001134693 | OST4 | 0.855546384 | 0.988178307 | 1.0107049 | down | mRNA |
| NM_001134709 | DEK | 0.213915787 | 0.706596013 | 1.095306 | down | mRNA |
| NM_001134758 | ARFRP1 | 0.599989622 | 0.940648177 | 1.0843648 | down | mRNA |
| NM_001134759 | CRYZ | 0.923725654 | 0.995456159 | 1.0255958 | down | mRNA |
| NM_001134772 | STX16 | 0.055250929 | 0.419634622 | 1.4339865 | down | mRNA |
| NM_001134773 | STX16 | 0.126044296 | 0.578441772 | 1.3214676 | down | mRNA |
| NM_001134774 | KLC2 | 0.547352776 | 0.925055514 | 1.0834495 | down | mRNA |
| NM_001134778 | PBX3 | 0.153890836 | 0.620938377 | 1.1442019 | down | mRNA |
| NM_001134779 | IPO11 | 0.040972415 | 0.375308608 | 1.2709988 | down | mRNA |
| NM_001134830 | AHI1 | 0.094757475 | 0.516057441 | 1.0520058 | down | mRNA |
| NM_001134848 | CCDC152 | 0.6545548 | 0.957205183 | 1.0421004 | down | mRNA |
| NM_001134855 | TRIM17 | 0.953954004 | 0.996710991 | 1.016725 | down | mRNA |
| NM_001134937 | FIP1L1 | 0.727626734 | 0.966191059 | 1.0243064 | down | mRNA |
| NM_001134938 | FIP1L1 | 0.132389037 | 0.587843174 | 1.2867805 | down | mRNA |
| NM_001134939 | OAZ3 | 0.238447214 | 0.736444734 | 1.1123311 | down | mRNA |
| NM_001134945 | GHRL | 0.194702882 | 0.682390954 | 1.5707395 | down | mRNA |
| NM_001134999 | FERMT2 | 0.455821632 | 0.891365829 | 1.1473417 | down | mRNA |
| NM_001135 | ACAN | 0.576567243 | 0.933656777 | 1.1643147 | down | mRNA |
| NM_001135000 | FERMT2 | 0.307822434 | 0.802596273 | 1.1726526 | down | mRNA |
| NM_001135005 | DNAJB5 | 0.755257317 | 0.971417904 | 1.0322592 | down | mRNA |
| NM_001135022 | ELMOD3 | 0.092469947 | 0.511338764 | 1.4463206 | down | mRNA |
| NM_001135024 | PDHX | 0.498737734 | 0.909361611 | 1.2000511 | down | mRNA |
| NM_001135036 | FAM18B2 | 0.363393095 | 0.843238468 | 1.8880766 | down | mRNA |
| NM_001135037 | MFAP3 | 0.103917539 | 0.533896783 | 1.1481059 | down | mRNA |
| NM_001135044 | MAPK9 | 0.059181969 | 0.432526163 | 1.0735643 | down | mRNA |
| NM_001135051 | FAM160B1 | 0.151207585 | 0.616500055 | 1.3466769 | down | mRNA |
| NM_001135054 | SIGIRR | 0.505129884 | 0.910568383 | 1.030054 | down | mRNA |
| NM_001135055 | TKT | 0.150611343 | 0.615623649 | 1.4109477 | down | mRNA |
| NM_001135086 | PRSS41 | 0.93821025 | 0.996710991 | 1.0060147 | down | mRNA |
| NM_001135098 | UPP2 | 0.903601874 | 0.99508468 | 1.0201641 | down | mRNA |
| NM_001135101 | CRELD2 | 0.779404531 | 0.976938981 | 1.030104 | down | mRNA |
| NM_001135106 | KCNK16 | 0.457977149 | 0.892646883 | 1.2105141 | down | mRNA |
| NM_001135110 | DNAJA3 | 0.000862326 | 0.083093876 | 1.3249091 | down | mRNA |
| NM_001135161 | COMT | 0.750927296 | 0.970725371 | 1.0456261 | down | mRNA |
| NM_001135168 | ALDH3A1 | 0.909569183 | 0.995342392 | 1.0078463 | down | mRNA |
| NM_001135177 | NEDD1 | 0.026744031 | 0.318484367 | 1.1444216 | down | mRNA |
| NM_001135179 | ZDHHC3 | 0.638370442 | 0.952902137 | 1.027049 | down | mRNA |
| NM_001135188 | AGFG1 | 0.390083837 | 0.858911043 | 1.1083584 | down | mRNA |
| NM_001135189 | AGFG1 | 0.666445923 | 0.958354006 | 1.1012338 | down | mRNA |
| NM_001135196 | C10orf71 | 0.334336312 | 0.824174306 | 1.0618247 | down | mRNA |
| NM_001135212 | FKBP7 | 0.578296313 | 0.933821303 | 1.0555909 | down | mRNA |
| NM_001135214 | GIT2 | 0.045039753 | 0.389144768 | 1.272151 | down | mRNA |
| NM_001135217 | LRRC23 | 0.460047149 | 0.89324475 | 1.2548435 | down | mRNA |
| NM_001135242 | NDRG1 | 0.722460809 | 0.966191059 | 1.0573657 | down | mRNA |
| NM_001135244 | TCOF1 | 0.023805733 | 0.303389731 | 1.2856224 | down | mRNA |
| NM_001135245 | TCOF1 | 0.204937304 | 0.694868044 | 1.1784287 | down | mRNA |
| NM_001135255 | RBBP4 | 0.064416738 | 0.443678541 | 1.1201136 | down | mRNA |
| NM_001135553 | MKNK1 | 0.222766424 | 0.717567638 | 1.0679523 | down | mRNA |
| NM_001135565 | HDHD1 | 0.775745444 | 0.97589756 | 1.0434405 | down | mRNA |
| NM_001135570 | C12orf73 | 0.087147191 | 0.500530139 | 1.2849186 | down | mRNA |
| NM_001135589 | GDAP2 | 0.596844871 | 0.939608975 | 1.0637539 | down | mRNA |
| NM_001135590 | ZNF577 | 0.066502445 | 0.450298982 | 1.2391187 | down | mRNA |
| NM_001135597 | CCDC88A | 0.107137138 | 0.542543535 | 1.1371225 | down | mRNA |
| NM_001135598 | CLHC1 | 0.130624874 | 0.585250095 | 1.3398219 | down | mRNA |
| NM_001135602 | GLB1 | 0.498921948 | 0.909361611 | 1.2343146 | down | mRNA |
| NM_001135608 | ARHGAP26 | 0.886099041 | 0.992981836 | 1.0500793 | down | mRNA |
| NM_001135629 | PPP1R21 | 0.040291539 | 0.371915487 | 1.2100696 | down | mRNA |
| NM_001135635 | C11orf68 | 0.567905695 | 0.931705674 | 1.152665 | down | mRNA |
| NM_001135643 | DCTN4 | 0.011825377 | 0.221393704 | 1.2741978 | down | mRNA |
| NM_001135644 | DCTN4 | 0.132703252 | 0.588376689 | 1.0736502 | down | mRNA |
| NM_001135648 | PTPRK | 0.411152185 | 0.868434057 | 1.1627457 | down | mRNA |
| NM_001135651 | EIF2AK2 | 0.366694258 | 0.845181673 | 1.0887339 | down | mRNA |
| NM_001135652 | EIF2AK2 | 0.812251773 | 0.98118927 | 1.017609 | down | mRNA |
| NM_001135653 | PABPC4 | 0.427875728 | 0.876992614 | 1.1427826 | down | mRNA |
| NM_001135654 | PABPC4 | 0.74448358 | 0.970330526 | 1.0344453 | down | mRNA |
| NM_001135655 | LY6H | 0.624897184 | 0.948658841 | 1.0494742 | down | mRNA |
| NM_001135662 | RAB7L1 | 0.718712364 | 0.966191059 | 1.0932002 | down | mRNA |
| NM_001135663 | RAB7L1 | 0.669274222 | 0.95930319 | 1.1069489 | down | mRNA |
| NM_001135664 | RAB7L1 | 0.749587813 | 0.970725371 | 1.1120435 | down | mRNA |
| NM_001135669 | XPR1 | 0.003855037 | 0.143554232 | 1.210414 | down | mRNA |
| NM_001135670 | MSRA | 0.631130731 | 0.950410442 | 1.0878171 | down | mRNA |
| NM_001135671 | MSRA | 0.548623093 | 0.92514258 | 1.119103 | down | mRNA |
| NM_001135673 | ATL2 | 0.008345523 | 0.198394583 | 1.2278761 | down | mRNA |
| NM_001135694 | VDAC3 | 0.294544481 | 0.791301949 | 1.0709519 | down | mRNA |
| NM_001135697 | SGCA | 0.288893537 | 0.784740601 | 1.0728988 | down | mRNA |
| NM_001135721 | PIWIL2 | 0.653399864 | 0.956860606 | 1.0859169 | down | mRNA |
| NM_001135729 | TOM1 | 0.683261769 | 0.960111041 | 1.0439774 | down | mRNA |
| NM_001135730 | TOM1 | 0.304375777 | 0.800038203 | 1.1598242 | down | mRNA |
| NM_001135731 | ZMAT4 | 0.897352566 | 0.994222243 | 1.0083951 | down | mRNA |
| NM_001135733 | TP53INP1 | 0.510034397 | 0.911891001 | 1.1363366 | down | mRNA |
| NM_001135734 | ZNF384 | 0.57555367 | 0.933656777 | 1.0228499 | down | mRNA |
| NM_001135745 | ALS2 | 0.030322487 | 0.337447626 | 1.3004714 | down | mRNA |
| NM_001135746 | NEIL2 | 0.246406168 | 0.744874529 | 1.3103319 | down | mRNA |
| NM_001135752 | ECD | 0.576506111 | 0.933656777 | 1.1114707 | down | mRNA |
| NM_001135768 | PVR | 0.673547057 | 0.959531882 | 1.0538872 | down | mRNA |
| NM_001135769 | PVR | 0.261257112 | 0.761996785 | 1.2212765 | down | mRNA |
| NM_001135770 | PVR | 0.770789154 | 0.975347132 | 1.0358953 | down | mRNA |
| NM_001135771 | RPN2 | 0.291502688 | 0.787986325 | 1.1282718 | down | mRNA |
| NM_001135811 | FAM60A | 0.009593589 | 0.205539348 | 1.7581801 | down | mRNA |
| NM_001135844 | SIRPB1 | 0.101749195 | 0.529637642 | 1.1663717 | down | mRNA |
| NM_001135863 | METTL20 | 0.021693538 | 0.29025889 | 1.4340281 | down | mRNA |
| NM_001135865 | LOC100132247 | 0.672611904 | 0.959531882 | 1.0761041 | down | mRNA |
| NM_001135911 | PIK3IP1 | 0.108071214 | 0.544140502 | 1.7000347 | down | mRNA |
| NM_001135919 | SLC46A3 | 0.689733688 | 0.961105466 | 1.2328765 | down | mRNA |
| NM_001135924 | VWDE | 0.37256236 | 0.84763089 | 1.0932163 | down | mRNA |
| NM_001135934 | POSTN | 0.652688989 | 0.95670626 | 1.2520611 | down | mRNA |
| NM_001135935 | POSTN | 0.608118509 | 0.942829459 | 1.3045278 | down | mRNA |
| NM_001135937 | SMAD2 | 0.198120222 | 0.686763988 | 1.1291843 | down | mRNA |
| NM_001135944 | MADD | 0.048206552 | 0.398753301 | 1.3631019 | down | mRNA |
| NM_001135947 | URM1 | 0.428268103 | 0.877211962 | 1.0840905 | down | mRNA |
| NM_001135954 | UCK1 | 0.094746781 | 0.516057441 | 1.1725811 | down | mRNA |
| NM_001135957 | TRPC4 | 0.938997193 | 0.996710991 | 1.0107827 | down | mRNA |
| NM_001135993 | TTC39C | 0.534673339 | 0.920879147 | 1.0802234 | down | mRNA |
| NM_001136 | AGER | 0.061014216 | 0.437118055 | 1.1680627 | down | mRNA |
| NM_001136006 | GART | 0.476239113 | 0.900415348 | 1.0397084 | down | mRNA |
| NM_001136012 | FXYD3 | 0.372579051 | 0.84763089 | 1.2445157 | down | mRNA |
| NM_001136015 | ANXA2 | 0.000835749 | 0.083093876 | 1.2746363 | down | mRNA |
| NM_001136022 | NFATC4 | 0.638979954 | 0.953242382 | 1.0267655 | down | mRNA |
| NM_001136025 | PLS3 | 0.575793965 | 0.933656777 | 1.0988443 | down | mRNA |
| NM_001136030 | TESPA1 | 0.791408123 | 0.978710629 | 1.0327698 | down | mRNA |
| NM_001136031 | ATG7 | 0.157725285 | 0.627322084 | 1.1061019 | down | mRNA |
| NM_001136032 | KLK11 | 0.856242623 | 0.988315074 | 1.0270403 | down | mRNA |
| NM_001136033 | PUF60 | 0.175040055 | 0.653821873 | 1.0295208 | down | mRNA |
| NM_001136036 | ZNF692 | 0.683224095 | 0.960111041 | 1.0330638 | down | mRNA |
| NM_001136040 | CPSF7 | 0.315608584 | 0.810346887 | 1.2561028 | down | mRNA |
| NM_001136041 | HDAC11 | 0.363805225 | 0.843238468 | 1.3895455 | down | mRNA |
| NM_001136044 | TMUB1 | 0.380011252 | 0.853273195 | 1.0795242 | down | mRNA |
| NM_001136051 | LRIG3 | 0.734518651 | 0.967777568 | 1.0282542 | down | mRNA |
| NM_001136052 | CPT1C | 0.045687693 | 0.39096051 | 1.3985784 | down | mRNA |
| NM_001136053 | TPRA1 | 0.251460918 | 0.751426128 | 1.0514063 | down | mRNA |
| NM_001136108 | R3HCC1 | 0.945802683 | 0.996710991 | 1.0040975 | down | mRNA |
| NM_001136115 | FBXO28 | 0.834848306 | 0.986575022 | 1.0200803 | down | mRNA |
| NM_001136116 | ZNF879 | 0.062230025 | 0.439379649 | 2.121311 | down | mRNA |
| NM_001136123 | FAM178A | 0.15070178 | 0.615769413 | 1.3629225 | down | mRNA |
| NM_001136125 | CCND3 | 0.739253982 | 0.969564098 | 1.0575685 | down | mRNA |
| NM_001136126 | CCND3 | 0.644234826 | 0.954657712 | 1.0912025 | down | mRNA |
| NM_001136130 | APP | 0.813828582 | 0.98120545 | 1.0263679 | down | mRNA |
| NM_001136135 | RPL28 | 0.968322185 | 0.99812049 | 1.0032907 | down | mRNA |
| NM_001136139 | TCF3 | 0.631939382 | 0.950683967 | 1.0654645 | down | mRNA |
| NM_001136140 | CMPK1 | 0.045673998 | 0.39096051 | 1.0736586 | down | mRNA |
| NM_001136152 | ALG1L2 | 0.109995743 | 0.549011353 | 1.212325 | down | mRNA |
| NM_001136153 | ATF6B | 0.548248472 | 0.92514258 | 1.103781 | down | mRNA |
| NM_001136154 | ERG | 0.697597135 | 0.962410025 | 1.1633112 | down | mRNA |
| NM_001136155 | ERG | 0.85903747 | 0.988512246 | 1.0736076 | down | mRNA |
| NM_001136180 | HSBP1L1 | 0.518139948 | 0.916125349 | 1.1727243 | down | mRNA |
| NM_001136191 | KANK2 | 0.243222407 | 0.741678336 | 1.3258363 | down | mRNA |
| NM_001136193 | FASTKD2 | 0.063617811 | 0.440919074 | 1.158916 | down | mRNA |
| NM_001136201 | ISOC2 | 0.002334074 | 0.114906521 | 1.2535316 | down | mRNA |
| NM_001136202 | ISOC2 | 0.033095931 | 0.346127643 | 1.1912735 | down | mRNA |
| NM_001136203 | CCDC124 | 0.684178708 | 0.960111041 | 1.0720896 | down | mRNA |
| NM_001136205 | KCTD1 | 0.086002205 | 0.498317708 | 1.348497 | down | mRNA |
| NM_001136223 | RCOR3 | 0.053512071 | 0.416064022 | 1.3338567 | down | mRNA |
| NM_001136224 | RCOR3 | 0.001309127 | 0.093126586 | 1.2731231 | down | mRNA |
| NM_001136225 | RCOR3 | 0.158799383 | 0.630361004 | 1.2927697 | down | mRNA |
| NM_001136234 | FAM48B1 | 0.598759699 | 0.940648177 | 1.046892 | down | mRNA |
| NM_001136262 | ATXN7L3B | 0.038206865 | 0.365047918 | 1.1826143 | down | mRNA |
| NM_001136264 | DAZAP2 | 0.678861084 | 0.95996398 | 1.033754 | down | mRNA |
| NM_001136266 | DAZAP2 | 0.675851765 | 0.959531882 | 1.0216885 | down | mRNA |
| NM_001136267 | DAZAP2 | 0.122743626 | 0.574983504 | 1.0926373 | down | mRNA |
| NM_001136478 | ANP32E | 0.53856052 | 0.923577434 | 1.1190481 | down | mRNA |
| NM_001136479 | ANP32E | 0.39155437 | 0.859202394 | 1.1145939 | down | mRNA |
| NM_001136493 | MFSD2A | 0.726826513 | 0.966191059 | 1.1025663 | down | mRNA |
| NM_001136497 | BTF3L4 | 0.182348819 | 0.665741817 | 1.0412922 | down | mRNA |
| NM_001136506 | SLC22A24 | 0.797840323 | 0.980749101 | 1.0411035 | down | mRNA |
| NM_001136509 | ZNF843 | 0.58086511 | 0.933994051 | 1.0564719 | down | mRNA |
| NM_001136538 | ACAD10 | 0.431342191 | 0.879996932 | 1.1252185 | down | mRNA |
| NM_001136543 | LYSMD1 | 0.196419204 | 0.684890285 | 1.3691147 | down | mRNA |
| NM_001136554 | TLK1 | 0.052919754 | 0.413038171 | 1.137806 | down | mRNA |
| NM_001136557 | GPR107 | 0.701851441 | 0.963247543 | 1.0844247 | down | mRNA |
| NM_001136574 | LANCL1 | 0.599741745 | 0.940648177 | 1.0608486 | down | mRNA |
| NM_001137550 | LRRFIP1 | 0.70942981 | 0.965462762 | 1.0374698 | down | mRNA |
| NM_001137553 | LRRFIP1 | 0.659584647 | 0.958354006 | 1.0690839 | down | mRNA |
| NM_001137554 | MCTS1 | 0.873310159 | 0.990580595 | 1.0196893 | down | mRNA |
| NM_001137559 | ANAPC5 | 0.195124205 | 0.682833964 | 1.1448099 | down | mRNA |
| NM_001137560 | TMEM151B | 0.713002934 | 0.966110889 | 1.0721778 | down | mRNA |
| NM_001137602 | MEMO1 | 0.8870605 | 0.993099104 | 1.0097386 | down | mRNA |
| NM_001137604 | POLR1B | 0.116988485 | 0.56398437 | 1.5357476 | down | mRNA |
| NM_001137610 | FAM86B2 | 0.294678455 | 0.791301949 | 1.0990089 | down | mRNA |
| NM_001137664 | ANAPC7 | 0.115936513 | 0.562046062 | 1.2050031 | down | mRNA |
| NM_001137667 | CASP8AP2 | 0.045082533 | 0.389222104 | 1.2826134 | down | mRNA |
| NM_001137674 | ZNF860 | 0.003954852 | 0.144623204 | 1.402479 | down | mRNA |
| NM_001139441 | BCAP31 | 0.359919081 | 0.841449472 | 1.1615348 | down | mRNA |
| NM_001139457 | BCAP31 | 0.544008736 | 0.923577434 | 1.0982664 | down | mRNA |
| NM_001139459 | CNST | 0.053093168 | 0.413914989 | 1.3027978 | down | mRNA |
| NM_001139490 | TRAF3IP1 | 0.072658801 | 0.463830063 | 1.1073503 | down | mRNA |
| NM_001139510 | ECHDC1 | 0.271990267 | 0.771957246 | 1.0900794 | down | mRNA |
| NM_001139517 | PRKRA | 0.607756551 | 0.942627675 | 1.0677312 | down | mRNA |
| NM_001139518 | PRKRA | 0.990561699 | 0.99935992 | 1.0015459 | down | mRNA |
| NM_001141945 | ACTA2 | 0.94451491 | 0.996710991 | 1.0670398 | down | mRNA |
| NM_001141947 | CCDC66 | 0.045665121 | 0.39096051 | 1.4568548 | down | mRNA |
| NM_001141974 | ATP13A2 | 0.08216981 | 0.489369308 | 1.2674308 | down | mRNA |
| NM_001141979 | TP53BP1 | 0.085893691 | 0.498103045 | 1.2122903 | down | mRNA |
| NM_001141980 | TP53BP1 | 0.061459785 | 0.437893235 | 1.5184399 | down | mRNA |
| NM_001142269 | GGCX | 0.501402191 | 0.910061537 | 1.0289161 | down | mRNA |
| NM_001142272 | RAB11FIP3 | 0.071575149 | 0.462000629 | 1.177334 | down | mRNA |
| NM_001142273 | CLASP1 | 0.494571953 | 0.906975462 | 1.1018512 | down | mRNA |
| NM_001142275 | NIPA1 | 0.661317359 | 0.958354006 | 1.0467784 | down | mRNA |
| NM_001142277 | APLP2 | 0.968286627 | 0.99812049 | 1.0027704 | down | mRNA |
| NM_001142278 | APLP2 | 0.977031458 | 0.998501532 | 1.0009934 | down | mRNA |
| NM_001142279 | RNASEH2B | 0.054663174 | 0.419400623 | 1.1130222 | down | mRNA |
| NM_001142281 | ARCN1 | 0.667527946 | 0.958354006 | 1.0177104 | down | mRNA |
| NM_001142285 | RPS24 | 0.149554395 | 0.61391883 | 1.3360198 | down | mRNA |
| NM_001142287 | SEMA4D | 0.84402271 | 0.987338076 | 1.0254305 | down | mRNA |
| NM_001142288 | EMC8 | 0.217369216 | 0.711339274 | 1.1327776 | down | mRNA |
| NM_001142290 | MGRN1 | 0.646718512 | 0.95550188 | 1.0729365 | down | mRNA |
| NM_001142292 | LMAN2L | 0.061876501 | 0.438960161 | 2.5513779 | down | mRNA |
| NM_001142296 | SPG20 | 0.499705341 | 0.909460484 | 1.0500413 | down | mRNA |
| NM_001142302 | CCDC113 | 0.469176052 | 0.897810636 | 1.0917708 | down | mRNA |
| NM_001142306 | C5orf60 | 0.812798417 | 0.98120545 | 1.0307861 | down | mRNA |
| NM_001142315 | LMO2 | 0.36454765 | 0.843907627 | 1.0861159 | down | mRNA |
| NM_001142319 | C2orf44 | 0.620089349 | 0.947444797 | 1.2105835 | down | mRNA |
| NM_001142325 | SLC18A1 | 0.051884677 | 0.410474414 | 1.3405955 | down | mRNA |
| NM_001142326 | DMTF1 | 0.133887587 | 0.590871682 | 1.2120111 | down | mRNA |
| NM_001142339 | GNAL | 0.164623422 | 0.637911838 | 1.2161066 | down | mRNA |
| NM_001142350 | THOC6 | 0.004834506 | 0.158753988 | 1.1769809 | down | mRNA |
| NM_001142353 | PPP3CB | 0.343849898 | 0.831629639 | 1.2021948 | down | mRNA |
| NM_001142354 | PPP3CB | 0.473238728 | 0.899184677 | 1.173434 | down | mRNA |
| NM_001142355 | NIF3L1 | 0.062981525 | 0.439746003 | 1.1928686 | down | mRNA |
| NM_001142356 | NIF3L1 | 0.162929159 | 0.635098861 | 1.1490233 | down | mRNA |
| NM_001142391 | SLC10A3 | 0.279080769 | 0.777816764 | 1.1014891 | down | mRNA |
| NM_001142392 | SLC10A3 | 0.000153366 | 0.044621794 | 1.6334891 | down | mRNA |
| NM_001142401 | CD164 | 0.343665253 | 0.831536584 | 1.0610928 | down | mRNA |
| NM_001142402 | CD164 | 0.472808436 | 0.899184677 | 1.09002 | down | mRNA |
| NM_001142403 | CD164 | 0.517560759 | 0.915525087 | 1.1314676 | down | mRNA |
| NM_001142404 | CD164 | 0.388999635 | 0.85800274 | 1.1667779 | down | mRNA |
| NM_001142413 | C9orf47 | 0.91134986 | 0.995342392 | 1.0493993 | down | mRNA |
| NM_001142414 | CRCP | 0.176136698 | 0.6551228 | 1.1808667 | down | mRNA |
| NM_001142426 | MORF4L2 | 0.50900592 | 0.911674633 | 1.0567004 | down | mRNA |
| NM_001142445 | ANK1 | 0.719074003 | 0.966191059 | 1.075984 | down | mRNA |
| NM_001142449 | SPNS1 | 0.029339458 | 0.333629679 | 1.1595834 | down | mRNA |
| NM_001142450 | SPNS1 | 0.022791177 | 0.297426317 | 1.2043628 | down | mRNA |
| NM_001142451 | SPNS1 | 0.03149288 | 0.341322176 | 1.1780773 | down | mRNA |
| NM_001142462 | OSR2 | 0.100038053 | 0.525710439 | 1.5376314 | down | mRNA |
| NM_001142464 | ECSIT | 0.491976416 | 0.905806259 | 1.036837 | down | mRNA |
| NM_001142465 | ECSIT | 0.890657691 | 0.9937521 | 1.004325 | down | mRNA |
| NM_001142466 | GPT2 | 0.09125639 | 0.50968981 | 1.3765019 | down | mRNA |
| NM_001142468 | C6orf203 | 0.003435145 | 0.137823993 | 1.4358253 | down | mRNA |
| NM_001142470 | C6orf203 | 0.014316453 | 0.241161654 | 1.307425 | down | mRNA |
| NM_001142475 | NREP | 0.56805108 | 0.931705674 | 1.2343496 | down | mRNA |
| NM_001142477 | NREP | 0.534257941 | 0.920652989 | 1.3232954 | down | mRNA |
| NM_001142497 | MLKL | 0.158835187 | 0.630380056 | 1.2037623 | down | mRNA |
| NM_001142500 | FLYWCH2 | 0.492720219 | 0.906047853 | 1.0833785 | down | mRNA |
| NM_001142501 | MON1A | 0.492599431 | 0.906047853 | 1.1238299 | down | mRNA |
| NM_001142503 | STARD8 | 0.715398649 | 0.966191059 | 1.0473824 | down | mRNA |
| NM_001142522 | FBXO5 | 0.279999581 | 0.778697234 | 1.2848747 | down | mRNA |
| NM_001142533 | SH2D3C | 0.844033805 | 0.987338076 | 1.0231514 | down | mRNA |
| NM_001142534 | SH2D3C | 0.052868246 | 0.413038171 | 1.791538 | down | mRNA |
| NM_001142535 | LGALS12 | 0.419758673 | 0.872365335 | 1.2588049 | down | mRNA |
| NM_001142536 | LGALS12 | 0.665706852 | 0.958354006 | 1.0726809 | down | mRNA |
| NM_001142546 | LUZP1 | 0.331756052 | 0.822255387 | 1.1165269 | down | mRNA |
| NM_001142547 | RPUSD3 | 0.205682907 | 0.696062726 | 1.1404159 | down | mRNA |
| NM_001142550 | WDR47 | 0.267565955 | 0.768382909 | 1.1478173 | down | mRNA |
| NM_001142551 | WDR47 | 0.249379672 | 0.747403815 | 1.1241083 | down | mRNA |
| NM_001142554 | TRMT1 | 0.001947121 | 0.107343066 | 1.22789 | down | mRNA |
| NM_001142560 | RRNAD1 | 0.632664226 | 0.951123179 | 1.0930797 | down | mRNA |
| NM_001142565 | CPSF7 | 0.876007286 | 0.991213905 | 1.0116385 | down | mRNA |
| NM_001142568 | BBX | 0.932924861 | 0.996710991 | 1.0183951 | down | mRNA |
| NM_001142569 | C1orf106 | 0.609962018 | 0.943169861 | 1.0495302 | down | mRNA |
| NM_001142571 | RAD51D | 0.123783935 | 0.577744894 | 1.3606845 | down | mRNA |
| NM_001142572 | ZNF669 | 0.110889422 | 0.551247853 | 1.0921251 | down | mRNA |
| NM_001142574 | IMPDH1 | 0.004227328 | 0.148390786 | 1.4918529 | down | mRNA |
| NM_001142575 | IMPDH1 | 0.025831886 | 0.315177872 | 1.2765854 | down | mRNA |
| NM_001142577 | ZNF780A | 0.604609392 | 0.94174007 | 1.0991583 | down | mRNA |
| NM_001142578 | ZNF780A | 0.001683167 | 0.10523567 | 1.5400273 | down | mRNA |
| NM_001142579 | ZNF780A | 0.262935196 | 0.763125731 | 1.3649277 | down | mRNA |
| NM_001142587 | NFYC | 0.561049177 | 0.928248992 | 1.0803487 | down | mRNA |
| NM_001142588 | NFYC | 0.346477895 | 0.833284666 | 1.0600852 | down | mRNA |
| NM_001142589 | NFYC | 0.899991531 | 0.994752372 | 1.0129353 | down | mRNA |
| NM_001142590 | NFYC | 0.097811562 | 0.520535504 | 1.1739947 | down | mRNA |
| NM_001142593 | ITPK1 | 0.020670927 | 0.286034385 | 1.1053115 | down | mRNA |
| NM_001142596 | P4HA1 | 0.407497204 | 0.867591329 | 1.0801255 | down | mRNA |
| NM_001142597 | CLP1 | 0.01087207 | 0.213674567 | 1.4260641 | down | mRNA |
| NM_001142603 | KIAA0141 | 0.39519813 | 0.861590143 | 1.1158171 | down | mRNA |
| NM_001142604 | PPT1 | 0.154199373 | 0.621674306 | 1.156561 | down | mRNA |
| NM_001142610 | ULK2 | 0.708307878 | 0.965462762 | 1.0774072 | down | mRNA |
| NM_001142615 | EHBP1 | 0.975471697 | 0.998501532 | 1.0088341 | down | mRNA |
| NM_001142620 | STRA6 | 0.610999423 | 0.943644684 | 1.1667777 | down | mRNA |
| NM_001142622 | GHDC | 0.577972686 | 0.933821303 | 1.0230829 | down | mRNA |
| NM_001142625 | RAB34 | 0.504936194 | 0.910568383 | 1.0295805 | down | mRNA |
| NM_001142627 | SEC61A2 | 0.689797924 | 0.961105466 | 1.0827047 | down | mRNA |
| NM_001142646 | TPRA1 | 0.697982054 | 0.962410025 | 1.0147751 | down | mRNA |
| NM_001142650 | HNRPLL | 0.059836289 | 0.434186803 | 1.8596411 | down | mRNA |
| NM_001142675 | CHID1 | 0.998635856 | 0.999889342 | 1.0002331 | down | mRNA |
| NM_001142676 | CHID1 | 0.792461222 | 0.978996327 | 1.0176436 | down | mRNA |
| NM_001142704 | FAM111B | 0.875715764 | 0.991145401 | 1.1096331 | down | mRNA |
| NM_001142725 | ERI2 | 0.006788883 | 0.183552613 | 1.35024 | down | mRNA |
| NM_001142726 | C1orf122 | 0.574516503 | 0.933656777 | 1.0338859 | down | mRNA |
| NM_001142730 | KCTD1 | 0.014888124 | 0.244907235 | 1.4490218 | down | mRNA |
| NM_001142761 | C15orf23 | 0.693062071 | 0.961584652 | 1.1273109 | down | mRNA |
| NM_001142762 | C15orf23 | 0.222720865 | 0.717565338 | 1.4160156 | down | mRNA |
| NM_001142782 | MAGI3 | 0.108794297 | 0.546082252 | 1.6565318 | down | mRNA |
| NM_001142797 | CXCR3 | 0.6550008 | 0.957205183 | 1.0309599 | down | mRNA |
| NM_001142806 | SLC6A8 | 0.408229217 | 0.867591329 | 1.0679632 | down | mRNA |
| NM_001142861 | SMAD6 | 0.996002256 | 0.999872237 | 1.0008211 | down | mRNA |
| NM_001142930 | API5 | 0.218290403 | 0.712269655 | 1.0466768 | down | mRNA |
| NM_001142931 | API5 | 0.743385132 | 0.970330526 | 1.0343707 | down | mRNA |
| NM_001142936 | DAGLB | 0.485832442 | 0.903250293 | 1.1223133 | down | mRNA |
| NM_001142958 | FBXO15 | 0.031824512 | 0.341995764 | 1.1434405 | down | mRNA |
| NM_001143667 | ZBED5 | 0.004148889 | 0.147175197 | 1.6638815 | down | mRNA |
| NM_001143678 | SGK1 | 0.000222559 | 0.049726123 | 2.0200417 | down | mRNA |
| NM_001143679 | GSTK1 | 0.317510899 | 0.811824899 | 1.0604271 | down | mRNA |
| NM_001143680 | GSTK1 | 0.723925654 | 0.966191059 | 1.0670867 | down | mRNA |
| NM_001143681 | GSTK1 | 0.822695335 | 0.983305175 | 1.0225608 | down | mRNA |
| NM_001143757 | LRRC27 | 0.067731303 | 0.451808679 | 1.0956587 | down | mRNA |
| NM_001143759 | LRRC27 | 0.604777417 | 0.94174007 | 1.0158356 | down | mRNA |
| NM_001143778 | ASAP3 | 0.234489041 | 0.731905307 | 1.193183 | down | mRNA |
| NM_001143781 | FKBP11 | 0.917310677 | 0.995349793 | 1.0135423 | down | mRNA |
| NM_001143783 | FES | 0.860757789 | 0.989013989 | 1.0929638 | down | mRNA |
| NM_001143785 | FES | 0.026257164 | 0.317396349 | 1.3330914 | down | mRNA |
| NM_001143787 | PPHLN1 | 0.452367277 | 0.888914959 | 1.1036103 | down | mRNA |
| NM_001143788 | PPHLN1 | 0.186594523 | 0.669953595 | 1.0787592 | down | mRNA |
| NM_001143810 | BDNF | 0.371038966 | 0.846402038 | 1.7777037 | down | mRNA |
| NM_001143811 | BDNF | 0.160414544 | 0.631980676 | 1.8507819 | down | mRNA |
| NM_001143826 | MAPRE2 | 0.158285642 | 0.628995854 | 1.1970567 | down | mRNA |
| NM_001143835 | NFRKB | 0.018991932 | 0.276409417 | 1.237115 | down | mRNA |
| NM_001143837 | NOX4 | 0.938452488 | 0.996710991 | 1.0055511 | down | mRNA |
| NM_001143838 | SLC13A5 | 0.642635037 | 0.95447352 | 1.0452287 | down | mRNA |
| NM_001143839 | PDE2A | 0.64466156 | 0.954657712 | 1.113521 | down | mRNA |
| NM_001143850 | TCTN2 | 0.26337534 | 0.763792241 | 1.0670593 | down | mRNA |
| NM_001143853 | WIBG | 0.110324553 | 0.549784024 | 1.2698278 | down | mRNA |
| NM_001143886 | PPP1R12A | 0.008271863 | 0.197905968 | 1.3590162 | down | mRNA |
| NM_001143887 | COPS2 | 0.011006818 | 0.215013404 | 1.2211592 | down | mRNA |
| NM_001143909 | LOC729020 | 0.298059574 | 0.794295449 | 1.2419112 | down | mRNA |
| NM_001143912 | FAM76A | 0.278872763 | 0.777783404 | 1.0746133 | down | mRNA |
| NM_001143914 | FAM76A | 0.000948876 | 0.084526081 | 1.3063437 | down | mRNA |
| NM_001143915 | FAM76A | 0.463927778 | 0.894989844 | 1.0692115 | down | mRNA |
| NM_001143917 | LYSMD2 | 0.06358047 | 0.440919074 | 1.2395228 | down | mRNA |
| NM_001143936 | C15orf61 | 0.300461809 | 0.795797471 | 1.1674892 | down | mRNA |
| NM_001143942 | RBM24 | 0.148020696 | 0.611080968 | 1.6165588 | down | mRNA |
| NM_001143944 | LEMD2 | 0.3401448 | 0.828242404 | 1.1464041 | down | mRNA |
| NM_001143945 | GAS6 | 0.581071463 | 0.934005566 | 1.1544764 | down | mRNA |
| NM_001143946 | GAS6 | 0.52535564 | 0.917686109 | 1.1506074 | down | mRNA |
| NM_001143958 | TMEM30A | 0.564589127 | 0.929793142 | 1.0495215 | down | mRNA |
| NM_001143963 | L1CAM | 0.567869014 | 0.931705674 | 1.0970327 | down | mRNA |
| NM_001143966 | TBC1D7 | 0.775393328 | 0.97589756 | 1.04189 | down | mRNA |
| NM_001143976 | WEE1 | 0.094496321 | 0.51550913 | 1.2490082 | down | mRNA |
| NM_001143980 | CCDC154 | 0.320239812 | 0.814552264 | 1.1266978 | down | mRNA |
| NM_001143981 | CHRDL1 | 0.894623526 | 0.993978977 | 1.0589591 | down | mRNA |
| NM_001143985 | BANF1 | 0.676251075 | 0.959531882 | 1.034174 | down | mRNA |
| NM_001143994 | RASSF7 | 0.06741048 | 0.451808679 | 1.1328788 | down | mRNA |
| NM_001143997 | OGDHL | 0.297681214 | 0.794023716 | 1.1529324 | down | mRNA |
| NM_001143999 | SEC14L1 | 0.984307609 | 0.998971807 | 1.0018244 | down | mRNA |
| NM_001144001 | SEC14L1 | 0.321445101 | 0.815220381 | 1.0911199 | down | mRNA |
| NM_001144012 | TMX2 | 0.614230514 | 0.944827645 | 1.0210768 | down | mRNA |
| NM_001144013 | RGPD3 | 0.005220433 | 0.165444121 | 1.2226298 | down | mRNA |
| NM_001144037 | TMEM25 | 0.312208664 | 0.80730339 | 1.1635785 | down | mRNA |
| NM_001144061 | COPB1 | 0.306952637 | 0.801927429 | 1.0799411 | down | mRNA |
| NM_001144070 | ABCC3 | 0.821607325 | 0.982815798 | 1.0283117 | down | mRNA |
| NM_001144072 | UBAC2 | 0.621975229 | 0.948047106 | 1.0447795 | down | mRNA |
| NM_001144073 | CHORDC1 | 0.520565788 | 0.916605148 | 1.0482295 | down | mRNA |
| NM_001144074 | DET1 | 0.105822 | 0.538296946 | 1.2389878 | down | mRNA |
| NM_001144755 | MYBL1 | 0.95902523 | 0.997335225 | 1.02481 | down | mRNA |
| NM_001144759 | PHLDB1 | 0.09297881 | 0.511788177 | 1.1456018 | down | mRNA |
| NM_001144824 | ZNF234 | 0.20337815 | 0.692876095 | 1.1494095 | down | mRNA |
| NM_001144825 | RUNDC3A | 0.66674862 | 0.958354006 | 1.0432633 | down | mRNA |
| NM_001144827 | RPUSD4 | 0.565844557 | 0.930458047 | 1.0321588 | down | mRNA |
| NM_001144831 | PHB2 | 0.35561096 | 0.838390774 | 1.0929487 | down | mRNA |
| NM_001144856 | PLEKHG6 | 0.516160705 | 0.914546356 | 1.1231778 | down | mRNA |
| NM_001144857 | PLEKHG6 | 0.382135096 | 0.853514109 | 1.3758617 | down | mRNA |
| NM_001144876 | DOK3 | 0.271322709 | 0.77133889 | 1.3200028 | down | mRNA |
| NM_001144877 | SCAI | 0.562415752 | 0.929041598 | 1.0961559 | down | mRNA |
| NM_001144879 | IMPA1 | 0.337120532 | 0.826919371 | 1.0827344 | down | mRNA |
| NM_001144886 | CITED1 | 0.787458526 | 0.977492986 | 1.0319727 | down | mRNA |
| NM_001144888 | BAIAP2 | 0.017792926 | 0.268173293 | 1.2614153 | down | mRNA |
| NM_001144891 | MBIP | 0.002983448 | 0.129891793 | 1.5140222 | down | mRNA |
| NM_001144894 | CD209 | 0.959790594 | 0.997693936 | 1.0086676 | down | mRNA |
| NM_001144900 | SMCR7 | 0.009517112 | 0.204959264 | 1.3573471 | down | mRNA |
| NM_001144912 | ATG7 | 0.401131485 | 0.864768873 | 1.0430856 | down | mRNA |
| NM_001144915 | FGFR2 | 0.134555247 | 0.591135973 | 1.1341154 | down | mRNA |
| NM_001144923 | TTC26 | 0.32060952 | 0.814552264 | 1.0366251 | down | mRNA |
| NM_001144924 | LOC81691 | 0.916129976 | 0.995349793 | 1.0308512 | down | mRNA |
| NM_001144927 | NKIRAS2 | 0.70591902 | 0.965020205 | 1.0697894 | down | mRNA |
| NM_001144928 | NKIRAS2 | 0.006155688 | 0.178714838 | 1.2535403 | down | mRNA |
| NM_001144929 | NKIRAS2 | 0.023823447 | 0.303389731 | 1.2081404 | down | mRNA |
| NM_001144931 | LOC391322 | 0.976538186 | 0.998501532 | 1.0096935 | down | mRNA |
| NM_001144935 | FGF1 | 0.285094294 | 0.781963593 | 1.4424587 | down | mRNA |
| NM_001144939 | VMO1 | 0.98384776 | 0.998971807 | 1.0045119 | down | mRNA |
| NM_001144942 | RAB34 | 0.264936929 | 0.76613535 | 1.0858539 | down | mRNA |
| NM_001144961 | NFKBIL1 | 0.913101921 | 0.995342392 | 1.0085304 | down | mRNA |
| NM_001144962 | NFKBIL1 | 0.071570291 | 0.462000629 | 1.1074808 | down | mRNA |
| NM_001144978 | MTHFD2L | 0.76720593 | 0.975033576 | 1.0440525 | down | mRNA |
| NM_001144983 | CCDC169 | 0.933683218 | 0.996710991 | 1.0220832 | down | mRNA |
| NM_001144995 | CCDC85C | 0.413951852 | 0.86961884 | 1.2161591 | down | mRNA |
| NM_001144997 | ITGA7 | 0.29304908 | 0.790364658 | 1.2358584 | down | mRNA |
| NM_001145008 | BTN3A1 | 0.89537927 | 0.993978977 | 1.0245599 | down | mRNA |
| NM_001145023 | SCRN2 | 0.001111605 | 0.087009974 | 2.0417711 | down | mRNA |
| NM_001145036 | COG2 | 0.030506314 | 0.338388833 | 1.1964154 | down | mRNA |
| NM_001145048 | GPN1 | 0.059141719 | 0.432387428 | 1.1829884 | down | mRNA |
| NM_001145049 | GPN1 | 0.141611312 | 0.601858131 | 1.0512634 | down | mRNA |
| NM_001145051 | TMEM247 | 0.165732605 | 0.639669852 | 1.9479536 | down | mRNA |
| NM_001145055 | IL18BP | 0.26157834 | 0.762395628 | 1.560855 | down | mRNA |
| NM_001145093 | ZNF619 | 0.069864186 | 0.457827646 | 1.4150073 | down | mRNA |
| NM_001145098 | SKIL | 0.20480577 | 0.694829118 | 1.1420459 | down | mRNA |
| NM_001145103 | SMAD3 | 0.820869367 | 0.982756843 | 1.0709215 | down | mRNA |
| NM_001145107 | NELL2 | 0.234937065 | 0.732404233 | 1.260587 | down | mRNA |
| NM_001145109 | NELL2 | 0.08142399 | 0.487632566 | 1.4982384 | down | mRNA |
| NM_001145112 | PATL2 | 0.247003975 | 0.745152522 | 1.1362858 | down | mRNA |
| NM_001145114 | RRP12 | 0.865597905 | 0.989401142 | 1.0099025 | down | mRNA |
| NM_001145118 | GRID2IP | 0.034769739 | 0.352074963 | 1.5698349 | down | mRNA |
| NM_001145134 | CPT1B | 0.185045798 | 0.668387133 | 1.078353 | down | mRNA |
| NM_001145137 | CPT1B | 0.094675748 | 0.516031114 | 1.3190877 | down | mRNA |
| NM_001145149 | VAMP7 | 0.837756379 | 0.986863424 | 1.0240903 | down | mRNA |
| NM_001145152 | VPS37A | 0.86743527 | 0.989481868 | 1.0196523 | down | mRNA |
| NM_001145157 | NR2F2 | 0.249215057 | 0.747319103 | 1.3581991 | down | mRNA |
| NM_001145158 | HBS1L | 0.557815951 | 0.926833616 | 1.0160689 | down | mRNA |
| NM_001145161 | UBE2QL1 | 0.18382528 | 0.667497719 | 1.2301592 | down | mRNA |
| NM_001145166 | PRMT3 | 0.086489327 | 0.49936346 | 1.1980552 | down | mRNA |
| NM_001145169 | GPR113 | 0.020705557 | 0.286034385 | 1.5728518 | down | mRNA |
| NM_001145191 | FAM200B | 0.093031364 | 0.511788177 | 1.434934 | down | mRNA |
| NM_001145206 | KIAA1671 | 0.800758705 | 0.98118927 | 1.1313761 | down | mRNA |
| NM_001145208 | DEPDC1B | 0.558795638 | 0.927153474 | 1.401974 | down | mRNA |
| NM_001145211 | SLCO2B1 | 0.834176366 | 0.986475536 | 1.0284954 | down | mRNA |
| NM_001145248 | FAM157A | 0.249226486 | 0.747319103 | 1.1155001 | down | mRNA |
| NM_001145255 | IKBKG | 0.976934126 | 0.998501532 | 1.0039513 | down | mRNA |
| NM_001145263 | NCOA4 | 0.177369323 | 0.65723949 | 1.1278145 | down | mRNA |
| NM_001145264 | LAMTOR2 | 0.49998042 | 0.909550837 | 1.0449491 | down | mRNA |
| NM_001145265 | SLC35C1 | 0.286293634 | 0.782895119 | 1.6847985 | down | mRNA |
| NM_001145268 | FAM185A | 0.031520613 | 0.341436922 | 1.2132737 | down | mRNA |
| NM_001145269 | FAM185A | 0.654648679 | 0.957205183 | 1.0331512 | down | mRNA |
| NM_001145277 | NECAP2 | 0.613398373 | 0.94460469 | 1.048438 | down | mRNA |
| NM_001145278 | NECAP2 | 0.890668474 | 0.9937521 | 1.0189014 | down | mRNA |
| NM_001145285 | OPRM1 | 0.471923125 | 0.899010679 | 1.0637226 | down | mRNA |
| NM_001145286 | OPRM1 | 0.962987323 | 0.99793927 | 1.0128101 | down | mRNA |
| NM_001145293 | NGLY1 | 0.031346962 | 0.341322176 | 1.1805434 | down | mRNA |
| NM_001145294 | NGLY1 | 0.983889443 | 0.998971807 | 1.0032495 | down | mRNA |
| NM_001145295 | NGLY1 | 0.054741474 | 0.419413793 | 1.1528226 | down | mRNA |
| NM_001145297 | EXOC7 | 0.834665225 | 0.986575022 | 1.0114951 | down | mRNA |
| NM_001145298 | EXOC7 | 0.665244769 | 0.958354006 | 1.0697249 | down | mRNA |
| NM_001145299 | EXOC7 | 0.734606612 | 0.967777568 | 1.0132499 | down | mRNA |
| NM_001145302 | RARA | 0.39175203 | 0.859419753 | 1.218739 | down | mRNA |
| NM_001145303 | TMC4 | 0.540241823 | 0.923577434 | 1.0624029 | down | mRNA |
| NM_001145305 | KIAA1683 | 0.896144086 | 0.99418353 | 1.0125496 | down | mRNA |
| NM_001145307 | LRTOMT | 0.376330776 | 0.849903337 | 1.0380574 | down | mRNA |
| NM_001145317 | DSN1 | 0.350767811 | 0.834911159 | 1.287992 | down | mRNA |
| NM_001145335 | UBE2Q2 | 0.013784958 | 0.236719404 | 1.1714719 | down | mRNA |
| NM_001145344 | ZNF566 | 0.995446375 | 0.999872237 | 1.0034025 | down | mRNA |
| NM_001145355 | CBWD1 | 0.41418837 | 0.86961884 | 1.15865 | down | mRNA |
| NM_001145356 | CBWD1 | 0.502486391 | 0.910335149 | 1.136968 | down | mRNA |
| NM_001145364 | ABHD11 | 0.71484223 | 0.966110889 | 1.073092 | down | mRNA |
| NM_001145373 | OTUD1 | 0.895456037 | 0.993978977 | 1.0155793 | down | mRNA |
| NM_001145375 | ALKBH2 | 0.018110833 | 0.269173581 | 1.2886765 | down | mRNA |
| NM_001145391 | OXSM | 0.00017825 | 0.044742906 | 1.7668357 | down | mRNA |
| NM_001145392 | TSEN2 | 0.432518464 | 0.880491224 | 1.0573932 | down | mRNA |
| NM_001145394 | TSEN2 | 0.040524687 | 0.373164825 | 1.6227343 | down | mRNA |
| NM_001145396 | ALDH16A1 | 0.659388693 | 0.958354006 | 1.0829581 | down | mRNA |
| NM_001145404 | ZNF506 | 0.002694608 | 0.122795128 | 1.3970823 | down | mRNA |
| NM_001145410 | NONO | 0.218439773 | 0.712320363 | 1.0834889 | down | mRNA |
| NM_001145411 | CCDC110 | 0.066285702 | 0.449693785 | 1.5122162 | down | mRNA |
| NM_001145414 | CHM | 0.950548563 | 0.996710991 | 1.0091666 | down | mRNA |
| NM_001145415 | SETDB1 | 0.713946033 | 0.966110889 | 1.0258407 | down | mRNA |
| NM_001145417 | MSL2 | 0.703125416 | 0.963625105 | 1.0522522 | down | mRNA |
| NM_001145418 | TTC28 | 0.575964551 | 0.933656777 | 1.0730218 | down | mRNA |
| NM_001145426 | CSDA | 0.69206902 | 0.961451327 | 1.0130069 | down | mRNA |
| NM_001145427 | SNX18 | 0.105744459 | 0.538171802 | 1.3025923 | down | mRNA |
| NM_001145432 | C4orf52 | 0.181349479 | 0.664839093 | 1.1422906 | down | mRNA |
| NM_001145434 | ZNF880 | 0.294890718 | 0.791301949 | 1.1772482 | down | mRNA |
| NM_001145438 | PGAP2 | 0.565908136 | 0.930458047 | 1.085118 | down | mRNA |
| NM_001145441 | TYW1B | 0.209048454 | 0.700221058 | 1.3362861 | down | mRNA |
| NM_001145442 | POTEM | 0.48766591 | 0.903757389 | 1.1008671 | down | mRNA |
| NM_001145443 | PFKFB3 | 0.297943462 | 0.794295449 | 1.0936498 | down | mRNA |
| NM_001145445 | SCAF4 | 0.90723168 | 0.995145546 | 1.0109534 | down | mRNA |
| NM_001145447 | ZNF200 | 0.176473409 | 0.655832089 | 1.2494924 | down | mRNA |
| NM_001145454 | STMN1 | 0.78208717 | 0.976984292 | 1.0745134 | down | mRNA |
| NM_001145462 | YIF1B | 0.692747684 | 0.961584652 | 1.1076922 | down | mRNA |
| NM_001145464 | EXOG | 0.0193855 | 0.280114993 | 1.4317646 | down | mRNA |
| NM_001145474 | ATPAF1-AS1 | 0.721529926 | 0.966191059 | 1.0759787 | down | mRNA |
| NM_001145515 | SCRN1 | 0.508376409 | 0.911674633 | 1.1970495 | down | mRNA |
| NM_001145524 | YPEL3 | 0.747712353 | 0.970660679 | 1.0465774 | down | mRNA |
| NM_001145531 | C7orf71 | 0.792667915 | 0.979003983 | 1.0380931 | down | mRNA |
| NM_001145536 | C17orf107 | 0.130066518 | 0.584037049 | 1.1262067 | down | mRNA |
| NM_001145548 | ZDHHC7 | 0.184779094 | 0.668017166 | 1.2504716 | down | mRNA |
| NM_001145549 | TXNDC5 | 0.343375224 | 0.831536584 | 1.1897708 | down | mRNA |
| NM_001145641 | SRRM5 | 0.104796687 | 0.535896943 | 1.1917917 | down | mRNA |
| NM_001145645 | TNFSF13B | 0.670925291 | 0.959531882 | 1.0166517 | down | mRNA |
| NM_001145646 | APH1B | 0.932074679 | 0.996710991 | 1.0153542 | down | mRNA |
| NM_001145659 | CTAGE9 | 0.117518167 | 0.564147103 | 1.1545794 | down | mRNA |
| NM_001145664 | RFX8 | 0.663726255 | 0.958354006 | 1.0634209 | down | mRNA |
| NM_001145665 | ZNF181 | 0.108534688 | 0.545544443 | 1.1644218 | down | mRNA |
| NM_001145667 | GLG1 | 0.823743985 | 0.983568283 | 1.0529384 | down | mRNA |
| NM_001145713 | SAE1 | 0.040994372 | 0.375308608 | 1.1731899 | down | mRNA |
| NM_001145714 | SAE1 | 0.96742188 | 0.99793927 | 1.0021244 | down | mRNA |
| NM_001145725 | LYAR | 0.865280754 | 0.989401142 | 1.0112451 | down | mRNA |
| NM_001145769 | XPC | 0.441328987 | 0.883920737 | 1.1833468 | down | mRNA |
| NM_001145784 | MEF2BNB | 0.927110632 | 0.995829678 | 1.0108795 | down | mRNA |
| NM_001145797 | SH2B1 | 0.162864318 | 0.635098861 | 1.237167 | down | mRNA |
| NM_001145798 | CACNB4 | 0.231898257 | 0.729717132 | 1.105606 | down | mRNA |
| NM_001145809 | MYH14 | 0.420207434 | 0.872365335 | 1.0523479 | down | mRNA |
| NM_001145815 | AMDHD2 | 0.767526988 | 0.975033576 | 1.0259031 | down | mRNA |
| NM_001145838 | MTFR1 | 0.060639453 | 0.435971562 | 1.3590818 | down | mRNA |
| NM_001145901 | SARS2 | 0.969836351 | 0.998245244 | 1.0058162 | down | mRNA |
| NM_001145923 | SCAPER | 0.519444604 | 0.916145597 | 1.1345305 | down | mRNA |
| NM_001145934 | TKTL1 | 0.439233017 | 0.882895421 | 1.0512261 | down | mRNA |
| NM_001145963 | SLC12A4 | 0.258143788 | 0.758903917 | 1.0822134 | down | mRNA |
| NM_001145964 | SLC12A4 | 0.288696519 | 0.784624733 | 1.0667682 | down | mRNA |
| NM_001145966 | MKI67 | 0.737073721 | 0.968834005 | 1.2077213 | down | mRNA |
| NM_001145998 | SLC15A2 | 0.554912008 | 0.926833616 | 1.1634361 | down | mRNA |
| NM_001146003 | NEK11 | 0.921783218 | 0.995349793 | 1.0206061 | down | mRNA |
| NM_001146006 | IGFALS | 0.535774422 | 0.921994545 | 1.0648235 | down | mRNA |
| NM_001146015 | DLGAP5 | 0.80777722 | 0.98118927 | 1.0737266 | down | mRNA |
| NM_001146029 | SEMA7A | 0.326032711 | 0.818786695 | 1.4851949 | down | mRNA |
| NM_001146030 | SEMA7A | 0.482083334 | 0.902092906 | 1.2907514 | down | mRNA |
| NM_001146041 | KRTAP4-9 | 0.223261103 | 0.71761506 | 1.3765727 | down | mRNA |
| NM_001146051 | HSDL1 | 0.160864625 | 0.63226359 | 1.3692233 | down | mRNA |
| NM_001146056 | MBOAT7 | 0.459820809 | 0.893024805 | 1.0503331 | down | mRNA |
| NM_001146068 | CAPN2 | 0.427580655 | 0.876992614 | 1.1395747 | down | mRNA |
| NM_001146070 | TDRD3 | 0.173807431 | 0.652002339 | 1.1266258 | down | mRNA |
| NM_001146071 | TDRD3 | 0.734184669 | 0.967777568 | 1.0565659 | down | mRNA |
| NM_001146075 | RPAP3 | 0.013073162 | 0.233776196 | 1.2510129 | down | mRNA |
| NM_001146076 | RPAP3 | 0.008303797 | 0.198160569 | 1.2686443 | down | mRNA |
| NM_001146099 | NEK3 | 0.109178203 | 0.547155834 | 1.1406092 | down | mRNA |
| NM_001146102 | PARP9 | 0.931037268 | 0.996710991 | 1.0084258 | down | mRNA |
| NM_001146106 | PARP9 | 0.526062658 | 0.917975454 | 1.1113013 | down | mRNA |
| NM_001146114 | DIP2A | 0.121606303 | 0.573155624 | 1.1561696 | down | mRNA |
| NM_001146115 | DIP2A | 0.007578349 | 0.193257016 | 1.1651336 | down | mRNA |
| NM_001146116 | DIP2A | 0.336481185 | 0.826506012 | 1.2245154 | down | mRNA |
| NM_001146151 | AVPR2 | 0.365376432 | 0.844953778 | 1.3400255 | down | mRNA |
| NM_001146152 | CYP51A1 | 0.482932543 | 0.902654749 | 1.1335961 | down | mRNA |
| NM_001146158 | MOGS | 0.328649602 | 0.821614764 | 1.1721358 | down | mRNA |
| NM_001146160 | TATDN1 | 0.4325372 | 0.880491224 | 1.1062036 | down | mRNA |
| NM_001146169 | TATDN3 | 0.562921392 | 0.929041598 | 1.0559199 | down | mRNA |
| NM_001146175 | ZNF414 | 0.382894489 | 0.853514109 | 1.0922145 | down | mRNA |
| NM_001146192 | ZMYND12 | 0.988368296 | 0.999140676 | 1.0076496 | down | mRNA |
| NM_001146209 | PDE2A | 0.477684838 | 0.900870802 | 1.1639171 | down | mRNA |
| NM_001146213 | TBC1D15 | 0.117558062 | 0.564147103 | 1.1721727 | down | mRNA |
| NM_001146226 | KRT72 | 0.490868972 | 0.90553335 | 1.132675 | down | mRNA |
| NM_001146258 | PIP4K2C | 0.628466872 | 0.949961866 | 1.0447356 | down | mRNA |
| NM_001146262 | SYT14 | 0.627326028 | 0.949299851 | 1.1209512 | down | mRNA |
| NM_001146265 | GPR85 | 0.974247009 | 0.998501532 | 1.0271987 | down | mRNA |
| NM_001146274 | TCF7L2 | 0.152594202 | 0.618255775 | 1.3807666 | down | mRNA |
| NM_001146277 | NCEH1 | 0.898870904 | 0.99453218 | 1.0477039 | down | mRNA |
| NM_001146280 | SHBG | 0.536089518 | 0.922302595 | 1.0355338 | down | mRNA |
| NM_001146282 | SRP54 | 0.284257691 | 0.78179061 | 1.1439915 | down | mRNA |
| NM_001146283 | TCF7L2 | 0.198350618 | 0.686799061 | 1.156428 | down | mRNA |
| NM_001146288 | AIFM3 | 0.954327399 | 0.996710991 | 1.0107716 | down | mRNA |
| NM_001146291 | ZNF674 | 0.016058109 | 0.25477722 | 1.3973596 | down | mRNA |
| NM_001146312 | MYOCD | 0.852333099 | 0.987678166 | 1.1054379 | down | mRNA |
| NM_001146316 | SPSB2 | 0.004767044 | 0.158085585 | 1.3503131 | down | mRNA |
| NM_001146326 | ZIM2 | 0.653129568 | 0.956860606 | 1.0640255 | down | mRNA |
| NM_001146333 | SUMF2 | 0.531797634 | 0.920228311 | 1.1377562 | down | mRNA |
| NM_001146337 | AFAP1L1 | 0.272303256 | 0.772141487 | 1.202187 | down | mRNA |
| NM_001146343 | PQLC1 | 0.911673219 | 0.995342392 | 1.0131633 | down | mRNA |
| NM_001146627 | AGXT2L1 | 0.019709084 | 0.282421182 | 1.5020706 | down | mRNA |
| NM_001146694 | KDM4C | 0.624207644 | 0.948341211 | 1.1143548 | down | mRNA |
| NM_001146729 | HRASLS5 | 0.937773726 | 0.996710991 | 1.0069957 | down | mRNA |
| NM_001150 | ANPEP | 0.499338206 | 0.909361611 | 1.0600092 | down | mRNA |
| NM_001152 | SLC25A5 | 0.877753829 | 0.991306146 | 1.0112654 | down | mRNA |
| NM_001153 | ANXA4 | 0.670121161 | 0.959531882 | 1.0464106 | down | mRNA |
| NM_001153484 | SCOC | 0.450708647 | 0.888732431 | 1.1927334 | down | mRNA |
| NM_001153552 | SCOC | 0.14450798 | 0.605676409 | 1.0607402 | down | mRNA |
| NM_001154 | ANXA5 | 0.875330267 | 0.991094934 | 1.0143468 | down | mRNA |
| NM_001156 | ANXA7 | 0.569403673 | 0.932435203 | 1.0290461 | down | mRNA |
| NM_001159291 | MICAL1 | 0.337907481 | 0.827394958 | 1.1019519 | down | mRNA |
| NM_001159293 | ZNF737 | 0.338497019 | 0.827403747 | 1.3131537 | down | mRNA |
| NM_001159390 | GUK1 | 0.814770465 | 0.981277402 | 1.0234043 | down | mRNA |
| NM_001159542 | POU5F1B | 0.976854656 | 0.998501532 | 1.0042808 | down | mRNA |
| NM_001159554 | P2RX6 | 0.231626666 | 0.729296323 | 1.2791724 | down | mRNA |
| NM_001159560 | BEX5 | 0.874062904 | 0.990793509 | 1.0449567 | down | mRNA |
| NM_001159575 | SCNN1A | 0.163653002 | 0.636236851 | 1.3708988 | down | mRNA |
| NM_001159587 | CD109 | 0.335220334 | 0.825045374 | 1.0759855 | down | mRNA |
| NM_001159588 | CD109 | 0.738393614 | 0.96939491 | 1.0254642 | down | mRNA |
| NM_001159596 | BIVM | 0.241401037 | 0.739627167 | 1.2791641 | down | mRNA |
| NM_001159597 | SH3YL1 | 0.063648312 | 0.440919074 | 1.1612568 | down | mRNA |
| NM_001159651 | BOD1 | 0.25655816 | 0.757240077 | 1.1124828 | down | mRNA |
| NM_001159677 | SYNCRIP | 0.040190121 | 0.371915487 | 1.1314287 | down | mRNA |
| NM_001159699 | FHL1 | 0.303383315 | 0.798807246 | 1.2318379 | down | mRNA |
| NM_001159708 | BTNL8 | 0.665047694 | 0.958354006 | 1.1643753 | down | mRNA |
| NM_001159736 | BUD13 | 0.054608052 | 0.41929415 | 1.3994283 | down | mRNA |
| NM_001159746 | ABR | 0.43178191 | 0.880121832 | 1.036583 | down | mRNA |
| NM_001159749 | RIOK2 | 0.004311555 | 0.150034965 | 1.4383233 | down | mRNA |
| NM_001159846 | MPND | 0.328886161 | 0.821690024 | 1.0919165 | down | mRNA |
| NM_001160036 | RHOBTB2 | 0.356908254 | 0.839423386 | 1.3029888 | down | mRNA |
| NM_001160037 | RHOBTB2 | 0.286055923 | 0.782746332 | 1.2199742 | down | mRNA |
| NM_001160045 | GLI1 | 0.171950194 | 0.649834644 | 1.3149552 | down | mRNA |
| NM_001160094 | ACOT13 | 0.547919085 | 0.92514258 | 1.0156155 | down | mRNA |
| NM_001160115 | C15orf40 | 0.785263072 | 0.977411899 | 1.0124044 | down | mRNA |
| NM_001160116 | C15orf40 | 0.761290857 | 0.973219662 | 1.0346059 | down | mRNA |
| NM_001160130 | KCNQ5 | 0.897459321 | 0.99428634 | 1.0205622 | down | mRNA |
| NM_001160132 | KCNQ5 | 0.696700847 | 0.962410025 | 1.0721591 | down | mRNA |
| NM_001160138 | C7orf63 | 0.677902848 | 0.959531882 | 1.0986087 | down | mRNA |
| NM_001160147 | DDHD1 | 0.000966746 | 0.085290119 | 1.3315603 | down | mRNA |
| NM_001160148 | DDHD1 | 0.008770379 | 0.202329498 | 1.5001404 | down | mRNA |
| NM_001160169 | PRR5L | 0.768395605 | 0.975142015 | 1.0335095 | down | mRNA |
| NM_001160174 | NAT1 | 0.305867065 | 0.801497191 | 2.467664 | down | mRNA |
| NM_001160176 | NAT1 | 0.167529726 | 0.642346587 | 1.3819161 | down | mRNA |
| NM_001160210 | SLC25A13 | 0.625378038 | 0.948832915 | 1.2005746 | down | mRNA |
| NM_001160223 | RNF170 | 0.36204214 | 0.842995969 | 1.1128585 | down | mRNA |
| NM_001160225 | RNF170 | 0.499288483 | 0.909361611 | 1.0746198 | down | mRNA |
| NM_001160227 | SPG11 | 0.163838667 | 0.63628397 | 1.2404903 | down | mRNA |
| NM_001160234 | ATP1A1 | 0.232774404 | 0.730816736 | 1.1313728 | down | mRNA |
| NM_001160300 | PANX2 | 0.701298896 | 0.963015537 | 1.0708989 | down | mRNA |
| NM_001160308 | SETDB2 | 0.049184222 | 0.402199077 | 1.6138696 | down | mRNA |
| NM_001160329 | SYT3 | 0.185720987 | 0.669321303 | 1.3096088 | down | mRNA |
| NM_001160332 | NFASC | 0.637469299 | 0.952666356 | 1.0516167 | down | mRNA |
| NM_001160354 | LY6K | 0.972515675 | 0.998501532 | 1.0015198 | down | mRNA |
| NM_001160364 | TMC7 | 0.27431878 | 0.774430636 | 1.6542434 | down | mRNA |
| NM_001160389 | TRPT1 | 0.876181922 | 0.991213905 | 1.0061704 | down | mRNA |
| NM_001160407 | TLCD1 | 0.701326532 | 0.963015537 | 1.1062782 | down | mRNA |
| NM_001160408 | TULP3 | 0.267409887 | 0.768151713 | 1.0911319 | down | mRNA |
| NM_001160708 | POLR3B | 0.028463483 | 0.327889776 | 1.4719123 | down | mRNA |
| NM_001161009 | ARMC10 | 0.675829427 | 0.959531882 | 1.0303368 | down | mRNA |
| NM_001161010 | ARMC10 | 0.365987773 | 0.845164582 | 1.0836441 | down | mRNA |
| NM_001161011 | ARMC10 | 0.345317701 | 0.832533915 | 1.1636367 | down | mRNA |
| NM_001161012 | ARMC10 | 0.572508905 | 0.933460721 | 1.0559213 | down | mRNA |
| NM_001161013 | ARMC10 | 0.287131848 | 0.782954059 | 1.1464789 | down | mRNA |
| NM_001161331 | SSH1 | 0.952189609 | 0.996710991 | 1.0086349 | down | mRNA |
| NM_001161344 | CHFR | 0.684150014 | 0.960111041 | 1.0593946 | down | mRNA |
| NM_001161353 | KCNMA1 | 0.99837082 | 0.999889342 | 1.0004649 | down | mRNA |
| NM_001161404 | LIMS2 | 0.216839673 | 0.711074992 | 1.152766 | down | mRNA |
| NM_001161427 | ZNF610 | 0.144123337 | 0.605062089 | 1.3939404 | down | mRNA |
| NM_001161452 | CYBASC3 | 0.734155924 | 0.967777568 | 1.0788908 | down | mRNA |
| NM_001161521 | KLHL2 | 0.207807242 | 0.698556218 | 1.2511673 | down | mRNA |
| NM_001161532 | CSF2RA | 0.913865382 | 0.995342392 | 1.024162 | down | mRNA |
| NM_001161580 | POC1A | 0.783365111 | 0.977275912 | 1.1263447 | down | mRNA |
| NM_001161581 | POC1A | 0.902686684 | 0.99508468 | 1.0061683 | down | mRNA |
| NM_001161616 | RGL3 | 0.883358513 | 0.992766819 | 1.0256655 | down | mRNA |
| NM_001161619 | DUS3L | 0.952905503 | 0.996710991 | 1.0082683 | down | mRNA |
| NM_001161766 | AHCY | 0.392758587 | 0.859694706 | 1.1863541 | down | mRNA |
| NM_001161772 | HTR3A | 0.892143995 | 0.993812827 | 1.0291126 | down | mRNA |
| NM_001162427 | TSC1 | 0.005773186 | 0.172364776 | 1.7603168 | down | mRNA |
| NM_001162429 | PDCD6IP | 0.092591582 | 0.511338764 | 1.1496177 | down | mRNA |
| NM_001162505 | TMEM189 | 0.220771681 | 0.716020069 | 1.0375661 | down | mRNA |
| NM_001162529 | FAM135A | 0.277125263 | 0.777205298 | 1.3030581 | down | mRNA |
| NM_001162952 | SYTL2 | 0.75595165 | 0.971522741 | 1.1103725 | down | mRNA |
| NM_001162995 | C17orf109 | 0.214747803 | 0.707887861 | 1.2676665 | down | mRNA |
| NM_001163022 | PLK1S1 | 0.651765729 | 0.956204521 | 1.1193283 | down | mRNA |
| NM_001163023 | PLK1S1 | 0.044675115 | 0.388177111 | 1.2836429 | down | mRNA |
| NM_001163121 | RDM1 | 0.010878982 | 0.213674567 | 1.5369306 | down | mRNA |
| NM_001163130 | RDM1 | 0.030999851 | 0.340185047 | 1.6588263 | down | mRNA |
| NM_001163152 | ETV1 | 0.767612038 | 0.975033576 | 1.0508295 | down | mRNA |
| NM_001163280 | HTATSF1 | 0.381973981 | 0.853514109 | 1.0612405 | down | mRNA |
| NM_001163315 | FBXL17 | 0.935977835 | 0.996710991 | 1.0177835 | down | mRNA |
| NM_001163321 | CCDC120 | 0.77014727 | 0.975286259 | 1.0166467 | down | mRNA |
| NM_001163323 | CCDC120 | 0.841212504 | 0.987068842 | 1.0110286 | down | mRNA |
| NM_001163391 | ZSCAN12 | 0.084034886 | 0.493254641 | 1.5048734 | down | mRNA |
| NM_001163417 | FAM172A | 0.185449759 | 0.669014284 | 1.0474142 | down | mRNA |
| NM_001163437 | TBCK | 0.68747817 | 0.960205549 | 1.1254307 | down | mRNA |
| NM_001163484 | DCAF11 | 0.508325143 | 0.911674633 | 1.0947363 | down | mRNA |
| NM_001163547 | SYNRG | 0.006000737 | 0.176785634 | 1.54658 | down | mRNA |
| NM_001163677 | KCNMB3 | 0.545056945 | 0.924012779 | 1.1307326 | down | mRNA |
| NM_001163692 | UBAP1L | 0.349019115 | 0.834138149 | 1.0736012 | down | mRNA |
| NM_001163735 | MYO19 | 0.92750913 | 0.995829678 | 1.0185438 | down | mRNA |
| NM_001163771 | COL11A2 | 0.349159246 | 0.834138149 | 1.4003509 | down | mRNA |
| NM_001163788 | PTBP3 | 0.412981981 | 0.86947899 | 1.0753926 | down | mRNA |
| NM_001163790 | PTBP3 | 0.891635116 | 0.9937521 | 1.0038233 | down | mRNA |
| NM_001163809 | WDR81 | 0.601029502 | 0.941023551 | 1.1125361 | down | mRNA |
| NM_001163811 | WDR81 | 0.658707007 | 0.958354006 | 1.0853768 | down | mRNA |
| NM_001163922 | VSIG10L | 0.781927478 | 0.976963215 | 1.055532 | down | mRNA |
| NM_001163940 | PYGL | 0.165765806 | 0.639669852 | 1.474429 | down | mRNA |
| NM_001163941 | ABCB5 | 0.725840486 | 0.966191059 | 1.1027477 | down | mRNA |
| NM_001163989 | RAB37 | 0.681664988 | 0.960111041 | 1.0458329 | down | mRNA |
| NM_001164145 | CHAMP1 | 0.002103976 | 0.110028846 | 1.9911523 | down | mRNA |
| NM_001164160 | PPP6R3 | 0.239135578 | 0.737241028 | 1.0854609 | down | mRNA |
| NM_001164161 | PPP6R3 | 0.240190506 | 0.738437735 | 1.1218951 | down | mRNA |
| NM_001164162 | PPP6R3 | 0.167596685 | 0.642346587 | 1.1358948 | down | mRNA |
| NM_001164163 | PPP6R3 | 0.098692997 | 0.52242281 | 1.1729803 | down | mRNA |
| NM_001164164 | PPP6R3 | 0.283730052 | 0.781574233 | 1.1186457 | down | mRNA |
| NM_001164213 | LRCH1 | 0.632061713 | 0.950683967 | 1.0412528 | down | mRNA |
| NM_001164245 | C1orf27 | 0.657054825 | 0.957445617 | 1.0239009 | down | mRNA |
| NM_001164246 | C1orf27 | 0.148137013 | 0.611080968 | 1.1853117 | down | mRNA |
| NM_001164257 | C17orf72 | 0.709717151 | 0.965462762 | 1.0256793 | down | mRNA |
| NM_001164269 | RAD51 | 0.940338887 | 0.996710991 | 1.0395436 | down | mRNA |
| NM_001164270 | RAD51 | 0.648441798 | 0.95550188 | 1.3046402 | down | mRNA |
| NM_001164271 | DLC1 | 0.049218407 | 0.402199077 | 1.6829619 | down | mRNA |
| NM_001164276 | ZNF44 | 0.038281998 | 0.365250856 | 1.1045152 | down | mRNA |
| NM_001164277 | SLC37A4 | 0.742665927 | 0.970330526 | 1.041094 | down | mRNA |
| NM_001164279 | SLC37A4 | 0.552429301 | 0.926337111 | 1.0520041 | down | mRNA |
| NM_001164280 | SLC37A4 | 0.764768207 | 0.97415819 | 1.0428737 | down | mRNA |
| NM_001164309 | ZNF415 | 0.864843175 | 0.989401142 | 1.0217097 | down | mRNA |
| NM_001164315 | ANKRD36 | 0.667748458 | 0.958354006 | 1.0329373 | down | mRNA |
| NM_001164318 | FLNB | 0.857547101 | 0.988512246 | 1.0375929 | down | mRNA |
| NM_001164319 | FLNB | 0.935141108 | 0.996710991 | 1.0167002 | down | mRNA |
| NM_001164347 | ZBTB20 | 0.379171161 | 0.852705238 | 1.0777063 | down | mRNA |
| NM_001164373 | GPN3 | 0.247912382 | 0.746093848 | 1.6892543 | down | mRNA |
| NM_001164381 | STAU2 | 0.324921267 | 0.817191271 | 1.1157235 | down | mRNA |
| NM_001164382 | STAU2 | 0.228547988 | 0.724651871 | 1.2133108 | down | mRNA |
| NM_001164383 | STAU2 | 0.532168231 | 0.920228311 | 1.162805 | down | mRNA |
| NM_001164384 | STAU2 | 0.121010878 | 0.572400147 | 1.1775407 | down | mRNA |
| NM_001164385 | STAU2 | 0.961110693 | 0.99793927 | 1.0051199 | down | mRNA |
| NM_001164387 | RAPGEF6 | 0.633301215 | 0.951174475 | 1.0601264 | down | mRNA |
| NM_001164389 | RAPGEF6 | 0.665555602 | 0.958354006 | 1.129141 | down | mRNA |
| NM_001164392 | MTF2 | 0.141654098 | 0.6019041 | 1.3944514 | down | mRNA |
| NM_001164393 | MTF2 | 0.082860806 | 0.491030575 | 1.3391618 | down | mRNA |
| NM_001164405 | BHLHA9 | 0.803118132 | 0.98118927 | 1.0145871 | down | mRNA |
| NM_001164410 | CDK5 | 0.288578509 | 0.784514546 | 1.0686307 | down | mRNA |
| NM_001164419 | MBD3L4 | 0.812087671 | 0.98118927 | 1.032634 | down | mRNA |
| NM_001164440 | ANKRD33B | 0.422527584 | 0.873896892 | 1.0726485 | down | mRNA |
| NM_001164442 | FAM159B | 0.416907885 | 0.870739638 | 1.0320391 | down | mRNA |
| NM_001164458 | ACTR3C | 0.981693926 | 0.998971807 | 1.0035098 | down | mRNA |
| NM_001164463 | RGPD8 | 0.061998626 | 0.439101535 | 1.3918827 | down | mRNA |
| NM_001164465 | GOLGA6L10 | 0.035181555 | 0.352400835 | 1.1683812 | down | mRNA |
| NM_001164471 | TSPY4 | 0.218006014 | 0.712269655 | 1.1065696 | down | mRNA |
| NM_001164475 | SLC41A3 | 0.862497542 | 0.989401142 | 1.0149441 | down | mRNA |
| NM_001164490 | ADAM8 | 0.555029568 | 0.926833616 | 1.0144605 | down | mRNA |
| NM_001164501 | EIF4ENIF1 | 0.555241162 | 0.926833616 | 1.0934455 | down | mRNA |
| NM_001164502 | EIF4ENIF1 | 0.009731729 | 0.206100289 | 1.4079484 | down | mRNA |
| NM_001164579 | C16orf52 | 0.297700314 | 0.794023716 | 1.1742152 | down | mRNA |
| NM_001164629 | GTDC1 | 0.832135419 | 0.98620142 | 1.0700204 | down | mRNA |
| NM_001164637 | ENDOV | 0.189317524 | 0.674719659 | 1.1501285 | down | mRNA |
| NM_001164638 | ENDOV | 0.998184924 | 0.999889342 | 1.0005854 | down | mRNA |
| NM_001164644 | SFTPA1 | 0.147810924 | 0.610583444 | 1.1462264 | down | mRNA |
| NM_001164665 | KIAA1549 | 0.077972427 | 0.479677877 | 1.3745875 | down | mRNA |
| NM_001164673 | DOK7 | 0.063436625 | 0.440687083 | 1.2076301 | down | mRNA |
| NM_001164674 | SUMF1 | 0.470703121 | 0.898445976 | 1.1184167 | down | mRNA |
| NM_001164675 | SUMF1 | 0.688853715 | 0.960945025 | 1.0607483 | down | mRNA |
| NM_001164688 | RD3 | 0.309025046 | 0.803961878 | 1.0673167 | down | mRNA |
| NM_001164710 | AMT | 0.119759357 | 0.569711571 | 1.3318195 | down | mRNA |
| NM_001164716 | PYGM | 0.375775705 | 0.849862269 | 1.1187534 | down | mRNA |
| NM_001164722 | PTAFR | 0.44061379 | 0.883835791 | 1.0718395 | down | mRNA |
| NM_001164748 | RASSF8 | 0.039446996 | 0.370286706 | 1.1793462 | down | mRNA |
| NM_001164750 | ASPH | 0.709544517 | 0.965462762 | 1.075677 | down | mRNA |
| NM_001164751 | ASPH | 0.541358232 | 0.923577434 | 1.079897 | down | mRNA |
| NM_001164753 | ASPH | 0.261227679 | 0.761996785 | 1.1133631 | down | mRNA |
| NM_001164755 | ASPH | 0.198058746 | 0.686763988 | 1.1439225 | down | mRNA |
| NM_001164756 | ASPH | 0.031120268 | 0.34055236 | 1.1406191 | down | mRNA |
| NM_001164766 | ZFHX3 | 0.612790205 | 0.944398719 | 1.1422126 | down | mRNA |
| NM_001164773 | BCAT2 | 0.419708458 | 0.872365335 | 1.1013869 | down | mRNA |
| NM_001164780 | ATXN3 | 0.214162447 | 0.706760409 | 1.0885869 | down | mRNA |
| NM_001164803 | RBMX | 0.833656833 | 0.986434891 | 1.0187357 | down | mRNA |
| NM_001164811 | PET117 | 0.209523115 | 0.700367256 | 1.3686311 | down | mRNA |
| NM_001164815 | ACIN1 | 0.184749944 | 0.668017166 | 1.136429 | down | mRNA |
| NM_001164816 | ACIN1 | 0.417818616 | 0.871738133 | 1.1281665 | down | mRNA |
| NM_001164817 | ACIN1 | 0.514078254 | 0.91350662 | 1.0827304 | down | mRNA |
| NM_001164821 | DCAF17 | 0.569322275 | 0.932377004 | 1.0593372 | down | mRNA |
| NM_001164824 | C1orf212 | 0.927897134 | 0.995916751 | 1.0143179 | down | mRNA |
| NM_001164831 | FXYD6 | 0.938417722 | 0.996710991 | 1.0124788 | down | mRNA |
| NM_001165030 | TMEM41B | 0.353988333 | 0.836796976 | 1.1487601 | down | mRNA |
| NM_001165032 | RNF182 | 0.723664853 | 0.966191059 | 1.053138 | down | mRNA |
| NM_001165037 | GABRA5 | 0.997640843 | 0.999889342 | 1.0003245 | down | mRNA |
| NM_001165038 | GFRA2 | 0.953708696 | 0.996710991 | 1.0047507 | down | mRNA |
| NM_001165039 | GFRA2 | 0.443979399 | 0.885088246 | 1.0643197 | down | mRNA |
| NM_001165252 | KRTAP2-3 | 0.007301712 | 0.190890337 | 3.5702819 | down | mRNA |
| NM_001165257 | SCG3 | 0.801985384 | 0.98118927 | 1.078812 | down | mRNA |
| NM_001165413 | SERPINE1 | 0.141955454 | 0.60204132 | 1.4048334 | down | mRNA |
| NM_001165881 | ZNF268 | 0.552633607 | 0.926337111 | 1.1441305 | down | mRNA |
| NM_001165884 | ZNF268 | 0.063689891 | 0.441056837 | 1.2402273 | down | mRNA |
| NM_001165885 | ZNF268 | 0.408705602 | 0.867591329 | 1.1140062 | down | mRNA |
| NM_001165886 | ZNF268 | 0.459720474 | 0.892978158 | 1.1051129 | down | mRNA |
| NM_001165887 | ZNF268 | 0.449752377 | 0.888279993 | 1.1842046 | down | mRNA |
| NM_001165924 | EMX2 | 0.510997998 | 0.912481293 | 1.2369993 | down | mRNA |
| NM_001165931 | RRM2 | 0.12359244 | 0.577541136 | 1.2959354 | down | mRNA |
| NM_001165937 | STARD3 | 0.118014167 | 0.564712647 | 1.1894397 | down | mRNA |
| NM_001165947 | HTR2A | 0.419476539 | 0.872365335 | 1.4871779 | down | mRNA |
| NM_001165959 | GSDMB | 0.034498362 | 0.351589324 | 1.4605639 | down | mRNA |
| NM_001165962 | ELAC2 | 0.811889401 | 0.98118927 | 1.0466235 | down | mRNA |
| NM_001165966 | PITPNM3 | 0.728317065 | 0.966191059 | 1.0542078 | down | mRNA |
| NM_001165967 | HES7 | 0.850496302 | 0.987678166 | 1.036079 | down | mRNA |
| NM_001165970 | STRADA | 0.191923641 | 0.67769911 | 1.326745 | down | mRNA |
| NM_001165974 | UROC1 | 0.455646217 | 0.891148383 | 1.0985186 | down | mRNA |
| NM_001166017 | SKA3 | 0.739928273 | 0.96971907 | 1.2295738 | down | mRNA |
| NM_001166019 | AP1B1 | 0.464748616 | 0.894989844 | 1.0629782 | down | mRNA |
| NM_001166037 | ZNF260 | 0.543136335 | 0.923577434 | 1.0797139 | down | mRNA |
| NM_001166051 | APBB2 | 0.70166915 | 0.963236071 | 1.0519384 | down | mRNA |
| NM_001166059 | HMGCL | 0.047440482 | 0.397265185 | 1.2028807 | down | mRNA |
| NM_001166102 | NDUFV1 | 0.591905049 | 0.937421207 | 1.0700879 | down | mRNA |
| NM_001166103 | SPINT2 | 0.424793884 | 0.874854499 | 1.1161429 | down | mRNA |
| NM_001166107 | HMGCS2 | 0.965941591 | 0.99793927 | 1.0072757 | down | mRNA |
| NM_001166110 | PALLD | 0.910819742 | 0.995342392 | 1.0525121 | down | mRNA |
| NM_001166118 | NT5C3 | 0.183767928 | 0.667497719 | 1.1797402 | down | mRNA |
| NM_001166135 | RBM28 | 0.242353923 | 0.740622663 | 1.0800056 | down | mRNA |
| NM_001166136 | EVC2 | 0.054240976 | 0.417895991 | 1.2573326 | down | mRNA |
| NM_001166139 | LCORL | 0.003842895 | 0.143383256 | 1.8102617 | down | mRNA |
| NM_001166158 | PDHX | 0.646192957 | 0.95550188 | 1.0791298 | down | mRNA |
| NM_001166159 | NDUFS2 | 0.05926055 | 0.432586127 | 1.7287823 | down | mRNA |
| NM_001166169 | NEK6 | 0.253916363 | 0.754108603 | 1.482813 | down | mRNA |
| NM_001166170 | NEK6 | 0.004590198 | 0.155029743 | 1.415889 | down | mRNA |
| NM_001166176 | NKX2-5 | 0.687201138 | 0.960205549 | 1.1544299 | down | mRNA |
| NM_001166221 | UTP14A | 0.095733039 | 0.517208636 | 1.1108055 | down | mRNA |
| NM_001166222 | CARNS1 | 0.733924901 | 0.967777568 | 1.101167 | down | mRNA |
| NM_001166239 | CGREF1 | 0.72879213 | 0.966191059 | 1.0744718 | down | mRNA |
| NM_001166242 | C22orf39 | 0.555545962 | 0.926833616 | 1.1180832 | down | mRNA |
| NM_001166254 | TBXAS1 | 0.761953559 | 0.97339948 | 1.0165673 | down | mRNA |
| NM_001166264 | LTBP1 | 0.769719316 | 0.975286259 | 1.0639151 | down | mRNA |
| NM_001166265 | LTBP1 | 0.667533829 | 0.958354006 | 1.0775255 | down | mRNA |
| NM_001166269 | HAUS4 | 0.335431791 | 0.825166263 | 1.0946484 | down | mRNA |
| NM_001166278 | DLG3 | 0.982270396 | 0.998971807 | 1.0032181 | down | mRNA |
| NM_001166283 | RGMA | 0.462690906 | 0.894989844 | 1.2538292 | down | mRNA |
| NM_001166284 | CCT7 | 0.039518249 | 0.370322288 | 1.1468841 | down | mRNA |
| NM_001166285 | CCT7 | 0.308571571 | 0.803724978 | 1.0883209 | down | mRNA |
| NM_001166294 | SSX2IP | 0.826191115 | 0.984374112 | 1.0536893 | down | mRNA |
| NM_001166301 | DHX40 | 0.721627839 | 0.966191059 | 1.0829185 | down | mRNA |
| NM_001166304 | PIGX | 0.836144683 | 0.986575022 | 1.0116533 | down | mRNA |
| NM_001166349 | SLC26A11 | 0.623118766 | 0.948341211 | 1.1955172 | down | mRNA |
| NM_001166417 | SSX2IP | 0.400236767 | 0.864237287 | 1.3601792 | down | mRNA |
| NM_001166418 | HDAC8 | 0.072260583 | 0.463255488 | 1.1397451 | down | mRNA |
| NM_001166419 | HDAC8 | 0.57654356 | 0.933656777 | 1.049126 | down | mRNA |
| NM_001166448 | HDAC8 | 0.03514796 | 0.352396712 | 1.2890661 | down | mRNA |
| NM_001166461 | MPP1 | 0.539920779 | 0.923577434 | 1.0422025 | down | mRNA |
| NM_001166538 | LST1 | 0.441045816 | 0.883920737 | 1.0439865 | down | mRNA |
| NM_001166588 | EIF2B3 | 0.013635419 | 0.235988767 | 1.1581455 | down | mRNA |
| NM_001166599 | FAM122B | 0.329864581 | 0.821768501 | 1.0928654 | down | mRNA |
| NM_001166600 | FAM122B | 0.078279044 | 0.480303439 | 1.1793618 | down | mRNA |
| NM_001167575 | CAAP1 | 0.146811074 | 0.608803337 | 1.1456958 | down | mRNA |
| NM_001167582 | RBM6 | 0.428917307 | 0.877515262 | 1.1597967 | down | mRNA |
| NM_001167594 | CTSA | 0.656859543 | 0.957445617 | 1.0627298 | down | mRNA |
| NM_001167595 | AMACR | 0.847571879 | 0.987678166 | 1.0330242 | down | mRNA |
| NM_001167605 | KLK11 | 0.74633367 | 0.970394407 | 1.0638267 | down | mRNA |
| NM_001167608 | RHBDD1 | 0.346545565 | 0.83333713 | 1.9815035 | down | mRNA |
| NM_001167617 | MLH1 | 0.370474237 | 0.846215286 | 1.2303021 | down | mRNA |
| NM_001167618 | MLH1 | 0.376133626 | 0.849862269 | 1.1173628 | down | mRNA |
| NM_001167621 | ATXN10 | 0.187315226 | 0.671419231 | 1.1066155 | down | mRNA |
| NM_001167624 | CACNA1C | 0.205218163 | 0.695403826 | 1.1052224 | down | mRNA |
| NM_001167675 | CADM2 | 0.931036568 | 0.996710991 | 1.0071207 | down | mRNA |
| NM_001167734 | VARS2 | 0.083405799 | 0.492251581 | 1.056677 | down | mRNA |
| NM_001167741 | MTX3 | 0.396968355 | 0.862344286 | 1.2620238 | down | mRNA |
| NM_001167857 | PPP1R12B | 0.141548442 | 0.601858131 | 1.3974156 | down | mRNA |
| NM_001167867 | ATP5SL | 0.705913052 | 0.965020205 | 1.0419117 | down | mRNA |
| NM_001167868 | ATP5SL | 0.598240121 | 0.940397814 | 1.0392584 | down | mRNA |
| NM_001167880 | LHPP | 0.979737996 | 0.998971807 | 1.0031214 | down | mRNA |
| NM_001167882 | ANKRD50 | 0.237874595 | 0.735771951 | 1.0804391 | down | mRNA |
| NM_001167903 | MFSD1 | 0.426623673 | 0.876202345 | 1.0478682 | down | mRNA |
| NM_001167911 | VEPH1 | 0.711379683 | 0.965796723 | 1.1383214 | down | mRNA |
| NM_001167934 | CCM2 | 0.032811619 | 0.34498156 | 1.0754107 | down | mRNA |
| NM_001167935 | CCM2 | 0.346966707 | 0.833567116 | 1.4028204 | down | mRNA |
| NM_001167941 | WDR76 | 0.997743471 | 0.999889342 | 1.0011063 | down | mRNA |
| NM_001167947 | TIMM17B | 0.49292447 | 0.906130999 | 1.087834 | down | mRNA |
| NM_001167962 | SLC26A5 | 0.925161961 | 0.995465766 | 1.0108788 | down | mRNA |
| NM_001167970 | TRMT2B | 0.112074215 | 0.554156842 | 1.5617413 | down | mRNA |
| NM_001167971 | TRMT2B | 0.469624874 | 0.89787452 | 1.1511063 | down | mRNA |
| NM_001168 | BIRC5 | 0.771935273 | 0.975347132 | 1.1482711 | down | mRNA |
| NM_001168241 | FAM59B | 0.070409164 | 0.458686035 | 1.0962182 | down | mRNA |
| NM_001168243 | C4orf48 | 0.710162357 | 0.965462762 | 1.0242827 | down | mRNA |
| NM_001168331 | NDUFB4 | 0.01122184 | 0.217427139 | 1.6702565 | down | mRNA |
| NM_001168335 | ME2 | 0.556933579 | 0.926833616 | 1.0275242 | down | mRNA |
| NM_001168344 | RREB1 | 0.041822058 | 0.378824525 | 1.4741745 | down | mRNA |
| NM_001168347 | NSUN5 | 0.12125167 | 0.572790183 | 1.1615136 | down | mRNA |
| NM_001168348 | NSUN5 | 0.186246801 | 0.669888134 | 1.2487717 | down | mRNA |
| NM_001168357 | PLA2G7 | 0.670882525 | 0.959531882 | 1.1577368 | down | mRNA |
| NM_001168394 | CREBRF | 0.587160987 | 0.93579682 | 1.1827031 | down | mRNA |
| NM_001168398 | SLC35A1 | 0.140796838 | 0.60102484 | 1.2191326 | down | mRNA |
| NM_001168399 | BEX2 | 0.503567178 | 0.910553209 | 1.1473843 | down | mRNA |
| NM_001168408 | RIMS1 | 0.965957055 | 0.99793927 | 1.0284462 | down | mRNA |
| NM_001168411 | RIMS1 | 0.973434362 | 0.998501532 | 1.032671 | down | mRNA |
| NM_001168412 | TSPAN3 | 0.932204912 | 0.996710991 | 1.0157791 | down | mRNA |
| NM_001168466 | MAP7D2 | 0.451945789 | 0.888854908 | 1.0528018 | down | mRNA |
| NM_001168467 | MAP7D2 | 0.295385931 | 0.79157694 | 2.1948119 | down | mRNA |
| NM_001168478 | ARMCX5 | 0.065685467 | 0.448009698 | 1.1905711 | down | mRNA |
| NM_001168499 | CNDP2 | 0.237875567 | 0.735771951 | 1.0874187 | down | mRNA |
| NM_001168530 | ASB9 | 0.657283479 | 0.957641541 | 1.0523366 | down | mRNA |
| NM_001168683 | TXLNG | 0.170359712 | 0.647247575 | 1.1141266 | down | mRNA |
| NM_001168724 | TMEM135 | 0.75851229 | 0.972072638 | 1.1239233 | down | mRNA |
| NM_001169106 | FAM21C | 0.609035802 | 0.943053241 | 1.0629332 | down | mRNA |
| NM_001169117 | STIM2 | 0.645597372 | 0.955130468 | 1.0979758 | down | mRNA |
| NM_001170464 | CDK17 | 0.252760702 | 0.752986167 | 1.3533317 | down | mRNA |
| NM_001170535 | ATAD3A | 0.368631239 | 0.845559574 | 1.204787 | down | mRNA |
| NM_001170543 | PGAM5 | 0.619856914 | 0.947444797 | 1.0836421 | down | mRNA |
| NM_001170585 | PLB1 | 0.130721245 | 0.585423868 | 1.1454074 | down | mRNA |
| NM_001170629 | CHD8 | 0.920787201 | 0.995349793 | 1.0142403 | down | mRNA |
| NM_001170631 | FCAMR | 0.41757871 | 0.871416436 | 1.1289959 | down | mRNA |
| NM_001170637 | SRGAP2 | 0.184676826 | 0.668017166 | 1.7765738 | down | mRNA |
| NM_001170649 | LAS1L | 0.127991413 | 0.581241479 | 1.1387201 | down | mRNA |
| NM_001170650 | LAS1L | 0.786745613 | 0.977479172 | 1.0432412 | down | mRNA |
| NM_001170687 | MIB2 | 0.837222378 | 0.986775289 | 1.0307837 | down | mRNA |
| NM_001170689 | MIB2 | 0.4767022 | 0.900429473 | 1.0906627 | down | mRNA |
| NM_001170690 | SCUBE2 | 0.13155477 | 0.586786184 | 1.1169996 | down | mRNA |
| NM_001170693 | CAGE1 | 0.999147965 | 0.999897336 | 1.0001412 | down | mRNA |
| NM_001170702 | MBNL3 | 0.416758892 | 0.870736736 | 1.0986835 | down | mRNA |
| NM_001170714 | BCAR1 | 0.464410288 | 0.894989844 | 1.1369004 | down | mRNA |
| NM_001170717 | BCAR1 | 0.050333286 | 0.406008044 | 1.1579521 | down | mRNA |
| NM_001170718 | BCAR1 | 0.110196012 | 0.549412779 | 1.215982 | down | mRNA |
| NM_001170719 | BCAR1 | 0.00400945 | 0.145198162 | 1.2808939 | down | mRNA |
| NM_001170720 | BCAR1 | 0.5687327 | 0.932312606 | 1.1665509 | down | mRNA |
| NM_001170751 | SRPX | 0.88324544 | 0.992766819 | 1.1253075 | down | mRNA |
| NM_001170752 | SRPX | 0.921153654 | 0.995349793 | 1.0715254 | down | mRNA |
| NM_001170753 | PPP1R32 | 0.629949328 | 0.950410442 | 1.0891176 | down | mRNA |
| NM_001170755 | NBPF15 | 0.166969709 | 0.641987164 | 1.0649487 | down | mRNA |
| NM_001170756 | FAM122B | 0.051810263 | 0.41031217 | 1.2330777 | down | mRNA |
| NM_001170765 | LCOR | 0.009153281 | 0.203383174 | 1.2652878 | down | mRNA |
| NM_001170766 | LCOR | 0.008093733 | 0.196374442 | 1.0899736 | down | mRNA |
| NM_001170790 | MFSD5 | 0.524223886 | 0.917428132 | 1.2135018 | down | mRNA |
| NM_001170796 | ZFAND1 | 0.097207226 | 0.519831481 | 1.1905036 | down | mRNA |
| NM_001170797 | ZFAND1 | 0.148141751 | 0.611080968 | 1.1656686 | down | mRNA |
| NM_001170820 | IFITM10 | 0.043490319 | 0.384598968 | 1.3806354 | down | mRNA |
| NM_001170880 | GPR137 | 0.301845551 | 0.797029057 | 1.0664035 | down | mRNA |
| NM_001170881 | GPR137 | 0.660863569 | 0.958354006 | 1.0552395 | down | mRNA |
| NM_001170931 | FOXO4 | 0.445011569 | 0.885721054 | 1.1937887 | down | mRNA |
| NM_001171 | ABCC6 | 0.63408407 | 0.951206671 | 1.088343 | down | mRNA |
| NM_001171038 | ASMT | 0.674426638 | 0.959531882 | 1.1693054 | down | mRNA |
| NM_001171080 | RBM41 | 0.114046218 | 0.557835051 | 1.210202 | down | mRNA |
| NM_001171083 | SMPD4 | 0.426210176 | 0.875717896 | 1.0531272 | down | mRNA |
| NM_001171087 | CLCN2 | 0.051843988 | 0.41031217 | 1.1966191 | down | mRNA |
| NM_001171089 | CLCN2 | 0.2890105 | 0.784785662 | 1.3276886 | down | mRNA |
| NM_001171091 | UBXN6 | 0.506431911 | 0.911362118 | 1.1039968 | down | mRNA |
| NM_001171093 | FAM131A | 0.440425151 | 0.883544565 | 1.480581 | down | mRNA |
| NM_001171134 | FAM3A | 0.07040269 | 0.458686035 | 1.2576276 | down | mRNA |
| NM_001171158 | SIGLEC10 | 0.295127675 | 0.791537205 | 1.1334632 | down | mRNA |
| NM_001171162 | ZMYM3 | 0.524594778 | 0.917632259 | 1.1809462 | down | mRNA |
| NM_001171163 | ZMYM3 | 0.996134988 | 0.999872237 | 1.0003843 | down | mRNA |
| NM_001171166 | CAMTA2 | 0.429173229 | 0.877844001 | 1.0687516 | down | mRNA |
| NM_001171168 | CAMTA2 | 0.001870167 | 0.105670067 | 1.3565315 | down | mRNA |
| NM_001171603 | PREPL | 0.001124866 | 0.087572727 | 1.3608002 | down | mRNA |
| NM_001171613 | PREPL | 0.163303869 | 0.635463018 | 1.1365492 | down | mRNA |
| NM_001171689 | AMMECR1 | 0.045054436 | 0.389144768 | 1.3099854 | down | mRNA |
| NM_001171706 | RIPPLY1 | 0.805758274 | 0.98118927 | 1.0223376 | down | mRNA |
| NM_001171887 | FGFR1OP2 | 0.049381221 | 0.402500646 | 1.1970851 | down | mRNA |
| NM_001171888 | FGFR1OP2 | 0.725967963 | 0.966191059 | 1.0209227 | down | mRNA |
| NM_001171904 | VPRBP | 0.156013676 | 0.623931953 | 1.2659342 | down | mRNA |
| NM_001171937 | FUZ | 0.238731424 | 0.736777065 | 1.0939313 | down | mRNA |
| NM_001171967 | RBFA | 0.804239408 | 0.98118927 | 1.013551 | down | mRNA |
| NM_001171991 | HCCS | 0.344738542 | 0.8321226 | 1.0565918 | down | mRNA |
| NM_001172085 | TBP | 0.287931833 | 0.784247799 | 1.0656382 | down | mRNA |
| NM_001172087 | PEX2 | 0.546395308 | 0.92469697 | 1.0396342 | down | mRNA |
| NM_001172128 | IL16 | 0.321167619 | 0.815220381 | 1.3051651 | down | mRNA |
| NM_001172221 | SCMH1 | 0.228477545 | 0.724599197 | 1.0943018 | down | mRNA |
| NM_001172225 | ZNF540 | 0.420351432 | 0.872365335 | 1.2715059 | down | mRNA |
| NM_001172292 | NIPAL4 | 0.14045934 | 0.600490836 | 1.2722507 | down | mRNA |
| NM_001172309 | NEXN | 0.119720006 | 0.569657653 | 1.2395138 | down | mRNA |
| NM_001172357 | SFTPC | 0.730518961 | 0.966329458 | 1.0755553 | down | mRNA |
| NM_001172411 | VANGL1 | 0.17034357 | 0.647247575 | 1.1730096 | down | mRNA |
| NM_001172412 | VANGL1 | 0.085463512 | 0.497360491 | 1.345057 | down | mRNA |
| NM_001172415 | BAG1 | 0.97459046 | 0.998501532 | 1.0027248 | down | mRNA |
| NM_001172416 | KCNJ13 | 0.083764939 | 0.493003289 | 1.2340621 | down | mRNA |
| NM_001172420 | EFHC1 | 0.324976508 | 0.817191271 | 1.0756376 | down | mRNA |
| NM_001172435 | RAB3GAP1 | 0.009697122 | 0.206060838 | 1.2542567 | down | mRNA |
| NM_001172439 | ENDOU | 0.207732243 | 0.698556218 | 1.3885693 | down | mRNA |
| NM_001172478 | RRM2B | 0.67968264 | 0.960007989 | 1.0227727 | down | mRNA |
| NM_001172562 | INTS9 | 0.081974238 | 0.488911764 | 1.5614079 | down | mRNA |
| NM_001172568 | MYD88 | 0.662698038 | 0.958354006 | 1.0592646 | down | mRNA |
| NM_001172569 | MYD88 | 0.683664457 | 0.960111041 | 1.0891098 | down | mRNA |
| NM_001172574 | MCPH1 | 0.007940482 | 0.194982936 | 1.2569824 | down | mRNA |
| NM_001172575 | MCPH1 | 0.012123132 | 0.222675671 | 1.4293037 | down | mRNA |
| NM_001172633 | OLR1 | 0.550921773 | 0.925960004 | 1.067834 | down | mRNA |
| NM_001172638 | ZFP62 | 0.253600747 | 0.753611573 | 1.2172134 | down | mRNA |
| NM_001172643 | TK2 | 0.129386196 | 0.583907255 | 1.0701431 | down | mRNA |
| NM_001172644 | TK2 | 0.019239664 | 0.27881743 | 1.1670694 | down | mRNA |
| NM_001172645 | TK2 | 0.503912154 | 0.910568383 | 1.072615 | down | mRNA |
| NM_001172646 | PLCB4 | 0.052783966 | 0.413038171 | 1.376003 | down | mRNA |
| NM_001172649 | CELF3 | 0.524129191 | 0.917428132 | 1.0499025 | down | mRNA |
| NM_001172651 | ZNF177 | 0.023642125 | 0.302131789 | 1.3980755 | down | mRNA |
| NM_001172656 | ZFYVE28 | 0.020554226 | 0.286034385 | 1.8025648 | down | mRNA |
| NM_001172657 | ZFYVE28 | 0.407365476 | 0.867591329 | 1.0811337 | down | mRNA |
| NM_001172658 | ZFYVE28 | 0.300967231 | 0.795950279 | 1.3830578 | down | mRNA |
| NM_001172660 | ZFYVE28 | 0.020552301 | 0.286034385 | 1.360538 | down | mRNA |
| NM_001172675 | ZNF347 | 0.011515176 | 0.219219625 | 1.5584296 | down | mRNA |
| NM_001172679 | ZNF764 | 0.40590253 | 0.866541972 | 1.1546354 | down | mRNA |
| NM_001172688 | GGA1 | 0.851373775 | 0.987678166 | 1.0168354 | down | mRNA |
| NM_001172698 | PPARGC1B | 0.45909601 | 0.892904132 | 1.1130688 | down | mRNA |
| NM_001172699 | PPARGC1B | 0.631448139 | 0.950475493 | 1.1299183 | down | mRNA |
| NM_001172702 | SLC38A6 | 0.142684162 | 0.602753869 | 1.1130888 | down | mRNA |
| NM_001172703 | GGA3 | 0.367820263 | 0.845181673 | 1.3308457 | down | mRNA |
| NM_001172705 | EIF4G2 | 0.242167544 | 0.740610726 | 1.1278919 | down | mRNA |
| NM_001172713 | GOLGA4 | 0.518195824 | 0.916125349 | 1.0890266 | down | mRNA |
| NM_001172773 | ZNF548 | 0.313480051 | 0.808703036 | 1.0905023 | down | mRNA |
| NM_001172819 | PGM1 | 0.285149553 | 0.781963593 | 1.1590376 | down | mRNA |
| NM_001172828 | YTHDF2 | 0.160424734 | 0.631980676 | 1.2781194 | down | mRNA |
| NM_001173393 | HAVCR1 | 0.296698254 | 0.793167205 | 1.2438879 | down | mRNA |
| NM_001173408 | OBSL1 | 0.654369021 | 0.957205183 | 1.09645 | down | mRNA |
| NM_001173455 | PDHA1 | 0.007000271 | 0.186784121 | 1.2947697 | down | mRNA |
| NM_001173456 | PDHA1 | 0.05053107 | 0.406628421 | 1.1456915 | down | mRNA |
| NM_001173463 | KIF21A | 0.39526691 | 0.861590143 | 1.1294107 | down | mRNA |
| NM_001173465 | KIF21A | 0.331054859 | 0.821853462 | 1.1374677 | down | mRNA |
| NM_001173468 | PDHB | 0.041034381 | 0.375308608 | 1.2027768 | down | mRNA |
| NM_001173474 | ASMTL | 0.164835404 | 0.638119216 | 1.2801738 | down | mRNA |
| NM_001173476 | SPEG | 0.898491776 | 0.994454806 | 1.0044625 | down | mRNA |
| NM_001173480 | ARHGEF9 | 0.171332954 | 0.648825037 | 1.2535391 | down | mRNA |
| NM_001173482 | CRBN | 0.041421173 | 0.377487799 | 1.1991582 | down | mRNA |
| NM_001173512 | MRRF | 0.114891563 | 0.559704057 | 1.2156126 | down | mRNA |
| NM_001173517 | MAP7D3 | 0.748646697 | 0.970725371 | 1.0177283 | down | mRNA |
| NM_001173523 | PCDH7 | 0.557084811 | 0.926833616 | 1.0292243 | down | mRNA |
| NM_001173540 | BANP | 0.979824472 | 0.998971807 | 1.002415 | down | mRNA |
| NM_001173984 | BRD7 | 0.079021811 | 0.481760561 | 1.1547874 | down | mRNA |
| NM_001173990 | TMEM216 | 0.486912667 | 0.903606093 | 1.08836 | down | mRNA |
| NM_001174044 | SPAM1 | 0.366007975 | 0.845164582 | 1.0295884 | down | mRNA |
| NM_001174061 | SMG7 | 0.343505752 | 0.831536584 | 1.1347546 | down | mRNA |
| NM_001174072 | SERINC5 | 0.369332848 | 0.846020219 | 1.0858371 | down | mRNA |
| NM_001174088 | NCOA3 | 0.004013445 | 0.145198162 | 1.5729638 | down | mRNA |
| NM_001174089 | SLC4A11 | 0.911189465 | 0.995342392 | 1.0254855 | down | mRNA |
| NM_001174090 | SLC4A11 | 0.462832562 | 0.894989844 | 1.091628 | down | mRNA |
| NM_001174093 | ZEB1 | 0.276989538 | 0.777205298 | 1.0756257 | down | mRNA |
| NM_001174095 | ZEB1 | 0.522347199 | 0.917129814 | 1.0805776 | down | mRNA |
| NM_001174096 | ZEB1 | 0.395186771 | 0.861590143 | 1.0766705 | down | mRNA |
| NM_001174097 | LDHB | 0.073461343 | 0.466317837 | 1.1162144 | down | mRNA |
| NM_001174101 | PRR7 | 0.007039028 | 0.18703243 | 1.2795774 | down | mRNA |
| NM_001174108 | ZBED6 | 0.841960135 | 0.987123192 | 1.0173631 | down | mRNA |
| NM_001174118 | MEX3D | 0.710893005 | 0.96568622 | 1.077301 | down | mRNA |
| NM_001174119 | ZFYVE27 | 0.377908964 | 0.851373413 | 1.1348717 | down | mRNA |
| NM_001174122 | ZFYVE27 | 0.452932798 | 0.889563297 | 1.0731132 | down | mRNA |
| NM_001174129 | SLC11A2 | 0.679245979 | 0.959969193 | 1.3553758 | down | mRNA |
| NM_001174147 | LMX1B | 0.536618265 | 0.922509728 | 1.2080034 | down | mRNA |
| NM_001174150 | ARL13B | 0.326758163 | 0.81979849 | 1.1172824 | down | mRNA |
| NM_001174151 | ARL13B | 0.581949328 | 0.934005566 | 1.045156 | down | mRNA |
| NM_001174153 | RABEPK | 0.146379488 | 0.608122555 | 1.1135649 | down | mRNA |
| NM_001174157 | ZFAT | 0.769899801 | 0.975286259 | 1.0880102 | down | mRNA |
| NM_001174159 | SH2D4A | 0.41780011 | 0.871738133 | 1.2583646 | down | mRNA |
| NM_001174160 | SH2D4A | 0.310645586 | 0.805412658 | 1.3364331 | down | mRNA |
| NM_001174164 | PRRT4 | 0.692074937 | 0.961451327 | 1.0694369 | down | mRNA |
| NM_001174168 | SYK | 0.109405586 | 0.547509603 | 1.1401286 | down | mRNA |
| NM_001175 | ARHGDIB | 0.926001133 | 0.995699344 | 1.038051 | down | mRNA |
| NM_001177 | ARL1 | 0.422469256 | 0.873896892 | 1.0607181 | down | mRNA |
| NM_001177304 | PLSCR4 | 0.694250107 | 0.962004578 | 1.0981007 | down | mRNA |
| NM_001177306 | PAM | 0.242963734 | 0.741217225 | 1.1616295 | down | mRNA |
| NM_001177376 | DUX4L4 | 0.635633419 | 0.951814727 | 1.1499754 | down | mRNA |
| NM_001177433 | TRPV4 | 0.444328926 | 0.885088246 | 1.1623291 | down | mRNA |
| NM_001177478 | HDX | 0.777379371 | 0.976938981 | 1.1495348 | down | mRNA |
| NM_001177547 | SIGLEC6 | 0.088995645 | 0.504027702 | 1.5227166 | down | mRNA |
| NM_001177597 | THPO | 0.132137849 | 0.587843174 | 1.0990542 | down | mRNA |
| NM_001177612 | CINP | 0.777516967 | 0.976938981 | 1.0456669 | down | mRNA |
| NM_001177642 | DAG1 | 0.50432279 | 0.910568383 | 1.1090661 | down | mRNA |
| NM_001177651 | SLC9A6 | 0.182868026 | 0.666166668 | 1.2908622 | down | mRNA |
| NM_001177693 | ARHGEF28 | 0.716695396 | 0.966191059 | 1.0765357 | down | mRNA |
| NM_001177712 | RBMS3 | 0.614384976 | 0.944827645 | 1.123408 | down | mRNA |
| NM_001177801 | RANGRF | 0.107240889 | 0.542766422 | 1.0665091 | down | mRNA |
| NM_001177802 | RANGRF | 0.256157353 | 0.757240077 | 1.0360935 | down | mRNA |
| NM_001177963 | MNAT1 | 0.480320418 | 0.901538317 | 1.0634543 | down | mRNA |
| NM_001177969 | VIT | 0.239563464 | 0.737777091 | 1.4724229 | down | mRNA |
| NM_001177984 | SCN8A | 0.858925372 | 0.988512246 | 1.005835 | down | mRNA |
| NM_001177999 | SLC34A2 | 0.808908715 | 0.98118927 | 1.0204367 | down | mRNA |
| NM_001178 | ARNTL | 0.100989205 | 0.528251228 | 1.1857721 | down | mRNA |
| NM_001178003 | WDR66 | 0.464390703 | 0.894989844 | 1.1611844 | down | mRNA |
| NM_001178004 | PCCA | 0.581406988 | 0.934005566 | 1.0551705 | down | mRNA |
| NM_001178005 | BHMT2 | 0.893448941 | 0.993978977 | 1.0807792 | down | mRNA |
| NM_001178014 | PCCB | 0.50726357 | 0.911674633 | 1.102812 | down | mRNA |
| NM_001178030 | SF1 | 0.580298458 | 0.93393768 | 1.0515579 | down | mRNA |
| NM_001178034 | DPP10 | 0.823998851 | 0.983568283 | 1.0948103 | down | mRNA |
| NM_001178035 | CEP85L | 0.726770565 | 0.966191059 | 1.0854556 | down | mRNA |
| NM_001178040 | STX3 | 0.103980012 | 0.533896783 | 1.3170181 | down | mRNA |
| NM_001178056 | PARP8 | 0.305915101 | 0.801497191 | 1.1588597 | down | mRNA |
| NM_001178061 | SEMA6C | 0.489336837 | 0.904636895 | 1.0516195 | down | mRNA |
| NM_001178064 | NELF | 0.699216873 | 0.962701213 | 1.0937751 | down | mRNA |
| NM_001178075 | ASNS | 0.92043083 | 0.995349793 | 1.0216567 | down | mRNA |
| NM_001178076 | ASNS | 0.803003178 | 0.98118927 | 1.04617 | down | mRNA |
| NM_001178083 | EXT2 | 0.852714133 | 0.987678166 | 1.0451697 | down | mRNA |
| NM_001178087 | SERF1B | 0.377076558 | 0.850710713 | 1.1295202 | down | mRNA |
| NM_001178100 | CD8B | 0.82982583 | 0.985549562 | 1.0228615 | down | mRNA |
| NM_001178106 | ZNF185 | 0.932911188 | 0.996710991 | 1.0188717 | down | mRNA |
| NM_001178111 | SCAMP5 | 0.636009979 | 0.951814727 | 1.0939794 | down | mRNA |
| NM_001178113 | ZNF185 | 0.163135258 | 0.635414954 | 1.296528 | down | mRNA |
| NM_001178133 | SUFU | 0.407975571 | 0.867591329 | 1.5219189 | down | mRNA |
| NM_001178134 | CXCL12 | 0.795711695 | 0.980137767 | 1.1476221 | down | mRNA |
| NM_001178136 | HDHD1 | 0.043021766 | 0.383286307 | 1.3269337 | down | mRNA |
| NM_001178139 | TFDP2 | 0.209490853 | 0.700367256 | 1.0671744 | down | mRNA |
| NM_001178140 | TFDP2 | 0.093631438 | 0.512619241 | 1.1488877 | down | mRNA |
| NM_001178141 | TFDP2 | 0.732993318 | 0.967703318 | 1.0168691 | down | mRNA |
| NM_001181 | ASGR2 | 0.529986102 | 0.91899174 | 1.0743356 | down | mRNA |
| NM_001184 | ATR | 0.480403137 | 0.901538317 | 1.0474151 | down | mRNA |
| NM_001184700 | UGDH | 0.427481132 | 0.876959579 | 1.2733553 | down | mRNA |
| NM_001184701 | UGDH | 0.578992681 | 0.933821303 | 1.1274546 | down | mRNA |
| NM_001184717 | TIPARP | 0.039241594 | 0.369575679 | 1.9429513 | down | mRNA |
| NM_001184720 | GYG1 | 0.84981727 | 0.987678166 | 1.0344715 | down | mRNA |
| NM_001184721 | GYG1 | 0.624257842 | 0.948341211 | 1.101478 | down | mRNA |
| NM_001184728 | PIGT | 0.955541994 | 0.996883425 | 1.0059745 | down | mRNA |
| NM_001184730 | PIGT | 0.960378017 | 0.997738713 | 1.0048397 | down | mRNA |
| NM_001184731 | GHRH | 0.60518549 | 0.94174007 | 1.0426777 | down | mRNA |
| NM_001184732 | SLC22A8 | 0.696851437 | 0.962410025 | 1.1480307 | down | mRNA |
| NM_001184739 | TSSK4 | 0.846803337 | 0.987678166 | 1.0151268 | down | mRNA |
| NM_001184740 | CTTN | 0.026293024 | 0.317461702 | 1.3954692 | down | mRNA |
| NM_001184742 | ZBTB33 | 0.11522899 | 0.560621161 | 1.4949019 | down | mRNA |
| NM_001184746 | PAFAH1B2 | 0.491681646 | 0.905806259 | 1.1581954 | down | mRNA |
| NM_001184762 | CLTA | 0.21653113 | 0.711074992 | 1.112244 | down | mRNA |
| NM_001184763 | UHMK1 | 0.321438015 | 0.815220381 | 1.5347372 | down | mRNA |
| NM_001184766 | ODF2L | 0.254580937 | 0.754979524 | 1.2076751 | down | mRNA |
| NM_001184777 | SEZ6L | 0.469116309 | 0.897810636 | 1.0667761 | down | mRNA |
| NM_001184779 | NOX5 | 0.894412806 | 0.993978977 | 1.0220625 | down | mRNA |
| NM_001184780 | NOX5 | 0.894490316 | 0.993978977 | 1.0253408 | down | mRNA |
| NM_001184782 | CXorf57 | 0.745674272 | 0.970394407 | 1.019333 | down | mRNA |
| NM_001184783 | VDAC2 | 0.095380155 | 0.516597924 | 1.0972731 | down | mRNA |
| NM_001184785 | PARD3 | 0.393230394 | 0.859879584 | 1.1550003 | down | mRNA |
| NM_001184787 | PARD3 | 0.582034367 | 0.934005566 | 1.0351032 | down | mRNA |
| NM_001184788 | PARD3 | 0.157033991 | 0.625751326 | 1.2402217 | down | mRNA |
| NM_001184791 | PARD3 | 0.561859577 | 0.928808234 | 1.0726962 | down | mRNA |
| NM_001184796 | ESYT1 | 0.649563604 | 0.95550188 | 1.0265839 | down | mRNA |
| NM_001184797 | TMLHE | 0.552759778 | 0.926337111 | 1.0521575 | down | mRNA |
| NM_001184801 | UBR2 | 0.071426923 | 0.461912278 | 1.2697357 | down | mRNA |
| NM_001184808 | CD99L2 | 0.175608767 | 0.654053388 | 1.1210452 | down | mRNA |
| NM_001184813 | CEACAM1 | 0.487383021 | 0.903736579 | 1.082145 | down | mRNA |
| NM_001184824 | PRMT7 | 0.060580033 | 0.435971562 | 1.2467103 | down | mRNA |
| NM_001184825 | PSG1 | 0.84330231 | 0.987338076 | 1.2201609 | down | mRNA |
| NM_001184833 | GPR64 | 0.936996008 | 0.996710991 | 1.0336815 | down | mRNA |
| NM_001184856 | KLHDC4 | 0.660208823 | 0.958354006 | 1.0522238 | down | mRNA |
| NM_001184874 | GPRASP2 | 0.098644167 | 0.52242281 | 1.1338508 | down | mRNA |
| NM_001184891 | FLAD1 | 0.089154833 | 0.504744674 | 1.131613 | down | mRNA |
| NM_001184892 | FLAD1 | 0.128268082 | 0.581871132 | 1.2438421 | down | mRNA |
| NM_001184902 | CARD8 | 0.99524555 | 0.999872237 | 1.0010738 | down | mRNA |
| NM_001184904 | CARD8 | 0.705519615 | 0.964798549 | 1.1915448 | down | mRNA |
| NM_001184906 | FBXL20 | 0.396137886 | 0.861880738 | 1.1235974 | down | mRNA |
| NM_001184917 | PCYT2 | 0.245661168 | 0.743916784 | 1.0701517 | down | mRNA |
| NM_001184937 | EPB41L5 | 0.193432635 | 0.680621781 | 1.0843714 | down | mRNA |
| NM_001184960 | SH3KBP1 | 0.431504936 | 0.880121832 | 1.180157 | down | mRNA |
| NM_001184965 | WDR44 | 0.007682743 | 0.193257016 | 1.2862639 | down | mRNA |
| NM_001184966 | WDR44 | 0.069848538 | 0.457827646 | 1.3370555 | down | mRNA |
| NM_001184967 | MITF | 0.318257438 | 0.81230357 | 1.1024565 | down | mRNA |
| NM_001184970 | PACSIN2 | 0.046391995 | 0.392259245 | 1.1418393 | down | mRNA |
| NM_001184971 | PACSIN2 | 0.714811348 | 0.966110889 | 1.0277761 | down | mRNA |
| NM_001184975 | PACSIN3 | 0.932886223 | 0.996710991 | 1.0304551 | down | mRNA |
| NM_001184979 | NDUFS5 | 0.949302366 | 0.996710991 | 1.0034372 | down | mRNA |
| NM_001184992 | RNF135 | 0.329448572 | 0.821768501 | 1.1883635 | down | mRNA |
| NM_001184998 | KIAA0430 | 0.374800034 | 0.849578399 | 1.0579578 | down | mRNA |
| NM_001185010 | RNF4 | 0.639910097 | 0.953734314 | 1.0516759 | down | mRNA |
| NM_001185012 | NDUFA2 | 0.90281636 | 0.99508468 | 1.011479 | down | mRNA |
| NM_001185015 | SP110 | 0.789103165 | 0.977807732 | 1.0415255 | down | mRNA |
| NM_001185024 | ZDHHC8 | 0.254478365 | 0.754895553 | 1.4780929 | down | mRNA |
| NM_001185063 | ZNF75D | 0.628819185 | 0.950128593 | 1.1268772 | down | mRNA |
| NM_001185072 | CLDN12 | 0.182708604 | 0.666042256 | 1.1768775 | down | mRNA |
| NM_001185074 | ZCCHC6 | 0.295181672 | 0.791563407 | 1.2314487 | down | mRNA |
| NM_001185075 | FMR1 | 0.083519756 | 0.492495266 | 1.2731319 | down | mRNA |
| NM_001185076 | FMR1 | 0.010427297 | 0.209736088 | 1.1680747 | down | mRNA |
| NM_001185094 | NIT1 | 0.101365683 | 0.529124784 | 1.0669289 | down | mRNA |
| NM_001185097 | INS | 0.816701733 | 0.981692737 | 1.025816 | down | mRNA |
| NM_001185101 | CD22 | 0.747321389 | 0.970534111 | 1.0487347 | down | mRNA |
| NM_001185115 | CD1E | 0.348881132 | 0.834138149 | 1.1097013 | down | mRNA |
| NM_001185127 | VAMP4 | 0.36178306 | 0.842778778 | 1.0904536 | down | mRNA |
| NM_001185181 | PFDN6 | 0.812510781 | 0.98118927 | 1.0650809 | down | mRNA |
| NM_001185183 | VAMP7 | 0.126753418 | 0.580406758 | 1.1238127 | down | mRNA |
| NM_001188 | BAK1 | 0.216669937 | 0.711074992 | 1.2457134 | down | mRNA |
| NM_001190182 | CCDC17 | 0.332078889 | 0.822591128 | 1.5110284 | down | mRNA |
| NM_001190202 | CES4A | 0.824328867 | 0.983583012 | 1.0565028 | down | mRNA |
| NM_001190214 | PPIP5K1 | 0.583818327 | 0.934397621 | 1.0346925 | down | mRNA |
| NM_001190233 | DNAJC19 | 0.374315398 | 0.849321997 | 1.0883498 | down | mRNA |
| NM_001190241 | IFT80 | 0.793325947 | 0.979053303 | 1.070168 | down | mRNA |
| NM_001190347 | DMKN | 0.105266828 | 0.537137409 | 1.3671723 | down | mRNA |
| NM_001190383 | GNE | 0.351630066 | 0.835247013 | 1.2230109 | down | mRNA |
| NM_001190384 | GNE | 0.025362016 | 0.312332229 | 1.447165 | down | mRNA |
| NM_001190411 | RPH3AL | 0.78953329 | 0.978053425 | 1.0397425 | down | mRNA |
| NM_001190413 | RPH3AL | 0.494157163 | 0.906787313 | 1.2438586 | down | mRNA |
| NM_001190438 | NCOR1 | 0.656296353 | 0.957440295 | 1.0476008 | down | mRNA |
| NM_001190440 | NCOR1 | 0.009150789 | 0.203383174 | 1.2847681 | down | mRNA |
| NM_001190442 | CAST | 0.801394803 | 0.98118927 | 1.0097683 | down | mRNA |
| NM_001190447 | PPP2R3A | 0.702158756 | 0.963247543 | 1.0302324 | down | mRNA |
| NM_001190458 | DOCK8 | 0.743418353 | 0.970330526 | 1.128027 | down | mRNA |
| NM_001190468 | GDNF | 0.557523774 | 0.926833616 | 1.1118932 | down | mRNA |
| NM_001190469 | GDNF | 0.508457097 | 0.911674633 | 1.2994415 | down | mRNA |
| NM_001190707 | ALS2CL | 0.636975955 | 0.952419115 | 1.055909 | down | mRNA |
| NM_001190796 | NCK1 | 0.019989761 | 0.283521851 | 1.6147864 | down | mRNA |
| NM_001190799 | PLK4 | 0.856172885 | 0.988315074 | 1.1095044 | down | mRNA |
| NM_001190801 | PLK4 | 0.901219099 | 0.994819845 | 1.1229244 | down | mRNA |
| NM_001190836 | DCTN1 | 0.117381613 | 0.564147103 | 1.1195759 | down | mRNA |
| NM_001190847 | CNOT4 | 0.104305501 | 0.534999858 | 1.0652127 | down | mRNA |
| NM_001190849 | CNOT4 | 0.122365263 | 0.57455499 | 1.3320009 | down | mRNA |
| NM_001190864 | MRPS35 | 0.117404745 | 0.564147103 | 1.1286071 | down | mRNA |
| NM_001190880 | HYI | 0.680784132 | 0.960111041 | 1.0645696 | down | mRNA |
| NM_001190881 | ORC4 | 0.449823846 | 0.888279993 | 1.0755924 | down | mRNA |
| NM_001190882 | ORC4 | 0.641707339 | 0.954332328 | 1.0467534 | down | mRNA |
| NM_001190899 | PDYN | 0.559246909 | 0.927203296 | 1.1133105 | down | mRNA |
| NM_001190906 | AKR1D1 | 0.633337598 | 0.951174475 | 1.0705058 | down | mRNA |
| NM_001190946 | FAM193B | 0.938325523 | 0.996710991 | 1.0084169 | down | mRNA |
| NM_001190979 | YAF2 | 0.858673464 | 0.988512246 | 1.0048761 | down | mRNA |
| NM_001190983 | COQ7 | 0.735076018 | 0.96794858 | 1.0210647 | down | mRNA |
| NM_001190987 | SRSF11 | 0.43228386 | 0.880427272 | 1.0289325 | down | mRNA |
| NM_001190995 | IPO8 | 0.277461124 | 0.777234326 | 1.1858384 | down | mRNA |
| NM_001190997 | SLC6A13 | 0.252460279 | 0.752621656 | 1.1737451 | down | mRNA |
| NM_001191 | BCL2L1 | 0.577669573 | 0.933787388 | 1.0790775 | down | mRNA |
| NM_001191002 | GSTO1 | 0.749933102 | 0.970725371 | 1.0366175 | down | mRNA |
| NM_001191005 | SRSF10 | 0.175086705 | 0.653821873 | 1.0864583 | down | mRNA |
| NM_001191006 | SRSF10 | 0.015381002 | 0.248788014 | 1.2399253 | down | mRNA |
| NM_001191007 | SRSF10 | 0.276081181 | 0.775838072 | 1.0648493 | down | mRNA |
| NM_001191009 | SRSF10 | 0.672076073 | 0.959531882 | 1.030868 | down | mRNA |
| NM_001191013 | GSTO2 | 0.50833142 | 0.911674633 | 1.1823553 | down | mRNA |
| NM_001191014 | GSTO2 | 0.398596382 | 0.863643939 | 1.1951648 | down | mRNA |
| NM_001191022 | CTCF | 0.000951055 | 0.084526081 | 1.2809188 | down | mRNA |
| NM_001191028 | TNPO3 | 0.00324805 | 0.134059627 | 1.4766377 | down | mRNA |
| NM_001191029 | C17orf72 | 0.775468421 | 0.97589756 | 1.0576752 | down | mRNA |
| NM_001191031 | C17orf72 | 0.882474223 | 0.992766819 | 1.0162788 | down | mRNA |
| NM_001191036 | TRIQK | 0.751749325 | 0.970725371 | 1.0244161 | down | mRNA |
| NM_001191037 | PSMD1 | 0.683971412 | 0.960111041 | 1.0388394 | down | mRNA |
| NM_001191055 | ERVV-2 | 0.851254398 | 0.987678166 | 1.0136555 | down | mRNA |
| NM_001191059 | PDE1C | 0.101914483 | 0.52995944 | 1.1642029 | down | mRNA |
| NM_001191060 | SLC25A22 | 0.974823882 | 0.998501532 | 1.002775 | down | mRNA |
| NM_001193267 | SIRT5 | 0.687079221 | 0.960205549 | 1.0403472 | down | mRNA |
| NM_001193269 | EML2 | 0.163619804 | 0.636236851 | 1.3441924 | down | mRNA |
| NM_001193272 | RBBP5 | 0.202930923 | 0.692396631 | 1.8581683 | down | mRNA |
| NM_001193285 | SIRT6 | 0.571085494 | 0.933029103 | 1.073168 | down | mRNA |
| NM_001193308 | SYTL1 | 0.393527665 | 0.860252069 | 1.0626138 | down | mRNA |
| NM_001193312 | C7orf10 | 0.753669224 | 0.970725371 | 1.0402317 | down | mRNA |
| NM_001193313 | C7orf10 | 0.927584881 | 0.995829678 | 1.0218151 | down | mRNA |
| NM_001193316 | VIPAS39 | 0.01508233 | 0.246308378 | 1.4286646 | down | mRNA |
| NM_001193317 | VIPAS39 | 0.304865625 | 0.800533111 | 1.4712337 | down | mRNA |
| NM_001193318 | RNF212 | 0.999849614 | 0.999954265 | 1.0001671 | down | mRNA |
| NM_001193321 | IKBKE | 0.905118975 | 0.99508468 | 1.0232703 | down | mRNA |
| NM_001193328 | ZNF692 | 0.719041665 | 0.966191059 | 1.0502525 | down | mRNA |
| NM_001193333 | CORO1A | 0.805419581 | 0.98118927 | 1.0361988 | down | mRNA |
| NM_001193334 | WLS | 0.482642323 | 0.902654749 | 1.1980834 | down | mRNA |
| NM_001193336 | SEC14L6 | 0.62337995 | 0.948341211 | 1.095648 | down | mRNA |
| NM_001193341 | RSPH9 | 0.860480411 | 0.988975154 | 1.0146308 | down | mRNA |
| NM_001193343 | MRPS18A | 0.524172782 | 0.917428132 | 1.0274705 | down | mRNA |
| NM_001193348 | MEF2C | 0.717166888 | 0.966191059 | 1.025711 | down | mRNA |
| NM_001193361 | EXD2 | 0.007665841 | 0.193257016 | 1.7390253 | down | mRNA |
| NM_001193380 | IL17RE | 0.670669327 | 0.959531882 | 1.0356458 | down | mRNA |
| NM_001193426 | SUV39H2 | 0.273021229 | 0.772770327 | 1.2964728 | down | mRNA |
| NM_001193451 | TMTC1 | 0.729796144 | 0.966191059 | 1.046999 | down | mRNA |
| NM_001193455 | NSUN2 | 0.077558324 | 0.478499021 | 1.1804381 | down | mRNA |
| NM_001193475 | PPP1R21 | 0.139232645 | 0.598648178 | 1.210085 | down | mRNA |
| NM_001193482 | LIMS1 | 0.983067795 | 0.998971807 | 1.0038007 | down | mRNA |
| NM_001193484 | LIMS1 | 0.990226428 | 0.999170408 | 1.0010102 | down | mRNA |
| NM_001193485 | LIMS1 | 0.313550501 | 0.808703036 | 1.1891066 | down | mRNA |
| NM_001193487 | GTF2A1L | 0.407612998 | 0.867591329 | 1.2426653 | down | mRNA |
| NM_001193493 | SLC1A4 | 0.301435194 | 0.796566202 | 1.1088633 | down | mRNA |
| NM_001193495 | ADAR | 0.499455705 | 0.909451316 | 1.0331162 | down | mRNA |
| NM_001193513 | SLC30A6 | 0.623969779 | 0.948341211 | 1.0773331 | down | mRNA |
| NM_001193514 | SLC30A6 | 0.139853632 | 0.599515926 | 1.1143293 | down | mRNA |
| NM_001193515 | SLC30A6 | 0.247121697 | 0.745152522 | 1.2225427 | down | mRNA |
| NM_001193517 | CHMP3 | 0.958379444 | 0.997020613 | 1.0056883 | down | mRNA |
| NM_001193528 | SCRN3 | 0.149048863 | 0.612750316 | 1.2466997 | down | mRNA |
| NM_001193529 | CCT6B | 0.697793514 | 0.962410025 | 1.1386139 | down | mRNA |
| NM_001193533 | NEK4 | 0.137793227 | 0.596789644 | 1.1448157 | down | mRNA |
| NM_001193535 | FBXL5 | 0.1665089 | 0.641349924 | 1.4091281 | down | mRNA |
| NM_001193538 | TMEM126B | 0.595262413 | 0.938707475 | 1.0415419 | down | mRNA |
| NM_001193544 | ANXA6 | 0.048296903 | 0.398851601 | 1.1557402 | down | mRNA |
| NM_001193599 | SCP2 | 0.852525256 | 0.987678166 | 1.0307482 | down | mRNA |
| NM_001193609 | TUBD1 | 0.2012114 | 0.689305844 | 1.1967371 | down | mRNA |
| NM_001193610 | TUBD1 | 0.053915715 | 0.416977409 | 1.1931892 | down | mRNA |
| NM_001193611 | TUBD1 | 0.127671474 | 0.580991132 | 1.1520728 | down | mRNA |
| NM_001193617 | SCP2 | 0.646693643 | 0.95550188 | 1.0948615 | down | mRNA |
| NM_001194 | HCN2 | 0.002781259 | 0.124831263 | 1.263557 | down | mRNA |
| NM_001194937 | POGZ | 0.34958875 | 0.834138149 | 1.0792976 | down | mRNA |
| NM_001194938 | POGZ | 0.265387838 | 0.766537754 | 1.0899967 | down | mRNA |
| NM_001194955 | MATR3 | 0.626560148 | 0.949203235 | 1.0535247 | down | mRNA |
| NM_001194990 | MEPCE | 0.022948998 | 0.298169644 | 1.2376062 | down | mRNA |
| NM_001194999 | MR1 | 0.464123708 | 0.894989844 | 1.073428 | down | mRNA |
| NM_001195000 | MR1 | 0.338823706 | 0.827403747 | 1.1288666 | down | mRNA |
| NM_001195032 | C6orf99 | 0.912010229 | 0.995342392 | 1.0401272 | down | mRNA |
| NM_001195035 | MR1 | 0.753404838 | 0.970725371 | 1.021293 | down | mRNA |
| NM_001195044 | YAP1 | 0.309461803 | 0.803961878 | 1.1059131 | down | mRNA |
| NM_001195045 | YAP1 | 0.292726377 | 0.789666008 | 1.1245673 | down | mRNA |
| NM_001195081 | LOC100288814 | 0.44829064 | 0.887969953 | 1.0651937 | down | mRNA |
| NM_001195101 | PTP4A2 | 0.359434996 | 0.841033672 | 1.0921437 | down | mRNA |
| NM_001195125 | C16orf95 | 0.551628847 | 0.926153403 | 1.0348336 | down | mRNA |
| NM_001195131 | TSTD3 | 0.45685761 | 0.891568474 | 1.0645759 | down | mRNA |
| NM_001195135 | LOC646862 | 0.171781323 | 0.649522625 | 1.5075231 | down | mRNA |
| NM_001195304 | BBIP1 | 0.940170723 | 0.996710991 | 1.0059518 | down | mRNA |
| NM_001195518 | MICU1 | 0.028113547 | 0.326444685 | 1.1964717 | down | mRNA |
| NM_001195519 | MICU1 | 0.576393026 | 0.933656777 | 1.0932766 | down | mRNA |
| NM_001195528 | TPBGL | 0.80385579 | 0.98118927 | 1.0207496 | down | mRNA |
| NM_001195577 | TRPC5OS | 0.62918388 | 0.95027237 | 1.0695473 | down | mRNA |
| NM_001195578 | TRPC5OS | 0.738720383 | 0.969564098 | 1.0538097 | down | mRNA |
| NM_001195605 | ZNF865 | 0.094913657 | 0.516123157 | 1.179883 | down | mRNA |
| NM_001198 | PRDM1 | 0.780568161 | 0.976938981 | 1.1934032 | down | mRNA |
| NM_001198784 | FLJ27352 | 0.565325117 | 0.930020725 | 1.0760136 | down | mRNA |
| NM_001198815 | MUC22 | 0.42177025 | 0.873398499 | 1.2016927 | down | mRNA |
| NM_001199206 | FOLR4 | 0.069305286 | 0.456847153 | 1.1578543 | down | mRNA |
| NM_001199233 | LOC100505478 | 0.999175361 | 0.999897336 | 1.00009 | down | mRNA |
| NM_001199814 | ZNF812 | 0.215808004 | 0.710547135 | 1.1569518 | down | mRNA |
| NM_001204 | BMPR2 | 0.002612044 | 0.121807537 | 1.3533866 | down | mRNA |
| NM_001204210 | TMEM235 | 0.822037263 | 0.982983098 | 1.0334049 | down | mRNA |
| NM_001204211 | TMEM235 | 0.69862391 | 0.962701213 | 1.0171469 | down | mRNA |
| NM_001205 | BNIP1 | 0.847276565 | 0.987678166 | 1.0129682 | down | mRNA |
| NM_001205272 | KRBOX1 | 0.866673139 | 0.989481868 | 1.0404581 | down | mRNA |
| NM_001205273 | THEG5 | 0.961602306 | 0.99793927 | 1.0111073 | down | mRNA |
| NM_001206650 | PSG7 | 0.754152512 | 0.970944772 | 1.137466 | down | mRNA |
| NM_001206747 | CAV2 | 0.813463072 | 0.98120545 | 1.0481249 | down | mRNA |
| NM_001206847 | LOC100507341 | 0.867445797 | 0.989481868 | 1.0367886 | down | mRNA |
| NM_001207043 | LOC554223 | 0.88257992 | 0.992766819 | 1.023639 | down | mRNA |
| NM_001207044 | CLASP2 | 0.804024137 | 0.98118927 | 1.0437312 | down | mRNA |
| NM_001207052 | TMEM191C | 0.476322778 | 0.900415348 | 1.2081286 | down | mRNA |
| NM_001211 | BUB1B | 0.648329568 | 0.95550188 | 1.3681812 | down | mRNA |
| NM_001212 | C1QBP | 0.610113467 | 0.943169861 | 1.0670744 | down | mRNA |
| NM_001219 | CALU | 0.551352254 | 0.926149092 | 1.1320704 | down | mRNA |
| NM_001220475 | PPFIA2 | 0.41812775 | 0.871927185 | 1.0550709 | down | mRNA |
| NM_001220768 | IKZF1 | 0.257448315 | 0.757735833 | 1.2210467 | down | mRNA |
| NM_001221 | CAMK2D | 0.444253294 | 0.885088246 | 1.0797256 | down | mRNA |
| NM_001222 | CAMK2G | 0.038601334 | 0.366748746 | 1.3489396 | down | mRNA |
| NM_001226 | CASP6 | 0.448002558 | 0.88788264 | 1.1265196 | down | mRNA |
| NM_001229 | CASP9 | 0.858434698 | 0.988512246 | 1.0205212 | down | mRNA |
| NM_001235 | SERPINH1 | 0.821387734 | 0.982772998 | 1.0128115 | down | mRNA |
| NM_001237 | CCNA2 | 0.420135153 | 0.872365335 | 1.4734521 | down | mRNA |
| NM_001239 | CCNH | 0.027769145 | 0.32381669 | 1.2591816 | down | mRNA |
| NM_001240 | CCNT1 | 0.392069679 | 0.859461046 | 1.1185002 | down | mRNA |
| NM_001242313 | TMEM191B | 0.071687083 | 0.462237305 | 1.5161455 | down | mRNA |
| NM_001242607 | NCAM1 | 0.713942346 | 0.966110889 | 1.0755156 | down | mRNA |
| NM_001242640 | BRCC3 | 0.415639902 | 0.870382888 | 1.1080741 | down | mRNA |
| NM_001242659 | C1orf233 | 0.383633801 | 0.853956914 | 1.0496645 | down | mRNA |
| NM_001242699 | ENO4 | 0.50167236 | 0.910145661 | 1.8970911 | down | mRNA |
| NM_001242704 | ZNF385C | 0.06304592 | 0.439769486 | 1.0817234 | down | mRNA |
| NM_001242740 | LOC100507462 | 0.382291487 | 0.853514109 | 1.3293452 | down | mRNA |
| NM_001242757 | SLC22A31 | 0.945121884 | 0.996710991 | 1.0151818 | down | mRNA |
| NM_001242780 | LOC100506388 | 0.772093847 | 0.975347132 | 1.0382191 | down | mRNA |
| NM_001242956 | SEPT7 | 0.31024913 | 0.805036317 | 1.0309656 | down | mRNA |
| NM_001243212 | LOC643669 | 0.771503832 | 0.975347132 | 1.0941663 | down | mRNA |
| NM_001243237 | LOC100130301 | 0.955656394 | 0.996883425 | 1.0132162 | down | mRNA |
| NM_001243248 | NPRL3 | 0.358538169 | 0.840603483 | 1.0970399 | down | mRNA |
| NM_001243249 | NPRL3 | 0.396493639 | 0.861995469 | 1.1096556 | down | mRNA |
| NM_001243523 | LOC100130880 | 0.378578199 | 0.852220101 | 1.2647258 | down | mRNA |
| NM_001243531 | LOC100505679 | 0.963256812 | 0.99793927 | 1.0118796 | down | mRNA |
| NM_001243535 | ANKRD65 | 0.240168285 | 0.738437735 | 1.164331 | down | mRNA |
| NM_001243537 | LOC388849 | 0.177995101 | 0.65859807 | 1.318098 | down | mRNA |
| NM_001243783 | BBIP1 | 0.882704547 | 0.992766819 | 1.0095595 | down | mRNA |
| NM_001243812 | CACNA1B | 0.332270426 | 0.822707947 | 1.306083 | down | mRNA |
| NM_001245 | SIGLEC6 | 0.761225602 | 0.973219662 | 1.0543574 | down | mRNA |
| NM_001252402 | TMEM249 | 0.567375516 | 0.931514776 | 1.0682789 | down | mRNA |
| NM_001253790 | LOC440335 | 0.833743042 | 0.986475536 | 1.0934103 | down | mRNA |
| NM_001254 | CDC6 | 0.639936728 | 0.953734314 | 1.2511807 | down | mRNA |
| NM_001256 | CDC27 | 0.829307148 | 0.985532347 | 1.0029889 | down | mRNA |
| NM_001256408 | LOC158434 | 0.087247257 | 0.500533133 | 1.1029127 | down | mRNA |
| NM_001256857 | LOC100287327 | 0.138407548 | 0.597943156 | 1.4466239 | down | mRNA |
| NM_001256932 | LOC730183 | 0.847475807 | 0.987678166 | 1.0196033 | down | mRNA |
| NM_001257995 | LOC729420 | 0.011469805 | 0.218765541 | 2.6035061 | down | mRNA |
| NM_001258000 | LOC100130539 | 0.160624552 | 0.632017029 | 1.3868005 | down | mRNA |
| NM_001260 | CDK8 | 0.013441697 | 0.235533862 | 1.5375325 | down | mRNA |
| NM_001261 | CDK9 | 0.020185823 | 0.284617308 | 1.1894734 | down | mRNA |
| NM_001263 | CDS1 | 0.711962592 | 0.96592341 | 1.031742 | down | mRNA |
| NM_001267536 | GOLGA6L4 | 0.03647848 | 0.358673526 | 1.2130249 | down | mRNA |
| NM_001267716 | ZNF728 | 0.046172067 | 0.391970965 | 1.2313217 | down | mRNA |
| NM_001276 | CHI3L1 | 0.644903233 | 0.954749596 | 1.035183 | down | mRNA |
| NM_001277 | CHKA | 0.030988357 | 0.340185047 | 1.5971266 | down | mRNA |
| NM_001278 | CHUK | 0.087677005 | 0.501313828 | 1.1307597 | down | mRNA |
| NM_001281 | TBCB | 0.741158389 | 0.970205535 | 1.0366221 | down | mRNA |
| NM_001282 | AP2B1 | 0.07226327 | 0.463255488 | 1.1032526 | down | mRNA |
| NM_001283 | AP1S1 | 0.980320023 | 0.998971807 | 1.0060975 | down | mRNA |
| NM_001285 | CLCA1 | 0.815800661 | 0.981459009 | 1.0700377 | down | mRNA |
| NM_001288 | CLIC1 | 0.05661941 | 0.424009517 | 1.0696715 | down | mRNA |
| NM_001290 | LDB2 | 0.712701505 | 0.966110889 | 1.1434281 | down | mRNA |
| NM_001293 | CLNS1A | 0.279290459 | 0.777816764 | 1.1243345 | down | mRNA |
| NM_001294 | CLPTM1 | 0.866809615 | 0.989481868 | 1.0402469 | down | mRNA |
| NM_001303 | COX10 | 0.788460517 | 0.977641695 | 1.0165654 | down | mRNA |
| NM_001304 | CPD | 0.294415314 | 0.791301949 | 1.1026249 | down | mRNA |
| NM_001306 | CLDN3 | 0.012874821 | 0.231450797 | 1.4703755 | down | mRNA |
| NM_001308 | CPN1 | 0.998139488 | 0.999889342 | 1.0001946 | down | mRNA |
| NM_001310 | CREBL2 | 0.608961993 | 0.943053241 | 1.0617538 | down | mRNA |
| NM_001311 | CRIP1 | 0.771834978 | 0.975347132 | 1.0708361 | down | mRNA |
| NM_001312 | CRIP2 | 0.185760462 | 0.669321303 | 1.0473633 | down | mRNA |
| NM_001313 | CRMP1 | 0.783026158 | 0.977275912 | 1.0652729 | down | mRNA |
| NM_001315 | MAPK14 | 0.437736192 | 0.882591456 | 1.0347932 | down | mRNA |
| NM_001316 | CSE1L | 0.465180342 | 0.895224488 | 1.1261708 | down | mRNA |
| NM_001319 | CSNK1G2 | 0.64763738 | 0.95550188 | 1.0361043 | down | mRNA |
| NM_001322 | CST2 | 0.618762984 | 0.94711754 | 1.1417167 | down | mRNA |
| NM_001323 | CST6 | 0.231065429 | 0.728488496 | 1.6770674 | down | mRNA |
| NM_001324 | CSTF1 | 0.007331432 | 0.190938402 | 1.4246806 | down | mRNA |
| NM_001325 | CSTF2 | 0.553253916 | 0.926337111 | 1.100729 | down | mRNA |
| NM_001326 | CSTF3 | 0.981320855 | 0.998971807 | 1.0020551 | down | mRNA |
| NM_001332 | CTNND2 | 0.410842999 | 0.868341245 | 1.1978717 | down | mRNA |
| NM_001334 | CTSO | 0.521218568 | 0.916644975 | 1.1233385 | down | mRNA |
| NM_001336 | CTSZ | 0.133690613 | 0.59039912 | 1.2073203 | down | mRNA |
| NM_001343 | DAB2 | 0.964369582 | 0.99793927 | 1.0117031 | down | mRNA |
| NM_001344 | DAD1 | 0.667198004 | 0.958354006 | 1.0322089 | down | mRNA |
| NM_001347 | DGKQ | 0.84348177 | 0.987338076 | 1.018002 | down | mRNA |
| NM_001349 | DARS | 0.443419756 | 0.884425653 | 1.0590122 | down | mRNA |
| NM_001353 | AKR1C1 | 0.307395513 | 0.802168809 | 1.17121 | down | mRNA |
| NM_001356 | DDX3X | 0.667326596 | 0.958354006 | 1.0197767 | down | mRNA |
| NM_001358 | DHX15 | 0.18774399 | 0.672162493 | 1.1269249 | down | mRNA |
| NM_001360 | DHCR7 | 0.982335993 | 0.998971807 | 1.0074401 | down | mRNA |
| NM_001361 | DHODH | 0.431987572 | 0.880287969 | 1.0931814 | down | mRNA |
| NM_001376 | DYNC1H1 | 0.24025261 | 0.738437735 | 1.2837102 | down | mRNA |
| NM_001378 | DYNC1I2 | 0.108909482 | 0.546347791 | 1.0492021 | down | mRNA |
| NM_001381 | DOK1 | 0.180937004 | 0.664045338 | 1.587651 | down | mRNA |
| NM_001382 | DPAGT1 | 0.189191483 | 0.674719659 | 1.1134877 | down | mRNA |
| NM_001383 | DPH1 | 0.161919863 | 0.633838016 | 1.4891316 | down | mRNA |
| NM_001384 | DPH2 | 0.450134799 | 0.888463871 | 1.0429323 | down | mRNA |
| NM_001387 | DPYSL3 | 0.663195095 | 0.958354006 | 1.0801225 | down | mRNA |
| NM_001388 | DRG2 | 0.271607635 | 0.771734007 | 1.1748175 | down | mRNA |
| NM_001394 | DUSP4 | 0.163690486 | 0.636236851 | 2.1039105 | down | mRNA |
| NM_001396 | DYRK1A | 0.303995459 | 0.799797576 | 1.1009107 | down | mRNA |
| NM_001401 | LPAR1 | 0.027748173 | 0.32381669 | 1.1468592 | down | mRNA |
| NM_001402 | EEF1A1 | 0.484857249 | 0.903250293 | 1.1432273 | down | mRNA |
| NM_001407 | CELSR3 | 0.469839714 | 0.897941557 | 1.0858664 | down | mRNA |
| NM_001412 | EIF1AX | 0.221599011 | 0.716734257 | 1.0726226 | down | mRNA |
| NM_001414 | EIF2B1 | 0.055900133 | 0.421647851 | 1.1271228 | down | mRNA |
| NM_001416 | EIF4A1 | 0.617332648 | 0.946433977 | 1.1458229 | down | mRNA |
| NM_001417 | EIF4B | 0.692072302 | 0.961451327 | 1.0859535 | down | mRNA |
| NM_001419 | ELAVL1 | 0.095247356 | 0.516597924 | 1.1239802 | down | mRNA |
| NM_001423 | EMP1 | 0.368716488 | 0.845559574 | 1.1282529 | down | mRNA |
| NM_001428 | ENO1 | 0.480998959 | 0.901538317 | 1.0713003 | down | mRNA |
| NM_001429 | EP300 | 1.17954E-05 | 0.015988243 | 1.2888656 | down | mRNA |
| NM_001431 | EPB41L2 | 0.597910371 | 0.940150767 | 1.0462872 | down | mRNA |
| NM_001436 | FBL | 0.192213373 | 0.678323006 | 1.0875876 | down | mRNA |
| NM_001439 | EXTL2 | 0.392265188 | 0.859493185 | 1.0791444 | down | mRNA |
| NM_001444 | FABP5 | 0.71704869 | 0.966191059 | 1.1712121 | down | mRNA |
| NM_001446 | FABP7 | 0.161470804 | 0.633496168 | 1.4397747 | down | mRNA |
| NM_001448 | GPC4 | 0.824751584 | 0.983798721 | 1.0197072 | down | mRNA |
| NM_001466 | FZD2 | 0.530258686 | 0.91899174 | 1.3476339 | down | mRNA |
| NM_001469 | XRCC6 | 0.755931683 | 0.971522741 | 1.0288894 | down | mRNA |
| NM_001470 | GABBR1 | 0.696158182 | 0.962410025 | 1.0464527 | down | mRNA |
| NM_001483 | GBAS | 0.760413368 | 0.972923327 | 1.0526201 | down | mRNA |
| NM_001487 | BLOC1S1 | 0.891925968 | 0.9937521 | 1.0064964 | down | mRNA |
| NM_001488 | TADA2A | 0.062201869 | 0.439379649 | 1.1859864 | down | mRNA |
| NM_001489 | NR6A1 | 0.080361226 | 0.484119838 | 1.357741 | down | mRNA |
| NM_001490 | GCNT1 | 0.113670089 | 0.55757304 | 1.6582793 | down | mRNA |
| NM_001498 | GCLC | 0.024990081 | 0.308874358 | 1.4349038 | down | mRNA |
| NM_001499 | GLE1 | 0.52453258 | 0.917632259 | 1.0989885 | down | mRNA |
| NM_001500 | GMDS | 0.204885122 | 0.694868044 | 1.0933502 | down | mRNA |
| NM_001502 | GP2 | 0.422030179 | 0.873444381 | 1.1989175 | down | mRNA |
| NM_001503 | GPLD1 | 0.00795457 | 0.195093254 | 1.5007986 | down | mRNA |
| NM_001512 | GSTA4 | 0.778908973 | 0.976938981 | 1.0180578 | down | mRNA |
| NM_001513 | GSTZ1 | 0.954645583 | 0.996834354 | 1.0030679 | down | mRNA |
| NM_001514 | GTF2B | 0.781744585 | 0.976938981 | 1.0253795 | down | mRNA |
| NM_001515 | GTF2H2 | 0.821135748 | 0.982756843 | 1.0580822 | down | mRNA |
| NM_001516 | GTF2H3 | 0.606377733 | 0.941998171 | 1.0598153 | down | mRNA |
| NM_001517 | GTF2H4 | 0.055930762 | 0.421647851 | 1.0864946 | down | mRNA |
| NM_001518 | GTF2I | 0.07176432 | 0.462237305 | 1.1315873 | down | mRNA |
| NM_001520 | GTF3C1 | 0.904603242 | 0.99508468 | 1.0159251 | down | mRNA |
| NM_001530 | HIF1A | 0.343115164 | 0.831521302 | 1.1232074 | down | mRNA |
| NM_001531 | MR1 | 0.4865256 | 0.903524001 | 1.0282192 | down | mRNA |
| NM_001532 | SLC29A2 | 0.975531313 | 0.998501532 | 1.0050879 | down | mRNA |
| NM_001533 | HNRNPL | 0.545673357 | 0.924167487 | 1.0923794 | down | mRNA |
| NM_001535 | PRMT2 | 0.480920881 | 0.901538317 | 1.2683805 | down | mRNA |
| NM_001536 | PRMT1 | 0.609687962 | 0.943053241 | 1.0471771 | down | mRNA |
| NM_001537 | HSBP1 | 0.728842438 | 0.966191059 | 1.0224592 | down | mRNA |
| NM_001540 | HSPB1 | 0.707300964 | 0.965401045 | 1.0819851 | down | mRNA |
| NM_001541 | HSPB2 | 0.91827253 | 0.995349793 | 1.0121296 | down | mRNA |
| NM_001548 | IFIT1 | 0.995698768 | 0.999872237 | 1.001794 | down | mRNA |
| NM_001549 | IFIT3 | 0.466635976 | 0.89599965 | 1.0644597 | down | mRNA |
| NM_001550 | IFRD1 | 0.029414733 | 0.333925382 | 1.4212737 | down | mRNA |
| NM_001551 | IGBP1 | 0.445896155 | 0.886251358 | 1.1613912 | down | mRNA |
| NM_001554 | CYR61 | 0.898741889 | 0.99453218 | 1.0224349 | down | mRNA |
| NM_001560 | IL13RA1 | 0.972839304 | 0.998501532 | 1.0024844 | down | mRNA |
| NM_001564 | ING2 | 0.018096871 | 0.269173581 | 1.252233 | down | mRNA |
| NM_001566 | INPP4A | 0.034968456 | 0.352396712 | 1.7501431 | down | mRNA |
| NM_001567 | INPPL1 | 0.070090944 | 0.458080705 | 1.1122365 | down | mRNA |
| NM_001568 | EIF3E | 0.141315892 | 0.601679402 | 1.0632955 | down | mRNA |
| NM_001571 | IRF3 | 0.401556044 | 0.864768873 | 1.1344503 | down | mRNA |
| NM_001587 | OCRL | 0.096076561 | 0.51782059 | 1.1559302 | down | mRNA |
| NM_001605 | AARS | 0.171392237 | 0.648928672 | 1.1590821 | down | mRNA |
| NM_001614 | ACTG1 | 0.066519764 | 0.450298982 | 1.1574276 | down | mRNA |
| NM_001615 | ACTG2 | 0.481278007 | 0.901538317 | 1.165079 | down | mRNA |
| NM_001616 | ACVR2A | 0.057092107 | 0.425938435 | 1.2653098 | down | mRNA |
| NM_001618 | PARP1 | 0.033097406 | 0.346127643 | 1.3147934 | down | mRNA |
| NM_001619 | ADRBK1 | 0.141205307 | 0.601673229 | 1.155223 | down | mRNA |
| NM_001620 | AHNAK | 0.252086917 | 0.752621656 | 1.0674659 | down | mRNA |
| NM_001632 | ALPP | 0.640849233 | 0.953894897 | 1.0350463 | down | mRNA |
| NM_001634 | AMD1 | 0.855500185 | 0.988178307 | 1.0397967 | down | mRNA |
| NM_001636 | SLC25A6 | 0.540450531 | 0.923577434 | 1.1045677 | down | mRNA |
| NM_001638 | APOF | 0.806526405 | 0.98118927 | 1.0346983 | down | mRNA |
| NM_001640 | APEH | 0.459277796 | 0.892904132 | 1.0803569 | down | mRNA |
| NM_001642 | APLP2 | 0.085421635 | 0.497360491 | 1.0800502 | down | mRNA |
| NM_001643 | APOA2 | 0.83543474 | 0.986575022 | 1.044315 | down | mRNA |
| NM_001646 | APOC4 | 0.734916723 | 0.967892655 | 1.03584 | down | mRNA |
| NM_001654 | ARAF | 0.093844679 | 0.513134818 | 1.2002068 | down | mRNA |
| NM_001655 | ARCN1 | 0.132935305 | 0.588930122 | 1.0756583 | down | mRNA |
| NM_001656 | TRIM23 | 0.044853689 | 0.38849334 | 1.3014405 | down | mRNA |
| NM_001657 | AREG | 0.862566699 | 0.989401142 | 1.1189994 | down | mRNA |
| NM_001659 | ARF3 | 0.415000457 | 0.870239791 | 1.0779324 | down | mRNA |
| NM_001660 | ARF4 | 0.484267336 | 0.903250293 | 1.0610563 | down | mRNA |
| NM_001661 | ARL4D | 0.810911889 | 0.98118927 | 1.0316632 | down | mRNA |
| NM_001662 | ARF5 | 0.72051891 | 0.966191059 | 1.0401412 | down | mRNA |
| NM_001663 | ARF6 | 0.789051735 | 0.977807732 | 1.0141591 | down | mRNA |
| NM_001664 | RHOA | 0.810714289 | 0.98118927 | 1.0444638 | down | mRNA |
| NM_001667 | ARL2 | 0.796549332 | 0.98065038 | 1.031501 | down | mRNA |
| NM_001669 | ARSD | 0.580656195 | 0.93393768 | 1.0670809 | down | mRNA |
| NM_001671 | ASGR1 | 0.008821094 | 0.202329498 | 1.3651922 | down | mRNA |
| NM_001675 | ATF4 | 0.655261444 | 0.957222998 | 1.0625013 | down | mRNA |
| NM_001677 | ATP1B1 | 0.218430242 | 0.712320363 | 1.277436 | down | mRNA |
| NM_001678 | ATP1B2 | 0.448749302 | 0.888279993 | 1.0782716 | down | mRNA |
| NM_001679 | ATP1B3 | 0.410306763 | 0.86807846 | 1.3538742 | down | mRNA |
| NM_001681 | ATP2A2 | 0.144333325 | 0.605523499 | 1.4096085 | down | mRNA |
| NM_001687 | ATP5D | 0.03931633 | 0.36974081 | 1.1150286 | down | mRNA |
| NM_001688 | ATP5F1 | 0.526886893 | 0.917975454 | 1.0374875 | down | mRNA |
| NM_001690 | ATP6V1A | 0.254300951 | 0.75479352 | 1.1547713 | down | mRNA |
| NM_001692 | ATP6V1B1 | 0.950834037 | 0.996710991 | 1.007422 | down | mRNA |
| NM_001695 | ATP6V1C1 | 0.084001186 | 0.493254641 | 1.1622335 | down | mRNA |
| NM_001696 | ATP6V1E1 | 0.415500451 | 0.870382888 | 1.034326 | down | mRNA |
| NM_001698 | AUH | 0.569940167 | 0.932738884 | 1.0971231 | down | mRNA |
| NM_001701 | BAAT | 0.11625585 | 0.562521166 | 1.1052528 | down | mRNA |
| NM_001703 | BAI2 | 0.555173045 | 0.926833616 | 1.0367279 | down | mRNA |
| NM_001707 | BCL7B | 0.284597319 | 0.78179061 | 1.0611648 | down | mRNA |
| NM_001711 | BGN | 0.90326976 | 0.99508468 | 1.0131923 | down | mRNA |
| NM_001714 | BICD1 | 0.127848156 | 0.581220503 | 1.4196434 | down | mRNA |
| NM_001716 | CXCR5 | 0.166578006 | 0.641440015 | 1.4106009 | down | mRNA |
| NM_001718 | BMP6 | 0.837199092 | 0.986775289 | 1.094458 | down | mRNA |
| NM_001720 | BMP8B | 0.12976063 | 0.583907255 | 1.1177857 | down | mRNA |
| NM_001723 | DST | 0.001300025 | 0.093126586 | 1.329018 | down | mRNA |
| NM_001737 | C9 | 0.516678739 | 0.914937059 | 1.2670057 | down | mRNA |
| NM_001741 | CALCA | 0.324929495 | 0.817191271 | 1.0468966 | down | mRNA |
| NM_001745 | CAMLG | 0.300752891 | 0.795797471 | 1.1093418 | down | mRNA |
| NM_001747 | CAPG | 0.906112086 | 0.99508468 | 1.0516623 | down | mRNA |
| NM_001755 | CBFB | 0.008468529 | 0.198595296 | 1.5727594 | down | mRNA |
| NM_001757 | CBR1 | 0.07811335 | 0.480109018 | 1.0879565 | down | mRNA |
| NM_001762 | CCT6A | 0.716038022 | 0.966191059 | 1.0454803 | down | mRNA |
| NM_001766 | CD1D | 0.281263013 | 0.779126326 | 1.1789631 | down | mRNA |
| NM_001770 | CD19 | 0.767625621 | 0.975033576 | 1.0398252 | down | mRNA |
| NM_001774 | CD37 | 0.717496551 | 0.966191059 | 1.0483782 | down | mRNA |
| NM_001777 | CD47 | 0.648067274 | 0.95550188 | 1.021107 | down | mRNA |
| NM_001780 | CD63 | 0.959811793 | 0.997693936 | 1.0092822 | down | mRNA |
| NM_001784 | CD97 | 0.321850632 | 0.815271211 | 1.3983189 | down | mRNA |
| NM_001789 | CDC25A | 0.539401681 | 0.923577434 | 1.5335831 | down | mRNA |
| NM_001797 | CDH11 | 0.31183389 | 0.806920827 | 1.1556936 | down | mRNA |
| NM_001799 | CDK7 | 0.68626487 | 0.960205549 | 1.033744 | down | mRNA |
| NM_001806 | CEBPG | 0.677902248 | 0.959531882 | 1.0391882 | down | mRNA |
| NM_001809 | CENPA | 0.353331033 | 0.836312754 | 1.4377236 | down | mRNA |
| NM_001812 | CENPC1 | 0.402413756 | 0.864786348 | 1.1820049 | down | mRNA |
| NM_001821 | CHML | 0.558927805 | 0.927153474 | 1.1142796 | down | mRNA |
| NM_001829 | CLCN3 | 0.02198759 | 0.292481125 | 1.1410403 | down | mRNA |
| NM_001830 | CLCN4 | 0.344582482 | 0.831973758 | 1.0796664 | down | mRNA |
| NM_001833 | CLTA | 0.072744921 | 0.463915802 | 1.1158177 | down | mRNA |
| NM_001834 | CLTB | 0.943280342 | 0.996710991 | 1.0113103 | down | mRNA |
| NM_001835 | CLTCL1 | 0.588274515 | 0.93579682 | 1.1066075 | down | mRNA |
| NM_001839 | CNN3 | 0.224892219 | 0.719853368 | 1.1290844 | down | mRNA |
| NM_001842 | CNTFR | 0.453188231 | 0.889563297 | 1.0288065 | down | mRNA |
| NM_001846 | COL4A2 | 0.306855364 | 0.801822806 | 1.0882314 | down | mRNA |
| NM_001847 | COL4A6 | 0.422945381 | 0.873989281 | 1.0192205 | down | mRNA |
| NM_001849 | COL6A2 | 0.029412662 | 0.333925382 | 1.240201 | down | mRNA |
| NM_001852 | COL9A2 | 0.990878498 | 0.999376923 | 1.0031323 | down | mRNA |
| NM_001854 | COL11A1 | 0.931121646 | 0.996710991 | 1.1012628 | down | mRNA |
| NM_001859 | SLC31A1 | 0.014552424 | 0.243322267 | 1.2071268 | down | mRNA |
| NM_001861 | COX4I1 | 0.896762529 | 0.994222243 | 1.0091587 | down | mRNA |
| NM_001866 | COX7B | 0.862185605 | 0.989401142 | 1.0148353 | down | mRNA |
| NM_001867 | COX7C | 0.504619208 | 0.910568383 | 1.0252454 | down | mRNA |
| NM_001871 | CPB1 | 0.897771733 | 0.994326043 | 1.0076507 | down | mRNA |
| NM_001876 | CPT1A | 0.818829734 | 0.982093332 | 1.0663646 | down | mRNA |
| NM_001878 | CRABP2 | 0.156601195 | 0.625226111 | 1.1010528 | down | mRNA |
| NM_001881 | CREM | 0.285371329 | 0.781963593 | 1.045038 | down | mRNA |
| NM_001884 | HAPLN1 | 0.938532843 | 0.996710991 | 1.0726127 | down | mRNA |
| NM_001891 | CSN2 | 0.020822219 | 0.286633283 | 1.480556 | down | mRNA |
| NM_001893 | CSNK1D | 0.099643501 | 0.524450342 | 1.0771994 | down | mRNA |
| NM_001896 | CSNK2A2 | 0.082737785 | 0.490806175 | 1.1863204 | down | mRNA |
| NM_001900 | CST5 | 0.230826002 | 0.728071705 | 1.9460997 | down | mRNA |
| NM_001901 | CTGF | 0.370331009 | 0.846215286 | 1.2438986 | down | mRNA |
| NM_001902 | CTH | 0.525718443 | 0.917817911 | 1.1619625 | down | mRNA |
| NM_001903 | CTNNA1 | 0.586562608 | 0.935591978 | 1.0680568 | down | mRNA |
| NM_001904 | CTNNB1 | 0.722200667 | 0.966191059 | 1.0939002 | down | mRNA |
| NM_001905 | CTPS1 | 0.062345874 | 0.439381733 | 1.1655508 | down | mRNA |
| NM_001906 | CTRB1 | 0.432810003 | 0.88061258 | 1.0694757 | down | mRNA |
| NM_001912 | CTSL1 | 0.134538767 | 0.591135973 | 1.1350349 | down | mRNA |
| NM_001917 | DAO | 0.339555951 | 0.828051324 | 1.1304694 | down | mRNA |
| NM_001918 | DBT | 0.064438391 | 0.443678541 | 1.1818911 | down | mRNA |
| NM_001919 | ECI1 | 0.29410088 | 0.791146775 | 1.0617725 | down | mRNA |
| NM_001921 | DCTD | 0.147655325 | 0.610312677 | 1.1693515 | down | mRNA |
| NM_001930 | DHPS | 0.654702231 | 0.957205183 | 1.0148466 | down | mRNA |
| NM_001931 | DLAT | 0.175257191 | 0.653821873 | 1.2668909 | down | mRNA |
| NM_001932 | MPP3 | 0.844386916 | 0.987338076 | 1.0193196 | down | mRNA |
| NM_001933 | DLST | 0.736957065 | 0.968834005 | 1.0303813 | down | mRNA |
| NM_001936 | DPP6 | 0.161613784 | 0.633561373 | 1.3271747 | down | mRNA |
| NM_001939 | DRP2 | 0.903770392 | 0.99508468 | 1.0129245 | down | mRNA |
| NM_001943 | DSG2 | 0.877850003 | 0.991306146 | 1.0744518 | down | mRNA |
| NM_001947 | DUSP7 | 0.075584642 | 0.471986158 | 1.3260086 | down | mRNA |
| NM_001948 | DUT | 0.321379217 | 0.815220381 | 1.1822384 | down | mRNA |
| NM_001949 | E2F3 | 0.043957235 | 0.385691973 | 1.1868995 | down | mRNA |
| NM_001950 | E2F4 | 0.403102647 | 0.864786348 | 1.0623884 | down | mRNA |
| NM_001951 | E2F5 | 0.000601228 | 0.07439369 | 1.3855139 | down | mRNA |
| NM_001956 | EDN2 | 0.491901138 | 0.905806259 | 1.1271584 | down | mRNA |
| NM_001957 | EDNRA | 0.390268562 | 0.858920947 | 1.2328978 | down | mRNA |
| NM_001961 | EEF2 | 0.628986211 | 0.950128593 | 1.1420045 | down | mRNA |
| NM_001967 | EIF4A2 | 0.605594783 | 0.94174007 | 1.1508339 | down | mRNA |
| NM_001972 | ELANE | 0.434248633 | 0.880931467 | 1.2466482 | down | mRNA |
| NM_001973 | ELK4 | 0.653238889 | 0.956860606 | 1.0453348 | down | mRNA |
| NM_001979 | EPHX2 | 0.001995311 | 0.107649983 | 1.3681891 | down | mRNA |
| NM_001980 | STX2 | 0.12699052 | 0.580406758 | 1.212398 | down | mRNA |
| NM_001981 | EPS15 | 0.153064293 | 0.619205018 | 1.06033 | down | mRNA |
| NM_001982 | ERBB3 | 0.911418728 | 0.995342392 | 1.0301291 | down | mRNA |
| NM_001985 | ETFB | 0.876346021 | 0.991213905 | 1.0214864 | down | mRNA |
| NM_001990 | EYA3 | 0.195615136 | 0.683572955 | 1.2460784 | down | mRNA |
| NM_001995 | ACSL1 | 0.483665194 | 0.903102279 | 1.077444 | down | mRNA |
| NM_001996 | FBLN1 | 0.968065113 | 0.997956694 | 1.0263247 | down | mRNA |
| NM_001998 | FBLN2 | 0.470387869 | 0.89818991 | 1.1090522 | down | mRNA |
| NM_002000 | FCAR | 0.277587259 | 0.777234326 | 1.2009298 | down | mRNA |
| NM_002005 | FES | 0.150603665 | 0.615623649 | 1.1325052 | down | mRNA |
| NM_002010 | FGF9 | 0.167454507 | 0.642346587 | 1.6904705 | down | mRNA |
| NM_002011 | FGFR4 | 0.273474414 | 0.773550609 | 1.2088397 | down | mRNA |
| NM_002013 | FKBP3 | 0.576350386 | 0.933656777 | 1.0508125 | down | mRNA |
| NM_002014 | FKBP4 | 0.277417466 | 0.777234326 | 1.1653163 | down | mRNA |
| NM_002026 | FN1 | 0.81753733 | 0.981727075 | 1.0588892 | down | mRNA |
| NM_002027 | FNTA | 0.354042505 | 0.836827738 | 1.1404928 | down | mRNA |
| NM_002033 | FUT4 | 0.809368359 | 0.98118927 | 1.0190038 | down | mRNA |
| NM_002035 | KDSR | 0.751136978 | 0.970725371 | 1.0625806 | down | mRNA |
| NM_002036 | DARC | 0.992825161 | 0.999798141 | 1.0012096 | down | mRNA |
| NM_002039 | GAB1 | 0.067678202 | 0.451808679 | 1.263981 | down | mRNA |
| NM_002044 | GALK2 | 0.339440568 | 0.827966656 | 1.1668658 | down | mRNA |
| NM_002046 | GAPDH | 0.392017373 | 0.859445464 | 2.3289156 | down | mRNA |
| NM_002047 | GARS | 0.54904746 | 0.925428884 | 1.1062854 | down | mRNA |
| NM_002048 | GAS1 | 0.851284149 | 0.987678166 | 1.1051942 | down | mRNA |
| NM_002056 | GFPT1 | 0.259423285 | 0.75991849 | 1.1768186 | down | mRNA |
| NM_002061 | GCLM | 0.035010537 | 0.352396712 | 1.1502624 | down | mRNA |
| NM_002067 | GNA11 | 0.352639349 | 0.835946719 | 1.1220076 | down | mRNA |
| NM_002072 | GNAQ | 0.009842131 | 0.206905383 | 1.1645033 | down | mRNA |
| NM_002074 | GNB1 | 0.935586008 | 0.996710991 | 1.0035805 | down | mRNA |
| NM_002076 | GNS | 0.635011044 | 0.951399945 | 1.0625424 | down | mRNA |
| NM_002077 | GOLGA1 | 0.49179172 | 0.905806259 | 1.0326839 | down | mRNA |
| NM_002079 | GOT1 | 0.407820008 | 0.867591329 | 1.1536104 | down | mRNA |
| NM_002080 | GOT2 | 0.146378271 | 0.608122555 | 1.1101529 | down | mRNA |
| NM_002081 | GPC1 | 0.026939851 | 0.319244809 | 1.5696957 | down | mRNA |
| NM_002082 | GRK6 | 0.151906441 | 0.617341945 | 1.1484233 | down | mRNA |
| NM_002091 | GRP | 0.911887545 | 0.995342392 | 1.0289816 | down | mRNA |
| NM_002095 | GTF2E2 | 0.3502016 | 0.834344847 | 1.0590058 | down | mRNA |
| NM_002096 | GTF2F1 | 0.871792182 | 0.990183713 | 1.0101823 | down | mRNA |
| NM_002105 | H2AFX | 0.509187485 | 0.911674633 | 1.2146455 | down | mRNA |
| NM_002106 | H2AFZ | 0.848126323 | 0.987678166 | 1.0420966 | down | mRNA |
| NM_002107 | H3F3A | 0.834699495 | 0.986575022 | 1.0211452 | down | mRNA |
| NM_002109 | HARS | 0.413694908 | 0.86961884 | 1.0742842 | down | mRNA |
| NM_002111 | HTT | 0.295286076 | 0.79157694 | 1.1347168 | down | mRNA |
| NM_002113 | CFHR1 | 0.743943617 | 0.970330526 | 1.053794 | down | mRNA |
| NM_002115 | HK3 | 0.324353624 | 0.816688282 | 1.1495871 | down | mRNA |
| NM_002119 | HLA-DOA | 0.890745589 | 0.9937521 | 1.0258723 | down | mRNA |
| NM_002124 | HLA-DRB1 | 0.440948743 | 0.883920737 | 1.1964607 | down | mRNA |
| NM_002126 | HLF | 0.952583037 | 0.996710991 | 1.0067575 | down | mRNA |
| NM_002128 | HMGB1 | 0.778396906 | 0.976938981 | 1.0435693 | down | mRNA |
| NM_002133 | HMOX1 | 0.064888959 | 0.444815344 | 1.2524902 | down | mRNA |
| NM_002134 | HMOX2 | 0.00480508 | 0.158085585 | 1.2222461 | down | mRNA |
| NM_002136 | HNRNPA1 | 0.369005739 | 0.84565745 | 1.2769811 | down | mRNA |
| NM_002137 | HNRNPA2B1 | 0.508903082 | 0.911674633 | 1.0734189 | down | mRNA |
| NM_002138 | HNRNPD | 0.066247351 | 0.449693785 | 1.194769 | down | mRNA |
| NM_002143 | HPCA | 0.195806064 | 0.683572955 | 1.4342197 | down | mRNA |
| NM_002144 | HOXB1 | 0.484963691 | 0.903250293 | 1.196938 | down | mRNA |
| NM_002146 | HOXB3 | 0.865653006 | 0.989401142 | 1.0085372 | down | mRNA |
| NM_002149 | HPCAL1 | 0.783634528 | 0.977275912 | 1.0224168 | down | mRNA |
| NM_002153 | HSD17B2 | 0.354606056 | 0.83766056 | 1.05963 | down | mRNA |
| NM_002154 | HSPA4 | 0.13662481 | 0.595467448 | 1.1395319 | down | mRNA |
| NM_002155 | HSPA6 | 0.621651639 | 0.947997886 | 1.1912599 | down | mRNA |
| NM_002157 | HSPE1 | 0.464313511 | 0.894989844 | 1.0655163 | down | mRNA |
| NM_002158 | FOXN2 | 0.134399907 | 0.590964295 | 1.2028369 | down | mRNA |
| NM_002162 | ICAM3 | 0.298650249 | 0.795233272 | 1.32524 | down | mRNA |
| NM_002165 | ID1 | 0.006188041 | 0.178714838 | 4.7519588 | down | mRNA |
| NM_002168 | IDH2 | 0.5254129 | 0.917686109 | 1.0379543 | down | mRNA |
| NM_002181 | IHH | 0.923578872 | 0.995456159 | 1.0223018 | down | mRNA |
| NM_002183 | IL3RA | 0.767447661 | 0.975033576 | 1.042162 | down | mRNA |
| NM_002197 | ACO1 | 0.908990789 | 0.995333785 | 1.0228671 | down | mRNA |
| NM_002204 | ITGA3 | 0.710357885 | 0.965462762 | 1.1414817 | down | mRNA |
| NM_002205 | ITGA5 | 0.973028396 | 0.998501532 | 1.0017262 | down | mRNA |
| NM_002206 | ITGA7 | 0.554038944 | 0.926549129 | 1.0946373 | down | mRNA |
| NM_002213 | ITGB5 | 0.632534523 | 0.951123179 | 1.0659787 | down | mRNA |
| NM_002217 | ITIH3 | 0.961123259 | 0.99793927 | 1.0055083 | down | mRNA |
| NM_002221 | ITPKB | 0.763189041 | 0.973534073 | 1.0781792 | down | mRNA |
| NM_002237 | KCNG1 | 0.135563984 | 0.593260206 | 1.25341 | down | mRNA |
| NM_002238 | KCNH1 | 0.186482729 | 0.669888134 | 1.1193561 | down | mRNA |
| NM_002246 | KCNK3 | 0.584652766 | 0.93461341 | 1.0622688 | down | mRNA |
| NM_002248 | KCNN1 | 0.803103453 | 0.98118927 | 1.056211 | down | mRNA |
| NM_002252 | KCNS3 | 0.031252253 | 0.340775376 | 1.2387431 | down | mRNA |
| NM_002253 | KDR | 0.72095681 | 0.966191059 | 1.1085182 | down | mRNA |
| NM_002260 | KLRC2 | 0.838920347 | 0.987032229 | 1.0492994 | down | mRNA |
| NM_002263 | KIFC1 | 0.65178619 | 0.956204521 | 1.3956521 | down | mRNA |
| NM_002264 | KPNA1 | 0.804922321 | 0.98118927 | 1.0173697 | down | mRNA |
| NM_002265 | KPNB1 | 0.005118899 | 0.163643802 | 1.2204408 | down | mRNA |
| NM_002266 | KPNA2 | 0.131294987 | 0.586572112 | 1.2810281 | down | mRNA |
| NM_002269 | KPNA5 | 0.040280796 | 0.371915487 | 1.1591962 | down | mRNA |
| NM_002270 | TNPO1 | 0.702124887 | 0.963247543 | 1.0844827 | down | mRNA |
| NM_002271 | IPO5 | 0.608927895 | 0.943053241 | 1.0547258 | down | mRNA |
| NM_002273 | KRT8 | 0.644033247 | 0.954605197 | 1.0448844 | down | mRNA |
| NM_002275 | KRT15 | 0.201081207 | 0.689092046 | 1.4111517 | down | mRNA |
| NM_002276 | KRT19 | 0.339131075 | 0.827695551 | 1.3151345 | down | mRNA |
| NM_002277 | KRT31 | 0.630122952 | 0.950410442 | 1.4811256 | down | mRNA |
| NM_002281 | KRT81 | 0.676397281 | 0.959531882 | 1.110123 | down | mRNA |
| NM_002286 | LAG3 | 0.269611825 | 0.76993462 | 1.2015479 | down | mRNA |
| NM_002288 | LAIR2 | 0.023759027 | 0.303244528 | 1.1238908 | down | mRNA |
| NM_002291 | LAMB1 | 0.407969199 | 0.867591329 | 1.2531863 | down | mRNA |
| NM_002292 | LAMB2 | 0.6748601 | 0.959531882 | 1.0426382 | down | mRNA |
| NM_002303 | LEPR | 0.973873053 | 0.998501532 | 1.0109909 | down | mRNA |
| NM_002308 | LGALS9 | 0.512965194 | 0.913297047 | 1.2888455 | down | mRNA |
| NM_002314 | LIMK1 | 0.141202989 | 0.601673229 | 1.7072523 | down | mRNA |
| NM_002318 | LOXL2 | 0.84053347 | 0.987068842 | 1.0409948 | down | mRNA |
| NM_002319 | LRCH4 | 0.260209906 | 0.76057904 | 1.3440689 | down | mRNA |
| NM_002332 | LRP1 | 0.829547839 | 0.985549562 | 1.0070924 | down | mRNA |
| NM_002333 | LRP3 | 0.341503937 | 0.829961515 | 1.1705905 | down | mRNA |
| NM_002335 | LRP5 | 0.403341799 | 0.864786348 | 1.2614871 | down | mRNA |
| NM_002337 | LRPAP1 | 0.277722452 | 0.777240591 | 1.4800704 | down | mRNA |
| NM_002338 | LSAMP | 0.796674623 | 0.980665598 | 1.06694 | down | mRNA |
| NM_002349 | LY75 | 0.745408208 | 0.970394407 | 1.0263625 | down | mRNA |
| NM_002355 | M6PR | 0.207196481 | 0.698556218 | 1.1717824 | down | mRNA |
| NM_002358 | MAD2L1 | 0.760850386 | 0.972945894 | 1.1661942 | down | mRNA |
| NM_002360 | MAFK | 0.250081508 | 0.748734681 | 1.3130006 | down | mRNA |
| NM_002361 | MAG | 0.956870619 | 0.996883425 | 1.0100102 | down | mRNA |
| NM_002372 | MAN2A1 | 0.848194222 | 0.987678166 | 1.0189437 | down | mRNA |
| NM_002373 | MAP1A | 0.221520468 | 0.716734257 | 1.1265122 | down | mRNA |
| NM_002375 | MAP4 | 0.21819299 | 0.712269655 | 1.1129831 | down | mRNA |
| NM_002378 | MATK | 0.600347589 | 0.940648177 | 1.0618942 | down | mRNA |
| NM_002380 | MATN2 | 0.395135827 | 0.861590143 | 1.0807956 | down | mRNA |
| NM_002382 | MAX | 0.008273661 | 0.197905968 | 1.1211513 | down | mRNA |
| NM_002383 | MAZ | 0.200402174 | 0.68900633 | 1.1659161 | down | mRNA |
| NM_002384 | MBD1 | 0.60020262 | 0.940648177 | 1.0733601 | down | mRNA |
| NM_002388 | MCM3 | 0.873902114 | 0.990793509 | 1.0920721 | down | mRNA |
| NM_002390 | ADAM11 | 0.655558912 | 0.957222998 | 1.042065 | down | mRNA |
| NM_002392 | MDM2 | 0.380727892 | 0.853514109 | 1.195597 | down | mRNA |
| NM_002394 | SLC3A2 | 0.030845912 | 0.340185047 | 1.2620654 | down | mRNA |
| NM_002396 | ME2 | 0.196792184 | 0.685161614 | 1.0818375 | down | mRNA |
| NM_002397 | MEF2C | 0.285593019 | 0.78236018 | 1.2448673 | down | mRNA |
| NM_002399 | MEIS2 | 0.231471044 | 0.72920193 | 1.4130459 | down | mRNA |
| NM_002408 | MGAT2 | 0.132388376 | 0.587843174 | 1.1005234 | down | mRNA |
| NM_002410 | MGAT5 | 0.862776163 | 0.989401142 | 1.0190334 | down | mRNA |
| NM_002412 | MGMT | 0.860828034 | 0.989013989 | 1.0211388 | down | mRNA |
| NM_002414 | CD99 | 0.974903057 | 0.998501532 | 1.0073781 | down | mRNA |
| NM_002415 | MIF | 0.729340427 | 0.966191059 | 1.0399098 | down | mRNA |
| NM_002417 | MKI67 | 0.688087751 | 0.96053554 | 1.2155156 | down | mRNA |
| NM_002420 | TRPM1 | 0.789190679 | 0.977807732 | 1.0462075 | down | mRNA |
| NM_002429 | MMP19 | 0.530256108 | 0.91899174 | 1.1616927 | down | mRNA |
| NM_002431 | MNAT1 | 0.483277529 | 0.902875928 | 1.0733187 | down | mRNA |
| NM_002435 | MPI | 0.533463939 | 0.920530493 | 1.026079 | down | mRNA |
| NM_002436 | MPP1 | 0.398980566 | 0.863643939 | 1.4181034 | down | mRNA |
| NM_002439 | MSH3 | 0.337865853 | 0.827394958 | 1.1057781 | down | mRNA |
| NM_002446 | MAP3K10 | 0.324468508 | 0.816842093 | 1.1164415 | down | mRNA |
| NM_002448 | MSX1 | 0.200002708 | 0.68900633 | 1.7482674 | down | mRNA |
| NM_002454 | MTRR | 0.37031472 | 0.846215286 | 1.1046054 | down | mRNA |
| NM_002466 | MYBL2 | 0.249139962 | 0.747319103 | 1.3332864 | down | mRNA |
| NM_002467 | MYC | 0.300311801 | 0.795797471 | 1.3542915 | down | mRNA |
| NM_002469 | MYF6 | 0.062617802 | 0.439458294 | 1.1907975 | down | mRNA |
| NM_002473 | MYH9 | 0.793107931 | 0.979003983 | 1.0411602 | down | mRNA |
| NM_002477 | MYL5 | 0.62399915 | 0.948341211 | 1.1060967 | down | mRNA |
| NM_002480 | PPP1R12A | 0.041195117 | 0.376152917 | 1.551051 | down | mRNA |
| NM_002481 | PPP1R12B | 0.229932917 | 0.727173131 | 1.2225261 | down | mRNA |
| NM_002485 | NBN | 0.165227893 | 0.638608196 | 1.0971434 | down | mRNA |
| NM_002486 | NCBP1 | 0.045289263 | 0.389848135 | 1.2598955 | down | mRNA |
| NM_002487 | NDN | 0.48662101 | 0.903524001 | 1.0663775 | down | mRNA |
| NM_002488 | NDUFA2 | 0.831086243 | 0.985710869 | 1.00453 | down | mRNA |
| NM_002489 | NDUFA4 | 0.524791039 | 0.917632491 | 1.049771 | down | mRNA |
| NM_002493 | NDUFB6 | 0.93977966 | 0.996710991 | 1.0045102 | down | mRNA |
| NM_002495 | NDUFS4 | 0.429586414 | 0.878226842 | 1.0786716 | down | mRNA |
| NM_002496 | NDUFS8 | 0.950047371 | 0.996710991 | 1.0045994 | down | mRNA |
| NM_002497 | NEK2 | 0.220923311 | 0.71639757 | 1.7029241 | down | mRNA |
| NM_002504 | NFX1 | 0.509528493 | 0.911674633 | 1.0326357 | down | mRNA |
| NM_002505 | NFYA | 0.05369645 | 0.416065637 | 1.2528651 | down | mRNA |
| NM_002510 | GPNMB | 0.678896423 | 0.95996398 | 1.0643504 | down | mRNA |
| NM_002512 | NME2 | 0.882113288 | 0.992766819 | 1.0040132 | down | mRNA |
| NM_002513 | NME3 | 0.90626798 | 0.99508468 | 1.0080183 | down | mRNA |
| NM_002514 | NOV | 0.855482569 | 0.988178307 | 1.0493977 | down | mRNA |
| NM_002517 | NPAS1 | 0.35839729 | 0.840546205 | 1.4018671 | down | mRNA |
| NM_002520 | NPM1 | 0.45972119 | 0.892978158 | 1.2977637 | down | mRNA |
| NM_002524 | NRAS | 0.855959235 | 0.988315074 | 1.0211117 | down | mRNA |
| NM_002525 | NRD1 | 0.308860414 | 0.803961878 | 1.1902403 | down | mRNA |
| NM_002526 | NT5E | 0.468106474 | 0.897203143 | 1.0651599 | down | mRNA |
| NM_002530 | NTRK3 | 0.768526247 | 0.975142015 | 1.0335469 | down | mRNA |
| NM_002533 | NVL | 0.37755726 | 0.851053291 | 1.1788174 | down | mRNA |
| NM_002535 | OAS2 | 0.592802386 | 0.937820942 | 1.5517667 | down | mRNA |
| NM_002539 | ODC1 | 0.791060274 | 0.978610795 | 1.035147 | down | mRNA |
| NM_002542 | OGG1 | 0.594882561 | 0.938655001 | 1.0371528 | down | mRNA |
| NM_002545 | OPCML | 0.633333187 | 0.951174475 | 1.027917 | down | mRNA |
| NM_002547 | OPHN1 | 0.62228665 | 0.948166379 | 1.1561425 | down | mRNA |
| NM_002555 | SLC22A18 | 0.899852603 | 0.994752372 | 1.0079707 | down | mRNA |
| NM_002560 | P2RX4 | 0.739246657 | 0.969564098 | 1.0467107 | down | mRNA |
| NM_002561 | P2RX5 | 0.839773453 | 0.987068842 | 1.0201129 | down | mRNA |
| NM_002566 | P2RY11 | 0.09018782 | 0.507386748 | 1.1311285 | down | mRNA |
| NM_002567 | PEBP1 | 0.958823635 | 0.99728722 | 1.0036656 | down | mRNA |
| NM_002568 | PABPC1 | 0.42663797 | 0.876202345 | 1.0992351 | down | mRNA |
| NM_002570 | PCSK6 | 0.937564376 | 0.996710991 | 1.0060862 | down | mRNA |
| NM_002571 | PAEP | 0.779348456 | 0.976938981 | 1.0177851 | down | mRNA |
| NM_002572 | PAFAH1B2 | 0.986731229 | 0.99909491 | 1.0028877 | down | mRNA |
| NM_002576 | PAK1 | 0.19169531 | 0.67759806 | 1.1032113 | down | mRNA |
| NM_002579 | PALM | 0.252395924 | 0.752621656 | 1.5604981 | down | mRNA |
| NM_002580 | REG3A | 0.748420881 | 0.970725371 | 1.0521988 | down | mRNA |
| NM_002582 | PARN | 0.010007292 | 0.207620683 | 1.2633667 | down | mRNA |
| NM_002583 | PAWR | 0.19069496 | 0.676402188 | 1.1167116 | down | mRNA |
| NM_002588 | PCDHGC3 | 0.67203314 | 0.959531882 | 1.0629858 | down | mRNA |
| NM_002591 | PCK1 | 0.527657078 | 0.918678174 | 1.0803964 | down | mRNA |
| NM_002593 | PCOLCE | 0.905406247 | 0.99508468 | 1.0124544 | down | mRNA |
| NM_002595 | CDK17 | 0.705864983 | 0.965020205 | 1.1040177 | down | mRNA |
| NM_002598 | PDCD2 | 0.146796943 | 0.608803337 | 1.0848037 | down | mRNA |
| NM_002599 | PDE2A | 0.550641113 | 0.925928432 | 1.0593821 | down | mRNA |
| NM_002609 | PDGFRB | 0.942120301 | 0.996710991 | 1.0284191 | down | mRNA |
| NM_002610 | PDK1 | 0.876126088 | 0.991213905 | 1.0288481 | down | mRNA |
| NM_002613 | PDPK1 | 0.169570629 | 0.646121878 | 1.179495 | down | mRNA |
| NM_002616 | PER1 | 0.848340996 | 0.987678166 | 1.0253392 | down | mRNA |
| NM_002618 | PEX13 | 0.016347307 | 0.255922225 | 1.2472434 | down | mRNA |
| NM_002621 | CFP | 0.090707433 | 0.508762352 | 1.1304966 | down | mRNA |
| NM_002628 | PFN2 | 0.600690098 | 0.940757262 | 1.136437 | down | mRNA |
| NM_002629 | PGAM1 | 0.777877636 | 0.976938981 | 1.0639957 | down | mRNA |
| NM_002631 | PGD | 0.967578161 | 0.99793927 | 1.008065 | down | mRNA |
| NM_002632 | PGF | 0.036376126 | 0.357986155 | 1.6974711 | down | mRNA |
| NM_002634 | PHB | 0.460577534 | 0.893982093 | 1.0874924 | down | mRNA |
| NM_002645 | PIK3C2A | 0.77505361 | 0.97589756 | 1.0357523 | down | mRNA |
| NM_002646 | PIK3C2B | 0.107416017 | 0.543414396 | 1.5899478 | down | mRNA |
| NM_002647 | PIK3C3 | 0.142007327 | 0.60204132 | 1.3041852 | down | mRNA |
| NM_002650 | PI4KA | 0.841107185 | 0.987068842 | 1.0206001 | down | mRNA |
| NM_002654 | PKM | 0.646742029 | 0.95550188 | 1.0588851 | down | mRNA |
| NM_002660 | PLCG1 | 0.041444011 | 0.377526715 | 1.272586 | down | mRNA |
| NM_002662 | PLD1 | 0.107674062 | 0.543907835 | 1.6843802 | down | mRNA |
| NM_002663 | PLD2 | 0.049120662 | 0.402023817 | 1.1547915 | down | mRNA |
| NM_002668 | PLP2 | 0.366793456 | 0.845181673 | 1.08224 | down | mRNA |
| NM_002669 | PLRG1 | 0.031918696 | 0.342121122 | 1.1641745 | down | mRNA |
| NM_002676 | PMM1 | 0.334339449 | 0.824174306 | 1.1774539 | down | mRNA |
| NM_002685 | EXOSC10 | 0.424194967 | 0.874808 | 1.0354196 | down | mRNA |
| NM_002686 | PNMT | 0.588129344 | 0.93579682 | 1.1985763 | down | mRNA |
| NM_002687 | PNN | 0.584095312 | 0.934515729 | 1.0364911 | down | mRNA |
| NM_002688 | SEPT5 | 0.223428742 | 0.71765453 | 1.5943747 | down | mRNA |
| NM_002689 | POLA2 | 0.833568633 | 0.986434891 | 1.1388638 | down | mRNA |
| NM_002692 | POLE2 | 0.770222874 | 0.975286259 | 1.193751 | down | mRNA |
| NM_002698 | POU2F2 | 0.637265297 | 0.952624144 | 1.0552626 | down | mRNA |
| NM_002706 | PPM1B | 0.502558345 | 0.910335149 | 1.1589149 | down | mRNA |
| NM_002708 | PPP1CA | 0.056066887 | 0.422065984 | 1.1546127 | down | mRNA |
| NM_002709 | PPP1CB | 0.037282761 | 0.36076463 | 1.155237 | down | mRNA |
| NM_002710 | PPP1CC | 0.036851271 | 0.359628653 | 1.1639591 | down | mRNA |
| NM_002712 | PPP1R7 | 0.922906023 | 0.995349793 | 1.0042845 | down | mRNA |
| NM_002713 | PPP1R8 | 0.112369284 | 0.554686607 | 1.1670093 | down | mRNA |
| NM_002714 | PPP1R10 | 0.688286332 | 0.96055024 | 1.0781522 | down | mRNA |
| NM_002715 | PPP2CA | 0.374307099 | 0.849321997 | 1.0968311 | down | mRNA |
| NM_002717 | PPP2R2A | 0.054755514 | 0.419413793 | 1.1501578 | down | mRNA |
| NM_002719 | PPP2R5C | 0.683925969 | 0.960111041 | 1.0566916 | down | mRNA |
| NM_002720 | PPP4C | 0.851205486 | 0.987678166 | 1.01859 | down | mRNA |
| NM_002721 | PPP6C | 0.173738381 | 0.651863583 | 1.074838 | down | mRNA |
| NM_002726 | PREP | 0.035821597 | 0.355280342 | 1.399805 | down | mRNA |
| NM_002728 | PRG2 | 0.314499982 | 0.809726938 | 1.7049036 | down | mRNA |
| NM_002733 | PRKAG1 | 0.599363815 | 0.940648177 | 1.0202839 | down | mRNA |
| NM_002736 | PRKAR2B | 0.604513466 | 0.94174007 | 1.133388 | down | mRNA |
| NM_002737 | PRKCA | 0.597851319 | 0.940150767 | 1.0581794 | down | mRNA |
| NM_002739 | PRKCG | 0.835803698 | 0.986575022 | 1.058646 | down | mRNA |
| NM_002740 | PRKCI | 0.909621571 | 0.995342392 | 1.0149138 | down | mRNA |
| NM_002742 | PRKD1 | 0.357594515 | 0.839676686 | 1.1023779 | down | mRNA |
| NM_002743 | PRKCSH | 0.397241618 | 0.862344286 | 1.2215848 | down | mRNA |
| NM_002746 | MAPK3 | 0.95161251 | 0.996710991 | 1.0050666 | down | mRNA |
| NM_002750 | MAPK8 | 0.797238311 | 0.980667273 | 1.0323988 | down | mRNA |
| NM_002752 | MAPK9 | 0.086682516 | 0.499950487 | 1.0954523 | down | mRNA |
| NM_002754 | MAPK13 | 0.528657328 | 0.918883792 | 1.267382 | down | mRNA |
| NM_002755 | MAP2K1 | 0.275409137 | 0.775184524 | 1.3609064 | down | mRNA |
| NM_002756 | MAP2K3 | 0.882114084 | 0.992766819 | 1.0273162 | down | mRNA |
| NM_002757 | MAP2K5 | 0.455587959 | 0.891148383 | 1.1303429 | down | mRNA |
| NM_002764 | PRPS1 | 0.037807275 | 0.363212377 | 1.5704409 | down | mRNA |
| NM_002765 | PRPS2 | 0.222618904 | 0.717433439 | 1.2826021 | down | mRNA |
| NM_002767 | PRPSAP2 | 0.484112723 | 0.903250293 | 1.0761496 | down | mRNA |
| NM_002768 | CHMP1A | 0.022831081 | 0.297755956 | 1.0260897 | down | mRNA |
| NM_002770 | PRSS2 | 0.94870519 | 0.996710991 | 1.0111794 | down | mRNA |
| NM_002780 | PSG4 | 0.824082847 | 0.983568283 | 1.0880937 | down | mRNA |
| NM_002781 | PSG5 | 0.910522473 | 0.995342392 | 1.0536692 | down | mRNA |
| NM_002787 | PSMA2 | 0.56262998 | 0.929041598 | 1.0308097 | down | mRNA |
| NM_002792 | PSMA7 | 0.824425374 | 0.983640438 | 1.0233426 | down | mRNA |
| NM_002793 | PSMB1 | 0.429933324 | 0.878446786 | 1.0439157 | down | mRNA |
| NM_002794 | PSMB2 | 0.771403579 | 0.975347132 | 1.0116861 | down | mRNA |
| NM_002798 | PSMB6 | 0.984434479 | 0.998971807 | 1.0013197 | down | mRNA |
| NM_002801 | PSMB10 | 0.912973165 | 0.995342392 | 1.0096344 | down | mRNA |
| NM_002803 | PSMC2 | 0.263131801 | 0.763194832 | 1.0919496 | down | mRNA |
| NM_002804 | PSMC3 | 0.689384736 | 0.96108227 | 1.0460414 | down | mRNA |
| NM_002805 | PSMC5 | 0.187510905 | 0.671597204 | 2.0432211 | down | mRNA |
| NM_002806 | PSMC6 | 0.29873578 | 0.795233272 | 1.0967177 | down | mRNA |
| NM_002807 | PSMD1 | 0.10286078 | 0.532205007 | 1.1209876 | down | mRNA |
| NM_002808 | PSMD2 | 0.621650702 | 0.947997886 | 1.08244 | down | mRNA |
| NM_002810 | PSMD4 | 0.10330027 | 0.533095146 | 1.1757947 | down | mRNA |
| NM_002813 | PSMD9 | 0.286212639 | 0.782895119 | 1.0981253 | down | mRNA |
| NM_002814 | PSMD10 | 0.462679061 | 0.894989844 | 1.0964578 | down | mRNA |
| NM_002815 | PSMD11 | 0.251647278 | 0.751762041 | 1.3493625 | down | mRNA |
| NM_002816 | PSMD12 | 0.191790803 | 0.67759806 | 1.0783626 | down | mRNA |
| NM_002817 | PSMD13 | 0.160730032 | 0.632101162 | 1.0969683 | down | mRNA |
| NM_002821 | PTK7 | 0.975993135 | 0.998501532 | 1.0033069 | down | mRNA |
| NM_002824 | PTMS | 0.758387029 | 0.972072638 | 1.050839 | down | mRNA |
| NM_002826 | QSOX1 | 0.190843584 | 0.676402188 | 1.1487002 | down | mRNA |
| NM_002830 | PTPN4 | 0.836968897 | 0.986775289 | 1.0305721 | down | mRNA |
| NM_002833 | PTPN9 | 0.103774501 | 0.533622448 | 1.1414663 | down | mRNA |
| NM_002837 | PTPRB | 0.65907783 | 0.958354006 | 1.0981713 | down | mRNA |
| NM_002840 | PTPRF | 0.729840007 | 0.966191059 | 1.108846 | down | mRNA |
| NM_002842 | PTPRH | 0.964066009 | 0.99793927 | 1.0080725 | down | mRNA |
| NM_002843 | PTPRJ | 0.182184762 | 0.66538182 | 1.0991637 | down | mRNA |
| NM_002845 | PTPRM | 0.992870521 | 0.999798141 | 1.0004445 | down | mRNA |
| NM_002853 | RAD1 | 0.331322049 | 0.82187807 | 1.0980943 | down | mRNA |
| NM_002856 | PVRL2 | 0.997316963 | 0.999889342 | 1.0002077 | down | mRNA |
| NM_002857 | PEX19 | 0.670433927 | 0.959531882 | 1.032194 | down | mRNA |
| NM_002858 | ABCD3 | 0.137020365 | 0.595932894 | 1.1584995 | down | mRNA |
| NM_002859 | PXN | 0.284730369 | 0.781893282 | 1.2447777 | down | mRNA |
| NM_002860 | ALDH18A1 | 0.001825115 | 0.105670067 | 1.4575319 | down | mRNA |
| NM_002861 | PCYT2 | 0.487528646 | 0.903757389 | 1.0570208 | down | mRNA |
| NM_002862 | PYGB | 0.872756561 | 0.990534615 | 1.0464577 | down | mRNA |
| NM_002863 | PYGL | 0.2463294 | 0.744874529 | 1.3113572 | down | mRNA |
| NM_002865 | RAB2A | 0.424862865 | 0.874854499 | 1.0940291 | down | mRNA |
| NM_002866 | RAB3A | 0.07426663 | 0.469506874 | 1.4135616 | down | mRNA |
| NM_002869 | RAB6A | 0.071724277 | 0.462237305 | 1.1136352 | down | mRNA |
| NM_002870 | RAB13 | 0.961232369 | 0.99793927 | 1.0079728 | down | mRNA |
| NM_002874 | RAD23B | 0.905710873 | 0.99508468 | 1.0075564 | down | mRNA |
| NM_002875 | RAD51 | 0.786058389 | 0.977479172 | 1.1500989 | down | mRNA |
| NM_002876 | RAD51C | 0.246395713 | 0.744874529 | 1.1070217 | down | mRNA |
| NM_002878 | RAD51D | 0.049451437 | 0.402500646 | 1.301199 | down | mRNA |
| NM_002880 | RAF1 | 0.086469419 | 0.49936346 | 1.1288419 | down | mRNA |
| NM_002881 | RALB | 0.014739952 | 0.24380986 | 1.1753657 | down | mRNA |
| NM_002884 | RAP1A | 0.505320324 | 0.910772598 | 1.0911431 | down | mRNA |
| NM_002885 | RAP1GAP | 0.059866697 | 0.434252472 | 1.6702606 | down | mRNA |
| NM_002886 | RAP2B | 0.007754213 | 0.19345927 | 1.4328399 | down | mRNA |
| NM_002887 | RARS | 0.185117105 | 0.668407207 | 1.098741 | down | mRNA |
| NM_002889 | RARRES2 | 0.422971124 | 0.873989281 | 1.2413125 | down | mRNA |
| NM_002890 | RASA1 | 0.007021307 | 0.18703243 | 1.6670007 | down | mRNA |
| NM_002892 | ARID4A | 0.137871094 | 0.596789644 | 1.4088786 | down | mRNA |
| NM_002893 | RBBP7 | 0.332399954 | 0.822707947 | 1.0853879 | down | mRNA |
| NM_002895 | RBL1 | 0.781287427 | 0.976938981 | 1.1849641 | down | mRNA |
| NM_002896 | RBM4 | 0.581830494 | 0.934005566 | 1.113575 | down | mRNA |
| NM_002898 | RBMS2 | 0.151205163 | 0.616500055 | 1.1620998 | down | mRNA |
| NM_002901 | RCN1 | 0.339286079 | 0.827737528 | 1.2195804 | down | mRNA |
| NM_002902 | RCN2 | 0.72627454 | 0.966191059 | 1.0287468 | down | mRNA |
| NM_002906 | RDX | 0.677934759 | 0.959531882 | 1.0316461 | down | mRNA |
| NM_002911 | UPF1 | 0.098752983 | 0.522573019 | 1.2610742 | down | mRNA |
| NM_002913 | RFC1 | 0.032004997 | 0.342443112 | 1.3693541 | down | mRNA |
| NM_002914 | RFC2 | 0.313846311 | 0.808965923 | 1.1958333 | down | mRNA |
| NM_002915 | RFC3 | 0.951565134 | 0.996710991 | 1.0319309 | down | mRNA |
| NM_002924 | RGS7 | 0.29899252 | 0.795233272 | 1.0773742 | down | mRNA |
| NM_002931 | RING1 | 0.376781071 | 0.85040519 | 1.074689 | down | mRNA |
| NM_002936 | RNASEH1 | 0.402044738 | 0.864786348 | 1.0725416 | down | mRNA |
| NM_002938 | RNF4 | 0.367072416 | 0.845181673 | 1.0965839 | down | mRNA |
| NM_002945 | RPA1 | 0.207202964 | 0.698556218 | 1.3389921 | down | mRNA |
| NM_002946 | RPA2 | 0.681354172 | 0.960111041 | 1.0727878 | down | mRNA |
| NM_002948 | RPL15 | 0.984579899 | 0.998971807 | 1.0028122 | down | mRNA |
| NM_002949 | MRPL12 | 0.689039381 | 0.961072147 | 1.0888517 | down | mRNA |
| NM_002950 | RPN1 | 0.916566399 | 0.995349793 | 1.0097338 | down | mRNA |
| NM_002951 | RPN2 | 0.523421801 | 0.917239107 | 1.0825836 | down | mRNA |
| NM_002957 | RXRA | 0.052415518 | 0.412637354 | 1.2180665 | down | mRNA |
| NM_002958 | RYK | 0.036495166 | 0.358673526 | 1.2871354 | down | mRNA |
| NM_002960 | S100A3 | 0.984209403 | 0.998971807 | 1.0061639 | down | mRNA |
| NM_002967 | SAFB | 0.213778376 | 0.706323665 | 1.1101686 | down | mRNA |
| NM_002969 | MAPK12 | 0.018368548 | 0.270998737 | 1.1650907 | down | mRNA |
| NM_002972 | SBF1 | 0.372994519 | 0.84810582 | 1.0830586 | down | mRNA |
| NM_002975 | CLEC11A | 0.958163145 | 0.996933183 | 1.0065236 | down | mRNA |
| NM_002983 | CCL3 | 0.605697579 | 0.94174007 | 1.0730423 | down | mRNA |
| NM_002988 | CCL18 | 0.762734907 | 0.973443358 | 1.0765301 | down | mRNA |
| NM_002997 | SDC1 | 0.6547438 | 0.957205183 | 1.07293 | down | mRNA |
| NM_002998 | SDC2 | 0.758002291 | 0.972072638 | 1.1709761 | down | mRNA |
| NM_003004 | SECTM1 | 0.416720164 | 0.870736736 | 1.2552202 | down | mRNA |
| NM_003005 | SELP | 0.125074147 | 0.578441772 | 1.2559173 | down | mRNA |
| NM_003010 | MAP2K4 | 0.323870634 | 0.816280863 | 1.1921288 | down | mRNA |
| NM_003011 | SET | 0.265824996 | 0.766569994 | 1.0756418 | down | mRNA |
| NM_003012 | SFRP1 | 0.915200272 | 0.995349793 | 1.116972 | down | mRNA |
| NM_003017 | SRSF3 | 0.056469014 | 0.423551663 | 1.2680626 | down | mRNA |
| NM_003018 | SFTPC | 0.058576212 | 0.430419782 | 1.80175 | down | mRNA |
| NM_003020 | SCG5 | 0.948298733 | 0.996710991 | 1.0129382 | down | mRNA |
| NM_003025 | SH3GL1 | 0.448131245 | 0.887964572 | 1.0962065 | down | mRNA |
| NM_003029 | SHC1 | 0.630473129 | 0.950410442 | 1.0634799 | down | mRNA |
| NM_003031 | SIAH1 | 0.611201411 | 0.943794873 | 1.0821742 | down | mRNA |
| NM_003036 | SKI | 0.526546365 | 0.917975454 | 1.1320786 | down | mRNA |
| NM_003038 | SLC1A4 | 0.449558997 | 0.888279993 | 1.1430253 | down | mRNA |
| NM_003041 | SLC5A2 | 0.146344586 | 0.608122555 | 1.1736174 | down | mRNA |
| NM_003042 | SLC6A1 | 0.470813791 | 0.898445976 | 1.1524993 | down | mRNA |
| NM_003061 | SLIT1 | 0.759643656 | 0.972795541 | 1.1004643 | down | mRNA |
| NM_003064 | SLPI | 0.824022867 | 0.983568283 | 1.1099883 | down | mRNA |
| NM_003069 | SMARCA1 | 0.683953456 | 0.960111041 | 1.0897028 | down | mRNA |
| NM_003070 | SMARCA2 | 0.260010873 | 0.760388386 | 1.1192857 | down | mRNA |
| NM_003071 | HLTF | 0.182439865 | 0.66575662 | 1.2374768 | down | mRNA |
| NM_003074 | SMARCC1 | 0.581173067 | 0.934005566 | 1.0574482 | down | mRNA |
| NM_003076 | SMARCD1 | 0.154365596 | 0.621759137 | 1.1367569 | down | mRNA |
| NM_003078 | SMARCD3 | 0.649795819 | 0.95550188 | 1.0626956 | down | mRNA |
| NM_003079 | SMARCE1 | 0.110939306 | 0.551283668 | 1.0807761 | down | mRNA |
| NM_003083 | SNAPC2 | 0.673823276 | 0.959531882 | 1.015146 | down | mRNA |
| NM_003086 | SNAPC4 | 0.343179322 | 0.831521302 | 1.1642964 | down | mRNA |
| NM_003088 | FSCN1 | 0.625345702 | 0.948832915 | 1.1302849 | down | mRNA |
| NM_003089 | SNRNP70 | 0.030849852 | 0.340185047 | 1.2023773 | down | mRNA |
| NM_003090 | SNRPA1 | 0.633795509 | 0.951174475 | 1.1439571 | down | mRNA |
| NM_003091 | SNRPB | 0.858880756 | 0.988512246 | 1.0196083 | down | mRNA |
| NM_003097 | SNRPN | 0.952837047 | 0.996710991 | 1.0082469 | down | mRNA |
| NM_003098 | SNTA1 | 0.03969689 | 0.370777582 | 1.1640719 | down | mRNA |
| NM_003099 | SNX1 | 0.049951032 | 0.40410612 | 1.2171559 | down | mRNA |
| NM_003101 | SOAT1 | 0.08403224 | 0.493254641 | 1.1603998 | down | mRNA |
| NM_003106 | SOX2 | 0.233413661 | 0.731130266 | 1.1139473 | down | mRNA |
| NM_003107 | SOX4 | 0.00021633 | 0.048871331 | 1.9197969 | down | mRNA |
| NM_003112 | SP4 | 0.072500441 | 0.463255488 | 1.4135696 | down | mRNA |
| NM_003113 | SP100 | 0.881446598 | 0.992719893 | 1.0224727 | down | mRNA |
| NM_003116 | SPAG4 | 0.244378288 | 0.742705433 | 1.5765518 | down | mRNA |
| NM_003118 | SPARC | 0.387371458 | 0.856773191 | 1.1006142 | down | mRNA |
| NM_003119 | SPG7 | 0.009417175 | 0.204717305 | 1.2173511 | down | mRNA |
| NM_003125 | SPRR1B | 0.625863312 | 0.948851902 | 1.0866357 | down | mRNA |
| NM_003127 | SPTAN1 | 0.333188336 | 0.82335721 | 1.1857797 | down | mRNA |
| NM_003129 | SQLE | 0.686134934 | 0.960205549 | 1.0703336 | down | mRNA |
| NM_003131 | SRF | 0.269362961 | 0.769752802 | 1.2658943 | down | mRNA |
| NM_003132 | SRM | 0.503051612 | 0.910475258 | 1.1115411 | down | mRNA |
| NM_003133 | SRP9 | 0.076831646 | 0.476553092 | 1.0717866 | down | mRNA |
| NM_003134 | SRP14 | 0.862558557 | 0.989401142 | 1.0106086 | down | mRNA |
| NM_003135 | SRP19 | 0.145237237 | 0.606607127 | 1.0805765 | down | mRNA |
| NM_003136 | SRP54 | 0.662250151 | 0.958354006 | 1.03507 | down | mRNA |
| NM_003142 | SSB | 0.675708759 | 0.959531882 | 1.040532 | down | mRNA |
| NM_003143 | SSBP1 | 0.398615299 | 0.863643939 | 1.0616543 | down | mRNA |
| NM_003144 | SSR1 | 0.344389156 | 0.831959196 | 1.1640857 | down | mRNA |
| NM_003145 | SSR2 | 0.288821237 | 0.784712079 | 1.0760526 | down | mRNA |
| NM_003147 | SSX2 | 0.408766175 | 0.867591329 | 1.0449013 | down | mRNA |
| NM_003155 | STC1 | 0.258832698 | 0.759358563 | 1.8488087 | down | mRNA |
| NM_003156 | STIM1 | 0.872886594 | 0.990534615 | 1.0219641 | down | mRNA |
| NM_003160 | AURKC | 0.710401069 | 0.965462762 | 1.1815081 | down | mRNA |
| NM_003161 | RPS6KB1 | 0.125913072 | 0.578441772 | 1.1188359 | down | mRNA |
| NM_003162 | STRN | 0.062003763 | 0.439101535 | 1.2727883 | down | mRNA |
| NM_003169 | SUPT5H | 0.669267546 | 0.95930319 | 1.1184468 | down | mRNA |
| NM_003171 | SUPV3L1 | 0.428661903 | 0.877386753 | 1.0533396 | down | mRNA |
| NM_003179 | SYP | 0.470704055 | 0.898445976 | 1.133909 | down | mRNA |
| NM_003184 | TAF2 | 0.119906237 | 0.570276868 | 1.2092911 | down | mRNA |
| NM_003185 | TAF4 | 0.993221261 | 0.999872237 | 1.0008546 | down | mRNA |
| NM_003186 | TAGLN | 0.891452711 | 0.9937521 | 1.0483223 | down | mRNA |
| NM_003188 | MAP3K7 | 0.317560863 | 0.811850555 | 1.1889151 | down | mRNA |
| NM_003192 | TBCC | 0.684407106 | 0.960111041 | 1.0763479 | down | mRNA |
| NM_003194 | TBP | 0.472940258 | 0.899184677 | 1.065275 | down | mRNA |
| NM_003195 | TCEA2 | 0.85088161 | 0.987678166 | 1.0429843 | down | mRNA |
| NM_003198 | TCEB3 | 0.172282689 | 0.6499464 | 1.1125265 | down | mRNA |
| NM_003199 | TCF4 | 0.331822949 | 0.822255387 | 1.0669906 | down | mRNA |
| NM_003200 | TCF3 | 0.442400134 | 0.88419144 | 1.0286323 | down | mRNA |
| NM_003201 | TFAM | 0.947682323 | 0.996710991 | 1.0059821 | down | mRNA |
| NM_003202 | TCF7 | 0.587749774 | 0.93579682 | 1.2093344 | down | mRNA |
| NM_003203 | GCFC2 | 0.301724015 | 0.796890463 | 1.0912905 | down | mRNA |
| NM_003211 | TDG | 0.10031905 | 0.52637082 | 1.2118871 | down | mRNA |
| NM_003213 | TEAD4 | 0.000429833 | 0.064097442 | 1.7059385 | down | mRNA |
| NM_003214 | TEAD3 | 0.385002348 | 0.855147437 | 1.2497038 | down | mRNA |
| NM_003216 | TEF | 0.225321533 | 0.720303223 | 1.1097994 | down | mRNA |
| NM_003217 | TMBIM6 | 0.996757873 | 0.999872237 | 1.0005282 | down | mRNA |
| NM_003218 | TERF1 | 0.662238379 | 0.958354006 | 1.0812082 | down | mRNA |
| NM_003222 | TFAP2C | 0.794568836 | 0.979378567 | 1.0517462 | down | mRNA |
| NM_003223 | TFAP4 | 0.912174687 | 0.995342392 | 1.0138338 | down | mRNA |
| NM_003238 | TGFB2 | 0.844442375 | 0.987338076 | 1.0505851 | down | mRNA |
| NM_003246 | THBS1 | 0.623714315 | 0.948341211 | 1.2855856 | down | mRNA |
| NM_003247 | THBS2 | 0.763999461 | 0.973773636 | 1.1537292 | down | mRNA |
| NM_003249 | THOP1 | 0.72352632 | 0.966191059 | 1.1182834 | down | mRNA |
| NM_003252 | TIAL1 | 0.02032795 | 0.285032405 | 1.253104 | down | mRNA |
| NM_003253 | TIAM1 | 0.372996372 | 0.84810582 | 1.1408264 | down | mRNA |
| NM_003256 | TIMP4 | 0.666549572 | 0.958354006 | 1.0614573 | down | mRNA |
| NM_003257 | TJP1 | 0.531289725 | 0.919882712 | 1.0220693 | down | mRNA |
| NM_003258 | TK1 | 0.893842347 | 0.993978977 | 1.0589424 | down | mRNA |
| NM_003260 | TLE2 | 0.645265299 | 0.954911862 | 1.0942238 | down | mRNA |
| NM_003262 | SEC62 | 0.581577956 | 0.934005566 | 1.0570286 | down | mRNA |
| NM_003268 | TLR5 | 0.707193167 | 0.965401045 | 1.0853448 | down | mRNA |
| NM_003269 | NR2E1 | 0.507711525 | 0.911674633 | 1.1234976 | down | mRNA |
| NM_003270 | TSPAN6 | 0.66236361 | 0.958354006 | 1.0825962 | down | mRNA |
| NM_003272 | GPR137B | 0.504493972 | 0.910568383 | 1.0513245 | down | mRNA |
| NM_003274 | TRAPPC10 | 0.658687085 | 0.958354006 | 1.0481362 | down | mRNA |
| NM_003276 | TMPO | 0.631562262 | 0.950475493 | 1.1027504 | down | mRNA |
| NM_003279 | TNNC2 | 0.074739377 | 0.469867247 | 1.4833513 | down | mRNA |
| NM_003283 | TNNT1 | 0.516891866 | 0.91493866 | 1.0381407 | down | mRNA |
| NM_003288 | TPD52L2 | 0.390920911 | 0.858920947 | 1.0959156 | down | mRNA |
| NM_003289 | TPM2 | 0.817800092 | 0.981727075 | 1.1047518 | down | mRNA |
| NM_003292 | TPR | 0.660549995 | 0.958354006 | 1.0490682 | down | mRNA |
| NM_003297 | NR2C1 | 0.043506671 | 0.384598968 | 1.3358263 | down | mRNA |
| NM_003298 | NR2C2 | 0.130656476 | 0.585262717 | 1.2935485 | down | mRNA |
| NM_003299 | HSP90B1 | 0.944231012 | 0.996710991 | 1.0119501 | down | mRNA |
| NM_003301 | TRHR | 0.845808924 | 0.987338076 | 1.0505599 | down | mRNA |
| NM_003302 | TRIP6 | 0.125858146 | 0.578441772 | 1.1352441 | down | mRNA |
| NM_003308 | TSPY1 | 0.343949126 | 0.831629639 | 1.2182288 | down | mRNA |
| NM_003310 | TSSC1 | 0.723673163 | 0.966191059 | 1.0338477 | down | mRNA |
| NM_003312 | TST | 0.400323961 | 0.864237287 | 1.0322955 | down | mRNA |
| NM_003314 | TTC1 | 0.04805437 | 0.398338064 | 1.1595084 | down | mRNA |
| NM_003316 | TTC3 | 0.000484158 | 0.066965341 | 1.2072626 | down | mRNA |
| NM_003317 | NKX2-1 | 0.662404498 | 0.958354006 | 1.0356113 | down | mRNA |
| NM_003319 | TTN | 0.711105777 | 0.96568622 | 1.1367934 | down | mRNA |
| NM_003325 | HIRA | 0.077400774 | 0.478473478 | 1.1414585 | down | mRNA |
| NM_003329 | TXN | 0.898327109 | 0.994375399 | 1.0091928 | down | mRNA |
| NM_003330 | TXNRD1 | 0.42507657 | 0.874882692 | 1.0467844 | down | mRNA |
| NM_003336 | UBE2A | 0.71513919 | 0.966191059 | 1.0251192 | down | mRNA |
| NM_003338 | UBE2D1 | 0.808568794 | 0.98118927 | 1.0255959 | down | mRNA |
| NM_003342 | UBE2G1 | 0.008511452 | 0.199121848 | 1.1073037 | down | mRNA |
| NM_003348 | UBE2N | 0.625854104 | 0.948851902 | 1.022477 | down | mRNA |
| NM_003350 | UBE2V2 | 0.059273692 | 0.432586127 | 1.1554835 | down | mRNA |
| NM_003353 | UCN | 0.201849347 | 0.690328163 | 1.1704727 | down | mRNA |
| NM_003356 | UCP3 | 0.123188163 | 0.576181673 | 1.3784347 | down | mRNA |
| NM_003357 | SCGB1A1 | 0.92614404 | 0.995699344 | 1.013386 | down | mRNA |
| NM_003359 | UGDH | 0.449954621 | 0.888279993 | 1.1724868 | down | mRNA |
| NM_003362 | UNG | 0.026000258 | 0.315604326 | 1.2671905 | down | mRNA |
| NM_003363 | USP4 | 0.093382219 | 0.512497334 | 1.1593556 | down | mRNA |
| NM_003365 | UQCRC1 | 0.751971268 | 0.970725371 | 1.0890719 | down | mRNA |
| NM_003366 | UQCRC2 | 0.924167952 | 0.995456159 | 1.0088481 | down | mRNA |
| NM_003368 | USP1 | 0.656594155 | 0.957445617 | 1.1441514 | down | mRNA |
| NM_003370 | VASP | 0.520442817 | 0.916605148 | 1.0317006 | down | mRNA |
| NM_003371 | VAV2 | 0.511902648 | 0.912870256 | 1.07964 | down | mRNA |
| NM_003372 | VBP1 | 0.496225099 | 0.908286704 | 1.071189 | down | mRNA |
| NM_003373 | VCL | 0.410851996 | 0.868341245 | 1.1980521 | down | mRNA |
| NM_003378 | VGF | 0.877391123 | 0.991306146 | 1.0168642 | down | mRNA |
| NM_003383 | VLDLR | 0.128552456 | 0.582242044 | 1.1218409 | down | mRNA |
| NM_003384 | VRK1 | 0.973115052 | 0.998501532 | 1.010613 | down | mRNA |
| NM_003385 | VSNL1 | 0.481066192 | 0.901538317 | 1.1521448 | down | mRNA |
| NM_003387 | WIPF1 | 0.725614251 | 0.966191059 | 1.05585 | down | mRNA |
| NM_003390 | WEE1 | 0.477064645 | 0.900429473 | 1.2724461 | down | mRNA |
| NM_003392 | WNT5A | 0.738069104 | 0.969207218 | 1.0378032 | down | mRNA |
| NM_003400 | XPO1 | 0.022965457 | 0.298169644 | 1.2080985 | down | mRNA |
| NM_003403 | YY1 | 0.012041545 | 0.222675671 | 1.2447672 | down | mRNA |
| NM_003405 | YWHAH | 0.031100345 | 0.34055236 | 1.2718504 | down | mRNA |
| NM_003406 | YWHAZ | 0.432960279 | 0.88061258 | 1.1390726 | down | mRNA |
| NM_003408 | ZFP37 | 0.003022525 | 0.130211341 | 1.4053233 | down | mRNA |
| NM_003416 | ZNF7 | 0.043222755 | 0.383753983 | 1.1958393 | down | mRNA |
| NM_003418 | CNBP | 0.078370742 | 0.48040541 | 1.1578642 | down | mRNA |
| NM_003419 | ZNF345 | 0.009321589 | 0.204533521 | 1.5012984 | down | mRNA |
| NM_003423 | ZNF43 | 0.362359692 | 0.843173209 | 1.3131865 | down | mRNA |
| NM_003428 | ZNF84 | 0.194458765 | 0.682224934 | 1.1804661 | down | mRNA |
| NM_003430 | ZNF91 | 0.003784424 | 0.142490569 | 1.2584622 | down | mRNA |
| NM_003432 | ZNF131 | 0.001800483 | 0.105670067 | 1.5640129 | down | mRNA |
| NM_003433 | ZNF132 | 0.037916762 | 0.363471765 | 1.1934147 | down | mRNA |
| NM_003435 | ZNF134 | 0.512787617 | 0.913297047 | 1.1391444 | down | mRNA |
| NM_003436 | ZNF135 | 0.252728995 | 0.752986167 | 1.1140416 | down | mRNA |
| NM_003441 | ZNF141 | 0.088288158 | 0.502399895 | 1.3353507 | down | mRNA |
| NM_003450 | ZNF174 | 0.000902643 | 0.083882581 | 2.3660466 | down | mRNA |
| NM_003454 | ZNF200 | 0.175349927 | 0.653962789 | 1.2740599 | down | mRNA |
| NM_003455 | ZNF202 | 0.307642077 | 0.802348229 | 1.4223712 | down | mRNA |
| NM_003457 | ZNF207 | 0.680097892 | 0.960060427 | 1.0282805 | down | mRNA |
| NM_003461 | ZYX | 0.510714167 | 0.912267627 | 1.2216401 | down | mRNA |
| NM_003463 | PTP4A1 | 0.747000645 | 0.970432947 | 1.0236848 | down | mRNA |
| NM_003466 | PAX8 | 0.211981341 | 0.703329735 | 1.0717938 | down | mRNA |
| NM_003469 | SCG2 | 0.859177574 | 0.988512246 | 1.014728 | down | mRNA |
| NM_003472 | DEK | 0.714029637 | 0.966110889 | 1.0398809 | down | mRNA |
| NM_003473 | STAM | 0.589648515 | 0.936090876 | 1.0453821 | down | mRNA |
| NM_003474 | ADAM12 | 0.544149439 | 0.923577434 | 1.0420827 | down | mRNA |
| NM_003477 | PDHX | 0.356203248 | 0.838911669 | 1.0910069 | down | mRNA |
| NM_003478 | CUL5 | 0.093304079 | 0.512441525 | 1.1682626 | down | mRNA |
| NM_003483 | HMGA2 | 0.987969096 | 0.999140676 | 1.0018478 | down | mRNA |
| NM_003490 | SYN3 | 0.330326495 | 0.821797487 | 1.0861333 | down | mRNA |
| NM_003491 | NAA10 | 0.194927512 | 0.682615599 | 1.0673996 | down | mRNA |
| NM_003494 | DYSF | 0.603500337 | 0.941484604 | 1.0418519 | down | mRNA |
| NM_003495 | HIST1H4I | 0.677428821 | 0.959531882 | 1.5211341 | down | mRNA |
| NM_003500 | ACOX2 | 0.750735097 | 0.970725371 | 1.1863186 | down | mRNA |
| NM_003501 | ACOX3 | 0.496745668 | 0.908665955 | 1.0977411 | down | mRNA |
| NM_003502 | AXIN1 | 0.567844428 | 0.931705674 | 1.1323771 | down | mRNA |
| NM_003505 | FZD1 | 0.003944352 | 0.144623204 | 1.3543898 | down | mRNA |
| NM_003509 | HIST1H2AI | 0.904465046 | 0.99508468 | 1.1163653 | down | mRNA |
| NM_003522 | HIST1H2BF | 0.84133886 | 0.987068842 | 1.2888666 | down | mRNA |
| NM_003525 | HIST1H2BI | 0.556047448 | 0.926833616 | 1.5814827 | down | mRNA |
| NM_003526 | HIST1H2BC | 0.952817375 | 0.996710991 | 1.0337494 | down | mRNA |
| NM_003530 | HIST1H3D | 0.82489806 | 0.98381451 | 1.1044282 | down | mRNA |
| NM_003542 | HIST1H4C | 0.84490132 | 0.987338076 | 1.0636912 | down | mRNA |
| NM_003545 | HIST1H4E | 0.987708345 | 0.999140676 | 1.018934 | down | mRNA |
| NM_003547 | HIST1H4G | 0.841242388 | 0.987068842 | 1.2428486 | down | mRNA |
| NM_003549 | HYAL3 | 0.353229179 | 0.836266379 | 1.142808 | down | mRNA |
| NM_003559 | PIP4K2B | 0.765683564 | 0.974637089 | 1.0472191 | down | mRNA |
| NM_003562 | SLC25A11 | 0.24797561 | 0.746165474 | 1.0722127 | down | mRNA |
| NM_003566 | EEA1 | 0.287638846 | 0.783845732 | 1.0744578 | down | mRNA |
| NM_003569 | STX7 | 0.430538469 | 0.879151165 | 1.1109239 | down | mRNA |
| NM_003576 | STK24 | 0.284406739 | 0.78179061 | 1.1359966 | down | mRNA |
| NM_003580 | NSMAF | 0.578725744 | 0.933821303 | 1.0395588 | down | mRNA |
| NM_003581 | NCK2 | 0.157086052 | 0.625758935 | 1.1646484 | down | mRNA |
| NM_003583 | DYRK2 | 0.329256064 | 0.821768501 | 1.2866639 | down | mRNA |
| NM_003584 | DUSP11 | 0.400932751 | 0.864768873 | 1.0663936 | down | mRNA |
| NM_003585 | DOC2B | 0.432956918 | 0.88061258 | 1.3661372 | down | mRNA |
| NM_003586 | DOC2A | 0.905668715 | 0.99508468 | 1.0078108 | down | mRNA |
| NM_003587 | DHX16 | 0.992115639 | 0.999743458 | 1.0011109 | down | mRNA |
| NM_003589 | CUL4A | 0.445915375 | 0.886251358 | 1.1177028 | down | mRNA |
| NM_003590 | CUL3 | 0.121657562 | 0.573155624 | 1.1872331 | down | mRNA |
| NM_003591 | CUL2 | 0.351113705 | 0.835187247 | 1.0769197 | down | mRNA |
| NM_003592 | CUL1 | 0.001043448 | 0.086588663 | 1.5099042 | down | mRNA |
| NM_003593 | FOXN1 | 0.746542334 | 0.970432947 | 1.060059 | down | mRNA |
| NM_003594 | TTF2 | 0.779482081 | 0.976938981 | 1.0757767 | down | mRNA |
| NM_003595 | TPST2 | 0.71445778 | 0.966110889 | 1.1350386 | down | mRNA |
| NM_003596 | TPST1 | 0.941033115 | 0.996710991 | 1.0183514 | down | mRNA |
| NM_003599 | SUPT3H | 0.845919426 | 0.987338076 | 1.019144 | down | mRNA |
| NM_003600 | AURKA | 0.285820216 | 0.782635081 | 1.4582442 | down | mRNA |
| NM_003609 | HIRIP3 | 0.451030164 | 0.888854908 | 1.1138266 | down | mRNA |
| NM_003611 | OFD1 | 0.17734423 | 0.65723949 | 1.1196075 | down | mRNA |
| NM_003613 | CILP | 0.832455492 | 0.986218684 | 1.0164072 | down | mRNA |
| NM_003616 | GEMIN2 | 0.081934811 | 0.488911764 | 1.4075526 | down | mRNA |
| NM_003619 | PRSS12 | 0.623315244 | 0.948341211 | 1.2319601 | down | mRNA |
| NM_003620 | PPM1D | 0.009810662 | 0.206905383 | 1.5385779 | down | mRNA |
| NM_003622 | PPFIBP1 | 0.240996228 | 0.739278108 | 1.0726331 | down | mRNA |
| NM_003628 | PKP4 | 0.620178689 | 0.947444797 | 1.1402048 | down | mRNA |
| NM_003630 | PEX3 | 0.361836548 | 0.842806815 | 1.0724599 | down | mRNA |
| NM_003635 | NDST2 | 0.545896003 | 0.92424063 | 1.0397769 | down | mRNA |
| NM_003636 | KCNAB2 | 0.544266096 | 0.923577434 | 1.0274657 | down | mRNA |
| NM_003637 | ITGA10 | 0.857873611 | 0.988512246 | 1.0212197 | down | mRNA |
| NM_003642 | HAT1 | 0.584409749 | 0.93461341 | 1.0946663 | down | mRNA |
| NM_003646 | DGKZ | 0.855391358 | 0.988178307 | 1.0214449 | down | mRNA |
| NM_003648 | DGKD | 0.87768127 | 0.991306146 | 1.0254691 | down | mRNA |
| NM_003651 | CSDA | 0.634510716 | 0.951279916 | 1.0386004 | down | mRNA |
| NM_003652 | CPZ | 0.685031057 | 0.960111041 | 1.0844316 | down | mRNA |
| NM_003653 | COPS3 | 0.338940869 | 0.827491085 | 1.0568348 | down | mRNA |
| NM_003655 | CBX4 | 0.863984032 | 0.989401142 | 1.055476 | down | mRNA |
| NM_003656 | CAMK1 | 0.306604458 | 0.801653799 | 1.0503791 | down | mRNA |
| NM_003664 | AP3B1 | 0.82348995 | 0.983498765 | 1.0188125 | down | mRNA |
| NM_003665 | FCN3 | 0.091774051 | 0.510858344 | 1.2091483 | down | mRNA |
| NM_003666 | BLZF1 | 0.137896801 | 0.596789644 | 1.1379138 | down | mRNA |
| NM_003668 | MAPKAPK5 | 0.083666135 | 0.493003289 | 1.0526151 | down | mRNA |
| NM_003670 | BHLHE40 | 0.22114461 | 0.716640124 | 1.0875942 | down | mRNA |
| NM_003673 | TCAP | 0.726207301 | 0.966191059 | 1.0716421 | down | mRNA |
| NM_003675 | PRPF18 | 0.104706286 | 0.535631326 | 1.1074854 | down | mRNA |
| NM_003677 | DENR | 0.201413419 | 0.689610625 | 1.1270772 | down | mRNA |
| NM_003678 | THOC5 | 0.512777404 | 0.913297047 | 1.091496 | down | mRNA |
| NM_003680 | YARS | 0.032512014 | 0.344109456 | 1.2568181 | down | mRNA |
| NM_003681 | PDXK | 0.846105923 | 0.987338076 | 1.0239198 | down | mRNA |
| NM_003683 | RRP1 | 0.02898884 | 0.331249882 | 1.2572129 | down | mRNA |
| NM_003689 | AKR7A2 | 0.69231304 | 0.961562704 | 1.0437807 | down | mRNA |
| NM_003702 | RGS20 | 0.637988994 | 0.952852353 | 1.1523788 | down | mRNA |
| NM_003703 | NOP14 | 0.940977214 | 0.996710991 | 1.0062355 | down | mRNA |
| NM_003704 | FAM193A | 0.002000559 | 0.107649983 | 1.3909706 | down | mRNA |
| NM_003708 | RDH16 | 0.521747136 | 0.916788762 | 1.0763977 | down | mRNA |
| NM_003710 | SPINT1 | 0.296235407 | 0.79250767 | 1.0686155 | down | mRNA |
| NM_003713 | PPAP2B | 0.522658409 | 0.917200998 | 1.1052505 | down | mRNA |
| NM_003714 | STC2 | 0.365603956 | 0.845008704 | 1.2064846 | down | mRNA |
| NM_003718 | CDK13 | 0.06307988 | 0.439828575 | 1.2142468 | down | mRNA |
| NM_003719 | PDE8B | 0.830894547 | 0.985710869 | 1.0334414 | down | mRNA |
| NM_003720 | PSMG1 | 0.642670857 | 0.95447352 | 1.0664583 | down | mRNA |
| NM_003721 | RFXANK | 0.556719971 | 0.926833616 | 1.0278204 | down | mRNA |
| NM_003729 | RTCA | 0.319656679 | 0.813888641 | 1.0901374 | down | mRNA |
| NM_003730 | RNASET2 | 0.9402855 | 0.996710991 | 1.0260896 | down | mRNA |
| NM_003731 | SSNA1 | 0.864500431 | 0.989401142 | 1.0323113 | down | mRNA |
| NM_003738 | PTCH2 | 0.890866435 | 0.9937521 | 1.0094187 | down | mRNA |
| NM_003742 | ABCB11 | 0.967639675 | 0.99793927 | 1.002302 | down | mRNA |
| NM_003743 | NCOA1 | 0.092849538 | 0.511603469 | 1.1892662 | down | mRNA |
| NM_003745 | SOCS1 | 0.181860498 | 0.664924727 | 1.3379171 | down | mRNA |
| NM_003747 | TNKS | 0.99645246 | 0.999872237 | 1.0002684 | down | mRNA |
| NM_003749 | IRS2 | 0.919844534 | 0.995349793 | 1.0545357 | down | mRNA |
| NM_003751 | EIF3B | 0.007256337 | 0.190657536 | 1.1168842 | down | mRNA |
| NM_003753 | EIF3D | 0.951636386 | 0.996710991 | 1.008144 | down | mRNA |
| NM_003756 | EIF3H | 0.848928363 | 0.987678166 | 1.0219441 | down | mRNA |
| NM_003762 | VAMP4 | 0.015274491 | 0.248240953 | 1.1825929 | down | mRNA |
| NM_003763 | STX16 | 0.107024123 | 0.542376487 | 1.3367327 | down | mRNA |
| NM_003769 | SRSF9 | 0.377193848 | 0.850710713 | 1.0492944 | down | mRNA |
| NM_003770 | KRT37 | 0.649021928 | 0.95550188 | 1.346208 | down | mRNA |
| NM_003779 | B4GALT3 | 0.659393838 | 0.958354006 | 1.0433271 | down | mRNA |
| NM_003789 | TRADD | 0.359415747 | 0.841033672 | 1.1044823 | down | mRNA |
| NM_003790 | TNFRSF25 | 0.174532404 | 0.653035121 | 1.0751771 | down | mRNA |
| NM_003793 | CTSF | 0.915148984 | 0.995349793 | 1.0067283 | down | mRNA |
| NM_003794 | SNX4 | 0.035366463 | 0.353699423 | 1.2028192 | down | mRNA |
| NM_003795 | SNX3 | 0.601133937 | 0.941028182 | 1.0654914 | down | mRNA |
| NM_003796 | URI1 | 0.321866123 | 0.815271211 | 1.1172012 | down | mRNA |
| NM_003797 | EED | 0.209163008 | 0.700304482 | 1.3165385 | down | mRNA |
| NM_003800 | RNGTT | 0.08275052 | 0.490806175 | 1.2501917 | down | mRNA |
| NM_003801 | GPAA1 | 0.504350995 | 0.910568383 | 1.0491883 | down | mRNA |
| NM_003809 | TNFSF12 | 0.36763238 | 0.845181673 | 1.1099166 | down | mRNA |
| NM_003812 | ADAM23 | 0.857288628 | 0.988512246 | 1.0485106 | down | mRNA |
| NM_003816 | ADAM9 | 0.468453688 | 0.897362012 | 1.1213796 | down | mRNA |
| NM_003819 | PABPC4 | 0.392230345 | 0.859493185 | 1.1386933 | down | mRNA |
| NM_003823 | TNFRSF6B | 0.336621751 | 0.826506012 | 1.1622682 | down | mRNA |
| NM_003824 | FADD | 0.045533477 | 0.390957205 | 1.3890756 | down | mRNA |
| NM_003826 | NAPG | 0.608526698 | 0.943053241 | 1.1584215 | down | mRNA |
| NM_003827 | NAPA | 0.526153335 | 0.917975454 | 1.0188376 | down | mRNA |
| NM_003829 | MPDZ | 0.387619853 | 0.856919305 | 1.1347204 | down | mRNA |
| NM_003831 | RIOK3 | 0.080164573 | 0.483876854 | 1.2636033 | down | mRNA |
| NM_003837 | FBP2 | 0.00171625 | 0.105422342 | 1.206456 | down | mRNA |
| NM_003838 | FPGT | 0.011641266 | 0.22025265 | 1.3723542 | down | mRNA |
| NM_003839 | TNFRSF11A | 0.626935385 | 0.949203235 | 1.0886546 | down | mRNA |
| NM_003841 | TNFRSF10C | 0.740934793 | 0.970165142 | 1.0215134 | down | mRNA |
| NM_003844 | TNFRSF10A | 0.817962504 | 0.981806106 | 1.0341317 | down | mRNA |
| NM_003848 | SUCLG2 | 0.256390136 | 0.757240077 | 1.1224748 | down | mRNA |
| NM_003849 | SUCLG1 | 0.319019331 | 0.812974553 | 1.0564905 | down | mRNA |
| NM_003851 | CREG1 | 0.540223692 | 0.923577434 | 1.0732632 | down | mRNA |
| NM_003852 | TRIM24 | 0.392001919 | 0.859445464 | 1.1243641 | down | mRNA |
| NM_003861 | DCAF5 | 0.009301003 | 0.204533521 | 1.3550198 | down | mRNA |
| NM_003862 | FGF18 | 0.024183327 | 0.304025779 | 1.6889089 | down | mRNA |
| NM_003863 | DPM2 | 0.552040532 | 0.926337111 | 1.069913 | down | mRNA |
| NM_003864 | SAP30 | 0.415039513 | 0.870239791 | 1.0611737 | down | mRNA |
| NM_003865 | HESX1 | 0.780381402 | 0.976938981 | 1.0555794 | down | mRNA |
| NM_003869 | CES2 | 0.343270503 | 0.831521302 | 1.1067864 | down | mRNA |
| NM_003875 | GMPS | 0.036083449 | 0.356486245 | 1.2207132 | down | mRNA |
| NM_003876 | TMEM11 | 0.013886004 | 0.237651715 | 1.3486204 | down | mRNA |
| NM_003881 | WISP2 | 0.560145345 | 0.927810602 | 1.0561856 | down | mRNA |
| NM_003883 | HDAC3 | 0.058363319 | 0.429582432 | 1.1864672 | down | mRNA |
| NM_003884 | KAT2B | 0.075905088 | 0.473352674 | 1.1644959 | down | mRNA |
| NM_003893 | LDB1 | 0.124095244 | 0.577766088 | 1.2707413 | down | mRNA |
| NM_003895 | SYNJ1 | 0.994846505 | 0.999872237 | 1.0011196 | down | mRNA |
| NM_003899 | ARHGEF7 | 0.322344606 | 0.815372756 | 1.0616067 | down | mRNA |
| NM_003902 | FUBP1 | 0.038915899 | 0.368040971 | 1.147071 | down | mRNA |
| NM_003904 | ZNF259 | 0.22799341 | 0.723854154 | 1.2934892 | down | mRNA |
| NM_003905 | NAE1 | 0.137439104 | 0.596770033 | 1.1612688 | down | mRNA |
| NM_003908 | EIF2S2 | 0.965675095 | 0.99793927 | 1.0049263 | down | mRNA |
| NM_003910 | BUD31 | 0.478838684 | 0.901271944 | 1.0347646 | down | mRNA |
| NM_003913 | PRPF4B | 0.009498507 | 0.204959264 | 1.1730509 | down | mRNA |
| NM_003915 | CPNE1 | 0.169668705 | 0.646133006 | 1.2517128 | down | mRNA |
| NM_003917 | AP1G2 | 0.913774044 | 0.995342392 | 1.021594 | down | mRNA |
| NM_003918 | GYG2 | 0.320421931 | 0.814552264 | 1.0979191 | down | mRNA |
| NM_003920 | TIMELESS | 0.277656608 | 0.777240591 | 1.1781658 | down | mRNA |
| NM_003921 | BCL10 | 0.593414959 | 0.938165712 | 1.0842227 | down | mRNA |
| NM_003922 | HERC1 | 0.673244726 | 0.959531882 | 1.0613227 | down | mRNA |
| NM_003927 | MBD2 | 0.031612473 | 0.341807319 | 1.1055553 | down | mRNA |
| NM_003934 | FUBP3 | 0.146930642 | 0.609050728 | 1.1966889 | down | mRNA |
| NM_003936 | CDK5R2 | 0.802145145 | 0.98118927 | 1.0215121 | down | mRNA |
| NM_003938 | AP3D1 | 0.439785173 | 0.883132064 | 1.0352831 | down | mRNA |
| NM_003939 | BTRC | 0.324751279 | 0.817191271 | 1.1325619 | down | mRNA |
| NM_003941 | WASL | 0.128460024 | 0.582222295 | 1.2401471 | down | mRNA |
| NM_003943 | STBD1 | 0.000547865 | 0.071405024 | 1.5811729 | down | mRNA |
| NM_003952 | RPS6KB2 | 0.413820669 | 0.86961884 | 1.0577994 | down | mRNA |
| NM_003957 | BRSK2 | 0.529622896 | 0.91899174 | 1.2036964 | down | mRNA |
| NM_003961 | RHBDL1 | 0.048180625 | 0.398701045 | 1.4004314 | down | mRNA |
| NM_003968 | UBA3 | 0.455469816 | 0.891148383 | 1.0623412 | down | mRNA |
| NM_003969 | UBE2M | 0.472929236 | 0.899184677 | 1.112251 | down | mRNA |
| NM_003972 | BTAF1 | 0.504678079 | 0.910568383 | 1.1329348 | down | mRNA |
| NM_003977 | AIP | 0.509027768 | 0.911674633 | 1.117392 | down | mRNA |
| NM_003978 | PSTPIP1 | 0.877774311 | 0.991306146 | 1.0376189 | down | mRNA |
| NM_003981 | PRC1 | 0.603386378 | 0.941484604 | 1.2252453 | down | mRNA |
| NM_003983 | SLC7A6 | 0.122325829 | 0.57455499 | 1.2731429 | down | mRNA |
| NM_003988 | PAX2 | 0.755431626 | 0.971497464 | 1.0356676 | down | mRNA |
| NM_003994 | KITLG | 0.200161499 | 0.68900633 | 1.4552608 | down | mRNA |
| NM_003995 | NPR2 | 0.27400714 | 0.774088254 | 1.2059518 | down | mRNA |
| NM_004001 | FCGR2B | 0.926208031 | 0.995699344 | 1.0169162 | down | mRNA |
| NM_004004 | GJB2 | 0.358618869 | 0.840603483 | 2.3828877 | down | mRNA |
| NM_004016 | DMD | 0.890186369 | 0.9937521 | 1.016046 | down | mRNA |
| NM_004020 | DMD | 0.305547701 | 0.801185951 | 1.1677589 | down | mRNA |
| NM_004032 | DDO | 0.194666346 | 0.682390954 | 1.2623496 | down | mRNA |
| NM_004036 | ADCY3 | 0.507293172 | 0.911674633 | 1.0636731 | down | mRNA |
| NM_004037 | AMPD2 | 0.604440064 | 0.94174007 | 1.0877934 | down | mRNA |
| NM_004040 | RHOB | 0.046372726 | 0.392259245 | 1.3713796 | down | mRNA |
| NM_004044 | ATIC | 0.69727464 | 0.962410025 | 1.0669866 | down | mRNA |
| NM_004047 | ATP6V0B | 0.371123065 | 0.846402038 | 1.0810517 | down | mRNA |
| NM_004048 | B2M | 0.557524663 | 0.926833616 | 1.0669769 | down | mRNA |
| NM_004052 | BNIP3 | 0.159080995 | 0.630863036 | 1.1493307 | down | mRNA |
| NM_004058 | CAPS | 0.221083738 | 0.716640124 | 1.122939 | down | mRNA |
| NM_004060 | CCNG1 | 0.523468767 | 0.917239107 | 1.2295119 | down | mRNA |
| NM_004061 | CDH12 | 0.186943261 | 0.670712968 | 1.268599 | down | mRNA |
| NM_004068 | AP2M1 | 0.013313943 | 0.23544335 | 1.1729036 | down | mRNA |
| NM_004072 | CMKLR1 | 0.004523639 | 0.154319835 | 1.7648607 | down | mRNA |
| NM_004075 | CRY1 | 0.151792665 | 0.617002892 | 1.4326198 | down | mRNA |
| NM_004077 | CS | 0.618348127 | 0.946956498 | 1.0713515 | down | mRNA |
| NM_004083 | DDIT3 | 0.589611812 | 0.936090876 | 1.3631184 | down | mRNA |
| NM_004085 | TIMM8A | 0.004507962 | 0.154302821 | 1.4408498 | down | mRNA |
| NM_004089 | TSC22D3 | 0.010033033 | 0.207942544 | 1.9281528 | down | mRNA |
| NM_004092 | ECHS1 | 0.818143801 | 0.981818735 | 1.0190333 | down | mRNA |
| NM_004093 | EFNB2 | 0.894674327 | 0.993978977 | 1.1314143 | down | mRNA |
| NM_004094 | EIF2S1 | 0.327350094 | 0.820319134 | 1.1125319 | down | mRNA |
| NM_004095 | EIF4EBP1 | 0.272667786 | 0.772618648 | 1.0901503 | down | mRNA |
| NM_004096 | EIF4EBP2 | 0.242977218 | 0.741217225 | 1.0936283 | down | mRNA |
| NM_004098 | EMX2 | 0.008944042 | 0.202329498 | 1.4515073 | down | mRNA |
| NM_004099 | STOM | 0.67265133 | 0.959531882 | 1.1254466 | down | mRNA |
| NM_004101 | F2RL2 | 0.808293787 | 0.98118927 | 1.0714845 | down | mRNA |
| NM_004104 | FASN | 0.542671303 | 0.923577434 | 1.0284744 | down | mRNA |
| NM_004109 | FDX1 | 0.267331597 | 0.76803533 | 1.0690638 | down | mRNA |
| NM_004110 | FDXR | 0.91506 | 0.995349793 | 1.0090216 | down | mRNA |
| NM_004111 | FEN1 | 0.96876999 | 0.998196842 | 1.0090712 | down | mRNA |
| NM_004116 | FKBP1B | 0.802843985 | 0.98118927 | 1.0336645 | down | mRNA |
| NM_004124 | GMFB | 0.15717208 | 0.625935125 | 1.1032578 | down | mRNA |
| NM_004127 | GPS1 | 0.370541742 | 0.846215286 | 1.0865055 | down | mRNA |
| NM_004128 | GTF2F2 | 0.942324531 | 0.996710991 | 1.0093352 | down | mRNA |
| NM_004130 | GYG1 | 0.718066442 | 0.966191059 | 1.0724501 | down | mRNA |
| NM_004134 | HSPA9 | 0.439017435 | 0.882895421 | 1.1510403 | down | mRNA |
| NM_004136 | IREB2 | 0.223728256 | 0.717822772 | 1.0924096 | down | mRNA |
| NM_004138 | KRT33A | 0.131085884 | 0.586042863 | 1.3996761 | down | mRNA |
| NM_004147 | DRG1 | 0.126770386 | 0.580406758 | 1.1340299 | down | mRNA |
| NM_004152 | OAZ1 | 0.741958055 | 0.970330526 | 1.0260853 | down | mRNA |
| NM_004154 | P2RY6 | 0.921286478 | 0.995349793 | 1.0068584 | down | mRNA |
| NM_004157 | PRKAR2A | 0.5438776 | 0.923577434 | 1.0643486 | down | mRNA |
| NM_004159 | PSMB8 | 0.128764084 | 0.582691153 | 1.1376615 | down | mRNA |
| NM_004160 | PYY | 0.59387541 | 0.938299318 | 1.0859935 | down | mRNA |
| NM_004161 | RAB1A | 0.517941641 | 0.915959767 | 1.1097584 | down | mRNA |
| NM_004162 | RAB5A | 0.410786384 | 0.868341245 | 1.1079105 | down | mRNA |
| NM_004169 | SHMT1 | 0.710420471 | 0.965462762 | 1.0695771 | down | mRNA |
| NM_004170 | SLC1A1 | 0.027863961 | 0.324473118 | 1.3700215 | down | mRNA |
| NM_004175 | SNRPD3 | 0.607259852 | 0.942627675 | 1.057533 | down | mRNA |
| NM_004176 | SREBF1 | 0.481765615 | 0.901874457 | 1.1235376 | down | mRNA |
| NM_004177 | STX3 | 0.132087238 | 0.587843174 | 1.4028782 | down | mRNA |
| NM_004178 | TARBP2 | 0.92510349 | 0.995465766 | 1.0090341 | down | mRNA |
| NM_004180 | TANK | 0.739131508 | 0.969564098 | 1.0543821 | down | mRNA |
| NM_004181 | UCHL1 | 0.278007789 | 0.777290204 | 1.1229754 | down | mRNA |
| NM_004186 | SEMA3F | 0.778069823 | 0.976938981 | 1.0287295 | down | mRNA |
| NM_004187 | KDM5C | 0.236737953 | 0.73465251 | 1.2351529 | down | mRNA |
| NM_004188 | GFI1B | 0.858567948 | 0.988512246 | 1.0181356 | down | mRNA |
| NM_004189 | SOX14 | 0.666340924 | 0.958354006 | 1.0573683 | down | mRNA |
| NM_004192 | ASMTL | 0.094799498 | 0.516057441 | 1.2290414 | down | mRNA |
| NM_004193 | GBF1 | 0.381947188 | 0.853514109 | 1.0316689 | down | mRNA |
| NM_004197 | STK19 | 0.379445432 | 0.852847753 | 1.0517829 | down | mRNA |
| NM_004199 | P4HA2 | 0.939933093 | 0.996710991 | 1.0204543 | down | mRNA |
| NM_004204 | PIGQ | 0.883126418 | 0.992766819 | 1.0240458 | down | mRNA |
| NM_004205 | USP2 | 0.485821875 | 0.903250293 | 1.1087407 | down | mRNA |
| NM_004206 | SEC22C | 0.462765359 | 0.894989844 | 1.0474259 | down | mRNA |
| NM_004208 | AIFM1 | 0.774700499 | 0.97589756 | 1.012038 | down | mRNA |
| NM_004210 | NEURL | 0.996572082 | 0.999872237 | 1.0005931 | down | mRNA |
| NM_004213 | SLC28A1 | 0.040253495 | 0.371915487 | 1.371933 | down | mRNA |
| NM_004214 | FIBP | 0.922146149 | 0.995349793 | 1.0051007 | down | mRNA |
| NM_004218 | RAB11B | 0.130328306 | 0.584331216 | 1.059479 | down | mRNA |
| NM_004223 | UBE2L6 | 0.304569635 | 0.800038203 | 1.089186 | down | mRNA |
| NM_004224 | GPR50 | 0.549888457 | 0.925533435 | 1.0653561 | down | mRNA |
| NM_004227 | CYTH3 | 0.026721511 | 0.318484367 | 1.3210456 | down | mRNA |
| NM_004229 | MED14 | 0.019964991 | 0.283521851 | 1.3504563 | down | mRNA |
| NM_004232 | SOCS6 | 0.598547635 | 0.940648177 | 1.0978386 | down | mRNA |
| NM_004235 | KLF4 | 0.593753595 | 0.938299318 | 1.1209008 | down | mRNA |
| NM_004236 | COPS2 | 0.032163496 | 0.3425639 | 1.2223719 | down | mRNA |
| NM_004237 | TRIP13 | 0.68705911 | 0.960205549 | 1.1970573 | down | mRNA |
| NM_004238 | TRIP12 | 0.333215169 | 0.82335721 | 1.1141555 | down | mRNA |
| NM_004239 | TRIP11 | 0.074675976 | 0.469867247 | 1.1226502 | down | mRNA |
| NM_004241 | JMJD1C | 0.000580436 | 0.073247308 | 1.7886685 | down | mRNA |
| NM_004245 | TGM5 | 0.686016376 | 0.960205549 | 1.0522308 | down | mRNA |
| NM_004249 | RAB28 | 0.432004395 | 0.880287969 | 1.1685249 | down | mRNA |
| NM_004251 | RAB9A | 0.826571345 | 0.984436279 | 1.0179135 | down | mRNA |
| NM_004252 | SLC9A3R1 | 0.38946958 | 0.858209114 | 1.1324946 | down | mRNA |
| NM_004255 | COX5A | 0.693109067 | 0.961584652 | 1.0495639 | down | mRNA |
| NM_004256 | SLC22A13 | 0.66384499 | 0.958354006 | 1.0220406 | down | mRNA |
| NM_004257 | TGFBRAP1 | 0.007040368 | 0.18703243 | 1.4472053 | down | mRNA |
| NM_004259 | RECQL5 | 0.786693757 | 0.977479172 | 1.0493083 | down | mRNA |
| NM_004261 | SEP15 | 0.806533192 | 0.98118927 | 1.0162397 | down | mRNA |
| NM_004265 | FADS2 | 0.852011944 | 0.987678166 | 1.1736666 | down | mRNA |
| NM_004267 | CHST2 | 0.524086463 | 0.917428132 | 1.0356267 | down | mRNA |
| NM_004268 | MED17 | 0.305340575 | 0.801056074 | 1.1598479 | down | mRNA |
| NM_004269 | MED27 | 0.856819559 | 0.988512246 | 1.0226448 | down | mRNA |
| NM_004272 | HOMER1 | 0.581202921 | 0.934005566 | 1.1482098 | down | mRNA |
| NM_004275 | MED20 | 0.040609374 | 0.373775369 | 1.2707037 | down | mRNA |
| NM_004278 | PIGL | 0.741632844 | 0.970240145 | 1.0539127 | down | mRNA |
| NM_004279 | PMPCB | 0.412870291 | 0.869353641 | 1.0604153 | down | mRNA |
| NM_004280 | EEF1E1 | 0.943142309 | 0.996710991 | 1.003427 | down | mRNA |
| NM_004281 | BAG3 | 0.352295502 | 0.835946719 | 1.0634473 | down | mRNA |
| NM_004282 | BAG2 | 0.087283605 | 0.500533133 | 1.6199265 | down | mRNA |
| NM_004284 | CHD1L | 0.177149243 | 0.65715084 | 1.0861939 | down | mRNA |
| NM_004294 | MTRF1 | 0.002030132 | 0.108622769 | 1.1371354 | down | mRNA |
| NM_004295 | TRAF4 | 0.588284905 | 0.93579682 | 1.0481063 | down | mRNA |
| NM_004297 | GNA14 | 0.435972972 | 0.881677506 | 1.2465651 | down | mRNA |
| NM_004300 | ACP1 | 0.485191398 | 0.903250293 | 1.1065273 | down | mRNA |
| NM_004301 | ACTL6A | 0.340463145 | 0.828628442 | 1.1334678 | down | mRNA |
| NM_004305 | BIN1 | 0.151687428 | 0.616902989 | 1.0866165 | down | mRNA |
| NM_004308 | ARHGAP1 | 0.487077823 | 0.903621334 | 1.0816212 | down | mRNA |
| NM_004311 | ARL3 | 0.257348493 | 0.757735833 | 1.1056568 | down | mRNA |
| NM_004313 | ARRB2 | 0.418705734 | 0.872328878 | 1.0864849 | down | mRNA |
| NM_004315 | ASAH1 | 0.751510465 | 0.970725371 | 1.0366666 | down | mRNA |
| NM_004316 | ASCL1 | 0.499056571 | 0.909361611 | 1.1254523 | down | mRNA |
| NM_004318 | ASPH | 0.501143156 | 0.909916293 | 1.1407729 | down | mRNA |
| NM_004319 | ASTN1 | 0.558714769 | 0.927102643 | 1.1179833 | down | mRNA |
| NM_004324 | BAX | 0.706898767 | 0.965401045 | 1.0617112 | down | mRNA |
| NM_004326 | BCL9 | 0.008131857 | 0.196966143 | 1.3123357 | down | mRNA |
| NM_004327 | BCR | 0.804506326 | 0.98118927 | 1.0814764 | down | mRNA |
| NM_004329 | BMPR1A | 0.001095964 | 0.086778252 | 1.3982006 | down | mRNA |
| NM_004330 | BNIP2 | 0.50408554 | 0.910568383 | 1.0762628 | down | mRNA |
| NM_004331 | BNIP3L | 0.563450798 | 0.929047249 | 1.0549871 | down | mRNA |
| NM_004336 | BUB1 | 0.729210264 | 0.966191059 | 1.2101989 | down | mRNA |
| NM_004337 | OSGIN2 | 0.141254524 | 0.601673229 | 1.1343657 | down | mRNA |
| NM_004341 | CAD | 0.20895116 | 0.700205031 | 1.2428018 | down | mRNA |
| NM_004342 | CALD1 | 0.756648777 | 0.971824502 | 1.1377493 | down | mRNA |
| NM_004348 | RUNX2 | 0.773276205 | 0.97589756 | 1.0294623 | down | mRNA |
| NM_004349 | RUNX1T1 | 0.190400796 | 0.676402188 | 1.2348035 | down | mRNA |
| NM_004350 | RUNX3 | 0.435289623 | 0.881504842 | 1.1056488 | down | mRNA |
| NM_004354 | CCNG2 | 0.735793389 | 0.968398491 | 1.055487 | down | mRNA |
| NM_004356 | CD81 | 0.806144831 | 0.98118927 | 1.0307463 | down | mRNA |
| NM_004358 | CDC25B | 0.474906934 | 0.899637359 | 1.2158841 | down | mRNA |
| NM_004359 | CDC34 | 0.002153142 | 0.110274545 | 1.3435172 | down | mRNA |
| NM_004369 | COL6A3 | 0.048100843 | 0.398338064 | 1.099337 | down | mRNA |
| NM_004370 | COL12A1 | 0.41306168 | 0.869486498 | 1.0647089 | down | mRNA |
| NM_004375 | COX11 | 0.336043918 | 0.826072416 | 1.1014303 | down | mRNA |
| NM_004376 | COX15 | 0.442292218 | 0.88419144 | 1.1392776 | down | mRNA |
| NM_004379 | CREB1 | 0.001337683 | 0.093785428 | 1.2835626 | down | mRNA |
| NM_004380 | CREBBP | 0.013662923 | 0.236019158 | 1.2506287 | down | mRNA |
| NM_004382 | CRHR1 | 0.099411098 | 0.523769488 | 2.0028843 | down | mRNA |
| NM_004384 | CSNK1G3 | 0.253710554 | 0.753827705 | 1.1806312 | down | mRNA |
| NM_004385 | VCAN | 0.651685025 | 0.956204521 | 1.0413545 | down | mRNA |
| NM_004389 | CTNNA2 | 0.717719536 | 0.966191059 | 1.1186719 | down | mRNA |
| NM_004390 | CTSH | 0.42392994 | 0.874798629 | 1.1886507 | down | mRNA |
| NM_004395 | DBN1 | 0.013219893 | 0.234704726 | 1.6809366 | down | mRNA |
| NM_004397 | DDX6 | 0.035674793 | 0.354652995 | 1.1217079 | down | mRNA |
| NM_004398 | DDX10 | 0.026294992 | 0.317461702 | 1.5526323 | down | mRNA |
| NM_004399 | DDX11 | 0.466640778 | 0.89599965 | 1.2836004 | down | mRNA |
| NM_004401 | DFFA | 0.010049645 | 0.207961374 | 1.2587413 | down | mRNA |
| NM_004405 | DLX2 | 0.351223055 | 0.835187247 | 1.3340049 | down | mRNA |
| NM_004407 | DMP1 | 0.721058368 | 0.966191059 | 1.0847529 | down | mRNA |
| NM_004412 | TRDMT1 | 0.453392989 | 0.889563297 | 1.0562538 | down | mRNA |
| NM_004415 | DSP | 0.449302769 | 0.888279993 | 1.287042 | down | mRNA |
| NM_004416 | DTX1 | 0.274964223 | 0.774827385 | 1.1629965 | down | mRNA |
| NM_004418 | DUSP2 | 0.988715231 | 0.999140676 | 1.0082782 | down | mRNA |
| NM_004420 | DUSP8 | 0.510961645 | 0.912481293 | 1.1229533 | down | mRNA |
| NM_004423 | DVL3 | 0.336258723 | 0.826400624 | 1.1236368 | down | mRNA |
| NM_004425 | ECM1 | 0.643972504 | 0.954605197 | 1.0562782 | down | mRNA |
| NM_004429 | EFNB1 | 0.442524242 | 0.884228603 | 1.2071973 | down | mRNA |
| NM_004434 | EML1 | 0.929166839 | 0.996456574 | 1.0127754 | down | mRNA |
| NM_004435 | ENDOG | 0.446103331 | 0.886278379 | 1.0556332 | down | mRNA |
| NM_004437 | EPB41 | 0.380790284 | 0.853514109 | 1.1145237 | down | mRNA |
| NM_004438 | EPHA4 | 0.208176918 | 0.698793461 | 1.3424136 | down | mRNA |
| NM_004442 | EPHB2 | 0.275100031 | 0.774827385 | 1.1093335 | down | mRNA |
| NM_004443 | EPHB3 | 0.008977841 | 0.202329498 | 3.7818326 | down | mRNA |
| NM_004444 | EPHB4 | 0.406780598 | 0.867591329 | 1.1590314 | down | mRNA |
| NM_004446 | EPRS | 0.314699972 | 0.809824265 | 1.1368192 | down | mRNA |
| NM_004447 | EPS8 | 0.029842668 | 0.335098261 | 1.1818286 | down | mRNA |
| NM_004450 | ERH | 0.712063377 | 0.965950933 | 1.1095285 | down | mRNA |
| NM_004451 | ESRRA | 0.18614321 | 0.66985199 | 1.207811 | down | mRNA |
| NM_004453 | ETFDH | 0.264920349 | 0.76613535 | 1.1371531 | down | mRNA |
| NM_004462 | FDFT1 | 0.471214678 | 0.898840119 | 1.1398729 | down | mRNA |
| NM_004468 | FHL3 | 0.128955914 | 0.582929618 | 1.4254269 | down | mRNA |
| NM_004472 | FOXD1 | 0.696248366 | 0.962410025 | 1.3441708 | down | mRNA |
| NM_004479 | FUT7 | 0.511244355 | 0.912481293 | 1.1688807 | down | mRNA |
| NM_004480 | FUT8 | 0.120341586 | 0.571550658 | 1.2538356 | down | mRNA |
| NM_004481 | GALNT2 | 0.786829728 | 0.977479172 | 1.0552565 | down | mRNA |
| NM_004482 | GALNT3 | 0.658038476 | 0.95832951 | 1.485035 | down | mRNA |
| NM_004489 | GPS2 | 0.172311708 | 0.6499464 | 1.0923648 | down | mRNA |
| NM_004491 | ARHGAP35 | 0.153045096 | 0.619205018 | 1.1083973 | down | mRNA |
| NM_004493 | HSD17B10 | 0.74027557 | 0.969794316 | 1.0306951 | down | mRNA |
| NM_004499 | HNRNPAB | 0.976765061 | 0.998501532 | 1.0068957 | down | mRNA |
| NM_004503 | HOXC6 | 0.970342761 | 0.998245244 | 1.0058308 | down | mRNA |
| NM_004504 | AGFG1 | 0.753834985 | 0.970798893 | 1.0468478 | down | mRNA |
| NM_004505 | USP6 | 0.248110902 | 0.746351658 | 1.1443639 | down | mRNA |
| NM_004506 | HSF2 | 0.025792398 | 0.315150866 | 1.2176367 | down | mRNA |
| NM_004507 | HUS1 | 0.640456867 | 0.953894897 | 1.0275288 | down | mRNA |
| NM_004508 | IDI1 | 0.579385661 | 0.933821303 | 1.1044589 | down | mRNA |
| NM_004514 | FOXK2 | 0.199185602 | 0.687812782 | 1.1983621 | down | mRNA |
| NM_004515 | ILF2 | 0.228217145 | 0.724338276 | 1.071437 | down | mRNA |
| NM_004518 | KCNQ2 | 0.001596347 | 0.103366039 | 1.1038746 | down | mRNA |
| NM_004521 | KIF5B | 0.122042924 | 0.574392762 | 1.1823545 | down | mRNA |
| NM_004523 | KIF11 | 0.601260253 | 0.941028182 | 1.2961296 | down | mRNA |
| NM_004529 | MLLT3 | 0.309234123 | 0.803961878 | 1.296027 | down | mRNA |
| NM_004531 | MOCS2 | 0.14837998 | 0.611309209 | 1.1270681 | down | mRNA |
| NM_004532 | MUC4 | 0.480747354 | 0.901538317 | 1.0400578 | down | mRNA |
| NM_004537 | NAP1L1 | 0.670772273 | 0.959531882 | 1.044241 | down | mRNA |
| NM_004539 | NARS | 0.127564785 | 0.580991132 | 1.2264176 | down | mRNA |
| NM_004541 | NDUFA1 | 0.948379755 | 0.996710991 | 1.0043729 | down | mRNA |
| NM_004546 | NDUFB2 | 0.515697471 | 0.914422683 | 1.035276 | down | mRNA |
| NM_004548 | NDUFB10 | 0.286717162 | 0.782942227 | 1.0808438 | down | mRNA |
| NM_004550 | NDUFS2 | 0.154464379 | 0.621861274 | 1.130906 | down | mRNA |
| NM_004551 | NDUFS3 | 0.278990228 | 0.777816764 | 1.0678706 | down | mRNA |
| NM_004554 | NFATC4 | 0.424885146 | 0.874854499 | 1.1342479 | down | mRNA |
| NM_004555 | NFATC3 | 0.017365757 | 0.265474108 | 1.5707642 | down | mRNA |
| NM_004559 | YBX1 | 0.530236563 | 0.91899174 | 1.0867691 | down | mRNA |
| NM_004560 | ROR2 | 0.72759862 | 0.966191059 | 1.0228565 | down | mRNA |
| NM_004561 | OVOL1 | 0.922211738 | 0.995349793 | 1.0178249 | down | mRNA |
| NM_004563 | PCK2 | 0.649793381 | 0.95550188 | 1.0749175 | down | mRNA |
| NM_004564 | PET112 | 0.100101682 | 0.52577303 | 1.2098686 | down | mRNA |
| NM_004568 | SERPINB6 | 0.455391733 | 0.891148383 | 1.044237 | down | mRNA |
| NM_004569 | PIGH | 0.003436788 | 0.137823993 | 1.2135165 | down | mRNA |
| NM_004572 | PKP2 | 0.923709917 | 0.995456159 | 1.0133967 | down | mRNA |
| NM_004577 | PSPH | 0.519058629 | 0.916145597 | 1.021523 | down | mRNA |
| NM_004578 | RAB4A | 0.427154125 | 0.876731038 | 1.1183745 | down | mRNA |
| NM_004581 | RABGGTA | 0.883848508 | 0.992766819 | 1.0177844 | down | mRNA |
| NM_004587 | RRBP1 | 0.644282633 | 0.954657712 | 1.0351787 | down | mRNA |
| NM_004589 | SCO1 | 0.240398852 | 0.738437735 | 1.0966703 | down | mRNA |
| NM_004592 | SFSWAP | 0.23229895 | 0.730226074 | 1.0724501 | down | mRNA |
| NM_004593 | TRA2B | 0.164828573 | 0.638119216 | 1.1026173 | down | mRNA |
| NM_004597 | SNRPD2 | 0.70906833 | 0.965462762 | 1.0279409 | down | mRNA |
| NM_004600 | TROVE2 | 0.39911462 | 0.863643939 | 1.0541102 | down | mRNA |
| NM_004603 | STX1A | 0.468612879 | 0.89739427 | 1.1505977 | down | mRNA |
| NM_004606 | TAF1 | 0.320387692 | 0.814552264 | 1.1517123 | down | mRNA |
| NM_004613 | TGM2 | 0.250149444 | 0.748827812 | 1.2135152 | down | mRNA |
| NM_004616 | TSPAN8 | 0.84264702 | 0.987190965 | 1.1067272 | down | mRNA |
| NM_004617 | TM4SF4 | 0.665106552 | 0.958354006 | 1.0333044 | down | mRNA |
| NM_004620 | TRAF6 | 0.088467075 | 0.502587299 | 1.1008092 | down | mRNA |
| NM_004622 | TSN | 0.070029724 | 0.457987595 | 1.2129905 | down | mRNA |
| NM_004623 | TTC4 | 0.718657979 | 0.966191059 | 1.0811777 | down | mRNA |
| NM_004624 | VIPR1 | 0.550967152 | 0.925960004 | 1.1581994 | down | mRNA |
| NM_004627 | WRB | 0.142913338 | 0.603147721 | 1.1938545 | down | mRNA |
| NM_004628 | XPC | 0.826372896 | 0.984423205 | 1.0152677 | down | mRNA |
| NM_004631 | LRP8 | 0.995953999 | 0.999872237 | 1.0021422 | down | mRNA |
| NM_004633 | IL1R2 | 0.422644335 | 0.873989281 | 1.2281231 | down | mRNA |
| NM_004635 | MAPKAPK3 | 0.581696024 | 0.934005566 | 1.0872128 | down | mRNA |
| NM_004637 | RAB7A | 0.952099291 | 0.996710991 | 1.0035323 | down | mRNA |
| NM_004641 | MLLT10 | 0.078374225 | 0.48040541 | 1.4654358 | down | mRNA |
| NM_004642 | CDK2AP1 | 0.594717926 | 0.938655001 | 1.0523444 | down | mRNA |
| NM_004643 | PABPN1 | 0.948162937 | 0.996710991 | 1.0092069 | down | mRNA |
| NM_004645 | COIL | 0.001476611 | 0.098148183 | 1.4321499 | down | mRNA |
| NM_004647 | DPF1 | 0.047700344 | 0.397835916 | 1.1665774 | down | mRNA |
| NM_004649 | C21orf33 | 0.399269533 | 0.863703388 | 1.0730942 | down | mRNA |
| NM_004651 | USP11 | 0.167859567 | 0.6427346 | 1.222445 | down | mRNA |
| NM_004653 | KDM5D | 0.575604366 | 0.933656777 | 1.1010285 | down | mRNA |
| NM_004654 | USP9Y | 0.459043983 | 0.892904132 | 1.2237787 | down | mRNA |
| NM_004655 | AXIN2 | 0.36821484 | 0.845312226 | 1.1121673 | down | mRNA |
| NM_004661 | CDC23 | 0.12151009 | 0.573155624 | 1.0721218 | down | mRNA |
| NM_004663 | RAB11A | 0.77044688 | 0.975347132 | 1.0206928 | down | mRNA |
| NM_004664 | LIN7A | 0.965725407 | 0.99793927 | 1.0344553 | down | mRNA |
| NM_004669 | CLIC3 | 0.586449987 | 0.935504488 | 1.1130643 | down | mRNA |
| NM_004671 | PIAS2 | 0.063401992 | 0.440687083 | 1.1740772 | down | mRNA |
| NM_004674 | ASH2L | 0.244002957 | 0.742391426 | 1.1038087 | down | mRNA |
| NM_004679 | VCY | 0.20999524 | 0.700742363 | 1.4490591 | down | mRNA |
| NM_004685 | MTMR6 | 0.019476522 | 0.28064964 | 1.2678256 | down | mRNA |
| NM_004687 | MTMR4 | 0.599607949 | 0.940648177 | 1.0761424 | down | mRNA |
| NM_004689 | MTA1 | 0.463653717 | 0.894989844 | 1.0578641 | down | mRNA |
| NM_004691 | ATP6V0D1 | 0.390856005 | 0.858920947 | 1.0744706 | down | mRNA |
| NM_004695 | SLC16A5 | 0.846552637 | 0.98761236 | 1.0387428 | down | mRNA |
| NM_004697 | PRPF4 | 0.020038407 | 0.283521851 | 1.3205022 | down | mRNA |
| NM_004701 | CCNB2 | 0.914131792 | 0.995342392 | 1.0533051 | down | mRNA |
| NM_004703 | RABEP1 | 0.269696752 | 0.76993462 | 1.1472172 | down | mRNA |
| NM_004704 | RRP9 | 0.013985995 | 0.238634683 | 1.2195073 | down | mRNA |
| NM_004706 | ARHGEF1 | 0.27830064 | 0.77746752 | 1.1723232 | down | mRNA |
| NM_004707 | ATG12 | 0.26025277 | 0.760594985 | 1.1085472 | down | mRNA |
| NM_004711 | SYNGR1 | 0.456830207 | 0.891568474 | 1.0876762 | down | mRNA |
| NM_004713 | NEMF | 0.785720047 | 0.977479172 | 1.0190593 | down | mRNA |
| NM_004714 | DYRK1B | 0.842801233 | 0.987257859 | 1.0410229 | down | mRNA |
| NM_004716 | PCSK7 | 0.124439094 | 0.578441772 | 1.1886346 | down | mRNA |
| NM_004718 | COX7A2L | 0.685805268 | 0.960111041 | 1.0577699 | down | mRNA |
| NM_004719 | SCAF11 | 0.047737112 | 0.397835916 | 1.2186318 | down | mRNA |
| NM_004722 | AP4M1 | 0.315172561 | 0.810240865 | 1.0905932 | down | mRNA |
| NM_004724 | ZW10 | 0.184928523 | 0.668140272 | 1.1925221 | down | mRNA |
| NM_004725 | BUB3 | 0.313585222 | 0.808703036 | 1.0953255 | down | mRNA |
| NM_004726 | REPS2 | 0.927842889 | 0.995916751 | 1.024157 | down | mRNA |
| NM_004729 | ZBED1 | 0.122805573 | 0.574983504 | 1.1714291 | down | mRNA |
| NM_004730 | ETF1 | 0.6556309 | 0.957222998 | 1.0289432 | down | mRNA |
| NM_004731 | SLC16A7 | 0.884435729 | 0.99276855 | 1.026739 | down | mRNA |
| NM_004733 | SLC33A1 | 0.39070968 | 0.858920947 | 1.2216586 | down | mRNA |
| NM_004737 | LARGE | 0.144734358 | 0.605951408 | 1.1706502 | down | mRNA |
| NM_004738 | VAPB | 0.019447842 | 0.280600624 | 1.2517948 | down | mRNA |
| NM_004744 | LRAT | 0.809978453 | 0.98118927 | 1.0562797 | down | mRNA |
| NM_004750 | CRLF1 | 0.281495564 | 0.779126326 | 1.2007979 | down | mRNA |
| NM_004753 | DHRS3 | 0.598176295 | 0.940394373 | 1.1386305 | down | mRNA |
| NM_004755 | RPS6KA5 | 0.723107424 | 0.966191059 | 1.0992273 | down | mRNA |
| NM_004756 | NUMBL | 0.299549633 | 0.795720295 | 1.0851444 | down | mRNA |
| NM_004757 | AIMP1 | 0.944437128 | 0.996710991 | 1.0059433 | down | mRNA |
| NM_004760 | STK17A | 0.0134376 | 0.235533862 | 1.4466685 | down | mRNA |
| NM_004761 | RGL2 | 0.846102369 | 0.987338076 | 1.0503902 | down | mRNA |
| NM_004763 | ITGB1BP1 | 0.455351715 | 0.891148383 | 1.0791604 | down | mRNA |
| NM_004765 | BCL7C | 0.160426832 | 0.631980676 | 1.1450834 | down | mRNA |
| NM_004769 | ASIC3 | 0.374495582 | 0.84936907 | 1.0896585 | down | mRNA |
| NM_004773 | ZNHIT3 | 0.121667306 | 0.573155624 | 1.1677059 | down | mRNA |
| NM_004774 | MED1 | 0.242078621 | 0.740587198 | 1.1174011 | down | mRNA |
| NM_004779 | CNOT8 | 0.012089627 | 0.222675671 | 1.2725982 | down | mRNA |
| NM_004780 | TCEAL1 | 0.152435202 | 0.618255775 | 1.0663822 | down | mRNA |
| NM_004782 | SNAP29 | 0.308687928 | 0.803800359 | 1.1456174 | down | mRNA |
| NM_004785 | SLC9A3R2 | 0.220124272 | 0.715236067 | 1.0741292 | down | mRNA |
| NM_004786 | TXNL1 | 0.165832175 | 0.639669852 | 1.0774812 | down | mRNA |
| NM_004788 | UBE4A | 0.66508842 | 0.958354006 | 1.0269577 | down | mRNA |
| NM_004790 | SLC22A6 | 0.266860281 | 0.767440345 | 1.1724963 | down | mRNA |
| NM_004792 | PPIG | 0.20932791 | 0.700367256 | 1.1238237 | down | mRNA |
| NM_004797 | ADIPOQ | 0.412689709 | 0.869333523 | 1.0802544 | down | mRNA |
| NM_004799 | ZFYVE9 | 0.085394272 | 0.497360491 | 1.2120281 | down | mRNA |
| NM_004800 | TM9SF2 | 0.483114744 | 0.902654749 | 1.1244912 | down | mRNA |
| NM_004804 | CIAO1 | 0.239685248 | 0.737916177 | 1.0798933 | down | mRNA |
| NM_004805 | POLR2D | 0.705414176 | 0.964784139 | 1.0824169 | down | mRNA |
| NM_004807 | HS6ST1 | 0.47164594 | 0.898908705 | 1.2314958 | down | mRNA |
| NM_004808 | NMT2 | 0.912983571 | 0.995342392 | 1.0149688 | down | mRNA |
| NM_004809 | STOML1 | 0.233290166 | 0.731130266 | 1.1527163 | down | mRNA |
| NM_004810 | GRAP2 | 0.739317631 | 0.969564098 | 1.0434427 | down | mRNA |
| NM_004813 | PEX16 | 0.333279059 | 0.82335721 | 1.0817884 | down | mRNA |
| NM_004814 | SNRNP40 | 0.107513921 | 0.543639156 | 1.1405595 | down | mRNA |
| NM_004815 | ARHGAP29 | 0.079847747 | 0.483605717 | 1.316923 | down | mRNA |
| NM_004818 | DDX23 | 0.010083681 | 0.208143549 | 1.2308054 | down | mRNA |
| NM_004819 | SYMPK | 0.054936274 | 0.419520587 | 1.1045992 | down | mRNA |
| NM_004823 | KCNK6 | 0.466432536 | 0.89599965 | 1.0900744 | down | mRNA |
| NM_004826 | ECEL1 | 0.957056245 | 0.996883425 | 1.0160107 | down | mRNA |
| NM_004830 | MED23 | 0.0137325 | 0.236215185 | 1.2535352 | down | mRNA |
| NM_004834 | MAP4K4 | 0.965845467 | 0.99793927 | 1.0058394 | down | mRNA |
| NM_004836 | EIF2AK3 | 0.330424673 | 0.821797487 | 1.2027128 | down | mRNA |
| NM_004839 | HOMER2 | 0.153865457 | 0.620938377 | 1.1744552 | down | mRNA |
| NM_004841 | RASAL2 | 0.743864356 | 0.970330526 | 1.0705656 | down | mRNA |
| NM_004843 | IL27RA | 0.569015139 | 0.93232103 | 1.0432713 | down | mRNA |
| NM_004844 | SH3BP5 | 0.063049776 | 0.439769486 | 1.3489898 | down | mRNA |
| NM_004848 | THEMIS2 | 0.54894405 | 0.925390135 | 1.1480024 | down | mRNA |
| NM_004849 | ATG5 | 0.09548372 | 0.516597924 | 1.1924835 | down | mRNA |
| NM_004850 | ROCK2 | 0.364550496 | 0.843907627 | 1.1565524 | down | mRNA |
| NM_004854 | CHST10 | 0.172160633 | 0.6499464 | 1.3962923 | down | mRNA |
| NM_004855 | PIGB | 0.399457025 | 0.863982495 | 1.1096887 | down | mRNA |
| NM_004856 | KIF23 | 0.394473055 | 0.861165014 | 1.3474948 | down | mRNA |
| NM_004859 | CLTC | 0.09761178 | 0.520535504 | 1.347005 | down | mRNA |
| NM_004860 | FXR2 | 0.439914503 | 0.883272235 | 1.1009473 | down | mRNA |
| NM_004863 | SPTLC2 | 0.032973243 | 0.345751409 | 1.3280977 | down | mRNA |
| NM_004865 | TBPL1 | 0.009359365 | 0.204533521 | 1.2417396 | down | mRNA |
| NM_004866 | SCAMP1 | 0.726505322 | 0.966191059 | 1.0396171 | down | mRNA |
| NM_004869 | VPS4B | 0.051646096 | 0.41031217 | 1.1645635 | down | mRNA |
| NM_004870 | MPDU1 | 0.93022948 | 0.996649933 | 1.0126671 | down | mRNA |
| NM_004871 | GOSR1 | 0.07642956 | 0.475509737 | 1.1748658 | down | mRNA |
| NM_004874 | BAG4 | 0.030147546 | 0.336976308 | 1.1875569 | down | mRNA |
| NM_004879 | EI24 | 0.116508766 | 0.562996237 | 1.1436989 | down | mRNA |
| NM_004882 | CIR1 | 0.298937369 | 0.795233272 | 1.0885523 | down | mRNA |
| NM_004888 | ATP6V1G1 | 0.443246566 | 0.884425653 | 1.044593 | down | mRNA |
| NM_004890 | SPAG7 | 0.938916429 | 0.996710991 | 1.0065959 | down | mRNA |
| NM_004896 | VPS26A | 0.79806134 | 0.980749101 | 1.0222542 | down | mRNA |
| NM_004898 | CLOCK | 0.139417607 | 0.598648178 | 1.1089016 | down | mRNA |
| NM_004900 | APOBEC3B | 0.938215542 | 0.996710991 | 1.0371309 | down | mRNA |
| NM_004901 | ENTPD4 | 0.602898772 | 0.941464987 | 1.1249448 | down | mRNA |
| NM_004902 | RBM39 | 0.006345629 | 0.180328763 | 1.3446181 | down | mRNA |
| NM_004910 | PITPNM1 | 0.729573529 | 0.966191059 | 1.0826632 | down | mRNA |
| NM_004911 | PDIA4 | 0.861990599 | 0.989401142 | 1.0280656 | down | mRNA |
| NM_004913 | C16orf7 | 0.411115987 | 0.868434057 | 1.1460783 | down | mRNA |
| NM_004914 | RAB36 | 0.103961396 | 0.533896783 | 1.1438613 | down | mRNA |
| NM_004923 | MTL5 | 0.486328408 | 0.903524001 | 1.0456154 | down | mRNA |
| NM_004927 | MRPL49 | 0.697327672 | 0.962410025 | 1.0652711 | down | mRNA |
| NM_004933 | CDH15 | 0.883549328 | 0.992766819 | 1.0262273 | down | mRNA |
| NM_004935 | CDK5 | 0.122333918 | 0.57455499 | 1.1044275 | down | mRNA |
| NM_004937 | CTNS | 0.036963798 | 0.359628653 | 1.2974846 | down | mRNA |
| NM_004939 | DDX1 | 0.670035686 | 0.959531882 | 1.0377206 | down | mRNA |
| NM_004942 | DEFB4A | 0.348611947 | 0.834138149 | 1.0740071 | down | mRNA |
| NM_004951 | GPR183 | 0.167028854 | 0.642006233 | 1.4224322 | down | mRNA |
| NM_004953 | EIF4G1 | 0.193273816 | 0.68050113 | 1.0843554 | down | mRNA |
| NM_004955 | SLC29A1 | 0.54863894 | 0.92514258 | 1.1714139 | down | mRNA |
| NM_004956 | ETV1 | 0.34393511 | 0.831629639 | 1.3998945 | down | mRNA |
| NM_004960 | FUS | 0.288005434 | 0.78431911 | 1.086999 | down | mRNA |
| NM_004964 | HDAC1 | 0.832352481 | 0.986211576 | 1.0278739 | down | mRNA |
| NM_004965 | HMGN1 | 0.962116034 | 0.99793927 | 1.0013495 | down | mRNA |
| NM_004969 | IDE | 0.393874982 | 0.860826199 | 1.0263526 | down | mRNA |
| NM_004978 | KCNC4 | 0.557943513 | 0.926833616 | 1.147999 | down | mRNA |
| NM_004984 | KIF5A | 0.365342385 | 0.844953778 | 1.1180016 | down | mRNA |
| NM_004985 | KRAS | 0.279636723 | 0.77809961 | 1.1237663 | down | mRNA |
| NM_004986 | KTN1 | 0.806664151 | 0.98118927 | 1.0168964 | down | mRNA |
| NM_004988 | MAGEA1 | 0.823629425 | 0.983498765 | 1.0997598 | down | mRNA |
| NM_004990 | MARS | 0.462358701 | 0.89487645 | 1.1914267 | down | mRNA |
| NM_004993 | ATXN3 | 0.467386848 | 0.896646168 | 1.0701059 | down | mRNA |
| NM_004995 | MMP14 | 0.916309738 | 0.995349793 | 1.0127628 | down | mRNA |
| NM_004996 | ABCC1 | 0.883143917 | 0.992766819 | 1.0456184 | down | mRNA |
| NM_004997 | MYBPH | 0.868015158 | 0.989481868 | 1.0423466 | down | mRNA |
| NM_005001 | NDUFA7 | 0.529397257 | 0.91899174 | 1.0326272 | down | mRNA |
| NM_005002 | NDUFA9 | 0.434559872 | 0.880992255 | 1.0408088 | down | mRNA |
| NM_005004 | NDUFB8 | 0.62790549 | 0.949611308 | 1.0283655 | down | mRNA |
| NM_005006 | NDUFS1 | 0.551124438 | 0.926071245 | 1.034094 | down | mRNA |
| NM_005007 | NFKBIL1 | 0.682426714 | 0.960111041 | 1.0794924 | down | mRNA |
| NM_005009 | NME4 | 0.370357043 | 0.846215286 | 1.10886 | down | mRNA |
| NM_005010 | NRCAM | 0.958876054 | 0.99728722 | 1.0053526 | down | mRNA |
| NM_005012 | ROR1 | 0.23032922 | 0.727521159 | 1.2382691 | down | mRNA |
| NM_005016 | PCBP2 | 0.329926098 | 0.821768501 | 1.1511703 | down | mRNA |
| NM_005020 | PDE1C | 0.849019321 | 0.987678166 | 1.021972 | down | mRNA |
| NM_005022 | PFN1 | 0.297386984 | 0.793891363 | 1.0866492 | down | mRNA |
| NM_005027 | PIK3R2 | 0.166891174 | 0.641842579 | 1.3242869 | down | mRNA |
| NM_005028 | PIP4K2A | 0.222954313 | 0.71761506 | 1.1130583 | down | mRNA |
| NM_005030 | PLK1 | 0.424113119 | 0.874798629 | 1.3784004 | down | mRNA |
| NM_005033 | EXOSC9 | 0.361281151 | 0.842165256 | 1.1586078 | down | mRNA |
| NM_005037 | PPARG | 0.663138287 | 0.958354006 | 1.1997738 | down | mRNA |
| NM_005038 | PPID | 0.269751635 | 0.769983186 | 1.1123406 | down | mRNA |
| NM_005040 | PRCP | 0.769751028 | 0.975286259 | 1.0475565 | down | mRNA |
| NM_005047 | PSMD5 | 0.002628609 | 0.122061602 | 1.3444915 | down | mRNA |
| NM_005049 | PWP2 | 0.04382255 | 0.385351177 | 1.2479614 | down | mRNA |
| NM_005051 | QARS | 0.354997164 | 0.837818031 | 1.1635594 | down | mRNA |
| NM_005053 | RAD23A | 0.007285145 | 0.190890337 | 1.1334591 | down | mRNA |
| NM_005054 | RGPD5 | 0.092743217 | 0.511370653 | 1.3629941 | down | mRNA |
| NM_005057 | RBBP5 | 0.284831539 | 0.781963593 | 1.2302871 | down | mRNA |
| NM_005065 | SEL1L | 0.746181585 | 0.970394407 | 1.0492933 | down | mRNA |
| NM_005069 | SIM2 | 0.522550719 | 0.917200998 | 1.1208032 | down | mRNA |
| NM_005072 | SLC12A4 | 0.793189682 | 0.979003983 | 1.0193124 | down | mRNA |
| NM_005085 | NUP214 | 0.075410491 | 0.471913234 | 1.2019999 | down | mRNA |
| NM_005088 | AKAP17A | 0.424147397 | 0.874798629 | 1.1224041 | down | mRNA |
| NM_005094 | SLC27A4 | 0.048117169 | 0.398338064 | 1.2849853 | down | mRNA |
| NM_005102 | FEZ2 | 0.917917513 | 0.995349793 | 1.0107903 | down | mRNA |
| NM_005107 | EXOG | 0.0441641 | 0.386336269 | 1.2882456 | down | mRNA |
| NM_005109 | OXSR1 | 0.147519383 | 0.609998801 | 1.1309725 | down | mRNA |
| NM_005112 | WDR1 | 0.439134757 | 0.882895421 | 1.1299172 | down | mRNA |
| NM_005113 | GOLGA5 | 0.024001885 | 0.303389731 | 1.2063101 | down | mRNA |
| NM_005117 | FGF19 | 0.46859024 | 0.89739427 | 1.0603882 | down | mRNA |
| NM_005124 | NUP153 | 0.487341253 | 0.903736579 | 1.0639063 | down | mRNA |
| NM_005125 | CCS | 0.429194135 | 0.877844001 | 1.2110763 | down | mRNA |
| NM_005131 | THOC1 | 0.161909341 | 0.633838016 | 1.2347779 | down | mRNA |
| NM_005134 | PPP4R1 | 0.017381387 | 0.265513422 | 1.470645 | down | mRNA |
| NM_005135 | SLC12A6 | 0.154775431 | 0.622213869 | 1.2508889 | down | mRNA |
| NM_005146 | SART1 | 0.032455554 | 0.343869895 | 1.2518301 | down | mRNA |
| NM_005147 | DNAJA3 | 0.040045081 | 0.371915487 | 1.3395717 | down | mRNA |
| NM_005148 | UNC119 | 0.163059709 | 0.635317534 | 1.121952 | down | mRNA |
| NM_005151 | USP14 | 0.624340747 | 0.948341211 | 1.0341488 | down | mRNA |
| NM_005156 | PTBP3 | 0.441271721 | 0.883920737 | 1.0435924 | down | mRNA |
| NM_005158 | ABL2 | 0.45710308 | 0.89166457 | 1.1333569 | down | mRNA |
| NM_005159 | ACTC1 | 0.671375895 | 0.959531882 | 1.0686343 | down | mRNA |
| NM_005161 | APLNR | 0.08875141 | 0.503205148 | 1.4003916 | down | mRNA |
| NM_005165 | ALDOC | 0.044408805 | 0.387186892 | 1.1432982 | down | mRNA |
| NM_005168 | RND3 | 0.858806681 | 0.988512246 | 1.0117135 | down | mRNA |
| NM_005170 | ASCL2 | 0.402067685 | 0.864786348 | 1.1162305 | down | mRNA |
| NM_005171 | ATF1 | 0.104639673 | 0.535611239 | 1.1609741 | down | mRNA |
| NM_005173 | ATP2A3 | 0.533592543 | 0.92058057 | 1.1408077 | down | mRNA |
| NM_005174 | ATP5C1 | 0.241183586 | 0.73945497 | 1.0781904 | down | mRNA |
| NM_005180 | BMI1 | 0.017106527 | 0.262894868 | 1.3019006 | down | mRNA |
| NM_005184 | CALM3 | 0.463988943 | 0.894989844 | 1.0713248 | down | mRNA |
| NM_005188 | CBL | 0.008152773 | 0.196966143 | 1.2748643 | down | mRNA |
| NM_005189 | CBX2 | 0.632347641 | 0.950953568 | 1.0352304 | down | mRNA |
| NM_005190 | CCNC | 0.049105754 | 0.402023817 | 1.0731715 | down | mRNA |
| NM_005192 | CDKN3 | 0.784157363 | 0.977347603 | 1.0989504 | down | mRNA |
| NM_005197 | FOXN3 | 0.265663332 | 0.766537754 | 1.0775473 | down | mRNA |
| NM_005198 | CHKB | 0.909577008 | 0.995342392 | 1.0108594 | down | mRNA |
| NM_005201 | CCR8 | 0.635207975 | 0.95145488 | 1.1393398 | down | mRNA |
| NM_005202 | COL8A2 | 0.19874311 | 0.687087939 | 1.1120035 | down | mRNA |
| NM_005206 | CRK | 0.067307529 | 0.451808679 | 1.2947833 | down | mRNA |
| NM_005216 | DDOST | 0.271899004 | 0.771886421 | 1.1366419 | down | mRNA |
| NM_005217 | DEFA3 | 0.506866919 | 0.911517619 | 1.0903979 | down | mRNA |
| NM_005223 | DNASE1 | 0.644197994 | 0.954657712 | 1.1054879 | down | mRNA |
| NM_005227 | EFNA4 | 0.014209138 | 0.240149793 | 1.3295592 | down | mRNA |
| NM_005236 | ERCC4 | 0.574938737 | 0.933656777 | 1.0894086 | down | mRNA |
| NM_005242 | F2RL1 | 0.932396683 | 0.996710991 | 1.0185395 | down | mRNA |
| NM_005243 | EWSR1 | 0.588433026 | 0.93579682 | 1.0953132 | down | mRNA |
| NM_005245 | FAT1 | 0.680057037 | 0.960060427 | 1.0942697 | down | mRNA |
| NM_005246 | FER | 0.703379813 | 0.963625105 | 1.0298896 | down | mRNA |
| NM_005247 | FGF3 | 0.146523823 | 0.608122555 | 1.3804607 | down | mRNA |
| NM_005253 | FOSL2 | 0.647455948 | 0.95550188 | 1.0505675 | down | mRNA |
| NM_005269 | GLI1 | 0.582563742 | 0.934005566 | 1.0525577 | down | mRNA |
| NM_005270 | GLI2 | 0.160300984 | 0.631980676 | 1.2566603 | down | mRNA |
| NM_005271 | GLUD1 | 0.019975864 | 0.283521851 | 1.3075853 | down | mRNA |
| NM_005273 | GNB2 | 0.858139176 | 0.988512246 | 1.0214739 | down | mRNA |
| NM_005276 | GPD1 | 0.051820329 | 0.41031217 | 1.1374245 | down | mRNA |
| NM_005278 | GPM6B | 0.052757784 | 0.413038171 | 1.1588396 | down | mRNA |
| NM_005281 | GPR3 | 0.766228061 | 0.974737263 | 1.0733983 | down | mRNA |
| NM_005292 | GPR18 | 0.108014825 | 0.544140502 | 1.8771262 | down | mRNA |
| NM_005294 | GPR21 | 0.078628395 | 0.480619753 | 1.114064 | down | mRNA |
| NM_005295 | GPR22 | 0.14491409 | 0.606253761 | 1.2571089 | down | mRNA |
| NM_005301 | GPR35 | 0.880713688 | 0.992287164 | 1.0242492 | down | mRNA |
| NM_005302 | GPR37 | 0.51088261 | 0.912444239 | 1.0995081 | down | mRNA |
| NM_005308 | GRK5 | 0.228823014 | 0.725125586 | 1.2068258 | down | mRNA |
| NM_005313 | PDIA3 | 0.130911732 | 0.585829729 | 1.1362826 | down | mRNA |
| NM_005318 | H1F0 | 0.682733563 | 0.960111041 | 1.0431076 | down | mRNA |
| NM_005326 | HAGH | 0.663196885 | 0.958354006 | 1.0530594 | down | mRNA |
| NM_005328 | HAS2 | 0.01030226 | 0.209736088 | 1.7405646 | down | mRNA |
| NM_005330 | HBE1 | 0.837166978 | 0.986775289 | 1.057004 | down | mRNA |
| NM_005331 | HBQ1 | 0.565057526 | 0.929921137 | 1.0736163 | down | mRNA |
| NM_005334 | HCFC1 | 0.726040741 | 0.966191059 | 1.0379898 | down | mRNA |
| NM_005339 | UBE2K | 0.655623158 | 0.957222998 | 1.0233702 | down | mRNA |
| NM_005340 | HINT1 | 0.232135522 | 0.730050958 | 1.0654996 | down | mRNA |
| NM_005341 | ZBTB48 | 0.050854071 | 0.407751844 | 1.5824469 | down | mRNA |
| NM_005342 | HMGB3 | 0.751029385 | 0.970725371 | 1.0602095 | down | mRNA |
| NM_005343 | HRAS | 0.288179693 | 0.784355942 | 1.0858957 | down | mRNA |
| NM_005347 | HSPA5 | 0.136805607 | 0.595718028 | 1.2695588 | down | mRNA |
| NM_005349 | RBPJ | 0.769251423 | 0.975270932 | 1.0228709 | down | mRNA |
| NM_005358 | LMO7 | 0.010456975 | 0.209736088 | 2.2358686 | down | mRNA |
| NM_005359 | SMAD4 | 0.022858265 | 0.297919387 | 1.2337665 | down | mRNA |
| NM_005368 | MB | 0.847805826 | 0.987678166 | 1.0306445 | down | mRNA |
| NM_005370 | RAB8A | 0.201503872 | 0.689610625 | 1.1396396 | down | mRNA |
| NM_005373 | MPL | 0.042294749 | 0.380166594 | 1.0648174 | down | mRNA |
| NM_005374 | MPP2 | 0.133273181 | 0.589162633 | 1.1008021 | down | mRNA |
| NM_005376 | MYCL1 | 0.781840392 | 0.976963215 | 1.0949474 | down | mRNA |
| NM_005381 | NCL | 0.374920297 | 0.849710156 | 1.2526358 | down | mRNA |
| NM_005382 | NEFM | 0.07833896 | 0.48040541 | 1.2455357 | down | mRNA |
| NM_005384 | NFIL3 | 0.497461463 | 0.908789022 | 1.1487475 | down | mRNA |
| NM_005385 | NKTR | 0.029217707 | 0.332617253 | 1.4367747 | down | mRNA |
| NM_005387 | NUP98 | 0.91312661 | 0.995342392 | 1.0124138 | down | mRNA |
| NM_005388 | PDCL | 0.017095944 | 0.262894868 | 1.2016591 | down | mRNA |
| NM_005389 | PCMT1 | 0.727310018 | 0.966191059 | 1.0572225 | down | mRNA |
| NM_005392 | PHF2 | 0.173498353 | 0.65174838 | 1.1435478 | down | mRNA |
| NM_005397 | PODXL | 0.01846429 | 0.271518921 | 1.5375077 | down | mRNA |
| NM_005398 | PPP1R3C | 0.08743518 | 0.500769598 | 1.6023747 | down | mRNA |
| NM_005399 | PRKAB2 | 0.113183059 | 0.55632728 | 1.1898441 | down | mRNA |
| NM_005400 | PRKCE | 0.12716224 | 0.580481066 | 1.1947452 | down | mRNA |
| NM_005401 | PTPN14 | 0.351948273 | 0.835686762 | 1.1171532 | down | mRNA |
| NM_005402 | RALA | 0.403335306 | 0.864786348 | 1.0463869 | down | mRNA |
| NM_005406 | ROCK1 | 0.054067281 | 0.417190119 | 1.3623029 | down | mRNA |
| NM_005412 | SHMT2 | 0.370728673 | 0.846330273 | 1.0793009 | down | mRNA |
| NM_005413 | SIX3 | 0.075148579 | 0.470853901 | 1.2347909 | down | mRNA |
| NM_005414 | SKIL | 0.172419072 | 0.6499464 | 1.1666445 | down | mRNA |
| NM_005415 | SLC20A1 | 0.003223092 | 0.133466208 | 1.6479785 | down | mRNA |
| NM_005417 | SRC | 0.652439526 | 0.95661646 | 1.1000649 | down | mRNA |
| NM_005423 | TFF2 | 0.946669434 | 0.996710991 | 1.005411 | down | mRNA |
| NM_005424 | TIE1 | 0.650307772 | 0.955894361 | 1.0258447 | down | mRNA |
| NM_005428 | VAV1 | 0.706592025 | 0.965401045 | 1.1417991 | down | mRNA |
| NM_005429 | VEGFC | 0.835592999 | 0.986575022 | 1.0502519 | down | mRNA |
| NM_005430 | WNT1 | 0.660289447 | 0.958354006 | 1.0995684 | down | mRNA |
| NM_005436 | CCDC6 | 0.746791577 | 0.970432947 | 1.085879 | down | mRNA |
| NM_005439 | MLF2 | 0.300376403 | 0.795797471 | 1.0648835 | down | mRNA |
| NM_005441 | CHAF1B | 0.744218957 | 0.970330526 | 1.1172706 | down | mRNA |
| NM_005443 | PAPSS1 | 0.287392184 | 0.783383547 | 1.1488019 | down | mRNA |
| NM_005445 | SMC3 | 0.249095365 | 0.747319103 | 1.1315389 | down | mRNA |
| NM_005452 | WDR46 | 0.939895772 | 0.996710991 | 1.010612 | down | mRNA |
| NM_005453 | ZBTB22 | 0.613863646 | 0.944827645 | 1.0802035 | down | mRNA |
| NM_005462 | MAGEC1 | 0.822191558 | 0.983107122 | 1.0184508 | down | mRNA |
| NM_005465 | AKT3 | 0.063904933 | 0.441793638 | 1.1450308 | down | mRNA |
| NM_005466 | MED6 | 0.579360419 | 0.933821303 | 1.0549124 | down | mRNA |
| NM_005469 | ACOT8 | 0.840776519 | 0.987068842 | 1.0141776 | down | mRNA |
| NM_005471 | GNPDA1 | 0.293071039 | 0.790364658 | 1.1287995 | down | mRNA |
| NM_005476 | GNE | 0.011115436 | 0.216267027 | 1.1975267 | down | mRNA |
| NM_005480 | TROAP | 0.316004569 | 0.810623882 | 1.1880754 | down | mRNA |
| NM_005481 | MED16 | 0.069571185 | 0.457632202 | 1.1595615 | down | mRNA |
| NM_005482 | PIGK | 0.913844395 | 0.995342392 | 1.0293087 | down | mRNA |
| NM_005484 | PARP2 | 0.662181782 | 0.958354006 | 1.1736437 | down | mRNA |
| NM_005486 | TOM1L1 | 0.662971134 | 0.958354006 | 1.0967584 | down | mRNA |
| NM_005489 | SH2D3C | 0.188728183 | 0.674261364 | 1.2126473 | down | mRNA |
| NM_005490 | SH2D3A | 0.907008031 | 0.995145546 | 1.0250807 | down | mRNA |
| NM_005495 | SLC17A4 | 0.627875529 | 0.949611308 | 1.06076 | down | mRNA |
| NM_005499 | UBA2 | 0.433105025 | 0.880677205 | 1.0705103 | down | mRNA |
| NM_005500 | SAE1 | 0.148361778 | 0.611309209 | 1.0740877 | down | mRNA |
| NM_005501 | ITGA3 | 0.563275023 | 0.929041598 | 1.2324191 | down | mRNA |
| NM_005503 | APBA2 | 0.945279436 | 0.996710991 | 1.0189373 | down | mRNA |
| NM_005504 | BCAT1 | 0.679108964 | 0.959969193 | 1.0426542 | down | mRNA |
| NM_005505 | SCARB1 | 0.191816855 | 0.67759806 | 1.2413482 | down | mRNA |
| NM_005506 | SCARB2 | 0.650724663 | 0.955894361 | 1.0722681 | down | mRNA |
| NM_005507 | CFL1 | 0.79860922 | 0.980749101 | 1.0337185 | down | mRNA |
| NM_005508 | CCR4 | 0.208095788 | 0.698793461 | 1.2526719 | down | mRNA |
| NM_005509 | DMXL1 | 0.614345548 | 0.944827645 | 1.0460752 | down | mRNA |
| NM_005510 | DOM3Z | 0.389253348 | 0.858189054 | 1.296504 | down | mRNA |
| NM_005512 | LRRC32 | 0.90047198 | 0.994789506 | 1.0153484 | down | mRNA |
| NM_005515 | MNX1 | 0.20143867 | 0.689610625 | 1.1311403 | down | mRNA |
| NM_005517 | HMGN2 | 0.487098653 | 0.903621334 | 1.0930169 | down | mRNA |
| NM_005524 | HES1 | 0.005887101 | 0.174484737 | 2.0492268 | down | mRNA |
| NM_005530 | IDH3A | 0.451387476 | 0.888854908 | 1.1027102 | down | mRNA |
| NM_005531 | IFI16 | 0.091107783 | 0.509602046 | 1.0999353 | down | mRNA |
| NM_005536 | IMPA1 | 0.266465497 | 0.767064488 | 1.0768555 | down | mRNA |
| NM_005537 | ING1 | 0.396614579 | 0.861995469 | 1.2055801 | down | mRNA |
| NM_005539 | INPP5A | 0.00729582 | 0.190890337 | 1.4810307 | down | mRNA |
| NM_005540 | INPP5B | 0.558615851 | 0.927089828 | 1.2308041 | down | mRNA |
| NM_005545 | ISLR | 0.91131177 | 0.995342392 | 1.0053422 | down | mRNA |
| NM_005546 | ITK | 0.71495421 | 0.966131151 | 1.0268038 | down | mRNA |
| NM_005548 | KARS | 0.624534032 | 0.948341211 | 1.1267858 | down | mRNA |
| NM_005550 | KIFC3 | 0.071149545 | 0.46104346 | 1.2016027 | down | mRNA |
| NM_005552 | KLC1 | 0.380274164 | 0.853407568 | 1.0770829 | down | mRNA |
| NM_005556 | KRT7 | 0.311649964 | 0.806920827 | 1.1489949 | down | mRNA |
| NM_005557 | KRT16 | 0.51742213 | 0.91546111 | 1.7107651 | down | mRNA |
| NM_005558 | LAD1 | 0.563144254 | 0.929041598 | 1.175887 | down | mRNA |
| NM_005565 | LCP2 | 0.226159078 | 0.721636281 | 1.0834922 | down | mRNA |
| NM_005567 | LGALS3BP | 0.762136569 | 0.97339948 | 1.1038902 | down | mRNA |
| NM_005569 | LIMK2 | 0.239057994 | 0.737113607 | 1.2053326 | down | mRNA |
| NM_005570 | LMAN1 | 0.496673581 | 0.908665955 | 1.0786706 | down | mRNA |
| NM_005572 | LMNA | 0.359211915 | 0.841029094 | 1.0885231 | down | mRNA |
| NM_005573 | LMNB1 | 0.892492303 | 0.993978977 | 1.0153332 | down | mRNA |
| NM_005575 | LNPEP | 0.801860597 | 0.98118927 | 1.0802216 | down | mRNA |
| NM_005576 | LOXL1 | 0.577922873 | 0.933821303 | 1.0380692 | down | mRNA |
| NM_005581 | BCAM | 0.069254929 | 0.456727607 | 1.3664879 | down | mRNA |
| NM_005583 | LYL1 | 0.807970256 | 0.98118927 | 1.0226754 | down | mRNA |
| NM_005585 | SMAD6 | 0.610914235 | 0.943638094 | 1.1573466 | down | mRNA |
| NM_005586 | MDFI | 0.526844086 | 0.917975454 | 1.0725698 | down | mRNA |
| NM_005587 | MEF2A | 0.059910291 | 0.43441371 | 1.3077492 | down | mRNA |
| NM_005589 | ALDH6A1 | 0.942946893 | 0.996710991 | 1.020967 | down | mRNA |
| NM_005596 | NFIB | 0.776670173 | 0.976577486 | 1.0329939 | down | mRNA |
| NM_005597 | NFIC | 0.139495482 | 0.598648178 | 1.1023007 | down | mRNA |
| NM_005598 | NHLH1 | 0.651422353 | 0.956204521 | 1.1218108 | down | mRNA |
| NM_005602 | CLDN11 | 0.832269628 | 0.986211576 | 1.0874375 | down | mRNA |
| NM_005603 | ATP8B1 | 0.01114822 | 0.216420345 | 1.3784892 | down | mRNA |
| NM_005610 | RBBP4 | 0.763374263 | 0.973567683 | 1.0468047 | down | mRNA |
| NM_005611 | RBL2 | 0.021377896 | 0.288424269 | 1.4759498 | down | mRNA |
| NM_005612 | REST | 0.003251289 | 0.134059627 | 1.536327 | down | mRNA |
| NM_005613 | RGS4 | 0.031868829 | 0.342121122 | 2.3735958 | down | mRNA |
| NM_005618 | DLL1 | 0.734355373 | 0.967777568 | 1.0482437 | down | mRNA |
| NM_005622 | ACSM3 | 0.160793791 | 0.632229619 | 1.1356749 | down | mRNA |
| NM_005624 | CCL25 | 0.363622978 | 0.843238468 | 1.7396226 | down | mRNA |
| NM_005626 | SRSF4 | 0.817779222 | 0.981727075 | 1.0174992 | down | mRNA |
| NM_005627 | SGK1 | 0.000468489 | 0.066148041 | 2.0695246 | down | mRNA |
| NM_005629 | SLC6A8 | 0.033873022 | 0.34941973 | 1.1450055 | down | mRNA |
| NM_005637 | SS18 | 0.647424139 | 0.95550188 | 1.0447607 | down | mRNA |
| NM_005638 | VAMP7 | 0.521179849 | 0.916644975 | 1.0708793 | down | mRNA |
| NM_005641 | TAF6 | 0.833867406 | 0.986475536 | 1.0365416 | down | mRNA |
| NM_005642 | TAF7 | 0.592343833 | 0.937750901 | 1.0727977 | down | mRNA |
| NM_005643 | TAF11 | 0.37274455 | 0.847817673 | 1.1191459 | down | mRNA |
| NM_005644 | TAF12 | 0.31271526 | 0.808086566 | 1.0924821 | down | mRNA |
| NM_005648 | TCEB1 | 0.530788865 | 0.919328665 | 1.0467368 | down | mRNA |
| NM_005649 | ZNF354A | 0.066001497 | 0.449561954 | 1.2135213 | down | mRNA |
| NM_005650 | TCF20 | 0.851590165 | 0.987678166 | 1.049805 | down | mRNA |
| NM_005652 | TERF2 | 0.057116728 | 0.425938435 | 1.3634549 | down | mRNA |
| NM_005653 | TFCP2 | 0.302012277 | 0.797262221 | 1.2107153 | down | mRNA |
| NM_005655 | KLF10 | 0.039742813 | 0.370835651 | 1.1805098 | down | mRNA |
| NM_005657 | TP53BP1 | 0.003698363 | 0.141344215 | 1.3524639 | down | mRNA |
| NM_005662 | VDAC3 | 0.333410504 | 0.823381801 | 1.0739204 | down | mRNA |
| NM_005663 | WHSC2 | 0.651249472 | 0.956204521 | 1.1813253 | down | mRNA |
| NM_005664 | MKRN3 | 0.172387768 | 0.6499464 | 1.271732 | down | mRNA |
| NM_005667 | RNF103 | 0.474558466 | 0.899495615 | 1.0974992 | down | mRNA |
| NM_005671 | UBXN8 | 0.616713369 | 0.945912508 | 1.0535132 | down | mRNA |
| NM_005680 | TAF1B | 0.58714697 | 0.93579682 | 1.0479663 | down | mRNA |
| NM_005681 | TAF1A | 0.02570755 | 0.314812835 | 1.3212903 | down | mRNA |
| NM_005685 | GTF2IRD1 | 0.137616556 | 0.596789644 | 1.6022292 | down | mRNA |
| NM_005689 | ABCB6 | 0.077475337 | 0.478473478 | 1.2316867 | down | mRNA |
| NM_005691 | ABCC9 | 0.580529281 | 0.93393768 | 1.0967631 | down | mRNA |
| NM_005692 | ABCF2 | 0.371177864 | 0.84643207 | 1.1958663 | down | mRNA |
| NM_005698 | SCAMP3 | 0.759861581 | 0.972829524 | 1.0314018 | down | mRNA |
| NM_005701 | SNUPN | 0.021367298 | 0.288424269 | 1.1597276 | down | mRNA |
| NM_005702 | ERAL1 | 0.058741977 | 0.430953947 | 1.1470138 | down | mRNA |
| NM_005704 | PTPRU | 0.547312158 | 0.925055514 | 1.1252073 | down | mRNA |
| NM_005706 | TSSC4 | 0.081385955 | 0.48754839 | 1.0730276 | down | mRNA |
| NM_005708 | GPC6 | 0.812469002 | 0.98118927 | 1.0217373 | down | mRNA |
| NM_005710 | PQBP1 | 0.99813982 | 0.999889342 | 1.0003706 | down | mRNA |
| NM_005715 | UST | 0.994416682 | 0.999872237 | 1.0013445 | down | mRNA |
| NM_005717 | ARPC5 | 0.739193492 | 0.969564098 | 1.0308295 | down | mRNA |
| NM_005718 | ARPC4 | 0.35000924 | 0.834344847 | 1.1191012 | down | mRNA |
| NM_005719 | ARPC3 | 0.130106529 | 0.584037049 | 1.1329509 | down | mRNA |
| NM_005720 | ARPC1B | 0.062760035 | 0.439458294 | 1.1387206 | down | mRNA |
| NM_005721 | ACTR3 | 0.425091782 | 0.874882692 | 1.1451456 | down | mRNA |
| NM_005722 | ACTR2 | 0.545898373 | 0.92424063 | 1.0261019 | down | mRNA |
| NM_005723 | TSPAN5 | 0.492116683 | 0.905904609 | 1.17806 | down | mRNA |
| NM_005724 | TSPAN3 | 0.812127466 | 0.98118927 | 1.0466828 | down | mRNA |
| NM_005725 | TSPAN2 | 0.968949527 | 0.998196842 | 1.0124971 | down | mRNA |
| NM_005726 | TSFM | 0.753524136 | 0.970725371 | 1.0235225 | down | mRNA |
| NM_005731 | ARPC2 | 0.404420091 | 0.865726395 | 1.1359967 | down | mRNA |
| NM_005733 | KIF20A | 0.142081567 | 0.60204132 | 1.4139541 | down | mRNA |
| NM_005734 | HIPK3 | 0.564459843 | 0.929730843 | 1.1675305 | down | mRNA |
| NM_005735 | ACTR1B | 0.852126823 | 0.987678166 | 1.0243069 | down | mRNA |
| NM_005737 | ARL4C | 0.038540169 | 0.366681664 | 1.6259129 | down | mRNA |
| NM_005740 | DNAL4 | 0.172998068 | 0.650904119 | 1.2019971 | down | mRNA |
| NM_005741 | ZNF263 | 0.473419644 | 0.899184677 | 1.3462108 | down | mRNA |
| NM_005742 | PDIA6 | 0.059514478 | 0.432586127 | 1.2757866 | down | mRNA |
| NM_005744 | ARIH1 | 0.301038196 | 0.79596036 | 1.1436381 | down | mRNA |
| NM_005747 | CELA3A | 0.332820389 | 0.82332451 | 1.04795 | down | mRNA |
| NM_005749 | TOB1 | 0.04733216 | 0.396926965 | 1.6439387 | down | mRNA |
| NM_005751 | AKAP9 | 0.355296349 | 0.838369858 | 1.0537617 | down | mRNA |
| NM_005759 | ABI2 | 0.783666461 | 0.977275912 | 1.0589153 | down | mRNA |
| NM_005760 | CEBPZ | 0.255110068 | 0.75588719 | 1.0397048 | down | mRNA |
| NM_005764 | PDZK1IP1 | 0.756469345 | 0.971789861 | 1.0121383 | down | mRNA |
| NM_005765 | ATP6AP2 | 0.810059971 | 0.98118927 | 1.0485008 | down | mRNA |
| NM_005766 | FARP1 | 0.134265071 | 0.590871682 | 1.1655951 | down | mRNA |
| NM_005768 | LPCAT3 | 0.478448824 | 0.901271944 | 1.1311309 | down | mRNA |
| NM_005772 | RCL1 | 0.400320327 | 0.864237287 | 1.0709818 | down | mRNA |
| NM_005773 | ZNF256 | 0.049083382 | 0.402023817 | 1.1467946 | down | mRNA |
| NM_005776 | CNIH | 0.908292025 | 0.995302455 | 1.016157 | down | mRNA |
| NM_005777 | RBM6 | 0.160287945 | 0.631980676 | 1.1683164 | down | mRNA |
| NM_005781 | TNK2 | 0.102778369 | 0.531998421 | 1.299266 | down | mRNA |
| NM_005782 | ALYREF | 0.793025367 | 0.979003983 | 1.050265 | down | mRNA |
| NM_005783 | TXNDC9 | 0.551549473 | 0.926152612 | 1.0485608 | down | mRNA |
| NM_005785 | RNF41 | 0.122108681 | 0.57455499 | 1.1092834 | down | mRNA |
| NM_005787 | ALG3 | 0.264858607 | 0.76613535 | 1.2200916 | down | mRNA |
| NM_005788 | PRMT3 | 0.155856651 | 0.623587735 | 1.3474696 | down | mRNA |
| NM_005789 | PSME3 | 0.743224572 | 0.970330526 | 1.0399612 | down | mRNA |
| NM_005791 | MPHOSPH10 | 0.004942055 | 0.159495026 | 1.3727109 | down | mRNA |
| NM_005792 | MPHOSPH6 | 0.56808751 | 0.931705674 | 1.1098533 | down | mRNA |
| NM_005796 | NUTF2 | 0.614743804 | 0.944887438 | 1.0177705 | down | mRNA |
| NM_005800 | USPL1 | 0.000158569 | 0.044621794 | 1.4900559 | down | mRNA |
| NM_005801 | EIF1 | 0.956100224 | 0.996883425 | 1.0067596 | down | mRNA |
| NM_005802 | TOPORS | 0.488506943 | 0.904099228 | 1.1200075 | down | mRNA |
| NM_005803 | FLOT1 | 0.631518063 | 0.950475493 | 1.1065308 | down | mRNA |
| NM_005804 | DDX39A | 0.526626468 | 0.917975454 | 1.1506319 | down | mRNA |
| NM_005805 | PSMD14 | 0.705073052 | 0.964577129 | 1.0325884 | down | mRNA |
| NM_005808 | CTDSPL | 0.053155706 | 0.414084985 | 1.3074133 | down | mRNA |
| NM_005810 | KLRG1 | 0.480821984 | 0.901538317 | 1.0901555 | down | mRNA |
| NM_005811 | GDF11 | 0.512501864 | 0.91317044 | 1.0760594 | down | mRNA |
| NM_005813 | PRKD3 | 0.527224053 | 0.918238774 | 1.0516402 | down | mRNA |
| NM_005828 | DCAF7 | 0.464567643 | 0.894989844 | 1.082753 | down | mRNA |
| NM_005833 | RABEPK | 0.03606432 | 0.356485876 | 1.1703967 | down | mRNA |
| NM_005836 | HRSP12 | 0.466282442 | 0.895903857 | 1.0452404 | down | mRNA |
| NM_005837 | POP7 | 0.293128397 | 0.790364658 | 1.201809 | down | mRNA |
| NM_005839 | SRRM1 | 0.275049767 | 0.774827385 | 1.0867533 | down | mRNA |
| NM_005845 | ABCC4 | 0.018028606 | 0.269173581 | 1.2119211 | down | mRNA |
| NM_005848 | DENND4A | 0.877501862 | 0.991306146 | 1.0246945 | down | mRNA |
| NM_005850 | SF3B4 | 0.849212925 | 0.987678166 | 1.0146848 | down | mRNA |
| NM_005853 | IRX5 | 0.037884197 | 0.363330891 | 1.5556339 | down | mRNA |
| NM_005856 | RAMP3 | 0.249779911 | 0.748162221 | 1.1903823 | down | mRNA |
| NM_005857 | ZMPSTE24 | 0.307251663 | 0.802168809 | 1.159774 | down | mRNA |
| NM_005859 | PURA | 0.845908266 | 0.987338076 | 1.0083499 | down | mRNA |
| NM_005861 | STUB1 | 0.064533812 | 0.443826413 | 1.0678182 | down | mRNA |
| NM_005862 | STAG1 | 0.175440289 | 0.653962789 | 1.1133313 | down | mRNA |
| NM_005863 | NET1 | 0.639066191 | 0.953258011 | 1.3002277 | down | mRNA |
| NM_005868 | BET1 | 0.097928449 | 0.520535504 | 1.088039 | down | mRNA |
| NM_005869 | CWC27 | 0.456116963 | 0.891365829 | 1.0599322 | down | mRNA |
| NM_005871 | SMNDC1 | 0.048981965 | 0.401897225 | 1.1662564 | down | mRNA |
| NM_005879 | TRAIP | 0.725751424 | 0.966191059 | 1.0996549 | down | mRNA |
| NM_005880 | DNAJA2 | 0.224166894 | 0.718323293 | 1.0931675 | down | mRNA |
| NM_005881 | BCKDK | 0.031781029 | 0.341957288 | 1.1178759 | down | mRNA |
| NM_005882 | MAEA | 0.129482178 | 0.583907255 | 1.2259026 | down | mRNA |
| NM_005885 | MARCH6 | 0.833058495 | 0.986434891 | 1.0234759 | down | mRNA |
| NM_005888 | SLC25A3 | 0.739013778 | 0.969564098 | 1.0319489 | down | mRNA |
| NM_005896 | IDH1 | 0.540220739 | 0.923577434 | 1.1501651 | down | mRNA |
| NM_005897 | IPP | 0.046778427 | 0.393992951 | 1.1790257 | down | mRNA |
| NM_005898 | CAPRIN1 | 0.248107353 | 0.746351658 | 1.0998093 | down | mRNA |
| NM_005901 | SMAD2 | 0.421766506 | 0.873398499 | 1.1041955 | down | mRNA |
| NM_005903 | SMAD5 | 0.01268074 | 0.229995372 | 1.2140221 | down | mRNA |
| NM_005905 | SMAD9 | 0.615581429 | 0.945575702 | 1.1179988 | down | mRNA |
| NM_005908 | MANBA | 0.747878835 | 0.97069087 | 1.1209723 | down | mRNA |
| NM_005909 | MAP1B | 0.419562458 | 0.872365335 | 1.063615 | down | mRNA |
| NM_005910 | MAPT | 0.026909291 | 0.319244809 | 1.7953657 | down | mRNA |
| NM_005911 | MAT2A | 0.012092175 | 0.222675671 | 1.2744593 | down | mRNA |
| NM_005914 | MCM4 | 0.923923006 | 0.995456159 | 1.0163426 | down | mRNA |
| NM_005915 | MCM6 | 0.735392436 | 0.968017545 | 1.0915206 | down | mRNA |
| NM_005916 | MCM7 | 0.975826026 | 0.998501532 | 1.0140473 | down | mRNA |
| NM_005918 | MDH2 | 0.325821398 | 0.818530154 | 1.0600801 | down | mRNA |
| NM_005919 | MEF2BNB-MEF2B | 0.423083854 | 0.873989281 | 1.0766493 | down | mRNA |
| NM_005924 | MEOX2 | 0.858359843 | 0.988512246 | 1.130261 | down | mRNA |
| NM_005926 | MFAP1 | 0.04517469 | 0.389384436 | 1.192708 | down | mRNA |
| NM_005929 | MFI2 | 0.943039435 | 0.996710991 | 1.0454908 | down | mRNA |
| NM_005930 | CTAGE5 | 0.172179467 | 0.6499464 | 1.227868 | down | mRNA |
| NM_005932 | MIPEP | 0.773996858 | 0.97589756 | 1.0290887 | down | mRNA |
| NM_005933 | MLL | 0.39196033 | 0.859445464 | 1.0794254 | down | mRNA |
| NM_005934 | MLLT1 | 0.474605773 | 0.899495615 | 1.078374 | down | mRNA |
| NM_005935 | AFF1 | 0.044521874 | 0.387743424 | 1.2572841 | down | mRNA |
| NM_005938 | FOXO4 | 0.542967127 | 0.923577434 | 1.0904428 | down | mRNA |
| NM_005944 | CD200 | 0.863955623 | 0.989401142 | 1.0149847 | down | mRNA |
| NM_005955 | MTF1 | 0.061195885 | 0.437893235 | 1.2438957 | down | mRNA |
| NM_005957 | MTHFR | 0.112202752 | 0.554473407 | 1.3748265 | down | mRNA |
| NM_005958 | MTNR1A | 0.427620957 | 0.876992614 | 1.9625741 | down | mRNA |
| NM_005959 | MTNR1B | 0.485365375 | 0.903250293 | 1.6023152 | down | mRNA |
| NM_005961 | MUC6 | 0.256204894 | 0.757240077 | 1.0813715 | down | mRNA |
| NM_005964 | MYH10 | 0.095481259 | 0.516597924 | 1.3530268 | down | mRNA |
| NM_005966 | NAB1 | 0.842759158 | 0.987257859 | 1.0117307 | down | mRNA |
| NM_005968 | HNRNPM | 0.156893682 | 0.625751326 | 1.1341916 | down | mRNA |
| NM_005969 | NAP1L4 | 0.646523868 | 0.95550188 | 1.0564759 | down | mRNA |
| NM_005982 | SIX1 | 0.081602966 | 0.488129303 | 3.354737 | down | mRNA |
| NM_005983 | SKP2 | 0.00036504 | 0.057984307 | 1.3723742 | down | mRNA |
| NM_005984 | SLC25A1 | 0.231150661 | 0.728531274 | 1.1700439 | down | mRNA |
| NM_005990 | STK10 | 0.658248478 | 0.958354006 | 1.1091843 | down | mRNA |
| NM_005992 | TBX1 | 0.11152636 | 0.552792283 | 1.1579168 | down | mRNA |
| NM_005993 | TBCD | 0.06233803 | 0.439381733 | 1.2073924 | down | mRNA |
| NM_005997 | VPS72 | 0.114343276 | 0.558391858 | 1.1316261 | down | mRNA |
| NM_006000 | TUBA4A | 0.726778648 | 0.966191059 | 1.0589654 | down | mRNA |
| NM_006002 | UCHL3 | 0.80740792 | 0.98118927 | 1.0307847 | down | mRNA |
| NM_006004 | UQCRH | 0.339577609 | 0.828051324 | 1.0544367 | down | mRNA |
| NM_006010 | MANF | 0.118662852 | 0.566616513 | 1.1067344 | down | mRNA |
| NM_006011 | ST8SIA2 | 0.777345378 | 0.976938981 | 1.0449503 | down | mRNA |
| NM_006016 | CD164 | 0.221802673 | 0.716734257 | 1.0702036 | down | mRNA |
| NM_006019 | TCIRG1 | 0.881146797 | 0.992492198 | 1.0376907 | down | mRNA |
| NM_006020 | ALKBH1 | 0.015236975 | 0.248235712 | 1.2357342 | down | mRNA |
| NM_006022 | TSC22D1 | 0.674778585 | 0.959531882 | 1.0856901 | down | mRNA |
| NM_006023 | CDC123 | 0.447150704 | 0.886972498 | 1.0755104 | down | mRNA |
| NM_006024 | TAX1BP1 | 0.809144435 | 0.98118927 | 1.0446924 | down | mRNA |
| NM_006025 | ENDOU | 0.68108182 | 0.960111041 | 1.0921796 | down | mRNA |
| NM_006029 | PNMA1 | 0.016888926 | 0.260822801 | 1.2859801 | down | mRNA |
| NM_006034 | TP53I11 | 0.433579795 | 0.880871258 | 1.1498156 | down | mRNA |
| NM_006035 | CDC42BPB | 0.846955947 | 0.987678166 | 1.0108435 | down | mRNA |
| NM_006043 | HS3ST2 | 0.419724073 | 0.872365335 | 1.1403697 | down | mRNA |
| NM_006045 | ATP9A | 0.249300357 | 0.747319103 | 1.096664 | down | mRNA |
| NM_006048 | UBE4B | 0.008432985 | 0.198394583 | 1.2747096 | down | mRNA |
| NM_006049 | SNAPC5 | 0.242304867 | 0.740610726 | 1.1908025 | down | mRNA |
| NM_006051 | APBB3 | 0.147591959 | 0.610174807 | 1.1497335 | down | mRNA |
| NM_006052 | DSCR3 | 0.053607299 | 0.416065637 | 1.1648092 | down | mRNA |
| NM_006053 | TCIRG1 | 0.498119472 | 0.909116151 | 1.1295079 | down | mRNA |
| NM_006054 | RTN3 | 0.772210915 | 0.975427771 | 1.0496308 | down | mRNA |
| NM_006056 | NMUR1 | 0.58196661 | 0.934005566 | 1.1077135 | down | mRNA |
| NM_006060 | IKZF1 | 0.784013383 | 0.977347603 | 1.0410418 | down | mRNA |
| NM_006062 | SMYD5 | 0.038516836 | 0.366631229 | 1.3311992 | down | mRNA |
| NM_006064 | RRAGB | 0.771342676 | 0.975347132 | 1.1552936 | down | mRNA |
| NM_006067 | EMC8 | 0.633050494 | 0.951174475 | 1.0501953 | down | mRNA |
| NM_006076 | AGFG2 | 0.723161611 | 0.966191059 | 1.0311503 | down | mRNA |
| NM_006082 | TUBA1B | 0.675065619 | 0.959531882 | 1.0902409 | down | mRNA |
| NM_006083 | IK | 0.196944078 | 0.685161614 | 1.0683724 | down | mRNA |
| NM_006084 | IRF9 | 0.226230126 | 0.721639289 | 1.2393511 | down | mRNA |
| NM_006088 | TUBB4B | 0.746039273 | 0.970394407 | 1.0519023 | down | mRNA |
| NM_006089 | SCML2 | 0.026450063 | 0.318338323 | 1.1958134 | down | mRNA |
| NM_006092 | NOD1 | 0.10213684 | 0.53053362 | 1.2782073 | down | mRNA |
| NM_006094 | DLC1 | 0.006442025 | 0.18106035 | 1.2726743 | down | mRNA |
| NM_006097 | MYL9 | 0.744616208 | 0.970330526 | 1.0881386 | down | mRNA |
| NM_006099 | PIAS3 | 0.032377871 | 0.343869895 | 1.5020897 | down | mRNA |
| NM_006100 | ST3GAL6 | 0.280739037 | 0.779126326 | 1.6009867 | down | mRNA |
| NM_006105 | RAPGEF3 | 0.695144704 | 0.962214902 | 1.0300415 | down | mRNA |
| NM_006106 | YAP1 | 0.187110102 | 0.671074722 | 1.1200056 | down | mRNA |
| NM_006109 | PRMT5 | 0.060367343 | 0.435553161 | 1.115576 | down | mRNA |
| NM_006111 | ACAA2 | 0.382668395 | 0.853514109 | 1.0768276 | down | mRNA |
| NM_006112 | PPIE | 0.017613767 | 0.267267899 | 1.1569751 | down | mRNA |
| NM_006113 | VAV3 | 0.711995581 | 0.96592341 | 1.050307 | down | mRNA |
| NM_006117 | ECI2 | 0.129951816 | 0.583907255 | 1.0835477 | down | mRNA |
| NM_006118 | HAX1 | 0.369486161 | 0.846085441 | 1.0526091 | down | mRNA |
| NM_006123 | IDS | 0.902363236 | 0.99508468 | 1.0090568 | down | mRNA |
| NM_006134 | TMEM50B | 0.86858222 | 0.989481868 | 1.01289 | down | mRNA |
| NM_006135 | CAPZA1 | 0.646795437 | 0.95550188 | 1.0268908 | down | mRNA |
| NM_006136 | CAPZA2 | 0.405462156 | 0.866165103 | 1.0640686 | down | mRNA |
| NM_006141 | DYNC1LI2 | 0.923167061 | 0.995430245 | 1.0103072 | down | mRNA |
| NM_006154 | NEDD4 | 0.071737355 | 0.462237305 | 1.4734196 | down | mRNA |
| NM_006156 | NEDD8 | 0.836705304 | 0.986725216 | 1.0161319 | down | mRNA |
| NM_006165 | NFRKB | 0.05354292 | 0.416065637 | 1.2071768 | down | mRNA |
| NM_006166 | NFYB | 0.201053047 | 0.689092046 | 1.2765491 | down | mRNA |
| NM_006167 | NKX3-1 | 0.574699464 | 0.933656777 | 1.1608664 | down | mRNA |
| NM_006178 | NSF | 0.338910083 | 0.827491085 | 1.1652401 | down | mRNA |
| NM_006184 | NUCB1 | 0.983867556 | 0.998971807 | 1.0013917 | down | mRNA |
| NM_006185 | NUMA1 | 0.803451758 | 0.98118927 | 1.0354673 | down | mRNA |
| NM_006186 | NR4A2 | 0.281324003 | 0.779126326 | 1.3718343 | down | mRNA |
| NM_006190 | ORC2 | 0.072870094 | 0.464013386 | 1.2600964 | down | mRNA |
| NM_006191 | PA2G4 | 0.62659649 | 0.949203235 | 1.0656103 | down | mRNA |
| NM_006192 | PAX1 | 0.766197211 | 0.974737263 | 1.0294689 | down | mRNA |
| NM_006194 | PAX9 | 0.830940044 | 0.985710869 | 1.0234527 | down | mRNA |
| NM_006195 | PBX3 | 0.172428235 | 0.6499464 | 1.1700363 | down | mRNA |
| NM_006196 | PCBP1 | 0.422027127 | 0.873444381 | 1.0306654 | down | mRNA |
| NM_006197 | PCM1 | 0.100638407 | 0.527482692 | 1.1509972 | down | mRNA |
| NM_006200 | PCSK5 | 0.994462817 | 0.999872237 | 1.006902 | down | mRNA |
| NM_006201 | CDK16 | 0.69271185 | 0.961584652 | 1.1046896 | down | mRNA |
| NM_006204 | PDE6C | 0.134244207 | 0.590871682 | 1.3910789 | down | mRNA |
| NM_006206 | PDGFRA | 0.308980645 | 0.803961878 | 1.1239842 | down | mRNA |
| NM_006209 | ENPP2 | 0.21140619 | 0.702730777 | 1.135391 | down | mRNA |
| NM_006212 | PFKFB2 | 0.072353111 | 0.463255488 | 1.3553915 | down | mRNA |
| NM_006214 | PHYH | 0.55278397 | 0.926337111 | 1.0641545 | down | mRNA |
| NM_006218 | PIK3CA | 0.126872188 | 0.580406758 | 1.2852482 | down | mRNA |
| NM_006221 | PIN1 | 0.838343639 | 0.986897498 | 1.0283111 | down | mRNA |
| NM_006227 | PLTP | 0.802288002 | 0.98118927 | 1.0450104 | down | mRNA |
| NM_006233 | POLR2I | 0.9306981 | 0.996710991 | 1.0090376 | down | mRNA |
| NM_006234 | POLR2J | 0.940524417 | 0.996710991 | 1.0100682 | down | mRNA |
| NM_006237 | POU4F1 | 0.290303145 | 0.786468159 | 1.264213 | down | mRNA |
| NM_006241 | PPP1R2 | 0.550961418 | 0.925960004 | 1.1052658 | down | mRNA |
| NM_006242 | PPP1R3D | 0.001729297 | 0.105670067 | 1.6062385 | down | mRNA |
| NM_006243 | PPP2R5A | 0.13302425 | 0.588930122 | 1.2397016 | down | mRNA |
| NM_006244 | PPP2R5B | 0.106713978 | 0.541344461 | 1.096896 | down | mRNA |
| NM_006245 | PPP2R5D | 0.731743869 | 0.967217713 | 1.0564733 | down | mRNA |
| NM_006246 | PPP2R5E | 0.344887068 | 0.832215034 | 1.0246378 | down | mRNA |
| NM_006247 | PPP5C | 0.515222942 | 0.914319064 | 1.092352 | down | mRNA |
| NM_006251 | PRKAA1 | 0.062746673 | 0.439458294 | 1.2837503 | down | mRNA |
| NM_006256 | PKN2 | 0.577245468 | 0.933656777 | 1.029517 | down | mRNA |
| NM_006258 | PRKG1 | 0.717551477 | 0.966191059 | 1.0153085 | down | mRNA |
| NM_006260 | DNAJC3 | 0.616149037 | 0.945912508 | 1.0417446 | down | mRNA |
| NM_006262 | PRPH | 0.287940676 | 0.784247799 | 1.1986351 | down | mRNA |
| NM_006264 | PTPN13 | 0.173421265 | 0.65174838 | 1.3690713 | down | mRNA |
| NM_006265 | RAD21 | 0.017738187 | 0.267776588 | 1.1095886 | down | mRNA |
| NM_006266 | RALGDS | 0.687106361 | 0.960205549 | 1.0523019 | down | mRNA |
| NM_006268 | DPF2 | 0.479506754 | 0.901538317 | 1.4563993 | down | mRNA |
| NM_006275 | SRSF6 | 0.22292365 | 0.71761506 | 1.1535997 | down | mRNA |
| NM_006279 | ST3GAL3 | 0.389753007 | 0.858647539 | 1.0510223 | down | mRNA |
| NM_006285 | TESK1 | 0.132219439 | 0.587843174 | 1.2666393 | down | mRNA |
| NM_006292 | TSG101 | 0.938507744 | 0.996710991 | 1.0087901 | down | mRNA |
| NM_006294 | UQCRB | 0.611011325 | 0.943644684 | 1.0459127 | down | mRNA |
| NM_006298 | ZNF192 | 0.743202439 | 0.970330526 | 1.0222367 | down | mRNA |
| NM_006299 | ZNF193 | 0.016127583 | 0.255378523 | 1.2221985 | down | mRNA |
| NM_006303 | AIMP2 | 0.79141164 | 0.978710629 | 1.0129833 | down | mRNA |
| NM_006304 | SHFM1 | 0.263918654 | 0.764495522 | 1.0500581 | down | mRNA |
| NM_006306 | SMC1A | 0.92618738 | 0.995699344 | 1.0294654 | down | mRNA |
| NM_006307 | SRPX | 0.944547171 | 0.996710991 | 1.0607489 | down | mRNA |
| NM_006310 | NPEPPS | 0.125486204 | 0.578441772 | 1.2872242 | down | mRNA |
| NM_006311 | NCOR1 | 0.006171489 | 0.178714838 | 1.3243793 | down | mRNA |
| NM_006312 | NCOR2 | 0.015059403 | 0.246131665 | 1.1412401 | down | mRNA |
| NM_006314 | CNKSR1 | 0.814207231 | 0.98120545 | 1.0363327 | down | mRNA |
| NM_006315 | PCGF3 | 0.572541436 | 0.933460721 | 1.0678828 | down | mRNA |
| NM_006319 | CDIPT | 0.32223002 | 0.815372756 | 1.0974445 | down | mRNA |
| NM_006320 | PGRMC2 | 0.009278234 | 0.204533521 | 1.370839 | down | mRNA |
| NM_006321 | ARIH2 | 0.362884545 | 0.843238468 | 1.0962643 | down | mRNA |
| NM_006322 | TUBGCP3 | 0.195541567 | 0.683572955 | 1.3196009 | down | mRNA |
| NM_006323 | SEC24B | 0.178645399 | 0.65979922 | 1.206128 | down | mRNA |
| NM_006324 | CFDP1 | 0.334581501 | 0.82457104 | 1.1197659 | down | mRNA |
| NM_006325 | RAN | 0.809350924 | 0.98118927 | 1.0331087 | down | mRNA |
| NM_006327 | TIMM23 | 0.910764693 | 0.995342392 | 1.007114 | down | mRNA |
| NM_006329 | FBLN5 | 0.376151415 | 0.849862269 | 1.1882731 | down | mRNA |
| NM_006336 | ZER1 | 0.240016644 | 0.73834502 | 1.0919689 | down | mRNA |
| NM_006339 | HMG20B | 0.988670924 | 0.999140676 | 1.0008111 | down | mRNA |
| NM_006340 | BAIAP2 | 0.558209811 | 0.926833616 | 1.0947313 | down | mRNA |
| NM_006342 | TACC3 | 0.672981048 | 0.959531882 | 1.288871 | down | mRNA |
| NM_006345 | SLC30A9 | 0.463719702 | 0.894989844 | 1.073391 | down | mRNA |
| NM_006346 | PIBF1 | 0.070191367 | 0.458429109 | 1.1103778 | down | mRNA |
| NM_006347 | PPIH | 0.769502979 | 0.975286259 | 1.0720891 | down | mRNA |
| NM_006349 | ZNHIT1 | 0.95813097 | 0.996933183 | 1.002976 | down | mRNA |
| NM_006351 | TIMM44 | 0.036241498 | 0.35703653 | 1.2661285 | down | mRNA |
| NM_006353 | HMGN4 | 0.284889129 | 0.781963593 | 1.2422079 | down | mRNA |
| NM_006354 | TADA3 | 0.490710953 | 0.905398715 | 1.0838945 | down | mRNA |
| NM_006355 | TRIM38 | 0.853501891 | 0.987678166 | 1.0221194 | down | mRNA |
| NM_006358 | SLC25A17 | 0.275541848 | 0.775299869 | 1.0955336 | down | mRNA |
| NM_006359 | SLC9A6 | 0.45889671 | 0.892904132 | 1.072245 | down | mRNA |
| NM_006360 | EIF3M | 0.529333065 | 0.91899174 | 1.0521373 | down | mRNA |
| NM_006361 | HOXB13 | 0.459424598 | 0.892904132 | 1.0724974 | down | mRNA |
| NM_006364 | SEC23A | 0.079382851 | 0.482639336 | 1.1956198 | down | mRNA |
| NM_006366 | CAP2 | 0.856303226 | 0.988326362 | 1.0323719 | down | mRNA |
| NM_006368 | CREB3 | 0.508034853 | 0.911674633 | 1.0706696 | down | mRNA |
| NM_006369 | LRRC41 | 0.23079406 | 0.728071705 | 1.1940244 | down | mRNA |
| NM_006370 | VTI1B | 0.595973293 | 0.93896389 | 1.0735843 | down | mRNA |
| NM_006371 | CRTAP | 0.288582458 | 0.784514546 | 1.1539946 | down | mRNA |
| NM_006372 | SYNCRIP | 0.542693136 | 0.923577434 | 1.0428234 | down | mRNA |
| NM_006374 | STK25 | 0.672772649 | 0.959531882 | 1.0185443 | down | mRNA |
| NM_006377 | UNC13B | 0.926548975 | 0.99582096 | 1.0177533 | down | mRNA |
| NM_006378 | SEMA4D | 0.835169046 | 0.986575022 | 1.033694 | down | mRNA |
| NM_006383 | CIB2 | 0.721097042 | 0.966191059 | 1.0265973 | down | mRNA |
| NM_006384 | CIB1 | 0.991033938 | 0.999376923 | 1.0010988 | down | mRNA |
| NM_006385 | ZNF211 | 0.729333325 | 0.966191059 | 1.0487218 | down | mRNA |
| NM_006387 | CHERP | 0.714384842 | 0.966110889 | 1.0563311 | down | mRNA |
| NM_006388 | KAT5 | 0.115049321 | 0.560283303 | 1.238799 | down | mRNA |
| NM_006390 | IPO8 | 0.334155445 | 0.824174306 | 1.1817775 | down | mRNA |
| NM_006391 | IPO7 | 0.670835332 | 0.959531882 | 1.157589 | down | mRNA |
| NM_006392 | NOP56 | 0.226502686 | 0.721826428 | 1.3574186 | down | mRNA |
| NM_006395 | ATG7 | 0.034005213 | 0.34971876 | 1.148004 | down | mRNA |
| NM_006400 | DCTN2 | 0.751751028 | 0.970725371 | 1.0409062 | down | mRNA |
| NM_006401 | ANP32B | 0.648633091 | 0.95550188 | 1.06843 | down | mRNA |
| NM_006405 | TM9SF1 | 0.189770109 | 0.675788971 | 1.1702845 | down | mRNA |
| NM_006406 | PRDX4 | 0.597236866 | 0.939714862 | 1.0288162 | down | mRNA |
| NM_006409 | ARPC1A | 0.171724874 | 0.64946245 | 1.1562448 | down | mRNA |
| NM_006412 | AGPAT2 | 0.024285254 | 0.304521009 | 1.2155487 | down | mRNA |
| NM_006413 | RPP30 | 0.705248906 | 0.964687887 | 1.058062 | down | mRNA |
| NM_006414 | RPP38 | 0.19815197 | 0.686763988 | 1.2531118 | down | mRNA |
| NM_006415 | SPTLC1 | 0.594622305 | 0.938655001 | 1.0708336 | down | mRNA |
| NM_006416 | SLC35A1 | 0.227303577 | 0.72290573 | 1.2478034 | down | mRNA |
| NM_006423 | RABAC1 | 0.259630789 | 0.760307245 | 1.0489366 | down | mRNA |
| NM_006425 | SLU7 | 0.03055472 | 0.338388833 | 1.1757345 | down | mRNA |
| NM_006427 | SIVA1 | 0.843561128 | 0.987338076 | 1.0495096 | down | mRNA |
| NM_006428 | MRPL28 | 0.528446671 | 0.918883792 | 1.0715633 | down | mRNA |
| NM_006429 | CCT7 | 0.299890428 | 0.795797471 | 1.1320737 | down | mRNA |
| NM_006430 | CCT4 | 0.036510155 | 0.358673526 | 1.1052904 | down | mRNA |
| NM_006431 | CCT2 | 0.248325355 | 0.746629174 | 1.0481145 | down | mRNA |
| NM_006437 | PARP4 | 0.055348299 | 0.419636174 | 1.1434651 | down | mRNA |
| NM_006439 | MAB21L2 | 0.046139864 | 0.391970965 | 1.5701401 | down | mRNA |
| NM_006443 | C6orf108 | 0.485288359 | 0.903250293 | 1.0226145 | down | mRNA |
| NM_006453 | TBL3 | 0.461279194 | 0.89460367 | 1.1369446 | down | mRNA |
| NM_006454 | MXD4 | 0.526892266 | 0.917975454 | 1.1164058 | down | mRNA |
| NM_006455 | LEPREL4 | 0.030559521 | 0.338388833 | 1.1732887 | down | mRNA |
| NM_006459 | ERLIN1 | 0.52339343 | 0.917239107 | 1.0346871 | down | mRNA |
| NM_006461 | SPAG5 | 0.697411491 | 0.962410025 | 1.1652542 | down | mRNA |
| NM_006462 | RBCK1 | 0.669347483 | 0.959338293 | 1.0461032 | down | mRNA |
| NM_006466 | POLR3F | 0.00341279 | 0.137676295 | 1.2203929 | down | mRNA |
| NM_006469 | IVNS1ABP | 0.023181128 | 0.299440091 | 1.2994693 | down | mRNA |
| NM_006472 | TXNIP | 0.018521454 | 0.272094076 | 2.3284898 | down | mRNA |
| NM_006473 | TAF6L | 0.114557092 | 0.558689084 | 1.212784 | down | mRNA |
| NM_006474 | PDPN | 0.281088736 | 0.779126326 | 1.1279488 | down | mRNA |
| NM_006475 | POSTN | 0.613123857 | 0.944540087 | 1.3295074 | down | mRNA |
| NM_006479 | RAD51AP1 | 0.809502343 | 0.98118927 | 1.1753945 | down | mRNA |
| NM_006482 | DYRK2 | 0.67697309 | 0.959531882 | 1.0444513 | down | mRNA |
| NM_006484 | DYRK1B | 0.670726319 | 0.959531882 | 1.1296698 | down | mRNA |
| NM_006488 | KHK | 0.989489228 | 0.999140676 | 1.0018326 | down | mRNA |
| NM_006489 | NOVA1 | 0.77320014 | 0.97589756 | 1.0539307 | down | mRNA |
| NM_006492 | ALX3 | 0.618604968 | 0.946956498 | 1.0834496 | down | mRNA |
| NM_006493 | CLN5 | 0.880047431 | 0.992197209 | 1.0234848 | down | mRNA |
| NM_006496 | GNAI3 | 0.088499055 | 0.502587299 | 1.1865295 | down | mRNA |
| NM_006500 | MCAM | 0.375339093 | 0.849862269 | 1.0857724 | down | mRNA |
| NM_006502 | POLH | 0.661583199 | 0.958354006 | 1.025939 | down | mRNA |
| NM_006503 | PSMC4 | 0.746043269 | 0.970394407 | 1.0175064 | down | mRNA |
| NM_006504 | PTPRE | 0.611166378 | 0.943794873 | 1.0472524 | down | mRNA |
| NM_006505 | PVR | 0.397127844 | 0.862344286 | 1.0724413 | down | mRNA |
| NM_006510 | TRIM27 | 0.012319713 | 0.225255756 | 1.2896382 | down | mRNA |
| NM_006513 | SARS | 0.549180707 | 0.925481755 | 1.1805612 | down | mRNA |
| NM_006522 | WNT6 | 0.618803335 | 0.94711754 | 1.1753769 | down | mRNA |
| NM_006524 | ZNF138 | 0.010406008 | 0.209736088 | 1.2121959 | down | mRNA |
| NM_006527 | SLBP | 0.032017724 | 0.342443112 | 1.3535017 | down | mRNA |
| NM_006530 | YEATS4 | 0.270324863 | 0.770199084 | 1.1031677 | down | mRNA |
| NM_006537 | USP3 | 0.947951188 | 0.996710991 | 1.0071086 | down | mRNA |
| NM_006540 | NCOA2 | 0.918041114 | 0.995349793 | 1.0232188 | down | mRNA |
| NM_006541 | GLRX3 | 0.666336709 | 0.958354006 | 1.0349464 | down | mRNA |
| NM_006544 | EXOC5 | 0.114022046 | 0.557835051 | 1.1345099 | down | mRNA |
| NM_006545 | NPRL2 | 0.459306118 | 0.892904132 | 1.0756793 | down | mRNA |
| NM_006548 | IGF2BP2 | 0.565683781 | 0.930390118 | 1.1423111 | down | mRNA |
| NM_006554 | MTX2 | 0.790546286 | 0.978473677 | 1.0387246 | down | mRNA |
| NM_006556 | PMVK | 0.963148626 | 0.99793927 | 1.0020963 | down | mRNA |
| NM_006559 | KHDRBS1 | 0.351723617 | 0.835328183 | 1.0325393 | down | mRNA |
| NM_006565 | CTCF | 0.120331097 | 0.571550658 | 1.2447779 | down | mRNA |
| NM_006568 | CGRRF1 | 0.06149652 | 0.437893235 | 1.251469 | down | mRNA |
| NM_006570 | RRAGA | 0.037795968 | 0.363212377 | 1.1098261 | down | mRNA |
| NM_006572 | GNA13 | 0.025918663 | 0.315604326 | 1.1915772 | down | mRNA |
| NM_006573 | TNFSF13B | 0.875246055 | 0.991094934 | 1.0126741 | down | mRNA |
| NM_006574 | CSPG5 | 0.837512898 | 0.986775289 | 1.0080232 | down | mRNA |
| NM_006575 | MAP4K5 | 0.073029915 | 0.464657997 | 1.1820962 | down | mRNA |
| NM_006579 | EBP | 0.948729391 | 0.996710991 | 1.0127611 | down | mRNA |
| NM_006583 | RRH | 0.86268727 | 0.989401142 | 1.0383198 | down | mRNA |
| NM_006584 | CCT6B | 0.681414689 | 0.960111041 | 1.2701005 | down | mRNA |
| NM_006586 | CNPY3 | 0.704427668 | 0.96395365 | 1.0737791 | down | mRNA |
| NM_006591 | POLD3 | 0.838367774 | 0.986897498 | 1.0691701 | down | mRNA |
| NM_006594 | AP4B1 | 0.000459949 | 0.066148041 | 1.4072065 | down | mRNA |
| NM_006595 | API5 | 0.436647244 | 0.882102748 | 1.0316701 | down | mRNA |
| NM_006597 | HSPA8 | 0.142805428 | 0.602890358 | 1.1185238 | down | mRNA |
| NM_006599 | NFAT5 | 0.803017391 | 0.98118927 | 1.0287027 | down | mRNA |
| NM_006600 | NUDC | 0.328042918 | 0.820939843 | 1.095609 | down | mRNA |
| NM_006601 | PTGES3 | 0.045790341 | 0.391180343 | 1.1446937 | down | mRNA |
| NM_006609 | MAP3K2 | 0.007403103 | 0.190938402 | 1.3868403 | down | mRNA |
| NM_006620 | HBS1L | 0.160036187 | 0.631816652 | 1.0866641 | down | mRNA |
| NM_006621 | AHCYL1 | 0.75976521 | 0.972828657 | 1.0517931 | down | mRNA |
| NM_006623 | PHGDH | 0.670316793 | 0.959531882 | 1.163216 | down | mRNA |
| NM_006624 | ZMYND11 | 0.239375715 | 0.73766795 | 1.1020874 | down | mRNA |
| NM_006625 | SRSF10 | 0.685800054 | 0.960111041 | 1.0721032 | down | mRNA |
| NM_006626 | ZBTB6 | 0.810083561 | 0.98118927 | 1.0765451 | down | mRNA |
| NM_006628 | ARPP19 | 0.263835102 | 0.764495522 | 1.0448376 | down | mRNA |
| NM_006631 | ZNF266 | 0.090533686 | 0.508246703 | 1.1046692 | down | mRNA |
| NM_006638 | RPP40 | 0.106127574 | 0.539446458 | 1.1666626 | down | mRNA |
| NM_006642 | SDCCAG8 | 0.708262974 | 0.965462762 | 1.0296182 | down | mRNA |
| NM_006643 | SDCCAG3 | 0.453820435 | 0.890043126 | 1.1796405 | down | mRNA |
| NM_006649 | UTP14A | 0.155443746 | 0.623056705 | 1.0693293 | down | mRNA |
| NM_006653 | FRS3 | 0.72890715 | 0.966191059 | 1.0436943 | down | mRNA |
| NM_006654 | FRS2 | 0.115467915 | 0.561007186 | 1.2670983 | down | mRNA |
| NM_006658 | PPP1R17 | 0.239481983 | 0.73766795 | 1.1857993 | down | mRNA |
| NM_006660 | CLPX | 0.026670034 | 0.318484367 | 1.4557397 | down | mRNA |
| NM_006667 | PGRMC1 | 0.077209574 | 0.477585962 | 1.2013944 | down | mRNA |
| NM_006668 | CYP46A1 | 0.737267707 | 0.968917782 | 1.1319299 | down | mRNA |
| NM_006682 | FGL2 | 0.989312994 | 0.999140676 | 1.0191405 | down | mRNA |
| NM_006690 | MMP24 | 0.030319469 | 0.337447626 | 1.39311 | down | mRNA |
| NM_006693 | CPSF4 | 0.331016633 | 0.821853462 | 1.0820667 | down | mRNA |
| NM_006694 | JTB | 0.164896634 | 0.638119216 | 1.0873846 | down | mRNA |
| NM_006695 | RUNDC3A | 0.778049681 | 0.976938981 | 1.0354801 | down | mRNA |
| NM_006696 | BRD8 | 0.055332322 | 0.419636174 | 1.4201687 | down | mRNA |
| NM_006699 | MAN1A2 | 0.186011853 | 0.669616322 | 1.1435665 | down | mRNA |
| NM_006701 | TXNL4A | 0.343472672 | 0.831536584 | 1.0965447 | down | mRNA |
| NM_006703 | NUDT3 | 0.029385505 | 0.333925382 | 1.1380979 | down | mRNA |
| NM_006704 | SUGT1 | 0.952545836 | 0.996710991 | 1.008397 | down | mRNA |
| NM_006709 | EHMT2 | 0.693048583 | 0.961584652 | 1.0801547 | down | mRNA |
| NM_006712 | FASTK | 0.775175019 | 0.97589756 | 1.0069884 | down | mRNA |
| NM_006715 | MAN2C1 | 0.16535494 | 0.638919924 | 1.167879 | down | mRNA |
| NM_006717 | SPIN1 | 0.046287451 | 0.392057225 | 1.2384257 | down | mRNA |
| NM_006724 | MAP3K4 | 0.028996156 | 0.331249882 | 2.081478 | down | mRNA |
| NM_006729 | DIAPH2 | 0.099058518 | 0.523009123 | 1.1656726 | down | mRNA |
| NM_006733 | CENPI | 0.66780176 | 0.958354006 | 1.3208052 | down | mRNA |
| NM_006737 | KIR3DL2 | 0.396325535 | 0.861995469 | 1.6680597 | down | mRNA |
| NM_006739 | MCM5 | 0.974869499 | 0.998501532 | 1.0169418 | down | mRNA |
| NM_006741 | PPP1R1A | 0.78491671 | 0.977411899 | 1.0428888 | down | mRNA |
| NM_006743 | RBM3 | 0.954717223 | 0.996834354 | 1.0054145 | down | mRNA |
| NM_006748 | SLA | 0.424057324 | 0.874798629 | 1.1601233 | down | mRNA |
| NM_006749 | SLC20A2 | 0.319679062 | 0.813888641 | 1.1935461 | down | mRNA |
| NM_006750 | SNTB2 | 0.570393811 | 0.932929528 | 1.0568655 | down | mRNA |
| NM_006751 | SSFA2 | 0.220616983 | 0.715799221 | 1.2976942 | down | mRNA |
| NM_006753 | SURF6 | 0.040175111 | 0.371915487 | 1.2765728 | down | mRNA |
| NM_006754 | SYPL1 | 0.381748415 | 0.853514109 | 1.3294229 | down | mRNA |
| NM_006755 | TALDO1 | 0.760756974 | 0.972934381 | 1.0259367 | down | mRNA |
| NM_006756 | TCEA1 | 0.808604189 | 0.98118927 | 1.0376594 | down | mRNA |
| NM_006758 | U2AF1 | 0.044589802 | 0.387767262 | 1.1160657 | down | mRNA |
| NM_006761 | YWHAE | 0.415174404 | 0.870239791 | 1.02604 | down | mRNA |
| NM_006762 | LAPTM5 | 0.916861383 | 0.995349793 | 1.0124559 | down | mRNA |
| NM_006767 | LZTR1 | 0.456527415 | 0.891568474 | 1.0922484 | down | mRNA |
| NM_006768 | BRAP | 0.700929247 | 0.962991468 | 1.039123 | down | mRNA |
| NM_006771 | KRT38 | 0.282197404 | 0.779872438 | 1.4206651 | down | mRNA |
| NM_006772 | SYNGAP1 | 0.419329824 | 0.872365335 | 1.1030523 | down | mRNA |
| NM_006773 | DDX18 | 0.456103137 | 0.891365829 | 1.0606047 | down | mRNA |
| NM_006782 | ZFPL1 | 0.016519157 | 0.256583273 | 1.1996305 | down | mRNA |
| NM_006784 | WDR3 | 0.013743978 | 0.236215185 | 1.3362613 | down | mRNA |
| NM_006785 | MALT1 | 0.067777352 | 0.451819382 | 1.260904 | down | mRNA |
| NM_006788 | RALBP1 | 0.215998088 | 0.710711878 | 1.1787578 | down | mRNA |
| NM_006791 | MORF4L1 | 0.377557578 | 0.851053291 | 1.1204797 | down | mRNA |
| NM_006794 | GPR75 | 0.054111484 | 0.417214521 | 1.5986521 | down | mRNA |
| NM_006796 | AFG3L2 | 0.069655553 | 0.457632202 | 1.3121612 | down | mRNA |
| NM_006800 | MSL3 | 0.333158319 | 0.82335721 | 1.0461154 | down | mRNA |
| NM_006802 | SF3A3 | 0.002927264 | 0.128824971 | 1.2518433 | down | mRNA |
| NM_006804 | STARD3 | 0.189954039 | 0.675909261 | 1.1691762 | down | mRNA |
| NM_006805 | HNRNPA0 | 0.659803079 | 0.958354006 | 1.0443225 | down | mRNA |
| NM_006810 | PDIA5 | 0.19650718 | 0.68496211 | 1.1089252 | down | mRNA |
| NM_006812 | OS9 | 0.395597662 | 0.861590143 | 1.2147306 | down | mRNA |
| NM_006815 | TMED2 | 0.674063167 | 0.959531882 | 1.051213 | down | mRNA |
| NM_006817 | ERP29 | 0.710308319 | 0.965462762 | 1.0396532 | down | mRNA |
| NM_006818 | MLLT11 | 0.282788859 | 0.780627498 | 1.138108 | down | mRNA |
| NM_006819 | STIP1 | 0.05487822 | 0.419520587 | 1.0811929 | down | mRNA |
| NM_006822 | RAB40B | 0.07201193 | 0.462605549 | 1.2235651 | down | mRNA |
| NM_006824 | EBNA1BP2 | 0.091279562 | 0.50968981 | 1.0983714 | down | mRNA |
| NM_006825 | CKAP4 | 0.140981398 | 0.601181581 | 1.1774642 | down | mRNA |
| NM_006826 | YWHAQ | 0.758471488 | 0.972072638 | 1.0293581 | down | mRNA |
| NM_006827 | TMED10 | 0.037307174 | 0.36076463 | 1.1091168 | down | mRNA |
| NM_006828 | ASCC3 | 0.058289931 | 0.429558128 | 1.2454556 | down | mRNA |
| NM_006831 | CLP1 | 0.032433947 | 0.343869895 | 1.1987593 | down | mRNA |
| NM_006832 | FERMT2 | 0.402601337 | 0.864786348 | 1.1601079 | down | mRNA |
| NM_006833 | COPS6 | 0.732535338 | 0.967703318 | 1.032212 | down | mRNA |
| NM_006835 | CCNI | 0.57850678 | 0.933821303 | 1.1143976 | down | mRNA |
| NM_006836 | GCN1L1 | 0.566407964 | 0.930978716 | 1.0307747 | down | mRNA |
| NM_006837 | COPS5 | 0.095435836 | 0.516597924 | 1.1659171 | down | mRNA |
| NM_006838 | METAP2 | 0.649757135 | 0.95550188 | 1.034484 | down | mRNA |
| NM_006840 | LILRB5 | 0.781137561 | 0.976938981 | 1.0285611 | down | mRNA |
| NM_006841 | SLC38A3 | 0.864853319 | 0.989401142 | 1.0154747 | down | mRNA |
| NM_006842 | SF3B2 | 0.020806209 | 0.286620793 | 1.1777934 | down | mRNA |
| NM_006845 | KIF2C | 0.548914997 | 0.925390135 | 1.3716154 | down | mRNA |
| NM_006847 | LILRB4 | 0.677507331 | 0.959531882 | 1.0102133 | down | mRNA |
| NM_006848 | CCDC85B | 0.804458526 | 0.98118927 | 1.0341671 | down | mRNA |
| NM_006849 | PDIA2 | 0.811818835 | 0.98118927 | 1.0251518 | down | mRNA |
| NM_006851 | GLIPR1 | 0.19845918 | 0.686836451 | 1.0602809 | down | mRNA |
| NM_006852 | TLK2 | 0.031551402 | 0.341588447 | 1.1927511 | down | mRNA |
| NM_006854 | KDELR2 | 0.120912146 | 0.572400147 | 1.2069428 | down | mRNA |
| NM_006855 | KDELR3 | 0.464113933 | 0.894989844 | 1.118848 | down | mRNA |
| NM_006856 | ATF7 | 0.826555962 | 0.984436279 | 1.0377189 | down | mRNA |
| NM_006858 | TMED1 | 0.436799853 | 0.882131629 | 1.0676171 | down | mRNA |
| NM_006859 | LIAS | 0.13392272 | 0.590871682 | 1.3408862 | down | mRNA |
| NM_006860 | IFT27 | 0.587704876 | 0.93579682 | 1.0404999 | down | mRNA |
| NM_006863 | LILRA1 | 0.066552648 | 0.450298982 | 1.2049578 | down | mRNA |
| NM_006871 | RIPK3 | 0.896277194 | 0.994194586 | 1.0389305 | down | mRNA |
| NM_006873 | STON1 | 0.754437866 | 0.97108323 | 1.0709262 | down | mRNA |
| NM_006874 | ELF2 | 0.09357142 | 0.512619241 | 1.4272646 | down | mRNA |
| NM_006875 | PIM2 | 0.770059369 | 0.975286259 | 1.0370554 | down | mRNA |
| NM_006876 | B3GNT1 | 0.008383305 | 0.198394583 | 1.1352282 | down | mRNA |
| NM_006885 | ZFHX3 | 0.333276394 | 0.82335721 | 1.068154 | down | mRNA |
| NM_006887 | ZFP36L2 | 0.590200587 | 0.936215326 | 1.0656469 | down | mRNA |
| NM_006891 | CRYGD | 0.915079992 | 0.995349793 | 1.0073602 | down | mRNA |
| NM_006893 | EIF2D | 0.795199742 | 0.979701559 | 1.0539974 | down | mRNA |
| NM_006901 | MYO9A | 0.011621126 | 0.22025265 | 1.2620429 | down | mRNA |
| NM_006904 | PRKDC | 0.60926369 | 0.943053241 | 1.0801208 | down | mRNA |
| NM_006907 | PYCR1 | 0.020970291 | 0.287697675 | 1.0977661 | down | mRNA |
| NM_006910 | RBBP6 | 0.047587323 | 0.397835916 | 1.1679877 | down | mRNA |
| NM_006912 | RIT1 | 0.069961395 | 0.457987595 | 1.2884537 | down | mRNA |
| NM_006915 | RP2 | 0.233998804 | 0.731381183 | 1.1565013 | down | mRNA |
| NM_006924 | SRSF1 | 0.156133401 | 0.624287966 | 1.1073608 | down | mRNA |
| NM_006932 | SMTN | 0.107094195 | 0.54246118 | 1.1379798 | down | mRNA |
| NM_006934 | SLC6A9 | 0.793584768 | 0.97913499 | 1.0809748 | down | mRNA |
| NM_006937 | SUMO2 | 0.66039327 | 0.958354006 | 1.1556397 | down | mRNA |
| NM_006938 | SNRPD1 | 0.449994291 | 0.888279993 | 1.0852191 | down | mRNA |
| NM_006939 | SOS2 | 0.161017847 | 0.632499008 | 1.1466323 | down | mRNA |
| NM_006940 | SOX5 | 0.684674655 | 0.960111041 | 1.0316268 | down | mRNA |
| NM_006943 | SOX12 | 0.488149894 | 0.903757389 | 1.0375688 | down | mRNA |
| NM_006946 | SPTBN2 | 0.621845308 | 0.948047106 | 1.066449 | down | mRNA |
| NM_006947 | SRP72 | 0.85623816 | 0.988315074 | 1.0183795 | down | mRNA |
| NM_006948 | HSPA13 | 0.474206408 | 0.899418411 | 1.0826283 | down | mRNA |
| NM_006950 | SYN1 | 0.768381752 | 0.975142015 | 1.0587605 | down | mRNA |
| NM_006956 | ZNF12 | 0.594028567 | 0.938299318 | 1.0940711 | down | mRNA |
| NM_006963 | ZNF22 | 0.526324624 | 0.917975454 | 1.1078779 | down | mRNA |
| NM_006969 | ZNF28 | 0.003485406 | 0.138138947 | 1.6786521 | down | mRNA |
| NM_006974 | ZNF33A | 0.124662628 | 0.578441772 | 1.1254118 | down | mRNA |
| NM_006978 | RNF113A | 0.000561224 | 0.072220342 | 1.6860547 | down | mRNA |
| NM_006979 | SLC39A7 | 0.631141004 | 0.950410442 | 1.0445223 | down | mRNA |
| NM_006980 | MTERF | 0.086449432 | 0.49936346 | 1.277687 | down | mRNA |
| NM_006985 | NPIP | 0.054932065 | 0.419520587 | 1.1854797 | down | mRNA |
| NM_006986 | MAGED1 | 0.880344363 | 0.992245156 | 1.0201568 | down | mRNA |
| NM_006989 | RASA4 | 0.201357232 | 0.689610625 | 1.1589518 | down | mRNA |
| NM_006990 | WASF2 | 0.511592106 | 0.912662856 | 1.1110965 | down | mRNA |
| NM_006991 | ZNF197 | 0.01639371 | 0.255922225 | 1.5739704 | down | mRNA |
| NM_006992 | LRRC23 | 0.136429257 | 0.595125435 | 1.0557562 | down | mRNA |
| NM_006996 | SLC19A2 | 0.604579249 | 0.94174007 | 1.1182835 | down | mRNA |
| NM_007001 | SLC35D2 | 0.841994572 | 0.987123192 | 1.0425539 | down | mRNA |
| NM_007010 | DDX52 | 0.473420332 | 0.899184677 | 1.0504227 | down | mRNA |
| NM_007011 | ABHD2 | 0.263116473 | 0.763194832 | 1.2806433 | down | mRNA |
| NM_007014 | WWP2 | 0.09882664 | 0.522586017 | 1.1058442 | down | mRNA |
| NM_007019 | UBE2C | 0.83896823 | 0.987032229 | 1.1126827 | down | mRNA |
| NM_007022 | CYB561D2 | 0.204521332 | 0.694609801 | 1.0693939 | down | mRNA |
| NM_007024 | TMEM115 | 0.024400974 | 0.304929683 | 1.1500971 | down | mRNA |
| NM_007032 | TRIOBP | 0.952020161 | 0.996710991 | 1.0061894 | down | mRNA |
| NM_007033 | RER1 | 0.483549317 | 0.903010541 | 1.0765443 | down | mRNA |
| NM_007034 | DNAJB4 | 0.596157272 | 0.939108209 | 1.1438563 | down | mRNA |
| NM_007038 | ADAMTS5 | 0.905201246 | 0.99508468 | 1.0565138 | down | mRNA |
| NM_007039 | PTPN21 | 0.10060355 | 0.527455231 | 1.5714188 | down | mRNA |
| NM_007041 | ATE1 | 0.173572113 | 0.65174838 | 1.1692381 | down | mRNA |
| NM_007043 | KRR1 | 0.451317321 | 0.888854908 | 1.0905334 | down | mRNA |
| NM_007046 | EMILIN1 | 0.141613678 | 0.601858131 | 1.0914316 | down | mRNA |
| NM_007049 | BTN2A1 | 0.798901063 | 0.980813744 | 1.0304794 | down | mRNA |
| NM_007055 | POLR3A | 0.996199219 | 0.999872237 | 1.0005025 | down | mRNA |
| NM_007057 | ZWINT | 0.945380567 | 0.996710991 | 1.0292534 | down | mRNA |
| NM_007062 | PWP1 | 0.680659064 | 0.960111041 | 1.0370432 | down | mRNA |
| NM_007069 | PLA2G16 | 0.837369048 | 0.986775289 | 1.0223974 | down | mRNA |
| NM_007075 | WDR45 | 0.995736207 | 0.999872237 | 1.0010789 | down | mRNA |
| NM_007076 | FICD | 0.005973994 | 0.176289172 | 1.2816307 | down | mRNA |
| NM_007080 | LSM6 | 0.647305016 | 0.95550188 | 1.0161075 | down | mRNA |
| NM_007081 | RABL2B | 0.752263783 | 0.970725371 | 1.0536614 | down | mRNA |
| NM_007082 | RABL2A | 0.281123948 | 0.779126326 | 1.1617189 | down | mRNA |
| NM_007086 | WDHD1 | 0.593937529 | 0.938299318 | 1.0683179 | down | mRNA |
| NM_007099 | ACP1 | 0.335518003 | 0.825278496 | 1.1072774 | down | mRNA |
| NM_007103 | NDUFV1 | 0.88693092 | 0.993077367 | 1.0098176 | down | mRNA |
| NM_007106 | UBL3 | 0.543905111 | 0.923577434 | 1.1941499 | down | mRNA |
| NM_007108 | TCEB2 | 0.997356635 | 0.999889342 | 1.0001296 | down | mRNA |
| NM_007111 | TFDP1 | 0.16935374 | 0.64578024 | 1.1593843 | down | mRNA |
| NM_007112 | THBS3 | 0.261552556 | 0.762395628 | 1.1301127 | down | mRNA |
| NM_007114 | TMF1 | 0.044396602 | 0.387186892 | 1.1075412 | down | mRNA |
| NM_007121 | NR1H2 | 0.967234792 | 0.99793927 | 1.0017957 | down | mRNA |
| NM_007122 | USF1 | 0.043959631 | 0.385691973 | 1.2124809 | down | mRNA |
| NM_007124 | UTRN | 0.391362123 | 0.85910136 | 1.0903461 | down | mRNA |
| NM_007126 | VCP | 0.374003841 | 0.849308269 | 1.0639598 | down | mRNA |
| NM_007127 | VIL1 | 0.314987511 | 0.810161426 | 1.2206499 | down | mRNA |
| NM_007129 | ZIC2 | 0.413152998 | 0.869509111 | 1.120485 | down | mRNA |
| NM_007139 | ZNF92 | 0.045572475 | 0.39096051 | 1.2598001 | down | mRNA |
| NM_007145 | ZNF146 | 0.142100939 | 0.60204132 | 1.2244682 | down | mRNA |
| NM_007146 | VEZF1 | 0.36673792 | 0.845181673 | 1.1963143 | down | mRNA |
| NM_007147 | ZNF175 | 0.011617792 | 0.22025265 | 1.4569674 | down | mRNA |
| NM_007148 | RNF112 | 0.04186596 | 0.378824525 | 1.5692087 | down | mRNA |
| NM_007149 | ZNF184 | 0.002217151 | 0.111580485 | 1.4892957 | down | mRNA |
| NM_007150 | ZNF185 | 0.901489332 | 0.994902084 | 1.010379 | down | mRNA |
| NM_007152 | ZNF195 | 0.844640664 | 0.987338076 | 1.0148173 | down | mRNA |
| NM_007158 | CSDE1 | 0.724898366 | 0.966191059 | 1.0358376 | down | mRNA |
| NM_007165 | SF3A2 | 0.725979715 | 0.966191059 | 1.0479784 | down | mRNA |
| NM_007166 | PICALM | 0.201832844 | 0.690328163 | 1.1349969 | down | mRNA |
| NM_007167 | ZMYM6 | 0.119535939 | 0.56918143 | 1.2227705 | down | mRNA |
| NM_007171 | POMT1 | 0.525855958 | 0.917868103 | 1.0530004 | down | mRNA |
| NM_007172 | NUP50 | 0.217175193 | 0.711339274 | 1.2617469 | down | mRNA |
| NM_007173 | PRSS23 | 0.545400224 | 0.924012779 | 1.0564977 | down | mRNA |
| NM_007176 | C14orf1 | 0.700210098 | 0.962991468 | 1.0443369 | down | mRNA |
| NM_007178 | STRAP | 0.51992446 | 0.916357846 | 1.084621 | down | mRNA |
| NM_007189 | ABCF2 | 0.042123649 | 0.379087707 | 1.1771155 | down | mRNA |
| NM_007190 | SEC23IP | 0.50463242 | 0.910568383 | 1.0429107 | down | mRNA |
| NM_007194 | CHEK2 | 0.940222823 | 0.996710991 | 1.0089667 | down | mRNA |
| NM_007195 | POLI | 0.420272767 | 0.872365335 | 1.142641 | down | mRNA |
| NM_007198 | PROSC | 0.248224764 | 0.74647329 | 1.0652174 | down | mRNA |
| NM_007200 | AKAP13 | 0.868369829 | 0.989481868 | 1.0163409 | down | mRNA |
| NM_007202 | AKAP10 | 0.038831692 | 0.368040971 | 1.4504231 | down | mRNA |
| NM_007204 | DDX20 | 0.034925438 | 0.352338753 | 1.4206694 | down | mRNA |
| NM_007208 | MRPL3 | 0.096696335 | 0.518741393 | 1.1080597 | down | mRNA |
| NM_007211 | RASSF8 | 0.357364273 | 0.839643099 | 1.1359783 | down | mRNA |
| NM_007212 | RNF2 | 0.016932866 | 0.261015185 | 1.1391801 | down | mRNA |
| NM_007214 | SEC63 | 0.287598916 | 0.783841979 | 1.1905718 | down | mRNA |
| NM_007215 | POLG2 | 0.060985588 | 0.437066964 | 1.7986953 | down | mRNA |
| NM_007218 | RNF139 | 0.38008609 | 0.853346994 | 1.2268298 | down | mRNA |
| NM_007219 | RNF24 | 0.470142099 | 0.898057982 | 1.0559824 | down | mRNA |
| NM_007220 | CA5B | 0.322546887 | 0.815372756 | 1.0979276 | down | mRNA |
| NM_007221 | PMF1 | 0.465535544 | 0.895568993 | 1.0617406 | down | mRNA |
| NM_007225 | NXPH3 | 0.508978078 | 0.911674633 | 1.0400334 | down | mRNA |
| NM_007234 | DCTN3 | 0.362247785 | 0.843173209 | 1.0195317 | down | mRNA |
| NM_007235 | XPOT | 0.500721405 | 0.909680616 | 1.1793719 | down | mRNA |
| NM_007236 | CHP1 | 0.854447629 | 0.988044084 | 1.0165536 | down | mRNA |
| NM_007240 | DUSP12 | 0.042714855 | 0.382084661 | 1.2156398 | down | mRNA |
| NM_007241 | SNF8 | 0.830581927 | 0.985549562 | 1.0179178 | down | mRNA |
| NM_007242 | DDX19B | 0.426173173 | 0.875717896 | 1.1385176 | down | mRNA |
| NM_007245 | ATXN2L | 0.258825893 | 0.759358563 | 1.4356689 | down | mRNA |
| NM_007246 | KLHL2 | 0.407075234 | 0.867591329 | 1.1367497 | down | mRNA |
| NM_007247 | SYNRG | 0.787817992 | 0.977536468 | 1.0244783 | down | mRNA |
| NM_007249 | KLF12 | 0.562478699 | 0.929041598 | 1.0884227 | down | mRNA |
| NM_007250 | KLF8 | 0.048094218 | 0.398338064 | 1.158711 | down | mRNA |
| NM_007252 | POU6F2 | 0.132159934 | 0.587843174 | 1.4450715 | down | mRNA |
| NM_007253 | CYP4F8 | 0.936700421 | 0.996710991 | 1.0116893 | down | mRNA |
| NM_007256 | SLCO2B1 | 0.176596656 | 0.65602809 | 1.2772736 | down | mRNA |
| NM_007257 | PNMA2 | 0.868017311 | 0.989481868 | 1.0215841 | down | mRNA |
| NM_007259 | VPS45 | 0.001474784 | 0.098148183 | 1.182593 | down | mRNA |
| NM_007260 | LYPLA2 | 0.422549693 | 0.873896892 | 1.1304045 | down | mRNA |
| NM_007265 | ECD | 0.808390579 | 0.98118927 | 1.0361846 | down | mRNA |
| NM_007269 | STXBP3 | 0.307994474 | 0.802832011 | 1.0600966 | down | mRNA |
| NM_007275 | TUSC2 | 0.853903891 | 0.987855862 | 1.023284 | down | mRNA |
| NM_007276 | CBX3 | 0.446534256 | 0.886701288 | 1.0743825 | down | mRNA |
| NM_007277 | EXOC3 | 0.03475203 | 0.352074963 | 1.2736227 | down | mRNA |
| NM_007278 | GABARAP | 0.722969202 | 0.966191059 | 1.0380551 | down | mRNA |
| NM_007284 | TWF2 | 0.092139702 | 0.511207518 | 1.1105313 | down | mRNA |
| NM_007294 | BRCA1 | 0.637631792 | 0.952805964 | 1.5042972 | down | mRNA |
| NM_007297 | BRCA1 | 0.858297692 | 0.988512246 | 1.0796632 | down | mRNA |
| NM_007299 | BRCA1 | 0.723596101 | 0.966191059 | 1.1081767 | down | mRNA |
| NM_007314 | ABL2 | 0.687388871 | 0.960205549 | 1.0798245 | down | mRNA |
| NM_007315 | STAT1 | 0.74042364 | 0.969794316 | 1.1751415 | down | mRNA |
| NM_007317 | KIF22 | 0.198828791 | 0.687164198 | 1.3111755 | down | mRNA |
| NM_007320 | RANBP3 | 0.827712804 | 0.985006148 | 1.0420639 | down | mRNA |
| NM_007322 | RANBP3 | 0.634018142 | 0.951206671 | 1.0806202 | down | mRNA |
| NM_007324 | ZFYVE9 | 0.181571755 | 0.664924727 | 1.228695 | down | mRNA |
| NM_007329 | DMBT1 | 0.061893049 | 0.438960161 | 1.5545353 | down | mRNA |
| NM_007331 | WHSC1 | 0.195990172 | 0.683863426 | 1.2028796 | down | mRNA |
| NM_007335 | DLEC1 | 0.290291975 | 0.786468159 | 1.0830544 | down | mRNA |
| NM_007342 | NUPL2 | 0.824316435 | 0.983583012 | 1.0403552 | down | mRNA |
| NM_007343 | PRSS3 | 0.216093883 | 0.710711878 | 1.1216523 | down | mRNA |
| NM_007344 | TTF1 | 0.058283595 | 0.429558128 | 1.3510864 | down | mRNA |
| NM_007345 | ZNF236 | 0.256959628 | 0.75762862 | 1.1739681 | down | mRNA |
| NM_007347 | AP4E1 | 0.492773813 | 0.906047853 | 1.1340604 | down | mRNA |
| NM_007348 | ATF6 | 0.555480566 | 0.926833616 | 1.0859382 | down | mRNA |
| NM_007349 | PAXIP1 | 0.008156871 | 0.196966143 | 1.3019131 | down | mRNA |
| NM_007351 | MMRN1 | 0.317496757 | 0.811824899 | 1.7012819 | down | mRNA |
| NM_007357 | COG2 | 0.025668028 | 0.314576454 | 1.39281 | down | mRNA |
| NM_007358 | MTF2 | 0.017540033 | 0.267116092 | 1.528697 | down | mRNA |
| NM_007359 | CASC3 | 0.060463508 | 0.435925442 | 1.2968423 | down | mRNA |
| NM_007362 | NCBP2 | 0.165025054 | 0.638373173 | 1.0409191 | down | mRNA |
| NM_007364 | TMED3 | 0.104261759 | 0.534910443 | 1.0902261 | down | mRNA |
| NM_007366 | PLA2R1 | 0.495961858 | 0.908134987 | 1.1250067 | down | mRNA |
| NM_007367 | RALY | 0.543853614 | 0.923577434 | 1.0483724 | down | mRNA |
| NM_007368 | RASA3 | 0.608058522 | 0.942821299 | 1.0341354 | down | mRNA |
| NM_007371 | BRD3 | 0.154194886 | 0.621674306 | 1.2293416 | down | mRNA |
| NM_007373 | SHOC2 | 0.270077797 | 0.770135843 | 1.2606569 | down | mRNA |
| NM_007375 | TARDBP | 0.191288031 | 0.676939305 | 1.1666202 | down | mRNA |
| NM_009587 | LGALS9 | 0.607037483 | 0.942525113 | 1.1562694 | down | mRNA |
| NM_009588 | LTB | 0.63378978 | 0.951174475 | 1.1917675 | down | mRNA |
| NM_012064 | MIP | 0.730002792 | 0.966191059 | 1.0438537 | down | mRNA |
| NM_012067 | AKR7A3 | 0.548587339 | 0.92514258 | 1.0403536 | down | mRNA |
| NM_012073 | CCT5 | 0.838826533 | 0.987032229 | 1.0155644 | down | mRNA |
| NM_012079 | DGAT1 | 0.767636475 | 0.975033576 | 1.0644943 | down | mRNA |
| NM_012082 | ZFPM2 | 0.410326666 | 0.86807846 | 1.3755736 | down | mRNA |
| NM_012083 | FRAT2 | 0.016512986 | 0.256583273 | 1.8797468 | down | mRNA |
| NM_012084 | GLUD2 | 0.007764511 | 0.19345927 | 1.2510522 | down | mRNA |
| NM_012086 | GTF3C3 | 0.146469566 | 0.608122555 | 1.1950371 | down | mRNA |
| NM_012087 | GTF3C5 | 0.073706989 | 0.467293575 | 1.1179907 | down | mRNA |
| NM_012090 | MACF1 | 0.430976266 | 0.879515151 | 1.1332265 | down | mRNA |
| NM_012091 | ADAT1 | 0.821057042 | 0.982756843 | 1.0156506 | down | mRNA |
| NM_012092 | ICOS | 0.365964164 | 0.845164582 | 1.0942085 | down | mRNA |
| NM_012093 | AK5 | 0.733858112 | 0.967777568 | 1.224689 | down | mRNA |
| NM_012094 | PRDX5 | 0.755156394 | 0.971417904 | 1.0585277 | down | mRNA |
| NM_012095 | AP3M1 | 0.155866263 | 0.623587735 | 1.1511024 | down | mRNA |
| NM_012096 | APPL1 | 0.142605621 | 0.602672517 | 1.2602631 | down | mRNA |
| NM_012097 | ARL5A | 0.075577454 | 0.471986158 | 1.1091656 | down | mRNA |
| NM_012106 | ARL2BP | 0.099758828 | 0.524785637 | 1.0575166 | down | mRNA |
| NM_012109 | TMEM59L | 0.24022409 | 0.738437735 | 1.1982032 | down | mRNA |
| NM_012110 | CHIC2 | 0.52558059 | 0.917693484 | 1.0475384 | down | mRNA |
| NM_012111 | AHSA1 | 0.630430678 | 0.950410442 | 1.0315895 | down | mRNA |
| NM_012112 | TPX2 | 0.587814843 | 0.93579682 | 1.2539058 | down | mRNA |
| NM_012115 | CASP8AP2 | 0.213284058 | 0.7056943 | 1.122529 | down | mRNA |
| NM_012119 | CDK20 | 0.905097618 | 0.99508468 | 1.0098235 | down | mRNA |
| NM_012120 | CD2AP | 0.995240505 | 0.999872237 | 1.0004235 | down | mRNA |
| NM_012123 | MTO1 | 0.067440099 | 0.451808679 | 1.3015786 | down | mRNA |
| NM_012133 | COPG2 | 0.869809302 | 0.989592579 | 1.0209483 | down | mRNA |
| NM_012134 | LMOD1 | 0.412215353 | 0.869144722 | 1.44369 | down | mRNA |
| NM_012137 | DDAH1 | 0.515769922 | 0.914425711 | 1.1077739 | down | mRNA |
| NM_012138 | AATF | 0.109401115 | 0.547509603 | 1.2035986 | down | mRNA |
| NM_012139 | SERGEF | 0.303050349 | 0.798533134 | 1.168218 | down | mRNA |
| NM_012141 | INTS6 | 0.407587124 | 0.867591329 | 1.0707857 | down | mRNA |
| NM_012142 | CCNDBP1 | 0.348767899 | 0.834138149 | 1.0341518 | down | mRNA |
| NM_012145 | DTYMK | 0.92963076 | 0.996533591 | 1.0250389 | down | mRNA |
| NM_012155 | EML2 | 0.663855831 | 0.958354006 | 1.1081284 | down | mRNA |
| NM_012156 | EPB41L1 | 0.367440498 | 0.845181673 | 1.3123715 | down | mRNA |
| NM_012157 | FBXL2 | 0.093536518 | 0.512619241 | 1.3171046 | down | mRNA |
| NM_012158 | FBXL3 | 0.016894729 | 0.260822801 | 1.226996 | down | mRNA |
| NM_012161 | FBXL5 | 0.238110141 | 0.736088702 | 1.3586971 | down | mRNA |
| NM_012162 | FBXL6 | 0.055008384 | 0.419520587 | 1.3924895 | down | mRNA |
| NM_012164 | FBXW2 | 0.037614287 | 0.362248289 | 1.1402172 | down | mRNA |
| NM_012170 | FBXO22 | 0.960260687 | 0.997738713 | 1.0069346 | down | mRNA |
| NM_012171 | TSPAN17 | 0.323641018 | 0.816213518 | 1.1904853 | down | mRNA |
| NM_012173 | FBXO25 | 0.359324932 | 0.841033672 | 1.0797939 | down | mRNA |
| NM_012174 | FBXW8 | 0.533348938 | 0.920530493 | 1.0482928 | down | mRNA |
| NM_012175 | FBXO3 | 0.038884402 | 0.368040971 | 1.2797272 | down | mRNA |
| NM_012176 | FBXO4 | 0.165177617 | 0.638608196 | 1.1690022 | down | mRNA |
| NM_012177 | FBXO5 | 0.385561533 | 0.85515381 | 1.3258156 | down | mRNA |
| NM_012179 | FBXO7 | 0.033161679 | 0.346298535 | 1.1622803 | down | mRNA |
| NM_012180 | FBXO8 | 0.221703649 | 0.716734257 | 1.1291256 | down | mRNA |
| NM_012182 | FOXB1 | 0.440032962 | 0.883367909 | 1.172542 | down | mRNA |
| NM_012186 | FOXE3 | 0.939346345 | 0.996710991 | 1.0114786 | down | mRNA |
| NM_012190 | ALDH1L1 | 0.074287016 | 0.469506874 | 1.3752763 | down | mRNA |
| NM_012194 | KIAA1549L | 0.141252956 | 0.601673229 | 1.6813492 | down | mRNA |
| NM_012197 | RABGAP1 | 0.077153232 | 0.477382686 | 1.1533907 | down | mRNA |
| NM_012201 | GLG1 | 0.853645042 | 0.987737756 | 1.035202 | down | mRNA |
| NM_012203 | GRHPR | 0.411104357 | 0.868434057 | 1.0455806 | down | mRNA |
| NM_012204 | GTF3C4 | 0.002901544 | 0.12797004 | 1.4936078 | down | mRNA |
| NM_012205 | HAAO | 0.515484922 | 0.914319064 | 1.0763619 | down | mRNA |
| NM_012207 | HNRNPH3 | 0.549958269 | 0.925533435 | 1.067144 | down | mRNA |
| NM_012208 | HARS2 | 0.011678186 | 0.220344574 | 1.3070236 | down | mRNA |
| NM_012213 | MLYCD | 0.685579683 | 0.960111041 | 1.0409949 | down | mRNA |
| NM_012218 | ILF3 | 0.346239308 | 0.833163669 | 1.1143397 | down | mRNA |
| NM_012219 | MRAS | 0.544915436 | 0.924012779 | 1.0432783 | down | mRNA |
| NM_012222 | MUTYH | 0.662873522 | 0.958354006 | 1.0513037 | down | mRNA |
| NM_012223 | MYO1B | 0.372060397 | 0.847209316 | 1.1853837 | down | mRNA |
| NM_012225 | NUBP2 | 0.588580417 | 0.93579682 | 1.0857857 | down | mRNA |
| NM_012227 | GTPBP6 | 0.116303527 | 0.562576753 | 1.1669675 | down | mRNA |
| NM_012228 | MSRB2 | 0.353163107 | 0.836266379 | 1.0422134 | down | mRNA |
| NM_012233 | RAB3GAP1 | 0.005635856 | 0.170012215 | 1.2585263 | down | mRNA |
| NM_012236 | SCMH1 | 0.957481585 | 0.996883425 | 1.0051688 | down | mRNA |
| NM_012237 | SIRT2 | 0.922210733 | 0.995349793 | 1.0340412 | down | mRNA |
| NM_012239 | SIRT3 | 0.399929227 | 0.864205663 | 1.2188287 | down | mRNA |
| NM_012241 | SIRT5 | 0.743934618 | 0.970330526 | 1.0722266 | down | mRNA |
| NM_012242 | DKK1 | 0.059407621 | 0.432586127 | 1.8251554 | down | mRNA |
| NM_012243 | SLC35A3 | 0.472417054 | 0.899184677 | 1.0600874 | down | mRNA |
| NM_012247 | SEPHS1 | 0.081956782 | 0.488911764 | 1.2417674 | down | mRNA |
| NM_012248 | SEPHS2 | 0.251952508 | 0.752563302 | 1.2054707 | down | mRNA |
| NM_012254 | SLC27A5 | 0.407268567 | 0.867591329 | 1.2751215 | down | mRNA |
| NM_012260 | HACL1 | 0.41801409 | 0.871927185 | 1.0869982 | down | mRNA |
| NM_012262 | HS2ST1 | 0.235219004 | 0.73249698 | 1.1526958 | down | mRNA |
| NM_012263 | TTLL1 | 0.77814162 | 0.976938981 | 1.0274218 | down | mRNA |
| NM_012268 | PLD3 | 0.497847371 | 0.909116151 | 1.0907224 | down | mRNA |
| NM_012275 | IL36RN | 0.818980296 | 0.982103868 | 1.0362388 | down | mRNA |
| NM_012279 | ZNF346 | 0.81675443 | 0.981692737 | 1.0059915 | down | mRNA |
| NM_012280 | FTSJ1 | 0.349900504 | 0.834214008 | 1.177049 | down | mRNA |
| NM_012287 | ACAP2 | 0.056987606 | 0.425513039 | 1.2216845 | down | mRNA |
| NM_012288 | TRAM2 | 0.403136906 | 0.864786348 | 1.1423676 | down | mRNA |
| NM_012292 | HMHA1 | 0.880829054 | 0.992287164 | 1.0190451 | down | mRNA |
| NM_012293 | PXDN | 0.811331147 | 0.98118927 | 1.017494 | down | mRNA |
| NM_012296 | GAB2 | 0.697787915 | 0.962410025 | 1.10345 | down | mRNA |
| NM_012305 | AP2A2 | 0.243098532 | 0.741476049 | 1.064845 | down | mRNA |
| NM_012308 | KDM2A | 0.072322218 | 0.463255488 | 1.3121415 | down | mRNA |
| NM_012311 | KIN | 0.053644991 | 0.416065637 | 1.1579643 | down | mRNA |
| NM_012316 | KPNA6 | 0.973807863 | 0.998501532 | 1.003201 | down | mRNA |
| NM_012319 | SLC39A6 | 0.464749133 | 0.894989844 | 1.1083381 | down | mRNA |
| NM_012321 | LSM4 | 0.783294867 | 0.977275912 | 1.0335456 | down | mRNA |
| NM_012326 | MAPRE3 | 0.994602782 | 0.999872237 | 1.0006932 | down | mRNA |
| NM_012327 | PIGN | 0.218352644 | 0.712269655 | 1.1831052 | down | mRNA |
| NM_012328 | DNAJB9 | 0.638112831 | 0.952852353 | 1.1567875 | down | mRNA |
| NM_012329 | MMD | 0.950900302 | 0.996710991 | 1.0050531 | down | mRNA |
| NM_012330 | KAT6B | 0.043117977 | 0.383508423 | 1.4692842 | down | mRNA |
| NM_012333 | MYCBP | 0.489584332 | 0.904897251 | 1.1222768 | down | mRNA |
| NM_012336 | NARF | 0.575116068 | 0.933656777 | 1.0559088 | down | mRNA |
| NM_012337 | CCDC19 | 0.780720638 | 0.976938981 | 1.0239662 | down | mRNA |
| NM_012343 | NNT | 0.135500949 | 0.593260206 | 1.2598787 | down | mRNA |
| NM_012345 | NUFIP1 | 0.118076364 | 0.564877324 | 1.0897962 | down | mRNA |
| NM_012347 | FBXO9 | 0.108290653 | 0.544895409 | 2.2109373 | down | mRNA |
| NM_012360 | OR1F1 | 0.059520569 | 0.432586127 | 1.6116145 | down | mRNA |
| NM_012381 | ORC3 | 0.703303418 | 0.963625105 | 1.0638996 | down | mRNA |
| NM_012382 | TTC33 | 0.392423367 | 0.859680391 | 1.0832451 | down | mRNA |
| NM_012383 | OSTF1 | 0.243675824 | 0.742130994 | 1.0580789 | down | mRNA |
| NM_012385 | NUPR1 | 0.703890571 | 0.963625105 | 1.0867763 | down | mRNA |
| NM_012394 | PFDN2 | 0.459202559 | 0.892904132 | 1.0593999 | down | mRNA |
| NM_012396 | PHLDA3 | 0.052033615 | 0.411492592 | 1.1336498 | down | mRNA |
| NM_012399 | PITPNB | 0.382610796 | 0.853514109 | 1.0756144 | down | mRNA |
| NM_012401 | PLXNB2 | 0.208644747 | 0.699796271 | 1.1442652 | down | mRNA |
| NM_012402 | ARFIP2 | 0.317381801 | 0.811824899 | 1.2117196 | down | mRNA |
| NM_012405 | ICMT | 0.569666454 | 0.932573151 | 1.0571972 | down | mRNA |
| NM_012406 | PRDM4 | 0.056558428 | 0.423865077 | 1.4146866 | down | mRNA |
| NM_012408 | ZMYND8 | 0.000618599 | 0.074865247 | 1.8104956 | down | mRNA |
| NM_012411 | PTPN22 | 0.352135852 | 0.835819069 | 1.1327473 | down | mRNA |
| NM_012412 | H2AFV | 0.862297932 | 0.989401142 | 1.0295584 | down | mRNA |
| NM_012413 | QPCT | 0.821521631 | 0.982772998 | 1.045642 | down | mRNA |
| NM_012414 | RAB3GAP2 | 0.086749036 | 0.499950487 | 1.4134646 | down | mRNA |
| NM_012415 | RAD54B | 0.945161738 | 0.996710991 | 1.0340429 | down | mRNA |
| NM_012416 | RANBP6 | 0.036144099 | 0.356565658 | 1.2566198 | down | mRNA |
| NM_012417 | PITPNC1 | 0.981285503 | 0.998971807 | 1.0169731 | down | mRNA |
| NM_012425 | RSU1 | 0.03504085 | 0.352396712 | 1.1082603 | down | mRNA |
| NM_012426 | SF3B3 | 0.446906928 | 0.88691918 | 1.101579 | down | mRNA |
| NM_012428 | NPTN | 0.463181481 | 0.894989844 | 1.1263462 | down | mRNA |
| NM_012430 | SEC22A | 0.096244541 | 0.51782059 | 1.0898846 | down | mRNA |
| NM_012432 | SETDB1 | 0.194042916 | 0.681590151 | 1.2601639 | down | mRNA |
| NM_012433 | SF3B1 | 0.297513332 | 0.794005306 | 1.2008559 | down | mRNA |
| NM_012434 | SLC17A5 | 0.023012275 | 0.298396419 | 1.1492426 | down | mRNA |
| NM_012437 | SNAPIN | 0.579815852 | 0.933893479 | 1.0607429 | down | mRNA |
| NM_012446 | SSBP2 | 0.767977143 | 0.975114674 | 1.0581358 | down | mRNA |
| NM_012450 | SLC13A4 | 0.367682477 | 0.845181673 | 1.1713411 | down | mRNA |
| NM_012453 | TBL2 | 0.110496865 | 0.549968727 | 1.0834068 | down | mRNA |
| NM_012458 | TIMM13 | 0.893712594 | 0.993978977 | 1.0113416 | down | mRNA |
| NM_012460 | TIMM9 | 0.03518462 | 0.352400835 | 1.1913778 | down | mRNA |
| NM_012461 | TINF2 | 0.82196689 | 0.982983098 | 1.0310141 | down | mRNA |
| NM_012463 | ATP6V0A2 | 0.040447534 | 0.372623133 | 1.6619914 | down | mRNA |
| NM_012467 | TPSG1 | 0.779544317 | 0.976938981 | 1.0798166 | down | mRNA |
| NM_012469 | PRPF6 | 0.253201295 | 0.753288068 | 1.1934905 | down | mRNA |
| NM_012470 | TNPO3 | 0.030372257 | 0.337631891 | 1.257555 | down | mRNA |
| NM_012473 | TXN2 | 0.002671684 | 0.122697021 | 1.0792557 | down | mRNA |
| NM_012474 | UCK2 | 0.11125247 | 0.552242484 | 1.1810512 | down | mRNA |
| NM_012476 | VAX2 | 0.145443454 | 0.607094295 | 1.5602428 | down | mRNA |
| NM_012477 | WBP1 | 0.245621363 | 0.743916784 | 1.0621201 | down | mRNA |
| NM_012482 | ZNF281 | 0.133465947 | 0.589791271 | 1.7846461 | down | mRNA |
| NM_012483 | GNLY | 0.318462968 | 0.812422372 | 1.1708421 | down | mRNA |
| NM_012484 | HMMR | 0.390367 | 0.858920947 | 1.338889 | down | mRNA |
| NM_013230 | CD24 | 0.378904297 | 0.852482258 | 1.6193155 | down | mRNA |
| NM_013231 | FLRT2 | 0.792327405 | 0.978996327 | 1.0956626 | down | mRNA |
| NM_013235 | DROSHA | 0.623848152 | 0.948341211 | 1.1898888 | down | mRNA |
| NM_013236 | ATXN10 | 0.645073132 | 0.954828387 | 1.0217867 | down | mRNA |
| NM_013237 | PRELID1 | 0.804287142 | 0.98118927 | 1.0300884 | down | mRNA |
| NM_013239 | PPP2R3B | 0.921307583 | 0.995349793 | 1.0480148 | down | mRNA |
| NM_013240 | N6AMT1 | 0.709372206 | 0.965462762 | 1.0650381 | down | mRNA |
| NM_013242 | C16orf80 | 0.04560533 | 0.39096051 | 1.1992186 | down | mRNA |
| NM_013245 | VPS4A | 0.075114582 | 0.470853901 | 1.1446867 | down | mRNA |
| NM_013246 | CLCF1 | 0.77617309 | 0.976080759 | 1.1082344 | down | mRNA |
| NM_013247 | HTRA2 | 0.349118509 | 0.834138149 | 1.1798611 | down | mRNA |
| NM_013248 | NXT1 | 0.001243143 | 0.092399617 | 1.2784171 | down | mRNA |
| NM_013250 | ZNF215 | 0.024001458 | 0.303389731 | 1.8684875 | down | mRNA |
| NM_013254 | TBK1 | 0.909124876 | 0.995342392 | 1.0130757 | down | mRNA |
| NM_013256 | ZNF180 | 0.972055718 | 0.998501532 | 1.0074575 | down | mRNA |
| NM_013258 | PYCARD | 0.701724016 | 0.963236071 | 1.0258274 | down | mRNA |
| NM_013263 | BRD7 | 0.02176182 | 0.290701669 | 1.1687089 | down | mRNA |
| NM_013265 | VPS51 | 0.774009316 | 0.97589756 | 1.0621426 | down | mRNA |
| NM_013271 | PCSK1N | 0.742946004 | 0.970330526 | 1.0164653 | down | mRNA |
| NM_013274 | POLL | 0.015266348 | 0.248240953 | 1.600157 | down | mRNA |
| NM_013275 | ANKRD11 | 0.007116745 | 0.188654043 | 1.2749491 | down | mRNA |
| NM_013277 | RACGAP1 | 0.370132827 | 0.846215286 | 1.4406061 | down | mRNA |
| NM_013278 | IL17C | 0.816293398 | 0.981643774 | 1.0298883 | down | mRNA |
| NM_013282 | UHRF1 | 0.685083376 | 0.960111041 | 1.0391918 | down | mRNA |
| NM_013283 | MAT2B | 0.469599822 | 0.89787452 | 1.1015122 | down | mRNA |
| NM_013284 | POLM | 0.936354717 | 0.996710991 | 1.0098281 | down | mRNA |
| NM_013285 | GNL2 | 0.08544875 | 0.497360491 | 1.1390852 | down | mRNA |
| NM_013286 | RBM15B | 0.867215294 | 0.989481868 | 1.0226132 | down | mRNA |
| NM_013290 | PSMC3IP | 0.84131813 | 0.987068842 | 1.0913574 | down | mRNA |
| NM_013291 | CPSF1 | 0.078507959 | 0.480536 | 1.0842965 | down | mRNA |
| NM_013292 | MYLPF | 0.574041875 | 0.933656777 | 1.1170769 | down | mRNA |
| NM_013296 | GPSM2 | 2.04881E-05 | 0.018978458 | 1.4729528 | down | mRNA |
| NM_013299 | SAC3D1 | 0.503974186 | 0.910568383 | 1.167972 | down | mRNA |
| NM_013300 | FAM216A | 0.844165406 | 0.987338076 | 1.0283566 | down | mRNA |
| NM_013301 | CCDC106 | 0.762351338 | 0.97339948 | 1.0411052 | down | mRNA |
| NM_013302 | EEF2K | 0.62309981 | 0.948341211 | 1.1355373 | down | mRNA |
| NM_013309 | SLC30A4 | 0.991168388 | 0.999376923 | 1.0009654 | down | mRNA |
| NM_013310 | C2orf27A | 0.718437534 | 0.966191059 | 1.052228 | down | mRNA |
| NM_013316 | CNOT4 | 0.009119902 | 0.203095117 | 1.1844848 | down | mRNA |
| NM_013318 | PRRC2B | 0.410353718 | 0.86807846 | 1.0609914 | down | mRNA |
| NM_013319 | UBIAD1 | 0.271846503 | 0.771845146 | 1.1229645 | down | mRNA |
| NM_013320 | HCFC2 | 0.001774633 | 0.105670067 | 1.3712291 | down | mRNA |
| NM_013325 | ATG4B | 0.228565436 | 0.724651871 | 1.0912992 | down | mRNA |
| NM_013326 | C18orf8 | 0.04952281 | 0.40292028 | 1.0836433 | down | mRNA |
| NM_013328 | PYCR2 | 0.134477294 | 0.591048927 | 1.0972731 | down | mRNA |
| NM_013330 | NME7 | 0.41322745 | 0.869509111 | 1.2280454 | down | mRNA |
| NM_013333 | EPN1 | 0.085748385 | 0.497982338 | 1.445822 | down | mRNA |
| NM_013334 | GMPPB | 0.942952481 | 0.996710991 | 1.0183247 | down | mRNA |
| NM_013337 | TIMM22 | 0.002254293 | 0.112066979 | 1.5429806 | down | mRNA |
| NM_013341 | OLA1 | 0.054652254 | 0.419400623 | 1.1585773 | down | mRNA |
| NM_013345 | GPR132 | 0.631996091 | 0.950683967 | 1.1012623 | down | mRNA |
| NM_013354 | CNOT7 | 0.299014281 | 0.795233272 | 1.1842299 | down | mRNA |
| NM_013355 | PKN3 | 0.906316574 | 0.99508468 | 1.0456514 | down | mRNA |
| NM_013360 | ZNF222 | 0.052747081 | 0.413038171 | 1.3714896 | down | mRNA |
| NM_013363 | PCOLCE2 | 0.330188421 | 0.821769369 | 1.0498961 | down | mRNA |
| NM_013365 | GGA1 | 0.129944548 | 0.583907255 | 1.1605321 | down | mRNA |
| NM_013366 | ANAPC2 | 0.108012414 | 0.544140502 | 1.0992947 | down | mRNA |
| NM_013367 | ANAPC4 | 0.084036695 | 0.493254641 | 1.2173092 | down | mRNA |
| NM_013374 | PDCD6IP | 0.899483714 | 0.994708735 | 1.0047408 | down | mRNA |
| NM_013375 | ABT1 | 0.024659143 | 0.306827745 | 1.3063752 | down | mRNA |
| NM_013376 | SERTAD1 | 0.010582024 | 0.210159643 | 1.3841347 | down | mRNA |
| NM_013385 | CYTH4 | 0.718237414 | 0.966191059 | 1.0661796 | down | mRNA |
| NM_013386 | SLC25A24 | 0.41659481 | 0.870736736 | 1.0398466 | down | mRNA |
| NM_013388 | PREB | 0.042735006 | 0.382096804 | 1.2203985 | down | mRNA |
| NM_013391 | DMGDH | 0.52895069 | 0.918932853 | 1.4069645 | down | mRNA |
| NM_013392 | NRBP1 | 0.061935942 | 0.438960161 | 1.2214691 | down | mRNA |
| NM_013396 | USP25 | 0.756091705 | 0.971612725 | 1.0731053 | down | mRNA |
| NM_013403 | STRN4 | 0.55324625 | 0.926337111 | 1.1137015 | down | mRNA |
| NM_013406 | DHPS | 0.178253818 | 0.659195459 | 1.0420186 | down | mRNA |
| NM_013411 | AK2 | 0.269344131 | 0.769752802 | 1.1124963 | down | mRNA |
| NM_013417 | IARS | 0.411826574 | 0.869001481 | 1.0579358 | down | mRNA |
| NM_013427 | ARHGAP6 | 0.633262248 | 0.951174475 | 1.1387871 | down | mRNA |
| NM_013436 | NCKAP1 | 0.262199544 | 0.763125731 | 1.0948648 | down | mRNA |
| NM_013438 | UBQLN1 | 0.298477052 | 0.795148977 | 1.0743905 | down | mRNA |
| NM_013441 | RCAN3 | 0.486302657 | 0.903524001 | 1.294296 | down | mRNA |
| NM_013443 | ST6GALNAC6 | 0.366208007 | 0.845181673 | 1.1099286 | down | mRNA |
| NM_013450 | BAZ2B | 0.198201549 | 0.686763988 | 1.2486169 | down | mRNA |
| NM_013942 | PAX3 | 0.591476288 | 0.937253206 | 1.0717832 | down | mRNA |
| NM_013943 | CLIC4 | 0.200104098 | 0.68900633 | 1.065652 | down | mRNA |
| NM_013945 | PAX7 | 0.267558919 | 0.768382909 | 1.1637883 | down | mRNA |
| NM_013953 | PAX8 | 0.803417331 | 0.98118927 | 1.0296017 | down | mRNA |
| NM_013974 | DDAH2 | 0.303159138 | 0.798533134 | 1.120582 | down | mRNA |
| NM_013976 | GCDH | 0.107560187 | 0.543737871 | 1.1327292 | down | mRNA |
| NM_013980 | BNIP1 | 0.805236826 | 0.98118927 | 1.031806 | down | mRNA |
| NM_013992 | PAX8 | 0.217926062 | 0.712244444 | 1.2200765 | down | mRNA |
| NM_014001 | GGA3 | 0.23386392 | 0.73118887 | 1.3897613 | down | mRNA |
| NM_014003 | DHX38 | 0.767396634 | 0.975033576 | 1.0274858 | down | mRNA |
| NM_014015 | DEXI | 0.474304751 | 0.899418411 | 1.1230452 | down | mRNA |
| NM_014016 | SACM1L | 0.087320038 | 0.500533133 | 1.2244549 | down | mRNA |
| NM_014023 | WDR37 | 0.491016111 | 0.90553335 | 1.0942194 | down | mRNA |
| NM_014034 | ASF1A | 0.194457481 | 0.682224934 | 1.1459401 | down | mRNA |
| NM_014035 | SNX24 | 0.485123134 | 0.903250293 | 1.0852259 | down | mRNA |
| NM_014039 | C11orf54 | 0.830042606 | 0.985549562 | 1.0148757 | down | mRNA |
| NM_014041 | SPCS1 | 0.854756166 | 0.988178307 | 1.0363948 | down | mRNA |
| NM_014043 | CHMP2B | 0.423919704 | 0.874798629 | 1.0863839 | down | mRNA |
| NM_014044 | UNC50 | 0.267839046 | 0.768732846 | 1.2064468 | down | mRNA |
| NM_014045 | LRP10 | 0.926651567 | 0.99582096 | 1.0078254 | down | mRNA |
| NM_014046 | MRPS18B | 0.171804676 | 0.649522625 | 1.3962183 | down | mRNA |
| NM_014048 | MKL2 | 0.436824191 | 0.882131629 | 1.3723841 | down | mRNA |
| NM_014049 | ACAD9 | 0.305721136 | 0.801330686 | 1.1010041 | down | mRNA |
| NM_014051 | TMEM14A | 0.947201739 | 0.996710991 | 1.0053211 | down | mRNA |
| NM_014059 | RGCC | 0.516260302 | 0.914577368 | 1.6363412 | down | mRNA |
| NM_014060 | MCTS1 | 0.98222718 | 0.998971807 | 1.0032323 | down | mRNA |
| NM_014061 | MAGEH1 | 0.332559021 | 0.822878182 | 1.1039262 | down | mRNA |
| NM_014064 | NTMT1 | 0.60771704 | 0.942627675 | 1.0626207 | down | mRNA |
| NM_014065 | ASTE1 | 0.049944534 | 0.40410612 | 1.4298678 | down | mRNA |
| NM_014066 | COMMD5 | 0.148321983 | 0.611309209 | 1.1265574 | down | mRNA |
| NM_014067 | MACROD1 | 0.665439506 | 0.958354006 | 1.047737 | down | mRNA |
| NM_014071 | NCOA6 | 0.000809772 | 0.083087642 | 1.398228 | down | mRNA |
| NM_014077 | FAM32A | 0.723823697 | 0.966191059 | 1.0264118 | down | mRNA |
| NM_014106 | ZNF770 | 0.148300624 | 0.611309209 | 1.2990574 | down | mRNA |
| NM_014109 | ATAD2 | 0.924842385 | 0.995456159 | 1.0559464 | down | mRNA |
| NM_014117 | C16orf72 | 0.137458175 | 0.596770033 | 1.1394338 | down | mRNA |
| NM_014138 | FAM156A | 0.003648716 | 0.140237595 | 1.4700152 | down | mRNA |
| NM_014140 | SMARCAL1 | 0.059662141 | 0.433232378 | 1.3384362 | down | mRNA |
| NM_014142 | NUDT5 | 0.551087775 | 0.926071245 | 1.0580492 | down | mRNA |
| NM_014145 | TMEM230 | 0.747538497 | 0.970620903 | 1.0297411 | down | mRNA |
| NM_014146 | LAT2 | 0.894096046 | 0.993978977 | 1.0432255 | down | mRNA |
| NM_014149 | WDR91 | 0.675300095 | 0.959531882 | 1.1242161 | down | mRNA |
| NM_014153 | ZC3H7A | 0.141813185 | 0.60204132 | 1.1690365 | down | mRNA |
| NM_014154 | ARMC8 | 0.030828303 | 0.340185047 | 1.2441065 | down | mRNA |
| NM_014155 | ZBTB44 | 0.364403789 | 0.843856246 | 1.1240551 | down | mRNA |
| NM_014157 | CCDC113 | 0.50622696 | 0.911278334 | 1.0757527 | down | mRNA |
| NM_014159 | SETD2 | 0.274208093 | 0.774333188 | 1.0726637 | down | mRNA |
| NM_014160 | MKRN2 | 0.039269534 | 0.369575679 | 1.2998272 | down | mRNA |
| NM_014161 | MRPL18 | 0.700218337 | 0.962991468 | 1.0320843 | down | mRNA |
| NM_014165 | NDUFAF4 | 0.061609745 | 0.437989276 | 1.2834827 | down | mRNA |
| NM_014166 | MED4 | 0.167152407 | 0.642168434 | 1.1036918 | down | mRNA |
| NM_014168 | METTL5 | 0.945793295 | 0.996710991 | 1.0066611 | down | mRNA |
| NM_014169 | CHMP4A | 0.839170536 | 0.987032229 | 1.0242679 | down | mRNA |
| NM_014170 | GTPBP8 | 0.106650347 | 0.541344461 | 1.1692024 | down | mRNA |
| NM_014171 | CRIPT | 0.922944409 | 0.995349793 | 1.0166816 | down | mRNA |
| NM_014175 | MRPL15 | 0.445413066 | 0.885807324 | 1.144385 | down | mRNA |
| NM_014176 | UBE2T | 0.921448302 | 0.995349793 | 1.0646658 | down | mRNA |
| NM_014177 | TIMM21 | 0.122934402 | 0.57525944 | 1.2361125 | down | mRNA |
| NM_014178 | STXBP6 | 0.881410878 | 0.992719893 | 1.0179747 | down | mRNA |
| NM_014180 | MRPL22 | 0.260605058 | 0.761186904 | 1.1001691 | down | mRNA |
| NM_014183 | DYNLRB1 | 0.661028291 | 0.958354006 | 1.043545 | down | mRNA |
| NM_014184 | CNIH4 | 0.883389927 | 0.992766819 | 1.01337 | down | mRNA |
| NM_014186 | COMMD9 | 0.244711933 | 0.743386078 | 1.1610344 | down | mRNA |
| NM_014188 | SSU72 | 0.760755732 | 0.972934381 | 1.0277153 | down | mRNA |
| NM_014206 | C11orf10 | 0.799160644 | 0.980917337 | 1.0259568 | down | mRNA |
| NM_014214 | IMPA2 | 0.807362206 | 0.98118927 | 1.0196821 | down | mRNA |
| NM_014215 | INSRR | 0.174700424 | 0.653303113 | 1.1393201 | down | mRNA |
| NM_014217 | KCNK2 | 0.137623288 | 0.596789644 | 1.255315 | down | mRNA |
| NM_014222 | NDUFA8 | 0.260022189 | 0.760388386 | 1.1068809 | down | mRNA |
| NM_014223 | NFYC | 0.112330578 | 0.554686607 | 1.0587332 | down | mRNA |
| NM_014226 | MOK | 0.438079702 | 0.882682266 | 1.1776254 | down | mRNA |
| NM_014230 | SRP68 | 0.177852075 | 0.658188638 | 1.2201226 | down | mRNA |
| NM_014231 | VAMP1 | 0.985019563 | 0.998971807 | 1.0022083 | down | mRNA |
| NM_014233 | UBTF | 0.138679888 | 0.598141596 | 1.3535133 | down | mRNA |
| NM_014235 | UBL4A | 0.013231766 | 0.234704726 | 1.8728503 | down | mRNA |
| NM_014236 | GNPAT | 0.589006398 | 0.935965462 | 1.0506955 | down | mRNA |
| NM_014239 | EIF2B2 | 0.38988307 | 0.858747978 | 1.1613577 | down | mRNA |
| NM_014241 | PTPLA | 0.885781108 | 0.992981836 | 1.0143402 | down | mRNA |
| NM_014245 | RNF7 | 0.684634418 | 0.960111041 | 1.0610137 | down | mRNA |
| NM_014247 | RAPGEF2 | 0.424108799 | 0.874798629 | 1.0586302 | down | mRNA |
| NM_014248 | RBX1 | 0.895700298 | 0.993978977 | 1.013782 | down | mRNA |
| NM_014251 | SLC25A13 | 0.846677525 | 0.987644719 | 1.0751215 | down | mRNA |
| NM_014252 | SLC25A15 | 0.200858219 | 0.68900633 | 1.1567858 | down | mRNA |
| NM_014253 | ODZ1 | 0.152373276 | 0.618255775 | 1.3089171 | down | mRNA |
| NM_014254 | TMEM5 | 0.011943058 | 0.222368362 | 1.2811358 | down | mRNA |
| NM_014255 | CNPY2 | 0.596918894 | 0.939652752 | 1.0295846 | down | mRNA |
| NM_014263 | YME1L1 | 0.821851302 | 0.982940803 | 1.0095395 | down | mRNA |
| NM_014264 | PLK4 | 0.920426351 | 0.995349793 | 1.0700122 | down | mRNA |
| NM_014265 | ADAM28 | 0.870326654 | 0.989592579 | 1.019008 | down | mRNA |
| NM_014267 | C11orf58 | 0.103554126 | 0.533123263 | 1.1075764 | down | mRNA |
| NM_014268 | MAPRE2 | 0.299318684 | 0.79562655 | 1.3517641 | down | mRNA |
| NM_014276 | RBPJL | 0.801478335 | 0.98118927 | 1.0228994 | down | mRNA |
| NM_014279 | OLFM1 | 0.616951265 | 0.945960598 | 1.1460414 | down | mRNA |
| NM_014280 | DNAJC8 | 0.569537257 | 0.932573151 | 1.0461074 | down | mRNA |
| NM_014282 | HABP4 | 0.86783491 | 0.989481868 | 1.0221921 | down | mRNA |
| NM_014283 | SUCO | 0.698646827 | 0.962701213 | 1.0609895 | down | mRNA |
| NM_014284 | NCDN | 0.409582194 | 0.867822547 | 1.0728123 | down | mRNA |
| NM_014285 | EXOSC2 | 0.776172003 | 0.976080759 | 1.0395262 | down | mRNA |
| NM_014287 | NOMO1 | 0.454040892 | 0.89021788 | 1.1039777 | down | mRNA |
| NM_014288 | ITGB3BP | 0.967243942 | 0.99793927 | 1.015623 | down | mRNA |
| NM_014291 | GCAT | 0.616387119 | 0.945912508 | 1.0207284 | down | mRNA |
| NM_014294 | TRAM1 | 0.612009406 | 0.944027644 | 1.0689001 | down | mRNA |
| NM_014296 | CAPN7 | 0.080589973 | 0.484779682 | 1.3320359 | down | mRNA |
| NM_014298 | QPRT | 0.332496951 | 0.822824733 | 1.2350119 | down | mRNA |
| NM_014300 | SEC11A | 0.743178243 | 0.970330526 | 1.0436741 | down | mRNA |
| NM_014301 | ISCU | 0.879712 | 0.992178089 | 1.0492851 | down | mRNA |
| NM_014306 | C22orf28 | 0.175194501 | 0.653821873 | 1.172176 | down | mRNA |
| NM_014309 | RBFOX2 | 0.01132576 | 0.218270471 | 1.1277787 | down | mRNA |
| NM_014311 | SMUG1 | 0.80002602 | 0.981186955 | 1.0212844 | down | mRNA |
| NM_014313 | TMEM50A | 0.74959933 | 0.970725371 | 1.0400081 | down | mRNA |
| NM_014315 | KLHDC2 | 0.184606928 | 0.668017166 | 1.1074642 | down | mRNA |
| NM_014316 | CARHSP1 | 0.656845459 | 0.957445617 | 1.0362356 | down | mRNA |
| NM_014319 | LEMD3 | 0.00674942 | 0.183552613 | 1.3270821 | down | mRNA |
| NM_014320 | HEBP2 | 0.465327508 | 0.895338213 | 1.0606498 | down | mRNA |
| NM_014322 | OPN3 | 0.74732845 | 0.970534111 | 1.0430872 | down | mRNA |
| NM_014323 | PATZ1 | 0.992433504 | 0.999781402 | 1.0010471 | down | mRNA |
| NM_014324 | AMACR | 0.671348357 | 0.959531882 | 1.1360998 | down | mRNA |
| NM_014326 | DAPK2 | 0.394781739 | 0.861511465 | 1.2551704 | down | mRNA |
| NM_014333 | CADM1 | 0.593176283 | 0.938046215 | 1.1025668 | down | mRNA |
| NM_014335 | EID1 | 0.674837446 | 0.959531882 | 1.0556381 | down | mRNA |
| NM_014338 | PISD | 0.155763 | 0.623419945 | 1.1858384 | down | mRNA |
| NM_014339 | IL17RA | 0.059115355 | 0.43235014 | 1.4027822 | down | mRNA |
| NM_014341 | MTCH1 | 0.035365593 | 0.353699423 | 1.1926296 | down | mRNA |
| NM_014342 | MTCH2 | 0.252560263 | 0.752702237 | 1.0544415 | down | mRNA |
| NM_014347 | ZNF324 | 0.000256555 | 0.052661938 | 1.2823659 | down | mRNA |
| NM_014351 | SULT4A1 | 0.041486068 | 0.377571504 | 1.1327926 | down | mRNA |
| NM_014353 | RAB26 | 0.855566191 | 0.988178307 | 1.0287371 | down | mRNA |
| NM_014366 | GNL3 | 0.396972873 | 0.862344286 | 1.1074347 | down | mRNA |
| NM_014367 | FAM162A | 0.351294577 | 0.835187247 | 1.0292898 | down | mRNA |
| NM_014372 | RNF11 | 0.786798643 | 0.977479172 | 1.0434472 | down | mRNA |
| NM_014374 | REPIN1 | 0.288844442 | 0.784712079 | 1.1455397 | down | mRNA |
| NM_014377 | DNAJC2 | 0.135139752 | 0.592787801 | 1.1703156 | down | mRNA |
| NM_014379 | KCNV1 | 0.553175437 | 0.926337111 | 1.0925008 | down | mRNA |
| NM_014381 | MLH3 | 0.11207213 | 0.554156842 | 1.5933436 | down | mRNA |
| NM_014382 | ATP2C1 | 0.307310601 | 0.802168809 | 1.1208716 | down | mRNA |
| NM_014384 | ACAD8 | 0.000811063 | 0.083087642 | 1.6458685 | down | mRNA |
| NM_014387 | LAT | 0.619229695 | 0.947444797 | 1.0952866 | down | mRNA |
| NM_014388 | DIEXF | 0.068916262 | 0.455528425 | 1.1548459 | down | mRNA |
| NM_014389 | PELP1 | 0.18226941 | 0.665571416 | 1.0928179 | down | mRNA |
| NM_014394 | GHITM | 0.602966409 | 0.941464987 | 1.0237386 | down | mRNA |
| NM_014396 | VPS41 | 0.912376894 | 0.995342392 | 1.0157514 | down | mRNA |
| NM_014399 | TSPAN13 | 0.40008604 | 0.864237287 | 1.0741998 | down | mRNA |
| NM_014402 | UQCRQ | 0.618395981 | 0.946956498 | 1.0537469 | down | mRNA |
| NM_014408 | TRAPPC3 | 0.011297573 | 0.217933835 | 1.092626 | down | mRNA |
| NM_014409 | TAF5L | 0.782713837 | 0.977106756 | 1.0504584 | down | mRNA |
| NM_014412 | CACYBP | 0.9316668 | 0.996710991 | 1.014393 | down | mRNA |
| NM_014413 | EIF2AK1 | 0.008251168 | 0.197897997 | 1.3136817 | down | mRNA |
| NM_014415 | ZBTB11 | 0.011337439 | 0.218288653 | 1.4694194 | down | mRNA |
| NM_014423 | AFF4 | 0.96676669 | 0.99793927 | 1.0038894 | down | mRNA |
| NM_014429 | MORC1 | 0.7826201 | 0.977106756 | 1.0223436 | down | mRNA |
| NM_014430 | CIDEB | 0.279143038 | 0.777816764 | 1.2213871 | down | mRNA |
| NM_014434 | NDOR1 | 0.763970638 | 0.973773636 | 1.1679691 | down | mRNA |
| NM_014444 | TUBGCP4 | 0.034078172 | 0.349898235 | 1.2727646 | down | mRNA |
| NM_014445 | SERP1 | 0.519326014 | 0.916145597 | 1.0453958 | down | mRNA |
| NM_014452 | TNFRSF21 | 0.620620807 | 0.9478661 | 1.056202 | down | mRNA |
| NM_014454 | SESN1 | 0.382950997 | 0.853514109 | 1.2287624 | down | mRNA |
| NM_014455 | RNF115 | 0.204646225 | 0.694679453 | 1.14354 | down | mRNA |
| NM_014456 | PDCD4 | 0.941204979 | 0.996710991 | 1.016409 | down | mRNA |
| NM_014458 | KLHL20 | 0.093638957 | 0.512619241 | 1.341419 | down | mRNA |
| NM_014460 | CSDC2 | 0.839416341 | 0.987032229 | 1.057247 | down | mRNA |
| NM_014462 | LSM1 | 0.071873514 | 0.46230063 | 1.1168463 | down | mRNA |
| NM_014463 | LSM3 | 0.654564662 | 0.957205183 | 1.0481465 | down | mRNA |
| NM_014467 | SRPX2 | 0.661920667 | 0.958354006 | 1.0376883 | down | mRNA |
| NM_014473 | DIMT1 | 0.651040876 | 0.956204521 | 1.0495687 | down | mRNA |
| NM_014478 | CRCP | 0.00030017 | 0.055482352 | 1.2968234 | down | mRNA |
| NM_014480 | ZNF544 | 0.512324198 | 0.91317044 | 1.17024 | down | mRNA |
| NM_014481 | APEX2 | 0.198433581 | 0.686836451 | 1.4746555 | down | mRNA |
| NM_014483 | RBMS3 | 0.76861632 | 0.975191334 | 1.0860619 | down | mRNA |
| NM_014487 | ZNF330 | 0.071201872 | 0.46104346 | 1.5008686 | down | mRNA |
| NM_014488 | RAB30 | 0.782706758 | 0.977106756 | 1.0587654 | down | mRNA |
| NM_014491 | FOXP2 | 0.407343496 | 0.867591329 | 1.1057155 | down | mRNA |
| NM_014497 | ZNF638 | 0.339929332 | 0.828118322 | 1.0934194 | down | mRNA |
| NM_014498 | GOLIM4 | 0.067380914 | 0.451808679 | 1.2507846 | down | mRNA |
| NM_014501 | UBE2S | 0.206653109 | 0.698066071 | 1.2796983 | down | mRNA |
| NM_014502 | PRPF19 | 0.573435326 | 0.933656777 | 1.0592325 | down | mRNA |
| NM_014503 | UTP20 | 0.069364799 | 0.456861883 | 1.141394 | down | mRNA |
| NM_014507 | MCAT | 0.014610181 | 0.24380986 | 1.2577957 | down | mRNA |
| NM_014508 | APOBEC3C | 0.408054167 | 0.867591329 | 1.1652876 | down | mRNA |
| NM_014515 | CNOT2 | 0.064428132 | 0.443678541 | 1.4321542 | down | mRNA |
| NM_014516 | CNOT3 | 0.146441399 | 0.608122555 | 1.1781907 | down | mRNA |
| NM_014520 | MYBBP1A | 0.024009027 | 0.303389731 | 1.1423622 | down | mRNA |
| NM_014550 | CARD10 | 0.004097132 | 0.14691868 | 1.8174704 | down | mRNA |
| NM_014551 | NCAPH2 | 0.665922893 | 0.958354006 | 1.0604315 | down | mRNA |
| NM_014562 | OTX1 | 0.812615246 | 0.98118927 | 1.0510287 | down | mRNA |
| NM_014567 | BCAR1 | 0.615347132 | 0.945385967 | 1.1225486 | down | mRNA |
| NM_014568 | GALNT5 | 0.418843421 | 0.872328878 | 1.0946055 | down | mRNA |
| NM_014572 | LATS2 | 0.28526033 | 0.781963593 | 1.2707983 | down | mRNA |
| NM_014577 | BRD1 | 0.885203338 | 0.992981836 | 1.0144167 | down | mRNA |
| NM_014578 | RHOD | 0.747638724 | 0.970660679 | 1.1001925 | down | mRNA |
| NM_014580 | SLC2A8 | 0.161189638 | 0.632807052 | 1.231933 | down | mRNA |
| NM_014584 | ERO1L | 0.193976104 | 0.681590151 | 1.1162385 | down | mRNA |
| NM_014585 | SLC40A1 | 0.373199885 | 0.848112577 | 1.5922681 | down | mRNA |
| NM_014592 | KCNIP1 | 0.837699864 | 0.986854026 | 1.0230028 | down | mRNA |
| NM_014593 | CXXC1 | 0.09731121 | 0.519982003 | 1.2195293 | down | mRNA |
| NM_014594 | ZNF354C | 0.134348142 | 0.590871682 | 1.5741911 | down | mRNA |
| NM_014595 | NT5C | 0.117776606 | 0.564437853 | 1.0978533 | down | mRNA |
| NM_014597 | DNTTIP2 | 0.151185572 | 0.616500055 | 1.1059729 | down | mRNA |
| NM_014598 | SOCS7 | 0.161270558 | 0.633002505 | 1.1652072 | down | mRNA |
| NM_014601 | EHD2 | 0.714700957 | 0.966110889 | 1.0313615 | down | mRNA |
| NM_014602 | PIK3R4 | 0.655147911 | 0.957205183 | 1.1124009 | down | mRNA |
| NM_014603 | CDR2L | 0.657104142 | 0.957448862 | 1.0624566 | down | mRNA |
| NM_014604 | TAX1BP3 | 0.270196726 | 0.770135843 | 1.059049 | down | mRNA |
| NM_014607 | UBXN4 | 0.1339927 | 0.590871682 | 1.0840287 | down | mRNA |
| NM_014616 | ATP11B | 0.243438893 | 0.741870658 | 1.1467916 | down | mRNA |
| NM_014623 | MEA1 | 0.676173946 | 0.959531882 | 1.0288627 | down | mRNA |
| NM_014624 | S100A6 | 0.827486274 | 0.984980441 | 1.0125014 | down | mRNA |
| NM_014630 | ZNF592 | 0.212312912 | 0.70408516 | 1.139759 | down | mRNA |
| NM_014632 | MICAL2 | 0.207288822 | 0.698556218 | 1.1124823 | down | mRNA |
| NM_014633 | CTR9 | 0.223346007 | 0.71761506 | 1.1390499 | down | mRNA |
| NM_014634 | PPM1F | 0.564011986 | 0.929400006 | 1.1455621 | down | mRNA |
| NM_014637 | MTFR1 | 0.067389383 | 0.451808679 | 1.1427107 | down | mRNA |
| NM_014639 | TTC37 | 0.400996201 | 0.864768873 | 1.077267 | down | mRNA |
| NM_014647 | KIAA0430 | 0.066882098 | 0.451176783 | 1.1903808 | down | mRNA |
| NM_014648 | DZIP3 | 0.451711266 | 0.888854908 | 1.128858 | down | mRNA |
| NM_014649 | SAFB2 | 0.541173036 | 0.923577434 | 1.0465631 | down | mRNA |
| NM_014650 | ZNF432 | 0.965768397 | 0.99793927 | 1.0050355 | down | mRNA |
| NM_014652 | IPO13 | 0.671679338 | 0.959531882 | 1.0316174 | down | mRNA |
| NM_014656 | KIAA0040 | 0.963287824 | 0.99793927 | 1.0176202 | down | mRNA |
| NM_014657 | TTI1 | 0.254896278 | 0.755363815 | 1.1065078 | down | mRNA |
| NM_014660 | PHF14 | 0.20082759 | 0.68900633 | 1.1206424 | down | mRNA |
| NM_014663 | KDM4A | 0.098531923 | 0.522386194 | 1.3456077 | down | mRNA |
| NM_014666 | CLINT1 | 0.10527703 | 0.537137409 | 1.2003485 | down | mRNA |
| NM_014668 | GREB1 | 0.545044469 | 0.924012779 | 1.0654359 | down | mRNA |
| NM_014669 | NUP93 | 0.854190756 | 0.98790708 | 1.0275329 | down | mRNA |
| NM_014670 | BZW1 | 0.78087394 | 0.976938981 | 1.0429317 | down | mRNA |
| NM_014671 | UBE3C | 0.125830124 | 0.578441772 | 1.1754471 | down | mRNA |
| NM_014672 | KIAA0391 | 0.183940522 | 0.667532183 | 1.1050025 | down | mRNA |
| NM_014673 | EMC2 | 0.590215878 | 0.936215326 | 1.06506 | down | mRNA |
| NM_014674 | EDEM1 | 0.197188027 | 0.685161614 | 1.075189 | down | mRNA |
| NM_014676 | PUM1 | 0.058315262 | 0.429582432 | 1.2913995 | down | mRNA |
| NM_014678 | PPP6R2 | 0.50477698 | 0.910568383 | 1.0619972 | down | mRNA |
| NM_014680 | KIAA0100 | 0.145152188 | 0.606482829 | 1.0835779 | down | mRNA |
| NM_014685 | HERPUD1 | 0.266747036 | 0.767278687 | 1.5936765 | down | mRNA |
| NM_014687 | KIAA0226 | 0.009102918 | 0.203095117 | 1.2117638 | down | mRNA |
| NM_014691 | AQR | 0.021534156 | 0.289533061 | 1.3513074 | down | mRNA |
| NM_014699 | ZNF646 | 0.315412773 | 0.810346887 | 1.2480049 | down | mRNA |
| NM_014700 | RAB11FIP3 | 0.076591657 | 0.475659052 | 1.1196218 | down | mRNA |
| NM_014703 | VPRBP | 0.214029247 | 0.706760409 | 1.8445672 | down | mRNA |
| NM_014704 | CEP104 | 0.388713247 | 0.857898453 | 1.0573646 | down | mRNA |
| NM_014706 | SART3 | 0.021631426 | 0.29025889 | 1.4299012 | down | mRNA |
| NM_014708 | KNTC1 | 0.820286872 | 0.982623736 | 1.107263 | down | mRNA |
| NM_014709 | USP34 | 0.174690583 | 0.653303113 | 1.2298979 | down | mRNA |
| NM_014711 | CCP110 | 0.0045745 | 0.155014562 | 1.3866894 | down | mRNA |
| NM_014712 | SETD1A | 0.205558105 | 0.695756183 | 1.2714898 | down | mRNA |
| NM_014714 | IFT140 | 0.154917684 | 0.62235348 | 1.2131261 | down | mRNA |
| NM_014715 | ARHGAP32 | 0.067184728 | 0.45156203 | 1.2353975 | down | mRNA |
| NM_014716 | ACAP1 | 0.515533993 | 0.914326338 | 1.109147 | down | mRNA |
| NM_014718 | CLSTN3 | 0.882002967 | 0.992766819 | 1.0236943 | down | mRNA |
| NM_014719 | FAM115A | 0.233825129 | 0.73118887 | 1.1302958 | down | mRNA |
| NM_014723 | SNPH | 0.987715747 | 0.999140676 | 1.0037475 | down | mRNA |
| NM_014727 | MLL4 | 0.93418127 | 0.996710991 | 1.0117443 | down | mRNA |
| NM_014730 | MLEC | 0.294794002 | 0.791301949 | 1.082517 | down | mRNA |
| NM_014732 | KIAA0513 | 0.872724365 | 0.990534615 | 1.0140935 | down | mRNA |
| NM_014733 | ZFYVE16 | 0.835551743 | 0.986575022 | 1.0290818 | down | mRNA |
| NM_014738 | KIAA0195 | 0.123689019 | 0.577726888 | 1.0757997 | down | mRNA |
| NM_014741 | ATG13 | 0.532057958 | 0.920228311 | 1.0473082 | down | mRNA |
| NM_014746 | RNF144A | 0.069699569 | 0.457632202 | 1.0738762 | down | mRNA |
| NM_014748 | SNX17 | 0.067131425 | 0.451445852 | 1.1582326 | down | mRNA |
| NM_014749 | KIAA0586 | 0.855479601 | 0.988178307 | 1.0476575 | down | mRNA |
| NM_014750 | DLGAP5 | 0.71854249 | 0.966191059 | 1.1826542 | down | mRNA |
| NM_014753 | BMS1 | 0.328425629 | 0.821245835 | 1.1097016 | down | mRNA |
| NM_014754 | PTDSS1 | 0.00489289 | 0.158753988 | 1.2182866 | down | mRNA |
| NM_014756 | CKAP5 | 0.067688893 | 0.451808679 | 1.2036096 | down | mRNA |
| NM_014758 | SNX19 | 0.563693528 | 0.929146073 | 1.1809276 | down | mRNA |
| NM_014762 | DHCR24 | 0.0926506 | 0.511338764 | 1.0929624 | down | mRNA |
| NM_014763 | MRPL19 | 0.307706307 | 0.802396387 | 1.3529961 | down | mRNA |
| NM_014765 | TOMM20 | 0.117057547 | 0.56398437 | 1.2029689 | down | mRNA |
| NM_014781 | RB1CC1 | 0.732975988 | 0.967703318 | 1.010706 | down | mRNA |
| NM_014782 | ARMCX2 | 0.096184243 | 0.51782059 | 1.2206828 | down | mRNA |
| NM_014783 | ARHGAP11A | 0.35480192 | 0.83766056 | 1.590941 | down | mRNA |
| NM_014786 | ARHGEF17 | 0.093590446 | 0.512619241 | 1.2063134 | down | mRNA |
| NM_014793 | LCMT2 | 0.412263646 | 0.869156414 | 1.0562531 | down | mRNA |
| NM_014797 | ZBTB24 | 0.093884592 | 0.513134818 | 1.3272697 | down | mRNA |
| NM_014798 | PLEKHM1 | 0.007173808 | 0.189833754 | 1.1758072 | down | mRNA |
| NM_014802 | KIAA0528 | 0.163859083 | 0.63628397 | 1.2492801 | down | mRNA |
| NM_014803 | ZNF518A | 0.414626267 | 0.870239791 | 1.285647 | down | mRNA |
| NM_014804 | KIAA0753 | 0.028603894 | 0.328520317 | 1.3192311 | down | mRNA |
| NM_014812 | CEP170 | 0.057807153 | 0.428173053 | 1.2783625 | down | mRNA |
| NM_014813 | LRIG2 | 0.035655471 | 0.354652995 | 1.3510618 | down | mRNA |
| NM_014814 | PSMD6 | 0.124831444 | 0.578441772 | 1.1195909 | down | mRNA |
| NM_014819 | PJA2 | 0.328675516 | 0.821614764 | 1.229923 | down | mRNA |
| NM_014820 | TOMM70A | 0.001933803 | 0.107205943 | 1.2469849 | down | mRNA |
| NM_014822 | SEC24D | 0.070031646 | 0.457987595 | 1.1689664 | down | mRNA |
| NM_014823 | WNK1 | 0.153640447 | 0.620667112 | 1.1599102 | down | mRNA |
| NM_014824 | FCHSD2 | 0.710970072 | 0.96568622 | 1.0371458 | down | mRNA |
| NM_014825 | URB1 | 0.255596691 | 0.756337058 | 1.3929202 | down | mRNA |
| NM_014827 | ZC3H11A | 0.253235032 | 0.753288068 | 1.2658275 | down | mRNA |
| NM_014828 | TOX4 | 0.542088699 | 0.923577434 | 1.0503974 | down | mRNA |
| NM_014832 | TBC1D4 | 0.194711883 | 0.682390954 | 1.0907176 | down | mRNA |
| NM_014835 | OSBPL2 | 0.384426816 | 0.85485898 | 1.0678703 | down | mRNA |
| NM_014838 | ZBED4 | 0.098599086 | 0.52242281 | 1.1324292 | down | mRNA |
| NM_014840 | NUAK1 | 0.001837731 | 0.105670067 | 1.8781221 | down | mRNA |
| NM_014845 | FIG4 | 0.216791206 | 0.711074992 | 1.2036465 | down | mRNA |
| NM_014846 | KIAA0196 | 0.074390621 | 0.469723636 | 1.2287109 | down | mRNA |
| NM_014847 | UBAP2L | 0.082785682 | 0.490850228 | 1.1309377 | down | mRNA |
| NM_014853 | SGSM2 | 0.44292438 | 0.884304813 | 1.0759703 | down | mRNA |
| NM_014857 | RABGAP1L | 0.136467674 | 0.595165326 | 1.0869649 | down | mRNA |
| NM_014858 | TMCC2 | 0.589928738 | 0.936114189 | 1.2886715 | down | mRNA |
| NM_014859 | ARHGAP44 | 0.245002333 | 0.743548975 | 1.2745875 | down | mRNA |
| NM_014862 | ARNT2 | 0.655791428 | 0.957319882 | 1.0698153 | down | mRNA |
| NM_014864 | FAM20B | 0.4746763 | 0.8995357 | 1.0694033 | down | mRNA |
| NM_014865 | NCAPD2 | 0.860900585 | 0.989013989 | 1.0706889 | down | mRNA |
| NM_014866 | SEC16A | 0.452098015 | 0.888854908 | 1.0689391 | down | mRNA |
| NM_014867 | KBTBD11 | 0.441670816 | 0.88412435 | 1.0328335 | down | mRNA |
| NM_014868 | RNF10 | 0.187717103 | 0.672162493 | 1.0918679 | down | mRNA |
| NM_014869 | IQSEC1 | 0.285355355 | 0.781963593 | 1.1088381 | down | mRNA |
| NM_014877 | HELZ | 0.085832979 | 0.498103045 | 1.2230021 | down | mRNA |
| NM_014878 | KIAA0020 | 0.219476015 | 0.714276371 | 1.0915738 | down | mRNA |
| NM_014881 | DCLRE1A | 0.010001723 | 0.207620683 | 1.3292584 | down | mRNA |
| NM_014882 | ARHGAP25 | 0.817709957 | 0.981727075 | 1.02295 | down | mRNA |
| NM_014885 | ANAPC10 | 0.089707228 | 0.506107454 | 1.1375638 | down | mRNA |
| NM_014887 | N4BP2L2 | 0.562156897 | 0.928947824 | 1.0605226 | down | mRNA |
| NM_014888 | FAM3C | 0.33666746 | 0.826506012 | 1.1088241 | down | mRNA |
| NM_014891 | PDAP1 | 0.00142216 | 0.09664422 | 1.2339349 | down | mRNA |
| NM_014892 | SCAF8 | 0.042657083 | 0.382084661 | 1.3005849 | down | mRNA |
| NM_014895 | KIAA1009 | 0.132506724 | 0.588108866 | 1.2533633 | down | mRNA |
| NM_014897 | ZNF652 | 0.004452513 | 0.153101361 | 1.3025497 | down | mRNA |
| NM_014899 | RHOBTB3 | 0.887835235 | 0.993391292 | 1.0512121 | down | mRNA |
| NM_014901 | RNF44 | 0.400748411 | 0.864768873 | 1.0602337 | down | mRNA |
| NM_014905 | GLS | 0.774116309 | 0.97589756 | 1.1776622 | down | mRNA |
| NM_014910 | ZNF507 | 0.032480225 | 0.34395205 | 1.2976489 | down | mRNA |
| NM_014916 | LMTK2 | 0.038743554 | 0.367756276 | 1.434412 | down | mRNA |
| NM_014922 | NLRP1 | 0.000502695 | 0.068138676 | 1.2900767 | down | mRNA |
| NM_014923 | FNDC3A | 0.317937401 | 0.811908136 | 1.0840086 | down | mRNA |
| NM_014924 | ATG14 | 0.356068526 | 0.838847221 | 1.0929006 | down | mRNA |
| NM_014935 | PLEKHA6 | 0.646168293 | 0.95550188 | 1.0827416 | down | mRNA |
| NM_014937 | INPP5F | 0.044763708 | 0.38849334 | 1.3972342 | down | mRNA |
| NM_014938 | MLXIP | 0.087930688 | 0.501543709 | 1.117657 | down | mRNA |
| NM_014939 | TRAPPC8 | 0.016421642 | 0.255922225 | 1.4227184 | down | mRNA |
| NM_014941 | MORC2 | 0.212660387 | 0.704662808 | 1.1289567 | down | mRNA |
| NM_014942 | ANKRD6 | 0.080183347 | 0.483876854 | 1.1652982 | down | mRNA |
| NM_014949 | KIAA0907 | 0.24994075 | 0.748423466 | 1.2459733 | down | mRNA |
| NM_014951 | ZNF365 | 0.334186088 | 0.824174306 | 1.1215244 | down | mRNA |
| NM_014954 | RPH3A | 0.603633146 | 0.941514447 | 1.0460118 | down | mRNA |
| NM_014955 | METTL13 | 0.034690711 | 0.352074963 | 1.3956401 | down | mRNA |
| NM_014961 | RUFY3 | 0.012157904 | 0.223099738 | 1.6123303 | down | mRNA |
| NM_014963 | SBNO2 | 0.625820287 | 0.948851902 | 1.139988 | down | mRNA |
| NM_014965 | TRAK1 | 0.238035607 | 0.735970189 | 1.3664599 | down | mRNA |
| NM_014967 | FAN1 | 2.73485E-05 | 0.022241958 | 1.5245797 | down | mRNA |
| NM_014969 | WDR47 | 0.496853065 | 0.908665955 | 1.0895068 | down | mRNA |
| NM_014971 | EFR3B | 0.55793946 | 0.926833616 | 1.2435264 | down | mRNA |
| NM_014972 | TCF25 | 0.587973973 | 0.93579682 | 1.0574593 | down | mRNA |
| NM_014976 | PDCD11 | 0.590222706 | 0.936215326 | 1.0796592 | down | mRNA |
| NM_014979 | SV2C | 0.828645352 | 0.985493382 | 1.0314783 | down | mRNA |
| NM_014984 | AZI1 | 0.115739692 | 0.561584069 | 1.2588864 | down | mRNA |
| NM_014985 | CEP152 | 0.391016338 | 0.858960277 | 1.4195935 | down | mRNA |
| NM_014989 | RIMS1 | 0.469578873 | 0.89787452 | 1.2191175 | down | mRNA |
| NM_014991 | WDFY3 | 0.161852961 | 0.633820185 | 1.1785727 | down | mRNA |
| NM_014997 | KLHDC10 | 0.557581336 | 0.926833616 | 1.0640578 | down | mRNA |
| NM_015000 | STK38L | 0.466391888 | 0.89599965 | 1.2616053 | down | mRNA |
| NM_015002 | FBXO21 | 0.519249082 | 0.916145597 | 1.0528397 | down | mRNA |
| NM_015005 | KIAA0284 | 0.378903877 | 0.852482258 | 1.1798709 | down | mRNA |
| NM_015012 | TMEM41B | 0.031234404 | 0.340775376 | 1.4845766 | down | mRNA |
| NM_015013 | KDM1A | 0.697789767 | 0.962410025 | 1.0258938 | down | mRNA |
| NM_015014 | RBM34 | 0.033967899 | 0.349511805 | 1.2574311 | down | mRNA |
| NM_015015 | KDM4B | 0.367661655 | 0.845181673 | 1.1513287 | down | mRNA |
| NM_015017 | USP33 | 0.18057027 | 0.663537814 | 1.285179 | down | mRNA |
| NM_015021 | ZNF292 | 0.000196724 | 0.047904279 | 1.933451 | down | mRNA |
| NM_015024 | XPO7 | 0.80166519 | 0.98118927 | 1.0294338 | down | mRNA |
| NM_015026 | MON2 | 0.365703944 | 0.845038366 | 1.1901029 | down | mRNA |
| NM_015027 | PDXDC1 | 0.262955543 | 0.763125731 | 1.2061376 | down | mRNA |
| NM_015032 | PDS5B | 0.031673413 | 0.341807319 | 1.3686977 | down | mRNA |
| NM_015041 | CLUAP1 | 0.089829761 | 0.506494371 | 1.1874788 | down | mRNA |
| NM_015046 | SETX | 0.430316477 | 0.87878612 | 1.1585018 | down | mRNA |
| NM_015047 | EMC1 | 0.092533939 | 0.511338764 | 2.6106133 | down | mRNA |
| NM_015049 | TRAK2 | 0.393103624 | 0.859795559 | 1.1520561 | down | mRNA |
| NM_015051 | ERP44 | 0.511243267 | 0.912481293 | 1.0653685 | down | mRNA |
| NM_015054 | UHRF1BP1L | 0.433187989 | 0.880722826 | 1.0786805 | down | mRNA |
| NM_015055 | SWAP70 | 0.860950511 | 0.989013989 | 1.0581768 | down | mRNA |
| NM_015056 | RRP1B | 0.197845145 | 0.686504248 | 1.1580934 | down | mRNA |
| NM_015059 | TLN2 | 0.572021174 | 0.933338778 | 1.1736784 | down | mRNA |
| NM_015060 | AVL9 | 0.439618387 | 0.883054028 | 1.0931497 | down | mRNA |
| NM_015061 | KDM4C | 0.238974829 | 0.736968939 | 1.321237 | down | mRNA |
| NM_015071 | ARHGAP26 | 0.975623891 | 0.998501532 | 1.0052684 | down | mRNA |
| NM_015072 | TTLL5 | 0.778153851 | 0.976938981 | 1.0961324 | down | mRNA |
| NM_015073 | SIPA1L3 | 0.011377788 | 0.218702965 | 1.1243358 | down | mRNA |
| NM_015075 | IQSEC2 | 0.471659988 | 0.898908705 | 1.1698981 | down | mRNA |
| NM_015076 | CDK19 | 0.890691095 | 0.9937521 | 1.0158895 | down | mRNA |
| NM_015083 | CDK12 | 0.793483522 | 0.979128905 | 1.0301402 | down | mRNA |
| NM_015084 | MRPS27 | 0.209617924 | 0.700367256 | 1.1291138 | down | mRNA |
| NM_015085 | RAP1GAP2 | 0.565015565 | 0.929921137 | 1.0884026 | down | mRNA |
| NM_015088 | TNRC6B | 0.707244103 | 0.965401045 | 1.0752163 | down | mRNA |
| NM_015090 | NFASC | 0.825982182 | 0.984306256 | 1.0274709 | down | mRNA |
| NM_015091 | FAM179B | 0.07698207 | 0.477122268 | 1.4643357 | down | mRNA |
| NM_015094 | HIC2 | 0.868838274 | 0.989481868 | 1.0181031 | down | mRNA |
| NM_015097 | CLASP2 | 0.047087185 | 0.395610183 | 1.29572 | down | mRNA |
| NM_015099 | CAMTA2 | 0.710531221 | 0.965504602 | 1.0198578 | down | mRNA |
| NM_015101 | GLT25D2 | 0.124862697 | 0.578441772 | 1.1165537 | down | mRNA |
| NM_015103 | PLXND1 | 0.640620237 | 0.953894897 | 1.1849239 | down | mRNA |
| NM_015104 | ATG2A | 0.097701983 | 0.520535504 | 1.0831065 | down | mRNA |
| NM_015106 | RAD54L2 | 0.651837539 | 0.956204521 | 1.0488785 | down | mRNA |
| NM_015107 | PHF8 | 0.628511067 | 0.949961866 | 1.0257077 | down | mRNA |
| NM_015113 | ZZEF1 | 0.503577034 | 0.910553209 | 1.0252786 | down | mRNA |
| NM_015117 | ZC3H3 | 0.17079262 | 0.647381721 | 1.2414574 | down | mRNA |
| NM_015123 | FRMD4B | 0.438748756 | 0.882895421 | 1.5537134 | down | mRNA |
| NM_015129 | SEPT6 | 0.385010341 | 0.855147437 | 1.1867645 | down | mRNA |
| NM_015130 | TBC1D9 | 0.408501808 | 0.867591329 | 1.1031281 | down | mRNA |
| NM_015131 | WDR43 | 0.08454423 | 0.494805206 | 1.1655905 | down | mRNA |
| NM_015132 | SNX13 | 0.466579102 | 0.89599965 | 1.095379 | down | mRNA |
| NM_015133 | MAPK8IP3 | 0.607594188 | 0.942627675 | 1.0969842 | down | mRNA |
| NM_015134 | MPRIP | 0.373213915 | 0.848112577 | 1.0863895 | down | mRNA |
| NM_015135 | NUP205 | 0.348213107 | 0.834138149 | 1.1062932 | down | mRNA |
| NM_015139 | SLC35D1 | 0.900101857 | 0.994774743 | 1.0147485 | down | mRNA |
| NM_015140 | TTLL12 | 0.402360639 | 0.864786348 | 1.0646982 | down | mRNA |
| NM_015141 | GPD1L | 0.529148166 | 0.91899174 | 1.1373866 | down | mRNA |
| NM_015143 | METAP1 | 0.92795482 | 0.995916751 | 1.0201062 | down | mRNA |
| NM_015144 | ZCCHC14 | 0.010371466 | 0.209736088 | 1.518512 | down | mRNA |
| NM_015146 | SEPT8 | 0.867964535 | 0.989481868 | 1.0126203 | down | mRNA |
| NM_015147 | CEP68 | 0.559393475 | 0.927203296 | 1.1527616 | down | mRNA |
| NM_015151 | DIP2A | 0.325515112 | 0.81809311 | 1.2417883 | down | mRNA |
| NM_015156 | RCOR1 | 0.078026967 | 0.479868206 | 1.3777768 | down | mRNA |
| NM_015157 | PHLDB1 | 0.75660976 | 0.971824502 | 1.0443301 | down | mRNA |
| NM_015160 | PMPCA | 0.144053738 | 0.604894797 | 1.184647 | down | mRNA |
| NM_015161 | ARL6IP1 | 0.043278387 | 0.383916304 | 1.2093923 | down | mRNA |
| NM_015167 | JMJD6 | 0.426173056 | 0.875717896 | 1.058023 | down | mRNA |
| NM_015168 | ZC3H4 | 0.036244511 | 0.35703653 | 1.1209258 | down | mRNA |
| NM_015169 | RRS1 | 0.000393079 | 0.061008278 | 1.6791979 | down | mRNA |
| NM_015177 | DTX4 | 0.442390333 | 0.88419144 | 2.1254505 | down | mRNA |
| NM_015178 | RHOBTB2 | 0.263903629 | 0.764495522 | 1.2688231 | down | mRNA |
| NM_015185 | ARHGEF9 | 0.056237131 | 0.422391335 | 1.4293749 | down | mRNA |
| NM_015190 | DNAJC9 | 0.86552084 | 0.989401142 | 1.0778855 | down | mRNA |
| NM_015196 | KIAA0922 | 0.824837024 | 0.98381451 | 1.0255194 | down | mRNA |
| NM_015202 | KIAA0556 | 0.000465079 | 0.066148041 | 1.7133569 | down | mRNA |
| NM_015203 | RPRD2 | 0.111903646 | 0.554156842 | 1.4092756 | down | mRNA |
| NM_015208 | ANKRD12 | 0.838278821 | 0.986897498 | 1.0186443 | down | mRNA |
| NM_015216 | PPIP5K2 | 0.042677147 | 0.382084661 | 1.2651499 | down | mRNA |
| NM_015219 | EXOC7 | 0.131564843 | 0.586786184 | 1.1005769 | down | mRNA |
| NM_015224 | FAM208A | 0.092072007 | 0.511207518 | 1.1592313 | down | mRNA |
| NM_015225 | PRUNE2 | 0.575520038 | 0.933656777 | 1.0489012 | down | mRNA |
| NM_015231 | NUP160 | 0.152477933 | 0.618255775 | 1.1563307 | down | mRNA |
| NM_015232 | IQSEC3 | 0.983819223 | 0.998971807 | 1.0029992 | down | mRNA |
| NM_015235 | CSTF2T | 0.554810997 | 0.926833616 | 1.0514663 | down | mRNA |
| NM_015239 | AGTPBP1 | 0.883493172 | 0.992766819 | 1.0342887 | down | mRNA |
| NM_015242 | ARAP1 | 0.001290398 | 0.093126586 | 1.3562991 | down | mRNA |
| NM_015245 | ANKS1A | 0.103390439 | 0.533123263 | 1.2130488 | down | mRNA |
| NM_015250 | BICD2 | 0.408093707 | 0.867591329 | 1.0606322 | down | mRNA |
| NM_015252 | EHBP1 | 0.030139013 | 0.336976308 | 1.2031977 | down | mRNA |
| NM_015255 | UBR2 | 0.234033345 | 0.731381183 | 1.2187762 | down | mRNA |
| NM_015257 | TMEM194A | 0.526500603 | 0.917975454 | 1.5001342 | down | mRNA |
| NM_015261 | NCAPD3 | 0.434415814 | 0.880931467 | 1.3966442 | down | mRNA |
| NM_015262 | FAM21C | 0.925495386 | 0.995465766 | 1.0186637 | down | mRNA |
| NM_015264 | KIAA0930 | 0.589708579 | 0.936090876 | 1.0842937 | down | mRNA |
| NM_015268 | DNAJC13 | 0.018040528 | 0.269173581 | 1.3266456 | down | mRNA |
| NM_015275 | KIAA1033 | 0.462123396 | 0.894754673 | 1.1402288 | down | mRNA |
| NM_015276 | USP22 | 0.408323067 | 0.867591329 | 1.0533485 | down | mRNA |
| NM_015278 | SASH1 | 0.505778263 | 0.911021812 | 1.1100123 | down | mRNA |
| NM_015282 | CLASP1 | 0.838602307 | 0.98694974 | 1.0291113 | down | mRNA |
| NM_015283 | DPY19L1 | 0.351291956 | 0.835187247 | 1.1408711 | down | mRNA |
| NM_015291 | DNAJC16 | 0.602293272 | 0.941406011 | 1.0475602 | down | mRNA |
| NM_015292 | ESYT1 | 0.14312798 | 0.603147721 | 1.089085 | down | mRNA |
| NM_015294 | TRIM37 | 0.220507305 | 0.715799221 | 1.1661249 | down | mRNA |
| NM_015295 | SMCHD1 | 0.430275904 | 0.87878612 | 1.0807996 | down | mRNA |
| NM_015297 | KIAA1045 | 0.446225782 | 0.886435036 | 1.0777117 | down | mRNA |
| NM_015305 | ANGEL1 | 0.220765524 | 0.716020069 | 1.1809959 | down | mRNA |
| NM_015307 | FAM189A1 | 0.798198024 | 0.980749101 | 1.0706571 | down | mRNA |
| NM_015308 | FNBP4 | 0.387451066 | 0.856773191 | 1.1351575 | down | mRNA |
| NM_015317 | PUM2 | 0.12618569 | 0.578621438 | 1.2407137 | down | mRNA |
| NM_015318 | ARHGEF18 | 0.745645679 | 0.970394407 | 1.0499058 | down | mRNA |
| NM_015319 | TENC1 | 0.570535397 | 0.93296476 | 1.1419941 | down | mRNA |
| NM_015320 | ARHGEF4 | 0.858850078 | 0.988512246 | 1.0237539 | down | mRNA |
| NM_015321 | CRTC1 | 0.481242207 | 0.901538317 | 1.0881669 | down | mRNA |
| NM_015322 | FEM1B | 0.092327004 | 0.511338764 | 1.3081198 | down | mRNA |
| NM_015323 | UFL1 | 0.013880769 | 0.237651715 | 1.297995 | down | mRNA |
| NM_015324 | RRP8 | 0.132711759 | 0.588376689 | 1.1983973 | down | mRNA |
| NM_015325 | KIAA0947 | 0.137262516 | 0.596584327 | 1.2103637 | down | mRNA |
| NM_015326 | SRGAP2 | 0.057487336 | 0.42700171 | 1.1756804 | down | mRNA |
| NM_015327 | SMG5 | 0.888055703 | 0.993440915 | 1.0293031 | down | mRNA |
| NM_015328 | AHCYL2 | 0.277113519 | 0.777205298 | 1.4107087 | down | mRNA |
| NM_015329 | MAU2 | 0.887734067 | 0.993363184 | 1.0275163 | down | mRNA |
| NM_015331 | NCSTN | 0.38845534 | 0.857898453 | 1.0481322 | down | mRNA |
| NM_015332 | NUDCD3 | 0.127462352 | 0.580991132 | 1.2600645 | down | mRNA |
| NM_015335 | MED13L | 0.254470472 | 0.754895553 | 1.1132844 | down | mRNA |
| NM_015336 | ZDHHC17 | 0.123891597 | 0.577744894 | 1.0668014 | down | mRNA |
| NM_015339 | ADNP | 0.036767877 | 0.359628653 | 1.1825203 | down | mRNA |
| NM_015342 | PPWD1 | 0.002978231 | 0.129891793 | 1.3352874 | down | mRNA |
| NM_015343 | CTDNEP1 | 0.024978896 | 0.308874358 | 1.1799996 | down | mRNA |
| NM_015344 | LEPROTL1 | 0.450748024 | 0.888732431 | 1.0848214 | down | mRNA |
| NM_015352 | POFUT1 | 0.311518337 | 0.806852334 | 1.1316026 | down | mRNA |
| NM_015353 | KCTD2 | 0.969604169 | 0.998245244 | 1.0037022 | down | mRNA |
| NM_015354 | NUP188 | 0.438107311 | 0.882682266 | 1.0717574 | down | mRNA |
| NM_015355 | SUZ12 | 0.286382947 | 0.782941789 | 1.0845857 | down | mRNA |
| NM_015360 | SKIV2L2 | 0.143591208 | 0.603870418 | 1.1349678 | down | mRNA |
| NM_015361 | R3HDM1 | 0.037202548 | 0.360363126 | 1.2390196 | down | mRNA |
| NM_015365 | AMMECR1 | 0.231592151 | 0.729296323 | 1.9908315 | down | mRNA |
| NM_015370 | C22orf31 | 0.380355377 | 0.853407568 | 1.055554 | down | mRNA |
| NM_015375 | DSTYK | 0.311811416 | 0.806920827 | 1.1421199 | down | mRNA |
| NM_015379 | BRI3 | 0.228334307 | 0.724597025 | 1.1865896 | down | mRNA |
| NM_015380 | SAMM50 | 0.011815937 | 0.221393704 | 1.2031961 | down | mRNA |
| NM_015384 | NIPBL | 0.392832471 | 0.859694706 | 1.0771794 | down | mRNA |
| NM_015386 | COG4 | 0.060860836 | 0.436633496 | 1.1257088 | down | mRNA |
| NM_015387 | MOB4 | 0.083363636 | 0.492251581 | 1.3284941 | down | mRNA |
| NM_015392 | NPDC1 | 0.48507341 | 0.903250293 | 1.0426009 | down | mRNA |
| NM_015393 | PARM1 | 0.473474359 | 0.899184677 | 1.1520756 | down | mRNA |
| NM_015394 | ZNF10 | 0.026061716 | 0.315973057 | 1.3944118 | down | mRNA |
| NM_015395 | TECPR1 | 0.218312548 | 0.712269655 | 1.2734957 | down | mRNA |
| NM_015396 | ARMC8 | 0.089749703 | 0.506183348 | 1.1552079 | down | mRNA |
| NM_015399 | BRMS1 | 0.050891335 | 0.407751844 | 1.3220328 | down | mRNA |
| NM_015401 | HDAC7 | 0.052446553 | 0.412637354 | 1.3288543 | down | mRNA |
| NM_015411 | SUMF2 | 0.556896344 | 0.926833616 | 1.082522 | down | mRNA |
| NM_015412 | C3orf17 | 0.06040484 | 0.435669103 | 1.2529701 | down | mRNA |
| NM_015416 | LETMD1 | 0.003521643 | 0.13849526 | 1.442142 | down | mRNA |
| NM_015419 | MXRA5 | 0.980010229 | 0.998971807 | 1.0034804 | down | mRNA |
| NM_015420 | DCAF13 | 0.718248251 | 0.966191059 | 1.0393818 | down | mRNA |
| NM_015423 | AASDHPPT | 0.522234003 | 0.917010256 | 1.0283272 | down | mRNA |
| NM_015425 | POLR1A | 0.338559894 | 0.827403747 | 1.1137782 | down | mRNA |
| NM_015426 | POC1A | 0.733284227 | 0.967777568 | 1.1532705 | down | mRNA |
| NM_015428 | ZNF473 | 0.133180866 | 0.589162633 | 1.3252013 | down | mRNA |
| NM_015430 | PAMR1 | 0.9811086 | 0.998971807 | 1.0123133 | down | mRNA |
| NM_015434 | INTS7 | 0.014002744 | 0.238634683 | 1.2823086 | down | mRNA |
| NM_015436 | RCHY1 | 0.008147522 | 0.196966143 | 1.3460886 | down | mRNA |
| NM_015440 | MTHFD1L | 0.63872668 | 0.953122601 | 1.0555744 | down | mRNA |
| NM_015444 | TMEM158 | 0.799272768 | 0.980917337 | 1.0164932 | down | mRNA |
| NM_015446 | AHCTF1 | 0.424658984 | 0.874854499 | 1.0456268 | down | mRNA |
| NM_015447 | CAMSAP1 | 0.202206728 | 0.69131473 | 1.1364879 | down | mRNA |
| NM_015448 | DPCD | 0.953192315 | 0.996710991 | 1.0048194 | down | mRNA |
| NM_015449 | C1orf43 | 0.226019597 | 0.721636281 | 1.1260351 | down | mRNA |
| NM_015450 | POT1 | 0.062129575 | 0.439379649 | 1.1478496 | down | mRNA |
| NM_015456 | COBRA1 | 0.015051145 | 0.246131665 | 1.2155439 | down | mRNA |
| NM_015460 | MYRIP | 0.852500249 | 0.987678166 | 1.0315578 | down | mRNA |
| NM_015461 | ZNF521 | 0.279305538 | 0.777816764 | 1.4465598 | down | mRNA |
| NM_015462 | NOL11 | 0.033953725 | 0.349511805 | 1.1713772 | down | mRNA |
| NM_015463 | CNRIP1 | 0.315960746 | 0.810613741 | 2.2283744 | down | mRNA |
| NM_015465 | GEMIN5 | 0.095215101 | 0.516597924 | 1.4118764 | down | mRNA |
| NM_015471 | NSL1 | 0.079724205 | 0.483289365 | 1.0978876 | down | mRNA |
| NM_015478 | L3MBTL1 | 0.327323471 | 0.820319134 | 1.3727266 | down | mRNA |
| NM_015480 | PVRL3 | 0.027131694 | 0.320017762 | 1.1534765 | down | mRNA |
| NM_015483 | KBTBD2 | 0.947870692 | 0.996710991 | 1.0112846 | down | mRNA |
| NM_015484 | SYF2 | 0.27496986 | 0.774827385 | 1.0768662 | down | mRNA |
| NM_015490 | SEC31B | 0.519468532 | 0.916145597 | 1.1336625 | down | mRNA |
| NM_015491 | PNISR | 0.214117762 | 0.706760409 | 1.1936266 | down | mRNA |
| NM_015493 | KANK2 | 0.337987441 | 0.827394958 | 1.0664211 | down | mRNA |
| NM_015496 | KIAA1429 | 0.170562548 | 0.64732212 | 1.1612266 | down | mRNA |
| NM_015497 | TMEM87A | 0.329238094 | 0.821768501 | 1.09037 | down | mRNA |
| NM_015509 | NECAP1 | 0.352796826 | 0.836119019 | 1.1296428 | down | mRNA |
| NM_015511 | AAR2 | 0.348851265 | 0.834138149 | 1.044737 | down | mRNA |
| NM_015512 | DNAH1 | 0.549917798 | 0.925533435 | 1.112896 | down | mRNA |
| NM_015514 | NGDN | 0.474442569 | 0.899495615 | 1.1031191 | down | mRNA |
| NM_015515 | KRT23 | 0.635181631 | 0.95145488 | 1.4562995 | down | mRNA |
| NM_015516 | TSKU | 0.644764888 | 0.954657712 | 1.0654371 | down | mRNA |
| NM_015520 | MAGI1 | 0.0559828 | 0.42172741 | 1.263264 | down | mRNA |
| NM_015522 | DYNC2LI1 | 0.109398033 | 0.547509603 | 1.2238356 | down | mRNA |
| NM_015527 | TBC1D10B | 0.685360956 | 0.960111041 | 1.0814489 | down | mRNA |
| NM_015528 | RNF167 | 0.58535748 | 0.934708084 | 1.0292485 | down | mRNA |
| NM_015529 | MOXD1 | 0.741237136 | 0.970240145 | 1.0137235 | down | mRNA |
| NM_015530 | GORASP2 | 0.013500947 | 0.235623837 | 1.2520475 | down | mRNA |
| NM_015533 | DAK | 0.636892353 | 0.952364148 | 1.058988 | down | mRNA |
| NM_015534 | ZZZ3 | 0.29241778 | 0.788954556 | 1.092177 | down | mRNA |
| NM_015535 | SPATS2L | 0.601263875 | 0.941028182 | 1.0270953 | down | mRNA |
| NM_015537 | NELF | 0.947677241 | 0.996710991 | 1.0108746 | down | mRNA |
| NM_015543 | RAB1A | 0.447692889 | 0.887547822 | 1.0857199 | down | mRNA |
| NM_015544 | TMEM98 | 0.732897816 | 0.967703318 | 1.0491316 | down | mRNA |
| NM_015545 | PTCD1 | 0.950998158 | 0.996710991 | 1.030954 | down | mRNA |
| NM_015548 | DST | 0.746606294 | 0.970432947 | 1.0546565 | down | mRNA |
| NM_015549 | PLEKHG3 | 0.861127599 | 0.989013989 | 1.1047266 | down | mRNA |
| NM_015550 | OSBPL3 | 0.0057309 | 0.17178751 | 1.3715982 | down | mRNA |
| NM_015555 | ZNF451 | 0.013396111 | 0.23544335 | 1.5212895 | down | mRNA |
| NM_015557 | CHD5 | 0.786217876 | 0.977479172 | 1.0421018 | down | mRNA |
| NM_015560 | OPA1 | 0.12464199 | 0.578441772 | 1.115533 | down | mRNA |
| NM_015571 | SENP6 | 0.381757041 | 0.853514109 | 1.0991377 | down | mRNA |
| NM_015575 | GIGYF2 | 0.217640304 | 0.711768162 | 1.1782542 | down | mRNA |
| NM_015584 | POLDIP2 | 0.321816081 | 0.815271211 | 1.0682345 | down | mRNA |
| NM_015600 | ABHD12 | 0.275995883 | 0.775741613 | 1.0296116 | down | mRNA |
| NM_015601 | HERC4 | 0.415628188 | 0.870382888 | 1.3117062 | down | mRNA |
| NM_015602 | TOR1AIP1 | 0.219619533 | 0.714276371 | 1.080663 | down | mRNA |
| NM_015604 | DCAF4 | 0.71112102 | 0.96568622 | 1.122901 | down | mRNA |
| NM_015607 | CHTOP | 0.04966093 | 0.403398333 | 1.0870142 | down | mRNA |
| NM_015608 | C10orf137 | 0.020836466 | 0.286635334 | 1.2312565 | down | mRNA |
| NM_015609 | SZRD1 | 0.21854536 | 0.712342894 | 1.2175083 | down | mRNA |
| NM_015617 | PYGO1 | 0.391610692 | 0.859202394 | 1.153064 | down | mRNA |
| NM_015621 | CCDC69 | 0.501886188 | 0.910194076 | 1.1863328 | down | mRNA |
| NM_015622 | CCZ1 | 0.346196355 | 0.833163669 | 1.0699984 | down | mRNA |
| NM_015630 | EPC2 | 0.146202128 | 0.608122555 | 1.183528 | down | mRNA |
| NM_015633 | FGFR1OP2 | 0.137712544 | 0.596789644 | 1.2139511 | down | mRNA |
| NM_015640 | SERBP1 | 0.327910692 | 0.820765751 | 1.9961763 | down | mRNA |
| NM_015645 | C1QTNF5 | 0.452078729 | 0.888854908 | 1.1485753 | down | mRNA |
| NM_015650 | TRAF3IP1 | 0.476936607 | 0.900429473 | 1.0748771 | down | mRNA |
| NM_015652 | C10orf12 | 0.08638852 | 0.49936346 | 1.2648331 | down | mRNA |
| NM_015653 | RIBC2 | 0.726933938 | 0.966191059 | 1.1746947 | down | mRNA |
| NM_015655 | ZNF337 | 0.89053401 | 0.9937521 | 1.0392399 | down | mRNA |
| NM_015656 | KIF26A | 0.247199181 | 0.745152522 | 1.1472936 | down | mRNA |
| NM_015658 | NOC2L | 0.257660211 | 0.7580303 | 1.5185604 | down | mRNA |
| NM_015659 | RSL1D1 | 0.376378598 | 0.849903337 | 1.0859721 | down | mRNA |
| NM_015662 | IFT172 | 0.565275868 | 0.930020145 | 1.0607854 | down | mRNA |
| NM_015665 | AAAS | 0.764670228 | 0.97415819 | 1.0291864 | down | mRNA |
| NM_015676 | TMEM251 | 0.079543168 | 0.482639336 | 1.2629106 | down | mRNA |
| NM_015677 | SH3YL1 | 0.082693697 | 0.490755473 | 1.1973534 | down | mRNA |
| NM_015679 | TRUB2 | 0.009182852 | 0.203383174 | 1.1914736 | down | mRNA |
| NM_015684 | ATP5S | 0.903176044 | 0.99508468 | 1.0174307 | down | mRNA |
| NM_015690 | STK36 | 0.382979998 | 0.853514109 | 1.3390569 | down | mRNA |
| NM_015693 | INTU | 0.067437846 | 0.451808679 | 1.6029953 | down | mRNA |
| NM_015696 | GPX7 | 0.756280493 | 0.97167109 | 1.1082034 | down | mRNA |
| NM_015698 | GPKOW | 0.580621674 | 0.93393768 | 1.0544042 | down | mRNA |
| NM_015701 | ERLEC1 | 0.340987985 | 0.829004868 | 1.1293582 | down | mRNA |
| NM_015702 | MMADHC | 0.482947571 | 0.902654749 | 1.0637742 | down | mRNA |
| NM_015713 | RRM2B | 0.634619807 | 0.951279916 | 1.058785 | down | mRNA |
| NM_015717 | CD207 | 0.173737618 | 0.651863583 | 1.25414 | down | mRNA |
| NM_015721 | GEMIN4 | 0.381765238 | 0.853514109 | 1.0557598 | down | mRNA |
| NM_015722 | CALY | 0.941552754 | 0.996710991 | 1.0053951 | down | mRNA |
| NM_015832 | MBD2 | 0.031208234 | 0.340775376 | 1.1762638 | down | mRNA |
| NM_015834 | ADARB1 | 0.267088338 | 0.76755337 | 1.4938662 | down | mRNA |
| NM_015842 | LMO7 | 0.262440683 | 0.763125731 | 1.3411439 | down | mRNA |
| NM_015844 | MBD1 | 0.893733872 | 0.993978977 | 1.0637538 | down | mRNA |
| NM_015845 | MBD1 | 0.33016789 | 0.821769369 | 1.8611207 | down | mRNA |
| NM_015847 | MBD1 | 0.189937313 | 0.675909261 | 1.1976407 | down | mRNA |
| NM_015852 | ZNF117 | 0.000352025 | 0.057900793 | 1.372647 | down | mRNA |
| NM_015871 | ZNF593 | 0.106247048 | 0.539783854 | 1.0544731 | down | mRNA |
| NM_015884 | MBTPS2 | 0.819656401 | 0.982331503 | 1.0165611 | down | mRNA |
| NM_015885 | PCF11 | 0.305767652 | 0.801349305 | 1.1968779 | down | mRNA |
| NM_015889 | MED15 | 0.394495915 | 0.861165014 | 1.0684221 | down | mRNA |
| NM_015891 | CDC40 | 0.004110245 | 0.146933952 | 1.2229718 | down | mRNA |
| NM_015895 | GMNN | 0.851767744 | 0.987678166 | 1.0554169 | down | mRNA |
| NM_015897 | PIAS4 | 0.072318686 | 0.463255488 | 1.2439089 | down | mRNA |
| NM_015900 | PLA1A | 0.671541157 | 0.959531882 | 1.0399224 | down | mRNA |
| NM_015902 | UBR5 | 0.112676924 | 0.55538204 | 1.2255701 | down | mRNA |
| NM_015904 | EIF5B | 0.843036009 | 0.987338076 | 1.0227449 | down | mRNA |
| NM_015908 | SRRT | 0.764924036 | 0.97415819 | 1.0659812 | down | mRNA |
| NM_015909 | NBAS | 0.006124454 | 0.17839885 | 1.1674672 | down | mRNA |
| NM_015910 | WDPCP | 0.454818644 | 0.890969522 | 1.0890718 | down | mRNA |
| NM_015911 | ZNF691 | 0.257879812 | 0.75845687 | 1.1840723 | down | mRNA |
| NM_015913 | TXNDC12 | 0.037314972 | 0.36076463 | 1.1303908 | down | mRNA |
| NM_015914 | TXNDC11 | 0.368057099 | 0.845288256 | 1.0642846 | down | mRNA |
| NM_015915 | ATL1 | 0.066539358 | 0.450298982 | 1.1665358 | down | mRNA |
| NM_015916 | CALHM2 | 0.009536271 | 0.204959264 | 1.200096 | down | mRNA |
| NM_015918 | POP5 | 0.794607738 | 0.979378567 | 1.0193661 | down | mRNA |
| NM_015919 | ZNF226 | 0.789123292 | 0.977807732 | 1.0429444 | down | mRNA |
| NM_015920 | RPS27L | 0.981535944 | 0.998971807 | 1.0038478 | down | mRNA |
| NM_015922 | NSDHL | 0.098328397 | 0.521707362 | 1.1658432 | down | mRNA |
| NM_015933 | TMA7 | 0.320797075 | 0.814711917 | 1.0568608 | down | mRNA |
| NM_015934 | NOP58 | 0.971980994 | 0.998501532 | 1.0028228 | down | mRNA |
| NM_015935 | METTL13 | 0.075493912 | 0.471940255 | 1.1664033 | down | mRNA |
| NM_015938 | NMD3 | 0.040822155 | 0.375054703 | 1.1832498 | down | mRNA |
| NM_015939 | TRMT6 | 0.384367903 | 0.85485898 | 1.0486673 | down | mRNA |
| NM_015942 | MTERFD1 | 0.011228531 | 0.217427139 | 1.2180897 | down | mRNA |
| NM_015944 | AMDHD2 | 0.72431307 | 0.966191059 | 1.0504721 | down | mRNA |
| NM_015945 | SLC35C2 | 0.544840949 | 0.924012779 | 1.0852086 | down | mRNA |
| NM_015946 | PELO | 0.000668211 | 0.076919551 | 1.6935164 | down | mRNA |
| NM_015948 | SLC35B3 | 0.210249187 | 0.700954461 | 1.0957458 | down | mRNA |
| NM_015949 | GET4 | 0.454400567 | 0.890473771 | 1.1586928 | down | mRNA |
| NM_015950 | MRPL2 | 0.01320984 | 0.234704726 | 1.2051414 | down | mRNA |
| NM_015952 | RWDD1 | 0.865232557 | 0.989401142 | 1.0084175 | down | mRNA |
| NM_015953 | NOSIP | 0.032891211 | 0.345072008 | 1.1091332 | down | mRNA |
| NM_015955 | MEMO1 | 0.424710531 | 0.874854499 | 1.1831331 | down | mRNA |
| NM_015957 | APIP | 0.419220342 | 0.872365335 | 1.0659088 | down | mRNA |
| NM_015959 | TMX2 | 0.126882044 | 0.580406758 | 1.0574013 | down | mRNA |
| NM_015960 | CUTC | 0.190618382 | 0.676402188 | 1.067999 | down | mRNA |
| NM_015962 | FCF1 | 0.164415022 | 0.637590354 | 1.1328153 | down | mRNA |
| NM_015963 | THAP4 | 0.13207729 | 0.587843174 | 1.1323638 | down | mRNA |
| NM_015965 | NDUFA13 | 0.671545524 | 0.959531882 | 1.0219427 | down | mRNA |
| NM_015966 | ERGIC3 | 0.79217374 | 0.978996327 | 1.0550256 | down | mRNA |
| NM_015969 | MRPS17 | 0.428380758 | 0.877211962 | 1.072639 | down | mRNA |
| NM_015971 | MRPS7 | 0.641923669 | 0.954332328 | 1.0479547 | down | mRNA |
| NM_015972 | POLR1D | 0.579762838 | 0.933893479 | 1.0338662 | down | mRNA |
| NM_015976 | SNX7 | 0.753285424 | 0.970725371 | 1.0650307 | down | mRNA |
| NM_015979 | MED23 | 0.609747094 | 0.943053241 | 1.0884246 | down | mRNA |
| NM_015982 | YBX2 | 0.005248449 | 0.165444121 | 1.1165508 | down | mRNA |
| NM_015983 | UBE2D4 | 0.952328829 | 0.996710991 | 1.0076598 | down | mRNA |
| NM_015984 | UCHL5 | 0.593929715 | 0.938299318 | 1.113836 | down | mRNA |
| NM_015986 | CRLF3 | 0.022512874 | 0.295888941 | 1.1852045 | down | mRNA |
| NM_015987 | HEBP1 | 0.769077866 | 0.975270932 | 1.0630086 | down | mRNA |
| NM_015994 | ATP6V1D | 0.686927115 | 0.960205549 | 1.0325042 | down | mRNA |
| NM_015995 | KLF13 | 0.402014876 | 0.864786348 | 1.2638264 | down | mRNA |
| NM_015997 | RRNAD1 | 0.758585095 | 0.972072638 | 1.0295143 | down | mRNA |
| NM_015999 | ADIPOR1 | 0.242579755 | 0.740802161 | 1.0975689 | down | mRNA |
| NM_016001 | UTP18 | 0.00772992 | 0.19345927 | 1.3285956 | down | mRNA |
| NM_016002 | SCCPDH | 0.299962706 | 0.795797471 | 1.0742842 | down | mRNA |
| NM_016003 | WIPI2 | 0.148654416 | 0.61203168 | 1.0752511 | down | mRNA |
| NM_016004 | IFT52 | 0.912756821 | 0.995342392 | 1.0262783 | down | mRNA |
| NM_016006 | ABHD5 | 0.234220486 | 0.731403918 | 1.2238747 | down | mRNA |
| NM_016009 | SH3GLB1 | 0.504014368 | 0.910568383 | 1.0637939 | down | mRNA |
| NM_016010 | ZC2HC1A | 0.01922153 | 0.27881743 | 1.257919 | down | mRNA |
| NM_016011 | MECR | 0.127224554 | 0.580546411 | 1.0995525 | down | mRNA |
| NM_016013 | NDUFAF1 | 0.323710378 | 0.816213518 | 1.0666627 | down | mRNA |
| NM_016014 | FAM108B1 | 0.051841607 | 0.41031217 | 1.4983279 | down | mRNA |
| NM_016016 | SLC25A39 | 0.015954005 | 0.254213821 | 1.1084414 | down | mRNA |
| NM_016018 | PHF20L1 | 0.104913056 | 0.535979888 | 1.2137795 | down | mRNA |
| NM_016019 | LUC7L2 | 0.002560769 | 0.120522127 | 1.1705635 | down | mRNA |
| NM_016020 | TFB1M | 0.249283703 | 0.747319103 | 1.0595155 | down | mRNA |
| NM_016022 | APH1A | 0.362383724 | 0.843173209 | 1.0816319 | down | mRNA |
| NM_016025 | METTL9 | 0.386332229 | 0.855747763 | 1.0984609 | down | mRNA |
| NM_016026 | RDH11 | 0.121626698 | 0.573155624 | 1.1316611 | down | mRNA |
| NM_016027 | LACTB2 | 0.64725548 | 0.95550188 | 1.0371841 | down | mRNA |
| NM_016028 | SUV420H1 | 0.061722073 | 0.438481196 | 1.225603 | down | mRNA |
| NM_016029 | DHRS7 | 0.568823265 | 0.93232103 | 1.0796517 | down | mRNA |
| NM_016030 | TRAPPC12 | 0.897209683 | 0.994222243 | 1.0202693 | down | mRNA |
| NM_016033 | FAM82B | 0.864014635 | 0.989401142 | 1.0142047 | down | mRNA |
| NM_016034 | MRPS2 | 0.145601783 | 0.607496425 | 1.0928763 | down | mRNA |
| NM_016035 | COQ4 | 0.443275596 | 0.884425653 | 1.0488769 | down | mRNA |
| NM_016037 | UTP11L | 0.183053777 | 0.666166668 | 1.1350086 | down | mRNA |
| NM_016038 | SBDS | 0.347835095 | 0.834082222 | 1.0439303 | down | mRNA |
| NM_016039 | C14orf166 | 0.313242575 | 0.808636478 | 1.0577668 | down | mRNA |
| NM_016040 | TMED5 | 0.391395627 | 0.85910136 | 1.0575115 | down | mRNA |
| NM_016041 | DERL2 | 0.233185913 | 0.731100552 | 1.064292 | down | mRNA |
| NM_016042 | EXOSC3 | 0.041674511 | 0.377943419 | 1.2152794 | down | mRNA |
| NM_016044 | FAHD2A | 0.056719989 | 0.424322651 | 1.2337272 | down | mRNA |
| NM_016045 | SLMO2 | 0.519148943 | 0.916145597 | 1.0741201 | down | mRNA |
| NM_016046 | EXOSC1 | 0.404985864 | 0.866165103 | 1.0548549 | down | mRNA |
| NM_016047 | SF3B14 | 0.313174809 | 0.808636478 | 1.0746131 | down | mRNA |
| NM_016048 | ISOC1 | 0.976414667 | 0.998501532 | 1.0014955 | down | mRNA |
| NM_016055 | MRPL48 | 0.574125263 | 0.933656777 | 1.0384063 | down | mRNA |
| NM_016058 | TPRKB | 0.965999511 | 0.99793927 | 1.0075618 | down | mRNA |
| NM_016061 | YPEL5 | 0.576476037 | 0.933656777 | 1.1196729 | down | mRNA |
| NM_016067 | MRPS18C | 0.586046099 | 0.935061546 | 1.0637376 | down | mRNA |
| NM_016072 | GOLT1B | 0.684582961 | 0.960111041 | 1.0106539 | down | mRNA |
| NM_016073 | HDGFRP3 | 0.275005876 | 0.774827385 | 1.1282529 | down | mRNA |
| NM_016074 | BOLA1 | 0.048717334 | 0.400696128 | 1.1121489 | down | mRNA |
| NM_016075 | VPS36 | 0.574893304 | 0.933656777 | 1.0549663 | down | mRNA |
| NM_016076 | DESI2 | 0.337636711 | 0.827187565 | 1.081586 | down | mRNA |
| NM_016077 | PTRH2 | 0.541536326 | 0.923577434 | 1.0476949 | down | mRNA |
| NM_016078 | FAM18B1 | 0.376809972 | 0.85040519 | 1.0858476 | down | mRNA |
| NM_016085 | ATRAID | 0.842870398 | 0.987281999 | 1.026449 | down | mRNA |
| NM_016086 | STYXL1 | 0.996555151 | 0.999872237 | 1.0003754 | down | mRNA |
| NM_016087 | WNT16 | 0.16385487 | 0.63628397 | 1.3061428 | down | mRNA |
| NM_016090 | RBM7 | 0.363743859 | 0.843238468 | 1.1056691 | down | mRNA |
| NM_016091 | EIF3L | 0.737108657 | 0.968834005 | 1.0907093 | down | mRNA |
| NM_016093 | RPL26L1 | 0.649307672 | 0.95550188 | 1.0349025 | down | mRNA |
| NM_016095 | GINS2 | 0.983865261 | 0.998971807 | 1.0068383 | down | mRNA |
| NM_016097 | IER3IP1 | 0.811083964 | 0.98118927 | 1.0256049 | down | mRNA |
| NM_016100 | NAA20 | 0.94985843 | 0.996710991 | 1.0030624 | down | mRNA |
| NM_016101 | NIP7 | 0.321798458 | 0.815271211 | 1.0806348 | down | mRNA |
| NM_016102 | TRIM17 | 0.967058471 | 0.99793927 | 1.0015411 | down | mRNA |
| NM_016103 | SAR1B | 0.523717726 | 0.917239107 | 1.0663654 | down | mRNA |
| NM_016104 | RWDD1 | 0.801726738 | 0.98118927 | 1.0156624 | down | mRNA |
| NM_016106 | SCFD1 | 0.075182252 | 0.470919763 | 1.1455063 | down | mRNA |
| NM_016108 | AIG1 | 0.925418296 | 0.995465766 | 1.0473812 | down | mRNA |
| NM_016115 | ASB3 | 0.146657509 | 0.608414707 | 1.0638071 | down | mRNA |
| NM_016120 | RLIM | 0.007647719 | 0.193257016 | 1.2132228 | down | mRNA |
| NM_016121 | KCTD3 | 0.111321314 | 0.552449342 | 1.1194043 | down | mRNA |
| NM_016122 | CCDC41 | 0.06558262 | 0.447458331 | 1.1288577 | down | mRNA |
| NM_016124 | RHD | 0.625255566 | 0.948832915 | 1.0899339 | down | mRNA |
| NM_016125 | RNFT1 | 0.145834451 | 0.607759658 | 1.1463923 | down | mRNA |
| NM_016126 | HSPB11 | 0.906443621 | 0.99508468 | 1.0199817 | down | mRNA |
| NM_016127 | TMEM66 | 0.294563287 | 0.791301949 | 1.1755745 | down | mRNA |
| NM_016129 | COPS4 | 0.855514717 | 0.988178307 | 1.0120635 | down | mRNA |
| NM_016131 | RAB10 | 0.381286006 | 0.853514109 | 1.110842 | down | mRNA |
| NM_016133 | INSIG2 | 0.636347755 | 0.951899835 | 1.047609 | down | mRNA |
| NM_016142 | HSD17B12 | 0.712399647 | 0.966072854 | 1.0374639 | down | mRNA |
| NM_016144 | COMMD10 | 0.363553631 | 0.843238468 | 1.0778928 | down | mRNA |
| NM_016146 | TRAPPC4 | 0.2002932 | 0.68900633 | 1.3315508 | down | mRNA |
| NM_016147 | PPME1 | 0.358104672 | 0.840469144 | 1.1162339 | down | mRNA |
| NM_016150 | ASB2 | 0.981068015 | 0.998971807 | 1.0024481 | down | mRNA |
| NM_016151 | TAOK2 | 0.851573487 | 0.987678166 | 1.02924 | down | mRNA |
| NM_016153 | HSFX1 | 0.064006436 | 0.442124523 | 1.3037434 | down | mRNA |
| NM_016155 | MMP17 | 0.125235679 | 0.578441772 | 1.2427391 | down | mRNA |
| NM_016156 | MTMR2 | 0.434730344 | 0.881074297 | 1.0515346 | down | mRNA |
| NM_016162 | ING4 | 0.356915954 | 0.839423386 | 1.1595692 | down | mRNA |
| NM_016166 | PIAS1 | 0.006251456 | 0.179020559 | 1.4636791 | down | mRNA |
| NM_016169 | SUFU | 0.696606101 | 0.962410025 | 1.0604193 | down | mRNA |
| NM_016173 | HEMK1 | 0.386358272 | 0.855747763 | 1.0412622 | down | mRNA |
| NM_016174 | CERCAM | 0.266305243 | 0.767064488 | 1.1162742 | down | mRNA |
| NM_016175 | C5orf45 | 0.443430804 | 0.884425653 | 1.123063 | down | mRNA |
| NM_016176 | SDF4 | 0.441056697 | 0.883920737 | 1.085446 | down | mRNA |
| NM_016183 | MRTO4 | 0.523291613 | 0.917239107 | 1.0754164 | down | mRNA |
| NM_016185 | HN1 | 0.941421408 | 0.996710991 | 1.019519 | down | mRNA |
| NM_016186 | SERPINA10 | 0.728079728 | 0.966191059 | 1.0506217 | down | mRNA |
| NM_016195 | KIF20B | 0.642820831 | 0.954490115 | 1.2440551 | down | mRNA |
| NM_016199 | LSM7 | 0.458050796 | 0.892646883 | 1.0374394 | down | mRNA |
| NM_016200 | NAA38 | 0.487042112 | 0.903621334 | 1.0884776 | down | mRNA |
| NM_016202 | ZNF580 | 0.685976148 | 0.960205549 | 1.0637568 | down | mRNA |
| NM_016205 | PDGFC | 0.302189156 | 0.797522056 | 1.1186558 | down | mRNA |
| NM_016206 | VGLL3 | 0.877456158 | 0.991306146 | 1.1145919 | down | mRNA |
| NM_016207 | CPSF3 | 0.286080464 | 0.782746332 | 1.0628864 | down | mRNA |
| NM_016209 | TRAPPC2L | 0.309446818 | 0.803961878 | 1.1196105 | down | mRNA |
| NM_016211 | SEC31A | 0.12700378 | 0.580406758 | 1.2772082 | down | mRNA |
| NM_016216 | DBR1 | 0.200723839 | 0.68900633 | 1.1445839 | down | mRNA |
| NM_016217 | HECA | 0.280634322 | 0.779126326 | 1.3173061 | down | mRNA |
| NM_016218 | POLK | 0.053627737 | 0.416065637 | 1.1923005 | down | mRNA |
| NM_016221 | DCTN4 | 0.588670142 | 0.93579682 | 1.0470456 | down | mRNA |
| NM_016222 | DDX41 | 0.001464713 | 0.098148183 | 1.1457352 | down | mRNA |
| NM_016224 | SNX9 | 0.163446803 | 0.635775857 | 1.1667979 | down | mRNA |
| NM_016228 | AADAT | 0.031793635 | 0.341957288 | 1.1865779 | down | mRNA |
| NM_016230 | CYB5R4 | 0.142512674 | 0.602652383 | 1.1450857 | down | mRNA |
| NM_016231 | NLK | 0.652023655 | 0.956271055 | 1.0635364 | down | mRNA |
| NM_016234 | ACSL5 | 0.743284725 | 0.970330526 | 1.0534628 | down | mRNA |
| NM_016235 | GPRC5B | 0.199059891 | 0.687651648 | 2.174922 | down | mRNA |
| NM_016240 | SCARA3 | 0.570479952 | 0.93296476 | 1.055079 | down | mRNA |
| NM_016243 | CYB5R1 | 0.538799142 | 0.923577434 | 1.0360563 | down | mRNA |
| NM_016245 | HSD17B11 | 0.624031072 | 0.948341211 | 1.0345461 | down | mRNA |
| NM_016250 | NDRG2 | 0.385178056 | 0.855147437 | 1.1275316 | down | mRNA |
| NM_016252 | BIRC6 | 0.029470618 | 0.333953485 | 1.2976075 | down | mRNA |
| NM_016255 | FAM8A1 | 0.047999801 | 0.398338064 | 1.1155642 | down | mRNA |
| NM_016256 | NAGPA | 0.143754238 | 0.604261149 | 1.0530406 | down | mRNA |
| NM_016257 | HPCAL4 | 0.021681643 | 0.29025889 | 1.3034975 | down | mRNA |
| NM_016258 | YTHDF2 | 0.485313041 | 0.903250293 | 1.0641592 | down | mRNA |
| NM_016261 | TUBD1 | 0.178676692 | 0.65979922 | 1.1236928 | down | mRNA |
| NM_016264 | ZNF44 | 0.050898789 | 0.407751844 | 1.2575883 | down | mRNA |
| NM_016265 | ZNF12 | 0.170088493 | 0.646926635 | 1.2658453 | down | mRNA |
| NM_016270 | KLF2 | 0.700470661 | 0.962991468 | 1.0936566 | down | mRNA |
| NM_016272 | TOB2 | 0.585792243 | 0.934949987 | 1.1024582 | down | mRNA |
| NM_016274 | PLEKHO1 | 0.061245433 | 0.437893235 | 1.3870864 | down | mRNA |
| NM_016275 | SELT | 0.288506628 | 0.784514546 | 1.0404156 | down | mRNA |
| NM_016277 | RAB23 | 0.632256422 | 0.95088672 | 1.2179954 | down | mRNA |
| NM_016282 | AK3 | 0.635563133 | 0.951814727 | 1.0208681 | down | mRNA |
| NM_016284 | CNOT1 | 0.919425774 | 0.995349793 | 1.009379 | down | mRNA |
| NM_016287 | HP1BP3 | 0.053641929 | 0.416065637 | 1.1663521 | down | mRNA |
| NM_016289 | CAB39 | 0.805806393 | 0.98118927 | 1.0575371 | down | mRNA |
| NM_016291 | IP6K2 | 0.311744918 | 0.806920827 | 1.0823958 | down | mRNA |
| NM_016297 | PCYOX1 | 0.983504627 | 0.998971807 | 1.0031897 | down | mRNA |
| NM_016299 | HSPA14 | 0.428661967 | 0.877386753 | 1.1943074 | down | mRNA |
| NM_016301 | GPN3 | 0.513975323 | 0.91350662 | 1.1326114 | down | mRNA |
| NM_016302 | CRBN | 0.066134904 | 0.449693785 | 1.1798837 | down | mRNA |
| NM_016306 | DNAJB11 | 0.259072381 | 0.759547173 | 1.1032976 | down | mRNA |
| NM_016307 | PRRX2 | 0.558406133 | 0.926833616 | 1.1120629 | down | mRNA |
| NM_016309 | LCMT1 | 0.832580197 | 0.98623676 | 1.0202476 | down | mRNA |
| NM_016311 | ATPIF1 | 0.898371125 | 0.994375399 | 1.0139175 | down | mRNA |
| NM_016312 | WBP11 | 0.919771142 | 0.995349793 | 1.0072821 | down | mRNA |
| NM_016315 | GULP1 | 0.545311922 | 0.924012779 | 1.0238429 | down | mRNA |
| NM_016316 | REV1 | 0.006677217 | 0.183552613 | 1.355353 | down | mRNA |
| NM_016318 | P2RX2 | 0.887020992 | 0.993099104 | 1.0396398 | down | mRNA |
| NM_016322 | RAB14 | 0.020304914 | 0.285032405 | 1.2282616 | down | mRNA |
| NM_016324 | ZNF274 | 0.906309975 | 0.99508468 | 1.0247861 | down | mRNA |
| NM_016325 | ZNF274 | 0.473163155 | 0.899184677 | 1.189877 | down | mRNA |
| NM_016326 | CKLF | 0.984728496 | 0.998971807 | 1.0021374 | down | mRNA |
| NM_016328 | GTF2IRD1 | 0.081156311 | 0.486746344 | 1.4790793 | down | mRNA |
| NM_016329 | SFMBT1 | 0.056173055 | 0.422065984 | 1.3122859 | down | mRNA |
| NM_016331 | ZNF639 | 0.000329755 | 0.056781055 | 1.5731023 | down | mRNA |
| NM_016332 | MSRB1 | 0.218230681 | 0.712269655 | 1.1497809 | down | mRNA |
| NM_016334 | GPR89B | 0.048597988 | 0.400524645 | 1.1953659 | down | mRNA |
| NM_016335 | PRODH | 0.749681433 | 0.970725371 | 1.0709405 | down | mRNA |
| NM_016338 | IPO11 | 0.199472261 | 0.688451878 | 1.1182525 | down | mRNA |
| NM_016343 | CENPF | 0.660809364 | 0.958354006 | 1.3083113 | down | mRNA |
| NM_016347 | NAT8B | 0.124977821 | 0.578441772 | 1.3501503 | down | mRNA |
| NM_016352 | CPA4 | 0.792331607 | 0.978996327 | 1.0720368 | down | mRNA |
| NM_016353 | ZDHHC2 | 0.744470783 | 0.970330526 | 1.0468741 | down | mRNA |
| NM_016359 | NUSAP1 | 0.88866116 | 0.993440915 | 1.0746948 | down | mRNA |
| NM_016364 | DUSP13 | 0.130181778 | 0.584037049 | 1.5277632 | down | mRNA |
| NM_016366 | CABP2 | 0.395177229 | 0.861590143 | 1.2740711 | down | mRNA |
| NM_016369 | CLDN18 | 0.746326912 | 0.970394407 | 1.0465548 | down | mRNA |
| NM_016371 | HSD17B7 | 0.829057906 | 0.98552522 | 1.0840952 | down | mRNA |
| NM_016373 | WWOX | 0.37568876 | 0.849862269 | 1.1336681 | down | mRNA |
| NM_016376 | ANKFY1 | 0.409181863 | 0.867591329 | 6.2906781 | down | mRNA |
| NM_016377 | AKAP7 | 0.368574538 | 0.845559574 | 1.0604712 | down | mRNA |
| NM_016391 | NOP16 | 0.17048021 | 0.64730412 | 1.1501892 | down | mRNA |
| NM_016395 | PTPLAD1 | 0.262556556 | 0.763125731 | 1.0970511 | down | mRNA |
| NM_016396 | CTDSPL2 | 0.092399073 | 0.511338764 | 1.2790954 | down | mRNA |
| NM_016400 | C15orf63 | 0.983288685 | 0.998971807 | 1.0029723 | down | mRNA |
| NM_016403 | CWC15 | 0.307370258 | 0.802168809 | 1.0800225 | down | mRNA |
| NM_016404 | TRMT112 | 0.947650314 | 0.996710991 | 1.0035179 | down | mRNA |
| NM_016406 | UFC1 | 0.497442307 | 0.908789022 | 1.0480375 | down | mRNA |
| NM_016407 | C20orf43 | 0.375890772 | 0.849862269 | 1.0970015 | down | mRNA |
| NM_016408 | CDK5RAP1 | 0.562792113 | 0.929041598 | 1.1465321 | down | mRNA |
| NM_016417 | GLRX5 | 0.134279648 | 0.590871682 | 1.0614914 | down | mRNA |
| NM_016422 | RNF141 | 0.589281212 | 0.936090876 | 1.053325 | down | mRNA |
| NM_016424 | LUC7L3 | 0.013342298 | 0.23544335 | 1.2509022 | down | mRNA |
| NM_016429 | COPZ2 | 0.034367544 | 0.351431016 | 1.1379175 | down | mRNA |
| NM_016431 | MAPK8IP2 | 0.57016402 | 0.932853855 | 1.1080951 | down | mRNA |
| NM_016436 | PHF20 | 0.047393864 | 0.397201997 | 1.2224463 | down | mRNA |
| NM_016441 | CRIM1 | 0.467687904 | 0.896909118 | 1.1773253 | down | mRNA |
| NM_016446 | TMEM8B | 0.967387482 | 0.99793927 | 1.0061239 | down | mRNA |
| NM_016447 | MPP6 | 0.009837813 | 0.206905383 | 1.1565626 | down | mRNA |
| NM_016448 | DTL | 0.424467219 | 0.874854499 | 1.0836094 | down | mRNA |
| NM_016453 | NCKIPSD | 0.701448731 | 0.963118287 | 1.075981 | down | mRNA |
| NM_016463 | CXXC5 | 0.043329423 | 0.384201411 | 1.3839988 | down | mRNA |
| NM_016464 | TMEM138 | 0.058377742 | 0.429582432 | 1.1609398 | down | mRNA |
| NM_016466 | ANKRD39 | 0.446387398 | 0.886582845 | 1.1017273 | down | mRNA |
| NM_016467 | ORMDL1 | 0.790835747 | 0.978565927 | 1.0597407 | down | mRNA |
| NM_016468 | COX16 | 0.884045689 | 0.992766819 | 1.0168941 | down | mRNA |
| NM_016472 | C14orf129 | 0.031230157 | 0.340775376 | 1.1930781 | down | mRNA |
| NM_016474 | C3orf19 | 0.159143457 | 0.630864648 | 1.2592243 | down | mRNA |
| NM_016475 | JKAMP | 0.072745361 | 0.463915802 | 1.2310228 | down | mRNA |
| NM_016479 | SHISA5 | 0.922323933 | 0.995349793 | 1.0183865 | down | mRNA |
| NM_016481 | C9orf156 | 0.143583333 | 0.603870418 | 1.1793903 | down | mRNA |
| NM_016485 | VTA1 | 0.104037923 | 0.533896783 | 1.1889831 | down | mRNA |
| NM_016486 | TMEM69 | 0.162794574 | 0.635098861 | 1.2724346 | down | mRNA |
| NM_016488 | PPHLN1 | 0.839129354 | 0.987032229 | 1.0237123 | down | mRNA |
| NM_016489 | NT5C3 | 0.193994561 | 0.681590151 | 1.2001724 | down | mRNA |
| NM_016491 | MRPL37 | 0.013677126 | 0.236063947 | 1.0763722 | down | mRNA |
| NM_016494 | RNF181 | 0.370249674 | 0.846215286 | 1.0933849 | down | mRNA |
| NM_016497 | MRPL51 | 0.868553535 | 0.989481868 | 1.0140619 | down | mRNA |
| NM_016498 | MTFP1 | 0.854982009 | 0.988178307 | 1.0288722 | down | mRNA |
| NM_016504 | MRPL27 | 0.778225106 | 0.976938981 | 1.0222988 | down | mRNA |
| NM_016506 | KBTBD4 | 0.00087662 | 0.08328711 | 1.5498728 | down | mRNA |
| NM_016510 | SCLY | 0.346875068 | 0.833567116 | 1.2702071 | down | mRNA |
| NM_016516 | VPS54 | 0.199322316 | 0.688167995 | 1.0985086 | down | mRNA |
| NM_016519 | AMBN | 0.772003214 | 0.975347132 | 1.0235588 | down | mRNA |
| NM_016520 | C9orf78 | 0.020776148 | 0.286581173 | 1.0721831 | down | mRNA |
| NM_016522 | NTM | 0.442322087 | 0.88419144 | 1.1033218 | down | mRNA |
| NM_016530 | RAB8B | 0.942566868 | 0.996710991 | 1.0182711 | down | mRNA |
| NM_016533 | NINJ2 | 0.043664441 | 0.385351177 | 1.1061063 | down | mRNA |
| NM_016538 | SIRT7 | 0.211503354 | 0.702875483 | 1.2443088 | down | mRNA |
| NM_016547 | SDF4 | 0.391077872 | 0.858960277 | 1.0916989 | down | mRNA |
| NM_016556 | PSMC3IP | 0.810150418 | 0.98118927 | 1.0912575 | down | mRNA |
| NM_016558 | SCAND1 | 0.730153723 | 0.966191059 | 1.012049 | down | mRNA |
| NM_016561 | BFAR | 0.006034292 | 0.176785634 | 1.2573568 | down | mRNA |
| NM_016564 | CEND1 | 0.55444685 | 0.926549129 | 1.0636789 | down | mRNA |
| NM_016565 | CHCHD8 | 0.948189047 | 0.996710991 | 1.006816 | down | mRNA |
| NM_016567 | BCCIP | 0.719203736 | 0.966191059 | 1.0452975 | down | mRNA |
| NM_016571 | LGSN | 0.884098111 | 0.992766819 | 1.0432885 | down | mRNA |
| NM_016576 | GMPR2 | 0.762334778 | 0.97339948 | 1.0195664 | down | mRNA |
| NM_016577 | RAB6B | 0.654222246 | 0.957205183 | 1.1043784 | down | mRNA |
| NM_016578 | RSF1 | 0.245293158 | 0.743589595 | 1.1832234 | down | mRNA |
| NM_016581 | ECSIT | 0.688741414 | 0.960854291 | 1.0177098 | down | mRNA |
| NM_016586 | MBIP | 0.001429905 | 0.09664422 | 1.387774 | down | mRNA |
| NM_016589 | TIMMDC1 | 0.941548136 | 0.996710991 | 1.007651 | down | mRNA |
| NM_016602 | CCR10 | 0.168060139 | 0.642954036 | 1.1869799 | down | mRNA |
| NM_016603 | FAM13B | 0.159882968 | 0.631698386 | 1.151929 | down | mRNA |
| NM_016608 | ARMCX1 | 0.108323672 | 0.544895409 | 1.0931846 | down | mRNA |
| NM_016613 | FAM198B | 0.862365042 | 0.989401142 | 1.1128021 | down | mRNA |
| NM_016615 | SLC6A13 | 0.690703458 | 0.961105466 | 1.0321319 | down | mRNA |
| NM_016616 | NME8 | 0.003707334 | 0.141421215 | 1.7022133 | down | mRNA |
| NM_016620 | ZNF644 | 0.253506603 | 0.753559682 | 1.1276969 | down | mRNA |
| NM_016621 | PHF21A | 0.123875371 | 0.577744894 | 1.2427792 | down | mRNA |
| NM_016623 | FAM49B | 0.052842961 | 0.413038171 | 1.2059106 | down | mRNA |
| NM_016625 | RSRC1 | 0.812055169 | 0.98118927 | 1.0199172 | down | mRNA |
| NM_016626 | MEX3C | 0.547123454 | 0.925005327 | 1.6443419 | down | mRNA |
| NM_016628 | WAC | 0.233717666 | 0.73118887 | 1.072519 | down | mRNA |
| NM_016632 | ARL17A | 0.002577872 | 0.120730042 | 1.4302085 | down | mRNA |
| NM_016638 | ARL6IP4 | 0.997837757 | 0.999889342 | 1.0002375 | down | mRNA |
| NM_016640 | MRPS30 | 0.025999844 | 0.315604326 | 1.285359 | down | mRNA |
| NM_016641 | GDE1 | 0.20321441 | 0.692876095 | 1.0776385 | down | mRNA |
| NM_016642 | SPTBN5 | 0.553699422 | 0.926337111 | 1.0920121 | down | mRNA |
| NM_016648 | LARP7 | 0.651886032 | 0.956204521 | 1.0548734 | down | mRNA |
| NM_016651 | DACT1 | 0.000888649 | 0.083478018 | 2.7252009 | down | mRNA |
| NM_016653 | ZAK | 0.734975672 | 0.967907596 | 1.0556784 | down | mRNA |
| NM_016657 | KDELR3 | 0.480659448 | 0.901538317 | 1.1183667 | down | mRNA |
| NM_016732 | RALY | 0.242036714 | 0.740570425 | 1.0780893 | down | mRNA |
| NM_016733 | LIMK2 | 0.399253144 | 0.863703388 | 1.1716432 | down | mRNA |
| NM_016815 | GYPC | 0.379943529 | 0.853273195 | 1.0689803 | down | mRNA |
| NM_016816 | OAS1 | 0.983119438 | 0.998971807 | 1.0225187 | down | mRNA |
| NM_016819 | OGG1 | 0.404167978 | 0.865617032 | 1.0858292 | down | mRNA |
| NM_016820 | OGG1 | 0.461547944 | 0.894667841 | 1.0797677 | down | mRNA |
| NM_016821 | OGG1 | 0.222602574 | 0.717433439 | 1.216262 | down | mRNA |
| NM_016823 | CRK | 0.162639564 | 0.63494386 | 1.1992531 | down | mRNA |
| NM_016824 | ADD3 | 0.125533547 | 0.578441772 | 1.4138144 | down | mRNA |
| NM_016826 | OGG1 | 0.976116279 | 0.998501532 | 1.0036159 | down | mRNA |
| NM_016827 | OGG1 | 0.906907642 | 0.995145546 | 1.0204305 | down | mRNA |
| NM_016829 | OGG1 | 0.984955757 | 0.998971807 | 1.0051021 | down | mRNA |
| NM_016830 | VAMP1 | 0.638673248 | 0.953122601 | 1.0858868 | down | mRNA |
| NM_016836 | RBMS1 | 0.508317531 | 0.911674633 | 1.0447176 | down | mRNA |
| NM_016930 | STX18 | 0.108325165 | 0.544895409 | 1.1688834 | down | mRNA |
| NM_016932 | SIX2 | 0.352497801 | 0.835946719 | 1.3087672 | down | mRNA |
| NM_016936 | UBN1 | 0.026985345 | 0.319549234 | 1.3252924 | down | mRNA |
| NM_016938 | EFEMP2 | 0.616216812 | 0.945912508 | 1.120611 | down | mRNA |
| NM_016940 | RWDD2B | 0.055239506 | 0.419634622 | 1.0892358 | down | mRNA |
| NM_016941 | DLL3 | 0.998775327 | 0.999897336 | 1.0001026 | down | mRNA |
| NM_016946 | F11R | 0.447702659 | 0.887547822 | 1.1916761 | down | mRNA |
| NM_016948 | PARD6A | 0.117845859 | 0.564437853 | 1.2447224 | down | mRNA |
| NM_016951 | CKLF | 0.753427144 | 0.970725371 | 1.0291225 | down | mRNA |
| NM_016954 | TBX22 | 0.752854293 | 0.970725371 | 1.054773 | down | mRNA |
| NM_017411 | SMN2 | 0.656750879 | 0.957445617 | 1.0975411 | down | mRNA |
| NM_017412 | FZD3 | 0.204996057 | 0.694889593 | 1.2209114 | down | mRNA |
| NM_017414 | USP18 | 0.578926896 | 0.933821303 | 1.1914917 | down | mRNA |
| NM_017420 | SIX4 | 0.634435172 | 0.95127846 | 1.0857348 | down | mRNA |
| NM_017421 | COQ3 | 0.41495231 | 0.870239791 | 1.1003796 | down | mRNA |
| NM_017425 | SPA17 | 0.661021726 | 0.958354006 | 1.0389505 | down | mRNA |
| NM_017426 | NUP54 | 0.775648303 | 0.97589756 | 1.0215353 | down | mRNA |
| NM_017432 | PTOV1 | 0.895381742 | 0.993978977 | 1.0236719 | down | mRNA |
| NM_017434 | DUOX1 | 0.787167273 | 0.977479172 | 1.0209332 | down | mRNA |
| NM_017437 | CPSF2 | 0.232449304 | 0.730317988 | 1.0908981 | down | mRNA |
| NM_017438 | SETD4 | 0.329680566 | 0.821768501 | 1.1438112 | down | mRNA |
| NM_017443 | POLE3 | 0.779309358 | 0.976938981 | 1.0857513 | down | mRNA |
| NM_017451 | BAIAP2 | 0.598935043 | 0.940648177 | 1.2026263 | down | mRNA |
| NM_017455 | NPTN | 0.27102275 | 0.770905786 | 1.1822543 | down | mRNA |
| NM_017459 | MFAP2 | 0.78464747 | 0.977411899 | 1.0276602 | down | mRNA |
| NM_017482 | ADD2 | 0.166436021 | 0.641269125 | 1.1547677 | down | mRNA |
| NM_017490 | MARK2 | 0.763929124 | 0.973773636 | 1.051807 | down | mRNA |
| NM_017491 | WDR1 | 0.278248475 | 0.777440204 | 1.1915411 | down | mRNA |
| NM_017503 | SURF2 | 0.177348623 | 0.65723949 | 1.1426754 | down | mRNA |
| NM_017510 | TMED9 | 0.125385745 | 0.578441772 | 1.3087785 | down | mRNA |
| NM_017514 | PLXNA3 | 0.416375334 | 0.870736736 | 1.0649151 | down | mRNA |
| NM_017518 | HAUS7 | 0.5967365 | 0.939585033 | 1.0685233 | down | mRNA |
| NM_017519 | ARID1B | 0.612312615 | 0.944159427 | 1.0380479 | down | mRNA |
| NM_017520 | MPHOSPH8 | 0.047300623 | 0.396911378 | 1.3043757 | down | mRNA |
| NM_017522 | LRP8 | 0.684492791 | 0.960111041 | 1.1808572 | down | mRNA |
| NM_017525 | CDC42BPG | 0.75318316 | 0.970725371 | 1.0578316 | down | mRNA |
| NM_017526 | LEPROT | 0.906947797 | 0.995145546 | 1.0119993 | down | mRNA |
| NM_017527 | LY6K | 0.787439403 | 0.977492986 | 1.032336 | down | mRNA |
| NM_017528 | WBSCR22 | 0.181202667 | 0.664543371 | 1.1724921 | down | mRNA |
| NM_017546 | C2orf29 | 0.080937834 | 0.486296704 | 1.1139153 | down | mRNA |
| NM_017547 | FOXRED1 | 0.360665565 | 0.841780454 | 1.284419 | down | mRNA |
| NM_017552 | ATAD2B | 0.009204722 | 0.203645714 | 1.3417216 | down | mRNA |
| NM_017553 | INO80 | 0.713632547 | 0.966110889 | 1.0685489 | down | mRNA |
| NM_017565 | FAM20A | 0.729907349 | 0.966191059 | 1.1260788 | down | mRNA |
| NM_017569 | FAM48A | 0.005430918 | 0.168942963 | 1.3898245 | down | mRNA |
| NM_017570 | OPLAH | 0.536472028 | 0.922509728 | 1.21242 | down | mRNA |
| NM_017572 | MKNK2 | 0.081663242 | 0.488346186 | 1.1596752 | down | mRNA |
| NM_017579 | DMBT1 | 0.81270049 | 0.98118927 | 1.0190433 | down | mRNA |
| NM_017580 | ZRANB1 | 0.019389106 | 0.280114993 | 1.1251224 | down | mRNA |
| NM_017582 | UBE2Q1 | 0.166009359 | 0.640231845 | 1.0807866 | down | mRNA |
| NM_017583 | TRIM44 | 0.541759455 | 0.923577434 | 1.1072715 | down | mRNA |
| NM_017586 | CACFD1 | 0.334552222 | 0.82457104 | 1.0983885 | down | mRNA |
| NM_017592 | MED29 | 0.934512607 | 0.996710991 | 1.0096286 | down | mRNA |
| NM_017593 | BMP2K | 0.163687201 | 0.636236851 | 1.28036 | down | mRNA |
| NM_017594 | DIRAS2 | 0.361671569 | 0.842615599 | 1.2001586 | down | mRNA |
| NM_017596 | KIF21B | 0.508780811 | 0.911674633 | 1.1215074 | down | mRNA |
| NM_017607 | PPP1R12C | 0.141274247 | 0.601673229 | 1.6451982 | down | mRNA |
| NM_017610 | RNF111 | 0.069183089 | 0.456727607 | 1.2354354 | down | mRNA |
| NM_017612 | ZCCHC8 | 0.225616325 | 0.720696169 | 1.1680155 | down | mRNA |
| NM_017614 | BHMT2 | 0.825248695 | 0.98381451 | 1.1461919 | down | mRNA |
| NM_017615 | NSMCE4A | 0.684166927 | 0.960111041 | 1.0582056 | down | mRNA |
| NM_017617 | NOTCH1 | 0.449323311 | 0.888279993 | 1.1032486 | down | mRNA |
| NM_017619 | RNPC3 | 0.003547828 | 0.138758659 | 1.4880677 | down | mRNA |
| NM_017620 | ILF3 | 0.20544671 | 0.695610743 | 1.1011668 | down | mRNA |
| NM_017621 | ALKBH4 | 0.01715892 | 0.263302002 | 2.3770973 | down | mRNA |
| NM_017622 | C17orf59 | 0.060321702 | 0.435378361 | 1.214579 | down | mRNA |
| NM_017629 | EIF2C4 | 0.03442176 | 0.351513418 | 1.291799 | down | mRNA |
| NM_017632 | CDKN2AIP | 0.145140812 | 0.606482829 | 1.1790368 | down | mRNA |
| NM_017633 | FAM46A | 0.08640743 | 0.49936346 | 1.4072313 | down | mRNA |
| NM_017634 | KCTD9 | 0.905250201 | 0.99508468 | 1.0218029 | down | mRNA |
| NM_017635 | SUV420H1 | 0.046925359 | 0.394576676 | 1.6654359 | down | mRNA |
| NM_017637 | BNC2 | 0.074747515 | 0.469867247 | 1.3223089 | down | mRNA |
| NM_017638 | MED18 | 0.078076262 | 0.480026175 | 1.4388929 | down | mRNA |
| NM_017641 | KIF21A | 0.394629736 | 0.861364659 | 1.1430481 | down | mRNA |
| NM_017644 | KLHL24 | 0.990872516 | 0.999376923 | 1.0044264 | down | mRNA |
| NM_017645 | HAUS6 | 0.808406743 | 0.98118927 | 1.0669771 | down | mRNA |
| NM_017646 | TRIT1 | 0.008953784 | 0.202329498 | 1.2924633 | down | mRNA |
| NM_017652 | ZNF586 | 0.766030386 | 0.974734429 | 1.0205813 | down | mRNA |
| NM_017658 | KLHL28 | 0.486908911 | 0.903606093 | 1.0802489 | down | mRNA |
| NM_017661 | ZNF280D | 0.062685074 | 0.439458294 | 1.4633211 | down | mRNA |
| NM_017666 | ZNF280C | 0.19867618 | 0.687087939 | 1.1990783 | down | mRNA |
| NM_017667 | CCDC132 | 0.002122376 | 0.110028846 | 1.1965741 | down | mRNA |
| NM_017669 | ERCC6L | 0.024558556 | 0.306334086 | 2.3252788 | down | mRNA |
| NM_017670 | OTUB1 | 0.313327077 | 0.808636478 | 1.0500755 | down | mRNA |
| NM_017676 | GIN1 | 0.013464 | 0.235533862 | 1.328925 | down | mRNA |
| NM_017677 | MTMR8 | 0.534837377 | 0.921043919 | 1.1879035 | down | mRNA |
| NM_017679 | BCAS3 | 0.295378955 | 0.79157694 | 1.1889152 | down | mRNA |
| NM_017686 | GDAP2 | 0.029984978 | 0.335897836 | 1.2172195 | down | mRNA |
| NM_017693 | BIVM | 0.347412677 | 0.833659217 | 1.1604907 | down | mRNA |
| NM_017698 | TMEM164 | 0.620010231 | 0.947444797 | 1.1548098 | down | mRNA |
| NM_017699 | SIDT1 | 0.582831899 | 0.934269626 | 1.3110321 | down | mRNA |
| NM_017704 | ANKRD49 | 0.161770905 | 0.633721912 | 1.1710165 | down | mRNA |
| NM_017705 | PAQR5 | 0.236243786 | 0.734460585 | 1.0931136 | down | mRNA |
| NM_017706 | WDR55 | 0.825848618 | 0.984306256 | 1.0578899 | down | mRNA |
| NM_017711 | GDPD2 | 0.082390652 | 0.489943729 | 1.4124177 | down | mRNA |
| NM_017714 | TASP1 | 0.638800518 | 0.953122601 | 1.0644094 | down | mRNA |
| NM_017719 | SNRK | 0.0643584 | 0.443678541 | 1.525332 | down | mRNA |
| NM_017720 | STAP2 | 0.034862185 | 0.352338753 | 1.1641786 | down | mRNA |
| NM_017722 | TRMT1 | 0.694144665 | 0.961951598 | 1.0781756 | down | mRNA |
| NM_017724 | LRRFIP2 | 0.539098464 | 0.923577434 | 1.0734568 | down | mRNA |
| NM_017726 | PPP1R14D | 0.348742659 | 0.834138149 | 1.085942 | down | mRNA |
| NM_017727 | TMEM214 | 0.653465406 | 0.956860606 | 1.0526578 | down | mRNA |
| NM_017728 | TMEM104 | 0.024654141 | 0.306827745 | 1.1697912 | down | mRNA |
| NM_017733 | PIGG | 0.141750976 | 0.60204132 | 1.1205338 | down | mRNA |
| NM_017734 | PALMD | 0.813725569 | 0.98120545 | 1.0691981 | down | mRNA |
| NM_017735 | TTC27 | 0.338102 | 0.827403747 | 1.0978683 | down | mRNA |
| NM_017736 | THUMPD1 | 0.001864927 | 0.105670067 | 1.4827914 | down | mRNA |
| NM_017738 | CNTLN | 0.097524903 | 0.520535504 | 1.1720593 | down | mRNA |
| NM_017739 | POMGNT1 | 0.4826166 | 0.902654749 | 1.0273692 | down | mRNA |
| NM_017740 | ZDHHC7 | 0.017836784 | 0.268635185 | 1.2192305 | down | mRNA |
| NM_017742 | ZCCHC2 | 0.01055873 | 0.210159643 | 1.5079994 | down | mRNA |
| NM_017743 | DPP8 | 0.236740861 | 0.73465251 | 1.1539952 | down | mRNA |
| NM_017744 | ST7L | 0.065807248 | 0.448620218 | 1.1397005 | down | mRNA |
| NM_017746 | TEX10 | 0.261560407 | 0.762395628 | 1.1217888 | down | mRNA |
| NM_017748 | CWC25 | 0.018949991 | 0.276065624 | 1.1603717 | down | mRNA |
| NM_017749 | AMBRA1 | 0.057217223 | 0.426252791 | 1.1706169 | down | mRNA |
| NM_017751 | SMPD4 | 0.674916514 | 0.959531882 | 1.0202209 | down | mRNA |
| NM_017752 | TBC1D8B | 0.054104141 | 0.417214521 | 1.3272579 | down | mRNA |
| NM_017754 | UHRF1BP1 | 0.815425863 | 0.981459009 | 1.0378602 | down | mRNA |
| NM_017755 | NSUN2 | 0.160553017 | 0.632016252 | 1.1184662 | down | mRNA |
| NM_017766 | CASZ1 | 0.268554878 | 0.769158724 | 1.4377593 | down | mRNA |
| NM_017767 | SLC39A4 | 0.268362146 | 0.768988803 | 1.3001363 | down | mRNA |
| NM_017768 | LRRC40 | 0.148031362 | 0.611080968 | 1.1018415 | down | mRNA |
| NM_017774 | CDKAL1 | 0.312999461 | 0.808422897 | 1.1247107 | down | mRNA |
| NM_017775 | TTC19 | 0.134713721 | 0.591704336 | 1.1655338 | down | mRNA |
| NM_017776 | ZNF673 | 0.980490032 | 0.998971807 | 1.0041923 | down | mRNA |
| NM_017779 | DEPDC1 | 0.611595228 | 0.943901183 | 1.3269397 | down | mRNA |
| NM_017784 | OSBPL10 | 0.75527121 | 0.971417904 | 1.0548414 | down | mRNA |
| NM_017785 | CCDC99 | 0.010470299 | 0.209736088 | 1.9389577 | down | mRNA |
| NM_017798 | YTHDF1 | 0.154226107 | 0.621674306 | 1.1652203 | down | mRNA |
| NM_017799 | C14orf101 | 0.159217401 | 0.630911751 | 1.1763936 | down | mRNA |
| NM_017801 | CMTM6 | 0.408759498 | 0.867591329 | 1.1112526 | down | mRNA |
| NM_017803 | DUS2L | 0.01401386 | 0.238634683 | 1.4067273 | down | mRNA |
| NM_017807 | OSGEP | 0.614338654 | 0.944827645 | 1.0626413 | down | mRNA |
| NM_017811 | UBE2R2 | 0.190217713 | 0.676255734 | 1.2920453 | down | mRNA |
| NM_017812 | CHCHD3 | 0.33397322 | 0.824079937 | 1.0816402 | down | mRNA |
| NM_017813 | IMPAD1 | 0.78512962 | 0.977411899 | 1.0337361 | down | mRNA |
| NM_017818 | WRAP73 | 0.199925443 | 0.68900633 | 1.0998775 | down | mRNA |
| NM_017819 | TRMT10C | 0.019176802 | 0.278501956 | 1.1181802 | down | mRNA |
| NM_017820 | EXD3 | 0.111811319 | 0.553934632 | 1.765043 | down | mRNA |
| NM_017822 | KANSL2 | 0.077747107 | 0.479016418 | 1.2866953 | down | mRNA |
| NM_017823 | DUSP23 | 0.745137303 | 0.970378122 | 1.0289369 | down | mRNA |
| NM_017832 | FAM206A | 0.491038426 | 0.90553335 | 1.1248209 | down | mRNA |
| NM_017836 | SLC41A3 | 0.629518335 | 0.950410442 | 1.1120337 | down | mRNA |
| NM_017839 | LPCAT2 | 0.988435386 | 0.999140676 | 1.0009047 | down | mRNA |
| NM_017841 | SDHAF2 | 0.965175371 | 0.99793927 | 1.0037635 | down | mRNA |
| NM_017843 | BCAS4 | 0.646437394 | 0.95550188 | 1.0888912 | down | mRNA |
| NM_017845 | COMMD8 | 0.00360816 | 0.139735427 | 1.1919684 | down | mRNA |
| NM_017846 | TRNAU1AP | 0.684319757 | 0.960111041 | 1.0651943 | down | mRNA |
| NM_017847 | C1orf27 | 0.542578049 | 0.923577434 | 1.0287773 | down | mRNA |
| NM_017851 | PARP16 | 0.216063689 | 0.710711878 | 1.1647643 | down | mRNA |
| NM_017852 | NLRP2 | 0.768277576 | 0.975142015 | 1.1466302 | down | mRNA |
| NM_017856 | GEMIN8 | 0.416895337 | 0.870739638 | 1.0930787 | down | mRNA |
| NM_017857 | SSH3 | 0.175483791 | 0.653962789 | 1.0914159 | down | mRNA |
| NM_017858 | TIPIN | 0.64981282 | 0.95550188 | 1.0993201 | down | mRNA |
| NM_017859 | UCKL1 | 0.995458352 | 0.999872237 | 1.001144 | down | mRNA |
| NM_017860 | C1orf56 | 0.526313747 | 0.917975454 | 1.0311742 | down | mRNA |
| NM_017861 | PIGX | 0.224456019 | 0.719023126 | 1.0635239 | down | mRNA |
| NM_017864 | INTS8 | 0.039222881 | 0.369575679 | 1.3569367 | down | mRNA |
| NM_017866 | TMEM70 | 0.040944397 | 0.375308608 | 1.2661567 | down | mRNA |
| NM_017869 | BANP | 0.169582881 | 0.646121878 | 1.1536245 | down | mRNA |
| NM_017871 | CPSF3L | 0.609385681 | 0.943053241 | 1.0376201 | down | mRNA |
| NM_017872 | THG1L | 0.415358553 | 0.870382888 | 1.0513697 | down | mRNA |
| NM_017873 | ASB6 | 0.149247823 | 0.613155535 | 1.0904848 | down | mRNA |
| NM_017875 | SLC25A38 | 0.004737942 | 0.157921059 | 1.3108411 | down | mRNA |
| NM_017879 | ZNF416 | 0.028977951 | 0.331249882 | 1.534449 | down | mRNA |
| NM_017880 | C2orf42 | 0.136232733 | 0.594906342 | 1.3411105 | down | mRNA |
| NM_017881 | NMRK1 | 0.882086019 | 0.992766819 | 1.041637 | down | mRNA |
| NM_017883 | WDR13 | 0.090436932 | 0.508086128 | 1.0820581 | down | mRNA |
| NM_017886 | ULK4 | 0.210352531 | 0.701096222 | 1.1297634 | down | mRNA |
| NM_017892 | PRPF40A | 0.357131076 | 0.839540876 | 1.0931805 | down | mRNA |
| NM_017894 | ZSCAN2 | 0.667219961 | 0.958354006 | 1.0467712 | down | mRNA |
| NM_017896 | GID8 | 0.047896316 | 0.398293541 | 1.1536811 | down | mRNA |
| NM_017897 | OXSM | 0.000946539 | 0.084526081 | 1.4253031 | down | mRNA |
| NM_017898 | MARC2 | 0.696075287 | 0.962410025 | 1.04574 | down | mRNA |
| NM_017900 | AURKAIP1 | 0.631782288 | 0.950595536 | 1.1285753 | down | mRNA |
| NM_017901 | TPCN1 | 0.36082723 | 0.841780454 | 1.0577515 | down | mRNA |
| NM_017902 | HIF1AN | 0.591287059 | 0.937026383 | 1.0268941 | down | mRNA |
| NM_017904 | TTC22 | 0.131602646 | 0.586786184 | 1.3605665 | down | mRNA |
| NM_017905 | TMCO3 | 0.193639011 | 0.680918086 | 1.1966444 | down | mRNA |
| NM_017906 | PAK1IP1 | 0.039258477 | 0.369575679 | 1.1457375 | down | mRNA |
| NM_017909 | RMND1 | 0.677609116 | 0.959531882 | 1.0357949 | down | mRNA |
| NM_017910 | TRMT61B | 0.135207166 | 0.592787801 | 1.141837 | down | mRNA |
| NM_017913 | CDC37L1 | 0.514083354 | 0.91350662 | 1.1038226 | down | mRNA |
| NM_017914 | C19orf24 | 0.408482342 | 0.867591329 | 1.0720729 | down | mRNA |
| NM_017915 | PARPBP | 0.550473995 | 0.925909806 | 1.1049135 | down | mRNA |
| NM_017917 | PPP2R3C | 0.697637433 | 0.962410025 | 1.0213774 | down | mRNA |
| NM_017918 | CCDC109B | 0.64376522 | 0.954605197 | 1.0884324 | down | mRNA |
| NM_017919 | STX17 | 0.552655379 | 0.926337111 | 1.0699804 | down | mRNA |
| NM_017924 | C14orf119 | 0.07915148 | 0.482239465 | 1.1214266 | down | mRNA |
| NM_017934 | PHIP | 0.01470847 | 0.24380986 | 1.2102466 | down | mRNA |
| NM_017940 | NBPF1 | 0.888479248 | 0.993440915 | 1.0110043 | down | mRNA |
| NM_017941 | C17orf80 | 0.001467787 | 0.098148183 | 1.8527532 | down | mRNA |
| NM_017944 | USP47 | 0.014693369 | 0.24380986 | 1.2253278 | down | mRNA |
| NM_017945 | SLC35A5 | 0.193513353 | 0.680621781 | 1.1320191 | down | mRNA |
| NM_017946 | FKBP14 | 0.579309238 | 0.933821303 | 1.0564594 | down | mRNA |
| NM_017947 | MOCOS | 0.345592477 | 0.832533915 | 1.0598099 | down | mRNA |
| NM_017948 | NOL8 | 0.056765572 | 0.424322651 | 1.3010311 | down | mRNA |
| NM_017950 | CCDC40 | 0.659025544 | 0.958354006 | 1.1276333 | down | mRNA |
| NM_017951 | SMPD4 | 0.31395814 | 0.809054816 | 1.0464054 | down | mRNA |
| NM_017953 | ZNHIT6 | 0.276213745 | 0.775901889 | 1.1623466 | down | mRNA |
| NM_017956 | TRMT12 | 0.01891854 | 0.275933826 | 1.5448052 | down | mRNA |
| NM_017957 | EPN3 | 0.839211367 | 0.987032229 | 1.0141866 | down | mRNA |
| NM_017958 | PLEKHB2 | 0.086058914 | 0.49850423 | 1.1519997 | down | mRNA |
| NM_017964 | SLC30A6 | 0.151401275 | 0.616599711 | 1.1216861 | down | mRNA |
| NM_017969 | IWS1 | 0.015401681 | 0.248924462 | 1.2404059 | down | mRNA |
| NM_017972 | C14orf118 | 0.385643044 | 0.85515381 | 1.0659174 | down | mRNA |
| NM_017974 | ATG16L1 | 0.888249876 | 0.993440915 | 1.0055713 | down | mRNA |
| NM_017978 | ANKHD1 | 0.914215747 | 0.995342392 | 1.0098706 | down | mRNA |
| NM_017980 | LIMS2 | 0.305478717 | 0.801185951 | 1.1654319 | down | mRNA |
| NM_017983 | WIPI1 | 0.401227354 | 0.864768873 | 1.1625916 | down | mRNA |
| NM_017984 | ZCWPW1 | 0.344337282 | 0.831959196 | 1.064048 | down | mRNA |
| NM_017986 | SLC52A1 | 0.677391756 | 0.959531882 | 1.0473807 | down | mRNA |
| NM_017988 | SCYL2 | 0.183117946 | 0.66623548 | 1.1258825 | down | mRNA |
| NM_017993 | ENOX1 | 0.579708366 | 0.933893479 | 1.0978177 | down | mRNA |
| NM_017994 | TMEM248 | 0.419884044 | 0.872365335 | 1.0441092 | down | mRNA |
| NM_017996 | DET1 | 0.012227073 | 0.224166676 | 1.2314031 | down | mRNA |
| NM_017998 | C9orf40 | 0.000858238 | 0.083093876 | 1.2529087 | down | mRNA |
| NM_018002 | OXR1 | 0.448631206 | 0.888279993 | 1.1377829 | down | mRNA |
| NM_018003 | UACA | 0.053732304 | 0.416184837 | 1.4491451 | down | mRNA |
| NM_018004 | TMEM45A | 0.531683868 | 0.920228311 | 1.0670272 | down | mRNA |
| NM_018006 | TRMU | 0.089630624 | 0.506107454 | 1.190791 | down | mRNA |
| NM_018009 | TAPBPL | 0.488873891 | 0.904356651 | 1.1578961 | down | mRNA |
| NM_018011 | ARGLU1 | 0.530156729 | 0.91899174 | 1.0853297 | down | mRNA |
| NM_018013 | SOBP | 0.525346688 | 0.917686109 | 1.4172646 | down | mRNA |
| NM_018017 | C10orf118 | 0.446637676 | 0.886733447 | 1.184791 | down | mRNA |
| NM_018019 | MED9 | 0.02239775 | 0.295133547 | 1.4253668 | down | mRNA |
| NM_018024 | WDYHV1 | 0.985944551 | 0.998971807 | 1.0011312 | down | mRNA |
| NM_018026 | PACS1 | 0.306555838 | 0.801653799 | 1.0539342 | down | mRNA |
| NM_018030 | OSBPL1A | 0.882899614 | 0.992766819 | 1.0229257 | down | mRNA |
| NM_018032 | LUC7L | 0.043135392 | 0.383508423 | 1.1266036 | down | mRNA |
| NM_018034 | WDR70 | 0.625430785 | 0.948832915 | 1.0261578 | down | mRNA |
| NM_018035 | ATP5SL | 0.570326618 | 0.932906321 | 1.0577293 | down | mRNA |
| NM_018036 | ATG2B | 0.088640701 | 0.502998249 | 1.1383155 | down | mRNA |
| NM_018039 | KDM4D | 0.530277941 | 0.91899174 | 1.3172202 | down | mRNA |
| NM_018040 | GPATCH2 | 0.19720469 | 0.685161614 | 1.1740347 | down | mRNA |
| NM_018044 | NSUN5 | 0.589673764 | 0.936090876 | 1.0814787 | down | mRNA |
| NM_018046 | AGGF1 | 0.031952454 | 0.342121122 | 1.1893253 | down | mRNA |
| NM_018049 | PLEKHJ1 | 0.004454936 | 0.153101361 | 1.1672915 | down | mRNA |
| NM_018051 | WDR60 | 0.0182538 | 0.26991728 | 1.1942203 | down | mRNA |
| NM_018052 | VAC14 | 0.08096764 | 0.486318878 | 1.2238569 | down | mRNA |
| NM_018053 | XKR8 | 0.173199629 | 0.651525414 | 1.2997686 | down | mRNA |
| NM_018055 | NODAL | 0.352641895 | 0.835946719 | 1.113232 | down | mRNA |
| NM_018056 | TMEM39B | 0.061299798 | 0.437893235 | 1.1044504 | down | mRNA |
| NM_018060 | IARS2 | 0.184032131 | 0.667572041 | 1.1969435 | down | mRNA |
| NM_018061 | PRPF38B | 0.05521554 | 0.419634622 | 1.22522 | down | mRNA |
| NM_018062 | FANCL | 0.641821414 | 0.954332328 | 1.0371134 | down | mRNA |
| NM_018064 | AKIRIN2 | 0.078448784 | 0.480427915 | 1.301019 | down | mRNA |
| NM_018066 | GPN2 | 0.149978454 | 0.614295311 | 1.1522185 | down | mRNA |
| NM_018072 | HEATR1 | 0.464611143 | 0.894989844 | 1.0683389 | down | mRNA |
| NM_018074 | CCDC94 | 0.237951702 | 0.735822663 | 1.230802 | down | mRNA |
| NM_018075 | ANO10 | 0.07496501 | 0.470573816 | 1.1240384 | down | mRNA |
| NM_018077 | RBM28 | 0.029018353 | 0.331275213 | 1.2236789 | down | mRNA |
| NM_018078 | LARP1B | 0.035742895 | 0.354907714 | 1.3030654 | down | mRNA |
| NM_018084 | CCDC88A | 0.68562256 | 0.960111041 | 1.0214892 | down | mRNA |
| NM_018085 | IPO9 | 0.430023676 | 0.878524598 | 1.062948 | down | mRNA |
| NM_018087 | TMEM48 | 0.462093481 | 0.894754673 | 1.3433383 | down | mRNA |
| NM_018090 | NECAP2 | 0.251129236 | 0.750876415 | 1.0816593 | down | mRNA |
| NM_018091 | ELP3 | 0.086940071 | 0.500185639 | 1.3031568 | down | mRNA |
| NM_018092 | NETO2 | 0.046771824 | 0.393992951 | 1.1822347 | down | mRNA |
| NM_018093 | WDR74 | 0.026495017 | 0.318338323 | 1.1132825 | down | mRNA |
| NM_018094 | GSPT2 | 0.010443949 | 0.209736088 | 1.3000472 | down | mRNA |
| NM_018096 | NLE1 | 0.211971306 | 0.703329735 | 1.2229073 | down | mRNA |
| NM_018101 | CDCA8 | 0.649093295 | 0.95550188 | 1.4145399 | down | mRNA |
| NM_018102 | ZNF334 | 0.71581444 | 0.966191059 | 1.0443548 | down | mRNA |
| NM_018103 | LRRC8D | 0.437095439 | 0.882217212 | 1.1774347 | down | mRNA |
| NM_018105 | THAP1 | 0.061901114 | 0.438960161 | 1.2107875 | down | mRNA |
| NM_018107 | RBM23 | 0.603326343 | 0.941484604 | 1.0889256 | down | mRNA |
| NM_018110 | DOK4 | 0.329724078 | 0.821768501 | 1.1307735 | down | mRNA |
| NM_018111 | C19orf73 | 0.105906886 | 0.538593998 | 1.1724845 | down | mRNA |
| NM_018112 | TMEM38B | 0.840150218 | 0.987068842 | 1.0130559 | down | mRNA |
| NM_018113 | LMBR1L | 0.424588238 | 0.874854499 | 1.0744535 | down | mRNA |
| NM_018114 | DALRD3 | 0.345549205 | 0.832533915 | 1.0781044 | down | mRNA |
| NM_018115 | SDAD1 | 0.030344444 | 0.337507242 | 1.1540326 | down | mRNA |
| NM_018116 | MSTO1 | 0.575818454 | 0.933656777 | 1.0867556 | down | mRNA |
| NM_018119 | POLR3E | 0.099816735 | 0.524954439 | 1.1928688 | down | mRNA |
| NM_018120 | ARMC1 | 0.061472946 | 0.437893235 | 1.2072684 | down | mRNA |
| NM_018121 | FAM178A | 0.078620603 | 0.480619753 | 1.4779327 | down | mRNA |
| NM_018122 | DARS2 | 0.336651686 | 0.826506012 | 1.0946875 | down | mRNA |
| NM_018124 | RFWD3 | 0.666047896 | 0.958354006 | 1.0726932 | down | mRNA |
| NM_018126 | TMEM33 | 0.423454293 | 0.874433545 | 1.038406 | down | mRNA |
| NM_018127 | ELAC2 | 0.247043832 | 0.745152522 | 1.1426634 | down | mRNA |
| NM_018128 | TSR1 | 0.512224306 | 0.91317044 | 1.078523 | down | mRNA |
| NM_018130 | SHQ1 | 0.08579347 | 0.498101894 | 1.333153 | down | mRNA |
| NM_018133 | MSL2 | 0.05881432 | 0.431078857 | 1.1405439 | down | mRNA |
| NM_018135 | MRPS18A | 0.231903055 | 0.729717132 | 1.047776 | down | mRNA |
| NM_018136 | ASPM | 0.767431263 | 0.975033576 | 1.169389 | down | mRNA |
| NM_018138 | TBCCD1 | 0.353892826 | 0.836765779 | 1.0594263 | down | mRNA |
| NM_018141 | MRPS10 | 0.718995675 | 0.966191059 | 1.0369046 | down | mRNA |
| NM_018142 | INTS10 | 0.055044168 | 0.419520587 | 1.3164956 | down | mRNA |
| NM_018143 | KLHL11 | 0.073380289 | 0.466094354 | 1.5790401 | down | mRNA |
| NM_018144 | SEC61A2 | 0.895505493 | 0.993978977 | 1.018905 | down | mRNA |
| NM_018146 | RNMTL1 | 0.048353596 | 0.399016869 | 1.3946838 | down | mRNA |
| NM_018149 | SMG8 | 0.018098599 | 0.269173581 | 1.2771662 | down | mRNA |
| NM_018153 | ANTXR1 | 0.639561861 | 0.953550763 | 1.0978839 | down | mRNA |
| NM_018155 | SLC25A36 | 0.16291876 | 0.635098861 | 1.2554531 | down | mRNA |
| NM_018157 | RIC8B | 0.017951083 | 0.269173581 | 1.189 | down | mRNA |
| NM_018158 | SLC4A1AP | 0.03891836 | 0.368040971 | 1.0937578 | down | mRNA |
| NM_018161 | NADSYN1 | 0.61611299 | 0.945912508 | 1.1063791 | down | mRNA |
| NM_018163 | DNAJC17 | 0.516895642 | 0.91493866 | 1.0699239 | down | mRNA |
| NM_018164 | ASUN | 0.037551902 | 0.362147666 | 1.1085157 | down | mRNA |
| NM_018167 | BTBD7 | 0.599544729 | 0.940648177 | 1.0401047 | down | mRNA |
| NM_018169 | C12orf35 | 0.033196017 | 0.346375523 | 1.2547251 | down | mRNA |
| NM_018170 | RPRD1A | 0.662816476 | 0.958354006 | 1.0809996 | down | mRNA |
| NM_018171 | APPL2 | 0.669155453 | 0.95930319 | 1.0804221 | down | mRNA |
| NM_018172 | FAM86C1 | 0.223322685 | 0.71761506 | 1.1476047 | down | mRNA |
| NM_018174 | MAP1S | 0.084282611 | 0.494150176 | 1.1324561 | down | mRNA |
| NM_018178 | GOLPH3L | 0.047547466 | 0.397833367 | 1.1646736 | down | mRNA |
| NM_018179 | ATF7IP | 0.162546328 | 0.63494386 | 1.1226554 | down | mRNA |
| NM_018181 | ZNF532 | 0.331213991 | 0.821853462 | 1.1161062 | down | mRNA |
| NM_018190 | BBS7 | 0.046284075 | 0.392057225 | 1.1388513 | down | mRNA |
| NM_018191 | RCBTB1 | 0.195161636 | 0.682847425 | 1.3673306 | down | mRNA |
| NM_018193 | FANCI | 0.877102904 | 0.991255275 | 1.0988528 | down | mRNA |
| NM_018196 | TMLHE | 0.937946839 | 0.996710991 | 1.0093228 | down | mRNA |
| NM_018197 | ZFP64 | 0.078638709 | 0.480619753 | 1.2198354 | down | mRNA |
| NM_018202 | TMEM57 | 0.603620092 | 0.941514447 | 1.0551617 | down | mRNA |
| NM_018204 | CKAP2 | 0.575754965 | 0.933656777 | 1.2396351 | down | mRNA |
| NM_018205 | LRRC20 | 0.860876374 | 0.989013989 | 1.0511594 | down | mRNA |
| NM_018206 | VPS35 | 0.20385205 | 0.693939432 | 1.1656417 | down | mRNA |
| NM_018210 | CARKD | 0.233194529 | 0.731100552 | 1.569533 | down | mRNA |
| NM_018212 | ENAH | 0.300633067 | 0.795797471 | 1.0810626 | down | mRNA |
| NM_018216 | PANK4 | 0.027126504 | 0.320017762 | 1.1447956 | down | mRNA |
| NM_018217 | EDEM2 | 0.240398993 | 0.738437735 | 1.1264841 | down | mRNA |
| NM_018221 | MOB1A | 0.682896574 | 0.960111041 | 1.0262577 | down | mRNA |
| NM_018222 | PARVA | 0.75713788 | 0.971960783 | 1.0642199 | down | mRNA |
| NM_018225 | SMU1 | 0.076617322 | 0.475659052 | 1.1220892 | down | mRNA |
| NM_018226 | RNPEPL1 | 0.136952289 | 0.595872872 | 1.1071569 | down | mRNA |
| NM_018227 | UBA6 | 0.868774251 | 0.989481868 | 1.0103583 | down | mRNA |
| NM_018228 | VRTN | 0.085257661 | 0.49712038 | 2.0788796 | down | mRNA |
| NM_018229 | AP5M1 | 0.086830485 | 0.499950487 | 1.2009051 | down | mRNA |
| NM_018230 | NUP133 | 0.105680699 | 0.537981967 | 1.1979141 | down | mRNA |
| NM_018231 | SLC38A7 | 0.054582142 | 0.419253535 | 1.3314417 | down | mRNA |
| NM_018232 | FAM21B | 0.377951315 | 0.851374488 | 1.1752197 | down | mRNA |
| NM_018233 | OGFOD1 | 0.028463774 | 0.327889776 | 1.1759097 | down | mRNA |
| NM_018235 | CNDP2 | 0.599265076 | 0.940648177 | 1.0738516 | down | mRNA |
| NM_018237 | CCAR1 | 0.084004322 | 0.493254641 | 1.2712191 | down | mRNA |
| NM_018238 | AGK | 0.17740971 | 0.657269354 | 1.1138782 | down | mRNA |
| NM_018241 | TMEM184C | 0.074890294 | 0.470395261 | 1.2599375 | down | mRNA |
| NM_018242 | SLC47A1 | 0.247568904 | 0.745868458 | 1.271949 | down | mRNA |
| NM_018243 | SEPT11 | 0.797298899 | 0.980672744 | 1.074108 | down | mRNA |
| NM_018244 | UQCC | 0.547960071 | 0.92514258 | 1.0202262 | down | mRNA |
| NM_018246 | CCDC25 | 0.781195369 | 0.976938981 | 1.0281794 | down | mRNA |
| NM_018247 | TMEM30A | 0.061272632 | 0.437893235 | 1.1142796 | down | mRNA |
| NM_018248 | NEIL3 | 0.400996084 | 0.864768873 | 1.5392327 | down | mRNA |
| NM_018249 | CDK5RAP2 | 0.130927124 | 0.585829729 | 1.1176421 | down | mRNA |
| NM_018252 | TMEM206 | 0.295446207 | 0.79157694 | 1.2943554 | down | mRNA |
| NM_018254 | RCOR3 | 0.149668627 | 0.614139764 | 1.2245315 | down | mRNA |
| NM_018255 | ELP2 | 0.097959315 | 0.520535504 | 1.1748212 | down | mRNA |
| NM_018257 | PCMTD2 | 0.689549484 | 0.96108227 | 1.0366878 | down | mRNA |
| NM_018259 | TTC17 | 0.35653393 | 0.839204431 | 1.1885203 | down | mRNA |
| NM_018263 | ASXL2 | 0.643695617 | 0.954605197 | 1.0729555 | down | mRNA |
| NM_018266 | TMEM39A | 0.380760916 | 0.853514109 | 1.0572921 | down | mRNA |
| NM_018268 | WDR41 | 0.51572324 | 0.914422683 | 1.0641395 | down | mRNA |
| NM_018279 | TMEM19 | 0.018032394 | 0.269173581 | 1.0711261 | down | mRNA |
| NM_018281 | ECHDC2 | 0.695130186 | 0.962214902 | 1.1870677 | down | mRNA |
| NM_018285 | IMP3 | 0.731866597 | 0.967258947 | 1.0176146 | down | mRNA |
| NM_018288 | PHF10 | 0.128578929 | 0.582242044 | 1.160401 | down | mRNA |
| NM_018289 | VPS53 | 0.516153212 | 0.914546356 | 1.1801548 | down | mRNA |
| NM_018290 | PGM2 | 0.761652636 | 0.97339948 | 1.0449126 | down | mRNA |
| NM_018292 | QRSL1 | 0.002057574 | 0.109514633 | 1.1993307 | down | mRNA |
| NM_018293 | ZNF654 | 0.055354329 | 0.419636174 | 1.5038353 | down | mRNA |
| NM_018295 | TMEM140 | 0.602876158 | 0.941464987 | 1.5433679 | down | mRNA |
| NM_018299 | UBE2W | 0.097089026 | 0.519544195 | 1.3333656 | down | mRNA |
| NM_018301 | RBM41 | 0.269687904 | 0.76993462 | 1.1145556 | down | mRNA |
| NM_018302 | C4orf19 | 0.416963764 | 0.870758755 | 1.1547989 | down | mRNA |
| NM_018303 | EXOC2 | 0.024081261 | 0.303619251 | 1.3357542 | down | mRNA |
| NM_018304 | PRR11 | 0.6668193 | 0.958354006 | 1.0778186 | down | mRNA |
| NM_018307 | RHOT1 | 0.088260382 | 0.502399895 | 1.2484938 | down | mRNA |
| NM_018309 | TBC1D23 | 0.004776258 | 0.158085585 | 1.2485019 | down | mRNA |
| NM_018312 | PPP6R3 | 0.145015371 | 0.606427917 | 1.1763095 | down | mRNA |
| NM_018313 | PBRM1 | 0.114066424 | 0.557835051 | 1.1584916 | down | mRNA |
| NM_018314 | UEVLD | 0.729851451 | 0.966191059 | 1.0386839 | down | mRNA |
| NM_018315 | FBXW7 | 0.027559228 | 0.323331929 | 1.627829 | down | mRNA |
| NM_018317 | TBC1D19 | 0.394103381 | 0.861008404 | 1.0332051 | down | mRNA |
| NM_018318 | CCDC91 | 0.583185357 | 0.934269626 | 1.0630548 | down | mRNA |
| NM_018320 | RNF121 | 0.847753214 | 0.987678166 | 1.0217799 | down | mRNA |
| NM_018321 | BRIX1 | 0.054050499 | 0.417190119 | 1.2048487 | down | mRNA |
| NM_018322 | SAYSD1 | 0.236691528 | 0.73465251 | 1.1400521 | down | mRNA |
| NM_018323 | PI4K2B | 0.535443285 | 0.921580706 | 1.0673877 | down | mRNA |
| NM_018327 | SPTLC3 | 0.792991486 | 0.979003983 | 1.080481 | down | mRNA |
| NM_018328 | MBD5 | 0.55536686 | 0.926833616 | 1.1007757 | down | mRNA |
| NM_018330 | KIAA1598 | 0.398849721 | 0.863643939 | 1.0845338 | down | mRNA |
| NM_018332 | DDX19A | 0.291203458 | 0.787647825 | 1.1347938 | down | mRNA |
| NM_018339 | RFK | 0.129698823 | 0.583907255 | 1.1301732 | down | mRNA |
| NM_018340 | CPPED1 | 0.801600932 | 0.98118927 | 1.0330251 | down | mRNA |
| NM_018341 | C6orf70 | 0.166770271 | 0.64182721 | 1.1353022 | down | mRNA |
| NM_018343 | RIOK2 | 0.03192567 | 0.342121122 | 1.380005 | down | mRNA |
| NM_018346 | RSAD1 | 0.359463887 | 0.841033672 | 1.0926058 | down | mRNA |
| NM_018347 | AP5S1 | 0.013603636 | 0.235988767 | 1.2211762 | down | mRNA |
| NM_018352 | TMA16 | 0.59037704 | 0.93638708 | 1.0555062 | down | mRNA |
| NM_018354 | TMEM74B | 0.901899144 | 0.994906068 | 1.0113793 | down | mRNA |
| NM_018357 | LARP6 | 0.030535851 | 0.338388833 | 1.4516623 | down | mRNA |
| NM_018358 | ABCF3 | 0.005492551 | 0.16971815 | 1.2908609 | down | mRNA |
| NM_018359 | UFSP2 | 0.558397502 | 0.926833616 | 1.060417 | down | mRNA |
| NM_018360 | TXLNG | 0.008334052 | 0.198394583 | 1.0983718 | down | mRNA |
| NM_018361 | AGPAT5 | 0.882787364 | 0.992766819 | 1.0274679 | down | mRNA |
| NM_018364 | RSBN1 | 0.283311891 | 0.780779444 | 1.212355 | down | mRNA |
| NM_018366 | BLOC1S4 | 0.113776462 | 0.557825663 | 1.2043381 | down | mRNA |
| NM_018367 | ACER3 | 0.493623282 | 0.906462119 | 1.0659982 | down | mRNA |
| NM_018368 | LMBRD1 | 0.051556137 | 0.41031217 | 1.340087 | down | mRNA |
| NM_018369 | DEPDC1B | 0.832347725 | 0.986211576 | 1.1188065 | down | mRNA |
| NM_018372 | LRIF1 | 0.567324689 | 0.931514776 | 1.049969 | down | mRNA |
| NM_018373 | SYNJ2BP | 0.652827088 | 0.956714149 | 1.0270502 | down | mRNA |
| NM_018375 | SLC39A9 | 0.567532052 | 0.931696542 | 1.0436839 | down | mRNA |
| NM_018379 | FAM63A | 0.143799735 | 0.604279163 | 1.1361116 | down | mRNA |
| NM_018383 | WDR33 | 0.292423841 | 0.788954556 | 1.2633322 | down | mRNA |
| NM_018387 | STRBP | 0.353226718 | 0.836266379 | 1.0924614 | down | mRNA |
| NM_018389 | SLC35C1 | 0.664209014 | 0.958354006 | 1.0564082 | down | mRNA |
| NM_018394 | ABHD10 | 0.002071555 | 0.109970903 | 1.4040094 | down | mRNA |
| NM_018400 | SCN3B | 0.63989479 | 0.953734314 | 1.0705558 | down | mRNA |
| NM_018401 | STK32B | 0.482077561 | 0.902092906 | 1.0596817 | down | mRNA |
| NM_018406 | MUC4 | 0.519716278 | 0.916187634 | 1.16165 | down | mRNA |
| NM_018407 | LAPTM4B | 0.320238338 | 0.814552264 | 1.0975055 | down | mRNA |
| NM_018409 | LRP2BP | 0.011436959 | 0.218702965 | 1.799365 | down | mRNA |
| NM_018412 | ST7 | 0.384925725 | 0.855147437 | 1.0366664 | down | mRNA |
| NM_018416 | FOXJ2 | 0.007889055 | 0.194660525 | 1.5847685 | down | mRNA |
| NM_018417 | ADCY10 | 0.054291879 | 0.418129726 | 1.3036277 | down | mRNA |
| NM_018421 | TBC1D2 | 0.000240954 | 0.052117825 | 1.5113666 | down | mRNA |
| NM_018424 | EPB41L4B | 0.036033772 | 0.356485876 | 1.1757489 | down | mRNA |
| NM_018427 | RRN3 | 0.173250788 | 0.651548201 | 1.2565909 | down | mRNA |
| NM_018429 | BDP1 | 0.266247358 | 0.767064488 | 1.1546844 | down | mRNA |
| NM_018431 | DOK5 | 0.255761509 | 0.756604539 | 1.2303709 | down | mRNA |
| NM_018437 | HEMGN | 0.479661746 | 0.901538317 | 1.0556144 | down | mRNA |
| NM_018438 | FBXO6 | 0.673698007 | 0.959531882 | 1.0915823 | down | mRNA |
| NM_018439 | IMPACT | 0.60115118 | 0.941028182 | 1.0658246 | down | mRNA |
| NM_018442 | DCAF6 | 0.900377127 | 0.994789506 | 1.0117651 | down | mRNA |
| NM_018443 | ZNF302 | 0.126061159 | 0.578441772 | 1.2435171 | down | mRNA |
| NM_018444 | PDP1 | 0.353618104 | 0.836408015 | 1.075754 | down | mRNA |
| NM_018445 | VIMP | 0.084685536 | 0.495154665 | 1.1739845 | down | mRNA |
| NM_018446 | GLT8D1 | 0.183796318 | 0.667497719 | 1.1887733 | down | mRNA |
| NM_018447 | EMC3 | 0.92299454 | 0.995349793 | 1.0046447 | down | mRNA |
| NM_018448 | CAND1 | 0.038886968 | 0.368040971 | 1.3382809 | down | mRNA |
| NM_018451 | CENPJ | 0.657977924 | 0.958309968 | 1.1157919 | down | mRNA |
| NM_018452 | TMEM242 | 0.295647004 | 0.791662837 | 1.0578644 | down | mRNA |
| NM_018453 | EAPP | 0.025460724 | 0.31316845 | 1.2247738 | down | mRNA |
| NM_018454 | NUSAP1 | 0.837420992 | 0.986775289 | 1.1148946 | down | mRNA |
| NM_018455 | CENPN | 0.379294242 | 0.852847753 | 1.1548448 | down | mRNA |
| NM_018456 | EAF2 | 0.723870754 | 0.966191059 | 1.0970938 | down | mRNA |
| NM_018457 | PRR13 | 0.237348795 | 0.735300273 | 1.1076057 | down | mRNA |
| NM_018461 | PPP2R2D | 0.683251433 | 0.960111041 | 1.0659726 | down | mRNA |
| NM_018463 | ITFG2 | 0.040173547 | 0.371915487 | 1.3717487 | down | mRNA |
| NM_018469 | TEX2 | 0.346923541 | 0.833567116 | 1.0471187 | down | mRNA |
| NM_018471 | ZC3H15 | 0.048356166 | 0.399016869 | 1.1809897 | down | mRNA |
| NM_018474 | PLK1S1 | 0.713446261 | 0.966110889 | 1.0606137 | down | mRNA |
| NM_018476 | BEX1 | 0.699298358 | 0.962701213 | 1.3084927 | down | mRNA |
| NM_018477 | ACTR10 | 0.227519539 | 0.723479398 | 1.1089997 | down | mRNA |
| NM_018479 | ECHDC1 | 0.139359247 | 0.598648178 | 1.0727917 | down | mRNA |
| NM_018480 | TMEM126B | 0.976902608 | 0.998501532 | 1.0021145 | down | mRNA |
| NM_018489 | ASH1L | 0.012694864 | 0.23004633 | 1.3264048 | down | mRNA |
| NM_018490 | LGR4 | 0.69741631 | 0.962410025 | 1.0308869 | down | mRNA |
| NM_018491 | CBWD1 | 0.806281002 | 0.98118927 | 1.0188597 | down | mRNA |
| NM_018492 | PBK | 0.945498415 | 0.996710991 | 1.0438101 | down | mRNA |
| NM_018502 | TMCO6 | 0.322298114 | 0.815372756 | 1.2892078 | down | mRNA |
| NM_018569 | AP1AR | 0.181326729 | 0.664839093 | 1.1120667 | down | mRNA |
| NM_018571 | STRADB | 0.80090197 | 0.98118927 | 1.0269416 | down | mRNA |
| NM_018590 | CSGALNACT2 | 0.117212316 | 0.564147103 | 1.076559 | down | mRNA |
| NM_018622 | PARL | 0.283496899 | 0.781143644 | 1.09344 | down | mRNA |
| NM_018639 | WSB2 | 0.129940179 | 0.583907255 | 1.1203201 | down | mRNA |
| NM_018641 | CHST12 | 0.090164213 | 0.507386748 | 1.1358945 | down | mRNA |
| NM_018644 | B3GAT1 | 0.12888276 | 0.582839032 | 1.2665322 | down | mRNA |
| NM_018645 | HES6 | 0.278789203 | 0.777783404 | 1.1870178 | down | mRNA |
| NM_018648 | NOP10 | 0.576973157 | 0.933656777 | 1.0455161 | down | mRNA |
| NM_018649 | H2AFY2 | 0.780860076 | 0.976938981 | 1.0368632 | down | mRNA |
| NM_018656 | SLC35E3 | 0.547622061 | 0.92514258 | 1.0371703 | down | mRNA |
| NM_018659 | CYTL1 | 0.036628116 | 0.359248842 | 1.2051706 | down | mRNA |
| NM_018660 | ZNF395 | 0.705047316 | 0.964577129 | 1.0654214 | down | mRNA |
| NM_018665 | DDX43 | 0.511929306 | 0.912870256 | 1.0697895 | down | mRNA |
| NM_018668 | VPS33B | 0.296996399 | 0.793620415 | 1.2083536 | down | mRNA |
| NM_018677 | ACSS2 | 0.448464184 | 0.888104977 | 1.2583038 | down | mRNA |
| NM_018682 | MLL5 | 0.028379564 | 0.327830694 | 1.1881904 | down | mRNA |
| NM_018684 | ZC4H2 | 0.930827526 | 0.996710991 | 1.0216974 | down | mRNA |
| NM_018685 | ANLN | 0.851663272 | 0.987678166 | 1.1031009 | down | mRNA |
| NM_018686 | CMAS | 0.297095207 | 0.793643704 | 1.0856348 | down | mRNA |
| NM_018688 | BIN3 | 0.194729113 | 0.682390954 | 1.1759176 | down | mRNA |
| NM_018689 | KIAA1199 | 0.996695586 | 0.999872237 | 1.0012165 | down | mRNA |
| NM_018691 | FAM114A2 | 0.188523605 | 0.673885712 | 1.1368538 | down | mRNA |
| NM_018695 | ERBB2IP | 0.015306659 | 0.248375884 | 1.294817 | down | mRNA |
| NM_018696 | ELAC1 | 0.02115314 | 0.288424269 | 1.3616399 | down | mRNA |
| NM_018698 | NXT2 | 0.751029076 | 0.970725371 | 1.070447 | down | mRNA |
| NM_018699 | PRDM5 | 0.350627585 | 0.834911159 | 1.1979634 | down | mRNA |
| NM_018703 | RBBP6 | 0.042803466 | 0.382359436 | 1.1931594 | down | mRNA |
| NM_018704 | CTTNBP2NL | 0.265478077 | 0.766537754 | 1.2031997 | down | mRNA |
| NM_018706 | DHTKD1 | 0.09780694 | 0.520535504 | 1.3323587 | down | mRNA |
| NM_018714 | COG1 | 0.131526801 | 0.586786184 | 1.0875753 | down | mRNA |
| NM_018715 | RCC2 | 0.610226969 | 0.943169861 | 1.068089 | down | mRNA |
| NM_018718 | CEP41 | 0.804664214 | 0.98118927 | 1.0173862 | down | mRNA |
| NM_018719 | CDCA7L | 0.251613525 | 0.751762041 | 1.1885908 | down | mRNA |
| NM_018835 | RC3H2 | 0.179809965 | 0.662598807 | 1.0873325 | down | mRNA |
| NM_018837 | SULF2 | 0.627515143 | 0.949374051 | 1.0465061 | down | mRNA |
| NM_018838 | NDUFA12 | 0.476301812 | 0.900415348 | 1.0465169 | down | mRNA |
| NM_018839 | NSFL1C | 0.888622463 | 0.993440915 | 1.0106554 | down | mRNA |
| NM_018841 | GNG12 | 0.37948621 | 0.852847753 | 1.0469443 | down | mRNA |
| NM_018843 | SLC25A40 | 0.491842526 | 0.905806259 | 1.102174 | down | mRNA |
| NM_018847 | KLHL9 | 0.113068907 | 0.556234457 | 1.1857846 | down | mRNA |
| NM_018849 | ABCB4 | 0.826639822 | 0.984436279 | 1.1787647 | down | mRNA |
| NM_018896 | CACNA1G | 0.667891206 | 0.958354006 | 1.0423385 | down | mRNA |
| NM_018898 | PCDHAC1 | 0.088035904 | 0.501570433 | 1.5115332 | down | mRNA |
| NM_018914 | PCDHGA11 | 0.989868209 | 0.999140676 | 1.0017705 | down | mRNA |
| NM_018927 | PCDHGB7 | 0.672129951 | 0.959531882 | 1.0401084 | down | mRNA |
| NM_018935 | PCDHB15 | 0.101512909 | 0.529356363 | 1.2475856 | down | mRNA |
| NM_018944 | MIS18A | 0.11638458 | 0.56274228 | 1.1591116 | down | mRNA |
| NM_018945 | PDE7B | 0.587534429 | 0.93579682 | 1.203878 | down | mRNA |
| NM_018946 | NANS | 0.769142403 | 0.975270932 | 1.0373193 | down | mRNA |
| NM_018956 | C9orf9 | 0.816716343 | 0.981692737 | 1.0492114 | down | mRNA |
| NM_018959 | DAZAP1 | 0.730203217 | 0.966191059 | 1.0500874 | down | mRNA |
| NM_018962 | DSCR6 | 0.634106591 | 0.951206671 | 1.0359528 | down | mRNA |
| NM_018963 | BRWD1 | 0.226583203 | 0.72196986 | 1.2143788 | down | mRNA |
| NM_018965 | TREM2 | 0.688925432 | 0.960979136 | 1.0518352 | down | mRNA |
| NM_018975 | TERF2IP | 0.762651668 | 0.973443358 | 1.1400659 | down | mRNA |
| NM_018976 | SLC38A2 | 0.000103678 | 0.037538791 | 2.6496185 | down | mRNA |
| NM_018981 | DNAJC10 | 0.442126006 | 0.88419144 | 1.0402529 | down | mRNA |
| NM_018982 | YIPF1 | 0.373499176 | 0.848504826 | 1.0815365 | down | mRNA |
| NM_018984 | SSH1 | 0.014685233 | 0.24380986 | 1.3747556 | down | mRNA |
| NM_018988 | GFOD1 | 0.043360751 | 0.384311589 | 1.5540972 | down | mRNA |
| NM_018989 | RBM27 | 0.26694124 | 0.767456064 | 1.1624022 | down | mRNA |
| NM_018992 | KCTD5 | 0.055238862 | 0.419634622 | 1.4579984 | down | mRNA |
| NM_018993 | RIN2 | 0.986247456 | 0.998971807 | 1.0048496 | down | mRNA |
| NM_018994 | FBXO42 | 0.025936136 | 0.315604326 | 1.1695461 | down | mRNA |
| NM_018997 | MRPS21 | 0.254369723 | 0.75479352 | 1.0623705 | down | mRNA |
| NM_018998 | FBXW5 | 0.067704651 | 0.451808679 | 1.0696454 | down | mRNA |
| NM_018999 | FAM190B | 0.695894064 | 0.962410025 | 1.0475968 | down | mRNA |
| NM_019001 | XRN1 | 0.700000206 | 0.962991468 | 1.0831079 | down | mRNA |
| NM_019002 | ETAA1 | 0.865402457 | 0.989401142 | 1.0146954 | down | mRNA |
| NM_019005 | MIOS | 0.005852303 | 0.173990061 | 1.5043678 | down | mRNA |
| NM_019008 | SMCR7L | 0.043726269 | 0.385351177 | 1.2439092 | down | mRNA |
| NM_019009 | TOLLIP | 0.014464027 | 0.242674797 | 1.1213218 | down | mRNA |
| NM_019010 | KRT20 | 0.699055544 | 0.962701213 | 1.0311589 | down | mRNA |
| NM_019013 | FAM64A | 0.533738944 | 0.92058057 | 1.2931549 | down | mRNA |
| NM_019014 | POLR1B | 0.128513384 | 0.582242044 | 1.532276 | down | mRNA |
| NM_019022 | TMX3 | 0.128841046 | 0.582780013 | 1.1335386 | down | mRNA |
| NM_019023 | PRMT7 | 0.329781758 | 0.821768501 | 1.1264081 | down | mRNA |
| NM_019024 | HEATR5B | 0.045379506 | 0.390294464 | 1.2829303 | down | mRNA |
| NM_019026 | TMCO1 | 0.543201966 | 0.923577434 | 1.0197461 | down | mRNA |
| NM_019028 | ZDHHC13 | 0.787573947 | 0.977492986 | 1.012559 | down | mRNA |
| NM_019030 | DHX29 | 0.130333566 | 0.584331216 | 1.0956239 | down | mRNA |
| NM_019035 | PCDH18 | 0.00631696 | 0.180328763 | 1.7959284 | down | mRNA |
| NM_019036 | HMGCLL1 | 0.059484134 | 0.432586127 | 1.0959427 | down | mRNA |
| NM_019037 | EXOSC4 | 0.052579622 | 0.413038171 | 1.221577 | down | mRNA |
| NM_019040 | ELP4 | 0.116140301 | 0.562521166 | 1.0861372 | down | mRNA |
| NM_019042 | PUS7 | 0.017614548 | 0.267267899 | 2.3905846 | down | mRNA |
| NM_019045 | WDR44 | 0.031473276 | 0.341322176 | 1.3070339 | down | mRNA |
| NM_019046 | ANKRD16 | 0.10261808 | 0.531600463 | 1.1831191 | down | mRNA |
| NM_019048 | ASNSD1 | 0.980971515 | 0.998971807 | 1.0020788 | down | mRNA |
| NM_019050 | USP53 | 0.819364267 | 0.982224663 | 1.0356364 | down | mRNA |
| NM_019051 | MRPL50 | 0.533051478 | 0.920530493 | 1.0293532 | down | mRNA |
| NM_019053 | EXOC6 | 0.374516861 | 0.84936907 | 1.1163193 | down | mRNA |
| NM_019054 | FAM35A | 0.057148076 | 0.425938435 | 1.2071235 | down | mRNA |
| NM_019067 | GNL3L | 0.878112605 | 0.991445937 | 1.0073417 | down | mRNA |
| NM_019071 | ING3 | 0.020369471 | 0.285032405 | 1.2888419 | down | mRNA |
| NM_019072 | SGTB | 0.699620615 | 0.962904533 | 1.0578723 | down | mRNA |
| NM_019080 | NDFIP2 | 0.512844865 | 0.913297047 | 1.179896 | down | mRNA |
| NM_019082 | DDX56 | 0.024785638 | 0.307526004 | 1.2400019 | down | mRNA |
| NM_019083 | TRMT13 | 0.019849156 | 0.283521851 | 1.4267574 | down | mRNA |
| NM_019086 | VSIG10 | 0.663798804 | 0.958354006 | 1.1081435 | down | mRNA |
| NM_019091 | PLEKHA3 | 0.411626431 | 0.869001481 | 1.1181645 | down | mRNA |
| NM_019095 | CRLS1 | 0.317942779 | 0.811908136 | 1.152235 | down | mRNA |
| NM_019106 | SEPT3 | 0.425802135 | 0.87545849 | 1.1854176 | down | mRNA |
| NM_019110 | ZKSCAN4 | 0.289330741 | 0.785298708 | 1.1602352 | down | mRNA |
| NM_019113 | FGF21 | 0.386203226 | 0.855747763 | 1.5109991 | down | mRNA |
| NM_019556 | MOSPD1 | 0.908803265 | 0.995333785 | 1.0070891 | down | mRNA |
| NM_019557 | FAM54B | 0.162453385 | 0.6347045 | 1.2209665 | down | mRNA |
| NM_019591 | ZNF26 | 0.102321051 | 0.531118612 | 2.1382082 | down | mRNA |
| NM_019592 | RNF20 | 0.110950854 | 0.551283668 | 1.2537801 | down | mRNA |
| NM_019594 | LRRC8A | 0.076775452 | 0.476495267 | 1.152435 | down | mRNA |
| NM_019597 | HNRNPH2 | 0.521469609 | 0.916644975 | 1.1069783 | down | mRNA |
| NM_019607 | C8orf44 | 0.681248989 | 0.960111041 | 1.0681109 | down | mRNA |
| NM_019616 | F7 | 0.181497096 | 0.664894785 | 1.0629707 | down | mRNA |
| NM_019854 | PRMT8 | 0.690418126 | 0.961105466 | 1.0264372 | down | mRNA |
| NM_019884 | GSK3A | 0.550675123 | 0.925928432 | 1.1691301 | down | mRNA |
| NM_019885 | CYP26B1 | 0.882924902 | 0.992766819 | 1.0250906 | down | mRNA |
| NM_019886 | CHST7 | 0.658749185 | 0.958354006 | 1.0476279 | down | mRNA |
| NM_019891 | ERO1LB | 0.692063739 | 0.961451327 | 1.0808053 | down | mRNA |
| NM_019903 | ADD3 | 0.610761385 | 0.94354536 | 1.1260825 | down | mRNA |
| NM_020039 | ASIC1 | 0.732989708 | 0.967703318 | 1.0631811 | down | mRNA |
| NM_020066 | FMN2 | 0.600117063 | 0.940648177 | 1.1280471 | down | mRNA |
| NM_020070 | IGLL1 | 0.485591092 | 0.903250293 | 1.1011944 | down | mRNA |
| NM_020120 | UGGT1 | 0.231921705 | 0.729717132 | 1.0875218 | down | mRNA |
| NM_020121 | UGGT2 | 0.750215685 | 0.970725371 | 1.0546996 | down | mRNA |
| NM_020122 | KCMF1 | 0.842448836 | 0.987182488 | 1.0179504 | down | mRNA |
| NM_020123 | TM9SF3 | 0.182559651 | 0.665794229 | 1.2191762 | down | mRNA |
| NM_020126 | SPHK2 | 0.053696117 | 0.416065637 | 1.3008032 | down | mRNA |
| NM_020128 | MDM1 | 0.125858104 | 0.578441772 | 1.3627049 | down | mRNA |
| NM_020133 | AGPAT4 | 0.120725222 | 0.572297789 | 1.3311188 | down | mRNA |
| NM_020135 | WRNIP1 | 0.047743441 | 0.397835916 | 1.3367463 | down | mRNA |
| NM_020141 | TMEM167B | 0.746957474 | 0.970432947 | 1.0485202 | down | mRNA |
| NM_020142 | NDUFA4L2 | 0.967156176 | 0.99793927 | 1.0316531 | down | mRNA |
| NM_020143 | PNO1 | 0.36098189 | 0.841780454 | 1.1080303 | down | mRNA |
| NM_020145 | SH3GLB2 | 0.729515327 | 0.966191059 | 1.0419215 | down | mRNA |
| NM_020148 | SPIRE1 | 0.027644091 | 0.32381669 | 1.2070735 | down | mRNA |
| NM_020153 | IFT46 | 0.438962658 | 0.882895421 | 1.1491101 | down | mRNA |
| NM_020154 | EMC7 | 0.864224357 | 0.989401142 | 1.0122568 | down | mRNA |
| NM_020155 | GPR137 | 0.370851919 | 0.846330273 | 1.0670433 | down | mRNA |
| NM_020158 | EXOSC5 | 0.791940507 | 0.978996327 | 1.0526893 | down | mRNA |
| NM_020159 | SMARCAD1 | 0.021637609 | 0.29025889 | 1.3968189 | down | mRNA |
| NM_020160 | MEIS3 | 0.438699318 | 0.882895421 | 1.0998193 | down | mRNA |
| NM_020165 | RAD18 | 0.870366962 | 0.989592579 | 1.0500667 | down | mRNA |
| NM_020166 | MCCC1 | 0.39640979 | 0.861995469 | 1.0879471 | down | mRNA |
| NM_020175 | DUS3L | 0.049588835 | 0.402973698 | 1.184857 | down | mRNA |
| NM_020179 | C11orf75 | 0.364020837 | 0.843353654 | 1.0688734 | down | mRNA |
| NM_020186 | ACN9 | 0.669939878 | 0.959531882 | 1.0296074 | down | mRNA |
| NM_020188 | CMC2 | 0.539800569 | 0.923577434 | 1.0584807 | down | mRNA |
| NM_020190 | OLFML3 | 0.555606941 | 0.926833616 | 1.1389833 | down | mRNA |
| NM_020191 | MRPS22 | 0.328091838 | 0.820939843 | 1.2136238 | down | mRNA |
| NM_020192 | YAE1D1 | 0.005386087 | 0.168450524 | 1.3974555 | down | mRNA |
| NM_020193 | C11orf30 | 0.035602673 | 0.354492429 | 1.6027608 | down | mRNA |
| NM_020195 | SDR39U1 | 0.281297894 | 0.779126326 | 1.1829582 | down | mRNA |
| NM_020196 | XAB2 | 0.640013327 | 0.953734314 | 1.0388692 | down | mRNA |
| NM_020197 | SMYD2 | 0.78411114 | 0.977347603 | 1.0999112 | down | mRNA |
| NM_020202 | NIT2 | 0.9755773 | 0.998501532 | 1.0031638 | down | mRNA |
| NM_020205 | OTUD7B | 0.758760204 | 0.972154041 | 1.0910386 | down | mRNA |
| NM_020211 | RGMA | 0.810192916 | 0.98118927 | 1.0627528 | down | mRNA |
| NM_020214 | PARP6 | 0.329968426 | 0.821768501 | 1.0818214 | down | mRNA |
| NM_020216 | RNPEP | 0.329220959 | 0.821768501 | 1.1805192 | down | mRNA |
| NM_020218 | ATXN7L3 | 0.673017429 | 0.959531882 | 1.0365884 | down | mRNA |
| NM_020219 | CEACAM19 | 0.131504299 | 0.586786184 | 1.2383765 | down | mRNA |
| NM_020223 | FAM20C | 0.092224532 | 0.511207518 | 1.3721095 | down | mRNA |
| NM_020230 | PPAN | 0.05055856 | 0.406628421 | 1.2461002 | down | mRNA |
| NM_020232 | PSMG2 | 0.169619698 | 0.646121878 | 1.0941808 | down | mRNA |
| NM_020234 | DTWD1 | 0.779783419 | 0.976938981 | 1.0614188 | down | mRNA |
| NM_020238 | INCENP | 0.851316196 | 0.987678166 | 1.0201495 | down | mRNA |
| NM_020240 | CDC42SE2 | 0.255460195 | 0.756286855 | 1.1958093 | down | mRNA |
| NM_020242 | KIF15 | 0.662888035 | 0.958354006 | 1.350749 | down | mRNA |
| NM_020243 | TOMM22 | 0.584015312 | 0.934461268 | 1.0780087 | down | mRNA |
| NM_020244 | CHPT1 | 0.720926777 | 0.966191059 | 1.0243539 | down | mRNA |
| NM_020248 | CTNNBIP1 | 0.886441365 | 0.992981836 | 1.0378788 | down | mRNA |
| NM_020299 | AKR1B10 | 0.673850298 | 0.959531882 | 1.0520613 | down | mRNA |
| NM_020310 | MNT | 0.010602845 | 0.210319067 | 2.5889246 | down | mRNA |
| NM_020312 | COQ9 | 0.256939949 | 0.75762862 | 1.0740994 | down | mRNA |
| NM_020313 | CIAPIN1 | 0.02394598 | 0.303389731 | 1.2506594 | down | mRNA |
| NM_020314 | C16orf62 | 0.125965293 | 0.578441772 | 1.280338 | down | mRNA |
| NM_020315 | PDXP | 0.956513487 | 0.996883425 | 1.0135969 | down | mRNA |
| NM_020317 | C1orf63 | 0.135136342 | 0.592787801 | 1.2508817 | down | mRNA |
| NM_020320 | RARS2 | 0.819649064 | 0.982331503 | 1.0594658 | down | mRNA |
| NM_020321 | ASIC3 | 0.844949483 | 0.987338076 | 1.023655 | down | mRNA |
| NM_020335 | VANGL2 | 0.205278021 | 0.695403826 | 1.2193991 | down | mRNA |
| NM_020336 | RALGAPB | 0.061940621 | 0.438960161 | 1.1536254 | down | mRNA |
| NM_020337 | ANKRD50 | 0.494693552 | 0.907034836 | 1.1151163 | down | mRNA |
| NM_020338 | ZMIZ1 | 0.034454677 | 0.351579889 | 1.1340506 | down | mRNA |
| NM_020345 | NKIRAS1 | 0.075021342 | 0.470782078 | 1.1604446 | down | mRNA |
| NM_020350 | AGTRAP | 0.921580574 | 0.995349793 | 1.009084 | down | mRNA |
| NM_020357 | PCNP | 0.41175734 | 0.869001481 | 1.0307015 | down | mRNA |
| NM_020360 | PLSCR3 | 0.859316937 | 0.988512246 | 1.0190981 | down | mRNA |
| NM_020362 | PITHD1 | 0.376902005 | 0.850518487 | 1.1168169 | down | mRNA |
| NM_020363 | DAZ2 | 0.432673355 | 0.88061258 | 1.1048345 | down | mRNA |
| NM_020364 | DAZ3 | 0.871860633 | 0.990206144 | 1.0331093 | down | mRNA |
| NM_020368 | UTP3 | 0.433932313 | 0.880923255 | 1.0603261 | down | mRNA |
| NM_020371 | AVEN | 0.609635723 | 0.943053241 | 1.1155627 | down | mRNA |
| NM_020372 | SLC22A17 | 0.387427018 | 0.856773191 | 1.1954837 | down | mRNA |
| NM_020374 | C12orf4 | 0.702003365 | 0.963247543 | 1.046377 | down | mRNA |
| NM_020375 | C12orf5 | 0.003556385 | 0.138787761 | 1.2614435 | down | mRNA |
| NM_020376 | PNPLA2 | 0.496675662 | 0.908665955 | 1.0251206 | down | mRNA |
| NM_020378 | NAT14 | 0.206180353 | 0.697397925 | 1.0867183 | down | mRNA |
| NM_020381 | PDSS2 | 0.740352863 | 0.969794316 | 1.0430056 | down | mRNA |
| NM_020387 | RAB25 | 0.397892554 | 0.863112281 | 1.036835 | down | mRNA |
| NM_020390 | EIF5A2 | 0.008576151 | 0.199736893 | 1.1676458 | down | mRNA |
| NM_020395 | INTS12 | 0.058678329 | 0.430858718 | 1.2470649 | down | mRNA |
| NM_020397 | CAMK1D | 0.977159844 | 0.998501532 | 1.0039936 | down | mRNA |
| NM_020401 | NUP107 | 0.500826439 | 0.909680616 | 1.1083538 | down | mRNA |
| NM_020406 | CD177 | 0.78656801 | 0.977479172 | 1.041881 | down | mRNA |
| NM_020409 | MRPL47 | 0.895784027 | 0.993978977 | 1.018878 | down | mRNA |
| NM_020412 | CHMP1B | 0.20897325 | 0.700205031 | 1.1208607 | down | mRNA |
| NM_020414 | DDX24 | 0.651354229 | 0.956204521 | 1.1174531 | down | mRNA |
| NM_020418 | PCBP4 | 0.723418469 | 0.966191059 | 1.0436214 | down | mRNA |
| NM_020436 | SALL4 | 0.409799227 | 0.86801103 | 1.1061874 | down | mRNA |
| NM_020437 | ASPHD2 | 0.208208282 | 0.698793461 | 1.1292581 | down | mRNA |
| NM_020440 | PTGFRN | 0.96339878 | 0.99793927 | 1.0035747 | down | mRNA |
| NM_020442 | VARS2 | 0.667146137 | 0.958354006 | 1.0138394 | down | mRNA |
| NM_020443 | NAV1 | 0.619686764 | 0.947444797 | 1.1961623 | down | mRNA |
| NM_020445 | ACTR3B | 0.241764508 | 0.7401831 | 1.526694 | down | mRNA |
| NM_020448 | NIPAL3 | 0.580047316 | 0.933893479 | 1.127235 | down | mRNA |
| NM_020452 | ATP8B2 | 0.133068315 | 0.588930122 | 1.1352172 | down | mRNA |
| NM_020453 | ATP10D | 0.110702097 | 0.550585871 | 1.2066808 | down | mRNA |
| NM_020455 | GPR126 | 0.046163731 | 0.391970965 | 1.34275 | down | mRNA |
| NM_020456 | SPRYD7 | 0.04940005 | 0.402500646 | 1.1478434 | down | mRNA |
| NM_020457 | THAP11 | 0.003806389 | 0.142765478 | 1.4363087 | down | mRNA |
| NM_020458 | TTC7A | 0.461689856 | 0.894667841 | 1.0851233 | down | mRNA |
| NM_020461 | TUBGCP6 | 0.52811394 | 0.918844141 | 1.0774707 | down | mRNA |
| NM_020463 | SMEK2 | 0.015280059 | 0.248240953 | 1.0868948 | down | mRNA |
| NM_020466 | LYRM2 | 0.796811936 | 0.980667273 | 1.0108426 | down | mRNA |
| NM_020468 | SNX14 | 0.175023205 | 0.653821873 | 1.2054963 | down | mRNA |
| NM_020470 | YIF1A | 0.787865931 | 0.977536468 | 1.023893 | down | mRNA |
| NM_020524 | PBXIP1 | 0.351552716 | 0.835247013 | 1.0530678 | down | mRNA |
| NM_020526 | EPHA8 | 0.97202457 | 0.998501532 | 1.003398 | down | mRNA |
| NM_020528 | PCBP3 | 0.774399625 | 0.97589756 | 1.027463 | down | mRNA |
| NM_020531 | APMAP | 0.310547588 | 0.805412658 | 1.1170102 | down | mRNA |
| NM_020532 | RTN4 | 0.062753466 | 0.439458294 | 1.0670652 | down | mRNA |
| NM_020640 | DCUN1D1 | 0.408331328 | 0.867591329 | 1.0625013 | down | mRNA |
| NM_020642 | AKIP1 | 0.227050449 | 0.722552782 | 1.0824675 | down | mRNA |
| NM_020644 | TMEM9B | 0.168879541 | 0.64469749 | 1.2319901 | down | mRNA |
| NM_020647 | JPH1 | 0.39722747 | 0.862344286 | 1.2107207 | down | mRNA |
| NM_020648 | TWSG1 | 0.98104412 | 0.998971807 | 1.0035898 | down | mRNA |
| NM_020649 | CBX8 | 0.864850468 | 0.989401142 | 1.0205254 | down | mRNA |
| NM_020650 | RCN3 | 0.009032438 | 0.202329498 | 1.0997204 | down | mRNA |
| NM_020651 | PELI1 | 0.496760525 | 0.908665955 | 1.2963741 | down | mRNA |
| NM_020653 | ZNF287 | 0.11146318 | 0.552748629 | 1.1846639 | down | mRNA |
| NM_020654 | SENP7 | 0.086614399 | 0.499870554 | 1.1287508 | down | mRNA |
| NM_020655 | JPH3 | 0.112372326 | 0.554686607 | 1.2162139 | down | mRNA |
| NM_020662 | MRS2 | 0.005196448 | 0.165084659 | 1.1753627 | down | mRNA |
| NM_020663 | RHOJ | 3.73032E-05 | 0.026153381 | 1.2924223 | down | mRNA |
| NM_020666 | CLK4 | 0.408977229 | 0.867591329 | 1.1163012 | down | mRNA |
| NM_020675 | SPC25 | 0.897682677 | 0.994326043 | 1.1177271 | down | mRNA |
| NM_020677 | NMRAL1 | 0.792634897 | 0.979003983 | 1.0320947 | down | mRNA |
| NM_020680 | SCYL1 | 0.195443231 | 0.683365329 | 1.1046536 | down | mRNA |
| NM_020690 | ANKHD1-EIF4EBP3 | 0.066697121 | 0.450526863 | 1.2170167 | down | mRNA |
| NM_020698 | TMCC3 | 0.983481458 | 0.998971807 | 1.0044134 | down | mRNA |
| NM_020699 | GATAD2B | 0.003163629 | 0.132624561 | 1.3056036 | down | mRNA |
| NM_020700 | PPM1H | 0.305687958 | 0.801330686 | 2.2062892 | down | mRNA |
| NM_020701 | ISY1 | 0.235408132 | 0.732838961 | 1.0741008 | down | mRNA |
| NM_020714 | ZNF490 | 0.045684193 | 0.39096051 | 1.372442 | down | mRNA |
| NM_020716 | GRAMD1B | 0.936766356 | 0.996710991 | 1.0036946 | down | mRNA |
| NM_020718 | USP31 | 0.305337123 | 0.801056074 | 1.1643529 | down | mRNA |
| NM_020719 | PRR12 | 0.037801508 | 0.363212377 | 1.4222502 | down | mRNA |
| NM_020728 | ESYT2 | 0.169936764 | 0.646789844 | 1.0769603 | down | mRNA |
| NM_020729 | ODF2L | 0.646937288 | 0.95550188 | 1.0647704 | down | mRNA |
| NM_020732 | ARID1B | 0.183845834 | 0.667497719 | 1.0292294 | down | mRNA |
| NM_020739 | CCPG1 | 0.790421741 | 0.978438986 | 1.0232233 | down | mRNA |
| NM_020744 | MTA3 | 0.012009195 | 0.222675671 | 1.303997 | down | mRNA |
| NM_020745 | AARS2 | 0.717179438 | 0.966191059 | 1.0837526 | down | mRNA |
| NM_020746 | MAVS | 0.21300278 | 0.705160241 | 1.0382038 | down | mRNA |
| NM_020750 | XPO5 | 0.558682961 | 0.927102643 | 1.1343811 | down | mRNA |
| NM_020751 | COG6 | 0.141969282 | 0.60204132 | 1.2190602 | down | mRNA |
| NM_020753 | CASKIN2 | 0.256709041 | 0.757240077 | 1.4724937 | down | mRNA |
| NM_020762 | SRGAP1 | 0.540379591 | 0.923577434 | 1.0524687 | down | mRNA |
| NM_020774 | MIB1 | 0.104563579 | 0.535513021 | 1.2043547 | down | mRNA |
| NM_020776 | KIAA1328 | 0.267786442 | 0.768690377 | 1.1753867 | down | mRNA |
| NM_020779 | WDR35 | 0.066550344 | 0.450298982 | 1.6017907 | down | mRNA |
| NM_020781 | ZNF398 | 0.011637278 | 0.22025265 | 2.934589 | down | mRNA |
| NM_020786 | PDP2 | 0.000661878 | 0.076919551 | 1.7850541 | down | mRNA |
| NM_020787 | ZNF624 | 0.916755323 | 0.995349793 | 1.018842 | down | mRNA |
| NM_020791 | TAOK1 | 0.367545392 | 0.845181673 | 1.1272502 | down | mRNA |
| NM_020798 | USP35 | 0.627484455 | 0.949374051 | 1.1818663 | down | mRNA |
| NM_020799 | STAMBPL1 | 0.85763902 | 0.988512246 | 1.1291786 | down | mRNA |
| NM_020800 | IFT80 | 0.225351604 | 0.720303223 | 1.310716 | down | mRNA |
| NM_020802 | KIAA1377 | 0.283246394 | 0.780770837 | 1.4513519 | down | mRNA |
| NM_020807 | ZNF319 | 0.4768511 | 0.900429473 | 1.0246368 | down | mRNA |
| NM_020810 | TRMT5 | 0.00112847 | 0.087572727 | 1.4764908 | down | mRNA |
| NM_020813 | ZNF471 | 0.331862468 | 0.822255387 | 1.1892672 | down | mRNA |
| NM_020814 | MARCH4 | 0.336827115 | 0.826617309 | 1.3680026 | down | mRNA |
| NM_020818 | UNC79 | 0.011745928 | 0.220719239 | 1.2933663 | down | mRNA |
| NM_020819 | FAM135A | 0.052469951 | 0.412637354 | 1.2852796 | down | mRNA |
| NM_020822 | KCNT1 | 0.594967147 | 0.938655001 | 1.1736387 | down | mRNA |
| NM_020823 | TMEM181 | 0.00163388 | 0.103894345 | 1.1520012 | down | mRNA |
| NM_020827 | KIAA1430 | 0.091686325 | 0.51073051 | 1.2539819 | down | mRNA |
| NM_020829 | KIAA1432 | 0.272993965 | 0.772770327 | 1.2854758 | down | mRNA |
| NM_020830 | WDFY1 | 0.040901514 | 0.375308608 | 1.2073457 | down | mRNA |
| NM_020832 | ZNF687 | 0.016862261 | 0.260717484 | 1.344781 | down | mRNA |
| NM_020839 | WDR48 | 0.203722857 | 0.693701746 | 1.1091114 | down | mRNA |
| NM_020843 | SCAPER | 0.990323152 | 0.999209835 | 1.0035185 | down | mRNA |
| NM_020844 | KIAA1456 | 0.270986033 | 0.770905786 | 1.0899313 | down | mRNA |
| NM_020846 | SLAIN2 | 0.335884802 | 0.825781112 | 1.1496192 | down | mRNA |
| NM_020854 | KIAA1468 | 0.514718284 | 0.913840427 | 1.1406515 | down | mRNA |
| NM_020856 | TSHZ3 | 0.079528507 | 0.482639336 | 1.4079467 | down | mRNA |
| NM_020861 | ZBTB2 | 0.204149456 | 0.694340371 | 1.2262805 | down | mRNA |
| NM_020863 | ZFAT | 0.169262323 | 0.645717918 | 1.3733194 | down | mRNA |
| NM_020865 | DHX36 | 0.280371339 | 0.778864608 | 1.0940266 | down | mRNA |
| NM_020866 | KLHL1 | 0.572266547 | 0.933397932 | 1.3661986 | down | mRNA |
| NM_020868 | DPP10 | 0.900163547 | 0.994782122 | 1.015437 | down | mRNA |
| NM_020870 | SH3RF1 | 0.011448193 | 0.218702965 | 1.3512154 | down | mRNA |
| NM_020871 | LRCH2 | 0.956619557 | 0.996883425 | 1.0295559 | down | mRNA |
| NM_020886 | USP28 | 0.311970471 | 0.80699254 | 1.1045722 | down | mRNA |
| NM_020888 | KIAA1522 | 0.73820298 | 0.969207218 | 1.0524947 | down | mRNA |
| NM_020890 | KIAA1524 | 0.814211636 | 0.98120545 | 1.0574591 | down | mRNA |
| NM_020901 | PHRF1 | 0.803825678 | 0.98118927 | 1.0431163 | down | mRNA |
| NM_020905 | RDH14 | 0.306624386 | 0.801653799 | 1.0319011 | down | mRNA |
| NM_020918 | GPAM | 0.026173809 | 0.316765402 | 1.8796255 | down | mRNA |
| NM_020919 | ALS2 | 0.061330636 | 0.437893235 | 1.5319987 | down | mRNA |
| NM_020920 | CHD8 | 0.996078449 | 0.999872237 | 1.0004571 | down | mRNA |
| NM_020923 | ZDBF2 | 0.209805098 | 0.700567788 | 1.1377735 | down | mRNA |
| NM_020928 | ZSWIM6 | 0.087285929 | 0.500533133 | 1.29414 | down | mRNA |
| NM_020931 | KIAA1586 | 0.057405048 | 0.426905423 | 1.5821565 | down | mRNA |
| NM_020932 | MAGEE1 | 0.743290077 | 0.970330526 | 1.1492233 | down | mRNA |
| NM_020936 | DDX55 | 0.108860486 | 0.54623677 | 1.2554557 | down | mRNA |
| NM_020937 | FANCM | 0.638247175 | 0.952852353 | 1.1316279 | down | mRNA |
| NM_020940 | FAM160B1 | 0.001702859 | 0.10523567 | 1.4388313 | down | mRNA |
| NM_020943 | CWC22 | 0.109762634 | 0.54819304 | 1.3790701 | down | mRNA |
| NM_020947 | KIAA1609 | 0.722208976 | 0.966191059 | 1.0967114 | down | mRNA |
| NM_020948 | MIER1 | 0.297537204 | 0.794005306 | 1.3651171 | down | mRNA |
| NM_020950 | KIAA1614 | 0.182900791 | 0.666166668 | 1.2008644 | down | mRNA |
| NM_020951 | ZNF529 | 0.470074111 | 0.898057982 | 1.0631033 | down | mRNA |
| NM_020960 | GPR107 | 0.433257412 | 0.880722826 | 1.0767535 | down | mRNA |
| NM_020961 | METTL14 | 0.002430959 | 0.117962433 | 1.3441952 | down | mRNA |
| NM_020964 | EPG5 | 0.630147545 | 0.950410442 | 1.0800736 | down | mRNA |
| NM_020971 | SPTBN4 | 0.832616269 | 0.98623676 | 1.0264347 | down | mRNA |
| NM_020972 | ZFYVE28 | 0.625747614 | 0.948851902 | 1.0682059 | down | mRNA |
| NM_020974 | SCUBE2 | 0.068792751 | 0.455285558 | 1.4054367 | down | mRNA |
| NM_020980 | AQP9 | 0.865072897 | 0.989401142 | 1.0311134 | down | mRNA |
| NM_020987 | ANK3 | 0.773588456 | 0.97589756 | 1.2299439 | down | mRNA |
| NM_020992 | PDLIM1 | 0.920799964 | 0.995349793 | 1.0838276 | down | mRNA |
| NM_020998 | MST1 | 0.957176791 | 0.996883425 | 1.0142043 | down | mRNA |
| NM_021004 | DHRS4 | 0.698115553 | 0.962441377 | 1.052201 | down | mRNA |
| NM_021005 | NR2F2 | 0.059502222 | 0.432586127 | 1.8615177 | down | mRNA |
| NM_021012 | KCNJ12 | 0.431662869 | 0.880121832 | 1.3894061 | down | mRNA |
| NM_021013 | KRT34 | 0.386374408 | 0.855747763 | 1.0980098 | down | mRNA |
| NM_021016 | PSG3 | 0.960416858 | 0.997738713 | 1.0299956 | down | mRNA |
| NM_021021 | SNTB1 | 0.464945421 | 0.895026539 | 1.2363696 | down | mRNA |
| NM_021033 | RAP2A | 0.100562426 | 0.527375612 | 1.1812526 | down | mRNA |
| NM_021038 | MBNL1 | 0.171671764 | 0.649425612 | 1.1992023 | down | mRNA |
| NM_021045 | ZNF248 | 0.029927814 | 0.335627308 | 1.97948 | down | mRNA |
| NM_021047 | ZNF253 | 0.009178635 | 0.203383174 | 1.3371679 | down | mRNA |
| NM_021066 | HIST1H2AJ | 0.966399764 | 0.99793927 | 1.0444271 | down | mRNA |
| NM_021067 | GINS1 | 0.844961639 | 0.987338076 | 1.1418605 | down | mRNA |
| NM_021070 | LTBP3 | 0.758435268 | 0.972072638 | 1.040621 | down | mRNA |
| NM_021072 | HCN1 | 0.812931056 | 0.98120545 | 1.1314417 | down | mRNA |
| NM_021075 | NDUFV3 | 0.020646401 | 0.286034385 | 1.1836922 | down | mRNA |
| NM_021079 | NMT1 | 0.59881739 | 0.940648177 | 1.0404726 | down | mRNA |
| NM_021081 | GHRH | 0.403051021 | 0.864786348 | 1.0978525 | down | mRNA |
| NM_021090 | MTMR3 | 0.483015477 | 0.902654749 | 1.1898359 | down | mRNA |
| NM_021095 | SLC5A6 | 0.536485931 | 0.922509728 | 1.0982262 | down | mRNA |
| NM_021098 | CACNA1H | 0.790422012 | 0.978438986 | 1.0685394 | down | mRNA |
| NM_021100 | NFS1 | 0.00990559 | 0.207085174 | 1.432281 | down | mRNA |
| NM_021105 | PLSCR1 | 0.804535358 | 0.98118927 | 1.0517806 | down | mRNA |
| NM_021107 | MRPS12 | 0.575987498 | 0.933656777 | 1.0688848 | down | mRNA |
| NM_021111 | RECK | 0.002190431 | 0.11120755 | 1.2281604 | down | mRNA |
| NM_021130 | PPIA | 0.45975287 | 0.892978158 | 1.2103704 | down | mRNA |
| NM_021132 | PPP3CB | 0.51285738 | 0.913297047 | 1.0568082 | down | mRNA |
| NM_021133 | RNASEL | 0.313163073 | 0.808636478 | 1.1719559 | down | mRNA |
| NM_021135 | RPS6KA2 | 0.077578503 | 0.478499021 | 1.2680173 | down | mRNA |
| NM_021141 | XRCC5 | 0.96339893 | 0.99793927 | 1.0038516 | down | mRNA |
| NM_021143 | ZNF20 | 0.012751243 | 0.230247141 | 1.2538264 | down | mRNA |
| NM_021148 | ZNF273 | 0.716609949 | 0.966191059 | 1.1345774 | down | mRNA |
| NM_021151 | CROT | 0.083317912 | 0.49216147 | 1.1921768 | down | mRNA |
| NM_021154 | PSAT1 | 0.570837067 | 0.93297904 | 1.1899044 | down | mRNA |
| NM_021156 | TMX4 | 0.8398717 | 0.987068842 | 1.0146692 | down | mRNA |
| NM_021159 | RAP1GDS1 | 0.038200081 | 0.365047918 | 1.2111624 | down | mRNA |
| NM_021167 | GATAD1 | 0.128189491 | 0.581774271 | 1.1516714 | down | mRNA |
| NM_021168 | RAB40C | 0.452244599 | 0.888854908 | 1.0855 | down | mRNA |
| NM_021173 | POLD4 | 0.438531712 | 0.882895421 | 1.1870442 | down | mRNA |
| NM_021177 | LSM2 | 0.215839872 | 0.710547135 | 1.147517 | down | mRNA |
| NM_021178 | CCNB1IP1 | 0.996981343 | 0.999889342 | 1.0009302 | down | mRNA |
| NM_021188 | ZNF410 | 0.217738362 | 0.711859845 | 1.1136792 | down | mRNA |
| NM_021190 | PTBP2 | 0.321408794 | 0.815220381 | 1.0942182 | down | mRNA |
| NM_021193 | HOXD12 | 0.707848793 | 0.965436846 | 1.0806858 | down | mRNA |
| NM_021194 | SLC30A1 | 0.027991065 | 0.325580278 | 1.2939483 | down | mRNA |
| NM_021202 | TP53INP2 | 0.803825006 | 0.98118927 | 1.0277069 | down | mRNA |
| NM_021203 | SRPRB | 0.807205435 | 0.98118927 | 1.017093 | down | mRNA |
| NM_021204 | ENOPH1 | 0.247126682 | 0.745152522 | 1.1889423 | down | mRNA |
| NM_021210 | TRAPPC1 | 0.568611224 | 0.932312606 | 1.0572186 | down | mRNA |
| NM_021213 | PCTP | 0.232471868 | 0.730317988 | 1.3872605 | down | mRNA |
| NM_021214 | FAM108C1 | 2.45118E-06 | 0.008848943 | 1.8986193 | down | mRNA |
| NM_021217 | ZNF77 | 0.262488063 | 0.763125731 | 1.1941018 | down | mRNA |
| NM_021218 | INIP | 0.121804514 | 0.573536217 | 1.1714887 | down | mRNA |
| NM_021223 | MYL7 | 0.452106719 | 0.888854908 | 1.1581566 | down | mRNA |
| NM_021225 | PROL1 | 0.997522354 | 0.999889342 | 1.0001847 | down | mRNA |
| NM_021226 | ARHGAP22 | 0.976406612 | 0.998501532 | 1.0031184 | down | mRNA |
| NM_021227 | OSTC | 0.532767683 | 0.92048203 | 1.0263692 | down | mRNA |
| NM_021228 | SCAF1 | 0.223876236 | 0.717844447 | 1.1300042 | down | mRNA |
| NM_021239 | RBM25 | 0.097090926 | 0.519544195 | 1.1455674 | down | mRNA |
| NM_021240 | DMRT3 | 0.377728957 | 0.851156505 | 1.539497 | down | mRNA |
| NM_021243 | ABRACL | 0.034748961 | 0.352074963 | 1.1979074 | down | mRNA |
| NM_021248 | CDH22 | 0.854752347 | 0.988178307 | 1.0584265 | down | mRNA |
| NM_021249 | SNX6 | 0.988106988 | 0.999140676 | 1.0017249 | down | mRNA |
| NM_021252 | RAB18 | 0.336097986 | 0.826105447 | 1.1292402 | down | mRNA |
| NM_021254 | C21orf59 | 0.067888706 | 0.452116989 | 1.1379258 | down | mRNA |
| NM_021258 | IL22RA1 | 0.660879897 | 0.958354006 | 1.1827275 | down | mRNA |
| NM_021260 | ZFYVE1 | 0.129802553 | 0.583907255 | 1.2032058 | down | mRNA |
| NM_021267 | CERS1 | 0.220055609 | 0.715236067 | 1.3251317 | down | mRNA |
| NM_021570 | BARX1 | 0.628373117 | 0.949894589 | 1.0613151 | down | mRNA |
| NM_021574 | BCR | 0.716150445 | 0.966191059 | 1.1151929 | down | mRNA |
| NM_021599 | ADAMTS2 | 0.903846887 | 0.99508468 | 1.0073506 | down | mRNA |
| NM_021615 | CHST6 | 0.427407643 | 0.876897294 | 1.5192665 | down | mRNA |
| NM_021626 | SCPEP1 | 0.602675782 | 0.941464987 | 1.1755285 | down | mRNA |
| NM_021627 | SENP2 | 0.430759474 | 0.879381002 | 1.0836679 | down | mRNA |
| NM_021629 | GNB4 | 0.963283488 | 0.99793927 | 1.0045004 | down | mRNA |
| NM_021630 | PDLIM2 | 0.408792685 | 0.867591329 | 1.2033513 | down | mRNA |
| NM_021633 | KLHL12 | 0.138668708 | 0.598141596 | 1.1145729 | down | mRNA |
| NM_021639 | GPBP1L1 | 0.193017851 | 0.680109784 | 1.2295651 | down | mRNA |
| NM_021640 | C12orf10 | 0.036926038 | 0.359628653 | 1.1103946 | down | mRNA |
| NM_021641 | ADAM12 | 0.615928476 | 0.945912508 | 1.1076831 | down | mRNA |
| NM_021643 | TRIB2 | 0.001160659 | 0.088716222 | 2.6458516 | down | mRNA |
| NM_021645 | UTP14C | 0.128973829 | 0.582929618 | 1.1312832 | down | mRNA |
| NM_021648 | TSPYL4 | 0.22461911 | 0.719318906 | 1.1635154 | down | mRNA |
| NM_021705 | NFYA | 0.0443958 | 0.387186892 | 1.2075737 | down | mRNA |
| NM_021709 | SIVA1 | 0.71607894 | 0.966191059 | 1.0947853 | down | mRNA |
| NM_021724 | NR1D1 | 0.498238443 | 0.909116151 | 1.3132639 | down | mRNA |
| NM_021732 | AVPI1 | 0.014503644 | 0.242706255 | 1.5899132 | down | mRNA |
| NM_021777 | ADAM28 | 0.685312601 | 0.960111041 | 1.0275638 | down | mRNA |
| NM_021794 | ADAM30 | 0.905610399 | 0.99508468 | 1.0212144 | down | mRNA |
| NM_021797 | CHIA | 0.994046081 | 0.999872237 | 1.0009836 | down | mRNA |
| NM_021801 | MMP26 | 0.244100931 | 0.742391426 | 1.1350454 | down | mRNA |
| NM_021807 | EXOC4 | 0.209955245 | 0.700729014 | 1.1933087 | down | mRNA |
| NM_021814 | ELOVL5 | 0.528426656 | 0.918883792 | 1.0823944 | down | mRNA |
| NM_021819 | LMAN1L | 0.806176574 | 0.98118927 | 1.0194078 | down | mRNA |
| NM_021821 | MRPS35 | 0.159944093 | 0.631698386 | 1.1351306 | down | mRNA |
| NM_021823 | PPCDC | 0.230703794 | 0.728071705 | 1.0790569 | down | mRNA |
| NM_021830 | C10orf2 | 0.29379295 | 0.791146775 | 1.2635141 | down | mRNA |
| NM_021871 | FGA | 0.382072549 | 0.853514109 | 1.0962991 | down | mRNA |
| NM_021872 | CDC25B | 0.905382525 | 0.99508468 | 1.0335962 | down | mRNA |
| NM_021903 | GABBR1 | 0.829083439 | 0.98552522 | 1.037559 | down | mRNA |
| NM_021907 | DTNB | 0.664259777 | 0.958354006 | 1.1523361 | down | mRNA |
| NM_021912 | GABRB3 | 0.466769817 | 0.896044904 | 1.0598533 | down | mRNA |
| NM_021914 | CFL2 | 0.209956775 | 0.700729014 | 1.0836109 | down | mRNA |
| NM_021915 | ZNF69 | 0.195617938 | 0.683572955 | 1.0907524 | down | mRNA |
| NM_021922 | FANCE | 0.52290699 | 0.917239107 | 1.0800295 | down | mRNA |
| NM_021925 | C2orf43 | 0.226837579 | 0.722135151 | 1.4508512 | down | mRNA |
| NM_021927 | GUF1 | 0.171450718 | 0.649029231 | 1.2094667 | down | mRNA |
| NM_021928 | SPCS3 | 0.753456855 | 0.970725371 | 1.0347431 | down | mRNA |
| NM_021933 | MIIP | 0.553122279 | 0.926337111 | 1.1012612 | down | mRNA |
| NM_021936 | PAPPA2 | 0.857108887 | 0.988512246 | 1.0292828 | down | mRNA |
| NM_021939 | FKBP10 | 0.013020122 | 0.233238 | 1.1077492 | down | mRNA |
| NM_021940 | SMAP1 | 0.0497184 | 0.403542715 | 1.2356454 | down | mRNA |
| NM_021942 | TRAPPC11 | 0.020256605 | 0.284825234 | 1.2811277 | down | mRNA |
| NM_021943 | ZFAND3 | 0.892880745 | 0.993978977 | 1.008653 | down | mRNA |
| NM_021947 | SRR | 0.449949301 | 0.888279993 | 1.0317956 | down | mRNA |
| NM_021948 | BCAN | 0.76457746 | 0.974143935 | 1.4023991 | down | mRNA |
| NM_021949 | ATP2B3 | 0.31063066 | 0.805412658 | 1.2732935 | down | mRNA |
| NM_021952 | ELAVL4 | 0.491723622 | 0.905806259 | 1.0858017 | down | mRNA |
| NM_021958 | HLX | 0.550915518 | 0.925960004 | 1.1612789 | down | mRNA |
| NM_021960 | MCL1 | 0.899544207 | 0.994708735 | 1.0142346 | down | mRNA |
| NM_021961 | TEAD1 | 0.349368982 | 0.834138149 | 1.1757738 | down | mRNA |
| NM_021962 | ABR | 0.75240933 | 0.970725371 | 1.1147294 | down | mRNA |
| NM_021964 | ZNF148 | 0.003619953 | 0.139792244 | 1.2420308 | down | mRNA |
| NM_021970 | LAMTOR3 | 0.510247827 | 0.912097614 | 1.0771611 | down | mRNA |
| NM_021974 | POLR2F | 0.725924812 | 0.966191059 | 1.0251597 | down | mRNA |
| NM_021976 | RXRB | 0.048456398 | 0.39968174 | 1.1764642 | down | mRNA |
| NM_021988 | UBE2V1 | 0.603398996 | 0.941484604 | 1.0253063 | down | mRNA |
| NM_021992 | TMSB15A | 0.876813257 | 0.991255275 | 1.0578746 | down | mRNA |
| NM_021994 | ZNF277 | 0.568691055 | 0.932312606 | 1.0937571 | down | mRNA |
| NM_021996 | GBGT1 | 0.545329022 | 0.924012779 | 1.1105211 | down | mRNA |
| NM_022037 | TIA1 | 0.322047268 | 0.815372756 | 1.1048786 | down | mRNA |
| NM_022041 | GAN | 0.520618226 | 0.916605148 | 1.239894 | down | mRNA |
| NM_022044 | SDF2L1 | 0.420253574 | 0.872365335 | 1.1471998 | down | mRNA |
| NM_022045 | MTBP | 0.476739476 | 0.900429473 | 1.1806272 | down | mRNA |
| NM_022048 | CSNK1G1 | 0.128389598 | 0.582032843 | 1.1609282 | down | mRNA |
| NM_022049 | GPR88 | 0.661699182 | 0.958354006 | 1.053556 | down | mRNA |
| NM_022060 | ABHD4 | 0.78155905 | 0.976938981 | 1.0917698 | down | mRNA |
| NM_022061 | MRPL17 | 0.336427336 | 0.826506012 | 1.1155104 | down | mRNA |
| NM_022064 | RNF123 | 0.349151393 | 0.834138149 | 1.1560195 | down | mRNA |
| NM_022066 | UBE2O | 0.549104721 | 0.925430801 | 1.0674334 | down | mRNA |
| NM_022070 | HEATR6 | 0.075449608 | 0.471918185 | 1.1249328 | down | mRNA |
| NM_022072 | NSUN3 | 0.016002313 | 0.254584533 | 1.2395088 | down | mRNA |
| NM_022079 | HERC4 | 0.50444612 | 0.910568383 | 1.1754955 | down | mRNA |
| NM_022081 | HPS4 | 0.119387207 | 0.568739618 | 1.2559539 | down | mRNA |
| NM_022087 | GALNT11 | 0.748567009 | 0.970725371 | 1.0232474 | down | mRNA |
| NM_022088 | ZFP64 | 0.596373052 | 0.939300503 | 1.0951108 | down | mRNA |
| NM_022089 | ATP13A2 | 0.159942205 | 0.631698386 | 1.803161 | down | mRNA |
| NM_022090 | C5orf54 | 0.326360624 | 0.819345076 | 1.1180024 | down | mRNA |
| NM_022098 | XPNPEP3 | 0.023566686 | 0.301884214 | 1.3581195 | down | mRNA |
| NM_022103 | ZNF667 | 0.436163823 | 0.881694456 | 1.1608935 | down | mRNA |
| NM_022104 | PCIF1 | 0.596417937 | 0.939300503 | 1.0755687 | down | mRNA |
| NM_022106 | FAM217B | 0.038018033 | 0.364270808 | 1.2698658 | down | mRNA |
| NM_022110 | FKBPL | 0.050842494 | 0.407751844 | 1.7748579 | down | mRNA |
| NM_022111 | CLSPN | 0.945368805 | 0.996710991 | 1.0524479 | down | mRNA |
| NM_022113 | KIF13A | 0.272530763 | 0.772493443 | 1.1664132 | down | mRNA |
| NM_022114 | PRDM16 | 0.866676757 | 0.989481868 | 1.0818972 | down | mRNA |
| NM_022116 | FIGNL1 | 0.07706443 | 0.477122268 | 1.9183654 | down | mRNA |
| NM_022117 | TSPYL2 | 0.99216057 | 0.999743458 | 1.000806 | down | mRNA |
| NM_022118 | RBM26 | 0.041637977 | 0.377938997 | 1.2071108 | down | mRNA |
| NM_022120 | OXCT2 | 0.2586068 | 0.759358563 | 1.1759307 | down | mRNA |
| NM_022126 | LHPP | 0.625664631 | 0.948851902 | 1.1642409 | down | mRNA |
| NM_022130 | GOLPH3 | 0.143274249 | 0.603450317 | 1.357369 | down | mRNA |
| NM_022133 | SNX16 | 0.410186361 | 0.86807846 | 1.0879027 | down | mRNA |
| NM_022134 | GAL3ST2 | 0.350747926 | 0.834911159 | 1.3263575 | down | mRNA |
| NM_022137 | SMOC1 | 0.93480937 | 0.996710991 | 1.0351305 | down | mRNA |
| NM_022143 | LRRC4 | 0.8511473 | 0.987678166 | 1.0159901 | down | mRNA |
| NM_022149 | MAGEF1 | 0.020118721 | 0.284065167 | 1.1856356 | down | mRNA |
| NM_022151 | MOAP1 | 0.061528095 | 0.437893235 | 1.3190748 | down | mRNA |
| NM_022156 | DUS1L | 0.530591535 | 0.919193443 | 1.0627542 | down | mRNA |
| NM_022158 | FN3K | 0.388408102 | 0.857898453 | 1.343317 | down | mRNA |
| NM_022161 | BIRC7 | 0.040446894 | 0.372623133 | 1.2558887 | down | mRNA |
| NM_022163 | MRPL46 | 0.963956425 | 0.99793927 | 1.0103565 | down | mRNA |
| NM_022167 | XYLT2 | 0.834574574 | 0.986575022 | 1.0127741 | down | mRNA |
| NM_022173 | TIA1 | 0.078844209 | 0.481399537 | 1.1600861 | down | mRNA |
| NM_022334 | ITGB1BP1 | 0.485030347 | 0.903250293 | 1.073485 | down | mRNA |
| NM_022338 | C11orf24 | 0.115308505 | 0.560638489 | 1.2892257 | down | mRNA |
| NM_022342 | KIF9 | 0.573370374 | 0.933656777 | 1.1338464 | down | mRNA |
| NM_022346 | NCAPG | 0.940538074 | 0.996710991 | 1.0530561 | down | mRNA |
| NM_022353 | OSGEPL1 | 0.147437643 | 0.609973207 | 1.1623544 | down | mRNA |
| NM_022356 | LEPRE1 | 0.175189653 | 0.653821873 | 1.2162895 | down | mRNA |
| NM_022359 | PDE4DIP | 0.92488454 | 0.995456159 | 1.0235987 | down | mRNA |
| NM_022366 | TFB2M | 0.052651245 | 0.413038171 | 1.1736756 | down | mRNA |
| NM_022368 | PJA1 | 0.626092199 | 0.948986625 | 1.1183989 | down | mRNA |
| NM_022371 | TOR3A | 0.005306652 | 0.16676175 | 1.2922969 | down | mRNA |
| NM_022373 | HERPUD2 | 0.474791669 | 0.899586638 | 1.0468175 | down | mRNA |
| NM_022374 | ATL2 | 0.059954981 | 0.434582768 | 1.2309567 | down | mRNA |
| NM_022406 | XRCC4 | 0.337982454 | 0.827394958 | 1.0910667 | down | mRNA |
| NM_022443 | MLF1 | 0.755895848 | 0.971522741 | 1.1491356 | down | mRNA |
| NM_022450 | RHBDF1 | 0.041936805 | 0.378937196 | 1.1255905 | down | mRNA |
| NM_022453 | RNF25 | 0.042951147 | 0.382891781 | 1.1947737 | down | mRNA |
| NM_022455 | NSD1 | 0.013569355 | 0.235988767 | 1.4865163 | down | mRNA |
| NM_022457 | RFWD2 | 0.841751927 | 0.987123192 | 1.018213 | down | mRNA |
| NM_022458 | LMBR1 | 0.19876426 | 0.687087939 | 1.1719549 | down | mRNA |
| NM_022461 | AZI2 | 0.644048939 | 0.954605197 | 1.1146531 | down | mRNA |
| NM_022466 | IKZF5 | 0.155514666 | 0.623056705 | 1.2068281 | down | mRNA |
| NM_022467 | CHST8 | 0.058186404 | 0.429558128 | 1.3644121 | down | mRNA |
| NM_022469 | GREM2 | 0.358436968 | 0.840546205 | 1.0772014 | down | mRNA |
| NM_022470 | ZMAT3 | 0.585328491 | 0.934708084 | 1.2058808 | down | mRNA |
| NM_022474 | MPP5 | 0.676702242 | 0.959531882 | 1.0512979 | down | mRNA |
| NM_022475 | HHIP | 0.181751643 | 0.664924727 | 1.1550426 | down | mRNA |
| NM_022478 | CDH24 | 0.116179578 | 0.562521166 | 1.2200311 | down | mRNA |
| NM_022479 | WBSCR17 | 0.640435649 | 0.953894897 | 1.0756018 | down | mRNA |
| NM_022483 | C5orf28 | 0.434443233 | 0.880931467 | 1.1336351 | down | mRNA |
| NM_022484 | TMEM168 | 0.036127006 | 0.356565658 | 1.2781044 | down | mRNA |
| NM_022485 | MTMR14 | 0.024369874 | 0.304929683 | 1.2142262 | down | mRNA |
| NM_022486 | SUSD1 | 0.579731451 | 0.933893479 | 1.0863435 | down | mRNA |
| NM_022487 | DCLRE1C | 0.023257588 | 0.300022844 | 1.3577582 | down | mRNA |
| NM_022488 | ATG3 | 0.109009566 | 0.54671497 | 1.137426 | down | mRNA |
| NM_022490 | POLR1E | 0.108003076 | 0.544140502 | 1.4021759 | down | mRNA |
| NM_022491 | SUDS3 | 0.954332232 | 0.996710991 | 1.0080834 | down | mRNA |
| NM_022492 | TTC31 | 0.690520078 | 0.961105466 | 1.0629289 | down | mRNA |
| NM_022493 | NARFL | 0.025750123 | 0.315012939 | 1.3190782 | down | mRNA |
| NM_022495 | PCNXL4 | 0.059530591 | 0.432586127 | 1.4170043 | down | mRNA |
| NM_022496 | ACTR6 | 0.303993225 | 0.799797576 | 1.1035243 | down | mRNA |
| NM_022552 | DNMT3A | 0.925748788 | 0.995677477 | 1.0063289 | down | mRNA |
| NM_022567 | NYX | 0.266313016 | 0.767064488 | 1.1027791 | down | mRNA |
| NM_022571 | GPR135 | 0.019952294 | 0.283521851 | 1.529564 | down | mRNA |
| NM_022572 | PNKD | 0.975668915 | 0.998501532 | 1.0063935 | down | mRNA |
| NM_022573 | TSPY2 | 0.270799372 | 0.77080958 | 1.4940083 | down | mRNA |
| NM_022648 | TNS1 | 0.13867367 | 0.598141596 | 1.0957791 | down | mRNA |
| NM_022650 | RASA1 | 0.000343463 | 0.057713112 | 2.1398724 | down | mRNA |
| NM_022658 | HOXC8 | 0.416074236 | 0.870736736 | 1.0680513 | down | mRNA |
| NM_022662 | ANAPC1 | 0.067976191 | 0.452403247 | 1.196016 | down | mRNA |
| NM_022664 | ECM1 | 0.741664855 | 0.970240145 | 1.0390974 | down | mRNA |
| NM_022717 | SNRNP35 | 0.986692048 | 0.99909491 | 1.0013512 | down | mRNA |
| NM_022719 | DGCR14 | 0.350177973 | 0.834344847 | 1.0736379 | down | mRNA |
| NM_022726 | ELOVL4 | 0.15079942 | 0.615920812 | 1.3185132 | down | mRNA |
| NM_022730 | COPS7B | 0.260714221 | 0.761396372 | 1.147517 | down | mRNA |
| NM_022731 | NUCKS1 | 0.268126915 | 0.768907819 | 1.0998979 | down | mRNA |
| NM_022734 | METTL17 | 0.209081352 | 0.700221058 | 1.218059 | down | mRNA |
| NM_022735 | ACBD3 | 0.037071635 | 0.359628653 | 1.3298351 | down | mRNA |
| NM_022736 | MFSD1 | 0.377358569 | 0.850887703 | 1.0463317 | down | mRNA |
| NM_022737 | LPPR2 | 0.147153136 | 0.609351845 | 1.0900273 | down | mRNA |
| NM_022746 | MARC1 | 0.395233297 | 0.861590143 | 1.2081864 | down | mRNA |
| NM_022751 | FAM59A | 0.080109991 | 0.483876854 | 1.469546 | down | mRNA |
| NM_022752 | ZNF574 | 0.348067855 | 0.834138149 | 1.133446 | down | mRNA |
| NM_022753 | S100PBP | 0.042709925 | 0.382084661 | 1.1865797 | down | mRNA |
| NM_022754 | SFXN1 | 0.480251136 | 0.901538317 | 1.1371636 | down | mRNA |
| NM_022756 | MEAF6 | 0.13672661 | 0.595655761 | 1.4928078 | down | mRNA |
| NM_022758 | C6orf106 | 0.764807633 | 0.97415819 | 1.026597 | down | mRNA |
| NM_022762 | RMND5B | 0.392998262 | 0.859795559 | 1.0645972 | down | mRNA |
| NM_022765 | MICAL1 | 0.259815147 | 0.760388386 | 1.3893408 | down | mRNA |
| NM_022766 | CERK | 0.064542995 | 0.443826413 | 1.3528849 | down | mRNA |
| NM_022767 | AEN | 0.230740677 | 0.728071705 | 1.1439021 | down | mRNA |
| NM_022768 | RBM15 | 0.239991339 | 0.73834502 | 1.1634715 | down | mRNA |
| NM_022771 | TBC1D15 | 0.075457703 | 0.471918185 | 1.1845271 | down | mRNA |
| NM_022774 | DEM1 | 0.143314376 | 0.603450317 | 1.2524847 | down | mRNA |
| NM_022776 | OSBPL11 | 0.026124839 | 0.316361069 | 1.302098 | down | mRNA |
| NM_022777 | RABL5 | 0.258896004 | 0.759358563 | 1.1394565 | down | mRNA |
| NM_022779 | DDX31 | 0.020673208 | 0.286034385 | 1.2257299 | down | mRNA |
| NM_022780 | RMND5A | 0.662118819 | 0.958354006 | 1.0329073 | down | mRNA |
| NM_022782 | MPHOSPH9 | 0.260031937 | 0.760388386 | 1.249513 | down | mRNA |
| NM_022783 | DEPTOR | 0.794457026 | 0.979333052 | 1.0919241 | down | mRNA |
| NM_022785 | EFCAB6 | 0.985857719 | 0.998971807 | 1.0027296 | down | mRNA |
| NM_022786 | ARV1 | 0.186367725 | 0.669888134 | 1.2099474 | down | mRNA |
| NM_022802 | CTBP2 | 0.497253832 | 0.908789022 | 1.0526788 | down | mRNA |
| NM_022803 | UCP3 | 0.719516483 | 0.966191059 | 1.1123922 | down | mRNA |
| NM_022804 | SNURF | 0.610039429 | 0.943169861 | 1.0939356 | down | mRNA |
| NM_022810 | SLC25A14 | 0.90799051 | 0.995150496 | 1.0126865 | down | mRNA |
| NM_022818 | MAP1LC3B | 0.758111316 | 0.972072638 | 1.1065895 | down | mRNA |
| NM_022820 | CYP3A43 | 0.700866501 | 0.962991468 | 1.164054 | down | mRNA |
| NM_022821 | ELOVL1 | 0.222943841 | 0.71761506 | 1.2145616 | down | mRNA |
| NM_022823 | FNDC4 | 0.729207341 | 0.966191059 | 1.0560648 | down | mRNA |
| NM_022825 | PORCN | 0.917656451 | 0.995349793 | 1.0262065 | down | mRNA |
| NM_022829 | SLC13A3 | 0.50597176 | 0.911278043 | 1.0396019 | down | mRNA |
| NM_022831 | AIDA | 0.835280329 | 0.986575022 | 1.018992 | down | mRNA |
| NM_022832 | USP46 | 0.610473022 | 0.943314902 | 1.068639 | down | mRNA |
| NM_022833 | FAM129B | 0.722625792 | 0.966191059 | 1.0760121 | down | mRNA |
| NM_022835 | PLEKHG2 | 0.811081306 | 0.98118927 | 1.0369467 | down | mRNA |
| NM_022836 | DCLRE1B | 0.500595172 | 0.909680616 | 1.0573345 | down | mRNA |
| NM_022841 | RFX7 | 0.172536378 | 0.65011298 | 1.4189828 | down | mRNA |
| NM_022842 | CDCP1 | 0.486598827 | 0.903524001 | 1.282884 | down | mRNA |
| NM_022845 | CBFB | 0.002438911 | 0.118066527 | 1.618677 | down | mRNA |
| NM_022875 | SMN2 | 0.456778179 | 0.891568474 | 1.1576469 | down | mRNA |
| NM_022876 | SMN2 | 0.362782769 | 0.843238468 | 1.0579922 | down | mRNA |
| NM_022892 | NAIP | 0.316111373 | 0.810795565 | 1.2875784 | down | mRNA |
| NM_022897 | RANBP17 | 0.381103369 | 0.853514109 | 1.0789368 | down | mRNA |
| NM_022898 | BCL11B | 0.71988987 | 0.966191059 | 1.2312245 | down | mRNA |
| NM_022899 | ACTR8 | 0.046912966 | 0.394576676 | 1.1716056 | down | mRNA |
| NM_022900 | CASD1 | 0.300947684 | 0.795950279 | 1.2139691 | down | mRNA |
| NM_022902 | SLC30A5 | 0.033459327 | 0.347621377 | 1.3237719 | down | mRNA |
| NM_022903 | CCDC71 | 0.628038513 | 0.949671207 | 1.0833506 | down | mRNA |
| NM_022913 | GPBP1 | 0.208696064 | 0.699852938 | 1.1490515 | down | mRNA |
| NM_022914 | ACD | 0.812169143 | 0.98118927 | 1.0340478 | down | mRNA |
| NM_022916 | VPS33A | 0.246187474 | 0.744847716 | 1.1987759 | down | mRNA |
| NM_022918 | TMEM135 | 0.432222405 | 0.880427272 | 1.2798864 | down | mRNA |
| NM_022963 | FGFR4 | 0.795200415 | 0.979701559 | 1.0440565 | down | mRNA |
| NM_022968 | SERF1A | 0.924556542 | 0.995456159 | 1.012837 | down | mRNA |
| NM_023000 | ARID4A | 0.206638468 | 0.698066071 | 1.4310239 | down | mRNA |
| NM_023001 | ARID4A | 0.659267889 | 0.958354006 | 1.1602254 | down | mRNA |
| NM_023010 | UPF3B | 0.467891829 | 0.897130958 | 1.2011365 | down | mRNA |
| NM_023011 | UPF3A | 0.946510869 | 0.996710991 | 1.0094198 | down | mRNA |
| NM_023012 | RSRC2 | 0.49301107 | 0.906130999 | 1.08159 | down | mRNA |
| NM_023016 | SOWAHC | 0.002769845 | 0.124594021 | 1.780597 | down | mRNA |
| NM_023019 | DCTN1 | 0.461552392 | 0.894667841 | 1.2102631 | down | mRNA |
| NM_023034 | WHSC1L1 | 0.001267185 | 0.092399617 | 1.473181 | down | mRNA |
| NM_023036 | DNAI2 | 0.954274921 | 0.996710991 | 1.0126471 | down | mRNA |
| NM_023070 | ZNF643 | 0.970515536 | 0.998245244 | 1.0072912 | down | mRNA |
| NM_023071 | SPATS2 | 0.052313212 | 0.412420403 | 1.2137352 | down | mRNA |
| NM_023074 | ZNF649 | 0.914508664 | 0.995342392 | 1.0060688 | down | mRNA |
| NM_023075 | MPPE1 | 0.303471278 | 0.79893539 | 1.0791921 | down | mRNA |
| NM_023076 | UNKL | 0.731472899 | 0.967176866 | 1.0577824 | down | mRNA |
| NM_023077 | SELRC1 | 0.004112012 | 0.146933952 | 1.809123 | down | mRNA |
| NM_023079 | UBE2Z | 0.899898658 | 0.994752372 | 1.0412629 | down | mRNA |
| NM_023080 | C8orf33 | 0.980363407 | 0.998971807 | 1.0030779 | down | mRNA |
| NM_023105 | FGFR1 | 0.270051145 | 0.770135843 | 1.0782188 | down | mRNA |
| NM_023106 | FGFR1 | 0.425763247 | 0.87545849 | 1.1202735 | down | mRNA |
| NM_023110 | FGFR1 | 0.507865562 | 0.911674633 | 1.0811846 | down | mRNA |
| NM_023112 | OTUB2 | 0.575405419 | 0.933656777 | 1.1024478 | down | mRNA |
| NM_023924 | BRD9 | 0.018227996 | 0.26991728 | 1.1400338 | down | mRNA |
| NM_023925 | CAPRIN2 | 0.229340037 | 0.726314896 | 1.0924267 | down | mRNA |
| NM_023928 | AACS | 0.139324473 | 0.598648178 | 1.1666662 | down | mRNA |
| NM_023930 | KCTD14 | 0.545573258 | 0.924167487 | 1.0548793 | down | mRNA |
| NM_023933 | FAM173A | 0.519443139 | 0.916145597 | 1.0405387 | down | mRNA |
| NM_023936 | MRPS34 | 0.579295046 | 0.933821303 | 1.0652322 | down | mRNA |
| NM_023938 | C1orf116 | 0.699263134 | 0.962701213 | 1.0275141 | down | mRNA |
| NM_023940 | RASL11B | 0.648517956 | 0.95550188 | 1.0561971 | down | mRNA |
| NM_023948 | MOSPD3 | 0.334991784 | 0.824836547 | 1.0897461 | down | mRNA |
| NM_024011 | CDK11A | 0.063149497 | 0.440012194 | 1.168369 | down | mRNA |
| NM_024012 | HTR5A | 0.830224934 | 0.985549562 | 1.0089136 | down | mRNA |
| NM_024013 | IFNA1 | 0.491300291 | 0.905806259 | 1.3944889 | down | mRNA |
| NM_024026 | MRP63 | 0.294704681 | 0.791301949 | 1.1076689 | down | mRNA |
| NM_024027 | COLEC11 | 0.646187859 | 0.95550188 | 1.2214517 | down | mRNA |
| NM_024028 | PCYOX1L | 0.697956704 | 0.962410025 | 1.0615805 | down | mRNA |
| NM_024029 | YIPF2 | 0.024087048 | 0.303619251 | 1.1732923 | down | mRNA |
| NM_024033 | C7orf49 | 0.009004302 | 0.202329498 | 1.2981182 | down | mRNA |
| NM_024036 | LRFN4 | 0.216903753 | 0.711074992 | 1.2209585 | down | mRNA |
| NM_024037 | AUNIP | 0.155709568 | 0.623328791 | 3.1922871 | down | mRNA |
| NM_024038 | C19orf43 | 0.359503425 | 0.841033672 | 1.0722242 | down | mRNA |
| NM_024039 | MIS12 | 0.491513426 | 0.905806259 | 1.2317512 | down | mRNA |
| NM_024041 | SCNM1 | 0.27864163 | 0.777783404 | 1.0959427 | down | mRNA |
| NM_024047 | NUDT9 | 0.95824637 | 0.996933183 | 1.007582 | down | mRNA |
| NM_024051 | GGCT | 0.170175184 | 0.646971174 | 1.191479 | down | mRNA |
| NM_024052 | GID4 | 0.506466682 | 0.911362118 | 1.0869867 | down | mRNA |
| NM_024053 | CENPM | 0.910786589 | 0.995342392 | 1.0200909 | down | mRNA |
| NM_024055 | SLC30A5 | 0.225218908 | 0.720245224 | 1.1491892 | down | mRNA |
| NM_024057 | NUP37 | 0.460677479 | 0.894090732 | 1.1058073 | down | mRNA |
| NM_024065 | PDCL3 | 0.240595547 | 0.738717708 | 1.124809 | down | mRNA |
| NM_024066 | ERI3 | 0.208963744 | 0.700205031 | 1.1742653 | down | mRNA |
| NM_024067 | C7orf26 | 0.475076522 | 0.89971438 | 1.1602132 | down | mRNA |
| NM_024068 | NABP2 | 0.445115409 | 0.885731305 | 1.0953542 | down | mRNA |
| NM_024071 | ZFYVE21 | 0.209891713 | 0.700729014 | 1.1855708 | down | mRNA |
| NM_024072 | DDX54 | 0.014124579 | 0.239317452 | 1.2094175 | down | mRNA |
| NM_024074 | TMEM38A | 0.32290409 | 0.815766149 | 1.1296534 | down | mRNA |
| NM_024075 | TSEN34 | 0.242459101 | 0.740636784 | 1.3055212 | down | mRNA |
| NM_024076 | KCTD15 | 0.004254514 | 0.148630199 | 1.3461405 | down | mRNA |
| NM_024077 | SECISBP2 | 0.300369348 | 0.795797471 | 1.1061814 | down | mRNA |
| NM_024079 | ALG8 | 0.979115589 | 0.998864935 | 1.0037022 | down | mRNA |
| NM_024086 | METTL16 | 0.246023517 | 0.744589184 | 1.0962176 | down | mRNA |
| NM_024089 | KDELC1 | 0.117590764 | 0.564147103 | 1.1775481 | down | mRNA |
| NM_024091 | FASTKD3 | 0.02412666 | 0.303741948 | 1.8636264 | down | mRNA |
| NM_024092 | TMEM109 | 0.360454559 | 0.841780454 | 1.0873603 | down | mRNA |
| NM_024093 | C2orf49 | 0.002496418 | 0.118591515 | 1.3721603 | down | mRNA |
| NM_024094 | DSCC1 | 0.336591983 | 0.826506012 | 1.2042887 | down | mRNA |
| NM_024095 | ASB8 | 0.01556028 | 0.250096132 | 1.3114927 | down | mRNA |
| NM_024096 | DCTPP1 | 0.478191878 | 0.901271944 | 1.2184518 | down | mRNA |
| NM_024097 | C1orf50 | 0.548248441 | 0.92514258 | 1.0465968 | down | mRNA |
| NM_024102 | WDR77 | 0.865446532 | 0.989401142 | 1.0214881 | down | mRNA |
| NM_024104 | C19orf42 | 0.029637967 | 0.334777298 | 1.1360829 | down | mRNA |
| NM_024105 | ALG12 | 0.126371872 | 0.579213911 | 1.2855658 | down | mRNA |
| NM_024107 | TMUB2 | 0.714329903 | 0.966110889 | 1.0306204 | down | mRNA |
| NM_024108 | TRAPPC6A | 0.677606417 | 0.959531882 | 1.093541 | down | mRNA |
| NM_024109 | METTL22 | 0.117997291 | 0.564712647 | 1.2556557 | down | mRNA |
| NM_024111 | CHAC1 | 0.938401314 | 0.996710991 | 1.0121854 | down | mRNA |
| NM_024113 | C11orf49 | 0.793177452 | 0.979003983 | 1.0202765 | down | mRNA |
| NM_024116 | TAF1D | 0.697203053 | 0.962410025 | 1.0285705 | down | mRNA |
| NM_024117 | MAPKAP1 | 0.488025724 | 0.903757389 | 1.0556408 | down | mRNA |
| NM_024120 | NDUFAF5 | 0.228176268 | 0.724321605 | 1.1445784 | down | mRNA |
| NM_024121 | TMEM185B | 0.479738226 | 0.901538317 | 1.1124604 | down | mRNA |
| NM_024164 | TPSB2 | 0.35958201 | 0.841120736 | 1.0777989 | down | mRNA |
| NM_024165 | PHF1 | 0.561467161 | 0.928513767 | 1.0756525 | down | mRNA |
| NM_024294 | C6orf106 | 0.669816042 | 0.959531882 | 1.0303762 | down | mRNA |
| NM_024295 | DERL1 | 0.637656441 | 0.952805964 | 1.0335625 | down | mRNA |
| NM_024298 | MBOAT7 | 0.014791869 | 0.244114105 | 1.2470561 | down | mRNA |
| NM_024299 | PPDPF | 0.864300825 | 0.989401142 | 1.0428689 | down | mRNA |
| NM_024307 | GDPD3 | 0.273063762 | 0.772770327 | 1.112332 | down | mRNA |
| NM_024310 | PLEKHF1 | 0.046134438 | 0.391970965 | 1.3278535 | down | mRNA |
| NM_024315 | C7orf23 | 0.281615157 | 0.779126326 | 1.0861545 | down | mRNA |
| NM_024319 | C1orf35 | 0.166138326 | 0.640243452 | 1.3130368 | down | mRNA |
| NM_024324 | CRELD2 | 0.630603686 | 0.950410442 | 1.1053087 | down | mRNA |
| NM_024325 | ZNF343 | 0.219708464 | 0.714395088 | 1.1669576 | down | mRNA |
| NM_024327 | ZNF576 | 0.217323018 | 0.711339274 | 1.1245866 | down | mRNA |
| NM_024332 | BRCC3 | 0.706949303 | 0.965401045 | 1.1096607 | down | mRNA |
| NM_024334 | TMEM43 | 0.647710888 | 0.95550188 | 1.0594717 | down | mRNA |
| NM_024336 | IRX3 | 0.194808678 | 0.682531124 | 1.6018982 | down | mRNA |
| NM_024339 | THOC6 | 0.01279442 | 0.230412891 | 1.1781542 | down | mRNA |
| NM_024344 | CAPN3 | 0.277050834 | 0.777205298 | 1.1108861 | down | mRNA |
| NM_024345 | DCAF10 | 0.474610907 | 0.899495615 | 1.0556487 | down | mRNA |
| NM_024348 | DCTN3 | 0.613280199 | 0.944565792 | 1.0990379 | down | mRNA |
| NM_024409 | NPPC | 0.966258303 | 0.99793927 | 1.0116534 | down | mRNA |
| NM_024418 | BZRAP1 | 0.700839246 | 0.962991468 | 1.04881 | down | mRNA |
| NM_024419 | PGS1 | 0.5027794 | 0.910354901 | 1.243268 | down | mRNA |
| NM_024490 | ATP10A | 0.604936646 | 0.94174007 | 1.1716136 | down | mRNA |
| NM_024491 | CEP70 | 0.39030223 | 0.858920947 | 1.0792627 | down | mRNA |
| NM_024493 | ZKSCAN3 | 0.136176285 | 0.594906342 | 1.2774806 | down | mRNA |
| NM_024496 | IRF2BPL | 0.022544959 | 0.295888941 | 1.2288536 | down | mRNA |
| NM_024506 | GLB1L | 0.057254396 | 0.426252791 | 1.2543554 | down | mRNA |
| NM_024507 | KREMEN2 | 0.417431388 | 0.871363651 | 1.057369 | down | mRNA |
| NM_024509 | LRFN3 | 0.572645245 | 0.933460721 | 1.1422847 | down | mRNA |
| NM_024511 | HAUS3 | 0.551396311 | 0.926149092 | 1.1694907 | down | mRNA |
| NM_024512 | LRRC2 | 0.023130631 | 0.299167937 | 1.2010199 | down | mRNA |
| NM_024513 | FYCO1 | 0.956957965 | 0.996883425 | 1.0191681 | down | mRNA |
| NM_024516 | C16orf53 | 0.013469432 | 0.235533862 | 1.2135487 | down | mRNA |
| NM_024520 | C2orf47 | 0.01380555 | 0.236813499 | 1.1798008 | down | mRNA |
| NM_024523 | GCC1 | 0.325890301 | 0.818530154 | 1.1317595 | down | mRNA |
| NM_024525 | TTC13 | 5.39172E-05 | 0.030102801 | 2.2290777 | down | mRNA |
| NM_024527 | ABHD8 | 0.433910737 | 0.880923255 | 1.0804469 | down | mRNA |
| NM_024528 | NKAP | 0.208063054 | 0.698793461 | 1.0602347 | down | mRNA |
| NM_024529 | CDC73 | 0.292161484 | 0.788954556 | 1.1259058 | down | mRNA |
| NM_024531 | SLC52A2 | 0.859335516 | 0.988512246 | 1.0111243 | down | mRNA |
| NM_024533 | CHST5 | 0.139183304 | 0.598648178 | 1.8514974 | down | mRNA |
| NM_024536 | CHPF | 0.904560287 | 0.99508468 | 1.0193656 | down | mRNA |
| NM_024537 | CARS2 | 0.896609669 | 0.994222243 | 1.0188737 | down | mRNA |
| NM_024540 | MRPL24 | 0.452217019 | 0.888854908 | 1.0685539 | down | mRNA |
| NM_024541 | C10orf76 | 0.04543103 | 0.39057239 | 1.3103868 | down | mRNA |
| NM_024544 | MUL1 | 0.353427346 | 0.836351835 | 1.1319195 | down | mRNA |
| NM_024545 | SAP130 | 0.020457787 | 0.285678379 | 1.3950247 | down | mRNA |
| NM_024548 | CEP97 | 0.449111595 | 0.888279993 | 1.1711256 | down | mRNA |
| NM_024549 | TCTN1 | 0.922591303 | 0.995349793 | 1.0140973 | down | mRNA |
| NM_024553 | CCDC132 | 0.449746979 | 0.888279993 | 1.1300321 | down | mRNA |
| NM_024555 | FBXL6 | 0.109623962 | 0.547904228 | 1.2545691 | down | mRNA |
| NM_024556 | FAM118B | 0.675412596 | 0.959531882 | 1.0647756 | down | mRNA |
| NM_024558 | METTL21D | 0.642709813 | 0.95447352 | 1.0337794 | down | mRNA |
| NM_024565 | CCNJL | 0.709234402 | 0.965462762 | 1.0680131 | down | mRNA |
| NM_024567 | HMBOX1 | 0.478730717 | 0.901271944 | 1.0926836 | down | mRNA |
| NM_024570 | RNASEH2B | 0.784077461 | 0.977347603 | 1.0324651 | down | mRNA |
| NM_024571 | SNRNP25 | 0.877563427 | 0.991306146 | 1.0149379 | down | mRNA |
| NM_024573 | C6orf211 | 0.142406549 | 0.602652383 | 1.1177482 | down | mRNA |
| NM_024574 | NDNF | 0.97817411 | 0.998641374 | 1.0223082 | down | mRNA |
| NM_024579 | C1orf54 | 0.922232902 | 0.995349793 | 1.0186524 | down | mRNA |
| NM_024580 | EFTUD1 | 0.007623686 | 0.193257016 | 1.4255181 | down | mRNA |
| NM_024582 | FAT4 | 0.502453186 | 0.910335149 | 1.2114462 | down | mRNA |
| NM_024583 | SCRN3 | 0.075966446 | 0.473352674 | 1.3271731 | down | mRNA |
| NM_024586 | OSBPL9 | 0.060903629 | 0.436633496 | 1.2820311 | down | mRNA |
| NM_024587 | TMEM53 | 0.983729783 | 0.998971807 | 1.0086017 | down | mRNA |
| NM_024590 | ARSJ | 0.811761205 | 0.98118927 | 1.0671101 | down | mRNA |
| NM_024592 | SRD5A3 | 0.718794488 | 0.966191059 | 1.055221 | down | mRNA |
| NM_024596 | MCPH1 | 0.001696473 | 0.10523567 | 1.4266656 | down | mRNA |
| NM_024597 | MAP7D3 | 0.111362924 | 0.552465871 | 1.0520133 | down | mRNA |
| NM_024598 | C16orf57 | 0.772368338 | 0.975448017 | 1.072142 | down | mRNA |
| NM_024600 | TMEM204 | 0.356845013 | 0.839423386 | 1.1170334 | down | mRNA |
| NM_024602 | HECTD3 | 0.213786929 | 0.706323665 | 1.1463901 | down | mRNA |
| NM_024604 | RPAP3 | 0.153758692 | 0.620938377 | 1.1946671 | down | mRNA |
| NM_024605 | ARHGAP10 | 0.167279446 | 0.642184223 | 1.145889 | down | mRNA |
| NM_024607 | PPP1R3B | 0.257408941 | 0.757735833 | 1.1963799 | down | mRNA |
| NM_024610 | HSPBAP1 | 0.376364313 | 0.849903337 | 1.1824519 | down | mRNA |
| NM_024612 | DHX40 | 0.180611122 | 0.663568002 | 1.2013025 | down | mRNA |
| NM_024613 | PLEKHF2 | 0.040670957 | 0.374172802 | 1.3706698 | down | mRNA |
| NM_024618 | NLRX1 | 0.006248613 | 0.179020559 | 1.1171195 | down | mRNA |
| NM_024622 | FASTKD1 | 0.008990866 | 0.202329498 | 1.255833 | down | mRNA |
| NM_024624 | SMC6 | 0.870620934 | 0.989592579 | 1.023444 | down | mRNA |
| NM_024627 | C22orf29 | 0.238447059 | 0.736444734 | 1.0970979 | down | mRNA |
| NM_024629 | MLF1IP | 0.904970759 | 0.99508468 | 1.0876934 | down | mRNA |
| NM_024630 | ZDHHC14 | 0.677113718 | 0.959531882 | 1.0542011 | down | mRNA |
| NM_024631 | MSANTD2 | 0.399843251 | 0.864196266 | 1.0777317 | down | mRNA |
| NM_024632 | SAP30L | 0.434373957 | 0.880931467 | 1.0695712 | down | mRNA |
| NM_024635 | NAA35 | 0.095826959 | 0.517216283 | 1.1398151 | down | mRNA |
| NM_024639 | ZNF322 | 0.002488122 | 0.118591515 | 1.5152839 | down | mRNA |
| NM_024641 | MANEA | 0.300803938 | 0.795828975 | 1.11743 | down | mRNA |
| NM_024645 | ZMAT4 | 0.709475517 | 0.965462762 | 1.102502 | down | mRNA |
| NM_024646 | ZYG11B | 0.18210609 | 0.665333517 | 1.0883095 | down | mRNA |
| NM_024648 | C17orf101 | 0.329939555 | 0.821768501 | 1.0688353 | down | mRNA |
| NM_024654 | NOL9 | 0.143601882 | 0.603870418 | 1.1250708 | down | mRNA |
| NM_024658 | IPO4 | 0.024587298 | 0.306504563 | 1.2371993 | down | mRNA |
| NM_024659 | GTDC1 | 0.66858623 | 0.958923197 | 1.1425558 | down | mRNA |
| NM_024663 | NPEPL1 | 0.881098414 | 0.992492198 | 1.0180592 | down | mRNA |
| NM_024664 | PPCS | 0.799909037 | 0.981152656 | 1.0110648 | down | mRNA |
| NM_024670 | SUV39H2 | 0.286057214 | 0.782746332 | 1.2191554 | down | mRNA |
| NM_024672 | THAP9 | 0.110553549 | 0.550116191 | 1.0563787 | down | mRNA |
| NM_024683 | TEFM | 0.020516654 | 0.286034385 | 1.1487476 | down | mRNA |
| NM_024685 | BBS10 | 0.034622846 | 0.35197585 | 1.4229445 | down | mRNA |
| NM_024691 | ZNF419 | 0.014879553 | 0.244907235 | 1.3556872 | down | mRNA |
| NM_024692 | CLIP4 | 0.494554684 | 0.906975462 | 1.1356718 | down | mRNA |
| NM_024693 | ECHDC3 | 0.805013048 | 0.98118927 | 1.0894256 | down | mRNA |
| NM_024697 | ZNF385D | 0.747788311 | 0.970686576 | 1.1369547 | down | mRNA |
| NM_024699 | ZFAND1 | 0.287102444 | 0.782954059 | 1.1724539 | down | mRNA |
| NM_024702 | ZNF750 | 0.491510332 | 0.905806259 | 1.0841764 | down | mRNA |
| NM_024704 | KIF16B | 0.346708251 | 0.833542883 | 1.272904 | down | mRNA |
| NM_024705 | DHRS12 | 0.340104752 | 0.828242404 | 1.0702941 | down | mRNA |
| NM_024708 | ASB7 | 0.596581748 | 0.939485718 | 1.0728073 | down | mRNA |
| NM_024710 | ISOC2 | 0.365023676 | 0.844618342 | 1.0632879 | down | mRNA |
| NM_024713 | C15orf29 | 0.18152818 | 0.664894785 | 1.1516665 | down | mRNA |
| NM_024718 | C9orf86 | 0.478454197 | 0.901271944 | 1.054736 | down | mRNA |
| NM_024721 | ZFHX4 | 0.525416947 | 0.917686109 | 1.0515843 | down | mRNA |
| NM_024725 | CCDC82 | 0.339854858 | 0.828118322 | 1.0617237 | down | mRNA |
| NM_024726 | IQCA1 | 0.176548998 | 0.655993096 | 1.1162359 | down | mRNA |
| NM_024731 | KLHL36 | 0.087244168 | 0.500533133 | 1.0987298 | down | mRNA |
| NM_024734 | CLMN | 0.3957837 | 0.861663366 | 1.0572025 | down | mRNA |
| NM_024738 | C12orf49 | 0.006549331 | 0.182886161 | 1.2467944 | down | mRNA |
| NM_024741 | ZNF408 | 0.223836094 | 0.717828939 | 1.6528691 | down | mRNA |
| NM_024745 | SHCBP1 | 0.759813649 | 0.972829416 | 1.1909885 | down | mRNA |
| NM_024746 | HHIPL2 | 0.345280182 | 0.832533915 | 1.5581515 | down | mRNA |
| NM_024753 | TTC21B | 0.253269587 | 0.753288068 | 1.1781645 | down | mRNA |
| NM_024754 | PTCD2 | 0.044287628 | 0.386873462 | 1.1501707 | down | mRNA |
| NM_024757 | EHMT1 | 0.984573555 | 0.998971807 | 1.0038018 | down | mRNA |
| NM_024759 | NIPAL2 | 0.627381697 | 0.949313438 | 1.4110225 | down | mRNA |
| NM_024763 | WDR78 | 0.875404376 | 0.991094934 | 1.0657326 | down | mRNA |
| NM_024766 | CAMKMT | 0.575806602 | 0.933656777 | 1.0499448 | down | mRNA |
| NM_024769 | CLMP | 0.037592637 | 0.36224336 | 1.190164 | down | mRNA |
| NM_024771 | NAA40 | 0.284562625 | 0.78179061 | 1.4843253 | down | mRNA |
| NM_024772 | ZMYM1 | 0.009741411 | 0.206100289 | 1.6090068 | down | mRNA |
| NM_024775 | GEMIN6 | 0.324142431 | 0.816523598 | 1.1770207 | down | mRNA |
| NM_024782 | NHEJ1 | 0.307421767 | 0.802168809 | 1.4593815 | down | mRNA |
| NM_024783 | AGBL2 | 0.564732935 | 0.929820378 | 1.039525 | down | mRNA |
| NM_024790 | CSPP1 | 0.151707404 | 0.616902989 | 1.292301 | down | mRNA |
| NM_024792 | FAM57A | 0.12730104 | 0.580723522 | 1.431004 | down | mRNA |
| NM_024793 | CLUAP1 | 0.085557771 | 0.497443696 | 1.2058941 | down | mRNA |
| NM_024800 | NEK11 | 0.488003827 | 0.903757389 | 1.177303 | down | mRNA |
| NM_024805 | RBFA | 0.963959257 | 0.99793927 | 1.0049815 | down | mRNA |
| NM_024806 | C11orf63 | 0.189387424 | 0.674719659 | 1.3497564 | down | mRNA |
| NM_024808 | BORA | 0.104874698 | 0.535979888 | 1.891909 | down | mRNA |
| NM_024811 | CPSF7 | 0.846044972 | 0.987338076 | 1.0184545 | down | mRNA |
| NM_024813 | RPAP2 | 0.037851127 | 0.363212377 | 1.2010253 | down | mRNA |
| NM_024814 | CBLL1 | 0.177329501 | 0.65723949 | 1.1889232 | down | mRNA |
| NM_024816 | RABEP2 | 0.311306605 | 0.806503245 | 1.1552817 | down | mRNA |
| NM_024818 | UBA5 | 0.020599128 | 0.286034385 | 1.2440372 | down | mRNA |
| NM_024827 | HDAC11 | 0.605027957 | 0.94174007 | 1.1057977 | down | mRNA |
| NM_024830 | LPCAT1 | 0.721596162 | 0.966191059 | 1.0591058 | down | mRNA |
| NM_024832 | RIN3 | 0.154327535 | 0.621759137 | 1.2180522 | down | mRNA |
| NM_024833 | ZNF671 | 0.137980208 | 0.596873691 | 1.1234315 | down | mRNA |
| NM_024834 | MCMBP | 0.153493756 | 0.620567716 | 1.1758565 | down | mRNA |
| NM_024835 | GGNBP2 | 0.080284429 | 0.483876854 | 1.2359515 | down | mRNA |
| NM_024836 | ZNF672 | 0.001042667 | 0.086588663 | 1.7854969 | down | mRNA |
| NM_024839 | RPP21 | 0.164889065 | 0.638119216 | 1.0633398 | down | mRNA |
| NM_024840 | ZNF613 | 0.224132408 | 0.718323293 | 1.2110903 | down | mRNA |
| NM_024841 | PRR5L | 0.023884007 | 0.303389731 | 1.5675228 | down | mRNA |
| NM_024843 | CYBRD1 | 0.915359324 | 0.995349793 | 1.0128358 | down | mRNA |
| NM_024847 | TMC7 | 0.013317959 | 0.23544335 | 1.3922326 | down | mRNA |
| NM_024848 | MORN1 | 0.603202846 | 0.941484604 | 1.0319011 | down | mRNA |
| NM_024852 | EIF2C3 | 0.464807626 | 0.894989844 | 1.0540371 | down | mRNA |
| NM_024854 | PYROXD1 | 0.533474858 | 0.920530493 | 1.0416624 | down | mRNA |
| NM_024860 | SETD6 | 0.062298784 | 0.439381733 | 1.5570246 | down | mRNA |
| NM_024866 | ADM2 | 0.910093767 | 0.995342392 | 1.0132763 | down | mRNA |
| NM_024874 | KIAA0319L | 0.226848181 | 0.722135151 | 1.0823713 | down | mRNA |
| NM_024881 | SLC35E1 | 0.190935718 | 0.676402188 | 1.1850256 | down | mRNA |
| NM_024884 | L2HGDH | 0.283623832 | 0.7813875 | 1.1763681 | down | mRNA |
| NM_024887 | DHDDS | 0.547550625 | 0.92514258 | 1.0484071 | down | mRNA |
| NM_024888 | LPPR3 | 0.704003648 | 0.963625105 | 1.0506963 | down | mRNA |
| NM_024893 | SYNDIG1 | 0.976745579 | 0.998501532 | 1.0030528 | down | mRNA |
| NM_024894 | NOL10 | 0.830549569 | 0.985549562 | 1.0159811 | down | mRNA |
| NM_024895 | PDZD7 | 0.743706647 | 0.970330526 | 1.0542721 | down | mRNA |
| NM_024898 | DENND1C | 0.751802293 | 0.970725371 | 1.0339845 | down | mRNA |
| NM_024899 | CEP76 | 0.178554795 | 0.65979922 | 1.4448601 | down | mRNA |
| NM_024900 | PHF17 | 0.000162631 | 0.044621794 | 2.4050633 | down | mRNA |
| NM_024908 | WDR76 | 0.807648466 | 0.98118927 | 1.1795577 | down | mRNA |
| NM_024911 | WLS | 0.474555857 | 0.899495615 | 1.219999 | down | mRNA |
| NM_024926 | TTC26 | 0.09767669 | 0.520535504 | 1.1022092 | down | mRNA |
| NM_024928 | OBFC1 | 0.872536127 | 0.990534615 | 1.0367445 | down | mRNA |
| NM_024939 | ESRP2 | 0.768499148 | 0.975142015 | 1.032849 | down | mRNA |
| NM_024940 | DOCK5 | 0.00429019 | 0.149619451 | 1.3296369 | down | mRNA |
| NM_024941 | C5orf44 | 0.142750062 | 0.602781779 | 1.0919404 | down | mRNA |
| NM_024942 | C10orf88 | 0.011251148 | 0.21765386 | 1.4145585 | down | mRNA |
| NM_024945 | RMI1 | 0.582099035 | 0.934005566 | 1.2261787 | down | mRNA |
| NM_024947 | PHC3 | 0.124157545 | 0.57792381 | 1.14614 | down | mRNA |
| NM_024948 | FAM188A | 0.648168911 | 0.95550188 | 1.0408538 | down | mRNA |
| NM_024949 | WWC2 | 0.532436124 | 0.920378445 | 1.3364609 | down | mRNA |
| NM_024953 | NAA25 | 0.84435657 | 0.987338076 | 1.0372606 | down | mRNA |
| NM_024954 | UBTD1 | 0.031395381 | 0.341322176 | 1.3397239 | down | mRNA |
| NM_024956 | TMEM62 | 0.036065879 | 0.356485876 | 1.1626919 | down | mRNA |
| NM_024963 | FBXL18 | 0.013376995 | 0.23544335 | 1.2017211 | down | mRNA |
| NM_024979 | MCF2L | 0.16807499 | 0.642954036 | 1.1580394 | down | mRNA |
| NM_024980 | GPR157 | 0.680793782 | 0.960111041 | 1.0441417 | down | mRNA |
| NM_024989 | PGAP1 | 0.379561414 | 0.852922487 | 1.153501 | down | mRNA |
| NM_025010 | KLHL18 | 0.041571619 | 0.377842715 | 1.3526432 | down | mRNA |
| NM_025014 | ARHGEF15 | 0.951115893 | 0.996710991 | 1.0092192 | down | mRNA |
| NM_025027 | ZNF606 | 0.021270275 | 0.288424269 | 1.4312433 | down | mRNA |
| NM_025049 | PIF1 | 0.29153453 | 0.787986325 | 1.3967713 | down | mRNA |
| NM_025054 | VCPIP1 | 0.198193292 | 0.686763988 | 1.1331807 | down | mRNA |
| NM_025057 | C14orf45 | 0.758396937 | 0.972072638 | 1.0449072 | down | mRNA |
| NM_025058 | TRIM46 | 0.742242231 | 0.970330526 | 1.0559789 | down | mRNA |
| NM_025065 | RPF1 | 0.683667243 | 0.960111041 | 1.0807393 | down | mRNA |
| NM_025075 | THOC7 | 0.767755758 | 0.975033576 | 1.0286496 | down | mRNA |
| NM_025077 | TOE1 | 0.098772777 | 0.522573019 | 1.3879987 | down | mRNA |
| NM_025083 | EDC3 | 0.174360935 | 0.652930084 | 1.1460075 | down | mRNA |
| NM_025087 | CWH43 | 0.50137118 | 0.910061537 | 1.1406318 | down | mRNA |
| NM_025092 | ATHL1 | 0.380221608 | 0.853407568 | 1.2290489 | down | mRNA |
| NM_025104 | DBF4B | 0.637459939 | 0.952666356 | 1.1792292 | down | mRNA |
| NM_025107 | MYCT1 | 0.498894465 | 0.909361611 | 1.0943979 | down | mRNA |
| NM_025108 | C16orf59 | 0.849048814 | 0.987678166 | 1.020283 | down | mRNA |
| NM_025109 | MYO19 | 0.860549318 | 0.988975154 | 1.0549374 | down | mRNA |
| NM_025114 | CEP290 | 0.198315999 | 0.686799061 | 1.1814801 | down | mRNA |
| NM_025125 | C10orf57 | 0.419149073 | 0.872365335 | 1.0972907 | down | mRNA |
| NM_025126 | RNF34 | 0.588055162 | 0.93579682 | 1.0479144 | down | mRNA |
| NM_025128 | MUS81 | 0.03752266 | 0.362147666 | 1.6223535 | down | mRNA |
| NM_025129 | FUZ | 0.049127704 | 0.402023817 | 1.1167352 | down | mRNA |
| NM_025134 | CHD9 | 0.04766473 | 0.397835916 | 1.3423829 | down | mRNA |
| NM_025136 | OPA3 | 0.441913498 | 0.88419144 | 1.3329825 | down | mRNA |
| NM_025137 | SPG11 | 0.085647844 | 0.497540559 | 1.259515 | down | mRNA |
| NM_025138 | PROSER1 | 0.003187056 | 0.133152455 | 1.5787264 | down | mRNA |
| NM_025139 | ARMC9 | 0.57857253 | 0.933821303 | 1.2972713 | down | mRNA |
| NM_025146 | NAA50 | 0.817932203 | 0.981806106 | 1.0191497 | down | mRNA |
| NM_025147 | COQ10B | 0.030904209 | 0.340185047 | 1.1927566 | down | mRNA |
| NM_025149 | ACSF2 | 0.920145217 | 0.995349793 | 1.0466908 | down | mRNA |
| NM_025150 | TARS2 | 0.142481393 | 0.602652383 | 1.2147186 | down | mRNA |
| NM_025154 | SUN1 | 0.449278889 | 0.888279993 | 1.064778 | down | mRNA |
| NM_025155 | PAAF1 | 0.309872531 | 0.80463963 | 1.0790679 | down | mRNA |
| NM_025161 | C17orf70 | 0.269643443 | 0.76993462 | 1.2699998 | down | mRNA |
| NM_025165 | ELL3 | 0.891436474 | 0.9937521 | 1.0279159 | down | mRNA |
| NM_025176 | NINL | 0.442290678 | 0.88419144 | 1.2758464 | down | mRNA |
| NM_025179 | PLXNA2 | 0.72481283 | 0.966191059 | 1.1918933 | down | mRNA |
| NM_025181 | SLC35F5 | 0.621865188 | 0.948047106 | 1.1232633 | down | mRNA |
| NM_025185 | TANC2 | 0.032891479 | 0.345072008 | 1.2929133 | down | mRNA |
| NM_025187 | C16orf70 | 0.216338121 | 0.711073448 | 1.102163 | down | mRNA |
| NM_025191 | EDEM3 | 0.089309753 | 0.505243712 | 1.1307751 | down | mRNA |
| NM_025193 | HSD3B7 | 0.750277589 | 0.970725371 | 1.0150504 | down | mRNA |
| NM_025196 | GRPEL1 | 0.517741007 | 0.915684599 | 1.0461338 | down | mRNA |
| NM_025201 | PLEKHO2 | 0.504942816 | 0.910568383 | 1.0334563 | down | mRNA |
| NM_025203 | C2orf44 | 0.191742112 | 0.67759806 | 1.4322721 | down | mRNA |
| NM_025204 | TRABD | 0.573238011 | 0.933656777 | 1.1191387 | down | mRNA |
| NM_025205 | MED28 | 0.557595426 | 0.926833616 | 1.0188944 | down | mRNA |
| NM_025207 | FLAD1 | 0.591617898 | 0.937303262 | 1.2244219 | down | mRNA |
| NM_025212 | CXXC4 | 0.373537072 | 0.848504826 | 1.2133598 | down | mRNA |
| NM_025215 | PUS1 | 0.194064794 | 0.681590151 | 1.3438452 | down | mRNA |
| NM_025217 | ULBP2 | 0.651761677 | 0.956204521 | 1.079522 | down | mRNA |
| NM_025219 | DNAJC5 | 0.448657971 | 0.888279993 | 1.1331037 | down | mRNA |
| NM_025220 | ADAM33 | 0.226333494 | 0.721667695 | 1.1946968 | down | mRNA |
| NM_025222 | WDR82 | 0.014749465 | 0.24380986 | 1.3092704 | down | mRNA |
| NM_025228 | TRAF3IP3 | 0.427727005 | 0.876992614 | 1.376866 | down | mRNA |
| NM_025231 | ZSCAN16 | 0.009844355 | 0.206905383 | 1.7660978 | down | mRNA |
| NM_025232 | REEP4 | 0.402141588 | 0.864786348 | 1.2181745 | down | mRNA |
| NM_025234 | WDR61 | 0.203963309 | 0.693939432 | 1.0584123 | down | mRNA |
| NM_025235 | TNKS2 | 0.24822417 | 0.74647329 | 1.1531123 | down | mRNA |
| NM_025236 | RNF39 | 0.715647474 | 0.966191059 | 1.0474534 | down | mRNA |
| NM_025237 | SOST | 0.656042716 | 0.957343034 | 1.1147629 | down | mRNA |
| NM_025238 | BTBD1 | 0.432880579 | 0.88061258 | 1.1095523 | down | mRNA |
| NM_025258 | VWA7 | 0.461566248 | 0.894667841 | 1.3370104 | down | mRNA |
| NM_025259 | MSH5 | 0.174412455 | 0.652930084 | 1.0829381 | down | mRNA |
| NM_025260 | C6orf25 | 0.498120148 | 0.909116151 | 1.0741228 | down | mRNA |
| NM_025263 | PRR3 | 0.797339225 | 0.980672744 | 1.0360062 | down | mRNA |
| NM_025264 | THUMPD2 | 0.000595671 | 0.074301778 | 1.6735923 | down | mRNA |
| NM_025267 | PTGES3L-AARSD1 | 0.975861384 | 0.998501532 | 1.0051385 | down | mRNA |
| NM_025268 | TMEM121 | 0.470831294 | 0.898445976 | 1.1941941 | down | mRNA |
| NM_030571 | NDFIP1 | 0.814774392 | 0.981277402 | 1.0085011 | down | mRNA |
| NM_030573 | THAP7 | 0.49051586 | 0.905300886 | 1.1991812 | down | mRNA |
| NM_030576 | LIMD2 | 0.447142478 | 0.886972498 | 1.071035 | down | mRNA |
| NM_030580 | ZNF34 | 0.622710406 | 0.948341211 | 1.0875897 | down | mRNA |
| NM_030581 | WDR59 | 0.071166031 | 0.46104346 | 1.2665787 | down | mRNA |
| NM_030583 | MATN2 | 0.728341118 | 0.966191059 | 1.0390019 | down | mRNA |
| NM_030593 | SIRT2 | 0.210576579 | 0.701214058 | 1.1794359 | down | mRNA |
| NM_030613 | ZFP2 | 0.912105487 | 0.995342392 | 1.0119297 | down | mRNA |
| NM_030629 | CMIP | 0.907710796 | 0.995145546 | 1.0142621 | down | mRNA |
| NM_030634 | ZNF436 | 0.077742965 | 0.479016418 | 1.2977284 | down | mRNA |
| NM_030637 | DDHD1 | 0.001070961 | 0.086778252 | 1.3039854 | down | mRNA |
| NM_030645 | SH3BP5L | 0.133606136 | 0.590154236 | 1.4751878 | down | mRNA |
| NM_030648 | SETD7 | 0.188074285 | 0.672871083 | 1.0567892 | down | mRNA |
| NM_030649 | ACAP3 | 0.137863719 | 0.596789644 | 1.1746985 | down | mRNA |
| NM_030652 | EGFL8 | 0.240115331 | 0.738437735 | 1.0745196 | down | mRNA |
| NM_030653 | DDX11 | 0.257819631 | 0.758389576 | 1.096995 | down | mRNA |
| NM_030660 | ATXN3 | 0.783660682 | 0.977275912 | 1.0372314 | down | mRNA |
| NM_030664 | PTER | 0.403359253 | 0.864786348 | 1.1337474 | down | mRNA |
| NM_030665 | RAI1 | 0.005505398 | 0.169833068 | 1.9390076 | down | mRNA |
| NM_030666 | SERPINB1 | 0.750271844 | 0.970725371 | 1.0434636 | down | mRNA |
| NM_030674 | SLC38A1 | 0.005878928 | 0.174484737 | 1.1404437 | down | mRNA |
| NM_030751 | ZEB1 | 0.280081294 | 0.778697234 | 1.2713118 | down | mRNA |
| NM_030752 | TCP1 | 0.376046125 | 0.849862269 | 1.0985047 | down | mRNA |
| NM_030753 | WNT3 | 0.4079416 | 0.867591329 | 1.1414857 | down | mRNA |
| NM_030755 | TMX1 | 0.246649796 | 0.745152522 | 1.0521346 | down | mRNA |
| NM_030756 | TCF7L2 | 0.718038768 | 0.966191059 | 1.0268474 | down | mRNA |
| NM_030759 | NRBF2 | 0.559821549 | 0.927576541 | 1.2646494 | down | mRNA |
| NM_030760 | S1PR5 | 0.863016695 | 0.989401142 | 1.0358299 | down | mRNA |
| NM_030761 | WNT4 | 0.794000075 | 0.97921643 | 1.0312406 | down | mRNA |
| NM_030770 | TMPRSS5 | 0.033083041 | 0.346127643 | 1.2427217 | down | mRNA |
| NM_030771 | CCDC34 | 0.801350762 | 0.98118927 | 1.0824879 | down | mRNA |
| NM_030777 | SLC2A10 | 0.002134934 | 0.110028846 | 1.2120191 | down | mRNA |
| NM_030782 | CLPTM1L | 0.049266316 | 0.402199077 | 1.1705982 | down | mRNA |
| NM_030786 | SYNC | 0.399112698 | 0.863643939 | 1.1401092 | down | mRNA |
| NM_030789 | HM13 | 0.114625853 | 0.558779196 | 1.1507213 | down | mRNA |
| NM_030790 | ITFG1 | 0.742496743 | 0.970330526 | 1.0418792 | down | mRNA |
| NM_030791 | SGPP1 | 0.191421798 | 0.676985215 | 1.2257689 | down | mRNA |
| NM_030793 | FBXO38 | 0.010435466 | 0.209736088 | 1.3440137 | down | mRNA |
| NM_030796 | VOPP1 | 0.084676035 | 0.495154665 | 1.1802488 | down | mRNA |
| NM_030801 | MAGED4B | 0.052054954 | 0.411501294 | 1.1559892 | down | mRNA |
| NM_030802 | FAM117A | 0.82274515 | 0.983305175 | 1.0111636 | down | mRNA |
| NM_030805 | LMAN2L | 0.005635291 | 0.170012215 | 1.163087 | down | mRNA |
| NM_030806 | C1orf21 | 0.435662612 | 0.881645489 | 1.0462282 | down | mRNA |
| NM_030808 | NDEL1 | 0.200729503 | 0.68900633 | 1.324435 | down | mRNA |
| NM_030810 | TXNDC5 | 0.017777689 | 0.268142409 | 1.1745787 | down | mRNA |
| NM_030811 | MRPS26 | 0.003964585 | 0.144718033 | 1.3098755 | down | mRNA |
| NM_030815 | PDRG1 | 0.928349805 | 0.996053205 | 1.0178563 | down | mRNA |
| NM_030816 | ANKRD13C | 0.011155249 | 0.216420345 | 1.2064253 | down | mRNA |
| NM_030818 | CCDC130 | 0.006597709 | 0.183508367 | 1.2974834 | down | mRNA |
| NM_030819 | GFOD2 | 0.008026959 | 0.195524479 | 1.3474864 | down | mRNA |
| NM_030821 | PLA2G12A | 0.802555056 | 0.98118927 | 1.0354195 | down | mRNA |
| NM_030877 | CTNNBL1 | 0.723878958 | 0.966191059 | 1.0535066 | down | mRNA |
| NM_030895 | ZNF696 | 0.070187317 | 0.458429109 | 1.2605362 | down | mRNA |
| NM_030900 | TBRG4 | 0.691973501 | 0.961451327 | 1.0261044 | down | mRNA |
| NM_030911 | CDADC1 | 0.399054244 | 0.863643939 | 1.1530815 | down | mRNA |
| NM_030913 | SEMA6C | 0.96936719 | 0.998245244 | 1.0030532 | down | mRNA |
| NM_030914 | URM1 | 0.766175589 | 0.974737263 | 1.027868 | down | mRNA |
| NM_030915 | LBH | 0.017731314 | 0.267776588 | 1.3065512 | down | mRNA |
| NM_030917 | FIP1L1 | 0.097707052 | 0.520535504 | 1.4665769 | down | mRNA |
| NM_030918 | SNX27 | 0.994660807 | 0.999872237 | 1.000683 | down | mRNA |
| NM_030926 | ITM2C | 0.852823752 | 0.987678166 | 1.0198804 | down | mRNA |
| NM_030927 | TSPAN14 | 0.369217146 | 0.845850481 | 1.0752817 | down | mRNA |
| NM_030928 | CDT1 | 0.796942665 | 0.980667273 | 1.0951857 | down | mRNA |
| NM_030929 | KAZALD1 | 0.744187166 | 0.970330526 | 1.0719563 | down | mRNA |
| NM_030934 | TRMT1L | 0.033253787 | 0.346405261 | 1.3494211 | down | mRNA |
| NM_030937 | CCNL2 | 0.01493443 | 0.245074119 | 1.1578933 | down | mRNA |
| NM_030938 | VMP1 | 0.486767693 | 0.903524001 | 1.0985793 | down | mRNA |
| NM_030940 | ISCA1 | 0.035660114 | 0.354652995 | 1.3798878 | down | mRNA |
| NM_030941 | LOC81691 | 0.846123286 | 0.987338076 | 1.1214954 | down | mRNA |
| NM_030943 | AMN | 0.886601653 | 0.992981836 | 1.011225 | down | mRNA |
| NM_030949 | PPP1R14C | 0.807123548 | 0.98118927 | 1.1172385 | down | mRNA |
| NM_030953 | TIGD6 | 0.366521165 | 0.845181673 | 1.290093 | down | mRNA |
| NM_030955 | ADAMTS12 | 0.727374626 | 0.966191059 | 1.0514554 | down | mRNA |
| NM_030963 | RNF146 | 0.065438215 | 0.447073184 | 1.1734385 | down | mRNA |
| NM_030965 | ST6GALNAC5 | 0.543663105 | 0.923577434 | 1.4612914 | down | mRNA |
| NM_030966 | KRTAP1-3 | 0.036793032 | 0.359628653 | 1.7104221 | down | mRNA |
| NM_030969 | TMEM14B | 0.732918115 | 0.967703318 | 1.0279202 | down | mRNA |
| NM_030971 | SFXN3 | 0.246528813 | 0.745120237 | 1.2827181 | down | mRNA |
| NM_030973 | MED25 | 0.456894325 | 0.891568474 | 1.1282269 | down | mRNA |
| NM_030974 | SHARPIN | 0.654978125 | 0.957205183 | 1.0245094 | down | mRNA |
| NM_030978 | ARPC5L | 0.236322955 | 0.734472382 | 1.1028727 | down | mRNA |
| NM_030979 | PABPC3 | 0.323213472 | 0.816040769 | 1.0787175 | down | mRNA |
| NM_030980 | ISG20L2 | 0.042692729 | 0.382084661 | 1.2614127 | down | mRNA |
| NM_031157 | HNRNPA1 | 0.752018494 | 0.970725371 | 1.0103662 | down | mRNA |
| NM_031203 | HNRNPM | 0.31204814 | 0.80699254 | 1.0767605 | down | mRNA |
| NM_031205 | CABP1 | 0.152369814 | 0.618255775 | 1.2570776 | down | mRNA |
| NM_031206 | LAS1L | 0.599246472 | 0.940648177 | 1.0419109 | down | mRNA |
| NM_031207 | HYI | 0.532948427 | 0.920530493 | 1.0897453 | down | mRNA |
| NM_031209 | QTRT1 | 0.351460878 | 0.835247013 | 1.0442962 | down | mRNA |
| NM_031213 | FAM108A1 | 0.985494481 | 0.998971807 | 1.0033933 | down | mRNA |
| NM_031216 | SEH1L | 0.034060543 | 0.349898235 | 1.1612844 | down | mRNA |
| NM_031217 | KIF18A | 0.046019975 | 0.391970965 | 1.8619222 | down | mRNA |
| NM_031219 | HDHD3 | 0.2441838 | 0.74244729 | 1.3243325 | down | mRNA |
| NM_031220 | PITPNM3 | 0.035422196 | 0.353725338 | 1.9266403 | down | mRNA |
| NM_031231 | NECAB3 | 0.71380999 | 0.966110889 | 1.0302607 | down | mRNA |
| NM_031232 | NECAB3 | 0.737804949 | 0.969060091 | 1.0348745 | down | mRNA |
| NM_031244 | SIRT5 | 0.260920466 | 0.761583524 | 1.0752085 | down | mRNA |
| NM_031246 | PSG2 | 0.855313785 | 0.988178307 | 1.1311987 | down | mRNA |
| NM_031266 | HNRNPAB | 0.574071744 | 0.933656777 | 1.072049 | down | mRNA |
| NM_031267 | CDK13 | 0.09251408 | 0.511338764 | 1.3939793 | down | mRNA |
| NM_031268 | PDPK1 | 0.238336998 | 0.736444734 | 1.1882725 | down | mRNA |
| NM_031271 | TEX15 | 0.940633221 | 0.996710991 | 1.0098152 | down | mRNA |
| NM_031279 | AGXT2L1 | 0.931954135 | 0.996710991 | 1.0096702 | down | mRNA |
| NM_031280 | MRPS15 | 0.503536287 | 0.910553209 | 1.0424475 | down | mRNA |
| NM_031284 | ADPGK | 0.781974196 | 0.976963215 | 1.0417014 | down | mRNA |
| NM_031286 | SH3BGRL3 | 0.539545666 | 0.923577434 | 1.0394081 | down | mRNA |
| NM_031289 | GSG1 | 0.308031306 | 0.802832011 | 1.5373335 | down | mRNA |
| NM_031291 | SLC25A31 | 0.162208801 | 0.634236412 | 1.4145595 | down | mRNA |
| NM_031299 | CDCA3 | 0.927079305 | 0.995829678 | 1.0492758 | down | mRNA |
| NM_031300 | MXD3 | 0.410102294 | 0.86807846 | 1.2631163 | down | mRNA |
| NM_031302 | GLT8D2 | 0.724184781 | 0.966191059 | 1.0774269 | down | mRNA |
| NM_031304 | DOHH | 0.351635317 | 0.835247013 | 1.0777097 | down | mRNA |
| NM_031307 | PUS3 | 0.021201636 | 0.288424269 | 1.1863393 | down | mRNA |
| NM_031308 | EPPK1 | 0.599235692 | 0.940648177 | 1.1589976 | down | mRNA |
| NM_031361 | COL4A3BP | 0.180494006 | 0.663497401 | 1.2449487 | down | mRNA |
| NM_031369 | HNRNPD | 0.14231917 | 0.602652383 | 1.1338327 | down | mRNA |
| NM_031370 | HNRNPD | 0.115595292 | 0.561328752 | 1.1943699 | down | mRNA |
| NM_031372 | HNRPDL | 0.633058151 | 0.951174475 | 1.070917 | down | mRNA |
| NM_031420 | MRPL9 | 0.945738867 | 0.996710991 | 1.0041961 | down | mRNA |
| NM_031423 | NUF2 | 0.65437157 | 0.957205183 | 1.2575669 | down | mRNA |
| NM_031427 | DNAL1 | 0.503130876 | 0.910475258 | 1.1062174 | down | mRNA |
| NM_031431 | COG3 | 0.330758669 | 0.821853462 | 1.1488788 | down | mRNA |
| NM_031432 | UCK1 | 0.076830432 | 0.476553092 | 1.322288 | down | mRNA |
| NM_031438 | NUDT12 | 0.022177868 | 0.293936579 | 1.3496674 | down | mRNA |
| NM_031443 | CCM2 | 0.016485718 | 0.25645571 | 1.0917429 | down | mRNA |
| NM_031446 | C18orf21 | 0.279596962 | 0.778095461 | 1.036152 | down | mRNA |
| NM_031452 | FAM103A1 | 0.041470284 | 0.377571504 | 1.0707297 | down | mRNA |
| NM_031454 | SELO | 0.231867223 | 0.729717132 | 1.1862319 | down | mRNA |
| NM_031459 | SESN2 | 0.126247185 | 0.578772889 | 1.1158787 | down | mRNA |
| NM_031460 | KCNK17 | 0.963958235 | 0.99793927 | 1.0036836 | down | mRNA |
| NM_031461 | CRISPLD1 | 0.398604828 | 0.863643939 | 1.4356343 | down | mRNA |
| NM_031463 | HSDL1 | 0.05725123 | 0.426252791 | 1.4459092 | down | mRNA |
| NM_031472 | TRPT1 | 0.329850928 | 0.821768501 | 1.0278813 | down | mRNA |
| NM_031473 | IFT81 | 0.691856539 | 0.961451327 | 1.0159313 | down | mRNA |
| NM_031474 | NRIP2 | 0.281247879 | 0.779126326 | 1.4043225 | down | mRNA |
| NM_031480 | RIOK1 | 0.067031747 | 0.451412357 | 1.1504237 | down | mRNA |
| NM_031486 | ZNF484 | 0.723538984 | 0.966191059 | 1.0559994 | down | mRNA |
| NM_031488 | L3MBTL2 | 0.232987464 | 0.731100552 | 1.3113885 | down | mRNA |
| NM_031490 | LONP2 | 0.072497034 | 0.463255488 | 1.0724523 | down | mRNA |
| NM_031492 | RBM4B | 0.105509264 | 0.537720406 | 1.411616 | down | mRNA |
| NM_031854 | KRTAP4-12 | 0.057638431 | 0.427646201 | 3.6175436 | down | mRNA |
| NM_031885 | BBS2 | 0.603495735 | 0.941484604 | 1.1431446 | down | mRNA |
| NM_031896 | CACNG7 | 0.01138547 | 0.218702965 | 1.2687209 | down | mRNA |
| NM_031897 | CACNG6 | 0.909672741 | 0.995342392 | 1.0146111 | down | mRNA |
| NM_031899 | GORASP1 | 0.081536863 | 0.488021046 | 1.1136123 | down | mRNA |
| NM_031902 | MRPS5 | 0.435414083 | 0.881627543 | 1.0866738 | down | mRNA |
| NM_031905 | ARMC10 | 0.223765194 | 0.71782801 | 1.073462 | down | mRNA |
| NM_031907 | USP26 | 0.784135006 | 0.977347603 | 1.114838 | down | mRNA |
| NM_031908 | C1QTNF2 | 0.864816035 | 0.989401142 | 1.0712372 | down | mRNA |
| NM_031909 | C1QTNF4 | 0.876624335 | 0.991255275 | 1.0401347 | down | mRNA |
| NM_031913 | ESYT3 | 0.751468773 | 0.970725371 | 1.0849491 | down | mRNA |
| NM_031914 | SYT16 | 0.100833308 | 0.527707289 | 1.3417276 | down | mRNA |
| NM_031918 | KLF16 | 0.139591021 | 0.598768913 | 1.5114656 | down | mRNA |
| NM_031921 | ATAD3B | 0.458441806 | 0.892667725 | 1.2753005 | down | mRNA |
| NM_031922 | REPS1 | 0.031010623 | 0.340185047 | 1.2094781 | down | mRNA |
| NM_031923 | TAF3 | 0.330699052 | 0.821853462 | 1.0688478 | down | mRNA |
| NM_031924 | RSPH3 | 0.405347138 | 0.866165103 | 1.1121437 | down | mRNA |
| NM_031925 | TMEM120A | 0.799818593 | 0.981152656 | 1.018599 | down | mRNA |
| NM_031934 | RAB34 | 0.135701315 | 0.593733407 | 1.1112896 | down | mRNA |
| NM_031935 | HMCN1 | 0.650456517 | 0.955894361 | 1.0477477 | down | mRNA |
| NM_031936 | GPR61 | 0.363799576 | 0.843238468 | 1.1771341 | down | mRNA |
| NM_031939 | MRO | 0.858026852 | 0.988512246 | 1.0191794 | down | mRNA |
| NM_031940 | TM2D2 | 0.383558037 | 0.853881747 | 1.1199193 | down | mRNA |
| NM_031941 | USHBP1 | 0.640904732 | 0.953894897 | 1.0633784 | down | mRNA |
| NM_031945 | TSPAN10 | 0.920150066 | 0.995349793 | 1.0152236 | down | mRNA |
| NM_031946 | AGAP3 | 0.67796005 | 0.959531882 | 1.0320588 | down | mRNA |
| NM_031952 | SPATA9 | 0.688394256 | 0.960567704 | 1.1911933 | down | mRNA |
| NM_031953 | SNX25 | 0.032729796 | 0.344901399 | 1.3721468 | down | mRNA |
| NM_031955 | SPATA16 | 0.492243332 | 0.906044608 | 1.2011658 | down | mRNA |
| NM_031957 | KRTAP1-5 | 0.411701491 | 0.869001481 | 2.3199644 | down | mRNA |
| NM_031966 | CCNB1 | 0.33148494 | 0.822021198 | 1.3544658 | down | mRNA |
| NM_031989 | PCBP2 | 0.502991644 | 0.910475258 | 1.1005848 | down | mRNA |
| NM_031992 | EIF4H | 0.353200968 | 0.836266379 | 1.0293907 | down | mRNA |
| NM_032012 | TMEM245 | 0.537403032 | 0.922702335 | 1.0945865 | down | mRNA |
| NM_032013 | NDRG3 | 0.381855812 | 0.853514109 | 1.1396323 | down | mRNA |
| NM_032015 | RNF26 | 0.659051661 | 0.958354006 | 1.1388708 | down | mRNA |
| NM_032016 | STARD3NL | 0.592143299 | 0.937519382 | 1.0420597 | down | mRNA |
| NM_032018 | SPRTN | 0.095620366 | 0.51694341 | 1.3196276 | down | mRNA |
| NM_032020 | FUCA2 | 0.680824496 | 0.960111041 | 1.065717 | down | mRNA |
| NM_032024 | C10orf11 | 0.893751442 | 0.993978977 | 1.1190171 | down | mRNA |
| NM_032025 | EIF2A | 0.591784477 | 0.937303262 | 1.0647211 | down | mRNA |
| NM_032026 | TATDN1 | 0.188978175 | 0.6744312 | 1.0831785 | down | mRNA |
| NM_032038 | SPNS1 | 0.426594866 | 0.876202345 | 1.0421573 | down | mRNA |
| NM_032039 | ITFG3 | 0.489215607 | 0.904620847 | 1.0843767 | down | mRNA |
| NM_032040 | CCDC8 | 0.029728384 | 0.334953885 | 2.0446128 | down | mRNA |
| NM_032042 | FAM172A | 0.461947613 | 0.894754673 | 1.0475883 | down | mRNA |
| NM_032051 | PATZ1 | 0.168338692 | 0.643478528 | 1.2495315 | down | mRNA |
| NM_032052 | PATZ1 | 0.227713236 | 0.723854154 | 1.2653961 | down | mRNA |
| NM_032088 | PCDHGA8 | 0.92018708 | 0.995349793 | 1.0157387 | down | mRNA |
| NM_032102 | SRSF8 | 0.203096383 | 0.692764888 | 1.1395686 | down | mRNA |
| NM_032107 | L3MBTL1 | 0.487277203 | 0.903705199 | 1.1861237 | down | mRNA |
| NM_032108 | SEMA6B | 0.931512436 | 0.996710991 | 1.0373228 | down | mRNA |
| NM_032109 | OTP | 0.43005879 | 0.878524598 | 1.152112 | down | mRNA |
| NM_032110 | DMRTA2 | 0.63021908 | 0.950410442 | 1.0280234 | down | mRNA |
| NM_032111 | MRPL14 | 0.294620906 | 0.791301949 | 1.05279 | down | mRNA |
| NM_032112 | MRPL43 | 0.685619627 | 0.960111041 | 1.0230986 | down | mRNA |
| NM_032117 | MND1 | 0.792248109 | 0.978996327 | 1.2256769 | down | mRNA |
| NM_032120 | RBM48 | 0.009699008 | 0.206060838 | 1.2722651 | down | mRNA |
| NM_032121 | MAGT1 | 0.380404451 | 0.853407568 | 1.0535409 | down | mRNA |
| NM_032122 | DTNBP1 | 0.029160816 | 0.332155583 | 1.1082267 | down | mRNA |
| NM_032124 | HDHD2 | 0.474721326 | 0.899537186 | 1.0794717 | down | mRNA |
| NM_032125 | TMEM222 | 0.24089274 | 0.739116169 | 1.1892192 | down | mRNA |
| NM_032142 | CEP192 | 0.045835555 | 0.391271907 | 1.9235116 | down | mRNA |
| NM_032145 | FBXO30 | 0.389579686 | 0.858358711 | 1.3354553 | down | mRNA |
| NM_032151 | PCBD2 | 0.584243026 | 0.93461341 | 1.1070505 | down | mRNA |
| NM_032153 | ZIC4 | 0.728018475 | 0.966191059 | 1.0948924 | down | mRNA |
| NM_032154 | PCGF6 | 0.121321678 | 0.572987773 | 1.3204947 | down | mRNA |
| NM_032156 | CAPRIN2 | 0.004804225 | 0.158085585 | 1.6686383 | down | mRNA |
| NM_032166 | ATRIP | 0.521606288 | 0.916644975 | 1.1473424 | down | mRNA |
| NM_032168 | WDR75 | 0.034901859 | 0.352338753 | 1.2666279 | down | mRNA |
| NM_032169 | ACAD11 | 0.243359783 | 0.741870658 | 1.2365296 | down | mRNA |
| NM_032171 | CEP78 | 0.148206807 | 0.611101359 | 1.240121 | down | mRNA |
| NM_032173 | ZNRF3 | 0.633494576 | 0.951174475 | 1.0585572 | down | mRNA |
| NM_032174 | TOMM40L | 0.486779555 | 0.903524001 | 1.051272 | down | mRNA |
| NM_032175 | UTP15 | 0.123531026 | 0.577386854 | 1.1846871 | down | mRNA |
| NM_032177 | PHAX | 0.019459319 | 0.280600624 | 1.1585757 | down | mRNA |
| NM_032180 | FAM161A | 0.300198006 | 0.795797471 | 1.4098503 | down | mRNA |
| NM_032182 | FAM175B | 0.612564927 | 0.944179372 | 1.0686542 | down | mRNA |
| NM_032184 | SYDE2 | 0.055523823 | 0.42076421 | 2.9508178 | down | mRNA |
| NM_032188 | KAT8 | 0.29195015 | 0.788723152 | 1.0717512 | down | mRNA |
| NM_032192 | PPP1R1B | 0.877179096 | 0.991258481 | 1.0121332 | down | mRNA |
| NM_032195 | SON | 0.309724043 | 0.804356781 | 1.1270039 | down | mRNA |
| NM_032199 | ARID5B | 0.008622404 | 0.199898185 | 1.5175027 | down | mRNA |
| NM_032208 | ANTXR1 | 0.409301755 | 0.867591329 | 1.2620342 | down | mRNA |
| NM_032211 | LOXL4 | 0.242201289 | 0.740610726 | 2.4554824 | down | mRNA |
| NM_032213 | ELMOD3 | 0.983570489 | 0.998971807 | 1.0046909 | down | mRNA |
| NM_032214 | SLA2 | 0.398353572 | 0.863627032 | 1.1181291 | down | mRNA |
| NM_032217 | ANKRD17 | 0.022009449 | 0.292481125 | 1.1962858 | down | mRNA |
| NM_032219 | MFSD7 | 0.290278444 | 0.786468159 | 1.2862205 | down | mRNA |
| NM_032226 | ZCCHC7 | 0.391233826 | 0.859096647 | 1.2482754 | down | mRNA |
| NM_032228 | FAR1 | 0.023080705 | 0.298902477 | 1.1691764 | down | mRNA |
| NM_032231 | FAM96A | 0.136232361 | 0.594906342 | 1.1774308 | down | mRNA |
| NM_032233 | SETD3 | 0.116218219 | 0.562521166 | 1.2995739 | down | mRNA |
| NM_032236 | USP48 | 0.543228372 | 0.923577434 | 1.0712233 | down | mRNA |
| NM_032237 | SGK196 | 0.006359223 | 0.180328763 | 1.5223509 | down | mRNA |
| NM_032246 | MEX3B | 0.061755877 | 0.438568109 | 1.2303183 | down | mRNA |
| NM_032256 | TMEM117 | 0.162184835 | 0.634236412 | 1.3286948 | down | mRNA |
| NM_032259 | WDR24 | 0.88956444 | 0.9937521 | 1.0163541 | down | mRNA |
| NM_032261 | C21orf56 | 0.502627502 | 0.910335149 | 1.1912269 | down | mRNA |
| NM_032264 | NBPF3 | 0.690858941 | 0.961105466 | 1.0941384 | down | mRNA |
| NM_032266 | C2orf16 | 0.065171787 | 0.445966419 | 1.2869932 | down | mRNA |
| NM_032272 | MAF1 | 0.369086877 | 0.84565745 | 1.1002562 | down | mRNA |
| NM_032273 | TMEM126A | 0.398066442 | 0.863397365 | 1.0497892 | down | mRNA |
| NM_032280 | ZCCHC9 | 0.03576657 | 0.354907714 | 1.4214459 | down | mRNA |
| NM_032283 | ZDHHC18 | 0.743371269 | 0.970330526 | 1.024643 | down | mRNA |
| NM_032286 | MED10 | 0.608733478 | 0.943053241 | 1.0255404 | down | mRNA |
| NM_032287 | LDOC1L | 0.216939533 | 0.711077638 | 1.0898741 | down | mRNA |
| NM_032292 | GON4L | 0.187520034 | 0.671597204 | 1.071238 | down | mRNA |
| NM_032293 | GARNL3 | 0.764911018 | 0.97415819 | 1.0236238 | down | mRNA |
| NM_032295 | SLC37A3 | 0.240519875 | 0.738596904 | 1.1456389 | down | mRNA |
| NM_032296 | FLYWCH1 | 0.557832725 | 0.926833616 | 1.1124765 | down | mRNA |
| NM_032299 | DCUN1D5 | 0.27475296 | 0.774774195 | 1.0718667 | down | mRNA |
| NM_032300 | TCHP | 0.544012785 | 0.923577434 | 1.1727516 | down | mRNA |
| NM_032303 | HSDL2 | 0.475311761 | 0.899815707 | 1.0465027 | down | mRNA |
| NM_032304 | HAGHL | 0.968842135 | 0.998196842 | 1.002802 | down | mRNA |
| NM_032307 | C9orf64 | 0.063619681 | 0.440919074 | 1.4954726 | down | mRNA |
| NM_032310 | C9orf89 | 0.579954495 | 0.933893479 | 1.0456734 | down | mRNA |
| NM_032312 | YIPF4 | 0.937412731 | 0.996710991 | 1.0097736 | down | mRNA |
| NM_032313 | NOA1 | 0.08612264 | 0.49873128 | 1.273152 | down | mRNA |
| NM_032314 | COQ5 | 0.262518042 | 0.763125731 | 1.0926548 | down | mRNA |
| NM_032317 | DNAJC30 | 0.016051436 | 0.25477722 | 1.2793292 | down | mRNA |
| NM_032320 | BTBD10 | 0.420853054 | 0.872753026 | 1.1046531 | down | mRNA |
| NM_032322 | RNF135 | 0.068198175 | 0.45284301 | 1.5087949 | down | mRNA |
| NM_032324 | NTPCR | 0.95896484 | 0.997323434 | 1.0153362 | down | mRNA |
| NM_032325 | EIF1AD | 0.408040439 | 0.867591329 | 1.0458135 | down | mRNA |
| NM_032328 | EFCAB2 | 0.945208911 | 0.996710991 | 1.0116027 | down | mRNA |
| NM_032329 | ING5 | 0.034771126 | 0.352074963 | 1.3239518 | down | mRNA |
| NM_032330 | CAPNS2 | 0.485441704 | 0.903250293 | 1.1166681 | down | mRNA |
| NM_032332 | WDR83 | 0.716940251 | 0.966191059 | 1.1323164 | down | mRNA |
| NM_032334 | UTP23 | 0.794649234 | 0.979378567 | 1.013384 | down | mRNA |
| NM_032338 | LLPH | 0.361592579 | 0.842528113 | 1.0818184 | down | mRNA |
| NM_032341 | DDI2 | 0.269633479 | 0.76993462 | 1.1888927 | down | mRNA |
| NM_032343 | CHCHD6 | 0.889032252 | 0.993470148 | 1.0077369 | down | mRNA |
| NM_032346 | PDCD2L | 0.06676596 | 0.450692395 | 1.2471798 | down | mRNA |
| NM_032347 | ZNF397 | 0.740379651 | 0.969794316 | 1.0506444 | down | mRNA |
| NM_032352 | BRMS1L | 0.014966037 | 0.24511734 | 1.2491778 | down | mRNA |
| NM_032353 | VPS25 | 0.637052819 | 0.952463999 | 1.0426334 | down | mRNA |
| NM_032355 | MON1A | 0.587525488 | 0.93579682 | 1.10156 | down | mRNA |
| NM_032357 | CCDC115 | 0.655712951 | 0.957274052 | 1.0797848 | down | mRNA |
| NM_032360 | ACBD6 | 0.76233833 | 0.97339948 | 1.0518524 | down | mRNA |
| NM_032367 | ZBED3 | 0.868289911 | 0.989481868 | 1.0175512 | down | mRNA |
| NM_032368 | LZIC | 0.320660846 | 0.814552264 | 1.0693214 | down | mRNA |
| NM_032373 | PCGF5 | 0.384895782 | 0.855147437 | 1.121855 | down | mRNA |
| NM_032374 | APOPT1 | 0.609115952 | 0.943053241 | 1.0707767 | down | mRNA |
| NM_032376 | TMEM101 | 0.589728911 | 0.936090876 | 1.0565233 | down | mRNA |
| NM_032383 | HPS3 | 0.233523431 | 0.73118887 | 1.1527005 | down | mRNA |
| NM_032385 | C5orf4 | 0.352372287 | 0.835946719 | 1.0789566 | down | mRNA |
| NM_032387 | WNK4 | 0.969854951 | 0.998245244 | 1.0212885 | down | mRNA |
| NM_032408 | BAZ1B | 0.257330665 | 0.757735833 | 1.0729428 | down | mRNA |
| NM_032410 | HOOK3 | 0.922978957 | 0.995349793 | 1.0050564 | down | mRNA |
| NM_032414 | PROK1 | 0.874422142 | 0.990846067 | 1.0058993 | down | mRNA |
| NM_032417 | SPANXD | 0.516186995 | 0.914546356 | 1.0608787 | down | mRNA |
| NM_032424 | MSANTD4 | 0.03312829 | 0.346127643 | 1.3874846 | down | mRNA |
| NM_032425 | HHIPL1 | 0.807571458 | 0.98118927 | 1.0311953 | down | mRNA |
| NM_032429 | LZTS2 | 0.043842681 | 0.385351177 | 1.5554626 | down | mRNA |
| NM_032430 | BRSK1 | 0.655544669 | 0.957222998 | 1.0886478 | down | mRNA |
| NM_032433 | ZNF333 | 0.232234615 | 0.730136725 | 1.1232557 | down | mRNA |
| NM_032434 | ZNF512 | 0.501920048 | 0.910194076 | 1.2228408 | down | mRNA |
| NM_032437 | EFCAB7 | 0.02984907 | 0.335098261 | 1.1529847 | down | mRNA |
| NM_032442 | NEURL4 | 0.088000598 | 0.501570433 | 2.1373222 | down | mRNA |
| NM_032444 | SLX4 | 0.320050836 | 0.814325317 | 1.3677367 | down | mRNA |
| NM_032448 | FAM120B | 0.458824837 | 0.892904132 | 1.2887325 | down | mRNA |
| NM_032449 | CC2D1B | 0.502598979 | 0.910335149 | 1.0548209 | down | mRNA |
| NM_032451 | SPIRE2 | 0.19483625 | 0.682531124 | 1.1998951 | down | mRNA |
| NM_032458 | PHF6 | 0.539056023 | 0.923577434 | 1.1141674 | down | mRNA |
| NM_032466 | ASPH | 0.809823765 | 0.98118927 | 1.0483807 | down | mRNA |
| NM_032468 | ASPH | 0.010214872 | 0.20936306 | 1.3056406 | down | mRNA |
| NM_032472 | PPIL3 | 0.168144343 | 0.642977391 | 1.1107186 | down | mRNA |
| NM_032478 | MRPL38 | 0.031736978 | 0.341807319 | 1.4050876 | down | mRNA |
| NM_032479 | MRPL36 | 0.548641366 | 0.92514258 | 1.0433885 | down | mRNA |
| NM_032486 | DCTN5 | 0.933544309 | 0.996710991 | 1.0089243 | down | mRNA |
| NM_032487 | ACTRT3 | 0.035363489 | 0.353699423 | 1.3565353 | down | mRNA |
| NM_032490 | C14orf142 | 0.044827398 | 0.38849334 | 1.1349835 | down | mRNA |
| NM_032494 | ZC3H8 | 0.004216993 | 0.148338914 | 1.3327595 | down | mRNA |
| NM_032495 | HOPX | 0.079139559 | 0.482239465 | 1.6589063 | down | mRNA |
| NM_032497 | ZNF559 | 0.124776187 | 0.578441772 | 1.2247193 | down | mRNA |
| NM_032499 | C15orf41 | 0.542255583 | 0.923577434 | 1.0697105 | down | mRNA |
| NM_032509 | MAK16 | 0.004504994 | 0.154302821 | 1.2349088 | down | mRNA |
| NM_032511 | FAXC | 0.091401637 | 0.509983006 | 2.1330978 | down | mRNA |
| NM_032522 | ZBTB37 | 0.139731889 | 0.599246732 | 1.3097137 | down | mRNA |
| NM_032525 | TUBB6 | 0.423119646 | 0.873989281 | 1.0629835 | down | mRNA |
| NM_032530 | ZNF594 | 0.663736943 | 0.958354006 | 1.0764606 | down | mRNA |
| NM_032531 | KIRREL3 | 0.086990727 | 0.500185639 | 1.1128501 | down | mRNA |
| NM_032534 | KRBA1 | 0.170560248 | 0.64732212 | 1.4559173 | down | mRNA |
| NM_032549 | IMMP2L | 0.76874965 | 0.97524194 | 1.016782 | down | mRNA |
| NM_032552 | DAB2IP | 0.35478155 | 0.83766056 | 1.0566949 | down | mRNA |
| NM_032557 | USP38 | 0.022663192 | 0.296708317 | 1.4340528 | down | mRNA |
| NM_032558 | HIATL1 | 0.186876244 | 0.670590858 | 1.1115424 | down | mRNA |
| NM_032560 | SMEK1 | 0.005246786 | 0.165444121 | 1.4505688 | down | mRNA |
| NM_032565 | EBPL | 0.083759269 | 0.493003289 | 1.1750335 | down | mRNA |
| NM_032584 | ZNF347 | 0.603669107 | 0.941514447 | 1.2203309 | down | mRNA |
| NM_032591 | SLC9A7 | 0.868775718 | 0.989481868 | 1.0337246 | down | mRNA |
| NM_032595 | PPP1R9B | 0.161985318 | 0.63397218 | 1.3598784 | down | mRNA |
| NM_032601 | MCEE | 0.622662903 | 0.948341211 | 1.1775065 | down | mRNA |
| NM_032603 | LOXL3 | 0.38142342 | 0.853514109 | 1.6121892 | down | mRNA |
| NM_032609 | COX4I2 | 0.958622841 | 0.99717178 | 1.0124176 | down | mRNA |
| NM_032622 | LNX1 | 0.781215753 | 0.976938981 | 1.0826469 | down | mRNA |
| NM_032627 | SSBP4 | 0.560076828 | 0.927772696 | 1.0619691 | down | mRNA |
| NM_032630 | CINP | 0.727649224 | 0.966191059 | 1.0488337 | down | mRNA |
| NM_032632 | PAPOLA | 0.685626598 | 0.960111041 | 1.0335477 | down | mRNA |
| NM_032634 | PIGO | 0.336916075 | 0.826617309 | 1.3549349 | down | mRNA |
| NM_032635 | TMEM147 | 0.580404909 | 0.93393768 | 1.018647 | down | mRNA |
| NM_032642 | WNT5B | 0.001499038 | 0.098955994 | 1.4900654 | down | mRNA |
| NM_032643 | IRF5 | 0.994777147 | 0.999872237 | 1.000434 | down | mRNA |
| NM_032647 | CBX2 | 0.737091445 | 0.968834005 | 1.0428139 | down | mRNA |
| NM_032663 | USP30 | 0.068423834 | 0.454193073 | 1.8606606 | down | mRNA |
| NM_032664 | FUT10 | 0.175069336 | 0.653821873 | 1.1451909 | down | mRNA |
| NM_032673 | PCGF1 | 0.089711355 | 0.506107454 | 1.1866728 | down | mRNA |
| NM_032679 | ZNF577 | 0.266736694 | 0.767278687 | 1.279241 | down | mRNA |
| NM_032683 | MPV17L2 | 0.030237184 | 0.337236658 | 1.3729232 | down | mRNA |
| NM_032687 | CYHR1 | 0.768977379 | 0.975270932 | 1.0896359 | down | mRNA |
| NM_032704 | TUBA1C | 0.988785071 | 0.999140676 | 1.0026309 | down | mRNA |
| NM_032709 | PYROXD2 | 0.151420537 | 0.616599711 | 1.1287497 | down | mRNA |
| NM_032717 | AGPAT9 | 0.416374486 | 0.870736736 | 1.2569641 | down | mRNA |
| NM_032718 | MFSD9 | 0.27860034 | 0.777783404 | 1.1027906 | down | mRNA |
| NM_032730 | RTN4IP1 | 0.153801295 | 0.620938377 | 1.1310081 | down | mRNA |
| NM_032731 | TXNDC17 | 0.692788739 | 0.961584652 | 1.0717858 | down | mRNA |
| NM_032737 | LMNB2 | 0.868987616 | 0.989541093 | 1.074719 | down | mRNA |
| NM_032740 | SFT2D3 | 0.148123476 | 0.611080968 | 1.2096623 | down | mRNA |
| NM_032750 | ABHD14B | 0.163178329 | 0.63542336 | 1.130064 | down | mRNA |
| NM_032756 | HPDL | 0.067212051 | 0.45156203 | 1.291158 | down | mRNA |
| NM_032758 | PHF5A | 0.490569673 | 0.905300886 | 1.2211957 | down | mRNA |
| NM_032765 | TRIM52 | 0.081892151 | 0.488911764 | 1.2293573 | down | mRNA |
| NM_032776 | JMJD1C | 0.001409888 | 0.096194096 | 1.8559387 | down | mRNA |
| NM_032779 | CCDC142 | 0.219463619 | 0.714276371 | 1.100095 | down | mRNA |
| NM_032783 | CBR4 | 0.729007154 | 0.966191059 | 1.0385382 | down | mRNA |
| NM_032786 | ZC3H10 | 0.226146821 | 0.721636281 | 1.2330173 | down | mRNA |
| NM_032789 | PARP10 | 0.725324844 | 0.966191059 | 1.0450537 | down | mRNA |
| NM_032790 | ORAI1 | 0.041899318 | 0.378877886 | 1.2334341 | down | mRNA |
| NM_032795 | RPUSD4 | 0.603876638 | 0.941514447 | 1.0646812 | down | mRNA |
| NM_032796 | SYAP1 | 0.984806434 | 0.998971807 | 1.0013863 | down | mRNA |
| NM_032797 | AIFM2 | 0.503197446 | 0.910475258 | 1.1011929 | down | mRNA |
| NM_032799 | ZDHHC12 | 0.880709418 | 0.992287164 | 1.0099278 | down | mRNA |
| NM_032801 | JAM3 | 0.559147886 | 0.927203296 | 1.1714089 | down | mRNA |
| NM_032804 | ADO | 0.007210109 | 0.190384335 | 1.2991467 | down | mRNA |
| NM_032805 | ZSCAN10 | 0.896760837 | 0.994222243 | 1.0217413 | down | mRNA |
| NM_032806 | GTDC2 | 0.298789373 | 0.795233272 | 1.2616044 | down | mRNA |
| NM_032808 | LINGO1 | 0.835881642 | 0.986575022 | 1.043159 | down | mRNA |
| NM_032809 | FAM73B | 0.970499187 | 0.998245244 | 1.0093489 | down | mRNA |
| NM_032811 | TBRG1 | 0.498883053 | 0.909361611 | 1.2257153 | down | mRNA |
| NM_032812 | PLXDC2 | 0.390811789 | 0.858920947 | 1.0767578 | down | mRNA |
| NM_032813 | TMTC4 | 0.523534142 | 0.917239107 | 1.1080955 | down | mRNA |
| NM_032816 | CEP89 | 0.142433521 | 0.602652383 | 1.0707279 | down | mRNA |
| NM_032822 | FAM136A | 0.641127733 | 0.954066388 | 1.0617066 | down | mRNA |
| NM_032825 | ZNF382 | 0.541596978 | 0.923577434 | 1.3325022 | down | mRNA |
| NM_032827 | ATOH8 | 0.36063816 | 0.841780454 | 1.137208 | down | mRNA |
| NM_032828 | ZNF587 | 0.888278447 | 0.993440915 | 1.0288526 | down | mRNA |
| NM_032830 | CIRH1A | 0.241195642 | 0.73945497 | 1.1734553 | down | mRNA |
| NM_032831 | ORAI2 | 0.374451576 | 0.84936907 | 1.0334563 | down | mRNA |
| NM_032832 | LRP11 | 0.730487169 | 0.966329458 | 1.0335745 | down | mRNA |
| NM_032834 | ALG10 | 0.234564137 | 0.731962397 | 1.1619176 | down | mRNA |
| NM_032836 | FIZ1 | 0.987774959 | 0.999140676 | 1.0011219 | down | mRNA |
| NM_032837 | FAM104A | 0.126963406 | 0.580406758 | 1.0516238 | down | mRNA |
| NM_032839 | DIRC2 | 0.48871587 | 0.904236483 | 1.1124715 | down | mRNA |
| NM_032840 | SPRYD3 | 0.053661025 | 0.416065637 | 1.3029561 | down | mRNA |
| NM_032848 | C12orf52 | 0.02003665 | 0.283521851 | 1.131233 | down | mRNA |
| NM_032849 | C13orf33 | 0.095259591 | 0.516597924 | 1.4602975 | down | mRNA |
| NM_032853 | MUM1 | 0.04373424 | 0.385351177 | 1.8716724 | down | mRNA |
| NM_032856 | WDR73 | 0.063402926 | 0.440687083 | 1.3025695 | down | mRNA |
| NM_032859 | ABHD13 | 0.106238339 | 0.539783854 | 1.2104995 | down | mRNA |
| NM_032860 | LTV1 | 0.019674242 | 0.282298294 | 1.2650457 | down | mRNA |
| NM_032861 | SERAC1 | 0.537007627 | 0.922702335 | 1.3405904 | down | mRNA |
| NM_032864 | PRPF38A | 0.781760737 | 0.976938981 | 1.0226423 | down | mRNA |
| NM_032871 | RELT | 0.501616069 | 0.910145661 | 1.1282249 | down | mRNA |
| NM_032873 | UBASH3B | 0.376079534 | 0.849862269 | 1.0941263 | down | mRNA |
| NM_032875 | FBXL20 | 0.520876248 | 0.916605148 | 1.0959758 | down | mRNA |
| NM_032876 | AJUBA | 0.006715274 | 0.183552613 | 2.224329 | down | mRNA |
| NM_032878 | ALKBH6 | 0.725237174 | 0.966191059 | 1.041794 | down | mRNA |
| NM_032885 | ATG4D | 0.12478658 | 0.578441772 | 2.193813 | down | mRNA |
| NM_032889 | MFSD5 | 0.026840351 | 0.319244809 | 1.1740714 | down | mRNA |
| NM_032890 | DISP1 | 0.020183779 | 0.284617308 | 1.3300969 | down | mRNA |
| NM_032892 | FRMD5 | 0.283109466 | 0.780710928 | 1.1006976 | down | mRNA |
| NM_032902 | PPP1R16A | 0.289991463 | 0.786468159 | 1.1317905 | down | mRNA |
| NM_032924 | ZNF3 | 0.004026027 | 0.145287251 | 1.4897441 | down | mRNA |
| NM_032927 | TMEM128 | 0.046083381 | 0.391970965 | 1.4272721 | down | mRNA |
| NM_032930 | C11orf70 | 0.826690194 | 0.984436279 | 1.0235695 | down | mRNA |
| NM_032936 | TMEM60 | 0.169399492 | 0.645833579 | 1.1754182 | down | mRNA |
| NM_032937 | C9orf37 | 0.083471422 | 0.492392714 | 1.1421387 | down | mRNA |
| NM_032940 | POLR2C | 0.197154038 | 0.685161614 | 1.130467 | down | mRNA |
| NM_032941 | RECQL | 0.345351828 | 0.832533915 | 1.1139211 | down | mRNA |
| NM_032943 | SYTL2 | 0.52536716 | 0.917686109 | 1.2557516 | down | mRNA |
| NM_032947 | C5orf62 | 0.001217485 | 0.09206987 | 2.0221554 | down | mRNA |
| NM_032952 | MLXIPL | 0.439483953 | 0.883054028 | 1.1508657 | down | mRNA |
| NM_032955 | AIF1 | 0.849301826 | 0.987678166 | 1.0195885 | down | mRNA |
| NM_032957 | RTEL1 | 0.977728478 | 0.998501954 | 1.0031128 | down | mRNA |
| NM_032959 | POLR2J2 | 0.982862358 | 0.998971807 | 1.0016647 | down | mRNA |
| NM_032963 | CCL14 | 0.296556597 | 0.793158213 | 1.0764845 | down | mRNA |
| NM_032970 | SEC22C | 0.284486563 | 0.78179061 | 1.1786533 | down | mRNA |
| NM_032992 | CASP6 | 0.116882921 | 0.563811043 | 1.1349425 | down | mRNA |
| NM_032999 | GTF2I | 0.002747212 | 0.12412513 | 1.2619465 | down | mRNA |
| NM_033000 | GTF2I | 0.000613255 | 0.074865247 | 1.3858145 | down | mRNA |
| NM_033001 | GTF2I | 0.004233064 | 0.148390786 | 1.4384595 | down | mRNA |
| NM_033004 | NLRP1 | 0.006801279 | 0.183643574 | 1.1864851 | down | mRNA |
| NM_033006 | NLRP1 | 0.747059776 | 0.970433131 | 1.0199324 | down | mRNA |
| NM_033008 | PCBP4 | 0.597059384 | 0.939714862 | 1.0618668 | down | mRNA |
| NM_033015 | FASTK | 0.296036697 | 0.792404038 | 1.073996 | down | mRNA |
| NM_033020 | TRIM33 | 0.003889081 | 0.143768711 | 1.4748535 | down | mRNA |
| NM_033032 | KRTAP2-2 | 0.879150336 | 0.992149178 | 1.0795703 | down | mRNA |
| NM_033034 | TRIM5 | 0.885664467 | 0.992981836 | 1.0447409 | down | mRNA |
| NM_033036 | GAL3ST3 | 0.919716717 | 0.995349793 | 1.0181242 | down | mRNA |
| NM_033046 | RTKN | 0.102623048 | 0.531600463 | 1.2950347 | down | mRNA |
| NM_033055 | HIAT1 | 0.403037202 | 0.864786348 | 1.1498357 | down | mRNA |
| NM_033056 | PCDH15 | 0.617842251 | 0.946754755 | 1.227849 | down | mRNA |
| NM_033058 | TRIM55 | 0.882434434 | 0.992766819 | 1.0142824 | down | mRNA |
| NM_033059 | KRTAP4-11 | 0.362300822 | 0.843173209 | 1.5897997 | down | mRNA |
| NM_033063 | MAP6 | 0.05151457 | 0.41031217 | 1.1996501 | down | mRNA |
| NM_033066 | MPP4 | 0.844403548 | 0.987338076 | 1.0176342 | down | mRNA |
| NM_033071 | SYNE1 | 0.00416656 | 0.147329558 | 1.5127895 | down | mRNA |
| NM_033081 | DIDO1 | 0.051127697 | 0.409102061 | 1.2913814 | down | mRNA |
| NM_033087 | ALG2 | 0.532893344 | 0.920530493 | 1.0528849 | down | mRNA |
| NM_033088 | FAM40A | 0.207386791 | 0.698556218 | 1.2579339 | down | mRNA |
| NM_033089 | ZCCHC3 | 0.638974837 | 0.953242382 | 1.0490552 | down | mRNA |
| NM_033091 | TRIM4 | 0.101099897 | 0.528558268 | 1.1133306 | down | mRNA |
| NM_033100 | CDHR1 | 0.790671168 | 0.978509111 | 1.0536232 | down | mRNA |
| NM_033101 | LGALS12 | 0.58865478 | 0.93579682 | 1.1075903 | down | mRNA |
| NM_033104 | STON2 | 0.874206034 | 0.990793509 | 1.026403 | down | mRNA |
| NM_033107 | GTPBP10 | 0.269012562 | 0.769710583 | 1.1525879 | down | mRNA |
| NM_033109 | PNPT1 | 0.288049778 | 0.784334819 | 1.2337226 | down | mRNA |
| NM_033110 | A2LD1 | 0.49870489 | 0.909361611 | 1.0775507 | down | mRNA |
| NM_033112 | RRP36 | 0.151305099 | 0.616500055 | 1.1415049 | down | mRNA |
| NM_033114 | ZCRB1 | 0.381175101 | 0.853514109 | 1.0567038 | down | mRNA |
| NM_033116 | NEK9 | 0.193471486 | 0.680621781 | 1.0960171 | down | mRNA |
| NM_033117 | RBM18 | 0.182956819 | 0.666166668 | 1.092593 | down | mRNA |
| NM_033121 | ANKRD13A | 0.031449244 | 0.341322176 | 1.1926163 | down | mRNA |
| NM_033124 | CCDC65 | 0.68689612 | 0.960205549 | 1.1376645 | down | mRNA |
| NM_033126 | PSKH2 | 0.839787322 | 0.987068842 | 1.0535725 | down | mRNA |
| NM_033129 | SCRT2 | 0.815060631 | 0.981277402 | 1.0346793 | down | mRNA |
| NM_033130 | SIGLEC10 | 0.67308708 | 0.959531882 | 1.114323 | down | mRNA |
| NM_033133 | CNP | 0.11085009 | 0.551187095 | 1.1957905 | down | mRNA |
| NM_033135 | PDGFD | 0.587727436 | 0.93579682 | 1.6391769 | down | mRNA |
| NM_033138 | CALD1 | 0.929481723 | 0.996533591 | 1.0257901 | down | mRNA |
| NM_033139 | CALD1 | 0.871246838 | 0.98985097 | 1.0571839 | down | mRNA |
| NM_033140 | CALD1 | 0.572345845 | 0.933397932 | 1.1064761 | down | mRNA |
| NM_033141 | MAP3K9 | 0.294950633 | 0.791301949 | 1.2396126 | down | mRNA |
| NM_033147 | DTNB | 0.366293294 | 0.845181673 | 1.1126581 | down | mRNA |
| NM_033150 | COL2A1 | 0.812982865 | 0.98120545 | 1.0451203 | down | mRNA |
| NM_033158 | HYAL2 | 0.764236161 | 0.973899357 | 1.0289615 | down | mRNA |
| NM_033160 | ZNF658 | 0.913986704 | 0.995342392 | 1.0060591 | down | mRNA |
| NM_033161 | SURF4 | 0.387711733 | 0.85702924 | 1.0692928 | down | mRNA |
| NM_033164 | FGF8 | 0.98932189 | 0.999140676 | 1.0023453 | down | mRNA |
| NM_033168 | B3GALNT1 | 0.120439421 | 0.571691731 | 1.1227822 | down | mRNA |
| NM_033177 | GPANK1 | 0.080269911 | 0.483876854 | 1.2645688 | down | mRNA |
| NM_033179 | OR51B4 | 0.815505338 | 0.981459009 | 1.0342229 | down | mRNA |
| NM_033183 | CGB8 | 0.139066501 | 0.598648178 | 1.7570518 | down | mRNA |
| NM_033198 | PIGS | 0.25672122 | 0.757240077 | 1.0852636 | down | mRNA |
| NM_033208 | TIGD7 | 0.583044128 | 0.934269626 | 1.0396631 | down | mRNA |
| NM_033211 | C5orf30 | 0.004914028 | 0.158842626 | 1.5403311 | down | mRNA |
| NM_033212 | CCDC102A | 0.011449393 | 0.218702965 | 1.2919459 | down | mRNA |
| NM_033225 | CSMD1 | 0.228403312 | 0.724599197 | 1.1928851 | down | mRNA |
| NM_033227 | TRIM23 | 0.04385594 | 0.385351177 | 1.2729995 | down | mRNA |
| NM_033228 | TRIM23 | 0.012721148 | 0.230112444 | 1.2918954 | down | mRNA |
| NM_033251 | RPL13 | 0.95292693 | 0.996710991 | 1.0064741 | down | mRNA |
| NM_033254 | BOC | 0.760647893 | 0.972931992 | 1.0884107 | down | mRNA |
| NM_033255 | EPSTI1 | 0.853126354 | 0.987678166 | 1.0537142 | down | mRNA |
| NM_033256 | PPP1R14A | 0.818137313 | 0.981818735 | 1.0426751 | down | mRNA |
| NM_033257 | DGCR6L | 0.819065847 | 0.982144775 | 1.0642953 | down | mRNA |
| NM_033260 | FOXQ1 | 0.075072349 | 0.470853901 | 1.8654942 | down | mRNA |
| NM_033266 | ERN2 | 0.399483545 | 0.863982495 | 1.30566 | down | mRNA |
| NM_033267 | IRX2 | 0.379028741 | 0.852573556 | 1.1808338 | down | mRNA |
| NM_033271 | BTBD6 | 0.034234754 | 0.350517496 | 1.1618889 | down | mRNA |
| NM_033276 | XRCC6BP1 | 0.541597071 | 0.923577434 | 1.1360589 | down | mRNA |
| NM_033278 | TRIM3 | 0.875272034 | 0.991094934 | 1.0168303 | down | mRNA |
| NM_033282 | OPN4 | 0.695501763 | 0.96236244 | 1.15455 | down | mRNA |
| NM_033286 | C15orf23 | 0.236551628 | 0.73465251 | 1.345837 | down | mRNA |
| NM_033290 | MID1 | 0.008910485 | 0.202329498 | 1.2606831 | down | mRNA |
| NM_033293 | CASP1 | 0.123744942 | 0.577744894 | 1.1572562 | down | mRNA |
| NM_033294 | CASP1 | 0.926651472 | 0.99582096 | 1.0692255 | down | mRNA |
| NM_033295 | CASP1 | 0.986393763 | 0.998971807 | 1.0121054 | down | mRNA |
| NM_033296 | MRFAP1 | 0.714253579 | 0.966110889 | 1.058526 | down | mRNA |
| NM_033297 | NLRP12 | 0.843340543 | 0.987338076 | 1.0555788 | down | mRNA |
| NM_033300 | LRP8 | 0.91257364 | 0.995342392 | 1.042346 | down | mRNA |
| NM_033309 | B3GNT9 | 0.65145431 | 0.956204521 | 1.0208938 | down | mRNA |
| NM_033312 | CDC14A | 0.31723544 | 0.811824899 | 1.0347421 | down | mRNA |
| NM_033316 | MFI2 | 0.816768088 | 0.981692737 | 1.3006605 | down | mRNA |
| NM_033317 | DMKN | 0.779529694 | 0.976938981 | 1.0660571 | down | mRNA |
| NM_033318 | C22orf32 | 0.603798134 | 0.941514447 | 1.0334515 | down | mRNA |
| NM_033319 | CENPL | 0.428708915 | 0.877386753 | 1.4476608 | down | mRNA |
| NM_033342 | TRIM7 | 0.564849034 | 0.929820378 | 1.3448266 | down | mRNA |
| NM_033360 | KRAS | 0.079960932 | 0.483876854 | 1.2824186 | down | mRNA |
| NM_033379 | CDK1 | 0.907604606 | 0.995145546 | 1.0858967 | down | mRNA |
| NM_033388 | ATG16L2 | 0.839295929 | 0.987032229 | 1.0352382 | down | mRNA |
| NM_033389 | SSH2 | 0.356125294 | 0.838847221 | 1.1046116 | down | mRNA |
| NM_033395 | KIAA1731 | 0.056743757 | 0.424322651 | 1.6829728 | down | mRNA |
| NM_033396 | TNKS1BP1 | 0.726785671 | 0.966191059 | 1.0199743 | down | mRNA |
| NM_033402 | LRRCC1 | 0.266074182 | 0.767023998 | 1.2896737 | down | mRNA |
| NM_033405 | PRIC285 | 0.700875641 | 0.962991468 | 1.0840623 | down | mRNA |
| NM_033406 | FBXO3 | 0.237209193 | 0.735183924 | 1.1653846 | down | mRNA |
| NM_033407 | DOCK7 | 0.078963593 | 0.481550021 | 1.225767 | down | mRNA |
| NM_033411 | RWDD2A | 0.540103748 | 0.923577434 | 1.0593711 | down | mRNA |
| NM_033414 | ZNF622 | 0.781711341 | 0.976938981 | 1.0444907 | down | mRNA |
| NM_033415 | ARMC6 | 0.091330914 | 0.509728287 | 1.2633363 | down | mRNA |
| NM_033417 | HAUS8 | 0.91569861 | 0.995349793 | 1.0264403 | down | mRNA |
| NM_033418 | METTL18 | 0.031493232 | 0.341322176 | 1.5855752 | down | mRNA |
| NM_033421 | SNX21 | 0.841870858 | 0.987123192 | 1.0284746 | down | mRNA |
| NM_033446 | FAM125B | 0.03103695 | 0.340185047 | 1.656714 | down | mRNA |
| NM_033452 | TRIM47 | 0.745937968 | 0.970394407 | 1.0769503 | down | mRNA |
| NM_033468 | ZNF257 | 0.195685012 | 0.683572955 | 1.1710738 | down | mRNA |
| NM_033480 | FBXO9 | 0.022899415 | 0.298107041 | 1.2393844 | down | mRNA |
| NM_033481 | FBXO9 | 0.292304491 | 0.788954556 | 1.1076829 | down | mRNA |
| NM_033484 | FBXO4 | 0.681623975 | 0.960111041 | 1.0397994 | down | mRNA |
| NM_033496 | HK1 | 0.445135811 | 0.885731305 | 1.2142604 | down | mRNA |
| NM_033500 | HK1 | 0.387421204 | 0.856773191 | 1.0687843 | down | mRNA |
| NM_033502 | TRERF1 | 0.464773173 | 0.894989844 | 1.1215628 | down | mRNA |
| NM_033504 | TMEM54 | 0.484347992 | 0.903250293 | 1.0843824 | down | mRNA |
| NM_033506 | FBXO24 | 0.147133363 | 0.609351845 | 1.2327656 | down | mRNA |
| NM_033512 | TSPYL5 | 0.425232192 | 0.875083091 | 1.1728276 | down | mRNA |
| NM_033513 | TPGS1 | 0.200597929 | 0.68900633 | 1.0878455 | down | mRNA |
| NM_033515 | ARHGAP18 | 0.893722082 | 0.993978977 | 1.0438397 | down | mRNA |
| NM_033518 | SLC38A5 | 0.740462668 | 0.969794316 | 1.1126658 | down | mRNA |
| NM_033540 | MFN1 | 0.254347314 | 0.75479352 | 1.2342278 | down | mRNA |
| NM_033547 | INTS4 | 0.079593237 | 0.482639336 | 1.3822663 | down | mRNA |
| NM_033549 | TRIM41 | 0.442980438 | 0.884304813 | 1.0872658 | down | mRNA |
| NM_033550 | TP53RK | 0.049301182 | 0.402199077 | 1.3943258 | down | mRNA |
| NM_033624 | FBXO21 | 0.502817506 | 0.910354901 | 1.1177421 | down | mRNA |
| NM_033625 | RPL34 | 0.994726769 | 0.999872237 | 1.0003321 | down | mRNA |
| NM_033626 | CCDC120 | 0.464236231 | 0.894989844 | 1.123389 | down | mRNA |
| NM_033632 | FBXW7 | 0.275761237 | 0.775616353 | 1.1752006 | down | mRNA |
| NM_033637 | BTRC | 0.342606018 | 0.831288811 | 1.1340858 | down | mRNA |
| NM_033641 | COL4A6 | 0.685565872 | 0.960111041 | 1.0235515 | down | mRNA |
| NM_033655 | CNTNAP3 | 0.585470937 | 0.934804075 | 1.1132652 | down | mRNA |
| NM_033656 | BRWD1 | 0.067050232 | 0.451412357 | 1.2513026 | down | mRNA |
| NM_033657 | DAP3 | 0.808958377 | 0.98118927 | 1.018172 | down | mRNA |
| NM_033661 | WDR4 | 0.779780009 | 0.976938981 | 1.0652411 | down | mRNA |
| NM_048368 | CTDP1 | 0.015778439 | 0.252653581 | 1.4695559 | down | mRNA |
| NM_052814 | CARD9 | 0.058245803 | 0.429558128 | 1.379235 | down | mRNA |
| NM_052821 | WDR5 | 0.509050808 | 0.911674633 | 1.0932987 | down | mRNA |
| NM_052831 | SLC18B1 | 0.125357003 | 0.578441772 | 1.3436344 | down | mRNA |
| NM_052837 | SCAMP3 | 0.963408063 | 0.99793927 | 1.0037441 | down | mRNA |
| NM_052839 | PANX2 | 0.790006066 | 0.978438986 | 1.091969 | down | mRNA |
| NM_052840 | CELF6 | 0.900848778 | 0.994789506 | 1.0206354 | down | mRNA |
| NM_052844 | WDR34 | 0.79621801 | 0.980479958 | 1.0484265 | down | mRNA |
| NM_052845 | MMAB | 0.026705514 | 0.318484367 | 1.1238683 | down | mRNA |
| NM_052850 | GADD45GIP1 | 0.740600952 | 0.96991295 | 1.0359457 | down | mRNA |
| NM_052852 | ZNF486 | 0.083775642 | 0.493003289 | 1.3143379 | down | mRNA |
| NM_052853 | ADCK2 | 0.119299885 | 0.568456822 | 1.1047804 | down | mRNA |
| NM_052855 | ANKRD40 | 0.475665015 | 0.900105754 | 1.0716668 | down | mRNA |
| NM_052859 | RFT1 | 0.097894378 | 0.520535504 | 1.1671977 | down | mRNA |
| NM_052863 | SCGB3A1 | 0.876677324 | 0.991255275 | 1.0421251 | down | mRNA |
| NM_052865 | C20orf72 | 0.482226885 | 0.902239535 | 1.203871 | down | mRNA |
| NM_052866 | ADAMTSL1 | 0.960429227 | 0.997738713 | 1.0124222 | down | mRNA |
| NM_052867 | NALCN | 0.04587851 | 0.391440141 | 1.4874148 | down | mRNA |
| NM_052868 | IGSF8 | 0.938465571 | 0.996710991 | 1.0118537 | down | mRNA |
| NM_052873 | IFT43 | 0.745322668 | 0.970378122 | 1.0813799 | down | mRNA |
| NM_052875 | VPS26B | 0.265693607 | 0.766537754 | 1.0833785 | down | mRNA |
| NM_052877 | MED8 | 0.957254247 | 0.996883425 | 1.0082755 | down | mRNA |
| NM_052880 | PIK3IP1 | 0.564377035 | 0.929730843 | 1.0536015 | down | mRNA |
| NM_052886 | MAL2 | 0.902028489 | 0.994906068 | 1.01757 | down | mRNA |
| NM_052897 | MBD6 | 0.342087187 | 0.830550068 | 1.0616803 | down | mRNA |
| NM_052899 | GPRIN1 | 0.914567508 | 0.995342392 | 1.0111639 | down | mRNA |
| NM_052903 | TUBGCP5 | 0.023147156 | 0.299191332 | 1.2964024 | down | mRNA |
| NM_052906 | ELFN2 | 0.949908515 | 0.996710991 | 1.0152237 | down | mRNA |
| NM_052909 | PLEKHG4B | 0.841959807 | 0.987123192 | 1.065328 | down | mRNA |
| NM_052911 | ESCO1 | 0.007766061 | 0.19345927 | 1.4773692 | down | mRNA |
| NM_052913 | TMEM200A | 0.363784428 | 0.843238468 | 1.2524067 | down | mRNA |
| NM_052917 | GALNT13 | 0.347620068 | 0.833960025 | 1.5082811 | down | mRNA |
| NM_052918 | SORCS1 | 0.6390842 | 0.953258011 | 1.0766392 | down | mRNA |
| NM_052920 | KLHL29 | 0.977058185 | 0.998501532 | 1.0017715 | down | mRNA |
| NM_052926 | PNMA5 | 0.133112431 | 0.58899716 | 1.0675638 | down | mRNA |
| NM_052927 | PWWP2A | 0.799485205 | 0.981056986 | 1.080159 | down | mRNA |
| NM_052928 | SMYD4 | 0.294132338 | 0.791146775 | 1.5158401 | down | mRNA |
| NM_052935 | NT5C3L | 0.110031929 | 0.549011353 | 1.1035316 | down | mRNA |
| NM_052936 | ATG4A | 0.574814025 | 0.933656777 | 1.0809451 | down | mRNA |
| NM_052940 | LRRC42 | 0.072834222 | 0.463930261 | 1.1815052 | down | mRNA |
| NM_052950 | WDFY2 | 0.451548506 | 0.888854908 | 1.0629507 | down | mRNA |
| NM_052953 | LRRC3B | 0.129916696 | 0.583907255 | 1.5186604 | down | mRNA |
| NM_052960 | RBP7 | 0.699635484 | 0.962904533 | 1.0915923 | down | mRNA |
| NM_052965 | TSEN15 | 0.404567612 | 0.865859862 | 1.1398848 | down | mRNA |
| NM_052978 | TRIM9 | 0.656344818 | 0.957440295 | 1.0999134 | down | mRNA |
| NM_052987 | CDK10 | 0.019985018 | 0.283521851 | 1.1189197 | down | mRNA |
| NM_052989 | IFT122 | 0.104718401 | 0.535631326 | 1.7089703 | down | mRNA |
| NM_052990 | IFT122 | 0.237711767 | 0.735771951 | 1.1303988 | down | mRNA |
| NM_053001 | OSR2 | 0.042319557 | 0.380221491 | 1.6143506 | down | mRNA |
| NM_053004 | GNB1L | 0.036936564 | 0.359628653 | 1.4864312 | down | mRNA |
| NM_053016 | PALM2 | 0.501922879 | 0.910194076 | 1.0978565 | down | mRNA |
| NM_053035 | MRPS33 | 0.943559304 | 0.996710991 | 1.0068017 | down | mRNA |
| NM_053041 | COMMD7 | 0.635683253 | 0.951814727 | 1.0949202 | down | mRNA |
| NM_053042 | ZNF518B | 0.184423318 | 0.66791858 | 1.1710941 | down | mRNA |
| NM_053043 | RBM33 | 0.367420345 | 0.845181673 | 1.1724875 | down | mRNA |
| NM_053044 | HTRA3 | 0.809325634 | 0.98118927 | 1.0272991 | down | mRNA |
| NM_053045 | TMEM203 | 0.006978014 | 0.186688411 | 1.3971202 | down | mRNA |
| NM_053046 | EGLN2 | 0.017578613 | 0.267267899 | 1.2596805 | down | mRNA |
| NM_053050 | MRPL53 | 0.074180881 | 0.469200549 | 1.2346553 | down | mRNA |
| NM_053052 | SNAP47 | 0.001066193 | 0.086778252 | 1.4010011 | down | mRNA |
| NM_053053 | TADA1 | 0.030195984 | 0.337147038 | 1.3433312 | down | mRNA |
| NM_053055 | THEM4 | 0.475696645 | 0.900105754 | 1.1208707 | down | mRNA |
| NM_053056 | CCND1 | 0.167818283 | 0.642697559 | 1.1296641 | down | mRNA |
| NM_053067 | UBQLN1 | 0.009426079 | 0.204717305 | 1.1952392 | down | mRNA |
| NM_053274 | GLMN | 0.034935812 | 0.352338753 | 1.0964716 | down | mRNA |
| NM_053284 | WFIKKN1 | 0.454984458 | 0.891140128 | 1.2138702 | down | mRNA |
| NM_053285 | TEKT1 | 0.288941043 | 0.784764798 | 1.1331453 | down | mRNA |
| NM_054016 | SRSF10 | 0.573871748 | 0.933656777 | 1.046773 | down | mRNA |
| NM_054026 | CNOT7 | 0.087062609 | 0.500185639 | 1.1543814 | down | mRNA |
| NM_054027 | ANKH | 0.060301439 | 0.435378361 | 1.4564492 | down | mRNA |
| NM_054033 | FKBP1B | 0.195262553 | 0.68296546 | 1.3339942 | down | mRNA |
| NM_054034 | FN1 | 0.811670944 | 0.98118927 | 1.0399086 | down | mRNA |
| NM_054035 | UNC119 | 0.088431953 | 0.502587299 | 1.4937474 | down | mRNA |
| NM_054112 | DEFB118 | 0.593976544 | 0.938299318 | 1.0537886 | down | mRNA |
| NM_057158 | DUSP4 | 0.135444006 | 0.593245913 | 1.9718794 | down | mRNA |
| NM_057161 | KLHDC3 | 0.539545748 | 0.923577434 | 1.0925686 | down | mRNA |
| NM_057165 | COL6A3 | 0.699505662 | 0.962856213 | 1.0777045 | down | mRNA |
| NM_057166 | COL6A3 | 0.457872917 | 0.892566841 | 1.0379097 | down | mRNA |
| NM_057168 | WNT16 | 0.058080785 | 0.42926155 | 1.0711328 | down | mRNA |
| NM_057169 | GIT2 | 0.021208488 | 0.288424269 | 1.1892232 | down | mRNA |
| NM_057170 | GIT2 | 0.145177647 | 0.606482829 | 1.2243022 | down | mRNA |
| NM_057175 | NAA15 | 0.039132736 | 0.369381053 | 1.1814 | down | mRNA |
| NM_057749 | CCNE2 | 0.855193752 | 0.988178307 | 1.1429623 | down | mRNA |
| NM_058163 | TSR2 | 0.558368404 | 0.926833616 | 1.0588604 | down | mRNA |
| NM_058167 | UBE2J2 | 0.167663454 | 0.642346587 | 1.0803112 | down | mRNA |
| NM_058169 | LOH12CR1 | 0.94646507 | 0.996710991 | 1.0047266 | down | mRNA |
| NM_058180 | C21orf58 | 0.843167113 | 0.987338076 | 1.0296549 | down | mRNA |
| NM_058186 | FAM3B | 0.399879917 | 0.864196266 | 1.0511953 | down | mRNA |
| NM_058187 | FAM176C | 0.026256601 | 0.317396349 | 1.2961967 | down | mRNA |
| NM_058200 | SPAG11B | 0.865460884 | 0.989401142 | 1.0518426 | down | mRNA |
| NM_058202 | SPAG11B | 0.185860271 | 0.669321303 | 1.204346 | down | mRNA |
| NM_058216 | RAD51C | 0.166839308 | 0.641842579 | 1.1365677 | down | mRNA |
| NM_058229 | FBXO32 | 0.484321682 | 0.903250293 | 1.1511584 | down | mRNA |
| NM_058230 | ZNF354B | 2.11662E-05 | 0.018978458 | 1.6907336 | down | mRNA |
| NM_058241 | CCNT2 | 0.789838614 | 0.978312439 | 1.025229 | down | mRNA |
| NM_058246 | DNAJB6 | 0.700692772 | 0.962991468 | 1.0869658 | down | mRNA |
| NM_078468 | BCCIP | 0.224699473 | 0.719349659 | 1.1118365 | down | mRNA |
| NM_078469 | BCCIP | 0.456904884 | 0.891568474 | 1.0284037 | down | mRNA |
| NM_078470 | COX15 | 0.306365282 | 0.801653799 | 1.0815257 | down | mRNA |
| NM_078471 | MYO18A | 0.387044196 | 0.856578056 | 1.2009901 | down | mRNA |
| NM_078473 | TM2D2 | 0.607297762 | 0.942627675 | 1.0353037 | down | mRNA |
| NM_078474 | TM2D3 | 0.207161419 | 0.698556218 | 1.3123179 | down | mRNA |
| NM_078476 | BTN2A1 | 0.218143225 | 0.712269655 | 1.1905355 | down | mRNA |
| NM_078483 | SLC36A1 | 0.002177481 | 0.11120755 | 1.179954 | down | mRNA |
| NM_078626 | CDKN2C | 0.26401853 | 0.764566979 | 1.3056517 | down | mRNA |
| NM_078628 | MSL3 | 0.595697066 | 0.938746918 | 1.0188866 | down | mRNA |
| NM_078629 | MSL3 | 0.756782281 | 0.971824502 | 1.0192789 | down | mRNA |
| NM_079420 | MYL1 | 0.906857914 | 0.995145546 | 1.0143816 | down | mRNA |
| NM_080388 | S100A16 | 0.510563124 | 0.912251098 | 1.0594416 | down | mRNA |
| NM_080390 | TCEAL2 | 0.722200484 | 0.966191059 | 1.057553 | down | mRNA |
| NM_080413 | VPS16 | 0.013045676 | 0.233490037 | 1.1578078 | down | mRNA |
| NM_080424 | SP110 | 0.481530114 | 0.901682656 | 1.0582026 | down | mRNA |
| NM_080426 | GNAS | 0.195250888 | 0.68296546 | 1.0922865 | down | mRNA |
| NM_080431 | ACTRT2 | 0.876768917 | 0.991255275 | 1.0408049 | down | mRNA |
| NM_080476 | PIGU | 0.044098491 | 0.386137176 | 1.2393621 | down | mRNA |
| NM_080539 | COLQ | 0.272124003 | 0.772141487 | 1.119028 | down | mRNA |
| NM_080546 | SLC44A1 | 0.368005348 | 0.845264883 | 1.1176468 | down | mRNA |
| NM_080550 | SYNRG | 0.005442344 | 0.168942963 | 1.3507238 | down | mRNA |
| NM_080590 | CAPS | 0.350932086 | 0.835106645 | 1.0551427 | down | mRNA |
| NM_080591 | PTGS1 | 0.891875511 | 0.9937521 | 1.0783925 | down | mRNA |
| NM_080592 | ATRAID | 0.013901508 | 0.237716955 | 1.2556708 | down | mRNA |
| NM_080593 | HIST1H2BK | 0.8352992 | 0.986575022 | 1.015711 | down | mRNA |
| NM_080594 | RNPS1 | 0.233854322 | 0.73118887 | 1.071548 | down | mRNA |
| NM_080597 | OSBPL1A | 0.577183148 | 0.933656777 | 1.1204503 | down | mRNA |
| NM_080598 | DDX39B | 0.807357946 | 0.98118927 | 1.032778 | down | mRNA |
| NM_080599 | UPF2 | 0.266642704 | 0.767248719 | 1.1614771 | down | mRNA |
| NM_080603 | ZSWIM1 | 0.97825793 | 0.998641374 | 1.003028 | down | mRNA |
| NM_080605 | B3GALT6 | 0.20266598 | 0.691983154 | 1.142719 | down | mRNA |
| NM_080612 | GAB3 | 0.398309598 | 0.863627032 | 1.0418933 | down | mRNA |
| NM_080616 | C20orf112 | 0.022171647 | 0.293936579 | 1.3516881 | down | mRNA |
| NM_080631 | VPS41 | 0.863921892 | 0.989401142 | 1.0341731 | down | mRNA |
| NM_080645 | COL12A1 | 0.220447848 | 0.715799221 | 1.0684064 | down | mRNA |
| NM_080648 | APEX1 | 0.218027593 | 0.712269655 | 1.1340818 | down | mRNA |
| NM_080650 | ATPBD4 | 0.985711394 | 0.998971807 | 1.0016059 | down | mRNA |
| NM_080654 | CCDC34 | 0.335779345 | 0.825621677 | 1.2050894 | down | mRNA |
| NM_080659 | C11orf52 | 0.788342701 | 0.977641695 | 1.0641216 | down | mRNA |
| NM_080664 | C14orf126 | 0.631279105 | 0.950410442 | 1.0716299 | down | mRNA |
| NM_080667 | CCDC104 | 0.751204683 | 0.970725371 | 1.0131956 | down | mRNA |
| NM_080677 | DYNLL2 | 0.852998092 | 0.987678166 | 1.0178935 | down | mRNA |
| NM_080678 | UBE2F | 0.173002452 | 0.650904119 | 1.1408981 | down | mRNA |
| NM_080681 | COL11A2 | 0.831008965 | 0.985710869 | 1.0127357 | down | mRNA |
| NM_080683 | PTPN13 | 0.810701773 | 0.98118927 | 1.1232566 | down | mRNA |
| NM_080684 | PTPN13 | 0.969364092 | 0.998245244 | 1.0096757 | down | mRNA |
| NM_080685 | PTPN13 | 0.431602639 | 0.880121832 | 1.2158135 | down | mRNA |
| NM_080723 | NRSN1 | 0.035466183 | 0.353826517 | 1.2241201 | down | mRNA |
| NM_080725 | SRXN1 | 0.570633291 | 0.93296476 | 1.2493335 | down | mRNA |
| NM_080738 | EDARADD | 0.317999183 | 0.811950192 | 1.3683709 | down | mRNA |
| NM_080742 | B3GAT2 | 0.922700268 | 0.995349793 | 1.0166793 | down | mRNA |
| NM_080745 | TRIM69 | 0.851325079 | 0.987678166 | 1.0235136 | down | mRNA |
| NM_080749 | NEURL2 | 0.784300781 | 0.977347603 | 1.0644 | down | mRNA |
| NM_080750 | DPH3P1 | 0.141504779 | 0.601858131 | 1.16366 | down | mRNA |
| NM_080764 | ZNF280B | 0.817647646 | 0.981727075 | 1.0299804 | down | mRNA |
| NM_080796 | DIDO1 | 0.518734868 | 0.916145597 | 1.0316615 | down | mRNA |
| NM_080797 | DIDO1 | 0.142057935 | 0.60204132 | 1.1255117 | down | mRNA |
| NM_080798 | COL13A1 | 0.216503121 | 0.711074992 | 1.1140464 | down | mRNA |
| NM_080801 | COL13A1 | 0.497437492 | 0.908789022 | 1.1149197 | down | mRNA |
| NM_080805 | COL13A1 | 0.806858252 | 0.98118927 | 1.0438261 | down | mRNA |
| NM_080820 | DTD1 | 0.5824853 | 0.934005566 | 1.0250185 | down | mRNA |
| NM_080821 | FAM210B | 0.150857691 | 0.616029255 | 1.0555641 | down | mRNA |
| NM_080833 | C20orf151 | 0.653807389 | 0.957103596 | 1.068905 | down | mRNA |
| NM_080861 | SPSB3 | 0.633883143 | 0.951174475 | 1.0261409 | down | mRNA |
| NM_080865 | GPR62 | 0.903964569 | 0.99508468 | 1.0054175 | down | mRNA |
| NM_080867 | SOCS4 | 0.03858654 | 0.366748746 | 1.1950769 | down | mRNA |
| NM_080869 | WFDC12 | 0.225196304 | 0.720245224 | 1.9174213 | down | mRNA |
| NM_080881 | DBN1 | 0.88343632 | 0.992766819 | 1.0121143 | down | mRNA |
| NM_080921 | PTPRC | 0.999418335 | 0.999897336 | 1.0001475 | down | mRNA |
| NM_080927 | DCBLD2 | 0.778312637 | 0.976938981 | 1.0345624 | down | mRNA |
| NM_100486 | WAC | 0.020662759 | 0.286034385 | 1.2749035 | down | mRNA |
| NM_130385 | MRVI1 | 0.258895812 | 0.759358563 | 1.0430151 | down | mRNA |
| NM_130388 | ASB12 | 0.228706502 | 0.724873046 | 1.1411054 | down | mRNA |
| NM_130395 | WRNIP1 | 0.014668762 | 0.24380986 | 1.3845046 | down | mRNA |
| NM_130434 | DPP8 | 0.361101035 | 0.841961726 | 1.0919427 | down | mRNA |
| NM_130436 | DYRK1A | 0.113948825 | 0.557835051 | 1.084994 | down | mRNA |
| NM_130438 | DYRK1A | 0.188631771 | 0.674131123 | 1.1461609 | down | mRNA |
| NM_130440 | PTPRF | 0.745036267 | 0.970378122 | 1.1031744 | down | mRNA |
| NM_130443 | DPP3 | 0.026596329 | 0.318484367 | 1.2260264 | down | mRNA |
| NM_130459 | TOR2A | 0.666927438 | 0.958354006 | 1.0391472 | down | mRNA |
| NM_130463 | ATP6V1G2 | 0.4508817 | 0.888823612 | 1.2037038 | down | mRNA |
| NM_130464 | NPIPL3 | 0.679699306 | 0.960007989 | 1.1113277 | down | mRNA |
| NM_130465 | TSPAN17 | 0.723546882 | 0.966191059 | 1.02682 | down | mRNA |
| NM_130472 | MADD | 0.099455109 | 0.523865616 | 1.4000311 | down | mRNA |
| NM_130474 | MADD | 0.411487075 | 0.86887062 | 1.2459169 | down | mRNA |
| NM_130475 | MADD | 0.336662379 | 0.826506012 | 1.2109285 | down | mRNA |
| NM_130476 | MADD | 0.143323902 | 0.603450317 | 1.3353398 | down | mRNA |
| NM_130760 | MADCAM1 | 0.853507052 | 0.987678166 | 1.0413482 | down | mRNA |
| NM_130767 | ACOT12 | 0.08749899 | 0.500993935 | 1.3984979 | down | mRNA |
| NM_130777 | XAGE2 | 0.816264601 | 0.981643774 | 1.0372939 | down | mRNA |
| NM_130781 | RAB24 | 0.200152621 | 0.68900633 | 1.1960544 | down | mRNA |
| NM_130809 | PRRC1 | 0.032435481 | 0.343869895 | 1.1648827 | down | mRNA |
| NM_130839 | UBE3A | 0.30424752 | 0.800038203 | 1.0907815 | down | mRNA |
| NM_130842 | PTPRN2 | 0.035987356 | 0.356229271 | 1.3257667 | down | mRNA |
| NM_130843 | PTPRN2 | 0.052841787 | 0.413038171 | 1.2763673 | down | mRNA |
| NM_130849 | SLC39A4 | 0.897931183 | 0.994326043 | 1.0073935 | down | mRNA |
| NM_130906 | PPIL3 | 0.731787826 | 0.967217713 | 1.0333796 | down | mRNA |
| NM_133173 | APBB3 | 0.477039041 | 0.900429473 | 1.1021713 | down | mRNA |
| NM_133174 | APBB3 | 0.146186403 | 0.608122555 | 1.3205805 | down | mRNA |
| NM_133180 | EPS8L1 | 0.118359131 | 0.565835163 | 1.1789391 | down | mRNA |
| NM_133259 | LRPPRC | 0.138294871 | 0.597621958 | 1.1540092 | down | mRNA |
| NM_133261 | GIPC3 | 0.234208158 | 0.731403918 | 1.1800854 | down | mRNA |
| NM_133264 | WIPF2 | 0.420233861 | 0.872365335 | 1.0584135 | down | mRNA |
| NM_133267 | GSX2 | 0.27875188 | 0.777783404 | 1.3056608 | down | mRNA |
| NM_133269 | FCAR | 0.266354798 | 0.767064488 | 1.6868232 | down | mRNA |
| NM_133271 | FCAR | 0.159193431 | 0.630911751 | 1.1495348 | down | mRNA |
| NM_133272 | FCAR | 0.502624121 | 0.910335149 | 1.0231916 | down | mRNA |
| NM_133273 | FCAR | 0.937557594 | 0.996710991 | 1.0163628 | down | mRNA |
| NM_133274 | FCAR | 0.35393683 | 0.836772515 | 1.1307354 | down | mRNA |
| NM_133278 | FCAR | 0.522013606 | 0.916999248 | 1.1524802 | down | mRNA |
| NM_133325 | PHF10 | 0.12513251 | 0.578441772 | 1.313773 | down | mRNA |
| NM_133328 | DEDD2 | 0.910921314 | 0.995342392 | 1.0145486 | down | mRNA |
| NM_133335 | WHSC1 | 0.569671382 | 0.932573151 | 1.1147733 | down | mRNA |
| NM_133338 | RAD17 | 0.019710587 | 0.282421182 | 1.2776854 | down | mRNA |
| NM_133339 | RAD17 | 0.030217794 | 0.33720537 | 1.3913759 | down | mRNA |
| NM_133340 | RAD17 | 0.103622272 | 0.533244253 | 1.2475209 | down | mRNA |
| NM_133368 | RSPRY1 | 0.16068988 | 0.632065515 | 1.3170955 | down | mRNA |
| NM_133370 | YTHDC1 | 0.038066064 | 0.364387577 | 1.2226643 | down | mRNA |
| NM_133372 | FNIP1 | 0.238715279 | 0.736777065 | 1.1321742 | down | mRNA |
| NM_133375 | DIS3L | 0.61403843 | 0.944827645 | 1.105053 | down | mRNA |
| NM_133376 | ITGB1 | 0.727577708 | 0.966191059 | 1.0152028 | down | mRNA |
| NM_133378 | TTN | 0.520148152 | 0.916394575 | 1.0578342 | down | mRNA |
| NM_133433 | NIPBL | 0.059008252 | 0.431877527 | 1.2825735 | down | mRNA |
| NM_133446 | AGAP4 | 0.819409626 | 0.982224663 | 1.0256565 | down | mRNA |
| NM_133448 | TMEM132D | 0.975161457 | 0.998501532 | 1.021236 | down | mRNA |
| NM_133450 | ANKS3 | 0.057149324 | 0.425938435 | 1.2557318 | down | mRNA |
| NM_133452 | RAVER1 | 0.611673786 | 0.943913476 | 1.0692011 | down | mRNA |
| NM_133459 | CCBE1 | 0.736608968 | 0.968717225 | 1.059575 | down | mRNA |
| NM_133462 | TTC14 | 0.217686652 | 0.711805244 | 1.0829384 | down | mRNA |
| NM_133466 | ZFP82 | 0.014813647 | 0.244234794 | 1.388677 | down | mRNA |
| NM_133474 | ZNF721 | 0.004786497 | 0.158085585 | 1.3371457 | down | mRNA |
| NM_133476 | ZNF384 | 0.161796697 | 0.633721912 | 1.1423559 | down | mRNA |
| NM_133489 | SLC26A10 | 0.101915277 | 0.52995944 | 1.2382815 | down | mRNA |
| NM_133491 | SAT2 | 0.374596324 | 0.84936907 | 1.1875451 | down | mRNA |
| NM_133493 | CD109 | 0.676921863 | 0.959531882 | 1.0560878 | down | mRNA |
| NM_133494 | NEK7 | 0.164883116 | 0.638119216 | 1.1245143 | down | mRNA |
| NM_133499 | SYN1 | 0.576809101 | 0.933656777 | 1.1222777 | down | mRNA |
| NM_133502 | ZNF274 | 0.27333339 | 0.773331313 | 1.4556977 | down | mRNA |
| NM_133503 | DCN | 0.933413251 | 0.996710991 | 1.0289881 | down | mRNA |
| NM_133505 | DCN | 0.847567198 | 0.987678166 | 1.0597159 | down | mRNA |
| NM_133506 | DCN | 0.897924924 | 0.994326043 | 1.0374833 | down | mRNA |
| NM_133625 | SYN2 | 0.375622836 | 0.849862269 | 1.1643903 | down | mRNA |
| NM_133629 | RAD51D | 0.009659442 | 0.205650016 | 1.1873533 | down | mRNA |
| NM_133645 | MTO1 | 0.049441394 | 0.402500646 | 1.263401 | down | mRNA |
| NM_133646 | ZAK | 0.964611107 | 0.99793927 | 1.0055205 | down | mRNA |
| NM_134263 | SLC26A6 | 0.573761292 | 0.933656777 | 1.0998575 | down | mRNA |
| NM_134265 | WSB1 | 0.984847758 | 0.998971807 | 1.0036433 | down | mRNA |
| NM_134269 | SMTN | 0.003873119 | 0.143701193 | 1.2499632 | down | mRNA |
| NM_134270 | SMTN | 0.014372026 | 0.24189738 | 1.2404945 | down | mRNA |
| NM_134323 | TARBP2 | 0.087662857 | 0.501313828 | 1.2309331 | down | mRNA |
| NM_134428 | RFX3 | 0.358879403 | 0.840767187 | 1.2489781 | down | mRNA |
| NM_134442 | CREB1 | 0.148196606 | 0.611101359 | 1.1029783 | down | mRNA |
| NM_134470 | IL1RAP | 0.250572987 | 0.749980154 | 1.1788861 | down | mRNA |
| NM_138270 | ATRX | 0.772461564 | 0.975448017 | 1.0328788 | down | mRNA |
| NM_138273 | C6orf25 | 0.627983038 | 0.949657949 | 1.0398802 | down | mRNA |
| NM_138275 | C6orf25 | 0.692730073 | 0.961584652 | 1.1121116 | down | mRNA |
| NM_138278 | BNIPL | 0.259348325 | 0.759808377 | 1.5613901 | down | mRNA |
| NM_138282 | ATP6V1G2 | 0.490728597 | 0.905398715 | 1.2860511 | down | mRNA |
| NM_138297 | MUC4 | 0.514018016 | 0.91350662 | 1.0308409 | down | mRNA |
| NM_138300 | PYGO2 | 0.402849655 | 0.864786348 | 1.2970688 | down | mRNA |
| NM_138316 | PANK1 | 0.775686033 | 0.97589756 | 1.034015 | down | mRNA |
| NM_138320 | PCSK6 | 0.375200366 | 0.849710156 | 1.242819 | down | mRNA |
| NM_138322 | PCSK6 | 0.133294713 | 0.589162633 | 1.1715489 | down | mRNA |
| NM_138334 | JOSD2 | 0.379142636 | 0.852705238 | 1.063428 | down | mRNA |
| NM_138338 | POLR3H | 0.20336324 | 0.692876095 | 1.307499 | down | mRNA |
| NM_138341 | TMEM116 | 0.816093636 | 0.981533026 | 1.0385422 | down | mRNA |
| NM_138345 | VWA5B2 | 0.667770049 | 0.958354006 | 1.0794255 | down | mRNA |
| NM_138346 | KIAA2013 | 0.57650189 | 0.933656777 | 1.0452147 | down | mRNA |
| NM_138347 | ZNF551 | 0.151096233 | 0.616500055 | 1.159734 | down | mRNA |
| NM_138348 | FAM105B | 0.097107377 | 0.519544195 | 1.232764 | down | mRNA |
| NM_138349 | TP53I13 | 0.010861903 | 0.213674567 | 1.0954278 | down | mRNA |
| NM_138352 | SAMD1 | 0.932389292 | 0.996710991 | 1.0121569 | down | mRNA |
| NM_138353 | DCAF15 | 0.382819896 | 0.853514109 | 1.1178161 | down | mRNA |
| NM_138355 | SCRN2 | 0.717898259 | 0.966191059 | 1.0685496 | down | mRNA |
| NM_138358 | C19orf52 | 0.560428252 | 0.92782421 | 1.2132579 | down | mRNA |
| NM_138363 | CEP95 | 0.784762636 | 0.977411899 | 1.0489196 | down | mRNA |
| NM_138364 | PRMT10 | 0.031014088 | 0.340185047 | 1.5323245 | down | mRNA |
| NM_138368 | AP5B1 | 0.617541596 | 0.94659436 | 1.0194489 | down | mRNA |
| NM_138369 | BOD1 | 0.464571531 | 0.894989844 | 1.1147231 | down | mRNA |
| NM_138370 | PKDCC | 0.432529036 | 0.880491224 | 1.3332121 | down | mRNA |
| NM_138376 | TTC5 | 0.184090043 | 0.667662996 | 1.3176497 | down | mRNA |
| NM_138381 | OXNAD1 | 0.985247593 | 0.998971807 | 1.0019267 | down | mRNA |
| NM_138382 | RIPPLY1 | 0.553588079 | 0.926337111 | 1.0960926 | down | mRNA |
| NM_138383 | MTSS1L | 0.803678645 | 0.98118927 | 1.0120075 | down | mRNA |
| NM_138386 | NAF1 | 0.924505996 | 0.995456159 | 1.0085475 | down | mRNA |
| NM_138391 | TMEM183A | 0.421868222 | 0.873398499 | 1.0704786 | down | mRNA |
| NM_138393 | REEP6 | 0.882851157 | 0.992766819 | 1.0264044 | down | mRNA |
| NM_138395 | MARS2 | 0.793817792 | 0.97921643 | 1.055139 | down | mRNA |
| NM_138396 | MARCH9 | 0.689750499 | 0.961105466 | 1.1021615 | down | mRNA |
| NM_138399 | TMEM44 | 0.180879707 | 0.663954902 | 1.2320484 | down | mRNA |
| NM_138401 | FAM125A | 0.73588093 | 0.968409778 | 1.0197261 | down | mRNA |
| NM_138402 | SP140L | 0.398793377 | 0.863643939 | 1.11774 | down | mRNA |
| NM_138408 | GTF3C6 | 0.537937702 | 0.923138873 | 1.0917015 | down | mRNA |
| NM_138412 | RDH13 | 0.033068754 | 0.346127643 | 1.4772709 | down | mRNA |
| NM_138414 | CCDC101 | 0.16995258 | 0.646789844 | 1.1871007 | down | mRNA |
| NM_138415 | PHF21B | 0.222183498 | 0.717189116 | 1.0922413 | down | mRNA |
| NM_138417 | KTI12 | 0.012841599 | 0.231057862 | 1.4013856 | down | mRNA |
| NM_138418 | FAM195A | 0.323538323 | 0.816213518 | 1.1323716 | down | mRNA |
| NM_138419 | FAM54A | 0.619819316 | 0.947444797 | 1.4529644 | down | mRNA |
| NM_138421 | SAAL1 | 0.593870528 | 0.938299318 | 1.0454132 | down | mRNA |
| NM_138422 | ADAT3 | 0.748853914 | 0.970725371 | 1.0548284 | down | mRNA |
| NM_138431 | MFSD3 | 0.732903918 | 0.967703318 | 1.0285603 | down | mRNA |
| NM_138432 | SDSL | 0.52835531 | 0.918883792 | 1.0545739 | down | mRNA |
| NM_138443 | HAUS1 | 0.453723973 | 0.890025645 | 1.1971646 | down | mRNA |
| NM_138444 | KCTD12 | 0.359974239 | 0.841457372 | 1.1995345 | down | mRNA |
| NM_138446 | MALSU1 | 0.619938629 | 0.947444797 | 1.0226825 | down | mRNA |
| NM_138459 | NUS1 | 0.115837045 | 0.561697782 | 1.1788241 | down | mRNA |
| NM_138462 | ZMYND19 | 0.002699657 | 0.122795128 | 1.4721456 | down | mRNA |
| NM_138464 | C5orf55 | 0.066308379 | 0.449693785 | 1.2090524 | down | mRNA |
| NM_138467 | TYW3 | 0.284891097 | 0.781963593 | 1.0920375 | down | mRNA |
| NM_138473 | SP1 | 0.000330533 | 0.056781055 | 1.1865888 | down | mRNA |
| NM_138476 | MDP1 | 0.6067761 | 0.942291918 | 1.0391049 | down | mRNA |
| NM_138477 | CDAN1 | 0.091622983 | 0.510719066 | 1.4258854 | down | mRNA |
| NM_138481 | CHADL | 0.904065589 | 0.99508468 | 1.0267385 | down | mRNA |
| NM_138484 | SGOL1 | 0.729885065 | 0.966191059 | 1.1738812 | down | mRNA |
| NM_138495 | ATXN7L1 | 0.795849346 | 0.980144703 | 1.0251042 | down | mRNA |
| NM_138496 | CYHR1 | 0.551582824 | 0.926152612 | 1.1016851 | down | mRNA |
| NM_138499 | PWWP2B | 0.025810908 | 0.315177872 | 2.1401225 | down | mRNA |
| NM_138501 | TECR | 0.771330291 | 0.975347132 | 1.0215704 | down | mRNA |
| NM_138555 | KIF23 | 0.445038672 | 0.885721054 | 1.3610085 | down | mRNA |
| NM_138558 | PPP1R8 | 0.041508974 | 0.377610942 | 1.4702774 | down | mRNA |
| NM_138565 | CTTN | 0.209641704 | 0.700367256 | 1.0669534 | down | mRNA |
| NM_138570 | SLC38A10 | 0.987956967 | 0.999140676 | 1.0012067 | down | mRNA |
| NM_138571 | HINT3 | 0.679930408 | 0.960023963 | 1.0247836 | down | mRNA |
| NM_138575 | PGAM5 | 0.329693962 | 0.821768501 | 1.5291225 | down | mRNA |
| NM_138578 | BCL2L1 | 0.626605563 | 0.949203235 | 1.0585557 | down | mRNA |
| NM_138609 | H2AFY | 0.273058344 | 0.772770327 | 1.1399597 | down | mRNA |
| NM_138610 | H2AFY | 0.507864411 | 0.911674633 | 1.1424674 | down | mRNA |
| NM_138617 | RHCE | 0.458434416 | 0.892667725 | 1.2681583 | down | mRNA |
| NM_138632 | TRIOBP | 0.858816667 | 0.988512246 | 1.0292121 | down | mRNA |
| NM_138633 | AKAP7 | 0.427334906 | 0.876836544 | 1.0632856 | down | mRNA |
| NM_138638 | CFL2 | 0.298520494 | 0.795148977 | 1.3895762 | down | mRNA |
| NM_138639 | BCL2L12 | 0.173900563 | 0.652129479 | 1.5128073 | down | mRNA |
| NM_138643 | CABYR | 0.970697914 | 0.998277062 | 1.005935 | down | mRNA |
| NM_138689 | PPP1R14B | 0.425878518 | 0.875527 | 1.0714581 | down | mRNA |
| NM_138701 | MPLKIP | 0.200002872 | 0.68900633 | 1.0633825 | down | mRNA |
| NM_138704 | NDNL2 | 0.035438644 | 0.353725338 | 1.3913069 | down | mRNA |
| NM_138706 | B3GNT6 | 0.649298044 | 0.95550188 | 1.0542832 | down | mRNA |
| NM_138713 | NFAT5 | 0.985246855 | 0.998971807 | 1.0022264 | down | mRNA |
| NM_138714 | NFAT5 | 0.866480173 | 0.989481868 | 1.0197445 | down | mRNA |
| NM_138727 | ST7L | 0.444475319 | 0.885206405 | 1.0852335 | down | mRNA |
| NM_138728 | ST7L | 0.044178233 | 0.386336269 | 1.1959483 | down | mRNA |
| NM_138729 | ST7L | 0.775853942 | 0.975973665 | 1.0278589 | down | mRNA |
| NM_138730 | HMGN3 | 0.828932663 | 0.98552522 | 1.0084081 | down | mRNA |
| NM_138731 | MIPOL1 | 0.270166425 | 0.770135843 | 1.1166116 | down | mRNA |
| NM_138740 | C1orf43 | 0.221348419 | 0.716734257 | 1.1573026 | down | mRNA |
| NM_138769 | RHOT2 | 0.422470994 | 0.873896892 | 1.1265312 | down | mRNA |
| NM_138773 | SLC25A46 | 0.056895258 | 0.424979572 | 1.1608725 | down | mRNA |
| NM_138775 | ALKBH8 | 0.401833328 | 0.864786348 | 1.2022678 | down | mRNA |
| NM_138779 | TEX30 | 0.962546952 | 0.99793927 | 1.0047614 | down | mRNA |
| NM_138783 | ZNF653 | 0.168686616 | 0.644327097 | 1.0984372 | down | mRNA |
| NM_138785 | GINM1 | 0.576041223 | 0.933656777 | 1.0369457 | down | mRNA |
| NM_138789 | PIH1D2 | 0.827750101 | 0.985006148 | 1.0369151 | down | mRNA |
| NM_138792 | LEO1 | 0.037062983 | 0.359628653 | 1.1495415 | down | mRNA |
| NM_138794 | LYPLAL1 | 0.989681598 | 0.999140676 | 1.0011491 | down | mRNA |
| NM_138795 | ARL8A | 0.007673842 | 0.193257016 | 1.1242921 | down | mRNA |
| NM_138796 | SPATA17 | 0.971324126 | 0.998501532 | 1.0077245 | down | mRNA |
| NM_138797 | ANKRD54 | 0.497383872 | 0.908789022 | 1.0220114 | down | mRNA |
| NM_138798 | MITD1 | 0.143115902 | 0.603147721 | 1.0828492 | down | mRNA |
| NM_138799 | MBOAT2 | 0.353731711 | 0.836545774 | 1.1357225 | down | mRNA |
| NM_138800 | TRIM43 | 0.639384327 | 0.953475593 | 1.0986554 | down | mRNA |
| NM_138802 | ZFAND2B | 0.103490149 | 0.533123263 | 1.0954786 | down | mRNA |
| NM_138807 | TAMM41 | 0.048780136 | 0.401050434 | 1.2544952 | down | mRNA |
| NM_138822 | PAM | 0.905257316 | 0.99508468 | 1.014026 | down | mRNA |
| NM_138924 | GAMT | 0.80163207 | 0.98118927 | 1.0204074 | down | mRNA |
| NM_138927 | SON | 0.399107131 | 0.863643939 | 1.118925 | down | mRNA |
| NM_138932 | A1CF | 0.069857907 | 0.457827646 | 1.2761037 | down | mRNA |
| NM_138957 | MAPK1 | 0.462776245 | 0.894989844 | 1.0870787 | down | mRNA |
| NM_138962 | MSI2 | 0.573926244 | 0.933656777 | 1.12385 | down | mRNA |
| NM_138970 | NRXN3 | 0.156286662 | 0.624655082 | 1.6752199 | down | mRNA |
| NM_138983 | OLIG1 | 0.652839023 | 0.956714149 | 1.0994081 | down | mRNA |
| NM_139003 | HFE | 0.639251971 | 0.953368377 | 1.0991616 | down | mRNA |
| NM_139004 | HFE | 0.053440413 | 0.415824909 | 1.1441802 | down | mRNA |
| NM_139006 | HFE | 0.605777758 | 0.94174007 | 1.0621141 | down | mRNA |
| NM_139007 | HFE | 0.833215733 | 0.986434891 | 1.0158682 | down | mRNA |
| NM_139008 | HFE | 0.235164781 | 0.73249698 | 1.2771644 | down | mRNA |
| NM_139012 | MAPK14 | 0.56652748 | 0.931095609 | 1.0559483 | down | mRNA |
| NM_139014 | MAPK14 | 0.013111122 | 0.234043321 | 1.2705764 | down | mRNA |
| NM_139015 | SPPL3 | 0.239717507 | 0.737916177 | 1.1900201 | down | mRNA |
| NM_139018 | CD300LF | 0.67895733 | 0.959969193 | 1.1382604 | down | mRNA |
| NM_139021 | MAPK15 | 0.653941649 | 0.957205183 | 1.1261242 | down | mRNA |
| NM_139022 | TSPAN32 | 0.734518985 | 0.967777568 | 1.027958 | down | mRNA |
| NM_139027 | ADAMTS13 | 0.689396021 | 0.96108227 | 1.1053455 | down | mRNA |
| NM_139032 | MAPK7 | 0.032674683 | 0.344901399 | 1.2185064 | down | mRNA |
| NM_139033 | MAPK7 | 0.392196086 | 0.859493185 | 1.4708727 | down | mRNA |
| NM_139035 | SMARCA1 | 0.987931332 | 0.999140676 | 1.0055219 | down | mRNA |
| NM_139045 | SMARCA2 | 0.301088488 | 0.79596036 | 1.0971159 | down | mRNA |
| NM_139046 | MAPK8 | 0.205028658 | 0.694889593 | 1.0989261 | down | mRNA |
| NM_139047 | MAPK8 | 0.998589277 | 0.999889342 | 1.000244 | down | mRNA |
| NM_139049 | MAPK8 | 0.047695425 | 0.397835916 | 1.2498189 | down | mRNA |
| NM_139057 | ADAMTS17 | 0.765961743 | 0.974734429 | 1.151065 | down | mRNA |
| NM_139058 | ARX | 0.392849868 | 0.859694706 | 1.0552037 | down | mRNA |
| NM_139062 | CSNK1D | 0.191232633 | 0.676939305 | 1.0806208 | down | mRNA |
| NM_139068 | MAPK9 | 0.127971976 | 0.581241479 | 1.1126938 | down | mRNA |
| NM_139070 | MAPK9 | 0.079290768 | 0.482639336 | 1.2972317 | down | mRNA |
| NM_139071 | SMARCD1 | 0.682213855 | 0.960111041 | 1.0707796 | down | mRNA |
| NM_139072 | DNER | 0.783812787 | 0.9773385 | 1.1109738 | down | mRNA |
| NM_139075 | TPCN2 | 0.429740254 | 0.878226842 | 1.0670878 | down | mRNA |
| NM_139078 | MAPKAPK5 | 0.264226249 | 0.764950604 | 1.2186727 | down | mRNA |
| NM_139118 | YY1AP1 | 0.914220241 | 0.995342392 | 1.0142221 | down | mRNA |
| NM_139125 | MASP1 | 0.940687198 | 0.996710991 | 1.0280521 | down | mRNA |
| NM_139126 | PPIL4 | 0.149978445 | 0.614295311 | 1.1625498 | down | mRNA |
| NM_139132 | NUP98 | 0.140666191 | 0.600786526 | 1.1380678 | down | mRNA |
| NM_139156 | AMPD2 | 0.666067271 | 0.958354006 | 1.0363868 | down | mRNA |
| NM_139157 | ST5 | 0.699763273 | 0.962977276 | 1.0941412 | down | mRNA |
| NM_139159 | DPP9 | 0.972418839 | 0.998501532 | 1.0058443 | down | mRNA |
| NM_139161 | CRB3 | 0.78506615 | 0.977411899 | 1.0262868 | down | mRNA |
| NM_139164 | STARD4 | 0.758001825 | 0.972072638 | 1.1786719 | down | mRNA |
| NM_139168 | SREK1 | 0.096722768 | 0.518746328 | 1.219523 | down | mRNA |
| NM_139169 | TRUB1 | 0.880033321 | 0.992197209 | 1.019923 | down | mRNA |
| NM_139170 | C16orf71 | 0.64578014 | 0.955256588 | 1.1022148 | down | mRNA |
| NM_139174 | ADAD2 | 0.939935057 | 0.996710991 | 1.01427 | down | mRNA |
| NM_139177 | SLC39A11 | 0.595034691 | 0.938655001 | 1.0283192 | down | mRNA |
| NM_139178 | ALKBH3 | 0.404523125 | 0.865855793 | 1.0420841 | down | mRNA |
| NM_139179 | DAGLB | 0.041986193 | 0.378937196 | 1.1812009 | down | mRNA |
| NM_139201 | GIT2 | 0.022535323 | 0.295888941 | 1.3230164 | down | mRNA |
| NM_139209 | GRK7 | 0.384210371 | 0.854819314 | 1.2813339 | down | mRNA |
| NM_139235 | NOL6 | 0.257055687 | 0.757669958 | 1.2524235 | down | mRNA |
| NM_139241 | FGD4 | 0.882101337 | 0.992766819 | 1.0561397 | down | mRNA |
| NM_139242 | MTFMT | 0.302517668 | 0.79797107 | 1.1552119 | down | mRNA |
| NM_139244 | STXBP5 | 0.322450809 | 0.815372756 | 1.249398 | down | mRNA |
| NM_139250 | CTAG1A | 0.739725012 | 0.969564098 | 1.0793577 | down | mRNA |
| NM_139265 | EHD4 | 0.036239365 | 0.35703653 | 1.3774359 | down | mRNA |
| NM_139266 | STAT1 | 0.861868663 | 0.989401142 | 1.074171 | down | mRNA |
| NM_139273 | CARS | 0.674208779 | 0.959531882 | 1.0318558 | down | mRNA |
| NM_139280 | ORMDL3 | 0.255582374 | 0.756337058 | 1.3950347 | down | mRNA |
| NM_139281 | WDR36 | 0.480471711 | 0.901538317 | 1.0676307 | down | mRNA |
| NM_139283 | PPTC7 | 0.544367465 | 0.923577434 | 1.0674423 | down | mRNA |
| NM_139286 | CDC26 | 0.122383085 | 0.57455499 | 1.1242371 | down | mRNA |
| NM_139317 | BIRC7 | 0.207482016 | 0.698556218 | 1.1067471 | down | mRNA |
| NM_139321 | ATRN | 0.700832943 | 0.962991468 | 1.0546376 | down | mRNA |
| NM_139323 | YWHAB | 0.534561133 | 0.920848299 | 1.0611467 | down | mRNA |
| NM_139344 | BIN1 | 0.509734273 | 0.911674633 | 1.3513863 | down | mRNA |
| NM_139346 | BIN1 | 0.350188097 | 0.834344847 | 1.1032024 | down | mRNA |
| NM_139347 | BIN1 | 0.813844356 | 0.98120545 | 1.0282185 | down | mRNA |
| NM_139348 | BIN1 | 0.944509213 | 0.996710991 | 1.0073139 | down | mRNA |
| NM_139350 | BIN1 | 0.918053324 | 0.995349793 | 1.0247149 | down | mRNA |
| NM_139351 | BIN1 | 0.125629334 | 0.578441772 | 1.0884411 | down | mRNA |
| NM_139352 | TAF1A | 0.020446937 | 0.285678379 | 1.3390674 | down | mRNA |
| NM_139353 | TAF1C | 0.624223106 | 0.948341211 | 1.0602364 | down | mRNA |
| NM_139355 | MATK | 0.604179195 | 0.94174007 | 1.0708121 | down | mRNA |
| NM_144489 | RGS3 | 0.9284599 | 0.996066203 | 1.0145582 | down | mRNA |
| NM_144498 | OSBPL2 | 0.954199216 | 0.996710991 | 1.0051243 | down | mRNA |
| NM_144563 | RPIA | 0.189951262 | 0.675909261 | 1.1249799 | down | mRNA |
| NM_144564 | SLC39A3 | 0.564450972 | 0.929730843 | 1.0617235 | down | mRNA |
| NM_144569 | SPOCD1 | 0.255969834 | 0.757000534 | 1.1178896 | down | mRNA |
| NM_144573 | NEXN | 0.017867027 | 0.268891484 | 1.2779945 | down | mRNA |
| NM_144574 | WDR20 | 0.014638463 | 0.24380986 | 1.1081425 | down | mRNA |
| NM_144578 | MAPK1IP1L | 0.404280606 | 0.865617032 | 1.0410836 | down | mRNA |
| NM_144580 | C1orf85 | 0.237682053 | 0.735771951 | 1.1822229 | down | mRNA |
| NM_144581 | C14orf149 | 0.165242855 | 0.638608196 | 1.1569416 | down | mRNA |
| NM_144586 | LYPD1 | 0.886292394 | 0.992981836 | 1.0264014 | down | mRNA |
| NM_144588 | ZFYVE27 | 0.262257366 | 0.763125731 | 1.0899035 | down | mRNA |
| NM_144589 | COMTD1 | 0.084336944 | 0.494303472 | 1.2859101 | down | mRNA |
| NM_144596 | TTC8 | 0.372451665 | 0.84763089 | 1.1430301 | down | mRNA |
| NM_144600 | FOPNL | 0.067030655 | 0.451412357 | 1.2675757 | down | mRNA |
| NM_144611 | CYB5D2 | 0.081191741 | 0.486780649 | 1.2037385 | down | mRNA |
| NM_144613 | COX6B2 | 0.792198949 | 0.978996327 | 1.0875099 | down | mRNA |
| NM_144614 | MBD3L2 | 0.686278693 | 0.960205549 | 1.0280153 | down | mRNA |
| NM_144617 | HSPB6 | 0.875567029 | 0.991094934 | 1.0764866 | down | mRNA |
| NM_144618 | GABPB2 | 0.738026759 | 0.969207218 | 1.0258771 | down | mRNA |
| NM_144620 | LRRC39 | 0.314414966 | 0.809707041 | 1.125952 | down | mRNA |
| NM_144628 | TBC1D20 | 0.246999869 | 0.745152522 | 1.0840261 | down | mRNA |
| NM_144631 | ZNF513 | 0.427261172 | 0.876836544 | 1.080815 | down | mRNA |
| NM_144638 | TMEM42 | 0.6624358 | 0.958354006 | 1.0639422 | down | mRNA |
| NM_144643 | SCLT1 | 0.288165763 | 0.784355942 | 1.0980941 | down | mRNA |
| NM_144645 | C4orf36 | 0.886509065 | 0.992981836 | 1.051303 | down | mRNA |
| NM_144648 | LRGUK | 0.397118467 | 0.862344286 | 1.1724604 | down | mRNA |
| NM_144653 | NACC2 | 0.75634439 | 0.971691782 | 1.0299641 | down | mRNA |
| NM_144654 | C9orf116 | 0.727177494 | 0.966191059 | 1.0699663 | down | mRNA |
| NM_144658 | DOCK11 | 0.239472219 | 0.73766795 | 1.1368767 | down | mRNA |
| NM_144664 | FAM76B | 0.096620856 | 0.518665334 | 1.3467619 | down | mRNA |
| NM_144665 | SESN3 | 0.887571238 | 0.993263732 | 1.0170262 | down | mRNA |
| NM_144668 | WDR66 | 0.575621624 | 0.933656777 | 1.2035002 | down | mRNA |
| NM_144675 | GSG1L | 0.576779024 | 0.933656777 | 1.1896381 | down | mRNA |
| NM_144677 | MGAT5B | 0.245254203 | 0.743589595 | 1.1024197 | down | mRNA |
| NM_144679 | ENTHD2 | 0.687059066 | 0.960205549 | 1.0758947 | down | mRNA |
| NM_144684 | ZNF480 | 0.625110199 | 0.948700498 | 1.0408062 | down | mRNA |
| NM_144686 | TMC4 | 0.777725541 | 0.976938981 | 1.112611 | down | mRNA |
| NM_144689 | ZNF420 | 0.063220629 | 0.440210745 | 1.3197259 | down | mRNA |
| NM_144697 | C1orf51 | 0.785160406 | 0.977411899 | 1.0311495 | down | mRNA |
| NM_144705 | TEKT4 | 0.676395281 | 0.959531882 | 1.1959791 | down | mRNA |
| NM_144709 | PUS10 | 0.746288275 | 0.970394407 | 1.0646091 | down | mRNA |
| NM_144711 | KLHL23 | 0.757727495 | 0.972072638 | 1.0766168 | down | mRNA |
| NM_144712 | SLC23A3 | 0.40845777 | 0.867591329 | 1.0437533 | down | mRNA |
| NM_144716 | CCDC12 | 0.05180351 | 0.41031217 | 1.2480582 | down | mRNA |
| NM_144718 | SPICE1 | 0.236310307 | 0.734472382 | 1.11325 | down | mRNA |
| NM_144721 | THAP6 | 0.104903505 | 0.535979888 | 1.1040716 | down | mRNA |
| NM_144722 | SPEF2 | 0.875758761 | 0.991145401 | 1.0469484 | down | mRNA |
| NM_144725 | TTC23L | 0.518828728 | 0.916145597 | 1.1331898 | down | mRNA |
| NM_144729 | DUSP10 | 0.984733495 | 0.998971807 | 1.0093692 | down | mRNA |
| NM_144736 | C2orf56 | 0.089391311 | 0.505564434 | 1.1197107 | down | mRNA |
| NM_144775 | SMCR8 | 0.986761011 | 0.99909491 | 1.0024097 | down | mRNA |
| NM_144781 | PDCD2 | 0.29104142 | 0.787419048 | 1.1176919 | down | mRNA |
| NM_144957 | PRSS21 | 0.204896872 | 0.694868044 | 1.30103 | down | mRNA |
| NM_144963 | FAM91A1 | 0.285032451 | 0.781963593 | 1.188198 | down | mRNA |
| NM_144964 | TRMT10B | 0.074381877 | 0.469723636 | 1.383707 | down | mRNA |
| NM_144969 | ZDHHC15 | 0.808006325 | 0.98118927 | 1.0927264 | down | mRNA |
| NM_144970 | CXorf38 | 0.76012452 | 0.972883253 | 1.0331815 | down | mRNA |
| NM_144973 | DENND5B | 0.006029649 | 0.176785634 | 1.623287 | down | mRNA |
| NM_144976 | ZNF564 | 0.003843393 | 0.143383256 | 1.8117466 | down | mRNA |
| NM_144978 | CCDC138 | 0.459547571 | 0.892920604 | 1.204255 | down | mRNA |
| NM_144980 | C6orf118 | 0.416260911 | 0.870736736 | 1.2033248 | down | mRNA |
| NM_144987 | U2AF1L4 | 0.620880294 | 0.947926299 | 1.0356448 | down | mRNA |
| NM_144988 | ALG14 | 0.374267278 | 0.849321997 | 1.0734825 | down | mRNA |
| NM_144992 | VWA3B | 0.34305236 | 0.831521302 | 1.0905188 | down | mRNA |
| NM_144996 | ARL13B | 0.6055445 | 0.94174007 | 1.0431076 | down | mRNA |
| NM_144999 | LRRC45 | 0.9706729 | 0.998277062 | 1.0035914 | down | mRNA |
| NM_145004 | ADAM32 | 0.427928572 | 0.876992614 | 1.1832605 | down | mRNA |
| NM_145011 | ZNF25 | 0.032241334 | 0.343117475 | 1.2979987 | down | mRNA |
| NM_145012 | CCNY | 0.486738207 | 0.903524001 | 1.0224651 | down | mRNA |
| NM_145014 | HYLS1 | 0.679983732 | 0.960032584 | 1.1617179 | down | mRNA |
| NM_145017 | PPP1R32 | 0.773385166 | 0.97589756 | 1.0452904 | down | mRNA |
| NM_145018 | C11orf82 | 0.771169659 | 0.975347132 | 1.2142009 | down | mRNA |
| NM_145025 | AKD1 | 0.708685418 | 0.965462762 | 1.1461904 | down | mRNA |
| NM_145026 | SPATS1 | 0.541419339 | 0.923577434 | 1.1610531 | down | mRNA |
| NM_145030 | PPP1R35 | 0.529059019 | 0.91899174 | 1.0946012 | down | mRNA |
| NM_145034 | TOR1AIP2 | 0.217225423 | 0.711339274 | 1.0921425 | down | mRNA |
| NM_145039 | CENPBD1 | 0.148407548 | 0.611309209 | 1.2455879 | down | mRNA |
| NM_145040 | PRKCDBP | 0.766465401 | 0.974838893 | 1.0530302 | down | mRNA |
| NM_145044 | ZNF501 | 0.00211824 | 0.110028846 | 1.5334399 | down | mRNA |
| NM_145048 | PACRGL | 0.210912841 | 0.701961023 | 1.1464626 | down | mRNA |
| NM_145049 | UBLCP1 | 0.325244213 | 0.817614409 | 1.1159868 | down | mRNA |
| NM_145052 | UPRT | 0.095374052 | 0.516597924 | 1.268428 | down | mRNA |
| NM_145057 | CDC42EP5 | 0.88589595 | 0.992981836 | 1.0267998 | down | mRNA |
| NM_145059 | FUK | 0.574430872 | 0.933656777 | 1.1087635 | down | mRNA |
| NM_145062 | ZUFSP | 0.063804179 | 0.441397267 | 1.2217739 | down | mRNA |
| NM_145063 | C6orf130 | 0.383520915 | 0.853881747 | 1.128005 | down | mRNA |
| NM_145071 | CISH | 0.516914243 | 0.91493866 | 1.163053 | down | mRNA |
| NM_145074 | HTRA2 | 0.115758353 | 0.561584069 | 1.2766002 | down | mRNA |
| NM_145080 | NSMCE1 | 0.326967674 | 0.81991943 | 1.0299384 | down | mRNA |
| NM_145109 | MAP2K3 | 0.82467113 | 0.983779567 | 1.0432407 | down | mRNA |
| NM_145111 | FAM200A | 0.00617413 | 0.178714838 | 1.4271525 | down | mRNA |
| NM_145112 | MAX | 0.021048987 | 0.288193947 | 1.1572004 | down | mRNA |
| NM_145113 | MAX | 0.986039159 | 0.998971807 | 1.0014255 | down | mRNA |
| NM_145116 | MAX | 0.00553175 | 0.169833068 | 1.1561848 | down | mRNA |
| NM_145160 | MAP2K5 | 0.078645579 | 0.480619753 | 1.2845581 | down | mRNA |
| NM_145165 | CHURC1 | 0.673388983 | 0.959531882 | 1.0518566 | down | mRNA |
| NM_145169 | SFT2D1 | 0.260094618 | 0.760388386 | 1.075242 | down | mRNA |
| NM_145171 | GPHB5 | 0.773464743 | 0.97589756 | 1.0429487 | down | mRNA |
| NM_145173 | DIRAS1 | 0.009366491 | 0.204533521 | 1.3890376 | down | mRNA |
| NM_145176 | SLC2A12 | 0.54232912 | 0.923577434 | 1.0884248 | down | mRNA |
| NM_145182 | PYCARD | 0.4310733 | 0.879624883 | 1.0713974 | down | mRNA |
| NM_145201 | NAPRT1 | 0.156457415 | 0.625091798 | 1.0406777 | down | mRNA |
| NM_145203 | CSNK1A1L | 0.208395597 | 0.699191299 | 1.1067489 | down | mRNA |
| NM_145206 | VTI1A | 0.893082471 | 0.993978977 | 1.0213906 | down | mRNA |
| NM_145207 | SPATA5 | 0.743635494 | 0.970330526 | 1.062001 | down | mRNA |
| NM_145212 | MRPL30 | 0.800719105 | 0.98118927 | 1.0336814 | down | mRNA |
| NM_145233 | ZNF625 | 0.281011228 | 0.779126326 | 1.2974412 | down | mRNA |
| NM_145236 | B3GNT7 | 0.966537176 | 0.99793927 | 1.0075818 | down | mRNA |
| NM_145243 | OMA1 | 0.453773101 | 0.890036146 | 1.1075911 | down | mRNA |
| NM_145252 | ZG16B | 0.918849914 | 0.995349793 | 1.0087376 | down | mRNA |
| NM_145253 | FAM100A | 0.227178636 | 0.722847579 | 1.0977797 | down | mRNA |
| NM_145254 | TMEM170A | 0.021303034 | 0.288424269 | 1.1538661 | down | mRNA |
| NM_145255 | MRPL10 | 0.009014369 | 0.202329498 | 1.2273513 | down | mRNA |
| NM_145256 | LRRC25 | 0.837031713 | 0.986775289 | 1.0234061 | down | mRNA |
| NM_145257 | CCSAP | 0.988288296 | 0.999140676 | 1.0018106 | down | mRNA |
| NM_145260 | OSR1 | 0.006718967 | 0.183552613 | 2.0671631 | down | mRNA |
| NM_145261 | DNAJC19 | 0.812687146 | 0.98118927 | 1.0310381 | down | mRNA |
| NM_145263 | SPATA18 | 0.867954539 | 0.989481868 | 1.107073 | down | mRNA |
| NM_145265 | CCDC127 | 0.001075131 | 0.086778252 | 1.1511548 | down | mRNA |
| NM_145267 | C6orf57 | 0.951103433 | 0.996710991 | 1.0040493 | down | mRNA |
| NM_145270 | C16orf11 | 0.183054947 | 0.666166668 | 1.237584 | down | mRNA |
| NM_145274 | TMEM99 | 0.418630065 | 0.872328878 | 1.0575746 | down | mRNA |
| NM_145276 | ZNF563 | 0.000553572 | 0.071689326 | 1.3576996 | down | mRNA |
| NM_145282 | SLC25A48 | 0.96799771 | 0.99793927 | 1.0019587 | down | mRNA |
| NM_145284 | FAM122B | 0.072368032 | 0.463255488 | 1.162227 | down | mRNA |
| NM_145286 | STOML3 | 0.180427741 | 0.66337375 | 1.457339 | down | mRNA |
| NM_145290 | GPR125 | 0.838486935 | 0.98692349 | 1.0137618 | down | mRNA |
| NM_145291 | ZBTB49 | 0.770313724 | 0.975340556 | 1.0870264 | down | mRNA |
| NM_145292 | GALNTL5 | 0.686619412 | 0.960205549 | 1.101784 | down | mRNA |
| NM_145294 | WDR90 | 0.354954246 | 0.837818031 | 1.1569366 | down | mRNA |
| NM_145305 | SLC25A43 | 0.374577181 | 0.84936907 | 1.1775848 | down | mRNA |
| NM_145306 | C10orf35 | 0.401384865 | 0.864768873 | 1.189309 | down | mRNA |
| NM_145315 | LACE1 | 0.756255729 | 0.97167109 | 1.0291752 | down | mRNA |
| NM_145320 | OSBPL3 | 0.002996232 | 0.130169625 | 1.2349852 | down | mRNA |
| NM_145321 | OSBPL3 | 0.121734511 | 0.573339375 | 1.1012239 | down | mRNA |
| NM_145322 | OSBPL3 | 0.004186208 | 0.147767322 | 1.2064257 | down | mRNA |
| NM_145326 | ZNF493 | 0.478758319 | 0.901271944 | 1.1467348 | down | mRNA |
| NM_145330 | MRPL33 | 0.670552646 | 0.959531882 | 1.0307406 | down | mRNA |
| NM_145332 | MAP3K7 | 0.388184933 | 0.857609048 | 1.141176 | down | mRNA |
| NM_145333 | MAP3K7 | 0.549922083 | 0.925533435 | 1.10466 | down | mRNA |
| NM_145343 | APOL1 | 0.787992849 | 0.97758108 | 1.0566434 | down | mRNA |
| NM_145638 | OSBPL5 | 0.595531594 | 0.938746918 | 1.2038933 | down | mRNA |
| NM_145644 | MRPL35 | 0.011924935 | 0.222234451 | 1.1983075 | down | mRNA |
| NM_145647 | WDR67 | 0.290520598 | 0.786596721 | 1.3142602 | down | mRNA |
| NM_145648 | SLC15A4 | 0.939909631 | 0.996710991 | 1.0059503 | down | mRNA |
| NM_145685 | BRF1 | 0.026411403 | 0.318338323 | 1.6601247 | down | mRNA |
| NM_145691 | ATPAF2 | 0.180799576 | 0.663780602 | 1.1234549 | down | mRNA |
| NM_145701 | CDCA4 | 0.169964789 | 0.646789844 | 1.7816342 | down | mRNA |
| NM_145714 | ATXN2L | 0.659113914 | 0.958354006 | 1.0517861 | down | mRNA |
| NM_145715 | TIGD2 | 0.035433172 | 0.353725338 | 2.136786 | down | mRNA |
| NM_145719 | TIGD3 | 0.459148856 | 0.892904132 | 1.0201592 | down | mRNA |
| NM_145730 | AP1B1 | 0.60942945 | 0.943053241 | 1.0878552 | down | mRNA |
| NM_145735 | ARHGEF7 | 0.126096926 | 0.578475338 | 1.1939095 | down | mRNA |
| NM_145754 | KIFC2 | 0.761971211 | 0.97339948 | 1.0393973 | down | mRNA |
| NM_145759 | TRAF5 | 0.815051939 | 0.981277402 | 1.0747335 | down | mRNA |
| NM_145792 | MGST1 | 0.870732932 | 0.989592579 | 1.032538 | down | mRNA |
| NM_145796 | POGZ | 0.474187147 | 0.899418411 | 1.0423374 | down | mRNA |
| NM_145806 | ZNF511 | 0.032288073 | 0.343300278 | 1.1211966 | down | mRNA |
| NM_145814 | CACNG6 | 0.928349523 | 0.996053205 | 1.0152802 | down | mRNA |
| NM_145815 | CACNG6 | 0.664889561 | 0.958354006 | 1.0570093 | down | mRNA |
| NM_145858 | CRYZL1 | 0.682167418 | 0.960111041 | 1.0195296 | down | mRNA |
| NM_145860 | PDCD10 | 0.050389069 | 0.4062294 | 1.1410418 | down | mRNA |
| NM_145863 | ASB3 | 0.181675944 | 0.664924727 | 1.151984 | down | mRNA |
| NM_145870 | GSTZ1 | 0.535156429 | 0.921242952 | 1.037408 | down | mRNA |
| NM_145886 | PIDD | 0.217526809 | 0.711740438 | 1.2321637 | down | mRNA |
| NM_145887 | PIDD | 0.241736163 | 0.7401831 | 1.1853141 | down | mRNA |
| NM_145895 | KLK12 | 0.501469773 | 0.910102957 | 1.0864058 | down | mRNA |
| NM_145910 | NEK11 | 0.996872424 | 0.999872237 | 1.0005041 | down | mRNA |
| NM_145914 | ZSCAN21 | 0.001693926 | 0.10523567 | 1.4000107 | down | mRNA |
| NM_146387 | MRPL4 | 0.216653304 | 0.711074992 | 1.1350142 | down | mRNA |
| NM_147127 | EVC2 | 0.928574499 | 0.996136586 | 1.0103665 | down | mRNA |
| NM_147133 | NFX1 | 0.484696711 | 0.903250293 | 1.0560982 | down | mRNA |
| NM_147134 | NFX1 | 0.0116476 | 0.22025265 | 1.5971409 | down | mRNA |
| NM_147148 | GSTM4 | 0.666394229 | 0.958354006 | 1.1031454 | down | mRNA |
| NM_147150 | PALM2-AKAP2 | 0.726531791 | 0.966191059 | 1.0340653 | down | mRNA |
| NM_147156 | SGMS1 | 0.035558499 | 0.354399703 | 1.1582767 | down | mRNA |
| NM_147157 | SIGMAR1 | 0.203428416 | 0.692931236 | 1.0902275 | down | mRNA |
| NM_147172 | NUDT2 | 0.646883754 | 0.95550188 | 1.1033459 | down | mRNA |
| NM_147188 | FBXO22 | 0.450334355 | 0.888519952 | 1.0777222 | down | mRNA |
| NM_147189 | FAM110B | 0.088425077 | 0.502587299 | 1.1142588 | down | mRNA |
| NM_147190 | CERS5 | 0.012779215 | 0.23037549 | 1.2407757 | down | mRNA |
| NM_147193 | GLIS1 | 0.008793145 | 0.202329498 | 1.1108917 | down | mRNA |
| NM_147202 | FAM219A | 0.920977215 | 0.995349793 | 1.0296807 | down | mRNA |
| NM_147204 | TRPV4 | 0.722372921 | 0.966191059 | 1.0581286 | down | mRNA |
| NM_147223 | NCOA1 | 0.478758683 | 0.901271944 | 1.1392272 | down | mRNA |
| NM_147233 | NCOA1 | 0.137347358 | 0.596770033 | 1.2859963 | down | mRNA |
| NM_147777 | SNX15 | 0.283953175 | 0.78179061 | 1.1269971 | down | mRNA |
| NM_147781 | CTSB | 0.951596755 | 0.996710991 | 1.0101938 | down | mRNA |
| NM_148176 | PPIL2 | 0.723963943 | 0.966191059 | 1.0728936 | down | mRNA |
| NM_148179 | RPP25L | 0.995912292 | 0.999872237 | 1.0005169 | down | mRNA |
| NM_148414 | ATXN2L | 0.598032749 | 0.940241406 | 1.1452376 | down | mRNA |
| NM_148672 | CCL28 | 0.803972433 | 0.98118927 | 1.0516274 | down | mRNA |
| NM_148674 | SMC1B | 0.709478861 | 0.965462762 | 1.035312 | down | mRNA |
| NM_148894 | BOD1L1 | 0.12833255 | 0.581903971 | 1.1233317 | down | mRNA |
| NM_148901 | TNFRSF18 | 0.469491065 | 0.89787452 | 1.2384666 | down | mRNA |
| NM_148902 | TNFRSF18 | 0.523267188 | 0.917239107 | 1.2819955 | down | mRNA |
| NM_148904 | OSBPL9 | 0.218594936 | 0.712369327 | 1.2815733 | down | mRNA |
| NM_148906 | OSBPL9 | 0.360548266 | 0.841780454 | 1.2481898 | down | mRNA |
| NM_148907 | OSBPL9 | 0.863902206 | 0.989401142 | 1.0598244 | down | mRNA |
| NM_148910 | TIRAP | 0.152263973 | 0.618255775 | 1.109743 | down | mRNA |
| NM_148912 | ABHD11 | 0.005545921 | 0.169833068 | 1.2178372 | down | mRNA |
| NM_148918 | SHMT1 | 0.505107895 | 0.910568383 | 1.0901192 | down | mRNA |
| NM_148955 | SNX1 | 0.297253761 | 0.79373308 | 1.1563039 | down | mRNA |
| NM_148956 | NSUN5 | 0.257034129 | 0.757669958 | 1.2884175 | down | mRNA |
| NM_148962 | OXER1 | 0.942880859 | 0.996710991 | 1.0108075 | down | mRNA |
| NM_148965 | TNFRSF25 | 0.56549508 | 0.930155001 | 1.0541815 | down | mRNA |
| NM_148966 | TNFRSF25 | 0.353429814 | 0.836351835 | 1.1322894 | down | mRNA |
| NM_148967 | TNFRSF25 | 0.133058491 | 0.588930122 | 1.1659309 | down | mRNA |
| NM_148970 | TNFRSF25 | 0.028501312 | 0.327950578 | 1.5788921 | down | mRNA |
| NM_148975 | MS4A4A | 0.657785662 | 0.958167222 | 1.2680417 | down | mRNA |
| NM_148978 | PANK1 | 0.096309663 | 0.51798222 | 1.0933409 | down | mRNA |
| NM_152133 | TAGAP | 0.42356017 | 0.874474551 | 1.0968376 | down | mRNA |
| NM_152227 | SNX5 | 0.330828351 | 0.821853462 | 1.1119408 | down | mRNA |
| NM_152237 | GAS2L1 | 0.614658112 | 0.944827645 | 1.1130376 | down | mRNA |
| NM_152238 | SNX7 | 0.664202307 | 0.958354006 | 1.096309 | down | mRNA |
| NM_152240 | ZMAT3 | 0.316726866 | 0.811452953 | 1.3304504 | down | mRNA |
| NM_152243 | CDC42EP1 | 0.302140129 | 0.797496185 | 1.1207683 | down | mRNA |
| NM_152244 | SNX11 | 0.078930114 | 0.481490271 | 1.1638046 | down | mRNA |
| NM_152261 | C12orf23 | 0.182641338 | 0.665972684 | 1.1087753 | down | mRNA |
| NM_152262 | ZNF439 | 0.002640096 | 0.122061602 | 1.5697638 | down | mRNA |
| NM_152265 | BTF3L4 | 0.641792748 | 0.954332328 | 1.0219254 | down | mRNA |
| NM_152269 | C12orf65 | 0.151647849 | 0.616902989 | 1.2865581 | down | mRNA |
| NM_152272 | CHMP7 | 0.326867066 | 0.819869378 | 1.1857599 | down | mRNA |
| NM_152274 | FAM58A | 0.830062465 | 0.985549562 | 1.0268293 | down | mRNA |
| NM_152277 | UBTD2 | 0.944048429 | 0.996710991 | 1.0097469 | down | mRNA |
| NM_152278 | TCEAL7 | 0.851266863 | 0.987678166 | 1.1081442 | down | mRNA |
| NM_152279 | ZNF585B | 0.330804979 | 0.821853462 | 1.2653266 | down | mRNA |
| NM_152282 | ACPL2 | 0.814898467 | 0.981277402 | 1.0299068 | down | mRNA |
| NM_152283 | ZFP62 | 0.006620873 | 0.183552613 | 1.3645734 | down | mRNA |
| NM_152288 | ORAI3 | 0.09924808 | 0.523453169 | 1.2172539 | down | mRNA |
| NM_152289 | ZNF561 | 0.069201146 | 0.456727607 | 1.2708567 | down | mRNA |
| NM_152295 | TARS | 0.871990825 | 0.990243379 | 1.0103451 | down | mRNA |
| NM_152298 | NASP | 0.126866104 | 0.580406758 | 1.1289981 | down | mRNA |
| NM_152299 | NCAPH2 | 0.579377862 | 0.933821303 | 1.1654792 | down | mRNA |
| NM_152305 | POGLUT1 | 0.703716877 | 0.963625105 | 1.1073896 | down | mRNA |
| NM_152307 | TRMT61A | 0.700452972 | 0.962991468 | 1.0203127 | down | mRNA |
| NM_152316 | ARL14EP | 0.042060184 | 0.379087707 | 1.3158681 | down | mRNA |
| NM_152318 | C12orf45 | 0.393127669 | 0.859795559 | 1.0475741 | down | mRNA |
| NM_152326 | ANKRD9 | 0.702629516 | 0.963478781 | 1.0664801 | down | mRNA |
| NM_152328 | ADSSL1 | 0.897947313 | 0.994326043 | 1.0149805 | down | mRNA |
| NM_152331 | ACOT4 | 0.529823998 | 0.91899174 | 1.3289692 | down | mRNA |
| NM_152334 | TARSL2 | 0.204278265 | 0.694397489 | 1.2814326 | down | mRNA |
| NM_152337 | C16orf46 | 0.573492985 | 0.933656777 | 1.0541056 | down | mRNA |
| NM_152339 | SPATA2L | 0.382969256 | 0.853514109 | 1.1951631 | down | mRNA |
| NM_152341 | PAQR4 | 0.801358137 | 0.98118927 | 1.0950183 | down | mRNA |
| NM_152342 | CDYL2 | 0.659693367 | 0.958354006 | 1.2502629 | down | mRNA |
| NM_152343 | TEX34 | 0.143847686 | 0.604279163 | 1.26881 | down | mRNA |
| NM_152345 | ANKRD13B | 0.500477833 | 0.909680616 | 1.1672461 | down | mRNA |
| NM_152357 | ZNF440 | 0.001371268 | 0.094832054 | 1.6291736 | down | mRNA |
| NM_152358 | IZUMO2 | 0.582417027 | 0.934005566 | 1.036879 | down | mRNA |
| NM_152363 | ANKLE1 | 0.082904606 | 0.491146983 | 1.132308 | down | mRNA |
| NM_152366 | KLHDC9 | 0.30679993 | 0.801817496 | 1.2268523 | down | mRNA |
| NM_152369 | SLC44A3 | 0.05302461 | 0.413697761 | 1.1476954 | down | mRNA |
| NM_152371 | FAM213B | 0.973413675 | 0.998501532 | 1.003451 | down | mRNA |
| NM_152374 | C1orf216 | 0.441484664 | 0.883920737 | 1.1297897 | down | mRNA |
| NM_152379 | C1orf131 | 0.220178169 | 0.715236067 | 1.2568551 | down | mRNA |
| NM_152380 | TBX15 | 0.994021345 | 0.999872237 | 1.0046031 | down | mRNA |
| NM_152384 | BBS5 | 0.007379336 | 0.190938402 | 1.7217402 | down | mRNA |
| NM_152385 | CLHC1 | 0.677885939 | 0.959531882 | 1.2297422 | down | mRNA |
| NM_152387 | KCTD18 | 0.320489784 | 0.814552264 | 1.1942457 | down | mRNA |
| NM_152390 | TMEM178A | 0.033402547 | 0.347386493 | 1.2393227 | down | mRNA |
| NM_152392 | AHSA2 | 0.677595756 | 0.959531882 | 1.1567395 | down | mRNA |
| NM_152402 | TRAM1L1 | 0.049009708 | 0.401962642 | 1.261473 | down | mRNA |
| NM_152405 | JMY | 0.169634005 | 0.646121878 | 1.4048238 | down | mRNA |
| NM_152406 | AFAP1L1 | 0.234879401 | 0.732404233 | 1.1106946 | down | mRNA |
| NM_152413 | GOT1L1 | 0.979614989 | 0.998971807 | 1.0024407 | down | mRNA |
| NM_152415 | VPS37A | 0.677494937 | 0.959531882 | 1.0561349 | down | mRNA |
| NM_152417 | TMEM68 | 0.027275447 | 0.3212251 | 1.1556113 | down | mRNA |
| NM_152418 | DCAF4L2 | 0.449977316 | 0.888279993 | 1.3244793 | down | mRNA |
| NM_152419 | HGSNAT | 0.971423093 | 0.998501532 | 1.007838 | down | mRNA |
| NM_152420 | C9orf41 | 0.050341643 | 0.406008044 | 1.2533061 | down | mRNA |
| NM_152422 | PTPDC1 | 0.091784651 | 0.510858344 | 1.2887091 | down | mRNA |
| NM_152433 | KBTBD3 | 0.943538584 | 0.996710991 | 1.0053844 | down | mRNA |
| NM_152434 | CWF19L2 | 0.621375455 | 0.947985725 | 1.1097292 | down | mRNA |
| NM_152436 | GLIPR1L2 | 0.784910722 | 0.977411899 | 1.0552025 | down | mRNA |
| NM_152437 | ZNF664 | 0.415583722 | 0.870382888 | 1.1260423 | down | mRNA |
| NM_152440 | C12orf66 | 0.039699999 | 0.370777582 | 1.5439165 | down | mRNA |
| NM_152446 | CEP128 | 0.960717063 | 0.997798988 | 1.0202463 | down | mRNA |
| NM_152448 | C15orf43 | 0.185363778 | 0.668941487 | 1.3834919 | down | mRNA |
| NM_152451 | MYZAP | 0.187300684 | 0.671419231 | 1.8201551 | down | mRNA |
| NM_152463 | EME1 | 0.829031907 | 0.98552522 | 1.1197026 | down | mRNA |
| NM_152464 | TMEM199 | 0.931635247 | 0.996710991 | 1.0054069 | down | mRNA |
| NM_152465 | PROCA1 | 0.671813285 | 0.959531882 | 1.1591387 | down | mRNA |
| NM_152474 | C19orf18 | 0.691629037 | 0.961451327 | 1.0438433 | down | mRNA |
| NM_152477 | ZNF565 | 0.216687609 | 0.711074992 | 1.5075045 | down | mRNA |
| NM_152482 | C19orf25 | 0.693417491 | 0.961584652 | 1.0255959 | down | mRNA |
| NM_152484 | ZNF569 | 0.055852044 | 0.421575513 | 1.4701501 | down | mRNA |
| NM_152485 | C1orf74 | 0.790356526 | 0.978438986 | 1.0537454 | down | mRNA |
| NM_152486 | SAMD11 | 0.056300364 | 0.422710116 | 2.4659446 | down | mRNA |
| NM_152487 | TMEM56 | 0.895009735 | 0.993978977 | 1.0181011 | down | mRNA |
| NM_152490 | B3GALNT2 | 0.077838476 | 0.479367476 | 1.1622316 | down | mRNA |
| NM_152492 | CCDC27 | 0.819422326 | 0.982224663 | 1.0228315 | down | mRNA |
| NM_152504 | C20orf196 | 0.010068177 | 0.208034735 | 1.4488088 | down | mRNA |
| NM_152511 | DUSP18 | 0.029725991 | 0.334953885 | 1.3281862 | down | mRNA |
| NM_152519 | KANSL1L | 0.521047912 | 0.916644975 | 1.1517121 | down | mRNA |
| NM_152522 | ARL6IP6 | 0.183458711 | 0.666919813 | 1.2186851 | down | mRNA |
| NM_152523 | CCNYL1 | 0.226114382 | 0.721636281 | 1.1452175 | down | mRNA |
| NM_152524 | SGOL2 | 0.434254338 | 0.880931467 | 1.4351342 | down | mRNA |
| NM_152526 | PARD3B | 0.808705305 | 0.98118927 | 1.0832912 | down | mRNA |
| NM_152528 | WDSUB1 | 0.693223391 | 0.961584652 | 1.0755925 | down | mRNA |
| NM_152531 | XXYLT1 | 0.995954501 | 0.999872237 | 1.0025276 | down | mRNA |
| NM_152533 | C3orf22 | 0.843065712 | 0.987338076 | 1.0309109 | down | mRNA |
| NM_152540 | SCFD2 | 0.891330609 | 0.9937521 | 1.0587204 | down | mRNA |
| NM_152544 | TRMT44 | 0.195007344 | 0.682660006 | 1.1309834 | down | mRNA |
| NM_152549 | CCDC112 | 0.416294451 | 0.870736736 | 1.2011827 | down | mRNA |
| NM_152550 | SH3RF2 | 0.53375709 | 0.92058057 | 1.0633601 | down | mRNA |
| NM_152551 | SNRNP48 | 0.12409231 | 0.577766088 | 1.1212525 | down | mRNA |
| NM_152556 | C7orf60 | 0.229595973 | 0.726672685 | 1.3361923 | down | mRNA |
| NM_152559 | WBSCR27 | 0.828029455 | 0.985280908 | 1.0374692 | down | mRNA |
| NM_152562 | CDCA2 | 0.577270507 | 0.933656777 | 1.3909281 | down | mRNA |
| NM_152563 | FAM86C1 | 0.597488952 | 0.939895193 | 1.0805162 | down | mRNA |
| NM_152564 | VPS13B | 0.744737747 | 0.970330526 | 1.0418963 | down | mRNA |
| NM_152581 | MOSPD2 | 0.528009427 | 0.918844141 | 1.1639968 | down | mRNA |
| NM_152589 | C12orf50 | 0.864685188 | 0.989401142 | 1.0289719 | down | mRNA |
| NM_152594 | SPRED1 | 0.288518003 | 0.784514546 | 1.150445 | down | mRNA |
| NM_152603 | ZNF567 | 0.000299703 | 0.055482352 | 1.3751411 | down | mRNA |
| NM_152604 | ZNF383 | 0.000168006 | 0.044621794 | 1.5544878 | down | mRNA |
| NM_152608 | SDE2 | 0.401186348 | 0.864768873 | 1.2543899 | down | mRNA |
| NM_152609 | CNST | 0.004853533 | 0.158753988 | 1.2744617 | down | mRNA |
| NM_152612 | CCDC116 | 0.695813096 | 0.962410025 | 1.0645886 | down | mRNA |
| NM_152617 | RNF168 | 0.70068525 | 0.962991468 | 1.10123 | down | mRNA |
| NM_152622 | MIER3 | 0.008449418 | 0.198394583 | 1.3206029 | down | mRNA |
| NM_152626 | ZNF92 | 0.002317001 | 0.114621062 | 1.232176 | down | mRNA |
| NM_152633 | FANCB | 0.54564353 | 0.924167487 | 1.1925678 | down | mRNA |
| NM_152637 | METTL7B | 0.20788317 | 0.698624894 | 1.3997448 | down | mRNA |
| NM_152640 | DCP1B | 0.016352629 | 0.255922225 | 1.7741275 | down | mRNA |
| NM_152641 | ARID2 | 0.058109625 | 0.42931864 | 1.4462572 | down | mRNA |
| NM_152644 | FAM24B | 0.504047678 | 0.910568383 | 1.1999246 | down | mRNA |
| NM_152647 | FAM227B | 0.674940138 | 0.959531882 | 1.1804075 | down | mRNA |
| NM_152649 | MLKL | 0.651819377 | 0.956204521 | 1.0673042 | down | mRNA |
| NM_152665 | TCTEX1D1 | 0.905140495 | 0.99508468 | 1.0215783 | down | mRNA |
| NM_152667 | NANP | 0.085249462 | 0.49712038 | 1.2920966 | down | mRNA |
| NM_152670 | C2orf51 | 0.506620424 | 0.911415165 | 1.1718988 | down | mRNA |
| NM_152671 | PIKFYVE | 0.353758635 | 0.836545774 | 1.1137181 | down | mRNA |
| NM_152672 | SLC51A | 0.258391124 | 0.759296063 | 1.0816675 | down | mRNA |
| NM_152678 | FAM116A | 0.039696851 | 0.370777582 | 1.337035 | down | mRNA |
| NM_152679 | SLC10A4 | 0.62885027 | 0.950128593 | 1.096064 | down | mRNA |
| NM_152682 | RWDD4 | 0.977291276 | 0.998501532 | 1.0105559 | down | mRNA |
| NM_152692 | C1GALT1C1 | 0.978162716 | 0.998641374 | 1.0044233 | down | mRNA |
| NM_152695 | ZNF449 | 0.196924075 | 0.685161614 | 1.4380211 | down | mRNA |
| NM_152696 | HIPK1 | 0.151768441 | 0.617002892 | 1.164907 | down | mRNA |
| NM_152705 | POLR1D | 0.42928001 | 0.877903959 | 1.0615048 | down | mRNA |
| NM_152706 | C7orf62 | 0.221581666 | 0.716734257 | 1.2929909 | down | mRNA |
| NM_152707 | SLC25A16 | 0.029688805 | 0.334953885 | 1.1423067 | down | mRNA |
| NM_152713 | STT3A | 0.318300315 | 0.812311032 | 1.0658961 | down | mRNA |
| NM_152718 | VWCE | 0.275768769 | 0.775616353 | 1.192568 | down | mRNA |
| NM_152724 | RSU1 | 0.616800196 | 0.945960598 | 1.0398386 | down | mRNA |
| NM_152729 | NT5DC1 | 0.679458554 | 0.959969193 | 1.05456 | down | mRNA |
| NM_152730 | C6orf170 | 0.268523616 | 0.769158724 | 1.2594486 | down | mRNA |
| NM_152731 | BEND6 | 0.464025001 | 0.894989844 | 1.1868703 | down | mRNA |
| NM_152732 | RSPH9 | 0.593705195 | 0.938299318 | 1.1572056 | down | mRNA |
| NM_152734 | C6orf89 | 0.6974323 | 0.962410025 | 1.0390817 | down | mRNA |
| NM_152735 | ZBTB9 | 0.000694271 | 0.078859848 | 1.6434358 | down | mRNA |
| NM_152736 | ZNF187 | 0.132572558 | 0.588144285 | 1.3240262 | down | mRNA |
| NM_152739 | HOXA9 | 0.734228566 | 0.967777568 | 1.032321 | down | mRNA |
| NM_152740 | HIBADH | 0.646614207 | 0.95550188 | 1.1135034 | down | mRNA |
| NM_152743 | BRAT1 | 0.506145211 | 0.911278334 | 1.1113813 | down | mRNA |
| NM_152749 | ATXN7L1 | 0.347125643 | 0.833588015 | 1.2730732 | down | mRNA |
| NM_152751 | BEND7 | 0.563612277 | 0.929146073 | 1.1444043 | down | mRNA |
| NM_152753 | SCUBE3 | 0.895774981 | 0.993978977 | 1.1128789 | down | mRNA |
| NM_152756 | RICTOR | 0.899986862 | 0.994752372 | 1.0220405 | down | mRNA |
| NM_152758 | YTHDF3 | 0.382240654 | 0.853514109 | 1.0925059 | down | mRNA |
| NM_152764 | C16orf73 | 0.885931669 | 0.992981836 | 1.030299 | down | mRNA |
| NM_152766 | C17orf61 | 0.785724339 | 0.977479172 | 1.0268326 | down | mRNA |
| NM_152772 | TCP11L2 | 0.695617978 | 0.96236244 | 1.2699456 | down | mRNA |
| NM_152774 | TMEM196 | 0.407939837 | 0.867591329 | 1.1309261 | down | mRNA |
| NM_152775 | CCDC110 | 0.02258383 | 0.295888941 | 1.6759922 | down | mRNA |
| NM_152778 | MFSD8 | 0.047641823 | 0.397835916 | 1.1245242 | down | mRNA |
| NM_152780 | MAP7D2 | 0.551748786 | 0.92621751 | 1.0535678 | down | mRNA |
| NM_152792 | ASPRV1 | 0.837290619 | 0.986775289 | 1.0366397 | down | mRNA |
| NM_152827 | SNX3 | 0.402077894 | 0.864786348 | 1.1253677 | down | mRNA |
| NM_152829 | TES | 0.810607081 | 0.98118927 | 1.0844903 | down | mRNA |
| NM_152830 | ACE | 0.726726823 | 0.966191059 | 1.0321948 | down | mRNA |
| NM_152835 | PDIK1L | 0.000997786 | 0.085705167 | 1.8177477 | down | mRNA |
| NM_152837 | SNX16 | 0.027458352 | 0.322334413 | 1.0981171 | down | mRNA |
| NM_152838 | RBM12 | 0.468262397 | 0.897203143 | 1.1217115 | down | mRNA |
| NM_152855 | IGLL1 | 0.427850639 | 0.876992614 | 1.2124607 | down | mRNA |
| NM_152856 | RBM10 | 0.640224403 | 0.953891706 | 1.088427 | down | mRNA |
| NM_152868 | KCNJ4 | 0.90055076 | 0.994789506 | 1.0102766 | down | mRNA |
| NM_152872 | FAS | 0.869923221 | 0.989592579 | 1.0230479 | down | mRNA |
| NM_152879 | DGKD | 0.06772624 | 0.451808679 | 1.211017 | down | mRNA |
| NM_152880 | PTK7 | 0.720697403 | 0.966191059 | 1.1050547 | down | mRNA |
| NM_152881 | PTK7 | 0.959513005 | 0.99758736 | 1.0087516 | down | mRNA |
| NM_152882 | PTK7 | 0.663191756 | 0.958354006 | 1.0530938 | down | mRNA |
| NM_152892 | LRWD1 | 0.018663913 | 0.273264013 | 1.5199436 | down | mRNA |
| NM_152896 | UHRF2 | 0.006692824 | 0.183552613 | 1.5925783 | down | mRNA |
| NM_152900 | MAGI3 | 0.172177116 | 0.6499464 | 1.1877448 | down | mRNA |
| NM_152903 | KBTBD6 | 0.842178518 | 0.987123192 | 1.0342477 | down | mRNA |
| NM_152908 | SLC47A2 | 0.191988673 | 0.677811026 | 1.3344495 | down | mRNA |
| NM_152909 | ZNF548 | 0.002820941 | 0.125779344 | 1.2917635 | down | mRNA |
| NM_152912 | MTIF3 | 0.628618847 | 0.950054144 | 1.0406246 | down | mRNA |
| NM_152925 | CPNE1 | 0.097373207 | 0.520175647 | 1.111213 | down | mRNA |
| NM_152945 | RBM45 | 0.011286045 | 0.217918205 | 1.1399403 | down | mRNA |
| NM_152991 | EED | 0.490366232 | 0.905264563 | 1.2411892 | down | mRNA |
| NM_152992 | POMZP3 | 0.942595891 | 0.996710991 | 1.008933 | down | mRNA |
| NM_152994 | PPP1R21 | 0.046617563 | 0.39300536 | 1.181126 | down | mRNA |
| NM_152995 | NFXL1 | 0.00306442 | 0.131446817 | 1.4664214 | down | mRNA |
| NM_152997 | FDCSP | 0.62308724 | 0.948341211 | 1.0534192 | down | mRNA |
| NM_153018 | ZFP3 | 0.008017178 | 0.195524479 | 2.3477816 | down | mRNA |
| NM_153028 | ZNF75A | 0.059474155 | 0.432586127 | 1.315856 | down | mRNA |
| NM_153045 | C9orf91 | 0.349716476 | 0.834138149 | 1.0576438 | down | mRNA |
| NM_153046 | TDRD9 | 0.894058529 | 0.993978977 | 1.092125 | down | mRNA |
| NM_153050 | MTMR3 | 0.015322586 | 0.248436066 | 1.3422283 | down | mRNA |
| NM_153051 | MTMR3 | 0.777433893 | 0.976938981 | 1.0265697 | down | mRNA |
| NM_153182 | MINA | 0.883731731 | 0.992766819 | 1.0375548 | down | mRNA |
| NM_153187 | SLC22A1 | 0.513279628 | 0.91350662 | 1.0795998 | down | mRNA |
| NM_153200 | EDF1 | 0.522759608 | 0.917239107 | 1.0453695 | down | mRNA |
| NM_153201 | HSPA8 | 0.093647944 | 0.512619241 | 1.1282945 | down | mRNA |
| NM_153207 | AEBP2 | 0.052916805 | 0.413038171 | 1.266364 | down | mRNA |
| NM_153210 | USP43 | 0.989513647 | 0.999140676 | 1.0011062 | down | mRNA |
| NM_153211 | TTC39C | 0.979631384 | 0.998971807 | 1.0032918 | down | mRNA |
| NM_153217 | TMEM174 | 0.977341077 | 0.998501532 | 1.005636 | down | mRNA |
| NM_153223 | CEP120 | 0.737212264 | 0.968907547 | 1.0594702 | down | mRNA |
| NM_153232 | EID2 | 0.100710392 | 0.527535765 | 1.2038874 | down | mRNA |
| NM_153240 | NPHP3 | 0.018954732 | 0.276065624 | 1.403506 | down | mRNA |
| NM_153246 | C6orf223 | 0.143998186 | 0.604786431 | 1.0682901 | down | mRNA |
| NM_153251 | ZDHHC20 | 0.174235597 | 0.652872869 | 1.0698932 | down | mRNA |
| NM_153252 | BRWD3 | 0.019531883 | 0.281049008 | 1.3001722 | down | mRNA |
| NM_153255 | MCM9 | 0.085518998 | 0.497360491 | 1.9454868 | down | mRNA |
| NM_153261 | CNEP1R1 | 0.75860105 | 0.972072638 | 1.0443008 | down | mRNA |
| NM_153262 | SYT14 | 0.754090252 | 0.970944772 | 1.2034808 | down | mRNA |
| NM_153263 | ZNF549 | 0.105349199 | 0.537370779 | 1.1894899 | down | mRNA |
| NM_153266 | TMEM151A | 0.941618399 | 0.996710991 | 1.0140086 | down | mRNA |
| NM_153267 | MAMDC2 | 0.715579571 | 0.966191059 | 1.0646327 | down | mRNA |
| NM_153269 | C20orf96 | 0.801271828 | 0.98118927 | 1.0379976 | down | mRNA |
| NM_153273 | IP6K1 | 0.092782134 | 0.511370653 | 1.3548157 | down | mRNA |
| NM_153274 | BEST4 | 0.738509152 | 0.969444674 | 1.034261 | down | mRNA |
| NM_153276 | SLC22A6 | 0.989884657 | 0.999140676 | 1.0036084 | down | mRNA |
| NM_153280 | UBA1 | 0.272330641 | 0.772141487 | 1.0845126 | down | mRNA |
| NM_153282 | HYAL1 | 0.473883133 | 0.899418411 | 1.0862546 | down | mRNA |
| NM_153320 | SLC22A7 | 0.197861248 | 0.686504248 | 1.173294 | down | mRNA |
| NM_153322 | PMP22 | 0.077008001 | 0.477122268 | 1.2266517 | down | mRNA |
| NM_153329 | ALDH16A1 | 0.911604344 | 0.995342392 | 1.0178821 | down | mRNA |
| NM_153333 | TCEAL8 | 0.382441063 | 0.853514109 | 1.1094577 | down | mRNA |
| NM_153334 | SCARF2 | 0.325321344 | 0.817707203 | 1.2445571 | down | mRNA |
| NM_153335 | STRADA | 0.432200915 | 0.880427272 | 1.1366769 | down | mRNA |
| NM_153339 | PUSL1 | 0.232589324 | 0.730348439 | 1.2275736 | down | mRNA |
| NM_153340 | ATXN7L2 | 0.541254226 | 0.923577434 | 1.0457815 | down | mRNA |
| NM_153348 | FBXW8 | 0.719122339 | 0.966191059 | 1.0846666 | down | mRNA |
| NM_153358 | ZNF791 | 0.50541104 | 0.910772598 | 1.0527098 | down | mRNA |
| NM_153365 | TAPT1 | 0.018380251 | 0.270998737 | 1.5905185 | down | mRNA |
| NM_153366 | SVEP1 | 0.947706293 | 0.996710991 | 1.0095538 | down | mRNA |
| NM_153367 | ZCCHC24 | 0.909216917 | 0.995342392 | 1.0127622 | down | mRNA |
| NM_153369 | KIAA1919 | 0.026309412 | 0.317461702 | 1.3819878 | down | mRNA |
| NM_153371 | LNX2 | 0.026484796 | 0.318338323 | 1.7819882 | down | mRNA |
| NM_153373 | AGXT2L2 | 0.002099592 | 0.110028846 | 1.2611876 | down | mRNA |
| NM_153374 | LYSMD2 | 0.419303082 | 0.872365335 | 1.1149629 | down | mRNA |
| NM_153376 | CCDC96 | 0.024371178 | 0.304929683 | 2.6298276 | down | mRNA |
| NM_153380 | ZNF41 | 0.755973898 | 0.971522741 | 1.0800395 | down | mRNA |
| NM_153427 | PITX2 | 0.683611677 | 0.960111041 | 1.134773 | down | mRNA |
| NM_153442 | GPR26 | 0.400865591 | 0.864768873 | 1.1225047 | down | mRNA |
| NM_153451 | ORAOV1 | 0.165517779 | 0.639363423 | 1.1182419 | down | mRNA |
| NM_153460 | IL17RC | 0.812010374 | 0.98118927 | 1.0521158 | down | mRNA |
| NM_153486 | LDHD | 0.990875291 | 0.999376923 | 1.0013216 | down | mRNA |
| NM_153487 | MDGA1 | 0.681437565 | 0.960111041 | 1.0301277 | down | mRNA |
| NM_153498 | CAMK1D | 0.425938639 | 0.875562067 | 1.0721831 | down | mRNA |
| NM_153603 | COG7 | 0.006709227 | 0.183552613 | 1.3093208 | down | mRNA |
| NM_153604 | MYOCD | 0.186422659 | 0.669888134 | 1.9317218 | down | mRNA |
| NM_153607 | CREBRF | 0.923701122 | 0.995456159 | 1.0365022 | down | mRNA |
| NM_153640 | PANK2 | 0.209300249 | 0.700367256 | 1.0813253 | down | mRNA |
| NM_153645 | NUP50 | 0.247915111 | 0.746093848 | 1.2117287 | down | mRNA |
| NM_153686 | LCORL | 0.106353452 | 0.540189454 | 1.1534522 | down | mRNA |
| NM_153687 | IKBIP | 0.553141293 | 0.926337111 | 1.1204156 | down | mRNA |
| NM_153688 | ZFP1 | 0.041536682 | 0.377694015 | 1.7412369 | down | mRNA |
| NM_153698 | AAED1 | 0.810026951 | 0.98118927 | 1.0364542 | down | mRNA |
| NM_153701 | IL12RB1 | 0.513449288 | 0.91350662 | 1.0755203 | down | mRNA |
| NM_153703 | PODN | 0.5757813 | 0.933656777 | 1.0483923 | down | mRNA |
| NM_153704 | TMEM67 | 0.561389699 | 0.928513767 | 1.1863952 | down | mRNA |
| NM_153710 | C9orf96 | 0.558232186 | 0.926833616 | 1.0992448 | down | mRNA |
| NM_153711 | FAM26E | 0.256707706 | 0.757240077 | 1.0747352 | down | mRNA |
| NM_153713 | LIX1L | 0.345325217 | 0.832533915 | 1.1142572 | down | mRNA |
| NM_153717 | EVC | 0.146213421 | 0.608122555 | 1.240325 | down | mRNA |
| NM_153741 | DPM3 | 0.815713621 | 0.981459009 | 1.0573559 | down | mRNA |
| NM_153742 | CTH | 0.312048758 | 0.80699254 | 1.171519 | down | mRNA |
| NM_153747 | PIGC | 0.717158832 | 0.966191059 | 1.0663935 | down | mRNA |
| NM_153757 | NAP1L5 | 0.120636605 | 0.572011066 | 1.4020583 | down | mRNA |
| NM_153759 | DNMT3A | 0.357480486 | 0.839643099 | 1.3127714 | down | mRNA |
| NM_153809 | TAF1L | 0.67038371 | 0.959531882 | 1.0989307 | down | mRNA |
| NM_153810 | CACUL1 | 0.172950556 | 0.650904119 | 1.1533958 | down | mRNA |
| NM_153811 | SLC38A6 | 0.165139179 | 0.638608196 | 1.1740621 | down | mRNA |
| NM_153812 | PHF13 | 0.087966972 | 0.501554816 | 1.6003623 | down | mRNA |
| NM_153816 | SNX14 | 0.480218507 | 0.901538317 | 1.1419926 | down | mRNA |
| NM_153818 | PEX10 | 0.753001494 | 0.970725371 | 1.0938786 | down | mRNA |
| NM_153824 | PYCR1 | 0.003366988 | 0.137189565 | 1.2022906 | down | mRNA |
| NM_153826 | CD46 | 0.842571529 | 0.987190965 | 1.0231722 | down | mRNA |
| NM_153828 | RTN4 | 0.604866233 | 0.94174007 | 1.0728408 | down | mRNA |
| NM_170589 | CASC5 | 0.57692859 | 0.933656777 | 1.4764867 | down | mRNA |
| NM_170662 | CBLB | 0.07478094 | 0.469867247 | 1.4317617 | down | mRNA |
| NM_170663 | MINK1 | 0.532365428 | 0.920378445 | 1.2392093 | down | mRNA |
| NM_170665 | ATP2A2 | 0.038909092 | 0.368040971 | 1.2193962 | down | mRNA |
| NM_170675 | MEIS2 | 0.434866193 | 0.881261779 | 1.3194153 | down | mRNA |
| NM_170676 | MEIS2 | 0.028342054 | 0.327787624 | 1.6250366 | down | mRNA |
| NM_170677 | MEIS2 | 0.500625098 | 0.909680616 | 1.1819267 | down | mRNA |
| NM_170686 | ZNF398 | 0.834591453 | 0.986575022 | 1.0226185 | down | mRNA |
| NM_170691 | GFM2 | 0.220515275 | 0.715799221 | 1.0863066 | down | mRNA |
| NM_170692 | RASAL2 | 0.115473887 | 0.561007186 | 1.1395109 | down | mRNA |
| NM_170695 | TGIF1 | 0.995420418 | 0.999872237 | 1.0004006 | down | mRNA |
| NM_170707 | LMNA | 0.256535807 | 0.757240077 | 1.1160313 | down | mRNA |
| NM_170709 | SGK3 | 0.233203279 | 0.731100552 | 1.0703926 | down | mRNA |
| NM_170712 | RASSF1 | 0.047341525 | 0.396926965 | 1.28742 | down | mRNA |
| NM_170713 | RASSF1 | 0.007469465 | 0.192233838 | 1.3157782 | down | mRNA |
| NM_170719 | PROSER1 | 0.170099832 | 0.646926635 | 1.2258471 | down | mRNA |
| NM_170722 | NLRX1 | 0.106833469 | 0.541545276 | 1.1036931 | down | mRNA |
| NM_170726 | ALDH4A1 | 0.818179963 | 0.981818735 | 1.0253802 | down | mRNA |
| NM_170734 | BDNF | 0.417050175 | 0.870760336 | 1.6804037 | down | mRNA |
| NM_170739 | MRPL11 | 0.48170175 | 0.901837935 | 1.1552514 | down | mRNA |
| NM_170743 | IL28RA | 0.579868632 | 0.933893479 | 1.0756915 | down | mRNA |
| NM_170753 | PGBD3 | 0.926995095 | 0.995829678 | 1.018484 | down | mRNA |
| NM_170754 | TENC1 | 0.996656529 | 0.999872237 | 1.0009422 | down | mRNA |
| NM_170774 | RASSF2 | 0.526439932 | 0.917975454 | 1.0434976 | down | mRNA |
| NM_170784 | MKKS | 0.009731664 | 0.206100289 | 1.2496827 | down | mRNA |
| NM_171828 | KCNMB3 | 0.768985721 | 0.975270932 | 1.0193377 | down | mRNA |
| NM_171829 | KCNMB3 | 0.695574478 | 0.96236244 | 1.0509414 | down | mRNA |
| NM_171846 | LACTB | 0.670072229 | 0.959531882 | 1.1380032 | down | mRNA |
| NM_171982 | TRIM35 | 0.710070168 | 0.965462762 | 1.0297687 | down | mRNA |
| NM_172002 | HSCB | 0.475619004 | 0.900105754 | 1.0607711 | down | mRNA |
| NM_172003 | CBWD2 | 0.536404276 | 0.922509728 | 1.1145918 | down | mRNA |
| NM_172004 | CLECL1 | 0.885285221 | 0.992981836 | 1.0055335 | down | mRNA |
| NM_172020 | POM121 | 0.378063381 | 0.851532587 | 1.0624773 | down | mRNA |
| NM_172069 | PLEKHH2 | 0.354341592 | 0.837242678 | 1.1508874 | down | mRNA |
| NM_172070 | UBR3 | 0.031953965 | 0.342121122 | 1.23233 | down | mRNA |
| NM_172071 | RC3H1 | 0.595230265 | 0.938707475 | 1.0823815 | down | mRNA |
| NM_172081 | CAMK2B | 0.394346702 | 0.86105621 | 1.1289656 | down | mRNA |
| NM_172089 | TNFSF12-TNFSF13 | 0.97416338 | 0.998501532 | 1.0052736 | down | mRNA |
| NM_172103 | EYA4 | 0.370318037 | 0.846215286 | 1.4262803 | down | mRNA |
| NM_172114 | CAMK2D | 0.691764135 | 0.961451327 | 1.1134128 | down | mRNA |
| NM_172128 | CAMK2D | 0.488656115 | 0.904236483 | 1.0714252 | down | mRNA |
| NM_172129 | CAMK2D | 0.458140643 | 0.892646883 | 1.0892736 | down | mRNA |
| NM_172165 | MSH5 | 0.164587147 | 0.637911838 | 1.1555881 | down | mRNA |
| NM_172169 | CAMK2G | 0.004380564 | 0.151730183 | 1.3165187 | down | mRNA |
| NM_172171 | CAMK2G | 0.934845582 | 0.996710991 | 1.0136068 | down | mRNA |
| NM_172177 | MRPL42 | 0.603134412 | 0.941484604 | 1.1012105 | down | mRNA |
| NM_172195 | EIF2B4 | 0.191345641 | 0.676939305 | 1.0961408 | down | mRNA |
| NM_172206 | CAMKK1 | 0.034303796 | 0.351013984 | 2.6531049 | down | mRNA |
| NM_172207 | CAMKK1 | 0.04184423 | 0.378824525 | 1.8443446 | down | mRNA |
| NM_172213 | CD8B | 0.006737253 | 0.183552613 | 1.1424626 | down | mRNA |
| NM_172229 | KREMEN2 | 0.87465399 | 0.990905383 | 1.0146692 | down | mRNA |
| NM_172230 | SYVN1 | 0.773790127 | 0.97589756 | 1.0415264 | down | mRNA |
| NM_172238 | TFAP2D | 0.487799743 | 0.903757389 | 1.1422421 | down | mRNA |
| NM_172240 | POC1B | 0.684011511 | 0.960111041 | 1.0595691 | down | mRNA |
| NM_172313 | CSF3R | 0.924021691 | 0.995456159 | 1.0367885 | down | mRNA |
| NM_172315 | MEIS2 | 0.673446354 | 0.959531882 | 1.1059354 | down | mRNA |
| NM_172316 | MEIS2 | 0.399647608 | 0.864061588 | 1.2022467 | down | mRNA |
| NM_172349 | NSD1 | 0.202383016 | 0.691421917 | 1.1906558 | down | mRNA |
| NM_172350 | CD46 | 0.544179839 | 0.923577434 | 1.068722 | down | mRNA |
| NM_172351 | CD46 | 0.914200312 | 0.995342392 | 1.0092363 | down | mRNA |
| NM_172352 | CD46 | 0.444416616 | 0.885176198 | 1.0922044 | down | mRNA |
| NM_172353 | CD46 | 0.416911783 | 0.870739638 | 1.0834807 | down | mRNA |
| NM_172366 | FBXO16 | 0.58310903 | 0.934269626 | 1.0731033 | down | mRNA |
| NM_173039 | AQP11 | 0.673005526 | 0.959531882 | 1.0506305 | down | mRNA |
| NM_173073 | SLC35C2 | 0.235203286 | 0.73249698 | 1.253952 | down | mRNA |
| NM_173082 | SHPRH | 0.101321518 | 0.529124784 | 1.4852398 | down | mRNA |
| NM_173083 | LIN9 | 0.345155045 | 0.832533915 | 1.5438489 | down | mRNA |
| NM_173084 | TRIM59 | 0.028186714 | 0.326447555 | 1.7094329 | down | mRNA |
| NM_173156 | SMG7 | 0.620182117 | 0.947444797 | 1.0689212 | down | mRNA |
| NM_173157 | NR4A1 | 0.005235789 | 0.165444121 | 1.9135267 | down | mRNA |
| NM_173163 | NFATC3 | 0.066687393 | 0.450526863 | 1.2584283 | down | mRNA |
| NM_173165 | NFATC3 | 2.14688E-05 | 0.018978458 | 1.836928 | down | mRNA |
| NM_173177 | C1D | 0.184151716 | 0.667687419 | 1.0898803 | down | mRNA |
| NM_173191 | KCNIP2 | 0.966771105 | 0.99793927 | 1.0040896 | down | mRNA |
| NM_173192 | KCNIP2 | 0.951958178 | 0.996710991 | 1.0058568 | down | mRNA |
| NM_173203 | IL37 | 0.804221198 | 0.98118927 | 1.0892604 | down | mRNA |
| NM_173206 | PIAS2 | 0.483886176 | 0.903204457 | 1.0701186 | down | mRNA |
| NM_173341 | PHF7 | 0.117219001 | 0.564147103 | 1.2190994 | down | mRNA |
| NM_173348 | FAM149B1 | 0.67267555 | 0.959531882 | 1.0884025 | down | mRNA |
| NM_173359 | EIF4E3 | 0.937457089 | 0.996710991 | 1.0173466 | down | mRNA |
| NM_173362 | RFESD | 0.401304391 | 0.864768873 | 1.2185704 | down | mRNA |
| NM_173452 | FCN3 | 0.517724728 | 0.915684599 | 1.1506493 | down | mRNA |
| NM_173460 | DEFB119 | 0.936052685 | 0.996710991 | 1.0125341 | down | mRNA |
| NM_173463 | CCDC149 | 0.333567935 | 0.823670543 | 1.2361781 | down | mRNA |
| NM_173464 | L3MBTL4 | 0.957801848 | 0.996933183 | 1.0101089 | down | mRNA |
| NM_173465 | COL23A1 | 0.344807835 | 0.8321226 | 1.5244752 | down | mRNA |
| NM_173467 | MCAT | 0.066120763 | 0.449693785 | 1.2078982 | down | mRNA |
| NM_173468 | MOB1B | 0.076190253 | 0.474456427 | 1.0886506 | down | mRNA |
| NM_173469 | UBE2Q2 | 0.025599843 | 0.314309189 | 1.147816 | down | mRNA |
| NM_173474 | NTAN1 | 0.546905129 | 0.924803608 | 1.0403181 | down | mRNA |
| NM_173483 | CYP4F22 | 0.81427698 | 0.98120545 | 1.038862 | down | mRNA |
| NM_173487 | C4orf33 | 0.55232294 | 0.926337111 | 1.4178228 | down | mRNA |
| NM_173491 | LSM11 | 0.011403393 | 0.218702965 | 1.4387624 | down | mRNA |
| NM_173497 | HECTD2 | 0.319487817 | 0.813605498 | 1.1116364 | down | mRNA |
| NM_173500 | TTBK2 | 0.831104981 | 0.985710869 | 1.0249534 | down | mRNA |
| NM_173502 | PRSS36 | 0.381272703 | 0.853514109 | 1.2335997 | down | mRNA |
| NM_173511 | FAM117B | 0.618559827 | 0.946956498 | 1.2814158 | down | mRNA |
| NM_173514 | SLC38A9 | 0.474850923 | 0.899615074 | 1.1299896 | down | mRNA |
| NM_173515 | CNKSR3 | 0.898230848 | 0.994360027 | 1.026306 | down | mRNA |
| NM_173517 | VKORC1L1 | 0.035129104 | 0.352396712 | 1.1701768 | down | mRNA |
| NM_173526 | FAM71D | 0.813727874 | 0.98120545 | 1.0165811 | down | mRNA |
| NM_173529 | C18orf54 | 0.528794772 | 0.918927803 | 1.3881701 | down | mRNA |
| NM_173530 | ZNF610 | 0.277267033 | 0.777205298 | 1.9380992 | down | mRNA |
| NM_173531 | ZNF100 | 0.192109458 | 0.678119705 | 1.1926351 | down | mRNA |
| NM_173536 | GABRG1 | 0.770107078 | 0.975286259 | 1.0269945 | down | mRNA |
| NM_173540 | FUT11 | 0.637886864 | 0.952852353 | 1.0151597 | down | mRNA |
| NM_173543 | DZIP1L | 0.015606012 | 0.250633046 | 1.3237516 | down | mRNA |
| NM_173546 | KLHDC8B | 0.093189806 | 0.512313794 | 1.4596892 | down | mRNA |
| NM_173551 | ANKS6 | 0.024194064 | 0.304025779 | 1.5493413 | down | mRNA |
| NM_173554 | C10orf107 | 0.720678024 | 0.966191059 | 1.1257723 | down | mRNA |
| NM_173555 | TYSND1 | 0.211129647 | 0.702369538 | 1.5169478 | down | mRNA |
| NM_173561 | UNC5CL | 0.267611169 | 0.76840422 | 1.48833 | down | mRNA |
| NM_173562 | KCTD20 | 0.523610811 | 0.917239107 | 1.2198421 | down | mRNA |
| NM_173566 | PRR14L | 0.040187082 | 0.371915487 | 1.1630757 | down | mRNA |
| NM_173569 | UBN2 | 0.553678331 | 0.926337111 | 1.059093 | down | mRNA |
| NM_173574 | ZNF683 | 0.583250107 | 0.934269626 | 1.120093 | down | mRNA |
| NM_173576 | MKX | 0.401443108 | 0.864768873 | 1.1483974 | down | mRNA |
| NM_173595 | ANKRD52 | 0.519341835 | 0.916145597 | 1.0642695 | down | mRNA |
| NM_173596 | SLC39A5 | 0.178440326 | 0.659645218 | 1.4525357 | down | mRNA |
| NM_173601 | GXYLT1 | 0.927977712 | 0.995916751 | 1.0099877 | down | mRNA |
| NM_173602 | DIP2B | 0.340827212 | 0.828854153 | 1.1120175 | down | mRNA |
| NM_173620 | HEXDC | 0.493873004 | 0.906630188 | 1.1490041 | down | mRNA |
| NM_173623 | TTLL6 | 0.94439092 | 0.996710991 | 1.0032759 | down | mRNA |
| NM_173627 | ENDOV | 0.183997882 | 0.667566906 | 1.2628595 | down | mRNA |
| NM_173637 | SLC25A41 | 0.285140773 | 0.781963593 | 1.2069588 | down | mRNA |
| NM_173647 | RNF149 | 0.943381188 | 0.996710991 | 1.0195727 | down | mRNA |
| NM_173650 | DNAJC5G | 0.337479791 | 0.827187565 | 1.1971898 | down | mRNA |
| NM_173654 | EOGT | 0.65556738 | 0.957222998 | 1.1210815 | down | mRNA |
| NM_173659 | RPUSD3 | 0.045787097 | 0.391180343 | 1.2318004 | down | mRNA |
| NM_173660 | DOK7 | 0.011715153 | 0.220344574 | 1.2602781 | down | mRNA |
| NM_173664 | ARL10 | 0.130496464 | 0.58480364 | 1.6967171 | down | mRNA |
| NM_173665 | KIAA0825 | 0.026929587 | 0.319244809 | 1.2536115 | down | mRNA |
| NM_173674 | DCBLD1 | 0.72420472 | 0.966191059 | 1.0334013 | down | mRNA |
| NM_173683 | XKR6 | 0.705598542 | 0.964841587 | 1.0596862 | down | mRNA |
| NM_173689 | CRB2 | 0.62417365 | 0.948341211 | 1.0624304 | down | mRNA |
| NM_173791 | PDZD8 | 0.01638381 | 0.255922225 | 1.3796172 | down | mRNA |
| NM_173794 | FUNDC1 | 0.066655356 | 0.450526863 | 1.0999785 | down | mRNA |
| NM_173807 | C1orf101 | 0.453062065 | 0.889563297 | 1.2076063 | down | mRNA |
| NM_173808 | NEGR1 | 0.30947316 | 0.803961878 | 1.2231574 | down | mRNA |
| NM_173810 | TTC9C | 0.308157206 | 0.803057205 | 1.1871797 | down | mRNA |
| NM_173811 | HARBI1 | 0.000785761 | 0.082351036 | 1.6629015 | down | mRNA |
| NM_173815 | CES4A | 0.798215669 | 0.980749101 | 1.0303491 | down | mRNA |
| NM_173821 | CXXC11 | 0.041675642 | 0.377943419 | 1.2242632 | down | mRNA |
| NM_173822 | FAM126B | 0.213608945 | 0.706194645 | 1.1910602 | down | mRNA |
| NM_173824 | C3orf38 | 0.032114003 | 0.3425639 | 1.1911312 | down | mRNA |
| NM_173826 | C3orf23 | 0.917140369 | 0.995349793 | 1.0140665 | down | mRNA |
| NM_173827 | COX18 | 0.991730532 | 0.999548897 | 1.0011685 | down | mRNA |
| NM_173832 | ZFP41 | 0.827598956 | 0.984999238 | 1.0786747 | down | mRNA |
| NM_173834 | YIPF6 | 0.527403474 | 0.918472585 | 1.0422824 | down | mRNA |
| NM_173844 | MALT1 | 0.034091501 | 0.349898235 | 1.2322367 | down | mRNA |
| NM_173854 | SLC41A1 | 0.030648443 | 0.338914937 | 1.2003043 | down | mRNA |
| NM_173872 | CLCN3 | 0.928847189 | 0.99632398 | 1.0111756 | down | mRNA |
| NM_174869 | IDH3G | 0.999020702 | 0.999897336 | 1.0001816 | down | mRNA |
| NM_174871 | PSMD12 | 0.131672912 | 0.586842099 | 1.1043019 | down | mRNA |
| NM_174892 | CD300LB | 0.294160221 | 0.791146775 | 1.1175686 | down | mRNA |
| NM_174907 | PPP4R2 | 0.910240579 | 0.995342392 | 1.012352 | down | mRNA |
| NM_174916 | UBR1 | 0.527954874 | 0.918844141 | 1.0551288 | down | mRNA |
| NM_174919 | ARHGAP27 | 0.609106786 | 0.943053241 | 1.1085342 | down | mRNA |
| NM_174921 | C4orf34 | 0.893058558 | 0.993978977 | 1.0138974 | down | mRNA |
| NM_174922 | ADCK5 | 0.097779164 | 0.520535504 | 1.1397158 | down | mRNA |
| NM_174923 | CCDC107 | 0.472199453 | 0.899115872 | 1.068628 | down | mRNA |
| NM_174931 | CCDC75 | 0.12613171 | 0.578504384 | 1.4072944 | down | mRNA |
| NM_174933 | PHYHD1 | 0.580698948 | 0.93393768 | 1.0325865 | down | mRNA |
| NM_174934 | SCN4B | 0.345792845 | 0.832819251 | 1.0335459 | down | mRNA |
| NM_174940 | TMEM80 | 0.331425214 | 0.821973342 | 1.2753151 | down | mRNA |
| NM_174943 | C14orf178 | 0.082171297 | 0.489369308 | 1.0822417 | down | mRNA |
| NM_174945 | ZNF575 | 0.414968236 | 0.870239791 | 1.1140894 | down | mRNA |
| NM_174957 | ATP2A3 | 0.830387369 | 0.985549562 | 1.0703289 | down | mRNA |
| NM_174965 | ST3GAL3 | 0.262697071 | 0.763125731 | 1.0620523 | down | mRNA |
| NM_174967 | ST3GAL3 | 0.651923424 | 0.956204521 | 1.0407447 | down | mRNA |
| NM_174968 | ST3GAL3 | 0.679118466 | 0.959969193 | 1.0604632 | down | mRNA |
| NM_174970 | ST3GAL3 | 0.534616505 | 0.920859266 | 1.0467795 | down | mRNA |
| NM_175057 | TAAR9 | 0.738526881 | 0.969444674 | 1.0474411 | down | mRNA |
| NM_175061 | JAZF1 | 0.667637551 | 0.958354006 | 1.0594141 | down | mRNA |
| NM_175062 | RASGEF1C | 0.811413842 | 0.98118927 | 1.0336559 | down | mRNA |
| NM_175063 | EMC10 | 0.658498662 | 0.958354006 | 1.0609491 | down | mRNA |
| NM_175069 | APTX | 0.371394029 | 0.846675738 | 1.0930103 | down | mRNA |
| NM_175073 | APTX | 0.033771879 | 0.348587681 | 1.1737286 | down | mRNA |
| NM_175075 | C8orf42 | 0.559505077 | 0.927203296 | 1.3249176 | down | mRNA |
| NM_175078 | KRT77 | 0.60393623 | 0.941514447 | 1.0646599 | down | mRNA |
| NM_175085 | GART | 0.760909535 | 0.972945894 | 1.015616 | down | mRNA |
| NM_175607 | CNTN4 | 0.402871896 | 0.864786348 | 1.2102813 | down | mRNA |
| NM_175609 | ARFGAP1 | 0.523099392 | 0.917239107 | 1.1222602 | down | mRNA |
| NM_175610 | TJP1 | 0.117873457 | 0.564437853 | 1.0781202 | down | mRNA |
| NM_175611 | GRIK1 | 0.122164852 | 0.57455499 | 1.1533074 | down | mRNA |
| NM_175614 | NDUFA11 | 0.687598116 | 0.960243201 | 1.0276297 | down | mRNA |
| NM_175634 | RUNX1T1 | 0.001870784 | 0.105670067 | 1.3943357 | down | mRNA |
| NM_175635 | RUNX1T1 | 0.179078113 | 0.661041429 | 1.2400508 | down | mRNA |
| NM_175698 | SSX2 | 0.993814534 | 0.999872237 | 1.0016842 | down | mRNA |
| NM_175709 | CBX7 | 0.487936563 | 0.903757389 | 1.0957932 | down | mRNA |
| NM_175711 | SSX3 | 0.783201993 | 0.977275912 | 1.0511691 | down | mRNA |
| NM_175722 | TPO | 0.984863804 | 0.998971807 | 1.007794 | down | mRNA |
| NM_175734 | C17orf74 | 0.620072416 | 0.947444797 | 1.091021 | down | mRNA |
| NM_175738 | RAB37 | 0.826414333 | 0.984423205 | 1.023801 | down | mRNA |
| NM_175768 | GRIK2 | 0.180046944 | 0.662747609 | 1.4451023 | down | mRNA |
| NM_175854 | PAN3 | 0.223485486 | 0.717723408 | 1.1909958 | down | mRNA |
| NM_175861 | TMTC1 | 0.236246913 | 0.734460585 | 1.3326708 | down | mRNA |
| NM_175868 | MAGEA6 | 0.901749323 | 0.994906068 | 1.0072962 | down | mRNA |
| NM_175875 | SIX5 | 0.90100428 | 0.994789506 | 1.0243186 | down | mRNA |
| NM_175884 | CCDC71L | 0.022906806 | 0.298107041 | 1.2789099 | down | mRNA |
| NM_175885 | FAM181B | 0.381000996 | 0.853514109 | 1.0882296 | down | mRNA |
| NM_175886 | PRPS1L1 | 0.083048589 | 0.491579514 | 1.459659 | down | mRNA |
| NM_175900 | C16orf54 | 0.305275827 | 0.801056074 | 1.2406224 | down | mRNA |
| NM_175902 | C17orf101 | 0.605349074 | 0.94174007 | 1.0769157 | down | mRNA |
| NM_175910 | ZNF493 | 0.002580836 | 0.120730042 | 1.4471361 | down | mRNA |
| NM_175918 | CRIPAK | 0.209564668 | 0.700367256 | 1.1203626 | down | mRNA |
| NM_175920 | LNPEP | 0.40167604 | 0.864768873 | 1.1845657 | down | mRNA |
| NM_175921 | C5orf51 | 0.098680035 | 0.52242281 | 1.3418384 | down | mRNA |
| NM_175931 | CBFA2T3 | 0.734696352 | 0.967777568 | 1.0422623 | down | mRNA |
| NM_176072 | P2RY2 | 0.190919999 | 0.676402188 | 1.1967623 | down | mRNA |
| NM_176677 | NHLRC4 | 0.642943383 | 0.954522001 | 1.1575677 | down | mRNA |
| NM_176791 | GTSF1L | 0.660300427 | 0.958354006 | 1.0630875 | down | mRNA |
| NM_176795 | HRAS | 0.027264584 | 0.3212251 | 1.0746824 | down | mRNA |
| NM_176805 | MRPS11 | 0.12030461 | 0.571550658 | 1.1800805 | down | mRNA |
| NM_176806 | MOCS2 | 0.642445218 | 0.954350564 | 1.0659047 | down | mRNA |
| NM_176814 | ZNF800 | 0.88831719 | 0.993440915 | 1.0127012 | down | mRNA |
| NM_176816 | CCDC125 | 0.224371351 | 0.718865159 | 1.1397562 | down | mRNA |
| NM_176819 | CXorf36 | 0.191527231 | 0.67724029 | 1.2286819 | down | mRNA |
| NM_176821 | NLRP10 | 0.274783431 | 0.774774195 | 1.4950965 | down | mRNA |
| NM_176824 | BBS7 | 0.24188623 | 0.740332805 | 1.0968665 | down | mRNA |
| NM_176863 | PSME3 | 0.866457525 | 0.989481868 | 1.01818 | down | mRNA |
| NM_176866 | PPA2 | 0.6493145 | 0.95550188 | 1.0315533 | down | mRNA |
| NM_176867 | PPA2 | 0.361341493 | 0.842165256 | 1.0414515 | down | mRNA |
| NM_176869 | PPA2 | 0.925276832 | 0.995465766 | 1.0067643 | down | mRNA |
| NM_176875 | CCKBR | 0.357418555 | 0.839643099 | 18.9409467 | down | mRNA |
| NM_176880 | NR2C2AP | 0.686732897 | 0.960205549 | 1.1111373 | down | mRNA |
| NM_176882 | TAS2R40 | 0.712967864 | 0.966110889 | 1.1127303 | down | mRNA |
| NM_176888 | TAS2R19 | 0.011131035 | 0.216363484 | 1.2894915 | down | mRNA |
| NM_176889 | TAS2R20 | 0.052130504 | 0.411765599 | 1.6737903 | down | mRNA |
| NM_177401 | MIDN | 0.006765164 | 0.183552613 | 1.8437158 | down | mRNA |
| NM_177417 | KLC3 | 0.04213743 | 0.379087707 | 1.3240133 | down | mRNA |
| NM_177422 | EIF2C3 | 0.406010661 | 0.866541972 | 1.1028455 | down | mRNA |
| NM_177423 | PPFIA1 | 0.611784366 | 0.943913476 | 1.0408511 | down | mRNA |
| NM_177439 | FTSJ1 | 0.437065049 | 0.882217212 | 1.1338085 | down | mRNA |
| NM_177452 | TRAPPC6B | 0.327856101 | 0.820730147 | 1.0711145 | down | mRNA |
| NM_177454 | FAM171B | 0.761333158 | 0.973219662 | 1.1536718 | down | mRNA |
| NM_177537 | MAGED4B | 0.921475575 | 0.995349793 | 1.0143817 | down | mRNA |
| NM_177538 | CYP20A1 | 0.0623172 | 0.439381733 | 1.3012169 | down | mRNA |
| NM_177540 | TAS1R1 | 0.815627228 | 0.981459009 | 1.1366561 | down | mRNA |
| NM_177542 | SNRPD2 | 0.405339679 | 0.866165103 | 1.061242 | down | mRNA |
| NM_177552 | SULT1A3 | 0.343875367 | 0.831629639 | 1.062493 | down | mRNA |
| NM_177556 | TRO | 0.792411176 | 0.978996327 | 1.0243632 | down | mRNA |
| NM_177560 | CSNK2A1 | 0.675888255 | 0.959531882 | 1.0461016 | down | mRNA |
| NM_177937 | GOLM1 | 0.064781324 | 0.444527127 | 1.204295 | down | mRNA |
| NM_177939 | P4HTM | 0.630104705 | 0.950410442 | 1.0473619 | down | mRNA |
| NM_177947 | ARMCX3 | 0.453884465 | 0.890082845 | 1.1444616 | down | mRNA |
| NM_177951 | PPM1A | 0.269894839 | 0.770082629 | 1.1326744 | down | mRNA |
| NM_177952 | PPM1A | 0.31477724 | 0.80982549 | 1.1770087 | down | mRNA |
| NM_177966 | PDE12 | 0.043037706 | 0.383286307 | 1.2323883 | down | mRNA |
| NM_177968 | PPM1B | 0.247368077 | 0.745551102 | 1.2256281 | down | mRNA |
| NM_177969 | PPM1B | 0.432376606 | 0.880428759 | 1.1549862 | down | mRNA |
| NM_177974 | CASC4 | 0.203312278 | 0.692876095 | 1.0641728 | down | mRNA |
| NM_177976 | ARL6 | 0.343228705 | 0.831521302 | 1.1162247 | down | mRNA |
| NM_177983 | PPM1G | 0.327585554 | 0.820456945 | 1.1035766 | down | mRNA |
| NM_177988 | MRPL47 | 0.72290649 | 0.966191059 | 1.0572271 | down | mRNA |
| NM_177999 | ASB6 | 0.458451524 | 0.892667725 | 1.068625 | down | mRNA |
| NM_178000 | PPP2R4 | 0.160501239 | 0.632016252 | 1.1639989 | down | mRNA |
| NM_178012 | TUBB2B | 0.5533851 | 0.926337111 | 1.1792265 | down | mRNA |
| NM_178014 | TUBB | 0.601611193 | 0.941064685 | 1.0624198 | down | mRNA |
| NM_178040 | ERC1 | 0.288569264 | 0.784514546 | 1.1756733 | down | mRNA |
| NM_178042 | ACTL6A | 0.286308645 | 0.782895119 | 1.1240278 | down | mRNA |
| NM_178120 | DLX1 | 0.086414421 | 0.49936346 | 1.5877421 | down | mRNA |
| NM_178122 | STPG1 | 0.830110443 | 0.985549562 | 1.022108 | down | mRNA |
| NM_178123 | SESTD1 | 0.487842441 | 0.903757389 | 1.1118391 | down | mRNA |
| NM_178124 | CXorf40A | 0.817629461 | 0.981727075 | 1.0251486 | down | mRNA |
| NM_178134 | CYP4Z1 | 0.991024542 | 0.999376923 | 1.0019984 | down | mRNA |
| NM_178136 | POLDIP3 | 0.135875153 | 0.594011904 | 1.1139276 | down | mRNA |
| NM_178138 | LHX3 | 0.543711996 | 0.923577434 | 1.122351 | down | mRNA |
| NM_178140 | PDZD2 | 0.319468257 | 0.813605498 | 1.2130363 | down | mRNA |
| NM_178148 | SLC35B2 | 0.758598122 | 0.972072638 | 1.0154667 | down | mRNA |
| NM_178152 | DCX | 0.958207615 | 0.996933183 | 1.0101401 | down | mRNA |
| NM_178176 | MOGAT3 | 0.522937938 | 0.917239107 | 1.0606017 | down | mRNA |
| NM_178190 | ATPIF1 | 0.436681525 | 0.882102748 | 1.1139198 | down | mRNA |
| NM_178221 | ATG4C | 0.06130464 | 0.437893235 | 1.1671419 | down | mRNA |
| NM_178230 | PPIAL4A | 0.745920221 | 0.970394407 | 1.0240235 | down | mRNA |
| NM_178232 | HAPLN3 | 0.322102154 | 0.815372756 | 1.2257226 | down | mRNA |
| NM_178233 | OTOP3 | 0.422883674 | 0.873989281 | 1.5825856 | down | mRNA |
| NM_178234 | TUSC3 | 0.618424441 | 0.946956498 | 1.1066339 | down | mRNA |
| NM_178237 | EXOC1 | 0.119022769 | 0.568068298 | 1.1909405 | down | mRNA |
| NM_178238 | PILRB | 0.643560436 | 0.954605197 | 1.0278787 | down | mRNA |
| NM_178270 | ATG4A | 0.026751597 | 0.318484367 | 1.3448034 | down | mRNA |
| NM_178272 | PILRA | 0.521620223 | 0.916644975 | 1.0438475 | down | mRNA |
| NM_178276 | SERINC5 | 0.280133863 | 0.778736902 | 1.1057198 | down | mRNA |
| NM_178314 | RILPL1 | 0.006764155 | 0.183552613 | 1.3158669 | down | mRNA |
| NM_178326 | ATG4B | 0.113940423 | 0.557835051 | 1.1017283 | down | mRNA |
| NM_178353 | LCE1E | 0.851268293 | 0.987678166 | 1.0518045 | down | mRNA |
| NM_178422 | PAQR7 | 0.267968532 | 0.768778917 | 1.3887211 | down | mRNA |
| NM_178427 | ARNT | 0.134915369 | 0.59246205 | 1.1108724 | down | mRNA |
| NM_178432 | CDK20 | 0.0755653 | 0.471986158 | 1.2815286 | down | mRNA |
| NM_178439 | GMCL1 | 0.048652194 | 0.400568526 | 1.1992018 | down | mRNA |
| NM_178448 | SAPCD2 | 0.57199734 | 0.933338778 | 1.0372195 | down | mRNA |
| NM_178454 | DRAM2 | 0.812556978 | 0.98118927 | 1.0710848 | down | mRNA |
| NM_178469 | LCN8 | 0.333106438 | 0.82335721 | 1.1398777 | down | mRNA |
| NM_178496 | MB21D2 | 0.029682731 | 0.334953885 | 1.8464043 | down | mRNA |
| NM_178509 | STXBP4 | 0.466568536 | 0.89599965 | 1.0486343 | down | mRNA |
| NM_178547 | ZBTB8OS | 0.340598914 | 0.828777111 | 1.1251881 | down | mRNA |
| NM_178548 | TFAP2E | 0.522075208 | 0.916999248 | 1.1552953 | down | mRNA |
| NM_178558 | ZNF680 | 0.002400827 | 0.117191396 | 1.254835 | down | mRNA |
| NM_178561 | CTAGE6P | 0.974416913 | 0.998501532 | 1.0032609 | down | mRNA |
| NM_178565 | RSPO2 | 0.500412136 | 0.909680616 | 1.369386 | down | mRNA |
| NM_178566 | ZDHHC21 | 0.266508978 | 0.767081052 | 1.1300407 | down | mRNA |
| NM_178571 | TBC1D26 | 0.986214974 | 0.998971807 | 1.00296 | down | mRNA |
| NM_178581 | HM13 | 0.140888527 | 0.601037671 | 1.1765315 | down | mRNA |
| NM_178586 | PPP2R5C | 0.414723182 | 0.870239791 | 1.0912815 | down | mRNA |
| NM_178587 | PPP2R5C | 0.690819099 | 0.961105466 | 1.0664108 | down | mRNA |
| NM_178812 | MTDH | 0.012045341 | 0.222675671 | 1.195786 | down | mRNA |
| NM_178819 | AGPAT6 | 0.221105576 | 0.716640124 | 1.1853694 | down | mRNA |
| NM_178821 | WDR69 | 0.355430429 | 0.838369858 | 1.3086092 | down | mRNA |
| NM_178824 | WDR49 | 0.812078934 | 0.98118927 | 1.0210378 | down | mRNA |
| NM_178828 | FAM75E1 | 0.3152575 | 0.810240865 | 1.0229876 | down | mRNA |
| NM_178831 | GATS | 0.367618752 | 0.845181673 | 1.0558302 | down | mRNA |
| NM_178835 | ZNF827 | 0.109512278 | 0.547615256 | 2.3362735 | down | mRNA |
| NM_178849 | HNF4A | 0.81069286 | 0.98118927 | 1.0375457 | down | mRNA |
| NM_178862 | STT3B | 0.342622479 | 0.831288811 | 1.2050249 | down | mRNA |
| NM_178865 | SERINC2 | 0.961600367 | 0.99793927 | 1.0036294 | down | mRNA |
| NM_180699 | SNRNP35 | 0.041602776 | 0.377938997 | 1.2522635 | down | mRNA |
| NM_180976 | PPP2R5D | 0.982793483 | 0.998971807 | 1.0068789 | down | mRNA |
| NM_180982 | MRPL52 | 0.74833173 | 0.970725371 | 1.0445547 | down | mRNA |
| NM_180989 | GPR180 | 0.00222261 | 0.111580485 | 1.3803426 | down | mRNA |
| NM_181050 | AXIN1 | 0.305012712 | 0.800815917 | 1.1149578 | down | mRNA |
| NM_181054 | HIF1A | 0.258834207 | 0.759358563 | 1.1720419 | down | mRNA |
| NM_181077 | GOLGA8A | 0.978745242 | 0.998661138 | 1.005818 | down | mRNA |
| NM_181268 | CMTM1 | 0.783533798 | 0.977275912 | 1.0576043 | down | mRNA |
| NM_181272 | CMTM1 | 0.751115399 | 0.970725371 | 1.057975 | down | mRNA |
| NM_181291 | WDR20 | 0.057693957 | 0.427646201 | 1.268492 | down | mRNA |
| NM_181307 | MRPL52 | 0.682422215 | 0.960111041 | 1.0605463 | down | mRNA |
| NM_181308 | WDR20 | 0.170375222 | 0.647247575 | 1.2334609 | down | mRNA |
| NM_181309 | IL22RA2 | 0.798863059 | 0.980813744 | 1.1011414 | down | mRNA |
| NM_181311 | TAZ | 0.194994539 | 0.682660006 | 1.104738 | down | mRNA |
| NM_181312 | TAZ | 0.998937461 | 0.999897336 | 1.0001767 | down | mRNA |
| NM_181313 | TAZ | 0.983702192 | 0.998971807 | 1.0032625 | down | mRNA |
| NM_181334 | PRR5-ARHGAP8 | 0.904451314 | 0.99508468 | 1.0148843 | down | mRNA |
| NM_181336 | LEMD2 | 0.23492586 | 0.732404233 | 1.109814 | down | mRNA |
| NM_181341 | DCAF4 | 0.000227925 | 0.050371507 | 1.5508942 | down | mRNA |
| NM_181342 | FKBP7 | 0.58707835 | 0.93579682 | 1.0421026 | down | mRNA |
| NM_181351 | NCAM1 | 0.6677322 | 0.958354006 | 1.0333065 | down | mRNA |
| NM_181353 | ID1 | 0.000996093 | 0.085705167 | 2.9324046 | down | mRNA |
| NM_181354 | OXR1 | 0.405797694 | 0.866541972 | 1.076115 | down | mRNA |
| NM_181356 | SUPT3H | 0.96297307 | 0.99793927 | 1.0125038 | down | mRNA |
| NM_181357 | DCAF11 | 0.331140802 | 0.821853462 | 1.0540275 | down | mRNA |
| NM_181358 | HIPK1 | 0.03543743 | 0.353725338 | 1.1797818 | down | mRNA |
| NM_181425 | FXN | 0.003580723 | 0.139262737 | 1.1804454 | down | mRNA |
| NM_181443 | BTBD3 | 0.430824029 | 0.879381002 | 1.3476828 | down | mRNA |
| NM_181455 | MRPL55 | 0.098352563 | 0.521707362 | 1.1033037 | down | mRNA |
| NM_181466 | EIF6 | 0.608358108 | 0.942985214 | 1.076508 | down | mRNA |
| NM_181489 | ZNF445 | 0.934673806 | 0.996710991 | 1.028604 | down | mRNA |
| NM_181492 | TCF20 | 0.162913285 | 0.635098861 | 1.2407139 | down | mRNA |
| NM_181493 | ITPA | 0.624465935 | 0.948341211 | 1.0145939 | down | mRNA |
| NM_181501 | ITGA1 | 0.626909769 | 0.949203235 | 1.0905107 | down | mRNA |
| NM_181503 | EXOSC8 | 0.688530639 | 0.960626155 | 1.0900112 | down | mRNA |
| NM_181505 | PPP1R1B | 0.574982843 | 0.933656777 | 1.0768649 | down | mRNA |
| NM_181508 | HPS5 | 0.515432066 | 0.914319064 | 1.0319019 | down | mRNA |
| NM_181514 | MRPL21 | 0.641863711 | 0.954332328 | 1.0234517 | down | mRNA |
| NM_181515 | MRPL21 | 0.721417812 | 0.966191059 | 1.0523984 | down | mRNA |
| NM_181521 | CMTM4 | 0.833540039 | 0.986434891 | 1.0229928 | down | mRNA |
| NM_181526 | MYL9 | 0.69447543 | 0.962054537 | 1.1036711 | down | mRNA |
| NM_181528 | NAA20 | 0.405379669 | 0.866165103 | 1.0610324 | down | mRNA |
| NM_181553 | CMTM3 | 0.207690014 | 0.698556218 | 1.1307008 | down | mRNA |
| NM_181571 | CREM | 0.604836027 | 0.94174007 | 1.0229187 | down | mRNA |
| NM_181573 | RFC4 | 0.621968744 | 0.948047106 | 1.257316 | down | mRNA |
| NM_181575 | AUP1 | 0.814426977 | 0.981277402 | 1.0232868 | down | mRNA |
| NM_181578 | RFC5 | 0.296704817 | 0.793167205 | 1.2049336 | down | mRNA |
| NM_181581 | DUS4L | 0.33110723 | 0.821853462 | 1.1668985 | down | mRNA |
| NM_181605 | KRTAP6-3 | 0.808351858 | 0.98118927 | 1.0214956 | down | mRNA |
| NM_181607 | KRTAP19-1 | 0.496529973 | 0.908599354 | 1.0580156 | down | mRNA |
| NM_181614 | KRTAP19-7 | 0.75375818 | 0.970761469 | 1.0410937 | down | mRNA |
| NM_181616 | KRTAP20-2 | 0.929309322 | 0.996504253 | 1.0054272 | down | mRNA |
| NM_181623 | KRTAP15-1 | 0.463360053 | 0.894989844 | 1.6309273 | down | mRNA |
| NM_181640 | CKLF | 0.661209814 | 0.958354006 | 1.045974 | down | mRNA |
| NM_181644 | MFSD4 | 0.280700416 | 0.779126326 | 1.0629713 | down | mRNA |
| NM_181646 | ZNF804B | 0.383183002 | 0.853514109 | 1.1553448 | down | mRNA |
| NM_181651 | PRDX5 | 0.679479203 | 0.959969193 | 1.0653398 | down | mRNA |
| NM_181652 | PRDX5 | 0.880106154 | 0.992197209 | 1.0238729 | down | mRNA |
| NM_181654 | CPLX4 | 0.053869007 | 0.416926015 | 1.7462611 | down | mRNA |
| NM_181659 | NCOA3 | 0.122388238 | 0.57455499 | 1.1334226 | down | mRNA |
| NM_181661 | VPS13B | 0.94694923 | 0.996710991 | 1.0146279 | down | mRNA |
| NM_181670 | ANKS1B | 0.779815045 | 0.976938981 | 1.0422879 | down | mRNA |
| NM_181671 | PITPNC1 | 0.94981289 | 0.996710991 | 1.0304101 | down | mRNA |
| NM_181689 | NNAT | 0.401623834 | 0.864768873 | 1.0416957 | down | mRNA |
| NM_181690 | AKT3 | 0.8648137 | 0.989401142 | 1.0195409 | down | mRNA |
| NM_181696 | PRDX1 | 0.865047391 | 0.989401142 | 1.0307847 | down | mRNA |
| NM_181700 | PPP2R1B | 0.783663702 | 0.977275912 | 1.0519617 | down | mRNA |
| NM_181702 | GEM | 0.838272665 | 0.986897498 | 1.033477 | down | mRNA |
| NM_181703 | GJA5 | 0.64877449 | 0.95550188 | 1.0924661 | down | mRNA |
| NM_181705 | LYRM7 | 0.216378283 | 0.711073448 | 1.1935423 | down | mRNA |
| NM_181706 | DNAJC24 | 0.538472693 | 0.923577434 | 1.0720015 | down | mRNA |
| NM_181708 | BCDIN3D | 0.023314778 | 0.300022844 | 1.1229936 | down | mRNA |
| NM_181709 | FAM101A | 0.664528351 | 0.958354006 | 1.0595772 | down | mRNA |
| NM_181713 | UBXN2A | 0.024009152 | 0.303389731 | 1.1910489 | down | mRNA |
| NM_181715 | CRTC2 | 0.984935655 | 0.998971807 | 1.0015708 | down | mRNA |
| NM_181716 | CENPV | 0.633918576 | 0.951174475 | 1.1711283 | down | mRNA |
| NM_181718 | ASPHD1 | 0.390844786 | 0.858920947 | 1.0865835 | down | mRNA |
| NM_181721 | FOXR1 | 0.071576922 | 0.462000629 | 1.314598 | down | mRNA |
| NM_181725 | METTL2A | 0.322748352 | 0.815766149 | 1.0636027 | down | mRNA |
| NM_181733 | COG5 | 0.787278463 | 0.977479172 | 1.0262182 | down | mRNA |
| NM_181747 | ORC5 | 0.078251013 | 0.480303439 | 1.3306453 | down | mRNA |
| NM_181762 | UBE2A | 0.944822363 | 0.996710991 | 1.0046234 | down | mRNA |
| NM_181777 | UBE2A | 0.46986033 | 0.897941557 | 1.0538415 | down | mRNA |
| NM_181782 | NCOA7 | 0.459034095 | 0.892904132 | 1.076734 | down | mRNA |
| NM_181784 | SPRED2 | 0.069254334 | 0.456727607 | 1.1472541 | down | mRNA |
| NM_181785 | SLC46A3 | 0.801006859 | 0.98118927 | 1.1051226 | down | mRNA |
| NM_181787 | DPY19L4 | 0.402150789 | 0.864786348 | 1.126242 | down | mRNA |
| NM_181799 | UBE2C | 0.737315603 | 0.968918099 | 1.261842 | down | mRNA |
| NM_181800 | UBE2C | 0.69416456 | 0.961951598 | 1.2596401 | down | mRNA |
| NM_181802 | UBE2C | 0.703499869 | 0.963625105 | 1.2579656 | down | mRNA |
| NM_181803 | UBE2C | 0.73660012 | 0.968717225 | 1.2319568 | down | mRNA |
| NM_181805 | PKIG | 0.605488071 | 0.94174007 | 1.0754539 | down | mRNA |
| NM_181806 | AASDH | 0.03575734 | 0.354907714 | 1.4621648 | down | mRNA |
| NM_181825 | NF2 | 0.276394708 | 0.776195746 | 1.0887895 | down | mRNA |
| NM_181828 | NF2 | 0.033257086 | 0.346405261 | 1.4511484 | down | mRNA |
| NM_181830 | NF2 | 0.107606699 | 0.543837782 | 1.1496521 | down | mRNA |
| NM_181833 | NF2 | 0.243483322 | 0.741870658 | 1.1033991 | down | mRNA |
| NM_181836 | TMED7 | 0.230861127 | 0.728071705 | 1.0823363 | down | mRNA |
| NM_181837 | ORC3 | 0.513808622 | 0.91350662 | 1.0855439 | down | mRNA |
| NM_181838 | UBE2D2 | 0.562960532 | 0.929041598 | 1.040662 | down | mRNA |
| NM_181843 | NUDT8 | 0.16445749 | 0.637633425 | 1.2187852 | down | mRNA |
| NM_181845 | ZNF283 | 0.002326156 | 0.114794657 | 1.7348192 | down | mRNA |
| NM_181861 | APAF1 | 0.450218535 | 0.888463871 | 1.0688268 | down | mRNA |
| NM_181864 | ACOT7 | 0.814830289 | 0.981277402 | 1.0106039 | down | mRNA |
| NM_181866 | ACOT7 | 0.904373045 | 0.99508468 | 1.009315 | down | mRNA |
| NM_181869 | APAF1 | 0.79265348 | 0.979003983 | 1.0442279 | down | mRNA |
| NM_181871 | PKLR | 0.02072244 | 0.286034385 | 1.5873779 | down | mRNA |
| NM_181873 | MTMR11 | 0.630121297 | 0.950410442 | 1.0679373 | down | mRNA |
| NM_181889 | UBE2D3 | 0.608066874 | 0.942821299 | 1.0666158 | down | mRNA |
| NM_181892 | UBE2D3 | 0.480161167 | 0.901538317 | 1.0664759 | down | mRNA |
| NM_181893 | UBE2D3 | 0.317656486 | 0.811908136 | 1.0625767 | down | mRNA |
| NM_181900 | STARD5 | 0.08422873 | 0.49409652 | 1.3851621 | down | mRNA |
| NM_181985 | LILRA5 | 0.880322491 | 0.992245156 | 1.0207996 | down | mRNA |
| NM_182472 | EPHA5 | 0.464882341 | 0.894989844 | 1.2308083 | down | mRNA |
| NM_182490 | ZNF227 | 0.495324299 | 0.907415174 | 1.0226852 | down | mRNA |
| NM_182503 | ADAT2 | 0.214293428 | 0.706979283 | 1.1857955 | down | mRNA |
| NM_182515 | ZNF714 | 0.031436658 | 0.341322176 | 1.6828731 | down | mRNA |
| NM_182520 | C22orf15 | 0.569956123 | 0.932738884 | 1.0874579 | down | mRNA |
| NM_182523 | CMC1 | 0.829785222 | 0.985549562 | 1.0302461 | down | mRNA |
| NM_182524 | ZNF595 | 0.024831736 | 0.307665355 | 1.7095041 | down | mRNA |
| NM_182529 | THAP5 | 0.053916664 | 0.416977409 | 1.234587 | down | mRNA |
| NM_182531 | ZNF778 | 0.662168304 | 0.958354006 | 1.0352435 | down | mRNA |
| NM_182533 | C1orf86 | 0.927440498 | 0.995829678 | 1.0133897 | down | mRNA |
| NM_182540 | DDX26B | 0.649684005 | 0.95550188 | 1.057827 | down | mRNA |
| NM_182541 | TMEM31 | 0.041737653 | 0.378337034 | 1.2717777 | down | mRNA |
| NM_182543 | NSUN6 | 0.334754433 | 0.824597423 | 1.2048454 | down | mRNA |
| NM_182552 | WDR27 | 0.975028702 | 0.998501532 | 1.0067384 | down | mRNA |
| NM_182559 | TMPRSS12 | 0.365328233 | 0.844953778 | 1.1737121 | down | mRNA |
| NM_182563 | C16orf79 | 0.0267497 | 0.318484367 | 1.2661676 | down | mRNA |
| NM_182565 | FAM100B | 0.952656696 | 0.996710991 | 1.0070737 | down | mRNA |
| NM_182566 | VMO1 | 0.86889046 | 0.989485876 | 1.0656765 | down | mRNA |
| NM_182578 | THEM5 | 0.926550305 | 0.99582096 | 1.0165519 | down | mRNA |
| NM_182584 | C20orf203 | 0.881672671 | 0.992766819 | 1.0121894 | down | mRNA |
| NM_182587 | UNC80 | 0.524443832 | 0.917632259 | 1.1156509 | down | mRNA |
| NM_182588 | RGPD4 | 0.809328584 | 0.98118927 | 1.0436807 | down | mRNA |
| NM_182603 | ANKRD42 | 0.505082917 | 0.910568383 | 1.1294589 | down | mRNA |
| NM_182609 | ZNF677 | 0.207497347 | 0.698556218 | 1.120693 | down | mRNA |
| NM_182625 | GEN1 | 0.787623666 | 0.977492986 | 1.0747243 | down | mRNA |
| NM_182626 | C2orf48 | 0.330374157 | 0.821797487 | 1.0670624 | down | mRNA |
| NM_182627 | WDR53 | 0.02312168 | 0.299167937 | 1.5522329 | down | mRNA |
| NM_182633 | ZNF713 | 0.007388534 | 0.190938402 | 1.5411392 | down | mRNA |
| NM_182639 | HPS1 | 0.969565927 | 0.998245244 | 1.0066186 | down | mRNA |
| NM_182640 | MRPS9 | 0.050593671 | 0.406749912 | 1.1913058 | down | mRNA |
| NM_182641 | BPTF | 0.088825794 | 0.503486489 | 1.2193469 | down | mRNA |
| NM_182645 | VGLL2 | 0.734250774 | 0.967777568 | 1.0513241 | down | mRNA |
| NM_182649 | PCNA | 0.963165928 | 0.99793927 | 1.0244681 | down | mRNA |
| NM_182679 | GPATCH4 | 0.902029425 | 0.994906068 | 1.0117838 | down | mRNA |
| NM_182681 | AMELX | 0.117345556 | 0.564147103 | 1.1471542 | down | mRNA |
| NM_182689 | EFNA4 | 0.04969534 | 0.403516637 | 1.1223325 | down | mRNA |
| NM_182690 | EFNA4 | 0.063463101 | 0.440687083 | 1.1140094 | down | mRNA |
| NM_182691 | SRPK2 | 0.930064398 | 0.996649933 | 1.0183886 | down | mRNA |
| NM_182709 | KAT5 | 0.811088043 | 0.98118927 | 1.0565782 | down | mRNA |
| NM_182710 | KAT5 | 0.985819482 | 0.998971807 | 1.0028587 | down | mRNA |
| NM_182719 | CREM | 0.827333543 | 0.984913965 | 1.0628145 | down | mRNA |
| NM_182720 | CREM | 0.718216384 | 0.966191059 | 1.022321 | down | mRNA |
| NM_182721 | CREM | 0.889880668 | 0.9937521 | 1.0206178 | down | mRNA |
| NM_182723 | CREM | 0.24520757 | 0.743589595 | 1.1319956 | down | mRNA |
| NM_182724 | CREM | 0.195444097 | 0.683365329 | 1.1366449 | down | mRNA |
| NM_182734 | PLCB1 | 0.869971651 | 0.989592579 | 1.0100063 | down | mRNA |
| NM_182739 | NDUFB6 | 0.65413455 | 0.957205183 | 1.0369117 | down | mRNA |
| NM_182742 | TXNRD1 | 0.402479309 | 0.864786348 | 1.060826 | down | mRNA |
| NM_182749 | N6AMT1 | 0.995484633 | 0.999872237 | 1.000729 | down | mRNA |
| NM_182751 | MCM10 | 0.6776861 | 0.959531882 | 1.2465629 | down | mRNA |
| NM_182752 | TPRG1L | 0.185557811 | 0.669285333 | 1.0980849 | down | mRNA |
| NM_182758 | WDR72 | 0.606165271 | 0.94174007 | 1.1526803 | down | mRNA |
| NM_182759 | FAM19A3 | 0.028194074 | 0.326447555 | 1.2676532 | down | mRNA |
| NM_182760 | SUMF1 | 0.767753328 | 0.975033576 | 1.0571929 | down | mRNA |
| NM_182765 | HECTD2 | 0.644732491 | 0.954657712 | 1.044039 | down | mRNA |
| NM_182769 | CREM | 0.37111744 | 0.846402038 | 1.0697826 | down | mRNA |
| NM_182796 | MAT2B | 0.78545828 | 0.977425166 | 1.0790625 | down | mRNA |
| NM_182797 | PLCB4 | 0.255896341 | 0.756893281 | 1.2671775 | down | mRNA |
| NM_182801 | EGFLAM | 0.555987223 | 0.926833616 | 1.281592 | down | mRNA |
| NM_182811 | PLCG1 | 0.37515221 | 0.849710156 | 1.1219348 | down | mRNA |
| NM_182826 | SCARA3 | 0.181896091 | 0.664924727 | 1.1317873 | down | mRNA |
| NM_182835 | SCFD1 | 0.23089742 | 0.728071705 | 1.1104719 | down | mRNA |
| NM_182899 | CREB5 | 0.248842073 | 0.747319103 | 1.142491 | down | mRNA |
| NM_182904 | P4HA3 | 0.343290862 | 0.831521302 | 1.1334967 | down | mRNA |
| NM_182905 | WASH1 | 0.101801978 | 0.529641203 | 1.1863938 | down | mRNA |
| NM_182907 | PRDM1 | 0.656847327 | 0.957445617 | 1.0539624 | down | mRNA |
| NM_182909 | FILIP1L | 0.968387773 | 0.998137494 | 1.0059645 | down | mRNA |
| NM_182910 | SYNE2 | 0.939043655 | 0.996710991 | 1.0178548 | down | mRNA |
| NM_182914 | SYNE2 | 0.236870869 | 0.734939495 | 1.2050881 | down | mRNA |
| NM_182915 | STEAP3 | 0.152633887 | 0.618255775 | 1.0934364 | down | mRNA |
| NM_182916 | TRNT1 | 0.626446966 | 0.949203235 | 1.1411826 | down | mRNA |
| NM_182918 | ERG | 0.512866329 | 0.913297047 | 1.2796452 | down | mRNA |
| NM_182922 | HEATR3 | 0.056156703 | 0.422065984 | 1.5179896 | down | mRNA |
| NM_182923 | KLC1 | 0.647294321 | 0.95550188 | 1.0679502 | down | mRNA |
| NM_182924 | MICALL2 | 0.878373639 | 0.991675986 | 1.0300372 | down | mRNA |
| NM_182926 | KTN1 | 0.701227586 | 0.963015537 | 1.0221427 | down | mRNA |
| NM_182935 | MOBP | 0.301058491 | 0.79596036 | 1.1742705 | down | mRNA |
| NM_182943 | PLOD2 | 0.13010515 | 0.584037049 | 1.081433 | down | mRNA |
| NM_182946 | NIN | 0.968727075 | 0.998196842 | 1.0042635 | down | mRNA |
| NM_182958 | KAT8 | 0.122568017 | 0.574983504 | 1.0687961 | down | mRNA |
| NM_182982 | GRK4 | 0.128559065 | 0.582242044 | 1.149144 | down | mRNA |
| NM_182984 | TRMT2A | 0.005853295 | 0.173990061 | 1.2576786 | down | mRNA |
| NM_183001 | SHC1 | 0.420738978 | 0.872676791 | 1.1129172 | down | mRNA |
| NM_183004 | EIF5 | 0.21113922 | 0.702369538 | 1.2627832 | down | mRNA |
| NM_183006 | DLGAP4 | 0.315616233 | 0.810346887 | 1.1157643 | down | mRNA |
| NM_183009 | KIAA1429 | 0.030437805 | 0.338175657 | 1.219381 | down | mRNA |
| NM_183011 | CREM | 0.206897127 | 0.698502807 | 1.1335652 | down | mRNA |
| NM_183044 | RNF6 | 0.067594048 | 0.451808679 | 1.3320698 | down | mRNA |
| NM_183047 | ZMYND8 | 0.026698102 | 0.318484367 | 1.1503192 | down | mRNA |
| NM_183048 | ZMYND8 | 0.023298417 | 0.300022844 | 2.8322434 | down | mRNA |
| NM_183060 | CREM | 0.142552364 | 0.602652383 | 1.2398908 | down | mRNA |
| NM_183075 | CYP2U1 | 0.047847452 | 0.398293541 | 1.136512 | down | mRNA |
| NM_183078 | RNF8 | 0.010843843 | 0.213674567 | 1.4815575 | down | mRNA |
| NM_183228 | IKZF3 | 0.136220304 | 0.594906342 | 1.1325516 | down | mRNA |
| NM_183230 | IKZF3 | 0.707860897 | 0.965436846 | 1.1134754 | down | mRNA |
| NM_183237 | RNF7 | 0.331180599 | 0.821853462 | 1.1641629 | down | mRNA |
| NM_183238 | ZNF605 | 0.506157426 | 0.911278334 | 1.0785452 | down | mRNA |
| NM_183239 | GSTO2 | 0.099484221 | 0.523883236 | 1.2697306 | down | mRNA |
| NM_183240 | TMEM37 | 0.271420427 | 0.771497292 | 1.0754414 | down | mRNA |
| NM_183243 | IMPDH1 | 0.495362471 | 0.907415174 | 1.1605957 | down | mRNA |
| NM_183245 | INVS | 0.048682118 | 0.400568526 | 1.3243963 | down | mRNA |
| NM_183323 | PAIP1 | 0.067652808 | 0.451808679 | 1.1759502 | down | mRNA |
| NM_183352 | SEC13 | 0.102891096 | 0.532205007 | 1.2275851 | down | mRNA |
| NM_183356 | ASNS | 0.590916498 | 0.93680423 | 1.1396768 | down | mRNA |
| NM_183373 | PXDC1 | 4.87161E-05 | 0.02935779 | 1.748964 | down | mRNA |
| NM_183376 | ARRDC4 | 0.641916046 | 0.954332328 | 1.0257008 | down | mRNA |
| NM_183378 | OVCH1 | 0.555772661 | 0.926833616 | 1.1816575 | down | mRNA |
| NM_183381 | RNF13 | 0.849301566 | 0.987678166 | 1.0117798 | down | mRNA |
| NM_183397 | PXMP4 | 0.487434384 | 0.903749397 | 1.0592828 | down | mRNA |
| NM_183398 | RNF14 | 0.06693984 | 0.451282995 | 1.1886289 | down | mRNA |
| NM_183399 | RNF14 | 0.204344137 | 0.694397489 | 1.128267 | down | mRNA |
| NM_183422 | TSC22D1 | 0.17058587 | 0.64732212 | 1.1868725 | down | mRNA |
| NM_183425 | RBM38 | 0.833144286 | 0.986434891 | 1.0288573 | down | mRNA |
| NM_184234 | RBM39 | 0.002493065 | 0.118591515 | 1.2867267 | down | mRNA |
| NM_194071 | CREB3L2 | 0.885833766 | 0.992981836 | 1.0178946 | down | mRNA |
| NM_194247 | HNRNPA3 | 0.33972689 | 0.828117387 | 1.1380648 | down | mRNA |
| NM_194249 | DND1 | 0.282477298 | 0.780131544 | 1.2970503 | down | mRNA |
| NM_194252 | TTLL11 | 0.266447794 | 0.767064488 | 1.2941372 | down | mRNA |
| NM_194271 | RNF34 | 0.543883745 | 0.923577434 | 1.1029433 | down | mRNA |
| NM_194279 | ISCA2 | 0.021444997 | 0.288950479 | 1.2313059 | down | mRNA |
| NM_194285 | SPTY2D1 | 0.154896714 | 0.62235348 | 1.19043 | down | mRNA |
| NM_194291 | TMEM65 | 0.427904394 | 0.876992614 | 1.1089746 | down | mRNA |
| NM_194292 | SASS6 | 0.236364842 | 0.734490289 | 2.0097678 | down | mRNA |
| NM_194298 | SLC16A9 | 0.357504934 | 0.839643099 | 1.2127151 | down | mRNA |
| NM_194301 | RALGAPA1 | 0.125737007 | 0.578441772 | 1.1613462 | down | mRNA |
| NM_194314 | ZBTB41 | 0.257132734 | 0.757669958 | 1.3235982 | down | mRNA |
| NM_194315 | UBE2J2 | 0.05448529 | 0.418826057 | 1.1347823 | down | mRNA |
| NM_194318 | B3GALTL | 0.177610167 | 0.657722936 | 1.1681071 | down | mRNA |
| NM_194320 | ZNF169 | 0.122678403 | 0.574983504 | 1.1680148 | down | mRNA |
| NM_194324 | TMSB15B | 0.896943772 | 0.994222243 | 1.0152977 | down | mRNA |
| NM_194329 | RNF38 | 0.001880739 | 0.105670067 | 1.9906389 | down | mRNA |
| NM_194356 | STX2 | 0.08243643 | 0.489943729 | 1.184927 | down | mRNA |
| NM_194358 | RNF41 | 0.007645834 | 0.193257016 | 1.2761117 | down | mRNA |
| NM_194429 | FGFR1OP | 0.907488677 | 0.995145546 | 1.0077532 | down | mRNA |
| NM_194431 | RNASE4 | 0.910930516 | 0.995342392 | 1.0105998 | down | mRNA |
| NM_194434 | VAPA | 0.256676909 | 0.757240077 | 1.0705327 | down | mRNA |
| NM_194436 | LDHD | 0.527964059 | 0.918844141 | 1.054946 | down | mRNA |
| NM_194439 | RNF212 | 0.809938958 | 0.98118927 | 1.0201009 | down | mRNA |
| NM_194448 | CLEC4A | 0.02611339 | 0.316361069 | 1.6627503 | down | mRNA |
| NM_194449 | PHLPP1 | 0.783351371 | 0.977275912 | 1.0541857 | down | mRNA |
| NM_194450 | CLEC4A | 0.092680401 | 0.511364429 | 1.247905 | down | mRNA |
| NM_194451 | LIAS | 0.090845644 | 0.508835711 | 1.5434316 | down | mRNA |
| NM_194457 | UBE2J2 | 0.145867522 | 0.607759658 | 1.1054871 | down | mRNA |
| NM_197939 | RNF135 | 0.009966035 | 0.207399614 | 1.2327089 | down | mRNA |
| NM_197941 | ADAMTS6 | 0.270480525 | 0.77028705 | 1.0999664 | down | mRNA |
| NM_197956 | NAIF1 | 0.016426249 | 0.255922225 | 1.1882553 | down | mRNA |
| NM_197957 | MAX | 0.984539218 | 0.998971807 | 1.0016461 | down | mRNA |
| NM_197960 | DPP8 | 0.232563562 | 0.730348439 | 1.165793 | down | mRNA |
| NM_197961 | DPP8 | 0.121108406 | 0.572400147 | 1.1707529 | down | mRNA |
| NM_197972 | NME7 | 0.395427573 | 0.861590143 | 1.0596348 | down | mRNA |
| NM_198040 | PHC2 | 0.759927448 | 0.972852592 | 1.0312204 | down | mRNA |
| NM_198042 | PDLIM2 | 0.323571073 | 0.816213518 | 1.1945557 | down | mRNA |
| NM_198043 | ZDHHC16 | 0.389184321 | 0.858138555 | 1.0662868 | down | mRNA |
| NM_198044 | ZDHHC16 | 0.060129652 | 0.435030895 | 1.1327079 | down | mRNA |
| NM_198046 | ZDHHC16 | 0.301272573 | 0.796239953 | 1.0563432 | down | mRNA |
| NM_198047 | HIBCH | 0.116577393 | 0.562996237 | 1.0583466 | down | mRNA |
| NM_198055 | MZF1 | 0.344313666 | 0.831959196 | 1.1165134 | down | mRNA |
| NM_198056 | SCN5A | 0.193084558 | 0.680144706 | 1.1814104 | down | mRNA |
| NM_198061 | CES2 | 0.38980625 | 0.858671797 | 1.0962759 | down | mRNA |
| NM_198066 | GNPNAT1 | 0.005741697 | 0.17178751 | 1.2040197 | down | mRNA |
| NM_198076 | COX20 | 0.595039285 | 0.938655001 | 1.1292159 | down | mRNA |
| NM_198077 | C1orf52 | 0.035535477 | 0.35434395 | 1.3133162 | down | mRNA |
| NM_198086 | AJUBA | 0.003589093 | 0.139262737 | 2.2571166 | down | mRNA |
| NM_198087 | ZNF200 | 0.02154552 | 0.289533061 | 1.3777279 | down | mRNA |
| NM_198089 | ZNF155 | 0.556384101 | 0.926833616 | 1.1153838 | down | mRNA |
| NM_198120 | EBAG9 | 0.026865168 | 0.319244809 | 1.1952091 | down | mRNA |
| NM_198128 | RNF138 | 0.029569201 | 0.334186207 | 1.2707574 | down | mRNA |
| NM_198147 | ABHD15 | 0.161129705 | 0.632747516 | 1.294739 | down | mRNA |
| NM_198152 | UTS2D | 0.801475295 | 0.98118927 | 1.0601717 | down | mRNA |
| NM_198155 | C21orf33 | 0.683113452 | 0.960111041 | 1.0264371 | down | mRNA |
| NM_198159 | MITF | 0.516197815 | 0.914546356 | 1.1160714 | down | mRNA |
| NM_198175 | NME1 | 0.4879758 | 0.903757389 | 1.0539586 | down | mRNA |
| NM_198177 | MITF | 0.671830623 | 0.959531882 | 1.1037359 | down | mRNA |
| NM_198189 | COPS8 | 0.114407073 | 0.558493302 | 1.0871695 | down | mRNA |
| NM_198194 | STOM | 0.636166759 | 0.951839175 | 1.1386215 | down | mRNA |
| NM_198195 | UBA3 | 0.379478251 | 0.852847753 | 1.0800221 | down | mRNA |
| NM_198202 | POP5 | 0.785468709 | 0.977425166 | 1.0122504 | down | mRNA |
| NM_198204 | MLX | 0.699849542 | 0.962991468 | 1.0552828 | down | mRNA |
| NM_198215 | FAM13C | 0.437631899 | 0.882468688 | 1.1142001 | down | mRNA |
| NM_198229 | RGS12 | 0.113045058 | 0.556234457 | 1.1019029 | down | mRNA |
| NM_198239 | WISP3 | 0.979331881 | 0.998935223 | 1.0115701 | down | mRNA |
| NM_198241 | EIF4G1 | 0.716233822 | 0.966191059 | 1.0699623 | down | mRNA |
| NM_198242 | EIF4G1 | 0.74520522 | 0.970378122 | 1.0431276 | down | mRNA |
| NM_198243 | ASB7 | 0.096129706 | 0.51782059 | 1.2153608 | down | mRNA |
| NM_198256 | E2F6 | 0.102697936 | 0.531852889 | 1.2452522 | down | mRNA |
| NM_198264 | FAM189B | 0.405375592 | 0.866165103 | 1.0497577 | down | mRNA |
| NM_198268 | HIPK1 | 0.64969857 | 0.95550188 | 1.0261299 | down | mRNA |
| NM_198273 | LYSMD3 | 0.001078527 | 0.086778252 | 1.5193092 | down | mRNA |
| NM_198276 | TMEM17 | 0.867852073 | 0.989481868 | 1.0117377 | down | mRNA |
| NM_198277 | SLC37A2 | 0.714999776 | 0.966131151 | 1.0486221 | down | mRNA |
| NM_198279 | CXorf23 | 0.028615457 | 0.328520317 | 1.360712 | down | mRNA |
| NM_198309 | TTC8 | 0.299522842 | 0.795720295 | 1.2721406 | down | mRNA |
| NM_198310 | TTC8 | 0.497231899 | 0.908789022 | 1.1817811 | down | mRNA |
| NM_198316 | TENC1 | 0.630977648 | 0.950410442 | 1.1234341 | down | mRNA |
| NM_198317 | KLHL17 | 0.132375509 | 0.587843174 | 1.0891391 | down | mRNA |
| NM_198318 | PRMT1 | 0.132269658 | 0.587843174 | 1.0921457 | down | mRNA |
| NM_198320 | CPM | 0.821474089 | 0.982772998 | 1.0095046 | down | mRNA |
| NM_198321 | GALNT10 | 0.113259112 | 0.556496924 | 1.1200075 | down | mRNA |
| NM_198329 | UBA5 | 0.162415455 | 0.634678268 | 1.1524093 | down | mRNA |
| NM_198334 | GANAB | 0.233570158 | 0.73118887 | 1.1484069 | down | mRNA |
| NM_198376 | CACNA1G | 0.74287529 | 0.970330526 | 1.0276673 | down | mRNA |
| NM_198383 | CACNA1G | 0.85490632 | 0.988178307 | 1.015718 | down | mRNA |
| NM_198384 | CACNA1G | 0.927648773 | 0.995829678 | 1.0287773 | down | mRNA |
| NM_198385 | CACNA1G | 0.457645009 | 0.892293664 | 1.0771098 | down | mRNA |
| NM_198398 | ERGIC3 | 0.597281879 | 0.939714862 | 1.1024454 | down | mRNA |
| NM_198400 | NEDD4 | 0.188944627 | 0.6744312 | 1.6025443 | down | mRNA |
| NM_198401 | ANKRD46 | 0.05004061 | 0.404543016 | 1.320558 | down | mRNA |
| NM_198402 | PTPLB | 0.711992352 | 0.96592341 | 1.0200225 | down | mRNA |
| NM_198404 | KCTD4 | 0.524957538 | 0.917632491 | 1.1254287 | down | mRNA |
| NM_198406 | PAQR6 | 0.900206387 | 0.994782122 | 1.0132723 | down | mRNA |
| NM_198428 | BBS9 | 0.517495057 | 0.915488514 | 1.218119 | down | mRNA |
| NM_198440 | DERL3 | 0.604598351 | 0.94174007 | 1.0543055 | down | mRNA |
| NM_198472 | FUOM | 0.631264294 | 0.950410442 | 1.0536539 | down | mRNA |
| NM_198474 | OLFML1 | 0.551456661 | 0.926149092 | 1.1131243 | down | mRNA |
| NM_198475 | FAM171A2 | 0.872917279 | 0.990534615 | 1.0193397 | down | mRNA |
| NM_198480 | ZNF615 | 0.092545797 | 0.511338764 | 2.6605742 | down | mRNA |
| NM_198481 | VSTM1 | 0.444606642 | 0.88538122 | 1.2389448 | down | mRNA |
| NM_198484 | ZNF621 | 0.159037945 | 0.630863036 | 1.2523245 | down | mRNA |
| NM_198486 | RPL7L1 | 0.088862366 | 0.503553406 | 1.1263761 | down | mRNA |
| NM_198495 | CTAGE4 | 0.106681744 | 0.541344461 | 1.1743624 | down | mRNA |
| NM_198501 | SMTNL2 | 0.424077243 | 0.874798629 | 1.0873951 | down | mRNA |
| NM_198507 | FAM174A | 0.051728614 | 0.41031217 | 1.1433457 | down | mRNA |
| NM_198508 | KLRG2 | 0.456775851 | 0.891568474 | 1.0893423 | down | mRNA |
| NM_198513 | PHF20L1 | 0.197035307 | 0.685161614 | 1.1953813 | down | mRNA |
| NM_198514 | NHLRC2 | 0.315567313 | 0.810346887 | 1.136542 | down | mRNA |
| NM_198516 | GALNTL4 | 0.974365316 | 0.998501532 | 1.018578 | down | mRNA |
| NM_198517 | TBC1D10C | 0.123964141 | 0.577766088 | 1.1562591 | down | mRNA |
| NM_198524 | TEX9 | 0.891942852 | 0.9937521 | 1.0057169 | down | mRNA |
| NM_198530 | MXRA7 | 0.290268097 | 0.786468159 | 1.1728377 | down | mRNA |
| NM_198531 | ATP9B | 0.149048319 | 0.612750316 | 1.1089892 | down | mRNA |
| NM_198533 | HSD11B1L | 0.424058137 | 0.874798629 | 1.2542872 | down | mRNA |
| NM_198539 | ZNF568 | 0.00773683 | 0.19345927 | 1.3580445 | down | mRNA |
| NM_198540 | B3GNT8 | 0.967128709 | 0.99793927 | 1.0057691 | down | mRNA |
| NM_198549 | FAM73A | 0.4855312 | 0.903250293 | 1.0823226 | down | mRNA |
| NM_198551 | MIA3 | 0.200234672 | 0.68900633 | 1.2810853 | down | mRNA |
| NM_198552 | FAM89A | 0.421898261 | 0.873398499 | 1.3507764 | down | mRNA |
| NM_198562 | C3orf62 | 0.211339751 | 0.70269171 | 1.135614 | down | mRNA |
| NM_198565 | LRRC33 | 0.919506717 | 0.995349793 | 1.0274446 | down | mRNA |
| NM_198567 | C5orf25 | 0.00315746 | 0.132624561 | 1.2841709 | down | mRNA |
| NM_198569 | GPR126 | 0.522182633 | 0.916999248 | 1.0671831 | down | mRNA |
| NM_198572 | SPATC1 | 0.17231395 | 0.6499464 | 1.2612065 | down | mRNA |
| NM_198596 | SULF2 | 0.322253242 | 0.815372756 | 1.1174304 | down | mRNA |
| NM_198679 | RAPGEF1 | 0.443368785 | 0.884425653 | 1.1055401 | down | mRNA |
| NM_198681 | PLEKHG5 | 0.896571542 | 0.994222243 | 1.0269775 | down | mRNA |
| NM_198690 | KRTAP10-9 | 0.957449003 | 0.996883425 | 1.0058455 | down | mRNA |
| NM_198691 | KRTAP10-1 | 0.873603219 | 0.990580595 | 1.0103833 | down | mRNA |
| NM_198693 | KRTAP10-2 | 0.746950258 | 0.970432947 | 1.0166023 | down | mRNA |
| NM_198722 | AMIGO3 | 0.057351778 | 0.426665332 | 1.1535752 | down | mRNA |
| NM_198723 | TCEA2 | 0.650887179 | 0.956064017 | 1.1231067 | down | mRNA |
| NM_198793 | CD47 | 0.839738955 | 0.987068842 | 1.0139063 | down | mRNA |
| NM_198794 | MAP4K5 | 0.083902679 | 0.493179896 | 1.1520652 | down | mRNA |
| NM_198798 | ANKRD5 | 0.442145795 | 0.88419144 | 1.3332924 | down | mRNA |
| NM_198799 | BCAS4 | 0.468282498 | 0.897203143 | 1.1799959 | down | mRNA |
| NM_198827 | GPR133 | 0.923956959 | 0.995456159 | 1.0172145 | down | mRNA |
| NM_198830 | ACLY | 0.992265002 | 0.999781402 | 1.0006656 | down | mRNA |
| NM_198849 | SIAH3 | 0.36857271 | 0.845559574 | 1.3331669 | down | mRNA |
| NM_198855 | ZNF211 | 0.016465487 | 0.256337115 | 1.2147261 | down | mRNA |
| NM_198880 | QRICH1 | 0.939937718 | 0.996710991 | 1.0100236 | down | mRNA |
| NM_198881 | TBC1D8B | 0.345720105 | 0.832742705 | 1.1949105 | down | mRNA |
| NM_198883 | MTX1 | 0.463998114 | 0.894989844 | 1.0843231 | down | mRNA |
| NM_198887 | NUP43 | 0.445174513 | 0.885731305 | 1.1105959 | down | mRNA |
| NM_198889 | ANKRD17 | 0.00878602 | 0.202329498 | 1.2647511 | down | mRNA |
| NM_198890 | ATG16L1 | 0.485183834 | 0.903250293 | 1.2652935 | down | mRNA |
| NM_198896 | RAB6A | 0.067493799 | 0.451808679 | 1.1984085 | down | mRNA |
| NM_198901 | SRI | 0.675813101 | 0.959531882 | 1.1478938 | down | mRNA |
| NM_198902 | TSPAN3 | 0.857479251 | 0.988512246 | 1.0334781 | down | mRNA |
| NM_198904 | GABRG2 | 0.714122428 | 0.966110889 | 1.080118 | down | mRNA |
| NM_198920 | UBE3D | 0.167174775 | 0.642168434 | 1.3610649 | down | mRNA |
| NM_198926 | FAM212B | 0.005822242 | 0.173574514 | 1.2633365 | down | mRNA |
| NM_198928 | PYHIN1 | 0.509727353 | 0.911674633 | 1.0970579 | down | mRNA |
| NM_198929 | PYHIN1 | 0.736569932 | 0.968717225 | 1.0379823 | down | mRNA |
| NM_198935 | SS18L1 | 0.020807215 | 0.286620793 | 1.5206086 | down | mRNA |
| NM_198941 | SERINC3 | 0.729202729 | 0.966191059 | 1.0533121 | down | mRNA |
| NM_198956 | SP8 | 0.478479896 | 0.901271944 | 1.1040328 | down | mRNA |
| NM_198968 | DZIP1 | 0.632699561 | 0.951123179 | 1.1197443 | down | mRNA |
| NM_198971 | HINFP | 0.007240295 | 0.190657536 | 1.5719045 | down | mRNA |
| NM_198973 | MKNK1 | 0.821050436 | 0.982756843 | 1.0346646 | down | mRNA |
| NM_198990 | NAPEPLD | 0.003380648 | 0.137400229 | 1.4343725 | down | mRNA |
| NM_198996 | LIPI | 0.851665066 | 0.987678166 | 1.0588577 | down | mRNA |
| NM_199003 | THAP1 | 0.294372904 | 0.791301949 | 1.0564841 | down | mRNA |
| NM_199006 | APITD1-CORT | 0.867072346 | 0.989481868 | 1.0458877 | down | mRNA |
| NM_199044 | NSUN4 | 0.086724585 | 0.499950487 | 1.1461851 | down | mRNA |
| NM_199046 | TEPP | 0.816653633 | 0.981692737 | 1.0185301 | down | mRNA |
| NM_199053 | TRAPPC11 | 0.140454724 | 0.600490836 | 1.3627908 | down | mRNA |
| NM_199069 | NDUFAF3 | 0.054020609 | 0.417146607 | 1.1075441 | down | mRNA |
| NM_199077 | CNNM2 | 0.954602514 | 0.996834354 | 1.007457 | down | mRNA |
| NM_199078 | CNNM3 | 0.460763644 | 0.894172608 | 1.0673739 | down | mRNA |
| NM_199126 | ZNF585A | 0.001615109 | 0.103549765 | 1.4874132 | down | mRNA |
| NM_199127 | GGTLC2 | 0.779730859 | 0.976938981 | 1.0405147 | down | mRNA |
| NM_199129 | TMEM189 | 0.064566137 | 0.443826413 | 1.1250088 | down | mRNA |
| NM_199132 | ZNF468 | 0.503412235 | 0.910539771 | 1.1334091 | down | mRNA |
| NM_199133 | FAM173B | 0.213018601 | 0.705160241 | 1.1025452 | down | mRNA |
| NM_199139 | XAF1 | 0.058885626 | 0.431383592 | 1.3039509 | down | mRNA |
| NM_199160 | LHX6 | 0.040179746 | 0.371915487 | 1.1959774 | down | mRNA |
| NM_199168 | CXCL12 | 0.837451038 | 0.986775289 | 1.1151399 | down | mRNA |
| NM_199173 | BGLAP | 0.286787095 | 0.782942227 | 1.1139791 | down | mRNA |
| NM_199177 | MRRF | 0.184401272 | 0.66791858 | 1.146141 | down | mRNA |
| NM_199184 | C6orf108 | 0.771500642 | 0.975347132 | 1.0292811 | down | mRNA |
| NM_199185 | NPM1 | 0.007374935 | 0.190938402 | 1.1442503 | down | mRNA |
| NM_199190 | LARP4 | 0.108773841 | 0.546082252 | 1.0543281 | down | mRNA |
| NM_199191 | BRE | 0.982616142 | 0.998971807 | 1.0032789 | down | mRNA |
| NM_199202 | THEG | 0.556896694 | 0.926833616 | 1.1098269 | down | mRNA |
| NM_199229 | RPE | 0.057504115 | 0.42700171 | 1.1676593 | down | mRNA |
| NM_199243 | GPR150 | 0.66483226 | 0.958354006 | 1.0615377 | down | mRNA |
| NM_199248 | CACNB1 | 0.052137444 | 0.411765599 | 1.2109682 | down | mRNA |
| NM_199249 | C19orf48 | 0.594806243 | 0.938655001 | 1.2265434 | down | mRNA |
| NM_199297 | THYN1 | 0.739743009 | 0.969564098 | 1.0243447 | down | mRNA |
| NM_199330 | HOMER2 | 0.38170608 | 0.853514109 | 1.0403861 | down | mRNA |
| NM_199334 | THRA | 0.59852087 | 0.940648177 | 1.0651412 | down | mRNA |
| NM_199336 | FAHD2B | 0.543327537 | 0.923577434 | 1.1013707 | down | mRNA |
| NM_199351 | ILDR2 | 0.19908783 | 0.687651648 | 1.0795923 | down | mRNA |
| NM_199353 | PRB1 | 0.735244829 | 0.96794858 | 1.0586976 | down | mRNA |
| NM_199359 | TPD52L2 | 0.058161302 | 0.429544346 | 1.1509916 | down | mRNA |
| NM_199360 | TPD52L2 | 0.036290992 | 0.35732128 | 1.1058268 | down | mRNA |
| NM_199361 | TPD52L2 | 0.151451029 | 0.616600386 | 1.0833862 | down | mRNA |
| NM_199362 | TPD52L2 | 0.152638386 | 0.618255775 | 1.1300477 | down | mRNA |
| NM_199363 | TPD52L2 | 0.972356971 | 0.998501532 | 1.0015635 | down | mRNA |
| NM_199368 | TRPC4AP | 0.205406074 | 0.695588989 | 1.0732627 | down | mRNA |
| NM_199413 | PRC1 | 0.599141001 | 0.940648177 | 1.228879 | down | mRNA |
| NM_199415 | UBOX5 | 0.606644815 | 0.942197111 | 1.0568035 | down | mRNA |
| NM_199420 | POLQ | 0.764202105 | 0.973899357 | 1.1863596 | down | mRNA |
| NM_199425 | VSX1 | 0.014674638 | 0.24380986 | 1.0604465 | down | mRNA |
| NM_199426 | ZFP64 | 0.005913637 | 0.175016104 | 1.2582995 | down | mRNA |
| NM_199427 | ZFP64 | 0.69396108 | 0.96187237 | 1.1206289 | down | mRNA |
| NM_199436 | SPAST | 0.001226019 | 0.09206987 | 1.2010777 | down | mRNA |
| NM_199437 | PRDM10 | 0.112658375 | 0.55538204 | 1.2918647 | down | mRNA |
| NM_199438 | PRDM10 | 0.356664183 | 0.839337376 | 1.0748946 | down | mRNA |
| NM_199439 | PRDM10 | 0.751833752 | 0.970725371 | 1.0265757 | down | mRNA |
| NM_199440 | HSPD1 | 0.010511897 | 0.210032734 | 1.1417111 | down | mRNA |
| NM_199441 | ZNF334 | 0.557524195 | 0.926833616 | 1.0794201 | down | mRNA |
| NM_199443 | USP4 | 0.062885552 | 0.439491843 | 1.3780872 | down | mRNA |
| NM_199451 | ZNF365 | 0.955817004 | 0.996883425 | 1.0044149 | down | mRNA |
| NM_199453 | HTR4 | 0.526989573 | 0.918066318 | 1.0544782 | down | mRNA |
| NM_199462 | DSTYK | 0.007235943 | 0.190657536 | 1.3206857 | down | mRNA |
| NM_199464 | KCNRG | 0.918663584 | 0.995349793 | 1.027452 | down | mRNA |
| NM_199483 | C20orf24 | 0.258431384 | 0.759296063 | 1.0755077 | down | mRNA |
| NM_199487 | UQCC | 0.88689171 | 0.993077367 | 1.0023618 | down | mRNA |
| NM_201222 | MAGED2 | 0.661944451 | 0.958354006 | 1.0437868 | down | mRNA |
| NM_201253 | CRB1 | 0.361374386 | 0.842165256 | 1.0960978 | down | mRNA |
| NM_201263 | WARS2 | 0.983487174 | 0.998971807 | 1.0023698 | down | mRNA |
| NM_201265 | UBL7 | 0.634181939 | 0.951249516 | 1.0360551 | down | mRNA |
| NM_201269 | ZNF644 | 0.184755592 | 0.668017166 | 1.1633415 | down | mRNA |
| NM_201274 | MPRIP | 0.123850111 | 0.577744894 | 1.1344576 | down | mRNA |
| NM_201277 | CNN2 | 0.688334497 | 0.96055024 | 1.0492066 | down | mRNA |
| NM_201280 | BLOC1S5 | 0.05686264 | 0.424892026 | 1.4721713 | down | mRNA |
| NM_201281 | MTMR2 | 0.43948318 | 0.883054028 | 1.0559058 | down | mRNA |
| NM_201382 | PLEC | 0.307998531 | 0.802832011 | 1.3446611 | down | mRNA |
| NM_201398 | FLAD1 | 0.149809708 | 0.614198134 | 1.106302 | down | mRNA |
| NM_201412 | LUC7L | 0.101657959 | 0.529590777 | 1.0667229 | down | mRNA |
| NM_201429 | RTN3 | 0.546845617 | 0.924803608 | 1.100163 | down | mRNA |
| NM_201430 | RTN3 | 0.825778077 | 0.984306256 | 1.0410826 | down | mRNA |
| NM_201434 | RAB5C | 0.383340468 | 0.853658125 | 1.0689687 | down | mRNA |
| NM_201436 | H2AFV | 0.96009886 | 0.997738713 | 1.0058528 | down | mRNA |
| NM_201437 | TCEA1 | 0.625392183 | 0.948832915 | 1.1174854 | down | mRNA |
| NM_201441 | TEAD4 | 0.000540903 | 0.070952549 | 1.850374 | down | mRNA |
| NM_201442 | C1S | 0.780656275 | 0.976938981 | 1.0518524 | down | mRNA |
| NM_201443 | TEAD4 | 0.001589892 | 0.103276923 | 1.4161904 | down | mRNA |
| NM_201444 | DGKA | 0.145272018 | 0.606627782 | 1.1945782 | down | mRNA |
| NM_201446 | EGFL7 | 0.434326659 | 0.880931467 | 1.1295729 | down | mRNA |
| NM_201453 | CBWD3 | 0.549718318 | 0.925533435 | 1.1123119 | down | mRNA |
| NM_201516 | H2AFV | 0.687335138 | 0.960205549 | 1.0568813 | down | mRNA |
| NM_201517 | H2AFV | 0.825654882 | 0.984241034 | 1.0196745 | down | mRNA |
| NM_201542 | MED8 | 0.210584942 | 0.701214058 | 1.154569 | down | mRNA |
| NM_201543 | LGALS8 | 0.772089296 | 0.975347132 | 1.0253782 | down | mRNA |
| NM_201557 | FHL2 | 0.007046372 | 0.18703243 | 1.380564 | down | mRNA |
| NM_201559 | FOXO3 | 0.637964397 | 0.952852353 | 1.1300878 | down | mRNA |
| NM_201568 | SMG7 | 0.25885382 | 0.759358563 | 1.3369234 | down | mRNA |
| NM_201569 | SMG7 | 0.300715378 | 0.795797471 | 1.1354836 | down | mRNA |
| NM_201571 | CACNB2 | 0.644920861 | 0.954749596 | 1.1626057 | down | mRNA |
| NM_201574 | SLC4A3 | 0.811608544 | 0.98118927 | 1.046197 | down | mRNA |
| NM_201575 | SEZ6L2 | 0.443336119 | 0.884425653 | 1.1402966 | down | mRNA |
| NM_201590 | CACNB2 | 0.679034354 | 0.959969193 | 1.0676779 | down | mRNA |
| NM_201595 | GTF2A1 | 0.237832707 | 0.735771951 | 1.1710194 | down | mRNA |
| NM_201612 | IKBIP | 0.30049824 | 0.795797471 | 1.0977757 | down | mRNA |
| NM_201613 | IKBIP | 0.088942262 | 0.503865719 | 1.1196393 | down | mRNA |
| NM_201624 | USP33 | 0.253376576 | 0.753496058 | 1.2711232 | down | mRNA |
| NM_201626 | USP33 | 0.444787154 | 0.885567217 | 1.1027746 | down | mRNA |
| NM_201627 | TRIM41 | 0.1562535 | 0.624645331 | 1.1822363 | down | mRNA |
| NM_201628 | KAZN | 0.402059862 | 0.864786348 | 1.5121334 | down | mRNA |
| NM_201653 | CHIA | 0.713082755 | 0.966110889 | 1.0721795 | down | mRNA |
| NM_201997 | SF1 | 0.125536753 | 0.578441772 | 1.0648351 | down | mRNA |
| NM_202001 | ERCC1 | 0.274529291 | 0.774594718 | 1.1156785 | down | mRNA |
| NM_202002 | FOXM1 | 0.696994963 | 0.962410025 | 1.2480405 | down | mRNA |
| NM_202003 | FOXM1 | 0.571172826 | 0.933029103 | 1.1004502 | down | mRNA |
| NM_202468 | GIPC1 | 0.446033633 | 0.886278379 | 1.0346999 | down | mRNA |
| NM_202494 | GIPC1 | 0.361395429 | 0.842165256 | 1.0681256 | down | mRNA |
| NM_202758 | ENOSF1 | 0.780998306 | 0.976938981 | 1.0604584 | down | mRNA |
| NM_203282 | ZNF254 | 0.150512662 | 0.615491439 | 1.3693323 | down | mRNA |
| NM_203283 | RBPJ | 0.55425224 | 0.926549129 | 1.026486 | down | mRNA |
| NM_203285 | PVRL1 | 0.435567021 | 0.881627543 | 1.0495299 | down | mRNA |
| NM_203288 | RP9 | 0.081765997 | 0.488477232 | 1.3550687 | down | mRNA |
| NM_203290 | POLR1C | 0.167153272 | 0.642168434 | 1.1454607 | down | mRNA |
| NM_203293 | TRIM7 | 0.648642991 | 0.95550188 | 1.1588656 | down | mRNA |
| NM_203295 | TRIM7 | 0.697518353 | 0.962410025 | 1.2135453 | down | mRNA |
| NM_203298 | CHCHD1 | 0.386033489 | 0.855738431 | 1.0892439 | down | mRNA |
| NM_203301 | FBXO33 | 0.028595162 | 0.328520317 | 1.2598573 | down | mRNA |
| NM_203304 | MEX3D | 0.350656487 | 0.834911159 | 1.2356062 | down | mRNA |
| NM_203305 | FAM102A | 0.034473886 | 0.351579889 | 1.2713847 | down | mRNA |
| NM_203309 | HEATR4 | 0.29890045 | 0.795233272 | 1.1472323 | down | mRNA |
| NM_203318 | MYO18A | 0.290049173 | 0.786468159 | 1.774431 | down | mRNA |
| NM_203341 | SEP15 | 0.676765913 | 0.959531882 | 1.0329353 | down | mRNA |
| NM_203349 | SHC4 | 0.179350261 | 0.661565587 | 1.3963784 | down | mRNA |
| NM_203352 | PDLIM7 | 0.590663471 | 0.936549145 | 1.0905302 | down | mRNA |
| NM_203355 | CTAGE5 | 0.019881807 | 0.283521851 | 1.4241875 | down | mRNA |
| NM_203364 | CAPRIN1 | 0.000308503 | 0.055508758 | 1.1862448 | down | mRNA |
| NM_203365 | RAPH1 | 0.04286424 | 0.382411463 | 1.2250994 | down | mRNA |
| NM_203373 | FBXL22 | 0.984934287 | 0.998971807 | 1.0086645 | down | mRNA |
| NM_203376 | TMEM81 | 0.212936334 | 0.705117516 | 1.1452366 | down | mRNA |
| NM_203379 | ACSL5 | 0.80854874 | 0.98118927 | 1.0771667 | down | mRNA |
| NM_203382 | AMACR | 0.408538556 | 0.867591329 | 1.1953033 | down | mRNA |
| NM_203384 | RNH1 | 0.694420475 | 0.962043956 | 1.017202 | down | mRNA |
| NM_203390 | RBM12B | 0.023895231 | 0.303389731 | 1.277451 | down | mRNA |
| NM_203394 | E2F7 | 0.917075427 | 0.995349793 | 1.0150053 | down | mRNA |
| NM_203397 | MBLAC1 | 0.277767111 | 0.777258589 | 1.1441434 | down | mRNA |
| NM_203399 | STMN1 | 0.694378422 | 0.962043956 | 1.1040744 | down | mRNA |
| NM_203402 | FITM1 | 0.149864103 | 0.614198134 | 1.1140765 | down | mRNA |
| NM_203403 | LURAP1L | 0.787547973 | 0.977492986 | 1.1909842 | down | mRNA |
| NM_203404 | AMPD2 | 0.274479421 | 0.774561497 | 1.2062824 | down | mRNA |
| NM_203413 | ELP5 | 0.12400889 | 0.577766088 | 1.2381094 | down | mRNA |
| NM_203414 | ELP5 | 0.915530613 | 0.995349793 | 1.014559 | down | mRNA |
| NM_203433 | PSMG1 | 0.420983632 | 0.872788744 | 1.1028371 | down | mRNA |
| NM_203434 | IER5L | 0.000489038 | 0.067183255 | 3.4645044 | down | mRNA |
| NM_203451 | SERTM1 | 0.003906516 | 0.144151159 | 1.5098427 | down | mRNA |
| NM_203453 | PPAPDC2 | 0.02450289 | 0.306015212 | 1.2675425 | down | mRNA |
| NM_203456 | PPIE | 0.30050716 | 0.795797471 | 1.0968344 | down | mRNA |
| NM_203459 | CAMSAP2 | 0.045177392 | 0.389384436 | 1.1945037 | down | mRNA |
| NM_203462 | MRFAP1L1 | 0.666576393 | 0.958354006 | 1.024733 | down | mRNA |
| NM_203463 | CERS6 | 0.724414792 | 0.966191059 | 1.0374615 | down | mRNA |
| NM_203467 | LRR1 | 0.570333738 | 0.932906321 | 1.2209 | down | mRNA |
| NM_203468 | ENTPD2 | 0.485139685 | 0.903250293 | 1.3515581 | down | mRNA |
| NM_203473 | PORCN | 0.903766685 | 0.99508468 | 1.0285859 | down | mRNA |
| NM_203486 | DLL3 | 0.044142971 | 0.386336269 | 1.1232106 | down | mRNA |
| NM_203488 | ACYP1 | 0.367812256 | 0.845181673 | 1.1038244 | down | mRNA |
| NM_203497 | COMMD6 | 0.795821368 | 0.980144703 | 1.039588 | down | mRNA |
| NM_203499 | DDX42 | 0.938138783 | 0.996710991 | 1.0089626 | down | mRNA |
| NM_203504 | G3BP2 | 0.074457431 | 0.469775665 | 1.1976685 | down | mRNA |
| NM_203505 | G3BP2 | 0.34820349 | 0.834138149 | 1.1193178 | down | mRNA |
| NM_205836 | FBXO38 | 0.281499659 | 0.779126326 | 1.2500738 | down | mRNA |
| NM_205842 | NCKAP1 | 0.277209353 | 0.777205298 | 1.0857401 | down | mRNA |
| NM_205847 | GMPPA | 0.030281005 | 0.337447626 | 1.111341 | down | mRNA |
| NM_205855 | FAM180A | 0.55904064 | 0.927203296 | 1.1291137 | down | mRNA |
| NM_205861 | DHDDS | 0.481517502 | 0.901682656 | 1.0929196 | down | mRNA |
| NM_205863 | PARD3B | 0.210712632 | 0.701524355 | 1.3004764 | down | mRNA |
| NM_206538 | EMC10 | 0.751271915 | 0.970725371 | 1.0137526 | down | mRNA |
| NM_206810 | MOG | 0.650639982 | 0.955894361 | 1.0516537 | down | mRNA |
| NM_206812 | MOG | 0.733636895 | 0.967777568 | 1.056197 | down | mRNA |
| NM_206826 | GNL3 | 0.503805484 | 0.910568383 | 1.0883762 | down | mRNA |
| NM_206831 | DPH3 | 0.277289358 | 0.777205298 | 1.0570685 | down | mRNA |
| NM_206833 | CTXN1 | 0.475132544 | 0.899729429 | 1.2278191 | down | mRNA |
| NM_206836 | ECI2 | 0.131324377 | 0.586574523 | 1.0646604 | down | mRNA |
| NM_206840 | NVL | 0.613335616 | 0.94457959 | 1.0785066 | down | mRNA |
| NM_206860 | TACC2 | 0.073660276 | 0.467143085 | 1.1099832 | down | mRNA |
| NM_206862 | TACC2 | 0.724225034 | 0.966191059 | 1.0562222 | down | mRNA |
| NM_206873 | PPP1CA | 0.003022809 | 0.130211341 | 1.1578291 | down | mRNA |
| NM_206886 | CCDC18 | 0.215869056 | 0.710547135 | 1.2988479 | down | mRNA |
| NM_206889 | DIP2A | 0.226736591 | 0.722135151 | 1.1787214 | down | mRNA |
| NM_206890 | DIP2A | 0.65983836 | 0.958354006 | 1.038401 | down | mRNA |
| NM_206898 | MRAP | 0.000431898 | 0.064097442 | 1.5119872 | down | mRNA |
| NM_206900 | RTN2 | 0.012505138 | 0.227664282 | 1.4559779 | down | mRNA |
| NM_206907 | PRKAA1 | 0.020644947 | 0.286034385 | 1.2175736 | down | mRNA |
| NM_206915 | NGFRAP1 | 0.8940754 | 0.993978977 | 1.0143874 | down | mRNA |
| NM_206925 | CA12 | 0.619929592 | 0.947444797 | 1.0395766 | down | mRNA |
| NM_206926 | SEPN1 | 0.033117527 | 0.346127643 | 1.3141175 | down | mRNA |
| NM_206929 | SYTL2 | 0.638376943 | 0.952902137 | 1.1248748 | down | mRNA |
| NM_206930 | SYTL2 | 0.908313843 | 0.995302455 | 1.0335672 | down | mRNA |
| NM_206947 | TRPM3 | 0.322537912 | 0.815372756 | 1.104666 | down | mRNA |
| NM_206999 | CNOT1 | 0.16305267 | 0.635317534 | 1.1011364 | down | mRNA |
| NM_207006 | FAM83A | 0.660089954 | 0.958354006 | 1.0477395 | down | mRNA |
| NM_207009 | FAM45A | 0.129514879 | 0.583907255 | 1.226576 | down | mRNA |
| NM_207013 | TCEB2 | 0.808466828 | 0.98118927 | 1.0301679 | down | mRNA |
| NM_207014 | WDR78 | 0.548683462 | 0.92514258 | 1.1466036 | down | mRNA |
| NM_207037 | TCF12 | 0.273020112 | 0.772770327 | 1.1339735 | down | mRNA |
| NM_207038 | TCF12 | 0.166606567 | 0.641440015 | 1.1888575 | down | mRNA |
| NM_207040 | TCF12 | 0.571563373 | 0.933223365 | 1.163775 | down | mRNA |
| NM_207044 | ENSA | 0.702057074 | 0.963247543 | 1.0454558 | down | mRNA |
| NM_207046 | ENSA | 0.006210164 | 0.178855089 | 1.0919829 | down | mRNA |
| NM_207103 | SCIMP | 0.765482283 | 0.974502898 | 1.0091097 | down | mRNA |
| NM_207111 | RNF216 | 0.004617257 | 0.155233834 | 1.490892 | down | mRNA |
| NM_207113 | SLC37A3 | 0.172798892 | 0.650632622 | 1.2027146 | down | mRNA |
| NM_207116 | RNF216 | 0.004555401 | 0.155014562 | 1.3796307 | down | mRNA |
| NM_207122 | EXT2 | 0.556293913 | 0.926833616 | 1.0542739 | down | mRNA |
| NM_207123 | GAB1 | 0.001389487 | 0.095442746 | 1.42581 | down | mRNA |
| NM_207127 | PAOX | 0.158907144 | 0.630542556 | 1.2031882 | down | mRNA |
| NM_207128 | PAOX | 0.340252112 | 0.828404495 | 1.0601595 | down | mRNA |
| NM_207181 | NPHP1 | 0.677788335 | 0.959531882 | 1.0698961 | down | mRNA |
| NM_207191 | ADAM15 | 0.569286043 | 0.932377004 | 1.0513898 | down | mRNA |
| NM_207194 | ADAM15 | 0.497527572 | 0.908789022 | 1.0560004 | down | mRNA |
| NM_207197 | ADAM15 | 0.922880316 | 0.995349793 | 1.0095964 | down | mRNA |
| NM_207292 | MBNL1 | 0.068609238 | 0.454916653 | 1.2743 | down | mRNA |
| NM_207293 | MBNL1 | 0.026463012 | 0.318338323 | 1.0690598 | down | mRNA |
| NM_207294 | MBNL1 | 0.055050213 | 0.419520587 | 1.2672493 | down | mRNA |
| NM_207295 | MBNL1 | 0.167305225 | 0.642184223 | 1.2778937 | down | mRNA |
| NM_207296 | MBNL1 | 0.371842267 | 0.846997196 | 1.0788164 | down | mRNA |
| NM_207297 | MBNL1 | 0.243770915 | 0.742190811 | 1.2305023 | down | mRNA |
| NM_207299 | LPPR1 | 0.101512447 | 0.529356363 | 1.186254 | down | mRNA |
| NM_207304 | MBNL2 | 0.011687119 | 0.220344574 | 1.2056976 | down | mRNA |
| NM_207311 | CCDC64 | 0.211826291 | 0.703274354 | 1.2561871 | down | mRNA |
| NM_207318 | FAM199X | 0.006916132 | 0.18551293 | 1.2331604 | down | mRNA |
| NM_207325 | DPY19L3 | 0.180679147 | 0.663697999 | 1.1943945 | down | mRNA |
| NM_207327 | CDPF1 | 0.268530405 | 0.769158724 | 1.2000622 | down | mRNA |
| NM_207330 | NIPAL1 | 0.124785766 | 0.578441772 | 1.424136 | down | mRNA |
| NM_207332 | ERICH1 | 0.428266505 | 0.877211962 | 1.0894537 | down | mRNA |
| NM_207333 | ZNF320 | 0.021459533 | 0.288950479 | 1.3431692 | down | mRNA |
| NM_207338 | LCTL | 0.083121708 | 0.491675352 | 1.3588974 | down | mRNA |
| NM_207340 | ZDHHC24 | 0.510738855 | 0.912267627 | 1.0305824 | down | mRNA |
| NM_207341 | ZP1 | 0.464078791 | 0.894989844 | 1.1252006 | down | mRNA |
| NM_207342 | STEAP1B | 0.984168697 | 0.998971807 | 1.0020385 | down | mRNA |
| NM_207344 | SPRYD4 | 0.984908148 | 0.998971807 | 1.0010845 | down | mRNA |
| NM_207346 | TSEN54 | 0.425705672 | 0.87545849 | 1.0723407 | down | mRNA |
| NM_207352 | CYP4V2 | 0.599752287 | 0.940648177 | 1.0471089 | down | mRNA |
| NM_207354 | ANKRD13D | 0.825180837 | 0.98381451 | 1.0553503 | down | mRNA |
| NM_207356 | C1orf174 | 0.011847541 | 0.221526283 | 1.5175792 | down | mRNA |
| NM_207360 | ZC3H12D | 0.743747301 | 0.970330526 | 1.0558579 | down | mRNA |
| NM_207363 | NCKAP5 | 0.750308418 | 0.970725371 | 1.045724 | down | mRNA |
| NM_207411 | XKR5 | 0.131090052 | 0.586042863 | 1.145401 | down | mRNA |
| NM_207417 | C9orf171 | 0.946536746 | 0.996710991 | 1.0129803 | down | mRNA |
| NM_207517 | ADAMTSL3 | 0.096172402 | 0.51782059 | 1.2100548 | down | mRNA |
| NM_207518 | PRKACA | 0.830158386 | 0.985549562 | 1.0254786 | down | mRNA |
| NM_207520 | RTN4 | 0.528523688 | 0.918883792 | 1.0805946 | down | mRNA |
| NM_207521 | RTN4 | 0.515446032 | 0.914319064 | 1.0753299 | down | mRNA |
| NM_207578 | PRKACB | 0.185889898 | 0.669321303 | 1.257757 | down | mRNA |
| NM_207644 | FAM211B | 0.892610023 | 0.993978977 | 1.0480685 | down | mRNA |
| NM_207660 | ZC3H14 | 0.306295609 | 0.801653799 | 1.1571212 | down | mRNA |
| NM_207661 | ZC3H14 | 0.11836007 | 0.565835163 | 1.2190886 | down | mRNA |
| NM_207662 | ZC3H14 | 0.037417142 | 0.361408713 | 1.3187057 | down | mRNA |
| NM_212460 | ARL4A | 0.003399189 | 0.137400229 | 2.4799021 | down | mRNA |
| NM_212469 | CHKA | 0.223263807 | 0.71761506 | 1.1839504 | down | mRNA |
| NM_212471 | PRKAR1A | 0.131530971 | 0.586786184 | 1.2536526 | down | mRNA |
| NM_212474 | FN1 | 0.900315771 | 0.994789506 | 1.0288638 | down | mRNA |
| NM_212476 | FN1 | 0.781762602 | 0.976938981 | 1.061522 | down | mRNA |
| NM_212481 | ARID5A | 0.532683744 | 0.92042723 | 1.0950642 | down | mRNA |
| NM_212503 | CDK18 | 0.818367126 | 0.981943961 | 1.0352273 | down | mRNA |
| NM_212550 | BLOC1S3 | 0.000467928 | 0.066148041 | 1.630487 | down | mRNA |
| NM_212551 | LYSMD1 | 0.169204888 | 0.645697032 | 1.1186712 | down | mRNA |
| NM_212554 | METTL10 | 0.474045019 | 0.899418411 | 1.1293179 | down | mRNA |
| NM_213566 | DFFA | 0.865587049 | 0.989401142 | 1.0241533 | down | mRNA |
| NM_213569 | NEBL | 0.315617598 | 0.810346887 | 1.0627741 | down | mRNA |
| NM_213589 | RAPH1 | 0.239484625 | 0.73766795 | 1.0634527 | down | mRNA |
| NM_213590 | TRIM13 | 0.050462489 | 0.40649894 | 1.3401687 | down | mRNA |
| NM_213595 | ISCU | 0.885360529 | 0.992981836 | 1.0464966 | down | mRNA |
| NM_213596 | FOXN4 | 0.555097913 | 0.926833616 | 1.0777484 | down | mRNA |
| NM_213603 | ZNF789 | 0.016305463 | 0.255922225 | 1.6453775 | down | mRNA |
| NM_213608 | C2orf66 | 0.320291441 | 0.814552264 | 1.4005654 | down | mRNA |
| NM_213611 | SLC25A3 | 0.74944858 | 0.970725371 | 1.0439608 | down | mRNA |
| NM_213619 | ATP6V1H | 0.298552395 | 0.795148977 | 1.1115593 | down | mRNA |
| NM_213620 | ATP6V1H | 0.070856846 | 0.460275207 | 1.1615312 | down | mRNA |
| NM_213621 | HTR3A | 0.371026057 | 0.846402038 | 1.1112739 | down | mRNA |
| NM_213632 | C20orf132 | 0.534891559 | 0.921043919 | 1.1357108 | down | mRNA |
| NM_213633 | PSG4 | 0.800462776 | 0.98118927 | 1.1062306 | down | mRNA |
| NM_213636 | PDLIM7 | 0.855332732 | 0.988178307 | 1.0067587 | down | mRNA |
| NM_213648 | TCF7 | 0.273674058 | 0.773899991 | 1.2527831 | down | mRNA |
| NM_213674 | TPM2 | 0.691778872 | 0.961451327 | 1.1061001 | down | mRNA |
| AA010334 |  | 0.483265039 | 0.919439531 | 1.0444312 | up | lncRNA |
| AA164863 |  | 0.636132766 | 0.96564589 | 1.132391 | up | lncRNA |
| AA166697 |  | 0.534396152 | 0.935381352 | 1.1840932 | up | lncRNA |
| AA219324 |  | 0.281353295 | 0.832297359 | 1.4873326 | up | lncRNA |
| AA309472 |  | 0.279471401 | 0.832297359 | 1.0894932 | up | lncRNA |
| AA420706 |  | 0.858772977 | 0.996142678 | 1.0116612 | up | lncRNA |
| AA457003 |  | 0.34336479 | 0.863514929 | 1.1440473 | up | lncRNA |
| AA504663 |  | 0.858498463 | 0.996142678 | 1.0863625 | up | lncRNA |
| AA584758 |  | 0.803753995 | 0.992578294 | 1.0789257 | up | lncRNA |
| AA593324 |  | 0.613802283 | 0.958235371 | 1.0732185 | up | lncRNA |
| AA594211 |  | 0.480606487 | 0.919198334 | 1.16182 | up | lncRNA |
| AA601302 |  | 0.412189673 | 0.890899058 | 1.3062136 | up | lncRNA |
| AA679157 |  | 0.534524424 | 0.935381352 | 1.1041782 | up | lncRNA |
| AA683203 |  | 0.284684749 | 0.834623138 | 1.3465291 | up | lncRNA |
| AA782034 |  | 0.003302176 | 0.167945066 | 1.7736539 | up | lncRNA |
| AA808940 |  | 0.514313766 | 0.929636449 | 1.0519322 | up | lncRNA |
| AA810436 |  | 0.217507156 | 0.785656962 | 1.3315426 | up | lncRNA |
| AA828826R |  | 0.133912985 | 0.689601422 | 1.560378 | up | lncRNA |
| AA878381 |  | 0.712953394 | 0.98251396 | 1.0610588 | up | lncRNA |
| AA879429 |  | 0.852576299 | 0.995900317 | 1.0379792 | up | lncRNA |
| AF056438 |  | 0.584853542 | 0.949634351 | 1.1152169 | up | lncRNA |
| AF070541 |  | 0.931555819 | 0.998875101 | 1.0063229 | up | lncRNA |
| AF193050R |  | 0.783512625 | 0.99121281 | 1.0291453 | up | lncRNA |
| AI015500 |  | 0.010459257 | 0.261616866 | 2.1874703 | up | lncRNA |
| AI091628 |  | 0.987174604 | 0.99906281 | 1.0020069 | up | lncRNA |
| AI161328 |  | 0.209874099 | 0.777319501 | 1.3648827 | up | lncRNA |
| AI201251 |  | 0.777185838 | 0.991152912 | 1.1603824 | up | lncRNA |
| AI203030 |  | 0.966794068 | 0.998875101 | 1.0032074 | up | lncRNA |
| AI307105 |  | 0.405276838 | 0.889198786 | 1.2339484 | up | lncRNA |
| AI379096 |  | 0.480976091 | 0.9192486 | 1.1292321 | up | lncRNA |
| AI547046 |  | 0.673287976 | 0.97507861 | 1.0826807 | up | lncRNA |
| AI767017 |  | 0.000635906 | 0.094750704 | 2.7905953 | up | lncRNA |
| AI808306 |  | 0.550486126 | 0.942167708 | 1.1527635 | up | lncRNA |
| AI889529 |  | 0.141616153 | 0.698568819 | 1.0615302 | up | lncRNA |
| AI927525 |  | 0.363350709 | 0.874234597 | 1.1056393 | up | lncRNA |
| AJ001873 |  | 0.887684383 | 0.998875101 | 1.0144251 | up | lncRNA |
| AK001840 |  | 0.228016774 | 0.798130144 | 1.1874415 | up | lncRNA |
| AK021960 |  | 0.465726553 | 0.913821992 | 1.0587833 | up | lncRNA |
| AK022294 |  | 0.288627653 | 0.837264328 | 1.220834 | up | lncRNA |
| AK022421 |  | 2.63438E-07 | 0.001260287 | 13.2354567 | up | lncRNA |
| AK022427 |  | 0.101931297 | 0.632351488 | 1.387382 | up | lncRNA |
| AK023372 |  | 0.97481154 | 0.998875101 | 1.0065194 | up | lncRNA |
| AK023412 |  | 0.805311746 | 0.992578294 | 1.0353696 | up | lncRNA |
| AK025564 |  | 0.028999476 | 0.407241179 | 1.2512028 | up | lncRNA |
| AK026788 |  | 0.010718995 | 0.261630988 | 3.164006 | up | lncRNA |
| AK026847 |  | 0.801463495 | 0.992578294 | 1.2102039 | up | lncRNA |
| AK055084R |  | 0.583902609 | 0.949379205 | 1.12976 | up | lncRNA |
| AK055531 |  | 0.021885844 | 0.361215409 | 1.5661472 | up | lncRNA |
| AK091312 |  | 0.155948278 | 0.711341825 | 1.4364612 | up | lncRNA |
| AK091319R |  | 0.349920556 | 0.867400431 | 1.1221148 | up | lncRNA |
| AK093610 |  | 0.076589628 | 0.570723954 | 1.4866235 | up | lncRNA |
| AK095276 |  | 0.543966639 | 0.939697392 | 1.0620132 | up | lncRNA |
| AK098597 |  | 0.815016062 | 0.99257857 | 1.0112843 | up | lncRNA |
| AK098597R |  | 0.43945048 | 0.9037775 | 1.1642037 | up | lncRNA |
| AK123125 |  | 0.05340649 | 0.515925762 | 1.16617 | up | lncRNA |
| AK123196 |  | 0.83856373 | 0.995583664 | 1.1832327 | up | lncRNA |
| AK123317R |  | 0.638584138 | 0.96564589 | 1.0453353 | up | lncRNA |
| AK126099 |  | 0.354797637 | 0.870139387 | 1.2414102 | up | lncRNA |
| AK309032R |  | 0.692192016 | 0.97895216 | 1.1135431 | up | lncRNA |
| AK310205 |  | 0.069636553 | 0.55572632 | 2.0812198 | up | lncRNA |
| AL038759 |  | 0.744392052 | 0.985094857 | 1.0799506 | up | lncRNA |
| AL046627 |  | 0.657494492 | 0.971418669 | 1.1292939 | up | lncRNA |
| AL049990 |  | 0.946980065 | 0.998875101 | 1.1461592 | up | lncRNA |
| AL080072 |  | 0.714699292 | 0.98251396 | 1.1370471 | up | lncRNA |
| AL137474 |  | 0.875932297 | 0.998411428 | 1.0326025 | up | lncRNA |
| AL713779 |  | 0.291294289 | 0.837264328 | 1.1575747 | up | lncRNA |
| AL713779R |  | 0.73034628 | 0.983295479 | 1.0236826 | up | lncRNA |
| AL832168 |  | 0.004101561 | 0.183182652 | 1.6893458 | up | lncRNA |
| AU185412 |  | 0.749286239 | 0.985227311 | 1.0331308 | up | lncRNA |
| AV648285 |  | 0.138140951 | 0.69662647 | 1.3521574 | up | lncRNA |
| AV730121 |  | 0.957805812 | 0.998875101 | 1.0048926 | up | lncRNA |
| AV730200 |  | 0.161318166 | 0.716150139 | 1.285507 | up | lncRNA |
| AV731389 |  | 0.92788337 | 0.998875101 | 1.0226966 | up | lncRNA |
| AW076083 |  | 0.398537693 | 0.887618401 | 1.0944704 | up | lncRNA |
| AW151284 |  | 0.406531807 | 0.889488526 | 1.2199386 | up | lncRNA |
| AW235165 |  | 0.029759434 | 0.413940496 | 1.4339534 | up | lncRNA |
| AW270668 |  | 0.582113358 | 0.949239022 | 1.1977377 | up | lncRNA |
| AW513776 |  | 0.03467543 | 0.429764781 | 1.222794 | up | lncRNA |
| AW631458 |  | 0.516468151 | 0.930031728 | 1.1396588 | up | lncRNA |
| AW820382 |  | 0.147858092 | 0.704258422 | 1.1821938 | up | lncRNA |
| AW833912R |  | 0.1456414 | 0.70076997 | 1.9078513 | up | lncRNA |
| AW847630R |  | 0.080753652 | 0.582801546 | 1.1977852 | up | lncRNA |
| AY010115 |  | 0.47253874 | 0.916418344 | 1.1569564 | up | lncRNA |
| AY203949 |  | 0.42043417 | 0.895254723 | 1.0288117 | up | lncRNA |
| AY203949R |  | 0.610031609 | 0.957150753 | 1.0388255 | up | lncRNA |
| AY665172R |  | 0.239894366 | 0.809169724 | 1.7052177 | up | lncRNA |
| AY927492 |  | 0.397438514 | 0.887348439 | 1.1294584 | up | lncRNA |
| AY927495R |  | 0.071385815 | 0.559511998 | 1.2197363 | up | lncRNA |
| AY927632 |  | 0.18812817 | 0.754410181 | 1.2542401 | up | lncRNA |
| BC006431 |  | 0.624520532 | 0.96133281 | 1.0608702 | up | lncRNA |
| BC009871 |  | 0.474424579 | 0.917441334 | 1.1391058 | up | lncRNA |
| BC030084 |  | 0.354031942 | 0.869401398 | 1.0498304 | up | lncRNA |
| BC034527R |  | 0.307327842 | 0.847137912 | 1.1070019 | up | lncRNA |
| BC042589 |  | 0.764313184 | 0.989189555 | 1.0778169 | up | lncRNA |
| BC046172 |  | 0.000613886 | 0.094750704 | 3.6078895 | up | lncRNA |
| BE083440 |  | 0.487386558 | 0.920771743 | 1.1318223 | up | lncRNA |
| BE092230 |  | 0.288336693 | 0.837198948 | 1.2683323 | up | lncRNA |
| BE174175 |  | 0.051474087 | 0.511249893 | 1.3050683 | up | lncRNA |
| BE174175R |  | 0.44404184 | 0.90519468 | 1.1554198 | up | lncRNA |
| BE327666 |  | 0.539685199 | 0.937437834 | 1.1019645 | up | lncRNA |
| BE675158 |  | 0.817587518 | 0.992651327 | 1.0595169 | up | lncRNA |
| BE729704 |  | 0.178588065 | 0.743651912 | 1.4321724 | up | lncRNA |
| BF377574R |  | 0.388404633 | 0.88524429 | 1.1512781 | up | lncRNA |
| BF379117 |  | 0.283929172 | 0.834126377 | 1.2405997 | up | lncRNA |
| BF379117R |  | 0.713409928 | 0.98251396 | 1.0468085 | up | lncRNA |
| BF379338R |  | 0.579540592 | 0.948676882 | 1.207547 | up | lncRNA |
| BF511054 |  | 0.648549233 | 0.969810478 | 1.0338462 | up | lncRNA |
| BF515427 |  | 0.149410675 | 0.704640207 | 1.1206969 | up | lncRNA |
| BF575745 |  | 0.638857796 | 0.96564589 | 1.0629588 | up | lncRNA |
| BF770830R |  | 0.179476184 | 0.744893635 | 1.1330015 | up | lncRNA |
| BF795336 |  | 0.82764199 | 0.994074191 | 1.016297 | up | lncRNA |
| BF799448R |  | 0.049210864 | 0.501683335 | 1.4177183 | up | lncRNA |
| BF803219 |  | 0.858357188 | 0.996142678 | 1.02011 | up | lncRNA |
| BF822271R |  | 0.321631811 | 0.853039439 | 1.3348018 | up | lncRNA |
| BF939605 |  | 0.328832313 | 0.85540421 | 1.2563343 | up | lncRNA |
| BF940279 |  | 0.91990977 | 0.998875101 | 1.0147784 | up | lncRNA |
| BF950033 |  | 0.149473715 | 0.704640207 | 1.3350914 | up | lncRNA |
| BF990071R |  | 0.009970166 | 0.25643695 | 1.2869215 | up | lncRNA |
| BG013335 |  | 0.256782721 | 0.822133742 | 1.5215094 | up | lncRNA |
| BG185212R |  | 0.908499002 | 0.998875101 | 1.0095956 | up | lncRNA |
| BG619841 |  | 0.161480323 | 0.716182201 | 1.1075666 | up | lncRNA |
| BG679513 |  | 0.039636979 | 0.454493742 | 1.734995 | up | lncRNA |
| BG925420 |  | 0.268154957 | 0.829967639 | 1.1481874 | up | lncRNA |
| BG925695 |  | 0.988922572 | 0.99906281 | 1.0053962 | up | lncRNA |
| BG926920 |  | 0.463291678 | 0.913747281 | 1.2049551 | up | lncRNA |
| BI116755 |  | 0.936630262 | 0.998875101 | 1.0223673 | up | lncRNA |
| BI763082 |  | 0.925664944 | 0.998875101 | 1.0085603 | up | lncRNA |
| BM699739 |  | 0.282235627 | 0.832609603 | 1.1017696 | up | lncRNA |
| BM728564 |  | 0.639486944 | 0.96564589 | 1.0801572 | up | lncRNA |
| BM990693 |  | 0.005215889 | 0.201643161 | 1.7230426 | up | lncRNA |
| BQ638469 |  | 0.914403806 | 0.998875101 | 1.042866 | up | lncRNA |
| BU564349 |  | 4.50177E-05 | 0.02692056 | 8.3760638 | up | lncRNA |
| BU594464 |  | 0.655473381 | 0.971258623 | 1.0385939 | up | lncRNA |
| BU624640 |  | 0.886092889 | 0.998875101 | 1.0137389 | up | lncRNA |
| BU662692 |  | 0.981782251 | 0.998875101 | 1.0020786 | up | lncRNA |
| BU663707 |  | 0.774103383 | 0.990435061 | 1.0333362 | up | lncRNA |
| BU739297 |  | 0.717664715 | 0.98251396 | 1.080362 | up | lncRNA |
| BU753580 |  | 0.6394996 | 0.96564589 | 1.1481723 | up | lncRNA |
| BX088948 |  | 0.384030095 | 0.882574488 | 1.0416943 | up | lncRNA |
| BX088953 |  | 0.917366823 | 0.998875101 | 1.0159146 | up | lncRNA |
| BX093283 |  | 0.794821366 | 0.991420185 | 1.0119648 | up | lncRNA |
| BX093893 |  | 0.09065265 | 0.604992392 | 1.3051859 | up | lncRNA |
| BX096636 |  | 0.405115083 | 0.889198786 | 1.0339848 | up | lncRNA |
| BX105843 |  | 0.968433375 | 0.998875101 | 1.0022361 | up | lncRNA |
| BX114362 |  | 0.338363694 | 0.86050695 | 1.4765134 | up | lncRNA |
| BX471032 |  | 0.178858358 | 0.743651912 | 1.0879445 | up | lncRNA |
| BX478947 |  | 0.708500023 | 0.981609897 | 1.0760268 | up | lncRNA |
| BX956284 |  | 0.008028763 | 0.240060015 | 1.3196737 | up | lncRNA |
| CA423547 |  | 0.268448572 | 0.830054406 | 1.0845999 | up | lncRNA |
| CA433290 |  | 0.195356934 | 0.761823233 | 1.6502901 | up | lncRNA |
| CA442294 |  | 0.238234733 | 0.807544847 | 1.3437973 | up | lncRNA |
| CB112975 |  | 0.373966728 | 0.879143403 | 1.2976545 | up | lncRNA |
| CD110511 |  | 0.680175349 | 0.976675999 | 1.0546174 | up | lncRNA |
| CD245860 |  | 0.310941764 | 0.848894083 | 1.1643834 | up | lncRNA |
| CD357052 |  | 0.098526675 | 0.624143367 | 1.7275513 | up | lncRNA |
| CD514953 |  | 0.79967652 | 0.992228977 | 1.0155888 | up | lncRNA |
| CD557263 |  | 0.243560515 | 0.810061605 | 1.2599022 | up | lncRNA |
| CF129145 |  | 0.121413969 | 0.672928817 | 1.5292273 | up | lncRNA |
| chr1:105438075-105481400- | chr1:105438075-105481400 | 0.978301611 | 0.998875101 | 1.0030141 | up | lncRNA |
| chr1:10834200-10846175- | chr1:10834200-10846175 | 0.727276081 | 0.98251396 | 1.0476244 | up | lncRNA |
| chr1:192286275-192304050- | chr1:192286275-192304050 | 0.00120661 | 0.122817539 | 1.8557852 | up | lncRNA |
| chr1:212355225-212367225- | chr1:212355225-212367225 | 0.933980505 | 0.998875101 | 1.007092 | up | lncRNA |
| chr1:232737950-232749825- | chr1:232737950-232749825 | 0.828923994 | 0.994074191 | 1.051269 | up | lncRNA |
| chr1:233220100-233231000+ | chr1:233220100-233231000 | 0.266618099 | 0.828881561 | 1.2410931 | up | lncRNA |
| chr1:242433950-242449875- | chr1:242433950-242449875 | 0.738939775 | 0.984978514 | 1.0148356 | up | lncRNA |
| chr1:36804800-36856000- | chr1:36804800-36856000 | 0.228120655 | 0.798130144 | 1.8103972 | up | lncRNA |
| chr1:38888750-38900850+ | chr1:38888750-38900850 | 0.367729547 | 0.875668569 | 1.1128886 | up | lncRNA |
| chr1:4325150-4335375- | chr1:4325150-4335375 | 0.687898247 | 0.97861545 | 1.0991863 | up | lncRNA |
| chr1:47977525-47990050- | chr1:47977525-47990050 | 0.162924188 | 0.718368032 | 1.5112744 | up | lncRNA |
| chr1:47977525-47990050+ | chr1:47977525-47990050 | 0.060222615 | 0.531246381 | 1.2722959 | up | lncRNA |
| chr1:54700800-54732968+ | chr1:54700800-54732968 | 0.459655136 | 0.91221792 | 1.0862764 | up | lncRNA |
| chr1:79669875-79726875- | chr1:79669875-79726875 | 0.635191144 | 0.965194977 | 1.1043994 | up | lncRNA |
| chr10:102320175-102331900- | chr10:102320175-102331900 | 0.6058152 | 0.956038292 | 1.0480204 | up | lncRNA |
| chr10:112068400-112115800- | chr10:112068400-112115800 | 0.162564239 | 0.718236848 | 1.1415856 | up | lncRNA |
| chr10:113880000-113890875- | chr10:113880000-113890875 | 0.120274973 | 0.669556486 | 1.1689676 | up | lncRNA |
| chr10:14583425-14595550- | chr10:14583425-14595550 | 0.846590197 | 0.995583664 | 1.0686093 | up | lncRNA |
| chr10:1842775-1853600+ | chr10:1842775-1853600 | 0.675922837 | 0.976096237 | 1.0907175 | up | lncRNA |
| chr10:44057525-44069225+ | chr10:44057525-44069225 | 0.261520604 | 0.82544125 | 1.0807356 | up | lncRNA |
| chr10:54673950-54691500+ | chr10:54673950-54691500 | 0.498827513 | 0.921805853 | 1.3536032 | up | lncRNA |
| chr10:7584675-7594825- | chr10:7584675-7594825 | 0.367505962 | 0.87558047 | 1.2151857 | up | lncRNA |
| chr11:113400350-113410875- | chr11:113400350-113410875 | 0.752797677 | 0.986562183 | 1.0805673 | up | lncRNA |
| chr11:39334375-39350600- | chr11:39334375-39350600 | 0.19756398 | 0.763778725 | 1.1450747 | up | lncRNA |
| chr11:8171725-8184025+ | chr11:8171725-8184025 | 0.945578168 | 0.998875101 | 1.0141429 | up | lncRNA |
| chr11:8260275-8275625- | chr11:8260275-8275625 | 0.172088839 | 0.733319188 | 1.1311691 | up | lncRNA |
| chr11:8260275-8275625+ | chr11:8260275-8275625 | 0.15123481 | 0.706090436 | 1.1225832 | up | lncRNA |
| chr12:125949425-125960625- | chr12:125949425-125960625 | 0.077872325 | 0.575629572 | 1.3870146 | up | lncRNA |
| chr12:53675775-53689275- | chr12:53675775-53689275 | 0.458988227 | 0.91221792 | 1.1061516 | up | lncRNA |
| chr12:60293850-60330425+ | chr12:60293850-60330425 | 0.969312934 | 0.998875101 | 1.0032742 | up | lncRNA |
| chr12:62078650-62106025+ | chr12:62078650-62106025 | 0.852152554 | 0.995900317 | 1.015116 | up | lncRNA |
| chr13:102433875-102457175+ | chr13:102433875-102457175 | 0.949442325 | 0.998875101 | 1.0157843 | up | lncRNA |
| chr13:104871700-104904225- | chr13:104871700-104904225 | 0.354168049 | 0.869401398 | 1.1105093 | up | lncRNA |
| chr13:19779350-19799600+ | chr13:19779350-19799600 | 0.460295144 | 0.91221792 | 1.0611157 | up | lncRNA |
| chr13:24548975-24561125+ | chr13:24548975-24561125 | 0.019568681 | 0.348881619 | 1.1936746 | up | lncRNA |
| chr13:52409200-52419600- | chr13:52409200-52419600 | 0.392822859 | 0.885711362 | 1.0579459 | up | lncRNA |
| chr13:52409200-52419600+ | chr13:52409200-52419600 | 0.002784946 | 0.154536879 | 1.1710903 | up | lncRNA |
| chr13:57331500-57346525+ | chr13:57331500-57346525 | 0.241710882 | 0.809272444 | 1.1857749 | up | lncRNA |
| chr13:72717475-72744200+ | chr13:72717475-72744200 | 0.390698227 | 0.885711362 | 1.1568326 | up | lncRNA |
| chr13:90287700-90298800- | chr13:90287700-90298800 | 0.683984406 | 0.977836377 | 1.0540026 | up | lncRNA |
| chr14:101121575-101132150- | chr14:101121575-101132150 | 0.395365791 | 0.885711362 | 1.0542949 | up | lncRNA |
| chr14:58685825-58705500+ | chr14:58685825-58705500 | 0.768621715 | 0.989189555 | 1.1578818 | up | lncRNA |
| chr14:75090375-75100625+ | chr14:75090375-75100625 | 0.74677887 | 0.985094857 | 1.201636 | up | lncRNA |
| chr15:35840950-35861750+ | chr15:35840950-35861750 | 0.85218429 | 0.995900317 | 1.0306559 | up | lncRNA |
| chr16:82854175-82865325+ | chr16:82854175-82865325 | 0.482837768 | 0.919300564 | 1.1978572 | up | lncRNA |
| chr17:21173800-21193125- | chr17:21173800-21193125 | 0.097615397 | 0.62265608 | 1.2165202 | up | lncRNA |
| chr18:10217025-10229050- | chr18:10217025-10229050 | 0.184872306 | 0.751257244 | 1.0920089 | up | lncRNA |
| chr18:18297450-18310625- | chr18:18297450-18310625 | 0.049287451 | 0.501683335 | 1.1748235 | up | lncRNA |
| chr18:45525275-45537075- | chr18:45525275-45537075 | 0.009813438 | 0.255612442 | 1.2800783 | up | lncRNA |
| chr2:102247075-102260750- | chr2:102247075-102260750 | 0.691410235 | 0.978695455 | 1.0485258 | up | lncRNA |
| chr2:102247075-102260750+ | chr2:102247075-102260750 | 0.407800352 | 0.889546439 | 1.5586675 | up | lncRNA |
| chr2:120864150-120877825+ | chr2:120864150-120877825 | 0.422498554 | 0.895936649 | 1.1069576 | up | lncRNA |
| chr2:122289675-122301200+ | chr2:122289675-122301200 | 0.602007657 | 0.954578533 | 1.0407943 | up | lncRNA |
| chr2:134177450-134192075- | chr2:134177450-134192075 | 0.082430599 | 0.585414993 | 1.171899 | up | lncRNA |
| chr2:148061475-148073025- | chr2:148061475-148073025 | 0.555251317 | 0.943023022 | 1.0966463 | up | lncRNA |
| chr2:148061475-148073025+ | chr2:148061475-148073025 | 0.017773358 | 0.337638939 | 1.1976426 | up | lncRNA |
| chr2:192023525-192035025+ | chr2:192023525-192035025 | 0.540971489 | 0.937910463 | 1.104206 | up | lncRNA |
| chr2:231247700-231258825- | chr2:231247700-231258825 | 0.315846314 | 0.851223284 | 1.1379793 | up | lncRNA |
| chr2:237208375-237219525+ | chr2:237208375-237219525 | 0.745060016 | 0.985094857 | 1.0233377 | up | lncRNA |
| chr2:60376250-60386500- | chr2:60376250-60386500 | 0.488620536 | 0.920777644 | 1.0687956 | up | lncRNA |
| chr2:88735550-88749975+ | chr2:88735550-88749975 | 0.129988881 | 0.682619986 | 1.2305448 | up | lncRNA |
| chr20:24726575-24738550- | chr20:24726575-24738550 | 0.60747834 | 0.956065104 | 1.0708718 | up | lncRNA |
| chr21:20864700-20924525+ | chr21:20864700-20924525 | 0.52520792 | 0.932778625 | 1.0735971 | up | lncRNA |
| chr3:128335025-128366725- | chr3:128335025-128366725 | 0.329109057 | 0.85540421 | 1.135942 | up | lncRNA |
| chr3:188521875-188539200+ | chr3:188521875-188539200 | 0.388621062 | 0.885276359 | 1.154918 | up | lncRNA |
| chr3:45182150-45196400+ | chr3:45182150-45196400 | 0.362038545 | 0.873747871 | 1.1913916 | up | lncRNA |
| chr3:72052025-72073225- | chr3:72052025-72073225 | 0.053121406 | 0.515925762 | 1.502762 | up | lncRNA |
| chr4:103259950-103278600- | chr4:103259950-103278600 | 0.167689794 | 0.723906495 | 1.4141669 | up | lncRNA |
| chr4:4855350-4865400- | chr4:4855350-4865400 | 0.275149026 | 0.832297359 | 1.4488153 | up | lncRNA |
| chr4:60023500-60045050- | chr4:60023500-60045050 | 0.215396041 | 0.783218643 | 1.5793393 | up | lncRNA |
| chr4:92760700-92774625- | chr4:92760700-92774625 | 0.825955375 | 0.993856674 | 1.0542229 | up | lncRNA |
| chr5:158012750-158032025- | chr5:158012750-158032025 | 0.214049275 | 0.781610853 | 1.4506709 | up | lncRNA |
| chr5:170925150-170938100- | chr5:170925150-170938100 | 0.952036885 | 0.998875101 | 1.0114488 | up | lncRNA |
| chr5:3618525-3632100- | chr5:3618525-3632100 | 0.442565337 | 0.90512482 | 1.1813819 | up | lncRNA |
| chr5:3618525-3632100+ | chr5:3618525-3632100 | 0.194053203 | 0.760765035 | 1.1926612 | up | lncRNA |
| chr5:3790400-3802175- | chr5:3790400-3802175 | 0.093053683 | 0.612479847 | 1.1819345 | up | lncRNA |
| chr5:3790400-3802175+ | chr5:3790400-3802175 | 0.006131731 | 0.212479102 | 1.1188612 | up | lncRNA |
| chr5:3881075-3894525+ | chr5:3881075-3894525 | 0.496106196 | 0.921717611 | 1.1437572 | up | lncRNA |
| chr5:67711025-67754775- | chr5:67711025-67754775 | 0.683556144 | 0.977836377 | 1.0506212 | up | lncRNA |
| chr6:125885000-125895925+ | chr6:125885000-125895925 | 0.235118657 | 0.804681389 | 1.4447569 | up | lncRNA |
| chr6:47425050-47444950- | chr6:47425050-47444950 | 0.608794504 | 0.956372451 | 1.078693 | up | lncRNA |
| chr6:48613250-48635850- | chr6:48613250-48635850 | 0.259703967 | 0.824094195 | 1.2884802 | up | lncRNA |
| chr6:63131625-63144250+ | chr6:63131625-63144250 | 0.341093721 | 0.862165742 | 1.1239263 | up | lncRNA |
| chr6:91443800-91462575+ | chr6:91443800-91462575 | 0.248199762 | 0.814856288 | 1.2304296 | up | lncRNA |
| chr7:1314975-1333125- | chr7:1314975-1333125 | 0.060291804 | 0.531246381 | 1.150357 | up | lncRNA |
| chr7:1314975-1333125+ | chr7:1314975-1333125 | 0.34328801 | 0.863514929 | 1.1323103 | up | lncRNA |
| chr8:140577700-140593825+ | chr8:140577700-140593825 | 0.003667209 | 0.175033407 | 1.2505286 | up | lncRNA |
| chr8:20622500-20634450+ | chr8:20622500-20634450 | 0.278585873 | 0.832297359 | 1.1567881 | up | lncRNA |
| chr8:50050850-50118750- | chr8:50050850-50118750 | 0.149584556 | 0.704805498 | 1.1972512 | up | lncRNA |
| chr8:66380600-66391625+ | chr8:66380600-66391625 | 0.778421979 | 0.99121281 | 1.1469234 | up | lncRNA |
| chr8:80224875-80250800- | chr8:80224875-80250800 | 0.803557564 | 0.992578294 | 1.0799811 | up | lncRNA |
| chr8:99411775-99424650+ | chr8:99411775-99424650 | 0.681767995 | 0.977676223 | 1.0422234 | up | lncRNA |
| chr9:110109500-110126925- | chr9:110109500-110126925 | 0.859054666 | 0.996142678 | 1.0306108 | up | lncRNA |
| chrX:115315750-115327875- | chrX:115315750-115327875 | 0.509415082 | 0.926768238 | 1.094272 | up | lncRNA |
| chrX:136212300-136224525- | chrX:136212300-136224525 | 0.039837874 | 0.45449377 | 1.2781492 | up | lncRNA |
| chrX:16349775-16384450- | chrX:16349775-16384450 | 0.531640171 | 0.935101828 | 1.4197608 | up | lncRNA |
| chrX:30716225-30736150- | chrX:30716225-30736150 | 0.076146019 | 0.569191494 | 1.1608387 | up | lncRNA |
| chrX:30716225-30736150+ | chrX:30716225-30736150 | 0.262002077 | 0.82544125 | 1.2195987 | up | lncRNA |
| CN266134 |  | 0.164495558 | 0.719521661 | 1.5566416 | up | lncRNA |
| CN281635 |  | 0.166720197 | 0.722295284 | 1.6062303 | up | lncRNA |
| CN282981 |  | 0.090681462 | 0.604992392 | 1.7578581 | up | lncRNA |
| CN285536 |  | 0.942840997 | 0.998875101 | 1.0079975 | up | lncRNA |
| CN412465 |  | 0.96198711 | 0.998875101 | 1.010712 | up | lncRNA |
| CR744969 |  | 0.176956504 | 0.741512332 | 1.314165 | up | lncRNA |
| CR745936 |  | 0.172436861 | 0.734089358 | 1.0861496 | up | lncRNA |
| CV326886R |  | 0.294100951 | 0.838742488 | 1.186249 | up | lncRNA |
| CX166999 |  | 0.424459698 | 0.89750944 | 1.1151553 | up | lncRNA |
| D38733 |  | 0.625598706 | 0.96133281 | 1.126233 | up | lncRNA |
| DA096519 |  | 0.280626495 | 0.832297359 | 1.3266489 | up | lncRNA |
| DA100307 |  | 0.533649609 | 0.935381352 | 1.212876 | up | lncRNA |
| DA249124 |  | 0.531639366 | 0.935101828 | 1.0219559 | up | lncRNA |
| DA269862 |  | 0.039567823 | 0.454493742 | 1.1204086 | up | lncRNA |
| DA312854 |  | 0.758041724 | 0.988042026 | 1.0487325 | up | lncRNA |
| DA326600 |  | 0.292731174 | 0.837871806 | 1.1515859 | up | lncRNA |
| DA446470 |  | 0.666102756 | 0.97282047 | 1.0473321 | up | lncRNA |
| DA706379 |  | 0.282394613 | 0.832629955 | 1.0735696 | up | lncRNA |
| DA802942 |  | 0.006842214 | 0.220481232 | 1.2518551 | up | lncRNA |
| DA807888 |  | 0.158057941 | 0.714689048 | 1.7025793 | up | lncRNA |
| DA957234 |  | 0.689899552 | 0.97861545 | 1.169263 | up | lncRNA |
| DA962001 |  | 0.86709016 | 0.996767702 | 1.0185047 | up | lncRNA |
| DB025955 |  | 0.884610209 | 0.998875101 | 1.0214963 | up | lncRNA |
| DB049930 |  | 0.500589609 | 0.922148899 | 1.0629897 | up | lncRNA |
| DB123109 |  | 0.162007322 | 0.717077405 | 1.1291468 | up | lncRNA |
| DB163079 |  | 0.136284669 | 0.693269931 | 1.3754853 | up | lncRNA |
| DB224754 |  | 0.083500202 | 0.586585851 | 1.1860743 | up | lncRNA |
| DB297878 |  | 0.577026745 | 0.947948855 | 1.0599947 | up | lncRNA |
| DB308427 |  | 0.144340155 | 0.699853429 | 1.0862249 | up | lncRNA |
| DB338868 |  | 0.473674765 | 0.91743323 | 1.0712971 | up | lncRNA |
| DB339348 |  | 0.816030301 | 0.992651327 | 1.0224037 | up | lncRNA |
| DB378707 |  | 0.957538611 | 0.998875101 | 1.0180472 | up | lncRNA |
| DN831128 |  | 0.843857191 | 0.995583664 | 1.0212353 | up | lncRNA |
| DN831128R |  | 0.466030704 | 0.913821992 | 1.0510027 | up | lncRNA |
| DT216591 |  | 0.043567618 | 0.475753 | 1.1275699 | up | lncRNA |
| DW409420R |  | 0.489054169 | 0.920777644 | 1.0854839 | up | lncRNA |
| EF565097R |  | 0.376048378 | 0.879353561 | 1.1606379 | up | lncRNA |
| EF565124R |  | 0.020859659 | 0.359097712 | 2.3637054 | up | lncRNA |
| ENST00000235290 | DLEU2 | 0.090721972 | 0.604992392 | 1.3824167 | up | lncRNA |
| ENST00000266746 | GOLGA2B | 0.815534201 | 0.99257857 | 1.0298922 | up | lncRNA |
| ENST00000273411 | RPL39P5 | 0.463200852 | 0.913747281 | 1.0651589 | up | lncRNA |
| ENST00000275590 | AC006014.8 | 0.468844292 | 0.914494873 | 1.0926712 | up | lncRNA |
| ENST00000307533 | AC093323.3 | 0.1549203 | 0.711341825 | 1.3747401 | up | lncRNA |
| ENST00000309865 | RP11-368J21.1 | 0.315156325 | 0.851223284 | 1.0799505 | up | lncRNA |
| ENST00000309874 | RP11-23P13.6 | 0.132131528 | 0.687332981 | 1.0787771 | up | lncRNA |
| ENST00000314246 | GOLGA8DP | 0.403079865 | 0.889146721 | 1.674652 | up | lncRNA |
| ENST00000321800 | LINC00469 | 0.63936205 | 0.96564589 | 1.1186662 | up | lncRNA |
| ENST00000325407 | HSP90AA6P | 0.806725055 | 0.992578294 | 1.017419 | up | lncRNA |
| ENST00000325660 | ZNRF3-AS1 | 0.053153583 | 0.515925762 | 1.3649013 | up | lncRNA |
| ENST00000326391 | POLR2J4 | 0.631843592 | 0.964380442 | 1.0421565 | up | lncRNA |
| ENST00000326734 | FAM87B | 0.385306296 | 0.882848407 | 1.1633435 | up | lncRNA |
| ENST00000328344 | LINC00334 | 0.182139449 | 0.74840533 | 1.4040532 | up | lncRNA |
| ENST00000329395 | ATP8B5P | 0.992690037 | 0.999156445 | 1.001241 | up | lncRNA |
| ENST00000330048 | SLC6A10P | 0.608482012 | 0.956231092 | 1.0480706 | up | lncRNA |
| ENST00000331787 | TTTY14 | 0.684336996 | 0.977836377 | 1.011526 | up | lncRNA |
| ENST00000332088 | AL353997.4 | 0.522977983 | 0.932117592 | 1.0964671 | up | lncRNA |
| ENST00000332123 | RP11-178A10.1 | 0.877371417 | 0.998682284 | 1.0189171 | up | lncRNA |
| ENST00000333854 | RP11-181G12.2 | 0.059065879 | 0.52982093 | 1.1415994 | up | lncRNA |
| ENST00000335223 | RP11-56D16.2 | 0.875620935 | 0.998245425 | 1.0255139 | up | lncRNA |
| ENST00000336399 | PLEKHA8P1 | 0.144056678 | 0.699853429 | 1.038611 | up | lncRNA |
| ENST00000338498 | C11orf95 | 0.898342861 | 0.998875101 | 1.0083654 | up | lncRNA |
| ENST00000338711 | FAM86FP | 0.842443734 | 0.995583664 | 1.0164437 | up | lncRNA |
| ENST00000340196 | CCDC144C | 0.073109172 | 0.56397543 | 1.1580937 | up | lncRNA |
| ENST00000340286 | WHAMMP3 | 0.274497329 | 0.832297359 | 1.1306103 | up | lncRNA |
| ENST00000340779 | RP11-797H7.5 | 0.596938549 | 0.952062956 | 1.3262833 | up | lncRNA |
| ENST00000342892 | AC005042.4 | 0.38211366 | 0.881294633 | 1.1846957 | up | lncRNA |
| ENST00000354527 | RP11-57C13.3 | 0.328388087 | 0.854893129 | 1.1150002 | up | lncRNA |
| ENST00000356047 | AC092415.1 | 0.503513463 | 0.92405258 | 1.1940171 | up | lncRNA |
| ENST00000356133 | RP11-208N14.4 | 0.967288489 | 0.998875101 | 1.013844 | up | lncRNA |
| ENST00000356555 | ZNF300P1 | 0.082311608 | 0.585414993 | 1.3628653 | up | lncRNA |
| ENST00000356672 | AC025165.8 | 0.744035917 | 0.985094857 | 1.027545 | up | lncRNA |
| ENST00000356684 | FLVCR1-AS1 | 0.469623088 | 0.91468974 | 1.5184359 | up | lncRNA |
| ENST00000357671 | ARID4B-IT1 | 0.104858831 | 0.635942989 | 1.2279865 | up | lncRNA |
| ENST00000358393 | LINC00176 | 0.745419749 | 0.985094857 | 1.0302588 | up | lncRNA |
| ENST00000359455 | LINC00615 | 0.325236374 | 0.854556781 | 1.2726382 | up | lncRNA |
| ENST00000359859 | TMEM191A | 0.860269163 | 0.996142678 | 1.0397304 | up | lncRNA |
| ENST00000360899 | LINC00523 | 0.189105548 | 0.75621143 | 1.2219588 | up | lncRNA |
| ENST00000366152 | RP11-277A4.4 | 0.003448881 | 0.170097393 | 1.5655494 | up | lncRNA |
| ENST00000366181 | RP11-296A18.3 | 0.99531382 | 0.999156445 | 1.0007177 | up | lncRNA |
| ENST00000366185 | RP11-258C19.5 | 0.179457155 | 0.744893635 | 1.5951445 | up | lncRNA |
| ENST00000366189 | RP11-423H2.3 | 0.548041008 | 0.940847434 | 1.1026576 | up | lncRNA |
| ENST00000366209 | AC009499.1 | 0.05985767 | 0.531246381 | 1.4467557 | up | lncRNA |
| ENST00000366224 | RP11-571E6.3 | 0.229165159 | 0.798130144 | 1.2203644 | up | lncRNA |
| ENST00000366234 | PCBP1-AS1 | 0.289083672 | 0.837264328 | 1.0938762 | up | lncRNA |
| ENST00000366264 | AC034193.5 | 0.8731902 | 0.997909851 | 1.0454835 | up | lncRNA |
| ENST00000366273 | RP11-288H12.3 | 0.344223503 | 0.864204712 | 1.0820968 | up | lncRNA |
| ENST00000366375 | RP11-570D4.2 | 0.256549459 | 0.822133742 | 1.7383225 | up | lncRNA |
| ENST00000366441 | AC093415.2 | 0.781818074 | 0.99121281 | 1.0606953 | up | lncRNA |
| ENST00000366446 | RP11-322M19.1 | 0.124126036 | 0.675318444 | 1.1299981 | up | lncRNA |
| ENST00000369027 | RP11-10J18.3 | 0.385700964 | 0.883149368 | 1.0304714 | up | lncRNA |
| ENST00000369120 | BVES-AS1 | 0.006497285 | 0.217096533 | 1.1362698 | up | lncRNA |
| ENST00000369843 | RP5-1160K1.6 | 0.141300962 | 0.698568819 | 1.1791199 | up | lncRNA |
| ENST00000369884 | CYP17A1-AS1 | 0.132272244 | 0.687815669 | 1.1053238 | up | lncRNA |
| ENST00000370257 | RP11-261N11.8 | 0.055897123 | 0.521270634 | 1.2401364 | up | lncRNA |
| ENST00000373171 | RP11-552E20.3 | 0.678453574 | 0.976096237 | 1.054486 | up | lncRNA |
| ENST00000375465 | BPIFA4P | 0.430335492 | 0.898685776 | 1.1369209 | up | lncRNA |
| ENST00000376482 | AC073842.19 | 0.006537466 | 0.217096533 | 1.2573994 | up | lncRNA |
| ENST00000377614 | RP11-111F5.5 | 0.916031266 | 0.998875101 | 1.0242468 | up | lncRNA |
| ENST00000377662 | BTN2A3P | 0.385321043 | 0.882848407 | 1.1695726 | up | lncRNA |
| ENST00000378432 | CTC-425O23.2 | 0.793956306 | 0.991420185 | 1.0264891 | up | lncRNA |
| ENST00000378769 | ABHD11-AS2 | 0.876162564 | 0.998411428 | 1.0114896 | up | lncRNA |
| ENST00000379050 | TPT1-AS1 | 0.594156656 | 0.951072365 | 1.0602158 | up | lncRNA |
| ENST00000379317 | TTLL10-AS1 | 0.41630903 | 0.892649611 | 1.0942247 | up | lncRNA |
| ENST00000380387 | AC013472.3 | 0.553789164 | 0.943023022 | 1.1859549 | up | lncRNA |
| ENST00000380722 | RP11-497E19.1 | 0.544796503 | 0.939697392 | 1.2606309 | up | lncRNA |
| ENST00000380981 | WI2-81516E3.1 | 0.892960184 | 0.998875101 | 1.0160366 | up | lncRNA |
| ENST00000381181 | AP000569.2 | 0.649564541 | 0.969927925 | 1.0834512 | up | lncRNA |
| ENST00000381790 | RP11-319G6.1 | 0.029723992 | 0.413940496 | 1.2024398 | up | lncRNA |
| ENST00000382007 | AC024132.1 | 0.119184416 | 0.66739553 | 1.6226972 | up | lncRNA |
| ENST00000382215 | ABC12-49244600F4.3 | 0.170997361 | 0.732321722 | 1.2033954 | up | lncRNA |
| ENST00000383038 | AP000525.8 | 0.930457348 | 0.998875101 | 1.0504002 | up | lncRNA |
| ENST00000388970 | AFG3L1P | 0.349900121 | 0.867400431 | 1.0909202 | up | lncRNA |
| ENST00000389600 | GUCY1B2 | 0.570234317 | 0.944496311 | 1.0897527 | up | lncRNA |
| ENST00000390681 | PRSS30P | 0.101780589 | 0.632351488 | 1.1194031 | up | lncRNA |
| ENST00000391394 | RP11-551L14.1 | 0.995381373 | 0.999156445 | 1.0039705 | up | lncRNA |
| ENST00000391707 | PPP2R3B-AS1 | 0.539760683 | 0.937437834 | 1.3580872 | up | lncRNA |
| ENST00000394517 | RP11-465B22.3 | 0.231578894 | 0.799139284 | 1.1683794 | up | lncRNA |
| ENST00000394662 | GBP1P1 | 0.034965208 | 0.430429421 | 1.6887842 | up | lncRNA |
| ENST00000395215 | RP11-24M17.5 | 0.427072611 | 0.897780553 | 1.0571792 | up | lncRNA |
| ENST00000395675 | FOXO3B | 0.507839764 | 0.926113887 | 1.0555324 | up | lncRNA |
| ENST00000396588 | AC097662.2 | 0.412867654 | 0.890927603 | 1.3184133 | up | lncRNA |
| ENST00000396645 | HMGN2P46 | 0.766942788 | 0.989189555 | 1.1058455 | up | lncRNA |
| ENST00000397184 | AP000692.9 | 0.006191029 | 0.212479102 | 1.3949071 | up | lncRNA |
| ENST00000397264 | RP11-536K7.3 | 0.734441545 | 0.983763148 | 1.208592 | up | lncRNA |
| ENST00000398173 | AC011491.6 | 0.737271663 | 0.984316636 | 1.041467 | up | lncRNA |
| ENST00000398460 | MEG3 | 0.046603692 | 0.48744626 | 1.094499 | up | lncRNA |
| ENST00000398461 | MEG3 | 0.565358892 | 0.943572364 | 1.0681478 | up | lncRNA |
| ENST00000398669 | RP11-989E6.3 | 0.067000262 | 0.547601226 | 1.2225085 | up | lncRNA |
| ENST00000398777 | RP11-16N2.1 | 0.253059061 | 0.819692089 | 1.2036462 | up | lncRNA |
| ENST00000398957 | RP11-717D12.1 | 0.598684669 | 0.952796892 | 1.0978732 | up | lncRNA |
| ENST00000399003 | RP11-1036E20.9 | 0.503809815 | 0.92405258 | 1.1302266 | up | lncRNA |
| ENST00000399070 | ZNF271 | 0.894771018 | 0.998875101 | 1.0165722 | up | lncRNA |
| ENST00000399186 | XXyac-YM21GA2.7 | 0.212048393 | 0.780026858 | 1.1500881 | up | lncRNA |
| ENST00000399711 | LINC00269 | 0.76101761 | 0.988045734 | 1.047128 | up | lncRNA |
| ENST00000400178 | LINC00478 | 0.811661264 | 0.99257857 | 1.0245639 | up | lncRNA |
| ENST00000400418 | CES5AP1 | 0.174616413 | 0.736164062 | 1.2367102 | up | lncRNA |
| ENST00000400432 | RP11-172E9.2 | 0.339171947 | 0.860518281 | 1.2193978 | up | lncRNA |
| ENST00000400461 | FER1L4 | 0.439657364 | 0.9037775 | 1.0567844 | up | lncRNA |
| ENST00000400581 | TTTY5 | 0.050778701 | 0.508649264 | 1.118098 | up | lncRNA |
| ENST00000400768 | AC114730.8 | 0.392371877 | 0.885711362 | 1.1152407 | up | lncRNA |
| ENST00000400892 | NPPA-AS1 | 0.245137211 | 0.811577674 | 1.1961435 | up | lncRNA |
| ENST00000400902 | RP11-703H8.7 | 0.711781395 | 0.98251396 | 1.027079 | up | lncRNA |
| ENST00000401014 | NBPF7 | 0.108724185 | 0.642195263 | 1.3741022 | up | lncRNA |
| ENST00000403324 | AC093642.5 | 0.204046163 | 0.771026327 | 1.2166462 | up | lncRNA |
| ENST00000403651 | RAET1K | 0.071150975 | 0.559511998 | 1.1239048 | up | lncRNA |
| ENST00000404689 | RP11-127B16.1 | 0.46880843 | 0.914494873 | 1.2363264 | up | lncRNA |
| ENST00000407767 | RP3-406A7.3 | 0.333367971 | 0.857704692 | 1.0453739 | up | lncRNA |
| ENST00000407889 | RP11-96L14.7 | 0.727464378 | 0.98251396 | 1.1528854 | up | lncRNA |
| ENST00000409054 | LINC00152 | 0.574178395 | 0.946652306 | 1.2569611 | up | lncRNA |
| ENST00000409590 | AC007131.1 | 0.855312646 | 0.996142678 | 1.017484 | up | lncRNA |
| ENST00000411427 | AP001610.5 | 0.901313688 | 0.998875101 | 1.0460044 | up | lncRNA |
| ENST00000411431 | RP11-569A11.2 | 0.825859085 | 0.993856674 | 1.0555545 | up | lncRNA |
| ENST00000411451 | RP11-308N19.4 | 0.357981466 | 0.872424004 | 1.1201278 | up | lncRNA |
| ENST00000411453 | RP11-112L6.4 | 0.439472203 | 0.9037775 | 1.1338892 | up | lncRNA |
| ENST00000411489 | RP1-86D1.4 | 0.005785584 | 0.208598143 | 2.0229336 | up | lncRNA |
| ENST00000411525 | LINC00422 | 0.116709495 | 0.662785634 | 1.0933932 | up | lncRNA |
| ENST00000411579 | CTD-3184A7.4 | 0.622328145 | 0.960457826 | 1.0603592 | up | lncRNA |
| ENST00000411694 | AP001055.6 | 0.000683805 | 0.094750704 | 4.8900572 | up | lncRNA |
| ENST00000411738 | AC159540.1 | 0.476270062 | 0.917441334 | 1.1036225 | up | lncRNA |
| ENST00000411759 | AC019349.5 | 0.947303871 | 0.998875101 | 1.008002 | up | lncRNA |
| ENST00000411804 | RP1-91G5.3 | 0.011702679 | 0.275339088 | 1.1899881 | up | lncRNA |
| ENST00000411815 | RP11-4M23.3 | 0.700699057 | 0.980170937 | 1.2287883 | up | lncRNA |
| ENST00000411824 | RP11-93B14.5 | 0.633247145 | 0.964607725 | 1.0448476 | up | lncRNA |
| ENST00000411903 | RP4-598G3.1 | 0.298128783 | 0.841354434 | 1.0644132 | up | lncRNA |
| ENST00000411904 | RP11-251M1.1 | 0.086582044 | 0.597129025 | 1.1272134 | up | lncRNA |
| ENST00000411909 | AZGP1P1 | 0.434617541 | 0.901001147 | 1.0942536 | up | lncRNA |
| ENST00000411941 | AL592284.1 | 0.205604385 | 0.772818733 | 1.6784176 | up | lncRNA |
| ENST00000411989 | LINC00114 | 0.581428134 | 0.949239022 | 1.0820957 | up | lncRNA |
| ENST00000412017 | AL589743.1 | 0.912905647 | 0.998875101 | 1.0889816 | up | lncRNA |
| ENST00000412060 | Z83851.1 | 0.381652037 | 0.881294633 | 1.0280029 | up | lncRNA |
| ENST00000412084 | AC091814.2 | 0.759853838 | 0.988045734 | 1.0843941 | up | lncRNA |
| ENST00000412119 | LINC00354 | 0.136485973 | 0.693269931 | 1.6404927 | up | lncRNA |
| ENST00000412143 | PSORS1C3 | 0.231921869 | 0.79936183 | 1.7699678 | up | lncRNA |
| ENST00000412154 | HERC2P3 | 0.540369392 | 0.937772856 | 1.1023726 | up | lncRNA |
| ENST00000412192 | CRYGEP | 0.337411812 | 0.859588936 | 1.0363213 | up | lncRNA |
| ENST00000412194 | BX248398.1 | 0.098805305 | 0.624143367 | 2.3662684 | up | lncRNA |
| ENST00000412197 | AC091705.1 | 0.346892955 | 0.865873516 | 1.1683917 | up | lncRNA |
| ENST00000412203 | LEPREL1-AS1 | 0.08424218 | 0.589155194 | 1.1207271 | up | lncRNA |
| ENST00000412204 | BX571672.2 | 0.479827252 | 0.918546036 | 1.0617765 | up | lncRNA |
| ENST00000412228 | RP1-140A9.1 | 0.404774765 | 0.889198786 | 1.0613681 | up | lncRNA |
| ENST00000412242 | KDM5C-IT1 | 0.025657178 | 0.38237988 | 1.3777122 | up | lncRNA |
| ENST00000412266 | AC006028.9 | 0.498026156 | 0.921805853 | 1.1056814 | up | lncRNA |
| ENST00000412294 | AC096559.1 | 0.481747983 | 0.9192486 | 1.204755 | up | lncRNA |
| ENST00000412295 | CTC-338M12.9 | 0.527131067 | 0.934113479 | 1.1375863 | up | lncRNA |
| ENST00000412357 | RP5-1114G22.2 | 0.059382677 | 0.530697632 | 1.4319218 | up | lncRNA |
| ENST00000412362 | RP11-236B18.2 | 0.347563698 | 0.866313684 | 1.0853613 | up | lncRNA |
| ENST00000412393 | LINC00342 | 0.003930884 | 0.179554598 | 1.1600717 | up | lncRNA |
| ENST00000412405 | RP4-734C18.1 | 0.557837331 | 0.943023022 | 1.1407503 | up | lncRNA |
| ENST00000412410 | AC073071.1 | 0.131065611 | 0.686015191 | 1.3022402 | up | lncRNA |
| ENST00000412436 | RP13-228J13.1 | 0.364983653 | 0.874818743 | 1.5635898 | up | lncRNA |
| ENST00000412439 | RP11-810B23.1 | 0.750193802 | 0.98551982 | 1.0496652 | up | lncRNA |
| ENST00000412497 | RP11-314N13.3 | 0.245031836 | 0.811577674 | 1.1576136 | up | lncRNA |
| ENST00000412549 | AC009542.2 | 0.055582438 | 0.520364745 | 1.2966523 | up | lncRNA |
| ENST00000412563 | AC007091.1 | 0.239297975 | 0.808658327 | 1.3561233 | up | lncRNA |
| ENST00000412595 | RP11-472B18.2 | 0.447429333 | 0.90584085 | 1.1578957 | up | lncRNA |
| ENST00000412602 | RP11-557H15.5 | 0.324412073 | 0.853958969 | 1.2289506 | up | lncRNA |
| ENST00000412701 | RP11-533O20.2 | 0.310443735 | 0.848664472 | 1.1500383 | up | lncRNA |
| ENST00000412712 | AC011747.7 | 0.91382997 | 0.998875101 | 1.0284001 | up | lncRNA |
| ENST00000412740 | AC092296.1 | 0.425645556 | 0.897780553 | 1.0457608 | up | lncRNA |
| ENST00000412754 | RP11-126L15.4 | 0.433902597 | 0.900875689 | 1.1005278 | up | lncRNA |
| ENST00000412772 | RP11-134P9.3 | 0.49428735 | 0.921717611 | 1.1385692 | up | lncRNA |
| ENST00000412804 | AC024168.2 | 0.746423969 | 0.985094857 | 1.0268175 | up | lncRNA |
| ENST00000412871 | RP11-354K1.1 | 0.225000418 | 0.794551181 | 1.3282261 | up | lncRNA |
| ENST00000412906 | ZNF295-AS1 | 0.575287343 | 0.946770106 | 1.2171495 | up | lncRNA |
| ENST00000412946 | TPT1-AS1 | 0.262036472 | 0.82544125 | 1.5480252 | up | lncRNA |
| ENST00000412951 | AC012462.1 | 0.011893973 | 0.27801677 | 1.461931 | up | lncRNA |
| ENST00000412954 | RP1-283K11.3 | 0.307601067 | 0.847137912 | 1.1060687 | up | lncRNA |
| ENST00000413005 | RP1-79C4.1 | 0.38503463 | 0.882848407 | 1.8311493 | up | lncRNA |
| ENST00000413035 | IPO9-AS1 | 0.997244366 | 0.999193741 | 1.0002396 | up | lncRNA |
| ENST00000413069 | PCBP1-AS1 | 0.331607296 | 0.856326799 | 1.1270846 | up | lncRNA |
| ENST00000413074 | RP11-378J18.3 | 0.734521271 | 0.983763148 | 1.0357493 | up | lncRNA |
| ENST00000413076 | RP13-507I23.1 | 0.41786412 | 0.893502063 | 1.2251944 | up | lncRNA |
| ENST00000413077 | AC012146.7 | 0.769238432 | 0.989189555 | 1.0196128 | up | lncRNA |
| ENST00000413103 | RP11-148B18.1 | 0.720739564 | 0.98251396 | 1.1137124 | up | lncRNA |
| ENST00000413202 | LINC00152 | 0.150451687 | 0.705518742 | 1.6257324 | up | lncRNA |
| ENST00000413220 | RP11-227H15.5 | 0.287504271 | 0.836961572 | 1.2486902 | up | lncRNA |
| ENST00000413231 | RP11-389O22.1 | 0.471101821 | 0.915538705 | 1.2090331 | up | lncRNA |
| ENST00000413254 | AC009228.1 | 0.250802013 | 0.817015761 | 1.5005649 | up | lncRNA |
| ENST00000413311 | AC093382.1 | 0.011058035 | 0.266400549 | 2.0021465 | up | lncRNA |
| ENST00000413339 | RP11-464C19.2 | 0.220665792 | 0.790478735 | 1.1842302 | up | lncRNA |
| ENST00000413347 | SNAP47-AS1 | 0.5263025 | 0.93359637 | 1.0643292 | up | lncRNA |
| ENST00000413352 | RP11-375O18.2 | 0.865358774 | 0.996767702 | 1.0482127 | up | lncRNA |
| ENST00000413436 | PCBP1-AS1 | 0.516590162 | 0.930031728 | 1.1815193 | up | lncRNA |
| ENST00000413451 | RP5-930J4.4 | 0.402770807 | 0.889146721 | 1.0615999 | up | lncRNA |
| ENST00000413452 | DGUOK-AS1 | 0.00086861 | 0.104758688 | 10.2108731 | up | lncRNA |
| ENST00000413483 | AC129929.5 | 0.029381101 | 0.411392746 | 1.6297144 | up | lncRNA |
| ENST00000413501 | RP11-165I9.4 | 0.711345031 | 0.98251396 | 1.1017588 | up | lncRNA |
| ENST00000413567 | AC083867.4 | 0.849979067 | 0.995900317 | 1.0578773 | up | lncRNA |
| ENST00000413669 | AC009236.1 | 0.389833031 | 0.885317362 | 1.1687716 | up | lncRNA |
| ENST00000413721 | LINC00313 | 0.183734654 | 0.751257244 | 1.6418132 | up | lncRNA |
| ENST00000413791 | PCBP1-AS1 | 0.001631655 | 0.135515114 | 1.6453023 | up | lncRNA |
| ENST00000413848 | AC005037.3 | 0.159859908 | 0.714806047 | 1.3472412 | up | lncRNA |
| ENST00000413913 | RP11-229P13.19 | 0.504949331 | 0.924602295 | 1.0877901 | up | lncRNA |
| ENST00000413954 | AC064871.3 | 0.842665203 | 0.995583664 | 1.0468548 | up | lncRNA |
| ENST00000413961 | RP11-322M19.1 | 0.054596322 | 0.51754717 | 1.2778788 | up | lncRNA |
| ENST00000413986 | RP3-512E2.2 | 0.321242104 | 0.852662264 | 1.2074028 | up | lncRNA |
| ENST00000413989 | AC009228.1 | 0.051244165 | 0.509318737 | 1.1455138 | up | lncRNA |
| ENST00000414006 | RP11-429G19.2 | 0.358203775 | 0.872424004 | 1.0809099 | up | lncRNA |
| ENST00000414030 | LINC00152 | 0.016155213 | 0.321135203 | 1.4547877 | up | lncRNA |
| ENST00000414039 | RP11-148B18.4 | 0.187005635 | 0.753717042 | 1.481105 | up | lncRNA |
| ENST00000414065 | AC018685.1 | 0.559175508 | 0.943152767 | 1.1373451 | up | lncRNA |
| ENST00000414086 | AC096559.2 | 0.965105425 | 0.998875101 | 1.0100344 | up | lncRNA |
| ENST00000414106 | RP11-140A10.3 | 0.701279956 | 0.980170937 | 1.0901749 | up | lncRNA |
| ENST00000414120 | RP11-513G11.1 | 0.554651855 | 0.943023022 | 1.0533723 | up | lncRNA |
| ENST00000414126 | AC073316.2 | 0.904545361 | 0.998875101 | 1.0146045 | up | lncRNA |
| ENST00000414128 | RP11-108M9.2 | 0.631142512 | 0.964380442 | 1.1828809 | up | lncRNA |
| ENST00000414189 | AP001422.3 | 0.403367024 | 0.889146721 | 1.1100492 | up | lncRNA |
| ENST00000414195 | AC074093.1 | 0.265712489 | 0.828662678 | 1.4440373 | up | lncRNA |
| ENST00000414201 | RP11-196P2.1 | 0.690485179 | 0.97861545 | 1.1649456 | up | lncRNA |
| ENST00000414203 | RP5-1170K4.7 | 0.470516303 | 0.915388501 | 1.2089197 | up | lncRNA |
| ENST00000414227 | AC000120.7 | 0.262035515 | 0.82544125 | 1.2933876 | up | lncRNA |
| ENST00000414282 | SNHG7 | 0.004514796 | 0.188910659 | 1.3338124 | up | lncRNA |
| ENST00000414295 | RP11-264A11.1 | 0.028468013 | 0.402931873 | 1.6775304 | up | lncRNA |
| ENST00000414344 | AL592284.1 | 0.005773066 | 0.208598143 | 1.3994113 | up | lncRNA |
| ENST00000414404 | AC078941.1 | 0.307138493 | 0.846887924 | 1.3091865 | up | lncRNA |
| ENST00000414407 | LINC00398 | 0.848748548 | 0.995876116 | 1.0145206 | up | lncRNA |
| ENST00000414416 | LINC00116 | 0.668235946 | 0.97405264 | 1.0771518 | up | lncRNA |
| ENST00000414483 | LL22NC03-121E8.4 | 0.503482853 | 0.92405258 | 1.0251205 | up | lncRNA |
| ENST00000414529 | AC092937.2 | 0.756738391 | 0.98762882 | 1.0234282 | up | lncRNA |
| ENST00000414532 | RP5-1166H10.2 | 0.212331069 | 0.780026858 | 1.136988 | up | lncRNA |
| ENST00000414538 | AC092687.5 | 0.899484026 | 0.998875101 | 1.0139675 | up | lncRNA |
| ENST00000414548 | CTD-2192J16.11 | 0.698773665 | 0.980170937 | 1.1363083 | up | lncRNA |
| ENST00000414600 | AC005152.2 | 0.392606674 | 0.885711362 | 1.1012016 | up | lncRNA |
| ENST00000414640 | RP5-956O18.3 | 0.844519215 | 0.995583664 | 1.0630574 | up | lncRNA |
| ENST00000414659 | MCM3AP-AS1 | 0.334792088 | 0.858024294 | 1.1720037 | up | lncRNA |
| ENST00000414676 | RP5-1103G7.4 | 0.41330223 | 0.890927603 | 1.6306314 | up | lncRNA |
| ENST00000414686 | RP11-421L21.2 | 0.799511119 | 0.992228977 | 1.0287528 | up | lncRNA |
| ENST00000414699 | AL773572.7 | 0.572155096 | 0.945484468 | 1.1808707 | up | lncRNA |
| ENST00000414744 | SPAG5-AS1 | 0.280758591 | 0.832297359 | 1.1942865 | up | lncRNA |
| ENST00000414776 | AL450226.2 | 0.91749631 | 0.998875101 | 1.0385166 | up | lncRNA |
| ENST00000414795 | AC017076.5 | 0.454659018 | 0.910032467 | 1.0935381 | up | lncRNA |
| ENST00000414796 | AC019064.1 | 0.00390226 | 0.179554598 | 2.331208 | up | lncRNA |
| ENST00000414797 | MAGI2-AS3 | 0.951402986 | 0.998875101 | 1.0084515 | up | lncRNA |
| ENST00000414828 | RP1-163M9.6 | 0.16647452 | 0.722043613 | 1.1294394 | up | lncRNA |
| ENST00000414844 | PLCL2-AS1 | 0.756095846 | 0.98762882 | 1.0455128 | up | lncRNA |
| ENST00000414877 | LINC00159 | 0.026942937 | 0.390590948 | 2.01421 | up | lncRNA |
| ENST00000414890 | RABGAP1L-IT1 | 0.034883584 | 0.43017068 | 1.1581841 | up | lncRNA |
| ENST00000414896 | AC005104.3 | 0.693284245 | 0.978978277 | 1.0721222 | up | lncRNA |
| ENST00000414915 | AC023669.1 | 0.327185377 | 0.854556781 | 1.1886011 | up | lncRNA |
| ENST00000414927 | RP11-307B6.3 | 0.005399318 | 0.204219613 | 1.2841385 | up | lncRNA |
| ENST00000414992 | DCUN1D2-AS2 | 0.672567445 | 0.974876341 | 1.0422278 | up | lncRNA |
| ENST00000415000 | RP11-280O1.2 | 0.699952146 | 0.980170937 | 1.048263 | up | lncRNA |
| ENST00000415054 | RP1-85F18.6 | 0.509712279 | 0.926820047 | 1.1042471 | up | lncRNA |
| ENST00000415062 | RP11-426A6.5 | 0.737040791 | 0.984316636 | 1.0835238 | up | lncRNA |
| ENST00000415101 | RP11-402G3.5 | 0.972139736 | 0.998875101 | 1.0052739 | up | lncRNA |
| ENST00000415104 | RP11-666A1.5 | 0.269693134 | 0.830995087 | 1.1948931 | up | lncRNA |
| ENST00000415106 | RP11-397G17.1 | 0.505291768 | 0.924770972 | 1.2996591 | up | lncRNA |
| ENST00000415121 | TPTEP1 | 0.910201859 | 0.998875101 | 1.1174103 | up | lncRNA |
| ENST00000415124 | AC113189.5 | 0.279242482 | 0.832297359 | 1.0836884 | up | lncRNA |
| ENST00000415129 | AC017104.6 | 0.983589701 | 0.998875101 | 1.0028608 | up | lncRNA |
| ENST00000415141 | RP11-203J24.8 | 0.40509288 | 0.889198786 | 1.1463581 | up | lncRNA |
| ENST00000415159 | AC007179.1 | 0.526992817 | 0.933983812 | 1.2855189 | up | lncRNA |
| ENST00000415194 | RP1-225E12.2 | 0.618795633 | 0.95998809 | 1.03336 | up | lncRNA |
| ENST00000415212 | CFL1P1 | 0.872525547 | 0.997909851 | 1.0188468 | up | lncRNA |
| ENST00000415218 | RP11-12M5.3 | 0.848975816 | 0.995876116 | 1.0075408 | up | lncRNA |
| ENST00000415294 | LINC00343 | 0.088106583 | 0.598724282 | 1.2399822 | up | lncRNA |
| ENST00000415356 | AC007349.5 | 0.763037942 | 0.988817776 | 1.0533535 | up | lncRNA |
| ENST00000415358 | RP11-184A2.2 | 0.158047811 | 0.714689048 | 1.5314476 | up | lncRNA |
| ENST00000415391 | RP11-544A12.4 | 0.909048368 | 0.998875101 | 1.0287376 | up | lncRNA |
| ENST00000415394 | RP1-241P17.1 | 0.595165145 | 0.951199074 | 1.1695821 | up | lncRNA |
| ENST00000415405 | TTTY7B | 0.185293654 | 0.751649101 | 1.2089598 | up | lncRNA |
| ENST00000415441 | XXyac-YM21GA2.6 | 0.996760075 | 0.999156445 | 1.0005912 | up | lncRNA |
| ENST00000415451 | AC091633.3 | 0.153889276 | 0.711341825 | 1.1417338 | up | lncRNA |
| ENST00000415471 | RP11-100G15.10 | 0.286971403 | 0.836961572 | 1.2547943 | up | lncRNA |
| ENST00000415503 | AC006547.13 | 0.059810027 | 0.531246381 | 1.4711427 | up | lncRNA |
| ENST00000415506 | AC073254.1 | 0.832984744 | 0.994074191 | 1.0483063 | up | lncRNA |
| ENST00000415556 | AC137723.5 | 0.55692964 | 0.943023022 | 1.1254712 | up | lncRNA |
| ENST00000415590 | SFTA1P | 0.65251642 | 0.969927925 | 1.3717579 | up | lncRNA |
| ENST00000415628 | AC012507.3 | 0.087708796 | 0.598469221 | 1.1311006 | up | lncRNA |
| ENST00000415637 | RP5-1018K9.1 | 0.622863777 | 0.960457826 | 1.0922757 | up | lncRNA |
| ENST00000415647 | RP11-46A10.4 | 0.041058446 | 0.462853115 | 1.3379587 | up | lncRNA |
| ENST00000415657 | AC097517.2 | 0.019147348 | 0.344796409 | 1.9232639 | up | lncRNA |
| ENST00000415661 | RP11-464F9.19 | 0.513649643 | 0.929152972 | 1.0622267 | up | lncRNA |
| ENST00000415663 | RP3-445N2.1 | 0.104090156 | 0.634228154 | 1.2175117 | up | lncRNA |
| ENST00000415702 | CTA-397C4.2 | 0.890057224 | 0.998875101 | 1.013732 | up | lncRNA |
| ENST00000415714 | RP1-60O19.1 | 0.153233497 | 0.709879648 | 1.1949381 | up | lncRNA |
| ENST00000415746 | RP11-432J9.3 | 0.6890439 | 0.97861545 | 1.0588861 | up | lncRNA |
| ENST00000415836 | RP11-1217F2.15 | 0.708203472 | 0.981606358 | 1.0364234 | up | lncRNA |
| ENST00000415872 | RP11-262H14.3 | 0.213876022 | 0.781610853 | 1.1229401 | up | lncRNA |
| ENST00000415918 | AC078851.1 | 0.229534276 | 0.79852974 | 1.1330588 | up | lncRNA |
| ENST00000415934 | AC007036.4 | 0.054993452 | 0.519180132 | 1.6086499 | up | lncRNA |
| ENST00000415963 | DNM1P46 | 0.756558164 | 0.98762882 | 1.0484572 | up | lncRNA |
| ENST00000415964 | RP11-73O6.3 | 0.892219191 | 0.998875101 | 1.01491 | up | lncRNA |
| ENST00000415982 | RP13-131K19.1 | 0.94917984 | 0.998875101 | 1.0233991 | up | lncRNA |
| ENST00000416017 | RP4-612J11.1 | 0.77307302 | 0.99026518 | 1.042094 | up | lncRNA |
| ENST00000416022 | LENG8-AS1 | 0.012262255 | 0.282031859 | 1.3999727 | up | lncRNA |
| ENST00000416028 | RP11-119F7.4 | 0.906575212 | 0.998875101 | 1.0149108 | up | lncRNA |
| ENST00000416057 | AC108868.4 | 0.072517641 | 0.562580097 | 1.1270824 | up | lncRNA |
| ENST00000416067 | MAFIPL | 0.852677194 | 0.995900317 | 1.0201547 | up | lncRNA |
| ENST00000416099 | RP11-552M11.4 | 0.349956634 | 0.867400431 | 1.0695916 | up | lncRNA |
| ENST00000416119 | RP11-240D10.4 | 0.054140831 | 0.516622604 | 1.9735328 | up | lncRNA |
| ENST00000416220 | AC018642.1 | 0.4647653 | 0.913821992 | 1.0662794 | up | lncRNA |
| ENST00000416221 | RP11-295G20.2 | 0.346240263 | 0.865832425 | 1.2394393 | up | lncRNA |
| ENST00000416229 | AC007040.6 | 0.607043123 | 0.956038292 | 1.0763734 | up | lncRNA |
| ENST00000416242 | RP11-143M1.4 | 0.117576773 | 0.664878585 | 1.6909668 | up | lncRNA |
| ENST00000416330 | XIST | 0.499080956 | 0.921805853 | 1.0630443 | up | lncRNA |
| ENST00000416351 | RP11-169O17.5 | 0.982804833 | 0.998875101 | 1.0053968 | up | lncRNA |
| ENST00000416361 | AC068039.4 | 0.413494279 | 0.890927603 | 1.2280002 | up | lncRNA |
| ENST00000416406 | RP3-508I15.14 | 0.00505834 | 0.198896715 | 2.1393367 | up | lncRNA |
| ENST00000416416 | RP11-576I22.2 | 0.622500301 | 0.960457826 | 1.4768763 | up | lncRNA |
| ENST00000416432 | NUCB1-AS1 | 0.117843328 | 0.66559915 | 1.1955273 | up | lncRNA |
| ENST00000416438 | LINC00475 | 0.019874434 | 0.3518771 | 1.3446015 | up | lncRNA |
| ENST00000416455 | RP11-49O14.2 | 0.033350701 | 0.42757611 | 1.6598088 | up | lncRNA |
| ENST00000416507 | RP11-787B4.2 | 0.189906819 | 0.756884941 | 1.0841677 | up | lncRNA |
| ENST00000416527 | BX004987.3 | 0.72458434 | 0.98251396 | 1.1341585 | up | lncRNA |
| ENST00000416554 | RP5-1092A11.2 | 0.029725249 | 0.413940496 | 1.5059271 | up | lncRNA |
| ENST00000416578 | RP5-1198E17.1 | 0.993175515 | 0.999156445 | 1.0017952 | up | lncRNA |
| ENST00000416595 | RP3-425P12.1 | 0.712223288 | 0.98251396 | 1.0343493 | up | lncRNA |
| ENST00000416643 | AC005488.13 | 0.135001381 | 0.693269931 | 1.225215 | up | lncRNA |
| ENST00000416646 | RP11-347D21.2 | 0.781789258 | 0.99121281 | 1.0384727 | up | lncRNA |
| ENST00000416648 | RP11-315J22.5 | 0.552850865 | 0.943013504 | 1.0849255 | up | lncRNA |
| ENST00000416684 | DDX39B-AS1 | 0.070946057 | 0.559359114 | 1.4644371 | up | lncRNA |
| ENST00000416689 | RP5-848E13.3 | 0.058310038 | 0.527325561 | 2.0259692 | up | lncRNA |
| ENST00000416725 | AP003039.3 | 0.589088271 | 0.950589384 | 1.0851843 | up | lncRNA |
| ENST00000416764 | RP11-443P15.2 | 0.140630135 | 0.698568819 | 1.2200179 | up | lncRNA |
| ENST00000416860 | RP3-395M20.8 | 0.528270163 | 0.934238148 | 1.0769364 | up | lncRNA |
| ENST00000416902 | USP17L7 | 0.376298162 | 0.879423449 | 1.2664782 | up | lncRNA |
| ENST00000416903 | RP3-477O4.14 | 0.140877188 | 0.698568819 | 1.1888316 | up | lncRNA |
| ENST00000416989 | RP11-493K23.1 | 0.035293623 | 0.431092836 | 1.1615284 | up | lncRNA |
| ENST00000416999 | AC058791.2 | 0.992066267 | 0.999156445 | 1.0015931 | up | lncRNA |
| ENST00000417038 | AC010894.3 | 0.476664718 | 0.917651513 | 1.1658422 | up | lncRNA |
| ENST00000417079 | LINC00426 | 0.289034452 | 0.837264328 | 1.2079432 | up | lncRNA |
| ENST00000417083 | CTA-211A9.5 | 0.61612969 | 0.959051918 | 1.0184793 | up | lncRNA |
| ENST00000417099 | RP1-283E3.4 | 0.866013184 | 0.996767702 | 1.0535984 | up | lncRNA |
| ENST00000417112 | RP11-554I8.2 | 0.247388424 | 0.814489937 | 1.1141449 | up | lncRNA |
| ENST00000417120 | AKT3-IT1 | 0.255508088 | 0.821835543 | 1.2620141 | up | lncRNA |
| ENST00000417123 | RP4-591N18.2 | 0.977765025 | 0.998875101 | 1.0049955 | up | lncRNA |
| ENST00000417213 | AC009229.6 | 0.098387345 | 0.624143367 | 1.2120675 | up | lncRNA |
| ENST00000417221 | AC068831.10 | 0.276208149 | 0.832297359 | 1.1573629 | up | lncRNA |
| ENST00000417267 | AC114730.7 | 0.608012797 | 0.956065104 | 1.0318611 | up | lncRNA |
| ENST00000417281 | CTC-338M12.4 | 0.125252265 | 0.67731705 | 1.4040177 | up | lncRNA |
| ENST00000417299 | RP1-278O22.1 | 0.001538775 | 0.135129436 | 1.7077075 | up | lncRNA |
| ENST00000417311 | RP11-84P7.2 | 0.265366322 | 0.828345538 | 1.2724273 | up | lncRNA |
| ENST00000417333 | RP11-10A14.5 | 0.576415876 | 0.947511242 | 1.0381024 | up | lncRNA |
| ENST00000417346 | RP4-800J21.3 | 0.260244808 | 0.824565516 | 1.0575722 | up | lncRNA |
| ENST00000417354 | DNM3OS | 0.230756808 | 0.79852974 | 1.3621871 | up | lncRNA |
| ENST00000417359 | RP11-379F12.3 | 0.463910284 | 0.913755581 | 1.1236285 | up | lncRNA |
| ENST00000417381 | TTC28-AS1 | 0.133875065 | 0.689601422 | 1.2225229 | up | lncRNA |
| ENST00000417384 | RP11-30O15.1 | 0.831751462 | 0.994074191 | 1.0296059 | up | lncRNA |
| ENST00000417401 | RP5-1033H22.2 | 0.326594645 | 0.854556781 | 1.2422196 | up | lncRNA |
| ENST00000417409 | RP11-161I10.1 | 0.363255459 | 0.874234597 | 1.664599 | up | lncRNA |
| ENST00000417447 | RP11-135A24.2 | 0.293284259 | 0.838358604 | 1.8421086 | up | lncRNA |
| ENST00000417481 | AC012513.6 | 0.633951494 | 0.964757805 | 1.0539859 | up | lncRNA |
| ENST00000417513 | ZDHHC20-IT1 | 0.729150134 | 0.982841582 | 1.12289 | up | lncRNA |
| ENST00000417516 | AC004041.2 | 0.028672083 | 0.405420433 | 1.3937955 | up | lncRNA |
| ENST00000417554 | RP11-495P10.2 | 0.02359245 | 0.37091715 | 1.3684926 | up | lncRNA |
| ENST00000417565 | RP11-475O6.1 | 0.954622904 | 0.998875101 | 1.0237668 | up | lncRNA |
| ENST00000417650 | RP11-439L18.1 | 0.000100992 | 0.046755988 | 11.8855355 | up | lncRNA |
| ENST00000417670 | AC108868.4 | 0.793086937 | 0.991420185 | 1.0334308 | up | lncRNA |
| ENST00000417692 | AC079807.2 | 0.90279662 | 0.998875101 | 1.0190481 | up | lncRNA |
| ENST00000417696 | RP11-23O13.1 | 0.406375355 | 0.889488526 | 1.1017774 | up | lncRNA |
| ENST00000417715 | AC073928.2 | 0.377130656 | 0.879520864 | 1.5917154 | up | lncRNA |
| ENST00000417742 | GRIFIN | 0.154810846 | 0.711341825 | 1.088723 | up | lncRNA |
| ENST00000417773 | LINC00222 | 0.901797451 | 0.998875101 | 1.0134299 | up | lncRNA |
| ENST00000417844 | AC068542.1 | 0.424553483 | 0.89750944 | 1.1772571 | up | lncRNA |
| ENST00000417860 | RP11-322M19.1 | 0.025965619 | 0.383305135 | 1.2089347 | up | lncRNA |
| ENST00000417942 | XIST | 0.582062104 | 0.949239022 | 1.0523267 | up | lncRNA |
| ENST00000417957 | RP11-375H17.1 | 0.650326122 | 0.969927925 | 1.0495424 | up | lncRNA |
| ENST00000417968 | RP11-354M20.3 | 0.064398231 | 0.539271375 | 1.1350907 | up | lncRNA |
| ENST00000417969 | RP11-104L21.2 | 0.352718373 | 0.868751345 | 1.1251206 | up | lncRNA |
| ENST00000417976 | BX248398.1 | 0.29792161 | 0.841190427 | 1.5406355 | up | lncRNA |
| ENST00000418106 | AC007405.8 | 0.807639437 | 0.992578294 | 1.0272617 | up | lncRNA |
| ENST00000418134 | RP1-60O19.1 | 0.272366017 | 0.832297359 | 1.2677686 | up | lncRNA |
| ENST00000418161 | CCDC13-AS1 | 0.650592492 | 0.969927925 | 1.427762 | up | lncRNA |
| ENST00000418192 | BX284650.1 | 0.001070126 | 0.116876278 | 1.6834814 | up | lncRNA |
| ENST00000418204 | AC104135.3 | 0.780062304 | 0.99121281 | 1.075917 | up | lncRNA |
| ENST00000418209 | IGKJ5 | 0.012635627 | 0.286112184 | 1.2852713 | up | lncRNA |
| ENST00000418237 | RP11-78J21.4 | 0.852044561 | 0.995900317 | 1.0208684 | up | lncRNA |
| ENST00000418242 | RP11-148B18.3 | 0.002103269 | 0.141857833 | 5.8462962 | up | lncRNA |
| ENST00000418246 | RP4-545L17.7 | 0.591712776 | 0.950912706 | 1.114671 | up | lncRNA |
| ENST00000418292 | CTA-292E10.6 | 0.347711491 | 0.866454524 | 1.1633453 | up | lncRNA |
| ENST00000418308 | PCBP1-AS1 | 0.70844036 | 0.981609897 | 1.1224027 | up | lncRNA |
| ENST00000418362 | RP11-364B6.1 | 0.746497857 | 0.985094857 | 1.0571299 | up | lncRNA |
| ENST00000418372 | RP11-67B16.1 | 0.698452866 | 0.980170937 | 1.030717 | up | lncRNA |
| ENST00000418402 | RP11-522M21.2 | 0.882201328 | 0.998875101 | 1.0167192 | up | lncRNA |
| ENST00000418415 | AC016912.3 | 0.261724378 | 0.82544125 | 1.1244886 | up | lncRNA |
| ENST00000418474 | AC009506.1 | 0.637341885 | 0.96564589 | 1.040315 | up | lncRNA |
| ENST00000418515 | RP11-619F23.2 | 0.088392839 | 0.599406853 | 1.4540651 | up | lncRNA |
| ENST00000418525 | RP11-169K16.4 | 0.197462568 | 0.763778725 | 1.2071391 | up | lncRNA |
| ENST00000418602 | RP11-536C5.7 | 0.783325566 | 0.99121281 | 1.0407042 | up | lncRNA |
| ENST00000418607 | BX004987.7 | 0.993772201 | 0.999156445 | 1.0008314 | up | lncRNA |
| ENST00000418618 | RP11-439L8.3 | 0.912329925 | 0.998875101 | 1.0296477 | up | lncRNA |
| ENST00000418656 | RP11-508N12.2 | 0.043274919 | 0.473947772 | 1.1059513 | up | lncRNA |
| ENST00000418669 | AC006995.8 | 0.492015545 | 0.920777644 | 1.0339086 | up | lncRNA |
| ENST00000418741 | LINC00419 | 0.796005878 | 0.991420185 | 1.0504266 | up | lncRNA |
| ENST00000418743 | RP4-749H3.1 | 0.498429379 | 0.921805853 | 1.1816386 | up | lncRNA |
| ENST00000418756 | PMS2P3 | 0.01068196 | 0.261616866 | 1.1801717 | up | lncRNA |
| ENST00000418821 | AC015922.6 | 0.614908496 | 0.958423841 | 1.1399876 | up | lncRNA |
| ENST00000418836 | AC104654.1 | 0.543515119 | 0.939697392 | 1.1495303 | up | lncRNA |
| ENST00000418837 | RP11-557H15.4 | 0.258364833 | 0.823936049 | 1.0852002 | up | lncRNA |
| ENST00000418855 | FTX | 0.852532414 | 0.995900317 | 1.0466855 | up | lncRNA |
| ENST00000418875 | RP11-92J19.3 | 0.698516542 | 0.980170937 | 1.0676931 | up | lncRNA |
| ENST00000418907 | AC073333.8 | 0.360900053 | 0.873342057 | 1.7121144 | up | lncRNA |
| ENST00000418927 | AL162759.1 | 0.789531005 | 0.991420185 | 1.0261742 | up | lncRNA |
| ENST00000418943 | RP11-473M10.3 | 0.105071628 | 0.635942989 | 1.9952774 | up | lncRNA |
| ENST00000418957 | AC011747.7 | 0.600344983 | 0.953841565 | 1.0724093 | up | lncRNA |
| ENST00000418972 | RP11-204C23.1 | 0.070972195 | 0.559359114 | 1.1651856 | up | lncRNA |
| ENST00000418990 | AC005041.9 | 0.919592658 | 0.998875101 | 1.0103895 | up | lncRNA |
| ENST00000419028 | AC141930.2 | 6.51913E-05 | 0.037425003 | 30.6692865 | up | lncRNA |
| ENST00000419029 | AC133106.2 | 0.002241953 | 0.143044381 | 1.566745 | up | lncRNA |
| ENST00000419083 | MYCNOS | 0.0994706 | 0.625213612 | 1.3090964 | up | lncRNA |
| ENST00000419105 | RP11-277L2.2 | 0.031462883 | 0.418106757 | 2.4272022 | up | lncRNA |
| ENST00000419134 | RP1-12G14.7 | 0.024799958 | 0.377450959 | 1.2387237 | up | lncRNA |
| ENST00000419220 | RP11-356I2.1 | 0.010347373 | 0.26053596 | 3.1948941 | up | lncRNA |
| ENST00000419223 | AC023347.1 | 0.950779997 | 0.998875101 | 1.0133925 | up | lncRNA |
| ENST00000419243 | AC093590.1 | 0.468842512 | 0.914494873 | 1.1715632 | up | lncRNA |
| ENST00000419299 | LINC00320 | 0.305353244 | 0.846407029 | 1.3376567 | up | lncRNA |
| ENST00000419303 | PRAMEL | 0.369432369 | 0.876569032 | 1.0832849 | up | lncRNA |
| ENST00000419361 | RP11-439E19.3 | 0.854951274 | 0.996142678 | 1.0489445 | up | lncRNA |
| ENST00000419415 | KIRREL-IT1 | 0.000398558 | 0.084119144 | 2.0383309 | up | lncRNA |
| ENST00000419425 | AC006369.2 | 0.077884141 | 0.575629572 | 1.3565348 | up | lncRNA |
| ENST00000419440 | AP004372.1 | 0.940104112 | 0.998875101 | 1.0184295 | up | lncRNA |
| ENST00000419458 | RP11-21M7.1 | 0.670460302 | 0.974341942 | 1.1452806 | up | lncRNA |
| ENST00000419467 | AP000472.2 | 0.249652177 | 0.816027969 | 1.1832741 | up | lncRNA |
| ENST00000419481 | HCG21 | 0.308615297 | 0.847582029 | 1.1417056 | up | lncRNA |
| ENST00000419627 | RP3-323P13.2 | 0.31862366 | 0.851223284 | 1.0941983 | up | lncRNA |
| ENST00000419667 | RP11-174G17 | 0.090340909 | 0.604992392 | 1.3942481 | up | lncRNA |
| ENST00000419668 | AC002454.1 | 0.00655787 | 0.217096533 | 1.385675 | up | lncRNA |
| ENST00000419680 | LINC00152 | 0.151371791 | 0.706270462 | 1.8810314 | up | lncRNA |
| ENST00000419694 | AP000221.1 | 0.214512118 | 0.781610853 | 1.3467331 | up | lncRNA |
| ENST00000419697 | RP11-773D16.1 | 0.734286258 | 0.983763148 | 1.0267345 | up | lncRNA |
| ENST00000419703 | FNDC1-IT1 | 0.054154294 | 0.516622604 | 1.9894883 | up | lncRNA |
| ENST00000419736 | AC068491.1 | 0.149312782 | 0.704640207 | 1.1978415 | up | lncRNA |
| ENST00000419815 | RP11-380F14.2 | 0.221357742 | 0.790635384 | 1.3437664 | up | lncRNA |
| ENST00000419817 | RP11-458D21.1 | 0.184575118 | 0.751257244 | 1.1085299 | up | lncRNA |
| ENST00000419897 | RP11-465L10.10 | 0.028363752 | 0.402249575 | 1.3967795 | up | lncRNA |
| ENST00000419922 | LINC00607 | 0.27368009 | 0.832297359 | 1.1241852 | up | lncRNA |
| ENST00000419931 | RP11-445H22.4 | 0.853276334 | 0.996142678 | 1.0384285 | up | lncRNA |
| ENST00000419952 | LINC00478 | 0.068305197 | 0.55181901 | 1.1646511 | up | lncRNA |
| ENST00000419963 | PCBP1-AS1 | 0.284589931 | 0.834623138 | 1.1353128 | up | lncRNA |
| ENST00000420006 | SNHG11 | 0.607715878 | 0.956065104 | 1.0405988 | up | lncRNA |
| ENST00000420038 | CTD-3105H18.7 | 0.747141223 | 0.985094857 | 1.0807685 | up | lncRNA |
| ENST00000420074 | MCM3AP-AS1 | 0.045817949 | 0.483158858 | 1.1604443 | up | lncRNA |
| ENST00000420080 | RP11-395B7.2 | 0.959662587 | 0.998875101 | 1.0064184 | up | lncRNA |
| ENST00000420081 | LINC00240 | 0.593690411 | 0.951058356 | 1.0664653 | up | lncRNA |
| ENST00000420110 | HLA-L | 0.692037233 | 0.97895216 | 1.112622 | up | lncRNA |
| ENST00000420118 | RP3-508I15.18 | 0.281841916 | 0.832297359 | 1.118531 | up | lncRNA |
| ENST00000420140 | LINC00357 | 0.746886706 | 0.985094857 | 1.4548059 | up | lncRNA |
| ENST00000420153 | AL928742.12 | 0.594654924 | 0.951072365 | 1.0906553 | up | lncRNA |
| ENST00000420156 | RP4-543J13.1 | 0.022800131 | 0.364395852 | 1.2282917 | up | lncRNA |
| ENST00000420168 | RP11-31F15.1 | 0.012457841 | 0.284705301 | 1.1616768 | up | lncRNA |
| ENST00000420169 | BPIFA4P | 0.589763264 | 0.950589384 | 1.0423366 | up | lncRNA |
| ENST00000420180 | RP1-76B20.12 | 0.466560022 | 0.914151254 | 1.0702491 | up | lncRNA |
| ENST00000420211 | RP11-422P22.1 | 0.785072446 | 0.991253313 | 1.029944 | up | lncRNA |
| ENST00000420213 | AC128709.2 | 0.653070224 | 0.97018076 | 1.0586485 | up | lncRNA |
| ENST00000420219 | LINC00426 | 0.642661855 | 0.967175883 | 1.0865527 | up | lncRNA |
| ENST00000420225 | AC007050.17 | 0.143281205 | 0.699684196 | 1.1471229 | up | lncRNA |
| ENST00000420226 | TM4SF19-AS1 | 0.555227756 | 0.943023022 | 1.1179156 | up | lncRNA |
| ENST00000420227 | RP5-1177E19.2 | 0.162764583 | 0.718236848 | 1.5012014 | up | lncRNA |
| ENST00000420237 | RP11-135J2.3 | 0.714561579 | 0.98251396 | 1.0402014 | up | lncRNA |
| ENST00000420255 | AP000472.2 | 0.568841185 | 0.943923733 | 1.1798701 | up | lncRNA |
| ENST00000420279 | RP5-907D15.2 | 0.72458238 | 0.98251396 | 1.0529674 | up | lncRNA |
| ENST00000420293 | RP11-61I13.3 | 0.393529984 | 0.885711362 | 1.1559088 | up | lncRNA |
| ENST00000420350 | RP11-365O16.6 | 0.686386224 | 0.97861545 | 1.0990657 | up | lncRNA |
| ENST00000420365 | AC079790.2 | 0.644004628 | 0.967175883 | 1.2429586 | up | lncRNA |
| ENST00000420373 | AC005488.12 | 0.366611542 | 0.875184441 | 1.1427563 | up | lncRNA |
| ENST00000420389 | RP1-290I10.3 | 0.652102438 | 0.969927925 | 1.0448489 | up | lncRNA |
| ENST00000420391 | CTA-796E4.3 | 0.295758901 | 0.839987155 | 1.0952663 | up | lncRNA |
| ENST00000420403 | RP11-1L9.1 | 0.812213295 | 0.99257857 | 1.0591442 | up | lncRNA |
| ENST00000420427 | AC004562.1 | 0.913669072 | 0.998875101 | 1.0237364 | up | lncRNA |
| ENST00000420452 | MYCNOS | 0.788779529 | 0.991420185 | 1.0245474 | up | lncRNA |
| ENST00000420498 | RP11-297K8.2 | 0.495943152 | 0.921717611 | 1.1473433 | up | lncRNA |
| ENST00000420508 | KB-1592A4.15 | 0.614168528 | 0.958418342 | 1.0396514 | up | lncRNA |
| ENST00000420520 | DDX39B-AS1 | 0.143966849 | 0.699853429 | 1.4090127 | up | lncRNA |
| ENST00000420524 | FAM197Y7 | 0.684225266 | 0.977836377 | 1.0528375 | up | lncRNA |
| ENST00000420537 | RP1-85F18.5 | 0.441510477 | 0.904580493 | 1.0778539 | up | lncRNA |
| ENST00000420546 | RP13-100B2.4 | 0.080481068 | 0.582052437 | 1.4799123 | up | lncRNA |
| ENST00000420549 | RP11-147G16.1 | 0.412221764 | 0.890899058 | 1.1294579 | up | lncRNA |
| ENST00000420573 | RP11-195E11.3 | 0.187176802 | 0.753960556 | 1.9364457 | up | lncRNA |
| ENST00000420579 | AC104653.1 | 0.306367911 | 0.846407029 | 1.1630644 | up | lncRNA |
| ENST00000420651 | TRAF3IP2-AS1 | 0.789061366 | 0.991420185 | 1.0859378 | up | lncRNA |
| ENST00000420695 | BX470102.3 | 0.271996536 | 0.832297359 | 1.2107824 | up | lncRNA |
| ENST00000420759 | XXbac-BPG55C20.7 | 0.941316436 | 0.998875101 | 1.0506733 | up | lncRNA |
| ENST00000420762 | RP11-95P13.1 | 0.099079429 | 0.624500644 | 2.4158183 | up | lncRNA |
| ENST00000420777 | RP1-290I10.6 | 0.562315949 | 0.943393901 | 1.1488601 | up | lncRNA |
| ENST00000420830 | RP11-261C10.2 | 0.352850138 | 0.868926763 | 1.0984422 | up | lncRNA |
| ENST00000420832 | AC018816.4 | 0.329891332 | 0.855547596 | 1.6036396 | up | lncRNA |
| ENST00000420865 | RP11-309M23.1 | 0.006185587 | 0.212479102 | 5.8551666 | up | lncRNA |
| ENST00000420870 | AC093415.2 | 0.792426812 | 0.991420185 | 1.0416511 | up | lncRNA |
| ENST00000420876 | RP11-511I2.2 | 0.333714722 | 0.85798034 | 1.0992145 | up | lncRNA |
| ENST00000420928 | RP5-828K20.2 | 0.013270677 | 0.290336527 | 1.7228385 | up | lncRNA |
| ENST00000420956 | LINC00413 | 0.057170609 | 0.52454156 | 1.3385096 | up | lncRNA |
| ENST00000420968 | ZDHHC8P1 | 0.389744281 | 0.885317362 | 1.0567057 | up | lncRNA |
| ENST00000421013 | RP11-421L21.3 | 0.904408377 | 0.998875101 | 1.0319172 | up | lncRNA |
| ENST00000421055 | RP11-335O13.7 | 0.291584245 | 0.837264328 | 1.139571 | up | lncRNA |
| ENST00000421116 | RRP7B | 0.734916998 | 0.983763148 | 1.0196636 | up | lncRNA |
| ENST00000421132 | RP11-351M16.3 | 0.011193241 | 0.267742326 | 1.318106 | up | lncRNA |
| ENST00000421166 | RP4-681L3.2 | 0.75585796 | 0.98762882 | 1.0871389 | up | lncRNA |
| ENST00000421170 | RP11-69I22.3 | 0.123387811 | 0.67474001 | 1.5967409 | up | lncRNA |
| ENST00000421181 | AC080094.1 | 0.465356474 | 0.913821992 | 1.0804438 | up | lncRNA |
| ENST00000421237 | RP11-439L18.2 | 0.002182075 | 0.142436072 | 3.5807387 | up | lncRNA |
| ENST00000421275 | RP11-148G20.1 | 0.977180328 | 0.998875101 | 1.0038477 | up | lncRNA |
| ENST00000421297 | RP5-1050E16.2 | 0.836909945 | 0.995078046 | 1.0496209 | up | lncRNA |
| ENST00000421298 | AC011747.7 | 0.413632282 | 0.890927603 | 1.0981622 | up | lncRNA |
| ENST00000421310 | RP11-69I8.2 | 0.593553655 | 0.951058356 | 1.0982785 | up | lncRNA |
| ENST00000421320 | RP11-124O11.1 | 0.088353788 | 0.599406853 | 1.1190631 | up | lncRNA |
| ENST00000421449 | IPO9-AS1 | 0.897260194 | 0.998875101 | 1.015045 | up | lncRNA |
| ENST00000421483 | RP5-842K24.2 | 0.00074747 | 0.098419223 | 3.0123189 | up | lncRNA |
| ENST00000421500 | RP11-310H4.2 | 0.329463123 | 0.85540421 | 1.4613306 | up | lncRNA |
| ENST00000421599 | SNHG11 | 0.520984963 | 0.932117592 | 1.051451 | up | lncRNA |
| ENST00000421601 | LINC00443 | 0.363094083 | 0.874234597 | 1.0879991 | up | lncRNA |
| ENST00000421604 | AP001255.2 | 0.694096016 | 0.979611174 | 1.0670181 | up | lncRNA |
| ENST00000421610 | AC090627.1 | 0.078949736 | 0.577356551 | 1.6892833 | up | lncRNA |
| ENST00000421637 | RP4-784A16.2 | 0.903147236 | 0.998875101 | 1.0157062 | up | lncRNA |
| ENST00000421648 | AC092214.10 | 0.640556683 | 0.966298892 | 1.2401558 | up | lncRNA |
| ENST00000421657 | RP11-186N15.3 | 0.163829221 | 0.718769923 | 1.2068857 | up | lncRNA |
| ENST00000421718 | AC012314.6 | 0.018511711 | 0.340811763 | 1.130605 | up | lncRNA |
| ENST00000421730 | AC005682.8 | 0.051188379 | 0.50923846 | 2.3225148 | up | lncRNA |
| ENST00000421734 | RP11-575L7.4 | 0.238005847 | 0.807531256 | 1.3074161 | up | lncRNA |
| ENST00000421735 | U73167.7 | 0.467803508 | 0.914494873 | 1.120069 | up | lncRNA |
| ENST00000421764 | RP11-315I20.1 | 0.164500831 | 0.719521661 | 1.8033469 | up | lncRNA |
| ENST00000421788 | RP4-697P8.2 | 0.872262688 | 0.997909851 | 1.0621147 | up | lncRNA |
| ENST00000421796 | AC004448.5 | 0.004565433 | 0.189321509 | 1.4335644 | up | lncRNA |
| ENST00000421820 | AC018866.1 | 0.503480208 | 0.92405258 | 1.0949623 | up | lncRNA |
| ENST00000421843 | PCBP1-AS1 | 0.979234778 | 0.998875101 | 1.002436 | up | lncRNA |
| ENST00000421862 | AC004540.5 | 0.651503023 | 0.969927925 | 1.0500624 | up | lncRNA |
| ENST00000421866 | RP11-61L14.6 | 0.839937879 | 0.995583664 | 1.0145401 | up | lncRNA |
| ENST00000421867 | MIAT | 0.805744614 | 0.992578294 | 1.0829693 | up | lncRNA |
| ENST00000421868 | AC114812.5 | 0.378022863 | 0.879833243 | 1.1691475 | up | lncRNA |
| ENST00000421878 | RP11-561I11.2 | 0.377278235 | 0.879525191 | 1.098674 | up | lncRNA |
| ENST00000421927 | MCM3AP-AS1 | 0.916189319 | 0.998875101 | 1.0118163 | up | lncRNA |
| ENST00000421937 | RP11-458D21.2 | 0.086712193 | 0.597529798 | 1.1298989 | up | lncRNA |
| ENST00000421943 | RP11-426L16.3 | 0.005742746 | 0.208598143 | 2.0684298 | up | lncRNA |
| ENST00000421964 | AC083900.1 | 0.419819435 | 0.894750301 | 1.0956822 | up | lncRNA |
| ENST00000421965 | AC006159.4 | 0.446748503 | 0.905331314 | 1.1042481 | up | lncRNA |
| ENST00000421996 | AC093734.14 | 0.150350284 | 0.705518742 | 1.3078637 | up | lncRNA |
| ENST00000421997 | RP11-86H7.1 | 0.679244695 | 0.976414249 | 1.0433933 | up | lncRNA |
| ENST00000422023 | RP11-242F11.2 | 0.30388847 | 0.845119652 | 1.1740731 | up | lncRNA |
| ENST00000422042 | AC009276.4 | 0.112328389 | 0.651593972 | 2.8062557 | up | lncRNA |
| ENST00000422045 | AC008440.10 | 0.500584067 | 0.922148899 | 1.2689254 | up | lncRNA |
| ENST00000422047 | RP11-245M24.1 | 0.222823667 | 0.791379677 | 1.2663612 | up | lncRNA |
| ENST00000422060 | ZNF890P | 0.650678379 | 0.969927925 | 1.083244 | up | lncRNA |
| ENST00000422064 | PMS2P3 | 0.224430817 | 0.794080348 | 1.1711347 | up | lncRNA |
| ENST00000422124 | RP11-108M9.4 | 0.761144185 | 0.988045734 | 1.022782 | up | lncRNA |
| ENST00000422150 | RP11-252M18.3 | 0.760650945 | 0.988045734 | 1.1166595 | up | lncRNA |
| ENST00000422151 | CES5AP1 | 0.770177714 | 0.989686083 | 1.0400837 | up | lncRNA |
| ENST00000422162 | RP11-465K1.2 | 0.600278158 | 0.953841565 | 1.1449121 | up | lncRNA |
| ENST00000422178 | AC016745.3 | 0.004397794 | 0.188856054 | 2.46785 | up | lncRNA |
| ENST00000422194 | RP11-402P6.9 | 0.465517356 | 0.913821992 | 1.1241169 | up | lncRNA |
| ENST00000422203 | AC096669.1 | 0.17796609 | 0.743651912 | 1.2767129 | up | lncRNA |
| ENST00000422204 | AC116366.5 | 0.231068765 | 0.79852974 | 1.3791263 | up | lncRNA |
| ENST00000422230 | AC091729.9 | 0.905187913 | 0.998875101 | 1.0225775 | up | lncRNA |
| ENST00000422239 | AC007285.6 | 0.713086372 | 0.98251396 | 1.2421819 | up | lncRNA |
| ENST00000422253 | RP5-968D22.3 | 0.616440197 | 0.959144591 | 1.0558366 | up | lncRNA |
| ENST00000422306 | RP11-296A18.6 | 0.491985628 | 0.920777644 | 1.0917396 | up | lncRNA |
| ENST00000422343 | RP11-180I4.2 | 0.703339087 | 0.980562547 | 1.0712531 | up | lncRNA |
| ENST00000422403 | MIAT | 0.364319211 | 0.874818743 | 1.1819655 | up | lncRNA |
| ENST00000422415 | AC010145.4 | 0.036900316 | 0.43898603 | 1.1122995 | up | lncRNA |
| ENST00000422521 | AC008280.5 | 0.9184089 | 0.998875101 | 1.0419681 | up | lncRNA |
| ENST00000422542 | AC005682.5 | 0.660320722 | 0.971704562 | 1.0631566 | up | lncRNA |
| ENST00000422555 | AC073135.3 | 0.787736183 | 0.991420185 | 1.1082008 | up | lncRNA |
| ENST00000422629 | BX248398.1 | 0.412301529 | 0.890899058 | 1.2549688 | up | lncRNA |
| ENST00000422638 | RP4-656G21.1 | 0.150524834 | 0.705518742 | 1.2052314 | up | lncRNA |
| ENST00000422649 | AC018737.3 | 0.555202868 | 0.943023022 | 1.0667744 | up | lncRNA |
| ENST00000422657 | AC090957.2 | 0.868263422 | 0.997224443 | 1.0258462 | up | lncRNA |
| ENST00000422665 | RP11-263K19.4 | 0.84735799 | 0.995583664 | 1.0083988 | up | lncRNA |
| ENST00000422679 | MIR1302-10 | 0.968795803 | 0.998875101 | 1.004113 | up | lncRNA |
| ENST00000422826 | H19 | 0.352988853 | 0.86894522 | 1.0560375 | up | lncRNA |
| ENST00000422831 | AC004920.3 | 0.975676903 | 0.998875101 | 1.0021697 | up | lncRNA |
| ENST00000422836 | ARHGEF7-AS2 | 0.927444317 | 0.998875101 | 1.0182426 | up | lncRNA |
| ENST00000422858 | RP11-46A10.2 | 0.380908226 | 0.881030597 | 1.1923216 | up | lncRNA |
| ENST00000422931 | LMO7-AS1 | 0.452798814 | 0.909172908 | 1.142616 | up | lncRNA |
| ENST00000422966 | RP11-523O18.1 | 0.982901231 | 0.998875101 | 1.0020568 | up | lncRNA |
| ENST00000422980 | DPYD-AS1 | 0.158788291 | 0.714694414 | 1.5877242 | up | lncRNA |
| ENST00000423054 | RP11-459F3.6 | 0.244144946 | 0.810806356 | 1.5218977 | up | lncRNA |
| ENST00000423062 | RP1-40E16.3 | 0.968019212 | 0.998875101 | 1.0045008 | up | lncRNA |
| ENST00000423074 | RP4-553F4.6 | 0.154611375 | 0.711341825 | 1.1104741 | up | lncRNA |
| ENST00000423083 | NCF1B | 0.006564931 | 0.217096533 | 1.2256599 | up | lncRNA |
| ENST00000423084 | AC004980.7 | 0.034237264 | 0.42757611 | 1.7948936 | up | lncRNA |
| ENST00000423099 | RP11-505P4.6 | 0.992092297 | 0.999156445 | 1.0025985 | up | lncRNA |
| ENST00000423171 | RP11-151D14.1 | 0.471038196 | 0.915538705 | 1.0379737 | up | lncRNA |
| ENST00000423187 | RP11-99H8.1 | 0.250512497 | 0.817015761 | 1.0918263 | up | lncRNA |
| ENST00000423193 | AC090044.2 | 0.47295754 | 0.91677171 | 1.1786308 | up | lncRNA |
| ENST00000423232 | RP13-137A17.5 | 0.334441436 | 0.85798034 | 1.4514845 | up | lncRNA |
| ENST00000423249 | BX004987.4 | 0.395589019 | 0.885711362 | 1.3686623 | up | lncRNA |
| ENST00000423268 | RP11-631F7.1 | 0.043885217 | 0.478238902 | 1.2820395 | up | lncRNA |
| ENST00000423283 | RP11-123B3.2 | 0.28145776 | 0.832297359 | 1.2289883 | up | lncRNA |
| ENST00000423311 | CTA-714B7.5 | 0.784418929 | 0.99121281 | 1.0203915 | up | lncRNA |
| ENST00000423367 | AC015933.2 | 0.543327841 | 0.939697392 | 1.4318578 | up | lncRNA |
| ENST00000423380 | RP11-305L7.1 | 0.887361188 | 0.998875101 | 1.0292227 | up | lncRNA |
| ENST00000423408 | RP4-794H19.1 | 0.300548852 | 0.842264993 | 1.1898499 | up | lncRNA |
| ENST00000423440 | RP11-321L2.1 | 0.861917564 | 0.996142678 | 1.020002 | up | lncRNA |
| ENST00000423442 | LINC00458 | 0.689491901 | 0.97861545 | 1.0196938 | up | lncRNA |
| ENST00000423466 | RP11-114M1.1 | 0.930290659 | 0.998875101 | 1.0630727 | up | lncRNA |
| ENST00000423473 | RP11-344E13.3 | 0.340534062 | 0.861386906 | 1.074739 | up | lncRNA |
| ENST00000423580 | ANKRD62P1 | 0.028041664 | 0.400053645 | 1.2730075 | up | lncRNA |
| ENST00000423617 | RP11-622K12.1 | 0.795612845 | 0.991420185 | 1.0335958 | up | lncRNA |
| ENST00000423706 | AC012506.1 | 0.407045583 | 0.889505404 | 1.1639855 | up | lncRNA |
| ENST00000423714 | ZEB1-AS1 | 0.463374413 | 0.913747281 | 1.0893996 | up | lncRNA |
| ENST00000423793 | RP11-432J22.2 | 0.721696025 | 0.98251396 | 1.0408964 | up | lncRNA |
| ENST00000423808 | AL035610.1 | 0.95350858 | 0.998875101 | 1.0059356 | up | lncRNA |
| ENST00000423824 | RP11-326L24.4 | 0.440590652 | 0.904456709 | 1.120094 | up | lncRNA |
| ENST00000423907 | RP4-655J12.4 | 0.295907331 | 0.839987155 | 1.3141781 | up | lncRNA |
| ENST00000423925 | AC008069.1 | 0.310367402 | 0.848664472 | 1.2158247 | up | lncRNA |
| ENST00000423963 | RP11-443B7.2 | 0.20813032 | 0.775639766 | 1.5731219 | up | lncRNA |
| ENST00000423984 | WARS2-IT1 | 0.001595933 | 0.135515114 | 1.1540332 | up | lncRNA |
| ENST00000424029 | CES5AP1 | 0.030211104 | 0.414324281 | 1.0678119 | up | lncRNA |
| ENST00000424036 | RP11-340I6.3 | 0.285735754 | 0.835890651 | 1.2899557 | up | lncRNA |
| ENST00000424084 | RP4-799D16.1 | 0.507669752 | 0.9259215 | 1.1663845 | up | lncRNA |
| ENST00000424106 | KPNA3-IT1 | 0.17568913 | 0.738380482 | 1.1407989 | up | lncRNA |
| ENST00000424116 | AC098617.2 | 0.822965776 | 0.993856674 | 1.0508853 | up | lncRNA |
| ENST00000424179 | LINC00487 | 0.083264716 | 0.585999222 | 1.2293167 | up | lncRNA |
| ENST00000424191 | ZEB1-AS1 | 0.46489033 | 0.913821992 | 1.0841693 | up | lncRNA |
| ENST00000424205 | RP5-907D15.2 | 0.967989986 | 0.998875101 | 1.0111226 | up | lncRNA |
| ENST00000424235 | RP4-564F22.2 | 0.171603593 | 0.732960438 | 1.0966345 | up | lncRNA |
| ENST00000424252 | RP4-724E16.2 | 0.302853842 | 0.843991909 | 1.1122106 | up | lncRNA |
| ENST00000424308 | RP4-794H19.4 | 0.552131939 | 0.942347198 | 1.1027699 | up | lncRNA |
| ENST00000424313 | AP000997.1 | 0.843854521 | 0.995583664 | 1.0237559 | up | lncRNA |
| ENST00000424343 | RP11-503C24.2 | 0.904285236 | 0.998875101 | 1.0201236 | up | lncRNA |
| ENST00000424391 | AC012594.1 | 0.065341656 | 0.542384873 | 1.0944608 | up | lncRNA |
| ENST00000424404 | RP11-560I19.4 | 0.044023963 | 0.478635427 | 1.3699158 | up | lncRNA |
| ENST00000424417 | RP11-84E24.2 | 0.186876506 | 0.753717042 | 1.3552066 | up | lncRNA |
| ENST00000424418 | RP1-144F13.3 | 0.005598583 | 0.206027856 | 2.032248 | up | lncRNA |
| ENST00000424435 | RP11-350G8.5 | 0.025133237 | 0.380009848 | 3.1311949 | up | lncRNA |
| ENST00000424450 | RP11-122K13.12 | 0.316783122 | 0.851223284 | 1.2484925 | up | lncRNA |
| ENST00000424477 | MAGI2-AS3 | 0.601876811 | 0.954578533 | 1.1981972 | up | lncRNA |
| ENST00000424493 | AC024560.2 | 0.884956009 | 0.998875101 | 1.0334213 | up | lncRNA |
| ENST00000424517 | RP4-717I23.3 | 0.918315701 | 0.998875101 | 1.0280869 | up | lncRNA |
| ENST00000424521 | RP3-453D15.1 | 0.317569644 | 0.851223284 | 1.1116944 | up | lncRNA |
| ENST00000424523 | AC002454.1 | 0.043590802 | 0.475753 | 1.28379 | up | lncRNA |
| ENST00000424592 | RP11-63G10.2 | 0.092386199 | 0.610803569 | 1.2198274 | up | lncRNA |
| ENST00000424612 | AC017074.2 | 0.928124296 | 0.998875101 | 1.0166385 | up | lncRNA |
| ENST00000424613 | CTA-250D10.19 | 0.477859463 | 0.917735717 | 1.1152946 | up | lncRNA |
| ENST00000424617 | AC068491.1 | 0.091961331 | 0.609339348 | 1.4065586 | up | lncRNA |
| ENST00000424640 | RP11-423O2.7 | 0.155478334 | 0.711341825 | 1.3143581 | up | lncRNA |
| ENST00000424655 | AC104820.2 | 0.078568479 | 0.57678507 | 1.4934962 | up | lncRNA |
| ENST00000424662 | RP4-614C15.2 | 0.28671245 | 0.836701318 | 1.4369317 | up | lncRNA |
| ENST00000424682 | RP11-58A12.2 | 0.197679637 | 0.763778725 | 1.9220535 | up | lncRNA |
| ENST00000424711 | RP11-86H7.1 | 0.084133355 | 0.589015565 | 1.2624968 | up | lncRNA |
| ENST00000424748 | AC016700.3 | 0.794790155 | 0.991420185 | 1.0605463 | up | lncRNA |
| ENST00000424770 | LA16c-4G1.3 | 0.503528639 | 0.92405258 | 1.0594664 | up | lncRNA |
| ENST00000424851 | AC068491.1 | 0.126166609 | 0.678900542 | 1.4553983 | up | lncRNA |
| ENST00000424853 | STARD13-AS2 | 0.25587274 | 0.822133742 | 1.0876659 | up | lncRNA |
| ENST00000424886 | AC105749.5 | 0.25959196 | 0.824094195 | 1.2370366 | up | lncRNA |
| ENST00000424904 | AC034193.5 | 0.650622222 | 0.969927925 | 1.0244456 | up | lncRNA |
| ENST00000424921 | AP001055.6 | 0.075992503 | 0.569109083 | 1.3754675 | up | lncRNA |
| ENST00000424941 | RP11-408H1.3 | 0.299807586 | 0.8422517 | 1.1452159 | up | lncRNA |
| ENST00000424968 | LINC00240 | 0.283139616 | 0.833653196 | 1.1675865 | up | lncRNA |
| ENST00000424982 | DPYD-IT1 | 2.13519E-05 | 0.020429495 | 1.9834174 | up | lncRNA |
| ENST00000425010 | AL157902.3 | 0.312081405 | 0.849269161 | 1.2971423 | up | lncRNA |
| ENST00000425045 | DNM1P46 | 0.731892001 | 0.983763148 | 1.0351021 | up | lncRNA |
| ENST00000425048 | RP11-114G1.2 | 0.299908552 | 0.8422517 | 1.2978682 | up | lncRNA |
| ENST00000425076 | RP11-342M1.3 | 0.442550405 | 0.90512482 | 1.1164926 | up | lncRNA |
| ENST00000425077 | AC013436.6 | 0.306374339 | 0.846407029 | 1.1588792 | up | lncRNA |
| ENST00000425089 | RP11-524H19.2 | 0.343510394 | 0.863514929 | 1.3815747 | up | lncRNA |
| ENST00000425109 | RP4-704D21.2 | 0.283286416 | 0.833653196 | 1.500074 | up | lncRNA |
| ENST00000425137 | RP11-218D6.4 | 0.129553651 | 0.682265946 | 1.384484 | up | lncRNA |
| ENST00000425150 | RP11-348F1.3 | 0.846960846 | 0.995583664 | 1.0369947 | up | lncRNA |
| ENST00000425153 | RP11-205K6.2 | 0.215837781 | 0.783396803 | 1.1750694 | up | lncRNA |
| ENST00000425161 | RP11-343H5.6 | 0.007438492 | 0.229585454 | 3.481609 | up | lncRNA |
| ENST00000425189 | DBH-AS1 | 0.153905921 | 0.711341825 | 1.4357042 | up | lncRNA |
| ENST00000425192 | AC073641.2 | 0.000281988 | 0.079354723 | 4.1113222 | up | lncRNA |
| ENST00000425194 | RP13-614K11.2 | 0.954613249 | 0.998875101 | 1.0255618 | up | lncRNA |
| ENST00000425205 | RP11-40H20.4 | 0.181745321 | 0.747823638 | 1.2522009 | up | lncRNA |
| ENST00000425212 | AC016735.2 | 0.327837053 | 0.854556781 | 1.1151151 | up | lncRNA |
| ENST00000425214 | CCDC144B | 0.744817071 | 0.985094857 | 1.2868175 | up | lncRNA |
| ENST00000425256 | GTF2IRD2P1 | 0.538066753 | 0.936418354 | 1.1338884 | up | lncRNA |
| ENST00000425271 | RP11-479J7.2 | 0.843459824 | 0.995583664 | 1.0799844 | up | lncRNA |
| ENST00000425290 | RP11-436D10.3 | 0.434453061 | 0.900880094 | 1.2730963 | up | lncRNA |
| ENST00000425333 | PCBP1-AS1 | 0.430816886 | 0.89909611 | 1.2956927 | up | lncRNA |
| ENST00000425350 | LINC00462 | 0.46613286 | 0.913821992 | 1.0838166 | up | lncRNA |
| ENST00000425352 | RP11-5P4.1 | 0.243471993 | 0.810061605 | 1.275669 | up | lncRNA |
| ENST00000425364 | RP11-428F8.2 | 0.756143737 | 0.98762882 | 1.0785544 | up | lncRNA |
| ENST00000425370 | RP11-123K19.1 | 0.267348946 | 0.829082126 | 1.1275494 | up | lncRNA |
| ENST00000425399 | AC131180.4 | 0.616868441 | 0.959290367 | 1.2173868 | up | lncRNA |
| ENST00000425412 | RP11-295G20.2 | 0.017439596 | 0.333873701 | 1.7230262 | up | lncRNA |
| ENST00000425425 | SDHAP2 | 0.984805632 | 0.998971647 | 1.0011904 | up | lncRNA |
| ENST00000425470 | AC009313.1 | 0.687579311 | 0.97861545 | 1.0817729 | up | lncRNA |
| ENST00000425481 | AC021016.6 | 0.741825166 | 0.985094857 | 1.0422034 | up | lncRNA |
| ENST00000425497 | RP11-290F20.3 | 0.053333764 | 0.515925762 | 1.1722907 | up | lncRNA |
| ENST00000425499 | RP11-112J3.15 | 0.357020491 | 0.872002738 | 1.0578095 | up | lncRNA |
| ENST00000425533 | TOPORS-AS1 | 0.634296314 | 0.96485646 | 1.0616308 | up | lncRNA |
| ENST00000425554 | RP11-399E6.1 | 0.586371599 | 0.950589384 | 1.0530997 | up | lncRNA |
| ENST00000425578 | RP11-363D14.1 | 0.678167851 | 0.976096237 | 1.071903 | up | lncRNA |
| ENST00000425586 | DLEU2 | 0.96854289 | 0.998875101 | 1.0027917 | up | lncRNA |
| ENST00000425587 | RP11-561O23.8 | 0.006563371 | 0.217096533 | 2.1918666 | up | lncRNA |
| ENST00000425588 | RP11-16C18.3 | 0.51488607 | 0.930031728 | 1.1697291 | up | lncRNA |
| ENST00000425595 | HOTAIR | 0.795994729 | 0.991420185 | 1.050246 | up | lncRNA |
| ENST00000425601 | PCBP1-AS1 | 0.780302049 | 0.99121281 | 1.0577909 | up | lncRNA |
| ENST00000425604 | ZNRD1-AS1 | 0.855081294 | 0.996142678 | 1.0683898 | up | lncRNA |
| ENST00000425648 | AL163953.3 | 0.940258251 | 0.998875101 | 1.017401 | up | lncRNA |
| ENST00000425653 | DANCR | 0.693160369 | 0.978978277 | 1.0337445 | up | lncRNA |
| ENST00000425698 | RP11-134P9.1 | 0.55294817 | 0.943023022 | 1.0582651 | up | lncRNA |
| ENST00000425712 | AC073871.2 | 0.434308159 | 0.900880094 | 1.144477 | up | lncRNA |
| ENST00000425740 | ASMTL-AS1 | 0.56846013 | 0.943923733 | 1.043921 | up | lncRNA |
| ENST00000425750 | RP11-76N22.2 | 0.711652566 | 0.98251396 | 1.0851418 | up | lncRNA |
| ENST00000425779 | AC107081.5 | 0.413346673 | 0.890927603 | 1.3550315 | up | lncRNA |
| ENST00000425797 | DTX2P1 | 0.207822448 | 0.775413441 | 1.2230625 | up | lncRNA |
| ENST00000425814 | hsa-mir-107 | 0.782254426 | 0.99121281 | 1.0272237 | up | lncRNA |
| ENST00000425939 | RP4-705D16.3 | 0.956035766 | 0.998875101 | 1.0133825 | up | lncRNA |
| ENST00000426017 | RP11-191G24.1 | 0.550236496 | 0.942167708 | 1.0538686 | up | lncRNA |
| ENST00000426030 | RP11-492I21.1 | 0.749136198 | 0.985210549 | 1.1439058 | up | lncRNA |
| ENST00000426044 | CTD-2666L21.1 | 0.948996221 | 0.998875101 | 1.0183038 | up | lncRNA |
| ENST00000426066 | LL22NC03-80A10.6 | 0.118102374 | 0.66612699 | 1.1726963 | up | lncRNA |
| ENST00000426067 | RP11-253D19.2 | 0.162765405 | 0.718236848 | 1.303205 | up | lncRNA |
| ENST00000426109 | AC091633.3 | 0.178387028 | 0.743651912 | 1.1523361 | up | lncRNA |
| ENST00000426178 | RP11-38C18.3 | 0.243339406 | 0.810061605 | 1.210046 | up | lncRNA |
| ENST00000426185 | HCG22 | 0.463386985 | 0.913747281 | 1.0542822 | up | lncRNA |
| ENST00000426270 | RP11-326F20.5 | 0.060367724 | 0.531246381 | 1.1570608 | up | lncRNA |
| ENST00000426350 | RP11-381O7.3 | 0.103002181 | 0.632351488 | 1.4208555 | up | lncRNA |
| ENST00000426354 | RP1-90G24.8 | 0.270970095 | 0.831567323 | 1.420942 | up | lncRNA |
| ENST00000426358 | RP11-465M18.1 | 0.235951848 | 0.805225869 | 1.4620897 | up | lncRNA |
| ENST00000426367 | RP1-315G1.3 | 0.970766332 | 0.998875101 | 1.0070529 | up | lncRNA |
| ENST00000426408 | AL583842.3 | 0.99400677 | 0.999156445 | 1.0016238 | up | lncRNA |
| ENST00000426471 | RP11-20F24.4 | 0.043816591 | 0.477853889 | 1.1143761 | up | lncRNA |
| ENST00000426495 | AC090939.2 | 0.385397276 | 0.88287657 | 1.1154473 | up | lncRNA |
| ENST00000426501 | HERC2P3 | 0.652509439 | 0.969927925 | 1.4066147 | up | lncRNA |
| ENST00000426528 | LL0XNC01-237H1.2 | 0.786864322 | 0.991316428 | 1.0482122 | up | lncRNA |
| ENST00000426697 | AY269186.2 | 0.13058974 | 0.684132143 | 1.2452336 | up | lncRNA |
| ENST00000426737 | ARMC2-AS1 | 0.124940027 | 0.67731705 | 1.9082122 | up | lncRNA |
| ENST00000426753 | RP5-1043L13.1 | 0.511082673 | 0.927835265 | 1.1169802 | up | lncRNA |
| ENST00000426775 | RP3-333A15.1 | 0.08970496 | 0.603136158 | 1.6248895 | up | lncRNA |
| ENST00000426786 | AL592284.1 | 0.021310004 | 0.359097712 | 1.3797418 | up | lncRNA |
| ENST00000426854 | RP4-738P15.1 | 0.077947729 | 0.575629572 | 1.4416514 | up | lncRNA |
| ENST00000426962 | AC093642.3 | 0.604784152 | 0.955931955 | 1.1345145 | up | lncRNA |
| ENST00000427019 | AC113607.3 | 0.100755479 | 0.628381708 | 1.1668439 | up | lncRNA |
| ENST00000427042 | AC092570.2 | 0.483570388 | 0.919439531 | 1.2188186 | up | lncRNA |
| ENST00000427046 | RP11-467D18.2 | 0.717844902 | 0.98251396 | 1.0846074 | up | lncRNA |
| ENST00000427049 | RP3-400B16.1 | 0.327605072 | 0.854556781 | 1.1355287 | up | lncRNA |
| ENST00000427054 | AC104654.2 | 0.003713939 | 0.176498201 | 2.0029164 | up | lncRNA |
| ENST00000427055 | ANKRD36BP2 | 0.952068748 | 0.998875101 | 1.0071522 | up | lncRNA |
| ENST00000427073 | RP5-1121A15.3 | 0.021224323 | 0.359097712 | 1.6979826 | up | lncRNA |
| ENST00000427076 | POLR2J4 | 0.629519946 | 0.963822303 | 1.0723162 | up | lncRNA |
| ENST00000427085 | AL132709.5 | 0.932358975 | 0.998875101 | 1.0085051 | up | lncRNA |
| ENST00000427109 | RP11-65J3.1 | 0.500344372 | 0.922148899 | 1.3424885 | up | lncRNA |
| ENST00000427154 | RP1-184J9.2 | 0.778494503 | 0.99121281 | 1.0436564 | up | lncRNA |
| ENST00000427169 | RP11-495P10.5 | 0.49163518 | 0.920777644 | 1.1758435 | up | lncRNA |
| ENST00000427243 | RP5-1033H22.2 | 0.419655733 | 0.894750301 | 1.2659972 | up | lncRNA |
| ENST00000427252 | AC000093.3 | 0.275895974 | 0.832297359 | 1.4254917 | up | lncRNA |
| ENST00000427273 | GRM7-AS1 | 0.100479862 | 0.628381708 | 1.3622958 | up | lncRNA |
| ENST00000427278 | ZEB2-AS1 | 0.324410543 | 0.853958969 | 1.386223 | up | lncRNA |
| ENST00000427292 | RP4-794H19.1 | 0.093718065 | 0.61382632 | 1.1149788 | up | lncRNA |
| ENST00000427303 | RP11-445H22.4 | 0.41717214 | 0.89285465 | 1.1598845 | up | lncRNA |
| ENST00000427317 | CAMTA1-IT1 | 0.925518014 | 0.998875101 | 1.0095139 | up | lncRNA |
| ENST00000427392 | AC073342.12 | 0.554080516 | 0.943023022 | 1.0908167 | up | lncRNA |
| ENST00000427415 | SDHAP1 | 0.793830381 | 0.991420185 | 1.0194863 | up | lncRNA |
| ENST00000427439 | RP11-16L9.3 | 0.230344913 | 0.79852974 | 1.1955423 | up | lncRNA |
| ENST00000427492 | RP11-537A6.9 | 0.579576393 | 0.948676882 | 1.1497651 | up | lncRNA |
| ENST00000427501 | SNHG5 | 0.459293123 | 0.91221792 | 1.1066391 | up | lncRNA |
| ENST00000427525 | SCAND2 | 0.665776642 | 0.972635141 | 1.0369019 | up | lncRNA |
| ENST00000427547 | AL162400.1 | 0.694303668 | 0.979750131 | 1.047809 | up | lncRNA |
| ENST00000427598 | RP11-445H22.4 | 0.433762554 | 0.900875689 | 1.1862292 | up | lncRNA |
| ENST00000427609 | RP1-90K10.4 | 0.795565447 | 0.991420185 | 1.0693814 | up | lncRNA |
| ENST00000427669 | RP4-717I23.3 | 0.81540965 | 0.99257857 | 1.091457 | up | lncRNA |
| ENST00000427732 | RP11-89F3.2 | 0.276234038 | 0.832297359 | 1.125385 | up | lncRNA |
| ENST00000427778 | RP11-492E3.51 | 0.764520489 | 0.989189555 | 1.0279728 | up | lncRNA |
| ENST00000427824 | RP3-467K16.4 | 0.00035474 | 0.084119144 | 11.8017566 | up | lncRNA |
| ENST00000427859 | CHRM3-AS2 | 0.497130317 | 0.921717611 | 1.2953084 | up | lncRNA |
| ENST00000427911 | AP000281.2 | 0.389261222 | 0.885276359 | 1.1124616 | up | lncRNA |
| ENST00000427974 | RP1-56L9.7 | 0.003302503 | 0.167945066 | 14.9775274 | up | lncRNA |
| ENST00000428006 | RP11-536O18.1 | 0.00433553 | 0.1879865 | 1.4323248 | up | lncRNA |
| ENST00000428008 | ZNFX1-AS1 | 0.26654009 | 0.828881561 | 1.1028891 | up | lncRNA |
| ENST00000428009 | RP4-723E3.1 | 0.141995508 | 0.698926614 | 1.7964617 | up | lncRNA |
| ENST00000428044 | RP11-390P2.4 | 0.000311035 | 0.082361798 | 1.3654074 | up | lncRNA |
| ENST00000428083 | RHOA-IT1 | 0.4852243 | 0.920060662 | 1.151126 | up | lncRNA |
| ENST00000428134 | AC087499.10 | 0.129372028 | 0.682265946 | 1.2932915 | up | lncRNA |
| ENST00000428190 | RP1-155G6.4 | 0.637353971 | 0.96564589 | 1.0326371 | up | lncRNA |
| ENST00000428244 | AC098971.2 | 0.65326146 | 0.97018076 | 1.0531055 | up | lncRNA |
| ENST00000428254 | RP4-694B14.5 | 0.983571801 | 0.998875101 | 1.0017452 | up | lncRNA |
| ENST00000428276 | RP11-175B12.2 | 0.518355056 | 0.930711226 | 1.256712 | up | lncRNA |
| ENST00000428292 | RP11-18B16.2 | 0.001699499 | 0.138586398 | 2.0239791 | up | lncRNA |
| ENST00000428300 | AC008268.2 | 0.537747515 | 0.936418354 | 1.0202546 | up | lncRNA |
| ENST00000428367 | MPRIP-AS1 | 0.251269163 | 0.817550448 | 1.1936845 | up | lncRNA |
| ENST00000428487 | AC012668.2 | 0.523739713 | 0.932117592 | 1.1697732 | up | lncRNA |
| ENST00000428504 | RP11-206L10.2 | 0.085995162 | 0.594686635 | 1.1366792 | up | lncRNA |
| ENST00000428508 | RP11-353N4.1 | 0.991587863 | 0.999156445 | 1.0079916 | up | lncRNA |
| ENST00000428531 | RP11-261N11.8 | 0.060337682 | 0.531246381 | 1.1376365 | up | lncRNA |
| ENST00000428541 | AC079305.10 | 0.046320503 | 0.485742152 | 1.381283 | up | lncRNA |
| ENST00000428557 | RP4-756H11.3 | 0.906910323 | 0.998875101 | 1.0108741 | up | lncRNA |
| ENST00000428561 | RP11-314A5.3 | 0.869178279 | 0.997455886 | 1.0159955 | up | lncRNA |
| ENST00000428576 | ANKRD20A11P | 0.923205808 | 0.998875101 | 1.0299675 | up | lncRNA |
| ENST00000428581 | RP11-444I9.3 | 0.932583577 | 0.998875101 | 1.0154973 | up | lncRNA |
| ENST00000428643 | HMGA1P4 | 0.187706478 | 0.753977992 | 1.1160015 | up | lncRNA |
| ENST00000428649 | FAM75C2 | 0.701381193 | 0.980170937 | 1.0538623 | up | lncRNA |
| ENST00000428651 | AC093375.1 | 0.335380324 | 0.858730619 | 1.2632114 | up | lncRNA |
| ENST00000428656 | DIAPH3-AS2 | 0.977926833 | 0.998875101 | 1.008139 | up | lncRNA |
| ENST00000428669 | LINC00478 | 0.163601826 | 0.718706279 | 1.1261337 | up | lncRNA |
| ENST00000428678 | UPK3BP1 | 0.966308197 | 0.998875101 | 1.0105296 | up | lncRNA |
| ENST00000428681 | AC093495.4 | 0.998109621 | 0.999477117 | 1.0003405 | up | lncRNA |
| ENST00000428732 | RP4-694A7.4 | 0.727040172 | 0.98251396 | 1.2743994 | up | lncRNA |
| ENST00000428752 | TMEM191A | 0.662631739 | 0.971730383 | 1.0521771 | up | lncRNA |
| ENST00000428769 | RP5-986I17.2 | 0.212344268 | 0.780026858 | 1.0586869 | up | lncRNA |
| ENST00000428777 | KLF7-IT1 | 0.063761866 | 0.537558012 | 1.582945 | up | lncRNA |
| ENST00000428818 | TTC28-AS1 | 0.256163432 | 0.822133742 | 2.0493328 | up | lncRNA |
| ENST00000428838 | RP5-1177I5.3 | 0.969196403 | 0.998875101 | 1.0044694 | up | lncRNA |
| ENST00000428862 | RP11-202P11.1 | 0.88723095 | 0.998875101 | 1.0154407 | up | lncRNA |
| ENST00000428888 | AC009495.4 | 0.951392487 | 0.998875101 | 1.0372957 | up | lncRNA |
| ENST00000428914 | LINC00310 | 0.504520347 | 0.92405258 | 1.229921 | up | lncRNA |
| ENST00000428936 | RP11-180I22.2 | 0.222126196 | 0.790839 | 1.1935735 | up | lncRNA |
| ENST00000429014 | AC004240.2 | 0.818075649 | 0.992651327 | 1.0374945 | up | lncRNA |
| ENST00000429037 | HLA-P | 0.881905593 | 0.998875101 | 1.033423 | up | lncRNA |
| ENST00000429044 | RP11-555F9.2 | 0.533375954 | 0.935381352 | 1.3017316 | up | lncRNA |
| ENST00000429070 | RP11-589B3.6 | 0.002583855 | 0.15074586 | 1.2707722 | up | lncRNA |
| ENST00000429074 | RP11-413E1.4 | 0.004724255 | 0.191133826 | 2.0541852 | up | lncRNA |
| ENST00000429099 | RP11-117D22.1 | 0.650050939 | 0.969927925 | 1.1924465 | up | lncRNA |
| ENST00000429104 | RP11-324H6.6 | 0.452322135 | 0.908696429 | 1.2489125 | up | lncRNA |
| ENST00000429110 | RP11-432B10.1 | 0.403802149 | 0.889146721 | 1.5552288 | up | lncRNA |
| ENST00000429132 | AL844908.5 | 0.850724106 | 0.995900317 | 1.0116515 | up | lncRNA |
| ENST00000429151 | AF230666.2 | 0.524373757 | 0.932117592 | 1.123241 | up | lncRNA |
| ENST00000429159 | MEG3 | 0.769133443 | 0.989189555 | 1.0500803 | up | lncRNA |
| ENST00000429207 | RP1-163M9.6 | 0.348558099 | 0.867156419 | 1.1099229 | up | lncRNA |
| ENST00000429210 | RP11-312O7.2 | 0.73052341 | 0.98341516 | 1.0233332 | up | lncRNA |
| ENST00000429224 | RP11-611D20.2 | 0.038396869 | 0.44875559 | 1.5770505 | up | lncRNA |
| ENST00000429250 | RP11-90J7.3 | 0.611137773 | 0.957448851 | 1.0540545 | up | lncRNA |
| ENST00000429268 | SHANK2-AS2 | 0.088544568 | 0.5997129 | 1.0806051 | up | lncRNA |
| ENST00000429388 | BX284650.1 | 0.007084407 | 0.224630786 | 1.3652846 | up | lncRNA |
| ENST00000429456 | AC093642.3 | 0.69122906 | 0.978695455 | 1.0694994 | up | lncRNA |
| ENST00000429482 | RP11-85O21.5 | 0.570509298 | 0.944646886 | 1.0591166 | up | lncRNA |
| ENST00000429505 | RP11-206L10.6 | 0.37243933 | 0.878574831 | 1.3711786 | up | lncRNA |
| ENST00000429521 | ANKRD20A11P | 0.542246625 | 0.938790368 | 1.0646625 | up | lncRNA |
| ENST00000429532 | AC093415.2 | 0.136430265 | 0.693269931 | 1.1951576 | up | lncRNA |
| ENST00000429599 | PCBP1-AS1 | 0.490731774 | 0.920777644 | 1.4082293 | up | lncRNA |
| ENST00000429614 | CCDC162P | 0.022602818 | 0.363379513 | 1.3259221 | up | lncRNA |
| ENST00000429618 | XXbac-B476C20.14 | 0.062640895 | 0.534813875 | 1.5504385 | up | lncRNA |
| ENST00000429634 | RP11-153K11.3 | 0.161691759 | 0.716676999 | 1.2462876 | up | lncRNA |
| ENST00000429648 | RP11-21N7.2 | 0.186983505 | 0.753717042 | 1.1548072 | up | lncRNA |
| ENST00000429680 | RP11-78C6.1 | 0.408769965 | 0.890173322 | 1.2469021 | up | lncRNA |
| ENST00000429681 | RP13-131K19.1 | 0.426241069 | 0.897780553 | 1.3258493 | up | lncRNA |
| ENST00000429698 | TPTE2P1 | 0.974789582 | 0.998875101 | 1.0099467 | up | lncRNA |
| ENST00000429707 | AC004980.7 | 0.018339838 | 0.339651019 | 1.262259 | up | lncRNA |
| ENST00000429796 | AC067945.4 | 0.296141346 | 0.839987155 | 1.2497545 | up | lncRNA |
| ENST00000429809 | RP11-159H3.2 | 0.347883236 | 0.866508192 | 1.1541971 | up | lncRNA |
| ENST00000429832 | KCNQ5-AS1 | 0.32325549 | 0.853556911 | 1.4933038 | up | lncRNA |
| ENST00000429859 | RP4-717I23.3 | 0.873545809 | 0.997909851 | 1.0580036 | up | lncRNA |
| ENST00000429872 | AC147651.4 | 0.387995492 | 0.88501451 | 1.2036811 | up | lncRNA |
| ENST00000429929 | AC008174.3 | 0.4173125 | 0.89285465 | 1.1675061 | up | lncRNA |
| ENST00000429947 | AC131097.3 | 0.04283289 | 0.470702635 | 1.3017889 | up | lncRNA |
| ENST00000429962 | THAP7-AS1 | 0.451763059 | 0.908696429 | 1.1024983 | up | lncRNA |
| ENST00000429998 | RP11-513I15.6 | 0.191708778 | 0.760520345 | 1.3367264 | up | lncRNA |
| ENST00000430014 | AL592284.1 | 0.664402648 | 0.972257778 | 1.0678858 | up | lncRNA |
| ENST00000430048 | AC012456.3 | 0.240279408 | 0.80919875 | 1.1238392 | up | lncRNA |
| ENST00000430057 | WWC3-AS1 | 0.449481283 | 0.907307366 | 1.0583418 | up | lncRNA |
| ENST00000430078 | RP3-393E18.2 | 0.780099725 | 0.99121281 | 1.0924783 | up | lncRNA |
| ENST00000430128 | AC018712.3 | 0.16258377 | 0.718236848 | 1.1194538 | up | lncRNA |
| ENST00000430181 | TSPEAR-AS1 | 0.871835732 | 0.997909851 | 1.0459396 | up | lncRNA |
| ENST00000430235 | LINC00106 | 0.820019068 | 0.993333549 | 1.0217855 | up | lncRNA |
| ENST00000430245 | GAS5 | 0.320233335 | 0.852213764 | 1.973096 | up | lncRNA |
| ENST00000430247 | AF131217.1 | 0.62619648 | 0.961734235 | 1.2022065 | up | lncRNA |
| ENST00000430306 | GGT3P | 0.239496971 | 0.808658327 | 1.1001858 | up | lncRNA |
| ENST00000430320 | RP1-65J11.1 | 0.592911431 | 0.950912706 | 1.2381496 | up | lncRNA |
| ENST00000430356 | RP11-298E9.3 | 0.904460424 | 0.998875101 | 1.0033246 | up | lncRNA |
| ENST00000430391 | AF127577.8 | 0.270509216 | 0.830995087 | 1.0610275 | up | lncRNA |
| ENST00000430428 | RP3-523C21.2 | 0.877255436 | 0.998682284 | 1.0232643 | up | lncRNA |
| ENST00000430520 | AC068490.1 | 0.000841311 | 0.104758688 | 6.0847723 | up | lncRNA |
| ENST00000430530 | ZNF815P | 0.408578964 | 0.890091879 | 1.1009418 | up | lncRNA |
| ENST00000430586 | AC092168.2 | 0.001786156 | 0.141460375 | 4.8038515 | up | lncRNA |
| ENST00000430595 | RP11-61I13.3 | 0.404414296 | 0.889198786 | 1.1126935 | up | lncRNA |
| ENST00000430598 | HERC2P3 | 0.863127632 | 0.996142678 | 1.0276026 | up | lncRNA |
| ENST00000430605 | NEGR1-AS1 | 0.129803913 | 0.682265946 | 1.5282105 | up | lncRNA |
| ENST00000430621 | AC034193.5 | 0.86819754 | 0.997224443 | 1.0531114 | up | lncRNA |
| ENST00000430647 | RP4-742C19.8 | 0.78674408 | 0.991316428 | 1.0415301 | up | lncRNA |
| ENST00000430672 | RP11-138M12.1 | 0.375863007 | 0.879312727 | 1.2198837 | up | lncRNA |
| ENST00000430733 | SPATA13-AS1 | 0.360860722 | 0.873342057 | 1.1061128 | up | lncRNA |
| ENST00000430748 | RP5-837I24.4 | 0.309965557 | 0.848664472 | 1.1223332 | up | lncRNA |
| ENST00000430772 | FTX | 0.413229703 | 0.890927603 | 1.061383 | up | lncRNA |
| ENST00000430801 | AC090421.1 | 0.022440768 | 0.363100225 | 1.202629 | up | lncRNA |
| ENST00000430820 | DDX26B-AS1 | 0.971118713 | 0.998875101 | 1.0095997 | up | lncRNA |
| ENST00000430825 | AC005534.9 | 0.206193799 | 0.773187432 | 1.0901682 | up | lncRNA |
| ENST00000430842 | RP1-20B11.2 | 0.293169415 | 0.838358604 | 1.2155449 | up | lncRNA |
| ENST00000430859 | AC005083.1 | 0.725772697 | 0.98251396 | 1.0439276 | up | lncRNA |
| ENST00000430907 | FTH1P10 | 0.14728832 | 0.703667281 | 1.1057593 | up | lncRNA |
| ENST00000430920 | RP5-1050D4.2 | 0.82497055 | 0.993856674 | 1.0678222 | up | lncRNA |
| ENST00000430963 | RP11-773D16.1 | 0.033634912 | 0.42757611 | 1.1668062 | up | lncRNA |
| ENST00000430978 | LINC00454 | 0.826750418 | 0.993856674 | 1.0717385 | up | lncRNA |
| ENST00000431017 | RP11-252P19.2 | 0.12905586 | 0.682265946 | 1.1536682 | up | lncRNA |
| ENST00000431027 | RP3-340N1.2 | 0.488416806 | 0.920777644 | 1.0957367 | up | lncRNA |
| ENST00000431043 | SNHG5 | 0.562170818 | 0.943393901 | 1.0772629 | up | lncRNA |
| ENST00000431095 | H19 | 0.772758528 | 0.990198305 | 1.0807103 | up | lncRNA |
| ENST00000431103 | DLG3-AS1 | 0.521803504 | 0.932117592 | 1.0764987 | up | lncRNA |
| ENST00000431108 | RP11-505P4.7 | 0.165157446 | 0.719579787 | 1.1245223 | up | lncRNA |
| ENST00000431144 | RP11-356I2.4 | 0.330136055 | 0.85556386 | 1.2449333 | up | lncRNA |
| ENST00000431145 | ZFY-AS1 | 0.218959379 | 0.787447617 | 1.2284712 | up | lncRNA |
| ENST00000431237 | CES5AP1 | 0.323457217 | 0.853615917 | 1.117046 | up | lncRNA |
| ENST00000431244 | AC159540.1 | 0.206619366 | 0.773246712 | 1.2254848 | up | lncRNA |
| ENST00000431308 | LRRC37BP1 | 0.299163628 | 0.842046751 | 1.0580371 | up | lncRNA |
| ENST00000431321 | RP4-669L17.4 | 0.980006985 | 0.998875101 | 1.0026209 | up | lncRNA |
| ENST00000431327 | EFCAB6-AS1 | 0.042544229 | 0.469333989 | 1.0998254 | up | lncRNA |
| ENST00000431376 | AC074011.2 | 0.401818209 | 0.889134718 | 1.1587164 | up | lncRNA |
| ENST00000431464 | RP11-308D16.4 | 0.112980819 | 0.653040963 | 1.3691275 | up | lncRNA |
| ENST00000431553 | RP11-69E11.4 | 0.645809981 | 0.968512523 | 1.0584717 | up | lncRNA |
| ENST00000431616 | LL0XNC01-157D4.1 | 0.288900248 | 0.837264328 | 1.1814414 | up | lncRNA |
| ENST00000431637 | LINC00210 | 0.432758386 | 0.900592919 | 1.1434889 | up | lncRNA |
| ENST00000431650 | AC012457.2 | 0.722507308 | 0.98251396 | 1.0464218 | up | lncRNA |
| ENST00000431697 | AC106053.1 | 0.360065456 | 0.873342057 | 1.3070644 | up | lncRNA |
| ENST00000431700 | BX004987.5 | 0.994098533 | 0.999156445 | 1.0013264 | up | lncRNA |
| ENST00000431730 | AC107070.1 | 0.429257603 | 0.898454882 | 1.0839606 | up | lncRNA |
| ENST00000431761 | KIAA0664L3 | 0.317304674 | 0.851223284 | 1.4017954 | up | lncRNA |
| ENST00000431796 | AC093642.5 | 0.7962647 | 0.991420185 | 1.0315448 | up | lncRNA |
| ENST00000431803 | CDC42-IT1 | 0.023647519 | 0.37091715 | 1.3639868 | up | lncRNA |
| ENST00000431856 | AC098820.3 | 0.29879134 | 0.841989654 | 1.1190154 | up | lncRNA |
| ENST00000431906 | RP11-512N4.3 | 0.874335382 | 0.997909851 | 1.0279685 | up | lncRNA |
| ENST00000431983 | RP11-380D15.3 | 0.61654993 | 0.959203037 | 1.1296238 | up | lncRNA |
| ENST00000431994 | SLC6A10P | 0.59771702 | 0.952312908 | 1.0648282 | up | lncRNA |
| ENST00000431997 | RP1-288M22.2 | 0.799244565 | 0.992228977 | 1.0514726 | up | lncRNA |
| ENST00000431999 | CYP1B1-AS1 | 0.80016189 | 0.992303072 | 1.0350267 | up | lncRNA |
| ENST00000432001 | AL589743.1 | 0.700636644 | 0.980170937 | 1.145885 | up | lncRNA |
| ENST00000432015 | ARHGAP26-AS1 | 0.255522381 | 0.821835543 | 1.1047907 | up | lncRNA |
| ENST00000432032 | AP000355.2 | 0.449402957 | 0.907276866 | 1.0695891 | up | lncRNA |
| ENST00000432047 | AC127904.2 | 0.199241873 | 0.766542641 | 1.2559114 | up | lncRNA |
| ENST00000432062 | RP4-537K23.4 | 0.055579168 | 0.520364745 | 1.1710775 | up | lncRNA |
| ENST00000432108 | RP11-219F10.1 | 0.752645601 | 0.986481248 | 1.0813639 | up | lncRNA |
| ENST00000432162 | RP11-86H7.1 | 0.464729005 | 0.913821992 | 1.1423873 | up | lncRNA |
| ENST00000432163 | AC098973.1 | 0.688305168 | 0.97861545 | 1.1052338 | up | lncRNA |
| ENST00000432211 | AC104655.2 | 0.721308934 | 0.98251396 | 1.0706756 | up | lncRNA |
| ENST00000432239 | RP5-1104E15.6 | 0.238138953 | 0.807531256 | 1.1612476 | up | lncRNA |
| ENST00000432267 | AC012307.3 | 0.819930524 | 0.993333549 | 1.0250515 | up | lncRNA |
| ENST00000432268 | AC017002.2 | 0.2820138 | 0.832297359 | 1.4867255 | up | lncRNA |
| ENST00000432331 | RP11-478K15.1 | 0.481451401 | 0.9192486 | 1.0649779 | up | lncRNA |
| ENST00000432334 | RP1-122P22.2 | 0.474859205 | 0.917441334 | 1.1644011 | up | lncRNA |
| ENST00000432373 | AP1B1P1 | 0.102610321 | 0.632351488 | 1.1205689 | up | lncRNA |
| ENST00000432395 | RP5-943J3.1 | 0.001803738 | 0.141460375 | 3.8161914 | up | lncRNA |
| ENST00000432411 | AP001619.2 | 0.846596671 | 0.995583664 | 1.0273945 | up | lncRNA |
| ENST00000432418 | RP11-123K19.1 | 0.408243413 | 0.889766054 | 1.1574865 | up | lncRNA |
| ENST00000432429 | RP11-242F24.1 | 0.213138236 | 0.781610853 | 1.1752088 | up | lncRNA |
| ENST00000432473 | Z83851.1 | 0.290077888 | 0.837264328 | 1.1066758 | up | lncRNA |
| ENST00000432488 | RP11-16L9.4 | 0.194370386 | 0.760765035 | 1.3229445 | up | lncRNA |
| ENST00000432502 | CTA-217C2.1 | 0.64260976 | 0.967175883 | 1.0413187 | up | lncRNA |
| ENST00000432511 | RP11-134P9.1 | 0.91663138 | 0.998875101 | 1.0162481 | up | lncRNA |
| ENST00000432536 | GAS5 | 0.060849177 | 0.532876797 | 1.4710888 | up | lncRNA |
| ENST00000432537 | LINC00466 | 0.664896015 | 0.972257778 | 1.0792563 | up | lncRNA |
| ENST00000432559 | RP11-219C24.10 | 0.652657547 | 0.969936263 | 1.1227335 | up | lncRNA |
| ENST00000432568 | CTA-85E5.10 | 0.474995751 | 0.917441334 | 1.1726675 | up | lncRNA |
| ENST00000432604 | PCBP1-AS1 | 0.443922353 | 0.90512482 | 1.2330988 | up | lncRNA |
| ENST00000432668 | AC005682.5 | 0.940093847 | 0.998875101 | 1.0232629 | up | lncRNA |
| ENST00000432673 | RP11-390P2.4 | 0.145265205 | 0.700631851 | 1.2560337 | up | lncRNA |
| ENST00000432697 | LINC00358 | 0.56882912 | 0.943923733 | 1.188664 | up | lncRNA |
| ENST00000432703 | RP11-415J8.5 | 0.246406803 | 0.813908042 | 1.1629118 | up | lncRNA |
| ENST00000432741 | RP4-717I23.3 | 0.723812874 | 0.98251396 | 1.0582433 | up | lncRNA |
| ENST00000432751 | RP11-328M4.2 | 0.806527355 | 0.992578294 | 1.0235519 | up | lncRNA |
| ENST00000432818 | AC068491.1 | 0.298949386 | 0.842046751 | 1.1399978 | up | lncRNA |
| ENST00000432822 | AC019055.1 | 0.123475055 | 0.67476384 | 1.2180793 | up | lncRNA |
| ENST00000432866 | AC018647.3 | 0.328576458 | 0.854918296 | 1.2845586 | up | lncRNA |
| ENST00000432892 | FAM197Y7 | 0.647027755 | 0.969646614 | 1.0320595 | up | lncRNA |
| ENST00000432908 | RP11-513G11.4 | 0.738662585 | 0.984700484 | 1.0947383 | up | lncRNA |
| ENST00000432960 | RP11-710A11.2 | 0.240301357 | 0.80919875 | 1.1335919 | up | lncRNA |
| ENST00000432994 | RP5-1063M23.2 | 0.042645067 | 0.46935736 | 1.2654953 | up | lncRNA |
| ENST00000433001 | AC003665.1 | 0.971466997 | 0.998875101 | 1.0117571 | up | lncRNA |
| ENST00000433005 | AC003075.4 | 0.622323997 | 0.960457826 | 1.1478624 | up | lncRNA |
| ENST00000433012 | AC016738.3 | 0.718291724 | 0.98251396 | 1.1189515 | up | lncRNA |
| ENST00000433033 | HNF1A-AS1 | 0.928375908 | 0.998875101 | 1.0103225 | up | lncRNA |
| ENST00000433059 | AC002398.5 | 0.161423195 | 0.716150139 | 1.1535213 | up | lncRNA |
| ENST00000433084 | RP11-403I13.8 | 0.385586768 | 0.88302877 | 1.1138025 | up | lncRNA |
| ENST00000433106 | AL078621.4 | 0.259019775 | 0.824094195 | 1.0765344 | up | lncRNA |
| ENST00000433110 | RP11-4C20.4 | 0.108600928 | 0.642195263 | 1.4363922 | up | lncRNA |
| ENST00000433116 | RP11-637A17.2 | 0.098796468 | 0.624143367 | 1.1947594 | up | lncRNA |
| ENST00000433121 | RP5-908M14.5 | 0.073134456 | 0.56397543 | 1.1689009 | up | lncRNA |
| ENST00000433182 | E2F3-IT1 | 0.16449939 | 0.719521661 | 1.1369312 | up | lncRNA |
| ENST00000433198 | AC068491.1 | 0.337377118 | 0.859588936 | 1.3270158 | up | lncRNA |
| ENST00000433232 | AC074138.3 | 0.322703119 | 0.853463264 | 1.0985116 | up | lncRNA |
| ENST00000433265 | CTC-338M12.3 | 0.937857247 | 0.998875101 | 1.009396 | up | lncRNA |
| ENST00000433290 | AC079776.2 | 0.709136918 | 0.981727542 | 1.1269579 | up | lncRNA |
| ENST00000433303 | AL035610.2 | 0.961721547 | 0.998875101 | 1.0100341 | up | lncRNA |
| ENST00000433308 | RP11-497D6.4 | 0.689608888 | 0.97861545 | 1.1451684 | up | lncRNA |
| ENST00000433310 | AF131217.1 | 0.930368965 | 0.998875101 | 1.0576621 | up | lncRNA |
| ENST00000433324 | AC097662.2 | 0.923247998 | 0.998875101 | 1.0155615 | up | lncRNA |
| ENST00000433344 | AJ006995.3 | 0.386065207 | 0.883419618 | 1.1112873 | up | lncRNA |
| ENST00000433357 | RP11-255A11.21 | 0.625248795 | 0.96133281 | 1.1168735 | up | lncRNA |
| ENST00000433391 | RP11-378J18.5 | 0.658013817 | 0.971704562 | 1.03292 | up | lncRNA |
| ENST00000433396 | AC007392.3 | 0.763842259 | 0.989015485 | 1.0761384 | up | lncRNA |
| ENST00000433419 | CCDC144C | 0.789091965 | 0.991420185 | 1.1452232 | up | lncRNA |
| ENST00000433431 | AC079776.7 | 0.639352197 | 0.96564589 | 1.0416712 | up | lncRNA |
| ENST00000433465 | LINC00205 | 0.394983031 | 0.885711362 | 1.0752952 | up | lncRNA |
| ENST00000433499 | SYP-AS1 | 0.442768702 | 0.90512482 | 1.1308725 | up | lncRNA |
| ENST00000433546 | RP11-284G10.1 | 0.000225917 | 0.072435429 | 1.901854 | up | lncRNA |
| ENST00000433581 | TCF7L1-IT1 | 0.148165147 | 0.704640207 | 1.741796 | up | lncRNA |
| ENST00000433598 | BX248398.1 | 0.003878724 | 0.179554598 | 1.5606588 | up | lncRNA |
| ENST00000433601 | AC091132.1 | 0.280009853 | 0.832297359 | 1.1493516 | up | lncRNA |
| ENST00000433614 | RP11-95H8.5 | 0.398357569 | 0.887618401 | 1.1533426 | up | lncRNA |
| ENST00000433671 | RP5-864K19.4 | 0.38596676 | 0.883335185 | 1.4261639 | up | lncRNA |
| ENST00000433673 | AC068282.3 | 0.814644364 | 0.99257857 | 1.2426953 | up | lncRNA |
| ENST00000433695 | RP11-465B22.3 | 0.167998552 | 0.724711516 | 1.4844338 | up | lncRNA |
| ENST00000433761 | RP1-40E16.2 | 0.306368562 | 0.846407029 | 1.2275071 | up | lncRNA |
| ENST00000433843 | SNHG5 | 0.240584002 | 0.809272444 | 1.1980159 | up | lncRNA |
| ENST00000433871 | CIDECP | 7.18638E-05 | 0.039668797 | 4.4297916 | up | lncRNA |
| ENST00000433873 | AC005324.6 | 0.189906483 | 0.756884941 | 1.3670289 | up | lncRNA |
| ENST00000433882 | BSN-AS2 | 0.980990382 | 0.998875101 | 1.0039251 | up | lncRNA |
| ENST00000433893 | AC007391.2 | 0.466566188 | 0.914151254 | 1.0230276 | up | lncRNA |
| ENST00000433905 | RP4-583P15.10 | 0.025442708 | 0.381452143 | 1.3380275 | up | lncRNA |
| ENST00000433918 | RP11-340I6.7 | 0.657212566 | 0.971290366 | 1.1730781 | up | lncRNA |
| ENST00000433945 | AC006028.10 | 0.96073884 | 0.998875101 | 1.021055 | up | lncRNA |
| ENST00000434081 | LINC00163 | 0.36332838 | 0.874234597 | 1.0416503 | up | lncRNA |
| ENST00000434097 | RP13-39P12.3 | 0.7935173 | 0.991420185 | 1.153599 | up | lncRNA |
| ENST00000434098 | RP11-85G21.2 | 0.554842767 | 0.943023022 | 1.1593512 | up | lncRNA |
| ENST00000434117 | LINC00353 | 0.714901844 | 0.98251396 | 1.0208212 | up | lncRNA |
| ENST00000434159 | AL592284.1 | 0.224225559 | 0.793609179 | 1.1816601 | up | lncRNA |
| ENST00000434218 | SERHL | 0.644937631 | 0.967912253 | 1.8125182 | up | lncRNA |
| ENST00000434221 | TTC28-AS1 | 0.080079511 | 0.581177305 | 1.0726301 | up | lncRNA |
| ENST00000434223 | RP11-1008C21.2 | 0.001107422 | 0.118308113 | 2.7265079 | up | lncRNA |
| ENST00000434255 | RP1-32B1.4 | 0.334572617 | 0.85798034 | 1.3636232 | up | lncRNA |
| ENST00000434273 | ENOX1-AS2 | 0.209391181 | 0.776733582 | 1.1133754 | up | lncRNA |
| ENST00000434292 | RP5-1077H22.1 | 0.115856243 | 0.65904431 | 1.3186074 | up | lncRNA |
| ENST00000434301 | AC092667.2 | 0.212516432 | 0.78025987 | 1.6849662 | up | lncRNA |
| ENST00000434321 | AC005019.3 | 0.86052322 | 0.996142678 | 1.027405 | up | lncRNA |
| ENST00000434375 | RP11-524G24.2 | 0.611839215 | 0.957636208 | 1.0602993 | up | lncRNA |
| ENST00000434384 | AC004383.5 | 0.078722724 | 0.577270023 | 1.0718973 | up | lncRNA |
| ENST00000434493 | RP1-177I10.1 | 0.397188404 | 0.887348439 | 1.2582501 | up | lncRNA |
| ENST00000434504 | AC083863.7 | 0.888821666 | 0.998875101 | 1.0388633 | up | lncRNA |
| ENST00000434510 | CTA-796E4.4 | 0.494729678 | 0.921717611 | 1.0833812 | up | lncRNA |
| ENST00000434531 | AC005522.7 | 0.526318857 | 0.93359637 | 1.0921267 | up | lncRNA |
| ENST00000434552 | RP11-490O24.2 | 0.557058285 | 0.943023022 | 1.0677385 | up | lncRNA |
| ENST00000434560 | RP1-86D1.2 | 0.001819154 | 0.141494996 | 2.1912483 | up | lncRNA |
| ENST00000434579 | RP11-203P23.1 | 0.070830189 | 0.559211931 | 1.1879016 | up | lncRNA |
| ENST00000434596 | RP11-503C24.3 | 0.568244784 | 0.943923733 | 1.0621229 | up | lncRNA |
| ENST00000434606 | RP13-221M14.3 | 0.953584442 | 0.998875101 | 1.0165621 | up | lncRNA |
| ENST00000434639 | RP11-261C10.3 | 0.818869576 | 0.992925826 | 1.035815 | up | lncRNA |
| ENST00000434661 | AC140481.7 | 0.651333363 | 0.969927925 | 1.0735915 | up | lncRNA |
| ENST00000434733 | AC003991.3 | 0.736023656 | 0.984025164 | 1.0273121 | up | lncRNA |
| ENST00000434779 | MTUS2-AS2 | 0.891228996 | 0.998875101 | 1.0137455 | up | lncRNA |
| ENST00000434790 | AC096579.13 | 0.07832685 | 0.5767814 | 1.2657743 | up | lncRNA |
| ENST00000434792 | RP11-127L20.3 | 0.690307858 | 0.97861545 | 1.0205722 | up | lncRNA |
| ENST00000434807 | AC012507.3 | 0.186191146 | 0.753336876 | 1.1292681 | up | lncRNA |
| ENST00000434828 | FER1L4 | 0.327667437 | 0.854556781 | 1.3946566 | up | lncRNA |
| ENST00000434849 | TPT1-AS1 | 0.395302743 | 0.885711362 | 1.233852 | up | lncRNA |
| ENST00000434902 | RP11-34C15.1 | 0.001245293 | 0.12293504 | 1.3202388 | up | lncRNA |
| ENST00000434914 | RP11-160C18.4 | 0.034030236 | 0.42757611 | 1.1264452 | up | lncRNA |
| ENST00000434938 | LINC00106 | 0.000317776 | 0.082361798 | 1.690063 | up | lncRNA |
| ENST00000434942 | RP1-90G24.10 | 0.010705736 | 0.261630988 | 1.109831 | up | lncRNA |
| ENST00000434951 | AC079742.4 | 0.935355241 | 0.998875101 | 1.0096724 | up | lncRNA |
| ENST00000434969 | RP11-71G7.1 | 0.427403953 | 0.897780553 | 1.4272929 | up | lncRNA |
| ENST00000435037 | ARHGEF7-AS1 | 0.849613658 | 0.995876116 | 1.0277035 | up | lncRNA |
| ENST00000435042 | AC004041.2 | 0.568033133 | 0.943923733 | 1.07243 | up | lncRNA |
| ENST00000435044 | RP11-490N5.1 | 0.117365431 | 0.664206886 | 1.1541373 | up | lncRNA |
| ENST00000435074 | HLA-DPB2 | 0.229644364 | 0.79852974 | 1.4197097 | up | lncRNA |
| ENST00000435097 | RP11-429J17.6 | 0.121835671 | 0.672928817 | 1.1138227 | up | lncRNA |
| ENST00000435108 | RP11-239E10.2 | 0.328492519 | 0.854918296 | 1.191404 | up | lncRNA |
| ENST00000435156 | RP11-203P2.2 | 0.697213948 | 0.980170937 | 1.0504452 | up | lncRNA |
| ENST00000435281 | LINC00439 | 0.031907669 | 0.420127403 | 1.8798767 | up | lncRNA |
| ENST00000435287 | RP11-69I8.3 | 0.28477953 | 0.834624426 | 1.1946014 | up | lncRNA |
| ENST00000435300 | RP11-206L10.10 | 0.55413949 | 0.943023022 | 1.1058436 | up | lncRNA |
| ENST00000435311 | RP5-898J17.1 | 0.554139603 | 0.943023022 | 1.4262696 | up | lncRNA |
| ENST00000435317 | RP11-464F9.9 | 0.293140414 | 0.838358604 | 1.1192399 | up | lncRNA |
| ENST00000435322 | AL163953.3 | 0.745149232 | 0.985094857 | 1.1451928 | up | lncRNA |
| ENST00000435380 | NEURL3 | 0.144359851 | 0.699853429 | 1.1808335 | up | lncRNA |
| ENST00000435389 | AC007392.3 | 0.452462791 | 0.908851781 | 1.3843662 | up | lncRNA |
| ENST00000435412 | C20orf90 | 0.530668365 | 0.934908142 | 1.0485241 | up | lncRNA |
| ENST00000435429 | RP3-470L22.1 | 0.080047734 | 0.581177305 | 1.4195922 | up | lncRNA |
| ENST00000435434 | RP11-127L20.6 | 0.000941184 | 0.108214114 | 3.2917676 | up | lncRNA |
| ENST00000435444 | RP11-29B9.2 | 0.155674591 | 0.711341825 | 1.0754107 | up | lncRNA |
| ENST00000435483 | RP11-244K5.4 | 0.131699334 | 0.686764863 | 1.2389559 | up | lncRNA |
| ENST00000435491 | AC091133.1 | 0.246969669 | 0.814268018 | 1.2459401 | up | lncRNA |
| ENST00000435492 | RP11-317P15.3 | 0.229129702 | 0.798130144 | 1.175578 | up | lncRNA |
| ENST00000435493 | LINC00323 | 0.006612058 | 0.217651979 | 1.7486171 | up | lncRNA |
| ENST00000435524 | RP5-1132H15.1 | 0.550808711 | 0.942167708 | 1.0666313 | up | lncRNA |
| ENST00000435526 | RP11-414H17.6 | 0.280056803 | 0.832297359 | 1.1444722 | up | lncRNA |
| ENST00000435539 | RP11-69C17.2 | 0.149103893 | 0.704640207 | 1.7148934 | up | lncRNA |
| ENST00000435548 | RP11-78H24.1 | 0.436383661 | 0.901926599 | 1.1279083 | up | lncRNA |
| ENST00000435554 | RP11-476H20.1 | 0.607476677 | 0.956065104 | 1.1257496 | up | lncRNA |
| ENST00000435585 | BX571672.5 | 0.665368381 | 0.972501549 | 1.1674218 | up | lncRNA |
| ENST00000435597 | RP11-1114A5.4 | 0.187988624 | 0.754266909 | 1.3619524 | up | lncRNA |
| ENST00000435612 | XXyac-YX65C7_A.3 | 0.22504276 | 0.794551181 | 1.1779473 | up | lncRNA |
| ENST00000435645 | RP11-14C22.4 | 0.290950584 | 0.837264328 | 1.3740097 | up | lncRNA |
| ENST00000435695 | AC002454.1 | 0.031261046 | 0.417820172 | 1.2389538 | up | lncRNA |
| ENST00000435697 | LINC00478 | 0.342918501 | 0.863514929 | 1.0915503 | up | lncRNA |
| ENST00000435718 | RP4-564F22.2 | 0.313380724 | 0.851097229 | 1.0655188 | up | lncRNA |
| ENST00000435726 | RP11-69D4.3 | 0.183782738 | 0.751257244 | 1.2031145 | up | lncRNA |
| ENST00000435731 | SDHAP1 | 0.159442252 | 0.714806047 | 1.0720323 | up | lncRNA |
| ENST00000435749 | MAGI2-AS3 | 0.143050291 | 0.69945173 | 1.5031429 | up | lncRNA |
| ENST00000435769 | TYW1B | 0.753641796 | 0.986873349 | 1.1173746 | up | lncRNA |
| ENST00000435789 | RP11-431J24.2 | 0.145987756 | 0.701210268 | 1.3307604 | up | lncRNA |
| ENST00000435793 | RP11-261C10.3 | 0.564688089 | 0.943393901 | 1.1312735 | up | lncRNA |
| ENST00000435800 | RP11-31F15.1 | 0.321911712 | 0.853269484 | 1.1206274 | up | lncRNA |
| ENST00000435925 | AC090774.2 | 0.497325162 | 0.921785486 | 1.1887721 | up | lncRNA |
| ENST00000435968 | AC005062.2 | 0.702346566 | 0.980170937 | 1.095852 | up | lncRNA |
| ENST00000436028 | AC024619.2 | 0.115831074 | 0.65904431 | 1.1308001 | up | lncRNA |
| ENST00000436039 | RP4-781K5.2 | 0.047547897 | 0.492357445 | 1.2345129 | up | lncRNA |
| ENST00000436056 | AC073115.7 | 0.386488938 | 0.883865828 | 1.3040259 | up | lncRNA |
| ENST00000436060 | LINC00606 | 0.232151688 | 0.79956859 | 1.2313002 | up | lncRNA |
| ENST00000436077 | RP11-20F24.2 | 0.835772565 | 0.995060648 | 1.0434859 | up | lncRNA |
| ENST00000436078 | RP11-91K9.1 | 0.047225852 | 0.490633373 | 1.4093338 | up | lncRNA |
| ENST00000436082 | AC021021.2 | 0.539541067 | 0.937437834 | 1.0840831 | up | lncRNA |
| ENST00000436112 | RP3-523C21.1 | 0.586526744 | 0.950589384 | 1.110694 | up | lncRNA |
| ENST00000436123 | AC098971.2 | 0.992060856 | 0.999156445 | 1.0007484 | up | lncRNA |
| ENST00000436140 | RP11-80K21.3 | 0.951838179 | 0.998875101 | 1.0117526 | up | lncRNA |
| ENST00000436151 | AC011899.9 | 0.884756464 | 0.998875101 | 1.0202075 | up | lncRNA |
| ENST00000436180 | RP11-508M1.3 | 0.647730109 | 0.969779566 | 1.0454633 | up | lncRNA |
| ENST00000436200 | RP4-717I23.3 | 0.948903629 | 0.998875101 | 1.0025971 | up | lncRNA |
| ENST00000436238 | MIAT | 0.516047986 | 0.930031728 | 1.0894647 | up | lncRNA |
| ENST00000436249 | RP11-637O19.2 | 0.084668659 | 0.590458988 | 1.1002046 | up | lncRNA |
| ENST00000436263 | RP11-358D14.2 | 0.414946769 | 0.892075779 | 1.105343 | up | lncRNA |
| ENST00000436303 | AP000688.11 | 0.900367265 | 0.998875101 | 1.009417 | up | lncRNA |
| ENST00000436304 | AC091729.9 | 0.41928777 | 0.894732097 | 1.0657194 | up | lncRNA |
| ENST00000436334 | RP5-1061H20.4 | 0.560410937 | 0.943393901 | 1.1858744 | up | lncRNA |
| ENST00000436340 | RP11-195B3.1 | 0.375058068 | 0.879312727 | 1.0948168 | up | lncRNA |
| ENST00000436416 | RP5-1160K1.3 | 0.829782429 | 0.994074191 | 1.0229563 | up | lncRNA |
| ENST00000436432 | KCNMB2-IT1 | 0.709413067 | 0.981727542 | 1.023145 | up | lncRNA |
| ENST00000436475 | LINC00466 | 0.110035262 | 0.645372325 | 1.4421056 | up | lncRNA |
| ENST00000436484 | XX-CR54.3 | 0.76299199 | 0.988817776 | 1.0190489 | up | lncRNA |
| ENST00000436492 | RP1-125N5.2 | 0.51259735 | 0.928771262 | 1.0868027 | up | lncRNA |
| ENST00000436493 | SDHAP3 | 0.194538155 | 0.760765035 | 1.1048529 | up | lncRNA |
| ENST00000436500 | RP11-514F8.2 | 0.60742856 | 0.956065104 | 1.1279848 | up | lncRNA |
| ENST00000436506 | AC009299.3 | 0.894480503 | 0.998875101 | 1.0413215 | up | lncRNA |
| ENST00000436510 | RP11-65J3.3 | 0.136261251 | 0.693269931 | 1.1973252 | up | lncRNA |
| ENST00000436539 | AP006285.6 | 0.496612382 | 0.921717611 | 1.225124 | up | lncRNA |
| ENST00000436551 | AC104654.2 | 0.041142883 | 0.462853115 | 2.4712949 | up | lncRNA |
| ENST00000436578 | AC005532.5 | 0.433625359 | 0.900875689 | 1.1667543 | up | lncRNA |
| ENST00000436616 | AC009948.5 | 0.734196069 | 0.983763148 | 1.0266901 | up | lncRNA |
| ENST00000436618 | TMEM191A | 0.852457584 | 0.995900317 | 1.0642071 | up | lncRNA |
| ENST00000436642 | RP5-930J4.2 | 0.380598 | 0.880821433 | 1.2304514 | up | lncRNA |
| ENST00000436671 | RP11-389K14.3 | 0.839689642 | 0.995583664 | 1.050577 | up | lncRNA |
| ENST00000436706 | RP11-365O16.3 | 0.305731293 | 0.846407029 | 1.1843415 | up | lncRNA |
| ENST00000436710 | RP11-65J3.1 | 0.647803561 | 0.969779566 | 1.0456729 | up | lncRNA |
| ENST00000436713 | RP1-92O14.6 | 0.215505854 | 0.783221074 | 1.0909328 | up | lncRNA |
| ENST00000436715 | H19 | 0.19710778 | 0.763327268 | 1.2688681 | up | lncRNA |
| ENST00000436716 | RP11-287A8.6 | 0.04700531 | 0.489564734 | 1.1788249 | up | lncRNA |
| ENST00000436739 | RP5-858B6.1 | 0.313030693 | 0.850391162 | 1.1689269 | up | lncRNA |
| ENST00000436756 | RP11-261C10.2 | 0.395284017 | 0.885711362 | 1.1262297 | up | lncRNA |
| ENST00000436764 | RP4-564F22.2 | 0.568670782 | 0.943923733 | 1.0264154 | up | lncRNA |
| ENST00000436845 | AP001422.3 | 0.129964289 | 0.682619986 | 1.1261117 | up | lncRNA |
| ENST00000436878 | AL035610.1 | 0.494443888 | 0.921717611 | 1.0688669 | up | lncRNA |
| ENST00000436893 | MID1IP1-AS1 | 0.009447397 | 0.253701351 | 1.3416918 | up | lncRNA |
| ENST00000436934 | HERC2P3 | 0.913339806 | 0.998875101 | 1.0131532 | up | lncRNA |
| ENST00000436942 | RP11-462G8.3 | 0.899416126 | 0.998875101 | 1.0243145 | up | lncRNA |
| ENST00000436950 | AC074289.1 | 0.264315011 | 0.827574498 | 1.0874241 | up | lncRNA |
| ENST00000437012 | XXyac-YX60D10.1 | 0.876840782 | 0.998660569 | 1.0251175 | up | lncRNA |
| ENST00000437040 | RP1-67A8.3 | 0.33938628 | 0.860518281 | 1.1429557 | up | lncRNA |
| ENST00000437091 | AC034220.3 | 0.422139797 | 0.895936649 | 1.1697381 | up | lncRNA |
| ENST00000437132 | AC114808.3 | 0.339244786 | 0.860518281 | 1.1700511 | up | lncRNA |
| ENST00000437157 | RP3-510D11.1 | 0.221457235 | 0.790635384 | 1.4569069 | up | lncRNA |
| ENST00000437190 | RP11-296O14.3 | 0.097457605 | 0.62265608 | 1.2735385 | up | lncRNA |
| ENST00000437201 | SBDSP1 | 0.839814907 | 0.995583664 | 1.035411 | up | lncRNA |
| ENST00000437204 | RP11-493K19.3 | 0.414205916 | 0.891256868 | 2.8182509 | up | lncRNA |
| ENST00000437233 | AC084149.2 | 0.705473808 | 0.980849889 | 1.1340187 | up | lncRNA |
| ENST00000437261 | AC108066.1 | 0.549665506 | 0.942167708 | 1.3067131 | up | lncRNA |
| ENST00000437289 | RP11-526P5.2 | 0.587866543 | 0.950589384 | 1.4948687 | up | lncRNA |
| ENST00000437294 | GUSBP11 | 0.338072277 | 0.86050695 | 1.1476285 | up | lncRNA |
| ENST00000437308 | RP4-655J12.4 | 0.053395423 | 0.515925762 | 1.2243856 | up | lncRNA |
| ENST00000437331 | DLX6-AS1 | 0.891274085 | 0.998875101 | 1.0253403 | up | lncRNA |
| ENST00000437557 | AP001057.1 | 0.813972261 | 0.99257857 | 1.0574109 | up | lncRNA |
| ENST00000437559 | RP11-456H18.2 | 0.4088564 | 0.890173322 | 1.1252239 | up | lncRNA |
| ENST00000437561 | LINC00152 | 0.159496685 | 0.714806047 | 1.6414161 | up | lncRNA |
| ENST00000437589 | AC017076.4 | 0.395830036 | 0.885711362 | 1.0615787 | up | lncRNA |
| ENST00000437597 | AC046143.7 | 0.188732827 | 0.756051633 | 1.9147039 | up | lncRNA |
| ENST00000437610 | AC011995.3 | 0.182014324 | 0.748286903 | 1.1765958 | up | lncRNA |
| ENST00000437631 | RP13-221M14.2 | 0.650839504 | 0.969927925 | 1.068067 | up | lncRNA |
| ENST00000437691 | RP11-261C10.2 | 0.782690906 | 0.99121281 | 1.05842 | up | lncRNA |
| ENST00000437697 | RP11-190J1.7 | 0.308832216 | 0.847582029 | 1.1284953 | up | lncRNA |
| ENST00000437712 | RP11-168K11.3 | 0.08398718 | 0.588566413 | 1.2054123 | up | lncRNA |
| ENST00000437730 | RP5-995J12.2 | 0.592338541 | 0.950912706 | 1.2530809 | up | lncRNA |
| ENST00000437753 | RP11-184I16.4 | 0.39501384 | 0.885711362 | 1.1272227 | up | lncRNA |
| ENST00000437774 | AFG3L1P | 0.597375713 | 0.952287813 | 1.0731075 | up | lncRNA |
| ENST00000437797 | RP11-315I20.1 | 0.000451997 | 0.088863929 | 1.2658036 | up | lncRNA |
| ENST00000437798 | AC079779.7 | 0.17834385 | 0.743651912 | 1.6420673 | up | lncRNA |
| ENST00000437830 | XX-CR54.1 | 0.028832605 | 0.406089833 | 1.5627995 | up | lncRNA |
| ENST00000437831 | RP11-441L11.1 | 0.491596735 | 0.920777644 | 1.1700816 | up | lncRNA |
| ENST00000437838 | RP11-498J9.2 | 0.397719777 | 0.887348439 | 1.0861962 | up | lncRNA |
| ENST00000437864 | RP11-244N9.4 | 0.636400357 | 0.96564589 | 1.0922617 | up | lncRNA |
| ENST00000437867 | C13orf44-AS1 | 0.478575265 | 0.918024039 | 1.0826648 | up | lncRNA |
| ENST00000437898 | RP1-43E13.2 | 0.663832726 | 0.971990255 | 1.0578036 | up | lncRNA |
| ENST00000437930 | RP11-399O19.5 | 0.272067567 | 0.832297359 | 1.2607686 | up | lncRNA |
| ENST00000438000 | BX571672.1 | 0.366317607 | 0.875064962 | 1.4004908 | up | lncRNA |
| ENST00000438010 | RP11-508M1.5 | 0.376236436 | 0.879423449 | 1.0868345 | up | lncRNA |
| ENST00000438017 | RP4-613B23.1 | 0.647710008 | 0.969779566 | 1.1041282 | up | lncRNA |
| ENST00000438080 | RP11-177A2.4 | 0.248510728 | 0.814856288 | 1.1811639 | up | lncRNA |
| ENST00000438131 | KRT42P | 0.019108561 | 0.344796409 | 1.2302851 | up | lncRNA |
| ENST00000438158 | RP11-400N13.3 | 0.844621772 | 0.995583664 | 1.0846713 | up | lncRNA |
| ENST00000438181 | RP11-342D14.1 | 0.782994901 | 0.99121281 | 1.0383091 | up | lncRNA |
| ENST00000438195 | RP4-794H19.4 | 0.060120533 | 0.531246381 | 1.5368122 | up | lncRNA |
| ENST00000438217 | XX-C2158C12.2 | 0.350463391 | 0.867400431 | 1.2042577 | up | lncRNA |
| ENST00000438219 | HCFC1-AS1 | 0.992945365 | 0.999156445 | 1.0028256 | up | lncRNA |
| ENST00000438246 | RP11-414H17.8 | 0.108170187 | 0.64098205 | 1.2590009 | up | lncRNA |
| ENST00000438248 | CFL1P1 | 0.300128649 | 0.8422517 | 1.1688532 | up | lncRNA |
| ENST00000438275 | RP11-280O1.2 | 0.415234804 | 0.892075779 | 1.0973241 | up | lncRNA |
| ENST00000438368 | AC092296.1 | 0.399671778 | 0.888347161 | 1.2496383 | up | lncRNA |
| ENST00000438378 | AC093838.4 | 0.677747387 | 0.976096237 | 1.0381873 | up | lncRNA |
| ENST00000438380 | RP11-175D17.3 | 0.332813258 | 0.85731789 | 1.0535587 | up | lncRNA |
| ENST00000438401 | RP5-845O24.8 | 0.361964709 | 0.873747871 | 1.2024955 | up | lncRNA |
| ENST00000438431 | RP11-214D15.2 | 0.997017547 | 0.999156445 | 1.0006584 | up | lncRNA |
| ENST00000438488 | RP11-197K6.1 | 0.729120909 | 0.982841582 | 1.0307123 | up | lncRNA |
| ENST00000438538 | AC003092.1 | 0.323799438 | 0.853615917 | 1.4711072 | up | lncRNA |
| ENST00000438589 | RP11-543D5.2 | 0.054192157 | 0.516622604 | 1.1387557 | up | lncRNA |
| ENST00000438640 | LINC00350 | 0.345049141 | 0.86477904 | 1.065729 | up | lncRNA |
| ENST00000438705 | SNHG15 | 3.47697E-05 | 0.025006259 | 2.2382443 | up | lncRNA |
| ENST00000438736 | AC016735.3 | 0.20910835 | 0.776733582 | 1.2039615 | up | lncRNA |
| ENST00000438824 | AC007879.6 | 0.298968455 | 0.842046751 | 1.1313951 | up | lncRNA |
| ENST00000438893 | AP000356.2 | 0.121352717 | 0.672928817 | 1.8533585 | up | lncRNA |
| ENST00000438905 | RP11-748L13.1 | 0.776813868 | 0.991030431 | 1.0642491 | up | lncRNA |
| ENST00000438934 | DGCR5 | 0.394916991 | 0.885711362 | 1.0817657 | up | lncRNA |
| ENST00000438935 | AC007390.6 | 0.705678481 | 0.980849889 | 1.0513007 | up | lncRNA |
| ENST00000438967 | RP11-298J23.5 | 0.16243159 | 0.718236848 | 1.0924632 | up | lncRNA |
| ENST00000439058 | AC005083.1 | 0.00634244 | 0.21515583 | 1.4893904 | up | lncRNA |
| ENST00000439074 | RP11-528A4.2 | 0.899724358 | 0.998875101 | 1.0075292 | up | lncRNA |
| ENST00000439076 | RP11-229P13.15 | 0.708507311 | 0.981609897 | 1.0411333 | up | lncRNA |
| ENST00000439104 | AP006216.10 | 0.101177213 | 0.630249723 | 1.4114913 | up | lncRNA |
| ENST00000439157 | RP11-94M14.2 | 0.277459115 | 0.832297359 | 1.1529825 | up | lncRNA |
| ENST00000439177 | RP11-30B1.1 | 0.545878927 | 0.939898322 | 1.0286291 | up | lncRNA |
| ENST00000439180 | MYCNOS | 0.799793573 | 0.992228977 | 1.0291741 | up | lncRNA |
| ENST00000439192 | DGUOK-AS1 | 0.00040764 | 0.084789048 | 11.0068527 | up | lncRNA |
| ENST00000439196 | AC092159.3 | 0.093268317 | 0.612906085 | 1.2672255 | up | lncRNA |
| ENST00000439208 | AC017076.4 | 0.025793954 | 0.382432679 | 1.1553226 | up | lncRNA |
| ENST00000439232 | SNORA71B | 0.791597497 | 0.991420185 | 1.0269476 | up | lncRNA |
| ENST00000439245 | RP11-327I22.4 | 0.853058999 | 0.996142678 | 1.0382179 | up | lncRNA |
| ENST00000439270 | AC114730.3 | 0.812250873 | 0.99257857 | 1.034113 | up | lncRNA |
| ENST00000439293 | ENTPD3-AS1 | 0.048945798 | 0.501307136 | 2.3247399 | up | lncRNA |
| ENST00000439298 | RP11-228B15.4 | 0.976835953 | 0.998875101 | 1.0034205 | up | lncRNA |
| ENST00000439325 | AC026202.3 | 0.113767091 | 0.654979643 | 1.1269117 | up | lncRNA |
| ENST00000439341 | RP5-858B6.3 | 0.851410678 | 0.995900317 | 1.1300198 | up | lncRNA |
| ENST00000439364 | RP5-982E9.1 | 0.20795523 | 0.775616804 | 1.306478 | up | lncRNA |
| ENST00000439386 | RP11-328M4.2 | 0.021458304 | 0.359357733 | 1.1959923 | up | lncRNA |
| ENST00000439413 | RP11-745C15.2 | 0.654291892 | 0.970670225 | 1.1368052 | up | lncRNA |
| ENST00000439433 | MEIS1-AS2 | 4.24174E-05 | 0.026468475 | 4.3311243 | up | lncRNA |
| ENST00000439434 | RP11-265D19.6 | 0.712891753 | 0.98251396 | 1.0426864 | up | lncRNA |
| ENST00000439438 | RP11-305L7.6 | 0.511375603 | 0.9278461 | 1.0833657 | up | lncRNA |
| ENST00000439444 | RP4-553F4.6 | 0.035536662 | 0.432931851 | 1.5179771 | up | lncRNA |
| ENST00000439446 | AC005083.1 | 0.947395148 | 0.998875101 | 1.0067943 | up | lncRNA |
| ENST00000439473 | DPY19L2P2 | 0.590582086 | 0.950912706 | 1.0417015 | up | lncRNA |
| ENST00000439494 | AC068491.1 | 0.17663967 | 0.74061716 | 1.466832 | up | lncRNA |
| ENST00000439529 | RP4-742J24.2 | 0.562898378 | 0.943393901 | 1.0920567 | up | lncRNA |
| ENST00000439562 | RP11-261C10.5 | 0.949400516 | 0.998875101 | 1.0144134 | up | lncRNA |
| ENST00000439577 | RP1-20B21.4 | 0.517276041 | 0.93019846 | 1.0186624 | up | lncRNA |
| ENST00000439588 | RP11-247I13.8 | 0.082496968 | 0.585414993 | 1.3461732 | up | lncRNA |
| ENST00000439621 | RP11-334A14.8 | 0.061435374 | 0.534457693 | 1.5657881 | up | lncRNA |
| ENST00000439670 | PCBP1-AS1 | 0.843558192 | 0.995583664 | 1.0386218 | up | lncRNA |
| ENST00000439694 | AC009264.1 | 0.809579101 | 0.992578294 | 1.0733373 | up | lncRNA |
| ENST00000439719 | RP11-403I13.4 | 0.745570156 | 0.985094857 | 1.0304297 | up | lncRNA |
| ENST00000439786 | RP4-781B1.2 | 0.392718757 | 0.885711362 | 1.1496779 | up | lncRNA |
| ENST00000439788 | RP3-467K16.7 | 0.228185513 | 0.798130144 | 1.0892019 | up | lncRNA |
| ENST00000439794 | RP11-344E13.3 | 0.14423106 | 0.699853429 | 1.2201385 | up | lncRNA |
| ENST00000439804 | RP11-197K6.1 | 0.851574152 | 0.995900317 | 1.020656 | up | lncRNA |
| ENST00000439810 | RP11-33A14.1 | 0.964951755 | 0.998875101 | 1.0063387 | up | lncRNA |
| ENST00000439823 | AC002480.4 | 0.000305188 | 0.082361798 | 1.7302591 | up | lncRNA |
| ENST00000439870 | AC079807.2 | 0.106566786 | 0.638286705 | 1.1734352 | up | lncRNA |
| ENST00000439876 | RP11-4E23.2 | 0.21820155 | 0.785656962 | 1.193765 | up | lncRNA |
| ENST00000439892 | PCBP1-AS1 | 0.231174745 | 0.79852974 | 1.4008157 | up | lncRNA |
| ENST00000439912 | SNORA71A | 0.618843826 | 0.95998809 | 1.0147168 | up | lncRNA |
| ENST00000439926 | RP11-402P6.11 | 0.189804801 | 0.756884941 | 1.0634614 | up | lncRNA |
| ENST00000439955 | AC009404.2 | 0.574769348 | 0.946756534 | 1.1424416 | up | lncRNA |
| ENST00000439960 | RP11-296L22.8 | 0.102044505 | 0.632351488 | 1.1562001 | up | lncRNA |
| ENST00000439964 | AC007365.3 | 0.307627383 | 0.847137912 | 1.2920168 | up | lncRNA |
| ENST00000439972 | ATP8B5P | 0.554579534 | 0.943023022 | 1.2328611 | up | lncRNA |
| ENST00000440013 | ZMYND10-AS1 | 0.293562289 | 0.838742488 | 1.0472028 | up | lncRNA |
| ENST00000440028 | MSL3P1 | 0.620685068 | 0.9603355 | 1.0531304 | up | lncRNA |
| ENST00000440038 | RP4-669L17.10 | 0.247887682 | 0.814489937 | 1.0835908 | up | lncRNA |
| ENST00000440084 | RP11-277L2.2 | 0.469422036 | 0.91468974 | 1.0979885 | up | lncRNA |
| ENST00000440100 | RP4-739H11.3 | 0.110182234 | 0.645443029 | 1.1076282 | up | lncRNA |
| ENST00000440123 | RP11-302K17.7 | 0.619455418 | 0.960028194 | 1.0483512 | up | lncRNA |
| ENST00000440196 | RP5-857K21.3 | 0.029274735 | 0.410303709 | 1.3953018 | up | lncRNA |
| ENST00000440220 | RP11-415D17.3 | 0.578718837 | 0.948580716 | 1.1807442 | up | lncRNA |
| ENST00000440221 | AL773572.7 | 0.692224829 | 0.97895216 | 1.214585 | up | lncRNA |
| ENST00000440249 | RP11-486B10.3 | 0.291238948 | 0.837264328 | 1.0843383 | up | lncRNA |
| ENST00000440351 | AC020743.4 | 0.434820918 | 0.901162429 | 1.138758 | up | lncRNA |
| ENST00000440357 | RP4-738P15.1 | 0.558042442 | 0.943023022 | 1.0480056 | up | lncRNA |
| ENST00000440377 | RP11-337C18.8 | 0.593722982 | 0.951058356 | 1.0594971 | up | lncRNA |
| ENST00000440388 | RP11-500G10.5 | 0.499224894 | 0.921805853 | 1.0775593 | up | lncRNA |
| ENST00000440414 | RP11-499O7.7 | 0.113613158 | 0.654586931 | 1.5593958 | up | lncRNA |
| ENST00000440441 | HERC2P3 | 0.179727529 | 0.745659191 | 1.595341 | up | lncRNA |
| ENST00000440451 | CCDC162P | 0.129215479 | 0.682265946 | 1.1307093 | up | lncRNA |
| ENST00000440477 | RP11-46E17.6 | 0.72804083 | 0.982841582 | 1.0640142 | up | lncRNA |
| ENST00000440568 | AC034193.5 | 0.290889946 | 0.837264328 | 1.1496112 | up | lncRNA |
| ENST00000440578 | RP11-356I2.2 | 0.020001716 | 0.352820395 | 3.6467137 | up | lncRNA |
| ENST00000440595 | RP5-1125A11.1 | 0.468829039 | 0.914494873 | 1.1078261 | up | lncRNA |
| ENST00000440629 | AP001432.14 | 0.243532194 | 0.810061605 | 1.4010108 | up | lncRNA |
| ENST00000440657 | USP12-AS1 | 0.407828441 | 0.889546439 | 1.2032514 | up | lncRNA |
| ENST00000440674 | RP11-358M14.2 | 0.664635139 | 0.972257778 | 1.0479413 | up | lncRNA |
| ENST00000440688 | RP5-1028L10.2 | 0.002402965 | 0.145414844 | 1.3612801 | up | lncRNA |
| ENST00000440719 | BX248398.1 | 0.054084723 | 0.516622604 | 2.539121 | up | lncRNA |
| ENST00000440729 | RP5-956O18.2 | 0.008759574 | 0.247532275 | 1.1943106 | up | lncRNA |
| ENST00000440750 | RP11-534L6.2 | 0.172446012 | 0.734089358 | 1.1450696 | up | lncRNA |
| ENST00000440762 | RP4-598G3.1 | 0.713477802 | 0.98251396 | 1.0941358 | up | lncRNA |
| ENST00000440763 | AF121898.3 | 0.14589823 | 0.701026582 | 1.0940304 | up | lncRNA |
| ENST00000440778 | RP4-717I23.3 | 0.568693766 | 0.943923733 | 1.1721672 | up | lncRNA |
| ENST00000440802 | AC097532.2 | 0.127712103 | 0.680549576 | 1.3536139 | up | lncRNA |
| ENST00000440860 | LINC00354 | 0.647155329 | 0.969646614 | 1.2268516 | up | lncRNA |
| ENST00000440862 | BX284650.1 | 0.024275484 | 0.374301178 | 1.4002839 | up | lncRNA |
| ENST00000440874 | TRIM31-AS1 | 0.281223278 | 0.832297359 | 1.5483519 | up | lncRNA |
| ENST00000440887 | KCNQ1-AS1 | 0.803327346 | 0.992578294 | 1.0375668 | up | lncRNA |
| ENST00000440889 | RP4-614C15.3 | 0.036334179 | 0.436010148 | 1.4765636 | up | lncRNA |
| ENST00000440971 | AC002467.7 | 0.796137521 | 0.991420185 | 1.0419653 | up | lncRNA |
| ENST00000440985 | RP11-269F19.2 | 0.06802351 | 0.550752865 | 1.258819 | up | lncRNA |
| ENST00000441013 | LINC00158 | 0.099395896 | 0.625122655 | 1.2700884 | up | lncRNA |
| ENST00000441075 | AC068491.1 | 0.479670053 | 0.918546036 | 1.0652273 | up | lncRNA |
| ENST00000441101 | RP11-374M1.3 | 0.760560778 | 0.988045734 | 1.0213132 | up | lncRNA |
| ENST00000441146 | GK-IT1 | 0.002615079 | 0.151532046 | 2.6601421 | up | lncRNA |
| ENST00000441167 | XXbac-B476C20.13 | 0.237549168 | 0.806512655 | 1.1629475 | up | lncRNA |
| ENST00000441214 | RP11-290F20.2 | 0.343089667 | 0.863514929 | 1.4120984 | up | lncRNA |
| ENST00000441215 | AC126365.1 | 0.316009509 | 0.851223284 | 1.2828401 | up | lncRNA |
| ENST00000441231 | PLCB1-IT1 | 0.617198467 | 0.959348191 | 1.0753101 | up | lncRNA |
| ENST00000441238 | TGFA-IT1 | 0.193702683 | 0.760765035 | 1.1195111 | up | lncRNA |
| ENST00000441245 | RP5-857K21.4 | 0.987197977 | 0.99906281 | 1.0028545 | up | lncRNA |
| ENST00000441266 | AC068134.8 | 0.476213993 | 0.917441334 | 1.1435568 | up | lncRNA |
| ENST00000441272 | RP11-66D17.3 | 0.556150806 | 0.943023022 | 1.1423482 | up | lncRNA |
| ENST00000441277 | RP13-379L11.1 | 0.42005524 | 0.894854783 | 1.2993514 | up | lncRNA |
| ENST00000441283 | LINC00313 | 0.55476521 | 0.943023022 | 1.0496232 | up | lncRNA |
| ENST00000441287 | AC011193.1 | 0.350005672 | 0.867400431 | 1.2263421 | up | lncRNA |
| ENST00000441289 | RP11-247I13.7 | 0.774501197 | 0.990435061 | 1.0419163 | up | lncRNA |
| ENST00000441295 | RP11-153N17.1 | 0.547443046 | 0.940721096 | 1.3593921 | up | lncRNA |
| ENST00000441331 | RP11-135J2.4 | 0.158653798 | 0.714689048 | 1.1598786 | up | lncRNA |
| ENST00000441338 | RP11-278H7.4 | 0.119443641 | 0.667912342 | 1.2821316 | up | lncRNA |
| ENST00000441348 | RP11-472G21.2 | 0.067452488 | 0.548255059 | 1.1982638 | up | lncRNA |
| ENST00000441360 | RP4-781K5.5 | 0.060789545 | 0.532876797 | 1.2690767 | up | lncRNA |
| ENST00000441399 | RP5-842K24.2 | 0.14224654 | 0.698941469 | 1.1624627 | up | lncRNA |
| ENST00000441414 | FMR1-IT1 | 0.098750477 | 0.624143367 | 1.7170735 | up | lncRNA |
| ENST00000441444 | AC144525.1 | 0.186088996 | 0.753336876 | 1.1826209 | up | lncRNA |
| ENST00000441539 | AC007566.10 | 0.981005465 | 0.998875101 | 1.0064322 | up | lncRNA |
| ENST00000441558 | MORC2-AS1 | 0.181383184 | 0.747757045 | 1.2089481 | up | lncRNA |
| ENST00000441570 | LINC00472 | 0.195929974 | 0.762235583 | 1.1832845 | up | lncRNA |
| ENST00000441613 | UBE2Q1-AS1 | 0.711310317 | 0.98251396 | 1.0923348 | up | lncRNA |
| ENST00000441660 | RP5-827L5.1 | 0.042492972 | 0.469333989 | 1.7625499 | up | lncRNA |
| ENST00000441665 | AP002856.4 | 0.863101385 | 0.996142678 | 1.0321181 | up | lncRNA |
| ENST00000441699 | FKBP9L | 0.478599572 | 0.918024039 | 1.1367094 | up | lncRNA |
| ENST00000441741 | RP11-69E11.4 | 0.635237812 | 0.965194977 | 1.0641048 | up | lncRNA |
| ENST00000441773 | RP11-38C18.3 | 0.656809861 | 0.971290366 | 1.0524159 | up | lncRNA |
| ENST00000441776 | RP11-344L13.2 | 0.397525218 | 0.887348439 | 1.2938721 | up | lncRNA |
| ENST00000441790 | RP11-392O17.1 | 0.157439763 | 0.713946155 | 1.4546015 | up | lncRNA |
| ENST00000441841 | RP3-473B4.3 | 0.320225003 | 0.852213764 | 1.1772306 | up | lncRNA |
| ENST00000442008 | RP11-116G8.5 | 0.059926363 | 0.531246381 | 1.1091775 | up | lncRNA |
| ENST00000442049 | RP11-403I13.4 | 0.07600362 | 0.569109083 | 1.1544006 | up | lncRNA |
| ENST00000442069 | RP11-143M1.2 | 0.836087718 | 0.995078046 | 1.048814 | up | lncRNA |
| ENST00000442072 | RP11-440G5.2 | 0.066968129 | 0.547601226 | 1.3355148 | up | lncRNA |
| ENST00000442133 | RP11-137L10.6 | 0.621969739 | 0.960457826 | 1.0660007 | up | lncRNA |
| ENST00000442150 | MDC1-AS1 | 0.30883182 | 0.847582029 | 1.1233717 | up | lncRNA |
| ENST00000442188 | RP11-107I14.2 | 0.677594879 | 0.976096237 | 1.0684397 | up | lncRNA |
| ENST00000442197 | AL132709.8 | 0.613097491 | 0.957994032 | 1.0936852 | up | lncRNA |
| ENST00000442213 | AC093642.5 | 0.239271501 | 0.808658327 | 1.3159041 | up | lncRNA |
| ENST00000442226 | RP3-340N1.5 | 0.261094517 | 0.82544125 | 1.3063475 | up | lncRNA |
| ENST00000442252 | AC002480.5 | 0.001920902 | 0.141494996 | 5.8842273 | up | lncRNA |
| ENST00000442274 | RP11-956J14.1 | 0.188296089 | 0.75486745 | 1.4764567 | up | lncRNA |
| ENST00000442293 | AC068491.1 | 0.128572588 | 0.682171456 | 1.6115925 | up | lncRNA |
| ENST00000442305 | RP3-395M20.3 | 0.173250757 | 0.735214331 | 1.2282954 | up | lncRNA |
| ENST00000442326 | AC016700.3 | 0.616611428 | 0.959203037 | 1.0450382 | up | lncRNA |
| ENST00000442329 | AC009948.3 | 0.744581861 | 0.985094857 | 1.0792804 | up | lncRNA |
| ENST00000442358 | RP11-404O13.1 | 0.256804709 | 0.822133742 | 1.1845128 | up | lncRNA |
| ENST00000442382 | RP4-781K5.6 | 0.577491649 | 0.947948855 | 1.108275 | up | lncRNA |
| ENST00000442417 | AC093818.1 | 0.174632934 | 0.736164062 | 1.1186393 | up | lncRNA |
| ENST00000442432 | RP11-326F20.5 | 0.005253501 | 0.201643161 | 1.1743035 | up | lncRNA |
| ENST00000442435 | RP11-54A4.2 | 0.336608281 | 0.859588936 | 1.1186715 | up | lncRNA |
| ENST00000442440 | RP3-322G13.5 | 0.744847678 | 0.985094857 | 1.0343098 | up | lncRNA |
| ENST00000442500 | LINC00454 | 0.487459846 | 0.920771743 | 1.2634302 | up | lncRNA |
| ENST00000442533 | RP1-215K18.1 | 0.383656504 | 0.882562871 | 1.2118157 | up | lncRNA |
| ENST00000442558 | RP11-147C23.1 | 0.828191426 | 0.994074191 | 1.0637026 | up | lncRNA |
| ENST00000442583 | CCDC144B | 0.853830375 | 0.996142678 | 1.0332019 | up | lncRNA |
| ENST00000442637 | RP5-1103G7.4 | 0.715841652 | 0.98251396 | 1.0531715 | up | lncRNA |
| ENST00000442749 | CNTN4-AS1 | 0.05204177 | 0.513193572 | 1.3748714 | up | lncRNA |
| ENST00000442751 | RP11-88H9.2 | 0.125224913 | 0.67731705 | 1.8825943 | up | lncRNA |
| ENST00000442765 | MAGI2-AS3 | 0.261376695 | 0.82544125 | 1.2340819 | up | lncRNA |
| ENST00000442794 | AC096558.1 | 0.348804561 | 0.867156419 | 1.2703179 | up | lncRNA |
| ENST00000442821 | AC009236.2 | 0.862546281 | 0.996142678 | 1.0211871 | up | lncRNA |
| ENST00000442849 | RP11-520B13.5 | 0.206280287 | 0.773187432 | 1.5209587 | up | lncRNA |
| ENST00000442956 | LINC00299 | 0.375684837 | 0.879312727 | 1.3826624 | up | lncRNA |
| ENST00000442957 | WT1-AS | 0.22533904 | 0.79487168 | 1.1990627 | up | lncRNA |
| ENST00000442967 | AC017096.1 | 0.522018411 | 0.932117592 | 1.1080491 | up | lncRNA |
| ENST00000443030 | AC092168.4 | 0.360110067 | 0.873342057 | 1.0820735 | up | lncRNA |
| ENST00000443123 | AC006227.1 | 0.003271455 | 0.167945066 | 1.3408767 | up | lncRNA |
| ENST00000443154 | RP11-386I8.6 | 0.523398272 | 0.932117592 | 1.1044395 | up | lncRNA |
| ENST00000443161 | RP5-864K19.4 | 0.138891118 | 0.69776743 | 1.834466 | up | lncRNA |
| ENST00000443163 | RP11-395D3.1 | 0.547780194 | 0.940847434 | 1.0937144 | up | lncRNA |
| ENST00000443171 | RP4-553F4.6 | 0.989634689 | 0.999156445 | 1.0007695 | up | lncRNA |
| ENST00000443207 | RP4-580N22.2 | 0.071420428 | 0.559511998 | 1.187639 | up | lncRNA |
| ENST00000443252 | AL132709.5 | 0.651737033 | 0.969927925 | 1.0607385 | up | lncRNA |
| ENST00000443294 | RP11-480I12.5 | 0.167285589 | 0.723499899 | 1.2041619 | up | lncRNA |
| ENST00000443319 | AP003025.2 | 0.0120444 | 0.280618888 | 1.8425267 | up | lncRNA |
| ENST00000443346 | AC136289.1 | 0.600236619 | 0.953841565 | 1.0489997 | up | lncRNA |
| ENST00000443347 | RP11-374M1.2 | 0.370154736 | 0.876933109 | 1.3801809 | up | lncRNA |
| ENST00000443374 | RP11-50E11.3 | 0.029849999 | 0.413940496 | 1.1181946 | up | lncRNA |
| ENST00000443380 | RP11-235G24.1 | 0.070756278 | 0.559211931 | 1.2895265 | up | lncRNA |
| ENST00000443406 | AC005029.1 | 0.105645396 | 0.635999463 | 2.1550206 | up | lncRNA |
| ENST00000443504 | RP11-686D16.1 | 0.617231864 | 0.959348191 | 1.0578141 | up | lncRNA |
| ENST00000443508 | RP11-344E13.3 | 0.345315203 | 0.864915148 | 1.0734329 | up | lncRNA |
| ENST00000443548 | BX004987.4 | 0.984066772 | 0.998875101 | 1.0038641 | up | lncRNA |
| ENST00000443554 | LINC00420 | 0.88714825 | 0.998875101 | 1.2002761 | up | lncRNA |
| ENST00000443562 | RP5-1007M22.2 | 0.091607051 | 0.608114895 | 1.2816957 | up | lncRNA |
| ENST00000443587 | DLEU2 | 0.131852429 | 0.687126382 | 1.5822232 | up | lncRNA |
| ENST00000443622 | RP11-176D17.3 | 0.254286847 | 0.820374967 | 1.1669058 | up | lncRNA |
| ENST00000443685 | RP11-662M24.1 | 0.02390746 | 0.3733622 | 1.2528198 | up | lncRNA |
| ENST00000443770 | ANKRD36BP2 | 0.488768122 | 0.920777644 | 1.086158 | up | lncRNA |
| ENST00000443772 | RP11-206L10.9 | 0.318733721 | 0.851223284 | 1.1879048 | up | lncRNA |
| ENST00000443776 | RP11-407B7.1 | 0.643925833 | 0.967175883 | 1.1489372 | up | lncRNA |
| ENST00000443783 | LINC00229 | 0.345080098 | 0.86477904 | 1.0807799 | up | lncRNA |
| ENST00000443837 | RNF216-IT1 | 0.983009031 | 0.998875101 | 1.0024338 | up | lncRNA |
| ENST00000443838 | RP11-536D16.2 | 0.462167329 | 0.912913065 | 1.1497663 | up | lncRNA |
| ENST00000443921 | PRINS | 0.038831773 | 0.451631772 | 1.4067039 | up | lncRNA |
| ENST00000443930 | RP11-547D24.1 | 0.034290698 | 0.42757611 | 1.568912 | up | lncRNA |
| ENST00000443935 | AC004019.13 | 0.148597668 | 0.704640207 | 1.279863 | up | lncRNA |
| ENST00000443946 | AC016747.3 | 0.250820195 | 0.817015761 | 1.1216753 | up | lncRNA |
| ENST00000443966 | GNAS-AS1 | 0.538780827 | 0.936942013 | 1.1218566 | up | lncRNA |
| ENST00000443988 | AC018730.1 | 0.614657303 | 0.958423841 | 1.0805537 | up | lncRNA |
| ENST00000443993 | RP11-492E3.2 | 0.000547353 | 0.094750704 | 2.1562012 | up | lncRNA |
| ENST00000444023 | MT1JP | 0.047304299 | 0.490897542 | 1.1904894 | up | lncRNA |
| ENST00000444038 | RP1-8B1.4 | 0.944823354 | 0.998875101 | 1.0071524 | up | lncRNA |
| ENST00000444046 | DSCAM-AS1 | 0.509165337 | 0.926768238 | 1.0377299 | up | lncRNA |
| ENST00000444093 | KB-318B8.7 | 0.697165481 | 0.980170937 | 1.1210336 | up | lncRNA |
| ENST00000444112 | AC005808.3 | 0.141303907 | 0.698568819 | 1.4248172 | up | lncRNA |
| ENST00000444126 | HCG18 | 0.6621015 | 0.971730383 | 1.0894522 | up | lncRNA |
| ENST00000444188 | XXyac-YX65C7_A.2 | 0.808814802 | 0.992578294 | 1.0221002 | up | lncRNA |
| ENST00000444200 | AP006216.12 | 0.159790643 | 0.714806047 | 1.1895161 | up | lncRNA |
| ENST00000444220 | GUSBP11 | 0.407048776 | 0.889505404 | 1.0892816 | up | lncRNA |
| ENST00000444229 | RP11-527F13.1 | 0.463014059 | 0.913747281 | 1.1583217 | up | lncRNA |
| ENST00000444266 | AC007403.1 | 0.525029448 | 0.932692492 | 1.0540412 | up | lncRNA |
| ENST00000444301 | AC068491.1 | 0.078036045 | 0.575629572 | 1.1640972 | up | lncRNA |
| ENST00000444320 | PCBP1-AS1 | 0.982859879 | 0.998875101 | 1.0068215 | up | lncRNA |
| ENST00000444330 | RP11-527D7.1 | 0.309808758 | 0.848664472 | 1.1177092 | up | lncRNA |
| ENST00000444349 | RP11-182I10.3 | 0.847512665 | 0.995583664 | 1.0588304 | up | lncRNA |
| ENST00000444357 | AL589743.2 | 0.36007693 | 0.873342057 | 1.2655181 | up | lncRNA |
| ENST00000444359 | RP11-316M21.6 | 0.491978938 | 0.920777644 | 1.0802548 | up | lncRNA |
| ENST00000444374 | RP11-316P17.2 | 0.66096543 | 0.971704562 | 1.1276139 | up | lncRNA |
| ENST00000444388 | CTA-211A9.5 | 0.582162895 | 0.949239022 | 1.0533187 | up | lncRNA |
| ENST00000444406 | AC093391.2 | 0.690178212 | 0.97861545 | 1.1827552 | up | lncRNA |
| ENST00000444410 | PCBP1-AS1 | 0.311372994 | 0.849269161 | 1.2077429 | up | lncRNA |
| ENST00000444433 | RP13-439H18.4 | 0.731861516 | 0.983763148 | 1.0191761 | up | lncRNA |
| ENST00000444436 | RP5-1031J8.1 | 0.943863253 | 0.998875101 | 1.0135093 | up | lncRNA |
| ENST00000444464 | AC084809.3 | 0.137550502 | 0.695137712 | 1.4725537 | up | lncRNA |
| ENST00000444465 | LINC00473 | 0.952223946 | 0.998875101 | 1.0167562 | up | lncRNA |
| ENST00000444489 | RP13-507I23.1 | 0.625567552 | 0.96133281 | 1.2296153 | up | lncRNA |
| ENST00000444529 | RP11-181G12.2 | 0.803030167 | 0.992578294 | 1.0404277 | up | lncRNA |
| ENST00000444563 | RP11-342M1.3 | 0.818692645 | 0.992881067 | 1.0201319 | up | lncRNA |
| ENST00000444568 | RP11-198M6.5 | 0.009610475 | 0.25448255 | 1.7778784 | up | lncRNA |
| ENST00000444586 | RP3-495K2.3 | 0.931818029 | 0.998875101 | 1.0279262 | up | lncRNA |
| ENST00000444660 | RP11-139J15.2 | 0.869437928 | 0.997455886 | 1.0493867 | up | lncRNA |
| ENST00000444663 | C13orf44-AS1 | 0.186499381 | 0.753717042 | 1.2336472 | up | lncRNA |
| ENST00000444665 | RP11-57H12.5 | 0.96351566 | 0.998875101 | 1.0047173 | up | lncRNA |
| ENST00000444682 | RP11-96L14.7 | 0.111312544 | 0.650206605 | 1.149832 | up | lncRNA |
| ENST00000444722 | RP13-228J13.1 | 0.700773487 | 0.980170937 | 1.0581599 | up | lncRNA |
| ENST00000444728 | AD000090.2 | 0.32551051 | 0.854556781 | 1.1407808 | up | lncRNA |
| ENST00000444745 | RP11-358M3.1 | 0.088977263 | 0.601480869 | 1.2936467 | up | lncRNA |
| ENST00000444755 | SEPT7P2 | 0.780062618 | 0.99121281 | 1.106874 | up | lncRNA |
| ENST00000444809 | ZNF733P | 0.012348099 | 0.283551867 | 1.2504043 | up | lncRNA |
| ENST00000444873 | AC009970.1 | 0.055021808 | 0.519180132 | 1.224203 | up | lncRNA |
| ENST00000444879 | CHL1-AS2 | 0.954223862 | 0.998875101 | 1.0055222 | up | lncRNA |
| ENST00000444883 | AL592284.1 | 0.011472104 | 0.271964925 | 2.0887469 | up | lncRNA |
| ENST00000444887 | RP11-445P19.1 | 0.708158309 | 0.981606358 | 1.0253355 | up | lncRNA |
| ENST00000444923 | RP4-745E8.2 | 0.670872276 | 0.974341942 | 1.0479989 | up | lncRNA |
| ENST00000444933 | RP3-477O4.14 | 0.789166629 | 0.991420185 | 1.0149146 | up | lncRNA |
| ENST00000444965 | RP11-348J12.2 | 0.586738343 | 0.950589384 | 1.1549755 | up | lncRNA |
| ENST00000444978 | RP3-380B8.3 | 0.329664445 | 0.85540421 | 1.312732 | up | lncRNA |
| ENST00000444986 | HCG14 | 0.104876302 | 0.635942989 | 1.1116291 | up | lncRNA |
| ENST00000445003 | RP11-290F20.3 | 0.024384866 | 0.374301178 | 1.1134074 | up | lncRNA |
| ENST00000445039 | RP4-784A16.5 | 0.162650918 | 0.718236848 | 1.2162201 | up | lncRNA |
| ENST00000445051 | RP11-4E23.2 | 0.911934564 | 0.998875101 | 1.0057173 | up | lncRNA |
| ENST00000445072 | RP11-373J16.1 | 0.189367339 | 0.756484902 | 1.4869767 | up | lncRNA |
| ENST00000445091 | U82695.5 | 0.51024743 | 0.927088381 | 1.0370127 | up | lncRNA |
| ENST00000445172 | RP1-153P14.3 | 0.165509967 | 0.720254409 | 1.0281506 | up | lncRNA |
| ENST00000445184 | RP11-325F22.3 | 0.017152904 | 0.330420153 | 1.117215 | up | lncRNA |
| ENST00000445225 | RP11-277L2.2 | 0.718272205 | 0.98251396 | 1.1011624 | up | lncRNA |
| ENST00000445272 | RP1-28O10.1 | 0.180177849 | 0.745729299 | 1.2708581 | up | lncRNA |
| ENST00000445306 | RP11-119F19.2 | 0.512870776 | 0.928980962 | 1.1966846 | up | lncRNA |
| ENST00000445382 | AC017099.3 | 0.08252452 | 0.585414993 | 1.3040108 | up | lncRNA |
| ENST00000445398 | AC008103.5 | 0.949253834 | 0.998875101 | 1.0085547 | up | lncRNA |
| ENST00000445485 | RP11-125K10.4 | 0.21453916 | 0.781610853 | 1.1757013 | up | lncRNA |
| ENST00000445520 | AC007246.3 | 0.92791602 | 0.998875101 | 1.0185838 | up | lncRNA |
| ENST00000445532 | AP001271.3 | 0.774331536 | 0.990435061 | 1.0713589 | up | lncRNA |
| ENST00000445544 | HCG16 | 0.062938637 | 0.53608031 | 1.7020122 | up | lncRNA |
| ENST00000445565 | RP11-418J17.1 | 0.180088242 | 0.745729299 | 1.1217702 | up | lncRNA |
| ENST00000445586 | AF196972.9 | 0.730579795 | 0.98341516 | 1.0307426 | up | lncRNA |
| ENST00000445600 | RP11-547D24.3 | 0.661750306 | 0.971704562 | 1.0136894 | up | lncRNA |
| ENST00000445662 | AL583842.1 | 0.212060243 | 0.780026858 | 1.4185807 | up | lncRNA |
| ENST00000445680 | RP11-552M11.4 | 0.401524538 | 0.8890764 | 1.0461897 | up | lncRNA |
| ENST00000445681 | GS1-124K5.4 | 0.962305409 | 0.998875101 | 1.0108046 | up | lncRNA |
| ENST00000445730 | RP11-525A16.1 | 0.406831141 | 0.889505404 | 1.2474891 | up | lncRNA |
| ENST00000445745 | AC016683.6 | 0.196757516 | 0.763206451 | 1.7205043 | up | lncRNA |
| ENST00000445808 | RP11-452K12.4 | 0.054107719 | 0.516622604 | 1.176044 | up | lncRNA |
| ENST00000445868 | RP11-462G8.2 | 0.89959403 | 0.998875101 | 1.016345 | up | lncRNA |
| ENST00000445878 | AC104695.3 | 0.860551241 | 0.996142678 | 1.0272663 | up | lncRNA |
| ENST00000445956 | RP4-813D12.2 | 0.502378396 | 0.923357158 | 1.0838342 | up | lncRNA |
| ENST00000445974 | RP11-437J19.1 | 0.105513267 | 0.635999463 | 1.310025 | up | lncRNA |
| ENST00000445984 | RP5-907D15.2 | 0.363863136 | 0.874818743 | 1.1055761 | up | lncRNA |
| ENST00000446001 | GFOD1-AS1 | 0.198970892 | 0.766200761 | 1.1181179 | up | lncRNA |
| ENST00000446002 | RP11-412H9.2 | 0.672797196 | 0.974876341 | 1.1992059 | up | lncRNA |
| ENST00000446029 | AC079612.2 | 0.724323116 | 0.98251396 | 1.0386454 | up | lncRNA |
| ENST00000446184 | RP11-473E2.3 | 0.126262915 | 0.678900542 | 1.1726065 | up | lncRNA |
| ENST00000446192 | AC073342.12 | 0.698035245 | 0.980170937 | 1.0408489 | up | lncRNA |
| ENST00000446201 | RP11-406A20.1 | 0.198485238 | 0.765216944 | 1.2527547 | up | lncRNA |
| ENST00000446211 | RP11-51B10.2 | 0.002110063 | 0.141857833 | 2.0031402 | up | lncRNA |
| ENST00000446220 | RP11-10N16.3 | 0.281614517 | 0.832297359 | 1.1777176 | up | lncRNA |
| ENST00000446281 | AC087859.1 | 0.363886908 | 0.874818743 | 1.0351108 | up | lncRNA |
| ENST00000446290 | RP11-274B18.2 | 0.579284298 | 0.948676882 | 1.0591426 | up | lncRNA |
| ENST00000446347 | RP11-978I15.10 | 0.135083553 | 0.693269931 | 1.2547872 | up | lncRNA |
| ENST00000446358 | RP3-399L15.1 | 0.365856169 | 0.874818743 | 1.622266 | up | lncRNA |
| ENST00000446373 | DPY19L2P2 | 0.428381085 | 0.897780553 | 1.0818909 | up | lncRNA |
| ENST00000446401 | AC104653.1 | 0.012530639 | 0.285740719 | 1.2621068 | up | lncRNA |
| ENST00000446404 | AP000402.3 | 0.171819871 | 0.733318903 | 1.3475743 | up | lncRNA |
| ENST00000446473 | RP1-7G5.6 | 0.010531462 | 0.261616866 | 1.2066236 | up | lncRNA |
| ENST00000446476 | RP5-826L7.1 | 0.120326991 | 0.669556486 | 1.3769516 | up | lncRNA |
| ENST00000446492 | AC093391.2 | 0.016931846 | 0.328386288 | 1.4748072 | up | lncRNA |
| ENST00000446528 | RP4-717I23.3 | 0.783516293 | 0.99121281 | 1.0853866 | up | lncRNA |
| ENST00000446578 | Z83851.3 | 0.669426866 | 0.974341942 | 1.0470129 | up | lncRNA |
| ENST00000446590 | AC016683.5 | 0.165304805 | 0.719579787 | 1.7038065 | up | lncRNA |
| ENST00000446592 | CCDC26 | 0.407400984 | 0.889546439 | 1.3469782 | up | lncRNA |
| ENST00000446628 | CEACAM22P | 0.906327842 | 0.998875101 | 1.0169767 | up | lncRNA |
| ENST00000446649 | FTCD-AS1 | 0.137463203 | 0.695137712 | 1.512625 | up | lncRNA |
| ENST00000446686 | RP4-784A16.4 | 0.325435658 | 0.854556781 | 1.0871144 | up | lncRNA |
| ENST00000446698 | AC007246.3 | 0.760361857 | 0.988045734 | 1.0601857 | up | lncRNA |
| ENST00000446713 | RP11-298J20.1 | 0.979763857 | 0.998875101 | 1.0037633 | up | lncRNA |
| ENST00000446733 | RP11-550C4.6 | 0.42016833 | 0.894854783 | 1.3231757 | up | lncRNA |
| ENST00000446754 | RP11-321L2.2 | 0.71831305 | 0.98251396 | 1.1593359 | up | lncRNA |
| ENST00000446789 | MCF2L-AS1 | 0.297516326 | 0.840892921 | 1.1398242 | up | lncRNA |
| ENST00000446849 | RP11-97N19.2 | 0.960967943 | 0.998875101 | 1.0028381 | up | lncRNA |
| ENST00000446887 | RP1-111C20.3 | 0.47444217 | 0.917441334 | 1.1380511 | up | lncRNA |
| ENST00000446911 | AC017096.1 | 0.868862783 | 0.997455886 | 1.0225159 | up | lncRNA |
| ENST00000446945 | LYST-IT1 | 0.133257762 | 0.689055558 | 1.163621 | up | lncRNA |
| ENST00000446954 | RP11-244K5.1 | 0.000663543 | 0.094750704 | 1.6608103 | up | lncRNA |
| ENST00000446986 | SLC25A5-AS1 | 0.305730445 | 0.846407029 | 1.0773531 | up | lncRNA |
| ENST00000446989 | AL845154.2 | 0.032075383 | 0.421176484 | 1.68918 | up | lncRNA |
| ENST00000447019 | AC019186.1 | 0.047747455 | 0.493001057 | 1.355073 | up | lncRNA |
| ENST00000447028 | AP003774.1 | 0.264441466 | 0.827574498 | 1.1399619 | up | lncRNA |
| ENST00000447058 | AC005075.5 | 0.177882972 | 0.743651912 | 1.6020395 | up | lncRNA |
| ENST00000447139 | AC046143.3 | 0.952712244 | 0.998875101 | 1.0192023 | up | lncRNA |
| ENST00000447147 | LINC00426 | 0.682056325 | 0.977676223 | 1.0526169 | up | lncRNA |
| ENST00000447148 | RP11-202I11.2 | 0.157198125 | 0.713732203 | 1.3009118 | up | lncRNA |
| ENST00000447149 | RP1-90L6.3 | 0.751748248 | 0.98644685 | 1.0471127 | up | lncRNA |
| ENST00000447181 | OSBPL10-AS1 | 0.765928873 | 0.989189555 | 1.1103696 | up | lncRNA |
| ENST00000447194 | AC006196.1 | 0.156390644 | 0.711867593 | 1.2068519 | up | lncRNA |
| ENST00000447297 | RP11-428L9.1 | 0.008385359 | 0.244110885 | 1.4436636 | up | lncRNA |
| ENST00000447307 | AC058791.1 | 0.110089152 | 0.645424639 | 1.5371307 | up | lncRNA |
| ENST00000447329 | RP4-794H19.4 | 0.08955294 | 0.603136158 | 1.2853251 | up | lncRNA |
| ENST00000447336 | RP11-645N11.3 | 0.625732371 | 0.961407878 | 1.2102455 | up | lncRNA |
| ENST00000447343 | RP4-583P15.10 | 0.021841057 | 0.361132318 | 1.3808091 | up | lncRNA |
| ENST00000447344 | RP11-108L7.4 | 0.521889986 | 0.932117592 | 1.0768583 | up | lncRNA |
| ENST00000447396 | RP1-302D9.1 | 0.375531143 | 0.879312727 | 1.1999909 | up | lncRNA |
| ENST00000447412 | RP11-291L22.3 | 0.953926445 | 0.998875101 | 1.0087338 | up | lncRNA |
| ENST00000447478 | RP4-534N18.2 | 0.507057557 | 0.925438288 | 1.0914606 | up | lncRNA |
| ENST00000447488 | AC073254.1 | 0.475936641 | 0.917441334 | 1.0661757 | up | lncRNA |
| ENST00000447524 | XXyac-YM21GA2.4 | 0.807906942 | 0.992578294 | 1.0662718 | up | lncRNA |
| ENST00000447538 | HOXD-AS1 | 0.927885871 | 0.998875101 | 1.0113222 | up | lncRNA |
| ENST00000447563 | AC004863.6 | 0.440056925 | 0.904111922 | 1.1893129 | up | lncRNA |
| ENST00000447577 | RP4-717I23.3 | 0.991847309 | 0.999156445 | 1.0022566 | up | lncRNA |
| ENST00000447600 | RP4-635E18.6 | 0.445972707 | 0.90519468 | 1.1429346 | up | lncRNA |
| ENST00000447651 | AC092198.1 | 0.009401765 | 0.253701351 | 4.2560266 | up | lncRNA |
| ENST00000447686 | RP11-315I20.1 | 0.594912082 | 0.951072365 | 1.0604384 | up | lncRNA |
| ENST00000447687 | CD27-AS1 | 0.563805689 | 0.943393901 | 1.177051 | up | lncRNA |
| ENST00000447702 | RP1-81D8.3 | 0.801982092 | 0.992578294 | 1.0121919 | up | lncRNA |
| ENST00000447775 | NCBP2-AS1 | 0.002183215 | 0.142436072 | 1.2787122 | up | lncRNA |
| ENST00000447835 | AC012462.2 | 0.002257537 | 0.143044381 | 2.1942304 | up | lncRNA |
| ENST00000447848 | RP11-394G3.2 | 0.269557319 | 0.830995087 | 1.1964039 | up | lncRNA |
| ENST00000447864 | AP002856.6 | 0.338580565 | 0.86050695 | 1.1696297 | up | lncRNA |
| ENST00000447867 | RP11-334A14.5 | 0.766238347 | 0.989189555 | 1.0345323 | up | lncRNA |
| ENST00000447880 | AC016999.2 | 0.023510139 | 0.370381471 | 1.556965 | up | lncRNA |
| ENST00000447883 | RP11-277L2.3 | 0.004304545 | 0.187425199 | 1.9075116 | up | lncRNA |
| ENST00000447898 | AP000525.8 | 0.996233172 | 0.999156445 | 1.0047237 | up | lncRNA |
| ENST00000447933 | AC073871.2 | 0.091846618 | 0.609226977 | 1.1841492 | up | lncRNA |
| ENST00000447937 | TTTY14 | 0.550600598 | 0.942167708 | 1.056843 | up | lncRNA |
| ENST00000448015 | RP11-460I13.2 | 0.568571241 | 0.943923733 | 1.0469554 | up | lncRNA |
| ENST00000448056 | AC004540.5 | 0.281550583 | 0.832297359 | 1.1196852 | up | lncRNA |
| ENST00000448195 | MAGI2-AS3 | 0.742329676 | 0.985094857 | 1.0812308 | up | lncRNA |
| ENST00000448204 | AC114752.1 | 0.276026921 | 0.832297359 | 1.084609 | up | lncRNA |
| ENST00000448211 | RP11-328M4.3 | 0.283868975 | 0.834126377 | 1.1600558 | up | lncRNA |
| ENST00000448256 | RP11-13J8.1 | 0.460252955 | 0.91221792 | 1.0628076 | up | lncRNA |
| ENST00000448318 | RP1-272J12.1 | 0.85107065 | 0.995900317 | 1.0327299 | up | lncRNA |
| ENST00000448341 | RP11-231P20.2 | 0.153026106 | 0.709376834 | 1.3481324 | up | lncRNA |
| ENST00000448371 | RP11-157P1.4 | 0.619673695 | 0.960029748 | 1.0664565 | up | lncRNA |
| ENST00000448374 | RP5-907D15.2 | 0.121642758 | 0.672928817 | 1.1040647 | up | lncRNA |
| ENST00000448379 | AC113608.1 | 0.538606905 | 0.936769998 | 1.1181293 | up | lncRNA |
| ENST00000448465 | LINC00476 | 0.761411517 | 0.988045734 | 1.048311 | up | lncRNA |
| ENST00000448475 | RP11-403N16.2 | 0.031528469 | 0.418199276 | 1.2757239 | up | lncRNA |
| ENST00000448513 | RP11-395B7.4 | 0.701259959 | 0.980170937 | 1.0698278 | up | lncRNA |
| ENST00000448559 | TDRG1 | 0.73586759 | 0.984025164 | 1.0283975 | up | lncRNA |
| ENST00000448605 | RP5-857K21.4 | 0.673761609 | 0.975272478 | 1.1011078 | up | lncRNA |
| ENST00000448613 | SRGAP2-IT1 | 0.661332012 | 0.971704562 | 1.0559691 | up | lncRNA |
| ENST00000448636 | MAGI2-AS3 | 0.556751259 | 0.943023022 | 1.2880322 | up | lncRNA |
| ENST00000448671 | RP11-282I1.1 | 0.161748249 | 0.716706043 | 1.306608 | up | lncRNA |
| ENST00000448680 | RP4-666F24.3 | 0.923581477 | 0.998875101 | 1.0526173 | up | lncRNA |
| ENST00000448686 | RP1-102G20.5 | 0.05586906 | 0.521270634 | 1.5747242 | up | lncRNA |
| ENST00000448698 | AC078942.1 | 0.860979442 | 0.996142678 | 1.0131853 | up | lncRNA |
| ENST00000448748 | LINC00349 | 0.422923516 | 0.896175742 | 1.1272602 | up | lncRNA |
| ENST00000448791 | RP1-50O24.6 | 0.957454498 | 0.998875101 | 1.0073681 | up | lncRNA |
| ENST00000448840 | AL132709.1 | 0.003932821 | 0.179554598 | 1.25597 | up | lncRNA |
| ENST00000448844 | BX004987.5 | 0.372390041 | 0.878574831 | 1.2334318 | up | lncRNA |
| ENST00000448869 | RP11-284F21.7 | 0.336674393 | 0.859588936 | 1.0910483 | up | lncRNA |
| ENST00000448887 | FREM2-AS1 | 0.605815658 | 0.956038292 | 1.2143792 | up | lncRNA |
| ENST00000448897 | RP11-149I23.3 | 0.982491816 | 0.998875101 | 1.0038564 | up | lncRNA |
| ENST00000448923 | RP11-96L14.7 | 0.476113488 | 0.917441334 | 1.1021863 | up | lncRNA |
| ENST00000448927 | AP001062.7 | 0.176025091 | 0.739552722 | 1.400487 | up | lncRNA |
| ENST00000448942 | RP11-356I2.4 | 0.113160597 | 0.653534309 | 1.4296922 | up | lncRNA |
| ENST00000448958 | AP006222.2 | 0.977852054 | 0.998875101 | 1.0023371 | up | lncRNA |
| ENST00000448978 | LENG8-AS1 | 0.197177201 | 0.763390122 | 1.6555955 | up | lncRNA |
| ENST00000448987 | AC068831.3 | 0.726534133 | 0.98251396 | 1.0596445 | up | lncRNA |
| ENST00000449009 | ATP8B5P | 0.484066548 | 0.919439531 | 1.0753735 | up | lncRNA |
| ENST00000449012 | RP4-781K5.4 | 0.41810876 | 0.893758851 | 1.1361126 | up | lncRNA |
| ENST00000449063 | RP11-70C1.3 | 0.917199815 | 0.998875101 | 1.0185731 | up | lncRNA |
| ENST00000449066 | AC140481.1 | 0.691111108 | 0.978695455 | 1.069604 | up | lncRNA |
| ENST00000449106 | TMEM212-AS1 | 0.129056621 | 0.682265946 | 1.132299 | up | lncRNA |
| ENST00000449143 | RP11-239H6.2 | 0.400944988 | 0.88878591 | 1.1353439 | up | lncRNA |
| ENST00000449151 | PDZK1P1 | 0.467989163 | 0.914494873 | 1.1454283 | up | lncRNA |
| ENST00000449158 | GRM7-AS3 | 0.074118637 | 0.565049692 | 1.1421138 | up | lncRNA |
| ENST00000449170 | AC018865.8 | 0.454899015 | 0.910032467 | 1.1098837 | up | lncRNA |
| ENST00000449178 | PCBP1-AS1 | 0.373682036 | 0.878762015 | 1.2411771 | up | lncRNA |
| ENST00000449235 | RP11-151D14.1 | 0.280995049 | 0.832297359 | 1.1358361 | up | lncRNA |
| ENST00000449242 | AC012307.2 | 0.135502242 | 0.693269931 | 1.101244 | up | lncRNA |
| ENST00000449391 | HS1BP3-IT1 | 0.332624718 | 0.857061033 | 1.2623088 | up | lncRNA |
| ENST00000449405 | AC113607.2 | 0.346406607 | 0.865832425 | 1.1230369 | up | lncRNA |
| ENST00000449419 | RP11-429G19.2 | 0.201766659 | 0.768512499 | 1.0787663 | up | lncRNA |
| ENST00000449436 | RP11-383C5.7 | 0.440555755 | 0.904456709 | 1.4058283 | up | lncRNA |
| ENST00000449453 | RP11-199F6.4 | 0.003091266 | 0.162565512 | 3.947762 | up | lncRNA |
| ENST00000449469 | RP4-564F22.2 | 0.103389126 | 0.632351488 | 1.3374955 | up | lncRNA |
| ENST00000449573 | AC005062.2 | 0.393279915 | 0.885711362 | 1.3516472 | up | lncRNA |
| ENST00000449579 | DLEU2 | 0.000365375 | 0.084119144 | 2.0977239 | up | lncRNA |
| ENST00000449585 | RP11-631M21.1 | 0.035747008 | 0.432946037 | 1.3413728 | up | lncRNA |
| ENST00000449689 | GTF2IRD2P1 | 0.029912156 | 0.41398193 | 1.1192803 | up | lncRNA |
| ENST00000449693 | RP11-383C5.3 | 0.03889925 | 0.451913909 | 1.3489712 | up | lncRNA |
| ENST00000449711 | AP000344.3 | 0.085831874 | 0.594526571 | 1.123095 | up | lncRNA |
| ENST00000449714 | AC144449.1 | 0.831211969 | 0.994074191 | 1.0754412 | up | lncRNA |
| ENST00000449741 | AC078937.4 | 0.914075658 | 0.998875101 | 1.016656 | up | lncRNA |
| ENST00000449772 | AC068535.3 | 0.211426747 | 0.779645856 | 1.4713707 | up | lncRNA |
| ENST00000449819 | AC093901.1 | 0.440842841 | 0.904456709 | 1.0796353 | up | lncRNA |
| ENST00000449841 | RP5-1069C8.1 | 0.602075774 | 0.954578533 | 1.0483336 | up | lncRNA |
| ENST00000449852 | RP11-126H7.3 | 0.952788245 | 0.998875101 | 1.0094632 | up | lncRNA |
| ENST00000449881 | UGGT2-IT1 | 0.990010867 | 0.999156445 | 1.0032321 | up | lncRNA |
| ENST00000449915 | RP11-480I12.5 | 0.033096939 | 0.427345115 | 1.1947727 | up | lncRNA |
| ENST00000449923 | BACH1-AS1 | 0.3014523 | 0.842735486 | 1.2116995 | up | lncRNA |
| ENST00000449941 | RP13-455A7.1 | 0.863536214 | 0.996259786 | 1.0163191 | up | lncRNA |
| ENST00000449954 | AL078621.3 | 0.0015156 | 0.135129436 | 2.8940352 | up | lncRNA |
| ENST00000449955 | AC093734.13 | 0.299113148 | 0.842046751 | 1.1423477 | up | lncRNA |
| ENST00000449984 | RP11-298J20.3 | 0.311899579 | 0.849269161 | 1.4853194 | up | lncRNA |
| ENST00000450026 | AFG3L1P | 0.874000119 | 0.997909851 | 1.01071 | up | lncRNA |
| ENST00000450028 | RPL13AP17 | 0.143038962 | 0.69945173 | 1.2439262 | up | lncRNA |
| ENST00000450072 | AC017099.3 | 0.560610049 | 0.943393901 | 1.1258589 | up | lncRNA |
| ENST00000450108 | RP11-242O24.5 | 0.854508136 | 0.996142678 | 1.026538 | up | lncRNA |
| ENST00000450112 | MINOS1P1 | 0.466129689 | 0.913821992 | 1.1235566 | up | lncRNA |
| ENST00000450187 | RP11-25J23.3 | 0.607333351 | 0.956065104 | 1.0952603 | up | lncRNA |
| ENST00000450206 | RP11-432J24.3 | 0.935228877 | 0.998875101 | 1.0108242 | up | lncRNA |
| ENST00000450226 | RP11-261C10.2 | 0.330736851 | 0.855705627 | 1.1029523 | up | lncRNA |
| ENST00000450264 | LINC00572 | 0.956217789 | 0.998875101 | 1.0072495 | up | lncRNA |
| ENST00000450277 | CCDC144B | 0.857007779 | 0.996142678 | 1.0569524 | up | lncRNA |
| ENST00000450299 | LINC00238 | 0.296004463 | 0.839987155 | 1.1972382 | up | lncRNA |
| ENST00000450360 | BMS1P2 | 0.562775151 | 0.943393901 | 1.1135327 | up | lncRNA |
| ENST00000450426 | AC083884.8 | 0.721330695 | 0.98251396 | 1.104403 | up | lncRNA |
| ENST00000450436 | SEPT7P2 | 0.879871716 | 0.998682284 | 1.0378776 | up | lncRNA |
| ENST00000450445 | RP11-62F24.2 | 0.55054244 | 0.942167708 | 1.1113645 | up | lncRNA |
| ENST00000450467 | AC097517.2 | 0.419754233 | 0.894750301 | 1.4069838 | up | lncRNA |
| ENST00000450483 | RP1-86D1.3 | 0.002356001 | 0.145121592 | 2.012566 | up | lncRNA |
| ENST00000450520 | RP11-216M21.7 | 0.801733503 | 0.992578294 | 1.0331952 | up | lncRNA |
| ENST00000450527 | RP11-342D14.1 | 0.044868479 | 0.48047812 | 1.1242646 | up | lncRNA |
| ENST00000450535 | ZNFX1-AS1 | 0.70181247 | 0.980170937 | 1.0667879 | up | lncRNA |
| ENST00000450540 | AC007255.8 | 0.04299398 | 0.471750461 | 1.1808227 | up | lncRNA |
| ENST00000450550 | AC073283.4 | 0.603071209 | 0.955012467 | 1.096455 | up | lncRNA |
| ENST00000450581 | RP11-14C22.3 | 0.083084299 | 0.585414993 | 1.579371 | up | lncRNA |
| ENST00000450591 | TTTY2B | 0.467015772 | 0.914238486 | 1.070367 | up | lncRNA |
| ENST00000450618 | RP3-340B19.3 | 0.170652945 | 0.73176309 | 1.2671156 | up | lncRNA |
| ENST00000450645 | RP11-498J9.4 | 0.281672585 | 0.832297359 | 1.2417126 | up | lncRNA |
| ENST00000450671 | RP3-475N16.1 | 0.180219539 | 0.745729299 | 1.1018204 | up | lncRNA |
| ENST00000450686 | RP11-325F22.3 | 0.020320887 | 0.355231872 | 1.5256687 | up | lncRNA |
| ENST00000450720 | ALMS1P | 0.206499491 | 0.773204016 | 1.2919226 | up | lncRNA |
| ENST00000450776 | AP000343.2 | 0.99211949 | 0.999156445 | 1.0016125 | up | lncRNA |
| ENST00000450783 | RP11-295G20.2 | 0.218182479 | 0.785656962 | 1.1416832 | up | lncRNA |
| ENST00000450834 | ZEB1-AS1 | 0.738031725 | 0.984316636 | 1.0562098 | up | lncRNA |
| ENST00000450916 | AC096559.1 | 0.970964689 | 0.998875101 | 1.0059985 | up | lncRNA |
| ENST00000450920 | SRGAP3-AS2 | 0.278092894 | 0.832297359 | 1.1511741 | up | lncRNA |
| ENST00000450963 | CTA-211A9.5 | 0.500300282 | 0.922148899 | 1.1149904 | up | lncRNA |
| ENST00000450980 | RP11-672F9.1 | 0.017525225 | 0.33447079 | 1.4879413 | up | lncRNA |
| ENST00000450990 | AC093415.2 | 0.588285228 | 0.950589384 | 1.1192904 | up | lncRNA |
| ENST00000450998 | RP11-154D6.1 | 0.35061581 | 0.867400431 | 1.4270843 | up | lncRNA |
| ENST00000451054 | RP11-465B22.3 | 0.308990002 | 0.847758462 | 1.4193555 | up | lncRNA |
| ENST00000451090 | RP11-145M4.2 | 0.762775333 | 0.988817776 | 1.1079983 | up | lncRNA |
| ENST00000451108 | RP11-410K21.2 | 0.200952135 | 0.767810338 | 1.2715618 | up | lncRNA |
| ENST00000451152 | RP11-63P12.6 | 0.12612018 | 0.678900542 | 1.4143336 | up | lncRNA |
| ENST00000451180 | RP1-76B20.11 | 0.544455509 | 0.939697392 | 1.0700837 | up | lncRNA |
| ENST00000451217 | RP1-150O5.3 | 0.441075623 | 0.904580493 | 1.2279304 | up | lncRNA |
| ENST00000451220 | RP11-14I4.3 | 0.300117931 | 0.8422517 | 1.1480034 | up | lncRNA |
| ENST00000451225 | RP11-414K1.3 | 0.661596716 | 0.971704562 | 1.0971078 | up | lncRNA |
| ENST00000451257 | KB-1183D5.11 | 0.941891971 | 0.998875101 | 1.0205485 | up | lncRNA |
| ENST00000451282 | RP11-111F5.5 | 0.911946969 | 0.998875101 | 1.0260919 | up | lncRNA |
| ENST00000451285 | RP11-401E14.2 | 0.020489291 | 0.356048364 | 1.2306266 | up | lncRNA |
| ENST00000451302 | RP4-717I23.3 | 0.460726154 | 0.91221792 | 1.3214476 | up | lncRNA |
| ENST00000451362 | RP11-139I14.2 | 0.32790163 | 0.854556781 | 1.267421 | up | lncRNA |
| ENST00000451364 | RP11-429G19.2 | 0.413659488 | 0.890927603 | 1.0990706 | up | lncRNA |
| ENST00000451381 | RP5-905H7.3 | 0.005516719 | 0.205651833 | 1.1350134 | up | lncRNA |
| ENST00000451392 | LINC00607 | 0.499880306 | 0.922144236 | 1.0603923 | up | lncRNA |
| ENST00000451395 | RP11-73B2.6 | 0.922722692 | 0.998875101 | 1.0134939 | up | lncRNA |
| ENST00000451400 | AC013402.2 | 0.068780203 | 0.55301595 | 1.2014705 | up | lncRNA |
| ENST00000451439 | RP11-297H3.4 | 0.155103123 | 0.711341825 | 1.7232117 | up | lncRNA |
| ENST00000451449 | RP11-123K19.1 | 0.678611041 | 0.976096237 | 1.0568469 | up | lncRNA |
| ENST00000451478 | AC074093.1 | 0.104135708 | 0.634228154 | 1.3444239 | up | lncRNA |
| ENST00000451482 | RP11-231P20.2 | 0.240711773 | 0.809272444 | 1.6474459 | up | lncRNA |
| ENST00000451486 | CTA-292E10.6 | 0.159811908 | 0.714806047 | 1.5613973 | up | lncRNA |
| ENST00000451488 | RP11-195E11.3 | 0.282687692 | 0.832745023 | 1.2945964 | up | lncRNA |
| ENST00000451511 | AC011752.1 | 0.644110026 | 0.967175883 | 1.0427599 | up | lncRNA |
| ENST00000451530 | RP11-342D11.2 | 0.314616353 | 0.851147568 | 1.1077541 | up | lncRNA |
| ENST00000451575 | RP11-499O7.7 | 0.877579722 | 0.998682284 | 1.025606 | up | lncRNA |
| ENST00000451583 | RP11-622K12.1 | 0.583179129 | 0.949379205 | 1.0531243 | up | lncRNA |
| ENST00000451595 | RP11-244N9.6 | 0.003783995 | 0.17862549 | 1.2778729 | up | lncRNA |
| ENST00000451598 | RP11-219C24.9 | 0.366048875 | 0.874818743 | 1.3041168 | up | lncRNA |
| ENST00000451601 | RP11-809C18.3 | 0.026187105 | 0.383507483 | 2.9748664 | up | lncRNA |
| ENST00000451607 | GAS5 | 0.215768023 | 0.783396803 | 1.1026323 | up | lncRNA |
| ENST00000451622 | AC007099.1 | 0.19508974 | 0.761263718 | 1.1991238 | up | lncRNA |
| ENST00000451644 | AC096559.1 | 0.100026727 | 0.627440378 | 1.4690289 | up | lncRNA |
| ENST00000451648 | RP11-93B14.5 | 0.374953974 | 0.879312727 | 1.0998255 | up | lncRNA |
| ENST00000451656 | RP11-549L6.3 | 0.471038317 | 0.915538705 | 1.1050442 | up | lncRNA |
| ENST00000451690 | RP11-139E24.1 | 0.634881794 | 0.965031085 | 1.0541463 | up | lncRNA |
| ENST00000451691 | CTD-2666L21.1 | 0.785703391 | 0.99125421 | 1.0808984 | up | lncRNA |
| ENST00000451704 | AC015818.3 | 0.458074107 | 0.912197894 | 1.1262996 | up | lncRNA |
| ENST00000451749 | AC073409.1 | 0.097949046 | 0.623398984 | 1.5897251 | up | lncRNA |
| ENST00000451760 | RP11-96C23.10 | 0.142492226 | 0.698941469 | 1.2394918 | up | lncRNA |
| ENST00000451809 | MAGI2-AS3 | 0.251869511 | 0.817890149 | 1.0855657 | up | lncRNA |
| ENST00000451829 | RP11-444D13.1 | 0.133839749 | 0.689601422 | 1.1816855 | up | lncRNA |
| ENST00000451902 | RP11-240D10.2 | 0.258891161 | 0.824081814 | 1.546422 | up | lncRNA |
| ENST00000451940 | RP11-322M19.1 | 0.761287322 | 0.988045734 | 1.0380308 | up | lncRNA |
| ENST00000451953 | RP5-1059M17.1 | 0.883380622 | 0.998875101 | 1.0372067 | up | lncRNA |
| ENST00000452042 | RP13-131K19.2 | 0.917293988 | 0.998875101 | 1.0073388 | up | lncRNA |
| ENST00000452050 | AC002454.1 | 0.47555694 | 0.917441334 | 1.2004888 | up | lncRNA |
| ENST00000452051 | TM4SF19-AS1 | 0.829501338 | 0.994074191 | 1.0337886 | up | lncRNA |
| ENST00000452053 | ADAM6 | 0.7336336 | 0.983763148 | 1.1255104 | up | lncRNA |
| ENST00000452057 | AC007038.7 | 0.286191908 | 0.835918864 | 1.2601012 | up | lncRNA |
| ENST00000452110 | PI4KAP1 | 0.34147796 | 0.862529336 | 1.0836926 | up | lncRNA |
| ENST00000452120 | MEG3 | 0.898437147 | 0.998875101 | 1.0381007 | up | lncRNA |
| ENST00000452219 | AC009518.4 | 0.707388512 | 0.981594335 | 1.0334441 | up | lncRNA |
| ENST00000452222 | USP12-AS2 | 0.659049391 | 0.971704562 | 1.0595192 | up | lncRNA |
| ENST00000452229 | ZNRD1-AS1 | 0.56889041 | 0.943923733 | 1.1050989 | up | lncRNA |
| ENST00000452249 | AC003075.4 | 0.660141513 | 0.971704562 | 1.123785 | up | lncRNA |
| ENST00000452252 | RP11-319I23.3 | 0.972211052 | 0.998875101 | 1.0065471 | up | lncRNA |
| ENST00000452320 | MAGI2-AS3 | 0.638131528 | 0.96564589 | 1.1928159 | up | lncRNA |
| ENST00000452342 | RP11-644K8.1 | 0.014653247 | 0.306534193 | 1.6532644 | up | lncRNA |
| ENST00000452347 | RP4-717I23.3 | 0.67902492 | 0.976196099 | 1.0648343 | up | lncRNA |
| ENST00000452349 | MEG9 | 0.422681383 | 0.896120672 | 1.2231757 | up | lncRNA |
| ENST00000452356 | RP11-277L2.2 | 0.000338232 | 0.084119144 | 2.2588461 | up | lncRNA |
| ENST00000452391 | RP11-483F11.7 | 0.305586751 | 0.846407029 | 1.2288925 | up | lncRNA |
| ENST00000452397 | AC007098.1 | 0.660296793 | 0.971704562 | 1.1288542 | up | lncRNA |
| ENST00000452429 | MIAT | 0.58911367 | 0.950589384 | 1.0538474 | up | lncRNA |
| ENST00000452431 | PCBP1-AS1 | 0.60684465 | 0.956038292 | 1.0930399 | up | lncRNA |
| ENST00000452435 | PI4KAP1 | 0.985578124 | 0.998971647 | 1.0029896 | up | lncRNA |
| ENST00000452466 | RP5-1024G6.2 | 0.971712729 | 0.998875101 | 1.0025947 | up | lncRNA |
| ENST00000452505 | RP1-302D9.2 | 0.332951336 | 0.85731789 | 1.2558209 | up | lncRNA |
| ENST00000452514 | MEG3 | 0.952714193 | 0.998875101 | 1.0108958 | up | lncRNA |
| ENST00000452532 | RP13-188A5.1 | 0.475861238 | 0.917441334 | 1.0212367 | up | lncRNA |
| ENST00000452565 | GS1-124K5.4 | 0.961947827 | 0.998875101 | 1.0081019 | up | lncRNA |
| ENST00000452578 | RP11-20J15.3 | 0.992432648 | 0.999156445 | 1.0123621 | up | lncRNA |
| ENST00000452584 | TTTY14 | 0.097665849 | 0.622701142 | 1.2758586 | up | lncRNA |
| ENST00000452591 | RP11-122K13.7 | 0.647376941 | 0.969646614 | 1.1009758 | up | lncRNA |
| ENST00000452599 | RP11-383G10.5 | 0.245108635 | 0.811577674 | 1.1438337 | up | lncRNA |
| ENST00000452600 | AC018804.7 | 0.292776415 | 0.837871806 | 1.0360685 | up | lncRNA |
| ENST00000452657 | RP11-655G22.1 | 0.331743102 | 0.856326799 | 1.1749988 | up | lncRNA |
| ENST00000452675 | RP11-367G18.1 | 0.611294442 | 0.957448851 | 1.1966651 | up | lncRNA |
| ENST00000452700 | AC003986.5 | 0.17308047 | 0.734926304 | 1.1369867 | up | lncRNA |
| ENST00000452716 | AC023115.3 | 0.000111528 | 0.048504344 | 9.3072492 | up | lncRNA |
| ENST00000452741 | AC002117.1 | 0.660215253 | 0.971704562 | 1.0402593 | up | lncRNA |
| ENST00000452766 | RP11-488L4.1 | 0.878359908 | 0.998682284 | 1.0435054 | up | lncRNA |
| ENST00000452785 | RP4-533D7.5 | 0.033804148 | 0.42757611 | 1.6720482 | up | lncRNA |
| ENST00000452788 | NHS-AS1 | 0.809128792 | 0.992578294 | 1.0278961 | up | lncRNA |
| ENST00000452826 | RP11-111F5.5 | 0.60866556 | 0.956293969 | 1.3776198 | up | lncRNA |
| ENST00000452842 | RP13-379L11.1 | 0.634669608 | 0.964937767 | 1.2053369 | up | lncRNA |
| ENST00000452844 | SDHAP2 | 0.882409707 | 0.998875101 | 1.0418917 | up | lncRNA |
| ENST00000452846 | RP4-639F20.1 | 0.017346235 | 0.332825087 | 1.1196761 | up | lncRNA |
| ENST00000452867 | RP11-21M7.1 | 0.80107051 | 0.992578294 | 1.089873 | up | lncRNA |
| ENST00000452883 | RP11-458D21.1 | 0.156811913 | 0.712655026 | 1.342832 | up | lncRNA |
| ENST00000452923 | RP11-397D12.4 | 0.053224928 | 0.515925762 | 1.1648885 | up | lncRNA |
| ENST00000452942 | RP11-429G19.2 | 0.103584384 | 0.632846721 | 1.3935121 | up | lncRNA |
| ENST00000453011 | RP11-421L21.3 | 0.689110163 | 0.97861545 | 1.1318776 | up | lncRNA |
| ENST00000453039 | AC007383.4 | 0.554804918 | 0.943023022 | 1.0761417 | up | lncRNA |
| ENST00000453087 | AC008154.5 | 0.13017348 | 0.683290083 | 1.2030899 | up | lncRNA |
| ENST00000453111 | RP11-25K21.1 | 0.622546173 | 0.960457826 | 1.1167929 | up | lncRNA |
| ENST00000453121 | NEGR1-IT1 | 0.365841414 | 0.874818743 | 1.3742126 | up | lncRNA |
| ENST00000453136 | RP11-263K19.4 | 0.000444415 | 0.088586663 | 2.0999803 | up | lncRNA |
| ENST00000453194 | MYH16 | 0.787563201 | 0.991420185 | 1.0471969 | up | lncRNA |
| ENST00000453206 | AC016751.3 | 0.548034968 | 0.940847434 | 1.1884873 | up | lncRNA |
| ENST00000453229 | RP11-230B22.1 | 0.954053782 | 0.998875101 | 1.0335853 | up | lncRNA |
| ENST00000453278 | RP11-807H17.1 | 0.445639313 | 0.90519468 | 1.2170822 | up | lncRNA |
| ENST00000453289 | AC074117.10 | 0.139987287 | 0.698568819 | 1.103382 | up | lncRNA |
| ENST00000453324 | SDHAP2 | 0.174873948 | 0.736657148 | 1.2171132 | up | lncRNA |
| ENST00000453339 | AC005703.2 | 0.049644303 | 0.503530065 | 1.0980258 | up | lncRNA |
| ENST00000453420 | AL035610.2 | 0.554645429 | 0.943023022 | 1.098207 | up | lncRNA |
| ENST00000453454 | AC024560.3 | 0.973738263 | 0.998875101 | 1.0032061 | up | lncRNA |
| ENST00000453478 | AC068491.1 | 0.967026698 | 0.998875101 | 1.0056862 | up | lncRNA |
| ENST00000453508 | RP13-228J13.5 | 0.963171195 | 0.998875101 | 1.0043037 | up | lncRNA |
| ENST00000453525 | ITPKB-AS1 | 0.00481653 | 0.192553858 | 1.9202746 | up | lncRNA |
| ENST00000453549 | MIS18A-AS1 | 0.036918627 | 0.43898603 | 1.2619904 | up | lncRNA |
| ENST00000453558 | HCG17 | 0.318852869 | 0.851223284 | 1.0500595 | up | lncRNA |
| ENST00000453569 | RP5-1051D14.1 | 0.48485705 | 0.919853038 | 1.1642263 | up | lncRNA |
| ENST00000453584 | DCUN1D2-AS2 | 0.665410062 | 0.972501549 | 1.0510063 | up | lncRNA |
| ENST00000453606 | AC016907.3 | 0.600405331 | 0.953841565 | 1.0427284 | up | lncRNA |
| ENST00000453677 | RP11-325F22.4 | 0.017174849 | 0.330420153 | 2.159394 | up | lncRNA |
| ENST00000453698 | SNHG11 | 0.488876008 | 0.920777644 | 1.1182678 | up | lncRNA |
| ENST00000453706 | AC068489.1 | 0.732720002 | 0.983763148 | 1.1419659 | up | lncRNA |
| ENST00000453714 | AC012370.3 | 0.344647335 | 0.864600342 | 1.4136196 | up | lncRNA |
| ENST00000453722 | LINC00511 | 0.898383372 | 0.998875101 | 1.0155049 | up | lncRNA |
| ENST00000453743 | CTA-85E5.10 | 0.782730508 | 0.99121281 | 1.049255 | up | lncRNA |
| ENST00000453783 | GGT3P | 0.294027465 | 0.838742488 | 1.1577356 | up | lncRNA |
| ENST00000453798 | AC073094.4 | 0.815164421 | 0.99257857 | 1.1078401 | up | lncRNA |
| ENST00000453802 | AP000233.2 | 0.382183732 | 0.881294633 | 1.3281744 | up | lncRNA |
| ENST00000453837 | RP11-84A19.2 | 0.338462397 | 0.86050695 | 1.1774005 | up | lncRNA |
| ENST00000453880 | AC107070.1 | 0.036118348 | 0.435028735 | 1.2813538 | up | lncRNA |
| ENST00000453889 | RP11-94M14.2 | 0.601893678 | 0.954578533 | 1.1055919 | up | lncRNA |
| ENST00000453910 | LINC00478 | 0.90043314 | 0.998875101 | 1.028501 | up | lncRNA |
| ENST00000453935 | RP4-669L17.4 | 0.069659396 | 0.55572632 | 1.8116344 | up | lncRNA |
| ENST00000453936 | AC016722.3 | 0.266392062 | 0.828881561 | 1.194059 | up | lncRNA |
| ENST00000453946 | RP11-327I22.4 | 0.547799993 | 0.940847434 | 1.0762125 | up | lncRNA |
| ENST00000453951 | AC104135.4 | 0.53080542 | 0.934908142 | 1.2090428 | up | lncRNA |
| ENST00000453955 | TTTY19 | 0.524446039 | 0.932117592 | 1.0529214 | up | lncRNA |
| ENST00000453968 | AC002115.5 | 0.105344789 | 0.635942989 | 1.3255241 | up | lncRNA |
| ENST00000453998 | RP11-399F4.4 | 0.00058559 | 0.094750704 | 1.1862796 | up | lncRNA |
| ENST00000454005 | RASA3-IT1 | 0.149794337 | 0.705330813 | 1.4330277 | up | lncRNA |
| ENST00000454058 | AC009950.2 | 0.745068649 | 0.985094857 | 1.041083 | up | lncRNA |
| ENST00000454082 | RPL31P11 | 0.884307739 | 0.998875101 | 1.005918 | up | lncRNA |
| ENST00000454104 | RP11-84A14.5 | 0.329608946 | 0.85540421 | 1.2116814 | up | lncRNA |
| ENST00000454137 | AC006509.6 | 0.721970193 | 0.98251396 | 1.0799672 | up | lncRNA |
| ENST00000454203 | AC010894.5 | 0.030152229 | 0.414324281 | 1.3260676 | up | lncRNA |
| ENST00000454205 | ITCH-AS1 | 0.369739566 | 0.876569032 | 1.1248405 | up | lncRNA |
| ENST00000454269 | HCG18 | 0.224921736 | 0.794551181 | 1.0787503 | up | lncRNA |
| ENST00000454321 | RP11-536K7.3 | 0.119462861 | 0.667912342 | 1.4226901 | up | lncRNA |
| ENST00000454388 | RP11-493K23.4 | 0.480130914 | 0.918546036 | 1.142402 | up | lncRNA |
| ENST00000454408 | RP11-65J3.3 | 0.642760173 | 0.967175883 | 1.102 | up | lncRNA |
| ENST00000454426 | HIST1H2APS2 | 0.400100308 | 0.888530576 | 1.0866626 | up | lncRNA |
| ENST00000454439 | RP1-102D24.5 | 0.881946767 | 0.998875101 | 1.0208944 | up | lncRNA |
| ENST00000454444 | AC007879.4 | 0.671443517 | 0.974377541 | 1.0981434 | up | lncRNA |
| ENST00000454484 | RP11-35J23.1 | 0.298412982 | 0.841479361 | 1.0823823 | up | lncRNA |
| ENST00000454490 | ANKRD36BP2 | 0.279320439 | 0.832297359 | 1.3800014 | up | lncRNA |
| ENST00000454503 | AC010976.2 | 0.973652809 | 0.998875101 | 1.0119405 | up | lncRNA |
| ENST00000454537 | AC012501.2 | 0.726799898 | 0.98251396 | 1.0612028 | up | lncRNA |
| ENST00000454551 | RP13-210D15.4 | 0.037256215 | 0.440489158 | 1.5187685 | up | lncRNA |
| ENST00000454562 | RP4-758J18.10 | 0.717695145 | 0.98251396 | 1.0517294 | up | lncRNA |
| ENST00000454588 | RP1-45I4.3 | 0.3926549 | 0.885711362 | 1.1137733 | up | lncRNA |
| ENST00000454625 | GS1-421I3.2 | 0.158257979 | 0.714689048 | 1.3190103 | up | lncRNA |
| ENST00000454631 | RP11-295G20.2 | 0.017130113 | 0.330420153 | 1.1308545 | up | lncRNA |
| ENST00000454645 | RP11-204M4.2 | 0.90679334 | 0.998875101 | 1.0256134 | up | lncRNA |
| ENST00000454647 | LINC00305 | 0.205920176 | 0.772818733 | 1.1690437 | up | lncRNA |
| ENST00000454672 | RP11-463P17.1 | 0.507129999 | 0.925438288 | 1.2828483 | up | lncRNA |
| ENST00000454719 | RP13-580B18.1 | 0.847155165 | 0.995583664 | 1.0719979 | up | lncRNA |
| ENST00000454768 | AC129778.2 | 0.469963918 | 0.91468974 | 1.1675001 | up | lncRNA |
| ENST00000454812 | RP11-328M4.2 | 0.334333624 | 0.85798034 | 1.2884431 | up | lncRNA |
| ENST00000454832 | SIK3-IT1 | 0.166602852 | 0.722163735 | 1.1456039 | up | lncRNA |
| ENST00000454833 | TMEM191A | 0.723657883 | 0.98251396 | 1.1372345 | up | lncRNA |
| ENST00000454857 | CASC2 | 0.378819731 | 0.88011421 | 1.1323513 | up | lncRNA |
| ENST00000454892 | HBBP1 | 0.049630671 | 0.503530065 | 1.6577852 | up | lncRNA |
| ENST00000454897 | AC073130.1 | 0.286712423 | 0.836701318 | 1.4349729 | up | lncRNA |
| ENST00000454977 | TSSC1-IT1 | 0.455999035 | 0.910972327 | 1.0960984 | up | lncRNA |
| ENST00000454980 | AP000695.4 | 8.68789E-05 | 0.042996088 | 1.9500431 | up | lncRNA |
| ENST00000455005 | RP5-1120P11.3 | 0.427072683 | 0.897780553 | 1.3121786 | up | lncRNA |
| ENST00000455071 | RP11-30P6.6 | 0.825834992 | 0.993856674 | 1.0481339 | up | lncRNA |
| ENST00000455074 | RP11-483H20.4 | 0.34265052 | 0.863514929 | 1.1761076 | up | lncRNA |
| ENST00000455081 | AC013472.3 | 0.641559369 | 0.967175883 | 1.2717078 | up | lncRNA |
| ENST00000455105 | RP11-763B22.4 | 0.158020246 | 0.714689048 | 1.1878313 | up | lncRNA |
| ENST00000455121 | AC105053.4 | 0.501248775 | 0.922721625 | 1.0698823 | up | lncRNA |
| ENST00000455126 | HTR2A-AS1 | 0.13290101 | 0.688794697 | 1.1919012 | up | lncRNA |
| ENST00000455128 | AC012507.3 | 0.512245392 | 0.928625804 | 1.0595929 | up | lncRNA |
| ENST00000455131 | LINC00152 | 0.187695191 | 0.753977992 | 1.5529568 | up | lncRNA |
| ENST00000455154 | AC108448.3 | 0.231170893 | 0.79852974 | 1.2752351 | up | lncRNA |
| ENST00000455257 | RP11-525G13.2 | 0.517677908 | 0.930458778 | 1.0982004 | up | lncRNA |
| ENST00000455286 | MEG3 | 0.051151163 | 0.50923846 | 1.0940845 | up | lncRNA |
| ENST00000455295 | RP11-277L2.2 | 0.000863982 | 0.104758688 | 2.6466696 | up | lncRNA |
| ENST00000455307 | KCNMB2-IT1 | 0.174298307 | 0.736164062 | 1.5682261 | up | lncRNA |
| ENST00000455309 | AC017002.1 | 0.629658832 | 0.96389872 | 1.147926 | up | lncRNA |
| ENST00000455442 | AC011625.1 | 0.383901966 | 0.882562871 | 1.3021012 | up | lncRNA |
| ENST00000455460 | AC004562.1 | 0.821388462 | 0.993333549 | 1.031526 | up | lncRNA |
| ENST00000455474 | RP4-717I23.3 | 0.792211832 | 0.991420185 | 1.0873362 | up | lncRNA |
| ENST00000455503 | RP11-126K1.6 | 0.834102232 | 0.99450938 | 1.0281426 | up | lncRNA |
| ENST00000455507 | OSTCP1 | 0.283667314 | 0.834126377 | 1.0591007 | up | lncRNA |
| ENST00000455530 | RP11-415D17.4 | 0.734142031 | 0.983763148 | 1.0891284 | up | lncRNA |
| ENST00000455557 | AC108676.1 | 0.299817811 | 0.8422517 | 1.1874683 | up | lncRNA |
| ENST00000455572 | BIRC6-AS1 | 0.062034026 | 0.534658422 | 1.1528559 | up | lncRNA |
| ENST00000455579 | AC010729.1 | 0.053757184 | 0.516622604 | 2.1930659 | up | lncRNA |
| ENST00000455598 | RP11-364P22.1 | 0.440604123 | 0.904456709 | 1.3931788 | up | lncRNA |
| ENST00000455607 | RP11-350J20.5 | 0.39079536 | 0.885711362 | 1.0919984 | up | lncRNA |
| ENST00000455629 | CCDC144B | 0.849055759 | 0.995876116 | 1.0177055 | up | lncRNA |
| ENST00000455671 | AC051649.16 | 0.197739499 | 0.763778725 | 1.1768949 | up | lncRNA |
| ENST00000455709 | AC005534.8 | 0.93188991 | 0.998875101 | 1.0064585 | up | lncRNA |
| ENST00000455777 | RP13-507I23.1 | 0.839900841 | 0.995583664 | 1.0794619 | up | lncRNA |
| ENST00000455857 | LINC00453 | 0.022003908 | 0.361714622 | 5.6578305 | up | lncRNA |
| ENST00000455896 | AC009955.8 | 0.381301098 | 0.88113876 | 1.2436413 | up | lncRNA |
| ENST00000455909 | FKBP9L | 0.714387175 | 0.98251396 | 1.0860318 | up | lncRNA |
| ENST00000455966 | RP1-224A6.3 | 0.008957306 | 0.249900233 | 1.5548241 | up | lncRNA |
| ENST00000455981 | RP11-344B5.2 | 0.404672173 | 0.889198786 | 1.0925234 | up | lncRNA |
| ENST00000455988 | AC007405.8 | 0.007570768 | 0.23118226 | 1.1957591 | up | lncRNA |
| ENST00000456027 | HERC2P3 | 0.972089397 | 0.998875101 | 1.0105955 | up | lncRNA |
| ENST00000456032 | RP11-127L21.1 | 0.964092272 | 0.998875101 | 1.0136089 | up | lncRNA |
| ENST00000456034 | RP11-144G6.10 | 0.534104946 | 0.935381352 | 1.2829721 | up | lncRNA |
| ENST00000456072 | L29074.3 | 0.463488667 | 0.913747281 | 1.0706778 | up | lncRNA |
| ENST00000456082 | RP11-478H13.1 | 0.304927647 | 0.845829453 | 1.3388328 | up | lncRNA |
| ENST00000456087 | STARD13-IT1 | 0.222326745 | 0.790839 | 1.3461318 | up | lncRNA |
| ENST00000456090 | SMCR2 | 0.0012194 | 0.12293504 | 1.2649175 | up | lncRNA |
| ENST00000456119 | AC013400.2 | 0.778910933 | 0.99121281 | 1.1183324 | up | lncRNA |
| ENST00000456161 | PCBP1-AS1 | 0.34999308 | 0.867400431 | 1.2991176 | up | lncRNA |
| ENST00000456176 | AC067945.4 | 0.535630309 | 0.936003031 | 1.1217428 | up | lncRNA |
| ENST00000456177 | RP11-77C3.3 | 0.19464394 | 0.760765035 | 1.2679426 | up | lncRNA |
| ENST00000456265 | RP5-1069C8.2 | 0.46493245 | 0.913821992 | 1.2026715 | up | lncRNA |
| ENST00000456312 | SBDSP1 | 0.788362193 | 0.991420185 | 1.0209878 | up | lncRNA |
| ENST00000456333 | MED14-AS1 | 0.267331692 | 0.829082126 | 1.2539744 | up | lncRNA |
| ENST00000456335 | RP11-528N21.1 | 0.860745716 | 0.996142678 | 1.0274705 | up | lncRNA |
| ENST00000456363 | AC093110.3 | 0.703784109 | 0.980759306 | 1.0495891 | up | lncRNA |
| ENST00000456375 | CFL1P1 | 0.867147659 | 0.996767702 | 1.019924 | up | lncRNA |
| ENST00000456382 | RP11-29H23.4 | 0.384404007 | 0.882848407 | 1.1133018 | up | lncRNA |
| ENST00000456391 | RP11-107I14.1 | 0.136228446 | 0.693269931 | 1.0796594 | up | lncRNA |
| ENST00000456414 | RP5-1086K13.1 | 0.001922486 | 0.141494996 | 2.3109025 | up | lncRNA |
| ENST00000456424 | RP1-249H1.2 | 0.818174025 | 0.992651327 | 1.0576053 | up | lncRNA |
| ENST00000456440 | RP3-448I9.2 | 0.13254001 | 0.688210645 | 1.7579032 | up | lncRNA |
| ENST00000456450 | AC010907.2 | 0.380617136 | 0.880821433 | 1.0491951 | up | lncRNA |
| ENST00000456469 | RP11-763B22.6 | 0.902347204 | 0.998875101 | 1.0097256 | up | lncRNA |
| ENST00000456514 | LINC00601 | 0.232204702 | 0.79956859 | 1.2024704 | up | lncRNA |
| ENST00000456519 | AC010884.1 | 0.527265037 | 0.934120209 | 1.1014735 | up | lncRNA |
| ENST00000456546 | RP11-342M3.1 | 0.058906065 | 0.52904871 | 1.3384642 | up | lncRNA |
| ENST00000456555 | AC011322.1 | 0.494485244 | 0.921717611 | 1.0590257 | up | lncRNA |
| ENST00000456582 | RP4-639F20.1 | 0.371134627 | 0.877177739 | 1.0763307 | up | lncRNA |
| ENST00000456623 | RP4-669L17.10 | 0.849662792 | 0.995876116 | 1.0188714 | up | lncRNA |
| ENST00000456685 | AC016683.6 | 0.102623508 | 0.632351488 | 1.332855 | up | lncRNA |
| ENST00000456687 | RP3-395M20.7 | 0.615030515 | 0.958509931 | 1.065366 | up | lncRNA |
| ENST00000456728 | RP11-45I20.1 | 0.199531093 | 0.766712248 | 1.4346625 | up | lncRNA |
| ENST00000456740 | CTA-747E2.10 | 0.265930824 | 0.828803299 | 1.1307751 | up | lncRNA |
| ENST00000456771 | RP11-540K16.2 | 0.778813875 | 0.99121281 | 1.0266859 | up | lncRNA |
| ENST00000456777 | AC003090.1 | 0.042610242 | 0.469333989 | 1.299795 | up | lncRNA |
| ENST00000456782 | DISC1-IT1 | 0.652481223 | 0.969927925 | 1.0803549 | up | lncRNA |
| ENST00000456813 | RP5-864K19.4 | 0.095661082 | 0.618714669 | 1.209325 | up | lncRNA |
| ENST00000456816 | RP11-513G11.1 | 0.288084599 | 0.836961572 | 1.2196804 | up | lncRNA |
| ENST00000456878 | RP11-575B7.3 | 0.158538654 | 0.714689048 | 1.4965298 | up | lncRNA |
| ENST00000456904 | AP001442.2 | 0.021315713 | 0.359097712 | 1.25486 | up | lncRNA |
| ENST00000456917 | MIR155HG | 0.009634747 | 0.254655403 | 2.781131 | up | lncRNA |
| ENST00000456932 | CSPG4P5 | 0.344743612 | 0.864690723 | 1.1168324 | up | lncRNA |
| ENST00000456938 | RP11-285G1.8 | 0.099033597 | 0.624500644 | 2.0343595 | up | lncRNA |
| ENST00000456944 | RP11-82L18.2 | 0.622247082 | 0.960457826 | 1.1427718 | up | lncRNA |
| ENST00000456949 | AC116614.1 | 0.283062063 | 0.833653196 | 4.8253791 | up | lncRNA |
| ENST00000456953 | RP4-564F22.2 | 0.266406008 | 0.828881561 | 1.1120783 | up | lncRNA |
| ENST00000456985 | RP11-525A16.4 | 0.423219508 | 0.896281227 | 1.0774796 | up | lncRNA |
| ENST00000456999 | AC013402.5 | 0.04150542 | 0.46501623 | 1.184918 | up | lncRNA |
| ENST00000457006 | RP11-304M2.2 | 0.026084984 | 0.383305135 | 1.1647478 | up | lncRNA |
| ENST00000457025 | RP4-717I23.3 | 0.834689276 | 0.994723946 | 1.0656322 | up | lncRNA |
| ENST00000457033 | AC073130.1 | 0.45731164 | 0.912197894 | 1.1256003 | up | lncRNA |
| ENST00000457043 | RP11-418J17.1 | 0.640100663 | 0.965955739 | 1.0264753 | up | lncRNA |
| ENST00000457097 | AC011247.3 | 0.811922187 | 0.99257857 | 1.0384722 | up | lncRNA |
| ENST00000457108 | AC009495.3 | 0.539784438 | 0.937437834 | 1.1599859 | up | lncRNA |
| ENST00000457113 | AC008746.3 | 0.263760128 | 0.827428492 | 1.0708647 | up | lncRNA |
| ENST00000457125 | RP11-706D8.3 | 0.396167097 | 0.886097826 | 1.1712199 | up | lncRNA |
| ENST00000457157 | AP000688.8 | 0.87546245 | 0.998245425 | 1.0164532 | up | lncRNA |
| ENST00000457233 | SDHAP2 | 0.549819967 | 0.942167708 | 1.0785801 | up | lncRNA |
| ENST00000457302 | RP11-229P13.20 | 0.159391738 | 0.714806047 | 1.191898 | up | lncRNA |
| ENST00000457319 | RP3-486I3.5 | 0.173053799 | 0.734926304 | 1.1644373 | up | lncRNA |
| ENST00000457321 | RP5-1011O1.3 | 0.438833065 | 0.903452621 | 1.0751586 | up | lncRNA |
| ENST00000457327 | TREML3P | 0.648334057 | 0.969810478 | 1.1546835 | up | lncRNA |
| ENST00000457328 | RP11-548B3.3 | 0.141534878 | 0.698568819 | 1.1293332 | up | lncRNA |
| ENST00000457371 | AC107070.1 | 0.621186504 | 0.960457826 | 1.0556884 | up | lncRNA |
| ENST00000457372 | GS1-278J22.1 | 0.127513232 | 0.680323382 | 1.5112855 | up | lncRNA |
| ENST00000457383 | RP11-70J12.1 | 0.221300296 | 0.790635384 | 1.1033989 | up | lncRNA |
| ENST00000457402 | RP11-470L19.2 | 0.031455341 | 0.418106757 | 1.490839 | up | lncRNA |
| ENST00000457427 | RP11-52J3.2 | 0.955713453 | 0.998875101 | 1.0159095 | up | lncRNA |
| ENST00000457443 | PRMT5-AS1 | 0.508906283 | 0.92652835 | 2.5966333 | up | lncRNA |
| ENST00000457449 | RP11-329B9.1 | 0.913514258 | 0.998875101 | 1.0158589 | up | lncRNA |
| ENST00000457465 | RP11-458D21.1 | 0.145836267 | 0.701026582 | 1.2938982 | up | lncRNA |
| ENST00000457499 | AC104113.3 | 0.539752637 | 0.937437834 | 1.0888887 | up | lncRNA |
| ENST00000457510 | AC002467.7 | 0.365958805 | 0.874818743 | 1.0375252 | up | lncRNA |
| ENST00000457548 | RP11-98D18.1 | 0.814588827 | 0.99257857 | 1.0291414 | up | lncRNA |
| ENST00000457558 | RP11-15B24.5 | 0.383790903 | 0.882562871 | 1.0680905 | up | lncRNA |
| ENST00000457601 | SDHAP1 | 0.456443283 | 0.911362548 | 1.2814394 | up | lncRNA |
| ENST00000457645 | BX248398.1 | 0.121366719 | 0.672928817 | 1.2932624 | up | lncRNA |
| ENST00000457668 | AC007381.3 | 0.278120421 | 0.832297359 | 1.1428779 | up | lncRNA |
| ENST00000457669 | AP000696.2 | 0.754010497 | 0.987008268 | 1.0443836 | up | lncRNA |
| ENST00000457694 | AC098820.2 | 0.202502197 | 0.769638667 | 1.1929899 | up | lncRNA |
| ENST00000457716 | RP11-402P6.7 | 0.344819304 | 0.864698435 | 1.3282558 | up | lncRNA |
| ENST00000457719 | RP11-418J17.3 | 0.300924218 | 0.842645237 | 1.1957295 | up | lncRNA |
| ENST00000457746 | AP006216.5 | 0.335719302 | 0.858998686 | 1.0535491 | up | lncRNA |
| ENST00000457756 | AC073636.1 | 0.057064308 | 0.524319427 | 1.53563 | up | lncRNA |
| ENST00000457770 | PCBP1-AS1 | 0.882752389 | 0.998875101 | 1.0144542 | up | lncRNA |
| ENST00000457775 | XX-FW81066F1.2 | 0.035546527 | 0.432931851 | 1.2771121 | up | lncRNA |
| ENST00000457791 | PMCHL2 | 0.805513415 | 0.992578294 | 1.2368643 | up | lncRNA |
| ENST00000457816 | LINC00489 | 0.111782393 | 0.651593972 | 1.1554766 | up | lncRNA |
| ENST00000457833 | XXyac-YX65C7_A.3 | 0.489945213 | 0.920777644 | 1.1141083 | up | lncRNA |
| ENST00000457852 | RP11-181K3.4 | 0.295346612 | 0.839966018 | 1.1824322 | up | lncRNA |
| ENST00000457853 | RP11-290F20.1 | 0.339361132 | 0.860518281 | 1.0502143 | up | lncRNA |
| ENST00000457870 | AC016700.3 | 0.629173142 | 0.963660225 | 1.0792497 | up | lncRNA |
| ENST00000457926 | AFG3L1P | 0.803190688 | 0.992578294 | 1.0204577 | up | lncRNA |
| ENST00000457975 | RP11-168O22.1 | 0.103348078 | 0.632351488 | 1.1404311 | up | lncRNA |
| ENST00000457991 | RP11-277L2.2 | 0.5534254 | 0.943023022 | 1.1910526 | up | lncRNA |
| ENST00000457996 | RP11-439A17.9 | 0.1278209 | 0.680549576 | 1.9080392 | up | lncRNA |
| ENST00000458016 | RP11-575L7.2 | 0.4442063 | 0.90519468 | 1.2079257 | up | lncRNA |
| ENST00000458028 | RP3-523C21.1 | 0.547931961 | 0.940847434 | 1.369274 | up | lncRNA |
| ENST00000458030 | AC008280.5 | 0.952327996 | 0.998875101 | 1.0249014 | up | lncRNA |
| ENST00000458036 | TOPORS-AS1 | 0.688473613 | 0.97861545 | 1.0536242 | up | lncRNA |
| ENST00000458080 | CTA-292E10.6 | 0.23046698 | 0.79852974 | 1.1796769 | up | lncRNA |
| ENST00000458107 | RP11-3G20.2 | 0.93258491 | 0.998875101 | 1.0257882 | up | lncRNA |
| ENST00000458127 | OSBPL10-AS1 | 0.010073301 | 0.257704119 | 1.4078463 | up | lncRNA |
| ENST00000458136 | RP11-456P18.2 | 0.871851797 | 0.997909851 | 1.0404686 | up | lncRNA |
| ENST00000458146 | RP11-339D23.1 | 0.163039386 | 0.718418509 | 1.5313812 | up | lncRNA |
| ENST00000458149 | AC017099.3 | 0.430369798 | 0.898685776 | 1.2767248 | up | lncRNA |
| ENST00000458171 | RP13-16H11.5 | 0.725802966 | 0.98251396 | 1.0982462 | up | lncRNA |
| ENST00000458207 | RP11-69E11.4 | 0.30768059 | 0.847137912 | 1.2453159 | up | lncRNA |
| ENST00000458228 | RP11-429G19.2 | 0.776183288 | 0.990907538 | 1.0754445 | up | lncRNA |
| ENST00000458254 | AC016751.2 | 0.263282392 | 0.827226956 | 1.1784329 | up | lncRNA |
| ENST00000458261 | RP11-65N13.7 | 0.339560095 | 0.860518281 | 1.0837781 | up | lncRNA |
| ENST00000458262 | AL592284.1 | 8.00382E-05 | 0.041025285 | 3.1660638 | up | lncRNA |
| ENST00000458274 | RP13-507I23.1 | 0.259941083 | 0.824094195 | 1.0900108 | up | lncRNA |
| ENST00000458293 | RP5-897D18.1 | 0.170311981 | 0.730956206 | 1.3931682 | up | lncRNA |
| ENST00000458302 | MIAT | 0.978266616 | 0.998875101 | 1.0011944 | up | lncRNA |
| ENST00000458314 | AC078883.3 | 0.821199882 | 0.993333549 | 1.0553017 | up | lncRNA |
| ENST00000458396 | RP11-835E18.2 | 0.318430786 | 0.851223284 | 1.049536 | up | lncRNA |
| ENST00000458474 | AC004878.7 | 0.349459361 | 0.867400431 | 1.0705347 | up | lncRNA |
| ENST00000458509 | AC004041.2 | 0.801010347 | 0.992578294 | 1.0279138 | up | lncRNA |
| ENST00000458519 | AC008268.2 | 0.811807813 | 0.99257857 | 1.0335569 | up | lncRNA |
| ENST00000458624 | AC007009.1 | 0.783305988 | 0.99121281 | 1.0647076 | up | lncRNA |
| ENST00000458633 | C4A-AS1 | 0.067659517 | 0.549236083 | 1.5587052 | up | lncRNA |
| ENST00000458645 | AC073871.2 | 0.006218034 | 0.212479102 | 1.2202086 | up | lncRNA |
| ENST00000458682 | LINC00202 | 0.541729178 | 0.938657149 | 1.0723131 | up | lncRNA |
| ENST00000458698 | PCBP1-AS1 | 0.317862758 | 0.851223284 | 1.1147641 | up | lncRNA |
| ENST00000458733 | RP11-390P2.4 | 0.034277369 | 0.42757611 | 1.2802053 | up | lncRNA |
| ENST00000460286 | SPATA1 | 0.006750592 | 0.220444566 | 1.884537 | up | lncRNA |
| ENST00000460574 | IQCJ-SCHIP1-AS1 | 0.037576741 | 0.443140005 | 1.3311011 | up | lncRNA |
| ENST00000460707 | RP11-572M11.4 | 0.597334409 | 0.952287813 | 1.0775863 | up | lncRNA |
| ENST00000460772 | AC007952.4 | 0.905014282 | 0.998875101 | 1.0257005 | up | lncRNA |
| ENST00000460796 | RP11-550I24.2 | 0.699753773 | 0.980170937 | 1.040657 | up | lncRNA |
| ENST00000460955 | RP4-545C24.1 | 0.402474121 | 0.889146721 | 1.0589835 | up | lncRNA |
| ENST00000461471 | OR2A9P | 0.621365025 | 0.960457826 | 1.2332886 | up | lncRNA |
| ENST00000461832 | SNHG12 | 0.726064818 | 0.98251396 | 1.03436 | up | lncRNA |
| ENST00000462083 | RP13-452N2.1 | 0.228894319 | 0.798130144 | 1.1195046 | up | lncRNA |
| ENST00000462131 | HOXB-AS4 | 0.441453368 | 0.904580493 | 1.3621026 | up | lncRNA |
| ENST00000462148 | HMCN2 | 0.624524509 | 0.96133281 | 1.0397357 | up | lncRNA |
| ENST00000462305 | RP4-798C17.6 | 0.47765009 | 0.917715179 | 1.0976235 | up | lncRNA |
| ENST00000462431 | CTD-2049J23.2 | 0.174375972 | 0.736164062 | 1.2865338 | up | lncRNA |
| ENST00000462717 | RP11-245J9.4 | 0.96343073 | 0.998875101 | 1.0047841 | up | lncRNA |
| ENST00000462836 | CYMP | 0.938244437 | 0.998875101 | 1.0075118 | up | lncRNA |
| ENST00000462928 | RP11-10O22.2 | 0.825051054 | 0.993856674 | 1.0256909 | up | lncRNA |
| ENST00000463348 | HLA-L | 0.251075229 | 0.817104689 | 1.3864722 | up | lncRNA |
| ENST00000463353 | RP11-331F4.4 | 0.757995618 | 0.988042026 | 1.0357846 | up | lncRNA |
| ENST00000463449 | TIPARP-AS1 | 0.310230362 | 0.848664472 | 1.3969029 | up | lncRNA |
| ENST00000463608 | PI4KAP2 | 0.659659094 | 0.971704562 | 1.0529937 | up | lncRNA |
| ENST00000463755 | RP11-103G8.2 | 0.270685754 | 0.831236357 | 1.131233 | up | lncRNA |
| ENST00000464115 | SNHG12 | 0.852627043 | 0.995900317 | 1.0217907 | up | lncRNA |
| ENST00000464125 | RP11-475O23.3 | 0.594623282 | 0.951072365 | 1.1234812 | up | lncRNA |
| ENST00000464209 | GUCY2GP | 0.287369755 | 0.836961572 | 1.327179 | up | lncRNA |
| ENST00000464216 | RP11-17A4.1 | 0.442684519 | 0.90512482 | 1.0981783 | up | lncRNA |
| ENST00000464612 | SNHG12 | 0.917521533 | 0.998875101 | 1.0213951 | up | lncRNA |
| ENST00000464823 | RP11-631B21.1 | 0.639627642 | 0.96564589 | 1.0665534 | up | lncRNA |
| ENST00000464929 | RP4-545C24.1 | 0.93853473 | 0.998875101 | 1.034524 | up | lncRNA |
| ENST00000465249 | RP11-197K3.1 | 0.694038406 | 0.979611174 | 1.0933967 | up | lncRNA |
| ENST00000465262 | RP11-129K20.2 | 0.806711586 | 0.992578294 | 1.0786747 | up | lncRNA |
| ENST00000465283 | RP11-2A4.3 | 0.568041656 | 0.943923733 | 1.0301971 | up | lncRNA |
| ENST00000465347 | RP11-62G11.2 | 0.12437513 | 0.675636587 | 1.7798361 | up | lncRNA |
| ENST00000465856 | BTN2A3P | 0.001240102 | 0.12293504 | 1.3439779 | up | lncRNA |
| ENST00000466206 | RP11-657O9.1 | 0.903696396 | 0.998875101 | 1.0193202 | up | lncRNA |
| ENST00000466225 | ADAMTS9-AS1 | 0.930256954 | 0.998875101 | 1.0369848 | up | lncRNA |
| ENST00000466343 | RP4-791M13.3 | 0.300096432 | 0.8422517 | 1.3478537 | up | lncRNA |
| ENST00000466677 | RP4-555L14.5 | 0.978900876 | 0.998875101 | 1.0028271 | up | lncRNA |
| ENST00000466717 | RP11-286H14.8 | 0.278855466 | 0.832297359 | 1.1168837 | up | lncRNA |
| ENST00000466730 | ABCA17P | 0.393470034 | 0.885711362 | 1.0587101 | up | lncRNA |
| ENST00000467082 | RP11-697M17.2 | 0.39447717 | 0.885711362 | 1.1581115 | up | lncRNA |
| ENST00000467342 | RP11-572M11.4 | 0.528543777 | 0.934265662 | 1.1194741 | up | lncRNA |
| ENST00000467458 | RP11-796I2.2 | 0.019446575 | 0.348001551 | 1.3538975 | up | lncRNA |
| ENST00000467484 | RP11-293A21.1 | 0.973312358 | 0.998875101 | 1.0018811 | up | lncRNA |
| ENST00000467614 | RP11-286H14.4 | 0.125271219 | 0.67731705 | 1.1980272 | up | lncRNA |
| ENST00000467677 | AC068610.3 | 0.835803798 | 0.995060648 | 1.0410673 | up | lncRNA |
| ENST00000467807 | ZNF542 | 0.704578614 | 0.980849889 | 1.0734475 | up | lncRNA |
| ENST00000467912 | ARHGEF26-AS1 | 0.561946157 | 0.943393901 | 1.0628635 | up | lncRNA |
| ENST00000467944 | RP4-798C17.6 | 0.935767081 | 0.998875101 | 1.0297377 | up | lncRNA |
| ENST00000467995 | RP11-6F2.6 | 0.158780042 | 0.714694414 | 1.422122 | up | lncRNA |
| ENST00000468266 | RP11-61L23.2 | 0.210083833 | 0.777895554 | 1.2645697 | up | lncRNA |
| ENST00000468356 | RP11-1191J2.2 | 0.889799709 | 0.998875101 | 1.0305831 | up | lncRNA |
| ENST00000468437 | SPATA1 | 0.00076956 | 0.098727717 | 1.8654486 | up | lncRNA |
| ENST00000468859 | RP11-379B18.5 | 0.803258092 | 0.992578294 | 1.090566 | up | lncRNA |
| ENST00000468961 | THOC7-AS1 | 0.818279107 | 0.992651327 | 1.0213559 | up | lncRNA |
| ENST00000469070 | RP11-18H7.1 | 0.007226762 | 0.225474959 | 2.014531 | up | lncRNA |
| ENST00000469148 | RP11-204J18.2 | 0.263293808 | 0.827226956 | 1.2176165 | up | lncRNA |
| ENST00000469178 | RP11-433A10.1 | 0.049273234 | 0.501683335 | 1.1173527 | up | lncRNA |
| ENST00000469196 | RP11-6F2.5 | 0.988696457 | 0.99906281 | 1.002896 | up | lncRNA |
| ENST00000469216 | RP11-2A4.3 | 0.028815752 | 0.406089833 | 1.3003936 | up | lncRNA |
| ENST00000469268 | RP11-166N6.2 | 0.585282373 | 0.950008213 | 1.1781644 | up | lncRNA |
| ENST00000469289 | MIR1302-10 | 0.090006142 | 0.604305136 | 1.2465275 | up | lncRNA |
| ENST00000469484 | WNT5A-AS1 | 0.69818434 | 0.980170937 | 1.1369451 | up | lncRNA |
| ENST00000469595 | AC007163.4 | 0.925605615 | 0.998875101 | 1.0344549 | up | lncRNA |
| ENST00000469928 | RPS27P25 | 0.587284612 | 0.950589384 | 1.0600204 | up | lncRNA |
| ENST00000470135 | RP5-884M6.1 | 0.158389029 | 0.714689048 | 1.2587861 | up | lncRNA |
| ENST00000470313 | RP11-572M11.4 | 0.521522214 | 0.932117592 | 1.1788272 | up | lncRNA |
| ENST00000470427 | RP11-755B10.2 | 0.716331136 | 0.98251396 | 1.1364016 | up | lncRNA |
| ENST00000470911 | AOX2P | 0.646626691 | 0.969404706 | 1.0240341 | up | lncRNA |
| ENST00000470997 | HLA-DPB2 | 0.668619225 | 0.974314461 | 1.0797952 | up | lncRNA |
| ENST00000471248 | RP11-34P13.7 | 0.29460123 | 0.839411724 | 1.2378645 | up | lncRNA |
| ENST00000471276 | TMEM198B | 0.229829603 | 0.79852974 | 1.1083384 | up | lncRNA |
| ENST00000471614 | RP11-359D24.1 | 0.060525829 | 0.531294612 | 1.4831056 | up | lncRNA |
| ENST00000471731 | MCCC1-AS1 | 0.744185473 | 0.985094857 | 1.0283457 | up | lncRNA |
| ENST00000472178 | LINC00518 | 0.959465346 | 0.998875101 | 1.0059544 | up | lncRNA |
| ENST00000472376 | AOX2P | 0.12911695 | 0.682265946 | 1.5339313 | up | lncRNA |
| ENST00000472513 | RP11-475O23.2 | 0.573401792 | 0.945806518 | 1.1410432 | up | lncRNA |
| ENST00000472666 | PRKY | 0.513799499 | 0.929306919 | 1.2131857 | up | lncRNA |
| ENST00000472761 | GLYCTK-AS1 | 0.425102125 | 0.897780553 | 1.0304311 | up | lncRNA |
| ENST00000472890 | RP11-167H9.6 | 0.053363254 | 0.515925762 | 1.3659128 | up | lncRNA |
| ENST00000472972 | KCNMB3P1 | 0.96435231 | 0.998875101 | 1.0038432 | up | lncRNA |
| ENST00000473001 | RP11-657O9.1 | 0.813427881 | 0.99257857 | 1.0726465 | up | lncRNA |
| ENST00000473164 | AC016629.2 | 0.820649809 | 0.993333549 | 1.0102526 | up | lncRNA |
| ENST00000473352 | RP11-392A22.2 | 0.745578703 | 0.985094857 | 1.0901333 | up | lncRNA |
| ENST00000473454 | CCDC162P | 0.25550196 | 0.821835543 | 1.0827555 | up | lncRNA |
| ENST00000473756 | CTD-2021J15.2 | 0.981030395 | 0.998875101 | 1.0067197 | up | lncRNA |
| ENST00000473885 | TUBA4B | 0.069189425 | 0.554442565 | 1.0870217 | up | lncRNA |
| ENST00000473958 | RP11-475N22.4 | 0.332150467 | 0.857061033 | 1.2484657 | up | lncRNA |
| ENST00000474264 | RPL17P42 | 0.935156033 | 0.998875101 | 1.007168 | up | lncRNA |
| ENST00000474419 | AC008888.7 | 0.766997791 | 0.989189555 | 1.0361885 | up | lncRNA |
| ENST00000474444 | RP11-167H9.5 | 0.413759124 | 0.890927603 | 1.0673349 | up | lncRNA |
| ENST00000474477 | RP11-6F2.6 | 0.702490149 | 0.980188885 | 1.0461778 | up | lncRNA |
| ENST00000474826 | RP11-4K3__A.2 | 0.382502731 | 0.881294633 | 1.052388 | up | lncRNA |
| ENST00000475089 | RP4-798C17.6 | 0.972413895 | 0.998875101 | 1.0127678 | up | lncRNA |
| ENST00000475102 | RP11-550I24.2 | 0.20438244 | 0.771514146 | 1.2382745 | up | lncRNA |
| ENST00000475362 | RP11-631B21.1 | 0.479657413 | 0.918546036 | 1.1683219 | up | lncRNA |
| ENST00000475393 | RP11-103G8.2 | 0.375710327 | 0.879312727 | 1.0406398 | up | lncRNA |
| ENST00000475791 | CTB-111H14.1 | 0.44678693 | 0.905331314 | 1.2898524 | up | lncRNA |
| ENST00000475886 | RP11-129K20.2 | 0.275351995 | 0.832297359 | 1.3683035 | up | lncRNA |
| ENST00000476276 | PRSS30P | 0.338482747 | 0.86050695 | 1.0685312 | up | lncRNA |
| ENST00000476301 | PVRL3-AS1 | 0.869008067 | 0.997455886 | 1.0058429 | up | lncRNA |
| ENST00000476426 | RP11-163E9.2 | 0.133905143 | 0.689601422 | 1.2645986 | up | lncRNA |
| ENST00000476560 | RP4-798C17.6 | 0.726567353 | 0.98251396 | 1.164234 | up | lncRNA |
| ENST00000476569 | RP4-778K6.3 | 0.642779682 | 0.967175883 | 1.1374889 | up | lncRNA |
| ENST00000477141 | CTD-3099C6.4 | 0.732295312 | 0.983763148 | 1.0340343 | up | lncRNA |
| ENST00000477296 | PI4KAP2 | 0.281538855 | 0.832297359 | 1.2299633 | up | lncRNA |
| ENST00000477404 | RPS27P25 | 0.160100261 | 0.714935095 | 1.0890662 | up | lncRNA |
| ENST00000477702 | LINC00173 | 0.269877967 | 0.830995087 | 1.3176198 | up | lncRNA |
| ENST00000478103 | C17orf76-AS1 | 0.632102175 | 0.964380442 | 1.2202469 | up | lncRNA |
| ENST00000478241 | TMEM198B | 0.33319367 | 0.857704692 | 1.2424431 | up | lncRNA |
| ENST00000478294 | RP13-463N16.6 | 0.780993769 | 0.99121281 | 1.1997696 | up | lncRNA |
| ENST00000478301 | ZBTB20-AS2 | 0.225045328 | 0.794551181 | 1.1742012 | up | lncRNA |
| ENST00000478379 | TCAM1P | 0.836780304 | 0.995078046 | 1.0362702 | up | lncRNA |
| ENST00000478468 | RP11-521D12.1 | 0.182431715 | 0.749358894 | 1.2607902 | up | lncRNA |
| ENST00000478745 | RP11-654C22.2 | 0.96007166 | 0.998875101 | 1.0025268 | up | lncRNA |
| ENST00000478823 | RP11-372E1.6 | 0.738470278 | 0.984535571 | 1.0476019 | up | lncRNA |
| ENST00000478824 | CTD-2377D24.6 | 0.404557775 | 0.889198786 | 1.0315035 | up | lncRNA |
| ENST00000478994 | SERPINA13 | 0.054293295 | 0.516748735 | 1.8275523 | up | lncRNA |
| ENST00000479041 | AC007163.4 | 0.1913566 | 0.760520345 | 1.7984907 | up | lncRNA |
| ENST00000479085 | RP11-148K1.10 | 0.676813819 | 0.976096237 | 1.0643517 | up | lncRNA |
| ENST00000479102 | OR2A9P | 0.706041505 | 0.981012299 | 1.1690719 | up | lncRNA |
| ENST00000479270 | ARHGEF26-AS1 | 0.632125346 | 0.964380442 | 1.0501735 | up | lncRNA |
| ENST00000479299 | DTX2 | 0.072638801 | 0.563215598 | 1.2787185 | up | lncRNA |
| ENST00000479612 | RP11-280F2.2 | 0.545484465 | 0.939697392 | 1.2409459 | up | lncRNA |
| ENST00000479887 | OR2A20P | 0.8284607 | 0.994074191 | 1.1206262 | up | lncRNA |
| ENST00000479920 | RP11-506M13.3 | 0.317203408 | 0.851223284 | 1.0993285 | up | lncRNA |
| ENST00000480074 | RP4-545C24.1 | 0.61985901 | 0.960092436 | 1.0645122 | up | lncRNA |
| ENST00000480237 | LINC00173 | 0.980006047 | 0.998875101 | 1.003568 | up | lncRNA |
| ENST00000480319 | PI4KAP2 | 0.81830053 | 0.992651327 | 1.0615122 | up | lncRNA |
| ENST00000480386 | HOXB-AS4 | 0.609550342 | 0.957036048 | 1.0975397 | up | lncRNA |
| ENST00000480427 | RPL22P19 | 0.414674636 | 0.891730652 | 1.0922759 | up | lncRNA |
| ENST00000480805 | MBL1P | 0.039744137 | 0.45449377 | 1.2388267 | up | lncRNA |
| ENST00000480817 | RP11-555M1.3 | 0.102534503 | 0.632351488 | 1.3979794 | up | lncRNA |
| ENST00000481351 | ADAM3A | 0.072301218 | 0.562178401 | 1.6857478 | up | lncRNA |
| ENST00000481582 | ABCC13 | 0.652165018 | 0.969927925 | 1.1032923 | up | lncRNA |
| ENST00000481651 | RP11-61L23.2 | 0.882806976 | 0.998875101 | 1.0142055 | up | lncRNA |
| ENST00000481968 | RP4-800G7.2 | 0.280564085 | 0.832297359 | 1.0971159 | up | lncRNA |
| ENST00000482003 | RP11-47P18.1 | 0.075318877 | 0.567570001 | 1.3354896 | up | lncRNA |
| ENST00000482005 | TCAM1P | 0.180820612 | 0.747570548 | 1.1578124 | up | lncRNA |
| ENST00000482249 | PLGLA | 0.676855885 | 0.976096237 | 1.0294453 | up | lncRNA |
| ENST00000482368 | CTD-2013N24.2 | 0.290901453 | 0.837264328 | 1.1055159 | up | lncRNA |
| ENST00000482378 | TMEM198B | 0.410656636 | 0.890697301 | 1.1643214 | up | lncRNA |
| ENST00000482381 | RP4-565E6.1 | 0.294869887 | 0.839677107 | 1.3459702 | up | lncRNA |
| ENST00000482382 | RP11-246A10.1 | 0.456022691 | 0.910972327 | 1.2397494 | up | lncRNA |
| ENST00000482559 | RP11-4B14.3 | 0.290861106 | 0.837264328 | 1.2600719 | up | lncRNA |
| ENST00000482985 | RP11-506M13.3 | 0.701944402 | 0.980170937 | 1.0661278 | up | lncRNA |
| ENST00000483080 | RP11-773D16.1 | 0.736099716 | 0.984025164 | 1.0527128 | up | lncRNA |
| ENST00000483289 | RP11-379K17.4 | 0.03240801 | 0.42322089 | 1.2147322 | up | lncRNA |
| ENST00000483588 | C17orf76-AS1 | 0.508812282 | 0.92652835 | 1.2590395 | up | lncRNA |
| ENST00000484046 | TM4SF1-AS1 | 0.002844446 | 0.155814844 | 1.1460569 | up | lncRNA |
| ENST00000484076 | RP11-18H7.1 | 0.184396721 | 0.751257244 | 1.5426195 | up | lncRNA |
| ENST00000484406 | AC007163.4 | 0.713775741 | 0.98251396 | 1.0540895 | up | lncRNA |
| ENST00000484794 | RP11-773D16.1 | 0.794385132 | 0.991420185 | 1.0331896 | up | lncRNA |
| ENST00000484859 | RP11-34P13.12 | 0.996294701 | 0.999156445 | 1.001865 | up | lncRNA |
| ENST00000484939 | SPATA1 | 0.000170544 | 0.066152698 | 2.4439974 | up | lncRNA |
| ENST00000485162 | MYLK-AS1 | 0.424475783 | 0.89750944 | 1.0823588 | up | lncRNA |
| ENST00000485185 | OR9A3P | 0.495709096 | 0.921717611 | 1.039622 | up | lncRNA |
| ENST00000485218 | RUVBL1-AS1 | 0.890675488 | 0.998875101 | 1.0278051 | up | lncRNA |
| ENST00000485404 | RP11-433A10.1 | 0.327617267 | 0.854556781 | 1.2681421 | up | lncRNA |
| ENST00000485827 | AC004057.1 | 0.259139505 | 0.824094195 | 1.1453255 | up | lncRNA |
| ENST00000485898 | RP11-18H7.1 | 0.004280623 | 0.187303349 | 2.6718957 | up | lncRNA |
| ENST00000485974 | RP11-148K1.12 | 0.358827013 | 0.873305967 | 1.2450149 | up | lncRNA |
| ENST00000486094 | RP4-798C17.6 | 0.783392979 | 0.99121281 | 1.0449757 | up | lncRNA |
| ENST00000486431 | RP11-666A20.1 | 0.529177382 | 0.934463866 | 1.0413056 | up | lncRNA |
| ENST00000486545 | RP11-439C8.2 | 0.972744142 | 0.998875101 | 1.0077276 | up | lncRNA |
| ENST00000486551 | MT1DP | 0.098413048 | 0.624143367 | 2.1540121 | up | lncRNA |
| ENST00000486997 | TUBA4B | 0.336760695 | 0.859588936 | 1.1352638 | up | lncRNA |
| ENST00000487102 | RP4-798C17.6 | 0.812048876 | 0.99257857 | 1.0961007 | up | lncRNA |
| ENST00000487111 | RP11-147I3.1 | 0.328273859 | 0.854893129 | 1.3262877 | up | lncRNA |
| ENST00000487580 | RP11-379K17.4 | 0.410425594 | 0.890570958 | 1.3748458 | up | lncRNA |
| ENST00000487582 | TMEM198B | 0.838211498 | 0.995583664 | 1.0420752 | up | lncRNA |
| ENST00000488041 | RP4-798C17.6 | 0.687128095 | 0.97861545 | 1.0257227 | up | lncRNA |
| ENST00000488192 | RPS20P22 | 0.32913217 | 0.85540421 | 1.0482915 | up | lncRNA |
| ENST00000489074 | INGX | 0.025978299 | 0.383305135 | 1.7501192 | up | lncRNA |
| ENST00000489077 | RP4-545C24.1 | 0.826410915 | 0.993856674 | 1.0928108 | up | lncRNA |
| ENST00000489205 | AP000221.2 | 0.763949234 | 0.989015485 | 1.040363 | up | lncRNA |
| ENST00000489257 | MT1P2 | 0.10465012 | 0.635875748 | 2.0088423 | up | lncRNA |
| ENST00000489312 | RP11-1191J2.2 | 0.870481026 | 0.997909851 | 1.0210294 | up | lncRNA |
| ENST00000489396 | RP11-320N7.1 | 0.50436825 | 0.92405258 | 1.0262313 | up | lncRNA |
| ENST00000489452 | LINC00173 | 0.308563342 | 0.847582029 | 1.118446 | up | lncRNA |
| ENST00000489488 | RP4-798C17.6 | 0.86621358 | 0.996767702 | 1.0197334 | up | lncRNA |
| ENST00000489609 | FAM86DP | 0.97785369 | 0.998875101 | 1.0023524 | up | lncRNA |
| ENST00000490139 | RP11-572M11.3 | 0.606412612 | 0.956038292 | 1.111806 | up | lncRNA |
| ENST00000490153 | SLC9A9-AS2 | 0.109833784 | 0.645245382 | 1.6955962 | up | lncRNA |
| ENST00000490162 | RP5-884M6.1 | 0.225258688 | 0.79487168 | 1.1187601 | up | lncRNA |
| ENST00000490269 | GUCY2GP | 0.244291801 | 0.810806356 | 1.3169693 | up | lncRNA |
| ENST00000490341 | TUBA4B | 0.433384875 | 0.900875689 | 1.0789805 | up | lncRNA |
| ENST00000490375 | RP11-274H2.3 | 0.860844462 | 0.996142678 | 1.0346465 | up | lncRNA |
| ENST00000490465 | RP11-639B1.1 | 0.002372585 | 0.145414844 | 1.8800334 | up | lncRNA |
| ENST00000490535 | RPS2P45 | 0.337136896 | 0.859588936 | 1.0660552 | up | lncRNA |
| ENST00000490916 | RP11-204J18.3 | 0.895721674 | 0.998875101 | 1.0596237 | up | lncRNA |
| ENST00000491264 | HOXB-AS3 | 0.481510267 | 0.9192486 | 1.0914147 | up | lncRNA |
| ENST00000491347 | RP13-635I23.3 | 0.616291753 | 0.959051918 | 1.0568785 | up | lncRNA |
| ENST00000491562 | PI4KAP2 | 0.003175932 | 0.164803479 | 1.3479787 | up | lncRNA |
| ENST00000491596 | MYADML | 0.562102941 | 0.943393901 | 1.2012999 | up | lncRNA |
| ENST00000492054 | RP11-545G3.1 | 0.178771534 | 0.743651912 | 1.1342043 | up | lncRNA |
| ENST00000492300 | RP11-734K21.3 | 0.268124681 | 0.829967639 | 1.3038259 | up | lncRNA |
| ENST00000492523 | RP11-758P17.2 | 0.247555016 | 0.814489937 | 1.2270704 | up | lncRNA |
| ENST00000492569 | RP11-292E2.3 | 0.063230679 | 0.536829664 | 1.4591719 | up | lncRNA |
| ENST00000492683 | RP11-64C1.1 | 0.523028902 | 0.932117592 | 1.0794527 | up | lncRNA |
| ENST00000492725 | ATP1B3-AS1 | 0.006293811 | 0.21455765 | 1.7649899 | up | lncRNA |
| ENST00000492786 | RPLP0P2 | 0.980206424 | 0.998875101 | 1.0021184 | up | lncRNA |
| ENST00000492937 | TIPARP-AS1 | 0.650450475 | 0.969927925 | 1.2215997 | up | lncRNA |
| ENST00000493028 | RPL21P123 | 0.300025551 | 0.8422517 | 1.0580697 | up | lncRNA |
| ENST00000493198 | ZNF767 | 0.831054111 | 0.994074191 | 1.0247952 | up | lncRNA |
| ENST00000493239 | RP11-61L23.2 | 0.813008073 | 0.99257857 | 1.036929 | up | lncRNA |
| ENST00000493248 | RP4-545C24.1 | 0.945028251 | 0.998875101 | 1.0296612 | up | lncRNA |
| ENST00000493539 | RP4-798C17.6 | 0.923987242 | 0.998875101 | 1.0314796 | up | lncRNA |
| ENST00000493616 | GLYCTK-AS1 | 0.956199384 | 0.998875101 | 1.006992 | up | lncRNA |
| ENST00000493696 | KCNMB3P1 | 0.7825752 | 0.99121281 | 1.0255679 | up | lncRNA |
| ENST00000494130 | RPS10P20 | 0.410340402 | 0.890549744 | 1.1211935 | up | lncRNA |
| ENST00000494289 | RP11-707F14.3 | 0.238097764 | 0.807531256 | 1.4172345 | up | lncRNA |
| ENST00000494367 | HLA-J | 0.737930208 | 0.984316636 | 1.0837734 | up | lncRNA |
| ENST00000494740 | PI4KAP2 | 0.589010274 | 0.950589384 | 1.0251321 | up | lncRNA |
| ENST00000495081 | AC007620.3 | 0.073651227 | 0.56397543 | 1.9977088 | up | lncRNA |
| ENST00000495094 | WWTR1-AS1 | 0.529607682 | 0.934578809 | 1.069911 | up | lncRNA |
| ENST00000495228 | RP11-446H18.3 | 0.426360236 | 0.897780553 | 1.2372556 | up | lncRNA |
| ENST00000495536 | CTD-2377D24.4 | 0.570068942 | 0.944496311 | 1.0548379 | up | lncRNA |
| ENST00000495542 | RP11-204J18.3 | 0.266055268 | 0.828881561 | 1.1976135 | up | lncRNA |
| ENST00000495939 | ARHGEF3-AS1 | 0.127395038 | 0.680198507 | 1.1763917 | up | lncRNA |
| ENST00000496154 | LSAMP-AS3 | 0.546056718 | 0.939898322 | 1.1632375 | up | lncRNA |
| ENST00000496192 | OR6W1P | 0.008653862 | 0.246429015 | 1.2560652 | up | lncRNA |
| ENST00000496247 | RP11-637O11.2 | 0.509917342 | 0.926917674 | 1.214281 | up | lncRNA |
| ENST00000496359 | RP11-773D16.1 | 0.724223997 | 0.98251396 | 1.0442113 | up | lncRNA |
| ENST00000496403 | RP11-12K11.2 | 0.275819924 | 0.832297359 | 1.1449818 | up | lncRNA |
| ENST00000496733 | TMEM161B-AS1 | 0.957876622 | 0.998875101 | 1.0172223 | up | lncRNA |
| ENST00000496773 | BAGE2 | 0.917169587 | 0.998875101 | 1.0179574 | up | lncRNA |
| ENST00000496968 | RP4-798C17.6 | 0.895899252 | 0.998875101 | 1.047441 | up | lncRNA |
| ENST00000496975 | AC084117.3 | 0.023397849 | 0.369670635 | 2.4575118 | up | lncRNA |
| ENST00000496994 | RP11-768G7.2 | 0.256117335 | 0.822133742 | 1.07215 | up | lncRNA |
| ENST00000497195 | CARM1P1 | 0.23109693 | 0.79852974 | 1.1156702 | up | lncRNA |
| ENST00000497258 | RP11-285B24.1 | 0.00443873 | 0.188856054 | 1.2406982 | up | lncRNA |
| ENST00000497329 | ANKRD36B | 0.820300793 | 0.993333549 | 1.032273 | up | lncRNA |
| ENST00000497397 | RP11-731F5.2 | 0.398445397 | 0.887618401 | 1.0701079 | up | lncRNA |
| ENST00000497576 | CTC-260E6.2 | 0.692424583 | 0.97895216 | 1.0350611 | up | lncRNA |
| ENST00000497652 | RP11-372E1.6 | 0.904168292 | 0.998875101 | 1.0342786 | up | lncRNA |
| ENST00000497718 | C3P1 | 0.672605283 | 0.974876341 | 1.0688162 | up | lncRNA |
| ENST00000497872 | AL928768.3 | 0.307413725 | 0.847137912 | 1.1857594 | up | lncRNA |
| ENST00000497961 | RP11-242C19.2 | 0.001683182 | 0.138499834 | 6.0763821 | up | lncRNA |
| ENST00000498005 | RP11-167H9.6 | 0.809213658 | 0.992578294 | 1.0413617 | up | lncRNA |
| ENST00000498233 | OR52A4 | 0.457586704 | 0.912197894 | 1.144371 | up | lncRNA |
| ENST00000498604 | RP11-656A15.1 | 0.135813926 | 0.693269931 | 1.2907321 | up | lncRNA |
| ENST00000498655 | RP11-204J18.3 | 0.97515658 | 0.998875101 | 1.0035919 | up | lncRNA |
| ENST00000498671 | RPL29P2 | 0.915453186 | 0.998875101 | 1.0061732 | up | lncRNA |
| ENST00000498687 | RP11-331F4.4 | 0.651078144 | 0.969927925 | 1.1314438 | up | lncRNA |
| ENST00000498693 | RP4-545C24.1 | 0.81300317 | 0.99257857 | 1.1050543 | up | lncRNA |
| ENST00000498745 | RP11-286H14.6 | 0.024533094 | 0.375772648 | 1.1137462 | up | lncRNA |
| ENST00000498872 | RP11-158I9.5 | 0.346447832 | 0.865832425 | 1.200193 | up | lncRNA |
| ENST00000498923 | NUDT16P | 0.423000123 | 0.896205753 | 1.1455886 | up | lncRNA |
| ENST00000499077 | NUDT16P | 0.237649249 | 0.806512655 | 1.0754543 | up | lncRNA |
| ENST00000499096 | CTC-338M12.7 | 0.201635631 | 0.768512499 | 1.2321902 | up | lncRNA |
| ENST00000499137 | RP11-887P2.1 | 0.789034554 | 0.991420185 | 1.0239971 | up | lncRNA |
| ENST00000499481 | RP11-620J15.2 | 0.493320012 | 0.921690406 | 1.099591 | up | lncRNA |
| ENST00000499504 | RP11-736K20.6 | 0.067332424 | 0.548255059 | 1.5183893 | up | lncRNA |
| ENST00000499525 | RP5-1186N24.3 | 0.843873403 | 0.995583664 | 1.0356089 | up | lncRNA |
| ENST00000499554 | CTD-2547L16.1 | 0.344400093 | 0.864282241 | 1.0533717 | up | lncRNA |
| ENST00000499653 | KB-431C1.4 | 0.551884996 | 0.942293226 | 1.0931053 | up | lncRNA |
| ENST00000499685 | RP11-796E2.4 | 0.443180711 | 0.90512482 | 1.1830502 | up | lncRNA |
| ENST00000499732 | NEAT1 | 0.107970495 | 0.640327498 | 1.1353268 | up | lncRNA |
| ENST00000499797 | RP11-325N19.3 | 0.291456993 | 0.837264328 | 1.2141819 | up | lncRNA |
| ENST00000499910 | CTD-2506J14.1 | 0.687571547 | 0.97861545 | 1.1778842 | up | lncRNA |
| ENST00000500180 | RP11-65D17.1 | 0.943580737 | 0.998875101 | 1.0238618 | up | lncRNA |
| ENST00000500215 | RP11-7F17.5 | 0.360988549 | 0.873342057 | 1.2701208 | up | lncRNA |
| ENST00000500262 | RP11-1379J22.2 | 0.144766103 | 0.69994374 | 2.1598207 | up | lncRNA |
| ENST00000500267 | RP11-325L7.1 | 0.405953612 | 0.889320642 | 1.1257769 | up | lncRNA |
| ENST00000500370 | SNHG10 | 0.800104013 | 0.992303072 | 1.0275231 | up | lncRNA |
| ENST00000500590 | RP3-330M21.5 | 0.04430729 | 0.478839027 | 1.4666642 | up | lncRNA |
| ENST00000500612 | RP11-510M2.2 | 0.083403497 | 0.586193429 | 1.2736555 | up | lncRNA |
| ENST00000500636 | RP3-510L9.1 | 0.13305438 | 0.688794697 | 1.233629 | up | lncRNA |
| ENST00000500941 | RP11-1000B6.5 | 0.969012283 | 0.998875101 | 1.0059347 | up | lncRNA |
| ENST00000500986 | RP11-887P2.1 | 0.484057818 | 0.919439531 | 1.0368829 | up | lncRNA |
| ENST00000501008 | RP11-796E2.4 | 0.045683299 | 0.482803175 | 2.4265886 | up | lncRNA |
| ENST00000501016 | KB-1208A12.3 | 0.071970349 | 0.5610638 | 1.2139758 | up | lncRNA |
| ENST00000501173 | CTC-228N24.3 | 0.10896344 | 0.642763377 | 1.0656842 | up | lncRNA |
| ENST00000501356 | RP11-712B9.2 | 0.040398755 | 0.458342242 | 1.4555759 | up | lncRNA |
| ENST00000501440 | CTD-3064M3.4 | 0.094550808 | 0.615695643 | 1.1134008 | up | lncRNA |
| ENST00000501541 | RP11-613D13.8 | 0.163195017 | 0.718418509 | 1.5525203 | up | lncRNA |
| ENST00000501652 | CTC-228N24.3 | 0.633216105 | 0.964607725 | 1.0374733 | up | lncRNA |
| ENST00000501718 | RP11-700H6.1 | 0.480164201 | 0.918546036 | 1.1241187 | up | lncRNA |
| ENST00000501725 | RP11-103J17.1 | 0.791135522 | 0.991420185 | 1.0184269 | up | lncRNA |
| ENST00000501818 | CTB-127C13.1 | 0.01052599 | 0.261616866 | 1.5477213 | up | lncRNA |
| ENST00000501976 | RP11-15B17.1 | 0.645383634 | 0.968341709 | 1.0714961 | up | lncRNA |
| ENST00000502027 | RP11-358M11.2 | 0.639606555 | 0.96564589 | 1.0675806 | up | lncRNA |
| ENST00000502069 | RP11-877E17.2 | 0.926100428 | 0.998875101 | 1.0078866 | up | lncRNA |
| ENST00000502253 | CTD-2269F5.1 | 0.653152569 | 0.97018076 | 1.0467159 | up | lncRNA |
| ENST00000502278 | ALDH1L1-AS2 | 0.191751612 | 0.760520345 | 1.0966999 | up | lncRNA |
| ENST00000502284 | RP11-849H4.4 | 0.829640953 | 0.994074191 | 1.0397553 | up | lncRNA |
| ENST00000502300 | RP11-94C24.6 | 0.701095335 | 0.980170937 | 1.1238514 | up | lncRNA |
| ENST00000502373 | LNX1-AS1 | 0.913292533 | 0.998875101 | 1.0243339 | up | lncRNA |
| ENST00000502419 | RP11-366H4.1 | 0.477758234 | 0.917735717 | 1.1504424 | up | lncRNA |
| ENST00000502421 | CTB-1I21.1 | 0.874730417 | 0.997909851 | 1.0388952 | up | lncRNA |
| ENST00000502435 | CTB-22K21.2 | 0.142314011 | 0.698941469 | 1.6210652 | up | lncRNA |
| ENST00000502496 | TMPRSS11GP | 0.272803959 | 0.832297359 | 1.2448492 | up | lncRNA |
| ENST00000502645 | CTD-2337A12.1 | 0.521952712 | 0.932117592 | 1.3461371 | up | lncRNA |
| ENST00000502712 | RP11-68L1.2 | 0.221550639 | 0.790772138 | 1.1121304 | up | lncRNA |
| ENST00000502767 | RP11-458I7.4 | 0.302690703 | 0.843987144 | 1.0930507 | up | lncRNA |
| ENST00000502790 | RP11-692D12.1 | 0.106759237 | 0.638686355 | 1.5554991 | up | lncRNA |
| ENST00000502934 | RP11-348J24.2 | 0.287835932 | 0.836961572 | 1.224186 | up | lncRNA |
| ENST00000502993 | CTD-2254N19.1 | 0.566938304 | 0.943923733 | 1.0922125 | up | lncRNA |
| ENST00000502999 | RP11-227H4.5 | 0.141934829 | 0.698926614 | 1.082907 | up | lncRNA |
| ENST00000503007 | CTD-2201I18.1 | 0.264357079 | 0.827574498 | 1.1869789 | up | lncRNA |
| ENST00000503066 | RP11-291L15.2 | 0.861728387 | 0.996142678 | 1.0499878 | up | lncRNA |
| ENST00000503091 | AC008592.5 | 0.018356269 | 0.339651019 | 1.6738866 | up | lncRNA |
| ENST00000503186 | RP11-774O3.1 | 0.394820533 | 0.885711362 | 1.1484211 | up | lncRNA |
| ENST00000503263 | RP11-423H2.3 | 0.873616674 | 0.997909851 | 1.0898684 | up | lncRNA |
| ENST00000503267 | RP11-321E2.4 | 0.494255 | 0.921717611 | 1.1934706 | up | lncRNA |
| ENST00000503268 | CTC-340D7.1 | 0.001544121 | 0.135129436 | 2.4771931 | up | lncRNA |
| ENST00000503309 | RP11-471J12.1 | 0.017843143 | 0.337638939 | 2.0783081 | up | lncRNA |
| ENST00000503325 | RP11-161D15.3 | 0.713835343 | 0.98251396 | 1.1478133 | up | lncRNA |
| ENST00000503386 | CTD-2194D22.1 | 0.698531668 | 0.980170937 | 1.1306725 | up | lncRNA |
| ENST00000503394 | RP11-552M14.1 | 0.692729639 | 0.978978277 | 1.0705761 | up | lncRNA |
| ENST00000503395 | TUBBP5 | 0.226399255 | 0.795886026 | 1.4001223 | up | lncRNA |
| ENST00000503405 | RP11-440L14.1 | 0.571590095 | 0.94531531 | 1.1319485 | up | lncRNA |
| ENST00000503428 | RP11-1379J22.5 | 0.959536724 | 0.998875101 | 1.0135387 | up | lncRNA |
| ENST00000503458 | RP11-219G10.3 | 0.715829035 | 0.98251396 | 1.4389722 | up | lncRNA |
| ENST00000503529 | CTC-210G5.1 | 0.432789114 | 0.900592919 | 1.0485992 | up | lncRNA |
| ENST00000503532 | RP11-341G5.1 | 0.262766327 | 0.826066228 | 1.2313202 | up | lncRNA |
| ENST00000503557 | RP11-26P13.2 | 0.361053452 | 0.873342057 | 1.0857062 | up | lncRNA |
| ENST00000503580 | RP11-219G10.3 | 0.943655604 | 0.998875101 | 1.0518766 | up | lncRNA |
| ENST00000503597 | RP11-55L3.1 | 0.510413947 | 0.927273541 | 1.0285579 | up | lncRNA |
| ENST00000503611 | RP11-597D13.9 | 0.96552467 | 0.998875101 | 1.0648405 | up | lncRNA |
| ENST00000503650 | RP11-6N13.1 | 0.231503346 | 0.799070713 | 1.1042153 | up | lncRNA |
| ENST00000503666 | RP11-529H2.1 | 0.756630358 | 0.98762882 | 1.0942814 | up | lncRNA |
| ENST00000503677 | RP11-91J3.3 | 0.434465946 | 0.900880094 | 1.1179456 | up | lncRNA |
| ENST00000503685 | CTD-2582M21.1 | 0.494127762 | 0.921717611 | 1.1892834 | up | lncRNA |
| ENST00000503710 | CTC-558O19.1 | 0.564076971 | 0.943393901 | 1.1102212 | up | lncRNA |
| ENST00000504082 | AC004069.2 | 0.159772774 | 0.714806047 | 1.3559385 | up | lncRNA |
| ENST00000504129 | CTC-498J12.3 | 0.066065507 | 0.543687263 | 1.6326091 | up | lncRNA |
| ENST00000504145 | RP11-37B2.1 | 0.012798319 | 0.287002307 | 3.3318891 | up | lncRNA |
| ENST00000504249 | RP13-560N11.1 | 0.074253325 | 0.565049692 | 1.4009906 | up | lncRNA |
| ENST00000504307 | HILS1 | 0.795264533 | 0.991420185 | 1.0807144 | up | lncRNA |
| ENST00000504322 | NUP210P1 | 0.223134218 | 0.792094292 | 1.2527792 | up | lncRNA |
| ENST00000504332 | RP11-521B24.3 | 0.877886827 | 0.998682284 | 1.0220773 | up | lncRNA |
| ENST00000504335 | RP3-412A9.10 | 0.003539984 | 0.172223227 | 1.4367791 | up | lncRNA |
| ENST00000504337 | RP1-5O6.6 | 0.242256461 | 0.809272444 | 1.2820359 | up | lncRNA |
| ENST00000504349 | RP11-155L15.1 | 0.707395936 | 0.981594335 | 1.0404596 | up | lncRNA |
| ENST00000504382 | CTD-2227C6.3 | 0.442472734 | 0.90512482 | 1.0784794 | up | lncRNA |
| ENST00000504402 | RP11-367J11.3 | 0.634097016 | 0.964757805 | 1.0633562 | up | lncRNA |
| ENST00000504429 | RP11-471J12.1 | 0.543208442 | 0.939697392 | 1.1339993 | up | lncRNA |
| ENST00000504469 | RP11-159F24.3 | 0.56000323 | 0.943393901 | 1.0555923 | up | lncRNA |
| ENST00000504506 | CTC-564N23.3 | 0.814395844 | 0.99257857 | 1.0145111 | up | lncRNA |
| ENST00000504512 | RP11-267A15.1 | 0.588659053 | 0.950589384 | 1.3065693 | up | lncRNA |
| ENST00000504539 | RP11-256P1.1 | 0.945167701 | 0.998875101 | 1.0154442 | up | lncRNA |
| ENST00000504578 | RP11-155G15.2 | 0.344308984 | 0.864204712 | 1.1772866 | up | lncRNA |
| ENST00000504610 | RP11-356J5.12 | 0.06361873 | 0.537558012 | 1.4429375 | up | lncRNA |
| ENST00000504628 | RP11-563E2.2 | 0.875881366 | 0.998411428 | 1.0247642 | up | lncRNA |
| ENST00000504670 | RP11-319G6.1 | 0.599045718 | 0.953054445 | 1.152393 | up | lncRNA |
| ENST00000504712 | GTF2H2B | 0.757266903 | 0.98777048 | 1.0291108 | up | lncRNA |
| ENST00000504737 | RP11-39E3.3 | 0.202222118 | 0.769430499 | 2.0170621 | up | lncRNA |
| ENST00000504748 | AC078852.1 | 0.002210901 | 0.143044381 | 1.1662667 | up | lncRNA |
| ENST00000504773 | RP11-510N19.5 | 0.491782415 | 0.920777644 | 1.1260325 | up | lncRNA |
| ENST00000504808 | RP11-77P16.4 | 0.548586188 | 0.941220439 | 1.1956841 | up | lncRNA |
| ENST00000504817 | RP11-5N11.3 | 0.476363769 | 0.917441334 | 1.0761074 | up | lncRNA |
| ENST00000504829 | CTC-441N14.2 | 0.051892611 | 0.513193572 | 2.9710933 | up | lncRNA |
| ENST00000504906 | AP000892.4 | 0.60256466 | 0.954842443 | 1.1315349 | up | lncRNA |
| ENST00000504916 | RP11-78C3.1 | 0.584183637 | 0.949470507 | 1.1328557 | up | lncRNA |
| ENST00000504932 | RP11-677M14.3 | 0.013757786 | 0.294694748 | 1.6023708 | up | lncRNA |
| ENST00000504955 | RP11-45L9.1 | 0.688033617 | 0.97861545 | 1.0661862 | up | lncRNA |
| ENST00000504998 | RP11-321E2.4 | 0.18314574 | 0.750893267 | 1.1427873 | up | lncRNA |
| ENST00000505151 | CTC-338M12.4 | 0.809711192 | 0.992578294 | 1.0229737 | up | lncRNA |
| ENST00000505175 | RP11-100N20.1 | 0.421146731 | 0.895572338 | 1.4141308 | up | lncRNA |
| ENST00000505196 | RP11-308B16.1 | 0.645081474 | 0.968026904 | 1.2101762 | up | lncRNA |
| ENST00000505200 | CTD-2503O16.4 | 0.454003486 | 0.909908955 | 1.1798909 | up | lncRNA |
| ENST00000505209 | CTC-441N14.1 | 0.64259777 | 0.967175883 | 1.0914288 | up | lncRNA |
| ENST00000505267 | EGFLAM-AS3 | 0.604668925 | 0.955931955 | 1.0844619 | up | lncRNA |
| ENST00000505276 | RP5-1063M23.1 | 0.417655793 | 0.893189679 | 1.1233935 | up | lncRNA |
| ENST00000505298 | RP11-722M1.1 | 0.147675993 | 0.703667281 | 1.3851687 | up | lncRNA |
| ENST00000505302 | RP11-53O19.1 | 0.488152272 | 0.920777644 | 1.1351745 | up | lncRNA |
| ENST00000505362 | CTC-463N11.3 | 0.923908518 | 0.998875101 | 1.023526 | up | lncRNA |
| ENST00000505371 | RP11-141O11.2 | 0.540094025 | 0.937635108 | 1.134986 | up | lncRNA |
| ENST00000505532 | RP11-597D13.9 | 0.223538588 | 0.792741738 | 1.094256 | up | lncRNA |
| ENST00000505546 | CTC-210G5.1 | 0.468073931 | 0.914494873 | 1.1927062 | up | lncRNA |
| ENST00000505637 | RP11-53O19.1 | 0.965510427 | 0.998875101 | 1.0036617 | up | lncRNA |
| ENST00000505677 | CTC-463N11.3 | 0.799825602 | 0.992228977 | 1.092962 | up | lncRNA |
| ENST00000505712 | RP11-61G23.1 | 0.670688416 | 0.974341942 | 1.1288616 | up | lncRNA |
| ENST00000505718 | ARHGAP22-IT1 | 0.394472429 | 0.885711362 | 1.145585 | up | lncRNA |
| ENST00000505731 | NOP14-AS1 | 0.268406897 | 0.830054406 | 1.1258932 | up | lncRNA |
| ENST00000505736 | RP11-138I17.1 | 0.261238344 | 0.82544125 | 1.190377 | up | lncRNA |
| ENST00000505776 | ZFAT-AS1 | 0.026427175 | 0.385842137 | 1.3368197 | up | lncRNA |
| ENST00000505817 | RP11-471J12.1 | 0.798730836 | 0.992228977 | 1.0448087 | up | lncRNA |
| ENST00000505873 | AC108142.1 | 0.333628964 | 0.857954289 | 1.2197293 | up | lncRNA |
| ENST00000505930 | RP11-542G1.1 | 0.4269649 | 0.897780553 | 1.1353813 | up | lncRNA |
| ENST00000505955 | RP11-79P5.8 | 0.117831978 | 0.66559915 | 1.7362932 | up | lncRNA |
| ENST00000505978 | RP11-1018N14.3 | 0.357712549 | 0.872424004 | 1.2013244 | up | lncRNA |
| ENST00000506005 | CTD-3028N15.1 | 0.826953324 | 0.993856674 | 1.0486167 | up | lncRNA |
| ENST00000506053 | CTC-210G5.1 | 0.314636827 | 0.851147568 | 1.1278879 | up | lncRNA |
| ENST00000506086 | RP11-229C3.2 | 0.099791839 | 0.626240697 | 2.837045 | up | lncRNA |
| ENST00000506090 | RP11-97O12.2 | 0.443238651 | 0.90512482 | 1.1292713 | up | lncRNA |
| ENST00000506106 | CTD-2023N9.1 | 0.586208603 | 0.950544104 | 1.0906092 | up | lncRNA |
| ENST00000506250 | CTC-347C20.2 | 0.848409845 | 0.995876116 | 1.0173828 | up | lncRNA |
| ENST00000506379 | RP11-16N2.1 | 0.337229156 | 0.859588936 | 1.1898596 | up | lncRNA |
| ENST00000506425 | RP11-412P11.1 | 0.197098215 | 0.763327268 | 1.4228137 | up | lncRNA |
| ENST00000506435 | RP11-506H20.1 | 0.973707815 | 0.998875101 | 1.0109531 | up | lncRNA |
| ENST00000506454 | RP11-696N14.1 | 0.640563521 | 0.966298892 | 1.1439507 | up | lncRNA |
| ENST00000506463 | RP11-122C5.1 | 0.737975168 | 0.984316636 | 1.0510125 | up | lncRNA |
| ENST00000506485 | AC010226.4 | 0.07900838 | 0.577356551 | 1.1982604 | up | lncRNA |
| ENST00000506514 | RP13-497K6.1 | 0.049065604 | 0.501361686 | 1.3514292 | up | lncRNA |
| ENST00000506562 | CTD-2215E18.2 | 0.063897305 | 0.537558012 | 1.409462 | up | lncRNA |
| ENST00000506593 | RPL32P3 | 0.360311035 | 0.873342057 | 1.0772074 | up | lncRNA |
| ENST00000506634 | HSP90AA6P | 0.788290181 | 0.991420185 | 1.030457 | up | lncRNA |
| ENST00000506645 | RP11-362F19.1 | 0.033434698 | 0.42757611 | 1.5937887 | up | lncRNA |
| ENST00000506655 | RP11-143A12.3 | 0.312043865 | 0.849269161 | 1.4531075 | up | lncRNA |
| ENST00000506656 | RP11-357F12.1 | 0.399205386 | 0.888157273 | 1.364023 | up | lncRNA |
| ENST00000506718 | U66061.31 | 0.709345448 | 0.981727542 | 1.0698637 | up | lncRNA |
| ENST00000507060 | CTD-2269F5.1 | 0.008260816 | 0.24294923 | 1.2346822 | up | lncRNA |
| ENST00000507072 | RP11-423H2.3 | 0.142384014 | 0.698941469 | 1.2541612 | up | lncRNA |
| ENST00000507161 | RP11-94C24.3 | 0.291268961 | 0.837264328 | 1.1565559 | up | lncRNA |
| ENST00000507238 | RPL32P3 | 0.12177203 | 0.672928817 | 1.1373539 | up | lncRNA |
| ENST00000507269 | CTC-551A13.2 | 0.021050596 | 0.359097712 | 3.5083188 | up | lncRNA |
| ENST00000507296 | RP11-364P22.2 | 0.660890846 | 0.971704562 | 1.0853159 | up | lncRNA |
| ENST00000507299 | CTD-2325B11.1 | 0.677362935 | 0.976096237 | 1.0439947 | up | lncRNA |
| ENST00000507304 | CTB-174D11.1 | 0.96565864 | 0.998875101 | 1.0087384 | up | lncRNA |
| ENST00000507337 | RP11-1079K10.3 | 0.589794809 | 0.950589384 | 1.0666028 | up | lncRNA |
| ENST00000507344 | RP11-51P8.1 | 0.638290424 | 0.96564589 | 1.0283212 | up | lncRNA |
| ENST00000507361 | RP11-267A15.1 | 0.999437737 | 0.999703145 | 1.0005517 | up | lncRNA |
| ENST00000507391 | CTB-1H10.1 | 0.61223247 | 0.957725078 | 1.0886494 | up | lncRNA |
| ENST00000507486 | RP11-284M14.1 | 0.541243267 | 0.938154996 | 1.1491575 | up | lncRNA |
| ENST00000507509 | CTC-228N24.2 | 0.715969891 | 0.98251396 | 1.1190049 | up | lncRNA |
| ENST00000507514 | CTD-2384B11.2 | 0.186995742 | 0.753717042 | 1.2031037 | up | lncRNA |
| ENST00000507521 | CTC-329D1.2 | 0.091551678 | 0.608086968 | 1.5595028 | up | lncRNA |
| ENST00000507558 | CTB-118N6.3 | 0.157312673 | 0.713946155 | 1.1361242 | up | lncRNA |
| ENST00000507565 | RP11-159K7.2 | 0.177016134 | 0.741545698 | 1.0551582 | up | lncRNA |
| ENST00000507628 | CTD-2195M18.1 | 0.300292778 | 0.8422517 | 1.2078299 | up | lncRNA |
| ENST00000507653 | CTC-493L21.2 | 0.373301597 | 0.878706238 | 1.2420588 | up | lncRNA |
| ENST00000507666 | RP11-380P13.2 | 0.195534758 | 0.761823233 | 1.2572186 | up | lncRNA |
| ENST00000507733 | CTC-537E7.3 | 0.341388437 | 0.862529336 | 1.1878004 | up | lncRNA |
| ENST00000507770 | RASA2-IT1 | 0.003806383 | 0.17862549 | 7.4684174 | up | lncRNA |
| ENST00000507776 | RP11-696F12.1 | 0.260302923 | 0.824565516 | 1.0593915 | up | lncRNA |
| ENST00000507856 | RP11-77P16.4 | 0.736167655 | 0.984025164 | 1.1061309 | up | lncRNA |
| ENST00000507869 | AC108142.1 | 0.643450968 | 0.967175883 | 1.1057858 | up | lncRNA |
| ENST00000507932 | AC006296.1 | 0.193688175 | 0.760765035 | 1.4115311 | up | lncRNA |
| ENST00000507983 | APOC1P1 | 0.600494878 | 0.953841565 | 1.0429787 | up | lncRNA |
| ENST00000507997 | RP11-254I22.1 | 0.100338444 | 0.628381708 | 1.2085962 | up | lncRNA |
| ENST00000508056 | RP11-844P9.2 | 0.931355948 | 0.998875101 | 1.0109902 | up | lncRNA |
| ENST00000508135 | RP11-4O3.2 | 0.714672267 | 0.98251396 | 1.1641315 | up | lncRNA |
| ENST00000508174 | RP11-234K19.2 | 0.210869928 | 0.778485565 | 1.0755366 | up | lncRNA |
| ENST00000508241 | RP11-231C18.2 | 0.442703091 | 0.90512482 | 1.0433911 | up | lncRNA |
| ENST00000508246 | TMEM198B | 0.413928739 | 0.890927603 | 1.0630731 | up | lncRNA |
| ENST00000508263 | RP11-158I23.1 | 0.275888384 | 0.832297359 | 1.2353835 | up | lncRNA |
| ENST00000508281 | RP11-381K20.2 | 0.846399981 | 0.995583664 | 1.0435014 | up | lncRNA |
| ENST00000508309 | CTC-205M6.2 | 0.967996258 | 0.998875101 | 1.0029122 | up | lncRNA |
| ENST00000508328 | RP11-12A1.1 | 0.85146181 | 0.995900317 | 1.033875 | up | lncRNA |
| ENST00000508352 | RP11-1E6.1 | 0.779386891 | 0.99121281 | 1.096148 | up | lncRNA |
| ENST00000508401 | CTC-564N23.3 | 0.920138783 | 0.998875101 | 1.0147408 | up | lncRNA |
| ENST00000508424 | CTB-118N6.3 | 0.40318158 | 0.889146721 | 1.3637416 | up | lncRNA |
| ENST00000508517 | AC010226.4 | 0.120871514 | 0.671500573 | 1.5305575 | up | lncRNA |
| ENST00000508534 | RP11-471J12.1 | 0.571000942 | 0.944998486 | 1.1316395 | up | lncRNA |
| ENST00000508547 | CTB-174D11.1 | 0.405561819 | 0.889198786 | 1.0462892 | up | lncRNA |
| ENST00000508688 | HOXB-AS1 | 0.975425426 | 0.998875101 | 1.0380649 | up | lncRNA |
| ENST00000508713 | CTB-131B5.2 | 0.499852348 | 0.922144236 | 1.1702339 | up | lncRNA |
| ENST00000508752 | RP11-597D13.9 | 0.386238304 | 0.883674819 | 1.2019559 | up | lncRNA |
| ENST00000508825 | RP11-12K22.1 | 0.150386351 | 0.705518742 | 1.6534018 | up | lncRNA |
| ENST00000508827 | RP11-47I22.2 | 0.081496284 | 0.584367002 | 1.7124021 | up | lncRNA |
| ENST00000508877 | CTC-338M12.5 | 0.702199348 | 0.980170937 | 1.0506798 | up | lncRNA |
| ENST00000508913 | AC025171.1 | 0.867963833 | 0.99713777 | 1.0216726 | up | lncRNA |
| ENST00000508945 | RP11-53O19.1 | 0.183860671 | 0.751257244 | 1.644702 | up | lncRNA |
| ENST00000508985 | RP11-635L1.3 | 0.200254062 | 0.767707124 | 1.2352303 | up | lncRNA |
| ENST00000508986 | EGFLAM-AS1 | 0.523487646 | 0.932117592 | 1.081203 | up | lncRNA |
| ENST00000509010 | RP11-395P13.6 | 0.633054364 | 0.964607725 | 1.1569081 | up | lncRNA |
| ENST00000509057 | CTD-2247C11.1 | 0.462166242 | 0.912913065 | 1.1647933 | up | lncRNA |
| ENST00000509191 | RP11-68L1.2 | 0.129250937 | 0.682265946 | 1.1808775 | up | lncRNA |
| ENST00000509252 | CTC-338M12.3 | 0.768961531 | 0.989189555 | 1.0267378 | up | lncRNA |
| ENST00000509260 | RP11-94C24.8 | 0.034588021 | 0.429619286 | 1.323806 | up | lncRNA |
| ENST00000509322 | RP11-778J15.1 | 0.607402253 | 0.956065104 | 1.0433504 | up | lncRNA |
| ENST00000509406 | CTD-2269F5.1 | 0.939384271 | 0.998875101 | 1.0121808 | up | lncRNA |
| ENST00000509456 | RP11-284A20.1 | 0.705469706 | 0.980849889 | 1.0788033 | up | lncRNA |
| ENST00000509458 | RP11-252I13.2 | 0.536685296 | 0.936418354 | 1.0493525 | up | lncRNA |
| ENST00000509470 | RP11-768F21.1 | 0.691537807 | 0.978695455 | 1.1270597 | up | lncRNA |
| ENST00000509629 | RP11-5N11.6 | 0.23576181 | 0.805225869 | 1.1932137 | up | lncRNA |
| ENST00000509655 | CTB-109A12.1 | 0.495935652 | 0.921717611 | 1.0442186 | up | lncRNA |
| ENST00000509713 | RP11-553P9.2 | 0.282437489 | 0.832629955 | 1.4295513 | up | lncRNA |
| ENST00000509733 | AC004507.1 | 0.960403919 | 0.998875101 | 1.0139303 | up | lncRNA |
| ENST00000509782 | RP11-340A13.2 | 0.449605996 | 0.907426685 | 1.2225207 | up | lncRNA |
| ENST00000509783 | LINC00461 | 0.271755907 | 0.832297359 | 1.0797616 | up | lncRNA |
| ENST00000509824 | RP11-577G20.1 | 0.94506084 | 0.998875101 | 1.0073679 | up | lncRNA |
| ENST00000509919 | RP11-90D4.1 | 0.833544433 | 0.994267761 | 1.1127553 | up | lncRNA |
| ENST00000509921 | CTC-205M6.2 | 0.409700131 | 0.890549744 | 1.0864459 | up | lncRNA |
| ENST00000509938 | RP11-148B6.1 | 0.587529666 | 0.950589384 | 1.040531 | up | lncRNA |
| ENST00000509939 | RP11-696N14.1 | 0.607014521 | 0.956038292 | 1.0910021 | up | lncRNA |
| ENST00000509943 | RP11-893F2.5 | 0.994615745 | 0.999156445 | 1.0006198 | up | lncRNA |
| ENST00000509956 | RP11-6E9.5 | 0.084830204 | 0.590846042 | 2.216415 | up | lncRNA |
| ENST00000509968 | RP11-161D15.1 | 0.963809127 | 0.998875101 | 1.0072079 | up | lncRNA |
| ENST00000509993 | CTC-210G5.1 | 0.630441054 | 0.964371833 | 1.3378374 | up | lncRNA |
| ENST00000509999 | AC008592.4 | 0.184541991 | 0.751257244 | 1.2477803 | up | lncRNA |
| ENST00000510017 | WI2-2373I1.2 | 0.33141791 | 0.856326799 | 1.2481487 | up | lncRNA |
| ENST00000510137 | CTD-2263F21.1 | 0.591224984 | 0.950912706 | 1.1441188 | up | lncRNA |
| ENST00000510145 | LINC00491 | 0.247779415 | 0.814489937 | 1.0547098 | up | lncRNA |
| ENST00000510150 | CTD-2532K18.2 | 0.323140982 | 0.853463264 | 1.1699142 | up | lncRNA |
| ENST00000510198 | CTD-2176I21.2 | 0.000124279 | 0.052460176 | 1.4730872 | up | lncRNA |
| ENST00000510200 | RP11-328K4.1 | 0.971943016 | 0.998875101 | 1.0071192 | up | lncRNA |
| ENST00000510240 | CTC-563A5.2 | 0.855121932 | 0.996142678 | 1.0746007 | up | lncRNA |
| ENST00000510261 | CTD-2116N20.1 | 0.96869217 | 0.998875101 | 1.0185725 | up | lncRNA |
| ENST00000510360 | RP11-1079K10.3 | 0.804191866 | 0.992578294 | 1.0218183 | up | lncRNA |
| ENST00000510456 | CTD-2350J17.1 | 0.38134953 | 0.88113876 | 1.1459932 | up | lncRNA |
| ENST00000510469 | CTD-2263F21.1 | 0.120118888 | 0.6694937 | 2.0737187 | up | lncRNA |
| ENST00000510550 | RP11-90J7.3 | 0.221024973 | 0.790618275 | 1.2377996 | up | lncRNA |
| ENST00000510619 | RP1-68D18.2 | 0.000686599 | 0.094750704 | 6.0257424 | up | lncRNA |
| ENST00000510667 | RP11-438E5.1 | 0.23170996 | 0.79921005 | 1.2123026 | up | lncRNA |
| ENST00000510758 | RP11-473L15.3 | 0.62318561 | 0.960579946 | 1.3036619 | up | lncRNA |
| ENST00000510844 | RP11-33B1.1 | 0.841129526 | 0.995583664 | 1.1302724 | up | lncRNA |
| ENST00000510853 | CTC-369A16.3 | 0.412135747 | 0.890899058 | 1.1213297 | up | lncRNA |
| ENST00000510859 | AC016683.6 | 0.292057743 | 0.837264328 | 1.547352 | up | lncRNA |
| ENST00000511053 | KHDC1P1 | 0.227284554 | 0.796967485 | 1.0435985 | up | lncRNA |
| ENST00000511164 | RAC1P2 | 0.998850354 | 0.999477117 | 1.0001669 | up | lncRNA |
| ENST00000511241 | RP11-438E5.1 | 0.329531722 | 0.85540421 | 1.158599 | up | lncRNA |
| ENST00000511256 | CTC-276P9.1 | 0.702055807 | 0.980170937 | 1.0772494 | up | lncRNA |
| ENST00000511329 | CTC-366B18.2 | 0.844436506 | 0.995583664 | 1.0599784 | up | lncRNA |
| ENST00000511330 | RP11-300M6.1 | 0.62902139 | 0.963660225 | 1.0172196 | up | lncRNA |
| ENST00000511361 | RP5-875H18.4 | 0.651932156 | 0.969927925 | 1.0595675 | up | lncRNA |
| ENST00000511418 | RP11-492L8.2 | 0.374791799 | 0.879312727 | 1.7858225 | up | lncRNA |
| ENST00000511422 | CTD-2334D19.1 | 0.194047248 | 0.760765035 | 1.3487993 | up | lncRNA |
| ENST00000511468 | RP11-346J10.1 | 0.406989288 | 0.889505404 | 1.2612495 | up | lncRNA |
| ENST00000511484 | CTC-458I2.2 | 0.575920618 | 0.947022539 | 1.1917037 | up | lncRNA |
| ENST00000511514 | CTD-2247C11.4 | 0.722623969 | 0.98251396 | 1.0780431 | up | lncRNA |
| ENST00000511537 | HNRNPKP3 | 0.173382658 | 0.735339215 | 1.2852333 | up | lncRNA |
| ENST00000511565 | RP11-1334A24.4 | 0.949469143 | 0.998875101 | 1.0115985 | up | lncRNA |
| ENST00000511596 | RP11-454P21.1 | 0.582340506 | 0.94928838 | 1.0545452 | up | lncRNA |
| ENST00000511627 | RP1-117B12.4 | 0.055558902 | 0.520364745 | 1.3511146 | up | lncRNA |
| ENST00000511631 | RP11-395P13.4 | 0.291107858 | 0.837264328 | 1.404529 | up | lncRNA |
| ENST00000511693 | RP11-259O2.1 | 0.637195369 | 0.96564589 | 1.0761787 | up | lncRNA |
| ENST00000511699 | RP11-933H2.4 | 0.914090455 | 0.998875101 | 1.0208994 | up | lncRNA |
| ENST00000511707 | RP11-267A15.1 | 0.333871943 | 0.85798034 | 1.3003843 | up | lncRNA |
| ENST00000511712 | RP11-53O19.2 | 0.099233705 | 0.624838831 | 1.1316425 | up | lncRNA |
| ENST00000511821 | CTD-2139B15.2 | 0.259374789 | 0.824094195 | 1.0831589 | up | lncRNA |
| ENST00000511846 | WWC2-AS1 | 0.643751676 | 0.967175883 | 1.143661 | up | lncRNA |
| ENST00000511867 | RP11-1094H24.3 | 0.894324927 | 0.998875101 | 1.017324 | up | lncRNA |
| ENST00000511897 | RP11-87E22.2 | 0.704319394 | 0.980849889 | 1.1059019 | up | lncRNA |
| ENST00000511918 | RP11-213H15.3 | 0.102350826 | 0.632351488 | 3.4010618 | up | lncRNA |
| ENST00000511927 | RP11-504G3.1 | 0.167097247 | 0.723214624 | 1.0562877 | up | lncRNA |
| ENST00000511929 | RP11-1149O23.4 | 0.84913972 | 0.995876116 | 1.1165946 | up | lncRNA |
| ENST00000511962 | RP11-319E12.2 | 0.367529808 | 0.87558047 | 1.2429921 | up | lncRNA |
| ENST00000511991 | RP11-159F24.2 | 0.163730064 | 0.718769923 | 1.0619118 | up | lncRNA |
| ENST00000512035 | CTD-2231H16.1 | 0.744390538 | 0.985094857 | 1.0310111 | up | lncRNA |
| ENST00000512039 | RP11-138I17.1 | 0.378484965 | 0.880076824 | 1.307255 | up | lncRNA |
| ENST00000512092 | FAM86EP | 0.506622064 | 0.92530413 | 1.1639752 | up | lncRNA |
| ENST00000512129 | LEF1-AS1 | 0.376793058 | 0.879423449 | 1.3496049 | up | lncRNA |
| ENST00000512130 | RP11-438E5.1 | 0.669859832 | 0.974341942 | 1.0735634 | up | lncRNA |
| ENST00000512155 | CTD-2297D10.2 | 0.784224325 | 0.99121281 | 1.0262763 | up | lncRNA |
| ENST00000512213 | CTC-564N23.3 | 0.293852044 | 0.838742488 | 1.2589243 | up | lncRNA |
| ENST00000512287 | CTC-281B15.1 | 0.501670529 | 0.922837148 | 1.1553158 | up | lncRNA |
| ENST00000512295 | RP11-23P13.4 | 0.180012554 | 0.745729299 | 1.2659757 | up | lncRNA |
| ENST00000512417 | CTB-174D11.1 | 0.228501561 | 0.798130144 | 1.5435556 | up | lncRNA |
| ENST00000512427 | HOXC-AS1 | 0.85248318 | 0.995900317 | 1.0400094 | up | lncRNA |
| ENST00000512498 | RP11-159F24.6 | 0.225600934 | 0.794953393 | 1.2014699 | up | lncRNA |
| ENST00000512500 | RP11-517I3.1 | 0.773417622 | 0.990435061 | 1.0516622 | up | lncRNA |
| ENST00000512508 | CTC-338M12.6 | 0.009084554 | 0.252136627 | 1.7791207 | up | lncRNA |
| ENST00000512519 | CTD-2127H9.1 | 0.087305997 | 0.597891607 | 2.8457877 | up | lncRNA |
| ENST00000512573 | CTC-347C20.2 | 0.196479641 | 0.762747039 | 1.1271562 | up | lncRNA |
| ENST00000512622 | RP11-319G6.1 | 0.620767816 | 0.960359997 | 1.1055006 | up | lncRNA |
| ENST00000512650 | CTC-564N23.2 | 0.106601687 | 0.638286705 | 1.3119837 | up | lncRNA |
| ENST00000512678 | RP11-328N19.1 | 0.667019539 | 0.973169099 | 1.0440273 | up | lncRNA |
| ENST00000512688 | RP11-774D14.1 | 0.301033401 | 0.842645237 | 1.2243804 | up | lncRNA |
| ENST00000512693 | CTD-2587M2.1 | 0.99620623 | 0.999156445 | 1.0012779 | up | lncRNA |
| ENST00000512765 | LINC00265 | 0.342901805 | 0.863514929 | 1.1633411 | up | lncRNA |
| ENST00000512835 | ANP32C | 0.852320193 | 0.995900317 | 1.0336088 | up | lncRNA |
| ENST00000512839 | RP11-219G10.3 | 0.670375037 | 0.974341942 | 1.7267921 | up | lncRNA |
| ENST00000512856 | CTD-2260A17.1 | 0.199891946 | 0.767353839 | 1.1488499 | up | lncRNA |
| ENST00000512882 | CTD-2170G1.2 | 0.306003714 | 0.846407029 | 1.5183036 | up | lncRNA |
| ENST00000512916 | HOXC-AS5 | 0.430597945 | 0.898769881 | 1.0943378 | up | lncRNA |
| ENST00000512966 | AC104650.2 | 0.528438216 | 0.934238148 | 1.1068697 | up | lncRNA |
| ENST00000513067 | RP11-597D13.8 | 0.756349707 | 0.98762882 | 1.1952181 | up | lncRNA |
| ENST00000513071 | RP11-286E11.1 | 0.114754505 | 0.657011458 | 1.3300431 | up | lncRNA |
| ENST00000513127 | RP11-632F7.3 | 0.411849307 | 0.890876411 | 1.0862674 | up | lncRNA |
| ENST00000513153 | RP11-438E5.1 | 0.312631884 | 0.850016827 | 1.235335 | up | lncRNA |
| ENST00000513179 | RP11-539L10.3 | 0.808997596 | 0.992578294 | 1.1446216 | up | lncRNA |
| ENST00000513283 | CTC-305H11.1 | 0.268819743 | 0.83059224 | 1.5338676 | up | lncRNA |
| ENST00000513313 | RP11-219G10.3 | 0.689910667 | 0.97861545 | 1.4019017 | up | lncRNA |
| ENST00000513379 | RP11-60A8.1 | 0.929105951 | 0.998875101 | 1.0099198 | up | lncRNA |
| ENST00000513381 | RP11-834C11.5 | 0.719095228 | 0.98251396 | 1.0349627 | up | lncRNA |
| ENST00000513406 | CTC-564N23.3 | 0.22991737 | 0.79852974 | 1.2168466 | up | lncRNA |
| ENST00000513466 | FAM86HP | 0.312715221 | 0.850016827 | 1.0751749 | up | lncRNA |
| ENST00000513480 | CTD-2127H9.1 | 0.009504371 | 0.25401626 | 1.7404023 | up | lncRNA |
| ENST00000513492 | RP11-213H15.3 | 0.01920078 | 0.345325309 | 4.2972749 | up | lncRNA |
| ENST00000513533 | HOXC-AS2 | 0.614409135 | 0.958423841 | 1.0713479 | up | lncRNA |
| ENST00000513542 | RP11-301H24.4 | 0.003103606 | 0.162565512 | 1.5956849 | up | lncRNA |
| ENST00000513573 | RP11-420O16.1 | 0.511698468 | 0.928079921 | 1.1800817 | up | lncRNA |
| ENST00000513626 | RP11-213H15.3 | 0.266149526 | 0.828881561 | 1.0963271 | up | lncRNA |
| ENST00000513638 | GBP1P1 | 0.000203794 | 0.069939941 | 13.8939519 | up | lncRNA |
| ENST00000513644 | RP11-974F13.6 | 0.423812943 | 0.897000937 | 1.1599202 | up | lncRNA |
| ENST00000513645 | RP11-650J17.2 | 0.416448672 | 0.892649611 | 1.1218967 | up | lncRNA |
| ENST00000513694 | TMEM161B-AS1 | 0.60585779 | 0.956038292 | 1.212523 | up | lncRNA |
| ENST00000513704 | CTC-454M9.1 | 0.355829092 | 0.870832491 | 1.2038669 | up | lncRNA |
| ENST00000513752 | AC108142.1 | 0.693364081 | 0.978978277 | 1.1174655 | up | lncRNA |
| ENST00000513758 | APOBEC3B-AS1 | 0.387822787 | 0.884936606 | 1.0742156 | up | lncRNA |
| ENST00000513764 | AC093484.6 | 0.078532986 | 0.57678507 | 1.458382 | up | lncRNA |
| ENST00000513771 | CTC-338M12.2 | 0.014180004 | 0.301052394 | 1.7708054 | up | lncRNA |
| ENST00000513843 | RP11-548L20.1 | 0.486740646 | 0.920642653 | 1.5121529 | up | lncRNA |
| ENST00000513844 | CTD-2195M18.1 | 0.037671589 | 0.443529657 | 1.3889532 | up | lncRNA |
| ENST00000513893 | LINC00461 | 0.274518066 | 0.832297359 | 1.1697988 | up | lncRNA |
| ENST00000513915 | RP11-257I8.2 | 0.252205398 | 0.818740529 | 1.5168742 | up | lncRNA |
| ENST00000513940 | RP11-265F19.1 | 0.064491381 | 0.539696965 | 1.1523779 | up | lncRNA |
| ENST00000514072 | AC016724.8 | 0.274549846 | 0.832297359 | 1.2214901 | up | lncRNA |
| ENST00000514136 | RP11-159F24.2 | 0.605379878 | 0.956038292 | 1.0385639 | up | lncRNA |
| ENST00000514201 | CTC-325J23.2 | 0.220811267 | 0.790478735 | 1.0838149 | up | lncRNA |
| ENST00000514241 | MAST4-IT1 | 0.253908671 | 0.820003881 | 1.3595187 | up | lncRNA |
| ENST00000514425 | RP11-534L6.5 | 0.794703905 | 0.991420185 | 1.1087027 | up | lncRNA |
| ENST00000514427 | EMCN-IT2 | 0.318834908 | 0.851223284 | 1.11207 | up | lncRNA |
| ENST00000514459 | TRPC7-AS1 | 0.087082113 | 0.597891607 | 1.2638028 | up | lncRNA |
| ENST00000514544 | CTC-428G20.2 | 0.639119838 | 0.96564589 | 1.0845434 | up | lncRNA |
| ENST00000514573 | CTC-228N24.3 | 0.582111218 | 0.949239022 | 1.0286377 | up | lncRNA |
| ENST00000514597 | RP11-53O19.1 | 0.52145758 | 0.932117592 | 1.0813358 | up | lncRNA |
| ENST00000514729 | RP11-319G6.1 | 0.32813344 | 0.854893129 | 1.0885588 | up | lncRNA |
| ENST00000514791 | RP11-434D9.2 | 0.762206072 | 0.98854315 | 1.1077971 | up | lncRNA |
| ENST00000514793 | RP11-1379J22.5 | 0.102409678 | 0.632351488 | 1.2009411 | up | lncRNA |
| ENST00000514843 | RP11-76G10.1 | 0.304345683 | 0.845358864 | 1.0834463 | up | lncRNA |
| ENST00000514966 | RP11-706F1.1 | 0.513434094 | 0.92902114 | 1.192351 | up | lncRNA |
| ENST00000514980 | RP11-582J16.4 | 0.309237088 | 0.847852517 | 1.382436 | up | lncRNA |
| ENST00000515003 | CTC-463N11.3 | 0.635393126 | 0.965194977 | 1.1469564 | up | lncRNA |
| ENST00000515019 | RP11-481K16.2 | 0.094803281 | 0.616146437 | 1.1732906 | up | lncRNA |
| ENST00000515092 | RP11-43D2.2 | 0.150618968 | 0.705518742 | 1.1911565 | up | lncRNA |
| ENST00000515097 | RP11-680H20.2 | 0.000682801 | 0.094750704 | 1.7120047 | up | lncRNA |
| ENST00000515199 | CTC-537E7.2 | 0.817109892 | 0.992651327 | 1.0373759 | up | lncRNA |
| ENST00000515205 | RP11-539L10.3 | 0.968514134 | 0.998875101 | 1.0196527 | up | lncRNA |
| ENST00000515245 | FAM86HP | 0.782735251 | 0.99121281 | 1.0138207 | up | lncRNA |
| ENST00000515247 | RP11-319G6.1 | 0.809059774 | 0.992578294 | 1.0950145 | up | lncRNA |
| ENST00000515288 | AC005592.2 | 0.506451181 | 0.925291133 | 1.086734 | up | lncRNA |
| ENST00000515319 | RP11-325D5.3 | 0.615883166 | 0.959051918 | 1.0324348 | up | lncRNA |
| ENST00000515464 | MYLK-AS2 | 0.39948602 | 0.888347161 | 1.3667703 | up | lncRNA |
| ENST00000515466 | RP11-159F24.1 | 0.817904952 | 0.992651327 | 1.0211801 | up | lncRNA |
| ENST00000515487 | STX18-IT1 | 0.901426832 | 0.998875101 | 1.0259588 | up | lncRNA |
| ENST00000515513 | RP11-267A15.1 | 0.767778007 | 0.989189555 | 1.0868831 | up | lncRNA |
| ENST00000515563 | CTC-459M5.2 | 0.478809706 | 0.918086425 | 1.1596373 | up | lncRNA |
| ENST00000515570 | AC010226.4 | 0.045572113 | 0.482418324 | 1.8997902 | up | lncRNA |
| ENST00000515608 | CTD-2541M15.1 | 0.022105352 | 0.361714622 | 1.3783568 | up | lncRNA |
| ENST00000515643 | RP11-274N19.2 | 0.995234506 | 0.999156445 | 1.0024999 | up | lncRNA |
| ENST00000515688 | CTD-2269F5.1 | 0.167711057 | 0.723906495 | 1.3070265 | up | lncRNA |
| ENST00000515692 | RP11-304F15.4 | 0.28655065 | 0.836569351 | 1.1081867 | up | lncRNA |
| ENST00000515703 | RP11-6L6.3 | 0.896548479 | 0.998875101 | 1.0256832 | up | lncRNA |
| ENST00000515882 | SLIT2-IT1 | 0.004687776 | 0.190726281 | 5.5903763 | up | lncRNA |
| ENST00000517292 | RP11-317N12.1 | 0.871866297 | 0.997909851 | 1.0228826 | up | lncRNA |
| ENST00000517335 | RP11-175K6.1 | 0.889280338 | 0.998875101 | 1.0249452 | up | lncRNA |
| ENST00000517345 | RP11-30J20.1 | 0.976267389 | 0.998875101 | 1.0054391 | up | lncRNA |
| ENST00000517357 | RP11-134O21.1 | 0.371022545 | 0.877177739 | 1.1786561 | up | lncRNA |
| ENST00000517363 | RP11-150O12.1 | 0.86004161 | 0.996142678 | 1.0252672 | up | lncRNA |
| ENST00000517365 | RP11-26J3.1 | 0.776696521 | 0.991030431 | 1.0262189 | up | lncRNA |
| ENST00000517369 | RP11-77B22.2 | 0.28621135 | 0.835918864 | 1.3873153 | up | lncRNA |
| ENST00000517384 | RP11-582J16.3 | 0.04206015 | 0.467219256 | 1.0808082 | up | lncRNA |
| ENST00000517509 | TPT1-AS1 | 0.65538141 | 0.971258623 | 1.0259579 | up | lncRNA |
| ENST00000517565 | RP11-61L23.2 | 0.932887556 | 0.998875101 | 1.0337733 | up | lncRNA |
| ENST00000517573 | RP11-421P23.1 | 0.426233774 | 0.897780553 | 1.2228488 | up | lncRNA |
| ENST00000517632 | CTD-2647L4.1 | 0.62779486 | 0.962703597 | 1.2560149 | up | lncRNA |
| ENST00000517635 | HOXA-AS2 | 0.183406258 | 0.751210293 | 1.295745 | up | lncRNA |
| ENST00000517641 | HOXA-AS2 | 0.205758706 | 0.772818733 | 1.1042364 | up | lncRNA |
| ENST00000517655 | KB-1047C11.2 | 0.502484037 | 0.923357158 | 1.0656835 | up | lncRNA |
| ENST00000517670 | RP11-363E6.3 | 0.581234183 | 0.949239022 | 1.0302525 | up | lncRNA |
| ENST00000517723 | HOXA11-AS | 0.942256879 | 0.998875101 | 1.005552 | up | lncRNA |
| ENST00000517725 | RP11-366K18.3 | 0.703378722 | 0.980562547 | 1.0209876 | up | lncRNA |
| ENST00000517726 | EVX1-AS | 0.049980924 | 0.505173876 | 1.3605071 | up | lncRNA |
| ENST00000517732 | RP11-981G7.2 | 0.382515532 | 0.881294633 | 1.4375136 | up | lncRNA |
| ENST00000517735 | RP11-597M17.1 | 0.493676087 | 0.921717611 | 1.0862608 | up | lncRNA |
| ENST00000517798 | CTD-2547L16.1 | 0.49886683 | 0.921805853 | 1.0461832 | up | lncRNA |
| ENST00000517838 | PVT1 | 0.317577986 | 0.851223284 | 1.0511877 | up | lncRNA |
| ENST00000517854 | RP11-131N11.4 | 0.200743555 | 0.767810338 | 1.1691815 | up | lncRNA |
| ENST00000517864 | RP11-320N21.1 | 0.264655505 | 0.827704469 | 1.1692029 | up | lncRNA |
| ENST00000517897 | RP11-150O12.2 | 0.116789452 | 0.662785634 | 1.2039739 | up | lncRNA |
| ENST00000517908 | RP11-809O17.1 | 0.042400621 | 0.469012344 | 1.3576173 | up | lncRNA |
| ENST00000517915 | RP11-89K10.1 | 0.95237054 | 0.998875101 | 1.0135318 | up | lncRNA |
| ENST00000517934 | RP11-461C13.1 | 0.943348264 | 0.998875101 | 1.0055682 | up | lncRNA |
| ENST00000517950 | CTD-2281E23.3 | 0.24483886 | 0.811577674 | 1.0889122 | up | lncRNA |
| ENST00000518024 | RP11-273G15.2 | 0.109791347 | 0.645245382 | 1.086291 | up | lncRNA |
| ENST00000518046 | HOXA-AS2 | 0.138607353 | 0.697263489 | 1.3667869 | up | lncRNA |
| ENST00000518073 | RP13-582O9.5 | 0.280410062 | 0.832297359 | 1.1724497 | up | lncRNA |
| ENST00000518090 | KB-1460A1.3 | 0.941197684 | 0.998875101 | 1.0117851 | up | lncRNA |
| ENST00000518096 | TUBBP1 | 0.611504566 | 0.957590063 | 1.1448428 | up | lncRNA |
| ENST00000518134 | RP11-54C4.1 | 0.347444371 | 0.866313684 | 1.0770673 | up | lncRNA |
| ENST00000518172 | MIR4454 | 0.707476705 | 0.98160341 | 1.1223427 | up | lncRNA |
| ENST00000518181 | RP11-89M20.2 | 0.295419973 | 0.839966018 | 1.3022529 | up | lncRNA |
| ENST00000518222 | RP11-10H3.1 | 0.611024194 | 0.957448851 | 1.0612168 | up | lncRNA |
| ENST00000518232 | CTD-3064M3.1 | 0.903545642 | 0.998875101 | 1.0202567 | up | lncRNA |
| ENST00000518376 | RP11-1136L8.1 | 0.206500209 | 0.773204016 | 1.2968524 | up | lncRNA |
| ENST00000518416 | RP11-122C21.1 | 0.257145929 | 0.822133742 | 1.278094 | up | lncRNA |
| ENST00000518425 | CTD-2363C16.2 | 0.008447963 | 0.244209562 | 2.0390323 | up | lncRNA |
| ENST00000518428 | RP11-465K16.1 | 0.207549019 | 0.775194643 | 1.1204331 | up | lncRNA |
| ENST00000518436 | RP11-61G23.1 | 0.2553911 | 0.821835543 | 1.2068395 | up | lncRNA |
| ENST00000518524 | CTD-2571E19.1 | 0.996526877 | 0.999156445 | 1.0013648 | up | lncRNA |
| ENST00000518535 | RP11-723D22.2 | 0.025265776 | 0.380009848 | 1.2353103 | up | lncRNA |
| ENST00000518539 | RP11-439C15.2 | 0.051072191 | 0.50923846 | 1.2137408 | up | lncRNA |
| ENST00000518556 | RP11-513O17.2 | 0.195671111 | 0.761823233 | 1.130139 | up | lncRNA |
| ENST00000518570 | RP11-351A11.1 | 0.458004555 | 0.912197894 | 1.2295639 | up | lncRNA |
| ENST00000518612 | KB-1930G5.3 | 0.241565202 | 0.809272444 | 1.2847668 | up | lncRNA |
| ENST00000518631 | RP11-586K2.1 | 0.794628391 | 0.991420185 | 1.0375372 | up | lncRNA |
| ENST00000518662 | RP11-17A4.2 | 0.081953638 | 0.585414993 | 1.4871615 | up | lncRNA |
| ENST00000518732 | RP11-706J10.1 | 0.983953634 | 0.998875101 | 1.0122723 | up | lncRNA |
| ENST00000518749 | KB-1107E3.1 | 0.651346455 | 0.969927925 | 1.0494032 | up | lncRNA |
| ENST00000518790 | CTB-131B5.4 | 0.808980714 | 0.992578294 | 1.049556 | up | lncRNA |
| ENST00000518818 | CTC-308K20.1 | 0.624871488 | 0.96133281 | 1.1627871 | up | lncRNA |
| ENST00000518865 | HAS2-AS1 | 0.901117971 | 0.998875101 | 1.0323379 | up | lncRNA |
| ENST00000518886 | RP1-170O19.19 | 0.592085683 | 0.950912706 | 1.0445815 | up | lncRNA |
| ENST00000518894 | CTC-308K20.1 | 0.985702968 | 0.998971647 | 1.0081638 | up | lncRNA |
| ENST00000518905 | CTB-113P19.1 | 0.00812952 | 0.242064044 | 1.4030719 | up | lncRNA |
| ENST00000518943 | RP11-16M8.2 | 0.194527608 | 0.760765035 | 1.1005035 | up | lncRNA |
| ENST00000518973 | RP11-238K6.1 | 0.366010343 | 0.874818743 | 1.1676152 | up | lncRNA |
| ENST00000518994 | RP11-231D20.2 | 0.312919461 | 0.850249924 | 1.4281943 | up | lncRNA |
| ENST00000519013 | RP11-62H7.2 | 0.782894532 | 0.99121281 | 1.0458387 | up | lncRNA |
| ENST00000519034 | RP11-347C18.3 | 0.577480064 | 0.947948855 | 1.0671462 | up | lncRNA |
| ENST00000519038 | RP11-156K13.3 | 0.583338978 | 0.949379205 | 1.136299 | up | lncRNA |
| ENST00000519040 | CTC-367J11.1 | 0.879528286 | 0.998682284 | 1.0707004 | up | lncRNA |
| ENST00000519048 | RP11-26E5.1 | 0.550972096 | 0.942167708 | 1.0509366 | up | lncRNA |
| ENST00000519050 | EVX1-AS | 0.93802278 | 0.998875101 | 1.0080641 | up | lncRNA |
| ENST00000519071 | RP11-1136L8.1 | 0.057509161 | 0.525082084 | 1.5569145 | up | lncRNA |
| ENST00000519134 | RP11-434I12.3 | 0.287045244 | 0.836961572 | 1.2335713 | up | lncRNA |
| ENST00000519144 | RP11-16M8.2 | 0.804463229 | 0.992578294 | 1.0663994 | up | lncRNA |
| ENST00000519233 | RP11-68L18.1 | 0.783040593 | 0.99121281 | 1.0453066 | up | lncRNA |
| ENST00000519281 | RP11-706C16.5 | 0.588166349 | 0.950589384 | 1.0635368 | up | lncRNA |
| ENST00000519319 | PCAT1 | 0.234169811 | 0.803248683 | 1.128721 | up | lncRNA |
| ENST00000519411 | RP11-706C16.5 | 0.09804076 | 0.623706113 | 1.1674476 | up | lncRNA |
| ENST00000519427 | RP11-313C15.1 | 0.174462973 | 0.736164062 | 1.1810522 | up | lncRNA |
| ENST00000519451 | RP11-962G15.1 | 0.624482884 | 0.96133281 | 1.1189322 | up | lncRNA |
| ENST00000519454 | TPT1-AS1 | 0.306923821 | 0.846715728 | 1.0643676 | up | lncRNA |
| ENST00000519481 | PVT1 | 0.19069044 | 0.758743885 | 1.053696 | up | lncRNA |
| ENST00000519499 | CTB-109A12.1 | 0.975563562 | 0.998875101 | 1.0054176 | up | lncRNA |
| ENST00000519500 | AC083843.3 | 0.275771476 | 0.832297359 | 1.3619233 | up | lncRNA |
| ENST00000519557 | RP11-744J10.3 | 0.805640629 | 0.992578294 | 1.0624597 | up | lncRNA |
| ENST00000519566 | KB-1991G8.1 | 0.420658387 | 0.895254723 | 1.1483744 | up | lncRNA |
| ENST00000519570 | CTC-340A15.2 | 0.962764706 | 0.998875101 | 1.0323379 | up | lncRNA |
| ENST00000519603 | RP11-443C10.1 | 0.270221224 | 0.830995087 | 1.3409443 | up | lncRNA |
| ENST00000519624 | RP11-459E5.1 | 0.346198507 | 0.865832425 | 1.0723735 | up | lncRNA |
| ENST00000519655 | RP11-37B2.1 | 0.150043669 | 0.705518742 | 1.5202007 | up | lncRNA |
| ENST00000519680 | RP11-1D12.1 | 0.019347961 | 0.346669087 | 3.0795209 | up | lncRNA |
| ENST00000519694 | HOXA-AS4 | 0.563798067 | 0.943393901 | 1.0736402 | up | lncRNA |
| ENST00000519727 | CTB-157D17.1 | 0.127911727 | 0.680549576 | 1.1499743 | up | lncRNA |
| ENST00000519738 | RP11-150O12.1 | 0.6655036 | 0.972539219 | 1.0705065 | up | lncRNA |
| ENST00000519750 | CTC-340A15.2 | 0.634685934 | 0.964937767 | 1.4795386 | up | lncRNA |
| ENST00000519753 | CTD-3107M8.4 | 0.064836309 | 0.541345997 | 1.2162785 | up | lncRNA |
| ENST00000519793 | KB-1930G5.4 | 0.908102638 | 0.998875101 | 1.0189605 | up | lncRNA |
| ENST00000519795 | CTB-178M22.2 | 0.290275064 | 0.837264328 | 1.4270077 | up | lncRNA |
| ENST00000519805 | RP11-642D21.1 | 0.598018091 | 0.952424484 | 1.9364624 | up | lncRNA |
| ENST00000519819 | CTD-3046C4.1 | 0.402172986 | 0.889146721 | 1.0745512 | up | lncRNA |
| ENST00000519854 | RP11-37B2.1 | 0.082489727 | 0.585414993 | 1.4508041 | up | lncRNA |
| ENST00000519976 | CTB-79E8.2 | 0.457540952 | 0.912197894 | 1.0503907 | up | lncRNA |
| ENST00000520017 | RP11-10A14.3 | 0.161166644 | 0.716150139 | 1.2598576 | up | lncRNA |
| ENST00000520023 | EXTL3-AS1 | 0.445096104 | 0.90519468 | 1.373477 | up | lncRNA |
| ENST00000520032 | RP11-61G23.2 | 0.662549481 | 0.971730383 | 1.1571089 | up | lncRNA |
| ENST00000520041 | CTC-558O2.2 | 0.310243883 | 0.848664472 | 1.2306698 | up | lncRNA |
| ENST00000520069 | MEG3 | 0.320138572 | 0.852213764 | 1.2067418 | up | lncRNA |
| ENST00000520129 | RP11-219B4.3 | 0.604067197 | 0.955851423 | 1.0754978 | up | lncRNA |
| ENST00000520300 | CTB-33O18.2 | 0.393868384 | 0.885711362 | 1.1376362 | up | lncRNA |
| ENST00000520310 | TPT1-AS1 | 0.806736243 | 0.992578294 | 1.0545993 | up | lncRNA |
| ENST00000520348 | SNHG6 | 0.463492217 | 0.913747281 | 1.1004887 | up | lncRNA |
| ENST00000520375 | RP11-213G6.2 | 0.407486056 | 0.889546439 | 1.2063991 | up | lncRNA |
| ENST00000520432 | TPT1-AS1 | 0.440884834 | 0.904456709 | 1.1546257 | up | lncRNA |
| ENST00000520433 | RP11-152P17.2 | 0.972487294 | 0.998875101 | 1.0213025 | up | lncRNA |
| ENST00000520443 | CTC-329D1.3 | 0.090022602 | 0.604305136 | 1.2102666 | up | lncRNA |
| ENST00000520494 | RP11-981G7.3 | 0.897865081 | 0.998875101 | 1.0205724 | up | lncRNA |
| ENST00000520496 | RP11-26M5.3 | 0.752234622 | 0.986481248 | 1.0271507 | up | lncRNA |
| ENST00000520562 | RP11-10N23.2 | 0.12671784 | 0.679616756 | 1.1165261 | up | lncRNA |
| ENST00000520582 | RP11-62H7.2 | 0.388779119 | 0.885276359 | 1.1415462 | up | lncRNA |
| ENST00000520585 | TPT1-AS1 | 0.23731588 | 0.806512655 | 1.1518476 | up | lncRNA |
| ENST00000520658 | CTB-47B11.3 | 0.198717484 | 0.765430307 | 1.1662223 | up | lncRNA |
| ENST00000520714 | MEG3 | 0.221364126 | 0.790635384 | 1.0666759 | up | lncRNA |
| ENST00000520820 | KB-431C1.4 | 0.622749968 | 0.960457826 | 1.0425034 | up | lncRNA |
| ENST00000520838 | AC034243.1 | 0.291586349 | 0.837264328 | 1.1321627 | up | lncRNA |
| ENST00000520842 | RP11-134O21.1 | 0.896899016 | 0.998875101 | 1.0076631 | up | lncRNA |
| ENST00000520849 | RP11-586K2.1 | 0.080138649 | 0.581177305 | 1.277971 | up | lncRNA |
| ENST00000520882 | AC005740.5 | 0.327218289 | 0.854556781 | 1.1597367 | up | lncRNA |
| ENST00000520885 | RP11-809O17.1 | 3.48471E-05 | 0.025006259 | 7.6098431 | up | lncRNA |
| ENST00000520944 | SNHG6 | 0.247760223 | 0.814489937 | 1.2083044 | up | lncRNA |
| ENST00000521030 | CTA-392C11.1 | 0.701067561 | 0.980170937 | 1.8025871 | up | lncRNA |
| ENST00000521100 | FAM90A24P | 0.731672089 | 0.983763148 | 1.0180316 | up | lncRNA |
| ENST00000521133 | CTB-131B5.4 | 0.381822189 | 0.881294633 | 1.0725929 | up | lncRNA |
| ENST00000521141 | RP11-459E5.1 | 0.014559964 | 0.306399703 | 1.9815565 | up | lncRNA |
| ENST00000521148 | RP11-100L22.2 | 0.941554482 | 0.998875101 | 1.0234804 | up | lncRNA |
| ENST00000521149 | RP11-981G7.4 | 0.745519899 | 0.985094857 | 1.0186167 | up | lncRNA |
| ENST00000521256 | MEG3 | 0.764954477 | 0.989189555 | 1.0141169 | up | lncRNA |
| ENST00000521257 | RP11-359E19.2 | 0.00149864 | 0.135129436 | 1.4196034 | up | lncRNA |
| ENST00000521336 | TPT1-AS1 | 0.791009266 | 0.991420185 | 1.0188785 | up | lncRNA |
| ENST00000521383 | RP11-318M2.2 | 0.899871296 | 0.998875101 | 1.0219277 | up | lncRNA |
| ENST00000521412 | AACSP1 | 0.687615195 | 0.97861545 | 1.0170698 | up | lncRNA |
| ENST00000521470 | RP11-231D20.2 | 0.13355897 | 0.689601422 | 1.0489551 | up | lncRNA |
| ENST00000521471 | CTB-114C7.3 | 0.269842234 | 0.830995087 | 1.074302 | up | lncRNA |
| ENST00000521482 | RP11-44N11.1 | 0.082059968 | 0.585414993 | 1.3803413 | up | lncRNA |
| ENST00000521483 | RP11-16M8.2 | 0.088022124 | 0.598480971 | 1.499385 | up | lncRNA |
| ENST00000521507 | TPT1-AS1 | 0.599396651 | 0.95320642 | 1.0545557 | up | lncRNA |
| ENST00000521511 | RP11-495O10.1 | 0.474610119 | 0.917441334 | 1.0544538 | up | lncRNA |
| ENST00000521586 | RP11-382A18.2 | 0.775690571 | 0.990727199 | 1.0703756 | up | lncRNA |
| ENST00000521612 | RP11-110G21.1 | 0.94339615 | 0.998875101 | 1.0174319 | up | lncRNA |
| ENST00000521660 | RP11-770E5.3 | 0.353680139 | 0.869401398 | 1.1112963 | up | lncRNA |
| ENST00000521666 | RP11-80G7.1 | 0.108261229 | 0.64125677 | 1.2216725 | up | lncRNA |
| ENST00000521696 | RP11-410L14.2 | 0.650989984 | 0.969927925 | 1.063321 | up | lncRNA |
| ENST00000521697 | CTB-180C19.1 | 0.012582785 | 0.285740719 | 1.6136465 | up | lncRNA |
| ENST00000521793 | RP11-489O18.1 | 0.004427484 | 0.188856054 | 1.2189659 | up | lncRNA |
| ENST00000521802 | RP11-231D20.2 | 0.436344071 | 0.901926599 | 1.0637959 | up | lncRNA |
| ENST00000521812 | MEG3 | 0.583408254 | 0.949379205 | 1.1232092 | up | lncRNA |
| ENST00000521851 | KB-1615E4.2 | 0.192258502 | 0.760662974 | 1.099201 | up | lncRNA |
| ENST00000521863 | LINC00599 | 0.882674677 | 0.998875101 | 1.0155729 | up | lncRNA |
| ENST00000521888 | RP3-399L15.3 | 0.059266684 | 0.530697632 | 1.1953985 | up | lncRNA |
| ENST00000521904 | RP11-1101K5.1 | 0.285341142 | 0.835236986 | 1.4097829 | up | lncRNA |
| ENST00000521908 | RP11-489E7.1 | 0.459578287 | 0.91221792 | 1.0779229 | up | lncRNA |
| ENST00000521965 | MIR4454 | 0.3896656 | 0.885317362 | 1.1332691 | up | lncRNA |
| ENST00000521991 | RP11-136O12.2 | 0.686783607 | 0.97861545 | 1.2312138 | up | lncRNA |
| ENST00000521996 | RP11-37B2.1 | 0.078967436 | 0.577356551 | 1.2384432 | up | lncRNA |
| ENST00000522019 | RP11-87E22.2 | 0.770968427 | 0.989973164 | 1.0850812 | up | lncRNA |
| ENST00000522028 | RP11-486M23.2 | 0.998720726 | 0.999477117 | 1.0010664 | up | lncRNA |
| ENST00000522087 | RP11-245A18.1 | 0.748790853 | 0.985094857 | 1.0695886 | up | lncRNA |
| ENST00000522123 | RP11-731N10.1 | 0.148927234 | 0.704640207 | 2.0057108 | up | lncRNA |
| ENST00000522132 | RP11-68L18.1 | 0.953863512 | 0.998875101 | 1.0202714 | up | lncRNA |
| ENST00000522197 | HAS2-AS1 | 0.918257242 | 0.998875101 | 1.0206631 | up | lncRNA |
| ENST00000522318 | IMPA1P | 0.614074314 | 0.958418342 | 1.0488659 | up | lncRNA |
| ENST00000522330 | RP11-114O8.1 | 0.137214714 | 0.694768806 | 1.3621062 | up | lncRNA |
| ENST00000522383 | RP11-539E17.5 | 0.277092597 | 0.832297359 | 1.191694 | up | lncRNA |
| ENST00000522393 | RP11-556O5.4 | 0.880899016 | 0.998875101 | 1.0365652 | up | lncRNA |
| ENST00000522414 | PVT1 | 0.987469826 | 0.99906281 | 1.0058798 | up | lncRNA |
| ENST00000522416 | KB-1639H6.2 | 0.115276597 | 0.657882593 | 1.3512929 | up | lncRNA |
| ENST00000522471 | RP11-150O12.3 | 0.80802103 | 0.992578294 | 1.0754267 | up | lncRNA |
| ENST00000522486 | CTA-392C11.1 | 0.963680693 | 0.998875101 | 1.0661601 | up | lncRNA |
| ENST00000522511 | RP11-17A4.2 | 0.5057392 | 0.925222307 | 1.3839137 | up | lncRNA |
| ENST00000522571 | CTB-43E15.4 | 0.264556109 | 0.827574498 | 1.0543548 | up | lncRNA |
| ENST00000522618 | MEG3 | 0.260770583 | 0.825083643 | 1.0502437 | up | lncRNA |
| ENST00000522627 | AC011343.1 | 0.129734681 | 0.682265946 | 1.2389112 | up | lncRNA |
| ENST00000522643 | RP11-150O12.5 | 0.452790936 | 0.909172908 | 1.0950511 | up | lncRNA |
| ENST00000522646 | CTC-340A15.2 | 0.775731007 | 0.990727199 | 1.1624571 | up | lncRNA |
| ENST00000522673 | TPT1-AS1 | 0.725704537 | 0.98251396 | 1.044265 | up | lncRNA |
| ENST00000522686 | CTC-340A15.2 | 0.963893464 | 0.998875101 | 1.0404398 | up | lncRNA |
| ENST00000522765 | RP11-115J16.2 | 0.005254699 | 0.201643161 | 2.4328269 | up | lncRNA |
| ENST00000522767 | KB-1448A5.1 | 0.632066499 | 0.964380442 | 1.0891361 | up | lncRNA |
| ENST00000522817 | GS1-251I9.4 | 0.379837787 | 0.880257051 | 1.1362521 | up | lncRNA |
| ENST00000522838 | RP11-6N13.1 | 0.242413397 | 0.809272444 | 1.2630406 | up | lncRNA |
| ENST00000522845 | TPT1-AS1 | 0.737643888 | 0.984316636 | 1.02389 | up | lncRNA |
| ENST00000522859 | TPT1-AS1 | 0.55123099 | 0.942167708 | 1.3300456 | up | lncRNA |
| ENST00000522875 | PVT1 | 0.361754182 | 0.873683336 | 1.0916368 | up | lncRNA |
| ENST00000522897 | CTD-2541M15.1 | 0.432094352 | 0.900451701 | 1.4227427 | up | lncRNA |
| ENST00000522918 | RP11-628E19.2 | 0.978359603 | 0.998875101 | 1.0029928 | up | lncRNA |
| ENST00000522960 | RP11-56F10.3 | 0.210509751 | 0.778067459 | 1.1008145 | up | lncRNA |
| ENST00000523011 | RP11-267M23.4 | 0.086876073 | 0.59772071 | 1.1124923 | up | lncRNA |
| ENST00000523024 | RP11-981G7.4 | 0.843601487 | 0.995583664 | 1.0325133 | up | lncRNA |
| ENST00000523050 | CTC-353G13.1 | 0.933147258 | 0.998875101 | 1.0111836 | up | lncRNA |
| ENST00000523063 | RP11-317N12.1 | 0.057539552 | 0.525082084 | 1.1868349 | up | lncRNA |
| ENST00000523081 | RP11-360L9.4 | 0.23753855 | 0.806512655 | 1.6468491 | up | lncRNA |
| ENST00000523087 | RP3-399L15.3 | 0.923530113 | 0.998875101 | 1.0169032 | up | lncRNA |
| ENST00000523166 | RP11-17A4.3 | 0.368197117 | 0.876138064 | 1.2699099 | up | lncRNA |
| ENST00000523194 | RP11-461C13.1 | 0.723554976 | 0.98251396 | 1.0632502 | up | lncRNA |
| ENST00000523246 | RP11-115J16.1 | 0.275518473 | 0.832297359 | 1.0706452 | up | lncRNA |
| ENST00000523301 | RP11-175K6.1 | 0.84171531 | 0.995583664 | 1.1036058 | up | lncRNA |
| ENST00000523307 | RP11-4K16.2 | 0.802444506 | 0.992578294 | 1.1070358 | up | lncRNA |
| ENST00000523328 | PVT1 | 0.600537373 | 0.953841565 | 1.0760269 | up | lncRNA |
| ENST00000523342 | RP11-89M20.2 | 0.590835361 | 0.950912706 | 1.149527 | up | lncRNA |
| ENST00000523380 | RP11-257P3.3 | 0.141579326 | 0.698568819 | 1.1539358 | up | lncRNA |
| ENST00000523394 | RP11-378A12.1 | 0.701163652 | 0.980170937 | 1.1224787 | up | lncRNA |
| ENST00000523445 | TPT1-AS1 | 0.96728303 | 0.998875101 | 1.0037205 | up | lncRNA |
| ENST00000523502 | KB-1568E2.1 | 0.379379303 | 0.880257051 | 1.0626691 | up | lncRNA |
| ENST00000523506 | TPT1-AS1 | 0.840177309 | 0.995583664 | 1.0191791 | up | lncRNA |
| ENST00000523563 | KB-1471A8.2 | 0.460236041 | 0.91221792 | 1.5440954 | up | lncRNA |
| ENST00000523584 | RP11-152K4.2 | 0.976625676 | 0.998875101 | 1.0091968 | up | lncRNA |
| ENST00000523602 | RP11-142A23.1 | 0.064804556 | 0.541345997 | 1.3508971 | up | lncRNA |
| ENST00000523605 | CTB-12O2.1 | 0.075669557 | 0.568987288 | 1.2595658 | up | lncRNA |
| ENST00000523614 | RP11-318M2.2 | 0.464223981 | 0.913755581 | 1.0712337 | up | lncRNA |
| ENST00000523648 | CTC-340A15.2 | 0.797506041 | 0.991665804 | 1.1966098 | up | lncRNA |
| ENST00000523657 | RP11-706C16.7 | 0.195519223 | 0.761823233 | 1.3812812 | up | lncRNA |
| ENST00000523664 | RP11-16M8.2 | 0.725515038 | 0.98251396 | 1.0455275 | up | lncRNA |
| ENST00000523671 | MEG3 | 0.553409541 | 0.943023022 | 1.0387674 | up | lncRNA |
| ENST00000523692 | RP11-325I22.2 | 0.899554303 | 0.998875101 | 1.0508778 | up | lncRNA |
| ENST00000523700 | RP11-624C23.1 | 0.618429776 | 0.95998809 | 1.0748835 | up | lncRNA |
| ENST00000523703 | RP11-37N22.1 | 0.287043711 | 0.836961572 | 1.1109695 | up | lncRNA |
| ENST00000523781 | CTB-17P3.4 | 0.373075658 | 0.878706238 | 1.0673994 | up | lncRNA |
| ENST00000523786 | RP11-16M8.2 | 0.575834414 | 0.947022539 | 1.175784 | up | lncRNA |
| ENST00000523789 | EXTL3-AS1 | 0.072084413 | 0.561089946 | 1.1124882 | up | lncRNA |
| ENST00000523790 | HOXA-AS4 | 0.109984341 | 0.645372325 | 1.2844734 | up | lncRNA |
| ENST00000523791 | KB-1507C5.3 | 0.859800634 | 0.996142678 | 1.0157136 | up | lncRNA |
| ENST00000523840 | RP11-395I14.2 | 0.499849429 | 0.922144236 | 1.1309518 | up | lncRNA |
| ENST00000523859 | RP11-37B2.1 | 0.038944155 | 0.451913909 | 1.7644819 | up | lncRNA |
| ENST00000523884 | RP11-875O11.1 | 0.152032454 | 0.707512899 | 1.22929 | up | lncRNA |
| ENST00000523905 | RP11-347C18.3 | 0.872992265 | 0.997909851 | 1.037771 | up | lncRNA |
| ENST00000523971 | RP11-134O21.1 | 0.994772628 | 0.999156445 | 1.001135 | up | lncRNA |
| ENST00000523995 | RP11-37B2.1 | 0.022602493 | 0.363379513 | 1.1802591 | up | lncRNA |
| ENST00000524035 | MEG3 | 0.865218678 | 0.996767702 | 1.0193456 | up | lncRNA |
| ENST00000524042 | RP11-855C21.1 | 0.311975005 | 0.849269161 | 1.1172687 | up | lncRNA |
| ENST00000524045 | RP11-152P17.2 | 0.918548433 | 0.998875101 | 1.1602934 | up | lncRNA |
| ENST00000524052 | RP11-317J10.2 | 0.912737653 | 0.998875101 | 1.0115813 | up | lncRNA |
| ENST00000524100 | RP11-473O4.5 | 0.902800122 | 0.998875101 | 1.0189676 | up | lncRNA |
| ENST00000524131 | MEG3 | 0.04422239 | 0.478642344 | 1.0784549 | up | lncRNA |
| ENST00000524166 | RP11-37B2.1 | 0.324458348 | 0.853958969 | 1.5521616 | up | lncRNA |
| ENST00000524198 | CTC-529G1.1 | 0.329614096 | 0.85540421 | 1.1954911 | up | lncRNA |
| ENST00000524284 | RP11-32K4.2 | 0.815142519 | 0.99257857 | 1.0642439 | up | lncRNA |
| ENST00000524338 | RP11-16M8.2 | 0.745818908 | 0.985094857 | 1.0606173 | up | lncRNA |
| ENST00000524444 | RP4-541C22.4 | 0.022669348 | 0.363433962 | 1.2514436 | up | lncRNA |
| ENST00000524565 | RP11-430H10.1 | 0.492082255 | 0.920777644 | 1.2862131 | up | lncRNA |
| ENST00000524619 | AP000487.5 | 0.222489199 | 0.790839 | 1.2415831 | up | lncRNA |
| ENST00000524707 | RP11-22P4.2 | 0.336996215 | 0.859588936 | 1.4059494 | up | lncRNA |
| ENST00000524768 | CTD-2530H12.2 | 0.407632521 | 0.889546439 | 1.1084265 | up | lncRNA |
| ENST00000524772 | RP11-27G22.1 | 0.720496912 | 0.98251396 | 1.1348367 | up | lncRNA |
| ENST00000524808 | RP11-661A12.12 | 0.478008039 | 0.917898232 | 1.0662596 | up | lncRNA |
| ENST00000524818 | RP11-522I20.3 | 0.557633108 | 0.943023022 | 1.0638337 | up | lncRNA |
| ENST00000524858 | RP11-430L3.1 | 0.375662419 | 0.879312727 | 1.120377 | up | lncRNA |
| ENST00000524885 | RP11-358H18.3 | 0.69728548 | 0.980170937 | 1.0491558 | up | lncRNA |
| ENST00000524962 | RP11-680F20.6 | 0.726529497 | 0.98251396 | 1.0825563 | up | lncRNA |
| ENST00000524978 | AC093734.11 | 0.533999642 | 0.935381352 | 1.0689715 | up | lncRNA |
| ENST00000525023 | GS1-393G12.12 | 0.911639037 | 0.998875101 | 1.0105437 | up | lncRNA |
| ENST00000525111 | RP11-445F12.1 | 0.497559388 | 0.921805853 | 1.212432 | up | lncRNA |
| ENST00000525142 | CTD-3074O7.5 | 0.312795778 | 0.850074797 | 1.0286527 | up | lncRNA |
| ENST00000525233 | FAR1-IT1 | 0.405633687 | 0.889198786 | 1.1062039 | up | lncRNA |
| ENST00000525262 | UNC93B6 | 0.115789293 | 0.65904431 | 1.128197 | up | lncRNA |
| ENST00000525302 | RP11-1L12.3 | 0.0603611 | 0.531246381 | 1.140095 | up | lncRNA |
| ENST00000525309 | RP11-406D1.2 | 0.874345815 | 0.997909851 | 1.0709952 | up | lncRNA |
| ENST00000525429 | RP11-687M24.4 | 0.40791184 | 0.889546439 | 1.4754923 | up | lncRNA |
| ENST00000525450 | RP11-689N19.3 | 0.196188617 | 0.762235583 | 1.1587855 | up | lncRNA |
| ENST00000525461 | CTD-2517M22.16 | 0.14916764 | 0.704640207 | 1.1054563 | up | lncRNA |
| ENST00000525484 | TMEM9B-AS1 | 0.326315302 | 0.854556781 | 1.0778666 | up | lncRNA |
| ENST00000525580 | RP11-535A19.2 | 0.959105648 | 0.998875101 | 1.0063676 | up | lncRNA |
| ENST00000525654 | RP11-347H15.4 | 0.372358335 | 0.878574831 | 1.1300044 | up | lncRNA |
| ENST00000525846 | RP11-1212A22.1 | 0.115738645 | 0.65904431 | 1.066504 | up | lncRNA |
| ENST00000525855 | RP11-958J22.1 | 0.553008183 | 0.943023022 | 1.0762727 | up | lncRNA |
| ENST00000525871 | RP5-1024C24.1 | 0.070056963 | 0.556731747 | 1.0788813 | up | lncRNA |
| ENST00000525893 | RP13-317D12.3 | 0.849668487 | 0.995876116 | 1.0378621 | up | lncRNA |
| ENST00000526061 | RP11-1L12.3 | 0.254075643 | 0.82035852 | 1.219755 | up | lncRNA |
| ENST00000526079 | GOLGA8E | 0.248586941 | 0.814920004 | 1.5548443 | up | lncRNA |
| ENST00000526174 | RP11-805J14.5 | 0.437526158 | 0.902506883 | 1.1000962 | up | lncRNA |
| ENST00000526225 | CTD-2210P24.3 | 0.340789771 | 0.861561333 | 1.0837372 | up | lncRNA |
| ENST00000526308 | PCDHB18 | 0.684639903 | 0.977836377 | 1.1445873 | up | lncRNA |
| ENST00000526310 | RP11-864G5.3 | 0.35820005 | 0.872424004 | 1.2008259 | up | lncRNA |
| ENST00000526344 | RP11-160H12.2 | 0.747705895 | 0.985094857 | 1.0957513 | up | lncRNA |
| ENST00000526431 | RP13-46H24.1 | 0.000392775 | 0.084119144 | 3.3386543 | up | lncRNA |
| ENST00000526456 | RP11-732A19.5 | 0.756537643 | 0.98762882 | 1.0437419 | up | lncRNA |
| ENST00000526458 | RP11-109D9.3 | 0.839690907 | 0.995583664 | 1.0149863 | up | lncRNA |
| ENST00000526556 | RP3-468B3.2 | 0.193216426 | 0.760765035 | 1.1353098 | up | lncRNA |
| ENST00000526566 | CTD-2653D5.1 | 0.731221715 | 0.983763148 | 1.0386212 | up | lncRNA |
| ENST00000526611 | RP1-179N16.6 | 0.397621875 | 0.887348439 | 1.1391214 | up | lncRNA |
| ENST00000526612 | RP11-326C3.7 | 0.92097504 | 0.998875101 | 1.0172892 | up | lncRNA |
| ENST00000526635 | CTD-2339L15.1 | 0.518646059 | 0.93080008 | 1.194764 | up | lncRNA |
| ENST00000526642 | NAV2-AS1 | 0.523237658 | 0.932117592 | 1.3242817 | up | lncRNA |
| ENST00000526646 | RP11-236J17.6 | 0.986127528 | 0.99906281 | 1.0011435 | up | lncRNA |
| ENST00000526694 | RP5-1007M22.2 | 0.050786956 | 0.508649264 | 1.7163345 | up | lncRNA |
| ENST00000526695 | CTD-2516F10.2 | 0.355279395 | 0.870470894 | 1.0383768 | up | lncRNA |
| ENST00000526706 | CTD-2516F10.2 | 0.37865956 | 0.88011421 | 1.1939484 | up | lncRNA |
| ENST00000526769 | GVINP1 | 0.024347769 | 0.374301178 | 1.0611249 | up | lncRNA |
| ENST00000526795 | RP11-113K21.5 | 0.04418315 | 0.478642344 | 1.2272304 | up | lncRNA |
| ENST00000526796 | RP11-687M24.8 | 0.62003341 | 0.96013301 | 1.0647333 | up | lncRNA |
| ENST00000526835 | TMCO5B | 0.447700023 | 0.905943546 | 1.1138952 | up | lncRNA |
| ENST00000526867 | RP11-567I13.1 | 0.751794568 | 0.98644685 | 1.0950105 | up | lncRNA |
| ENST00000526934 | RP11-831A10.2 | 0.097003757 | 0.622605316 | 1.1125892 | up | lncRNA |
| ENST00000526947 | RP11-550A5.2 | 0.01967984 | 0.350004957 | 2.0790878 | up | lncRNA |
| ENST00000526984 | RP11-672A2.2 | 0.745905612 | 0.985094857 | 1.0586417 | up | lncRNA |
| ENST00000527030 | RP11-452H21.2 | 0.550205083 | 0.942167708 | 1.1229565 | up | lncRNA |
| ENST00000527061 | RP11-867G23.10 | 0.051636877 | 0.512512076 | 1.1040015 | up | lncRNA |
| ENST00000527080 | USP17L8 | 0.244310809 | 0.810806356 | 1.0598013 | up | lncRNA |
| ENST00000527092 | CTD-3074O7.5 | 0.331719439 | 0.856326799 | 1.0568228 | up | lncRNA |
| ENST00000527113 | RP11-496I9.1 | 0.432602696 | 0.900592919 | 1.0603222 | up | lncRNA |
| ENST00000527191 | RP11-732A19.8 | 0.421510563 | 0.895783495 | 1.1316126 | up | lncRNA |
| ENST00000527274 | CTD-3074O7.5 | 0.29687234 | 0.840211364 | 1.0606395 | up | lncRNA |
| ENST00000527283 | RP11-2F20.1 | 0.277109089 | 0.832297359 | 1.4960425 | up | lncRNA |
| ENST00000527288 | RP11-265D17.2 | 0.970022163 | 0.998875101 | 1.0019782 | up | lncRNA |
| ENST00000527321 | RP11-705O3.1 | 0.303512184 | 0.844725814 | 1.1581587 | up | lncRNA |
| ENST00000527406 | RP11-540A21.3 | 0.342612232 | 0.863514929 | 1.1659617 | up | lncRNA |
| ENST00000527426 | RP11-750H9.5 | 0.4467322 | 0.905331314 | 1.1116832 | up | lncRNA |
| ENST00000527453 | RP11-770G2.4 | 0.591280862 | 0.950912706 | 1.0691487 | up | lncRNA |
| ENST00000527511 | RP11-356J5.12 | 0.918822996 | 0.998875101 | 1.0333746 | up | lncRNA |
| ENST00000527518 | RP11-234B24.2 | 0.204856752 | 0.771727492 | 1.1318253 | up | lncRNA |
| ENST00000527533 | RP11-109E10.1 | 0.519918074 | 0.93173 | 1.2129092 | up | lncRNA |
| ENST00000527617 | RP11-693N9.2 | 0.093007439 | 0.612479847 | 1.2389394 | up | lncRNA |
| ENST00000527627 | RP11-113K21.5 | 0.562350032 | 0.943393901 | 1.03179 | up | lncRNA |
| ENST00000527642 | RP11-958J22.1 | 0.328516941 | 0.854918296 | 1.1318027 | up | lncRNA |
| ENST00000527684 | RP5-945I17.2 | 0.045116924 | 0.481089193 | 1.3483766 | up | lncRNA |
| ENST00000527712 | RP11-713P17.3 | 0.598270218 | 0.952424484 | 1.0655975 | up | lncRNA |
| ENST00000527726 | RP11-483L5.1 | 0.689971689 | 0.97861545 | 1.0362869 | up | lncRNA |
| ENST00000527789 | AP003068.12 | 0.618863127 | 0.95998809 | 1.1286499 | up | lncRNA |
| ENST00000527801 | RP11-182J1.1 | 0.991788337 | 0.999156445 | 1.0048343 | up | lncRNA |
| ENST00000527880 | RP11-466P24.2 | 0.76863912 | 0.989189555 | 1.0393426 | up | lncRNA |
| ENST00000527908 | RP11-429J17.5 | 0.224043797 | 0.793609179 | 1.1313479 | up | lncRNA |
| ENST00000527960 | RP11-613D13.7 | 0.826096785 | 0.993856674 | 1.0435814 | up | lncRNA |
| ENST00000527997 | RP13-631K18.5 | 0.369095103 | 0.8763174 | 1.7202496 | up | lncRNA |
| ENST00000528056 | PRKY | 0.228884328 | 0.798130144 | 1.0260445 | up | lncRNA |
| ENST00000528075 | RP11-21L23.3 | 0.025728104 | 0.382432679 | 1.1562792 | up | lncRNA |
| ENST00000528083 | RP11-727A23.5 | 0.555748028 | 0.943023022 | 1.1002449 | up | lncRNA |
| ENST00000528139 | RP11-1134I14.8 | 0.906879126 | 0.998875101 | 1.0563996 | up | lncRNA |
| ENST00000528151 | RP11-236J17.6 | 0.284403011 | 0.834623138 | 1.1343977 | up | lncRNA |
| ENST00000528194 | RP11-222N13.1 | 0.096637886 | 0.621949299 | 1.1510239 | up | lncRNA |
| ENST00000528242 | UNC93B6 | 0.264556285 | 0.827574498 | 1.1846483 | up | lncRNA |
| ENST00000528366 | RP4-607I7.1 | 1.84772E-07 | 0.001260287 | 20.2333495 | up | lncRNA |
| ENST00000528381 | RP11-820L6.1 | 0.016827336 | 0.327871272 | 1.1272884 | up | lncRNA |
| ENST00000528437 | RP11-693N9.2 | 0.069957218 | 0.556731747 | 1.2391673 | up | lncRNA |
| ENST00000528497 | RP11-555G19.1 | 0.013732393 | 0.294694748 | 1.8107048 | up | lncRNA |
| ENST00000528510 | RP11-334E6.3 | 0.824275835 | 0.993856674 | 1.0255258 | up | lncRNA |
| ENST00000528520 | RP11-5L12.1 | 0.004365303 | 0.188707333 | 3.1183813 | up | lncRNA |
| ENST00000528524 | RP11-727A23.5 | 0.997141544 | 0.999160472 | 1.000273 | up | lncRNA |
| ENST00000528578 | RP11-770J1.3 | 0.055481337 | 0.520364745 | 1.2604358 | up | lncRNA |
| ENST00000528607 | AP000487.6 | 0.193566039 | 0.760765035 | 1.1839196 | up | lncRNA |
| ENST00000528646 | RP11-583F24.8 | 0.830109686 | 0.994074191 | 1.0346573 | up | lncRNA |
| ENST00000528690 | CTD-2517M22.14 | 0.177216998 | 0.742170516 | 1.1023372 | up | lncRNA |
| ENST00000528692 | RP5-1007M22.2 | 0.00148801 | 0.135129436 | 1.9412073 | up | lncRNA |
| ENST00000528781 | RP11-672A2.6 | 0.65089988 | 0.969927925 | 1.0649155 | up | lncRNA |
| ENST00000529014 | CTD-2003C8.1 | 0.021787119 | 0.361132318 | 1.1422689 | up | lncRNA |
| ENST00000529026 | AF186192.5 | 0.705703452 | 0.980849889 | 1.0608608 | up | lncRNA |
| ENST00000529031 | RP11-727A23.5 | 0.565364227 | 0.943572364 | 1.0676904 | up | lncRNA |
| ENST00000529069 | RP11-119D9.1 | 0.892573627 | 0.998875101 | 1.0422193 | up | lncRNA |
| ENST00000529081 | CTD-2026G22.1 | 0.80241515 | 0.992578294 | 1.3985277 | up | lncRNA |
| ENST00000529094 | RP11-539G18.2 | 0.096842102 | 0.622147652 | 1.3611939 | up | lncRNA |
| ENST00000529096 | AC005152.2 | 0.707989131 | 0.981606358 | 1.0499464 | up | lncRNA |
| ENST00000529127 | RP11-958J22.1 | 0.261816694 | 0.82544125 | 1.1013474 | up | lncRNA |
| ENST00000529215 | CTD-2562J17.7 | 0.544377458 | 0.939697392 | 1.0926613 | up | lncRNA |
| ENST00000529247 | RP11-661A12.7 | 0.063898688 | 0.537558012 | 1.2611619 | up | lncRNA |
| ENST00000529264 | RP11-445F12.1 | 0.338914506 | 0.860518281 | 1.1070906 | up | lncRNA |
| ENST00000529304 | CTD-2562J17.9 | 0.902251842 | 0.998875101 | 1.0152303 | up | lncRNA |
| ENST00000529314 | RP11-539G18.2 | 0.788353433 | 0.991420185 | 1.0703643 | up | lncRNA |
| ENST00000529369 | RP11-660L16.2 | 0.989088836 | 0.99906281 | 1.00317 | up | lncRNA |
| ENST00000529416 | RP11-839D17.3 | 0.061944053 | 0.534658422 | 1.2677151 | up | lncRNA |
| ENST00000529570 | RP11-147I3.1 | 0.032271576 | 0.421823005 | 1.1154848 | up | lncRNA |
| ENST00000529624 | HERC2P9 | 0.307017046 | 0.846715728 | 1.0701184 | up | lncRNA |
| ENST00000529656 | RSF1-IT2 | 0.883338602 | 0.998875101 | 1.0562971 | up | lncRNA |
| ENST00000529667 | AC005152.2 | 0.696205211 | 0.980170937 | 1.0526626 | up | lncRNA |
| ENST00000529696 | TMCO5B | 0.444771422 | 0.90519468 | 1.1098021 | up | lncRNA |
| ENST00000529719 | RP11-535A19.2 | 0.983801098 | 0.998875101 | 1.0051859 | up | lncRNA |
| ENST00000529733 | RP11-820L6.1 | 0.311948096 | 0.849269161 | 1.203743 | up | lncRNA |
| ENST00000529811 | RP11-727A23.5 | 0.284384075 | 0.834623138 | 1.1025285 | up | lncRNA |
| ENST00000529823 | RP11-166D19.1 | 0.72873789 | 0.982841582 | 1.0367684 | up | lncRNA |
| ENST00000529841 | RP11-108O10.2 | 0.419286701 | 0.894732097 | 1.0431876 | up | lncRNA |
| ENST00000529883 | RP11-152H18.3 | 0.184878935 | 0.751257244 | 1.1796647 | up | lncRNA |
| ENST00000529929 | CTD-2371O3.3 | 0.140230996 | 0.698568819 | 1.1620025 | up | lncRNA |
| ENST00000529961 | RP11-290F24.3 | 0.730054669 | 0.983087316 | 1.043706 | up | lncRNA |
| ENST00000530001 | CTD-2313N18.8 | 0.39269765 | 0.885711362 | 1.1904428 | up | lncRNA |
| ENST00000530045 | RP11-727A23.10 | 0.244337896 | 0.810806356 | 1.1191315 | up | lncRNA |
| ENST00000530072 | RP11-166D19.1 | 0.701618544 | 0.980170937 | 1.0221013 | up | lncRNA |
| ENST00000530196 | ADAM21P1 | 0.318712281 | 0.851223284 | 1.0699989 | up | lncRNA |
| ENST00000530246 | GOLGA8E | 0.561045134 | 0.943393901 | 1.2030217 | up | lncRNA |
| ENST00000530263 | RP4-607I7.1 | 0.002290719 | 0.143565088 | 3.3291809 | up | lncRNA |
| ENST00000530270 | RP11-113K21.5 | 0.060636068 | 0.531936951 | 1.2774038 | up | lncRNA |
| ENST00000530346 | RP1-80B9.2 | 0.973996667 | 0.998875101 | 1.006167 | up | lncRNA |
| ENST00000530379 | RP11-375D13.2 | 0.355685582 | 0.870832491 | 1.2853393 | up | lncRNA |
| ENST00000530420 | HERC2P9 | 0.551285395 | 0.942167708 | 1.0731264 | up | lncRNA |
| ENST00000530422 | SCARNA9 | 0.018104399 | 0.337638939 | 1.3047899 | up | lncRNA |
| ENST00000530430 | RP11-1L12.3 | 0.120618399 | 0.670714942 | 1.952992 | up | lncRNA |
| ENST00000530479 | RP11-261P9.4 | 0.067194461 | 0.548255059 | 1.2556201 | up | lncRNA |
| ENST00000530490 | OR7E14P | 0.359556453 | 0.873342057 | 1.1339579 | up | lncRNA |
| ENST00000530572 | RP11-688I9.4 | 0.511329567 | 0.9278461 | 1.1003166 | up | lncRNA |
| ENST00000530600 | RP11-661A12.5 | 0.791618425 | 0.991420185 | 1.0462757 | up | lncRNA |
| ENST00000530606 | ZNF503-AS1 | 0.42853406 | 0.897780553 | 1.1467499 | up | lncRNA |
| ENST00000530621 | RP11-448P19.1 | 0.304492622 | 0.845551016 | 1.3463251 | up | lncRNA |
| ENST00000530652 | NAV2-AS5 | 0.23742594 | 0.806512655 | 1.099256 | up | lncRNA |
| ENST00000530690 | AP000487.5 | 0.943741655 | 0.998875101 | 1.0122553 | up | lncRNA |
| ENST00000530792 | CTD-2562J17.4 | 0.422260883 | 0.895936649 | 1.182923 | up | lncRNA |
| ENST00000530825 | RP11-727A23.5 | 0.59095519 | 0.950912706 | 1.0554507 | up | lncRNA |
| ENST00000530963 | CTD-2516F10.4 | 0.09673234 | 0.622147652 | 1.5589131 | up | lncRNA |
| ENST00000530972 | RP11-91P24.6 | 0.36097587 | 0.873342057 | 1.1132046 | up | lncRNA |
| ENST00000531024 | RP11-385N17.1 | 0.935082865 | 0.998875101 | 1.0139217 | up | lncRNA |
| ENST00000531076 | RP11-326C3.12 | 0.69862264 | 0.980170937 | 1.1596304 | up | lncRNA |
| ENST00000531077 | RP11-27P7.1 | 0.796215753 | 0.991420185 | 1.0450557 | up | lncRNA |
| ENST00000531086 | RP11-755F10.1 | 0.391104648 | 0.885711362 | 1.2563166 | up | lncRNA |
| ENST00000531092 | RP1-80B9.2 | 0.994109643 | 0.999156445 | 1.0006624 | up | lncRNA |
| ENST00000531111 | CTD-2371O3.2 | 0.353374294 | 0.869401398 | 1.6419307 | up | lncRNA |
| ENST00000531193 | RP11-680F20.6 | 0.923897813 | 0.998875101 | 1.063573 | up | lncRNA |
| ENST00000531315 | FOLH1B | 0.474445932 | 0.917441334 | 1.244785 | up | lncRNA |
| ENST00000531363 | RP11-1L12.3 | 0.119901704 | 0.669062695 | 1.774699 | up | lncRNA |
| ENST00000531371 | RP11-693N9.2 | 0.27024525 | 0.830995087 | 1.5935844 | up | lncRNA |
| ENST00000531379 | RP11-91I20.4 | 0.515280574 | 0.930031728 | 1.087169 | up | lncRNA |
| ENST00000531381 | RP11-166D19.1 | 0.991517489 | 0.999156445 | 1.0015495 | up | lncRNA |
| ENST00000531511 | RP11-672A2.4 | 0.742771779 | 0.985094857 | 1.0371388 | up | lncRNA |
| ENST00000531585 | RP11-688I9.2 | 0.007425868 | 0.229585454 | 1.2566614 | up | lncRNA |
| ENST00000531617 | CTD-2514K5.2 | 0.437918017 | 0.902672564 | 1.1637086 | up | lncRNA |
| ENST00000531627 | RP1-17K7.2 | 0.308137933 | 0.847137912 | 1.2030522 | up | lncRNA |
| ENST00000531652 | RP11-939C17.2 | 0.534712317 | 0.935381352 | 1.0564138 | up | lncRNA |
| ENST00000531661 | RP11-522I20.3 | 0.471817076 | 0.916418344 | 1.0586451 | up | lncRNA |
| ENST00000531680 | OR1D4 | 0.33376828 | 0.85798034 | 1.441747 | up | lncRNA |
| ENST00000531692 | NAPSB | 0.169316016 | 0.727987857 | 1.2801005 | up | lncRNA |
| ENST00000531701 | GS1-24F4.2 | 0.174044445 | 0.736135912 | 1.1171784 | up | lncRNA |
| ENST00000531708 | AP001258.4 | 0.458554801 | 0.91221792 | 1.1059298 | up | lncRNA |
| ENST00000531730 | RP11-661A12.9 | 0.978260722 | 0.998875101 | 1.0098208 | up | lncRNA |
| ENST00000531763 | CTD-3096P4.1 | 0.425003939 | 0.897780553 | 1.060682 | up | lncRNA |
| ENST00000531887 | OR5G5P | 0.958470265 | 0.998875101 | 1.0261597 | up | lncRNA |
| ENST00000531938 | RP11-713P17.3 | 0.693095144 | 0.978978277 | 1.0582404 | up | lncRNA |
| ENST00000532153 | RP11-196E1.3 | 0.02480668 | 0.377450959 | 1.3321941 | up | lncRNA |
| ENST00000532249 | AC005152.2 | 0.499864381 | 0.922144236 | 1.4897655 | up | lncRNA |
| ENST00000532307 | CTD-2210P24.1 | 0.211685168 | 0.779801216 | 1.1282263 | up | lncRNA |
| ENST00000532357 | RP11-50B3.2 | 0.662406345 | 0.971730383 | 1.0292147 | up | lncRNA |
| ENST00000532358 | AC093734.11 | 0.031501527 | 0.418199276 | 1.2267803 | up | lncRNA |
| ENST00000532365 | RP11-685M7.5 | 0.084842067 | 0.590846042 | 1.1003156 | up | lncRNA |
| ENST00000532387 | RP11-445F12.1 | 0.949557979 | 0.998875101 | 1.0075466 | up | lncRNA |
| ENST00000532488 | CTD-2525J15.2 | 0.732545081 | 0.983763148 | 1.1131221 | up | lncRNA |
| ENST00000532524 | RP11-58K22.4 | 0.064215516 | 0.538644701 | 2.6379688 | up | lncRNA |
| ENST00000532599 | TMEM9B-AS1 | 0.563700104 | 0.943393901 | 1.3087639 | up | lncRNA |
| ENST00000532626 | RP11-831H9.3 | 0.383243727 | 0.882308947 | 1.1569559 | up | lncRNA |
| ENST00000532794 | CTD-2514K5.2 | 0.49938037 | 0.921814414 | 1.056323 | up | lncRNA |
| ENST00000532831 | RP11-705O3.1 | 0.313681094 | 0.851133753 | 1.0955477 | up | lncRNA |
| ENST00000532845 | AP001258.4 | 0.249694859 | 0.816027969 | 1.1565455 | up | lncRNA |
| ENST00000532874 | NAV2-AS4 | 0.461796783 | 0.912702045 | 1.169588 | up | lncRNA |
| ENST00000532875 | CTD-2313N18.4 | 0.181677036 | 0.747757045 | 1.0577504 | up | lncRNA |
| ENST00000532906 | RP11-689N19.3 | 0.001170814 | 0.121764606 | 1.3836157 | up | lncRNA |
| ENST00000532922 | RP13-25N22.1 | 0.885353396 | 0.998875101 | 1.0666411 | up | lncRNA |
| ENST00000532943 | RP11-750H9.5 | 0.213963249 | 0.781610853 | 1.0583105 | up | lncRNA |
| ENST00000532947 | RP11-22P4.1 | 0.930521716 | 0.998875101 | 1.01214 | up | lncRNA |
| ENST00000533009 | RP1-65P5.3 | 0.318090861 | 0.851223284 | 1.0658512 | up | lncRNA |
| ENST00000533167 | PMS2P3 | 0.495725819 | 0.921717611 | 1.1036688 | up | lncRNA |
| ENST00000533179 | AC005152.2 | 0.269176386 | 0.830995087 | 1.2853128 | up | lncRNA |
| ENST00000533203 | RP11-514F3.5 | 0.285444167 | 0.835236986 | 1.1498389 | up | lncRNA |
| ENST00000533287 | RP11-867G23.2 | 0.114852282 | 0.657011458 | 1.127422 | up | lncRNA |
| ENST00000533301 | RP11-350N15.3 | 0.87441314 | 0.997909851 | 1.0391303 | up | lncRNA |
| ENST00000533304 | RP3-468B3.3 | 0.366949687 | 0.875554764 | 1.1081245 | up | lncRNA |
| ENST00000533311 | RP11-655M14.13 | 0.675692934 | 0.976096237 | 1.0436535 | up | lncRNA |
| ENST00000533437 | RP11-21L23.4 | 0.035821708 | 0.433119759 | 1.6140309 | up | lncRNA |
| ENST00000533481 | RP11-98D18.9 | 0.753164884 | 0.986566987 | 1.0419482 | up | lncRNA |
| ENST00000533590 | RP11-535A19.2 | 0.346732688 | 0.865873516 | 1.1362953 | up | lncRNA |
| ENST00000533615 | FAM66E | 0.454868738 | 0.910032467 | 1.0850487 | up | lncRNA |
| ENST00000533767 | NAV2-AS2 | 0.084930002 | 0.590846042 | 1.7223676 | up | lncRNA |
| ENST00000533798 | RP11-474L11.5 | 0.343243647 | 0.863514929 | 1.1751795 | up | lncRNA |
| ENST00000533814 | RP11-58K22.5 | 0.194246354 | 0.760765035 | 1.1110045 | up | lncRNA |
| ENST00000533876 | RP11-655M14.12 | 0.744239316 | 0.985094857 | 1.0799655 | up | lncRNA |
| ENST00000533935 | AC093734.11 | 0.665347678 | 0.972501549 | 1.0286902 | up | lncRNA |
| ENST00000533941 | CTD-2003C8.2 | 0.022279524 | 0.361714622 | 3.8361304 | up | lncRNA |
| ENST00000534006 | RP11-299M14.2 | 0.010532802 | 0.261616866 | 1.1119456 | up | lncRNA |
| ENST00000534036 | NAV2-AS3 | 0.000527431 | 0.094750704 | 1.7362985 | up | lncRNA |
| ENST00000534135 | CTD-2140G10.1 | 0.931775011 | 0.998875101 | 1.0634369 | up | lncRNA |
| ENST00000534160 | RP1-80B9.2 | 0.523522311 | 0.932117592 | 1.0630347 | up | lncRNA |
| ENST00000534168 | RP11-452H21.1 | 0.879999788 | 0.998682284 | 1.0589638 | up | lncRNA |
| ENST00000534287 | RP11-613D13.7 | 0.823895122 | 0.993856674 | 1.0508337 | up | lncRNA |
| ENST00000534297 | RP11-166D19.1 | 0.482035665 | 0.9192486 | 1.070677 | up | lncRNA |
| ENST00000534342 | CTD-2560E9.5 | 0.30190186 | 0.843139812 | 1.0854531 | up | lncRNA |
| ENST00000534349 | TMEM9B-AS1 | 0.694684691 | 0.979851048 | 1.0337214 | up | lncRNA |
| ENST00000534388 | RP11-736I10.1 | 0.5185317 | 0.930711226 | 1.2535744 | up | lncRNA |
| ENST00000534398 | RP11-429J17.6 | 0.282474586 | 0.832629955 | 1.1698347 | up | lncRNA |
| ENST00000534477 | CTC-497E21.4 | 0.277264866 | 0.832297359 | 1.163379 | up | lncRNA |
| ENST00000534496 | RP11-820L6.1 | 0.287634445 | 0.836961572 | 1.2328346 | up | lncRNA |
| ENST00000534499 | RP11-113K21.5 | 0.564446326 | 0.943393901 | 1.0475788 | up | lncRNA |
| ENST00000534540 | MIR210HG | 0.861345614 | 0.996142678 | 1.0521201 | up | lncRNA |
| ENST00000534545 | RP11-939C17.2 | 0.363345164 | 0.874234597 | 1.1049139 | up | lncRNA |
| ENST00000534587 | RP11-21L19.1 | 0.067436716 | 0.548255059 | 1.6225964 | up | lncRNA |
| ENST00000534594 | RP5-916O11.2 | 0.330690573 | 0.855705627 | 1.1511308 | up | lncRNA |
| ENST00000534671 | RP11-540A21.3 | 0.786219934 | 0.991305067 | 1.0597731 | up | lncRNA |
| ENST00000534675 | RP11-354A14.1 | 0.636231927 | 0.96564589 | 1.2401475 | up | lncRNA |
| ENST00000534742 | RP11-326C3.2 | 0.416122074 | 0.892649611 | 1.2841189 | up | lncRNA |
| ENST00000534782 | RP11-820L6.1 | 0.570181616 | 0.944496311 | 1.0585106 | up | lncRNA |
| ENST00000534849 | RP5-916L7.2 | 0.297833842 | 0.841108087 | 1.1530411 | up | lncRNA |
| ENST00000534856 | RP11-286N22.10 | 0.365565702 | 0.874818743 | 1.1240401 | up | lncRNA |
| ENST00000534914 | LINC00467 | 0.395659429 | 0.885711362 | 1.0567802 | up | lncRNA |
| ENST00000535078 | ABC12-49244600F4.3 | 0.086250702 | 0.595989445 | 1.3980178 | up | lncRNA |
| ENST00000535133 | RP11-897M7.1 | 0.109653276 | 0.644976974 | 1.3054698 | up | lncRNA |
| ENST00000535188 | RP11-195E11.3 | 0.306010219 | 0.846407029 | 1.6469808 | up | lncRNA |
| ENST00000535247 | RP11-351O2.1 | 0.788129109 | 0.991420185 | 1.0225195 | up | lncRNA |
| ENST00000535249 | RP11-169K16.9 | 0.055269231 | 0.520364745 | 1.1859056 | up | lncRNA |
| ENST00000535320 | LINC00452 | 0.796343701 | 0.991420185 | 1.0467597 | up | lncRNA |
| ENST00000535337 | AC156455.1 | 0.818296523 | 0.992651327 | 1.0763056 | up | lncRNA |
| ENST00000535363 | RP11-27M24.1 | 0.096549041 | 0.621949299 | 1.4781676 | up | lncRNA |
| ENST00000535720 | RP11-173P15.3 | 0.680894587 | 0.977096946 | 1.072757 | up | lncRNA |
| ENST00000535721 | RP11-123O10.4 | 0.013492318 | 0.292952728 | 1.7275979 | up | lncRNA |
| ENST00000535745 | H19 | 0.779718622 | 0.99121281 | 1.0490926 | up | lncRNA |
| ENST00000535817 | RP11-727F15.9 | 0.453385751 | 0.909450994 | 1.1614453 | up | lncRNA |
| ENST00000535867 | RP11-727F15.9 | 0.98258556 | 0.998875101 | 1.001022 | up | lncRNA |
| ENST00000535914 | RP11-283G6.3 | 0.798118018 | 0.992168887 | 1.0815475 | up | lncRNA |
| ENST00000535921 | RP11-64B16.4 | 0.520829374 | 0.932117592 | 1.1291735 | up | lncRNA |
| ENST00000535922 | AP003419.16 | 0.391377708 | 0.885711362 | 1.1079776 | up | lncRNA |
| ENST00000536029 | RP11-392P7.8 | 0.451972739 | 0.908696429 | 1.1289368 | up | lncRNA |
| ENST00000536131 | RP11-625L16.1 | 0.296385324 | 0.839987155 | 1.1712795 | up | lncRNA |
| ENST00000536296 | LINC00271 | 0.692535863 | 0.97895216 | 1.0848136 | up | lncRNA |
| ENST00000536405 | RP11-467L20.10 | 0.02871279 | 0.405477148 | 1.522896 | up | lncRNA |
| ENST00000536487 | NKAPP1 | 0.644602434 | 0.967712776 | 1.1004415 | up | lncRNA |
| ENST00000536497 | RP11-885B4.2 | 0.242223686 | 0.809272444 | 1.2433145 | up | lncRNA |
| ENST00000536517 | RP11-173C20.2 | 1.10007E-05 | 0.014352949 | 33.7142516 | up | lncRNA |
| ENST00000536678 | RP11-181G12.2 | 0.99112672 | 0.999156445 | 1.000631 | up | lncRNA |
| ENST00000536679 | ABC12-49244600F4.3 | 0.127894554 | 0.680549576 | 1.0815987 | up | lncRNA |
| ENST00000536743 | AC062028.1 | 0.610801995 | 0.957448851 | 1.1062105 | up | lncRNA |
| ENST00000536744 | RP11-114G22.1 | 0.195564198 | 0.761823233 | 1.4172996 | up | lncRNA |
| ENST00000536804 | AC074391.1 | 0.712005871 | 0.98251396 | 1.0233946 | up | lncRNA |
| ENST00000536815 | RP11-395P17.3 | 0.34054552 | 0.861386906 | 1.1962214 | up | lncRNA |
| ENST00000536855 | RP11-809N8.4 | 0.274322506 | 0.832297359 | 1.4659638 | up | lncRNA |
| ENST00000536910 | RP11-87C12.4 | 0.343330509 | 0.863514929 | 1.1122344 | up | lncRNA |
| ENST00000536914 | IFNG-AS1 | 0.605895853 | 0.956038292 | 1.1792887 | up | lncRNA |
| ENST00000536922 | RP11-1060J15.4 | 0.791881827 | 0.991420185 | 1.0756233 | up | lncRNA |
| ENST00000536949 | RP11-443N24.2 | 0.078322948 | 0.5767814 | 1.3088668 | up | lncRNA |
| ENST00000537095 | RP11-143E21.7 | 0.879294047 | 0.998682284 | 1.0310947 | up | lncRNA |
| ENST00000537295 | RP11-598F7.6 | 0.084400868 | 0.589450732 | 1.209388 | up | lncRNA |
| ENST00000537298 | RP11-230G5.2 | 0.581134074 | 0.949239022 | 1.0544668 | up | lncRNA |
| ENST00000537346 | DENND5B-AS1 | 0.421277378 | 0.895596641 | 1.5039771 | up | lncRNA |
| ENST00000537361 | HNF1A-AS1 | 0.430354466 | 0.898685776 | 1.1236612 | up | lncRNA |
| ENST00000537401 | RP11-439A17.9 | 0.276307754 | 0.832297359 | 1.6740521 | up | lncRNA |
| ENST00000537478 | RP11-407A16.3 | 0.141031204 | 0.698568819 | 1.3223614 | up | lncRNA |
| ENST00000537525 | RP11-283G6.5 | 0.011401293 | 0.271539352 | 1.4719335 | up | lncRNA |
| ENST00000537555 | RP11-551L14.6 | 0.364981475 | 0.874818743 | 1.1152665 | up | lncRNA |
| ENST00000537616 | RP11-705C15.3 | 0.000227118 | 0.072435429 | 2.0157781 | up | lncRNA |
| ENST00000537659 | RP11-90D4.2 | 0.375408632 | 0.879312727 | 1.2004273 | up | lncRNA |
| ENST00000537724 | RP11-612B6.2 | 0.569236473 | 0.943923733 | 1.0736754 | up | lncRNA |
| ENST00000537780 | H1FX-AS1 | 0.03970518 | 0.454493742 | 1.286425 | up | lncRNA |
| ENST00000537801 | RP11-877E17.2 | 0.395205005 | 0.885711362 | 1.152374 | up | lncRNA |
| ENST00000537961 | RP11-598F7.6 | 0.683120057 | 0.977836377 | 1.0474155 | up | lncRNA |
| ENST00000537965 | SNHG1 | 0.824522326 | 0.993856674 | 1.0220319 | up | lncRNA |
| ENST00000537988 | GOLGA2B | 0.438884593 | 0.903452621 | 1.1647465 | up | lncRNA |
| ENST00000538025 | RP11-313F23.3 | 0.273134706 | 0.832297359 | 1.1787455 | up | lncRNA |
| ENST00000538062 | RP11-273B20.1 | 0.503827616 | 0.92405258 | 1.0637694 | up | lncRNA |
| ENST00000538101 | RP11-864I4.4 | 0.174051748 | 0.736135912 | 1.2485835 | up | lncRNA |
| ENST00000538111 | STARD10-AS1 | 0.449666847 | 0.907426685 | 1.049358 | up | lncRNA |
| ENST00000538113 | RP11-993B23.3 | 0.210335454 | 0.777960284 | 1.5094412 | up | lncRNA |
| ENST00000538202 | RP11-429A20.4 | 0.017786313 | 0.337638939 | 2.2048946 | up | lncRNA |
| ENST00000538306 | PPIAL4E | 0.007895232 | 0.23855236 | 2.5057726 | up | lncRNA |
| ENST00000538380 | AC091878.1 | 0.211744113 | 0.779818197 | 1.5890554 | up | lncRNA |
| ENST00000538405 | RP11-64B16.3 | 0.276325951 | 0.832297359 | 1.1251227 | up | lncRNA |
| ENST00000538624 | RP11-456I15.2 | 0.584325817 | 0.949470507 | 1.0580522 | up | lncRNA |
| ENST00000538693 | RP11-84E24.3 | 0.201011667 | 0.767810338 | 1.5769799 | up | lncRNA |
| ENST00000538710 | RP11-87C12.5 | 0.32031461 | 0.852272022 | 1.0490101 | up | lncRNA |
| ENST00000538717 | GHRLOS2 | 0.729720207 | 0.982972002 | 1.0329286 | up | lncRNA |
| ENST00000538790 | AC156455.1 | 0.619369023 | 0.960028194 | 1.1631481 | up | lncRNA |
| ENST00000538810 | RP11-84E24.3 | 0.007014823 | 0.223418931 | 1.2244877 | up | lncRNA |
| ENST00000538943 | RP5-1154L15.2 | 0.701228258 | 0.980170937 | 1.0268725 | up | lncRNA |
| ENST00000539116 | RP11-221N13.3 | 0.201052286 | 0.767810338 | 2.0899619 | up | lncRNA |
| ENST00000539135 | RP11-264F23.3 | 0.475791244 | 0.917441334 | 1.44218 | up | lncRNA |
| ENST00000539163 | HNF1A-AS1 | 0.816597233 | 0.992651327 | 1.0249076 | up | lncRNA |
| ENST00000539219 | RP11-380J14.1 | 0.125068853 | 0.67731705 | 1.136036 | up | lncRNA |
| ENST00000539315 | RP11-173C20.1 | 0.575437266 | 0.946770106 | 1.1477958 | up | lncRNA |
| ENST00000539328 | RP11-711K1.8 | 0.146500539 | 0.702967479 | 1.8495187 | up | lncRNA |
| ENST00000539391 | RP11-522N14.2 | 0.524405138 | 0.932117592 | 1.3681188 | up | lncRNA |
| ENST00000539446 | RP1-278C19.3 | 0.149364489 | 0.704640207 | 1.1436351 | up | lncRNA |
| ENST00000539453 | BCORP1 | 0.676626303 | 0.976096237 | 1.1281747 | up | lncRNA |
| ENST00000539532 | RP11-117L5.4 | 0.622694034 | 0.960457826 | 1.1117264 | up | lncRNA |
| ENST00000539685 | AP000462.1 | 0.594896442 | 0.951072365 | 1.038164 | up | lncRNA |
| ENST00000539734 | RERG-IT1 | 0.988602419 | 0.99906281 | 1.0024565 | up | lncRNA |
| ENST00000539784 | TSPAN9-IT1 | 0.201249145 | 0.767968021 | 1.2566329 | up | lncRNA |
| ENST00000539795 | RP11-90D4.3 | 0.898612029 | 0.998875101 | 1.0365603 | up | lncRNA |
| ENST00000539808 | RP11-152F13.3 | 0.836508076 | 0.995078046 | 1.0295385 | up | lncRNA |
| ENST00000539963 | RP11-783K16.14 | 0.169648903 | 0.728546096 | 1.2802484 | up | lncRNA |
| ENST00000539987 | RP11-256L11.3 | 0.220897298 | 0.790478735 | 1.1511138 | up | lncRNA |
| ENST00000540024 | RP11-766N7.3 | 0.783172 | 0.99121281 | 1.0282583 | up | lncRNA |
| ENST00000540066 | TOPORS-AS1 | 0.152862952 | 0.709376834 | 1.2763049 | up | lncRNA |
| ENST00000540093 | RP11-885B4.1 | 0.2352167 | 0.804727076 | 1.2441482 | up | lncRNA |
| ENST00000540171 | RP11-982M15.6 | 0.566841849 | 0.943923733 | 2.786613 | up | lncRNA |
| ENST00000540198 | RP11-392P7.6 | 0.909205938 | 0.998875101 | 1.019204 | up | lncRNA |
| ENST00000540250 | RP11-173C20.1 | 0.855217214 | 0.996142678 | 1.0643653 | up | lncRNA |
| ENST00000540307 | RP11-697H9.2 | 0.052063116 | 0.513193572 | 1.4402776 | up | lncRNA |
| ENST00000540392 | RP11-283G6.4 | 1.2744E-05 | 0.01524184 | 14.3503349 | up | lncRNA |
| ENST00000540547 | RP11-707G14.1 | 0.763662805 | 0.989015485 | 1.0425563 | up | lncRNA |
| ENST00000540619 | RP11-319E16.1 | 0.350973432 | 0.86767936 | 1.1242184 | up | lncRNA |
| ENST00000540625 | RP11-283G6.5 | 0.063604324 | 0.537558012 | 1.2910259 | up | lncRNA |
| ENST00000540665 | TTC28-AS1 | 0.275368545 | 0.832297359 | 1.1501555 | up | lncRNA |
| ENST00000540684 | RP11-173C20.2 | 0.014339808 | 0.303995459 | 1.7592495 | up | lncRNA |
| ENST00000540761 | RP11-335O4.1 | 0.293915218 | 0.838742488 | 1.5776939 | up | lncRNA |
| ENST00000540875 | RP11-221N13.4 | 0.492147643 | 0.920779947 | 1.1274566 | up | lncRNA |
| ENST00000540886 | RP11-167N4.2 | 0.021051839 | 0.359097712 | 1.1110545 | up | lncRNA |
| ENST00000540922 | AC114788.2 | 0.103666572 | 0.632846721 | 1.2170828 | up | lncRNA |
| ENST00000541002 | RP11-282O18.3 | 0.940105493 | 0.998875101 | 1.0153491 | up | lncRNA |
| ENST00000541258 | RP13-653N12.2 | 0.245131764 | 0.811577674 | 1.7602754 | up | lncRNA |
| ENST00000541282 | RMST | 0.917323571 | 0.998875101 | 1.0137259 | up | lncRNA |
| ENST00000541385 | LINC00525 | 0.554195577 | 0.943023022 | 1.0992589 | up | lncRNA |
| ENST00000541391 | RP11-221N13.3 | 0.118122435 | 0.66612699 | 1.9489793 | up | lncRNA |
| ENST00000541782 | SCARNA10 | 0.050584909 | 0.508203401 | 1.3498612 | up | lncRNA |
| ENST00000541797 | RP11-749H20.1 | 0.402580347 | 0.889146721 | 1.3238988 | up | lncRNA |
| ENST00000541803 | GNG12-AS1 | 0.427896164 | 0.897780553 | 1.1908202 | up | lncRNA |
| ENST00000541870 | RP11-274J7.2 | 0.37427997 | 0.879312727 | 1.4409128 | up | lncRNA |
| ENST00000541888 | RP1-102E24.8 | 0.987250022 | 0.99906281 | 1.0024727 | up | lncRNA |
| ENST00000541940 | RP11-283G6.5 | 0.0036397 | 0.175033407 | 14.9252549 | up | lncRNA |
| ENST00000542000 | RP11-143E21.7 | 0.861585435 | 0.996142678 | 1.03388 | up | lncRNA |
| ENST00000542070 | LINC00442 | 0.018926859 | 0.343923538 | 1.1871994 | up | lncRNA |
| ENST00000542086 | RP11-877E17.2 | 0.720510355 | 0.98251396 | 1.0421634 | up | lncRNA |
| ENST00000542114 | COL6A4P2 | 0.637380578 | 0.96564589 | 1.1369242 | up | lncRNA |
| ENST00000542121 | RP11-855O10.3 | 0.068437786 | 0.55181901 | 1.1517669 | up | lncRNA |
| ENST00000542172 | RP11-504G3.1 | 0.089135661 | 0.601480869 | 1.2131638 | up | lncRNA |
| ENST00000542314 | RP1-278C19.3 | 0.263636854 | 0.827360264 | 1.2098714 | up | lncRNA |
| ENST00000542331 | RP11-443D10.2 | 0.656657932 | 0.971290366 | 1.0460101 | up | lncRNA |
| ENST00000542435 | RP11-304M2.2 | 0.527725919 | 0.934238148 | 1.2042414 | up | lncRNA |
| ENST00000542477 | RP11-807H22.5 | 0.247115526 | 0.814489937 | 1.3991829 | up | lncRNA |
| ENST00000542489 | RP11-59N23.1 | 0.12211587 | 0.672986132 | 1.1519917 | up | lncRNA |
| ENST00000542598 | RP11-809N8.4 | 0.048347624 | 0.497052359 | 1.5470819 | up | lncRNA |
| ENST00000542660 | RP11-1060J15.4 | 0.719493284 | 0.98251396 | 1.1130604 | up | lncRNA |
| ENST00000543061 | RP11-273B20.3 | 0.424257004 | 0.89750944 | 1.0359589 | up | lncRNA |
| ENST00000543217 | RP11-282O18.3 | 0.887345257 | 0.998875101 | 1.0458544 | up | lncRNA |
| ENST00000543308 | RP11-1038A11.2 | 0.806412158 | 0.992578294 | 1.0441033 | up | lncRNA |
| ENST00000543387 | RP11-473M14.3 | 0.905893658 | 0.998875101 | 1.0303179 | up | lncRNA |
| ENST00000543486 | RP11-667M19.2 | 0.538413305 | 0.936643365 | 1.0712245 | up | lncRNA |
| ENST00000543515 | RP11-392P7.8 | 0.437098452 | 0.902234572 | 1.2489012 | up | lncRNA |
| ENST00000543573 | RP11-712B9.2 | 0.241358124 | 0.809272444 | 1.1634045 | up | lncRNA |
| ENST00000543603 | AP001053.11 | 0.077274213 | 0.574334288 | 1.195697 | up | lncRNA |
| ENST00000543651 | RP11-349K16.1 | 0.015910478 | 0.317148864 | 1.3815758 | up | lncRNA |
| ENST00000543907 | RP11-881M11.4 | 0.930231231 | 0.998875101 | 1.0122243 | up | lncRNA |
| ENST00000543969 | RP11-785H5.2 | 0.718810681 | 0.98251396 | 1.0872802 | up | lncRNA |
| ENST00000544004 | AP000439.2 | 0.243911351 | 0.810806356 | 1.1843844 | up | lncRNA |
| ENST00000544019 | RP11-867G2.6 | 0.999003626 | 0.999535085 | 1.0001012 | up | lncRNA |
| ENST00000544036 | RP11-174M13.2 | 0.940270592 | 0.998875101 | 1.0223668 | up | lncRNA |
| ENST00000544089 | RP11-71J4.2 | 0.194170418 | 0.760765035 | 1.1918062 | up | lncRNA |
| ENST00000544163 | RP5-1096D14.3 | 0.797941196 | 0.992035001 | 1.0767488 | up | lncRNA |
| ENST00000544386 | Z97634.5 | 0.037654035 | 0.443529657 | 1.1039815 | up | lncRNA |
| ENST00000544423 | AC084125.4 | 0.452812705 | 0.909172908 | 1.1277605 | up | lncRNA |
| ENST00000544517 | CACNA1C-AS1 | 0.766015073 | 0.989189555 | 1.0145606 | up | lncRNA |
| ENST00000544553 | RP11-783K16.5 | 0.542195751 | 0.938790368 | 1.0317053 | up | lncRNA |
| ENST00000544556 | NPSR1-AS1 | 0.950469522 | 0.998875101 | 1.012084 | up | lncRNA |
| ENST00000544565 | TP73-AS1 | 0.007341795 | 0.228072384 | 1.2001346 | up | lncRNA |
| ENST00000544674 | RP11-809N8.2 | 0.562021358 | 0.943393901 | 1.1548515 | up | lncRNA |
| ENST00000544704 | RP11-725K16.4 | 0.345585583 | 0.864988541 | 1.2565312 | up | lncRNA |
| ENST00000544842 | RP11-319E16.2 | 0.698466945 | 0.980170937 | 1.1430592 | up | lncRNA |
| ENST00000544890 | RP11-282O18.3 | 0.894834137 | 0.998875101 | 1.0132821 | up | lncRNA |
| ENST00000544939 | RP11-173P15.5 | 0.205003551 | 0.771727492 | 1.1745413 | up | lncRNA |
| ENST00000545158 | RP11-392P7.7 | 0.449002964 | 0.906657331 | 1.160096 | up | lncRNA |
| ENST00000545293 | RP11-324E6.6 | 0.012384658 | 0.283937083 | 1.6443691 | up | lncRNA |
| ENST00000545508 | RP11-173C20.2 | 0.000674494 | 0.094750704 | 30.503191 | up | lncRNA |
| ENST00000545535 | RP11-407A16.7 | 0.432707279 | 0.900592919 | 1.0527554 | up | lncRNA |
| ENST00000545572 | RP11-732A19.9 | 0.826822119 | 0.993856674 | 1.0366386 | up | lncRNA |
| ENST00000545706 | RP11-259O18.4 | 0.157146814 | 0.713725022 | 1.0619534 | up | lncRNA |
| ENST00000545709 | RP11-305O6.3 | 0.10425098 | 0.63448111 | 1.2197316 | up | lncRNA |
| ENST00000545731 | RP11-443D10.3 | 0.289956439 | 0.837264328 | 1.0756266 | up | lncRNA |
| ENST00000545739 | RP11-955H22.3 | 0.897978333 | 0.998875101 | 1.037497 | up | lncRNA |
| ENST00000545750 | RP11-221N13.2 | 0.490128958 | 0.920777644 | 1.3579269 | up | lncRNA |
| ENST00000545775 | ABC12-49244600F4.3 | 0.005295811 | 0.201643161 | 1.4569351 | up | lncRNA |
| ENST00000545800 | RP11-783K16.13 | 0.112476881 | 0.651702944 | 1.0807066 | up | lncRNA |
| ENST00000545820 | GOLGA2B | 0.052400468 | 0.513671228 | 1.8331437 | up | lncRNA |
| ENST00000545853 | RP11-407A16.3 | 0.418739897 | 0.894308781 | 1.0608147 | up | lncRNA |
| ENST00000545904 | RP11-860B13.1 | 0.044280117 | 0.478839027 | 1.3479981 | up | lncRNA |
| ENST00000546065 | AP000593.7 | 0.415357607 | 0.892129957 | 1.107254 | up | lncRNA |
| ENST00000546101 | RP11-685B13.2 | 0.424087621 | 0.897317637 | 1.1121132 | up | lncRNA |
| ENST00000546112 | SRGAP3-AS3 | 0.367386529 | 0.87558047 | 1.2217099 | up | lncRNA |
| ENST00000546134 | RP11-877E17.2 | 0.172627619 | 0.734089358 | 1.0771911 | up | lncRNA |
| ENST00000546135 | RP11-338E21.1 | 0.370807351 | 0.877177739 | 1.1343999 | up | lncRNA |
| ENST00000546214 | RP11-415I12.5 | 0.719354298 | 0.98251396 | 1.0959016 | up | lncRNA |
| ENST00000546259 | RP11-705C15.4 | 0.127007744 | 0.679843078 | 1.4938695 | up | lncRNA |
| ENST00000546273 | RP11-713B9.1 | 0.874110668 | 0.997909851 | 1.0233711 | up | lncRNA |
| ENST00000546324 | RP11-800A3.7 | 0.466953196 | 0.914238486 | 1.0526667 | up | lncRNA |
| ENST00000546413 | GOLGA2B | 0.010237668 | 0.258823411 | 1.346701 | up | lncRNA |
| ENST00000546607 | GLYCAM1 | 0.088509558 | 0.5997129 | 1.1455754 | up | lncRNA |
| ENST00000546674 | MIR3179-1 | 0.91104412 | 0.998875101 | 1.0056048 | up | lncRNA |
| ENST00000546678 | RP11-570L15.2 | 0.806580094 | 0.992578294 | 1.0227829 | up | lncRNA |
| ENST00000546696 | RP11-116D17.2 | 0.642664277 | 0.967175883 | 1.0867312 | up | lncRNA |
| ENST00000546734 | CTD-2314B22.1 | 0.396739277 | 0.886797236 | 1.0794834 | up | lncRNA |
| ENST00000546798 | HOXD-AS1 | 0.196108662 | 0.762235583 | 1.141862 | up | lncRNA |
| ENST00000546804 | RP1-228P16.3 | 0.914212171 | 0.998875101 | 1.0224571 | up | lncRNA |
| ENST00000546894 | RP11-793H13.4 | 0.03410288 | 0.42757611 | 1.1227707 | up | lncRNA |
| ENST00000546911 | RP11-70F11.11 | 0.746547583 | 0.985094857 | 1.0361591 | up | lncRNA |
| ENST00000546959 | CTD-2314B22.3 | 0.598135963 | 0.952424484 | 1.1189198 | up | lncRNA |
| ENST00000547004 | RP11-554E23.2 | 0.779078066 | 0.99121281 | 1.0598383 | up | lncRNA |
| ENST00000547009 | RP11-762I7.4 | 0.052737317 | 0.514888421 | 1.2248231 | up | lncRNA |
| ENST00000547089 | RP11-171L9.1 | 0.196241672 | 0.762235583 | 1.2368179 | up | lncRNA |
| ENST00000547093 | CTD-2213F21.4 | 0.302267652 | 0.843668873 | 1.0609129 | up | lncRNA |
| ENST00000547174 | RP3-416H24.4 | 0.856087023 | 0.996142678 | 1.0174827 | up | lncRNA |
| ENST00000547177 | RP11-968A15.2 | 0.636771274 | 0.96564589 | 1.2627369 | up | lncRNA |
| ENST00000547387 | RP11-386G11.10 | 0.29915357 | 0.842046751 | 1.1920008 | up | lncRNA |
| ENST00000547452 | RP11-554D14.6 | 0.527836087 | 0.934238148 | 1.272492 | up | lncRNA |
| ENST00000547607 | RP1-46F2.2 | 0.121782148 | 0.672928817 | 1.1189895 | up | lncRNA |
| ENST00000547626 | RP11-493L12.2 | 0.954941779 | 0.998875101 | 1.0158024 | up | lncRNA |
| ENST00000547712 | RP11-386G11.10 | 0.1940445 | 0.760765035 | 1.1569335 | up | lncRNA |
| ENST00000547748 | RP11-493L12.3 | 0.127185896 | 0.679843078 | 1.2684825 | up | lncRNA |
| ENST00000547845 | RP11-887P2.1 | 0.426100456 | 0.897780553 | 1.0822659 | up | lncRNA |
| ENST00000547866 | RP11-386G11.5 | 0.266624516 | 0.828881561 | 1.2513921 | up | lncRNA |
| ENST00000548027 | RP11-244H18.2 | 0.671665555 | 0.974377541 | 1.046398 | up | lncRNA |
| ENST00000548030 | RP11-386G11.8 | 0.288341304 | 0.837198948 | 1.0688537 | up | lncRNA |
| ENST00000548141 | CTD-2311B13.3 | 0.496860685 | 0.921717611 | 1.0653895 | up | lncRNA |
| ENST00000548149 | RP11-386G11.10 | 0.172713057 | 0.734235127 | 1.2627115 | up | lncRNA |
| ENST00000548172 | RP11-753N8.1 | 0.065577488 | 0.542942809 | 1.3160385 | up | lncRNA |
| ENST00000548266 | RP11-324P9.1 | 0.86184364 | 0.996142678 | 1.0696779 | up | lncRNA |
| ENST00000548268 | MIR3180-1 | 0.151116594 | 0.70579658 | 1.1298095 | up | lncRNA |
| ENST00000548271 | AL589743.1 | 0.84088462 | 0.995583664 | 1.1183718 | up | lncRNA |
| ENST00000548328 | NOVA1-AS1 | 0.752844832 | 0.986562183 | 1.1285291 | up | lncRNA |
| ENST00000548368 | RP1-46F2.2 | 0.00810907 | 0.241957124 | 1.1032801 | up | lncRNA |
| ENST00000548404 | RP11-175P13.3 | 0.050421347 | 0.508001392 | 1.2543102 | up | lncRNA |
| ENST00000548416 | CTD-2314B22.1 | 0.386764986 | 0.884034255 | 1.2365501 | up | lncRNA |
| ENST00000548419 | RP11-554D14.1 | 0.330207861 | 0.855595455 | 1.198392 | up | lncRNA |
| ENST00000548473 | RP11-128P10.1 | 0.355142964 | 0.870470894 | 1.3233346 | up | lncRNA |
| ENST00000548631 | RP11-1103G16.1 | 0.831827567 | 0.994074191 | 1.0528149 | up | lncRNA |
| ENST00000548666 | RP3-405J10.2 | 0.663269954 | 0.971748711 | 1.0595906 | up | lncRNA |
| ENST00000548756 | CTD-2252P21.1 | 0.369920749 | 0.87681298 | 1.2497629 | up | lncRNA |
| ENST00000548782 | RP11-175P13.3 | 0.08043022 | 0.582052437 | 1.2200653 | up | lncRNA |
| ENST00000548846 | RP3-473L9.4 | 0.069356914 | 0.554855315 | 1.4083673 | up | lncRNA |
| ENST00000548875 | CTD-2314B22.3 | 0.592812781 | 0.950912706 | 1.0958977 | up | lncRNA |
| ENST00000548897 | RP11-642P15.1 | 0.718929786 | 0.98251396 | 1.1209407 | up | lncRNA |
| ENST00000548903 | CTD-2314B22.3 | 0.872155049 | 0.997909851 | 1.0483904 | up | lncRNA |
| ENST00000548955 | RP11-620J15.2 | 0.375577272 | 0.879312727 | 1.1753781 | up | lncRNA |
| ENST00000548963 | RP11-754N21.1 | 0.90884442 | 0.998875101 | 1.0765258 | up | lncRNA |
| ENST00000548964 | AL512355.4 | 0.535403337 | 0.936003031 | 1.0434359 | up | lncRNA |
| ENST00000549032 | AC096558.1 | 0.601714753 | 0.95454959 | 1.1025479 | up | lncRNA |
| ENST00000549103 | RP11-136F16.1 | 0.214574054 | 0.781610853 | 1.2747811 | up | lncRNA |
| ENST00000549208 | RP11-754I20.1 | 0.160603435 | 0.716150139 | 1.1163418 | up | lncRNA |
| ENST00000549241 | RP11-449P15.1 | 0.069546517 | 0.55572632 | 1.1377574 | up | lncRNA |
| ENST00000549329 | HOXD-AS1 | 0.596729253 | 0.952007364 | 1.0589741 | up | lncRNA |
| ENST00000549330 | RP11-626P14.1 | 0.961678462 | 0.998875101 | 1.0166052 | up | lncRNA |
| ENST00000549360 | CTD-2251F13.1 | 0.069149431 | 0.554431642 | 1.0537184 | up | lncRNA |
| ENST00000549364 | RP11-804F13.1 | 0.472024648 | 0.916418344 | 1.1562642 | up | lncRNA |
| ENST00000549386 | RP1-292L20.1 | 0.966203192 | 0.998875101 | 1.0048893 | up | lncRNA |
| ENST00000549403 | RP11-210N13.1 | 0.851736613 | 0.995900317 | 1.0155663 | up | lncRNA |
| ENST00000549460 | RP11-588H23.3 | 0.748361823 | 0.985094857 | 1.110392 | up | lncRNA |
| ENST00000549470 | RP11-753N8.1 | 0.181648398 | 0.747757045 | 1.169756 | up | lncRNA |
| ENST00000549477 | RP11-571M6.7 | 0.937699158 | 0.998875101 | 1.012039 | up | lncRNA |
| ENST00000549484 | AL589743.1 | 0.881527336 | 0.998875101 | 1.0660896 | up | lncRNA |
| ENST00000549515 | RP11-260G13.1 | 0.520953222 | 0.932117592 | 1.1393191 | up | lncRNA |
| ENST00000549516 | RP11-386G11.3 | 0.132982323 | 0.688794697 | 1.1061866 | up | lncRNA |
| ENST00000549616 | RP11-588H23.3 | 0.293198539 | 0.838358604 | 1.2388664 | up | lncRNA |
| ENST00000549651 | RP11-611E13.2 | 0.750123962 | 0.98551982 | 1.0615809 | up | lncRNA |
| ENST00000549659 | NKX2-2-AS1 | 0.156225574 | 0.711341825 | 1.9136252 | up | lncRNA |
| ENST00000549666 | CITF22-92A6.1 | 0.189540318 | 0.756884941 | 1.5051046 | up | lncRNA |
| ENST00000549723 | RP11-887P2.1 | 0.950915492 | 0.998875101 | 1.0106006 | up | lncRNA |
| ENST00000549807 | RP11-818F20.5 | 0.956952699 | 0.998875101 | 1.0045013 | up | lncRNA |
| ENST00000549830 | LINC00592 | 0.061656241 | 0.534514328 | 1.1863244 | up | lncRNA |
| ENST00000549860 | RP11-977G19.11 | 0.767196608 | 0.989189555 | 1.0265973 | up | lncRNA |
| ENST00000549930 | RP11-511B23.2 | 0.07168427 | 0.560238874 | 1.1902619 | up | lncRNA |
| ENST00000550042 | RP1-34H18.1 | 0.587058515 | 0.950589384 | 1.276456 | up | lncRNA |
| ENST00000550116 | RP11-983P16.2 | 0.59209862 | 0.950912706 | 1.2370849 | up | lncRNA |
| ENST00000550185 | RP11-626I20.3 | 0.944460651 | 0.998875101 | 1.0082565 | up | lncRNA |
| ENST00000550206 | RP11-780K2.1 | 0.924280776 | 0.998875101 | 1.0120353 | up | lncRNA |
| ENST00000550263 | RP11-680A11.5 | 0.358755125 | 0.873279096 | 1.0634094 | up | lncRNA |
| ENST00000550290 | RP11-631N16.2 | 0.912325692 | 0.998875101 | 1.0617131 | up | lncRNA |
| ENST00000550426 | RP11-493L12.2 | 0.582788455 | 0.94928838 | 1.0780538 | up | lncRNA |
| ENST00000550468 | RP11-161H23.5 | 0.106889646 | 0.639107828 | 1.1914378 | up | lncRNA |
| ENST00000550506 | RP11-362A1.1 | 0.957067041 | 0.998875101 | 1.0051614 | up | lncRNA |
| ENST00000550530 | RP11-469H8.6 | 0.453243678 | 0.909402105 | 1.0625125 | up | lncRNA |
| ENST00000550644 | RP11-133N21.10 | 0.001277981 | 0.124738056 | 3.0800112 | up | lncRNA |
| ENST00000550687 | RP11-1060G2.1 | 0.873693141 | 0.997909851 | 1.0127644 | up | lncRNA |
| ENST00000550759 | RP11-74K11.2 | 0.052658335 | 0.514646985 | 1.3839165 | up | lncRNA |
| ENST00000550805 | CTD-2314B22.3 | 0.854579078 | 0.996142678 | 1.0391465 | up | lncRNA |
| ENST00000550827 | CTD-2384A14.1 | 0.821147352 | 0.993333549 | 1.0536714 | up | lncRNA |
| ENST00000550886 | RP11-175P13.2 | 0.280861919 | 0.832297359 | 1.0579915 | up | lncRNA |
| ENST00000550892 | CTD-2311B13.7 | 0.931640811 | 0.998875101 | 1.0192339 | up | lncRNA |
| ENST00000550926 | RP11-56G10.2 | 0.056824043 | 0.523711112 | 1.1005369 | up | lncRNA |
| ENST00000550947 | RP11-603J24.5 | 0.257789508 | 0.823250249 | 1.1051516 | up | lncRNA |
| ENST00000551021 | RP11-96H19.1 | 0.793503129 | 0.991420185 | 1.0695994 | up | lncRNA |
| ENST00000551029 | RP11-74K11.2 | 0.659054155 | 0.971704562 | 1.0302356 | up | lncRNA |
| ENST00000551040 | RP11-562L8.1 | 0.370505378 | 0.877177739 | 1.5896521 | up | lncRNA |
| ENST00000551082 | RP11-20E24.1 | 0.524290819 | 0.932117592 | 1.0840814 | up | lncRNA |
| ENST00000551089 | RP11-641A6.2 | 0.431414992 | 0.899690201 | 1.0612577 | up | lncRNA |
| ENST00000551125 | RP1-267L14.3 | 0.847622287 | 0.995583664 | 1.0414929 | up | lncRNA |
| ENST00000551144 | RP11-244H18.3 | 0.845853808 | 0.995583664 | 1.0342925 | up | lncRNA |
| ENST00000551146 | RP11-70F11.6 | 0.936564382 | 0.998875101 | 1.0170293 | up | lncRNA |
| ENST00000551152 | RP11-396F22.1 | 0.13695713 | 0.693854474 | 1.5128142 | up | lncRNA |
| ENST00000551187 | RP11-61A14.1 | 0.810549229 | 0.99257857 | 1.0781725 | up | lncRNA |
| ENST00000551271 | RP11-521B24.3 | 0.030100193 | 0.414324281 | 1.1882779 | up | lncRNA |
| ENST00000551284 | RP3-405J10.4 | 0.076205405 | 0.569253528 | 2.0165072 | up | lncRNA |
| ENST00000551287 | RP11-776A13.3 | 0.559995482 | 0.943393901 | 1.1822205 | up | lncRNA |
| ENST00000551395 | RP11-966I7.1 | 0.907363525 | 0.998875101 | 1.0232797 | up | lncRNA |
| ENST00000551408 | RP11-108M12.3 | 0.537363512 | 0.936418354 | 1.0727384 | up | lncRNA |
| ENST00000551496 | RP11-386G11.10 | 0.016556467 | 0.325245053 | 1.4828495 | up | lncRNA |
| ENST00000551571 | RP11-121E16.1 | 0.904569125 | 0.998875101 | 1.0243646 | up | lncRNA |
| ENST00000551610 | RP11-167N24.3 | 0.154880096 | 0.711341825 | 1.4099873 | up | lncRNA |
| ENST00000551844 | RP11-394J1.2 | 0.005812335 | 0.208598143 | 1.3370512 | up | lncRNA |
| ENST00000551847 | RP11-722P11.4 | 0.046263718 | 0.485742152 | 1.3581018 | up | lncRNA |
| ENST00000551893 | RP11-536G4.2 | 0.184290368 | 0.751257244 | 1.1890988 | up | lncRNA |
| ENST00000551905 | RP11-341G23.2 | 0.001904543 | 0.141494996 | 1.5572828 | up | lncRNA |
| ENST00000551982 | RP11-438N16.1 | 0.33341238 | 0.857704692 | 1.1826312 | up | lncRNA |
| ENST00000552015 | MIR3180-4 | 0.326331402 | 0.854556781 | 1.1783646 | up | lncRNA |
| ENST00000552063 | RP11-493L12.4 | 0.597502213 | 0.952287813 | 1.0540055 | up | lncRNA |
| ENST00000552070 | RP11-244H18.2 | 0.255886285 | 0.822133742 | 1.3672788 | up | lncRNA |
| ENST00000552096 | RP11-129J12.2 | 0.867493002 | 0.996977864 | 1.026064 | up | lncRNA |
| ENST00000552114 | RP11-542H1.1 | 0.898952667 | 0.998875101 | 1.0215302 | up | lncRNA |
| ENST00000552154 | RP11-554D14.7 | 0.901740882 | 0.998875101 | 1.0468033 | up | lncRNA |
| ENST00000552156 | HOXD-AS1 | 0.725757048 | 0.98251396 | 1.0336399 | up | lncRNA |
| ENST00000552284 | RP11-386G11.5 | 0.203476337 | 0.770729053 | 1.1717888 | up | lncRNA |
| ENST00000552290 | RP11-114H23.1 | 0.52038391 | 0.93173 | 1.1416864 | up | lncRNA |
| ENST00000552324 | RP11-320P7.2 | 0.567493733 | 0.943923733 | 1.2387845 | up | lncRNA |
| ENST00000552353 | RP11-511B23.1 | 0.588632533 | 0.950589384 | 1.2929088 | up | lncRNA |
| ENST00000552367 | RP11-290L1.3 | 0.395605251 | 0.885711362 | 1.1764497 | up | lncRNA |
| ENST00000552378 | RP11-316A16.1 | 0.523770221 | 0.932117592 | 1.5853013 | up | lncRNA |
| ENST00000552411 | RMST | 0.361624737 | 0.873683336 | 1.2597064 | up | lncRNA |
| ENST00000552415 | RP11-482D24.3 | 0.149304514 | 0.704640207 | 1.3715705 | up | lncRNA |
| ENST00000552466 | RP11-114H23.1 | 0.294172391 | 0.838742488 | 1.1014206 | up | lncRNA |
| ENST00000552486 | RP11-651L5.2 | 0.869284702 | 0.997455886 | 1.0223149 | up | lncRNA |
| ENST00000552576 | RP11-644F5.11 | 0.791322719 | 0.991420185 | 1.0156483 | up | lncRNA |
| ENST00000552615 | RP11-1016B18.1 | 0.052990361 | 0.515925762 | 1.3520756 | up | lncRNA |
| ENST00000552628 | RP11-70F11.11 | 0.204679127 | 0.771727492 | 1.1739831 | up | lncRNA |
| ENST00000552675 | RP11-521B24.5 | 0.572421238 | 0.945484468 | 1.0568054 | up | lncRNA |
| ENST00000552685 | AC004790.2 | 0.008824597 | 0.248217958 | 1.2151923 | up | lncRNA |
| ENST00000552712 | RP11-630C16.1 | 0.411061327 | 0.890876411 | 1.1687173 | up | lncRNA |
| ENST00000552753 | MIR3179-3 | 0.774862223 | 0.990435061 | 1.0299194 | up | lncRNA |
| ENST00000552778 | RP11-981P6.1 | 0.055018816 | 0.519180132 | 3.5429429 | up | lncRNA |
| ENST00000552835 | RP11-486A14.2 | 0.448528676 | 0.906276723 | 1.2249777 | up | lncRNA |
| ENST00000552893 | RP11-386G11.10 | 0.144804189 | 0.69994374 | 1.256552 | up | lncRNA |
| ENST00000553045 | RP1-197B17.3 | 0.934368807 | 0.998875101 | 1.0149867 | up | lncRNA |
| ENST00000553105 | RP11-996F15.2 | 0.933672488 | 0.998875101 | 1.031841 | up | lncRNA |
| ENST00000553163 | RP11-536G4.2 | 0.025612028 | 0.38237988 | 1.3140304 | up | lncRNA |
| ENST00000553174 | RP11-386G11.8 | 0.066451955 | 0.545605528 | 1.1726143 | up | lncRNA |
| ENST00000553177 | RP1-46F2.2 | 0.707798953 | 0.981606358 | 1.0767401 | up | lncRNA |
| ENST00000553227 | RP11-23J18.1 | 0.765639594 | 0.989189555 | 1.073236 | up | lncRNA |
| ENST00000553270 | RP11-1029J19.4 | 0.406566662 | 0.889488526 | 1.1065995 | up | lncRNA |
| ENST00000553288 | RP11-47I22.2 | 0.961822795 | 0.998875101 | 1.0069597 | up | lncRNA |
| ENST00000553318 | RP11-1029J19.2 | 0.796597667 | 0.991448346 | 1.2653807 | up | lncRNA |
| ENST00000553344 | CTD-3051D23.4 | 0.376580507 | 0.879423449 | 1.5678699 | up | lncRNA |
| ENST00000553382 | RP11-1084I9.3 | 0.250288891 | 0.81695387 | 1.1768148 | up | lncRNA |
| ENST00000553421 | MEG8 | 0.048823719 | 0.500529978 | 1.1755872 | up | lncRNA |
| ENST00000553433 | RP11-982M15.7 | 0.367151603 | 0.87558047 | 1.0819787 | up | lncRNA |
| ENST00000553465 | MEG8 | 0.746556892 | 0.985094857 | 1.0430528 | up | lncRNA |
| ENST00000553468 | RP11-384J4.2 | 0.26580099 | 0.828758593 | 1.3039605 | up | lncRNA |
| ENST00000553478 | RP11-266O8.1 | 0.308166085 | 0.847137912 | 1.5683696 | up | lncRNA |
| ENST00000553496 | RP11-300J18.3 | 0.087896967 | 0.598480971 | 1.3203472 | up | lncRNA |
| ENST00000553537 | RP11-472N19.3 | 0.958876837 | 0.998875101 | 1.0101083 | up | lncRNA |
| ENST00000553559 | SNHG10 | 0.600762548 | 0.95398806 | 1.0609229 | up | lncRNA |
| ENST00000553568 | RP11-203M5.6 | 0.750866224 | 0.98577793 | 1.035037 | up | lncRNA |
| ENST00000553760 | RP11-406A9.2 | 0.713599824 | 0.98251396 | 1.0911641 | up | lncRNA |
| ENST00000553762 | RP11-112J1.2 | 0.447197309 | 0.905615479 | 1.1475323 | up | lncRNA |
| ENST00000553767 | RP11-361H10.2 | 0.519895934 | 0.93173 | 1.1723797 | up | lncRNA |
| ENST00000553860 | RP11-638I2.3 | 0.719167184 | 0.98251396 | 1.0312413 | up | lncRNA |
| ENST00000553869 | AC016683.6 | 0.358221478 | 0.872424004 | 1.229795 | up | lncRNA |
| ENST00000553921 | RP11-300J18.1 | 0.059372408 | 0.530697632 | 1.1974685 | up | lncRNA |
| ENST00000553929 | RP11-300J18.3 | 0.435988354 | 0.901926599 | 1.0962789 | up | lncRNA |
| ENST00000553944 | ACTN1-AS1 | 0.065867894 | 0.543449026 | 2.8032861 | up | lncRNA |
| ENST00000553961 | ACTN1-AS1 | 0.183223817 | 0.750893267 | 1.0807116 | up | lncRNA |
| ENST00000553963 | RP11-185P18.2 | 0.411959364 | 0.890876411 | 1.0841978 | up | lncRNA |
| ENST00000553991 | RP11-488C13.4 | 0.008382599 | 0.244110885 | 1.1449575 | up | lncRNA |
| ENST00000554008 | RP11-486O13.2 | 0.392788141 | 0.885711362 | 1.0590497 | up | lncRNA |
| ENST00000554010 | RP11-124D2.3 | 0.122555466 | 0.673398182 | 1.1749498 | up | lncRNA |
| ENST00000554016 | MEG9 | 0.198588282 | 0.765278337 | 1.1127141 | up | lncRNA |
| ENST00000554022 | RP11-973D8.4 | 0.545215324 | 0.939697392 | 1.0730196 | up | lncRNA |
| ENST00000554032 | RP6-65G23.3 | 0.508045644 | 0.926253949 | 1.0482765 | up | lncRNA |
| ENST00000554036 | DHRS4-AS1 | 0.032541176 | 0.423455575 | 1.0964785 | up | lncRNA |
| ENST00000554053 | RP11-973D8.3 | 0.198496178 | 0.765216944 | 1.0910733 | up | lncRNA |
| ENST00000554055 | RP11-841O20.2 | 0.721841261 | 0.98251396 | 1.0252163 | up | lncRNA |
| ENST00000554058 | RP11-488C13.5 | 0.215881714 | 0.783396803 | 1.1728342 | up | lncRNA |
| ENST00000554149 | RP11-1085N6.5 | 0.397750776 | 0.887348439 | 1.118184 | up | lncRNA |
| ENST00000554181 | RP11-736P16.1 | 0.580971736 | 0.949239022 | 1.0574248 | up | lncRNA |
| ENST00000554197 | RP11-76E12.1 | 0.183192105 | 0.750893267 | 1.2460563 | up | lncRNA |
| ENST00000554205 | RP11-325D8.1 | 0.144264077 | 0.699853429 | 1.2321162 | up | lncRNA |
| ENST00000554211 | RP11-799P8.1 | 0.732470607 | 0.983763148 | 1.1229934 | up | lncRNA |
| ENST00000554221 | LINC00520 | 0.524319967 | 0.932117592 | 1.2540559 | up | lncRNA |
| ENST00000554286 | RP11-903H12.3 | 0.498228005 | 0.921805853 | 1.0567287 | up | lncRNA |
| ENST00000554297 | SPATA1 | 0.002831565 | 0.155703545 | 2.0599686 | up | lncRNA |
| ENST00000554321 | RP11-185P18.1 | 0.401547049 | 0.8890764 | 1.1502453 | up | lncRNA |
| ENST00000554369 | AL132709.5 | 0.926061927 | 0.998875101 | 1.040553 | up | lncRNA |
| ENST00000554407 | RP11-770J1.3 | 0.158804382 | 0.714694414 | 1.0810616 | up | lncRNA |
| ENST00000554430 | RP11-950C14.3 | 0.035356447 | 0.43149296 | 1.3719547 | up | lncRNA |
| ENST00000554433 | RP11-300J18.2 | 0.193418174 | 0.760765035 | 2.2355144 | up | lncRNA |
| ENST00000554440 | RP11-763K15.1 | 0.706267197 | 0.98102817 | 1.0663234 | up | lncRNA |
| ENST00000554454 | RP11-973N13.3 | 0.250887589 | 0.817049847 | 1.2305046 | up | lncRNA |
| ENST00000554458 | RP11-813I20.2 | 0.449780985 | 0.907529411 | 1.0951779 | up | lncRNA |
| ENST00000554485 | AL132709.5 | 0.561166487 | 0.943393901 | 1.2576223 | up | lncRNA |
| ENST00000554519 | RP11-1152H15.1 | 0.934606463 | 0.998875101 | 1.0052077 | up | lncRNA |
| ENST00000554526 | RP11-388E23.2 | 0.943801906 | 0.998875101 | 1.0371782 | up | lncRNA |
| ENST00000554540 | CTD-2644I21.1 | 0.824457023 | 0.993856674 | 1.0069925 | up | lncRNA |
| ENST00000554583 | RP11-398J10.2 | 0.103339812 | 0.632351488 | 1.1678187 | up | lncRNA |
| ENST00000554634 | RP1-261D10.2 | 0.897179833 | 0.998875101 | 1.0121593 | up | lncRNA |
| ENST00000554649 | RP11-255M2.1 | 0.097583238 | 0.62265608 | 1.1636661 | up | lncRNA |
| ENST00000554653 | RP11-862G15.1 | 0.326909929 | 0.854556781 | 1.2834153 | up | lncRNA |
| ENST00000554678 | RP11-203M5.8 | 0.910771254 | 0.998875101 | 1.0146504 | up | lncRNA |
| ENST00000554687 | CTD-2058B24.2 | 0.55981554 | 0.943393901 | 1.1452919 | up | lncRNA |
| ENST00000554693 | AL132709.8 | 0.587257853 | 0.950589384 | 1.0707975 | up | lncRNA |
| ENST00000554701 | CHEK2P2 | 0.200779271 | 0.767810338 | 1.0707226 | up | lncRNA |
| ENST00000554733 | RP11-998D10.7 | 0.15368287 | 0.711043375 | 1.1178904 | up | lncRNA |
| ENST00000554838 | RP11-404P21.2 | 0.14397642 | 0.699853429 | 1.1276843 | up | lncRNA |
| ENST00000554852 | MEG8 | 0.023416051 | 0.369670635 | 1.2999752 | up | lncRNA |
| ENST00000554859 | CTD-2017C7.2 | 0.656935675 | 0.971290366 | 1.118078 | up | lncRNA |
| ENST00000554889 | RP11-185P18.2 | 0.557681014 | 0.943023022 | 1.055442 | up | lncRNA |
| ENST00000554921 | RP11-1112J20.2 | 0.126317224 | 0.678900542 | 1.0760806 | up | lncRNA |
| ENST00000554926 | RP11-187O7.3 | 0.680945071 | 0.977096946 | 1.0553479 | up | lncRNA |
| ENST00000554939 | RP3-449M8.3 | 0.052286534 | 0.51320639 | 1.1340807 | up | lncRNA |
| ENST00000555016 | RP11-203M5.6 | 0.748906294 | 0.985094857 | 1.0549958 | up | lncRNA |
| ENST00000555103 | AL132709.1 | 0.527394847 | 0.93423486 | 1.1179783 | up | lncRNA |
| ENST00000555174 | DIO3OS | 0.818209945 | 0.992651327 | 1.0446949 | up | lncRNA |
| ENST00000555250 | RP11-909M7.3 | 0.680110745 | 0.976675999 | 1.1135984 | up | lncRNA |
| ENST00000555299 | RP11-862G15.2 | 0.524028541 | 0.932117592 | 1.1078794 | up | lncRNA |
| ENST00000555325 | CTD-2313J17.1 | 0.031963289 | 0.420473996 | 1.5457005 | up | lncRNA |
| ENST00000555332 | CTD-2536I1.1 | 0.309260586 | 0.847852517 | 1.1700862 | up | lncRNA |
| ENST00000555344 | CTC-349C3.2 | 0.65241763 | 0.969927925 | 1.0614805 | up | lncRNA |
| ENST00000555350 | RP11-572M18.1 | 0.121716121 | 0.672928817 | 1.0837867 | up | lncRNA |
| ENST00000555377 | RP11-72M17.1 | 0.530686726 | 0.934908142 | 1.1165967 | up | lncRNA |
| ENST00000555383 | RP11-404P21.5 | 0.001046509 | 0.115534581 | 4.7653483 | up | lncRNA |
| ENST00000555403 | RP11-247L20.4 | 0.879774182 | 0.998682284 | 1.0287326 | up | lncRNA |
| ENST00000555406 | CTD-3098H1.2 | 0.316199986 | 0.851223284 | 1.4351211 | up | lncRNA |
| ENST00000555407 | RP11-356K23.1 | 0.364517818 | 0.874818743 | 1.094008 | up | lncRNA |
| ENST00000555413 | RP11-1078H9.1 | 0.550435858 | 0.942167708 | 1.0740434 | up | lncRNA |
| ENST00000555435 | RP11-203M5.7 | 0.027071517 | 0.391663721 | 1.7044435 | up | lncRNA |
| ENST00000555442 | RP11-369C8.1 | 0.001449347 | 0.132490657 | 3.8956527 | up | lncRNA |
| ENST00000555460 | AE000661.50 | 0.262188235 | 0.82544125 | 1.4301115 | up | lncRNA |
| ENST00000555580 | RP11-597A11.6 | 0.826917333 | 0.993856674 | 1.0230007 | up | lncRNA |
| ENST00000555599 | AL132709.1 | 0.436319854 | 0.901926599 | 1.0709577 | up | lncRNA |
| ENST00000555649 | RP11-255G12.2 | 0.810617871 | 0.99257857 | 1.0413962 | up | lncRNA |
| ENST00000555689 | RP11-589M4.1 | 0.674504771 | 0.975659391 | 1.1278628 | up | lncRNA |
| ENST00000555766 | AC016683.6 | 0.028994067 | 0.407241179 | 1.3779666 | up | lncRNA |
| ENST00000555796 | CTD-2555O16.2 | 0.646957159 | 0.969646614 | 1.0559927 | up | lncRNA |
| ENST00000555801 | RP11-255G12.1 | 0.402705185 | 0.889146721 | 1.1150318 | up | lncRNA |
| ENST00000555853 | RP11-471B22.2 | 0.008967295 | 0.249900233 | 2.0511511 | up | lncRNA |
| ENST00000555875 | RP11-543C4.3 | 0.047658784 | 0.492794577 | 1.268604 | up | lncRNA |
| ENST00000555968 | RP11-66N24.3 | 0.865061146 | 0.996767702 | 1.0352692 | up | lncRNA |
| ENST00000555972 | RP3-414A15.2 | 0.682161157 | 0.977676223 | 1.0623573 | up | lncRNA |
| ENST00000556019 | RP11-58E21.5 | 0.12138529 | 0.672928817 | 1.2932231 | up | lncRNA |
| ENST00000556050 | SPAG5-AS1 | 0.299794085 | 0.8422517 | 1.1569235 | up | lncRNA |
| ENST00000556070 | AC016683.6 | 0.655660191 | 0.971258623 | 1.3898175 | up | lncRNA |
| ENST00000556073 | RP11-614O9.1 | 0.087758718 | 0.598469221 | 1.1831743 | up | lncRNA |
| ENST00000556080 | RP11-362L22.1 | 0.291558393 | 0.837264328 | 1.1205676 | up | lncRNA |
| ENST00000556099 | AL132709.5 | 0.700542011 | 0.980170937 | 1.0650723 | up | lncRNA |
| ENST00000556116 | RP11-566K8.2 | 0.391578593 | 0.885711362 | 1.055198 | up | lncRNA |
| ENST00000556130 | RP11-58E21.4 | 0.676434318 | 0.976096237 | 1.1831686 | up | lncRNA |
| ENST00000556136 | AL132709.1 | 0.867356623 | 0.996900957 | 1.0820827 | up | lncRNA |
| ENST00000556183 | RP11-665C16.6 | 0.375304684 | 0.879312727 | 1.0482964 | up | lncRNA |
| ENST00000556266 | DIO3OS | 0.437412018 | 0.902506883 | 1.0884273 | up | lncRNA |
| ENST00000556290 | FAM181A-AS1 | 0.324716125 | 0.854254771 | 1.1929532 | up | lncRNA |
| ENST00000556301 | CTD-2325P2.4 | 0.918202022 | 0.998875101 | 1.0266535 | up | lncRNA |
| ENST00000556332 | RP11-894P9.2 | 0.299924199 | 0.8422517 | 1.4797506 | up | lncRNA |
| ENST00000556346 | RP11-1070N10.4 | 0.05580847 | 0.521270634 | 1.1917268 | up | lncRNA |
| ENST00000556361 | RP11-72M17.1 | 0.772081798 | 0.989986416 | 1.0874612 | up | lncRNA |
| ENST00000556407 | MEG3 | 0.035043789 | 0.43097555 | 1.0770899 | up | lncRNA |
| ENST00000556434 | RP11-58E21.4 | 0.828163307 | 0.994074191 | 1.0652301 | up | lncRNA |
| ENST00000556458 | RP11-638I2.6 | 0.105704772 | 0.63605775 | 1.3262459 | up | lncRNA |
| ENST00000556475 | MEG8 | 0.300467201 | 0.842264993 | 1.0992169 | up | lncRNA |
| ENST00000556479 | RP11-255G12.1 | 0.007138524 | 0.224630786 | 2.4945484 | up | lncRNA |
| ENST00000556578 | RP4-647C14.3 | 0.202343044 | 0.769482611 | 1.5185781 | up | lncRNA |
| ENST00000556583 | RP11-770J1.3 | 0.923703099 | 0.998875101 | 1.0236516 | up | lncRNA |
| ENST00000556634 | RP11-973N13.3 | 0.279107551 | 0.832297359 | 1.0982348 | up | lncRNA |
| ENST00000556652 | CTD-2207P18.2 | 0.000372661 | 0.084119144 | 2.0567796 | up | lncRNA |
| ENST00000556653 | CTD-2134A5.3 | 0.202854692 | 0.770000141 | 1.1501214 | up | lncRNA |
| ENST00000556693 | RP11-671J11.6 | 0.611344662 | 0.957448851 | 1.2774037 | up | lncRNA |
| ENST00000556705 | RP11-85K15.2 | 0.804364338 | 0.992578294 | 1.068645 | up | lncRNA |
| ENST00000556720 | AL132709.5 | 0.977618144 | 0.998875101 | 1.0054588 | up | lncRNA |
| ENST00000556763 | RP11-76E17.4 | 0.031179081 | 0.417820172 | 1.5684126 | up | lncRNA |
| ENST00000556781 | RP11-7F17.3 | 0.055425924 | 0.520364745 | 1.4776086 | up | lncRNA |
| ENST00000556926 | RP11-356K23.2 | 0.376482575 | 0.879423449 | 1.5893418 | up | lncRNA |
| ENST00000556987 | LINC00524 | 0.363011878 | 0.874234597 | 1.1184516 | up | lncRNA |
| ENST00000557025 | RP11-100M12.1 | 0.54105018 | 0.937933588 | 1.1306507 | up | lncRNA |
| ENST00000557062 | RP11-816J8.1 | 0.273803547 | 0.832297359 | 1.1640565 | up | lncRNA |
| ENST00000557160 | RP11-831F12.2 | 0.081412562 | 0.584216547 | 1.1356605 | up | lncRNA |
| ENST00000557322 | CTD-2002H8.2 | 0.000380543 | 0.084119144 | 1.3786883 | up | lncRNA |
| ENST00000557350 | RP11-545M17.1 | 0.35898378 | 0.873342057 | 1.2731932 | up | lncRNA |
| ENST00000557368 | CTD-2201G16.1 | 0.678449751 | 0.976096237 | 1.0225995 | up | lncRNA |
| ENST00000557373 | RP11-73E17.2 | 0.001802715 | 0.141460375 | 2.9196127 | up | lncRNA |
| ENST00000557452 | RP11-74M13.4 | 0.854730677 | 0.996142678 | 1.0276127 | up | lncRNA |
| ENST00000557499 | RP11-167B3.1 | 0.373957652 | 0.879143403 | 1.1174293 | up | lncRNA |
| ENST00000557532 | DIO3OS | 0.468954621 | 0.914585776 | 1.0705611 | up | lncRNA |
| ENST00000557544 | RP11-618G20.4 | 0.24948997 | 0.815830497 | 1.3065018 | up | lncRNA |
| ENST00000557545 | CTD-2313J17.2 | 0.395114659 | 0.885711362 | 1.4533394 | up | lncRNA |
| ENST00000557546 | RP11-566J3.2 | 0.164657164 | 0.719579787 | 1.2047783 | up | lncRNA |
| ENST00000557615 | RP11-298I3.4 | 0.611832176 | 0.957636208 | 1.0898876 | up | lncRNA |
| ENST00000557625 | FRMD6-AS2 | 0.318975955 | 0.851275405 | 1.1257027 | up | lncRNA |
| ENST00000557642 | RP11-81F13.2 | 0.322548966 | 0.853463264 | 1.0943819 | up | lncRNA |
| ENST00000557661 | DIO3OS | 0.287712495 | 0.836961572 | 1.262507 | up | lncRNA |
| ENST00000557715 | CTD-3049M7.1 | 0.217514845 | 0.785656962 | 1.1867612 | up | lncRNA |
| ENST00000557749 | RP11-479H16.1 | 0.926181275 | 0.998875101 | 1.0148218 | up | lncRNA |
| ENST00000557775 | RP11-114N19.3 | 0.948629409 | 0.998875101 | 1.0158728 | up | lncRNA |
| ENST00000557784 | CTD-2007A10.1 | 0.138010529 | 0.696458197 | 1.121835 | up | lncRNA |
| ENST00000557800 | RP11-138H8.6 | 0.683063443 | 0.977836377 | 1.0649362 | up | lncRNA |
| ENST00000557839 | RP11-526I2.1 | 0.246310981 | 0.813853858 | 1.1579984 | up | lncRNA |
| ENST00000557891 | RP11-313P18.1 | 0.154448218 | 0.711341825 | 1.2338124 | up | lncRNA |
| ENST00000557908 | RP11-158M2.5 | 0.633973685 | 0.964757805 | 1.1324128 | up | lncRNA |
| ENST00000557989 | RP11-680F8.3 | 0.003305656 | 0.167945066 | 1.2192837 | up | lncRNA |
| ENST00000558006 | RP11-151N17.1 | 0.867725951 | 0.99713777 | 1.0189915 | up | lncRNA |
| ENST00000558015 | RP11-561C5.6 | 0.606245697 | 0.956038292 | 1.1512433 | up | lncRNA |
| ENST00000558042 | RP11-30K9.5 | 0.894738213 | 0.998875101 | 1.009346 | up | lncRNA |
| ENST00000558061 | RP11-227D13.1 | 0.95724965 | 0.998875101 | 1.0102558 | up | lncRNA |
| ENST00000558105 | RP11-387D10.2 | 0.722565791 | 0.98251396 | 1.0828378 | up | lncRNA |
| ENST00000558142 | RP11-56B16.5 | 0.587564324 | 0.950589384 | 1.1170927 | up | lncRNA |
| ENST00000558149 | DNM1P44 | 0.935345852 | 0.998875101 | 1.0129992 | up | lncRNA |
| ENST00000558156 | CTD-3032H12.1 | 0.098521343 | 0.624143367 | 1.2165927 | up | lncRNA |
| ENST00000558185 | RP11-552E10.1 | 0.165980137 | 0.721169506 | 1.1870935 | up | lncRNA |
| ENST00000558209 | RP11-624L4.1 | 0.757812826 | 0.988042026 | 1.0235895 | up | lncRNA |
| ENST00000558226 | RP11-543G18.1 | 0.724228064 | 0.98251396 | 1.1471054 | up | lncRNA |
| ENST00000558235 | RP11-219B17.1 | 0.446541328 | 0.905320121 | 1.1689383 | up | lncRNA |
| ENST00000558237 | RP11-120K9.2 | 0.193068636 | 0.760765035 | 1.0587692 | up | lncRNA |
| ENST00000558251 | UBE2Q2P1 | 0.139272457 | 0.698406118 | 1.1061297 | up | lncRNA |
| ENST00000558269 | RP11-352D13.5 | 0.15609805 | 0.711341825 | 1.5601266 | up | lncRNA |
| ENST00000558282 | RP11-561C5.3 | 0.240960376 | 0.809272444 | 1.2099334 | up | lncRNA |
| ENST00000558334 | RP11-617F23.1 | 0.005931693 | 0.210201607 | 1.2665678 | up | lncRNA |
| ENST00000558359 | RP11-616K22.3 | 0.502978028 | 0.923939672 | 1.091735 | up | lncRNA |
| ENST00000558365 | RP11-561C5.5 | 0.721255684 | 0.98251396 | 1.1244012 | up | lncRNA |
| ENST00000558369 | LINC00277 | 0.272164602 | 0.832297359 | 1.4320398 | up | lncRNA |
| ENST00000558372 | CTD-3110H11.1 | 0.256975471 | 0.822133742 | 1.3688455 | up | lncRNA |
| ENST00000558385 | LINC00593 | 0.986794135 | 0.99906281 | 1.0009826 | up | lncRNA |
| ENST00000558389 | CTD-3065B20.2 | 0.947681188 | 0.998875101 | 1.0071458 | up | lncRNA |
| ENST00000558417 | RP11-566K19.5 | 0.888651004 | 0.998875101 | 1.0070505 | up | lncRNA |
| ENST00000558419 | CTD-2008A1.1 | 0.24251744 | 0.809272444 | 1.1972365 | up | lncRNA |
| ENST00000558427 | RP11-66B24.2 | 0.017891253 | 0.337638939 | 1.6044728 | up | lncRNA |
| ENST00000558441 | RP11-758N13.1 | 0.712902672 | 0.98251396 | 1.0320976 | up | lncRNA |
| ENST00000558443 | RP11-702L15.3 | 0.012105919 | 0.280774438 | 1.725215 | up | lncRNA |
| ENST00000558478 | RP11-468E2.5 | 0.000547966 | 0.094750704 | 3.0458365 | up | lncRNA |
| ENST00000558515 | RP11-424I19.2 | 0.533445637 | 0.935381352 | 1.0297604 | up | lncRNA |
| ENST00000558536 | RP11-519G16.3 | 0.001074949 | 0.116876278 | 3.6883786 | up | lncRNA |
| ENST00000558556 | CTD-2008A1.2 | 0.969232825 | 0.998875101 | 1.0036658 | up | lncRNA |
| ENST00000558593 | CTD-3110H11.2 | 0.833913902 | 0.99450938 | 1.0280131 | up | lncRNA |
| ENST00000558607 | CTD-2184D3.5 | 0.242110534 | 0.809272444 | 1.1247141 | up | lncRNA |
| ENST00000558650 | RP11-152F13.3 | 0.638398054 | 0.96564589 | 1.0339528 | up | lncRNA |
| ENST00000558671 | CTD-3118D7.1 | 0.566096925 | 0.943849275 | 1.3447649 | up | lncRNA |
| ENST00000558749 | RP11-356M20.3 | 0.021709517 | 0.361037071 | 1.1188619 | up | lncRNA |
| ENST00000558754 | CTD-2335A18.1 | 0.481352352 | 0.9192486 | 1.0652809 | up | lncRNA |
| ENST00000558842 | RP11-346C20.1 | 0.325577608 | 0.854556781 | 1.0388033 | up | lncRNA |
| ENST00000558885 | RP11-327J17.1 | 0.634413215 | 0.964932012 | 1.2310024 | up | lncRNA |
| ENST00000558909 | RP13-98N21.2 | 0.669579426 | 0.974341942 | 1.0367631 | up | lncRNA |
| ENST00000558938 | RP11-394B5.2 | 0.371174778 | 0.877177739 | 1.0834128 | up | lncRNA |
| ENST00000558945 | OIP5-AS1 | 0.367829504 | 0.875679927 | 1.0912903 | up | lncRNA |
| ENST00000558967 | RP11-540O11.4 | 0.558411469 | 0.943023022 | 1.1165249 | up | lncRNA |
| ENST00000558971 | RP11-762H8.3 | 0.558357003 | 0.943023022 | 1.0475101 | up | lncRNA |
| ENST00000559012 | RP11-521C20.4 | 0.591269327 | 0.950912706 | 1.1232417 | up | lncRNA |
| ENST00000559022 | RP11-521C20.3 | 0.413323492 | 0.890927603 | 1.1614616 | up | lncRNA |
| ENST00000559026 | RP11-59H7.3 | 0.379759094 | 0.880257051 | 1.1126248 | up | lncRNA |
| ENST00000559072 | ELMO2P1 | 0.185166434 | 0.751649101 | 1.268939 | up | lncRNA |
| ENST00000559148 | RP11-627D16.1 | 0.617551853 | 0.95949503 | 1.0919287 | up | lncRNA |
| ENST00000559173 | RP11-707P17.2 | 0.187955618 | 0.754266909 | 1.1457127 | up | lncRNA |
| ENST00000559210 | RP11-702M1.1 | 0.632332204 | 0.964380442 | 1.0366132 | up | lncRNA |
| ENST00000559211 | CTD-2034I4.1 | 0.196595245 | 0.762783174 | 1.1981034 | up | lncRNA |
| ENST00000559285 | RP11-502I4.1 | 0.231850344 | 0.799307263 | 1.366186 | up | lncRNA |
| ENST00000559298 | RP11-502I4.1 | 0.723102178 | 0.98251396 | 1.0739492 | up | lncRNA |
| ENST00000559299 | RP11-276M12.1 | 0.319695885 | 0.852213764 | 1.1050685 | up | lncRNA |
| ENST00000559302 | RP11-56B16.4 | 0.319855301 | 0.852213764 | 1.0998241 | up | lncRNA |
| ENST00000559331 | RP11-66B24.2 | 0.585557326 | 0.950239568 | 1.1153638 | up | lncRNA |
| ENST00000559357 | RP11-265M18.2 | 0.436727229 | 0.901987219 | 1.0667374 | up | lncRNA |
| ENST00000559368 | OIP5-AS1 | 0.651607344 | 0.969927925 | 1.0697415 | up | lncRNA |
| ENST00000559481 | RP11-561C5.4 | 0.426851546 | 0.897780553 | 1.0635895 | up | lncRNA |
| ENST00000559614 | AC002306.1 | 0.874174659 | 0.997909851 | 1.0456239 | up | lncRNA |
| ENST00000559621 | RP11-403B2.6 | 0.598317945 | 0.952424484 | 1.4464771 | up | lncRNA |
| ENST00000559654 | AC103965.1 | 0.586057354 | 0.950494284 | 1.074865 | up | lncRNA |
| ENST00000559666 | RP11-7M10.2 | 0.267022785 | 0.829082126 | 1.1460508 | up | lncRNA |
| ENST00000559740 | RP11-566K19.5 | 0.545513683 | 0.939697392 | 1.0903422 | up | lncRNA |
| ENST00000559749 | CTD-2071N1.1 | 0.115254219 | 0.657882593 | 1.2009321 | up | lncRNA |
| ENST00000559752 | RP11-96C21.1 | 0.85123195 | 0.995900317 | 1.0168616 | up | lncRNA |
| ENST00000559755 | RP11-66B24.5 | 0.318602156 | 0.851223284 | 1.1449988 | up | lncRNA |
| ENST00000559756 | AC074212.5 | 0.008247901 | 0.24294923 | 3.200432 | up | lncRNA |
| ENST00000559783 | RP11-507B12.2 | 0.799076139 | 0.992228977 | 1.0278006 | up | lncRNA |
| ENST00000559802 | CTD-3032H12.1 | 0.94775705 | 0.998875101 | 1.0080521 | up | lncRNA |
| ENST00000559812 | RP11-182J1.9 | 0.313562657 | 0.851097229 | 1.2406245 | up | lncRNA |
| ENST00000559825 | CTD-2184D3.6 | 0.278584865 | 0.832297359 | 1.2260248 | up | lncRNA |
| ENST00000559843 | RP11-736N17.8 | 0.591555699 | 0.950912706 | 1.038636 | up | lncRNA |
| ENST00000559884 | WASH3P | 0.256907693 | 0.822133742 | 1.0915791 | up | lncRNA |
| ENST00000559899 | RP11-37C7.2 | 0.279394302 | 0.832297359 | 1.171737 | up | lncRNA |
| ENST00000559914 | LINC00277 | 0.298405802 | 0.841479361 | 1.1754443 | up | lncRNA |
| ENST00000559979 | RP11-17L5.4 | 0.025278439 | 0.380009848 | 1.1955398 | up | lncRNA |
| ENST00000560040 | DDX11L9 | 0.352623864 | 0.868751345 | 1.0747844 | up | lncRNA |
| ENST00000560053 | RP11-26F2.1 | 0.294238287 | 0.838742488 | 1.2566901 | up | lncRNA |
| ENST00000560128 | RP11-90E5.1 | 0.387391329 | 0.884620581 | 1.585159 | up | lncRNA |
| ENST00000560140 | RP11-463I20.3 | 0.447180007 | 0.905615479 | 1.1418774 | up | lncRNA |
| ENST00000560159 | RP11-562A8.5 | 0.304709336 | 0.845829453 | 1.4918316 | up | lncRNA |
| ENST00000560169 | RP11-29M5.1 | 0.056600373 | 0.523070548 | 1.2933169 | up | lncRNA |
| ENST00000560237 | RP11-485O10.3 | 0.966814769 | 0.998875101 | 1.0051873 | up | lncRNA |
| ENST00000560242 | RP11-522B15.4 | 0.640369527 | 0.966209362 | 1.0313467 | up | lncRNA |
| ENST00000560259 | RP11-265N7.2 | 0.273840174 | 0.832297359 | 1.1408867 | up | lncRNA |
| ENST00000560295 | CTA-204B4.6 | 0.127184488 | 0.679843078 | 1.2282961 | up | lncRNA |
| ENST00000560306 | RP11-182J1.9 | 0.192395254 | 0.760765035 | 1.4592711 | up | lncRNA |
| ENST00000560323 | RP11-154J22.1 | 0.004783403 | 0.191763694 | 2.0550361 | up | lncRNA |
| ENST00000560351 | RP11-66B24.4 | 0.050017564 | 0.505173876 | 1.2021951 | up | lncRNA |
| ENST00000560359 | CTD-3110H11.2 | 0.90484253 | 0.998875101 | 1.0097998 | up | lncRNA |
| ENST00000560360 | CTD-2544M6.1 | 0.318468185 | 0.851223284 | 1.5664915 | up | lncRNA |
| ENST00000560363 | RP11-758N13.1 | 0.757482946 | 0.987871271 | 1.0529637 | up | lncRNA |
| ENST00000560376 | RP11-521C20.2 | 0.77385658 | 0.990435061 | 1.0235595 | up | lncRNA |
| ENST00000560381 | CTD-2071L7.1 | 0.632502753 | 0.964403446 | 1.0203979 | up | lncRNA |
| ENST00000560391 | RP11-57P19.1 | 0.755136169 | 0.987491052 | 1.3226036 | up | lncRNA |
| ENST00000560405 | RP11-561C5.4 | 0.54534853 | 0.939697392 | 1.0408602 | up | lncRNA |
| ENST00000560452 | RP11-17L5.4 | 0.004475463 | 0.188856054 | 1.3074498 | up | lncRNA |
| ENST00000560484 | RP11-624L4.1 | 0.09092835 | 0.605464696 | 1.4054469 | up | lncRNA |
| ENST00000560551 | RP11-616K22.3 | 0.911201982 | 0.998875101 | 1.0074789 | up | lncRNA |
| ENST00000560578 | RP11-154B12.3 | 0.018760222 | 0.342989437 | 1.5195791 | up | lncRNA |
| ENST00000560622 | RP11-265M18.2 | 0.962262592 | 0.998875101 | 1.0048208 | up | lncRNA |
| ENST00000560623 | RP11-605F22.2 | 0.017821032 | 0.337638939 | 1.1299639 | up | lncRNA |
| ENST00000560647 | RP11-519G16.3 | 0.782660243 | 0.99121281 | 1.0280582 | up | lncRNA |
| ENST00000560655 | RP11-279F6.3 | 0.237072231 | 0.806461402 | 1.2773973 | up | lncRNA |
| ENST00000560683 | RP11-463I20.2 | 0.825186042 | 0.993856674 | 1.0382193 | up | lncRNA |
| ENST00000560705 | RP11-718O11.1 | 0.303633271 | 0.84485163 | 1.1103427 | up | lncRNA |
| ENST00000560740 | RP11-680F8.4 | 0.2237505 | 0.792938703 | 1.0867757 | up | lncRNA |
| ENST00000560778 | RP11-2E17.1 | 0.359690158 | 0.873342057 | 1.2724541 | up | lncRNA |
| ENST00000560811 | RP11-561C5.4 | 0.2195947 | 0.787803958 | 1.1868535 | up | lncRNA |
| ENST00000560819 | RP11-1008C21.2 | 0.068936356 | 0.553727975 | 2.8940844 | up | lncRNA |
| ENST00000560866 | RP11-702M1.1 | 0.128388446 | 0.681698476 | 1.1157321 | up | lncRNA |
| ENST00000560870 | RP11-253M7.6 | 0.214613893 | 0.781610853 | 1.1595309 | up | lncRNA |
| ENST00000560886 | RP11-184D12.1 | 0.631226589 | 0.964380442 | 1.2602665 | up | lncRNA |
| ENST00000560903 | RP11-244F12.3 | 0.158242044 | 0.714689048 | 1.2415418 | up | lncRNA |
| ENST00000560930 | RP11-778O17.4 | 0.189384772 | 0.756484902 | 1.6409806 | up | lncRNA |
| ENST00000560931 | RP11-365N19.2 | 0.528999819 | 0.93440905 | 1.0822744 | up | lncRNA |
| ENST00000560933 | RP4-742C19.12 | 0.618026948 | 0.959844471 | 1.1339267 | up | lncRNA |
| ENST00000560958 | RP11-752G15.9 | 0.664471353 | 0.972257778 | 1.0223414 | up | lncRNA |
| ENST00000560962 | RP11-265M18.2 | 0.647297166 | 0.969646614 | 1.0832409 | up | lncRNA |
| ENST00000560967 | RP11-109D20.2 | 0.706039142 | 0.981012299 | 1.0748422 | up | lncRNA |
| ENST00000560973 | RP11-761I4.3 | 0.393546162 | 0.885711362 | 1.2495749 | up | lncRNA |
| ENST00000560994 | RP11-680F8.1 | 0.950570487 | 0.998875101 | 1.0105789 | up | lncRNA |
| ENST00000561015 | AC103965.1 | 0.592142863 | 0.950912706 | 1.1932473 | up | lncRNA |
| ENST00000561037 | CTD-2335A18.1 | 0.231710773 | 0.79921005 | 1.494546 | up | lncRNA |
| ENST00000561039 | RP11-111A22.1 | 0.524965033 | 0.932692492 | 1.1756235 | up | lncRNA |
| ENST00000561101 | CTD-2315E11.1 | 0.759481579 | 0.988042026 | 1.033293 | up | lncRNA |
| ENST00000561111 | RP11-316M1.12 | 0.081248524 | 0.584216547 | 1.0753191 | up | lncRNA |
| ENST00000561123 | RP11-307C19.2 | 0.489348139 | 0.920777644 | 1.1505909 | up | lncRNA |
| ENST00000561134 | RP5-977B1.11 | 0.267874168 | 0.829789144 | 1.0948125 | up | lncRNA |
| ENST00000561159 | RP11-56B16.3 | 0.095713389 | 0.618722353 | 1.260405 | up | lncRNA |
| ENST00000561165 | CTD-3094K11.2 | 0.090719578 | 0.604992392 | 1.2725706 | up | lncRNA |
| ENST00000561181 | CTD-3065J16.6 | 0.298155564 | 0.841354434 | 1.11831 | up | lncRNA |
| ENST00000561202 | RP11-50C13.2 | 0.358535532 | 0.872892612 | 1.0994716 | up | lncRNA |
| ENST00000561215 | CTD-2544M6.2 | 0.497388894 | 0.921785486 | 1.0678756 | up | lncRNA |
| ENST00000561254 | RP11-408J6.1 | 0.849672731 | 0.995876116 | 1.1592404 | up | lncRNA |
| ENST00000561256 | RP11-265M18.2 | 0.701157206 | 0.980170937 | 1.0566767 | up | lncRNA |
| ENST00000561310 | AC011648.1 | 0.161128542 | 0.716150139 | 1.3634929 | up | lncRNA |
| ENST00000561316 | RP11-19J5.2 | 0.299850981 | 0.8422517 | 1.0884396 | up | lncRNA |
| ENST00000561358 | RP11-343B18.2 | 0.67635284 | 0.976096237 | 1.1206878 | up | lncRNA |
| ENST00000561384 | CTD-2008A1.2 | 0.378799615 | 0.88011421 | 1.0952441 | up | lncRNA |
| ENST00000561388 | RP11-540O11.6 | 0.870587194 | 0.997909851 | 1.0630345 | up | lncRNA |
| ENST00000561403 | UBE2Q2P1 | 0.217117133 | 0.785656962 | 1.0409007 | up | lncRNA |
| ENST00000561432 | RP11-210M15.3 | 0.181003586 | 0.747570548 | 1.2291829 | up | lncRNA |
| ENST00000561458 | RP11-489D6.2 | 0.694895319 | 0.979851048 | 1.1471505 | up | lncRNA |
| ENST00000561476 | RP11-360A18.2 | 0.729505961 | 0.982972002 | 1.0198408 | up | lncRNA |
| ENST00000561510 | RP11-345M22.1 | 0.485606891 | 0.920158099 | 1.7771012 | up | lncRNA |
| ENST00000561544 | RP11-304L19.8 | 0.002674296 | 0.153526012 | 1.2925832 | up | lncRNA |
| ENST00000561550 | AC124789.1 | 0.466207122 | 0.913821992 | 1.1476388 | up | lncRNA |
| ENST00000561559 | RP11-568N6.1 | 0.002393509 | 0.145414844 | 3.1333895 | up | lncRNA |
| ENST00000561567 | RP4-536B24.3 | 0.479954475 | 0.918546036 | 1.1463755 | up | lncRNA |
| ENST00000561577 | CTD-2012K14.1 | 0.689991465 | 0.97861545 | 1.102106 | up | lncRNA |
| ENST00000561588 | RP11-211G23.2 | 0.673703504 | 0.975272478 | 1.0767624 | up | lncRNA |
| ENST00000561599 | RP11-483P21.2 | 0.615136214 | 0.958570569 | 1.1730226 | up | lncRNA |
| ENST00000561606 | CTD-2256P15.2 | 0.251675811 | 0.817761204 | 1.0829065 | up | lncRNA |
| ENST00000561611 | AC004158.2 | 0.493172704 | 0.921690406 | 1.1851798 | up | lncRNA |
| ENST00000561642 | RP11-812E19.3 | 0.780644391 | 0.99121281 | 1.1091113 | up | lncRNA |
| ENST00000561655 | RP11-475B2.1 | 0.359864216 | 0.873342057 | 1.416551 | up | lncRNA |
| ENST00000561669 | AC009120.10 | 0.159469945 | 0.714806047 | 1.1990921 | up | lncRNA |
| ENST00000561676 | RP11-457I16.2 | 0.559837354 | 0.943393901 | 1.160925 | up | lncRNA |
| ENST00000561699 | RP5-1142A6.5 | 0.313412481 | 0.851097229 | 1.1192884 | up | lncRNA |
| ENST00000561744 | RP11-586K12.13 | 0.643894621 | 0.967175883 | 1.0813107 | up | lncRNA |
| ENST00000561746 | RP11-819C21.1 | 0.381699938 | 0.881294633 | 1.2421112 | up | lncRNA |
| ENST00000561761 | RP11-629O1.2 | 0.034604229 | 0.429619286 | 2.0862875 | up | lncRNA |
| ENST00000561764 | CTD-2196E14.3 | 0.934356125 | 0.998875101 | 1.0299965 | up | lncRNA |
| ENST00000561777 | MIR4313 | 0.332963344 | 0.85731789 | 1.152367 | up | lncRNA |
| ENST00000561794 | RP6-91H8.2 | 0.932133777 | 0.998875101 | 1.0348713 | up | lncRNA |
| ENST00000561800 | RP11-546B15.1 | 0.525733177 | 0.932903382 | 1.0574476 | up | lncRNA |
| ENST00000561802 | AC140912.1 | 0.113248316 | 0.653534309 | 1.5627014 | up | lncRNA |
| ENST00000561805 | FAM157C | 0.612258054 | 0.957725078 | 1.0464946 | up | lncRNA |
| ENST00000561822 | RP11-5N23.3 | 0.338941685 | 0.860518281 | 1.1421076 | up | lncRNA |
| ENST00000561876 | CTC-527H23.3 | 0.052159308 | 0.51320639 | 1.2925574 | up | lncRNA |
| ENST00000561900 | RP11-486L19.2 | 0.942929 | 0.998875101 | 1.0107571 | up | lncRNA |
| ENST00000561909 | CTD-2302E22.4 | 0.287892763 | 0.836961572 | 1.343379 | up | lncRNA |
| ENST00000561921 | AC009120.6 | 0.397571195 | 0.887348439 | 1.2205302 | up | lncRNA |
| ENST00000561923 | RP11-459F6.1 | 0.210425617 | 0.777960284 | 1.1993288 | up | lncRNA |
| ENST00000561926 | RP11-544D21.2 | 0.68129809 | 0.977505767 | 1.068581 | up | lncRNA |
| ENST00000561968 | CRYM-AS1 | 0.102936973 | 0.632351488 | 1.5854362 | up | lncRNA |
| ENST00000561973 | RP6-201G10.2 | 0.429637311 | 0.898463455 | 1.4140378 | up | lncRNA |
| ENST00000561980 | RP11-830F9.7 | 0.887644291 | 0.998875101 | 1.0263911 | up | lncRNA |
| ENST00000562009 | CTD-2311M21.2 | 0.853498822 | 0.996142678 | 1.0101161 | up | lncRNA |
| ENST00000562031 | RP11-8P11.3 | 0.511175731 | 0.927835348 | 1.1054356 | up | lncRNA |
| ENST00000562040 | CTD-2555A7.1 | 0.435672625 | 0.901926599 | 1.0531998 | up | lncRNA |
| ENST00000562054 | RP11-109E24.2 | 0.191939168 | 0.760520345 | 1.3193956 | up | lncRNA |
| ENST00000562098 | RP11-652G5.1 | 0.879078948 | 0.998682284 | 1.0546226 | up | lncRNA |
| ENST00000562130 | RP11-941F15.1 | 0.204982966 | 0.771727492 | 1.1115321 | up | lncRNA |
| ENST00000562175 | RP11-31I10.2 | 0.833216786 | 0.994074191 | 1.0560254 | up | lncRNA |
| ENST00000562178 | RP11-467J12.4 | 0.795644523 | 0.991420185 | 1.0352408 | up | lncRNA |
| ENST00000562220 | PCDHA14 | 0.413666551 | 0.890927603 | 1.0397236 | up | lncRNA |
| ENST00000562248 | LINC00304 | 0.513036727 | 0.928980962 | 1.097027 | up | lncRNA |
| ENST00000562284 | RP11-731J8.2 | 0.037705303 | 0.443562717 | 1.3196511 | up | lncRNA |
| ENST00000562328 | RP11-378A13.1 | 0.409650083 | 0.890549744 | 1.1166762 | up | lncRNA |
| ENST00000562341 | RP11-211G23.1 | 0.542274581 | 0.938790368 | 1.1133387 | up | lncRNA |
| ENST00000562343 | RP11-923I11.6 | 0.712789182 | 0.98251396 | 1.0290819 | up | lncRNA |
| ENST00000562349 | CTC-420A11.2 | 0.015556967 | 0.31394045 | 1.269365 | up | lncRNA |
| ENST00000562354 | KIAA0664L3 | 0.061193576 | 0.533992678 | 1.4591632 | up | lncRNA |
| ENST00000562355 | RP11-44F21.5 | 0.695318799 | 0.979987764 | 1.0416089 | up | lncRNA |
| ENST00000562376 | RP11-141O15.1 | 0.279272265 | 0.832297359 | 1.0358432 | up | lncRNA |
| ENST00000562405 | RP11-830F9.5 | 0.062023426 | 0.534658422 | 1.1018737 | up | lncRNA |
| ENST00000562409 | RP11-407G23.4 | 0.037166474 | 0.440489158 | 1.2890409 | up | lncRNA |
| ENST00000562450 | RP11-303E16.5 | 0.174622761 | 0.736164062 | 1.1581418 | up | lncRNA |
| ENST00000562471 | RP11-7K24.3 | 0.237572292 | 0.806512655 | 1.2065279 | up | lncRNA |
| ENST00000562506 | RP11-554A11.5 | 0.125175998 | 0.67731705 | 1.2135368 | up | lncRNA |
| ENST00000562525 | RP11-297C4.3 | 0.258225152 | 0.823932277 | 1.2519752 | up | lncRNA |
| ENST00000562538 | AC000032.2 | 0.791064664 | 0.991420185 | 1.0479607 | up | lncRNA |
| ENST00000562549 | RP11-46D6.1 | 0.803677103 | 0.992578294 | 1.0619454 | up | lncRNA |
| ENST00000562555 | RP11-259G18.1 | 0.99078514 | 0.999156445 | 1.0035474 | up | lncRNA |
| ENST00000562573 | RP11-1007O24.3 | 0.490714577 | 0.920777644 | 1.1404179 | up | lncRNA |
| ENST00000562574 | RP11-830F9.5 | 0.41434877 | 0.891430603 | 1.1568668 | up | lncRNA |
| ENST00000562644 | GS1-21A4.1 | 0.381920779 | 0.881294633 | 1.1419868 | up | lncRNA |
| ENST00000562656 | RP11-256I9.3 | 0.003447784 | 0.170097393 | 2.1398859 | up | lncRNA |
| ENST00000562658 | CTD-2524L6.3 | 0.564772704 | 0.943393901 | 1.2304167 | up | lncRNA |
| ENST00000562665 | RP11-1223D19.1 | 0.21518363 | 0.782855908 | 1.1218603 | up | lncRNA |
| ENST00000562672 | CTD-2526A2.2 | 0.150726229 | 0.705782327 | 1.23474 | up | lncRNA |
| ENST00000562681 | RP11-592N21.2 | 0.167677737 | 0.723906495 | 1.0790554 | up | lncRNA |
| ENST00000562687 | HERC2P5 | 0.310806306 | 0.848804431 | 1.2328808 | up | lncRNA |
| ENST00000562691 | RP11-174G6.5 | 0.18024909 | 0.745729299 | 1.2453699 | up | lncRNA |
| ENST00000562696 | CTD-2033A16.1 | 0.57815414 | 0.94830494 | 1.1686223 | up | lncRNA |
| ENST00000562700 | SLC25A3P1 | 0.127626099 | 0.680419676 | 1.1482861 | up | lncRNA |
| ENST00000562716 | RP11-114H24.6 | 0.280760609 | 0.832297359 | 1.1912031 | up | lncRNA |
| ENST00000562739 | RP11-941F15.1 | 0.834162759 | 0.99450938 | 1.0122176 | up | lncRNA |
| ENST00000562742 | RP11-95H3.1 | 0.184300898 | 0.751257244 | 1.2180727 | up | lncRNA |
| ENST00000562763 | RP11-432I5.2 | 0.202929053 | 0.770078732 | 1.3684008 | up | lncRNA |
| ENST00000562773 | RP11-812E19.6 | 0.481183964 | 0.9192486 | 1.0355458 | up | lncRNA |
| ENST00000562782 | CTD-2555A7.3 | 0.155876733 | 0.711341825 | 1.0877183 | up | lncRNA |
| ENST00000562802 | RP11-166B2.5 | 0.024121647 | 0.374301178 | 1.3008595 | up | lncRNA |
| ENST00000562807 | ERVK13-1 | 0.610079405 | 0.957150753 | 1.0450576 | up | lncRNA |
| ENST00000562822 | RP11-355E10.1 | 0.738372485 | 0.984496646 | 1.0619268 | up | lncRNA |
| ENST00000562846 | CTD-2012K14.7 | 0.33051 | 0.855705627 | 1.0988127 | up | lncRNA |
| ENST00000562848 | AC012531.25 | 0.304187787 | 0.845358864 | 1.7700414 | up | lncRNA |
| ENST00000562852 | RP11-143N13.2 | 0.895539345 | 0.998875101 | 1.0059593 | up | lncRNA |
| ENST00000562893 | RP11-473I1.6 | 0.85944625 | 0.996142678 | 1.0245987 | up | lncRNA |
| ENST00000562945 | RP11-488I20.9 | 0.021160093 | 0.359097712 | 1.6875457 | up | lncRNA |
| ENST00000562965 | RP11-941F15.1 | 0.1937063 | 0.760765035 | 1.1283529 | up | lncRNA |
| ENST00000562970 | RP11-322D14.2 | 0.77736629 | 0.991152912 | 1.0600778 | up | lncRNA |
| ENST00000562996 | RP11-923I11.5 | 0.166261641 | 0.721556419 | 1.1082979 | up | lncRNA |
| ENST00000563035 | RP11-1223D19.1 | 0.988343322 | 0.99906281 | 1.008898 | up | lncRNA |
| ENST00000563041 | CTD-2524L6.3 | 0.304334818 | 0.845358864 | 1.3097774 | up | lncRNA |
| ENST00000563083 | CTD-2012K14.3 | 0.595778853 | 0.951509512 | 1.0453342 | up | lncRNA |
| ENST00000563105 | RP11-425D10.10 | 0.427639876 | 0.897780553 | 1.0703081 | up | lncRNA |
| ENST00000563182 | RP11-46C24.6 | 0.715727777 | 0.98251396 | 1.0609836 | up | lncRNA |
| ENST00000563202 | RP11-578F21.12 | 0.186889483 | 0.753717042 | 1.1197808 | up | lncRNA |
| ENST00000563223 | LA16c-390E6.3 | 0.890134243 | 0.998875101 | 1.0287766 | up | lncRNA |
| ENST00000563230 | RP11-556H2.3 | 0.458139386 | 0.912197894 | 1.2134794 | up | lncRNA |
| ENST00000563256 | RP13-98N21.3 | 0.882390051 | 0.998875101 | 1.0193035 | up | lncRNA |
| ENST00000563267 | RP11-525K10.3 | 0.674259793 | 0.975501668 | 1.0674939 | up | lncRNA |
| ENST00000563268 | RP11-367F23.2 | 0.407070199 | 0.889505404 | 1.2113323 | up | lncRNA |
| ENST00000563280 | RP11-463O9.5 | 0.454691888 | 0.910032467 | 1.2491494 | up | lncRNA |
| ENST00000563287 | RP11-10O17.1 | 0.216253711 | 0.784389384 | 1.2032049 | up | lncRNA |
| ENST00000563344 | RP11-141O15.1 | 0.834117349 | 0.99450938 | 1.0576139 | up | lncRNA |
| ENST00000563349 | RP11-114H24.2 | 0.27257854 | 0.832297359 | 1.879422 | up | lncRNA |
| ENST00000563394 | RP5-882C2.2 | 0.321793863 | 0.853202571 | 1.3448675 | up | lncRNA |
| ENST00000563397 | RP11-525K10.1 | 0.770475316 | 0.989799889 | 1.0587352 | up | lncRNA |
| ENST00000563417 | RP11-410C4.5 | 0.682810099 | 0.977836377 | 1.0802072 | up | lncRNA |
| ENST00000563435 | AP006547.3 | 0.419556048 | 0.894750301 | 1.2855298 | up | lncRNA |
| ENST00000563472 | RP11-31E22.1 | 0.550281832 | 0.942167708 | 1.0897277 | up | lncRNA |
| ENST00000563487 | RP11-80F22.2 | 0.019096409 | 0.344796409 | 1.4341497 | up | lncRNA |
| ENST00000563521 | RP11-863P13.1 | 0.34296396 | 0.863514929 | 1.0705554 | up | lncRNA |
| ENST00000563553 | RP11-214L13.1 | 0.54220812 | 0.938790368 | 1.1572103 | up | lncRNA |
| ENST00000563557 | RP11-432I5.4 | 0.521386317 | 0.932117592 | 1.2369478 | up | lncRNA |
| ENST00000563565 | RP11-24N18.1 | 0.774808483 | 0.990435061 | 1.0261383 | up | lncRNA |
| ENST00000563568 | RP11-69H7.3 | 0.034188828 | 0.42757611 | 1.2248068 | up | lncRNA |
| ENST00000563574 | CTD-2547L24.4 | 0.071610812 | 0.560238874 | 1.3038421 | up | lncRNA |
| ENST00000563610 | LA16c-390E6.4 | 0.642532048 | 0.967175883 | 1.0618166 | up | lncRNA |
| ENST00000563631 | RP11-489G11.3 | 0.50442521 | 0.92405258 | 1.0842903 | up | lncRNA |
| ENST00000563632 | RP11-757F18.5 | 0.123118998 | 0.67474001 | 2.8326255 | up | lncRNA |
| ENST00000563684 | RP11-18F14.4 | 0.491929794 | 0.920777644 | 1.1472505 | up | lncRNA |
| ENST00000563709 | CTD-2524L6.2 | 0.058192096 | 0.527275332 | 1.2602173 | up | lncRNA |
| ENST00000563750 | MIR4720 | 0.939454143 | 0.998875101 | 1.0047896 | up | lncRNA |
| ENST00000563751 | RP11-297C4.1 | 0.273069118 | 0.832297359 | 1.120712 | up | lncRNA |
| ENST00000563759 | RP11-394I13.1 | 0.595332324 | 0.951199074 | 1.2373458 | up | lncRNA |
| ENST00000563777 | FBXL19-AS1 | 0.750003493 | 0.98551982 | 1.0644168 | up | lncRNA |
| ENST00000563806 | AC009133.14 | 0.806805728 | 0.992578294 | 1.0489775 | up | lncRNA |
| ENST00000563855 | RP11-368L12.1 | 0.585384441 | 0.950066437 | 1.211509 | up | lncRNA |
| ENST00000563867 | RP11-114H24.4 | 0.546539305 | 0.940293947 | 1.0701098 | up | lncRNA |
| ENST00000563931 | RP11-805I24.1 | 0.338514223 | 0.86050695 | 1.2760348 | up | lncRNA |
| ENST00000563938 | GHc-362H12.3 | 0.271620542 | 0.83225833 | 1.3202311 | up | lncRNA |
| ENST00000563946 | RP4-584D14.7 | 0.94713028 | 0.998875101 | 1.0248948 | up | lncRNA |
| ENST00000563960 | RP11-1437A8.4 | 0.866105991 | 0.996767702 | 1.0280644 | up | lncRNA |
| ENST00000563981 | RP11-298D21.1 | 0.490403446 | 0.920777644 | 1.0893074 | up | lncRNA |
| ENST00000563989 | RP11-45P15.4 | 0.982033248 | 0.998875101 | 1.0045544 | up | lncRNA |
| ENST00000563990 | RP11-553E24.2 | 0.556744211 | 0.943023022 | 1.1585045 | up | lncRNA |
| ENST00000563993 | RP4-536B24.4 | 0.4720395 | 0.916418344 | 1.165797 | up | lncRNA |
| ENST00000564016 | CTD-2583P5.1 | 0.365010979 | 0.874818743 | 1.3116028 | up | lncRNA |
| ENST00000564038 | RP11-1223D19.1 | 0.778578805 | 0.99121281 | 1.2299703 | up | lncRNA |
| ENST00000564048 | RP11-491F9.5 | 0.788497644 | 0.991420185 | 1.0386241 | up | lncRNA |
| ENST00000564063 | RP11-483I13.5 | 0.39454316 | 0.885711362 | 1.1759769 | up | lncRNA |
| ENST00000564147 | RP11-96D1.6 | 0.58269397 | 0.94928838 | 1.1173813 | up | lncRNA |
| ENST00000564152 | CTA-29F11.1 | 0.417471009 | 0.892927558 | 1.1700578 | up | lncRNA |
| ENST00000564165 | RP11-19N8.4 | 0.613062038 | 0.957994032 | 1.0549026 | up | lncRNA |
| ENST00000564248 | RP3-406A7.7 | 0.394427874 | 0.885711362 | 1.3959524 | up | lncRNA |
| ENST00000564287 | RP11-61J19.4 | 0.353845692 | 0.869401398 | 1.2471877 | up | lncRNA |
| ENST00000564299 | RP11-574O16.1 | 0.224807199 | 0.794551181 | 1.2941852 | up | lncRNA |
| ENST00000564300 | RP4-593C16.3 | 0.58434954 | 0.949470507 | 1.0145534 | up | lncRNA |
| ENST00000564371 | CTD-2576F9.2 | 0.802360454 | 0.992578294 | 1.0674635 | up | lncRNA |
| ENST00000564390 | RP11-161M6.5 | 0.289812626 | 0.837264328 | 1.0814983 | up | lncRNA |
| ENST00000564394 | AC137932.5 | 0.277500874 | 0.832297359 | 1.090195 | up | lncRNA |
| ENST00000564411 | RP11-525K10.3 | 0.016826342 | 0.327871272 | 1.1824884 | up | lncRNA |
| ENST00000564438 | AC005606.14 | 0.56872214 | 0.943923733 | 1.1200578 | up | lncRNA |
| ENST00000564479 | RP11-275F13.1 | 0.653993791 | 0.970589543 | 1.0966381 | up | lncRNA |
| ENST00000564485 | RP11-77H9.8 | 0.97358561 | 0.998875101 | 1.0071849 | up | lncRNA |
| ENST00000564498 | HERC2P8 | 0.236464629 | 0.805225869 | 1.0966712 | up | lncRNA |
| ENST00000564505 | CTD-3037G24.3 | 0.445904264 | 0.90519468 | 1.1194583 | up | lncRNA |
| ENST00000564527 | RP11-6O2.3 | 0.202593585 | 0.769638667 | 1.3359802 | up | lncRNA |
| ENST00000564577 | RP11-165M1.2 | 0.232134799 | 0.79956859 | 1.1482004 | up | lncRNA |
| ENST00000564585 | RP11-982M15.8 | 0.464347399 | 0.913755581 | 1.5991732 | up | lncRNA |
| ENST00000564612 | RP4-545K15.5 | 0.745761093 | 0.985094857 | 1.0418782 | up | lncRNA |
| ENST00000564613 | GS1-465N13.1 | 0.675928633 | 0.976096237 | 1.0686241 | up | lncRNA |
| ENST00000564629 | RP11-452L6.5 | 0.045613271 | 0.482418324 | 1.1817007 | up | lncRNA |
| ENST00000564680 | TREML3P | 0.662781324 | 0.971730383 | 1.120587 | up | lncRNA |
| ENST00000564681 | RP11-108B14.5 | 0.622087401 | 0.960457826 | 1.0953268 | up | lncRNA |
| ENST00000564694 | RP11-521I2.3 | 0.240498415 | 0.809272444 | 1.3576925 | up | lncRNA |
| ENST00000564743 | RP11-388M20.6 | 0.666680634 | 0.972894449 | 1.0499307 | up | lncRNA |
| ENST00000564834 | RP5-1129J21.3 | 0.990971107 | 0.999156445 | 1.0010694 | up | lncRNA |
| ENST00000564893 | CTD-2049O4.1 | 0.218187546 | 0.785656962 | 1.3671634 | up | lncRNA |
| ENST00000564919 | RP11-77H9.5 | 0.332256712 | 0.857061033 | 1.1158879 | up | lncRNA |
| ENST00000564933 | RP11-160C18.2 | 0.277963324 | 0.832297359 | 1.1578246 | up | lncRNA |
| ENST00000564977 | RP11-395B7.7 | 0.218098654 | 0.785656962 | 1.2326444 | up | lncRNA |
| ENST00000564984 | RP5-1142A6.9 | 0.348727994 | 0.867156419 | 1.1000757 | up | lncRNA |
| ENST00000565050 | RP11-525K10.3 | 0.673242607 | 0.97507861 | 1.1908892 | up | lncRNA |
| ENST00000565072 | RP11-42I10.1 | 0.166062533 | 0.721169506 | 1.1955286 | up | lncRNA |
| ENST00000565104 | RP11-65L3.1 | 0.678615402 | 0.976096237 | 1.0535579 | up | lncRNA |
| ENST00000565118 | ABCC6P1 | 0.770670833 | 0.989946102 | 1.1023836 | up | lncRNA |
| ENST00000565137 | AC026471.6 | 0.008672549 | 0.246472128 | 1.3364548 | up | lncRNA |
| ENST00000565145 | RP11-554A11.9 | 0.966216188 | 0.998875101 | 1.0074824 | up | lncRNA |
| ENST00000565152 | RP11-388M20.1 | 0.041448066 | 0.464736439 | 1.7115536 | up | lncRNA |
| ENST00000565153 | RP11-297L17.2 | 0.756961888 | 0.98762882 | 1.0631304 | up | lncRNA |
| ENST00000565166 | CTD-2576F9.2 | 0.935192771 | 0.998875101 | 1.0210968 | up | lncRNA |
| ENST00000565181 | RP11-1007O24.3 | 0.165239613 | 0.719579787 | 1.3044126 | up | lncRNA |
| ENST00000565189 | RP11-44F14.2 | 0.411231568 | 0.890876411 | 1.1517409 | up | lncRNA |
| ENST00000565229 | RP11-297L17.4 | 0.356148608 | 0.870922615 | 1.1483601 | up | lncRNA |
| ENST00000565257 | RP11-744D14.2 | 0.114167077 | 0.655262594 | 1.1495817 | up | lncRNA |
| ENST00000565259 | RP11-380D11.2 | 0.908211206 | 0.998875101 | 1.023629 | up | lncRNA |
| ENST00000565271 | RP11-318A15.2 | 0.142074452 | 0.698941469 | 1.4884101 | up | lncRNA |
| ENST00000565297 | RP11-79H23.3 | 1.48113E-08 | 0.000212572 | 22.073424 | up | lncRNA |
| ENST00000565305 | RP11-1006G14.2 | 0.737780906 | 0.984316636 | 1.0324899 | up | lncRNA |
| ENST00000565307 | RP11-212I21.2 | 0.975187011 | 0.998875101 | 1.004223 | up | lncRNA |
| ENST00000565310 | RP11-104N10.1 | 0.177386338 | 0.742662987 | 1.130832 | up | lncRNA |
| ENST00000565327 | RP11-327F22.5 | 0.023209836 | 0.36807466 | 2.2344821 | up | lncRNA |
| ENST00000565416 | RP11-941F15.1 | 0.931489917 | 0.998875101 | 1.0036805 | up | lncRNA |
| ENST00000565521 | CTD-2083E4.4 | 0.322756032 | 0.853463264 | 1.2555225 | up | lncRNA |
| ENST00000565543 | RP11-1006G14.2 | 0.924493873 | 0.998875101 | 1.0382102 | up | lncRNA |
| ENST00000565545 | RP11-114H24.1 | 0.128649241 | 0.682265946 | 1.2132595 | up | lncRNA |
| ENST00000565547 | RP11-326A19.3 | 0.127887306 | 0.680549576 | 1.1247368 | up | lncRNA |
| ENST00000565563 | RP5-902P8.12 | 0.50209737 | 0.923357158 | 1.0736491 | up | lncRNA |
| ENST00000565566 | ABCC6P1 | 0.035292236 | 0.431092836 | 1.1778835 | up | lncRNA |
| ENST00000565623 | RP11-104N10.1 | 0.078074374 | 0.575629572 | 1.0443854 | up | lncRNA |
| ENST00000565625 | RP11-352B15.1 | 0.939774126 | 0.998875101 | 1.022683 | up | lncRNA |
| ENST00000565633 | RP5-1142A6.3 | 0.349800378 | 0.867400431 | 1.3867893 | up | lncRNA |
| ENST00000565670 | SNX18P3 | 0.390490648 | 0.885711362 | 1.0824155 | up | lncRNA |
| ENST00000565695 | RP3-467N11.1 | 0.242522351 | 0.809272444 | 1.2982882 | up | lncRNA |
| ENST00000565700 | RP11-517C16.2 | 0.155852937 | 0.711341825 | 1.5255757 | up | lncRNA |
| ENST00000565714 | RP11-483P21.2 | 0.254248613 | 0.820374967 | 1.3743208 | up | lncRNA |
| ENST00000565735 | RP11-510C10.4 | 0.821578202 | 0.993343952 | 1.3559639 | up | lncRNA |
| ENST00000565748 | CTD-2636A23.2 | 0.187555186 | 0.753977992 | 1.2488342 | up | lncRNA |
| ENST00000565755 | RP11-297L17.1 | 0.66981864 | 0.974341942 | 1.1744541 | up | lncRNA |
| ENST00000565757 | FAM157C | 0.429199407 | 0.898454882 | 1.099725 | up | lncRNA |
| ENST00000565762 | RP11-294K24.4 | 0.666475012 | 0.972894449 | 1.0327177 | up | lncRNA |
| ENST00000565782 | CTD-2288F12.1 | 0.473104287 | 0.91677171 | 1.2424407 | up | lncRNA |
| ENST00000565783 | RP11-673P17.2 | 0.452932988 | 0.909287206 | 1.1511261 | up | lncRNA |
| ENST00000565824 | RP11-482M8.1 | 0.914128569 | 0.998875101 | 1.0453244 | up | lncRNA |
| ENST00000565827 | RP11-715J22.4 | 0.817250245 | 0.992651327 | 1.039412 | up | lncRNA |
| ENST00000565829 | RP11-407G23.1 | 0.077043739 | 0.573215003 | 2.0291895 | up | lncRNA |
| ENST00000565882 | RP11-65J3.14 | 0.196123414 | 0.762235583 | 1.5402498 | up | lncRNA |
| ENST00000565934 | RP11-77H9.2 | 0.912697398 | 0.998875101 | 1.0063746 | up | lncRNA |
| ENST00000565959 | AP000265.1 | 0.459122217 | 0.91221792 | 1.0757117 | up | lncRNA |
| ENST00000566043 | FAM157C | 0.985317677 | 0.998971647 | 1.0036919 | up | lncRNA |
| ENST00000566054 | RP11-65J21.1 | 0.938886419 | 0.998875101 | 1.0250088 | up | lncRNA |
| ENST00000566070 | RP11-426C22.4 | 0.565495421 | 0.943572364 | 1.1046855 | up | lncRNA |
| ENST00000566085 | RP11-715J22.4 | 0.932245964 | 0.998875101 | 1.0143007 | up | lncRNA |
| ENST00000566108 | CTD-2540M10.1 | 0.759262151 | 0.988042026 | 1.0359729 | up | lncRNA |
| ENST00000566140 | CTD-2319I12.1 | 0.004532449 | 0.189098001 | 1.2841781 | up | lncRNA |
| ENST00000566170 | RP1-223E5.4 | 0.044213918 | 0.478642344 | 1.3363769 | up | lncRNA |
| ENST00000566195 | RP11-426L16.9 | 0.808102726 | 0.992578294 | 1.0469141 | up | lncRNA |
| ENST00000566225 | RP11-243E13.1 | 0.033434623 | 0.42757611 | 1.2819684 | up | lncRNA |
| ENST00000566287 | LA16c-390E6.5 | 0.3298448 | 0.855547596 | 1.176229 | up | lncRNA |
| ENST00000566325 | RP11-27M24.2 | 0.005182838 | 0.201038095 | 3.2129582 | up | lncRNA |
| ENST00000566394 | RP11-553K8.5 | 0.776700084 | 0.991030431 | 1.2411683 | up | lncRNA |
| ENST00000566457 | CTD-3247F14.2 | 0.15108975 | 0.70579658 | 1.3554033 | up | lncRNA |
| ENST00000566475 | HCG22 | 0.924833086 | 0.998875101 | 1.012025 | up | lncRNA |
| ENST00000566529 | RP11-2A4.4 | 0.724536203 | 0.98251396 | 1.0340681 | up | lncRNA |
| ENST00000566537 | AC009133.15 | 0.587048335 | 0.950589384 | 1.0643195 | up | lncRNA |
| ENST00000566562 | RP11-80F22.14 | 0.49902218 | 0.921805853 | 1.2193418 | up | lncRNA |
| ENST00000566567 | RP11-83N9.5 | 0.536074865 | 0.93641313 | 1.1131225 | up | lncRNA |
| ENST00000566570 | RP11-760D2.11 | 0.480146665 | 0.918546036 | 1.0896945 | up | lncRNA |
| ENST00000566575 | CTA-250D10.23 | 0.759177775 | 0.988042026 | 1.210541 | up | lncRNA |
| ENST00000566645 | RP11-297L17.2 | 0.201027105 | 0.767810338 | 1.0530492 | up | lncRNA |
| ENST00000566676 | RP11-580I1.2 | 0.302797115 | 0.843991909 | 1.4134343 | up | lncRNA |
| ENST00000566733 | RP1-140K8.5 | 0.785307948 | 0.99125421 | 1.0243932 | up | lncRNA |
| ENST00000566734 | RP11-1021N1.2 | 0.69480297 | 0.979851048 | 1.0528136 | up | lncRNA |
| ENST00000566738 | RP11-432I5.8 | 0.660200849 | 0.971704562 | 1.0651069 | up | lncRNA |
| ENST00000566747 | RP11-21L23.2 | 0.865284171 | 0.996767702 | 1.015905 | up | lncRNA |
| ENST00000566781 | RP13-98N21.3 | 0.677883551 | 0.976096237 | 1.023634 | up | lncRNA |
| ENST00000566787 | RP11-27M24.3 | 0.075184856 | 0.567570001 | 1.7755038 | up | lncRNA |
| ENST00000566847 | RP11-85A1.3 | 0.024001451 | 0.373610439 | 1.9958098 | up | lncRNA |
| ENST00000566851 | RRP7B | 0.93330198 | 0.998875101 | 1.0092577 | up | lncRNA |
| ENST00000566927 | LA16c-313D11.12 | 0.610120904 | 0.957150753 | 1.1216055 | up | lncRNA |
| ENST00000566929 | CTD-2313J23.1 | 0.880857299 | 0.998875101 | 1.0233037 | up | lncRNA |
| ENST00000566949 | RP5-1042I8.7 | 0.083057595 | 0.585414993 | 1.8787284 | up | lncRNA |
| ENST00000566954 | RP13-122B23.8 | 0.447718984 | 0.905943546 | 1.1758642 | up | lncRNA |
| ENST00000566960 | RP11-529K1.4 | 0.150620779 | 0.705518742 | 1.068996 | up | lncRNA |
| ENST00000567002 | RP11-64K12.10 | 0.929331467 | 0.998875101 | 1.006535 | up | lncRNA |
| ENST00000567043 | AC002519.6 | 0.580331511 | 0.949213695 | 1.0315952 | up | lncRNA |
| ENST00000567054 | MT1CP | 0.096152943 | 0.620776893 | 2.115323 | up | lncRNA |
| ENST00000567073 | HERC2P8 | 0.85095581 | 0.995900317 | 1.0128014 | up | lncRNA |
| ENST00000567077 | RP11-432I5.6 | 0.981250036 | 0.998875101 | 1.0032204 | up | lncRNA |
| ENST00000567089 | RP11-265N6.2 | 0.399132262 | 0.888157273 | 1.2983093 | up | lncRNA |
| ENST00000567091 | LA16c-313D11.9 | 0.626944125 | 0.962239555 | 1.090427 | up | lncRNA |
| ENST00000567093 | CTD-2542L18.1 | 0.327541345 | 0.854556781 | 1.0683792 | up | lncRNA |
| ENST00000567108 | CTD-3203P2.1 | 0.455541954 | 0.91064808 | 1.4528266 | up | lncRNA |
| ENST00000567167 | RP11-923I11.4 | 0.693483344 | 0.979035309 | 1.0241559 | up | lncRNA |
| ENST00000567186 | RP11-394B2.6 | 0.15401208 | 0.711341825 | 1.0624962 | up | lncRNA |
| ENST00000567230 | RP11-299H22.5 | 0.271424299 | 0.832251135 | 1.2947707 | up | lncRNA |
| ENST00000567236 | RP11-626G11.3 | 0.867979526 | 0.99713777 | 1.0200825 | up | lncRNA |
| ENST00000567259 | RP5-963E22.5 | 0.426947858 | 0.897780553 | 1.2941046 | up | lncRNA |
| ENST00000567286 | RP11-10O17.2 | 0.178616562 | 0.743651912 | 1.1545224 | up | lncRNA |
| ENST00000567313 | AC002398.5 | 0.713272012 | 0.98251396 | 1.0171144 | up | lncRNA |
| ENST00000567327 | RP11-366L5.1 | 0.977802909 | 0.998875101 | 1.008071 | up | lncRNA |
| ENST00000567342 | RP11-483P21.2 | 0.003489995 | 0.171535633 | 3.1806443 | up | lncRNA |
| ENST00000567359 | RP11-22H5.2 | 0.002384393 | 0.145414844 | 3.9437318 | up | lncRNA |
| ENST00000567374 | CTC-400I9.1 | 0.752192445 | 0.986481248 | 1.0464306 | up | lncRNA |
| ENST00000567376 | RP11-116D2.1 | 0.133084427 | 0.688794697 | 1.3235032 | up | lncRNA |
| ENST00000567395 | RP11-20G6.2 | 0.192087332 | 0.760520345 | 1.2031143 | up | lncRNA |
| ENST00000567422 | RP11-23E10.4 | 0.680173713 | 0.976675999 | 1.0738043 | up | lncRNA |
| ENST00000567456 | CTD-2036P10.3 | 0.253148986 | 0.819692089 | 1.1619523 | up | lncRNA |
| ENST00000567465 | RP11-14N9.2 | 0.460431264 | 0.91221792 | 1.1544031 | up | lncRNA |
| ENST00000567474 | RP11-731K22.1 | 0.74050884 | 0.985094857 | 1.1744107 | up | lncRNA |
| ENST00000567484 | RP11-429B14.4 | 0.56782299 | 0.943923733 | 1.1157666 | up | lncRNA |
| ENST00000567527 | RP11-701H24.4 | 0.270382162 | 0.830995087 | 1.6334697 | up | lncRNA |
| ENST00000567540 | RP11-254F7.2 | 0.78619314 | 0.991305067 | 1.1486288 | up | lncRNA |
| ENST00000567574 | RP11-218F4.1 | 0.639485646 | 0.96564589 | 1.0570113 | up | lncRNA |
| ENST00000567594 | RP11-856B14.1 | 0.172824201 | 0.73449006 | 1.1205395 | up | lncRNA |
| ENST00000567624 | AC004125.3 | 0.237349148 | 0.806512655 | 3.7322998 | up | lncRNA |
| ENST00000567644 | RP11-941F15.1 | 0.577243921 | 0.947948855 | 1.0851765 | up | lncRNA |
| ENST00000567665 | RP11-345M22.1 | 0.75234391 | 0.986481248 | 1.0411461 | up | lncRNA |
| ENST00000567668 | LINC00273 | 0.507299608 | 0.925510828 | 1.0551795 | up | lncRNA |
| ENST00000567704 | RP11-114H24.1 | 0.993263257 | 0.999156445 | 1.0012743 | up | lncRNA |
| ENST00000567728 | RP11-327F22.2 | 0.025521104 | 0.381540504 | 2.7199044 | up | lncRNA |
| ENST00000567753 | RP11-524C21.2 | 0.464390552 | 0.913755581 | 1.2750534 | up | lncRNA |
| ENST00000567770 | RP11-932O9.4 | 0.4448307 | 0.90519468 | 1.1694691 | up | lncRNA |
| ENST00000567788 | RP11-474D1.3 | 0.921887355 | 0.998875101 | 1.009875 | up | lncRNA |
| ENST00000567829 | LA16c-358B7.3 | 0.045456841 | 0.482418324 | 1.11923 | up | lncRNA |
| ENST00000567853 | RP11-315F22.1 | 0.140853085 | 0.698568819 | 1.3055412 | up | lncRNA |
| ENST00000567904 | RP11-533E19.5 | 0.003665503 | 0.175033407 | 2.73732 | up | lncRNA |
| ENST00000567913 | RP11-384K6.6 | 0.844218488 | 0.995583664 | 1.0106248 | up | lncRNA |
| ENST00000567925 | RP11-554A11.7 | 0.317798014 | 0.851223284 | 1.4925653 | up | lncRNA |
| ENST00000567942 | RP11-152P23.2 | 0.412675911 | 0.890927603 | 1.0745817 | up | lncRNA |
| ENST00000568017 | RP11-541N10.3 | 0.277802861 | 0.832297359 | 1.2879033 | up | lncRNA |
| ENST00000568021 | RP4-668E10.1 | 0.270358575 | 0.830995087 | 1.3315697 | up | lncRNA |
| ENST00000568070 | FAM157C | 0.491133706 | 0.920777644 | 1.0637634 | up | lncRNA |
| ENST00000568083 | RP11-1166P10.9 | 0.917476777 | 0.998875101 | 1.0137049 | up | lncRNA |
| ENST00000568091 | RP11-616M22.6 | 0.025765504 | 0.382432679 | 1.4740414 | up | lncRNA |
| ENST00000568093 | RP11-133K1.7 | 0.041151099 | 0.462853115 | 1.3403037 | up | lncRNA |
| ENST00000568097 | HERC2P4 | 0.041752257 | 0.465644177 | 1.154673 | up | lncRNA |
| ENST00000568106 | LA16c-358B7.3 | 0.596181113 | 0.951580314 | 1.0711603 | up | lncRNA |
| ENST00000568134 | RP11-410D17.2 | 0.337184381 | 0.859588936 | 1.1136275 | up | lncRNA |
| ENST00000568137 | AC009120.4 | 0.591355879 | 0.950912706 | 1.1099156 | up | lncRNA |
| ENST00000568140 | RP11-252K23.1 | 0.329542138 | 0.85540421 | 1.0872916 | up | lncRNA |
| ENST00000568150 | RP11-42I10.1 | 0.712534698 | 0.98251396 | 1.067118 | up | lncRNA |
| ENST00000568177 | RP11-600F24.7 | 0.317399031 | 0.851223284 | 1.1122425 | up | lncRNA |
| ENST00000568184 | RP11-114H24.4 | 0.988265735 | 0.99906281 | 1.0010605 | up | lncRNA |
| ENST00000568229 | RP11-941F15.1 | 0.734979563 | 0.983763148 | 1.0180558 | up | lncRNA |
| ENST00000568244 | RP4-668J24.2 | 0.979860372 | 0.998875101 | 1.0061623 | up | lncRNA |
| ENST00000568280 | RP1-239B22.5 | 0.008464982 | 0.244209562 | 1.4994366 | up | lncRNA |
| ENST00000568302 | RP11-173B14.5 | 0.657605161 | 0.971482168 | 1.0603484 | up | lncRNA |
| ENST00000568337 | RP11-160C18.2 | 0.258570789 | 0.823936049 | 1.4558215 | up | lncRNA |
| ENST00000568344 | RP11-355I22.7 | 0.799776188 | 0.992228977 | 1.0766446 | up | lncRNA |
| ENST00000568362 | RP11-303E16.6 | 0.775903966 | 0.990727199 | 1.2128673 | up | lncRNA |
| ENST00000568384 | RP11-375I20.6 | 0.33648848 | 0.859588936 | 1.0756429 | up | lncRNA |
| ENST00000568389 | RP11-345M22.2 | 0.421203545 | 0.895572338 | 1.0496888 | up | lncRNA |
| ENST00000568414 | RP11-854K16.3 | 0.240130858 | 0.80919875 | 1.1193073 | up | lncRNA |
| ENST00000568425 | RP11-275F13.1 | 0.165290842 | 0.719579787 | 1.9405602 | up | lncRNA |
| ENST00000568427 | RP11-21B23.1 | 0.465447074 | 0.913821992 | 1.1056318 | up | lncRNA |
| ENST00000568430 | RP11-403P17.2 | 0.236062649 | 0.805225869 | 1.1548842 | up | lncRNA |
| ENST00000568457 | RP4-647J21.1 | 0.000706591 | 0.09566979 | 7.0990281 | up | lncRNA |
| ENST00000568458 | RP11-558A11.3 | 0.247382439 | 0.814489937 | 1.0686712 | up | lncRNA |
| ENST00000568471 | RP11-883G14.3 | 0.005531583 | 0.205671704 | 1.9213679 | up | lncRNA |
| ENST00000568506 | RP11-455F5.5 | 0.44336233 | 0.90512482 | 1.0750894 | up | lncRNA |
| ENST00000568525 | RP11-532F12.5 | 0.682828537 | 0.977836377 | 1.1233217 | up | lncRNA |
| ENST00000568541 | RP11-580I1.1 | 0.062978493 | 0.53610162 | 1.193622 | up | lncRNA |
| ENST00000568571 | RP11-469M7.1 | 0.761273922 | 0.988045734 | 1.1341992 | up | lncRNA |
| ENST00000568587 | RP11-863P13.2 | 0.598671911 | 0.952796892 | 1.153388 | up | lncRNA |
| ENST00000568659 | RP11-351A20.2 | 0.41004639 | 0.890549744 | 1.1090704 | up | lncRNA |
| ENST00000568695 | RP11-92G12.3 | 0.957021084 | 0.998875101 | 1.0184351 | up | lncRNA |
| ENST00000568735 | RP11-173B14.5 | 0.540694391 | 0.937855369 | 1.1042926 | up | lncRNA |
| ENST00000568764 | RP11-264L1.3 | 0.515603873 | 0.930031728 | 1.1327428 | up | lncRNA |
| ENST00000568775 | IGHV3OR16-6 | 0.033619605 | 0.42757611 | 1.3680158 | up | lncRNA |
| ENST00000568776 | RP11-525K10.3 | 0.781771556 | 0.99121281 | 1.0160685 | up | lncRNA |
| ENST00000568833 | CTD-2012K14.1 | 0.287388014 | 0.836961572 | 1.1859643 | up | lncRNA |
| ENST00000568835 | CTD-2576F9.2 | 0.984787231 | 0.998971647 | 1.0049075 | up | lncRNA |
| ENST00000568836 | RP11-2E17.2 | 0.474718167 | 0.917441334 | 1.046354 | up | lncRNA |
| ENST00000568884 | RP11-616M22.1 | 0.516855079 | 0.930031728 | 1.0585152 | up | lncRNA |
| ENST00000568947 | AC137934.1 | 0.697903894 | 0.980170937 | 1.0485094 | up | lncRNA |
| ENST00000568958 | RP11-223I10.1 | 0.31347861 | 0.851097229 | 1.1395097 | up | lncRNA |
| ENST00000568966 | RP11-71H17.7 | 0.858601299 | 0.996142678 | 1.0341117 | up | lncRNA |
| ENST00000568970 | ERVK13-1 | 0.902710476 | 0.998875101 | 1.0268805 | up | lncRNA |
| ENST00000569008 | RP11-434B12.1 | 0.398248937 | 0.887618401 | 1.2253163 | up | lncRNA |
| ENST00000569009 | RP11-883G14.4 | 0.064087672 | 0.537886703 | 1.181498 | up | lncRNA |
| ENST00000569013 | DEFB122 | 0.739288121 | 0.984985435 | 1.0268027 | up | lncRNA |
| ENST00000569032 | RP11-538I12.2 | 0.836399735 | 0.995078046 | 1.0567232 | up | lncRNA |
| ENST00000569034 | RP11-15N24.4 | 0.677283024 | 0.976096237 | 1.0652937 | up | lncRNA |
| ENST00000569039 | AC009133.20 | 0.136702474 | 0.693269931 | 1.1177207 | up | lncRNA |
| ENST00000569048 | CTA-481E9.4 | 0.320924651 | 0.852658967 | 1.0739067 | up | lncRNA |
| ENST00000569130 | CTD-2639E6.4 | 0.570526701 | 0.944646886 | 1.0530145 | up | lncRNA |
| ENST00000569178 | RP11-19D2.2 | 0.184411289 | 0.751257244 | 1.2150403 | up | lncRNA |
| ENST00000569195 | RP5-991G20.4 | 0.830896475 | 0.994074191 | 1.0447945 | up | lncRNA |
| ENST00000569274 | RP11-63M22.2 | 0.070662542 | 0.559211931 | 1.1413333 | up | lncRNA |
| ENST00000569288 | RP11-29B2.4 | 0.226347233 | 0.795886026 | 1.4441361 | up | lncRNA |
| ENST00000569291 | RP11-452L6.1 | 0.07172755 | 0.560238874 | 1.0399778 | up | lncRNA |
| ENST00000569326 | CTD-2517M14.5 | 0.004598309 | 0.189321509 | 1.2537729 | up | lncRNA |
| ENST00000569328 | RP11-700H13.1 | 0.05889272 | 0.52904871 | 1.6873584 | up | lncRNA |
| ENST00000569334 | VENTXP1 | 0.739476709 | 0.985094857 | 1.0813642 | up | lncRNA |
| ENST00000569345 | CTA-363E6.7 | 0.922143946 | 0.998875101 | 1.015359 | up | lncRNA |
| ENST00000569362 | LA16c-444G7.2 | 0.070733107 | 0.559211931 | 1.2498663 | up | lncRNA |
| ENST00000569373 | RP13-379O24.2 | 0.102722921 | 0.632351488 | 1.2286756 | up | lncRNA |
| ENST00000569428 | RP11-554A11.6 | 0.752164317 | 0.986481248 | 1.0293037 | up | lncRNA |
| ENST00000569432 | RP11-554A11.6 | 0.99558369 | 0.999156445 | 1.0009158 | up | lncRNA |
| ENST00000569467 | CTD-2026K11.1 | 0.374926204 | 0.879312727 | 1.239238 | up | lncRNA |
| ENST00000569484 | RP11-586K12.13 | 0.525385486 | 0.932863107 | 1.1138495 | up | lncRNA |
| ENST00000569543 | CTC-297N7.1 | 0.45932679 | 0.91221792 | 1.0872156 | up | lncRNA |
| ENST00000569583 | RP11-220I1.5 | 0.282394747 | 0.832629955 | 1.4325944 | up | lncRNA |
| ENST00000569586 | RP11-506G7.1 | 0.413111978 | 0.890927603 | 1.0642642 | up | lncRNA |
| ENST00000569596 | RP11-24M17.4 | 0.854530619 | 0.996142678 | 1.0074897 | up | lncRNA |
| ENST00000569618 | RP11-354P17.15 | 0.727444564 | 0.98251396 | 1.0496223 | up | lncRNA |
| ENST00000569625 | RP11-1012E15.1 | 0.364960649 | 0.874818743 | 1.0902941 | up | lncRNA |
| ENST00000569670 | LA16c-361A3.3 | 0.085917147 | 0.594543344 | 1.2408093 | up | lncRNA |
| ENST00000569697 | HERC2P5 | 0.723882736 | 0.98251396 | 1.0622861 | up | lncRNA |
| ENST00000569713 | RP11-142G1.2 | 0.676968701 | 0.976096237 | 1.0487111 | up | lncRNA |
| ENST00000569742 | RP11-797A18.4 | 0.486900524 | 0.920684627 | 1.0786568 | up | lncRNA |
| ENST00000569752 | AC002310.12 | 0.717328801 | 0.98251396 | 1.0507809 | up | lncRNA |
| ENST00000569754 | RP11-423G4.7 | 0.065699087 | 0.543152821 | 1.287151 | up | lncRNA |
| ENST00000569756 | AC002519.6 | 0.310239921 | 0.848664472 | 1.0927341 | up | lncRNA |
| ENST00000569778 | RP11-249C24.10 | 0.411980682 | 0.890876411 | 1.0345439 | up | lncRNA |
| ENST00000569846 | RP11-160C18.2 | 0.013267412 | 0.290336527 | 1.4628074 | up | lncRNA |
| ENST00000569881 | AC002551.1 | 0.687335467 | 0.97861545 | 1.0805716 | up | lncRNA |
| ENST00000569893 | RNF126P1 | 0.547248559 | 0.940721096 | 1.0378896 | up | lncRNA |
| ENST00000569940 | RP11-401P9.1 | 0.002470715 | 0.146699107 | 1.2391247 | up | lncRNA |
| ENST00000569966 | AP001505.9 | 0.71217788 | 0.98251396 | 1.221545 | up | lncRNA |
| ENST00000569974 | RP11-264B17.2 | 0.899791772 | 0.998875101 | 1.0119787 | up | lncRNA |
| ENST00000569981 | AC009133.14 | 0.807745007 | 0.992578294 | 1.0385533 | up | lncRNA |
| ENST00000569999 | RP11-340F14.5 | 0.259453876 | 0.824094195 | 1.0964114 | up | lncRNA |
| ENST00000570008 | RP11-553E24.2 | 0.602906494 | 0.954962366 | 1.3218059 | up | lncRNA |
| ENST00000570024 | AC140542.2 | 0.827066585 | 0.993892626 | 1.0255339 | up | lncRNA |
| ENST00000570072 | RP11-304L19.1 | 0.246329632 | 0.813853858 | 1.1425087 | up | lncRNA |
| ENST00000570084 | RP11-586K12.10 | 0.280294225 | 0.832297359 | 1.1854354 | up | lncRNA |
| ENST00000570096 | RP4-616B8.4 | 0.223522995 | 0.792741738 | 1.1320108 | up | lncRNA |
| ENST00000570105 | RP11-107D24.2 | 0.134608725 | 0.692112025 | 1.2963509 | up | lncRNA |
| ENST00000570118 | RP11-243A14.3 | 0.112335431 | 0.651593972 | 1.3757914 | up | lncRNA |
| ENST00000570141 | RP11-439E19.9 | 0.925164229 | 0.998875101 | 1.0335248 | up | lncRNA |
| ENST00000570167 | RP11-401P9.5 | 0.824319659 | 0.993856674 | 1.0271888 | up | lncRNA |
| ENST00000570207 | RP11-506G7.1 | 0.39502422 | 0.885711362 | 1.0579861 | up | lncRNA |
| ENST00000570221 | ULK4P2 | 0.524331932 | 0.932117592 | 1.1371785 | up | lncRNA |
| ENST00000570266 | RP11-196G11.2 | 0.003525462 | 0.172100128 | 2.5850789 | up | lncRNA |
| ENST00000570288 | RP11-1437A8.5 | 0.849806447 | 0.995900317 | 1.0257223 | up | lncRNA |
| ENST00000570290 | RP11-77H9.6 | 0.384133765 | 0.882658948 | 1.5794335 | up | lncRNA |
| ENST00000570301 | RP11-1055B8.3 | 0.717637454 | 0.98251396 | 1.0601473 | up | lncRNA |
| ENST00000570309 | RP11-334C17.5 | 0.516201275 | 0.930031728 | 1.0883203 | up | lncRNA |
| ENST00000570379 | RP11-156P1.3 | 0.410279784 | 0.890549744 | 1.0702425 | up | lncRNA |
| ENST00000570413 | RP11-149I9.2 | 0.186287108 | 0.753336876 | 1.375225 | up | lncRNA |
| ENST00000570416 | MIR22HG | 0.690762973 | 0.978695455 | 1.1648501 | up | lncRNA |
| ENST00000570440 | RP11-876N24.5 | 0.364447251 | 0.874818743 | 1.0936214 | up | lncRNA |
| ENST00000570501 | RP11-1260E13.1 | 0.793417269 | 0.991420185 | 1.0400953 | up | lncRNA |
| ENST00000570512 | RP11-353N14.1 | 0.007498352 | 0.230441855 | 1.3758228 | up | lncRNA |
| ENST00000570515 | LA16c-380H5.2 | 0.476019131 | 0.917441334 | 1.0857106 | up | lncRNA |
| ENST00000570572 | CTD-2033D24.2 | 0.913937615 | 0.998875101 | 1.0088187 | up | lncRNA |
| ENST00000570677 | CTD-2270P14.3 | 0.153530024 | 0.711006819 | 1.1522983 | up | lncRNA |
| ENST00000570835 | ALOX12P2 | 0.807887512 | 0.992578294 | 1.0596287 | up | lncRNA |
| ENST00000570843 | RP11-473M20.16 | 0.140480478 | 0.698568819 | 1.3258894 | up | lncRNA |
| ENST00000570869 | RP13-638C3.2 | 0.09061054 | 0.604992392 | 1.3347788 | up | lncRNA |
| ENST00000570919 | RP11-388C12.5 | 0.233806113 | 0.80277161 | 1.0690765 | up | lncRNA |
| ENST00000570924 | RP11-818O24.3 | 0.133656934 | 0.689601422 | 1.0836328 | up | lncRNA |
| ENST00000570929 | RP11-1055B8.3 | 0.089722387 | 0.603136158 | 1.1152681 | up | lncRNA |
| ENST00000570949 | RP11-473M20.7 | 0.939816085 | 0.998875101 | 1.0122358 | up | lncRNA |
| ENST00000571067 | RP11-81A22.5 | 0.639138162 | 0.96564589 | 1.0594119 | up | lncRNA |
| ENST00000571085 | RP11-455O6.2 | 0.789464632 | 0.991420185 | 1.0314948 | up | lncRNA |
| ENST00000571138 | AC012146.7 | 0.747227004 | 0.985094857 | 1.05383 | up | lncRNA |
| ENST00000571152 | LINC00514 | 0.736741679 | 0.984316636 | 1.1068199 | up | lncRNA |
| ENST00000571188 | RP3-406A7.3 | 0.374751128 | 0.879312727 | 1.3427657 | up | lncRNA |
| ENST00000571512 | RP11-1260E13.3 | 0.846882045 | 0.995583664 | 1.0251696 | up | lncRNA |
| ENST00000571523 | RP11-44F14.11 | 0.974536472 | 0.998875101 | 1.0041689 | up | lncRNA |
| ENST00000571591 | CTD-2561B21.3 | 0.781514251 | 0.99121281 | 1.0326248 | up | lncRNA |
| ENST00000571595 | MIR22HG | 0.788898627 | 0.991420185 | 1.0341175 | up | lncRNA |
| ENST00000571665 | RP11-156P1.3 | 0.199404271 | 0.766712248 | 1.1165632 | up | lncRNA |
| ENST00000571724 | RP11-1055B8.4 | 0.076773732 | 0.571502384 | 1.7440203 | up | lncRNA |
| ENST00000571772 | RP11-297D21.3 | 0.733039793 | 0.983763148 | 1.0441469 | up | lncRNA |
| ENST00000571911 | RP11-517A5.4 | 0.133731308 | 0.689601422 | 1.6369013 | up | lncRNA |
| ENST00000571933 | LA16c-313D11.9 | 0.717228419 | 0.98251396 | 1.0816321 | up | lncRNA |
| ENST00000571975 | RP11-96D1.10 | 0.721453521 | 0.98251396 | 1.0690377 | up | lncRNA |
| ENST00000572067 | CTC-479C5.10 | 0.41032404 | 0.890549744 | 1.2712849 | up | lncRNA |
| ENST00000572086 | LINC00514 | 0.706093719 | 0.981012299 | 1.0760131 | up | lncRNA |
| ENST00000572124 | RP11-504P24.6 | 0.401602602 | 0.8890764 | 1.1640581 | up | lncRNA |
| ENST00000572193 | RP5-867C24.5 | 0.429578379 | 0.898463455 | 1.3654735 | up | lncRNA |
| ENST00000572222 | RP11-473M20.7 | 0.330592417 | 0.855705627 | 1.0307266 | up | lncRNA |
| ENST00000572284 | RP3-509I19.6 | 0.078475956 | 0.57678507 | 1.032446 | up | lncRNA |
| ENST00000572287 | AC099684.1 | 0.079160632 | 0.577880666 | 1.1270604 | up | lncRNA |
| ENST00000572301 | RP11-1055B8.3 | 0.481727702 | 0.9192486 | 1.0994271 | up | lncRNA |
| ENST00000572343 | AC139099.4 | 0.107205795 | 0.639452435 | 1.1520889 | up | lncRNA |
| ENST00000572353 | RP11-353N14.4 | 0.264072312 | 0.827574498 | 1.0927657 | up | lncRNA |
| ENST00000572404 | RP11-667K14.4 | 0.662269715 | 0.971730383 | 1.0774254 | up | lncRNA |
| ENST00000572427 | RP11-473M20.7 | 0.453121702 | 0.909381287 | 1.0491426 | up | lncRNA |
| ENST00000572479 | RP11-65J21.4 | 0.320994732 | 0.852658967 | 1.3631779 | up | lncRNA |
| ENST00000572609 | LINC00085 | 0.566232845 | 0.943849275 | 1.1922854 | up | lncRNA |
| ENST00000572790 | RP11-667K14.3 | 0.656048063 | 0.971258623 | 1.4496792 | up | lncRNA |
| ENST00000572792 | RP11-420A6.2 | 0.626515991 | 0.962096887 | 1.0580716 | up | lncRNA |
| ENST00000572811 | RP11-490O6.2 | 0.153575888 | 0.711006819 | 1.3801581 | up | lncRNA |
| ENST00000572864 | RP11-156P1.3 | 0.977240419 | 0.998875101 | 1.0112875 | up | lncRNA |
| ENST00000573037 | BCAR4 | 0.413004642 | 0.890927603 | 1.0693936 | up | lncRNA |
| ENST00000573130 | RP11-473M20.7 | 0.778343034 | 0.99121281 | 1.0308093 | up | lncRNA |
| ENST00000573151 | LINC00085 | 0.496326206 | 0.921717611 | 1.261919 | up | lncRNA |
| ENST00000573177 | RP11-874J12.1 | 0.006355751 | 0.21515583 | 2.1188304 | up | lncRNA |
| ENST00000573220 | RP11-95P2.3 | 0.975437307 | 0.998875101 | 1.0164554 | up | lncRNA |
| ENST00000573268 | RP11-95P2.1 | 0.477570788 | 0.917715179 | 1.0723412 | up | lncRNA |
| ENST00000573312 | AC144831.1 | 0.786260541 | 0.991305067 | 1.1150654 | up | lncRNA |
| ENST00000573341 | RP11-156P1.3 | 0.175027799 | 0.737088901 | 1.1749834 | up | lncRNA |
| ENST00000573346 | RP11-334C17.5 | 0.038016699 | 0.44612892 | 1.2355313 | up | lncRNA |
| ENST00000573355 | RP11-874J12.2 | 0.000254807 | 0.073139875 | 1.5187243 | up | lncRNA |
| ENST00000573371 | RP11-810M2.2 | 0.26094476 | 0.82544125 | 1.0883277 | up | lncRNA |
| ENST00000573379 | RP11-876N24.4 | 0.003816388 | 0.17862549 | 2.6992063 | up | lncRNA |
| ENST00000573382 | RP1-302G2.5 | 0.508519216 | 0.92652835 | 1.1352381 | up | lncRNA |
| ENST00000573394 | CTD-2047H16.4 | 0.874036599 | 0.997909851 | 1.0151357 | up | lncRNA |
| ENST00000573479 | AC021224.1 | 0.819887291 | 0.993333549 | 1.0484413 | up | lncRNA |
| ENST00000573609 | LA16c-349E10.1 | 0.223525532 | 0.792741738 | 1.169712 | up | lncRNA |
| ENST00000573819 | RP11-189E14.3 | 0.383511171 | 0.882499972 | 1.1895122 | up | lncRNA |
| ENST00000573856 | CTA-972D3.2 | 0.728961782 | 0.982841582 | 1.072938 | up | lncRNA |
| ENST00000573861 | RP11-498D10.6 | 0.025739596 | 0.382432679 | 1.1547095 | up | lncRNA |
| ENST00000573896 | LINC00085 | 0.147898194 | 0.704258422 | 1.7161912 | up | lncRNA |
| ENST00000573901 | RP11-64J4.2 | 0.596196361 | 0.951580314 | 1.0844501 | up | lncRNA |
| ENST00000573953 | RP11-473M20.7 | 0.477086831 | 0.917715179 | 1.1044797 | up | lncRNA |
| ENST00000574016 | MIR22HG | 0.683593308 | 0.977836377 | 1.0212101 | up | lncRNA |
| ENST00000574072 | LINC00085 | 0.189383226 | 0.756484902 | 1.4234076 | up | lncRNA |
| ENST00000574183 | ALOX12P2 | 0.239458031 | 0.808658327 | 1.2925335 | up | lncRNA |
| ENST00000574260 | RP5-1050D4.5 | 0.204183077 | 0.771026327 | 1.1531766 | up | lncRNA |
| ENST00000574267 | RP11-1113L8.1 | 0.662891671 | 0.971730383 | 1.071598 | up | lncRNA |
| ENST00000574472 | RP11-1055B8.2 | 0.515227917 | 0.930031728 | 1.0784275 | up | lncRNA |
| ENST00000574560 | RP11-676J12.8 | 0.093907292 | 0.61382632 | 3.1936096 | up | lncRNA |
| ENST00000574565 | APOC1P1 | 0.623915051 | 0.96130052 | 1.0442887 | up | lncRNA |
| ENST00000574705 | RP11-295D4.1 | 0.543280774 | 0.939697392 | 1.0597098 | up | lncRNA |
| ENST00000574716 | RP11-515O17.2 | 0.700088791 | 0.980170937 | 1.0489388 | up | lncRNA |
| ENST00000574741 | RP11-156P1.3 | 0.439809529 | 0.903801026 | 1.2400348 | up | lncRNA |
| ENST00000574826 | RP11-670E13.5 | 0.735415046 | 0.983843842 | 1.0983418 | up | lncRNA |
| ENST00000575126 | RP11-63A1.2 | 0.959161934 | 0.998875101 | 1.0046771 | up | lncRNA |
| ENST00000575193 | ANKRD36BP2 | 0.389917158 | 0.885317362 | 1.2040808 | up | lncRNA |
| ENST00000575202 | CTD-2377D24.8 | 0.743425057 | 0.985094857 | 1.0739113 | up | lncRNA |
| ENST00000575312 | RP13-1032I1.7 | 0.36003632 | 0.873342057 | 1.0869031 | up | lncRNA |
| ENST00000575402 | RP1-59D14.6 | 0.227200569 | 0.796867685 | 1.1379935 | up | lncRNA |
| ENST00000575424 | CTD-2135D7.5 | 0.611172159 | 0.957448851 | 1.137365 | up | lncRNA |
| ENST00000575554 | LRRC37A6P | 0.909776491 | 0.998875101 | 1.0074031 | up | lncRNA |
| ENST00000575562 | RP11-2C24.6 | 0.259890492 | 0.824094195 | 1.1293755 | up | lncRNA |
| ENST00000575606 | RP11-874J12.2 | 0.001015244 | 0.114037466 | 1.4789565 | up | lncRNA |
| ENST00000575787 | ALOX12P2 | 0.340794571 | 0.861561333 | 1.3006969 | up | lncRNA |
| ENST00000575792 | RP11-65J21.3 | 0.996937501 | 0.999156445 | 1.0010871 | up | lncRNA |
| ENST00000575917 | RP11-327F22.4 | 0.915282621 | 0.998875101 | 1.010005 | up | lncRNA |
| ENST00000575924 | OR7E5P | 0.262341372 | 0.82544125 | 1.1427309 | up | lncRNA |
| ENST00000576014 | RP11-166P13.4 | 0.296485238 | 0.839987155 | 1.2145482 | up | lncRNA |
| ENST00000576021 | RP11-498C9.3 | 0.636079352 | 0.96564589 | 1.0629269 | up | lncRNA |
| ENST00000576080 | AC005356.1 | 0.3796092 | 0.880257051 | 2.4205744 | up | lncRNA |
| ENST00000576093 | LINC00085 | 0.955847277 | 0.998875101 | 1.0182454 | up | lncRNA |
| ENST00000576138 | CTC-281F24.1 | 0.242520838 | 0.809272444 | 1.3035765 | up | lncRNA |
| ENST00000576169 | RP11-461A8.5 | 0.754563374 | 0.987297642 | 1.0774294 | up | lncRNA |
| ENST00000576220 | RP11-1113L8.2 | 0.015064975 | 0.310650165 | 1.346675 | up | lncRNA |
| ENST00000576266 | ROCK1P1 | 0.526668494 | 0.933927598 | 1.0734925 | up | lncRNA |
| ENST00000576302 | CTD-2031P19.5 | 0.001912118 | 0.141494996 | 1.8498825 | up | lncRNA |
| ENST00000576345 | RP11-290H9.4 | 0.110030056 | 0.645372325 | 1.1762319 | up | lncRNA |
| ENST00000576476 | RP1-302G2.5 | 0.296458538 | 0.839987155 | 1.1225083 | up | lncRNA |
| ENST00000576494 | LINC00085 | 0.293978603 | 0.838742488 | 1.2229542 | up | lncRNA |
| ENST00000576554 | RP11-498C9.3 | 0.800474649 | 0.99243367 | 1.0290891 | up | lncRNA |
| ENST00000576606 | RP11-874J12.2 | 0.002889273 | 0.157315299 | 1.4417057 | up | lncRNA |
| ENST00000576636 | ALOX12P2 | 0.969438338 | 0.998875101 | 1.015695 | up | lncRNA |
| ENST00000576671 | RP11-189E14.5 | 0.245203055 | 0.811577674 | 1.1978408 | up | lncRNA |
| ENST00000576749 | MIR22HG | 0.19428435 | 0.760765035 | 1.250573 | up | lncRNA |
| ENST00000576762 | AJ003147.8 | 0.661280534 | 0.971704562 | 1.1233729 | up | lncRNA |
| ENST00000576784 | RP11-498C9.2 | 0.100833548 | 0.628381708 | 1.121146 | up | lncRNA |
| ENST00000576808 | CTD-2047H16.2 | 0.438669258 | 0.903449725 | 1.1604841 | up | lncRNA |
| ENST00000576825 | RP11-818O24.3 | 0.197106467 | 0.763327268 | 1.0858807 | up | lncRNA |
| ENST00000576871 | RP11-166P13.3 | 0.715740145 | 0.98251396 | 1.050929 | up | lncRNA |
| ENST00000576912 | RP13-638C3.4 | 0.230445096 | 0.79852974 | 1.1515992 | up | lncRNA |
| ENST00000576975 | LINC00085 | 0.714134726 | 0.98251396 | 1.0275854 | up | lncRNA |
| ENST00000577000 | LINC00482 | 0.720903501 | 0.98251396 | 1.0421277 | up | lncRNA |
| ENST00000577048 | AF001548.6 | 0.50446065 | 0.92405258 | 1.1178687 | up | lncRNA |
| ENST00000577055 | CTD-2270P14.3 | 0.426004029 | 0.897780553 | 1.2219495 | up | lncRNA |
| ENST00000577061 | CTD-2561B21.7 | 0.32788542 | 0.854556781 | 1.0264988 | up | lncRNA |
| ENST00000577089 | RP11-515E23.1 | 0.266408735 | 0.828881561 | 1.4088505 | up | lncRNA |
| ENST00000577140 | LA16c-325D7.1 | 0.142993743 | 0.69945173 | 1.2324835 | up | lncRNA |
| ENST00000577164 | MIR22HG | 0.587644278 | 0.950589384 | 1.1410657 | up | lncRNA |
| ENST00000577173 | RP11-429K17.1 | 0.20497271 | 0.771727492 | 1.5716616 | up | lncRNA |
| ENST00000577175 | RP11-893F2.15 | 0.42011744 | 0.894854783 | 1.3975914 | up | lncRNA |
| ENST00000577176 | RP13-580F15.2 | 0.185110448 | 0.751649101 | 1.2426527 | up | lncRNA |
| ENST00000577181 | CTC-297N7.7 | 0.425269973 | 0.897780553 | 1.1140634 | up | lncRNA |
| ENST00000577189 | RP11-118E18.2 | 0.332474068 | 0.857061033 | 1.052205 | up | lncRNA |
| ENST00000577295 | RP11-309N17.4 | 0.114504459 | 0.656559327 | 1.0922167 | up | lncRNA |
| ENST00000577325 | AC010761.9 | 0.777536532 | 0.991152912 | 1.0650818 | up | lncRNA |
| ENST00000577327 | RP11-91I8.3 | 0.41568343 | 0.892358512 | 1.3514038 | up | lncRNA |
| ENST00000577382 | RP11-100K18.1 | 0.826921072 | 0.993856674 | 1.0178668 | up | lncRNA |
| ENST00000577423 | RP11-583F2.3 | 0.895214621 | 0.998875101 | 1.0250628 | up | lncRNA |
| ENST00000577546 | RP11-118G23.2 | 0.592387661 | 0.950912706 | 1.0447636 | up | lncRNA |
| ENST00000577560 | CTD-2006K23.1 | 0.392751291 | 0.885711362 | 1.0908912 | up | lncRNA |
| ENST00000577584 | RP11-118G23.1 | 0.001341622 | 0.127516283 | 1.3384458 | up | lncRNA |
| ENST00000577672 | RP1-283E3.4 | 0.54496065 | 0.939697392 | 1.0348474 | up | lncRNA |
| ENST00000577684 | RP1-37N7.3 | 0.996712978 | 0.999156445 | 1.0005258 | up | lncRNA |
| ENST00000577698 | RP11-147L13.2 | 0.662986616 | 0.971730383 | 1.0903119 | up | lncRNA |
| ENST00000577709 | CTC-304I17.2 | 0.714508637 | 0.98251396 | 1.0858237 | up | lncRNA |
| ENST00000577719 | RP11-78F17.1 | 0.476547949 | 0.917651513 | 1.2010106 | up | lncRNA |
| ENST00000577743 | RP11-401O9.3 | 0.841968544 | 0.995583664 | 1.1641892 | up | lncRNA |
| ENST00000577746 | RP11-19P22.8 | 0.188930071 | 0.756051633 | 1.1424618 | up | lncRNA |
| ENST00000577781 | RP11-573D15.9 | 0.208113258 | 0.775639766 | 1.1847683 | up | lncRNA |
| ENST00000577806 | RP11-231E4.4 | 0.89692089 | 0.998875101 | 1.0198417 | up | lncRNA |
| ENST00000577807 | RP11-599B13.3 | 0.206399799 | 0.773204016 | 1.4054602 | up | lncRNA |
| ENST00000577828 | LINC00511 | 0.691135813 | 0.978695455 | 1.0597335 | up | lncRNA |
| ENST00000577847 | RP11-258F1.1 | 0.74158875 | 0.985094857 | 1.0447356 | up | lncRNA |
| ENST00000577848 | RP11-874J12.4 | 0.041228261 | 0.462995303 | 2.5491757 | up | lncRNA |
| ENST00000577863 | AC005358.1 | 0.81427318 | 0.99257857 | 1.0991228 | up | lncRNA |
| ENST00000577889 | AC005154.6 | 0.451011663 | 0.908000742 | 1.0567719 | up | lncRNA |
| ENST00000577995 | RP11-874J12.2 | 0.913183788 | 0.998875101 | 1.0239268 | up | lncRNA |
| ENST00000578003 | OR3A4P | 0.804608252 | 0.992578294 | 1.0871087 | up | lncRNA |
| ENST00000578024 | RPL21P122 | 0.132743957 | 0.688522325 | 1.1853495 | up | lncRNA |
| ENST00000578030 | CTD-2382H12.2 | 0.258518791 | 0.823936049 | 1.2159143 | up | lncRNA |
| ENST00000578036 | PLEKHM1P | 0.986828974 | 0.99906281 | 1.0041297 | up | lncRNA |
| ENST00000578037 | AC022596.2 | 0.496160506 | 0.921717611 | 1.1142292 | up | lncRNA |
| ENST00000578084 | KIRREL3-AS2 | 0.05669101 | 0.523571026 | 1.2879843 | up | lncRNA |
| ENST00000578216 | RP11-334E6.12 | 0.670397897 | 0.974341942 | 1.1121888 | up | lncRNA |
| ENST00000578406 | RP11-218E15.1 | 0.123922117 | 0.67522028 | 1.2446776 | up | lncRNA |
| ENST00000578482 | RP11-138C9.1 | 0.388272223 | 0.88524429 | 1.0416991 | up | lncRNA |
| ENST00000578492 | RP13-104F24.2 | 0.1475307 | 0.703667281 | 1.2163474 | up | lncRNA |
| ENST00000578500 | RP11-360L9.7 | 0.986301911 | 0.99906281 | 1.0144323 | up | lncRNA |
| ENST00000578583 | RP11-13N13.6 | 0.324675976 | 0.854254771 | 1.161192 | up | lncRNA |
| ENST00000578610 | RP11-202D1.3 | 0.062545704 | 0.534813875 | 1.2932281 | up | lncRNA |
| ENST00000578633 | RP11-484N16.1 | 0.496685046 | 0.921717611 | 1.0832421 | up | lncRNA |
| ENST00000578650 | RP1-41C23.1 | 0.189723353 | 0.756884941 | 1.8836732 | up | lncRNA |
| ENST00000578740 | RP11-126K15.1 | 0.873118028 | 0.997909851 | 1.0204259 | up | lncRNA |
| ENST00000578741 | RP11-627G18.4 | 0.030746517 | 0.415683747 | 3.0569513 | up | lncRNA |
| ENST00000578800 | RP13-270P17.1 | 0.759252361 | 0.988042026 | 1.0904619 | up | lncRNA |
| ENST00000578803 | RP11-111H3.2 | 0.313923664 | 0.851137751 | 1.0651828 | up | lncRNA |
| ENST00000578831 | RP11-739L10.1 | 0.654350225 | 0.970670225 | 1.037489 | up | lncRNA |
| ENST00000578881 | RP11-1109M24.5 | 0.015829717 | 0.317148864 | 1.1811277 | up | lncRNA |
| ENST00000578897 | RP11-789C17.1 | 0.026408331 | 0.385842137 | 1.2069059 | up | lncRNA |
| ENST00000578923 | RP11-305N23.1 | 0.598910816 | 0.952945458 | 1.0576803 | up | lncRNA |
| ENST00000578967 | RP11-676J15.1 | 0.51109418 | 0.927835265 | 1.1331173 | up | lncRNA |
| ENST00000579003 | hsa-mir-142 | 0.976532044 | 0.998875101 | 1.0048835 | up | lncRNA |
| ENST00000579012 | RP11-146G7.3 | 0.66039964 | 0.971704562 | 1.2261607 | up | lncRNA |
| ENST00000579019 | RP11-192H23.6 | 0.983689461 | 0.998875101 | 1.0011493 | up | lncRNA |
| ENST00000579095 | RP13-20L14.6 | 0.448335178 | 0.906140892 | 1.1014829 | up | lncRNA |
| ENST00000579114 | CTC-297N7.5 | 0.361355608 | 0.873342057 | 1.0427879 | up | lncRNA |
| ENST00000579160 | RP11-746B8.1 | 0.337790969 | 0.860180267 | 1.1579839 | up | lncRNA |
| ENST00000579174 | AC005154.6 | 0.761412055 | 0.988045734 | 1.0539699 | up | lncRNA |
| ENST00000579339 | LINC00271 | 0.154972947 | 0.711341825 | 1.1539364 | up | lncRNA |
| ENST00000579367 | CTC-297N7.9 | 0.228390346 | 0.798130144 | 1.1869812 | up | lncRNA |
| ENST00000579383 | SNHG15 | 3.82788E-05 | 0.025956561 | 2.3679391 | up | lncRNA |
| ENST00000579431 | RP11-627G18.3 | 0.158122367 | 0.714689048 | 1.7547703 | up | lncRNA |
| ENST00000579467 | RP11-143J12.3 | 0.030555786 | 0.415683747 | 1.2821441 | up | lncRNA |
| ENST00000579468 | AC010761.10 | 0.774476483 | 0.990435061 | 1.0119932 | up | lncRNA |
| ENST00000579480 | RP11-671C19.2 | 0.74301843 | 0.985094857 | 1.1391592 | up | lncRNA |
| ENST00000579499 | CTB-58E17.9 | 0.59370763 | 0.951058356 | 1.0381378 | up | lncRNA |
| ENST00000579506 | RP11-202D1.3 | 0.064386267 | 0.539271375 | 1.7868249 | up | lncRNA |
| ENST00000579529 | CTC-297N7.9 | 0.891150835 | 0.998875101 | 1.0275426 | up | lncRNA |
| ENST00000579581 | RP11-697E2.7 | 0.83162297 | 0.994074191 | 1.0343197 | up | lncRNA |
| ENST00000579588 | RP11-848P1.9 | 0.647245562 | 0.969646614 | 1.0243175 | up | lncRNA |
| ENST00000579595 | RP11-769O8.2 | 0.389089087 | 0.885276359 | 1.2178891 | up | lncRNA |
| ENST00000579633 | AC124789.1 | 0.913077588 | 0.998875101 | 1.0266877 | up | lncRNA |
| ENST00000579673 | RP11-20B24.7 | 0.319737096 | 0.852213764 | 1.2284327 | up | lncRNA |
| ENST00000579693 | MTND1P15 | 0.198150671 | 0.764889305 | 1.1291778 | up | lncRNA |
| ENST00000579805 | RP11-789C17.5 | 0.113772593 | 0.654979643 | 1.3284135 | up | lncRNA |
| ENST00000579914 | CTC-297N7.7 | 0.768530668 | 0.989189555 | 1.0357991 | up | lncRNA |
| ENST00000579981 | RP11-498C9.16 | 0.299548869 | 0.8422517 | 1.0987517 | up | lncRNA |
| ENST00000580056 | RP11-344E13.3 | 0.723944462 | 0.98251396 | 1.0319278 | up | lncRNA |
| ENST00000580121 | RP1-56K13.3 | 0.272946421 | 0.832297359 | 1.2753201 | up | lncRNA |
| ENST00000580194 | AC005324.6 | 0.284070663 | 0.834250491 | 1.0993887 | up | lncRNA |
| ENST00000580420 | RP11-326K13.4 | 0.982874894 | 0.998875101 | 1.0052287 | up | lncRNA |
| ENST00000580458 | SNHG15 | 7.1057E-07 | 0.002549527 | 2.320156 | up | lncRNA |
| ENST00000580487 | RP11-715C4.1 | 0.342170017 | 0.863365698 | 1.4193188 | up | lncRNA |
| ENST00000580500 | LINC00511 | 0.72837412 | 0.982841582 | 1.0357369 | up | lncRNA |
| ENST00000580524 | CTD-2015H3.2 | 0.498914487 | 0.921805853 | 1.065443 | up | lncRNA |
| ENST00000580537 | RP11-849F2.5 | 0.572663437 | 0.945492775 | 1.0560162 | up | lncRNA |
| ENST00000580580 | RP11-862L9.3 | 0.922771101 | 0.998875101 | 1.0203167 | up | lncRNA |
| ENST00000580587 | XXbac-BPG181M17.6 | 0.400433094 | 0.888530576 | 1.085194 | up | lncRNA |
| ENST00000580633 | RP5-1171I10.4 | 0.525336123 | 0.932863107 | 1.0643348 | up | lncRNA |
| ENST00000580639 | CTD-2382H12.1 | 0.345544397 | 0.864988541 | 1.1210372 | up | lncRNA |
| ENST00000580684 | RP11-835E18.2 | 0.857924552 | 0.996142678 | 1.2308862 | up | lncRNA |
| ENST00000580729 | RP11-855A2.5 | 0.429565957 | 0.898463455 | 1.1010458 | up | lncRNA |
| ENST00000580769 | RP11-112H10.4 | 0.036717263 | 0.438043359 | 1.2575396 | up | lncRNA |
| ENST00000580770 | C17orf76-AS1 | 0.866827783 | 0.996767702 | 1.0191939 | up | lncRNA |
| ENST00000580776 | CTD-2532D12.5 | 0.530926954 | 0.934908142 | 1.1004082 | up | lncRNA |
| ENST00000580853 | KIR3DP1 | 0.8119006 | 0.99257857 | 1.0491626 | up | lncRNA |
| ENST00000580867 | RP11-527H14.3 | 0.141266057 | 0.698568819 | 1.5308269 | up | lncRNA |
| ENST00000580884 | RP11-311F12.2 | 0.4338526 | 0.900875689 | 1.2453801 | up | lncRNA |
| ENST00000580897 | RP11-498C9.12 | 0.447971227 | 0.905943546 | 1.1483346 | up | lncRNA |
| ENST00000580927 | RP11-736G13.1 | 0.769436912 | 0.989335115 | 1.0723599 | up | lncRNA |
| ENST00000580996 | RP11-138I1.2 | 0.191613453 | 0.760520345 | 1.0967615 | up | lncRNA |
| ENST00000581028 | RP11-101O21.1 | 0.580439333 | 0.949213695 | 1.1187955 | up | lncRNA |
| ENST00000581072 | hsa-mir-133a-1 | 0.569072779 | 0.943923733 | 1.1322913 | up | lncRNA |
| ENST00000581080 | CTD-2267D19.2 | 0.833149119 | 0.994074191 | 1.1599129 | up | lncRNA |
| ENST00000581083 | RP1-56K13.2 | 0.032573506 | 0.423455575 | 1.1257396 | up | lncRNA |
| ENST00000581198 | RP11-202D1.3 | 0.691742428 | 0.978792006 | 1.0806475 | up | lncRNA |
| ENST00000581240 | RP11-68I3.5 | 0.054560256 | 0.517547122 | 1.2542149 | up | lncRNA |
| ENST00000581247 | AL353997.3 | 0.86341283 | 0.996197518 | 1.0601731 | up | lncRNA |
| ENST00000581274 | RP11-370A5.1 | 0.604280315 | 0.955872488 | 1.1279652 | up | lncRNA |
| ENST00000581333 | RP11-73M7.9 | 0.000233158 | 0.072745323 | 1.6457214 | up | lncRNA |
| ENST00000581360 | RP11-466A19.1 | 0.529755507 | 0.934603134 | 1.1691762 | up | lncRNA |
| ENST00000581398 | CTBP1-AS1 | 0.045959958 | 0.483606918 | 1.0800682 | up | lncRNA |
| ENST00000581442 | RP11-838N2.4 | 0.123739497 | 0.675092482 | 1.1655802 | up | lncRNA |
| ENST00000581457 | CXADRP3 | 0.631816716 | 0.964380442 | 1.3026355 | up | lncRNA |
| ENST00000581489 | RP13-516M14.4 | 0.722082174 | 0.98251396 | 1.037262 | up | lncRNA |
| ENST00000581532 | RP11-784B15.1 | 0.076701826 | 0.571263417 | 1.7928457 | up | lncRNA |
| ENST00000581622 | RP11-927P21.2 | 0.474654346 | 0.917441334 | 1.1880987 | up | lncRNA |
| ENST00000581636 | AC100830.5 | 0.002788814 | 0.154536879 | 1.5973339 | up | lncRNA |
| ENST00000581648 | RP11-178F10.3 | 0.537110347 | 0.936418354 | 1.074347 | up | lncRNA |
| ENST00000581657 | RP11-285E9.6 | 0.57174291 | 0.94531531 | 1.1919066 | up | lncRNA |
| ENST00000581673 | RP11-381P6.1 | 0.59387185 | 0.951072365 | 1.1406525 | up | lncRNA |
| ENST00000581712 | RP11-769O8.3 | 0.09954738 | 0.625253392 | 1.1975883 | up | lncRNA |
| ENST00000581798 | CTD-3096M3.2 | 0.006457696 | 0.217042786 | 3.2046203 | up | lncRNA |
| ENST00000581801 | LINC00511 | 0.805035363 | 0.992578294 | 1.1668823 | up | lncRNA |
| ENST00000581816 | MIR17HG | 0.731947474 | 0.983763148 | 1.1318975 | up | lncRNA |
| ENST00000581905 | RP13-270P17.1 | 0.495792068 | 0.921717611 | 1.0932772 | up | lncRNA |
| ENST00000581922 | AC124789.1 | 0.908713861 | 0.998875101 | 1.0176057 | up | lncRNA |
| ENST00000581935 | ANKRD20A5P | 0.008761594 | 0.247532275 | 1.0904395 | up | lncRNA |
| ENST00000581951 | CTD-2541J13.2 | 0.911115095 | 0.998875101 | 1.01902 | up | lncRNA |
| ENST00000581964 | RP11-321A17.5 | 0.304532693 | 0.845551016 | 1.1630282 | up | lncRNA |
| ENST00000581995 | RP11-338L22.3 | 0.43351103 | 0.900875689 | 1.0513696 | up | lncRNA |
| ENST00000581996 | RP11-17M16.2 | 0.877848949 | 0.998682284 | 1.1447425 | up | lncRNA |
| ENST00000582028 | RP11-453M23.1 | 0.266908777 | 0.828970953 | 1.5230614 | up | lncRNA |
| ENST00000582038 | RP11-88L24.4 | 0.192559801 | 0.760765035 | 1.2903179 | up | lncRNA |
| ENST00000582086 | RP11-161I6.2 | 0.108586435 | 0.642195263 | 1.5324272 | up | lncRNA |
| ENST00000582093 | RP11-474I11.7 | 0.591550486 | 0.950912706 | 1.1714351 | up | lncRNA |
| ENST00000582142 | RP11-6N17.6 | 0.396991989 | 0.887204768 | 1.0713191 | up | lncRNA |
| ENST00000582201 | PLEKHM1P | 0.635322447 | 0.965194977 | 1.054227 | up | lncRNA |
| ENST00000582225 | RP11-726O12.3 | 0.504127939 | 0.92405258 | 1.1150266 | up | lncRNA |
| ENST00000582233 | RP11-53I6.3 | 0.437770871 | 0.902584046 | 1.1761454 | up | lncRNA |
| ENST00000582249 | RP13-991F5.2 | 0.147352973 | 0.703667281 | 1.128554 | up | lncRNA |
| ENST00000582320 | MIR451B | 0.125022435 | 0.67731705 | 1.5308779 | up | lncRNA |
| ENST00000582324 | RP11-344E13.3 | 0.544903375 | 0.939697392 | 1.1441317 | up | lncRNA |
| ENST00000582334 | RP11-963H4.5 | 0.3014651 | 0.842735486 | 1.3396177 | up | lncRNA |
| ENST00000582348 | RP5-1171I10.4 | 0.406501715 | 0.889488526 | 1.0806245 | up | lncRNA |
| ENST00000582375 | RP11-143J12.2 | 0.579602161 | 0.948676882 | 1.1208703 | up | lncRNA |
| ENST00000582435 | RP11-692N5.1 | 0.786522111 | 0.991316428 | 1.1360515 | up | lncRNA |
| ENST00000582470 | RP11-419J16.1 | 0.775822414 | 0.990727199 | 1.0637277 | up | lncRNA |
| ENST00000582483 | RP11-848P1.9 | 0.323295385 | 0.853556911 | 1.3905493 | up | lncRNA |
| ENST00000582505 | RP11-180P8.1 | 0.711968007 | 0.98251396 | 1.0333923 | up | lncRNA |
| ENST00000582527 | RP11-744K17.2 | 0.740662002 | 0.985094857 | 1.0759865 | up | lncRNA |
| ENST00000582546 | RP11-862L9.3 | 0.846952401 | 0.995583664 | 1.0282074 | up | lncRNA |
| ENST00000582689 | RP11-267C16.1 | 0.773864072 | 0.990435061 | 1.0429628 | up | lncRNA |
| ENST00000582695 | RP11-110I1.12 | 0.950955227 | 0.998875101 | 1.0042858 | up | lncRNA |
| ENST00000582697 | RP11-958F21.1 | 0.086020726 | 0.594686635 | 1.2426082 | up | lncRNA |
| ENST00000582712 | LINC00511 | 0.987438403 | 0.99906281 | 1.0019974 | up | lncRNA |
| ENST00000582718 | AC010761.8 | 0.514031441 | 0.929375063 | 1.0999668 | up | lncRNA |
| ENST00000582726 | RP11-510D21.1 | 0.787295435 | 0.991420185 | 1.1104121 | up | lncRNA |
| ENST00000582727 | SNHG15 | 0.001538929 | 0.135129436 | 2.138646 | up | lncRNA |
| ENST00000582752 | AC015842.1 | 0.768666735 | 0.989189555 | 1.0303962 | up | lncRNA |
| ENST00000582755 | RP11-94B19.5 | 0.290473569 | 0.837264328 | 1.1029232 | up | lncRNA |
| ENST00000582780 | CTD-3157E16.1 | 0.614742934 | 0.958423841 | 1.0594051 | up | lncRNA |
| ENST00000582805 | RP11-671C19.1 | 0.143023206 | 0.69945173 | 1.376412 | up | lncRNA |
| ENST00000582842 | CTB-131K11.1 | 0.892370011 | 0.998875101 | 1.0147001 | up | lncRNA |
| ENST00000582896 | RP11-746M1.8 | 0.753200552 | 0.986566987 | 1.0480615 | up | lncRNA |
| ENST00000582940 | RP11-160O5.1 | 0.70740177 | 0.981594335 | 1.0975667 | up | lncRNA |
| ENST00000582965 | RP11-329L6.1 | 0.784770305 | 0.99121281 | 1.08669 | up | lncRNA |
| ENST00000582990 | RP11-728E14.2 | 0.068945923 | 0.553727975 | 1.1943704 | up | lncRNA |
| ENST00000583018 | CTD-2514K5.4 | 0.443146016 | 0.90512482 | 1.0277379 | up | lncRNA |
| ENST00000583044 | AC100830.4 | 0.121950482 | 0.672928817 | 1.229267 | up | lncRNA |
| ENST00000583065 | RPL6P27 | 0.626606076 | 0.962132278 | 1.0435657 | up | lncRNA |
| ENST00000583081 | RP11-143J12.2 | 0.861173772 | 0.996142678 | 1.0107719 | up | lncRNA |
| ENST00000583117 | LINC00200 | 0.286200755 | 0.835918864 | 2.2487928 | up | lncRNA |
| ENST00000583141 | RP11-160E2.19 | 0.489216557 | 0.920777644 | 1.171742 | up | lncRNA |
| ENST00000583156 | RP11-466A19.6 | 0.399981879 | 0.888530576 | 1.0514704 | up | lncRNA |
| ENST00000583175 | RP11-214C8.5 | 0.092309945 | 0.610803569 | 1.1780464 | up | lncRNA |
| ENST00000583236 | CTC-542B22.2 | 0.038221044 | 0.447678433 | 1.3575329 | up | lncRNA |
| ENST00000583262 | RP11-214O1.2 | 0.024480757 | 0.375371614 | 1.264025 | up | lncRNA |
| ENST00000583306 | RP11-527H14.1 | 0.409267336 | 0.890173322 | 1.0790356 | up | lncRNA |
| ENST00000583326 | RP11-862L9.3 | 0.528327895 | 0.934238148 | 1.1048634 | up | lncRNA |
| ENST00000583343 | CTC-297N7.5 | 0.898573034 | 0.998875101 | 1.0458095 | up | lncRNA |
| ENST00000583346 | RP11-640N20.1 | 0.63063518 | 0.964380442 | 1.0604919 | up | lncRNA |
| ENST00000583377 | RP11-848P1.5 | 0.399031947 | 0.888157273 | 1.182248 | up | lncRNA |
| ENST00000583400 | C17orf76-AS1 | 0.324459187 | 0.853958969 | 1.1025178 | up | lncRNA |
| ENST00000583409 | CTB-58E17.3 | 0.007974874 | 0.239948424 | 1.7135489 | up | lncRNA |
| ENST00000583426 | RP11-464D20.6 | 0.692378611 | 0.97895216 | 1.062449 | up | lncRNA |
| ENST00000583442 | RP11-627G18.1 | 0.134823111 | 0.692796739 | 1.3652298 | up | lncRNA |
| ENST00000583462 | RP11-387H17.6 | 0.05701266 | 0.524319427 | 1.2342435 | up | lncRNA |
| ENST00000583490 | RP11-627G18.3 | 0.168592389 | 0.726181864 | 2.423133 | up | lncRNA |
| ENST00000583574 | AC015818.11 | 0.336837345 | 0.859588936 | 1.0987086 | up | lncRNA |
| ENST00000583643 | RP11-720N19.2 | 0.97963448 | 0.998875101 | 1.0035958 | up | lncRNA |
| ENST00000583654 | RP11-571L19.7 | 0.007545927 | 0.230915009 | 1.3102579 | up | lncRNA |
| ENST00000583662 | RP11-524F11.2 | 0.604064236 | 0.955851423 | 1.0524061 | up | lncRNA |
| ENST00000583712 | RP11-449L23.3 | 0.585163166 | 0.949922154 | 1.0442827 | up | lncRNA |
| ENST00000583763 | CSAG4 | 0.066066791 | 0.543687263 | 1.1120999 | up | lncRNA |
| ENST00000583787 | RP11-192H23.7 | 0.677649567 | 0.976096237 | 1.0470792 | up | lncRNA |
| ENST00000583794 | RP11-449D8.5 | 0.352921349 | 0.86894522 | 1.1938614 | up | lncRNA |
| ENST00000583797 | RP11-434D2.11 | 0.002100983 | 0.141857833 | 1.4180003 | up | lncRNA |
| ENST00000583854 | CTD-2532D12.4 | 0.1585291 | 0.714689048 | 1.0778689 | up | lncRNA |
| ENST00000583910 | RP11-474I11.8 | 0.403808412 | 0.889146721 | 1.0759912 | up | lncRNA |
| ENST00000583916 | RP11-171G2.1 | 0.205416703 | 0.77277602 | 1.262427 | up | lncRNA |
| ENST00000583934 | RP11-138I1.4 | 0.020035721 | 0.35282536 | 1.1492157 | up | lncRNA |
| ENST00000583950 | SUZ12P1 | 0.620107611 | 0.96013301 | 1.0876694 | up | lncRNA |
| ENST00000583982 | RP11-267C16.1 | 0.873015063 | 0.997909851 | 1.0389041 | up | lncRNA |
| ENST00000583985 | RP11-92G19.2 | 0.569199782 | 0.943923733 | 1.1515263 | up | lncRNA |
| ENST00000584003 | RP11-101O21.1 | 0.733155486 | 0.983763148 | 1.0466703 | up | lncRNA |
| ENST00000584023 | AC005154.6 | 0.966728847 | 0.998875101 | 1.0081147 | up | lncRNA |
| ENST00000584036 | RP11-678G15.1 | 0.572916414 | 0.945492775 | 1.074275 | up | lncRNA |
| ENST00000584135 | KRT16P1 | 0.903288747 | 0.998875101 | 1.0139014 | up | lncRNA |
| ENST00000584151 | AC022596.2 | 0.905750993 | 0.998875101 | 1.0302486 | up | lncRNA |
| ENST00000584179 | LINC00473 | 0.288973491 | 0.837264328 | 1.4415175 | up | lncRNA |
| ENST00000584190 | RP13-104F24.1 | 0.082365968 | 0.585414993 | 1.1501952 | up | lncRNA |
| ENST00000584191 | RP11-680C21.2 | 0.398100522 | 0.887618401 | 1.1998124 | up | lncRNA |
| ENST00000584201 | RP11-627G18.3 | 0.058190641 | 0.527275332 | 2.3846989 | up | lncRNA |
| ENST00000584264 | CTB-104H12.4 | 0.985694038 | 0.998971647 | 1.0024318 | up | lncRNA |
| ENST00000584277 | RP11-349A8.3 | 0.091941462 | 0.609339348 | 1.1225272 | up | lncRNA |
| ENST00000584327 | SNHG15 | 0.000778588 | 0.098887607 | 2.3440245 | up | lncRNA |
| ENST00000584373 | RP11-627G18.3 | 0.058239191 | 0.527325561 | 2.2623293 | up | lncRNA |
| ENST00000584382 | RP11-142O6.1 | 0.874943505 | 0.997948755 | 1.0629182 | up | lncRNA |
| ENST00000584481 | AC022596.6 | 0.391954527 | 0.885711362 | 1.1079297 | up | lncRNA |
| ENST00000584531 | RP11-527H14.2 | 0.243543253 | 0.810061605 | 1.4293503 | up | lncRNA |
| ENST00000584540 | RP11-640I15.1 | 0.454225128 | 0.910032467 | 1.1457227 | up | lncRNA |
| ENST00000584560 | RP11-552O4.1 | 0.753098744 | 0.986566987 | 1.0267577 | up | lncRNA |
| ENST00000584585 | PLEKHM1P | 0.49208003 | 0.920777644 | 1.0814695 | up | lncRNA |
| ENST00000584621 | AC005154.6 | 0.108865262 | 0.64244922 | 1.1093612 | up | lncRNA |
| ENST00000584660 | AC125421.1 | 0.25430507 | 0.820374967 | 1.1780185 | up | lncRNA |
| ENST00000584683 | RP11-214O1.3 | 0.141919546 | 0.698926614 | 1.627434 | up | lncRNA |
| ENST00000584686 | SNHG15 | 1.85377E-05 | 0.020429495 | 2.2918024 | up | lncRNA |
| ENST00000584721 | RP11-227G15.8 | 0.681802854 | 0.977676223 | 1.049326 | up | lncRNA |
| ENST00000584749 | LINC00511 | 0.994196466 | 0.999156445 | 1.0014475 | up | lncRNA |
| ENST00000584758 | RP11-17M24.1 | 0.141042433 | 0.698568819 | 1.2306024 | up | lncRNA |
| ENST00000584759 | KRT16P3 | 0.36205653 | 0.873747871 | 1.0964257 | up | lncRNA |
| ENST00000584769 | KRT17P2 | 0.91880358 | 0.998875101 | 1.0068651 | up | lncRNA |
| ENST00000584779 | AC010761.6 | 0.518216149 | 0.930711226 | 1.0639458 | up | lncRNA |
| ENST00000584805 | RP11-159D12.10 | 0.279789647 | 0.832297359 | 1.3234513 | up | lncRNA |
| ENST00000584902 | RP11-849N15.5 | 0.974777291 | 0.998875101 | 1.0041894 | up | lncRNA |
| ENST00000584923 | SNORD3A | 0.012766402 | 0.287002307 | 2.0181145 | up | lncRNA |
| ENST00000584934 | RP5-1148A21.3 | 0.998718534 | 0.999477117 | 1.000404 | up | lncRNA |
| ENST00000584962 | KRT16P1 | 0.899835397 | 0.998875101 | 1.0402447 | up | lncRNA |
| ENST00000585003 | CTA-292E10.6 | 0.263680731 | 0.827360264 | 1.1948072 | up | lncRNA |
| ENST00000585025 | CTC-542B22.1 | 0.627117277 | 0.962299493 | 1.0606043 | up | lncRNA |
| ENST00000585028 | RP11-172F10.1 | 0.878199144 | 0.998682284 | 1.0193263 | up | lncRNA |
| ENST00000585030 | SNHG15 | 6.61576E-06 | 0.009494942 | 2.3779867 | up | lncRNA |
| ENST00000585184 | RP11-17J14.2 | 0.388328813 | 0.88524429 | 1.1304174 | up | lncRNA |
| ENST00000585189 | RP11-192H23.5 | 0.061950424 | 0.534658422 | 1.6664941 | up | lncRNA |
| ENST00000585193 | RP11-401F2.3 | 0.365600989 | 0.874818743 | 2.028651 | up | lncRNA |
| ENST00000585232 | RP11-549B18.2 | 0.484448281 | 0.919439531 | 1.1020948 | up | lncRNA |
| ENST00000585275 | RP11-849F2.4 | 0.581883331 | 0.949239022 | 1.0493047 | up | lncRNA |
| ENST00000585303 | CTC-297N7.7 | 0.267923836 | 0.829789144 | 1.167105 | up | lncRNA |
| FN091184 |  | 0.977656413 | 0.998875101 | 1.0054217 | up | lncRNA |
| H16291 |  | 0.330091144 | 0.85556386 | 1.2856155 | up | lncRNA |
| HMlincRNA1018- | HMlincRNA1018 | 0.564173084 | 0.943393901 | 1.0918114 | up | lncRNA |
| HMlincRNA1090+ | HMlincRNA1090 | 0.24468634 | 0.811577674 | 1.4031264 | up | lncRNA |
| HMlincRNA1103+ | HMlincRNA1103 | 0.7117642 | 0.98251396 | 1.1190237 | up | lncRNA |
| HMlincRNA1106- | HMlincRNA1106 | 0.34301317 | 0.863514929 | 1.1250007 | up | lncRNA |
| HMlincRNA1110- | HMlincRNA1110 | 0.567029225 | 0.943923733 | 1.1450571 | up | lncRNA |
| HMlincRNA1172+ | HMlincRNA1172 | 0.731356348 | 0.983763148 | 1.0745596 | up | lncRNA |
| HMlincRNA1179- | HMlincRNA1179 | 0.382265583 | 0.881294633 | 1.119721 | up | lncRNA |
| HMlincRNA1235+ | HMlincRNA1235 | 0.574439415 | 0.946756534 | 1.0424963 | up | lncRNA |
| HMlincRNA1256+ | HMlincRNA1256 | 0.43476371 | 0.901162429 | 1.2542737 | up | lncRNA |
| HMlincRNA1266+ | HMlincRNA1266 | 0.651525329 | 0.969927925 | 1.1337927 | up | lncRNA |
| HMlincRNA1294+ | HMlincRNA1294 | 0.203391734 | 0.770611976 | 1.1891555 | up | lncRNA |
| HMlincRNA1314+ | HMlincRNA1314 | 0.793992776 | 0.991420185 | 1.0801319 | up | lncRNA |
| HMlincRNA133- | HMlincRNA133 | 0.375030754 | 0.879312727 | 1.094521 | up | lncRNA |
| HMlincRNA136- | HMlincRNA136 | 0.763081263 | 0.988817776 | 1.043447 | up | lncRNA |
| HMlincRNA1397- | HMlincRNA1397 | 0.397435689 | 0.887348439 | 1.0702503 | up | lncRNA |
| HMlincRNA1397+ | HMlincRNA1397 | 0.547099913 | 0.94058193 | 1.1316794 | up | lncRNA |
| HMlincRNA1422+ | HMlincRNA1422 | 0.920005424 | 0.998875101 | 1.0102594 | up | lncRNA |
| HMlincRNA1441+ | HMlincRNA1441 | 0.034502053 | 0.429465272 | 1.1543661 | up | lncRNA |
| HMlincRNA1452+ | HMlincRNA1452 | 0.465711468 | 0.913821992 | 1.3153038 | up | lncRNA |
| HMlincRNA1466+ | HMlincRNA1466 | 0.163601462 | 0.718706279 | 1.1637956 | up | lncRNA |
| HMlincRNA1467- | HMlincRNA1467 | 0.036441159 | 0.436563873 | 1.2548217 | up | lncRNA |
| HMlincRNA152- | HMlincRNA152 | 0.824399127 | 0.993856674 | 1.0228165 | up | lncRNA |
| HMlincRNA152+ | HMlincRNA152 | 0.013733671 | 0.294694748 | 1.4442162 | up | lncRNA |
| HMlincRNA1546+ | HMlincRNA1546 | 0.46487082 | 0.913821992 | 1.1444106 | up | lncRNA |
| HMlincRNA1548- | HMlincRNA1548 | 0.076283073 | 0.569326396 | 1.347449 | up | lncRNA |
| HMlincRNA156+ | HMlincRNA156 | 0.352131261 | 0.868751345 | 1.2519327 | up | lncRNA |
| HMlincRNA1562+ | HMlincRNA1562 | 0.371386158 | 0.877243933 | 1.0638502 | up | lncRNA |
| HMlincRNA1573+ | HMlincRNA1573 | 0.824242851 | 0.993856674 | 1.0152146 | up | lncRNA |
| HMlincRNA158- | HMlincRNA158 | 0.486749728 | 0.920642653 | 1.0629049 | up | lncRNA |
| HMlincRNA1584- | HMlincRNA1584 | 0.092758205 | 0.612440005 | 1.5631819 | up | lncRNA |
| HMlincRNA1584+ | HMlincRNA1584 | 0.813287482 | 0.99257857 | 1.0284676 | up | lncRNA |
| HMlincRNA1593+ | HMlincRNA1593 | 0.70803114 | 0.981606358 | 1.0677483 | up | lncRNA |
| HMlincRNA1594+ | HMlincRNA1594 | 0.188838516 | 0.756051633 | 1.5998922 | up | lncRNA |
| HMlincRNA1602+ | HMlincRNA1602 | 0.996213642 | 0.999156445 | 1.0009357 | up | lncRNA |
| HMlincRNA1607- | HMlincRNA1607 | 0.155761677 | 0.711341825 | 1.524039 | up | lncRNA |
| HMlincRNA1613- | HMlincRNA1613 | 0.435511145 | 0.901926599 | 1.0279423 | up | lncRNA |
| HMlincRNA1623- | HMlincRNA1623 | 0.803000481 | 0.992578294 | 1.0398094 | up | lncRNA |
| HMlincRNA1636- | HMlincRNA1636 | 0.772002484 | 0.989973164 | 1.0843655 | up | lncRNA |
| HMlincRNA1639+ | HMlincRNA1639 | 0.060891719 | 0.532876797 | 1.1167532 | up | lncRNA |
| HMlincRNA181- | HMlincRNA181 | 0.392510135 | 0.885711362 | 1.1085706 | up | lncRNA |
| HMlincRNA230+ | HMlincRNA230 | 0.614860262 | 0.958423841 | 1.199147 | up | lncRNA |
| HMlincRNA250- | HMlincRNA250 | 0.265658683 | 0.828662678 | 1.1336636 | up | lncRNA |
| HMlincRNA250+ | HMlincRNA250 | 0.685139535 | 0.977836377 | 1.0425282 | up | lncRNA |
| HMlincRNA30- | HMlincRNA30 | 0.731855584 | 0.983763148 | 1.034924 | up | lncRNA |
| HMlincRNA346- | HMlincRNA346 | 0.69973315 | 0.980170937 | 1.066876 | up | lncRNA |
| HMlincRNA392- | HMlincRNA392 | 0.506199221 | 0.925291133 | 1.1016456 | up | lncRNA |
| HMlincRNA420- | HMlincRNA420 | 0.593095328 | 0.950912706 | 1.0695702 | up | lncRNA |
| HMlincRNA420+ | HMlincRNA420 | 0.445814623 | 0.90519468 | 1.4042204 | up | lncRNA |
| HMlincRNA425- | HMlincRNA425 | 0.074665506 | 0.566975999 | 1.2711834 | up | lncRNA |
| HMlincRNA429- | HMlincRNA429 | 0.887982159 | 0.998875101 | 1.03943 | up | lncRNA |
| HMlincRNA440+ | HMlincRNA440 | 0.181110841 | 0.747570548 | 1.3634934 | up | lncRNA |
| HMlincRNA463- | HMlincRNA463 | 0.259629638 | 0.824094195 | 1.0551191 | up | lncRNA |
| HMlincRNA472- | HMlincRNA472 | 0.437670562 | 0.902506883 | 1.1162816 | up | lncRNA |
| HMlincRNA478- | HMlincRNA478 | 0.14706304 | 0.703667281 | 1.2005915 | up | lncRNA |
| HMlincRNA482- | HMlincRNA482 | 0.896681064 | 0.998875101 | 1.0184414 | up | lncRNA |
| HMlincRNA482+ | HMlincRNA482 | 0.018072138 | 0.337638939 | 1.4298526 | up | lncRNA |
| HMlincRNA52- | HMlincRNA52 | 0.298698255 | 0.841989654 | 1.1316646 | up | lncRNA |
| HMlincRNA528- | HMlincRNA528 | 0.966052745 | 0.998875101 | 1.0069153 | up | lncRNA |
| HMlincRNA543+ | HMlincRNA543 | 0.073660915 | 0.56397543 | 1.3461841 | up | lncRNA |
| HMlincRNA561- | HMlincRNA561 | 0.640759806 | 0.966493404 | 1.0831768 | up | lncRNA |
| HMlincRNA577+ | HMlincRNA577 | 0.927175671 | 0.998875101 | 1.0249727 | up | lncRNA |
| HMlincRNA617+ | HMlincRNA617 | 0.138499196 | 0.697263489 | 1.2518737 | up | lncRNA |
| HMlincRNA634- | HMlincRNA634 | 0.346298195 | 0.865832425 | 1.1853628 | up | lncRNA |
| HMlincRNA639- | HMlincRNA639 | 0.58847996 | 0.950589384 | 1.0967917 | up | lncRNA |
| HMlincRNA705- | HMlincRNA705 | 0.5441419 | 0.939697392 | 1.3664873 | up | lncRNA |
| HMlincRNA716- | HMlincRNA716 | 0.232380272 | 0.799746466 | 1.1804229 | up | lncRNA |
| HMlincRNA745- | HMlincRNA745 | 0.403686643 | 0.889146721 | 1.3817886 | up | lncRNA |
| HMlincRNA791- | HMlincRNA791 | 0.13149179 | 0.686687521 | 1.2780363 | up | lncRNA |
| HMlincRNA81+ | HMlincRNA81 | 0.664734011 | 0.972257778 | 1.1871194 | up | lncRNA |
| HMlincRNA814+ | HMlincRNA814 | 0.485451914 | 0.920127558 | 1.1398406 | up | lncRNA |
| HMlincRNA815- | HMlincRNA815 | 0.269701485 | 0.830995087 | 1.3266746 | up | lncRNA |
| HMlincRNA824+ | HMlincRNA824 | 0.553807047 | 0.943023022 | 1.1605693 | up | lncRNA |
| HMlincRNA825- | HMlincRNA825 | 0.728962747 | 0.982841582 | 1.0235685 | up | lncRNA |
| HMlincRNA825+ | HMlincRNA825 | 0.902179591 | 0.998875101 | 1.0154907 | up | lncRNA |
| HMlincRNA858- | HMlincRNA858 | 0.162662983 | 0.718236848 | 1.2958092 | up | lncRNA |
| HMlincRNA892+ | HMlincRNA892 | 0.473036968 | 0.91677171 | 1.1266645 | up | lncRNA |
| HMlincRNA898+ | HMlincRNA898 | 0.304286401 | 0.845358864 | 1.2679362 | up | lncRNA |
| HMlincRNA901+ | HMlincRNA901 | 0.067947278 | 0.550752865 | 1.7035335 | up | lncRNA |
| HMlincRNA921+ | HMlincRNA921 | 0.024270412 | 0.374301178 | 2.4434534 | up | lncRNA |
| HMlincRNA954- | HMlincRNA954 | 0.871021591 | 0.997909851 | 1.0169216 | up | lncRNA |
| HMlincRNA963+ | HMlincRNA963 | 0.043293306 | 0.473947772 | 1.9008588 | up | lncRNA |
| HMlincRNA986- | HMlincRNA986 | 0.733895803 | 0.983763148 | 1.1415523 | up | lncRNA |
| K00627 |  | 0.432423739 | 0.900592919 | 1.3808067 | up | lncRNA |
| N66985 |  | 0.564774275 | 0.943393901 | 1.7658393 | up | lncRNA |
| N71455 |  | 0.908397541 | 0.998875101 | 1.0032184 | up | lncRNA |
| NR_001458 | MIR155HG | 0.000425617 | 0.0868848 | 37.4907409 | up | lncRNA |
| NR_001459 | SNHG10 | 0.963308372 | 0.998875101 | 1.0108084 | up | lncRNA |
| NR_001524 | TTTY3 | 0.84556765 | 0.995583664 | 1.0338816 | up | lncRNA |
| NR_001533 | TTTY8 | 0.221066829 | 0.790618275 | 1.0601105 | up | lncRNA |
| NR_001536 | TTTY2 | 0.943218229 | 0.998875101 | 1.011156 | up | lncRNA |
| NR_001566 | TERC | 0.918935089 | 0.998875101 | 1.0095789 | up | lncRNA |
| NR_002196 | H19 | 0.691659301 | 0.978770882 | 1.1058043 | up | lncRNA |
| NR_002312 | RPPH1 | 0.104287969 | 0.63448111 | 1.2345598 | up | lncRNA |
| NR_002612 | DLEU2 | 0.043455788 | 0.475363923 | 1.1618905 | up | lncRNA |
| NR_002765 | ASAP1-IT1 | 0.014555363 | 0.306399703 | 1.8997107 | up | lncRNA |
| NR_002766 | MEG3 | 0.824035887 | 0.993856674 | 1.01366 | up | lncRNA |
| NR_002791 | EMX2OS | 0.003953404 | 0.179554598 | 2.1371771 | up | lncRNA |
| NR_002795 | HOXA11-AS | 0.6593802 | 0.971704562 | 1.0930231 | up | lncRNA |
| NR_002813 | KCNIP4-IT1 | 0.282535231 | 0.832637708 | 1.4049178 | up | lncRNA |
| NR_003051 | RMRP | 0.01187455 | 0.278015572 | 1.1228421 | up | lncRNA |
| NR_003089 | OTUB1 | 0.663548958 | 0.971990255 | 1.0046191 | up | lncRNA |
| NR_003136 | FBXO22-AS1 | 0.811806157 | 0.99257857 | 1.0113408 | up | lncRNA |
| NR_003141 | SNHG4 | 0.930636832 | 0.998875101 | 1.0290462 | up | lncRNA |
| NR_003142 | SNHG9 | 0.595690104 | 0.951509512 | 1.0170895 | up | lncRNA |
| NR_003149 | GPR98 | 0.523337335 | 0.932117592 | 1.1062934 | up | lncRNA |
| NR_003189 | SYS1-DBNDD2 | 0.370399794 | 0.877177739 | 1.0210737 | up | lncRNA |
| NR_003530 | MEG3 | 0.555576203 | 0.943023022 | 1.0495401 | up | lncRNA |
| NR_003562 | MPP3 | 0.680630665 | 0.976938825 | 1.0291626 | up | lncRNA |
| NR_003589 | TTTY1B | 0.142861995 | 0.69945173 | 1.5154222 | up | lncRNA |
| NR_003592 | TTTY7B | 0.136833222 | 0.693687883 | 1.1003349 | up | lncRNA |
| NR_003604 | ZNFX1-AS1 | 0.771047018 | 0.989973164 | 1.0531061 | up | lncRNA |
| NR_003605 | ZNFX1-AS1 | 0.530981578 | 0.934908142 | 1.0677646 | up | lncRNA |
| NR_003606 | ZNFX1-AS1 | 0.679497489 | 0.976550965 | 1.0554033 | up | lncRNA |
| NR_003697 | SNHG15 | 2.24068E-06 | 0.004528265 | 2.4318344 | up | lncRNA |
| NR_004422 | HPVC1 | 0.156618328 | 0.71227166 | 1.2869217 | up | lncRNA |
| NR_004431 | GHRLOS | 0.560414469 | 0.943393901 | 1.054421 | up | lncRNA |
| NR_004855 | HULC | 0.280525569 | 0.832297359 | 1.0854382 | up | lncRNA |
| NR_015361 | LOC440896 | 0.815163302 | 0.99257857 | 1.0540664 | up | lncRNA |
| NR_015363 | MGC21881 | 0.218900054 | 0.787447617 | 1.2675186 | up | lncRNA |
| NR_015368 | LOC643837 | 0.793725137 | 0.991420185 | 1.1005542 | up | lncRNA |
| NR_015369 | FLJ42709 | 0.429874892 | 0.898685776 | 1.2143473 | up | lncRNA |
| NR_015381 | TP53TG1 | 0.581991246 | 0.949239022 | 1.1768705 | up | lncRNA |
| NR_015383 | LOC340357 | 0.924667056 | 0.998875101 | 1.0134254 | up | lncRNA |
| NR_015384 | LOC100126784 | 0.197003498 | 0.763327268 | 1.1414031 | up | lncRNA |
| NR_015389 | LOC339290 | 0.391405004 | 0.885711362 | 1.0819069 | up | lncRNA |
| NR_015390 | LINC00608 | 0.405991838 | 0.889320642 | 1.1141197 | up | lncRNA |
| NR_015396 | LOC440356 | 0.718779044 | 0.98251396 | 1.0223205 | up | lncRNA |
| NR_015407 | LOC339535 | 0.805012485 | 0.992578294 | 1.211581 | up | lncRNA |
| NR_015414 | LINC00635 | 0.853511812 | 0.996142678 | 1.0306946 | up | lncRNA |
| NR_015419 | LOC145783 | 0.391865972 | 0.885711362 | 1.2568951 | up | lncRNA |
| NR_015422 | LOC149134 | 0.021345612 | 0.359097712 | 1.0859727 | up | lncRNA |
| NR_015429 | LOC283050 | 0.570444388 | 0.944646886 | 1.0745727 | up | lncRNA |
| NR_015447 | LOC153684 | 0.783066162 | 0.99121281 | 1.0212091 | up | lncRNA |
| NR_015448 | DLX6-AS1 | 0.561989593 | 0.943393901 | 1.0984013 | up | lncRNA |
| NR_015453 | NOP14-AS1 | 0.977833759 | 0.998875101 | 1.0028055 | up | lncRNA |
| NR_021490 | FLJ42709 | 0.858323722 | 0.996142678 | 1.0983758 | up | lncRNA |
| NR_023314 | THTPA | 0.876352237 | 0.99852392 | 1.0179236 | up | lncRNA |
| NR_023318 | ADPGK | 0.458541494 | 0.91221792 | 1.0628841 | up | lncRNA |
| NR_023348 | GAS8 | 0.138826239 | 0.69776743 | 1.0512081 | up | lncRNA |
| NR_023359 | CABLES1 | 0.781649829 | 0.99121281 | 1.1168561 | up | lncRNA |
| NR_023389 | LINC00476 | 0.47175858 | 0.916418344 | 1.2147851 | up | lncRNA |
| NR_023920 | WT1-AS | 0.427809594 | 0.897780553 | 1.0748099 | up | lncRNA |
| NR_023921 | DHRS4-AS1 | 0.214718422 | 0.781745 | 1.0853022 | up | lncRNA |
| NR_023922 | DHRS4-AS1 | 0.0695411 | 0.55572632 | 1.227572 | up | lncRNA |
| NR_024006 | FP588 | 0.887791548 | 0.998875101 | 1.0504401 | up | lncRNA |
| NR_024012 | TEX264 | 0.718808676 | 0.98251396 | 1.0346692 | up | lncRNA |
| NR_024015 | TDRG1 | 0.277943771 | 0.832297359 | 1.1132176 | up | lncRNA |
| NR_024028 | LINC00112 | 0.037563474 | 0.443140005 | 1.2335392 | up | lncRNA |
| NR_024047 | WNT2 | 0.305108004 | 0.846002718 | 1.4067229 | up | lncRNA |
| NR_024048 | TAZ | 0.811162157 | 0.99257857 | 1.0219127 | up | lncRNA |
| NR_024052 | HCG18 | 0.659970902 | 0.971704562 | 1.0911271 | up | lncRNA |
| NR_024053 | HCG18 | 0.774132872 | 0.990435061 | 1.0424374 | up | lncRNA |
| NR_024065 | LINC00312 | 0.430551152 | 0.898769881 | 1.5177556 | up | lncRNA |
| NR_024089 | LINC00162 | 0.925280346 | 0.998875101 | 1.1431941 | up | lncRNA |
| NR_024091 | ZFP91-CNTF | 0.150374418 | 0.705518742 | 1.1647982 | up | lncRNA |
| NR_024092 | LINC00515 | 0.0021251 | 0.141857833 | 2.5071091 | up | lncRNA |
| NR_024101 | FLJ35776 | 0.006073922 | 0.212479102 | 1.7924164 | up | lncRNA |
| NR_024129 | LINC00092 | 0.010581907 | 0.261616866 | 1.139919 | up | lncRNA |
| NR_024144 | GHRLOS | 0.090151422 | 0.604886959 | 1.5723565 | up | lncRNA |
| NR_024145 | GHRLOS | 0.351417491 | 0.86822927 | 1.0442393 | up | lncRNA |
| NR_024146 | GHRLOS | 0.38752431 | 0.884642738 | 1.184861 | up | lncRNA |
| NR_024170 | LAMTOR3 | 0.453733782 | 0.909732981 | 1.0355257 | up | lncRNA |
| NR_024183 | LINC00521 | 0.772783226 | 0.990198305 | 1.0356966 | up | lncRNA |
| NR_024194 | TOM1 | 0.359627264 | 0.873342057 | 1.1119863 | up | lncRNA |
| NR_024199 | CBLL1 | 0.215241737 | 0.782855908 | 1.1672826 | up | lncRNA |
| NR_024204 | LINC00152 | 0.010934706 | 0.265092726 | 1.5159472 | up | lncRNA |
| NR_024205 | LINC00152 | 0.024775564 | 0.377450959 | 1.7297663 | up | lncRNA |
| NR_024206 | LINC00152 | 0.180197668 | 0.745729299 | 1.6195762 | up | lncRNA |
| NR_024212 | RNF185 | 0.767574395 | 0.989189555 | 1.0912473 | up | lncRNA |
| NR_024281 | LINC00599 | 0.083000668 | 0.585414993 | 1.2063122 | up | lncRNA |
| NR_024330 | LINC00085 | 0.831995272 | 0.994074191 | 1.031872 | up | lncRNA |
| NR_024334 | FUT8-AS1 | 0.495511285 | 0.921717611 | 1.0978889 | up | lncRNA |
| NR_024355 | LINC00634 | 0.920559797 | 0.998875101 | 1.0192034 | up | lncRNA |
| NR_024368 | FLJ45340 | 0.82441295 | 0.993856674 | 1.0617375 | up | lncRNA |
| NR_024373 | LOC541471 | 0.181089299 | 0.747570548 | 1.5368239 | up | lncRNA |
| NR_024388 | LOC152217 | 0.999269671 | 0.9996876 | 1.0000831 | up | lncRNA |
| NR_024396 | LINC00638 | 0.345516779 | 0.864988541 | 1.1741291 | up | lncRNA |
| NR_024408 | LOC253039 | 0.450797743 | 0.908000742 | 1.0212153 | up | lncRNA |
| NR_024410 | LOC254312 | 0.900661161 | 0.998875101 | 1.0422952 | up | lncRNA |
| NR_024411 | LOC254312 | 0.000695406 | 0.095052113 | 4.1049118 | up | lncRNA |
| NR_024430 | MIR100HG | 0.961471756 | 0.998875101 | 1.0066245 | up | lncRNA |
| NR_024431 | LOC283050 | 0.062254881 | 0.534765065 | 1.1814669 | up | lncRNA |
| NR_024434 | LOC100133991 | 0.419604362 | 0.894750301 | 1.1676151 | up | lncRNA |
| NR_024437 | LOC728323 | 0.5341255 | 0.935381352 | 1.0763408 | up | lncRNA |
| NR_024440 | LOC729609 | 0.219110355 | 0.787447617 | 1.0723909 | up | lncRNA |
| NR_024451 | LOC100134229 | 0.008367336 | 0.244110885 | 4.2163621 | up | lncRNA |
| NR_024453 | LOC100134368 | 0.914166823 | 0.998875101 | 1.0305078 | up | lncRNA |
[truncated: 993,738 more chars]
